# LncRNA PANTR1 is Associated with Poor Prognostic and Suppresses Apoptosis in Glioma

**DOI:** 10.1155/2023/8537036

**Published:** 2023-02-20

**Authors:** Fei Shi, Jie Hu, Ping Zheng, Yisong Lv, Hongyu Liu, Guiyun Zhang, Hongyu Jiang

**Affiliations:** ^1^Department of Neurovascular Intervention and Neurosurgery, Clinical Center of Neuroscience, Shanghai General Hospital, Shanghai Jiao Tong University School of Medicine, Shanghai, China; ^2^Department of Neurosurgery, Jiading Branch of Shanghai General Hospital, Shanghai Jiao Tong University School of Medicine, Shanghai, China; ^3^Department of Neurosurgery, Shanghai Pudong New Area People's Hospital, Shanghai, China; ^4^School of Continuing Education, Shanghai Jiao Tong University, Shanghai, China; ^5^Department of Neurosurgery, First Medical Center of Chinese PLA General Hospital, Beijing, China; ^6^Department of Neurosurgery, Hainan Hospital of Chinese PLA General Hospital, Beijing, China; ^7^Medical School of Chinese PLA, Beijing, China; ^8^Department of Anesthesiology, Wuxi Second People's Hospital, Wuxi, Jiangsu Province, China

## Abstract

Glioma is the most common tumor in the central nervous system. High-grade gliomas confer a poor prognosis, being a serious health and economic burden. Current literature suggests the important role of long noncoding RNA (lncRNA) in mammals, especially in tumorigenesis of various tumors. The functions of lncRNA POU3F3 adjacent noncoding transcript 1 (PANTR1) have been investigated in hepatocellular carcinoma but remain yet unclear in gliomas. We evaluated the role of PANTR1 in glioma cells using published data from The Cancer Genome Atlas (TCGA), then validated it by ex vivo experiments. To investigate the potential cellular mechanism of different levels of PANTR1 expression in glioma cells, we used siRNA-mediated knockdown in low-grade (grade II) cell lines and GBM (grade IV) cell lines (SW1088 and SHG44, respectively). On the molecular level, low expression of PANTR1 caused significantly reduced glioma cell viability and enhanced cell death. Moreover, we identified the importance of PANTR1 expression for cell migration in both cell lines, a critical foundation for invasiveness in recurrent gliomas. In conclusion, this study provides the first evidence that PANTR1 has a relevant role in human glioma by influencing cell viability and cell death.

## 1. Introduction

Glioma is the most common primary brain tumor, accounting for 81% of malignant intracranial tumors and resulting in severe morbidity and mortality [1]. Glioblastoma (GBM), the most prevalent glioma histology (∼45% of all gliomas), remains a poor median survival time which is 12–15 months, despite advancements in treatment techniques such as surgery, chemotherapy, and radiotherapy [2–4], since of substantial drug resistance and a near-total recurrence rate [5]. To achieve a favorable prognosis, it is essential to establish novel therapeutic interventions. Plenty of research has suggested that extracellular vehicles (EVs) may hold great potential for creating more effective therapies for tumor patients [6, 7]. These findings revealed an increasing recognition of long noncoding RNAs (LncRNAs) as crucial mediators of EV biological effects [6].

lncRNAs are defined as any RNA with a length of more than 200 bps but with no obvious coding function [8]. They have diverse roles in tumorigenesis, cell viability, cell death, as well as cell migration and invasiveness [9, 10]. Several recent studies have indicated that lncRNAs are related to the response of immunotherapy, TMZ resistance, and radiotherapy resistance, which could further affect the therapeutic outcomes of glioma patients [11, 12]. Nowadays, applications of microarray technology and bioinformatic analysis have enabled a comprehensive investigation for identifying novel functional RNAs [13]. Especially the regulatory role of lncRNAs in tumor cell microenvironment and tumor growth has received a lot of attention. Although previously published studies suggested that some lncRNAs may have been associated with gliomas, the specific characteristics by which lncRNAs act on glioma are not yet fully understood [14, 15]. The mechanism underlying the interaction between lncRNAs and glioma remains unknown.

Bian et al. found that glioma cells-derived sEVs delivering lncRNA-ATB can activate astrocytes by inhibiting miR-204-3p, and the activated astrocytes, in turn, promoted glioma cell invasion [16]. LncRNA AHIF, the natural antisense transcript of hypoxia-inducible factor 1*α* (HIF 1*α*), was reported to promote glioma cell proliferation, invasion, and radioresistance via sEVs and may act as a potential therapeutic target [17, 18]. Chai and colleagues found that lncRNA ROR1-AS1 was up-regulated in glioma tissues in comparison with normal tissue, and high expression of lncRNA ROR1-AS1 presented a poor prognosis. Further research showed that sEVs derived from tumor cells and decorated with lncRNA ROR1-AS1 could facilitate glioma progression via inhibiting miR-4686 [19].

POU3F3 adjacent noncoding transcript 1 (PANTR1) is an oncogenic long noncoding RNA that is located at 4 kb upstream of the protein-encoding POU3F3 gene that has a substantial influence on a variety of cellular features in various types of cancer. PANTR1 was initially found to be associating with the regulator of cortical development [20]. However, its role in tumorigenesis has not been fully investigated. Thus, the present study aimed to explore a novel interactome, PANTR1, and to discover the inner relationship between PANTR1 and gliomas. Considering PANTR1 has previously been reported to be associating with tumorigenesis of hepatocellular carcinoma, we hypothesize that PANTR1 may play a significant role in glioma tumorigenesis [21].

## 2. Material and Methods

### 2.1. Data Acquisition from TCGA

We started with data downloaded from GBM and LGG collection of TCGA datasets (https://portal.gdc.cancer.gov/). RNA-Seq data and their corresponding clinical data were obtained in Level 3 HTseq-FPKM format, and RNAseq data without corresponding clinical data were discarded. There were 670 cases with RNAseq data after the exclusion of cases without clinical data. For sample size calculation, we used Open Source Epidemiologic Statistics for Public Health (OpenEpi) to show that a size of at least 663 cases could achieve a 99% confidence level. RNAseq data in FPKM (fragments per kilobase per million) format were converted into TPM (transcripts per million reads) format for expression comparison among samples. The data of WHO grade, IDH mutation, and 1p/19q codeletion status were obtained from the study of Ceccarelli et al. [22].

RNAseq data in TPM format of TCGA and GTEx after unified processing with the toil pipeline were downloaded from UCSC XENA (https://xenabrowser.net/datapages/) [23].

The expression levels of PANTR1 in 5 normal adjacents to tumor (NAT) samples and 670 glioma samples of GBM and LGG collection were compared. Similarly, the expression levels of PANTR1 in tumor tissues were compared with NAT tissues of GTEx combined with TCGA.

### 2.2. Cell Culture and Transfection

The human glioma cell lines grade IV glioblastoma (SHG44) (cat#TCHu 48) and grade II astrocytoma (SW1088) (cat#HTB-12) were obtained from Professor Tianyi Liu at PLA301 Hospital in Beijing, China. SHG44 cells were cultured in minimal essential medium (MEM) with 1% penicillin/streptomycin, 1% nonessential amino acids, 10% fetal bovine serum (FBS), and L-15 medium for SW1088. All samples were cultured in 5% CO_2_ incubators at the temperature of 37°C.

### 2.3. Collection of Glioma Tissues

For PANTR1 expression comparison, glioma tissues (*n* = 15) and normal adjacent tissues (*n* = 15) were collected from Shanghai General Hospital between February 2017 and October 2021, consisting of 12 grade II/III glioma patients and 3 GBM patients. Inclusion criteria included: (i) patients who underwent gross total resection and postoperative pathology diagnosed as glioma; (ii) complete patient information acquired. Exclusion criteria included: (i) patients who underwent any preoperative therapies such as radiotherapy and chemotherapy; (ii) Patients with multiple histological malignant tumor history; (iii) Patients with severe heart, lung, liver, spleen, or kidney diseases. This study was performed under the approval of local medical ethics (No. 2020SQ119) Shanghai General Hospital. The processing of clinical tissue samples complies with the ethical standards of the Declaration of Helsinki. Informed consent of tissue sample donations for biomedical research was signed by all patients.

### 2.4. RNA Extraction and Reverse Transcription-Quantitative (RT-q) PCR

RNAs were extracted from the glioma tissue, the NAT tissues, and SHG44 and SW1088 cell lines using a standard TRIzol protocol following the manufacturer's instructions. A High-Capacity cDNA Reverse Transcription Kit (Aidlab, Beijing, China) was used for cDNA synthesis following the manufacturer's protocol. A 7500 Real-Time PCR System (Applied Biosystems, CA, USA) was used for qRT-PCR with an SYBR Green PCR Kit (Roche, USA). The expression fold-change was determined using the 2^−ΔΔCT^.

### 2.5. Wound-Healing Assay

The migratory ability of glioma cells was evaluated by wound-healing assay. Cells were cultured in 6-well plates (1 × 10⁵/well) until 100% confluence was achieved. Then, the cells were wounded using a plastic pipette tip, after which the cells were washed with PBS and incubated for 24 h. Thereafter, the wounded areas were analyzed using ImageJ software (NIH, Bethesda, MD, USA).

### 2.6. Cell Migration and Invasion Assays

The cell migration and invasion assays were carried out with 6-well Transwell insert chambers (Corning, NY, USA). Cells (5 × 10^4^) were cultured in the upper Matrigel chamber. After 48 h of incubation at 37°C, the cells from the upper chamber were removed by cotton swabs, and the residual cells in the bottom chamber were fixed with methanol and stained with 1% crystal violet for 30 min. Cells were then enumerated using a microscope.

### 2.7. Annexin V/Propidium Iodide Assay

To perform flow cytometric analysis, death cells were harvested using an Annexin V Cell death Detection Kit (BD Biosciences, CA, USA). Cells in the log phase were plated in 6-well plates at a density of 2 × 10^5^ cells/well. In different groups, glioma cells were harvested using the Accutase™ cell detachment solution (Sigma-Aldrich, USA) and then labeled with V-FITC reagents following the protocol of BD Bioscience. The stained cells were analyzed via flow cytometry and enumerated by FACSDiva software.

### 2.8. Western Blot Analysis

For Western blot analysis, we transferred total protein to a polyvinyldifluoridene membrane via RIPA lysis buffer. Protein expression was tested using the BCA protein assay. Total protein extracts were separated by sodium dodecyl sulfate-polyacrylamide gel electrophoresis (12% resolving gel) and then electro-transferred to nitrocellulose membranes. The primary antibodies for total or p-akt (ser 473) (Cat No.: # 5012), AKT (Cat No.: # 9272), and *β*-actin (Cat No.: # 3700s) rabbit mAb were obtained from Cell signaling Technology (CST), which have already been validated by the CST company. Rabbit anti-goat IgG horse-radish peroxidase-conjugated secondary antibodies were used for these experiments. We controlled the loaded amount of each lane and validated the expression of *β*-actin in the WB study. The immunoreactive bands were captured on an X-ray film as described previously [1]. The western blot assay was performed 6 times.

### 2.9. Gene Enrichment Analysis

Gene set enrichment analysis (GSEA) for high and low levels of expression of PANTR1 was performed using the clusterProfiler package [24]. The *a priori* gene sets were c2.cp.v7.0.symbols.gmt [Curated] from MSigDB Collections. Significant enrichment was defined as a false discovery rate (FDR) < 0.25 and p.adjust <0.05 [5].

### 2.10. Statistical Analysis

All the statistical analysis processes were verified by independent authors who were blinded to the experimental groups at least three times. The tumor cells were grouped randomly. To compare two groups of continuous variables, the statistical significance of normally distributed variables was estimated using the independent Student's *t*-test, and the differences between non-normally distributed variables were analyzed using the Mann–Whitney *U* test (i.e., Wilcoxon rank sum test). Chi-square or Fisher's exact tests were used to compare and analyze the statistical differences between the two groups of categorical variables. The sample size was determined by Shapiro–Wilk test. Pearson correlation analysis calculated the correlation coefficients between different gene sets. The survival package in R was used for survival analysis. The Kaplan–Meier survival curve was used to show the survival difference. The log-rank test was employed to determine the significance of different survival times between the two groups. Univariate and multivariate Cox analyses were used to determine independent prognostic factors. Statistical analysis and related graphing were carried out using GraphPad Prism (ver.8.0) application. A *p* value less than 0.05 was considered statistically significant.

## 3. Results

### 3.1. PANTR1 is Overexpressed in Grade II/III Gliomas

**Figure 1:**
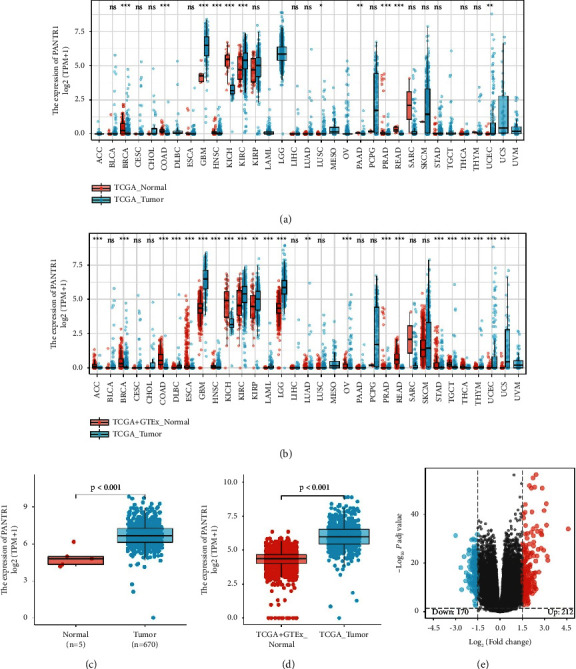
Differential expression of PANTR1. (a) The expression of PANTR1 was significantly higher in tumor (blue; TCGA) tissues than in NAT tissues (red; TCGA). We found that PANTR1 was significantly (*p* < 0.05) expressed in breast invasive carcinoma (BRCA), colon adenocarcinoma (COAD), glioblastoma multiforme (GBM), head and neck squamous cell carcinoma (HNSC), kidney chromophobe (KICH), kidney renal clear cell carcinoma (KIRC), lung squamous cell carcinoma (LUSC), pancreatic adenocarcinoma (PAAD), prostate adenocarcinoma (PRAD), rectum adenocarcinoma (READ), and uterine corpus endometrial carcinoma (UCEC). ns, *p* ≥ 0.05; ^*∗*^, *p* < 0.05; ^*∗∗*^, *p* < 0.01; ^*∗∗∗*^, and *p* < 0.001. (b) The expression of PANTR1 was significantly higher in tumor (blue; TCGA) tissues than in NAT tissues (red; TCGA and GTEx). We found that PANTR1 was significantly (*p* < 0.05) expressed in adrenocortical carcinoma (ACC), breast invasive carcinoma (BRCA), colon adenocarcinoma (COAD), diffuse large B-cell lymphoma (DLBC), esophageal carcinoma (ESCA), glioblastoma multiforme (GBM), head and neck squamous cell carcinoma (HNSC), kidney chromophobe (KICH), kidney renal clear cell carcinoma (KIRC), kidney renal papillary cell carcinoma (KIRP), acute myeloid leukemia (LAML), brain lower grade glioma (LGG), lung adenocarcinoma (LUAD), ovarian serous cystadenocarcinoma (OV), prostate adenocarcinoma (PRAD), rectum adenocarcinoma (READ), stomach adenocarcinoma (STAD), testicular germ cell tumors (TGCT), thyroid carcinoma (THCA), thymoma (THYM), uterine corpus endometrial carcinoma (UCEC), and uterine carcinosarcoma (UCS). ns, *p* ≥ 0.05; ^*∗*^, *p* < 0.05; ^*∗∗*^, *p* < 0.01; ^*∗∗∗*^, and *p* < 0.001. (c) The expression of PANTR1 was significantly higher in gliomas (blue; TCGA) tissues than in NAT tissues (red; TCGA) with a normal distribution. (d) The expression of PANTR1 was significantly higher in gliomas (blue; TCGA) tissues than in NAT tissues with a normal distribution (red; TCGA and GTEx). (e) Transcripts correlated with PANTR1 were identified and depicted in the volcano plot (red dots: up-regulated transcripts; blue dots: down-regulated transcripts). There were 212 up-regulated transcripts (|logFC| > 1.5 and padj < 0.05) and 170 down-regulated transcripts (|logFC| < −1.5 and padj < 0.05).

To elucidate whether PANTR1 is involved in glioma, we compared the expression of PANTR1 in normal adjacent to tumor (NAT) tissues and tumor tissues. The results showed that expression of PANTR1 was significantly increased in several tumor tissues including GBM (Figures 1(a) and 1(b)). Further analysis confirmed a significantly higher expression of PANTR1 in glioma tissues (Figures 1(c) and 1(d)). Reverse transcription-quantitative (RT-q) PCR showed that all the 15 glioma samples' PANTR1 expression outweighs normal adjacent tissues, whereas grade II and III glioma tend to have a higher expression rather than GBM compared with NAT (supplement 10). In the glioma samples of this study, 212 genes (red dots) were found to have closed correlations with PANTR1, while 170 genes (blue d-ots) were negatively associated. The top five genes substantially related to greater PANTR1 expression were EGFR, SEC61G, IGFBP2, and METTL7B. While low expression was related to the downregulation of CAMK2A, SYT1, VSNL1, and PACSIN1 (Figure 1(e)).

### 3.2. PANTR1 is Correlated with a Poor Prognosis

**Figure 2:**
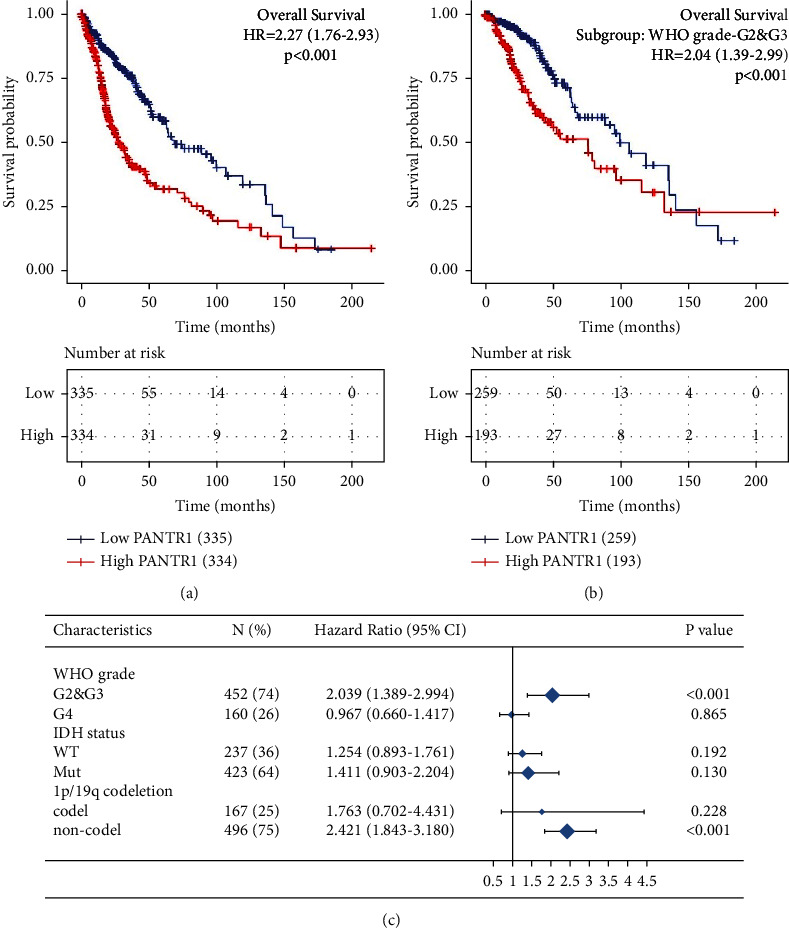
Prognosis analysis of PANTR1. (a) Using the survminer *R* package, a Kaplan–Meier survival plot was made to evaluate the prognostic value of PANTR1 in terms of overall survival. The lower portion was the risk table which recorded the number of censored observations. The prognostic data were derived from a study by Liu et al. [25]. (b) and (c) Subgroup analysis revealed PANTR1 as an important prognostic factor in patients with grade II/III gliomas.

To figure out whether PANTR1 affects glioma patients' prognosis, we used Kaplan–Meier survival analysis. Among other factors including WHO grade (*p* = 0.048), IDH status (*p* < 0.001), primary therapy outcome (*p* = 0.005), and age (*p* < 0.001), were independent prognostic factors in terms of overall survival (OS, age), progression-free survival, and disease-specific survival, respectively (Figure 2(a), Supplement Tables 7–9). Of note, high expression of PANTR1 was associated with poor OS (HR = 2.04, 95% CI: 1.39–2.99, *p* < 0.001) in patients with WHO grade II and III gliomas (Figure 2(b)). The result suggests that PANTR1 was correlated with a poor prognosis among glioma patients.

### 3.3. Genetic Functions of PANTR1

**Figure 3:**
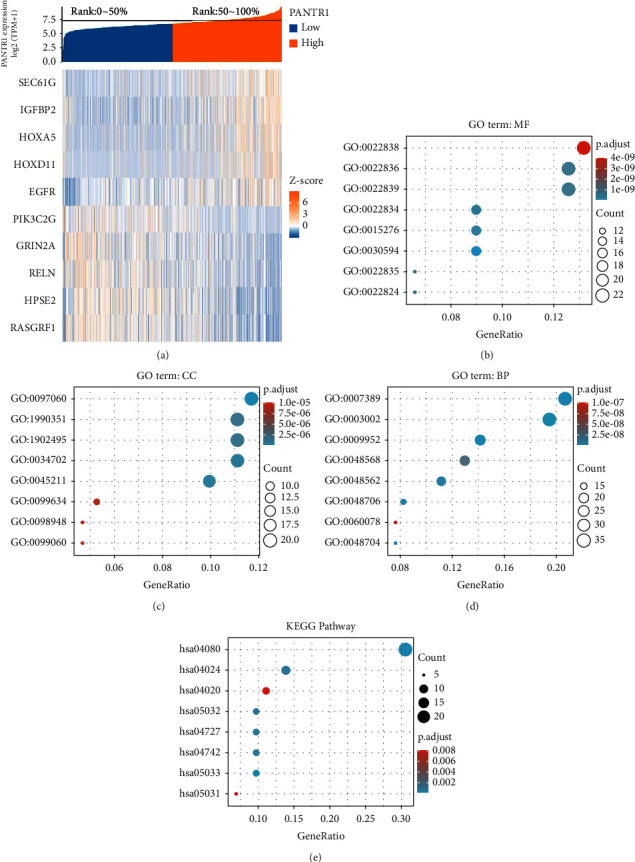
Gene set enrichment analysis (GSEA) of PANTR1. (a) Heat map depicting several genes up-regulated or down-regulated by PANTR1. (b)–(d) Gene ontology (GO) analysis revealed that PANTR1 was associated with several genes involved in molecular functions (MF), cell components (CC), and biological processes (BP). (e) KEGG analysis revealed PANTR1 involvement in several complex pathways.

To further explore the functions of PANTR1 up-regulated genes, we used Gene Ontology (GO) analysis. The result revealed that PANTR1 up-regulated genes which are expressed primarily in cell components including synaptic membrane and transporter complex, in biological processes including pattern-specific process and regionalization, and in molecular functions including substrate-specific channel activity and gated channel activity (Supplement Tables 1 and 2, Figures 3(a)–3(d)).

**Figure 4:**
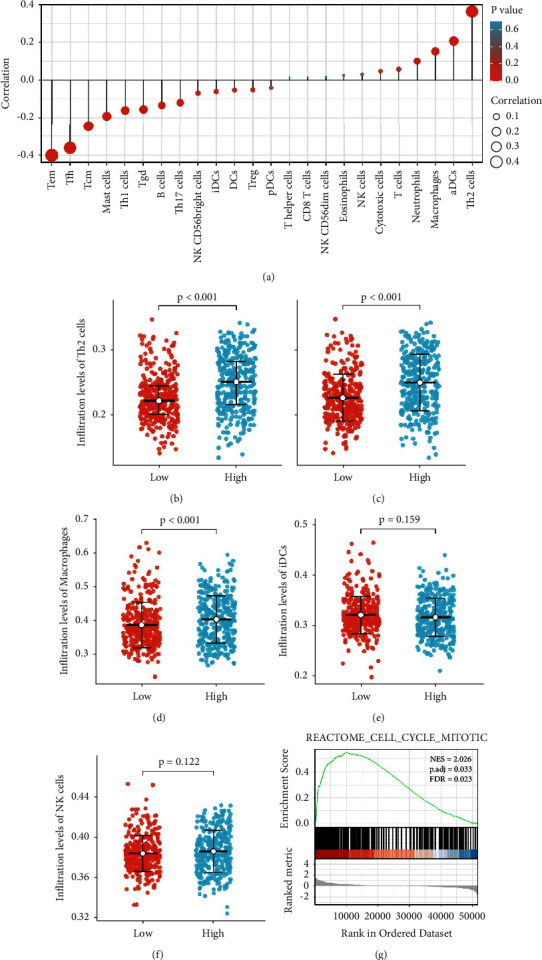
The association of PANTR1 and immune infiltrations. (a) The association of PANTR1 with infiltration levels of 24 different immune cells is shown in the plot. (b) and (c) Differences in infiltration levels of Th2 cells and aDCs were noted in different expression levels of PANTR1 in glioma cells. The results were of statistical significance (*p* < 0.001). (d) and (e) The expression of PANTR1 was not significantly associated with the infiltration levels of macrophages and iDCs. All data have a normal distribution here. (DCs, dendritic cells; aDCs, activated DCs; iDCs, immature DCs; Th, helper T cells; Treg, regulatory T cells; T regular; Tgd, T gamma delta; Tcm, T central memory; Tem, T effector memory; Tfh, T follicular helper). (g) Further KEGG analysis revealed positive PANTR1 correlation with the mitotic pathway. The reference gene set is h.all.v7.0.symbols.gmt [Hallmarks], REACTOME_CELL_CYCLE_MITOTIC, NES = 2.026, p.adj = 0.033, FDR = 0.023.

Kyoto Encyclopedia of Genes and Genomes (KEGG) pathway analysis showed enrichment of gene expression in neuroactive ligand-receptor interaction, cAMP signaling pathway, and calcium signaling pathway (Figure 2(e), Supplement Table 3). Based on these results, further KEGG analysis of GBM/LGG samples revealed a significant correlation of PANTR1 expression with pathways including mitosis, M-phase, DNA repair, and translation (Supplement Table 3, Figure 4(h)).

Next, immune signatures of GBM/LGG were investigated. Marker genes of 24 immune cells were derived from a study by Bindea et al. [226]. A high expression level of PANTR1 was found to be positively correlated with infiltration levels of Th2 cells, aDCs, and macrophages. Of note, the expression of PANTR1 was not associated with the infiltration of iDCs and NK cells (Figures 4(a)–4(f)).

**Figure 5:**
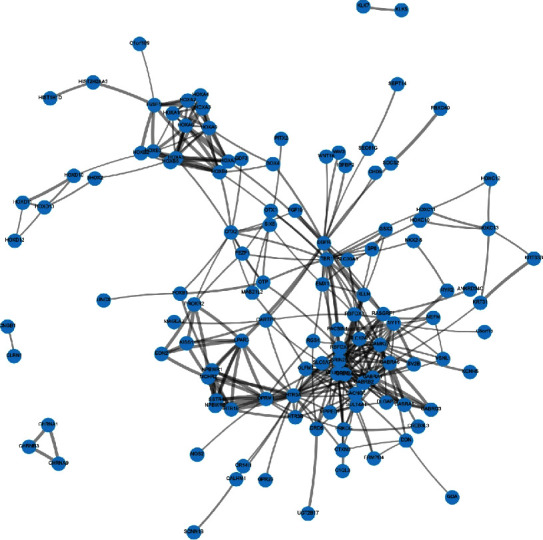
A complex protein-protein interaction network was derived from the STRING database (https://string-db.org) [27].

We also performed an analysis of protein-protein interaction associated with PANTR1, which indicated the involvement of several proteins including EGFR, GRIN2A, HOXA6, and RASGRF1 (Supplement Table 4, Figure 5).

### 3.4. PANTR1 is Associated with Cell Viability, Migration, and Cell Death

**Figure 6:**
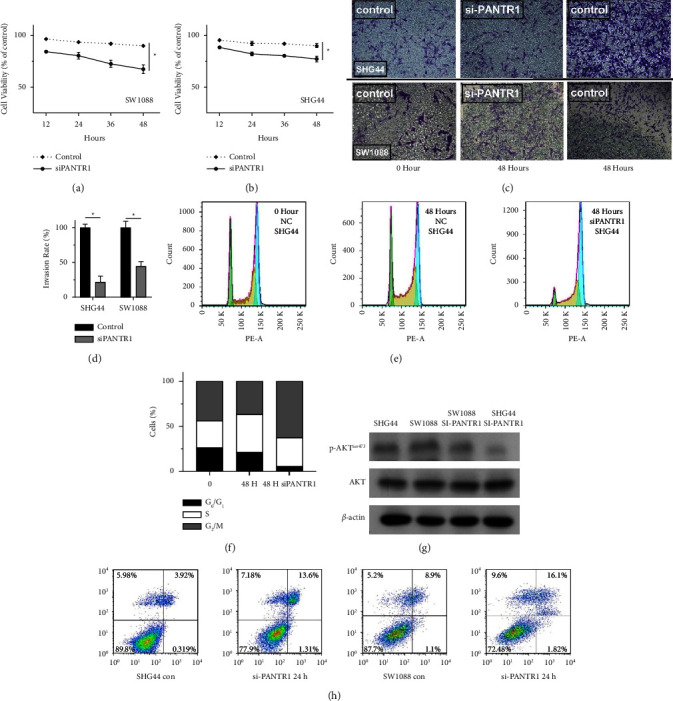
(a) si-RNA knockdown of PANTR1 led to decreased cell viability of SW1088 (WHO grade II) cell line. (b) si-RNA knockdown of PANTR1 leads to the decreased cell of the SHG44 (WHO grade IV) cell line. (c) and (d) Decreased invasiveness of both (SW1088 and SGH 44) cell lines compared with control was seen after PANTR1 knockdown. (e) and (f) Knockdown of PANTR1 contributed to an increased ratio of G2/M phase glioma cells and a decreased ratio of G0/G1 and S glioma cells after 48 hours. (g) The WB results show the reduced p-Akt activity in PANTR1 knockdown glioma cell lines, and p-Akt and Akt were compared to the same internal control (*β*-actin). (h) Representative results of the effects of control (con) and si-PANTR1 combinations in SHG44 and sw1088 cells are shown.

**Figure 7:**
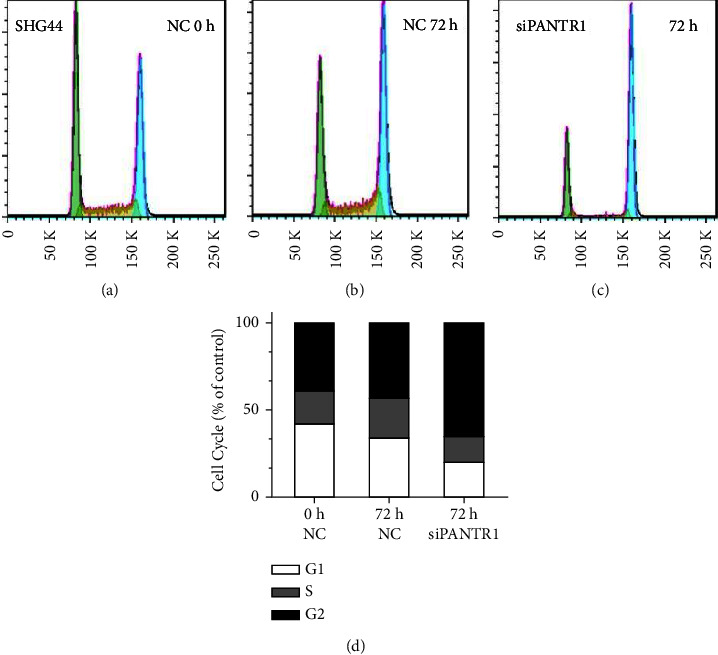
(a)–(c) Knockdown of PANTR1 showed an observable decreased S and G1 phase glioma cells and increased G2 phase glioma cells after 72 hours. (d) Histograms showing the percentage of different cell cycle phases are shown.

To detect the effects of PANTR1 on gliomas, we used Transwell and transfection assay; it was found that knockdown of PANTR1 was associated with attenuated cell viability and invasiveness (Figures 6(a)–6(d)). Moreover, an increased ratio of G2/M phase and a reduction of S and G0/G1 phase glioma cells were observed in PANTR1 knockdown group compared with normal glioma cells after 48 and 72 hours (Figures 6(e), 6(f), and 7(a)–7(d)), which indicated that the SHG44 cell cycle was blocked in G2 phase.

**Figure 8:**
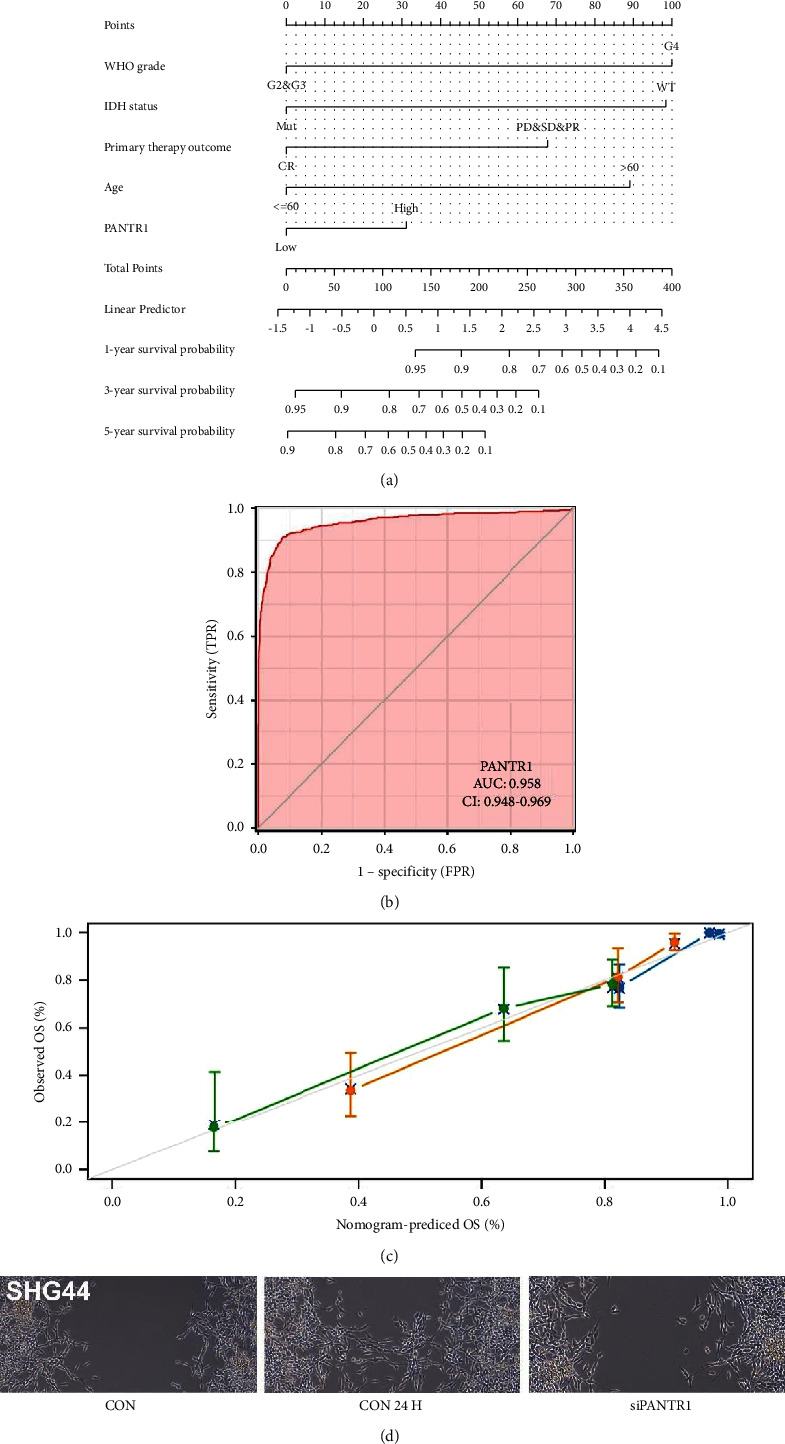
(a) We established a nomogram for glioma prognosis. Included in the parameters are WHO grade, IDH status, primary therapy outcome, and PANTR1. (b) ROC curve revealed that this nomogram was a reliable tool for predicting glioma prognosis. (c) Calibration curves for 1-year (blue), 3-year (red), and 5-year (green) outcomes were depicted. (d) A wound healing assay, as described in the “materials and methods” is shown.

Moreover, according to the result of the wound-healing assay, cells transfected with SiPANTR1 exhibited a perceptible migratory ability decrease after 24 hours compared with control cells (Figure 8(d)). According to the result of the correlation analysis presented by the volcano plot, one of the top five genes substantially related to the high expression of PANTR1 was EGFR (Figure 1(e)). The PI3K/Akt signaling pathway was the major molecular mechanism of EGFR-induced tumorigenic transformation [28]. To further investigate the potential functions of PANTR1 in cell death, we used a western blot assay to detect the PI3K/Akt signaling pathway. The result showed that Ser^473^ phosphorylated Akt1 expression by the western blot was markedly reduced in PANTR1 knockdown glioma cells, which plays an important role in cell death (Figure 6(g)). Annexin V/propidium iodide assay also confirmed that in the two cell lines, apoptosis was enhanced after silencing, especially the proportion of late apoptosis and death was increased (Figure 6(h)).

### 3.5. Construction of a Nomogram

We found that PANTR1 expression was significantly related to several clinical parameters of glioma prognosis, including WHO grade (*p* < 0.001), IDH status (*p* < 0.001), primary therapy outcome (*p*=0.02), gender (*p*=0.023), histological type (*p* < 0.001), and EGFR status (*p* < 0.001) (*t*-test for each group). PANTR1 expression was also significantly (*p* < 0.001) correlated with age (Wilcoxon rank sum test) (Supplement Tables 5–9).

Based on this, a nomogram with variables including WHO grade, IDH status, primary therapy outcome, age, and PANTR1 was constructed (Figure 8(a)). The receiver operating characteristic (ROC) curve of the nomogram indicated that PANTR1 could serve as a potential diagnostic parameter for gliomas (AUC of 0.958) (Figure 8(b)). Calibration curves for 1-year, 3-year, and 5-year outcomes were depicted in Figure 8(c).

## 4. Discussion

In recent years, an accumulating body of research revealed the regulatory role of lncRNAs in tumor treatment. Several lncRNAs were proven to be related to the resistance against glioma treatments, including immunotherapy, chemotherapy, and radiotherapy [11]. Furthermore, Zhang et al. established a lncRNAs signature based on the 16 most potent tumor-infiltrating immune cell-associated lncRNAs, which could predict the superior responses of immunotherapy in multiple datasets across cancer types [29]. Wu et al. reported an immune-related lncRNAs signature that could predict the therapeutic efficacy of low-grade glioma treatments [30]. In the current study, we first found the mRNA level of PANTR1 increased in glioma samples by a public dataset mining and verified by (RT-q) PCR of collected glioma samples. The Kaplan–Meier survival analysis showed that PANTR1 was correlated with a poor prognosis. The in-vitro study showed that the knockdown of PANTR1 caused significantly reduced glioma cells viability. In addition, according to the western blot and Annexin V/propidium iodide assay, PANTR1 is highly correlated with the upregulation of the phosphorylation of AKT1 at S473, which promotes cell survival by inhibiting apoptosis via phosphorylation and inactivation of several targets including Bad, Foxo1, c-Raf, and caspase-9. Hence, PANTR1 might be an oncogene in glioma. In addition, wound-healing assay that showed a migratory capacity decline of Si-PANTR1 transfected cells suggested PANTR1 is associated with tumor invasiveness and metastasis. Both genetic and epigenetic mechanisms play important roles in tumorigenesis.

PANTR1 is a nonprotein-coding transcript with four 4KB variants upstream of the POU3F3 gene on chromosome 2q12.1. Several studies have revealed that PANTR1 plays a vital function in the development of the human neurological and urinary systems [20, 31, 32]. At the epigenetic level, PANTR1 has been proven to be involved in the regulation of the expression of Delta1 and Sox2, both of which are key genes in the neural differentiation of stem cells [33]. Clinical presentations of POU3F3 mutations vary from cognitive impairment and malformation of several organs [34]. Meanwhile, PANTR1 played an important role in tumorigenesis. Some suggested that the combination of PANTR1 with other lncRNAs may identify the early progression of clear-cell renal cell cancer, breast cancer, and cervical cancer [21, 35, 36]. It was also demonstrated that the CpG island near the PANTR1 gene is highly methylated and that its transcriptional activity is generally inhibited in the process of tumor progression [37].

Several studies indicated PANTR1 is a driver gene of different types of cancers including cervical cancer, clear-cell renal cell cancer, esophageal squamous cell carcinoma, gastric cancer, and breast cancer, as PANTR1 can promote angiogenesis, cell proliferation, migration, and invasion, and inhibit angiogenesis cell-cell death [21, 35, 36, 38, 39].

Previous studies demonstrated that Linc-POU3F3 (PANTR1) could promote tumor cell invasion in hepatocellular carcinoma and colorectal cancer [40, 41]. In the present study, cell invasion assays showed decreased invasiveness in both SW1088 and SGH44 glioma cell lines after PANTR1 knockdown, which were consistent with the results in other tumor types.

After PANTR1 knockdown, we observed different degrees of cell growth and cell death in cell lines, which was more pronounced in low-grade glioma cells. To elucidate the cell behavior pattern observed by cell viability inhibition, we studied the apoptotic activity of PANTR1 knockdown cells.

In the present study, PANTR1 was found to be significantly up-regulated in glioma tissues. Kaplan–Meier survival analysis showed high PANTR1 expressed gliomas indicate poor prognosis. Bioinformatic techniques play a critical role in this study. At the epigenetic level, we demonstrated the upregulation of several genes including EGFR, SEC61G, IGFBP2, and METTL7B by PANTR1. Pathway analysis by KEGG revealed the regulatory role of PANTR1 in mitosis, M-phase, and translation. In terms of the immune microenvironment, we found that high expression of PANTR1 may be negatively associated with the infiltration of iDCs and NK cells. In addition, a major inhibition of cell viability (G2 phase blockage in the cell cycle), migration, and invasiveness was found in PANTR1 knock-out glioma cells. Meanwhile, the result of WB indicated that PI3K-Akt pathway signaling might be involved in glioma progression regulated by PANTR1. The PI3K/Akt signaling pathway was deactivated in glioma cells. Akt promotes cell survival by inhibiting cell death through phosphorylation and inactivation of several targets. Thus, we hypothesize that PANTRI may inhibit cell death by activating the PI3K/Akt signaling pathway. However, other pathways might also be affected by PANTR1. Ma et al. demonstrated that PANTR1 could promote tumorigenesis of hepatocellular carcinoma through the miR-587/BCL2A1 axis [42]. Seles et al. reported that PANTR1 could regulate the expression of VEGF-A and LAMC2 in clear-cell renal cell cancer [21]. There are many apoptosis-related pathways, such as PERK and caspase 7 pathways [41, 42]. Future experiments could be carried out to validate the role of these pathways in glioma progression.

Collectively, the nomogram we constructed proved that PANTR1 could serve as a potential biomarker of glioma prognosis, as high expression of PANTR1 may confer a variety of adverse events at the molecular level, especially in those with grade II/III gliomas. The role of lncRNAs in tumorigenesis is receiving increasing recognition, our study provides insight into future therapeutic targets for gliomas, and we believe that further studies are still needed to further establish the role of PANTR1.

## Data Availability

Publicly available datasets were analyzed in this study. This data can be found here: the datasets analyzed for this study can be found on the TCGA GDC official website (https://portal.gdc.cancer.gov/) and Genomics of Drug Sensitivity in Cancer (GDSC; https://www.cancerrxgene.org/)

## Supplementary Materials

Supplementary Materials Table 1: Differential expression analysis of PANTR1 in GBM/LGG. Table 2: Gene ontology enrichment analysis of PANTR1 using the clusterProfiler package. Table 3: Pathway enrichment analysis of PANTR1. Table 4: Protein-protein interaction network of PANTR1. Table 5: The association of PANTR1 expression level with clinical parameters of gliomas using the Chi-squared test or Fisher's exact test for analysis. Student's *t*-test or Wilcoxon rank sum test revealed that age was significantly (*p* < 0.001) associated with PANTR1 expression. Table 6: The association of PANTR1 expression level with pathological parameters of gliomas using logistics regression. PANTR1 expression was significantly correlated with these variables including WHO grade (*p* < 0.001), IDH status (*p* < 0.001), primary therapy outcome (*p* = 0.016), and EGFR status (*p* < 0.001). Table 7: Uni- and multivariate Cox regression analysis showed the prognostic value of PANTR1 in overall survival. We observed IDH status (*p* < 0.001), primary therapy outcome (*p* < 0.001), age (*p* = 0.022), and PANTR1 (*p* = 0.045) are independent prognostic factors in progression-free interval (*p* < 0.05) of gliomas. Table 8: Uni- and multivariate Cox regression analysis showed the prognostic value of PANTR1 in progression-free survival. Table 9: Uni- and multivariate Cox regression analysis showed the prognostic value of PANTR1 in disease-specific survival. Supplement 10: Relative PANTR1 expression. PCR showed that all 15 glioma samples' PANTR1 expression outweighs normal adjacent tissues, whereas grade II and III glioma tend to have a higher expression rather than GBM compared with NAT.
