# Supplementary material for: LncRNA PANTR1 is Associated with Poor Prognostic and Suppresses Apoptosis in Glioma
Source: J Oncol. 2023 Feb 20;2023:8537036. doi: 10.1155/2023/8537036 (PMC9970703; doi:10.1155/2023/8537036)
Supplement: Supplementary Materials — Table 1: Differential expression analysis of PANTR1 in GBM/LGG. Table 2: Gene ontology enrichment analysis of PANTR1 using the clusterProfiler package. Table 3: Pathway enrichment analysis of PANTR1. Table 4: Protein-protein interaction network of PANTR1. Table 5: The association of PANTR1 expression level with clinical parameters of gliomas using the Chi-squared test or Fisher's exact test for analysis. Student's t-test or Wilcoxon rank sum test revealed that age was significantly (p < 0.001) associated with PANTR1 expression. Table 6: The association of PANTR1 expression level with pathological parameters of gliomas using logistics regression. PANTR1 expression was significantly correlated with these variables including WHO grade (p < 0.001), IDH status (p < 0.001), primary therapy outcome (p = 0.016), and EGFR status (p < 0.001). Table 7: Uni- and multivariate Cox regression analysis showed the prognostic value of PANTR1 in overall survival. We observed IDH status (p < 0.001), primary therapy outcome (p < 0.001), age (p = 0.022), and PANTR1 (p = 0.045) are independent prognostic factors in progression-free interval (p < 0.05) of gliomas. Table 8: Uni- and multivariate Cox regression analysis showed the prognostic value of PANTR1 in progression-free survival. Table 9: Uni- and multivariate Cox regression analysis showed the prognostic value of PANTR1 in disease-specific survival. Supplement 10: Relative PANTR1 expression. PCR showed that all 15 glioma samples' PANTR1 expression outweighs normal adjacent tissues, whereas grade II and III glioma tend to have a higher expression rather than GBM compared with NAT. [file 8537036.f1.zip › Supplement table 2.pdf]

ONTOLOC	ID	Descriptio	GeneRatic	BgRatio	pvalue	p.adjust	qvalue	geneID
BP	GO:00030	regionaliz	33/169	351/1867(	3.35E-24	7.70E-21	6.92E-21	RELN/MEC
BP	GO:00073	pattern sp	35/169	446/1867(	5.10E-23	5.87E-20	5.28E-20	RELN/MEC
BP	GO:00099	anterior/p	24/169	219/1867(	2.49E-19	1.91E-16	1.72E-16	MEOX2/H
BP	GO:00487	embryonic	13/169	93/18670	2.38E-12	1.37E-09	1.23E-09	HOXB3/SH
BP	GO:00487	embryonic	14/169	126/1867(	8.25E-12	3.80E-09	3.41E-09	HOXB3/SH
BP	GO:00485	embryonic	19/169	288/1867(	1.61E-11	6.16E-09	5.53E-09	RYR2/HOX
BP	GO:00485	embryonic	22/169	428/1867(	4.91E-11	1.61E-08	1.45E-08	EGFR/RYR
BP	GO:00600	regulation	13/169	140/1867(	4.57E-10	1.31E-07	1.18E-07	RGS4/GRII
BP	GO:00487	skeletal sy	15/169	239/1867(	4.82E-09	1.23E-06	1.11E-06	HOXB3/SH
BP	GO:00487	appendag	13/169	179/1867(	9.35E-09	1.85E-06	1.66E-06	MEOX2/SH
BP	GO:00601	limb devel	13/169	179/1867(	9.35E-09	1.85E-06	1.66E-06	MEOX2/SH
BP	GO:00099	proximal/c	7/169	31/18670	9.63E-09	1.85E-06	1.66E-06	HOXA10/H
BP	GO:00309	forebrain (	18/169	381/1867(	1.18E-08	2.08E-06	1.87E-06	EGFR/CHD
BP	GO:00303	embryonic	10/169	125/1867(	2.05E-07	3.14E-05	2.82E-05	SHOX2/HC
BP	GO:00351	embryonic	10/169	125/1867(	2.05E-07	3.14E-05	2.82E-05	SHOX2/HC
BP	GO:00423	regulation	17/169	434/1867(	4.45E-07	6.40E-05	5.75E-05	RGS4/GRII
BP	GO:00351	appendag	10/169	148/1867(	9.84E-07	0.000126	0.000113	SHOX2/HC
BP	GO:00351	limb morp	10/169	148/1867(	9.84E-07	0.000126	0.000113	SHOX2/HC
BP	GO:00072	gamma-a	5/169	25/18670	2.63E-06	0.000318	0.000286	GABRA1/C
BP	GO:00215	telenceph	12/169	251/1867(	3.16E-06	0.000363	0.000326	EGFR/RELI
BP	GO:00600	excitatory	8/169	100/1867(	3.50E-06	0.000384	0.000345	RGS4/GRII
BP	GO:00076	learning o	12/169	256/1867(	3.87E-06	0.000404	0.000364	EGFR/SLC
BP	GO:00215	spinal corc	8/169	106/1867(	5.42E-06	0.000542	0.000487	RELN/HOX
BP	GO:00995	chemical s	8/169	108/1867(	6.23E-06	0.000597	0.000537	RGS4/GRII
BP	GO:00486	neuron fat	5/169	34/18670	1.29E-05	0.001186	0.001066	DMRTA2/I
BP	GO:00508	cognition	12/169	296/1867(	1.68E-05	0.001466	0.001317	EGFR/SLC
BP	GO:20003	regulation	5/169	36/18670	1.72E-05	0.001466	0.001317	RASGRF1/
BP	GO:19004	regulation	6/169	64/18670	2.44E-05	0.002008	0.001805	RASGRF1/
BP	GO:00486	neuron fat	6/169	65/18670	2.67E-05	0.00212	0.001905	TBR1/DMF
BP	GO:00451	cell fate cc	11/169	270/1867(	3.63E-05	0.002784	0.002503	CHD5/TBF
BP	GO:00308	thyroid gl	4/169	22/18670	4.17E-05	0.003079	0.002767	HOXB3/HC
BP	GO:00219	central ne	9/169	183/1867(	4.46E-05	0.003079	0.002767	CHD5/TBF
BP	GO:00072	neuropept	7/169	104/1867(	4.47E-05	0.003079	0.002767	CARTPT/N
BP	GO:00991	regulation	14/169	437/1867(	4.55E-05	0.003079	0.002767	EGFR/CAN
BP	GO:00519	synaptic tr	5/169	45/18670	5.22E-05	0.003394	0.003051	GABRA1/C
BP	GO:00076	learning	8/169	145/1867(	5.31E-05	0.003394	0.003051	SLC12A5/C
BP	GO:00996	regulation	6/169	74/18670	5.60E-05	0.003484	0.003132	RASGRF1/
BP	GO:00218	forebrain r	4/169	24/18670	5.98E-05	0.00356	0.0032	DMRTA2/I
BP	GO:00217	limbic syst	7/169	109/1867(	6.04E-05	0.00356	0.0032	RELN/TBR
BP	GO:00321	regulation	4/169	25/18670	7.07E-05	0.003963	0.003562	PRKCG/CY
BP	GO:00321	regulation	4/169	25/18670	7.07E-05	0.003963	0.003562	PRKCG/CY
BP	GO:00083	associative	6/169	78/18670	7.54E-05	0.004131	0.003713	GRIN2A/R
BP	GO:00216	nerve dev	6/169	79/18670	8.10E-05	0.004334	0.003895	GABRB2/F
BP	GO:00905	sensory or	10/169	256/1867(	0.000117	0.006107	0.005489	HCN1/OLI
BP	GO:00347	regulation	14/169	483/1867(	0.000132	0.00673	0.006049	RGS4/RAS
BP	GO:19024	chloride tr	6/169	88/18670	0.000148	0.007233	0.006502	SLC12A5/C
BP	GO:00508	positive re	8/169	168/1867(	0.000149	0.007233	0.006502	EGFR/SYT
BP	GO:00380	opioid rec	3/169	12/18670	0.000151	0.007233	0.006502	OPRM1/N
BP	GO:00099	dorsal/ver	6/169	90/18670	0.000167	0.007692	0.006914	HOXB2/EN
BP	GO:00309	midbrain (	6/169	90/18670	0.000167	0.007692	0.006914	OTX1/EN1
BP	GO:00508	modulatio	13/169	436/1867(	0.000174	0.007735	0.006953	EGFR/CAN
BP	GO:00215	pallium de	8/169	172/1867(	0.000175	0.007735	0.006953	EGFR/RELI
BP	GO:00215	spinal corc	3/169	13/18670	0.000195	0.008303	0.007463	HOXC10/H
BP	GO:00219	telenceph	3/169	13/18670	0.000195	0.008303	0.007463	DMRTA2/I
BP	GO:20012	regulation	8/169	178/1867(	0.000221	0.009244	0.008309	RASGRF1/
BP	GO:00017	cell fate sp	6/169	99/18670	0.000281	0.01156	0.010391	TBR1/DMF
BP	GO:00072	glutamate	6/169	100/1867(	0.000297	0.011993	0.01078	RASGRF1/
BP	GO:00085	epidermis	13/169	464/1867(	0.000319	0.012633	0.011355	EGFR/FAB

BP	GO:00507	positive re	13/169	474/1867(	0.00039	0.015213	0.013674	SYT1/PAC
BP	GO:00068	chloride tr	6/169	108/1867(	0.00045	0.017235	0.015492	SLC12A5/(
BP	GO:00458	positive re	4/169	40/18670	0.000459	0.017289	0.01554	RGS4/RYR
BP	GO:00708	divalent m	13/169	483/1867(	0.000466	0.017289	0.01554	CAMK2A/I
BP	GO:00309	hindbrain	7/169	152/1867(	0.000474	0.017289	0.01554	TBR1/HOX
BP	GO:00351	forelimb n	4/169	41/18670	0.000505	0.017677	0.015889	SHOX2/EN
BP	GO:00986	inorganic	6/169	111/1867(	0.00052	0.017677	0.015889	SLC12A5/(
BP	GO:00725	divalent in	13/169	489/1867(	0.000523	0.017677	0.015889	CAMK2A/I
BP	GO:00618	antimicrob	5/169	73/18670	0.000524	0.017677	0.015889	KLK7/KLK5
BP	GO:19035	positive re	5/169	73/18670	0.000524	0.017677	0.015889	EGFR/RGS
BP	GO:00607	mammary	3/169	18/18670	0.000538	0.017677	0.015889	SOCS2/HO
BP	GO:00613	mammary	3/169	18/18670	0.000538	0.017677	0.015889	SOCS2/HO
BP	GO:00068	calcium io	12/169	434/1867(	0.000609	0.019716	0.017721	CAMK2A/I
BP	GO:00324	regulation	9/169	260/1867(	0.000619	0.01976	0.017761	RASGRF1/
BP	GO:00320	regulation	3/169	19/18670	0.000635	0.019995	0.017973	PRKCG/CA
BP	GO:00602	definitive I	3/169	20/18670	0.000742	0.023052	0.02072	HOXB3/HO
BP	GO:00228	regulation	9/169	268/1867(	0.000767	0.023536	0.021156	RASGRF1/
BP	GO:00156	inorganic	7/169	169/1867(	0.000888	0.026871	0.024153	SLC12A5/(
BP	GO:00215	ventral spi	4/169	48/18670	0.000924	0.02761	0.024817	RELN/HOX
BP	GO:00215	dorsal spir	3/169	22/18670	0.000989	0.029153	0.026205	GSX2/HOX
BP	GO:00352	endocrine	6/169	127/1867(	0.001055	0.030718	0.027611	HOXB3/HO
BP	GO:00324	regulation	9/169	283/1867(	0.001125	0.032351	0.029079	RASGRF1/
BP	GO:19040	regulation	10/169	342/1867(	0.00114	0.032384	0.029109	RGS4/RAS
BP	GO:00986	anion tran	9/169	288/1867(	0.001271	0.035645	0.032039	RGS4/SLC
BP	GO:00218	forebrain r	4/169	53/18670	0.001343	0.037219	0.033455	TBR1/OTP
BP	GO:00456	negative r	5/169	91/18670	0.001423	0.038649	0.03474	HOXA5/HO
BP	GO:00215	cell differe	4/169	54/18670	0.00144	0.038649	0.03474	HOXC10/H
BP	GO:00603	response t	6/169	135/1867(	0.001445	0.038649	0.03474	RGS4/GAB
BP	GO:00104	positive re	3/169	26/18670	0.001625	0.042972	0.038626	RGS4/RYR
BP	GO:00603	innervatio	3/169	27/18670	0.001817	0.047479	0.042677	GABRB2/P
BP	GO:00487	gland dev	11/169	434/1867(	0.002043	0.052792	0.047452	EGFR/SOC
BP	GO:00076	sensory pe	6/169	145/1867(	0.002077	0.053082	0.047713	GABRB2/G
BP	GO:00435	negative r	6/169	147/1867(	0.002225	0.054796	0.049253	GABRB2/P
BP	GO:00199	second-m	11/169	439/1867(	0.002233	0.054796	0.049253	EGFR/GRIN
BP	GO:00030	respiratory	3/169	29/18670	0.002239	0.054796	0.049253	HOXA5/GS
BP	GO:00216	cranial ner	3/169	29/18670	0.002239	0.054796	0.049253	HOXB3/HO
BP	GO:00217	olfactory b	3/169	30/18670	0.002472	0.059846	0.053793	GSX2/FEZI
BP	GO:00219	olfactory l	3/169	31/18670	0.002719	0.065135	0.058547	GSX2/FEZI
BP	GO:00509	neuromus	5/169	107/1867(	0.002897	0.068694	0.061746	GRIN2A/C
BP	GO:00019	response t	3/169	32/18670	0.00298	0.069236	0.062233	RGS4/GRII
BP	GO:00519	positive re	3/169	32/18670	0.00298	0.069236	0.062233	EGFR/RELI
BP	GO:00218	forebrain (	4/169	66/18670	0.003019	0.069426	0.062404	TBR1/OTP
BP	GO:00140	response t	3/169	33/18670	0.003257	0.071334	0.064119	RGS4/PRK
BP	GO:00351	embryonic	3/169	33/18670	0.003257	0.071334	0.064119	SHOX2/EN
BP	GO:00432	response t	3/169	33/18670	0.003257	0.071334	0.064119	RGS4/PRK
BP	GO:00485	spleen dev	3/169	33/18670	0.003257	0.071334	0.064119	NKX2-5/H
BP	GO:00488	brain mor	3/169	33/18670	0.003257	0.071334	0.064119	OTX1/EMX
BP	GO:00075	skeletal m	6/169	160/1867(	0.003391	0.072865	0.065495	MEOX2/D
BP	GO:00069	hypotonic	2/169	10/18670	0.003495	0.072865	0.065495	SLC12A5/
BP	GO:00071	adenylate	2/169	10/18670	0.003495	0.072865	0.065495	DRD5/OPI
BP	GO:00900	primitive s	2/169	10/18670	0.003495	0.072865	0.065495	OTX2/GDI
BP	GO:19004	positive re	2/169	10/18670	0.003495	0.072865	0.065495	KLK7/KLK5
BP	GO:00432	response t	5/169	112/1867(	0.003526	0.072865	0.065495	RGS4/PRK
BP	GO:00215	spinal cor	3/169	34/18670	0.003548	0.072865	0.065495	HOXC10/H
BP	GO:00432	positive re	8/169	275/1867(	0.003638	0.07404	0.066552	CAMK2A/I
BP	GO:00519	regulation	4/169	70/18670	0.003734	0.075345	0.067724	EGFR/SYT
BP	GO:00509	sensory pe	6/169	165/1867(	0.003942	0.078844	0.070869	GABRB2/G
BP	GO:00219	cerebral c	5/169	116/1867(	0.004096	0.080047	0.071951	EGFR/RELI
BP	GO:00192	transmissi	4/169	72/18670	0.004132	0.080047	0.071951	CACNG3/(

BP	GO:00075	muscle or[illegible]	10/169	410/1867[illegible]	0.004242	0.080047	0.071951	RGS4/RYR
BP	GO:00347	response t	2/169	11/18670	0.004246	0.080047	0.071951	GABRA1/[illegible]
BP	GO:00488	forebrain [illegible]	2/169	11/18670	0.004246	0.080047	0.071951	OTX1/GS[illegible]
BP	GO:00602	embryonic	2/169	11/18670	0.004246	0.080047	0.071951	SHOX2/H[illegible]
BP	GO:01060	negative r	2/169	11/18670	0.004246	0.080047	0.071951	SSTR4/OP
BP	GO:00605	skeletal m	6/169	169/1867[illegible]	0.004429	0.082824	0.074447	MEOX2/D
BP	GO:00715	dopamine	3/169	37/18670	0.004517	0.08378	0.075306	DMRTA2/[illegible]
BP	GO:00215	diencepha	4/169	75/18670	0.004781	0.087971	0.079073	OTX1/OT[illegible]
BP	GO:00320	response t	3/169	38/18670	0.004872	0.088929	0.079934	PRKCG/C[illegible]
BP	GO:00104	regulation	6/169	173/1867[illegible]	0.004959	0.089711	0.080637	RASGRF1/
BP	GO:00218	olfactory [illegible]	2/169	12/18670	0.005065	0.089711	0.080637	GSX2/UN[illegible]
BP	GO:19048	inhibitory	2/169	12/18670	0.005065	0.089711	0.080637	GABRA1/[illegible]
BP	GO:00197	antimicro[illegible]	5/169	122/1867[illegible]	0.005071	0.089711	0.080637	KLK7/KLK[illegible]
BP	GO:00072	serotonin	3/169	39/18670	0.005243	0.092053	0.082743	HTR5A/HT
BP	GO:00099	epidermal	9/169	358/1867[illegible]	0.005414	0.094317	0.084778	CYP27B1/[illegible]
BP	GO:00723	response t	4/169	78/18670	0.005495	0.094317	0.084778	RGS4/PRK
BP	GO:20003	regulation	4/169	78/18670	0.005495	0.094317	0.084778	CAMK2A/[illegible]
BP	GO:00454	response t	5/169	125/1867[illegible]	0.005615	0.095658	0.085982	RGS4/GRI[illegible]
BP	GO:00016	eye devel[illegible]	9/169	362/1867[illegible]	0.00581	0.098263	0.088324	EGFR/HCN
BP	GO:00055	detection	2/169	13/18670	0.00595	0.099898	0.089794	SYT1/RYR[illegible]
CC	GO:00347	ion chann[illegible]	19/171	301/1971[illegible]	1.69E-11	3.90E-09	3.28E-09	GRIN2A/G
CC	GO:19024	transmem	19/171	324/1971[illegible]	6.03E-11	6.94E-09	5.84E-09	GRIN2A/G
CC	GO:19903	transporte	19/171	332/1971[illegible]	9.16E-11	7.02E-09	5.91E-09	GRIN2A/G
CC	GO:00970	synaptic m	20/171	432/1971[illegible]	1.19E-09	6.84E-08	5.76E-08	CAMK2A/[illegible]
CC	GO:00452	postsynap	17/171	323/1971[illegible]	3.35E-09	1.54E-07	1.30E-07	CAMK2A/[illegible]
CC	GO:00996	postsynap	9/171	101/1971[illegible]	2.37E-07	8.27E-06	6.96E-06	GRIN2A/G
CC	GO:00990	integral c[illegible]	8/171	74/19717	2.52E-07	8.27E-06	6.96E-06	GRIN2A/G
CC	GO:00989	intrinsic c[illegible]	8/171	77/19717	3.44E-07	9.88E-06	8.32E-06	GRIN2A/G
CC	GO:19027	GABA-A r[illegible]	5/171	19/19717	4.88E-07	1.25E-05	1.05E-05	GABRA1/[illegible]
CC	GO:19027	GABA rec[illegible]	5/171	20/19717	6.46E-07	1.48E-05	1.25E-05	GABRA1/[illegible]
CC	GO:00990	integral c[illegible]	9/171	117/1971[illegible]	8.33E-07	1.65E-05	1.39E-05	GRIN2A/G
CC	GO:00996	integral c[illegible]	10/171	152/1971[illegible]	8.60E-07	1.65E-05	1.39E-05	GRIN2A/G
CC	GO:00325	dendrite n	6/171	40/19717	1.17E-06	1.95E-05	1.64E-05	DDN/GAB
CC	GO:00989	intrinsic c[illegible]	9/171	122/1971[illegible]	1.18E-06	1.95E-05	1.64E-05	GRIN2A/G
CC	GO:00992	intrinsic c[illegible]	10/171	164/1971[illegible]	1.72E-06	2.64E-05	2.22E-05	GRIN2A/G
CC	GO:00995	postsynap	14/171	348/1971[illegible]	2.19E-06	3.15E-05	2.65E-05	CAMK2A/[illegible]
CC	GO:00347	cation cha	11/171	220/1971[illegible]	3.60E-06	4.87E-05	4.10E-05	GRIN2A/G
CC	GO:00325	neuron pr[illegible]	6/171	57/19717	9.81E-06	0.000125	0.000106	DDN/GAB
CC	GO:00347	chloride c[illegible]	5/171	50/19717	7.15E-05	0.000866	0.000729	GABRA1/[illegible]
CC	GO:00988	neurotran[illegible]	5/171	53/19717	9.48E-05	0.001091	0.000918	GRIN2A/G
CC	GO:00058	acetylchol	3/171	17/19717	0.000399	0.004365	0.003676	CHRNA1/[illegible]
CC	GO:00312	leading e[illegible]	7/171	170/1971[illegible]	0.000718	0.007505	0.00632	PACSIN1/[illegible]
CC	GO:00989	neuron to	10/171	350/1971[illegible]	0.000988	0.009521	0.008018	CAMK2A/[illegible]
CC	GO:00083	ionotropic	4/171	51/19717	0.000993	0.009521	0.008018	GRIN2A/G
CC	GO:00312	cell projec	9/171	345/1971[illegible]	0.003237	0.029781	0.025079	PACSIN1/[illegible]
CC	GO:00989	GABA-erg	4/171	71/19717	0.003378	0.029885	0.025166	GABRA1/[illegible]
CC	GO:00171	NMDA sel	2/171	11/19717	0.003907	0.033279	0.028025	GRIN2A/G
CC	GO:00988	plasma m[illegible]	8/171	295/1971[illegible]	0.0043	0.035322	0.029745	GRIN2A/G
CC	GO:00140	postsynap	8/171	324/1971[illegible]	0.007448	0.059068	0.049742	CAMK2A/[illegible]
CC	GO:00322	asymmetri	8/171	328/1971[illegible]	0.007991	0.061263	0.05159	CAMK2A/[illegible]
CC	GO:00425	lamellar b[illegible]	2/171	17/19717	0.009336	0.069268	0.058331	KLK7/KLK[illegible]
CC	GO:00451	basal part	3/171	51/19717	0.009841	0.070735	0.059566	EGFR/AQ[illegible]
MF	GO:00305	neurotran[illegible]	15/167	117/1769[illegible]	3.05E-13	1.05E-10	8.63E-11	GRIN2A/G
MF	GO:00152	ligand-ga[illegible]	15/167	138/1769[illegible]	3.55E-12	4.05E-10	3.35E-10	GRIN2A/G
MF	GO:00228	ligand-ga[illegible]	15/167	138/1769[illegible]	3.55E-12	4.05E-10	3.35E-10	GRIN2A/G
MF	GO:00228	ion gated	21/167	334/1769[illegible]	6.85E-12	5.55E-10	4.58E-10	GRIN2A/G
MF	GO:00228	transmitte	11/167	61/17697	1.05E-11	5.55E-10	4.58E-10	GRIN2A/G
MF	GO:00228	transmitte	11/167	61/17697	1.05E-11	5.55E-10	4.58E-10	GRIN2A/G
MF	GO:00228	gated cha	21/167	343/1769[illegible]	1.13E-11	5.55E-10	4.58E-10	GRIN2A/G

MF	GO:00228 substrate-	22/167	428/17697	1.06E-10	4.20E-09	3.47E-09	GRIN2A/G
MF	GO:00052 extracellul	11/167	75/17697	1.10E-10	4.20E-09	3.47E-09	GRIN2A/G
MF	GO:00152 channel ac	22/167	456/17697	3.54E-10	1.15E-08	9.50E-09	GRIN2A/G
MF	GO:00228 passive tra	22/167	457/17697	3.69E-10	1.15E-08	9.50E-09	GRIN2A/G
MF	GO:00052 ion channe	21/167	416/17697	4.06E-10	1.16E-08	9.58E-09	GRIN2A/G
MF	GO:00012 DNA-binc	20/167	439/17697	6.33E-09	1.67E-07	1.38E-07	MEOX2/TI
MF	GO:19043 transmitte	8/167	47/17697	1.22E-08	2.99E-07	2.47E-07	GABRA1/(
MF	GO:00995 neurotrans	8/167	50/17697	2.04E-08	4.66E-07	3.85E-07	GABRA1/(
MF	GO:00989 postsynap	8/167	52/17697	2.81E-08	6.02E-07	4.97E-07	GABRA1/(
MF	GO:00990 ligand-ga	10/167	105/17697	5.70E-08	1.15E-06	9.50E-07	GRIN2A/G
MF	GO:00052 cation cha	16/167	319/17697	6.11E-08	1.16E-06	9.61E-07	GRIN2A/G
MF	GO:00228 GABA-gat	5/167	13/17697	8.53E-08	1.54E-06	1.27E-06	GABRA1/(
MF	GO:00052 inhibitory	5/167	16/17697	2.83E-07	4.85E-06	4.01E-06	GABRA1/(
MF	GO:00048 GABA-A r	5/167	19/17697	7.36E-07	1.20E-05	9.93E-06	GABRA1/(
MF	GO:00990 ligand-ga	5/167	20/17697	9.74E-07	1.52E-05	1.25E-05	GABRA1/(
MF	GO:00169 GABA rece	5/167	22/17697	1.63E-06	2.43E-05	2.01E-05	GABRA1/(
MF	GO:00085 benzodiaz	4/167	11/17697	2.40E-06	3.43E-05	2.83E-05	GABRA1/(
MF	GO:00228 voltage-g	9/167	142/17697	8.17E-06	0.000112	9.26E-05	GRIN2A/G
MF	GO:00468 metal ion	15/167	438/17697	1.79E-05	0.000236	0.000195	SLC12A5/(
MF	GO:00019 G-protein	4/167	24/17697	7.01E-05	0.000859	0.000709	RGS4/DRD
MF	GO:00429 neuropept	4/167	24/17697	7.01E-05	0.000859	0.000709	SSTR4/OP
MF	GO:00081 neuropept	5/167	50/17697	0.000106	0.001192	0.000984	PROKR2/S
MF	GO:00052 voltage-g	9/167	197/17697	0.000108	0.001192	0.000984	GRIN2A/G
MF	GO:00228 voltage-g	9/167	197/17697	0.000108	0.001192	0.000984	GRIN2A/G
MF	GO:00055 calmoduli	9/167	200/17697	0.000121	0.001295	0.001069	EGFR/CAN
MF	GO:00052 calcium ch	7/167	123/17697	0.000167	0.001689	0.001395	GRIN2A/G
MF	GO:00421 neurotrans	5/167	55/17697	0.000167	0.001689	0.001395	GRIN2B/C
MF	GO:00150 calcium io	7/167	139/17697	0.000353	0.003461	0.002857	GRIN2A/G
MF	GO:00151 chloride tr	6/167	100/17697	0.00037	0.003526	0.002911	SLC12A5/(
MF	GO:00228 acetylchol	3/167	16/17697	0.000422	0.003915	0.003232	CHRNA1/(
MF	GO:00085 G protein-	7/167	146/17697	0.000475	0.004178	0.003449	MAS1/MC
MF	GO:00151 inorganic	7/167	146/17697	0.000475	0.004178	0.003449	SLC12A5/(
MF	GO:00016 peptide re	7/167	152/17697	0.000604	0.005182	0.004278	MAS1/MC
MF	GO:00052 chloride cl	5/167	75/17697	0.000714	0.005831	0.004814	GABRA1/(
MF	GO:00704 ammoniur	5/167	75/17697	0.000714	0.005831	0.004814	CHRNA1/I
MF	GO:00052 anion char	5/167	89/17697	0.001545	0.012323	0.010173	GABRA1/(
MF	GO:00996 ligand-ga	3/167	27/17697	0.002044	0.015933	0.013153	GRIN2A/G
MF	GO:00052 excitatory	3/167	31/17697	0.003055	0.023289	0.019226	CHRNA1/(
MF	GO:00052 intracellula	3/167	33/17697	0.003658	0.026531	0.021902	RYR2/HCN
MF	GO:00108 calcium-d	2/167	10/17697	0.00379	0.026531	0.021902	CAMK2A/I
MF	GO:00431 amine bin	2/167	10/17697	0.00379	0.026531	0.021902	HTR5A/HT
MF	GO:00513 serotonin	2/167	10/17697	0.00379	0.026531	0.021902	HTR5A/HT
MF	GO:00085 anion tran	9/167	327/17697	0.003936	0.027001	0.02229	SLC12A5/(
MF	GO:00052 intracellula	2/167	11/17697	0.004604	0.030367	0.025069	HCN1/CN
MF	GO:00438 cyclic nucl	2/167	11/17697	0.004604	0.030367	0.025069	HCN1/CN
MF	GO:00011 enhancer	5/167	119/17697	0.005426	0.035113	0.028987	MEOX2/H
MF	GO:00995 G protein-	3/167	41/17697	0.006765	0.042968	0.035471	HTR5A/HT
MF	GO:00422 peptide bi	8/167	295/17697	0.007021	0.043784	0.036145	GRIN2A/G
MF	GO:00336 activating	4/167	85/17697	0.008566	0.051744	0.042717	TBR1/HOX
MF	GO:00353 enhancer	5/167	133/17697	0.008599	0.051744	0.042717	MEOX2/H
MF	GO:00052 voltage-g	4/167	87/17697	0.009281	0.054887	0.045311	HCN1/KCI
MF	GO:00352 glutamate	3/167	47/17697	0.009875	0.05741	0.047394	CAMK2A/I
MF	GO:00052 voltage-g	3/167	48/17697	0.010462	0.059806	0.049372	CACNG3/(
MF	GO:00421 acetylchol	2/167	17/17697	0.010969	0.06168	0.050919	CHRNA1/(
MF	GO:00049 ionotropic	2/167	19/17697	0.013623	0.075368	0.062218	GRIN2A/G
MF	GO:00009 RNA polyr	4/167	99/17697	0.014395	0.078375	0.064701	MEOX2/H
MF	GO:00150 potassium	5/167	159/17697	0.017522	0.093905	0.077522	SLC12A5/I

Count
33
35
24
13
14
19
22
13
15
13
13
7
18
10
10
17
10
10
5
12
8
12
8
8
5
12
5
6
6
11
4
9
7
14
5
8
6
4
7
4
4
6
6
10
14
6
8
3
6
6
13
8
3
3
8
6
6
13

13
6
4
13
7
4
6
13
5
5
3
3
12
9
3
3
9
7
4
3
6
9
10
9
4
5
4
6
3
3
11
6
6
11
3
3
3
3
5
3
3
4
3
3
3
3
3
6
2
2
2
2
5
3
8
4
6
5
4

10
2
2
2
2
6
3
4
3
6
2
2
5
3
9
4
4
5
9
2
19
19
19
20
17
9
8
8
5
5
9
10
6
9
10
14
11
6
5
5
3
7
10
4
9
4
2
8
8
8
2
3
15
15
15
21
11
11
21

22
11
22
22
21
20
8
8
8
10
16
5
5
5
5
5
4
9
15
4
4
5
9
9
9
7
5
7
6
3
7
7
7
5
5
5
3
3
3
2
2
2
9
2
2
5
3
8
4
5
4
3
3
2
2
4
5
