# Supplementary material for: LncRNA PANTR1 is Associated with Poor Prognostic and Suppresses Apoptosis in Glioma
Source: J Oncol. 2023 Feb 20;2023:8537036. doi: 10.1155/2023/8537036 (PMC9970703; doi:10.1155/2023/8537036)
Supplement: Supplementary Materials — Table 1: Differential expression analysis of PANTR1 in GBM/LGG. Table 2: Gene ontology enrichment analysis of PANTR1 using the clusterProfiler package. Table 3: Pathway enrichment analysis of PANTR1. Table 4: Protein-protein interaction network of PANTR1. Table 5: The association of PANTR1 expression level with clinical parameters of gliomas using the Chi-squared test or Fisher's exact test for analysis. Student's t-test or Wilcoxon rank sum test revealed that age was significantly (p < 0.001) associated with PANTR1 expression. Table 6: The association of PANTR1 expression level with pathological parameters of gliomas using logistics regression. PANTR1 expression was significantly correlated with these variables including WHO grade (p < 0.001), IDH status (p < 0.001), primary therapy outcome (p = 0.016), and EGFR status (p < 0.001). Table 7: Uni- and multivariate Cox regression analysis showed the prognostic value of PANTR1 in overall survival. We observed IDH status (p < 0.001), primary therapy outcome (p < 0.001), age (p = 0.022), and PANTR1 (p = 0.045) are independent prognostic factors in progression-free interval (p < 0.05) of gliomas. Table 8: Uni- and multivariate Cox regression analysis showed the prognostic value of PANTR1 in progression-free survival. Table 9: Uni- and multivariate Cox regression analysis showed the prognostic value of PANTR1 in disease-specific survival. Supplement 10: Relative PANTR1 expression. PCR showed that all 15 glioma samples' PANTR1 expression outweighs normal adjacent tissues, whereas grade II and III glioma tend to have a higher expression rather than GBM compared with NAT. [file 8537036.f1.zip › Supplement table 3.pdf]

ID	setSize	enrichmer	NES	pvalue	p.adjust	FDR	rank	leading_ec
REACTOM	496	0.551	2.026	0.001	0.033	0.023	10147	tags=40%,
REACTOM	352	0.512	1.854	0.001	0.033	0.023	10249	tags=36%,
REACTOM	297	0.547	1.948	0.001	0.033	0.023	12824	tags=42%,
REACTOM	287	0.485	1.724	0.001	0.033	0.023	17345	tags=60%,
REACTOM	263	0.596	2.099	0.001	0.033	0.023	10737	tags=45%,
REACTOM	165	0.507	1.712	0.001	0.033	0.023	14566	tags=55%,
REACTOM	194	0.523	1.783	0.001	0.033	0.023	10123	tags=36%,
REACTOM	158	0.58	1.948	0.001	0.033	0.023	15391	tags=59%,
REACTOM	163	0.489	1.644	0.001	0.033	0.023	8268	tags=28%,
REACTOM	191	0.529	1.806	0.001	0.033	0.023	10147	tags=37%,
REACTOM	183	0.519	1.766	0.001	0.033	0.023	15733	tags=55%,
REACTOM	151	0.55	1.835	0.001	0.033	0.023	14048	tags=55%,
REACTOM	147	0.6	1.995	0.001	0.033	0.023	13502	tags=55%,
REACTOM	144	0.617	2.047	0.001	0.033	0.023	12557	tags=49%,
REACTOM	145	0.616	2.045	0.001	0.033	0.023	12557	tags=54%,
REACTOM	134	0.516	1.699	0.001	0.033	0.023	6451	tags=30%,
REACTOM	114	0.585	1.896	0.001	0.033	0.023	10959	tags=45%,
REACTOM	117	0.532	1.728	0.001	0.033	0.023	16368	tags=62%,
REACTOM	120	0.586	1.907	0.001	0.033	0.023	6436	tags=35%,
REACTOM	125	0.624	2.037	0.001	0.033	0.023	16383	tags=71%,
KEGG_CEL	124	0.636	2.075	0.001	0.033	0.023	8177	tags=40%,
REACTOM	116	0.646	2.094	0.001	0.033	0.023	12557	tags=54%,
REACTOM	115	0.56	1.813	0.001	0.033	0.023	14096	tags=57%,
REACTOM	109	0.605	1.947	0.001	0.033	0.023	14048	tags=63%,
REACTOM	112	0.544	1.749	0.001	0.033	0.023	15385	tags=58%,
REACTOM	111	0.558	1.793	0.001	0.033	0.023	9351	tags=41%,
REACTOM	106	0.596	1.912	0.001	0.033	0.023	4780	tags=30%,
REACTOM	77	0.568	1.736	0.001	0.033	0.023	13543	tags=49%,
REACTOM	93	0.595	1.868	0.001	0.033	0.023	14048	tags=60%,
REACTOM	90	0.627	1.962	0.001	0.033	0.023	7711	tags=40%,
REACTOM	80	0.599	1.845	0.001	0.033	0.023	13543	tags=50%,
REACTOM	76	0.647	1.973	0.001	0.033	0.023	12557	tags=57%,
REACTOM	80	0.624	1.92	0.001	0.033	0.023	8240	tags=38%,
REACTOM	76	0.633	1.933	0.001	0.033	0.023	13726	tags=64%,
PID_E2F_P	73	0.544	1.646	0.001	0.033	0.023	12854	tags=49%,
REACTOM	69	0.682	2.06	0.001	0.033	0.023	11413	tags=61%,
REACTOM	69	0.616	1.86	0.001	0.033	0.023	14493	tags=61%,
KEGG_RIB(	86	0.621	1.929	0.001	0.033	0.023	14048	tags=66%,
REACTOM	81	0.568	1.751	0.001	0.033	0.023	13543	tags=48%,
REACTOM	74	0.6	1.817	0.001	0.033	0.023	17165	tags=68%,
REACTOM	74	0.617	1.868	0.001	0.033	0.023	9542	tags=45%,
REACTOM	88	0.622	1.942	0.001	0.033	0.023	9185	tags=44%,
REACTOM	99	0.601	1.901	0.001	0.033	0.023	14493	tags=61%,
REACTOM	88	0.596	1.861	0.001	0.033	0.023	17165	tags=69%,
REACTOM	70	0.589	1.781	0.001	0.033	0.023	17165	tags=67%,
REACTOM	70	0.57	1.724	0.001	0.033	0.023	17165	tags=66%,
REACTOM	84	0.72	2.228	0.001	0.033	0.023	9745	tags=67%,
REACTOM	83	0.638	1.97	0.001	0.033	0.023	14493	tags=66%,
REACTOM	84	0.583	1.804	0.001	0.033	0.023	17165	tags=67%,
REACTOM	66	0.614	1.835	0.001	0.033	0.023	14493	tags=61%,
REACTOM	66	0.672	2.008	0.001	0.033	0.023	12557	tags=61%,
REACTOM	63	0.61	1.807	0.001	0.033	0.023	11361	tags=49%,
REACTOM	60	0.712	2.091	0.001	0.033	0.023	9745	tags=67%,
REACTOM	61	0.62	1.826	0.001	0.033	0.023	9185	tags=46%,
REACTOM	62	0.653	1.929	0.001	0.033	0.023	8240	tags=42%,
REACTOM	54	0.636	1.839	0.001	0.033	0.023	8240	tags=41%,
REACTOM	57	0.688	2.009	0.001	0.033	0.023	9185	tags=51%,
REACTOM	57	0.73	2.132	0.001	0.033	0.023	9185	tags=60%,

REACTOM	49	0.738	2.106	0.001	0.033	0.023	9745	tags=69%,
PID_AURC	37	0.735	1.991	0.002	0.033	0.023	3877	tags=41%,
REACTOM	38	0.721	1.958	0.002	0.033	0.023	10249	tags=66%,
REACTOM	37	0.775	2.1	0.002	0.033	0.023	7438	tags=68%,
PID_PLK1_	44	0.755	2.104	0.002	0.033	0.023	3385	tags=45%,
REACTOM	44	0.737	2.054	0.002	0.033	0.023	9351	tags=64%,
REACTOM	44	0.698	1.944	0.002	0.033	0.023	12145	tags=68%,
KEGG_DN/	36	0.731	1.977	0.002	0.033	0.023	9575	tags=75%,
PID_ATR_I	39	0.699	1.9	0.002	0.033	0.023	4727	tags=44%,
PID_FOXM	40	0.755	2.061	0.002	0.033	0.023	4851	tags=50%,
REACTOM	33	0.765	2.036	0.002	0.033	0.023	9575	tags=79%,
REACTOM	33	0.701	1.867	0.002	0.033	0.023	9435	tags=52%,
REACTOM	39	0.71	1.93	0.002	0.033	0.023	8240	tags=54%,
REACTOM	35	0.737	1.982	0.002	0.033	0.023	11361	tags=74%,
REACTOM	30	0.72	1.874	0.002	0.033	0.023	9745	tags=73%,
REACTOM	41	0.687	1.877	0.002	0.033	0.023	11413	tags=61%,
REACTOM	41	0.701	1.917	0.002	0.033	0.023	9542	tags=54%,
KEGG_HO	26	0.715	1.821	0.002	0.033	0.023	9169	tags=54%,
REACTOM	34	0.711	1.893	0.002	0.033	0.023	9281	tags=59%,
REACTOM	32	0.783	2.06	0.002	0.033	0.023	9435	tags=81%,
REACTOM	29	0.724	1.88	0.002	0.033	0.023	6684	tags=52%,
REACTOM	25	0.818	2.072	0.002	0.033	0.023	4174	tags=44%,
REACTOM	25	0.728	1.845	0.002	0.033	0.023	5848	tags=44%,
REACTOM	24	0.76	1.914	0.002	0.033	0.023	9575	tags=79%,
BIOCARTA	23	0.737	1.835	0.002	0.033	0.023	5992	tags=48%,
REACTOM	22	0.745	1.843	0.002	0.033	0.023	7438	tags=45%,
REACTOM	20	0.75	1.82	0.002	0.033	0.023	9435	tags=75%,
REACTOM	21	0.735	1.785	0.002	0.033	0.023	9575	tags=71%,
BIOCARTA	18	0.785	1.857	0.002	0.033	0.023	9159	tags=72%,
REACTOM	16	0.816	1.88	0.002	0.033	0.023	4174	tags=62%,
REACTOM	12	0.866	1.878	0.002	0.033	0.023	6885	tags=92%,
REACTOM	11	0.846	1.801	0.002	0.033	0.023	2479	tags=55%,
BIOCARTA	10	-0.869	-1.918	0.002	0.033	0.023	4141	tags=70%,
REACTOM	10	-0.9	-1.986	0.002	0.033	0.023	3490	tags=100%
REACTOM	10	-0.874	-1.927	0.002	0.033	0.023	5642	tags=80%,
BIOCARTA	11	-0.909	-2.067	0.002	0.033	0.023	2276	tags=73%,
REACTOM	11	-0.845	-1.922	0.002	0.033	0.023	4227	tags=64%,
REACTOM	233	0.426	1.481	0.002	0.033	0.023	17459	tags=48%,
REACTOM	12	-0.828	-1.933	0.002	0.033	0.023	5780	tags=58%,
REACTOM	12	-0.894	-2.087	0.002	0.033	0.023	2651	tags=75%,
BIOCARTA	16	-0.798	-1.987	0.002	0.033	0.023	6728	tags=62%,
BIOCARTA	16	-0.752	-1.872	0.002	0.033	0.023	8607	tags=56%,
REACTOM	16	-0.868	-2.161	0.002	0.033	0.023	3490	tags=81%,
REACTOM	16	-0.796	-1.983	0.002	0.033	0.023	5385	tags=69%,
REACTOM	195	0.436	1.49	0.003	0.033	0.023	14501	tags=43%,
BIOCARTA	17	-0.782	-1.987	0.003	0.033	0.023	5555	tags=65%,
REACTOM	17	-0.851	-2.161	0.003	0.033	0.023	4443	tags=88%,
REACTOM	17	-0.762	-1.935	0.003	0.033	0.023	7821	tags=59%,
REACTOM	17	-0.884	-2.245	0.003	0.033	0.023	3490	tags=82%,
REACTOM	17	-0.759	-1.928	0.003	0.033	0.023	9762	tags=82%,
REACTOM	191	0.484	1.651	0.003	0.033	0.023	15733	tags=54%,
REACTOM	18	-0.727	-1.859	0.003	0.033	0.023	7014	tags=39%,
REACTOM	18	-0.785	-2.006	0.003	0.033	0.023	5642	tags=67%,
BIOCARTA	21	-0.843	-2.258	0.003	0.033	0.023	5192	tags=71%,
BIOCARTA	15	-0.838	-2.05	0.003	0.033	0.023	4227	tags=60%,
PID_LPA4_	15	-0.807	-1.974	0.003	0.033	0.023	7441	tags=67%,
REACTOM	21	-0.757	-2.029	0.003	0.033	0.023	8770	tags=67%,
BIOCARTA	13	-0.84	-1.98	0.003	0.033	0.023	6728	tags=77%,
REACTOM	20	-0.819	-2.14	0.003	0.033	0.023	4308	tags=65%,

REACTOM	20	-0.812	-2.122	0.003	0.033	0.023	4696 tags=90%,
BIOCARTA	19	-0.742	-1.894	0.003	0.033	0.023	6728 tags=68%,
REACTOM	19	-0.854	-2.179	0.003	0.033	0.023	5553 tags=84%,
REACTOM	14	-0.81	-1.934	0.003	0.033	0.023	8007 tags=86%,
REACTOM	19	-0.799	-2.04	0.003	0.033	0.023	2406 tags=58%,
REACTOM	19	-0.776	-1.981	0.003	0.033	0.023	7821 tags=63%,
REACTOM	19	-0.821	-2.094	0.003	0.033	0.023	4308 tags=63%,
REACTOM	22	-0.862	-2.306	0.003	0.033	0.023	3490 tags=68%,
REACTOM	22	-0.841	-2.251	0.003	0.033	0.023	4696 tags=73%,
REACTOM	23	-0.866	-2.339	0.003	0.033	0.023	3490 tags=70%,
BIOCARTA	25	-0.719	-1.959	0.003	0.033	0.023	10156 tags=56%,
PID_IL8_C›	25	-0.735	-2.003	0.003	0.033	0.023	9978 tags=72%,
REACTOM	27	-0.779	-2.179	0.003	0.033	0.023	7700 tags=67%,
REACTOM	79	0.526	1.615	0.003	0.033	0.023	8240 tags=37%,
REACTOM	26	-0.752	-2.063	0.003	0.033	0.023	6948 tags=58%,
REACTOM	31	-0.731	-2.107	0.003	0.033	0.023	9170 tags=77%,
REACTOM	31	-0.741	-2.136	0.003	0.033	0.023	7024 tags=74%,
REACTOM	41	-0.807	-2.486	0.003	0.033	0.023	3268 tags=61%,
REACTOM	41	-0.63	-1.941	0.003	0.033	0.023	2930 tags=39%,
REACTOM	41	-0.637	-1.963	0.003	0.033	0.023	6277 tags=44%,
BIOCARTA	30	-0.722	-2.065	0.003	0.033	0.023	10542 tags=57%,
REACTOM	35	-0.784	-2.312	0.003	0.033	0.023	5374 tags=63%,
REACTOM	62	0.59	1.744	0.003	0.033	0.023	12145 tags=48%,
REACTOM	42	-0.801	-2.483	0.003	0.033	0.023	5301 tags=69%,
REACTOM	39	-0.625	-1.897	0.003	0.033	0.023	2847 tags=28%,
REACTOM	39	-0.796	-2.417	0.003	0.033	0.023	5192 tags=64%,
REACTOM	44	-0.723	-2.261	0.003	0.033	0.023	5750 tags=66%,
REACTOM	38	-0.672	-2.013	0.003	0.033	0.023	3333 tags=50%,
REACTOM	47	0.655	1.857	0.003	0.033	0.023	8240 tags=47%,
PID_FANC	43	0.628	1.745	0.003	0.033	0.023	11618 tags=58%,
REACTOM	46	-0.64	-2.014	0.003	0.033	0.023	6277 tags=41%,
SIG_BCR_S	46	-0.593	-1.866	0.003	0.033	0.023	13040 tags=61%,
REACTOM	36	0.657	1.775	0.003	0.033	0.023	9542 tags=47%,
REACTOM	25	0.7	1.773	0.003	0.033	0.023	12557 tags=68%,
REACTOM	48	-0.638	-2.004	0.003	0.033	0.023	10127 tags=58%,
REACTOM	49	-0.626	-1.986	0.003	0.033	0.023	8327 tags=45%,
REACTOM	50	-0.794	-2.535	0.003	0.033	0.023	7161 tags=72%,
REACTOM	51	-0.571	-1.824	0.003	0.033	0.023	11293 tags=55%,
REACTOM	56	-0.788	-2.581	0.003	0.033	0.023	7875 tags=68%,
REACTOM	57	-0.562	-1.846	0.003	0.033	0.023	7124 tags=33%,
KEGG_INC	53	-0.616	-1.975	0.003	0.033	0.023	7509 tags=57%,
PID_NFAT_	53	-0.608	-1.952	0.003	0.033	0.023	10213 tags=55%,
PID_TXA2I	53	-0.556	-1.783	0.003	0.033	0.023	10438 tags=49%,
REACTOM	53	-0.688	-2.208	0.003	0.033	0.023	5394 tags=45%,
KEGG_AM	52	-0.648	-2.078	0.003	0.033	0.023	4696 tags=37%,
REACTOM	52	-0.754	-2.42	0.003	0.033	0.023	6009 tags=60%,
REACTOM	54	-0.733	-2.368	0.003	0.033	0.023	4141 tags=61%,
REACTOM	62	-0.638	-2.12	0.003	0.033	0.023	7014 tags=40%,
REACTOM	55	-0.632	-2.052	0.003	0.033	0.023	9596 tags=55%,
PID_ENDC	63	-0.603	-2.001	0.003	0.033	0.023	9375 tags=56%,
REACTOM	87	-0.751	-2.683	0.003	0.033	0.023	8770 tags=68%,
REACTOM	97	-0.728	-2.658	0.003	0.033	0.023	5917 tags=65%,
REACTOM	89	-0.534	-1.913	0.004	0.033	0.023	3664 tags=33%,
KEGG_ADI	67	-0.566	-1.899	0.004	0.033	0.023	9375 tags=46%,
KEGG_HYI	83	-0.503	-1.77	0.004	0.033	0.023	8719 tags=41%,
KEGG_LOI	70	-0.733	-2.475	0.004	0.033	0.023	5427 tags=54%,
REACTOM	83	-0.52	-1.83	0.004	0.033	0.023	10156 tags=48%,
REACTOM	84	-0.466	-1.644	0.004	0.033	0.023	9845 tags=45%,
KEGG_GAI	88	-0.514	-1.832	0.004	0.033	0.023	7441 tags=42%,

REACTOM	85	-0.446	-1.578	0.004	0.033	0.023	4090	tags=26%,
REACTOM	99	-0.494	-1.813	0.004	0.033	0.023	10537	tags=43%,
REACTOM	85	-0.547	-1.933	0.004	0.033	0.023	5203	tags=40%,
REACTOM	98	-0.426	-1.559	0.004	0.033	0.023	9960	tags=38%,
REACTOM	288	0.406	1.444	0.004	0.033	0.023	9248	tags=30%,
BIOCARTA	81	-0.47	-1.64	0.004	0.033	0.023	9091	tags=31%,
KEGG_ERB	86	-0.513	-1.819	0.004	0.033	0.023	10603	tags=45%,
REACTOM	86	-0.68	-2.41	0.004	0.033	0.023	5374	tags=48%,
REACTOM	82	-0.524	-1.833	0.004	0.033	0.023	6862	tags=41%,
REACTOM	79	-0.483	-1.676	0.004	0.033	0.023	10575	tags=52%,
REACTOM	78	-0.549	-1.905	0.004	0.033	0.023	10156	tags=51%,
REACTOM	78	-0.492	-1.71	0.004	0.033	0.023	10537	tags=42%,
KEGG_LON	68	-0.602	-2.026	0.004	0.033	0.023	7502	tags=46%,
KEGG_B_C	75	-0.459	-1.58	0.004	0.033	0.023	11885	tags=48%,
KEGG_PHO	75	-0.662	-2.276	0.004	0.033	0.023	7894	tags=60%,
REACTOM	80	-0.47	-1.634	0.004	0.033	0.023	10905	tags=46%,
REACTOM	80	-0.575	-2	0.004	0.033	0.023	12651	tags=59%,
REACTOM	76	-0.676	-2.334	0.004	0.033	0.023	6277	tags=59%,
KEGG_DIL	90	-0.573	-2.049	0.004	0.033	0.023	8007	tags=46%,
KEGG_CAF	77	-0.55	-1.899	0.004	0.033	0.023	5549	tags=35%,
KEGG_GNI	101	-0.525	-1.923	0.004	0.033	0.023	7819	tags=44%,
KEGG_MEI	101	-0.588	-2.152	0.004	0.033	0.023	7441	tags=46%,
REACTOM	103	-0.486	-1.775	0.004	0.033	0.023	9762	tags=40%,
REACTOM	103	-0.455	-1.66	0.004	0.033	0.023	11069	tags=45%,
KEGG_FC_	91	-0.486	-1.729	0.004	0.033	0.023	10947	tags=44%,
REACTOM	91	-0.637	-2.263	0.004	0.033	0.023	8770	tags=55%,
REACTOM	105	-0.665	-2.445	0.004	0.033	0.023	8360	tags=55%,
REACTOM	106	-0.485	-1.785	0.004	0.033	0.023	6613	tags=35%,
REACTOM	106	-0.415	-1.526	0.004	0.033	0.023	6966	tags=31%,
REACTOM	106	-0.563	-2.073	0.004	0.033	0.023	8805	tags=45%,
REACTOM	109	-0.408	-1.501	0.004	0.034	0.023	10603	tags=45%,
REACTOM	118	-0.396	-1.471	0.004	0.034	0.023	11134	tags=37%,
REACTOM	119	-0.485	-1.802	0.004	0.034	0.023	7024	tags=39%,
REACTOM	121	-0.415	-1.541	0.004	0.034	0.023	5704	tags=26%,
REACTOM	115	-0.442	-1.629	0.004	0.034	0.023	9755	tags=46%,
KEGG_NEU	125	-0.469	-1.747	0.004	0.034	0.023	10161	tags=41%,
REACTOM	118	0.499	1.618	0.004	0.034	0.023	13543	tags=41%,
KEGG_VAS	113	-0.546	-2.01	0.004	0.034	0.023	8341	tags=48%,
PID_PDGF	127	-0.432	-1.608	0.004	0.034	0.023	13329	tags=43%,
REACTOM	106	0.507	1.627	0.004	0.034	0.023	9185	tags=39%,
REACTOM	105	0.494	1.583	0.004	0.034	0.023	11396	tags=34%,
KEGG_AXO	128	-0.583	-2.17	0.004	0.034	0.023	8402	tags=42%,
KEGG_TIG	128	-0.531	-1.978	0.004	0.034	0.023	7477	tags=34%,
REACTOM	128	-0.563	-2.098	0.004	0.034	0.023	7740	tags=41%,
REACTOM	83	0.517	1.595	0.004	0.034	0.023	17165	tags=61%,
REACTOM	89	0.519	1.617	0.004	0.034	0.023	10335	tags=35%,
REACTOM	138	-0.478	-1.799	0.004	0.034	0.023	10954	tags=49%,
REACTOM	137	-0.616	-2.317	0.004	0.034	0.023	8007	tags=54%,
REACTOM	137	-0.467	-1.755	0.004	0.034	0.023	8582	tags=42%,
REACTOM	52	0.566	1.632	0.004	0.034	0.023	17189	tags=67%,
REACTOM	47	0.616	1.745	0.004	0.035	0.024	7625	tags=32%,
KEGG_WN	150	-0.534	-2.03	0.004	0.035	0.024	10008	tags=47%,
REACTOM	142	-0.495	-1.859	0.005	0.035	0.024	9762	tags=37%,
REACTOM	38	0.636	1.728	0.005	0.035	0.024	11413	tags=55%,
REACTOM	37	0.621	1.682	0.005	0.035	0.024	12557	tags=51%,
KEGG_ALZ	151	-0.432	-1.635	0.005	0.035	0.024	5374	tags=24%,
REACTOM	182	-0.465	-1.813	0.005	0.035	0.024	5313	tags=32%,
KEGG_CAL	177	-0.684	-2.65	0.005	0.035	0.024	8770	tags=62%,
KEGG_CHE	175	-0.402	-1.552	0.005	0.035	0.024	12286	tags=46%,

KEGG_END	176	-0.442	-1.705	0.005	0.035	0.024	8656 tags=31%,
REACTOM	183	-0.473	-1.838	0.005	0.035	0.024	9663 tags=44%,
REACTOM	30	0.646	1.681	0.005	0.035	0.024	12557 tags=63%,
REACTOM	31	0.638	1.671	0.005	0.035	0.024	12557 tags=58%,
KEGG_BAS	34	0.628	1.672	0.005	0.035	0.024	14913 tags=62%,
REACTOM	34	0.649	1.729	0.005	0.035	0.024	13617 tags=65%,
REACTOM	25	0.681	1.725	0.005	0.035	0.024	9575 tags=60%,
REACTOM	10	-0.83	-1.83	0.005	0.035	0.024	3333 tags=70%,
REACTOM	10	-0.805	-1.776	0.005	0.035	0.024	4227 tags=70%,
REACTOM	10	-0.801	-1.768	0.005	0.035	0.024	10161 tags=90%,
REACTOM	185	-0.383	-1.495	0.005	0.035	0.024	10643 tags=33%,
REACTOM	197	-0.469	-1.837	0.005	0.035	0.024	11134 tags=44%,
BIOCARTA	11	-0.791	-1.8	0.005	0.035	0.024	5192 tags=64%,
BIOCARTA	11	-0.784	-1.783	0.005	0.035	0.024	7821 tags=82%,
SA_REG_C	13	0.8	1.769	0.005	0.035	0.024	3177 tags=46%,
BIOCARTA	21	0.697	1.694	0.005	0.035	0.024	11033 tags=62%,
REACTOM	213	0.408	1.408	0.005	0.035	0.024	14493 tags=41%,
REACTOM	18	0.73	1.727	0.005	0.035	0.024	7625 tags=56%,
REACTOM	12	-0.791	-1.847	0.005	0.035	0.024	5192 tags=67%,
REACTOM	201	-0.625	-2.453	0.005	0.035	0.024	8007 tags=55%,
REACTOM	12	-0.796	-1.858	0.005	0.035	0.024	6061 tags=83%,
BIOCARTA	16	0.737	1.697	0.005	0.035	0.024	2706 tags=38%,
BIOCARTA	18	-0.72	-1.839	0.005	0.035	0.024	7630 tags=56%,
REACTOM	203	-0.556	-2.176	0.005	0.035	0.024	8007 tags=48%,
REACTOM	11	0.799	1.7	0.005	0.035	0.024	7438 tags=64%,
BIOCARTA	15	-0.758	-1.853	0.005	0.035	0.024	5192 tags=60%,
KEGG_REG	210	-0.363	-1.435	0.005	0.035	0.024	10575 tags=43%,
BIOCARTA	13	-0.734	-1.732	0.005	0.035	0.024	8607 tags=69%,
REACTOM	13	-0.735	-1.733	0.005	0.035	0.024	4863 tags=77%,
REACTOM	212	-0.452	-1.786	0.005	0.036	0.024	8851 tags=43%,
REACTOM	14	-0.772	-1.841	0.005	0.036	0.025	3333 tags=64%,
BIOCARTA	22	-0.704	-1.884	0.005	0.036	0.025	7821 tags=59%,
REACTOM	215	-0.51	-2.01	0.005	0.036	0.025	7740 tags=27%,
REACTOM	248	-0.501	-2.018	0.005	0.036	0.025	8115 tags=38%,
REACTOM	92	0.502	1.575	0.005	0.036	0.025	17266 tags=65%,
REACTOM	93	0.491	1.541	0.006	0.036	0.025	17165 tags=55%,
REACTOM	29	-0.702	-1.988	0.006	0.036	0.025	5828 tags=45%,
PID_IL8_C)	32	-0.661	-1.928	0.006	0.036	0.025	9978 tags=47%,
REACTOM	81	0.494	1.523	0.006	0.036	0.025	17330 tags=58%,
REACTOM	96	0.513	1.616	0.006	0.036	0.025	8240 tags=34%,
BIOCARTA	34	-0.641	-1.882	0.006	0.036	0.025	7717 tags=35%,
REACTOM	84	0.5	1.548	0.006	0.036	0.025	18370 tags=56%,
BIOCARTA	26	-0.709	-1.944	0.006	0.036	0.025	8341 tags=65%,
KEGG_P53	65	0.558	1.661	0.006	0.036	0.025	9911 tags=42%,
REACTOM	65	0.558	1.66	0.006	0.036	0.025	14493 tags=55%,
REACTOM	30	-0.643	-1.839	0.006	0.036	0.025	3133 tags=50%,
REACTOM	252	-0.331	-1.332	0.006	0.036	0.025	10438 tags=35%,
REACTOM	54	0.555	1.606	0.006	0.036	0.025	17165 tags=67%,
REACTOM	57	0.571	1.668	0.006	0.036	0.025	11361 tags=47%,
REACTOM	58	0.587	1.713	0.006	0.036	0.025	17165 tags=67%,
REACTOM	56	0.562	1.634	0.006	0.036	0.025	13052 tags=45%,
KEGG_MA	265	-0.488	-1.962	0.006	0.036	0.025	8656 tags=40%,
REACTOM	265	-0.67	-2.693	0.006	0.036	0.025	8007 tags=58%,
REACTOM	46	0.589	1.661	0.006	0.037	0.026	12557 tags=48%,
KEGG_NEU	268	-0.666	-2.683	0.006	0.037	0.026	4275 tags=46%,
KEGG_PRC	44	0.578	1.609	0.006	0.038	0.026	17165 tags=68%,
REACTOM	44	0.578	1.609	0.006	0.038	0.026	13726 tags=48%,
REACTOM	27	0.676	1.734	0.006	0.038	0.026	6321 tags=41%,
BIOCARTA	28	0.657	1.694	0.006	0.038	0.026	11072 tags=50%,

PID_BARD	28	0.647	1.668	0.006	0.038	0.026	11618	tags=54%,
REACTOM	285	-0.428	-1.736	0.006	0.038	0.026	10604	tags=40%,
KEGG_MIS	23	0.678	1.688	0.006	0.039	0.027	13447	tags=70%,
REACTOM	13	0.791	1.751	0.006	0.039	0.027	5305	tags=54%,
REACTOM	293	-0.412	-1.684	0.006	0.039	0.027	11100	tags=41%,
REACTOM	298	-0.381	-1.553	0.007	0.039	0.027	10603	tags=35%,
REACTOM	15	0.763	1.737	0.007	0.039	0.027	9435	tags=80%,
PID_FAK_F	58	-0.479	-1.58	0.007	0.039	0.027	13301	tags=55%,
REACTOM	58	-0.527	-1.739	0.007	0.039	0.027	10575	tags=59%,
REACTOM	58	-0.491	-1.622	0.007	0.039	0.027	10905	tags=50%,
REACTOM	59	-0.489	-1.622	0.007	0.039	0.027	9529	tags=46%,
REACTOM	54	-0.464	-1.501	0.007	0.039	0.027	11643	tags=57%,
REACTOM	54	-0.514	-1.661	0.007	0.039	0.027	11643	tags=59%,
KEGG_NO	60	-0.487	-1.616	0.007	0.039	0.027	13662	tags=53%,
PID_TRKR_	61	-0.487	-1.613	0.007	0.039	0.027	10905	tags=44%,
REACTOM	60	-0.515	-1.707	0.007	0.039	0.027	7480	tags=38%,
REACTOM	309	-0.526	-2.159	0.007	0.039	0.027	5313	tags=34%,
REACTOM	63	-0.497	-1.651	0.007	0.039	0.027	11807	tags=62%,
PID_CD8_	65	-0.448	-1.495	0.007	0.039	0.027	9917	tags=35%,
PID_LYSOI	64	-0.463	-1.536	0.007	0.039	0.027	8607	tags=36%,
REACTOM	64	-0.485	-1.612	0.007	0.039	0.027	8446	tags=45%,
REACTOM	64	-0.453	-1.504	0.007	0.039	0.027	12428	tags=53%,
REACTOM	64	-0.495	-1.644	0.007	0.039	0.027	10537	tags=47%,
REACTOM	67	-0.492	-1.653	0.007	0.04	0.027	9529	tags=43%,
KEGG_ARI	74	-0.487	-1.668	0.007	0.04	0.027	9497	tags=45%,
REACTOM	85	-0.439	-1.551	0.007	0.04	0.027	9529	tags=44%,
REACTOM	69	-0.473	-1.591	0.007	0.04	0.027	9529	tags=46%,
REACTOM	95	-0.425	-1.537	0.007	0.04	0.028	10575	tags=42%,
BIOCARTA	11	-0.767	-1.743	0.007	0.041	0.028	9994	tags=73%,
REACTOM	92	-0.439	-1.563	0.007	0.041	0.028	5179	tags=25%,
REACTOM	12	-0.753	-1.76	0.007	0.041	0.028	2691	tags=33%,
REACTOM	12	-0.781	-1.823	0.007	0.041	0.028	10161	tags=83%,
REACTOM	12	-0.786	-1.835	0.007	0.041	0.028	8224	tags=67%,
REACTOM	16	-0.719	-1.791	0.007	0.041	0.028	5593	tags=50%,
BIOCARTA	17	-0.706	-1.793	0.008	0.041	0.028	4227	tags=47%,
BIOCARTA	17	-0.686	-1.741	0.008	0.041	0.028	4301	tags=47%,
REACTOM	18	-0.704	-1.8	0.008	0.041	0.028	4938	tags=56%,
REACTOM	41	0.588	1.606	0.008	0.042	0.029	13927	tags=49%,
REACTOM	118	-0.378	-1.403	0.008	0.042	0.029	13719	tags=48%,
BIOCARTA	24	0.651	1.639	0.008	0.042	0.029	8177	tags=54%,
REACTOM	20	-0.699	-1.827	0.008	0.042	0.029	8240	tags=70%,
REACTOM	14	-0.738	-1.761	0.008	0.042	0.029	3365	tags=71%,
NABA_SEC	331	-0.353	-1.452	0.008	0.042	0.029	9996	tags=39%,
REACTOM	23	-0.691	-1.865	0.008	0.043	0.029	11643	tags=78%,
REACTOM	24	-0.65	-1.761	0.008	0.043	0.03	1960	tags=46%,
REACTOM	24	-0.646	-1.75	0.008	0.043	0.03	11464	tags=67%,
BIOCARTA	25	-0.673	-1.834	0.008	0.043	0.03	10542	tags=60%,
PID_REELI	28	-0.679	-1.922	0.008	0.043	0.03	10156	tags=50%,
REACTOM	25	-0.664	-1.81	0.008	0.043	0.03	11643	tags=76%,
REACTOM	28	-0.699	-1.977	0.008	0.043	0.03	8435	tags=64%,
PID_NETRI	32	-0.615	-1.792	0.008	0.043	0.03	8327	tags=56%,
REACTOM	32	-0.597	-1.74	0.008	0.043	0.03	7665	tags=47%,
REACTOM	358	-0.4	-1.651	0.008	0.043	0.03	12258	tags=22%,
REACTOM	34	-0.609	-1.788	0.008	0.043	0.03	6805	tags=41%,
SIG_CD40I	34	-0.616	-1.809	0.008	0.043	0.03	7455	tags=41%,
REACTOM	26	-0.664	-1.822	0.008	0.043	0.03	7894	tags=58%,
PID_ALPHA	31	-0.599	-1.726	0.008	0.043	0.03	9285	tags=48%,
REACTOM	31	-0.595	-1.715	0.008	0.043	0.03	11643	tags=61%,
REACTOM	65	0.54	1.607	0.008	0.043	0.03	9185	tags=45%,

REACTOM	30	-0.626	-1.79	0.008	0.043	0.03	11643	tags=67%,
REACTOM	137	-0.379	-1.426	0.009	0.043	0.03	10575	tags=37%,
PID_THRO	42	-0.551	-1.708	0.009	0.043	0.03	10548	tags=50%,
KEGG_OLF	352	-0.467	-1.92	0.009	0.044	0.03	8923	tags=19%,
PID_ERBB4	38	-0.595	-1.781	0.009	0.044	0.03	6966	tags=45%,
KEGG_INS	136	-0.39	-1.458	0.009	0.044	0.03	10900	tags=42%,
REACTOM	50	0.565	1.615	0.009	0.044	0.03	17165	tags=66%,
KEGG_GLY	43	-0.58	-1.797	0.009	0.044	0.03	9112	tags=49%,
REACTOM	382	-0.319	-1.315	0.009	0.044	0.031	11298	tags=34%,
REACTOM	182	-0.346	-1.349	0.009	0.045	0.031	8612	tags=29%,
REACTOM	33	0.605	1.61	0.009	0.045	0.031	12145	tags=42%,
REACTOM	391	-0.538	-2.224	0.009	0.046	0.031	5374	tags=31%,
REACTOM	405	-0.694	-2.872	0.009	0.046	0.031	8007	tags=61%,
KEGG_TYP	47	-0.546	-1.717	0.009	0.046	0.031	8944	tags=57%,
REACTOM	110	0.45	1.45	0.009	0.046	0.032	18370	tags=55%,
BIOCARTA	22	0.666	1.648	0.01	0.047	0.032	8666	tags=55%,
PID_P73PA	77	0.483	1.477	0.01	0.047	0.032	8205	tags=31%,
BIOCARTA	19	0.683	1.639	0.01	0.047	0.032	8177	tags=47%,
BIOCARTA	11	-0.74	-1.682	0.01	0.047	0.032	7821	tags=64%,
REACTOM	69	0.517	1.562	0.01	0.047	0.032	11361	tags=45%,
REACTOM	11	-0.743	-1.69	0.01	0.047	0.032	11590	tags=82%,
REACTOM	17	0.715	1.671	0.01	0.047	0.033	6414	tags=47%,
REACTOM	61	-0.464	-1.536	0.01	0.048	0.033	8470	tags=38%,
REACTOM	437	-0.533	-2.235	0.01	0.048	0.033	3679	tags=30%,
REACTOM	463	-0.383	-1.612	0.01	0.048	0.033	10603	tags=38%,
REACTOM	11	0.778	1.655	0.01	0.048	0.033	9435	tags=82%,
BIOCARTA	21	-0.679	-1.821	0.01	0.048	0.033	9402	tags=67%,
BIOCARTA	13	-0.725	-1.709	0.01	0.049	0.034	7024	tags=69%,
REACTOM	20	-0.665	-1.736	0.01	0.049	0.034	6532	tags=65%,
BIOCARTA	19	-0.687	-1.752	0.011	0.049	0.034	7821	tags=47%,
BIOCARTA	19	-0.678	-1.73	0.011	0.049	0.034	8341	tags=47%,
REACTOM	19	-0.71	-1.811	0.011	0.049	0.034	7684	tags=63%,
BIOCARTA	23	-0.659	-1.78	0.011	0.049	0.034	11643	tags=61%,
REACTOM	23	-0.659	-1.781	0.011	0.049	0.034	5192	tags=43%,
REACTOM	23	-0.645	-1.742	0.011	0.049	0.034	4938	tags=70%,
REACTOM	69	-0.453	-1.524	0.011	0.049	0.034	9529	tags=42%,
REACTOM	496	-0.524	-2.226	0.011	0.05	0.034	8923	tags=25%,
REACTOM	77	-0.443	-1.527	0.011	0.05	0.034	9529	tags=40%,
REACTOM	91	0.464	1.461	0.011	0.05	0.034	15739	tags=51%,
BIOCARTA	24	-0.627	-1.699	0.011	0.05	0.034	10156	tags=62%,
PID_S1P_S	24	-0.632	-1.713	0.011	0.05	0.034	9994	tags=54%,
REACTOM	32	-0.573	-1.67	0.011	0.05	0.035	7821	tags=41%,
REACTOM	32	-0.578	-1.684	0.011	0.05	0.035	7124	tags=34%,
KEGG_BUT	34	-0.591	-1.736	0.011	0.05	0.035	8723	tags=50%,
REACTOM	34	-0.597	-1.752	0.011	0.05	0.035	11643	tags=68%,
PID_RXR_V	26	-0.621	-1.705	0.011	0.05	0.035	8262	tags=69%,
REACTOM	14	0.751	1.696	0.011	0.051	0.035	9169	tags=71%,
KEGG_T_C	107	-0.388	-1.43	0.011	0.051	0.035	10537	tags=37%,
REACTOM	53	0.548	1.584	0.011	0.051	0.035	18836	tags=72%,
BIOCARTA	39	-0.564	-1.712	0.011	0.051	0.035	10564	tags=44%,
BIOCARTA	33	-0.591	-1.711	0.011	0.051	0.035	11643	tags=58%,
REACTOM	33	-0.598	-1.732	0.011	0.051	0.035	7954	tags=42%,
SA_G1_AN	15	0.741	1.685	0.011	0.051	0.035	8177	tags=53%,
REACTOM	122	-0.38	-1.412	0.012	0.053	0.036	6183	tags=24%,
KEGG_CEL	127	-0.369	-1.374	0.012	0.054	0.037	8514	tags=27%,
REACTOM	106	0.446	1.43	0.012	0.054	0.037	16846	tags=56%,
BIOCARTA	16	-0.696	-1.733	0.012	0.055	0.038	6200	tags=38%,
REACTOM	48	-0.491	-1.543	0.013	0.055	0.038	13090	tags=56%,
REACTOM	48	-0.487	-1.531	0.013	0.055	0.038	11643	tags=60%,

REACTOM	21	-0.652	-1.747	0.013	0.055	0.038	11643	tags=71%,
REACTOM	53	0.54	1.561	0.013	0.056	0.038	17165	tags=62%,
REACTOM	59	0.516	1.507	0.013	0.056	0.038	18836	tags=64%,
REACTOM	53	0.541	1.562	0.013	0.056	0.038	17165	tags=66%,
REACTOM	20	-0.654	-1.707	0.013	0.056	0.039	4938	tags=75%,
BIOCARTA	19	-0.666	-1.7	0.013	0.057	0.039	9469	tags=53%,
REACTOM	121	0.434	1.412	0.013	0.057	0.04	11042	tags=35%,
REACTOM	142	-0.364	-1.365	0.014	0.058	0.04	9788	tags=28%,
KEGG_NU	44	0.55	1.533	0.014	0.059	0.04	13927	tags=50%,
BIOCARTA	29	-0.593	-1.678	0.014	0.059	0.04	13090	tags=55%,
REACTOM	154	-0.353	-1.344	0.014	0.059	0.04	7139	tags=21%,
REACTOM	27	-0.613	-1.715	0.014	0.059	0.04	6277	tags=44%,
REACTOM	27	-0.62	-1.736	0.014	0.059	0.04	8901	tags=56%,
REACTOM	78	0.475	1.454	0.014	0.059	0.04	18370	tags=59%,
REACTOM	41	-0.52	-1.604	0.014	0.059	0.041	10438	tags=46%,
REACTOM	41	-0.504	-1.555	0.014	0.059	0.041	14301	tags=66%,
REACTOM	74	-0.429	-1.471	0.014	0.059	0.041	10575	tags=46%,
REACTOM	161	-0.36	-1.368	0.014	0.059	0.041	9848	tags=31%,
PID_MTOF	69	-0.438	-1.474	0.014	0.059	0.041	13531	tags=55%,
REACTOM	10	-0.742	-1.638	0.014	0.059	0.041	3954	tags=40%,
PID_PI3K_I	36	-0.588	-1.739	0.014	0.06	0.041	12759	tags=58%,
KEGG_VEG	76	-0.434	-1.497	0.014	0.06	0.041	10537	tags=43%,
REACTOM	44	-0.511	-1.6	0.014	0.06	0.041	11643	tags=50%,
REACTOM	38	-0.555	-1.663	0.015	0.06	0.041	6369	tags=45%,
REACTOM	48	0.542	1.545	0.015	0.06	0.041	13052	tags=42%,
BIOCARTA	13	0.731	1.618	0.015	0.06	0.041	8152	tags=69%,
REACTOM	102	-0.401	-1.468	0.015	0.06	0.041	10603	tags=43%,
PID_PS1_P	45	-0.512	-1.607	0.015	0.06	0.041	9567	tags=42%,
REACTOM	45	-0.508	-1.593	0.015	0.06	0.041	10537	tags=49%,
REACTOM	166	-0.353	-1.345	0.015	0.06	0.041	10137	tags=32%,
REACTOM	104	-0.389	-1.429	0.015	0.06	0.041	8446	tags=38%,
KEGG_VAS	43	-0.526	-1.63	0.015	0.06	0.041	8067	tags=35%,
REACTOM	15	0.709	1.613	0.015	0.06	0.041	12697	tags=73%,
REACTOM	18	-0.672	-1.718	0.015	0.061	0.042	11643	tags=78%,
PID_MYC_	78	0.472	1.443	0.015	0.061	0.042	7141	tags=23%,
REACTOM	25	0.615	1.559	0.016	0.063	0.043	16560	tags=68%,
REACTOM	52	0.53	1.528	0.016	0.063	0.043	17165	tags=67%,
REACTOM	58	0.507	1.479	0.016	0.063	0.043	14566	tags=55%,
REACTOM	56	0.514	1.496	0.016	0.063	0.043	17172	tags=57%,
REACTOM	23	-0.619	-1.671	0.016	0.064	0.044	11643	tags=70%,
REACTOM	127	-0.367	-1.365	0.016	0.064	0.044	8607	tags=30%,
KEGG_TAS	51	-0.481	-1.537	0.016	0.065	0.044	2656	tags=31%,
REACTOM	58	-0.446	-1.471	0.016	0.065	0.045	3315	tags=26%,
REACTOM	28	-0.588	-1.663	0.016	0.065	0.045	3214	tags=29%,
REACTOM	28	-0.582	-1.647	0.016	0.065	0.045	6138	tags=36%,
PID_TGFBI	53	-0.459	-1.474	0.017	0.065	0.045	8588	tags=28%,
KEGG_O_C	29	-0.586	-1.66	0.017	0.065	0.045	7377	tags=48%,
KEGG_ALA	32	-0.566	-1.649	0.017	0.065	0.045	7733	tags=50%,
PID_WNT_	27	-0.582	-1.628	0.017	0.065	0.045	4385	tags=33%,
REACTOM	10	-0.731	-1.612	0.017	0.065	0.045	8607	tags=60%,
REACTOM	10	-0.729	-1.608	0.017	0.065	0.045	8435	tags=80%,
REACTOM	26	-0.585	-1.605	0.017	0.065	0.045	7917	tags=54%,
REACTOM	26	-0.604	-1.658	0.017	0.065	0.045	8007	tags=62%,
PID_PI3KC	35	-0.571	-1.682	0.017	0.065	0.045	13184	tags=57%,
PID_RAS_F	30	-0.572	-1.636	0.017	0.065	0.045	7388	tags=43%,
REACTOM	30	-0.59	-1.688	0.017	0.065	0.045	10537	tags=47%,
REACTOM	42	-0.492	-1.526	0.017	0.066	0.045	7821	tags=38%,
REACTOM	51	0.526	1.512	0.017	0.066	0.045	17165	tags=61%,
PID_RAC1_	38	-0.535	-1.602	0.017	0.066	0.046	10575	tags=53%,

BIOCARTA	16	-0.667	-1.66	0.017	0.066	0.046	8607	tags=56%,
REACTOM	16	-0.666	-1.658	0.017	0.066	0.046	11590	tags=81%,
REACTOM	73	-0.423	-1.448	0.018	0.067	0.046	6910	tags=26%,
REACTOM	78	-0.422	-1.467	0.018	0.067	0.046	10537	tags=49%,
REACTOM	182	0.394	1.338	0.018	0.068	0.046	14895	tags=37%,
BIOCARTA	14	-0.673	-1.606	0.018	0.07	0.048	5185	tags=36%,
REACTOM	14	-0.681	-1.624	0.018	0.07	0.048	9762	tags=64%,
REACTOM	286	-0.312	-1.27	0.019	0.07	0.048	7908	tags=23%,
KEGG_OO	111	-0.371	-1.377	0.019	0.071	0.049	5374	tags=34%,
REACTOM	10	-0.725	-1.599	0.019	0.071	0.049	4443	tags=50%,
REACTOM	10	-0.726	-1.601	0.019	0.071	0.049	4433	tags=60%,
REACTOM	25	-0.597	-1.625	0.019	0.072	0.049	11643	tags=64%,
REACTOM	14	0.702	1.586	0.019	0.072	0.049	6414	tags=43%,
PID_AR_N	31	-0.54	-1.557	0.02	0.073	0.05	12190	tags=52%,
KEGG_ARG	52	-0.459	-1.473	0.02	0.073	0.051	9254	tags=40%,
REACTOM	12	-0.713	-1.664	0.02	0.073	0.051	8571	tags=75%,
KEGG_SPL	123	0.422	1.377	0.02	0.073	0.051	18549	tags=52%,
PID_BCR_5	63	-0.436	-1.448	0.021	0.076	0.052	13323	tags=43%,
REACTOM	76	0.468	1.427	0.021	0.076	0.052	14493	tags=53%,
REACTOM	14	0.698	1.576	0.021	0.076	0.053	2174	tags=29%,
REACTOM	20	-0.641	-1.675	0.021	0.077	0.053	11643	tags=75%,
REACTOM	44	0.531	1.479	0.021	0.078	0.053	7159	tags=23%,
KEGG_PPA	69	-0.432	-1.453	0.021	0.078	0.053	8116	tags=35%,
PID_MET_I	79	-0.419	-1.453	0.021	0.078	0.053	10592	tags=35%,
REACTOM	18	0.642	1.518	0.021	0.078	0.053	12557	tags=56%,
REACTOM	17	0.671	1.569	0.021	0.078	0.053	12557	tags=59%,
REACTOM	29	-0.576	-1.632	0.022	0.079	0.055	6363	tags=41%,
REACTOM	29	-0.567	-1.606	0.022	0.079	0.055	11643	tags=66%,
BIOCARTA	27	-0.579	-1.621	0.022	0.08	0.055	8341	tags=44%,
REACTOM	32	-0.543	-1.583	0.022	0.08	0.055	11643	tags=62%,
REACTOM	73	0.465	1.408	0.022	0.08	0.055	18569	tags=59%,
REACTOM	12	-0.706	-1.65	0.022	0.08	0.055	2433	tags=50%,
BIOCARTA	14	0.697	1.574	0.022	0.08	0.055	14547	tags=86%,
REACTOM	41	-0.496	-1.528	0.022	0.08	0.055	13622	tags=49%,
BIOCARTA	17	-0.643	-1.632	0.023	0.081	0.056	13256	tags=71%,
BIOCARTA	21	-0.616	-1.651	0.023	0.081	0.056	12759	tags=57%,
BIOCARTA	21	-0.619	-1.659	0.023	0.081	0.056	4511	tags=48%,
REACTOM	21	-0.615	-1.647	0.023	0.081	0.056	7237	tags=33%,
REACTOM	112	-0.359	-1.334	0.023	0.081	0.056	9529	tags=35%,
PID_GMCS	36	-0.52	-1.54	0.023	0.081	0.056	12759	tags=53%,
REACTOM	54	-0.451	-1.458	0.023	0.081	0.056	13622	tags=39%,
REACTOM	16	0.678	1.562	0.023	0.082	0.056	6321	tags=38%,
REACTOM	34	0.56	1.491	0.023	0.082	0.056	13726	tags=53%,
PID_RHOA	43	-0.485	-1.5	0.024	0.082	0.057	11582	tags=47%,
REACTOM	43	-0.487	-1.509	0.024	0.082	0.057	10505	tags=35%,
BIOCARTA	19	-0.627	-1.6	0.024	0.082	0.057	10156	tags=58%,
REACTOM	19	-0.616	-1.571	0.024	0.082	0.057	4839	tags=63%,
REACTOM	22	-0.591	-1.581	0.024	0.083	0.057	5380	tags=41%,
REACTOM	65	0.476	1.416	0.024	0.083	0.057	12648	tags=42%,
REACTOM	65	-0.425	-1.418	0.024	0.083	0.057	6417	tags=32%,
REACTOM	19	0.63	1.511	0.024	0.083	0.057	6321	tags=37%,
REACTOM	50	0.504	1.44	0.025	0.085	0.059	13892	tags=44%,
BIOCARTA	12	-0.702	-1.639	0.025	0.085	0.059	10542	tags=67%,
KEGG_SYS	98	0.44	1.389	0.025	0.086	0.059	13970	tags=52%,
REACTOM	34	-0.521	-1.53	0.025	0.086	0.059	10227	tags=56%,
KEGG_PEN	28	0.583	1.502	0.025	0.086	0.059	4356	tags=21%,
PID_REG_(	80	-0.412	-1.432	0.025	0.086	0.059	8777	tags=34%,
SA_PTEN_	17	-0.633	-1.608	0.025	0.086	0.059	7700	tags=47%,
REACTOM	49	-0.458	-1.453	0.025	0.086	0.059	11643	tags=53%,

BIOCARTA	11	0.74	1.574	0.025	0.086	0.059	8152	tags=55%,
BIOCARTA	21	-0.606	-1.623	0.025	0.086	0.059	12759	tags=57%,
REACTOM	15	-0.654	-1.6	0.025	0.086	0.059	9721	tags=73%,
REACTOM	23	0.616	1.532	0.026	0.086	0.059	16560	tags=70%,
REACTOM	42	-0.485	-1.504	0.026	0.086	0.059	5052	tags=40%,
BIOCARTA	33	-0.529	-1.532	0.026	0.086	0.059	8607	tags=39%,
REACTOM	33	-0.538	-1.559	0.026	0.086	0.059	11885	tags=45%,
KEGG_PYR	98	0.439	1.386	0.026	0.088	0.061	14414	tags=56%,
REACTOM	28	0.57	1.47	0.027	0.089	0.061	5507	tags=29%,
REACTOM	19	0.626	1.503	0.027	0.091	0.063	2174	tags=21%,
REACTOM	12	-0.691	-1.614	0.027	0.091	0.063	7612	tags=50%,
REACTOM	13	0.687	1.52	0.028	0.091	0.063	4851	tags=46%,
REACTOM	87	-0.409	-1.462	0.028	0.092	0.063	6766	tags=26%,
REACTOM	18	-0.612	-1.564	0.028	0.092	0.063	4863	tags=67%,
BIOCARTA	21	-0.588	-1.575	0.028	0.092	0.063	12759	tags=52%,
PID_S1P_M	21	-0.595	-1.594	0.028	0.092	0.063	10074	tags=48%,
KEGG_PRI	35	-0.513	-1.513	0.028	0.093	0.064	6728	tags=37%,
REACTOM	347	0.349	1.258	0.028	0.093	0.064	16353	tags=40%,
BIOCARTA	16	0.665	1.532	0.028	0.093	0.064	9159	tags=56%,
REACTOM	48	-0.458	-1.438	0.028	0.093	0.064	13226	tags=42%,
REACTOM	10	-0.713	-1.573	0.029	0.093	0.064	12774	tags=80%,
PID_RET_P	39	-0.492	-1.494	0.029	0.093	0.064	11643	tags=44%,
PID_ATM_	31	0.561	1.47	0.029	0.096	0.066	5558	tags=26%,
REACTOM	12	-0.682	-1.594	0.03	0.097	0.067	5192	tags=33%,
BIOCARTA	46	-0.46	-1.447	0.03	0.098	0.067	8607	tags=41%,
PID_ANTH	18	-0.609	-1.557	0.03	0.098	0.068	7196	tags=44%,
REACTOM	21	-0.578	-1.55	0.031	0.098	0.068	11643	tags=67%,
REACTOM	15	-0.646	-1.581	0.031	0.098	0.068	9762	tags=80%,
PID_EPHB_	40	-0.489	-1.485	0.032	0.101	0.07	10905	tags=50%,
REACTOM	33	-0.512	-1.483	0.032	0.101	0.07	4994	tags=33%,
REACTOM	152	-0.338	-1.28	0.032	0.102	0.07	8639	tags=28%,
KEGG_PRO	23	-0.573	-1.548	0.032	0.102	0.07	7080	tags=52%,
REACTOM	75	-0.405	-1.394	0.032	0.103	0.071	10603	tags=45%,
KEGG_MT	51	-0.456	-1.456	0.033	0.104	0.071	10156	tags=45%,
REACTOM	101	-0.372	-1.362	0.033	0.104	0.072	7139	tags=25%,
REACTOM	27	0.576	1.477	0.033	0.104	0.072	12557	tags=59%,
BIOCARTA	24	-0.561	-1.522	0.033	0.104	0.072	12759	tags=54%,
REACTOM	58	-0.424	-1.398	0.033	0.104	0.072	9248	tags=34%,
REACTOM	21	-0.567	-1.518	0.033	0.104	0.072	7908	tags=38%,
REACTOM	15	-0.645	-1.577	0.033	0.104	0.072	8194	tags=60%,
REACTOM	12	0.705	1.529	0.033	0.105	0.072	2174	tags=17%,
REACTOM	66	0.46	1.374	0.034	0.106	0.073	14831	tags=45%,
REACTOM	20	0.61	1.482	0.034	0.106	0.073	2307	tags=20%,
REACTOM	42	-0.477	-1.479	0.034	0.107	0.074	8154	tags=48%,
REACTOM	54	0.48	1.389	0.034	0.107	0.074	16551	tags=54%,
REACTOM	10	0.728	1.504	0.034	0.107	0.074	2420	tags=20%,
REACTOM	39	-0.486	-1.476	0.034	0.107	0.074	2603	tags=23%,
BIOCARTA	22	-0.573	-1.532	0.034	0.107	0.074	6728	tags=50%,
REACTOM	37	-0.478	-1.425	0.035	0.108	0.075	12029	tags=57%,
REACTOM	85	-0.397	-1.405	0.035	0.11	0.075	3375	tags=20%,
REACTOM	15	-0.637	-1.557	0.036	0.11	0.076	6537	tags=53%,
REACTOM	42	0.515	1.42	0.037	0.114	0.078	8205	tags=33%,
KEGG_ALD	42	-0.471	-1.459	0.037	0.115	0.079	10537	tags=57%,
PID_AURO	31	0.555	1.453	0.037	0.115	0.079	8177	tags=39%,
REACTOM	179	-0.327	-1.271	0.037	0.115	0.079	9529	tags=28%,
REACTOM	25	0.573	1.45	0.038	0.115	0.079	1921	tags=16%,
REACTOM	71	0.45	1.361	0.038	0.115	0.079	17165	tags=54%,
PID_WNT_	32	-0.503	-1.465	0.039	0.119	0.082	9845	tags=44%,
BIOCARTA	11	-0.687	-1.563	0.039	0.119	0.082	5185	tags=36%,

REACTOM	13	0.676	1.495	0.039	0.119	0.082	8945	tags=38%,
REACTOM	51	-0.446	-1.423	0.039	0.119	0.082	8508	tags=29%,
SIG_INSUL	51	-0.441	-1.409	0.039	0.119	0.082	10161	tags=39%,
PID_IFNG_	40	-0.478	-1.451	0.04	0.122	0.084	10156	tags=50%,
REACTOM	56	0.471	1.37	0.04	0.122	0.084	17165	tags=59%,
REACTOM	62	0.461	1.361	0.041	0.125	0.086	17212	tags=60%,
SIG_PIP3_S	66	-0.409	-1.369	0.042	0.126	0.087	9350	tags=50%,
REACTOM	30	0.546	1.421	0.042	0.126	0.087	17172	tags=67%,
REACTOM	26	-0.55	-1.509	0.042	0.126	0.087	8194	tags=54%,
REACTOM	31	-0.51	-1.47	0.042	0.126	0.087	11643	tags=52%,
BIOCARTA	19	-0.568	-1.45	0.042	0.127	0.087	10156	tags=53%,
REACTOM	44	0.503	1.401	0.043	0.128	0.088	18400	tags=66%,
REACTOM	32	0.549	1.444	0.044	0.131	0.09	17172	tags=69%,
REACTOM	63	-0.411	-1.364	0.045	0.133	0.091	11643	tags=41%,
REACTOM	51	0.48	1.38	0.045	0.133	0.091	9801	tags=41%,
PID_TCR_F	14	-0.627	-1.495	0.045	0.133	0.092	10537	tags=64%,
KEGG_HED	56	-0.415	-1.358	0.046	0.136	0.094	9616	tags=48%,
REACTOM	56	-0.415	-1.358	0.046	0.136	0.094	10597	tags=39%,
BIOCARTA	11	-0.673	-1.531	0.046	0.136	0.094	11078	tags=55%,
NABA_CO	265	-0.297	-1.193	0.047	0.137	0.095	8567	tags=33%,
PID_MAPK	34	-0.487	-1.431	0.047	0.14	0.096	10620	tags=41%,
REACTOM	26	-0.542	-1.488	0.047	0.14	0.096	4233	tags=42%,
REACTOM	42	0.505	1.392	0.048	0.14	0.096	17331	tags=67%,
REACTOM	10	-0.684	-1.508	0.048	0.14	0.096	9764	tags=70%,
PID_RHOA	46	-0.449	-1.413	0.048	0.142	0.097	8328	tags=54%,
REACTOM	11	-0.665	-1.513	0.049	0.142	0.098	8327	tags=55%,
REACTOM	58	0.465	1.356	0.049	0.142	0.098	12983	tags=36%,
REACTOM	125	0.396	1.293	0.049	0.143	0.099	17172	tags=55%,
REACTOM	84	-0.389	-1.373	0.049	0.143	0.099	10561	tags=30%,
REACTOM	191	0.367	1.253	0.049	0.143	0.099	8240	tags=24%,
REACTOM	38	-0.466	-1.395	0.049	0.143	0.099	11643	tags=47%,
PID_LIS1_F	28	-0.513	-1.452	0.049	0.143	0.099	9469	tags=39%,

core_enrichment
H2BFS/HIST2H2AA3/CENPA/CENPK/BIRC5/HIST2H2AA4/CDKN2C/MYBL2/CDK4/PTTG1/SPC24/
H2BFS/HIST2H2AA3/CENPA/CENPK/BIRC5/HIST2H2AA4/PTTG1/SPC24/CDC20/UBE2C/NDC80/
H2BFS/HIST2H2AA3/NEIL3/HIST2H2AA4/HIST1H4J/EYA4/CCNA2/EXO1/RAD51/UBE2T/DTL/FA
SEC61G/MRPS17/RPL10L/TSFM/RPS3A/RPL39L/RPL39/RPL22L1/MRPS12/RPS5/RPS26/MRPL54/
H2BFS/CENPA/CENPK/BIRC5/SPC24/CDC25C/CDC20/UBE2C/NDC80/HIST1H4J/CCNB2/CENPU
HOXA2/RPL10L/RPS3A/LHX3/PSMB8/PSMB9/RPL39L/RPL39/RPL22L1/RPS5/PSMA2/RPS26/RPS
CENPA/CENPK/BIRC5/PTTG1/SPC24/CDC20/UBE2C/NDC80/CENPU/SKA1/NUF2/AURKB/KIF18A
CDK4/UBE2C/CCNA2/CDC45/ESCO2/GINS2/WEE1/ORC1/GINS1/CDC6/POLE2/ORC6/CDCA5/N
H2BFS/HIST2H2AA3/HIST1H1D/HIST2H2AA4/CDKN2C/CDK4/UBE2C/HIST1H4J/CCNA2/HIST2H
CENPA/CENPK/BIRC5/SPC24/CDC20/NDC80/NCAPG/CCNB2/CENPU/NEK2/SKA1/CCNB1/NUF
RPL10L/RPS3A/RPL39L/RPL39/RPL22L1/RPS5/RPS26/ERI1/RPS15/RPL35/RPL9/RPL18A/RPS7/N
RPL10L/RPS3A/NUP37/RPL39L/KPNA2/POLR2L/RPL39/RPL22L1/RPS5/RPS26/ISG15/RPS15/RPL
CDKN2C/MYBL2/CDK4/RRM2/CCNB1/CCNA2/TOP2A/CDC45/CDK1/TYMS/WEE1/TK1/ORC1/C
H2BFS/HIST1H4J/EYA4/CCNA2/EXO1/RAD51/RAD51AP1/BRIP1/RMI2/POLE2/XRCC2/CLSPN/P
H2BFS/CDC25C/HIST1H4J/CCNB2/CCNB1/CDC45/CDK1/EXO1/WEE1/GTSE1/ORC1/BRIP1/RMI
CENPA/CENPK/BIRC5/SPC24/CDC20/NDC80/CENPU/SKA1/NUF2/AURKB/KIF18A/KIF2C/CDCA
H2BFS/HIST2H2AA3/HIST2H2AA4/CDK4/HIST1H4J/HIST2H3C/HIST1H3J/RAD51/MND1/HIST2H
RPL10L/RPS3A/RPL39L/RPL39/RPL22L1/RPS5/RPS26/RPS15/RPL35/RPL9/RPL18A/RPS7/RPL26L
CENPA/CENPK/BIRC5/SPC24/CDC20/NDC80/CCNB2/CENPU/SKA1/CCNB1/NUF2/AURKB/KIF1
UBE2C/CCNA2/CDC45/GINS2/ORC1/GINS1/CDC6/POLE2/ORC6/E2F2/MCM2/CDK2/PCNA/DB
CDKN2C/CDK4/PTTG1/CDC25C/CDC20/CCNB2/CCNB1/CCNA2/CDC45/BUB1/CDK1/TTK/WEE1
H2BFS/HIST1H4J/CCNA2/EXO1/RAD51/RAD51AP1/BRIP1/RMI2/POLE2/XRCC2/CLSPN/POLQ/C
RPL10L/NNMT/RPS3A/RPL39L/RPL39/RPL22L1/RPS5/RPS26/RPS15/RPL35/RPL9/RPL18A/RPS7/
SEC61G/RPL10L/RPS3A/RPL39L/RPL39/RPL22L1/RPS5/RPS26/RPS15/RPL35/RPL9/RPL18A/RPS7
RPL10L/RPS3A/RPL39L/RPL39/RPL22L1/RPS5/RPS26/RPS15/RPL35/RPL9/RPL18A/RPS7/RPL26L
H2BFS/HIST2H2AA3/HIST2H2AA4/HIST1H4J/CCNB2/CCNB1/HIST2H3C/HIST1H3J/CDK1/HIST2
CENPA/CENPK/BIRC5/SPC24/CDC20/UBE2C/NDC80/CENPU/SKA1/NUF2/AURKB/KIF18A/KIF2C
H2BFS/HIST2H2AA3/HIST2H2AA4/HIST1H4J/HIST2H3C/HIST1H3J/HIST2H2AC/HIST1H2AD/HIS
RPL10L/RPS3A/RPL39L/RPL39/RPL22L1/RPS5/RPS26/RPS15/RPL35/RPL9/RPL18A/RPS7/RPL26L
HOXB3/HOXB4/HOXA4/HOXA3/HOXA2/H2BFS/HOXB2/HIST2H2AA3/HOXA1/HIST2H2AA4/HI
H2BFS/HIST2H2AA3/HIST2H2AA4/HIST1H4J/HIST2H3C/HIST1H3J/HIST2H2AC/HIST1H2AD/HIS
H2BFS/HIST1H4J/CCNA2/EXO1/BRIP1/RMI2/CLSPN/CDK2/CHEK1/BLM/HIST1H4C/HIST1H4A/R
H2BFS/HIST2H2AA3/HIST2H2AA4/CDKN2C/CDK4/UBE2C/HIST1H4J/CCNA2/HIST2H3C/HIST1H
H2BFS/HIST2H2AA3/HIST2H2AA4/HIST1H4J/HIST2H3C/HIST1H3J/HIST2H2AC/HIST1H2AD/HIS
E2F7/CDKN2C/MYBL2/RRM2/CCNA2/CDK1/TYMS/TK1/ORC1/CDC6/E2F2/CDK2/CDKN2A/E2F1
H2BFS/HIST2H2AA3/NEIL3/HIST2H2AA4/HIST1H4J/POLE2/HIST2H2AC/PCNA/HIST1H2AD/HIST
CCNA2/ORC1/CDC6/ORC6/MCM2/CDK2/CDT1/PSMB8/PSMB9/MCM7/MCM4/MCM6/PSMA2/
RPL10L/RPS3A/RPL39/RPL22L1/RPS5/RPS26/RPS15/RPL35/RPL9/RPL18A/RPS7/RPL26L1/RPS18
H2BFS/HIST2H2AA3/HIST2H2AA4/HIST1H4J/HIST2H3C/HIST1H3J/HIST2H2AC/HIST1H2AD/HIS
PTTG1/CDC20/UBE2C/NEK2/CCNB1/CCNA2/CDK1/BUB1B/PLK1/MAD2L1/PSMB8/PSMB9/PSM
H2BFS/CDC25C/HIST1H4J/CCNB1/CDK1/EXO1/WEE1/BRIP1/RMI2/CHEK1/BLM/HIST1H4C/HIST
H2BFS/HIST2H2AA3/HIST2H2AA4/CDK4/HIST1H4J/HIST2H3C/HIST1H3J/RAD51/MND1/HIST2H
H2BFS/HIST2H2AA3/HIST2H2AA4/HIST1H4J/LMO1/HIST2H3C/HIST1H3J/HIST2H2AC/HIST1H2
UBE2C/CCNA2/ORC1/CDC6/ORC6/MCM2/CDK2/CDT1/PSMB8/PSMB9/MCM7/MCM4/MCM6/
PTTG1/CDC20/UBE2C/AURKB/AURKA/PLK1/PSMB8/PSMB9/PSMA2/SKP2/PSME2/ANAPC11/PS
UBE2C/CCNA2/CDC6/CDK2/PSMB8/PSMB9/PSMA2/PSME2/ANAPC11/PSMA7/PSMD8/CCNE1/
H2BFS/HIST2H2AA3/TERT/CENPA/CENPK/HIST2H2AA4/HJURP/HIST1H4J/CENPU/OIP5/CENPM
CDC45/ORC1/CDC6/POLE2/ORC6/E2F2/MCM2/CDK2/DBF4/MCM10/CDT1/E2F1/RPA3/PSMB8
PTTG1/CDC20/UBE2C/NEK2/CCNB1/CCNA2/AURKB/CDK1/AURKA/BUB1B/PLK1/MAD2L1/CDK
ORC1/CDC6/ORC6/E2F2/MCM2/CDT1/E2F1/PSMB8/PSMB9/MCM7/MCM4/MCM6/PSMA2/MC
EXO1/RAD51/RAD51AP1/BRIP1/RMI2/POLE2/XRCC2/PCNA/CHEK1/BRCA2/EME1/BLM/RFC2/R
H2BFS/HIST2H2AA3/TERT/HIST2H2AA4/HIST1H4J/HIST2H3C/HIST1H3J/HIST2H2AC/HIST1H2A
H2BFS/HIST2H2AA3/TERT/HIST2H2AA4/HIST1H4J/POLE2/HIST2H2AC/PCNA/HIST1H2AD/HIST
H2BFS/HIST2H2AA3/HIST2H2AA4/CDK4/HIST1H4J/HIST2H3C/HIST1H3J/HIST2H2AC/CDK2/HIS
H2BFS/HIST2H2AA3/HIST2H2AA4/HIST1H4J/HIST2H3C/HIST1H3J/HIST2H2AC/HIST1H2AD/HIS
HIST2H2AA3/HIST2H2AA4/CDK4/HIST1H4J/HIST2H3C/HIST1H3J/HIST1H2AI/HIST2H2AC/HIST1
H2BFS/HIST2H2AA3/HIST1H1D/HIST2H2AA4/HIST1H4J/CCNA2/HIST1H1B/HIST2H2AC/CDK2/L
H2BFS/HIST2H2AA3/HIST2H2AA4/CDK4/HIST1H4J/HIST2H3C/HIST1H3J/RAD51/MND1/HIST2H

H2BFS/HIST2H2AA3/CENPA/CENPK/HIST2H2AA4/HJURP/HIST1H4J/CENPU/OIP5/CENPM/HIST
CENPA/BIRC5/NDC80/NCAPG/AURKB/KIF20A/KIF2C/CDCA8/BUB1/AURKA/NCAPH/SMC4/RAC
H2BFS/HIST2H2AA3/HIST2H2AA4/HIST1H4J/HIST2H3C/HIST1H3J/KLK2/HIST2H2AC/HIST1H2A
CDC25C/CDC45/ORC1/CDC6/ORC6/CLSPN/MCM2/CDK2/DBF4/CHEK1/MCM10/RFC2/RPA3/C
SPC24/CDC25C/CDC20/NDC80/CENPU/CCNB1/KIF20A/BUB1/CDK1/TPX2/WEE1/ERCC6L/CENI
H2BFS/HIST2H2AA3/HIST2H2AA4/HIST1H4J/CCNB1/HIST2H3C/HIST1H3J/CDK1/HIST2H2AC/PI
H2BFS/HIST2H2AA3/HIST2H2AA4/HIST1H4J/HIST2H3C/HIST1H3J/EZH2/HIST2H2AC/HIST1H2A
POLE2/MCM2/PCNA/RNASEH2A/RFC2/RPA3/RFC4/RFC3/MCM7/MCM4/MCM6/PRIM1/LIG1/P
CDC25C/CCNA2/RAD51/CDC6/PLK1/CLSPN/MCM2/CDK2/CHEK1/BRCA2/FANCD2/RFC2/TIME
CENPA/BIRC5/CDK4/GAS1/CCNB2/NEK2/CCNB1/CCNA2/AURKB/FOXM1/CDK1/CENPF/PLK1/C
CDC45/ORC1/CDC6/POLE2/ORC6/MCM2/CDK2/DBF4/MCM10/CDT1/RPA3/MCM7/MCM4/MC
EXO1/RAD51/RAD51AP1/BRIP1/RMI2/XRCC2/BRCA2/EME1/BLM/RBBP8/GEN1/BRCA1/BARD1/
H2BFS/HIST2H2AA3/HIST2H2AA4/HIST1H4J/HIST2H3C/HIST1H3J/HIST2H2AC/HIST1H2AD/HIS
H2BFS/HIST2H2AA3/HIST2H2AA4/HIST1H4J/HIST2H3C/HIST1H3J/HIST2H2AC/HIST1H2AD/HIS
TERT/POLE2/PCNA/RFC2/RPA3/RFC4/RFC3/PRIM1/LIG1/PRIM2/POLA2/POLD1/POLD3/WRAP5
H2BFS/HIST2H2AA3/NEIL3/HIST2H2AA4/HIST1H4J/HIST2H2AC/HIST1H2AD/HIST1H4C/HIST1H
EXO1/RAD51/RAD51AP1/BRIP1/RMI2/XRCC2/CHEK1/BRCA2/BLM/RFC2/RPA3/RFC4/RFC3/RBB
RAD51/XRCC2/RAD54L/BRCA2/EME1/BLM/RPA3/RAD54B/POLD1/SSBP1/POLD3/XRCC3/RPA2/
H2BFS/HIST2H2AA3/NEIL3/HIST2H2AA4/HIST1H4J/HIST2H2AC/HIST1H2AD/HIST1H4C/HIST1H
CDC45/GINS2/GINS1/MCM2/PCNA/RFC2/RPA3/RFC4/RFC3/MCM7/GINS4/MCM4/MCM6/MCN
RRM2/CDC45/CDK1/TYMS/TK1/ORC1/CDC6/PCNA/CDT1/E2F1/FBXO5/CDC25A/RBL1/LIN9/DH
CDC25C/CCNB2/CCNB1/CCNA2/FOXM1/CDK1/WEE1/PLK1/PKMYT1/CDK2/CDC25A
EXO1/RAD51/RAD51AP1/BRIP1/RMI2/XRCC2/BRCA2/BLM/RBBP8/BRCA1/BARD1
POLE2/PCNA/RFC2/RPA3/RFC4/RFC3/PRIM1/LIG1/PRIM2/POLA2/POLD1/POLD3/POLE4/FEN1/
CDKN2C/CDK4/CCNB1/CDK1/CDK6/CDK2/CDKN2A/E2F1/CDC25A/CCND2/RBL1
CCNB1/CDK1/ORC1/ORC6/E2F1/MCM8/PRIM1/PRIM2/POLA2/ORC5
PCNA/RFC2/RPA3/RFC4/RFC3/PRIM1/LIG1/PRIM2/POLA2/POLD1/POLD3/FEN1/RPA2/POLD2/I
POLE2/PCNA/RFC2/RPA3/RFC4/RFC3/LIG1/POLD1/POLD3/POLE4/FEN1/RPA2/POLD2/POLE/PC
ORC1/CDC6/ORC6/MCM2/CDK2/CDT1/MCM7/MCM4/MCM6/MCM5/MCM3/ORC5/CCNE1
MYBL2/CDC25C/CCNB2/CCNB1/FOXM1/WEE1/CENPF/PLK1/PKMYT1/CDC25A
CDC45/GINS2/GINS1/MCM2/MCM7/GINS4/MCM4/MCM6/MCM8/MCM5/MCM3
NCAPG/CCNB2/CCNB1/CDK1/NCAPH/SMC4
GABRA3/NSF/GABRA2/GABRA4/GABRA5/GABRA1/GABRA6
VAMP2/VAMP1/STX1B/STX1A/SNAP25/SV2C/SYT2/SYT1/SV2B
DIO2/DUOX2/DUOX1/CGA/TPO/TSHB/SLC5A5/DIO3
PPARA/KCNA3/KCNA2/PLCB1/ARRB1/ADCY1/DNM1/KCNA1
CALM1/PPP3R1/ITPR2/AHCYL1/PPP3CA/PPP3CB/ITPR1
NXT1/SNRPE/SNRPG/SNRPD2/LSM5/DDX39A/LSM7/NUP37/POLR2L/SNRPB/BUD31/LSM2/LSN
GLUL/RIMKLA/OAT/GLUD2/GLS/GLUD1/GLS2
HTR7/HTR4/HTR6/HTR1A/HTR2A/HTR2C/HTR1B/HTR1E/HTR5A
MAPK3/MAPK1/MAP2K1/KCNA3/KCNA2/PLCB1/ARRB1/ADCY1/DNM1/KCNA1
FOS/MYC/MAPK3/MAPK1/MAP2K1/PRKCA/PLCB1/TNF/PRKCB
VAMP2/PPFIA3/PPFIA2/CPLX1/PPFIA4/SLC18A3/SLC5A7/STXBP1/RIMS1/STX1A/RAB3A/SNAP2
PDE10A/KCNMB4/PDE11A/PRKG1/KCNMA1/PRKG2/MRVI1/PDE2A/PDE1B/PDE1A/ITPR1
MYBL2/CDC25C/CCNB2/NEK2/CCNB1/CCNA2/FOXM1/CDK1/TUBA1C/HMMR/TPX2/WEE1/GTS
NCAM1/SPTAN1/ANK1/DLG3/NFASC/SPTBN1/DLG4/DLG2/NRXN3/PCLO/GRIN1
UNC13B/SLC18A2/MAOA/VAMP2/SLC22A2/PPFIA3/PPFIA2/CPLX1/PPFIA4/STXBP1/RIMS1/STX
PRKAR2B/ADCY9/PRKACB/ADCY7/PRKAR1B/ADCY5/ADCY8/ADCY2/ADCY1/PRKACG
VAMP2/SYN3/PPFIA3/PPFIA2/CPLX1/PPFIA4/STXBP1/SYN1/RIMS1/STX1A/RAB3A/SYN2/SNAP2
AP2A1/AP2A2/GRIA3/TSPAN7/GRIA4/AP2B1/GRIP2/GRIA2/PRKCA/NSF/GRIA1/GRIP1/PRKCB/P
RPL10L/RPS3A/RPL39L/RPL39/RPL22L1/RPS5/RPS26/ERI1/RPS15/RPL35/RPL9/RPL18A/RPS7/N(
MECP2/PTPN4/GRIA2/OPRK1/GRIN2B/GRIN2A/OPRM1
AANAT/DIO2/DUOX2/TH/DBH/DUOX1/CGA/TPO/TSHB/SLC5A5/DIO3/TPH2
PRKACB/CALM1/PRKCA/PRKAR1B/CALM3/DLG4/PPP3CA/PPP3CB/GRIN2C/PRKACG/NOS1/PRI
CALM1/CALM3/YWHAH/PPP3CA/PPARA/PPP3CB/CAMK4/SLC2A4/CAMK1G
ADCY9/LPAR4/ADCY7/RPS6KA5/GNAL/ADCY5/ADCY8/ADCY2/PRKCE/ADCY1
GRIN2D/GRIA3/PTPRF/GRIA4/RTN3/DLG3/DLG4/PTPRD/GRIA1/GRIN2C/LRFN2/GRIN1/GRIN2B
MAPK3/MAPK1/MAP2K1/KCNA3/KCNA2/PLCB1/ARRB1/ADCY1/DNM1/KCNA1
DLG3/CALM1/DLG4/CAMK2G/CAMK2B/GRIN2C/CAMK4/DLG2/LRRC7/GRIN1/GRIN2B/GRIN2A

GRIN2D/GRIA3/ACTN2/GRIA4/GRIA2/DLG3/CALM1/DLG4/GRIA1/CAMK2G/CAMK2B/GRIN2C/
CCR3/ROCK2/GNAQ/MAPK3/MAPK1/MAP2K1/MYL2/PRKCA/CCL11/PPP1R12B/PLCB1/PRKCB/
DNAJC5/SLC6A12/SLC6A1/GAD1/VAMP2/ALDH5A1/SLC6A13/CPLX1/STXBP1/RIMS1/STX1A/SL
CACNG2/ADAM22/LGI1/ADAM23/LGI2/DLG4/LGI3/CACNG8/STX1B/ADAM11/STX1A/CACNG3
KCNK3/KCNK6/KCNK16/KCNK17/KCNK12/KCNK4/KCNK1/KCNK18/KCNK9/KCNJ4/KCNJ12
PRKAR2B/ADCY9/PRKACB/PRKX/CALM1/ADCY7/PRKAR1B/ADCY5/ADCY8/ADCY2/ADCY1/PRK
DLG3/CALM1/DLG4/CAMK2G/CAMK2B/DLG2/LRRC7/RASGRF2/GRIN1/GRIN2B/RASGRF1/CAM
VAMP2/APBA1/SYN3/PPFIA3/PPFIA2/CPLX1/PPFIA4/STXBP1/SYN1/RIMS1/STX1A/RAB3A/SYN2
GRIA2/DLG3/CALM1/DLG4/GRIA1/ERBB4/CAMK2G/CAMK2B/GRIN2C/DLG2/LRRC7/GRIN1/NR
VAMP2/GLS/SLC1A6/SLC1A2/PPFIA3/PPFIA2/CPLX1/PPFIA4/STXBP1/RIMS1/GLS2/STX1A/RAB3
PIK3R1/PIK3CG/NFATC2/HDAC5/CABIN1/AVP/CALM1/IGF1/CALM3/YWHAH/PPP3CA/PPP3CB/
ARRB2/GNG2/GNB1/PIK3CG/HCK/CBL/GNA15/PDPK1/PLCB2/PRKCA/GNA14/PLD1/PLCB1/PRK
PDPK1/ACTN2/MAPK3/RPS6KA2/MAPK1/DLG3/CALM1/RPS6KA6/DLG4/CAMK2G/CAMK2B/DL
H2BFS/HIST2H2AA3/HIST2H2AA4/HIST1H4J/LTF/HIST2H3C/HIST1H3J/HIST2H2AC/SAA1/FGA/
PLCB2/PTEN/ITPKB/PLCD3/PLCG2/ITPK1/CALM1/SYNJ1/PLCB4/PLCH1/PLCZ1/ITPKA/INPP5J/PL
AP2A1/AP2A2/MYO6/GRIA3/CACNG2/TSPAN7/GRIA4/AP2B1/GRIP2/GRIA2/PRKCA/DLG4/NSF
KCNQ2/SCN4B/SCN1A/SPTAN1/ANK1/L1CAM/NFASC/SPTBN1/ANK2/SCN11A/SCN9A/SPTBN
CHRM5/HTR7/ADRB2/TAAR5/ADRA2C/HTR4/ADRA1B/ADRB3/CHRM4/CHRM3/ADRA1A/HTR6
KIF17/APBA1/DLG4/GRIN3A/CAMK2G/TUBA8/CAMK2B/GRIN2C/TUBB4A/DLG2/LRRC7/TUBA4
GNB5/RAPGEF3/PRKACB/KCNG2/KCNS3/ITPR2/PRKAR1B/GLP1R/ADCY5/ADCY8/RAPGEF4/KC
PRKAR1A/NFATC2/FOS/GNAQ/PRKAR2B/MAPK3/PRKACB/MAP2K1/CALM1/PRKCA/PRKAR1B/
MAPK1/PRKACB/PRKCD/PRKX/CALM1/PRKCA/ADCY7/PRKAR1B/ADCY5/CAMKK2/ADCY8/CAM
HIST2H2AA3/HIST2H2AA4/HIST1H4J/HIST2H3C/HIST1H3J/HIST1H2AI/HIST2H2AC/HIST1H2AD
KCND2/KCNG2/KCNH6/KCNS3/KCNH4/KCNG1/KCND1/KCNA5/KCNAB1/KCNC4/KCNA3/KCN
CASR/TAS2R4/GABBR1/GABBR2/GRM5/TAS2R3/GRM3/GRM2/GRM7/GRM4/GRM1
PRKACB/PRKCD/PRKX/CALM1/PRKCA/ITPR2/ADCY7/AHCYL1/PRKAR1B/ADCY5/CAMKK2/ADC
SCN1A/CACNA2D1/CACNA1D/CALM1/CACNB3/SCN11A/SCN9A/CACNB4/CACNA1S/FGF12/C
ADCY9/KCNJ15/KCNJ5/KCNJ10/ADCY7/GNAI1/GNAL/ADCY5/GABBR1/KCNJ6/ADCY8/KCNJ9/A
H2BFS/HIST2H2AA3/HIST2H2AA4/HIST1H4J/HIST2H3C/HIST1H3J/HIST2H2AC/HIST1H2AD/HIS
UBE2T/FANCI/BRIP1/CHEK1/BRCA2/FANCA/BLM/FANCD2/RFC2/FANCB/RFC4/FANCC/RFC3/H
GNB5/GNAZ/PRKCD/PRKCA/PRKCQ/ADCY7/GNAI1/ADCY5/ADRA2C/ADCY8/ADCY2/PRKCE/A
GSK3A/ITPR3/SOS1/GSK3B/GRB2/INPP5D/PTPRC/PIK3R1/NFATC2/SOS2/VAV1/BLNK/PDPK1/B
EXO1/RAD51/BRIP1/RMI2/BLM/RFC2/RPA3/RFC4/RFC3/RBBP8/BRCA1/BARD1/RAD9B/RPA2/D
POLE2/PCNA/RFC2/RPA3/RFC4/RFC3/LIG1/POLD1/POLD3/POLE4/RPA2/POLD2/POLE/POLE3/
IPPK/NUDT11/PLD4/INPP1/IPMK/INPP4B/PLCB2/PTEN/NUDT3/PPIP5K1/INPP4A/ITPKB/IP6K3/
DCC/PTK2/MAPK8/EZR/DSCAML1/AGAP2/UNC5B/PTPN11/DOCK1/PRKCQ/NTN4/MAPK13/AB
SLC1A1/UNC13B/SLC18A2/SLC1A7/DNAJC5/SLC6A12/SLC6A1/GAD1/MAOA/VAMP2/SLC22A2
INPP5D/RAB4A/PIK3C2A/PIK3R1/PIK3CG/MTM1/PIK3R5/SBF2/MTMR3/PIP5K1C/INPP4B/PTEN/
PDLIM5/NRXN2/LRRTM3/NLGN2/NLGN4Y/LRRTM1/HOMER1/LRRTM4/DLG3/SYT12/NRXN1/S
SPARC/WWP1/WWOX/ERBB3/APOE/NRG4/GFAP/GABRB1/GABRB3/DLG4/CXCL12/ERBB4/AD
PIK3C2A/PIKFYVE/IPPK/PIK3CG/INPP1/PIP5K1C/IPMK/INPP4B/PLCB2/PTEN/INPP4A/ITPKB/PLC
PRKCH/YWHAB/NFATC2/FKBP8/MAP3K8/CHP1/MAPK8/EP300/CABIN1/BCL2/MAPK3/PRKCD/
SRC/NOS3/PRKCH/GNA13/PIK3CG/HCK/PIK3R5/GNAQ/ROCK1/GNA15/PLCB2/SYK/GNB5/TG
ATP1A4/TRDN/FXYD7/CALM1/ITPR2/PLN/ATP2B1/ATP1A2/ABCC9/AHCYL1/CASQ2/FXYD4/AT
GRIA2/PPP3R1/MAPK13/PPP3R2/GRIA1/PPP3CA/MAP3K5/PPP3CB/SLC1A2/GRIN2C/CHP2/PR
GNAZ/MAPK1/PRKACB/PRKCD/PRKX/CALM1/PRKCA/ITPR2/ADCY7/GNAI1/AHCYL1/PRKAR1B/
ADCY9/KCNJ15/KCNJ5/GABRR3/KCNJ10/NPTN/GABRA3/ARHGEF9/ADCY7/GABRB1/GNAI1/G
MECP2/PTEN/TNRC6B/HTT/TNRC6C/PTPN4/GRIA2/GAD1/CALM1/HIPK2/CAMK2G/CAMK2B/
FXYD2/ATP13A4/ATP2C2/ATP12A/CUTC/ATP13A5/ATP10A/ATP2A2/ATP13A2/ATP1A4/ATP10
JAK2/EDN3/MMP1/FOS/CYSLTR1/GNAQ/COL3A1/EDN1/GNA15/MAPK8/ADCY9/EDNRA/PLCB
GRIN2D/GRIA3/HOMER2/NTRK3/PTPRF/PPFIBP1/PDLIM5/SLITRK2/NRXN2/LRRTM3/NLGN2/G
KCNH8/KCNV2/KCNQ1/KCNK6/KCNJ11/KCNJ15/KCNQ2/KCNJ5/KCNK16/HCN4/KCNJ10/KCN
CALCRL/WNT8A/GIP/FZD9/WNT4/WNT8B/WNT2B/UCN3/GLP1R/WNT2/CALCR/WNT9B/WNT
JAK2/MTOR/TNFRSF1B/POMC/ADIPOR2/IRS1/CHUK/RXRA/ACSL1/MAPK8/AKT3/ACSL4/PCK1/
ITGB6/SLC8A1/CACNG2/ATP2A2/SGCG/MYBPC3/DMD/TPM1/ACTC1/CACNA2D1/PRKAA2/CA
RAPGEF3/MAPK1/PRKACB/MAP2K1/GRIA2/PRKX/CALM1/PPP3R1/PRKCA/ITPR2/RPS6KA6/CAL
PIK3R1/DAPP1/IGHV3-33/IGLV1-51/IGKV3-20/IGKV4-1/IGLV3-19/PIK3AP1/VAV1/BLNK/IGLV2
NFATC2/IGHV3-33/IGLV1-51/IGKV3-20/IGKV4-1/IGLV3-19/VAV1/IGLV2-23/IGLC3/IGKV1-17/
ADCY9/TJP1/PLCB2/TUBB8/MAPK3/GUCY1A2/HTR2B/ADRB1/MAPK1/PRKACB/MAP2K1/PRKX/

MME/PCSK1/KLF4/IGF1/VAMP2/GIP/GRP/CPE/MYO5A/CGA/SLC30A8/KIF5C/TSHB/MYRIP/CPB
KRAS/SRC/FRS3/YWHAB/PIK3R1/CRKL/AP2A1/KIDINS220/BRAF/AP2A2/FRS2/NTRK1/IRS1/NTR
SLC18A2/SLC22A16/SLC13A5/RHCG/SLC14A2/EMB/SLC6A12/SLC2A13/SLC44A1/SLC22A15/SL
RBP2/NMT1/HSD17B6/MYO7A/STRA6/LPL/AKR1C4/GUCA1A/LRP12/TTR/GPC5/GUCA1B/GNB5
H2BFS/HIST2H2AA3/CENPA/CENPK/BIRC5/HIST2H2AA4/SPC24/CDC25C/CDC20/NDC80/HIST1
MKNK1/MAP4K5/FOS/MAP3K8/RPS6KA4/CHUK/MAP3K3/MYC/MAPK8/MAP4K2/MAPK3/RPS6
AREG/KRAS/SRC/PIK3R1/CRKL/PIK3CG/TGFA/BRAF/SOS2/MTOR/PIK3R5/CBL/PTK2/ABL2/MYC/
GNB5/GNAZ/MAPK1/PRKACB/PRKCD/PDE4A/PRKX/CALM1/PPP3R1/PRKCA/ITPR2/ADCY7/GN
P2RX2/SLC8A1/P2RX6/ATP2A2/SLC8A3/P2RX7/GUCY1A2/STIM1/PPP2R5C/GNB5/PDE10A/PTP
TIAM2/ROCK2/AKAP13/ARHGEF7/ARHGEF10/GNA13/SOS2/FGD3/VAV1/ARHGEF11/RHOB/RO
PIK3R1/PLA2G6/PLD4/IGHV3-33/IGLV1-51/IGKV3-20/IGKV4-1/PLD3/IGLV3-19/IGLV2-23/IGL
KRAS/SRC/YWHAB/PIK3R1/CRKL/AP2A1/KIDINS220/BRAF/AP2A2/FRS2/NTRK1/IRS1/ADORA2A
PLA2G4E/GRID2/PLCB2/MAPK3/GUCY1A2/PLA2G2D/CACNA1A/GNAZ/MAPK1/MAP2K1/GRIA2
GSK3B/GRB2/RASGRP3/INPP5D/KRAS/PIK3R1/PIK3CG/DAPP1/NFATC2/SOS2/IFITM1/PIK3R5/F
PIK3R1/PIKFYVE/IPPK/PIK3CG/INPP1/PIK3R5/DGKH/DGKQ/PIP5K1C/INPP4B/PLCB2/PTEN/INPP
RASA1/KRAS/SRC/YWHAB/FGB/APBB1IP/BRAF/TLN1/JAK2/CNKSR1/QKI/RASAL3/AKAP9/VCL/K
GDPD1/MTMR1/TNFAIP8L1/PNPLA7/PIK3C3/INPP5D/MTMR4/RAB4A/MTMR12/PIK3C2A/PIK3R
PRKAR2B/GNA15/KCNJ11/PLCB2/GNB5/ACSL4/CACNA1A/RAPGEF3/PRKACB/KCNG2/CACNA1
CACNG2/ATP2A2/SGCG/MYBPC3/ADCY9/DMD/TPM1/ADRB1/ACTC1/PRKACB/CACNA2D1/CA
ACTC1/ATP1A4/CACNA2D1/CACNA1D/MYL2/CACNB3/ATP1A2/MT-CO3/SLC9A6/CACNB4/M
MAP3K3/PLA2G4E/MAPK8/ADCY9/PLCB2/MAPK3/GNRHR/PLA2G2D/MAPK1/PRKACB/MAP2K4
ADCY9/TCF7L2/EP300/PLCB2/MAPK3/WNT11/MITF/MAPK1/PRKACB/MAP2K1/PRKX/WNT5B/C
AP2A1/TGFA/TGOLN2/WNT5A/AP2A2/CD4/SYT11/CBL/IGF2R/CLTC/ITSN2/UBQLN2/AVP/AP2B
SHB/RASA1/NCKAP1/ROCK2/KRAS/SRC/PRR5/NOS3/PIK3R1/HSP90AA1/JUP/MTOR/ABI2/CYBB
DNM1L/WAS/PTPRC/PIK3R1/PIKFYVE/CRKL/PLA2G6/PIK3CG/HCK/PIK3R5/VAV1/PIP5K1C/PLA2
GRIN2D/GRIA3/TUBB2A/GIT1/PPM1E/PRKAR2B/PDPK1/ACTN2/GRIA4/TUBB8/MAPK3/RPS6KA2
ADIPOR2/GNAQ/PRKAR2B/GNA15/GCGR/KCNJ11/ADCY9/PLCB2/FASN/MLXIPL/GNB5/ACSL4/
ST6GAL1/SSPO/POMK/ADAMTSL1/GALNT6/SBSPON/GALNT8/GALNT18/ADAMTS2/ST6GALNA
WWP1/ANO2/NEDD4L/TRPV4/TRPV3/CLCN6/WNK1/TRPM1/TRDN/UNC80/STOM/BEST1/ANO
SLC20A2/SLC8A1/SLC12A6/SLC7A10/SLC8A3/SLC4A5/SLC6A6/SLC1A1/SLC9A7/SLC7A11/SLC7
AREG/PPP2R5B/MET/FGF7/SRC/CD28/PIK3R1/FGF4/TGFA/FRS2/IL33/FGF20/PHLPP1/PIK3AP1/
PNPLA3/ACHE/PLD6/LIPI/PLA2G12A/GPD1/PLA2G6/PISD/PLD4/GPD1L/LPCAT2/PHOSPHO1/D
KCNQ2/TUBB8/MAPK3/ALCAM/RPS6KA2/SCN4B/AP2B1/SCN1A/NCAM1/MAPK1/SPTAN1/MA
ACTN2/CDH10/CDH3/SIRPA/CLDN2/PARD3/PIK3CB/LAMA3/SPTAN1/PTPN11/SFTPA2/SPTBN1
ACTR2/WAS/NCKAP1/MYH9/HSP90AA1/IGHV3-33/IGLV1-51/IGKV3-20/NF2/ABI2/IGKV4-1/IG
YWHAB/PIK3R1/CRKL/PIK3CG/KIDINS220/BRAF/SOS2/FRS2/SORT1/PIK3R5/NTRK1/IRS1/NTRK3
H2BFS/HIST2H2AA3/HIST2H2AA4/HIST1H4J/HIST2H3C/HIST1H3J/EZH2/HIST2H2AC/HIST1H2A
GNAQ/ARHGEF11/ROCK1/ADORA2A/PLA2G4E/ADCY9/EDNRA/PLCB2/MAPK3/ARHGEF12/GU
WASF2/PAG1/BCAR1/FYN/SOS1/PDGFB/YWHAZ/ARAP1/USP6NL/SLA/GRB2/RAB4A/ACTR2/RA
H2BFS/HIST2H2AA3/HIST2H2AA4/HIST1H4J/HIST2H3C/HIST1H3J/HIST2H2AC/MAEL/HIST1H2
HIST2H2AA3/HIST2H2AA4/HIST1H4J/HIST2H3C/HIST1H3J/PAX3/HIST1H2AI/HIST2H2AC/HIST1
PLXNA2/DCC/PTK2/ROCK1/CHP1/SEMA7A/LRRC4C/PLXNB1/MAPK3/ARHGEF12/UNC5B/SRGA
ACTN2/TJP1/AKT3/PTEN/PPP2R2D/MYH10/TJP3/MRAS/CLDN2/PPP2R2B/PARD3/LLGL1/SPTAN
SPINK5/SPINK9/LCE1B/PPL/SPRR2B/LCE1A/LCE1F/KAZN/SPRR2D/DSC2/KRT24/LOR/LCE3D/SP
CDK4/CCNA2/WEE1/CDK2/E2F1/CKS1B/CDC25A/PSMB8/PSMB9/PSMA2/LIN9/SKP2/PSME2/CD
H2BFS/HIST2H2AA3/HIST2H2AA4/HIST1H4J/HIST2H3C/HIST1H3J/HIST2H2AC/HIST1H2AD/ELF
ACTR2/WAS/NCKAP1/SRC/MYH9/PIK3R1/PLA2G6/HSP90AA1/PLD4/IGHV3-33/IGLV1-51/HCK/
CACNG2/ATP2A2/HIPK1/AKAP9/SLC8A3/KCNQ1/KCNK6/KCNJ11/KCNIP3/SCN4B/STIM1/KCNK
GDI1/FGD3/VAV1/ARHGEF11/RHOB/RHOF/STARD13/ARHGAP22/ARHGAP30/BCR/STARD8/AR
SNRPE/SNRPG/SNRPD2/NUP37/SNRPB/GEMIN7/NDC1/NUP107/NUP210/NUP35/NUP85/NUP
E2F7/CDC25C/CCNB1/CCNA2/CDK1/E2F8/AURKA/CDK2/PCNA/E2F1/GADD45A/RBL1/TP53/BA
ROCK2/PPP2R5B/APC2/NKD2/DKK4/NFATC2/CHD8/TBL1X/WNT5A/DVL1/LRP6/WNT9A/SOX1
AP2A1/TGFA/TGOLN2/WNT5A/AP2A2/PACSIN3/CD4/SYT11/CBL/IGF2R/CLTC/ITSN2/PIP5K1C/
POLE2/PCNA/RFC2/RPA3/RFC4/RFC3/LIG1/POLD1/POLD3/POLE4/FEN1/RPA2/POLD2/POLE/M
POLE2/PCNA/RFC2/RPA3/RFC4/RFC3/MAD2L2/ISG15/POLD1/POLD3/POLE4/RPA2/POLD2/PO
MAPK1/APBB1/CACNA1D/MME/CALM1/PPP3R1/APOE/ITPR2/CDK5R1/MT-CO3/MAPT/IL1B/M
PNOC/BDKRB1/CXCL5/CCR1/GHSR/CXCL2/HCRTR1/NPY/MLNR/CORT/TACR2/APLNR/TAC1/C
GRIN2D/NTSR1/SLC8A1/CYSLTR1/GNAQ/P2RX6/ADORA2A/ATP2A2/CHP1/SLC8A3/GNA15/AD
ARRB2/PREX1/GNG2/CCR7/GSK3B/GNB1/GRB2/PF4V1/PF4/GNB4/PPBP/FOXO3/WAS/CCR3/TI

HSPA1L/GRK5/NTRK1/CBL/AGAP1/CLTC/GIT1/SH3GLB2/PIP5K1C/SMAP1/WWP1/AGAP2/RABE
ANO5/FXYD2/ATP13A4/ATP2C2/WNK4/ATP12A/ASPH/ATP6V0A4/CUTC/SLC9B2/ATP13A5/TP
DTL/POLE2/PCNA/RFC2/RPA3/RFC4/RFC3/RAD18/POLD1/POLD3/POLE4/RPA2/POLD2/POLE/
POLE2/PCNA/RFC2/RPA3/RFC4/RFC3/ISG15/POLD1/POLD3/POLE4/RPA2/POLD2/POLE/POLE3
NEIL3/POLE2/PCNA/LIG1/POLD1/POLD3/HMGB1P1/POLE4/FEN1/POLD2/POLE/MUTYH/UNG/
UBE2T/FANCI/FANCA/EME1/FANCD2/RPA3/FANCB/FANCC/SLX1A/FANCG/SLX1B/RPA2/USP1/
POLE2/PCNA/RFC2/RPA3/RFC4/RFC3/LIG1/POLD1/POLD3/POLE4/FEN1/RPA2/POLD2/POLE/P
ADCY9/ADCY7/GNAL/ADCY5/ADCY8/ADCY2/ADCY1
CALM1/CAMKK2/CAMK2G/CAMK2B/CAMK4/CAMKK1/CAMK2A
YWHAB/CRKL/BRAF/FRS2/NTRK1/RAPGEF1/MAPK3/NGF/MAPK1
ST8SIA2/SPTBN5/AREG/TUBAL3/MAN2A1/TUBB1/STX17/TRAPPC9/GOLGB1/TGFA/MIA3/TRAP
INPP5D/PNPLA3/MTMR4/RAB4A/ACHE/PLD6/LIPI/MTMR12/PIK3C2A/PLA2G12A/PIK3R4/GPD1
PRKAR2B/PRKACB/PRKCA/PRKAR1B/ARRB1/PRKACG/PRKCB
PRKAR1A/PRKAR2B/EZR/PRKACB/SLC9A3R1/PRKAR1B/ADRB2/ADCY1/PRKACG
CDK4/CCNA2/E2F2/CDK2/CDKN2A/E2F1
RAD51/CHEK1/BRCA2/FANCA/FANCD2/FANCC/CHEK2/BRCA1/TP53/FANCG/HUS1/FANCF/FA
EGFR/H2BFS/HIST2H2AA3/FABP7/DLGAP5/HIST2H2AA4/HIST1H4J/HIST2H3C/HIST1H3J/TACC
CDC25C/CCNB1/CDK1/AURKA/PCNA/GADD45A/RBL1/TP53/BAX/PRMT1
PRKAR2B/PRKACB/PRKX/CALM1/PRKAR1B/ADCY8/ADCY1/PRKACG
GLRA1/TUBB2A/NCALD/CACNG2/GIT1/PPM1E/PRKAR2B/PDPK1/GLRB/TSPAN7/ACTN2/ADCY
CACNG2/CACNA1A/CACNB3/CACNB4/CACNB2/CACNA2D2/CACNB1/CACNA1E/CACNA2D3/
CDK4/CCNB2/CCNB1/CDK1/CDK6/CDK2
CAPNS2/EP300/CABIN1/CALM1/PRKCA/MEF2D/CALM3/PPP3CA/PPP3CB/PRKCB
CACNG2/ATP2A2/HIPK1/AKAP9/DYSF/SLC8A3/KCNQ1/KCNK6/MYBPC3/KCNJ11/VCL/ACTN2/
ORC1/CDC6/ORC6/E2F2/E2F1/MCM8/ORC5
PRKAR2B/PRKACB/CDK5R1/PRKAR1B/PPP1R1B/PPP3CA/PLCB1/PRKACG/DRD1
TIAM2/NCKAP1/ROCK2/KRAS/APC2/FGF7/MYH9/ARHGEF7/DIAPH2/PIK3R1/ITGAD/PIKFYVE/C
PRKAR1A/FOS/PRKAR2B/MAPK3/KCNIP3/PRKACB/PRKAR1B/PRKACG/OPRK1
FGF4/FGF20/FGF2/FGF17/FGFR2/FGF9/FGF8/FGF18/FGF5/FGF16
RGS18/NMUR2/NTSR1/MCHR1/GRK5/XCR1/CYSLTR1/GNAQ/FFAR3/DGKH/EDN1/GNA15/GCG
GNAT3/ADCY9/ADCY7/GNAI1/GNAL/ADCY5/ADCY8/ADCY2/ADCY1
GRB2/PRKAR1A/PIK3R1/PRKAR2B/MAPK3/MAPK1/PRKACB/PRKCA/RPS6KA5/PRKAR1B/ADCY1
SPINK5/SPINK9/LCE1B/PPL/KRTAP5-6/SPRR2B/LCE1A/LCE1F/KAZN/SPRR2D/DSC2/KRT24/KRT
SLC2A6/SLC13A3/SLC28A3/SLC7A10/SLC8A3/SLC4A5/SLC29A3/SLC6A6/SLC1A1/SLC5A4/SLC1
MRPS17/TSFM/MRPS12/MRPL54/MRPL11/MRPS15/MRPL32/MRPL36/MRPS24/MRPL53/MRPL1
HIST2H2AA3/HIST2H2AA4/HIST1H2AI/HIST2H2AC/HIST1H2AD/HIST2H2AB/PSMB8/PSMB9/PS
SV2A/MAP2K4/MAP2K1/CALM1/VAMP2/VAMP1/STX1B/STX1A/SNAP25/SV2C/SYT2/SYT1/SV2
PIK3CG/HCK/CBL/GNA15/PDPK1/ELMO1/PLCB2/DOCK2/PRKCA/GNA14/PLCB1/ARRB1/DNM1
NXT1/DDX39A/NUP37/MAGOHB/CPSF4/NDC1/NUP107/NUP210/MAGOH/ALYREF/LUZP4/NU
H2BFS/HIST2H2AA3/HIST2H2AA4/CDKN2C/CDK4/HIST1H4J/HIST2H3C/HIST1H3J/IFNB1/CDK6
GNA15/MAPK3/MAPK1/MAP2K1/CALM1/CALM3/PPP3CA/PPP3CB/PAK1/PLCB1/CAMK1G/PIK
POLE2/PCNA/RFC2/RPA3/RFC4/RFC3/ACTL6A/LIG1/POLD1/POLD3/GTF2H2/POLE4/DDB2/RPA
PRKAR1A/NFATC2/GNAQ/CHUK/PRKAR2B/VIPR2/MYC/PRKACB/CALM1/PRKAR1B/CALM3/PPP
CDK4/RRM2/CCNB2/CCNB1/IGFBP3/CDK1/GTSE1/CDK6/STEAP3/CDK2/CHEK1/CDKN2A/CASP
CCNA2/CDK2/CHEK1/CDKN2A/CDC25A/PSMB8/PSMB9/CHEK2/PSMA2/TP53/PSME2/MDM2/P
KCNJ11/KCNJ15/KCNJ5/KCNJ10/ABCC9/KCNJ1/ABCC8/GABBR1/KCNJ6/KCNJ9/GABBR2/GNG3
SRC/BRPF3/PRKCH/NHLRC2/PIK3R1/GNA13/PIK3CG/FGB/ORM2/CDC37L1/GAS6/APBB1IP/PLE
CDKN2A/PSMB8/PSMB9/CHEK2/PSMA2/TP53/PSME2/MDM2/PSMA7/PSMD8/PSMD14/PSMC2
H2BFS/HIST2H2AA3/HIST2H2AA4/HIST1H4J/HIST2H2AC/LMNB1/HIST1H2AD/HIST1H4C/HIST1
CDK4/CCNA2/CDK2/CKS1B/PSMB8/PSMB9/PSMA2/SKP2/PSME2/PSMA7/PSMD8/CCNE1/PSM
H2BFS/HIST1H4J/EYA4/HIST1H4C/HIST1H4A/HIST1H2BJ/HIST1H2BL/H2AFX/KPNA2/CHEK2/EY
HSPA1L/FOS/TAB2/FGF11/NTRK1/MAP3K8/RPS6KA4/CHUK/CACNG2/BDNF/CHP1/MAP3K3/D
CACNG2/GIT1/PPM1E/PRKAR2B/PDPK1/SLC1A1/GLRB/TSPAN7/ACTN2/ADCY9/UNC13B/CHR
DTL/POLE2/PCNA/RFC2/RPA3/RFC4/RFC3/RAD18/MAD2L2/ISG15/POLD1/POLD3/POLE4/RPA
CHRNB2/HCRTR1/PRLR/GABRA3/S1PR1/GHR/MLNR/GRID1/CALCRL/TACR2/APLNR/LEPR/CHR
PSMC1P4/PSMB8/PSMB9/PSMA2/PSME2/PSMA7/PSMD8/PSMD14/PSMA6P4/PSMC2/PSMA1/
BIRC5/AURKB/TOP2A/CDCA8/AURKA/PCNA/NUP37/NDC1/NUP107/NUP210/NUP35/NUP85/
MYBL2/CCNA2/TOP2A/CDK1/CDC6/CDK2/PCNA/E2F1/CDC25A/RBL1/LIN9
CDK4/CDK1/CDK6/CDK2/CDKN2A/E2F1/CDC25A/TGFB2/TP53/DHFR/SKP2/CCNE1/HDAC1/TG

RAD51/CDK2/PCNA/FANCA/FANCD2/FANCC/RBBP8/BRCA1/BARD1/TP53/FANCG/CCNE1/FAN
SPTBN5/AREG/KRAS/PPP2R5B/MET/FGF7/SRC/FRS3/PIK3R4/YWHAB/PIK3R1/NRTN/FGB/IL16/F
EXO1/PCNA/RFC2/RPA3/RFC4/RFC3/LIG1/POLD1/SSBP1/POLD3/RPA2/POLD2/MLH1/MSH2/M
HIST1H1D/HIST1H1B/HIST1H1E/HMGB2/HIST1H1C/CASP3/HIST1H1A
KBTBD7/FOXO3/PTPRA/IL2RA/RASA1/CDC14A/SPTBN5/AREG/KRAS/PPP2R5B/MET/FGF7/SRC/
AREG/PRKAR1A/PPP2R5B/MET/FGF7/SRC/PRR5/ATN1/CD28/RNF146/PIK3R1/RPTOR/EGR1/OT
PCNA/RPA3/PRIM1/LIG1/PRIM2/POLA2/POLD1/POLD3/FEN1/RPA2/POLD2/DNA2
BCAR1/ASAP1/FYN/SOS1/GRB2/ACTA1/ITGB5/ARHGEF28/RASA1/ROCK2/SRC/ARHGEF7/PIK3R
TIAM2/AKAP13/ARHGEF7/ARHGEF10/GNA13/SOS2/FGD3/VAV1/ARHGEF11/MAPK8/ARHGEF1
RASA1/KRAS/SRC/YWHAB/FGB/APBB1IP/BRAF/TLN1/JAK2/CNKSR1/RASAL3/VCL/KSR1/DAB2IP
IGHV3-33/IGLV1-51/IGKV3-20/IGKV4-1/IGLV3-19/IGLV2-23/IGLC3/IGKV1-17/IGLV3-25/IGHV
GRB2/PIK3C3/FGF1/KRAS/FGF7/PIK3R4/PIK3R1/FGF4/FRS2/FGF20/IRS1/PDPK1/KL/MAPK3/GAB
GRB2/PIK3C3/FGF1/KRAS/FGF7/PIK3R4/PIK3R1/FGF4/FRS2/FGF20/IRS1/IGF2/PDPK1/KL/GAB2/I
NFKBIA/XIAP/CCL2/NLRP1/TAB1/NLRC4/MEFV/PSTPIP1/SUGT1/IL6/TAB3/HSP90AA1/TNFAIP3/
RASA1/KRAS/FRS3/PIK3R1/CRKL/FRS2/NTRK1/NTRK3/BDNF/RAPGEF1/ELMO1/MAPK3/MCF2L/
MYC/TCF7L2/NLK/PLCB2/TNRC6B/TNRC6C/WNT11/GNB5/AXIN2/CALM1/PPP3R1/PRKCA/ITPR
PNOC/BDKRB1/CXCL5/CCR1/PTGDR2/GHSR/GPR143/CXCL2/HCRTR1/S1PR1/NPY/MLNR/COR
COL9A2/COL6A3/GRB2/PTPRA/ST8SIA2/SPTBN5/KRAS/SRC/NRTN/GFRA1/PTK2/COL3A1/GDN
EGR1/NFATC2/BRAF/FOS/CD8A/MAPK8/MAPK3/IFNA2/MAPK1/IFNA8/MAP2K1/PPP3R1/PRKC
FOS/GNAQ/PTK2/GNA15/ADCY9/LPAR4/GNAZ/PIK3CB/PRKCD/GNA14/ADCY7/MAPT/GNAI1/
POMC/RXRA/CYP2W1/CYP8B1/NCOA1/CYP2A6/CYP4F2/CYP4F3/PTGIS/CYP3A43/CYP24A1/C
PLXNA4/SEMA6A/MYL9/GSK3B/PLXND1/DPYSL3/PTPRC/ROCK2/MET/DPYSL2/MYH9/PLXNB3/
KRAS/SRC/YWHAB/FGB/APBB1IP/BRAF/TLN1/JAK2/CNKSR1/QKI/AKAP9/VCL/KSR1/MAPK3/CL
IGHV3-33/IGLV1-51/HCK/IGKV3-20/IGKV4-1/IGLV3-19/IGLV2-23/IGLC3/IGKV1-17/IGLV3-25/
CACNG7/ITGB4/JUP/GJA1/ITGB6/SLC8A1/CACNG2/ATP2A2/SGCG/ACTN2/TCF7L2/DMD/DSC2
IGHV3-33/IGLV1-51/IGKV3-20/IGKV4-1/FOS/IGLV3-19/VAV1/IGLV2-23/MAPK8/IGLC3/IGKV1-
MS4A2/PIK3R1/IGHV3-33/IGLV1-51/IGKV3-20/IGKV4-1/IGLV3-19/IGLV2-23/PDPK1/IGLC3/IGK
TIAM2/AKAP13/ARHGEF7/ARHGEF10/GNA13/LINGO1/SOS2/RTN4/FGD3/VAV1/ARHGEF11/MA
GNA13/GNAQ/ROCK1/ARHGAP5/PRKCA/PPP1R12B/PLCB1/PRKCB
EPHA5/EFNA3/LIMK2/MYH14/EPHB1/CLTCL1/EPHA4/EFNA5/TIAM1/ITSN1/PAK3/EPHA10/EPH
PAK3/PAK1/SLIT2/PAK6
YWHAB/CRKL/KIDINS220/BRAF/FRS2/NTRK1/RAPGEF1/MAPK3/NGF/MAPK1
KCNK3/KCNK6/KCNK16/KCNK17/KCNK4/KCNK1/KCNK18/KCNK9
RASGRP1/RAPGEF3/PRKACB/RAP1GAP/PRKG1/RAPGEF4/RAP1GAP2/PRKACG
NFATC2/GNAQ/CALM1/PRKCA/CALM3/PPP3CA/PPP3CB/PRKCB
EPS15/CALM1/SYNJ2/CALM3/SYNJ1/PPP3CA/PPP3CB/DNM1
FGF22/FGF2/FGF17/FGFR2/FGF9/FGF8/FGF3/FGF18/FGF5/FGF16
POLE2/PCNA/RFC2/RPA3/RFC4/RFC3/POLD1/POLD3/GTF2H2/POLE4/DDB2/RPA2/POLD2/POL
TBC1D25/RAB39B/RAB33A/RAB7A/RIN3/RAB21/TSC2/TBC1D13/GAPVD1/HPS4/GDI2/TBC1D1
CDC25C/CCNB1/CDK1/WEE1/PLK1/CHEK1/CDC25A/CHEK2/GADD45A/BRCA1/TP53/CDC25B/
NTRK3/PTPRF/PPFIBP1/SLITRK2/SLITRK1/SLITRK5/PTPRD/IL1RAPL1/PPFIA3/PPFIA2/PPFIA4/SLIT
DKK4/WNT5A/LRP6/WNT9A/WNT4/WNT3A/SFRP1/DKK2/SFRP2/WIF1
NRTN/EGFL7/IL16/IL1RN/FGF4/TGFA/WNT5A/ANGPT4/TNFSF13/INS-IGF2/WNT9A/CXCL3/EGF
GRB2/FGF1/FGF7/PIK3R1/FGF4/FRS2/FGF20/FGF22/FGF2/PTPN11/FGF17/FGFR2/FGF9/FGF8/FG
KCNJ15/KCNJ5/KCNJ10/GABBR1/KCNJ6/KCNJ9/GABBR2/GNG3/KCNJ4/KCNJ3/KCNJ12
EXOC3/EXOC2/EXOC6/EXOC5/PCSK2/EXOC8/KIF5B/PCSK1/VAMP2/CPE/MYO5A/SLC30A8/KIF
PRKAR1A/PIK3R1/PIK3CG/PRKAR2B/BCL2/MAPK3/CSF2RB/MAPK1/PRKACB/IGF1/PRKAR1B/YW
PIK3R1/CRKL/CBL/DAB1/MAPK8/RAPGEF1/PAFAH1B1/VLDLR/MAP1B/CDK5R1/MAPT/GRIN2B/
GRB2/FGF1/KRAS/FGF7/FRS3/FGF4/FRS2/FGF20/FGF22/FGF2/PTPN11/FGF17/FGFR2/FGF9/FGF
CSF2RA/ADORA2A/ABCA3/ZDHHC2/PGA4/CSF2RB/SFTPC/GATA6/SFTA3/SFTPA2/PGA5/SFTPD
PITPNA/SRC/PIK3R1/DCC/PTK2/ELMO1/AGAP2/MAPK3/UNC5B/MAPK1/MAP2K1/MAP1B/DOC
GCGR/VIPR2/SCTR/SCT/GNB5/GHRHR/GIP/GLP1R/ADCYAP1R1/ADCYAP1/VIPR1/GNG3/GLP2R
OR52B6/OR13C9/OR2T33/REEP2/OR52I1/OR5B12/OR2T10/OR10J3/OR4C15/GNB1/OR4F6/OR
GUCA1B/GNB5/NMT2/CNGA1/CALM1/PRKCA/RCVRN/PRKCQ/RGS9BP/RGS9/RHO/PPEF1/GUC
MAPK8/MAPK8IP1/MAPK3/GORASP1/MAPK1/MAP2K4/PIK3CD/TRAF3/MAPK8IP3/MAPK9/MAP
DGKH/DGKQ/MGLL/DAGLA/ABHD6/RASGRP1/DGKZ/PRKCD/ITPR2/PRKCQ/DGKI/DGKB/PRKCE
SRC/HCK/UCHL1/GRK5/PLCB2/MAPK3/SYK/MAPK1/PRKCD/TH/PLD1/PTK2B/KLK6/SLC6A3/SNC
GRB2/FGF1/KRAS/FRS3/PIK3R1/FGF4/FRS2/FGF20/KL/FLRT1/FGF22/FGF2/PTPN11/FGF17/FGF9/
H2BFS/HIST2H2AA3/HIST2H2AA4/CDC25C/HIST1H4J/HIST2H3C/HIST1H3J/KLK2/HIST2H2AC/H

GRB2/FGF1/KRAS/FGF7/FRS3/PIK3R1/FGF4/FRS2/FGF20/FGF22/FGF2/PTPN11/FGF17/FGFR2/FG
TIAM2/AKAP13/ARHGEF7/ARHGEF10/GNA13/TNFRSF10A/LINGO1/OTUD7B/SOS2/TNFAIP3/RT
ROCK2/AKAP13/NOS3/PIK3R1/GNA13/GNAQ/ROCK1/GNA15/PLCB2/GNAZ/PRKCD/MYL2/PRK
OR2C3/OR4N2/OR2L3/OR8D4/OR10Q1/OR4C3/OR1D2/GUCA1A/OR2M3/OR2T6/OR52B2/OR
PIK3R1/JAK2/TAB2/WWP1/MAPK3/WWOX/MAPK1/PIK3CB/PIK3CD/PRLR/LRIG1/NRG4/DLG4/E
PHKA1/ACACB/PYGM/PRKAR1A/KRAS/PDE3A/PIK3R1/RPTOR/CRKL/PPP1R3D/PIK3CG/BRAF/S
APOBEC3G/PSMB8/PSMB9/PSMA2/PSME2/PSMA7/PSMD8/PSMD14/PSMC2/PSMA1/PSMB4/P
LPL/ALDH9A1/MBOAT2/DGKH/DGAT2/DGKQ/AGPAT4/MGLL/GPAT2/AGPAT3/DGKZ/LIPF/GPA
FGF1/MAMLD1/ZMYM2/MAML3/FGFR1OP2/FOXO3/PORCN/RASA1/IHH/AREG/NCOR2/KRAS/
IGKV4-1/FOS/TAB2/IGLV3-19/VAV1/CHUK/IGLV2-23/PDPK1/MAPK8/FBXW11/IGLC3/IGKV1-1
RRM2/RAD51/EZH2/CHEK1/E2F1/RBBP8/BRCA1/CBX3/CDC7/UXT/E2F6/RNF2/EED/RBBP4
MAPK1/PNOC/BDKRB1/CXCL5/PRKACB/CCR1/PTGDR2/RGS11/PRKCD/PDE4A/RPE65/NMT2/A
CACNG2/GIT1/KCNH8/PPFIBP1/PDLIM5/PPM1E/SLITRK2/PRKAR2B/KCNV2/KCNQ1/PDPK1/SLC
MTOR/PIK3R5/IRS1/KCNJ11/MAPK8/CACNA1G/MAPK3/CACNA1A/MAPK1/PIK3CB/CACNA1D/
POLE2/PCNA/RFC2/RPA3/RFC4/RFC3/POLR2L/ACTL6A/LIG1/POLR2J/POLD1/POLR2H/POLD3/
LMNB1/CASP6/CASP3/CASP4/CASP1/GZMB/CASP2/CASP8/PRF1/LMNB2/BIRC3/LMNA
GDF15/CCNB1/WT1/CCNA2/BUB1/CDK1/IL1RAP/RAD51/CDK6/PLK1/CDK2/CHEK1/BRCA2/S10
RAD51/CHEK1/RBBP8/CHEK2/GADD45A/BRCA1/TP73/TP53/MDM2
PRKAR1A/PRKAR2B/PRKACB/PRKAR1B/ADRB2/ADCY1/PRKACG
H2BFS/HIST2H2AA3/HIST2H2AA4/HIST1H4J/HIST2H3C/HIST1H3J/HIST2H2AC/HIST1H2AD/HIS
PIK3C3/MTMR4/PIK3C2A/PIK3R4/PIKFYVE/MTM1/FIG4/MTMR9/MTMR7
HIST1H1D/HIST1H1B/LMNB1/HIST1H1E/HIST1H1C/HMGA2/HIST1H1A/TP53
MUC15/B3GNT2/MUCL1/GCNT1/ST6GAL1/GALNT6/GALNT8/GALNT18/ST6GALNAC3/MUC5B/
CORT/CALCRL/WNT8A/TACR2/APLNR/TAC1/CX3CL1/GIP/FZD9/WNT4/GRP/INSL3/CHRM5/S1
AREG/NCKAP1/ROCK2/KRAS/MET/FGF7/SRC/PRR5/CMA1/FRS3/NOS3/PIK3R4/FGFBP1/ARHGE
PCNA/RPA3/LIG1/POLD1/POLD3/FEN1/RPA2/POLD2/DNA2
ARHGAP4/TLN1/ARHGEF11/ROCK1/VCL/GSN/BAIAP2/ARHGAP6/MYL2/ARHGAP5/OPHN1/AR
NOS3/KCNQ4/KCNQ2/GUCY1A2/TRPV4/KCNQ3/ADCY1/KCNQ5/TRPC5
GUCY1A2/PDE10A/KCNMB4/PDE11A/PRKG1/KCNMA1/PRKG2/MRVI1/PDE2A/PDE1B/PDE1A/I
PRKAR2B/MLXIPL/ACSBG1/PRKACB/PRKAA2/PRKAG2/PRKAR1B/ADCY1/PRKACG
GNAQ/MAPK3/MAPK1/MAP2K1/PRKCA/PTK2B/CXCL12/PRKCB/PIK3C2G
SLC6A6/SLC18A2/SLC6A12/SLC6A1/SLC6A5/SLC22A2/SLC6A13/SLC6A3/SLC6A18/SLC6A20/SL
SOS1/GRB2/PRKAR1A/PIK3R1/IRS1/PRKAR2B/MAPK3/MAPK1/PRKACB/MAP2K1/PRKAR1B/YW
PRKAR2B/PRKACB/PDE4A/CALM1/PPP3R1/PRKAR1B/PPP1R1B/PPP3CA/PPP3CB/PRKACG
GRB2/FGF1/KRAS/FGF7/FGF4/FGF20/FGF22/FGF2/FGF17/FGFR2/FGF9/FGF8/FGF3/FGF18/FGF5/
IGHV3-33/IGLV1-51/IGKV3-20/IGKV4-1/IGLV3-19/IGLV2-23/IGLC3/IGKV1-17/IGLV3-25/IGHV
OR2C3/GPR45/OR4N2/GNAT3/OR2L3/REEP5/GRK5/OR8D4/POMC/OR10Q1/OR4C3/OR1D2/P
IGHV3-33/IGLV1-51/IGKV3-20/IGKV4-1/IGLV3-19/IGLV2-23/IGLC3/IGKV1-17/IGLV3-25/IGHV
CCNA2/AURKB/EXO1/TPX2/AURKA/BRIP1/RMI2/CDK2/CHEK1/BLM/RFC2/RPA3/RFC4/RFC3/RB
EIF4A2/PIK3R1/PABPC1/MKNK1/MTOR/IRS1/EIF4G3/PDPK1/PTEN/MAPK3/PDK2/MAPK1/PRKC
GNA13/FOS/GNAQ/IRS1/GNA15/MAPK8/MAPK3/GNAZ/MAPK1/GNA14/GNAI1/GNAO1/PAK1
PRKAR2B/GCGR/ADCY9/PRKACB/ADCY7/PRKAR1B/ADCY5/ADCY8/ADCY2/ADCY1/PRKACG/G
TAB2/SPARC/WWOX/APOE/NRG4/GFAP/CXCL12/ERBB4/ADAP1/PGR/NRG3
HADHA/EHHADH/PDHA1/ECHS1/ABAT/ALDH9A1/HMGCS1/L2HGDH/AACS/OXCT1/GAD1/AL
GRB2/FGF1/FGF7/SRC/FGF4/BRAF/MKNK1/FRS2/FGF20/CBL/MAPK3/MAPK1/FGF22/FGF2/PTP
PPARG/PPARD/MED1/VDR/FAM120B/RARB/NCOR2/ABCA1/RXRA/BCL2/NCOA1/THRA/SREBF1
PCNA/RFC2/RFC4/RFC3/PRIM1/PRIM2/POLA2/POLD1/POLD3/POLD2
KRAS/CD28/PIK3R1/PIK3CG/NFATC2/SOS2/CD4/PIK3R5/FOS/CBL/VAV1/MAP3K8/CD8A/CHUK
AURKA/PSMB8/PSMB9/PSMA2/PSME2/PSMA7/PSMD8/PSMD14/PSMC2/PSMA1/PSMB4/PSM
MS4A2/PIK3R1/PIK3CG/NFATC2/FOS/VAV1/MAPK8/MAPK3/SYK/MAPK1/MAP2K4/MAP2K1/C
SOS1/GRB2/ACTA1/RASA1/MET/PIK3R1/CRKL/PIK3CG/FOS/MAPK8/RAPGEF1/PTEN/MAPK3/M
SLC7A10/SLC6A6/SLC7A11/SLC7A1/SLC6A12/SLC38A1/SLC7A8/SLC1A4/SLC36A2/SLC7A2/SLC
CDK4/E2F2/CDK2/CDKN2A/E2F1/NXT1/TP53/MDM2
GPC3/KERA/NDST1/VCAN/B4GALT1/UST/ST3GAL1/ST3GAL6/ACAN/B4GALT6/NDST4/STAB2/
OCLN/CD8A/PTPRF/NRXN2/NLGN2/SIGLEC1/CDH3/ALCAM/CLDN2/NCAM1/VCAN/ITGAM/SE
METTL1/RPPH1/NUP37/RPP40/CPSF4/NDC1/HSD17B10/NUP107/NUP210/POP7/QTRT1/NUP3
C7/SELP/SELPLG/C6/KNG1/TNF
FYN/PTPN6/SOS1/YWHAZ/GRB2/INPP5D/IL2RA/PIK3R1/CRKL/JAK2/HCK/CBL/CSF2RA/VAV1/B
GRB2/PIK3C3/FGF1/KRAS/FGF7/PIK3R4/PIK3R1/FGF4/FRS2/FGF20/IRS1/PDPK1/KL/GAB2/PIK3C

GRB2/FGF1/PIK3R1/FGF4/FRS2/FGF20/KL/FGF22/FGF2/PTPN11/FGF17/FGF9/FGF8/FGF3/FGF5
PSMB8/PSMB9/HSPB1/PSMA2/PSME2/PSMA7/PSMD8/PSMD14/PSMC2/PSMA1/PSMB4/PSMD
OS9/PSMB8/PSMB9/PSMA2/PSME2/PSMA7/PSMD8/DERL3/PSMD14/PSMC2/PSMA1/PSMB4/P
CDKN2A/PSMB8/PSMB9/PSMA2/PSME2/CBFB/MDM2/PSMA7/PSMD8/PSMD14/PSMC2/PSMA
FGF1/FGF7/FGFBP1/FGF4/FGF20/FGF22/FGF2/FGF17/FGFR2/FGF9/FGF8/FGF3/FGF18/FGF5/FGF
NDEL1/CLIP1/DNAH8/DNAH6/DNAH1/PAFAH1B1/DNAH2/DCTN1/CDK5R1/RELN
H2BFS/HIST2H2AA3/HIST2H2AA4/HIST1H4J/HIST2H3C/HIST1H3J/HIST2H2AC/CITED1/HIST1H
AGO4/AP2A1/WNT5A/DVL1/AGO1/AP2A2/DAAM1/AGO3/CLTC/MYC/TCF7L2/NLK/PLCB2/TN
POLE2/PCNA/RFC2/RPA3/RFC4/RFC3/LIG1/POLD1/POLD3/GTF2H2/POLE4/DDB2/RPA2/POLD
FYN/SOS1/F2/GRB2/AGT/JAK2/MAPK8/MAPK3/MAPK1/MAP2K1/CALM1/PRKCA/PTK2B/CALM
ACTR1A/ANKRD28/TUBB8/SEC24C/CD55/ARF3/GORASP1/DYNLL2/SEC16B/DYNC1LI2/SPTAN1
GNB5/CACNA1D/CACNB3/GNAI1/CACNB2/CACNA1C/CACNA2D2/ADCY5/ADRA2C/ADRA2A/
P2RX2/SLC8A1/P2RX6/ATP2A2/SLC8A3/P2RX7/STIM1/CALM1/ITPR2/ATP2B1/P2RX5/ATP2B2/I
POLE2/PCNA/RFC2/RPA3/RFC4/RFC3/POLR2L/LIG1/POLR2J/POLD1/POLR2H/POLD3/POLR2G/
SRC/PIK3R1/NRTN/GFRA1/FRS2/GDNF/GAB2/PIK3CB/PRKACB/PIK3CD/RAP1GAP/PTPN11/PRK
GABARAP/TBC1D20/RAB5A/RAB11A/GGA1/GGA2/GGA3/TBC1D25/RAB33A/RAB7A/TSC2/TBC
TIAM2/AKAP13/ARHGEF7/ARHGEF10/GNA13/SOS2/FGD3/VAV1/ARHGEF11/MAPK8/ARHGEF1
DAPP1/NFATC2/IGHV3-33/IGLV1-51/IGKV3-20/IGKV4-1/IGLV3-19/PIK3AP1/VAV1/BLNK/CHU
TSC2/DDIT4/POLDIP3/SGK1/ATG13/YWHAZ/RRAGB/YY1/EEF2/EIF4B/BNIP3/KRAS/PRR5/YWHA
PIK3R1/NRG4/ERBB4/NRG3
SOS1/YWHAZ/GSK3B/GRB2/FOXO3/TRPV1/KRAS/SRC/YWHAB/PIK3R1/EGR1/NTRK1/PDPK1/A
KRAS/SRC/PLA2G12A/NOS3/PIK3R1/PLA2G6/PIK3CG/NFATC2/PIK3R5/PTK2/CHP1/PLA2G4E/C
PTPN6/SOS1/GRB2/INPP5D/IL2RA/PIK3R1/JAK2/SOS2/CSF2RA/IL5/JAK1/SYK/GAB2/CSF2RB/JA
SSPO/ADAMTSL1/SBSPON/ADAMTS2/THSD7B/THBS1/ADAMTS17/ADAMTS4/ADAMTS12/SPO
H2BFS/HIST1H4J/HIST1H4C/HIST1H4A/HIST1H2BJ/HIST1H2BL/H2AFX/BRCA1/HIST1H2BK/BAR
CDK4/CDC25C/CDK1/WEE1/CDK2/CHEK1/CDC25A/TP53/CDC25B
AREG/MET/FGF7/SRC/PRR5/CD28/PIK3R1/FGF4/TGFA/FRS2/MTOR/FGF20/PIK3AP1/VAV1/IRS1
TAB1/CREBBP/FRAT1/RBPJ/GSK3B/DVL1/LRP6/FOS/MYC/FBXW11/NLK/MAPK3/SSPO/MAPK1/
KRAS/SRC/YWHAB/FGB/APBB1IP/BRAF/TLN1/JAK2/CNKSR1/VCL/KSR1/MAPK3/PEBP1/MAPK1/
PON3/AMACR/CPT1A/SCD5/ACOT6/PCCA/ACOXL/ALOX15/HSD17B4/CYP2C9/SLC25A1/PTGE
POMC/CYB5R3/RXRA/CYP2W1/CYP8B1/NCOA1/CYP2A6/CYP4F2/CYP4F3/PAOX/PTGIS/CYP3A
DYNC2H1/ADCY9/AVP/AQP3/DYNLL2/PRKACB/DYNC1LI2/DCTN1/PRKX/DYNC1H1/VAMP2/N
EXO1/PCNA/RPA3/LIG1/POLD1/POLD3/RPA2/POLD2/MLH1/MSH2/MSH6
GRB2/FGF1/PIK3R1/FGF4/FRS2/FGF20/FGF2/PTPN11/FGF17/FGF9/FGF8/FGF18/FGF5/FGF16
TERT/BIRC5/CDK4/CCNB1/BCAT1/MMP9/TK1/CDCA7/CDC25A/LDHA/TFRC/CCND2/NME1/AC
CDC20/UBE2C/NEK2/BUB1B/MAD2L1/ANAPC11/CDC26/UBA52/ANAPC7/UBB/RPS27A/ANAP
TACC3/PSMB8/PSMB9/PSMA2/PSME2/PSMA7/PSMD8/PSMD14/PSMC2/PSMA1/PSMB4/PSMD
RPS3A/RPS5/RPS26/RPS15/RPS7/EIF4EBP1/RPS18/RPS10/RPSA/RPS2/RPS19/RPS21/RPS29/RPS
RPPH1/NUP37/RPP40/CPSF4/NDC1/NUP107/NUP210/POP7/NUP35/ZBTB8OS/NUP85/NUP54/
GRB2/FGF1/KRAS/FRS3/FGF4/FRS2/FGF20/KL/FGF22/FGF2/PTPN11/FGF17/FGF9/FGF8/FGF3/F
PELI2/FOS/TAB2/MAP3K8/CHUK/DUSP3/IRAK3/MAPK8/FBXW11/MAPK3/RPS6KA2/BPI/TLR6/P
PLCB2/TAS1R2/CACNA1A/PRKACB/PRKX/TAS2R4/ADCY8/TAS2R3/KCNB1/PRKACG/GNG3/ASI
CDH10/CDH3/CLDN2/PARD3/SDK2/CDH13/CADM2/CLDN11/CADM3/CDH18/CDH7/CLDN16/
CDH13/CADM2/CADM3/CDH18/CDH7/CDH9/CDH12/CDH8
TRPV4/TRPV3/TRPM1/TRPV5/TRPM2/TRPM3/TRPM6/TRPA1/TRPV6/TRPC5
TAB2/OCLN/PDPK1/BAMBI/WWP1/ZFYVE16/SMAD3/TGFBR2/NEDD4L/SMAD7/ZFYVE9/SPTBN
GCNT1/ST6GALNAC1/GALNT6/GALNT8/GALNT18/GALNT16/ST3GAL1/GALNT13/GALNT14/GA
GPT2/CPS1/GLUL/ALDH4A1/GPT/GFPT2/GAD1/GLUD2/ALDH5A1/GLS/GLUD1/ASPA/GOT1/AC
CTHRC1/FZD9/ROR2/WNT2/RSPO1/WNT7B/WNT3A/WNT1/WIF1
FOS/MAPK8/MAPK3/MAPK1/MAPK9/MAPK10
CSF2RA/ABCA3/CSF2RB/SFTPC/SFTA3/SFTPA2/SFTPD/SFTPA1
ADD1/ADD2/LRRC8A/LRRC8C/TUSC3/NIPAL1/NIPAL2/ANKH/NIPAL3/NIPA1/LRRC8B/NIPAL4/
CACNG2/AKAP9/KCNQ1/CACNA2D1/CACNA1D/CACNB3/CACNB4/CACNA1S/CACNB2/CACN
KPNA1/GSK3A/YWHAZ/GSK3B/FOXO3/SRC/YWHAB/HSP90AA1/MTOR/CHUK/PDPK1/CASP9/
DAB2IP/NF1/RASGRP4/SYNGAP1/RASGRP1/PRKCA/CAMK2B/PRKCZ/PRKCE/RASAL1/RASGRF2
KRAS/PPP2R5B/SRC/YWHAB/BRAF/JAK2/KSR1/PPP2R5C/MAP2K1/CALM1/PPP2R5A/CAMK2G/
PRKAR2B/ADCY9/AVP/GNB5/AQP3/PRKACB/RAB11FIP2/ADCY7/PRKAR1B/ADCY5/ADCY8/AD
PSMB8/PSMB9/PSMA2/PSME2/PSMA7/PSMD8/PSMD14/PSMC2/PSMA1/PSMB4/PSMD13/PSM
TIAM2/ARHGEF7/DEF6/RAP1GDS1/VAV1/BCR/PREX2/ELMO1/CHN2/DOCK2/ABI1/DOCK1/TIA

FOS/GNAQ/MAPK8/CALM1/PRKCA/PTK2B/CALM3/CXCL12/PRKCB
PIK3C3/MTMR4/MTMR12/PIK3C2A/PIK3R4/PIKFYVE/MTM1/MTMR10/FIG4/INPP4B/PI4K2A/INP
PPRC1/RXRA/NCOA1/NR1D1/PPARGC1B/NCOA2/CRTC1/PRKAA2/CALM1/PRKAG2/MEF2D/GL
KRAS/FGF7/PIK3R4/PIK3R1/FGF4/ATP6V0A4/FRS2/FGF20/ATP6V1D/ATP6V1E1/IRS1/ATP6V0C/
SNRPE/SNRPG/SNRPD2/LSM5/LSM7/POLR2L/SNRPB/BUD31/LSM2/LSM4/SNRPC/PPIH/POLR2
ITGAM/SELP/SELPLG/SELL/TNF
AP2A1/AP2A2/NTRK1/CLTC/AP2B1/NGF/DNM3/SH3GL2/DNM1
FUT6/CHP1/EPM2A/PAPSS2/GPC5/RANBP2/B3GAT1/PYGB/PFKM/MDH1/SORD/GPC3/PCK1/KE
MAPK3/SPDYC/RPS6KA2/PPP2R5C/MAPK1/PRKACB/MAP2K1/YWHAG/PRKX/CALM1/PPP3R1/S
ACHE/MAOA/SLC22A2/ALDH2/SLC6A3
SLC17A6/SLC25A22/SLC25A18/SLC5A5/SLC5A8/SLC17A7
GRB2/FGF1/KRAS/FRS3/PIK3R1/FGF4/FRS2/FGF20/FGF2/PTPN11/FGF17/FGF9/FGF8/FGF18/FGF
E2F7/CCNA2/E2F8/CDK2/E2F1/TP53
GNG2/PELP1/GNB1/SRC/PIK3R1/FOS/PTK2/PLCB2/MAPK3/GNAZ/MAPK1/PLCG2/MAP2K1/GN
P4HA2/PRODH/ALDH9A1/ARG2/CPS1/OTC/GLUL/ALDH4A1/MAOA/AGMAT/OAT/GLUD2/GLS
ATP2A3/SLC8A1/ATP2A2/SLC8A3/CALM1/ATP2B1/ATP2B2/SLC8A2/ATP2B3
HNRNPA1P60/SNRPE/SNRPG/SNRPD2/LSM5/LSM7/SNRPB/BUD31/LSM2/LSM4/SNRPC/PPIH/S
PAG1/PTPN6/SOS1/IBTK/GRB2/RASA1/PTPRC/PIK3R1/DAPP1/FOS/BLNK/CHUK/PDPK1/POU2F
CCNB2/CCNB1/CDK1/TUBA1C/GTSE1/TUBA3C/PLK1/TUBA3E/PSMB8/PSMB9/PSMA2/TUBA1A
CCNB2/CCNB1/CDK1/PLK1
GRB2/FGF1/KRAS/FRS3/FGF4/FRS2/FGF20/FGF2/PTPN11/FGF17/FGF9/FGF8/FGF18/FGF5/FGF1
CDKN2C/CDK4/CDK6/E2F2/CDKN2A/E2F1/CKS1B/CCND2/RBL1/SKP2
RXRA/PDPK1/ACSL1/CYP8B1/FADS2/ACSL4/PCK1/FABP3/SLC27A6/ACSL6/ME1/SORBS1/PPAR
NUMB/MET/SRC/PIK3R1/RPTOR/CRKL/EGR1/SNAI1/MTOR/CBL/PTK2/PDPK1/MAPK8/RAPGEF1
PCNA/RFC2/RPA3/RFC4/RFC3/RPA2/UBA52/UBB/POLH/RPS27A
PCNA/RFC2/RPA3/RFC4/RFC3/MAD2L2/RPA2/UBA52/UBB/RPS27A
MYH10/EPHA5/EFNA3/MYH14/EPHA4/EFNA5/EPHA10/EPHA7/MYH11/NGEF/EPHA8/EPHA6
GRB2/FGF1/SRC/FGF4/BRAF/MKNK1/FRS2/FGF20/CBL/MAPK3/MAPK1/FGF2/PTPN11/FGF17/F
GNAQ/MAPK8/MAPK3/MAPK1/MAP2K4/MAP2K1/CALM1/PRKCA/PTK2B/CALM3/PAK1/PRKCB
GRB2/FGF1/SRC/FGF4/BRAF/MKNK1/FRS2/FGF20/CBL/KL/MAPK3/MAPK1/FGF22/FGF2/PTPN1
BLM/CDKN2A/NUP37/EID3/BRCA1/SP100/NDC1/NUP107/NUP210/CBX2/NSMCE2/NUP35/NU
GNRHR/CGA/TSHR/TSHB/FSHR/FSHB
SNRPE/SNRPG/SNRPD2/SNRPB/SNRPC/SNRPD1/SNRPB2/SNRPF/SNRPA1/SRSF2/U2AF1/SNRP
MED1/SC5D/NFYB/CREBBP/FDFT1/NFYA/ACACB/TBL1X/MTF1/HMGCS1/RXRA/IDI1/SREBF2/N
CYTH3/ASAP1/CLTB/GPLD1/ARAP1/ASAP2/ARFGEF1/CYTH4/ARAP2/CYTH1/ARFGEF2/GBF1
SOS1/GRB2/RASA1/PIK3R1/PIK3CG/FOS/IRS1/MAPK8/MAPK3/MAP2K1/PTPN11/SLC2A4
PIK3R1/PIK3CG/VAV1/MYL2/CDK5R1/PLD1/WASF1/PPP1R12B/PAK1/CHN1
EP300/PRKACB/RPS6KA5/PAK3/CD209/PAK1/PRKACG
IGHV3-33/IGLV1-51/IGKV3-20/IGKV4-1/IGLV3-19/IGLV2-23/C3AR1/IGLC3/IGKV1-17/IGLV3-2
SOS1/YWHAZ/GRB2/KRAS/PIK3R1/JAK2/FOS/CSF2RA/MAPK3/SYK/GAB2/CSF2RB/MAPK1/PRK
SC5D/NFYB/CREBBP/FDFT1/SEC23A/NFYA/ACACB/TBL1X/MTF1/HMGCS1/RXRA/IDI1/SREBF2/N
MYBL2/CCNA2/CDK1/E2F1/RBL1/LIN9
CCNB2/CCNB1/CDK1/NUP37/NEK6/NDC1/NUP107/NUP210/NUP35/NUP85/NUP54/NUP205/N
ACTA1/SLC9A3/ROCK2/TLN1/FOS/ROCK1/VCL/PIP5K1C/MAPK8/EZR/PTEN/SCAI/MAP2K4/LIM
MAN2A1/RGP1/CYTH4/GOLIM4/MAN1A2/RAB41/TRIP11/NAPG/MAN2A2/VTI1A/CYTH1/MAN
PIK3R1/GNA13/PIK3CG/GNAQ/ROCK1/PRKCA/PTK2B/PPP1R12B/PLCB1/ADCY1/PRKCB
RASA1/KRAS/SRC/PDPK1/PRKCD/CALM1/PRKCA/ITPR2/AHCYL1/PRKCZ/ITPR1/PRKCB
ALDH1A1/AKR1C3/CYP26C1/ALDH1A2/DHRS9/ALDH1A3/CYP26A1/CYP26B1/SDR16C5
POLE2/PCNA/RFC2/RPA3/RFC4/RFC3/POLR2L/POLR2J/POLD1/POLR2H/POLD3/POLR2G/GTF2
NOTCH2/SSPO/ADAMTSL1/SBSPON/ADAMTS2/MUC5B/THSD7B/MUC6/THBS1/ADAMTS17/A
TOP2A/CDC6/PCNA/E2F1/CDC25A/RBL1/LIN9
SNRPE/SNRPG/SNRPD2/POLR2L/SNRPB/LSM2/POLR2J/SNRNP40/POLR2H/POLR2G/POLR2I/SN
CREBBP/PRKAR1A/RXRA/PRKAR2B/EP300/PRKACB/PRKAR1B/PRKACG
HIST2H2AA3/HIST2H2AA4/HIST1H4J/HIST2H3C/HIST1H3J/HIST1H2AI/HIST2H2AC/HIST1H2AD
AC003665.1/AMACR/HSD17B4/OSBPL2/AKR1C4/RXRA/CYP8B1/NCOA1/PTGIS/BAAT/OSBPL1A
UGT2B17/UGT2B15/UGT2B7/UGT2B4/RPEL1/GUSB
MMP1/POU2F1/FOS/POMC/IL5/NR1I3/SMARCC2/MAPK8/EP300/NCOA1/MAPK3/MAPK1/NCO
PDPK1/AKT3/PTEN/MAPK3/MAPK1/PIK3CD/PTK2B/IPCEF1
GRB2/FGF1/KRAS/SRC/FRS3/PIK3R1/FGF4/BRAF/MKNK1/FRS2/FGF20/CBL/KL/MAPK3/MAPK1/

CDC25C/CCNB1/CDK1/CDC25A/SHH/CDC25B
SOS1/GRB2/RASA1/PIK3R1/PIK3CG/FOS/IRS1/MAPK8/MAPK3/MAP2K1/PTPN11/IGF1
FGF1/FGF4/FGF20/KL/FGF22/FGF2/FGF17/FGF9/FGF8/FGF3/FGF5
CDC20/UBE2C/CCNB1/CDK1/ANAPC11/CDC26/UBA52/ANAPC7/UBB/RPS27A/ANAPC10/CDC1
ARHGEF28/ACTR2/RASA1/ROCK2/EFNB3/SRC/PTK2/ROCK1/LIMK2/EPHB1/TIAM1/ITSN1/KALRI
NFATC2/FOS/VAV1/MAPK8/MAPK3/SYK/MAP2K1/CALM1/PRKCA/CALM3/PPP3CA/PPP3CB/PR
GSK3B/PORCN/PPP2R5B/DKK4/LRP6/AMER1/TCF7L2/CTBP2/PPP2R5C/TNKS2/APC/RNF43/PPP
RRM2/TYMS/TK1/POLE2/DTYMK/ENTPD8/DPYD/UPP1/POLR2L/NME1/PRIM1/PRIM2/POLA2/P
SOCS2/HIST2H3C/HIST1H3J/SOCS1/IL7/HIST1H3D/CISH/RAG1
UBE2C/CCNB1/CDK1/PLK1
CYP2W1/CYP4F2/CYP4F3/CYP3A43/CYP4A22/CYP4A11
CDC25C/CCNB1/CDK1/WEE1/CHEK1/CHEK2
ALPL/LYPD6B/LYPD3/LYPD5/MSLN/LSAMP/GP2/NTNG2/MDGA2/RTN4RL2/NTM/ART4/SPRN/L
GRB2/FGF1/KRAS/FGF4/FGF20/FGF2/FGF17/FGF9/FGF8/FGF18/FGF5/FGF16
SOS1/IL6/GRB2/JAK2/FOS/MAPK3/JAK1/JAK3/IL6R/MAP2K1/PTPN11
SGPL1/GNA13/GNAQ/GNA15/GNAZ/S1PR1/GNA14/S1PR5/GNAI1/GNAO1
MAPK3/C9/C7/NCAM1/MAPK1/PRKACB/MAP2K1/NCAM2/PRKX/PRNP/IL1B/C6/PRKACG
E2F7/BIRC5/CDC25C/CCNB1/IGFBP3/CCNA2/AURKB/CDK1/E2F8/EXO1/TPX2/AURKA/FANCI/BI
CDK4/CDK2/PCNA/E2F1/GADD45A/TP53/BAX/MDM2/CCNE1
TMF1/VPS52/VAMP4/COG1/USP6NL/RGP1/NAA30/RHOBTB3/RAB6A/TGOLN2/RAB9B/GOLGA
NUDT4/IP6K1/IPPK/NUDT11/NUDT3/PPIP5K1/IP6K3/ITPK1
GRB2/RASA1/SRC/PIK3R1/GFRA1/FRS2/IRS1/PTK2/GDNF/MAPK8/MAPK3/MAPK1/PTPN11/PRK
CDC25C/BLM/FANCD2/CDC25A/RBBP8/H2AFX/CHEK2/BRCA1
PRKACB/PFKFB3/PFKFB2/PRKACG
FOS/TNFRSF1B/CHUK/MAPK8/BCL2/MAPK3/MAPK1/MAP2K4/PRKCD/MAP2K1/PRKCA/PRKCQ/
ANTXR1/MAPK3/MAPK1/MAP2K4/MAP2K1/IL1B/PGR/TNF
SOS1/GRB2/FGF1/KRAS/FGF4/FGF20/KL/FGF22/FGF2/FGF17/FGF9/FGF8/FGF3/FGF5
AP2M1/FZD4/CLTB/ARRB2/AP2A1/WNT5A/AP2A2/CLTC/AP2B1/PRKCA/PRKCB/PRKCG
RASA1/EFNB3/KRAS/SRC/PIK3R1/PTK2/ROCK1/MAPK3/MAP4K4/MAPK1/MAP2K1/EPHB1/EFNA
POLR2F/FGF22/FGF2/FGF17/FGFR2/FGF9/FGF8/FGF3/FGF18/FGF5/FGF16
TLR8/FOS/TAB2/MAP3K8/CHUK/DUSP3/IRAK3/MAPK8/FBXW11/MAPK3/RPS6KA2/BPI/TLR7/TL
CA4/MDH1/PCK1/ATP1A4/SLC4A4/ATP1A2/GLUD2/GLS/GLUD1/ATP1B1/ATP1A3/GLS2
AREG/MET/FGF7/SRC/CD28/PIK3R1/FGF4/TGFA/FRS2/FGF20/PIK3AP1/VAV1/IRS1/KL/STRN/ERI
PIK3R1/RPTOR/PIK3CG/CAB39/BRAF/MTOR/PIK3R5/ULK1/TSC1/PDPK1/AKT3/MAPK3/RPS6KA2
ACTR1A/TUBB8/CD55/ARF3/GORASP1/DYNLL2/DYNC1LI2/SPTAN1/DCTN1/ANK1/GBF1/SPTBN
HIST2H2AA3/HIST2H2AA4/HIST1H2AI/HIST2H2AC/HIST1H2AD/HIST2H2AB/HIST1H2AG/BRCA1
SOS1/GRB2/RASA1/PIK3R1/PIK3CG/JAK2/THPO/FOS/MAPK3/MPL/MAP2K1/PRKCA/PRKCB
ALOX15/CYP2C9/PTGES/CYP8B1/PTGR2/CYP4F2/CYP4F3/PTGIS/CYP2J2/DPEP3/AKR1C3/FAAH
FUT6/ST6GALNAC6/B3GALT2/ST3GAL6/FUT5/FUT1/FUT3/FUT9
ST8SIA2/MGAT4A/ST6GAL1/MGAT5/B4GALT1/B4GALT6/ST8SIA6/ST8SIA3/MGAT4C
KIF20A/PLK1
SNRPE/SNRPG/DDX39A/SNRPB/LSM10/MAGOHB/CPSF4/MAGOH/ALYREF/ZNF473/LUZP4/TH
CDC20/UBE2C/BUB1B/MAD2L1
COL3A1/GDNF/COL4A5/CACNA1G/NCAM1/CACNA1H/CACNA1D/CACNB3/COL6A6/PRNP/CC
RPS7/NOP10/RPS2/PNO1/RPS14/DDX49/TRMT112/NOL11/DDX47/UTP15/RRP9/NOP56/IMP3/
ESCO2/CDCA5
CCR6/DEFB112/DEFB4A/TLR2/DEFB110/CCR2/DEFB1/DEFB124/DEFB119
ASAH1/PIK3R1/PIK3CG/MAPK3/MAPK1/PRKCA/S1PR1/SPHKAP/PLCB1/ADCY1/PRKCB
BCAR1/SOS1/F2/GRB2/COL1A1/SRC/FGB/APBB1IP/TLN1/THPO/PTK2/PDPK1/SYK/MPL/GP1BA/
CLDN2/PARD3/LAMA3/DST/SDK2/CDH13/KRT14/PLEC/CADM2/CLDN11/CADM3/CDH18/CDH
CYP4F2/CYP4F3/CYP2J2/CYP4F12/CYP2F1/CYP4A22/CYP2A7/CYP4A11
BIRC5/IGFBP3/STEAP3/CASP6/CASP1/TRIAP1/TP73/CASP2/TP53/BAX/PRELID1/TP53AIP1/TP63/
KRAS/PIK3R1/PIK3CG/FXYD2/PIK3R5/IRS1/PDPK1/MAPK3/NEDD4L/ATP1A4/MAPK1/PIK3CB/PII
CENPA/BIRC5/DLGAP5/AURKB/TACC3/TPX2/AURKA/GADD45A/BRCA1/TP53/CDC25B/MDM2
IGHV3-33/IFITM1/IGLV1-51/IGKV3-20/LILRB2/LILRB1/IGKV4-1/IGLV3-19/CD8A/COL3A1/SIGLE
UGT2B17/UGT2B15/UGT2B7/UGT2B4
VEGFA/CA9/EPO/PSMB8/PSMB9/PSMA2/PSME2/LIMD1/PSMA7/PSMD8/PSMD14/PSMC2/PSM
NFATC2/WNT5A/DVL1/TAB2/DAAM1/ROCK1/MAPK8/NLK/CTHRC1/MAPK9/MAPK10/ROR2/PF
ITGAM/SELP/SELE/SELL

LMNB1/TMPO/BANF1/LMNA/VRK1
PPRC1/RXRA/NCOA1/NR1D1/PPARGC1B/NCOA2/CRTC1/CALM1/MEF2D/GLUD2/GLUD1/USP4
YWHAB/PIK3R1/SOS2/LNPEP/CBL/IRS1/PDPK1/AKT3/PTEN/MAPK3/RPS6KA2/PARD3/MAPK1/
PIK3R1/CRKL/JAK2/MTOR/PIAS1/CBL/RAPGEF1/EP300/MAPK3/JAK1/MAPK1/SMAD7/PRKCD/M
PSMB8/PSMB9/PSMA2/ODC1/SMS/PSME2/PSMA7/PSMD8/PSMD14/PSMC2/PSMA1/SAT1/PSM
PSMB8/PSMB9/PSMA2/FZD7/FZD5/PSME2/PSMA7/PSMD8/PSMD14/PSMC2/PSMA1/PSMB4/P
GSK3A/SOS1/YWHAZ/PREX1/GSK3B/GRB2/FOXO3/MET/YWHAB/SOS2/MTOR/PIK3R5/IRS1/PT
GCK/NUP37/NDC1/NUP107/NUP210/NUP35/NUP85/NUP54/NUP205/NUP160/NUPL2/RAE1/A
ST8SIA2/MAN2A1/MGAT4A/MAN2A2/ST6GAL1/MGAT5/B4GALT1/B4GALT6/ST8SIA6/FUT3/M
GRB2/FGF1/ZMYM2/FGFR1OP2/PIK3R1/FGF4/FGF20/BAG4/BCR/GAB2/FGF2/FGF17/MYO18A/
PIK3R1/PIK3CG/TLN1/ROCK1/MAPK3/GSN/MAPK1/MAP2K1/MYL2/ARHGAP5
NUP37/NDC1/NUP107/NUP210/CBX2/NUP35/NUP85/NUP54/NUP205/NUP160/NUPL2/RAE1/
NUP37/RCC1/NDC1/NUP107/NUP210/NUP35/NUP85/NUP54/NUP205/NUP160/NUPL2/RAN/
GRB2/FGF1/ZMYM2/FGFR1OP2/KRAS/FGF7/PIK3R1/FGF4/FRS2/POLR2A/FGF20/BAG4/BCR/GA
CCNB2/CCNB1/CDK1/PLK1/LMNB1/LPIN3/NUP37/TMPO/NEK6/NDC1/NUP107/NUP210/NUP3
KRAS/BRAF/FOS/MAP3K8/MAPK3/MAPK1/MAP2K1/PRKCA/PRKCB
WNT5A/WNT9A/RAB23/BMP8A/BMP8B/BMP4/BMP6/FBXW11/WNT11/PRKACB/SUFU/PRKX/W
TUBAL3/DNAJA1/NR3C1/TUBB1/HSP90AA1/HSPA1L/TUBB2A/ACTR1A/TUBB8/DYNLL2/DYNC1
LPAR1/AKAP13/PRKAR2B/PRKACB/PRKACG/LPAR3
HAPLN2/VWCE/NDNF/COL21A1/LTBP2/HAPLN1/COL3A1/COL14A1/CRIM1/VWA2/IGFBP6/AM
TRPV1/KRAS/EGR1/BRAF/RUSC1/FOS/MAPK3/MAPK1/PRKCD/MAP2K1/CDK5R1/RPS6KA5/MEF
WNT5B/WNT8A/WNT4/WNT8B/WNT2B/WNT2/WNT9B/WNT7B/WNT3A/WNT1/WNT10B
NUP37/POLR2L/POLR2J/POLR2H/NDC1/NUP107/NUP210/POLR2G/NUP35/NUP85/NUP54/PO
LINGO1/RTN4/NGF/OMG/MAG/RTN4R/MCF2
ARHGEF28/ARHGAP4/AKAP13/ARHGEF10/DEF6/VAV1/ARHGEF11/BCR/ARHGEF10L/ARHGEF1
DSCAM/DCC/MAPK8/DSCAML1/MAPK13/PAK1
FANCI/CHEK1/FANCD2/FANCC/POLR2L/BRCA1/TP53/POLR2J/POLR2H/POLR2G/GTF2H2/DDB
PPIA/APOBEC3G/PSMB8/PSMB9/NUP37/HLA-A/PSMA2/AP1S3/AP1S2/RCC1/NDC1/NUP107/
NCOR2/ZNF638/CDK19/TBL1X/LPL/KLF5/RXRA/EP300/SREBF2/NCOA1/MED13L/PCK1/SREBF1/
EGFR/H2BFS/HIST2H2AA3/HIST2H2AA4/HIST1H4J/HIST2H3C/HIST1H3J/MMP9/HIST2H2AC/CI
GRB2/FGF1/ZMYM2/FGFR1OP2/KRAS/PIK3R1/FGF4/FRS2/FGF20/BAG4/BCR/GAB2/FGF2/FGF17
NDEL1/CLIP1/PLA2G7/DAB1/PAFAH1B1/VLDLR/MAP1B/CDK5R1/DYNC1H1/CDK5R2/RELN

/CDC25C/RRM2/CDC20/UBE2C/NDC80/HIST1H4J/NCAPG/CCNB2/CENPU/NEK2/SKA1/CCNB1/
/HIST1H4J/NCAPG/CCNB2/CENPU/NEK2/SKA1/CCNB1/NUF2/AURKB/KIF18A/KIF20A/HIST2H3C
NCI/RAD51AP1/BRIP1/RMI2/POLE2/HIST2H2AC/XRCC2/CLSPN/POLQ/CDK2/PCNA/CHEK1/BRC
/MRPL11/RPS15/RPL35/RPL9/MRPS15/RPL18A/MRPL32/RPS7/RPL26L1/EIF4EBP1/RPS18/RPL41/
I/SKA1/CCNB1/NUF2/CCNA2/AURKB/KIF18A/CDC45/KIF2C/CDCA8/BUB1/CDK1/EXO1/SPC25/V
S15/RPL35/RPL9/RPL18A/RPS7/RPL26L1/MAGOHB/RPS18/RPL41/RPS10/PSME2/RPL36/MAGOH
A/KIF2C/CDCA8/BUB1/TUBA1C/SPC25/ERCC6L/CENPE/CENPF/BUB1B/CENPM/ESPL1/TUBA3C/I
/MCM2/CDK2/PCNA/CDT1/E2F1/RFC2/RPA3/CKS1B/CDC25A/RFC4/PSMB8/RFC3/PSMB9/MCM7/
H3C/HIST1H3J/HIST1H1B/IFNB1/CDK6/EZH2/HIST2H2AC/E2F2/CDK2/LMNB1/HIST1H2AD/CDKN
2/AURKB/KIF18A/KIF2C/CDCA8/BUB1/CDK1/TUBA1C/SPC25/ERCC6L/CENPE/NCAPH/CENPF/BU
OP10/RPL26L1/RPP40/RPS18/RPL41/RPS10/RPL36/RPSA/RPS2/PNO1/RPS19/EXOSC8/RPL21/RP
L35/RPL9/RPL18A/RPS7/RPL26L1/POLR2J/CPSF4/RPS18/POLR2H/NDC1/RPL41/NUP107/RPS10/
DC6/CDK6/POLE2/ORC6/E2F2/MCM2/CDK2/PCNA/DBF4/MCM10/CDT1/CDKN2A/E2F1/RPA3/C
OLQ/CDK2/PCNA/CHEK1/BRCA2/EME1/BLM/HIST1H4C/HIST1H4A/RFC2/HIST1H2BJ/RPA3/HIST
2/CDC6/PKMYT1/ORC6/CLSPN/MCM2/CDK2/DBF4/CHEK1/MCM10/BLM/HIST1H4C/HIST1H4A/
8/BUB1/TUBA1C/SPC25/ERCC6L/CENPE/CENPF/BUB1B/CENPM/TUBA3C/PLK1/MAD2L1/CENPH
H2AC/CDK2/LMNB1/BRCA2/HIST1H2AD/BLM/HIST1H4C/HIST1H4A/HIST1H2BJ/RPA3/HIST1H2B
1/EIF4EBP1/RPS18/RPL41/RPS10/RPL36/RPSA/RPS2/RPS19/RPL21/RPS21/RPS29/RPL27/RPS14/
8A/KIF2C/CDCA8/BUB1/CDK1/TUBA1C/SPC25/ERCC6L/CENPE/CENPF/BUB1B/CENPM/TUBA3C/
F4/MCM10/CDT1/E2F1/RFC2/RPA3/RFC4/PSMB8/RFC3/PSMB9/MCM7/GINS4/MCM4/MCM6/PS
L/ORC1/BUB1B/CDC6/ESPL1/CDK6/PLK1/MAD2L1/PKMYT1/ORC6/E2F2/MCM2/CDK2/PCNA/DE
CDK2/PCNA/CHEK1/BRCA2/EME1/BLM/HIST1H4C/HIST1H4A/RFC2/HIST1H2BJ/RPA3/HIST1H2BI
/RPL26L1/RPS18/AHCY/RPL41/RPS10/RPL36/INMT/RPSA/RPS2/RPS19/MARS/RPL21/RPS21/RPS
7/RPL26L1/RPS18/RPL41/RPS10/SSR2/RPL36/DDOST/SRPRB/RPSA/RPS2/RPS19/TRAM1/RPL21/I
1/MAGOHB/RPS18/RPL41/RPS10/RPL36/MAGOH/RPSA/RPS2/RPS19/RPL21/RPS21/RPS29/RPL2
H2AC/PLK1/SMC4/LMNB1/LPIN3/HIST1H2AD/HIST1H4C/HIST1H4A/HIST1H2BJ/HIST1H2BL/HIS
/CDCA8/BUB1/SPC25/ERCC6L/CENPE/CENPF/BUB1B/CENPM/PLK1/MAD2L1/CENPH/CENPI/CE
T1H4C/HIST1H4A/HIST1H2BJ/HIST1H2BL/HIST1H3D/H2AFX/POLR2L/HIST1H2BK/H3F3A/H2AFZ
1/RPS18/RPL41/RPS10/RPL36/RPSA/RPS2/RPS19/RPL21/RPS21/RPS29/RPL27/RPS14/RPLP0/RPS
ST1H4J/HIST2H3C/HIST1H3J/EZH2/HOXC4/HIST2H2AC/MEIS1/HIST1H2AD/HIST1H4C/HIST1H4
T1H4C/HIST1H4A/HIST1H2BJ/HIST1H2BL/HIST1H3D/H2AFX/POLR2L/HIST1H2BK/H3F3A/H2AFZ
RFC2/HIST1H2BJ/RPA3/HIST1H2BL/TIMELESS/RFC4/RFC3/RBBP8/H2AFX/BRCA1/PPP4C/HIST1H2
H3J/CDK6/HIST2H2AC/CDK2/HIST1H2AD/CDKN2A/HIST1H4C/HIST1H4A/HIST1H2BJ/HIST1H2BL
T1H4C/HIST1H4A/HIST1H2BJ/HIST1H2BL/HIST1H3D/H2AFX/NUP37/POLR2L/HIST1H2BK/H3F3A
L/CDC25A/RBBP8/CES1/BRCA1/TP73/RBL1/DHFR/MCM3/UXT/RRM1/CCNE1/E2F6/RANBP1/SER
T1H4C/HIST1H4A/RFC2/HIST1H2BJ/RPA3/HIST1H2BL/RFC4/RFC3/H2AFX/HIST1H2BK/LIG1/H2A
/MCM8/MCM5/MCM3/SKP2/ORC5/PSME2/PSMA7/PSMD8/PSMD14/PSMC2/PSMA1/PSMB4/PS
/RPL41/RPS10/RPL36/RPSA/MRPL13/RPS2/RPS19/RPL21/RPS21/RPS29/RPL27/RPLP0/RPS15A/I
T1H4C/HIST1H4A/HIST1H2BJ/HIST1H2BL/HIST1H3D/H2AFX/POLR2L/HIST1H2BK/H3F3A/H2AFZ
A2/PSME2/ANAPC11/PSMA7/PSMD8/PSMD14/CDC26/PSMC2/PSMA1/PSMB4/PSMD13/PSMB1
T1H4A/RFC2/HIST1H2BJ/RPA3/HIST1H2BL/RFC4/RFC3/RBBP8/H2AFX/CHEK2/BRCA1/HIST1H2BI
H2AC/CDK2/LMNB1/BRCA2/HIST1H2AD/BLM/HIST1H4C/HIST1H4A/HIST1H2BJ/RPA3/HIST1H2B
AD/HIST1H4C/HIST1H4A/HIST1H2BJ/GATA3/HIST1H2BL/MYB/HIST1H3D/PSMB8/PSMB9/H2AFX
PSMA2/MCM8/MCM5/MCM3/SKP2/ORC5/PSME2/ANAPC11/PSMA7/PSMD8/CCNE1/PSMD14/
SMA7/PSMD8/PSMD14/CDC26/PSMC2/PSMA1/PSMB4/PSMD13/PSMB1/UBA52/PSME1/PSMA4
/PSMD14/CDC26/PSMC2/PSMA1/PSMB4/PSMD13/PSMB1/UBA52/PSME1/PSMA4/ANAPC7/UBI
1/POLE2/HIST2H2AC/CENPH/PCNA/CENPI/HIST1H2AD/HIST1H4C/HIST1H4A/RFC2/HIST1H2BJ/
/PSMB9/MCM7/MCM4/MCM6/PSMA2/MCM8/PRIM1/PRIM2/POLA2/CDC7/MCM5/MCM3/ORC
2/FBXO5/PSMB8/PSMB9/PSMA2/SKP2/PSME2/ANAPC11/PSMA7/PSMD8/PSMD14/CDC26/PSM
CM8/MCM5/MCM3/ORC5/PSME2/PSMA7/PSMD8/PSMD14/PSMC2/PSMA1/PSMB4/PSMD13/PS
PA3/RFC4/RFC3/RBBP8/GEN1/BRCA1/BARD1/POLD1/SLX1A/RAD9B/POLD3/SLX1B/POLE4/XRC
D/HIST1H4C/HIST1H4A/HIST1H2BJ/HIST1H2BL/HIST1H3D/H2AFX/TLE2/HIST1H2BK/H3F3A/H2/
1H4C/HIST1H4A/RFC2/HIST1H2BJ/RPA3/HIST1H2BL/RFC4/RFC3/H2AFX/PRIM1/HIST1H2BK/LIG
ST1H2AD/E2F1/HIST1H4C/HIST1H4A/HIST1H2BJ/HIST1H2BL/MYB/HIST1H3D/H2AFX/HIST1H2BI
T1H4C/HIST1H4A/HIST1H2BJ/HIST1H2BL/HIST1H3D/H2AFX/POLR2L/HIST1H2BK/H3F3A/H2AFZ
LH2AD/HIST1H4C/HIST1H4A/HIST1H3D/HIST2H2AB/H2AFX/HIST1H2AG/ACTL6A/H2AFZ/HIST1
LMNB1/HIST1H2AD/HIST1H4C/HIST1H4A/HIST1H2BJ/HIST1H2BL/HIST1H1E/HIST1H1C/H2AFX/I
H2AC/CDK2/BRCA2/HIST1H2AD/BLM/HIST1H4C/HIST1H4A/HIST1H2BJ/RPA3/HIST1H2BL/HIST1

'2H2AC/CENPH/CENPI/HIST1H2AD/HIST1H4C/HIST1H4A/HIST1H2BJ/HIST1H2BL/CENPL/ITGB3I
GAP1/DES/VIM
D/HIST1H4C/HIST1H4A/HIST1H2BJ/HIST1H2BL/HIST1H3D/H2AFX/HIST1H2BK/H3F3A/H2AFZ/H
DC25A/RFC4/RFC3/MCM7/MCM4/MCM6/MCM8/CDC7/MCM5/MCM3/RAD9B/ORC5
PE/AURKA/BUB1B/PLK1/CLSPN/PRC1/ECT2/FBXO5
LK1/SMC4/HIST1H2AD/HIST1H4C/HIST1H4A/HIST1H2BJ/HIST1H2BL/HIST1H3D/H2AFX/NCAPG
D/HIST1H4C/HIST1H4A/HIST1H2BJ/HIST1H2BL/HIST1H3D/H2AFX/HIST1H2BK/H3F3A/H2AFZ/H
RIM2/POLA2/MCM5/POLD1/MCM3/SSBP1/POLD3/POLE4/FEN1/RPA2/POLD2/POLE/DNA2/PO
LESS/CDC25A/RFC4/RFC3/MCM7
ONECUT1/CDK2/BRCA2/CDKN2A/CKS1B/ETV5/CHEK2
CM6/MCM8/PRIM1/PRIM2/POLA2/CDC7/MCM5/MCM3/ORC5/POLE4/RPA2/POLE/POLE3
SLX1A/SLX1B/XRCC3/DNA2
T1H4C/HIST1H4A/HIST1H2BJ/HIST1H2BL/HIST1H3D/H2AFX/HIST1H2BK/H3F3A/H2AFZ/HIST1H
T1H4C/HIST1H4A/HIST1H2BJ/HIST1H2BL/HIST1H3D/H2AFX/HIST1H2BK/H3F3A/H2AFZ/HIST1H
3/POLE4/FEN1/RPA2/POLD2/POLE/DNA2/POLE3/RUVBL1
4A/HIST1H2BJ/HIST1H2BL/H2AFX/HIST1H2BK/H2AFZ/HIST1H2AC/HIST1H2BD/HIST1H4H/POT
P8/BRCA1/BARD1/RAD9B/XRCC3/RPA2/DNA2/HUS1/RHNO1
/POLD2
4A/HIST1H2BJ/HIST1H2BL/H2AFX/HIST1H2BK/H2AFZ/HIST1H2AC/HIST1H2BD/HIST1H4H/POT
M8/PRIM1/LIG1/PRIM2/POLA2/MCM5/POLD1/MCM3/POLD3/FEN1/RPA2/POLD2/DNA2
HFR

/RPA2/POLD2/POLE/DNA2/POLE3

DNA2
OLE3

M4/SNRPC/PPIH/POLR2J/SNRNP40/MAGOHB/CPSF4/POLR2H/NDC1/NUP107/NUP210/PTBP1/I

5/SYT1

SE1/AURKA/CENPF/TUBA3C/PLK1/PKMYT1/PLK4/CDK2/E2F1/HAUS1/HAUS8/TUBA3E/CDC25A/

1A/RAB3A/SNAP25/SYT1

25/SYT1
RKCG
OP10/RPL26L1/RPP40/RPS18/HSD17B10/RPL41/RPS10/RPL36/RPSA/RPS2/PNO1/RPS19/EXOSC

KCB/GRIN1/GRIN2B/GRIN2A

/GRIN2A

/CAMK2A

DLG2/LRRC7/GRIN1/GRIN2B/GRIN2A/CAMK2A
PIK3C2G
LC32A1/RAB3A/GAD2/SNAP25/SYT1

ACG
IK2A
2/SNAP25/SYT1
GN/GRIN2B/GRIN2A/CAMK2A
3A/SNAP25/SLC17A7/SYT1
/MYOD1/CAMK1G
CE/ARRB1/DNM1/PRKCB/PRKCG
G2/LRRC7/RASGRF2/GRIN1/GRIN2B/RASGRF1/CAMK2A
HIST1H2AD/HIST1H4C/HIST1H4A/HIST1H2BJ/HIST1H2BL/HIST1H3D/NPPA/H2AFX/APOA1/HIST
LCB1/PLCH2
/GRIA1/EPB41L1/GRIP1/CAMK2G/CAMK2B/CACNG8/AKAP5/PRKCB/CACNG3/PRKCG/CAMK2A
4/ANK3/SPTBN2/SCN1B/SCN8A/SCN2A/SCN5A/KCNQ3/SPTB/SCN3B/SCN7A/SCN2B
/HRH3/ADRA2A/HTR1A/HTR2A/DRD1/CHRM1/HTR2C/HRH2/CHRM2/HTR1B/HTR1E/HTR5A/D
A/GRIN1/GRIN2B/GRIN2A/CAMK2A
NB1/AKAP5/PRKACG/GNG3/ITPR1/KCNC2/GNG13
CALM3/PPP3CA/PPP3CB/ADCY1/PRKACG/PRKCB
IK2G/CAMK2B/CAMK4/ADCY2/ADCY1/PRKACG/PDE1B/CAMKK1/PDE1A/PRKCG/CAMK2A
/HIST1H4C/HIST1H4A/HIST1H2BJ/HIST1H2BL/HIST1H3D/HIST2H2AB/HIST1H2AG/HIST1H2BK/H
C1/KCNC3/KCNA2/KCNH1/KCNB1/KCNQ3/KCNQ5/KCNA1/KCNH3/KCNAB2/KCNA4/KCNC2/KC

Y8/CAMK2G/CAMK2B/CAMK4/ADCY2/PRKCE/ADCY1/PRKACG/PDE1B/CAMKK1/PDE1A/ITPR1/P
CACNB2/CACNA1F/CACNA1C/CACNA2D2/CACNB1/CAMK2G/CAMK2B/SCN1B/CACNG8/SCN8A
ADCY2/GABBR2/ADCY1/GNG3/KCNJ4/KCNJ3/KCNJ12
T1H4C/HIST1H4A/HIST1H2BJ/HIST1H2BL/HIST1H3D/H2AFX/HIST1H2BK/H3F3A/H2AFZ/CBX3/H
2AFX/BRCA1/HES1/FANCG/XRCC3/RPA2/HUS1/USP1/RMI1/FANCF/FANCE/FANCL
DCY1/ADRA2A/GNG3/GNG13/PRKCB/RGS4/PRKCG
CR/AKT3/BCL2/MAPK3/SYK/MAPK1/PLCG2/PIK3CD/PPP1R13B/ITPR2/PPP3CA/CD22/PPP3CB/C
NA2/HUS1/RHNO1
UBA52/UBB/RPS27A
PLCD3/PLCG2/MTMR9/ITPK1/CALM1/INPP5A/SYNJ1/PLCB4/PLCH1/PLCZ1/ITPKA/INPP5J/PLCB1
LIM1/UNC5C/ABLIM2/UNC5A/UNC5D/PAK1/ABLIM3/SLIT2/SLIT3/TRPC5
/ALDH5A1/APBA1/GLS/SLC1A6/SYN3/SLC1A2/PPFIA3/PPFIA2/SLC6A13/CPLX1/PPFIA4/SLC18A
PI4K2A/INPP4A/PIK3CB/MTMR9/PIK3CD/MTMR8/BMX/PLEKHA6/SYNJ2/PIP4K2A/PLEKHA1/SYN
YT9/EPB41L2/SHANK3/APBA1/DLG4/EPB41L1/BEGAIN/DLGAP1/EPB41L3/GRIN2C/SHANK2/DLG
AP1/GABRG2/PGR/NRG3/GABRG3/GABRA1/GABRB2
D3/PIK3CB/PLCG2/PIK3CD/ITPK1/INPP5A/SYNJ2/PIP4K2A/SYNJ1/PLCB4/ALDH6A1/PLCZ1/PIP5K
/WHAG/PPP3R1/PRKCA/PRKCQ/NR4A1/MEF2D/MAPK9/YWHAH/PPP3CA/PPP3CB/CAMK4/PRK
M2/PRKCD/SLC9A3R1/PRKCA/PRKCQ/GNA14/PTGDR/SELE/PRKCZ/PRKCE/DNM1/PRKCB/PRKCG
P1B1/CAMK2G/CAMK2B/RYR1/ATP1A3/ATP2B2/ITPR1/SLC8A2/NOS1/ATP2B3/RYR2/CAMK2A
PH2/NEFH/TNF/NOS1/GRIN1/GRIN2B/GRIN2A/NEFM
GNAO1/PLCB4/GNAL/ADCY5/CAMKK2/ADCY8/CAMK2G/CAMK2B/CAMK4/PLCB1/ADCY2/ADC
ABRB3/GNAL/ADCY5/GABBR1/KCNJ6/ADCY8/KCNJ9/ADCY2/GABRG2/GABBR2/ADCY1/GABRA2
MOBP/MEF2C/CAMK4/SST/PVALB/GAD2/OPRK1/CRH/GRIN2B/GRIN2A/RBFOX1/OPRM1/CAMK2
3/ATP7B/ATP8B4/FXYD7/CALM1/PLN/ATP2B1/ATP1A2/ATP9A/FXYD4/ATP8A1/ATP4A/ATP1B1/
2/MAPK3/GNAZ/MAPK1/PRKCD/MAP2K1/PRKCA/PRKCQ/GNA14/ADCY7/EDNRB/GNAI1/PTK2B
RIA4/NLGN4Y/LRRTM1/RTN3/HOMER1/LRRTM4/DLG3/SYT12/SLITRK1/NRXN1/SLITRK5/SYT9/EP
D2/KCNG2/KCNH6/KCNS3/KCNN2/KCNH4/KCNMB4/KCNG1/KCND1/HCN2/KCNA5/ABCC9/KCN
7B/WNT3A/ADCYAP1R1/ADCYAP1/CRHR1/PTH/VIPR1/CRHBP/WNT1/PTH2R/GNG3/CRHR2/GLI
PRKAA2/PTPN11/G6PC/NPY/PRKCQ/PRKAG2/MAPK9/LEPR/MAPK10/ACSL6/PPARA/RXRG/ADIP
CNA1D/MYL2/CACNB3/IGF1/TTN/PRKAG2/ITGA8/CACNB4/CACNA1S/MYL3/CACNB2/CACNA1
M3/PLCB4/PPP3R2/GRIA1/PPP3CA/PPP3CB/CACNA1C/PPP1R1A/ADCY8/CAMK2G/CALML3/CA
-23/IGLC3/IGKV1-17/IGLV3-25/IGHV3-30/STIM1/SYK/IGLV1-47/IGLV3-1/IGLV1-40/IGHV1-2/P
IGLV3-25/IGHV3-30/TEC/SYK/IGLV1-47/IGLV3-1/IGLV1-40/IGHV1-2/PLCG2/IGHV3-23/IGHV2-
PRKCA/ITPR2/PRKG1/ADCY7/GNAI1/GJD2/PLCB4/ADCY5/ADCY8/PRKG2/TUBA8/PLCB1/TUBB4

1/FFAR1/KIF5A/INHBA/FSHB/STX1A/CRHR2/GNG13
K3/ADORA2A/CLTC/BDNF/DUSP3/RAPGEF1/MAPK3/RPS6KA2/AP2B1/NGF/MAPK1/PIK3CB/ME
.C6A1/SLC30A2/SLC13A4/SLC30A10/SLC16A7/HEPH/SLC16A8/SLC6A5/SLC22A2/SLC5A11/SLC1
5/GPC3/RPE65/NMT2/APOB/CNGA1/CALM1/APOE/PRKCA/RCVRN/PRKCQ/PNLIP/AKR1C3/RGS
LH4J/CENPU/SKA1/NUF2/AURKB/KIF18A/HIST2H3C/HIST1H3J/KIF2C/CDCA8/BUB1/TUBA1C/SP
KA2/MAP4K4/MAPK1/MAP2K4/MAP2K1/MAPK9/MAPK10/RAPGEF2/MAPK13/RPS6KA5/MAP3K
/MAPK8/AKT3/MAPK3/ERBB3/MAPK1/PIK3CB/PLCG2/MAP2K4/PIK3CD/MAP2K1/PRKCA/NRG4/
AI1/AHCYL1/PRKAR1B/GNAO1/PPP1R1B/PLCB4/PPP3CA/PPP3CB/GNAL/ADCY5/PDYN/CAMKK
N11/APOB/KCNMB4/CALM1/ITPR2/ATP2B1/PDE11A/PRKG1/PPP2R5A/P2RX5/KCNMA1/PRKG2
CK1/PLXNB1/ARHGEF10L/ARHGEF12/MCF2L/FGD4/GNB5/ARHGEF33/FGD2/ARHGEF17/ARHGE
C3/IGKV1-17/IGLV3-25/IGHV3-30/SYK/IGHG1/IGLV1-47/IGLV3-1/IGLV1-40/IGHV1-2/PIK3CB/P
/CLTC/DUSP3/RAPGEF1/MAPK3/RPS6KA2/AP2B1/NGF/MAPK1/PIK3CB/MEF2A/DUSP7/MAPK1
2/PRKCA/ITPR2/IGF1/PRKG1/GNAI1/GNAO1/PLCB4/GRIA1/PLA2G3/PRKG2/CRHR1/PLCB1/RYR1
OS/PIK3AP1/VAV1/BLNK/CHUK/CHP1/AKT3/MAPK3/SYK/NFAT5/MAPK1/PIK3CB/CARD11/PLCG
4A/CDS1/ITPKB/PLCD3/PIK3CB/PLCG2/PIK3CD/DGKZ/CDS2/ITPK1/CALM1/PRKCA/ITPR2/INPP5
KSR1/DAB2IP/RASAL2/MAPK3/NF1/SYNGAP1/CLCN6/PEBP1/KDM7A/MAPK1/TRAK1/MAP2K1/K
R4/PIK3R1/PIKFYVE/PIK3CG/MTM1/MTMR10/PIK3R5/FIG4/SBF2/MTMR3/PIP5K1C/INPP4B/PTEN
D/KCNS3/CACNB3/PRKCA/ITPR2/GNA14/VAMP2/GNAI1/AHCYL1/PRKAR1B/GLP1R/ABCC8/CA
CNA1D/MYL2/PRKX/CACNB3/PLN/IGF1/TTN/ITGA8/ADCY7/CACNB4/CACNA1S/MYL3/CACNB
T-CO2/CACNA1S/MYL3/CACNB2/CACNA1F/MT-CO1/CACNA1C/CACNA2D2/MT-CYB/CACNB1
/CACNA1D/PRKCD/MAP2K1/PRKX/CALM1/PRKCA/ITPR2/MAPK9/MAPK10/ADCY7/MAPK13/PL
CALM1/PRKCA/WNT8A/FZD9/WNT4/ADCY7/WNT8B/WNT2B/EDNRB/GNAI1/CALM3/GNAO1/W
B1/STAM/APOB/CLTCL1/EPS15/LDLRAP1/FCHO1/STON2/DAB2/VAMP2/STON1/SYT9/ITSN1/TF
3/VAV1/PTK2/ROCK1/PDPK1/ELMO2/ELMO1/AKT3/NCKAP1L/AXL/FLT1/RICTOR/PIK3CB/PRKAC
2G4E/AKT3/MAPK3/SCIN/SYK/GAB2/GSN/MAPK1/PIK3CB/PLCG2/LIMK2/PIK3CD/PRKCD/MAP2K
2/MAPK1/PRKACB/PRKAA2/GRIA2/PRKX/DLG3/CALM1/RPS6KA6/PRKAG2/MAPT/KIF17/PRKAR1
CACNA1A/RAPGEF3/PRKACB/KCNG2/PRKAA2/CACNA1D/KCNS3/CACNB3/PRKCA/ITPR2/GNA1
AC3/MUC5B/THSD7B/MUC6/GALNT16/THBS1/ST3GAL1/ADAMTS17/ADAMTS4/ADAMTS12/B4
04/CLCA2/TTYH2/CALM1/TRPV5/TRPM2/SGK2/CLCA4/CASQ2/TRPM3/CLCN4/NALCN/TRPM6/T
A1/SLC1A7/SLC12A2/SLC4A9/SLC9A9/SLC12A1/SLC4A4/SLC6A12/SLC17A6/CALM1/SLC25A22
VAV1/IRS1/KL/PIP5K1C/AKT3/PTEN/MAPK3/STRN/PPP2R5C/ERBB3/PHLPP2/MAPK1/PIK3CB/PIK
DHD1/MBOAT2/PLD3/PCYT1B/DGAT2/PLA2G4E/AGPAT4/MGLL/PLA2G2D/LPGAT1/CDS1/HAD
P2K1/ANK1/DLG3/L1CAM/RPS6KA6/NFASC/SPTBN1/ANK2/SCN11A/RPS6KA5/SCN9A/DLG4/CN
./DST/SDK2/CDH13/KRT14/PLEC/PTK2B/KIRREL3/CADM2/CLDN11/SFTPD/CADM3/NPHS1/CDH
LV3-19/WIPF1/VAV1/PTK2/IGLV2-23/ELMO2/IGLC3/IGKV1-17/IGLV3-25/ELMO1/WIPF2/IGHV3
3/RPS6KA4/BDNF/MAP3K3/IRAK3/PDPK1/MAPK8/RAPGEF1/AKT3/BCL2/MAPK3/RPS6KA2/NGF/
D/HIST1H4C/HIST1H4A/HIST1H2BJ/HIST1H2BL/HIST1H3D/H2AFX/POLR2L/HIST1H2BK/H3F3A/
CY1A2/PLA2G2D/MAPK1/PRKACB/CACNA1D/PRKCD/MAP2K1/PRKX/KCNMB4/CALM1/MYLK/PI
ASA1/NCKAP1/KRAS/SRC/YWHAB/PIK3R1/PIK3CG/BRAF/JAK2/HCK/PIK3R5/FOS/CBL/DOCK4/M
AD/HIST1H4C/HIST1H4A/HIST1H2BJ/HIST1H2BL/HIST1H3D/MIR23B/H2AFX/NUP37/POLR2L/HIS
.H2AD/HIST1H4C/HIST1H4A/HIST1H2BJ/HIST1H2BL/HIST1H3D/HIST2H2AB/HIST1H2AG/ACTL6
P3/NFAT5/MAPK1/SEMA4B/EPHA5/EFNA3/LIMK2/EPHB1/SEMA4G/EPHA4/L1CAM/PPP3R1/EFN
N1/ASH1L/PRKCD/MYH14/MPDZ/MYL2/MAGI3/PRKCA/AMOTL1/PRKCQ/EPB41L2/MYH7B/GNA
RR2A/KRT1/CELA2A/KRT84/PKP4/IVL/PKP3/DSC3/PKP1/KRT14/KRT35/SPRR1A/LCE1E/DSC1/KR
DC25B/PSMA7/PSMD8/CCNE1/PSMD14/PSMC2/PSMA1/PSMB4/PSMD13/PSMB1/UBA52/AKT2/
3/E2F1/HIST1H4C/HIST1H4A/HIST1H2BJ/HIST1H2BL/HIST1H3D/LFNG/H2AFX/HIST1H2BK/H3F3
IGKV3-20/NF2/ABI2/IGKV4-1/PLD3/IGLV3-19/WIPF1/VAV1/PTK2/IGLV2-23/ELMO2/IGLC3/IGK
16/SCN1A/KCNIP4/ATP1A4/KCND2/TRDN/CACNA2D1/CACNA1D/FXYD7/CALM1/CACNB3/ITP
HGAP12/ARHGEF10L/ARHGEF12/MCF2L/FGD4/ARHGAP21/FAM13A/SRGAP3/MYO9A/ARHGEF
54/SMN1/NUP205/NUP160/SNRPD1/SMN2/GEMIN6/NUPL2/SNRPF/WDR77/RAE1/GEMIN2/SN
AX/PRMT1
7/DAAM1/ROCK1/CHP1/MYC/MAPK8/FBXW11/TCF7L2/CTBP2/EP300/NLK/PLCB2/SMAD3/PPP
FNBP1/UBQLN2/AVP/AP2B1/STAM/SNX18/APOB/CLTCL1/BIN1/EPS15/LDLRAP1/FCHO1/STON
UTYH/UNG/POLE3/PNKP/TDG/ADPRHL2/NTHL1
LE/POLE3/UBA52/UBB/POLH/RPS27A
IT-CO2/CALM3/PLCB4/PPP3R2/CACNA1S/PPP3CA/MT-ATP6/PPP3CB/CACNA1F/MT-CO1/CAC
X3CL1/GRP/INSL3/SSTR2/EDNRB/OPRL1/TRHR/CX3CR1/QRFPR/NTSR2/UTS2R/CXCL12/SSTR1/
DCY9/PTAFR/CACNA1G/CHRNA7/EDNRA/PLCB2/P2RX7/HTR2B/ADRB1/CACNA1A/ITPKB/ERBB
AM2/ROCK2/KRAS/PIK3R1/CRKL/PIK3CG/BRAF/CCR2/JAK2/SOS2/CXCL3/HCK/CCL22/PIK3R5/G

IP1/FLT1/MVB12B/NEDD4L/AP2B1/ADRB1/ERBB3/RAB11FIP5/PARD3/STAM/SMAP2/CLTCL1/EP
CN1/ATP6V1D/CLCN3/ATP6V1E1/ATP10A/UNC79/ATP2A2/ATP6V0C/SGK3/WWP1/ANO2/ATP1
POLE3/USP1/UBA52/UBB/RPS27A
/UBA52/UBB/POLH/RPS27A
POLE3/TDG/NTHL1/MPG/HMGB1/SMUG1/APEX2/APEX1
/FANCF/UBA52/FANCE/FANCL/DCLRE1B/UBB/RPS27A/ERCC1/EME2
OLE3

PC6B/MAN1A2/TUBB2A/MGAT4A/NAPG/MAN2A2/ACTR1A/ANKRD28/TUBB8/ST6GAL1/SEC24
./PIK3R1/PIKFYVE/PLA2G6/PIK3CG/PISD/MTM1/PLD4/GPD1L/LPCAT2/PHOSPHO1/DDHD1/MTN

NCE
3/HIST2H2AC/MDK/HIST1H2AD/PTCRA/ELF3/E2F1/HIST1H4C/HIST1H4A/HIST1H2BJ/HIST1H2BI

9/CHRNA7/GRIA4/KCNJ15/PLCB2/KCNJ5/TUBB8/MAPK3/RPS6KA2/GABRR3/CHRNA3/AP2B1/GI
CACNA1E

MYBPC2/ANXA6/KCNIP3/GUCY1A2/SCN4B/DMD/STIM1/TPM1/KCNK16/SCN1A/KCNIP4/ACTC1

RKL/GNA13/PIK3CG/SSH1/FGF4/BRAF/SOS2/ITGB4/FGF20/ABI2/ITGB6/PIK3R5/FGF11/FGD3/VA

R/DGKQ/NMBR/FPR2/TAC3/LPAR4/PTAFR/EDNRA/FFAR4/PLCB2/MGLL/MAPK3/LPAR5/NMB/R

/PRKACG/PRKCB
AP5-1/KRTAP5-5/LOR/KRTAP11-1/LCE3D/SPRR2A/KRT1/KRTAP10-2/CELA2A/KRT84/PKP4/IVL/
8A2/SLC22A16/SLC9A7/SLC13A5/SLC7A11/SLC7A1/AVP/SLC1A7/SLC12A2/SLC4A9/SLC9A9/RH
3/MRPL23/MRPL47/MRPL22/GADD45GIP1/MRPS33/MRPL14/MRPL4/MRPS7/MRPL34/MRPS6/I
MA2/HIST1H2AG/ACTL6A/BARD1/MBD6/HIST1H2AC/PSME2/PSMA7/PSMD8/PSMD14/INO80C
B
/PRKCB/PRKCG
P35/NUP85/NUP54/THOC6/NUP205/NUP160/NUPL2/CPSF3/SRSF3/SLBP/U2AF1L4/RAE1/SRSF2
/EZH2/HIST2H2AC/E2F2/HIST1H2AD/CDKN2A/E2F1/HIST1H4C/HIST1H4A/HIST1H2BJ/HIST1H2
3C2G
2/POLD2/POLE/INO80C/POLE3/RUVBL1/COPS6/CETN2/COPS3/UBA52/CDK7/SUMO2/UBB/RPS
P3CA/PPP3CB/EGR2/PRKACG/EGR3/VIP
3/CHEK2/CCND2/GADD45A/TP73/CASP8/TP53/BAX/TP53AIP1/MDM2/TP53I3/DDB2/CCNE1/FA
PSMA7/PSMD8/CCNE1/PSMD14/PSMC2/PSMA1/PSMB4/PSMD13/PSMB1/UBA52/PSME1/PSMA
/KCNJ4/KCNJ3/KCNJ12
K/TLN1/THPO/GNAT3/ENDOD1/PIK3R5/GNAQ/VAV1/RHOB/PTK2/DGKH/IGF2/GNA15/PDPK1/
2/PSMA1/PSMB4/PSMD13/PSMB1/UBA52/PSME1/PSMA4/MDM4/UBB/PSMA5/RPS27A/PSMC4/
H4A/HIST1H2BJ/HIST1H2BL/H2AFX/BRCA1/HIST1H2BK/H2AFZ/HIST1H2AC/HIST1H2BD/HIST1H
D14/PSMC2/PSMA1/PSMB4/PSMD13/PSMB1/UBA52/PSME1/PSMA4/UBB/PSMA5/RPS27A/PSM
A2/BRCA1/HIST1H2BK/BARD1/TP53/HIST1H2BD/HIST1H4H/HIST2H2BE/UBA52/HIST1H2BN/UB
JSP3/PLA2G4E/TAOK1/MYC/MAPK8/MAP4K2/MAPK8IP1/CACNA1G/NLK/AKT3/MAPK3/RPS6KA
NA7/GRIA4/SLC18A2/KCNJ15/PLCB2/KCNJ5/TUBB8/MAPK3/RPS6KA2/GABRR3/CHRNA3/AP2B1
2/POLD2/POLE/POLE3/USP1/UBA52/UBB/POLH/RPS27A
M5/S1PR5/SSTR2/PLG/GABRB1/EDNRB/OPRL1/P2RX5/TRHR/GLP1R/HTR7/PTGER2/GABRB3/NT
PSMB4/PSMD13/PSMB1/PSME1/PSMA4/PSMA5/IFNG/PSMC4/PSMB7/PSMC3/PSMA3/PSMB6/P
NUP54/NUP205/NUP160/NUPL2/RAE1/SUMO2/AAAS/NUP43/NUP88

FB3

NCF/FANCE/FANCL
FGF4/APBB1IP/TGFA/GFRA1/BRAF/TLN1/JAK2/HCK/CNKSR1/FRS2/CD4/FGF20/GRIN2D/TEK/CSF
ISH6/RPA4

FRS3/YWHAB/NRTN/FGB/AGO4/FGF4/APBB1IP/TGFA/GFRA1/BRAF/TLN1/JAK2/AGO1/RAG2/C
UD3/AGO4/FGF4/SNAI1/TGFA/AGO1/FRS2/MTOR/IL33/FGF20/PHLPP1/PIK3AP1/VAV1/IRS1/AG

R1/BRAF/TLN1/ARHGEF11/PTK2/VCL/MAPK8/RAPGEF1/ELMO1/KLF8/MAPK1/MAP2K4/MAP2K1
0L/ARHGEF12/MCF2L/FGD4/NGF/ARHGEF33/FGD2/ARHGEF17/ARHGEF9/OBSCN/ARHGEF4/AR
P/RASAL2/MAPK3/NF1/SYNGAP1/PEBP1/MAPK1/MAP2K1/CALM1/CAMK2G/CNKSR2/CAMK2B/
3-30/IGLV1-47/IGLV3-1/IGLV1-40/IGHV1-2/IGHV3-23/IGHV2-5/IGKV2D-40/IGLV2-11/IGKV1-
32/MAPK1/PIK3CB/FGF22/FGF2/PTPN11/PDE3B/FGF17/KLB/FGFR2/FGF9/FGF8/FGF3/FLT3/FGF18
PIK3CB/FGF22/FGF2/PTPN11/PDE3B/FGF17/KLB/IGF1/FGFR2/FGF9/FGF8/FGF3/FLT3/FGF18/FGF
TAB2/CCL13/NAIP/CHUK/MAPK8/MAPK3/CARD18/RIPK2/MAPK1/CXCL2/MAPK9/MAPK10/MA
NEDD4L/GAB2/NGF/MAPK1/MAP2K1/PTPN11/DOCK1/TIAM1/NTRK2/PRKCZ/NTF3/DNM1/MA
R2/PRKG1/GNAO1/PPP3CA/PPP3CB/PRKG2/PLCB1/GNG3/ITPR1/GNG13/CAMK2A
T/TACR2/APLNR/TAC1/CX3CL1/GRP/INSL3/CHRM5/S1PR5/SSTR2/EDNRB/OPRL1/TRHR/CX3CR
F/COL4A5/CACNA1G/MAPK3/NCAM1/MAPK1/CACNA1H/CACNA1D/SPTAN1/CACNB3/COL6A
A/PRKCQ/MAPK9/IFNA21/PPP3CA/PPP3CB/PRKCE/EGR4/TNF/STAT4/PRKCB
TIAM1/PTK2B/GNAO1/ADCY5/ADCY8/ADRA1B/ADCY2/PRKCE/ADCY1/LPAR3
YP2J2/NCOA2/CYP4F12/ARNT2/CYP2E1/CYP21A2/CYP2F1/CYP26C1/CYP2C18/CYP1B1/CYP7B1
HSP90AA1/TLN1/PLXNA2/ARHGEF11/RHOB/ROCK1/PIP5K1C/DPYSL4/SEMA7A/PLXNB1/ARHG
CN6/PEBP1/KDM7A/MAPK1/TRAK1/MAP2K1/KIAA1549/MPRIP/CALM1/ESRP1/CAMK2G/CNKSR
IGHV3-30/SYK/IGHG1/IGLV1-47/IGLV3-1/IGLV1-40/IGHV1-2/IGHV3-23/IGHV2-5/IGKV2D-40/
2/CACNA2D1/CACNA1D/CACNB3/ITGA8/CACNB4/CACNA1S/CACNB2/CACNA1F/CACNA1C/CA
-17/IGLV3-25/IGHV3-30/MAPK3/SYK/IGLV1-47/IGLV3-1/IGLV1-40/IGHV1-2/MAPK1/PLCG2/M
KV1-17/IGLV3-25/IGHV3-30/SYK/GAB2/IGLV1-47/IGLV3-1/IGLV1-40/IGHV1-2/PIK3CB/IGHV3-
APK8/ARHGEF10L/ARHGEF12/MCF2L/FGD4/NGF/RIPK2/ARHGEF33/FGD2/OMG/ARHGEF17/ARH

A7/KALRN/PAK1/MYH11/DNM1/NGEF/EPHB6/EPHA8/GRIN1/EPHA6/GRIN2B

LE/POLE3/UBA52/UBB/RPS27A/ERCC1/RBX1
7/ALS2/TBC1D2/TRAPPC5/HPS1/RAB4A/MON1B/RGP1/ANKRD27/RAB6A/TRAPPC9/CHML/SYTL
MDM2
TRK6/SLITRK4/IL1RAPL2

FL6/IL20/CCL22/THPO/FGF20/RPTN/BMP8A/ANGPTL7/FGF11/BMP8B/CCL13/ISM1/MEGF6/MEG
GF3/FGF18/FGF5/FGF16

5C/MYRIP/KIF5A/STX1A
WHAH/KIT/ADCY1/PRKACG
GRIN2A/RELN
8/FGF3/FGF18/FGF5/FGF16
D/CTSH/ADRA2C/DMBT1/PGA3/SFTPA1/ADRA2A
CK1/DAPK1/UNC5C/UNC5A/PAK1/CAMK2A
2/GNG13/VIP
12D3/OR2A1/OR52A5/OR5B2/OR5K2/OR4K2/REEP3/OR2V1/OR6B2/OR6M1/OR1L3/OR1N2/RE
CA1C/CNGB1
PK10/MAPK13/MAPK8IP2/SYT1
E/DGKE/ITPR1
CA
/FGF8/FGF3/FGF5/FLRT2
HIST1H2AD/HIST1H4C/HIST1H4A/HIST1H2BJ/HIST1H2BL/HIST1H3D/H2AFX/HIST1H2BK/H3F3A/

GF9/FGF8/FGF3/FGF18/FGF5/FGF16
TN4/BAG4/TAB2/FGD3/VAV1/ARHGEF11/CHUK/MAPK8/SMPD3/ARHGEF10L/ARHGEF12/MCF2L
KCA/GNA14/GNAI1/GNAO1/PLCB1/ARRB1/DNM1/PRKCB/PRKCG
6T1/GUCA1B/OR51Q1/OR2AK2/OR6A2/OR2D2/OR2K2/PRKACB/OR2M4/CLCA2/PRKX/OR2C1/
RBB4/CBFA2T3/NRG3/GRIN2B
OS2/MKNK1/MTOR/PIK3R5/CBL/IRS1/TSC1/PTPRF/PRKAR2B/PDPK1/MAPK8/RAPGEF1/AKT3/PY
SMD13/PSMB1/UBA52/PSME1/PSMA4/UBB/PSMA5/RPS27A/PSMC4/PSMD5/PSMB7/RBX1/PSM
AM/PNLIP/DGKI/PNLIPRP2/ALDH2/DGKB/AWAT2/DGKE/GK2
PPP2R5B/MET/FGF7/SRC/PRR5/CD28/YWHAB/PIK3R1/MAML2/FGB/DKK4/TBL1X/HSP90AA1/FG
7/IGLV3-25/IGHV3-30/TEC/MAPK3/SYK/RASGRP4/GAB2/IGLV1-47/IGLV3-1/IGLV1-40/RASGRP

POB/RGS14/CXCL2/PRKX/RGS8/CNGA1/CALM1/PPP3R1/APOE/PRKCA/ITPR2/S1PR1/NPY/RCVR
C1A1/KCNK6/NRXN2/KCNJ11/GLRB/TSPAN7/ACTN2/ADCY9/LRRTM3/NLGN2/UNC13B/CHRNA
PIK3CD/PRKCD/HK1/MAPK9/MAPK10/ABCC8/CACNA1C/ADIPOQ/PKLR/CACNA1E/PRKCZ/PRK
POLR2G/GTF2H2/POLE4/DDB2/POLR2I/RPA2/POLD2/POLE/INO80C/POLE3/RUVBL1/COPS6/CE

0A2/AFP/AEN/TUBA1A/TP73/CASP2/BAX/TP53AIP1/TP63/MDM2/TP53I3

T1H4C/HIST1H4A/HIST1H2BJ/HIST1H2BL/MIR27A/HIST1H3D/H2AFX/HIST1H2BK/H3F3A/H2AF

/MUC6/GALNT16/ST3GAL1/B4GALT6/GALNT13/GALNT14/GALNT15/B3GNT4/GCNT4/GALNTL5/
PR5/WNT8B/SSTR2/WNT2B/UCN3/EDNRB/OPRL1/TRHR/CASR/CX3CR1/QRFPR/GLP1R/TAS2R4/
F7/YWHAB/PIK3R1/CRKL/AP2A1/KIDINS220/HSP90AA1/FGF4/TGFA/FER/BRAF/JAK2/ATP6V0A4

HGEF5/PPP1R12B/PIP5K1B

TPR1/NOS1

C6A15/SLC6A7
HAH/ADCY1/PRKACG

/FGF16
3-30/IGHG1/FCN1/IGLV1-47/IGLV3-1/IGLV1-40/IGHV1-2/IGHV3-23/IGHV2-5/IGKV2D-40/IGL
TH1R/ADORA2A/OR2M3/GCGR/OR2T6/VIPR2/ADCY9/SCTR/OR52B2/SCT/OR6T1/OR51Q1/AVP
3-30/IGHG1/FCN1/IGLV1-47/IGLV3-1/IGLV1-40/IGHV1-2/IGHV3-23/C4A/IGHV2-5/IGKV2D-4
BP8/CHEK2/BRCA1/BARD1/TP53/RAD9B/MDM2/RPA2/TAF7/DNA2/HUS1/RHNO1/RMI1/UBA5
A/GHR/PRKCB

NG3/GNG13

DH5A1/ALDH2/OXCT2/ACSM5/HMGCS2/GAD2
N11/FGF17/FGFR2/FGF9/FGF8/FGF3/FGF18/FGF5/FGF16
L/NR4A1/PPARA/RXRG/THRB/TNF

/IL5/CHP1/PDPK1/AKT3/TEC/MAPK3/NFAT5/RASGRP1/MAPK1/PIK3CB/CARD11/PIK3CD/MAP2
013/PSMB1/UBA52/PSME1/PSMA4/UBB/PSMA5/RPS27A/PSMC4/PSMD5/PSMB7/RBX1/PSMC3/
ALM1/CALM3/PPP3CA/PPP3CB/PRKCB
APK1/MAP2K1/PTPN11/DOCK1/PTK2B/PAK1
C6A18/SLC38A4/SLC6A20/SLC6A15

CSGALNACT1/CHST9/HS3ST2/CHST15/B3GNT4/HS3ST5/IDS/B3GAT2/CHST1/HAS1/OGN/OMD/
LP/NCAM2/L1CAM/SELPLG/NFASC/ITGA8/NRXN1/PTPRM/CLDN11/CNTNAP1/CD22/MAG/ITG
5/ZBTB8OS/PUS7/NUP85/NUP54/CTU1/NUP205/NUP160/POP5/TSEN34/TRMT112/POP4/NUP

LNK/IL5/RAPGEF1/TEC/JAK1/SYK/GAB2/CSF2RB/JAK3/PIK3CB/PIK3CD/PTPN11/CSF2
B/FGF22/FGF2/PTPN11/PDE3B/FGF17/KLB/FGFR2/FGF9/FGF8/FGF3/FLT3/FGF18/FGF5/FGF16

:13/PSMB1/UBA52/PSME1/PSMA4/UBB/PSMA5/RPS27A/PSMC4/PSMD5/PSMB7/PSMC3/PSMD9
SMD13/PSMB1/UBA52/PSME1/PSMA4/UBB/PSMA5/RPS27A/PSMC4/PSMD5/PSMB7/DERL2/PS
1/RUNX3/PSMB4/PSMD13/PSMB1/UBA52/PSME1/PSMA4/UBB/PSMA5/RPS27A/PSMC4/PSMD5
F16

2AD/HIST1H4C/HIST1H4A/HIST1H2BJ/GATA3/HIST1H2BL/MYB/HIST1H3D/TFF3/H2AFX/KPNA2/
RC6B/TNRC6C/AP2B1/WNT11/GNB5/AXIN2/WNT5B/CALM1/PPP3R1/PRKCA/ITPR2/WNT4/PRK(
2/POLE/POLE3/CETN2/CDK7/ERCC8/ERCC1/RPA4/RBX1
3/KNG1/PRKCB
./TRAPPC10/DCTN1/LMAN1L/ANK1/GBF1/COL7A1/SPTBN1/DYNC1H1/ANK2/NSF/GRIA1/SPTB
GNG3/GNG13
TPR1/SLC8A2/ATP2B3
GTF2H2/POLE4/POLR2I/RPA2/POLD2/POLE/POLE3/COPS6/COPS3/UBA52/CDK7/ERCC8/UBB/H
CA/SHANK3/DOK6/GFRA2/GFRA4/PRKACG/RET
1D13/TBC1D17/TBC1D2/RAB4A/RAB6A/SYTL1/TBC1D16/TBC1D24/TBC1D10A/ULK1/TSC1/RABI
0L/ARHGEF12/MCF2L/FGD4/NGF/ARHGEF33/FGD2/ARHGEF17/ARHGEF9/OBSCN/ARHGEF4/AR
K/IGLV2-23/FBXW11/IGLC3/IGKV1-17/IGLV3-25/IGHV3-30/STIM1/SYK/IGLV1-47/IGLV3-1/IGL
B/RPTOR/BRAF/MTOR/CLIP1/IRS1/ULK1/TSC1/PDPK1/FBXW11/RB1CC1/MAPK3/RRAGD/ULK2/

GAP2/NGF/PRKCD/YWHAG/YWHAH/EPB41L1/CAMK4/CAMK2A
ASP9/AKT3/MAPK3/PLA2G2D/NFAT5/MAPK1/PIK3CB/PLCG2/PIK3CD/MAP2K1/PPP3R1/PRKCA/
K3/PIK3CB/IL21R/PIK3CD/PTK2B/CSF2/IL9/STAT4
N1/ADAMTS8/THSD4/ADAMTS19/ADAMTSL5/ADAMTS16/ADAMTS20/ADAMTSL3
D1/HIST1H2BD/HIST1H4H/PAXIP1/HIST2H2BE/POLM/HIST1H2BN/UIMC1/BABAM1/TDP1/HIST1

/CHUK/PDPK1/KL/CASP9/AKT3/PTEN/STRN/RICTOR/ERBB3/PIK3CB/PIK3CD/FGF22/FGF2/PTPN
NKD1/APC/WNT1/DKK2/WIF1
MAP2K1/CALM1/CAMK2G/CNKSR2/CAMK2B/ARRB1/KSR2/CAMK2A
S/ELOVL3/RXRA/MID1IP1/ACSL1/CYP8B1/PTGR2/FASN/CYP4F2/CYP4F3/ACSBG1/FADS2/ACSL4
43/CYP24A1/EPHX1/AOC3/CYP2J2/ALDH1A1/NCOA2/CYP4F12/ARNT2/CYP2E1/AOC2/CYP21A
SF/DYNC1I1/PRKACG/CREB3L3

TL6A/TP53/ODC1/RCC1/BAX
C10/CDC16/CDC23/ANAPC15/ANAPC4/ANAPC5
013/PSMB1/UBA52/PSME1/PSMA4/UBB/PSMA5/RPS27A/NOTCH4/PSMC4/PSMD5/PSMB7/RBX1
S14/EIF3I/RPS15A/EIF3B/RPS27L/RPS28/RPS11/EIF3K/RPS16/RPS13/RPS27A/RPS3/RPS27/RPS9/I
NUP205/NUP160/POP5/TSEN34/POP4/NUPL2/CLP1/RAN/TSEN15/RAE1/RPP21/AAAS/NUP43/
GF5
TPN4/RIPK2/MAPK1/NKIRAS1/PLCG2/MEF2A/ITGAM/MAP2K4/MAP2K1/DUSP7/PTPN11/TRAF3/
C2/GRM4/PDE1A/GNG13/CACNA1B
CDH9/CDH12/CDH8

1/DAB2/TGFBR3/CAMK2A
LNT15/GCNT4/GALNTL5/GALNT9/GALNTL6
Y3/GLS2/GAD2

ADD3/DMTN
A1F/CACNA1C/CACNA2D2/CACNB1/CACNG8/CACNA2D3/CACNG3
KT3/TBC1D4/RICTOR/YWHAG/FOXO4/YWHAH/MAP3K5/SLC2A4
/PRKCB/RASGRF1
CAMK2B/CAMK2A
Y2/ADCY1/PRKACG/GNG3/GNG13
B1/UBA52/PSME1/PSMA4/UBB/PSMA5/RPS27A/PSMC4/PSMD5/PSMB7/PSMC3/PSMD9/PSMA
M1/ABR/MCF2/KALRN/CHN1/NGEF/RASGRF2/RASGRF1

P4A/INPP5F
UD2/GLUD1/USP46/MT-ATP6/PPARA/MT-ATP8/MEF2C/CAMK4
PDPK1/KL/MAPK3/ATP6V1H/GAB2/ATP6V1B2/MAPK1/PIK3CB/ATP6V1A/FGF22/FGF2/ATP6V0A
)/SNRNP40/MAGOHB/CPSF4/POLR2H/PTBP1/POLR2G/MAGOH/ALYREF/POLR2I/SNRPD1/SNRF

ERA/ST6GALNAC6/NDST1/PPP1R3C/ALDH1A1/VCAN/PRKACB/B4GALT1/G6PC/HK1/ALDOB/B3
LK/ITPR2/RPS6KA6/IGF1/BTRC/ADCY7/PPP2R5A/CPEB1/CALM3/YWHAH/PPP3R2/PPP3CA/PPP

5/FGF16

AI1/GNAO1/PLCB1
/GLUD1/CKMT1B/ALDH2/GOT1/CKMT1A/DAO/GLS2/NOS1

NRNP40/MAGOHB/MAGOH/ALYREF/SNRPD1/SNRPB2/PHF5A/SNRPF/SRSF3/PRPF3/PRPF4/PP
2/MAPK8/PTEN/MAPK3/SYK/MAPK1/CARD11/PLCG2/MAP2K1/PPP3CA/CD22/SH3BP5/PPP3CB
/TUBB6/TP53/PSME2/PSMA7/PSMD8/PSMD14/PSMC2/PSMA1/PSMB4/PSMD13/PSMB1/UBA52

6

A/RXRG/ADIPOQ/CYP4A22/AQP7/FABP6/SCD/HMGCS2/CYP4A11/SLC27A2/PLIN1/GK2
/MAPK3/RIN2/GAB2/MAPK1/MAP2K4/APC/MAP2K1/PTPN11/EPS15/ARHGEF4/DEPTOR/PAK1/

F9/FGF8/FGF18/FGF5/FGF16

1/FGF17/FGF9/FGF8/FGF3/FGF5
P85/NUP54/NUP205/NUP160/NUPL2/CETN2/TDG/RAE1/SUMO2/RNF2/SMC5/AAAS/PHC2/NU

A
COA1/FASN/SREBF1/NCOA2/GPAM/PPARA/SCD

5/IGHV3-30/CPN1/C9/CD55/C7/C5AR2/IGHG1/FCN1/IGLV1-47/IGLV3-1/IGLV1-40/IGHV1-2/I
ACB/IRF8/MAP2K1/PTPN11/CSF2/PRKACG
NCOA1/FASN/SEC24C/SREBF1/NCOA2/GPAM/PPARA/SCD

NUP160/NUPL2/RAE1/AAAS/NUP43/NUP88
K2/MYL2/PLD1/CIT/PIP5K1B/PRKCZ/SH3GL2
1A1/RAB30/NSF/NAPB

H2/POLE4/POLR2I/RPA2/POLD2/POLE/POLE3/UBA52/CDK7/ERCC8/UBB/HMGN1/POLR2C/RPS
DAMTS4/ADAMTS12/SPON1/ADAMTS8/THSD4/ADAMTS19/ADAMTSL5/ADAMTS16/ADAMTS2

NRPD1/YBX1/SNRPF/SF3B5/SRSF2/POLR2C/POLR2D/SNRNP25/SRSF7/SRSF6/DDX23

/HIST1H4C/HIST1H4A/HIST1H2BJ/H3F3C/HIST1H2BL/HIST1H3D/HIST2H2AB/ELANE/H2AFX/SN
/NCOA2/AKR1C2/AKR1C3/CYP7B1/AKR1C1/CYP46A1/CH25H/SLC27A2

DA2/PRKACB/POU1F1/CDK5R1/NR4A1/MAPK9/MAPK10/KRT14/YWHAH/CGA/CSF2/KRT17/SELE

FLRT1/FGF22/FGF2/PTPN11/FGF17/FGF9/FGF8/FGF3/FGF18/FGF5/FLRT2

16/CDC23/ANAPC15/ANAPC4/ANAPC5
N/PAK1/EPHB6/GRIN1/GRIN2B
KCB
2R5A/WNT3A/DKK2
OLR2J/POLD1/NT5E/POLR2H/NT5C3A/POLD3/POLR2G/ZNRD1/NME1-NME2/DCTD/POLE4/CA

LY6K/LY6D/LY6H/FOLR2/OPCML/CNTN5/NEGR1/CNTN4/CNTN3/RTN4RL1

RIP1/RMI2/STEAP3/CDK2/PCNA/CHEK1/BLM/CDKN2A/E2F1/FANCD2/RFC2/RPA3/CASP6/RFC4/

4/IGF2R/NAPG/VTI1A/VPS53/GCC2/NSF/RAB6B/NAPB

CA/SHANK3/DOK6/RET

/MAPK13/ETS2/MAP3K5/PRKCE/TNF/PRKCB/PRKCG

A5/ITSN1/SYNJ1/TF/GRIA1/KALRN/PAK1/DNM1

R6/PTPN4/RIPK2/MAPK1/NKIRAS1/PLCG2/MEF2A/ITGAM/MAP2K4/MAP2K1/DUSP7/PTPN11/A

BB3/PIK3CB/PIK3CD/FGF22/FGF2/PTPN11/FGF17/KLB/NRG4/FGFR2/FGF9/FGF8/FGF3/KIT/ERBB4
2/ULK2/RICTOR/MAPK1/PIK3CB/PRKAA2/PIK3CD/CAB39L/RPS6KA6/IGF1/EIF4E1B
N1/DYNC1H1/ANK2/NSF/SPTBN4/ANK3/SPTBN2/TUBA8/TUBB4A/SPTB/DYNC1I1/TUBA4A/CAP
1/BARD1/HIST1H2AC/PSMD14/UBA52/UBB/UIMC1/BABAM1/RPS27A

/PON1/CYP1B1/PTGS2/CYP4A22/ALOXE3/CYP1A1/PTGDS/CYP4A11

OC6/CPSF3/SNRPF/SRSF3/CLP1/SLBP/U2AF1L4/SRSF2/SRSF9/U2AF1/EIF4A3/SRSF7/SRSF6/THO

OL5A1/CACNB4/CACNA1S/CACNB2/CACNA1C/CACNB1/CNTN2/GFRA2/GFRA4/CACNA1
/UTP6/RRP36/UTP18/NHP2/DCAF13/EMG1/RPS9/WDR46/NOP2/DKC1/UTP20/NOC4L/FBL/NOP

/RASGRP1/RAPGEF3/ADRA2C/RAPGEF4/ADRA2A/GP9
7/CLDN16/CDH9/CDH12/CDH8

/TP53I3
K3CD/PRKCA/IGF1/ATP1A2/KCNJ1/NR3C2/FXYD4/ATP1B1/ATP1A3/PRKCB/IRS4/PRKCG

EC11/IGLV2-23/TRAV29DV5/IGLC3/IGKV1-17/IGLV3-25/KLRD1/CD200/LILRA1/SIGLEC1/IGHV3-

A1/CITED2/PSMB4/PSMD13/PSMB1/UBA52/PSME1/PSMA4/UBB/PSMA5/AJUBA/RPS27A/PSMC
RKCZ/CAMK2A

46/PPARA/MEF2C/CAMK4
PIK3CD/YWHAG/YWHAH/SORBS1/NPY4R/SLC2A4/IRS4
MAP2K1/PTPN11/DAPK1/IL1B/CAMK2G/CAMK2B/CAMK2A
MB4/PSMD13/PSMB1/PSME1/PSMA4/SRM/PSMA5/PSMC4/PSMD5/PSMB7/OAZ1/PSMC3/PSMI
SMD13/FZD2/PSMB1/UBA52/FZD1/PSME1/PSMA4/UBB/PSMA5/RPS27A/PSMC4/PSMD5/PSME
K2/TSC1/PDPK1/MYC/AKT3/PTEN/NOLC1/RPS6KA2/PARD3/PIK3CD/PPP1R13B/YWHAG/IGF1/Y
AAS/NUP43/NUP88/POM121/NUP155/NUP62/NUP188/SEC13
GAT3/CGA/ST8SIA3/MGAT4C
FGF9/FGF8/FGF5

SUMO2/RNF2/AAAS/PHC2/NUP43/NUP88/UBE2I/POM121/SCMH1/NOP58/NUP155/NUP62/NU
RAE1/AAAS/NUP43/NPM1/NUP88/POM121/NUP155/NUP62/NUP188/SEC13
B2/POLR2F/FGF22/FGF2/FGF17/MYO18A/FGFR2/FGF9/FGF8/FGF3/FGF18/FGF5/FGF16
35/BANF1/NUP85/NUP54/LMNA/VRK1/NUP205/NUP160/NUPL2

VNT5B/WNT8A/BTRC/WNT4/HHIP/WNT8B/WNT2B/WNT2/LRP2/WNT9B/WNT7B/WNT3A/WNT
LI2/DCTN1/DYNC1H1/HSPA2/NR3C2/DNAJA4/TUBA8/TUBB4A/PGR/DYNC1I1/TUBA4A/CAPZA

IELX/IGFALS/COL12A1/MFAP5/COL4A5/NYX/FGL1/COL13A1/SPARC/LGI1/TINAGL1/SSPO/LGI2/
F2C/NTF3

LR2I/NUP205/NUP160/NUPL2/RAE1/POLR2C/POLR2D/AAAS/NUP43/NUP88/POM121/NUP155

2/MCF2L/ARHGAP6/ARHGEF17/ARHGAP5/ARHGAP8/OBSCN/ARHGAP35/FARP1/OPHN1/ABR/N

2/POLR2I/MLH1/CDK7/POLR2C/NELFE/SUPT4H1/POLR2D/MSH2/NELFCD
NUP210/PSME2/B2M/PSMA7/PSMD8/NUP35/BANF1/NUP85/NUP54/AP2S1/NUP205/NUP160/P
/NCOA2/ZNF467/EBF1/KLF4/PPARA/ADIPOQ/EGR2/WNT1/SLC2A4/PLIN1/TNF/WNT10B
TED1/HIST1H2AD/HIST1H4C/HIST1H4A/HIST1H2BJ/GATA3/HIST1H2BL/MYB/HIST1H3D/GNG5/
7/MYO18A/FGF9/FGF8/FGF5

NUF2/CCNA2/AURKB/KIF18A/KIF20A/HIST2H3C/TOP2A/FOXM1/HIST1H3J/CDC45/KIF2C/CDCA
:/HIST1H3J/KIF2C/CDCA8/BUB1/CDK1/TUBA1C/SPC25/ERCC6L/CENPE/NCAPH/CENPF/BUB1B/(
CA2/FANCA/HIST1H2AD/EME1/BLM/HIST1H4C/HIST1H4A/FANCD2/RFC2/HIST1H2BJ/RPA3/HIST
/RPS10/MRPL36/SSR2/RPL36/MRPS24/DDOST/SRPRB/RPSA/MRPL53/MRPL13/RPS2/MRPL23/R
VEE1/ERCC6L/CENPE/GTSE1/CENPF/ORC1/BRIP1/RMI2/BUB1B/CENPM/CDC6/PLK1/MAD2L1/P
I/RPSA/RPS2/RPS19/PSMA7/PSMD8/RPL21/RPS21/RPS29/RPL27/RPS14/PSMD14/RPLP0/RPS15
PLK1/MAD2L1/CDCA5/CENPH/LMNB1/CENPI/FBXO5/CENPL/ZWINT/ITGB3BP/CENPN/DSN1/TU
/GINS4/MCM4/MCM6/PSMA2/MCM8/PRIM1/LIG1/PRIM2/POLA2/LIN9/MCM5/POLD1/MCM3/S
N2A/E2F1/HIST1H4C/HIST1H4A/HIST1H2BJ/HIST1H2BL/HIST1H1E/HIST1H3D/HIST1H1C/H2AFX
JB1B/CENPM/TUBA3C/PLK1/MAD2L1/CDCA5/SMC4/CENPH/PLK4/CENPI/HAUS1/CENPL/ZWIN
PS21/NIP7/RPS29/RPL27/RPS14/XRN2/DDX49/TRMT112/RPLP0/RPS15A/EXOSC9/RPL24/RPL17/
NUP210/RPL36/POLR2G/RPSA/KPNA7/RPS2/RPS19/NUP35/NUP85/RPL21/NUP54/RPS21/RPS2
CKS1B/FBXO5/CDC25A/PSMB8/PSMB9/MCM7/CCND2/MCM4/MCM6/PSMA2/MCM8/PRIM1/PF
T1H2BL/TIMELESS/RFC4/RFC3/RBBP8/H2AFX/KPNA2/CHEK2/EYA2/GEN1/BRCA1/PPP4C/HIST1H
/RFC2/HIST1H2BJ/RPA3/HIST1H2BL/CDC25A/RFC4/PSMB8/RFC3/RBBP8/PSMB9/H2AFX/MCM7/
I/CENPI/CENPL/ZWINT/ITGB3BP/CENPN/DSN1/TUBA3E/KNTC1/NUP37/ZWILCH/TUBA1A/TUBE
3L/HIST1H3D/CATSPER1/RBBP8/CATSPERB/H2AFX/ZP3/BRCA1/HIST1H2BK/H3F3A/H2AFZ/HIST
EIF3I/RPLP0/RPS15A/RPL24/EIF3B/RPL17/RPS27L/RPL23A/RPS28/UBA52/RPS11/RPL32/RPL29/R
/PLK1/MAD2L1/CDCA5/CENPH/CENPI/CENPL/ZWINT/ITGB3BP/CENPN/DSN1/TUBA3E/KNTC1/N
SMA2/MCM8/PRIM1/LIG1/PRIM2/POLA2/CDC7/MCM5/POLD1/MCM3/SKP2/POLD3/ORC5/PSM
3F4/CHEK1/CDKN2A/E2F1/CDC25A/MCM7/TGFB2/CHEK2/CCND2/MCM4/MCM6/GADD45A/MA
L/TIMELESS/RFC4/RFC3/RBBP8/H2AFX/GEN1/BRCA1/PPP4C/HIST1H2BK/BARD1/HIST1H2BD/PC
29/RPL27/RPS14/RPLP0/RPS15A/RPL24/RPL17/RPS27L/AIMP2/RPL23A/RPS28/UBA52/RPS11/RI
RPS21/RPS29/RPL27/RPS14/SSR4/RPLP0/RPS15A/RPN2/RPL24/RPL17/RPN1/RPS27L/SEC61A1/I
27/RPS14/RPLP0/RPS15A/RPL24/RPL17/RPS27L/RPL23A/RPS28/UBA52/RPS11/RPL32/RPL29/RP
T1H3D/H2AFX/NUP37/NCAPG2/TMPO/HIST1H2BK/H3F3A/NEK6/H2AFZ/HIST1H2AC/HIST1H2I
ENPL/ZWINT/ITGB3BP/CENPN/DSN1/KNTC1/NUP37
Z/CBX3/HIST1H2AC/HIST1H2BD/POLR2H/HIST1H4H/ZNRD1/HIST2H3A/H2AFJ/POLR1C/HIST2H
S15A/RPL24/RPL17/RPS27L/RPL23A/RPS28/UBA52/RPS11/RPL32/RPL29/RPL35A/RPL36A/RPL1(
A/HIST1H2BJ/HIST1H2BL/HOXD3/HIST1H3D/HOXD4/H2AFX/POLR2L/HIST1H2BK/H3F3A/H2AF
Z/HIST1H2AC/HIST1H2BD/POLR2H/HIST1H4H/ZNRD1/GTF2H2/HIST2H3A/H2AFJ/DNMT3B/POI
2BK/BARD1/HIST1H2BD/RAD9B/TIPIN/HIST1H4H/RPA2/DNA2/HUS1/RHNO1/HIST2H2BE/RMI1/
L/HIST1H3D/H2AFX/HIST1H2BK/H3F3A/H2AFZ/HIST1H2AC/HIST1H2BD/ANAPC11/HIST1H4H/I(
A/H2AFZ/HIST1H2AC/HIST1H2BD/POLR2J/POLR2H/NDC1/NUP107/NUP210/POLR2G/HIST1H4I
PINE1/HDAC1/PLAU/CASP7/TRIM28/HBP1/RBBP4/E2F5/CCNE2
FZ/HIST1H2AC/HIST1H2BD/POLD1/POLD3/HIST1H4H/POLE4/POT1/FEN1/RPA2/POLD2/H2AFJ
MD13/PSMB1/UBA52/PSME1/PSMA4/UBB/PSMA5/RPS27A/PSMC4/PSMD5/PSMB7/RBX1/PSM(
RPL24/RPL17/RPS27L/RPL23A/RPS28/UBA52/RPS11/RPL32/RPL29/RSL24D1P11/RPL35A/RPL36
Z/CBX3/HIST1H2AC/HIST1H2BD/POLR2H/HIST1H4H/ZNRD1/GTF2H2/HIST2H3A/H2AFJ/POLR1(
L/UBA52/PSME1/PSMA4/ANAPC7/UBB/PSMA5/RPS27A/PSMC4/PSMD5/PSMB7/ANAPC10/PSM
K/BARD1/TP53/HIST1H2BD/RAD9B/HIST1H4H/RPA2/DNA2/HUS1/RHNO1
3L/HIST1H3D/RBBP8/H2AFX/BRCA1/HIST1H2BK/H3F3A/H2AFZ/HIST1H2AC/HIST1H2BD/MSH5/
K/PSMA2/HIST1H2BK/H3F3A/LMO2/H2AFZ/TP73/HIST1H2AC/HIST1H2BD/PSME2/HIST1H4H/C
CDC26/PSMC2/PSMA1/PSMB4/PSMD13/PSMB1/UBA52/PSME1/PSMA4/ANAPC7/UBB/PSMA5/
/ANAPC7/UBB/PSMA5/RPS27A/PSMC4/PSMD5/PSMB7/ANAPC10/PSMC3/PSMD9/PSMA3/PSM
3/PSMA5/RPS27A/PSMC4/CCNE2/PSMD5/PSMB7/ANAPC10/PSMC3/PSMD9/PSMA3/PSMB6/CI
/RPA3/HIST1H2BL/CENPL/ITGB3BP/CENPN/RFC4/RFC3/CENPW/H2AFX/PRIM1/HIST1H2BK/LIG1
C5/PSME2/POLE4/PSMA7/PSMD8/RPA2/POLE/PSMD14/POLE3/PSMC2/PSMA1/PSMB4/PSMD1
IC2/PSMA1/PSMB4/PSMD13/PSMB1/UBA52/PSME1/PSMA4/ANAPC7/UBB/PSMA5/RPS27A/PSN
SMB1/UBA52/PSME1/PSMA4/UBB/PSMA5/RPS27A/PSMC4/PSMD5/PSMB7/PSMC3/PSMD9/PSM
C3/RPA2/POLD2/POLE/DNA2/HUS1/RHNO1/POLE3/RMI1/UBA52/UBB/RAD1/POLH/RPS27A
AFZ/HIST1H2AC/HIST1H2BD/HIST1H4H/HIST2H3A/LEF1/H2AFJ/RUVBL1/RUNX3/HIST2H2BE/HD
1/H2AFZ/PRIM2/POLA2/HIST1H2AC/HIST1H2BD/POLD1/POLD3/WRAP53/HIST1H4H/POLE4/P(
K/H3F3A/H2AFZ/HIST1H2AC/HIST1H2BD/HIST1H4H/CBFB/HIST2H3A/LEF1/H2AF
Z/CBX3/HIST1H2AC/HIST1H2BD/POLR2H/HIST1H4H/ZNRD1/GTF2H2/HIST2H3A
H2AC/PRMT1/HIST1H4H/RPS2/HIST2H3A
HMGA2/HIST1H1A/HIST1H2BK/H2AFZ/TP53/HIST1H2AC/HIST1H2BD/HIST1H4H/POT1/CCNE1/
H3D/RBBP8/H2AFX/BRCA1/HIST1H2BK/H3F3A/H2AFZ/HIST1H2AC/HIST1H2BD/MSH5/HIST1H4

3P/CENPN/CENPW/H2AFX/HIST1H2BK/H2AFZ/HIST1H2AC/HIST1H2BD/CENPO/HIST1H4H/CEN

IIST1H2AC/HIST1H2BD/HIST1H4H/HIST2H3A/H2AFJ/KLK3/HIST2H2BE

2/HIST1H2BK/H3F3A/H2AFZ/HIST1H2AC/HIST1H2BD/HIST1H4H/HIST2H3A/H2AFJ/SMC2
HIST1H2AC/HIST1H2BD/PHF19/HIST1H4H/HIST2H3A/H2AFJ/DNMT3B/HIST2H2BE/DNMT1/HIST
LE3

I2AC/HIST1H2BD/HIST1H4H/HIST2H3A
I2AC/HIST1H2BD/HIST1H4H/HIST2H3A/H2AFJ/DNMT3B/HIST2H2BE/DNMT1/HIST1H2BN

1/H2AFJ/MUTYH/UNG/HIST2H2BE/TDG/HIST1H2BN/NTHL1

1/H2AFJ/MUTYH

POLR2G/MAGOH/ALYREF/LUZP4/NUP35/NUP85/NUP54/THOC6/POLR2I/NUP205/NUP160/SNF

'PSMB8/PSMB9/PSMA2/BORA/TUBA1A/TUBB6/LIN9/TP53/DCTN2/CEP152/HAUS7/PSME2/CEP1

:8/RPL21/RPS21/NIP7/RPS29/RPL27/RPS14/XRN2/DDX49/TRMT112/RPLP0/RPS15A/EXOSC9/RF

T1H2BK/H3F3A/H2AFZ/HIST1H2AC/HIST1H2BD/CST3/B2M/APCS/HIST1H4H/HIST2H3A

RD5

HIST1H2AC/HIST1H2BD/HIST1H4H/HIST2H3A/HDAC3/GPS2/HMG20B/SAP30/HIST2H2BE/HDAC
CNG3/KCNB2/KCNS1/KCNH5/KCNS2/KCNV1

PRKCG/CAMK2A
A/SCN2A/SCN5A/FGF13/SCN3B/SCN7A/CACNA2D3/SCN2B/CACNG3/CAMK2A

HIST1H2AC/HIST1H2BD/HIST1H4H/HIST2H3A

R2/ITPR1

L/MTMR7/PLCH2

A3/SLC5A7/STXBP1/SYN1/RIMS1/GLS2/STX1A/SLC32A1/RAB3A/GAD2/SYN2/SNAP25/SLC17A7/
NJ1/PIP5K1B/PLEKHA5/INPP5J/PIK3C2G
G2/GRM5/STXBP1/DLGAP3/SYT7/STX1A/GRM1/SHANK1/SYT10/NRXN3/SYT2/GRIN1/DLGAP2/G

K1B/PI4KA/ITPKA/INPP5J/PLCB1/PIK3C2G
CZ/PRKCE/AKAP5/RCAN2/PRKCB/PRKCG
G

CY1/PRKACG/PDE1B/CAMKK1/PDE1A/ITPR1/PRKCG/CAMK2A
2/GNG3/GABRA4/KCNJ4/KCNJ3/KCNJ12/GABRG3/GABRA5/GABRA1/GABRA6/GABRB2
2A
/CAMK2G/CAMK2B/ATP1A3/ATP2B2/ATP8A2/ATP2B3/CAMK2A
B/GNAO1/GNAL/ADCY5/ADCY8/PLCB1/ADCY2/PRKCE/ADCY1/PRKCB/PRKCG
PB41L2/SHANK3/APBA1/DLG4/PTPRD/IL1RAPL1/GRIA1/PPFIA3/EPB41L1/PPFIA2/BEGAIN/PPFIA
NJ1/KCNAB1/ABCC8/KCNC4/KCNMA1/KCNK17/KCNA3/GABBR1/KCNJ6/KCNC1/KCNN3/KCNK4
P2R/GNG13/CRH/VIP/WNT10B
POQ/CAMKK2/SLC2A4/CAMKK1/TNF/IRS4
F/CACNA1C/CACNA2D2/ITGA9/CACNB1/CACNG8/SGCD/CACNA2D3/TNF/TNNT2/RYR2/CACN
MK2B/GRIN2C/CAMK4/PLCB1/CHP2/GRM5/ADCY1/PRKACG/GRM1/ITPR1/PRKCB/GRIN1/GRIN
PLCG2/PIK3CD/IGHV3-23/IGHV2-5/CALM1/ITPR2/IGKV2D-40/IGLV2-11/IGKV1-16/IGHV1-46/A
-5/CALM1/PPP3R1/ITPR2/IGKV2D-40/IGLV2-11/IGKV1-16/IGHV1-46/AHCYL1/IGKV3-15/IGHV
A/ADCY2/GRM5/ADCY1/PRKACG/TUBA4A/HTR2A/GRM1/DRD1/ITPR1/HTR2C/PRKCB/PRKCG

F2A/DUSP7/PTPN11/CDK5R1/MAPK13/RPS6KA5/TIAM1/DNM3/ADCYAP1R1/DOCK3/ADCYAP1
13A1/SLC30A8/SLC22A6/SLC6A13/SLC6A3/SLC13A2/SLC6A18/SLC5A7/SLC14A1/SLC6A20/SLC6
9BP/DHRS9/RGS9/LRP2/RHO/CLPS/AKR1C1/PPEF1/AWAT2/RBP4/GUCA1C/CNGB1
C25/ERCC6L/CENPE/CENPF/KLK2/BUB1B/CENPM/TUBA3C/KIF14/HIST2H2AC/PLK1/MAD2L1/PF
10/MAP3K5/PAK1/MAP3K9
MAPK9/MAPK10/PAK3/ERBB4/CAMK2G/CAMK2B/PAK1/NRG3/PAK6/PRKCB/PRKCG/CAMK2A
2/ADCY8/CAMK2G/CAMK2B/CAMK4/PLCB1/ADCY2/ADCY1/PRKACG/PDE1B/GNG3/CAMKK1/P
/MRVI1/PDE2A/ATP2B2/PDE1B/GNG3/PDE1A/ITPR1/GNG13/SLC8A2/NOS1/ATP2B3
F9/OBSCN/ARHGEF4/ARHGEF16/TIAM1/ITSN1/ARHGEF37/ARHGEF35/ABR/NET1/ARHGEF5/PL
LCG2/IGHV3-23/PRKCD/IGHV2-5/ITPR2/IGKV2D-40/IGLV2-11/IGKV1-16/IGHV1-46/PLD1/AHC
3/RPS6KA5/DNM3/ADCYAP1R1/ADCYAP1/MEF2C/NTRK2/SH3GL2/DNM1
/GRM5/GRM1/ITPR1/NOS1/PRKCB/CRH/PRKCG
G2/PIK3CD/MAP2K1/PPP3R1/PPP3R2/PPP3CA/CD22/PPP3CB/CR2/CHP2/PRKCE
A/SYNJ2/PIP4K2A/DGKI/CALM3/SYNJ1/PLCB4/PLCZ1/PIP5K1B/PI4KA/ITPKA/DGKB/CALML3/IN
IAA1549/MPRIP/CALM1/ESRP1/CAMK2G/CNKSR2/CAMK2B/ARRB1/KSR2/RASAL1/CAMK2A
/PI4K2A/INPP4A/ARF3/ENPP6/GDPD5/PIK3CB/MTMR9/PIK3CD/SBF1/MTMR8/BMX/PLEKHA6/T
CNB2/CACNA1C/CACNA2D2/ADCY5/ADRA2C/CACNA1E/ADCY8/FFAR1/PLCB1/CHRM3/RAPGE
2/CACNA1F/CACNA1C/CACNA2D2/ADCY5/ITGA9/CACNB1/ADCY8/CACNG8/ADCY2/SGCD/AD
1/ATP1B1/CACNG8/ATP1A3/CACNA2D3/TNNT2/RYR2/CACNG3
D1/PTK2B/CALM3/PLCB4/CACNA1S/CGA/PLA2G3/CACNA1F/CACNA1C/ADCY5/ADCY8/CAMK
NT2/PLCB4/KIT/WNT9B/WNT7B/ADCY5/WNT3A/ADCY8/CAMK2G/CALML3/CAMK2B/PLCB1/A
/LRP2/AAK1/ADRB2/SGIP1/SH3GL3/SLC18A3/REPS2/SNAP91/ARRB1/KIAA0319/SH3GL2/SYT2/
B/BAIAP2/PRKCD/ABI1/CALM1/PRKCA/ITPR2/DOCK1/MAPK13/AHCYL1/PTK2B/WASF1/PAK3/V
1/DOCK2/PRKCA/PLD1/WASF1/PLA2G4D/DNM3/WASF3/PIP5K1B/AMPH/PAK1/PRKCE/DNM1/
B/APBA1/DLG4/GRIA1/GRIN3A/ERBB4/CAMKK2/ADCY8/CAMK2G/TUBA8/CAMK2B/GRIN2C/CA
4/PRKAG2/VAMP2/ADCY7/GNAI1/AHCYL1/PRKAR1B/GLP1R/ABCC8/CACNB2/CACNA1C/CACN
GALT6/SPON1/GALNT13/ADAMTS8/GALNT14/GALNT15/THSD4/B3GNT4/ADAMTS19/GCNT4/A
TRPA1/RYR1/ASIC2/WNK2/TRPV6/TRPC5/ANO3/RYR2
/SLC25A18/SLC38A1/SLC9A5/SLC9A6/SLC7A8/SLC1A4/SLC26A9/SLC1A6/SLC1A2/SLC5A12/SLC
3CD/FGF22/FGF2/PTPN11/FGF17/KLB/NRG4/FGFR2/PIP4K2A/IER3/PPP2R5A/FGF9/FGF8/FGF3/
HB/PLA2G4C/GPAT2/AGPAT3/DDHD2/SLC44A1/PITPNM1/CDS2/GPAM/LPCAT4/PITPNM2/PLD
NTNAP1/DNM3/SPTBN4/ANK3/ITGA9/SPTBN2/CNTN2/TUBA8/CNTN1/SCN1B/PAK1/TUBB4A/S
18/SFTPA1/CDH7/CLDN16/CDH9/CDH12/CDH8
-30/NCKAP1L/MAPK3/SYK/NCKIPSD/IGHG1/IGLV1-47/IGLV3-1/IGLV1-40/IGHV1-2/MAPK1/IG
RIPK2/MAPK1/PIK3CB/PLCG2/PIK3CD/PRKCD/MAP2K1/PTPN11/YWHAG/CALM1/RPS6KA6/MA
H2AFZ/CBX3/HIST1H2AC/HIST1H2BD/PHF19/POLR2H/HIST1H4H/ZNRD1/GTF2H2/HIST2H3A/H
RKCA/ITPR2/PRKCQ/CALCRL/PRKG1/ADCY7/CALM3/PLCB4/KCNMA1/CACNA1S/PLA2G3/PPP1
YC/KSR1/MAPK8/RAPGEF1/PTEN/MAPK3/PPP2R2B/MAPK1/PIK3CB/MAP2K4/PIK3CD/BAIAP2/P
T1H2BK/H3F3A/H2AFZ/TARBP2/PIWIL4/HIST1H2AC/HIST1H2BD/POLR2J/POLR2H/NDC1/NUP
A/HIST1H2BK/KANSL2/ING3/HIST1H2AC/HIST1H2BD/YEATS4/MRGBP/HIST1H4H/HIST2H3A/EL
A5/RND1/NTN4/SEMA3C/GNAI1/SEMA6B/PAK3/SEMA3D/PPP3R2/CXCL12/PPP3CA/ABLIM1/U
1/CLDN11/EPB41L1/CTNNA3/EPB41L3/PRKCZ/MYH15/PRKCE/TJP2/MYH8/RAB3B/MYH11/MY
T78/DSP/KRT6C/SPRR2G/KRT17/PCSK6/KRT83/KRT6B/TCHH/KRT86/KRT81/KRT82/KRT33A/DSG
/PSME1/CDK7/PSMA4/UBB/PSMA5/RBBP4/RPS27A/E2F5/PSMC4/CCNE2/PSMD5/PSMB7/E2F4/
A/H2AFZ/TP53/HIST1H2AC/HIST1H2BD/HIST1H4H/HIST2H3A/H2AFJ/POGLUT1/POFUT1/MIR3
V1-17/IGLV3-25/ELMO1/WIPF2/IGHV3-30/NCKAP1L/MAPK3/SYK/NCKIPSD/IGHG1/IGLV1-47/I
R2/PLN/ATP2B1/KCND1/ATP1A2/SCN11A/ABCC9/SCN9A/AHCYL1/CACNB4/CASQ2/CACNA1S
33/CHN2/FGD2/RHOBTB2/ARHGAP6/ARHGAP24/TAGAP/ARHGEF17/ARHGAP5/ARHGEF9/ARH
UPN/AAAS/NUP43/DDX20/NUP88/POM121/GEMIN4/NUP155/NUP62/NUP188/SEC13/TGS1

2R5C/WNT11/TBL1Y/NFAT5/PRKACB/AXIN2/NKD1/APC/PRKX/WNT5B/PPP3R1/PRICKLE2/PRKC
2/DAB2/VAMP2/SYNJ2/STON1/SYT9/ITSN1/SYNJ1/TF/LRP2/AAK1/DNM3/ADRB2/AMPH/SGIP1

NA1C/MT-ATP8/MT-CYB/CALML3/GRIN2C/PLCB1/CHP2/SNCA/ITPR1/TNF/NOS1/GRIN1/GRIN
SSTR5/PENK/CCL21/AVPR1B/NPY5R/PDYN/MC3R/CCL19/XK/NPY1R/OPRD1/RXFP2/TACR3/CC
/PLCD3/CACNA1H/PLCG2/BDKRB1/PRKACB/CACNA1D/PRKX/CALM1/MYLK/PPP3R1/PRKCA/IT
RK5/XCR1/CCL13/VAV1/PTK2/ROCK1/CHUK/CXCR5/ADCY9/ELMO1/AKT3/CXCL6/PLCB2/MAP

S15/LDLRAP1/FGFR2/RAB11FIP2/DAB2/PLD1/HSPA2/CSF1R/IQSEC1/EHD3/KIT/DNM3/ADRB2/
L3A2/NEDD4L/ATP6V1H/TRPV4/TRPV3/CLCN6/ATP6V1B2/ATP1A4/WNK1/TRPM1/ATP10B/TRD

C/CD55/MGAT5/ARF3/GORASP1/DYNLL2/SEC16B/MAN1A1/DYNC1LI2/SPTAN1/TRAPPC10/B4
MR10/PIK3R5/MBOAT2/PLD3/FIG4/PCYT1B/SBF2/MTMR3/DGAT2/PIP5K1C/PLA2G4E/AGPAT4/I

L/JAG1/HIST1H3D/LFNG/PSMB8/PSMB9/H2AFX/TLE2/PSMA2/HIST1H2BK/H3F3A/H2AFZ/GZMB

NB5/GRIP2/KCNJ10/MAPK1/PRKACB/PRKAA2/GLRA4/NPTN/CHRNA4/GRIA2/PRKX/DLG3/CHRN

L/ATP1A4/KCND2/TRDN/CACNA2D1/CACNA1D/MYL2/FXYD7/CALM1/MYLK/CACNB3/ITPR2/PL

V1/PTK2/ROCK1/PFN2/GIT1/TMSB4Y/VCL/PIP5K1C/ACTN2/EZR/ENAH/SSH2/NCKAP1L/MAPK3

PS6KA2/AVP/HTR2B/GNB5/GNRHR/DAGLA/ABHD6/GPR132/RASGRP1/MAPK1/BDKRB1/DGKZ/

/PKP3/DSC3/PKP1/KRT14/KRT35/SPRR1A/KRTAP5-4/LCE1E/DSC1/KRT78/DSP/KRT6C/SPRR2G/K
HCG/SLC12A1/SLCO2B1/SLC14A2/EMB/SLC4A4/SLC6A12/SLC2A13/SLC44A1/SLC22A15/SLC6A1
MRPL12/MRPL15/MRPL21/MRPL49/MRPS26/MRPL33/MRPS18A/MRPL52/AURKAIP1/MRPL55/M
C/PSMC2/PSMA1/RUVBL1/PSMB4/PSMD13/PSMB1/UBA52/UCHL3/PSME1/PSMA4/UBB/PSMA5/

2/SRSF9/AAAS/NUP43/U2AF1/EIF4A3/SRSF7/SRSF6/NUP88/THOC1/THOC3/SARNP/U2AF2/RNF
BL/HIST1H3D/H2AFX/HIST1H2BK/H3F3A/H2AFZ/TP53/HIST1H2AC/HIST1H2BD/MIR24-2/HIST1

S27A/INO80B/ERCC1/ACTR5/RBX1/MCRS1/ACTB/UBE2N/RFC5/CCNH/UBE2I/INO80E/RPA1/CC

AS/CYCS
A4/MDM4/UBB/PSMA5/RPS27A/PSMC4/CCNE2/PSMD5/PSMB7/PSMC3/PSMD9/PSMA3/PSMB6

VTI1B/DGKQ/VCL/ACTN2/PSAP/GP6/SPARC/TIMP3/MGLL/MAPK3/SYK/GNB5/DAGLA/ABHD6/M
/PSMD5/PSMB7/PSMC3/PSMD9/PSMA3/PSMB6/PSMB2/PSMC6/PSMD3/PSMB10/PSMD12/PSM
H4H/SYCE3/POT1/LMNA/TEX12/H2AFJ/SUN1/HIST2H2BE/SYCP1/HIST1H2BN
IC4/CCNE2/PSMD5/PSMB7/PSMC3/PSMD9/PSMA3/PSMB6/PSMB2/PSMC6/PSMD3/CUL1/PSM
B/UIMC1/BABAM1/RPS27A/HIST1H2BC
A2/TGFBR2/NF1/RASGRP4/PLA2G2D/PPM1A/MAP4K4/CACNA1A/NGF/MRAS/RASGRP1/MAPK1
/GNB5/GRIP2/SLC1A7/CACNA1A/GLUL/DNAJC5/KCNJ10/MAPK1/PRKACB/PRKAA2/SLC6A12/G

TSR2/UTS2R/GRIA1/CGA/CALCR/GLRA3/P2RY13/SSTR1/PTGFR/ADRB2/SSTR5/PTGDR/GRIN3A/
POMP/PSMB2/PSMC6/PSMD3/PSMB10/PSMD12/PSMC1

-2RA/RASAL3/IRS1/PTK2/GDNF/IL5/PDPK1/KL/CSF1/VCL/ACTN2/KSR1/DAB2IP/RASAL2/MAPK3

NKSR1/FRS2/FGF20/GRIN2D/TEK/CSF2RA/RASAL3/IRS1/AGO3/PTK2/GDNF/IL5/KL/VCL/MYC/A
GO3/CHUK/HDAC5/PRKAR2B/PDPK1/KL/PIP5K1C/ADCY9/PREX2/CASP9/AKT3/PTEN/MTA3/MA

/BMX/MAPK8IP3/DOCK1/ARHGAP35/MAPK9/ARHGAP26/PAK1
RHGEF16/TIAM1/ITSN1/ARHGEF37/ARHGEF35/ABR/NET1/ARHGEF5/PLEKHG5/MCF2/KALRN/N
ARRB1/KSR2/RASAL1/CAMK2A
-16/IGHV1-46/IGKV3-15/IGHV4-59/IGKV1D-16/CD22/IGHV3-48/IGHV3-11/IGLV6-57
8/FGF5/FGF16
5/FGF16/IRS4
PK13/IL1B/CCL8/NLRP3/CCL11/PYDC1/TNF
TK/RASGRF1

1/QRFPR/HTR7/PTGER2/NTSR2/UTS2R/CXCL12/CGA/P2RY13/SSTR1/PTGFR/ADRB2/RHO/SSTR
6/PRNP/SPTBN1/COL5A1/RPS6KA5/CACNB4/CACNA1S/SPTBN4/CACNB2/CACNA1C/SPTBN2/

/CYP26A1/CYP4A22/CYP1A1/CYP2A7/CYP46A1/CYP4A11/CYP26B1
EF12/MYH10/LIMK2/MYH14/CDK5R1/RND1/ARHGAP35/PAK3/SEMA4D/SEMA4A/PAK1/MYH11
2/CAMK2B/ARRB1/KSR2/CAMK2A
IGLV2-11/IGKV1-16/IGHV1-46/IGKV3-15/IGHV4-59/IGKV1D-16/IGHV3-48/IGHV3-11/IGLV6-5
CNA2D2/ITGA9/CTNNA3/DSP/CACNB1/CACNG8/SGCD/PKP2/CACNA2D3/RYR2/CACNG3
AP2K4/IGHV3-23/IGHV2-5/IGKV2D-40/IGLV2-11/IGKV1-16/MAPK9/IGHV1-46/MAPK10/IGKV3
23/IGHV2-5/IGKV2D-40/IGLV2-11/IGKV1-16/IGHV1-46/IGKV3-15/IGHV4-59/IGKV1D-16/IGH
HGEF9/OBSCN/ARHGEF4/ARHGEF16/TIAM1/ITSN1/ARHGEF37/ARHGEF35/ABR/NET1/ARHGEF5

1/TBC1D16/DENND6B/DENND3/DENND4A/RAB9B/DENND4C/TRAPPC6B/DENND1C/DENND2

GF9/GDNF/IL5/BDNF/BMP4/CHRD/IGF2/CSF1/IL12B/EBI3/BMP6/IFNE/CXCL6/PIK3IP1/IFNK/CNT

EP6/OR2C3/OR4N2/OR2L3/REEP5/OR8D4/OR10Q1/OR4C3/OR1D2/OR2M3/OR2T6/OR52B2/C

/H2AFZ/PKN3/MYL6/RHOC/HIST1H2AC/HIST1H2BD/MYL12B/HIST1H4H/HIST2H3A/PPP1CB/H2

L/FGD4/NGF/RIPK2/CYLD/ARHGEF33/CLIP3/FGD2/OMG/MADD/ARHGEF17/ARHGEF9/OBSCN/A

OR2H2/CALM1/OR5AK2/OR3A3/OR10H2/CNGA4/OR13A1/OR13J1/OR4A47/PRKG1/OR2L2/OF

'GB/FASN/MAPK3/PCK1/SREBF1/PPP1R3C/MAPK1/PIK3CB/PRKACB/PRKAA2/PIK3CD/MAP2K1/L
IC3/PSMD9/PSMA3/PSMB6/PSMB2/PSMC6/PSMD3/PSMB10/PSMD12/PSMC1

GF4/APBB1IP/TGFA/BRAF/TLN1/JAK2/LRP6/AMER1/STRA6/CNKSR1/FRS2/POLR2A/MTOR/FGF2(
'1/IGHV1-2/MAPK1/PIK3CB/CARD11/PLCG2/MAP2K4/IGHV3-23/IGHV2-5/CALM1/PPP3R1/ITPF

RN/PRKCQ/PNLIP/CORT/AKR1C3/APLNR/CX3CL1/ADCY7/S1PR5/SSTR2/RGS9BP/OPRL1/GNAI1/
7/GRIA4/SLC18A2/KCNJ15/KCNQ2/PLCB2/KCNJ5/NLGN4Y/TUBB8/MAPK3/LRRTM1/RPS6KA2/G
CE/SLC2A4/TNF/IRS4/CACNA1E
TN2/COPS3/UBA52/CDK7/SUMO2/ERCC8/UBB/HMGN1/POLR2C/RPS27A/POLR2D/INO80B/ER

Z/HIST1H2AC/HIST1H2BD/PRMT1/HIST1H4H/CBFB/HIST2H3A/H2AFJ/DPY30/PRMT6/HIST2H2B

/GALNT9/GALNTL6/MUC7
/HTR7/WNT2/PTGER2/NTSR2/UTS2R/CXCL12/CGA/CALCR/WNT9B/P2RY13/SSTR1/PTGFR/ADR
-/AP2A2/JUP/MKNK1/FRS2/POLR2A/MTOR/FGF20/ATP6V1D/ABI2/ATP6V1E1/TAB2/NTRK1/CBL

V2-11/IGKV1-16/IGHV1-46/CRP/IGKV3-15/IGHV4-59/IGKV1D-16/IGHV3-48/IGHV3-11/IGLV6-
'/OR2AK2/OR6A2/OR2D2/GNB5/OR6J1/ADRB1/GHRHR/GNAZ/OR2K2/PDE10A/PDE4A/OR2M4
)/IGLV2-11/IGKV1-16/C4B/IGHV1-46/CRP/IGKV3-15/IGHV4-59/IGKV1D-16/IGHV3-48/IGHV3-
2/TAF12/MDM4/UBB/TAF7L/RAD1/RPS27A/TAF11/TAF13/PLK3/CDK5/CSNK2B/TAF10/PRKAB1/

K1/PPP3R1/PRKCQ/MAPK9/MAPK13/PAK3/PPP3R2/PPP3CA/PPP3CB/CSF2/PAK1/CHP2/PAK6/T
'PSMD9/PSMA3/PSMB6/PSMB2/PSMC6/PSMD3/CUL1/PSMB10/PSMD12/PSMC1/FBXL7/PSMA6

'LYVE1/NDST3/HS3ST4/HS6ST3/HPSE2
A9/CADM3/CNTN2/CNTN1/SELE/CNTNAP2/NEGR1/CLDN16/NRXN3/SELL
L2/TRMT10C/CLP1/RAN/THG1L/TSEN15/TYW1/RAE1/RPP21/CTU2/URM1/TRMU/LAGE3/TRMT1

)/PSMA3/PSMB6/PSMB2/PSMC6/PSMD3/HNRNPD/PSMB10/PSMD12/PSMC1
MC3/PSMD9/PSMA3/PSMB6/PSMB2/PSMC6/PSMD3/PSMB10/PSMD12/PSMC1/PSMA6/RNF5/I
5/PSMB7/PSMC3/PSMD9/PSMA3/PSMB6/PSMB2/PSMC6/PSMD3/PSMB10/PSMD12/PSMC1

/POLR2L/HIST1H2BK/H3F3A/H2AFZ/HIST1H2AC/HIST1H2BD/ZNF217/POLR2J/POLR2H/POLR2G
G1/GNAO1/ROR2/PRICKLE1/PPP3CA/PPP3CB/PRKG2/PLCB1/WNT1/GNG3/ITPR1/GNG13/PRKC

N4/ANK3/SPTBN2/TUBA8/TUBB4A/SPTB/DYNC1I1/TUBA4A/CAPZA3/NAPB/F5

HMGN1/POLR2C/RPS27A/POLR2D/ERCC1/RBX1/RFC5/CCNH/RPA1/TCEA1/COPS8/POLR2K/PPIE

EP1/RABGAP1/TBC1D14/OPTN/RAB6B
RHGEF16/TIAM1/ITSN1/ARHGEF37/ARHGEF35/ABR/NET1/ARHGEF5/PLEKHG5/MCF2/KALRN/NG
V1-40/RASGRP1/IGHV1-2/REL/CARD11/PLCG2/PIK3CD/IGHV3-23/IGHV2-5/CALM1/PPP3R1/IT
SSPO/RICTOR/PDCD4/SREBF1/MAPK1/MAP2K1/YWHAG/PRKCA/PLD1/YWHAH/DEPTOR

/MAPK13/PPP3R2/PPP3CA/PLA2G3/PPP3CB/PTGS2/CHP2/PRKCB/PRKCG

1H2BC

11/FGF17/KLB/NRG4/FGFR2/FOXO4/NR4A1/FGF9/FGF8/FGF3/KIT/ERBB4/FGF18/FGF5/NRG3/FG

4/PTGIS/FADS1/HADHB/OLAH/CYP2J2/ACOT7/ACOT4/ELOVL4/PRKAA2/DPEP3/TECRL/ACBD5/
2/MAOA/CYP2F1/CYP26C1/ADH1B/FMO2/ALDH2/CYP2C18/CYP1B1/CYP7B1/CYP26A1/CYP4A

1/PSMC3/PSMD9/PSMA3/PSMB6/PSMB2/PSMC6/PSMD3/CUL1/PSMB10/PSMD12/PSMC1
EIF4A1/RPS4X/RPS8/EIF3G/FAU
NUP88/TSEN54/POM121/NUP155/NUP62/NUP188/SEC13

/SFTPA2/BTRC/MAPK9/MAPK10/LBP/RPS6KA5/PELI3/DNM3/TLR4/SFTPD/MEF2C/SFTPA1/DNM

3/PSMB6/PSMB2/PSMC6/PSMD3/PSMB10/PSMD12/PSMC1

\1/PTPN11/PDE3B/ATP6V1C1/FGF17/KLB/FGFR2/FGF9/FGF8/FGF3/FLT3/ATP6V1G2/FGF18/FGF5
PB2/PHF5A/YBX1/PPIL3/CPSF3/SNRPF/HNRNPH1/SRSF3/CLP1/PRPF3/PRPF4/U2AF1L4/PPIL1/RE

GALT2/CALM1/UST/ST3GAL1/PFKFB3/ST3GAL6/ALDOC/ACAN/PC/GYS2/B4GALT6/NDST4/NHL
3CB/PLCZ1/ADCY5/ADCY8/CAMK2G/CALML3/CAMK2B/MOS/ADCY2/CHP2/PGR/ADCY1/PRKA

IL1/RBMX/SNRPA1/PRPF31/USP39/SF3B5/LSM6/SRSF2/SF3A2/SF3A3/TRA2B/SRSF9/LSM3/U2AF
/CAMK2G
/PSME1/PSMA4/UBB/PSMA5/FKBPL/TUBA1B/RPS27A/PSMC4/PSMD5/PSMB7/PSMC3/PSMD9/I

PRKCZ/SH3GL2

P43/NUP88/NSMCE1/UBE2I/POM121/SCMH1/RPA1/NUP155/NUP62/NUP188/SUMO1/SMC6/S

GHV3-23/C4A/IGHV2-5/IGKV2D-40/IGLV2-11/IGKV1-16/C4B/IGHV1-46/CRP/IGKV3-15/IGHV4

27A/POLR2D
0/ADAMTSL3/MUC7

RPB/HIST1H2AG/HIST1H2BK/H3F3A/H2AFZ/C8B/HIST1H2AC/HIST1H2BD/TRIM21/FCGR2B/HIS

E/VIPR1/PRKACG

ANT1/POLR3G/ITPA/RRM1/POLR2I/POLR2J3/POLD2/POLE/POLR2J2/CMPK2/POLE3/POLR1C/UC

/FANCC/RFC3/RBBP8/CHEK2/POLR2L/SMYD2/SETD9/CASP1/GADD45A/BRCA1/TRIAP1/BARD1/

POB/TLR5/TRAF3/SFTPA2/BTRC/MAPK9/MAPK10/LBP/RPS6KA5/PELI3/DNM3/TLR4/SFTPD/S10

4/FGF18/FGF5/NRG3/FGF16

ZA3/NAPE

C1/THOC3/SARNP/U2AF2/CSTF1/RNPS1

P14/NOP58/GAR1

-30/SIGLEC12/IGLV1-47/IGLV3-1/PIANP/IGLV1-40/SIGLEC8/IGHV1-2/COLEC12/IGHV3-23/IGH

C4/PSMD5/PSMB7/RBX1/PSMC3/PSMD9/PSMA3/PSMB6/PSMB2/PSMC6/PSMD3/PSMB10/PSMI

D9/PSMA3/PSMB6/PSMB2/PSMC6/PSMD3/PSMB10/PSMD12/PSMC1
37/PSMC3/PSMD9/PSMA3/PSMB6/PSMB2/PSMC6/PSMD3/PSMB10/DVL2/PSMD12/PSMC1/FZD
WHAH/PAK3/ERBB4/PAK1/SLC2A4/PAK6/IRS4

JP188/SUMO1/SEC13/HNRNPC/NUP93

1/PRKACG/WNT10B
3

/NTN5/SBSPON/KERA/COL24A1/IMPG1/FBN1/VCAN/TNR/CILP2/RSPO4/LAMA3/FRAS1/CTHRC

/NUP62/POLR2K/NUP188/SEC13/POLR2E

NET1/ARHGEF5/MCF2/ARHGDIG/NGEF

PSMD14/PSMC2/PSMA1/NUPL2/RANBP1/PSMB4/PSMD13/PSMB1/RAN/UBA52/RAE1/PSME1/PS

TFF3/ESR2/GNG12/H2AFX/KPNA2/POLR2L/MMP7/HSPB1/GNG11/HIST1H2BK/H3F3A/H2AFZ/H

A8/BUB1/CDK1/TUBA1C/ESCO2/HMMR/SPC25/GINS2/TYMS/TPX2/WEE1/ERCC6L/CENPE/GTSE1
CENPM/ESPL1/TUBA3C/HIST2H2AC/PLK1/MAD2L1/CDCA5/SMC4/CENPH/PLK4/LMNB1/LPIN3/
T1H2BL/TIMELESS/FANCB/RFC4/FANCC/RFC3/RBBP8/H2AFX/KPNA2/CHEK2/POLR2L/EYA2/GEI
PS19/MRPL47/MARS/TRAM1/RPL21/RPS21/MRPL22/RPS29/RPL27/RPS14/GADD45GIP1/SSR4/E
KMYT1/ORC6/CLSPN/MCM2/CENPH/CDK2/DBF4/CHEK1/CENPI/MCM10/BLM/CDKN2A/HIST1H
A/RPL24/RPL17/PSMC2/PSMA1/MSI1/PSMB4/PSMD13/RPS27L/PSMB1/ROBO2/RPL23A/RPS28/
UBA3E/PSMB8/KNTC1/PSMB9/NUP37/PSMA2/ZWILCH/TMPO/TUBA1A/TUBB6/B9D2/NUP107/(
SKP2/POLD3/ORC5/PSME2/ANAPC11/CDC25B/POLE4/PSMA7/PSMD8/FEN1/RPA2/CCNE1/POL
/HMGA2/HIST1H1A/HIST1H2BK/H3F3A/H2AFZ/TP53/HIST1H2AC/HIST1H2BD/MIR24-2/ANAPC
IT/ITGB3BP/CENPN/HAUS8/DSN1/TUBA3E/KNTC1/NUP37/ZWILCH/TUBA1A/TUBB6/B9D2/DCTI
/NOL11/DDX47/WDR18/RPS27L/UTP15/RPL23A/RPS28/UBA52/RPS11/RPL32/RPL29/RPL35A/RF
9/RPL27/CALR/RPS14/POLR2I/NUP205/NUP160/RPLP0/RPS15A/RPL24/RPL17/NUPL2/RPS27L/F
RIM2/RBL1/POLA2/LIN9/CDC7/DHFR/MCM5/MCM3/SKP2/ORC5/PSME2/POLE4/PSMA7/PSMD8
I2BK/BARD1/TP53/HIST1H2BD/POLD1/SLX1A/RAD9B/POLD3/TIPIN/SLX1B/HIST1H4H/POLE4/FI
/CHEK2/MCM4/MCM6/PSMA2/MCM8/BRCA1/HIST1H2BK/BARD1/TP53/CDC7/HIST1H2BD/MCI
36/RHOC/B9D2/DIAPH3
1H2AC/HIST1H2BD/ZP1/MSH5/HIST1H4H/PSMC3IP/SYCE3/CD9/HIST2H3A/POT1/LMNA/TEX12
RPL35A/RPL36A/EIF2B3/RPL10A/RPL27A/RPLP2/RPL12/RPL13/EIF3K/RPS16/RPS13/RPS27A/RPS3
NUP37/ZWILCH/TUBA1A/TUBB6/B9D2
ME2/ANAPC11/POLE4/PSMA7/PSMD8/FEN1/RPA2/CCNE1/POLD2/POLE/PSMD14/DNA2/CDC26
AD2L2/RBL1/TP53/CDC7/MCM5/MCM3/SKP2/ORC5/ANAPC11/CDC25B/MDM2
OLD1/SLX1A/RAD9B/POLD3/TIPIN/SLX1B/HIST1H4H/POLE4/FEN1/XRCC3/RPA2/POLD2/POLE/D
PL32/RPL29/RPL35A/RPL36A/RPL10A/RPL27A/RARS/RPLP2/RPL12/RPL13/RPS16/RPS13/RPS27A
RPL23A/RPS28/UBA52/RPS11/SEC61B/RPL32/RPL29/RPL35A/RPL36A/RPL10A/RPL27A/RPLP2/R
L35A/RPL36A/RPL10A/RPL27A/RPLP2/RPL12/RPL13/RPS16/RPS13/RPS27A/RPS3/RPS27/RPLP1/
3D/NDC1/NUP107/NUP210/HIST1H4H/HIST2H3A/NUP35/BANF1/NUP85/NUP54/LMNA/VRK1/

I2BE/HDAC1/HIST1H2BN/DEK/RBBP4/HIST1H2BC/TWISTNB/MBD3/H2AFV/POLR1D/HIST2H3D
0A/RPL27A/RPLP2/RPL12/RPL13/RPS16/RPS13/RPS27A/RPS3/RPS27/RPLP1/RPS9/RPL28/RPL38/
Z/HIST1H2AC/HIST1H2BD/POLR2J/POLR2H/POLR2G/HIST1H4H
LR1C/SAP30/HIST2H2BE/HDAC1/DNMT1/HIST1H2BN/SUV39H1/CDK7/HIST1H2BC/TWISTNB/H
/UBA52/HIST1H2BN/SUMO2/UBB/UIMC1/RAD1/BABAM1/RPS27A
GFBP7/HIST2H3A
H/HIST2H3A/NUP35/NUP85/NUP54/POLR2I/NUP205/NUP160/H2AFJ/NUPL2/HIST2H2BE/RAN/

/POLE/MUTYH/UNG/POLE3/HIST2H2BE/PNKP/TDG/ADPRHL2/HIST1H2BN/NTHL1
C3/PSMD9/PSMA3/ORC2/PSMB6
A/RSL24D1/RPL10A/RPL27A/RPLP2/RPL12/RPL13/RPS16/RPS13/RPS27A/RPS3/RPS27/RPLP1/RP
C/HIST2H2BE/HDAC1/HIST1H2BN/CDK7/RBBP4/HIST1H2BC/TWISTNB/MBD3/H2AFV/POLR1D/H
IC3/PSMD9/PSMA3/PSMB6/CDC16/CDC23/ANAPC15/PSMB2/ANAPC4/PSMC6/PSMD3/ANAPC

HIST1H4H/PSMC3IP/SYCE3/HIST2H3A/POT1/LMNA/TEX12/RPA2/H2AFJ
BFB/HIST2H3A/PSMA7/PSMD8/H2AFJ/PSMD14/PSMC2/PSMA1/PSMB4/PSMD13/HIST2H2BE/P
RPS27A/PSMC4/CCNE2/PSMD5/PSMB7/RBX1/ANAPC10/PSMC3/PSMD9/PSMA3/ORC2/PSMB6
MB6/CDC16/CDC23/ANAPC15/PSMB2/ANAPC4/PSMC6/PSMD3/ANAPC5/PSMB10/PSMD12/PSM
DC16/CDC23/ANAPC15/PSMB2/ANAPC4/PSMC6/PSMD3/ANAPC5/PSMB10/PSMD12/PSMC1
/H2AFZ/PRIM2/POLA2/HIST1H2AC/HIST1H2BD/POLD1/POLD3/WRAP53/CENPO/HIST1H4H/PO
3/PSMB1/UBA52/PSME1/PSMA4/UBB/PSMA5/RPS27A/PSMC4/PSMD5/RPA4/PSMB7/PSMC3/PS
MC4/PSMD5/PSMB7/ANAPC10/PSMC3/PSMD9/PSMA3/PSMB6/CDC16/CDC23/ANAPC15/PSMI
MA3/ORC2/PSMB6

DAC1/TCF7/HIST1H2BN
OT1/FEN1/RPA2/POLD2/H2AFJ/POLE/DNA2/POLE3/RUVBL1

H2AFJ
H/PSMC3IP/HIST2H3A/RPA2/H2AFJ

NPP/MIS18A/H2AFJ/CENPQ/RUVBL1

T1H2BN/EED/RBBP4

RPD1/SNRPB2/PHF5A/YBX1/PPIL3/NUPL2/WTAP/CPSF3/SNRPF/HNRNPH1/SRSF3/CLP1/PRPF3/

135/CDC25B/PSMA7/PSMD8/NDE1/TUBB/SSNA1/PPP1CB/HAUS5/ODF2/PSMD14/TUBG1/PSM

PL24/RPL17/NOL11/DDX47/WDR18/TRMT10C/RPS27L/UTP15/RPL23A/RPS28/UBA52/TFB1M/RP

C1/HIST1H2BN/HIST2H2BF/BRMS1/RBBP4

/SYT1

GRIN2B/SYT1/GRIN2A

A4/DLGAP1/EPB41L3/GRIN2C/SHANK2/DLG2/GRM5/STXBP1/SLITRK6/DLGAP3/LRFN2/SYT7/SLIT
/KCNC3/KCNA2/KCNJ9/KCNK1/KCNN1/GABBR2/KCNH1/KCNK18/KCNB1/KCNQ3/KCNK9/GNG

NG3
2B/GRIN2A/PRKCG/CAMK2A
AHCYL1/IGKV3-15/IGHV4-59/IGKV1D-16/CD22/IGHV3-48/IGHV3-11/IGLV6-57/ITPR1
4-59/IGKV1D-16/PPP3CA/PPP3CB/IGHV3-48/IGHV3-11/IGLV6-57/ITPR1

/MEF2C/PCSK6/NTRK2/NTF3/SH3GL2/DNM1/GRIN2
A15/SLC30A3/SLC22A8/SLC6A

C1/IQGAP3/CENPH/IQGAP2/CENPI/HIST1H2AD/HIST1H4C/HIST1H4A/HIST1H2BJ/HIST1H2BL/

DE1A/ITPR1/GNG13/OPRM1/PRKCG/CAMK2A

EKHG5/MCF2/KALRN/ADRA1B/ADRA1A/GNG3/NGEF/GNG13/RASGRF2
YL1/IGKV3-15/IGHV4-59/IGKV1D-16/IGHV3-48/IGHV3-11/PRKCE/IGLV6-57/ITPR1

PP5J/PLCB1/DGKE/ITPR1/PRKCB/PIK3C2G/PRKCG

NFAIP8L3/SYNJ2/PIP4K2A/PLEKHA1/SYNJ1/PIP5K1B/PLEKHA5/PI4KA/INPP5J/INPP5F/MTMR7/P
F4/STXBP1/KCNB1/AKAP5/ADRA2A/SYT5/PRKACG/GNG3/STX1A/ITPR1/KCNC2/SNAP25/GNG
CY1/PRKACG/CACNA2D3/TNF/TNNT2/RYR2/CACNG

2G/CALML3/CAMK2B/PLCB1/ADCY2/ADCY1/PRKACG/FSHB/ITPR1/PRKCB/CAMK2A
DCY2/WNT1/ADCY1/PRKACG/TYRP1/PRKCB/WNT10B/CREB3L3/PRKCG/CAMK2A
CHRM2/SYT1
WASF3/CYFIP2/PAK1/PRKCZ/PRKACG/ITPR1/PRKCB
/PRKCB/PRKCG
MK4/TUBB4A/DLG2/LRRC7/ADCY1/PRKACG/TUBA4A/CAMKK1/RASGRF2/GRIN1/NRGN/GRIN2
A2D2/ADCY5/ADIPOQ/ADRA2C/PKLR/CACNA1E/ADCY8/FFAR1/PLCB1/CHRM3/ADCY2/RAPG
DAMTSL5/ADAMTS16/GALNTL5/ADAMTS20/GALNT9/GALNTL6/ADAMTSL3/MUC7

15A2/SLC36A2/SLC7A2/SLC9A2/SLC6A18/SLC24A4/SLC38A4/SLC5A5/SLC24A2/SLC4A10/SLC
KIT/IL1RL1/PIP5K1B/ERBB4/FGF18/FGF5/NRG3/FGF16
1/LPIN1/PLA1A/PLA2G4D/PLA2G3/AWAT2/ETNPPL/CPNE7/PITPNM3/CPNE6
CN8A/SCN2A/SCN5A/KCNQ3/SPTB/SCN3B/SCN7A/TUBA4A/SH3GL2/DNM1/SCN2

HV3-23/BAIAP2/IGHV2-5/ABI1/DOCK1/IGKV2D-40/IGLV2-11/IGKV1-16/IGHV1-46/IGKV3-15/
PK9/MAPK10/MAPK13/RPS6KA5/CALM3/YWHAH/MAP3K5/CAMK2G/CALML3/CAMK2B/NTRK2
2AFJ/DNMT3B/POLR1C/SAP30/HIST2H2BE/TDG/HDAC1/DNMT1/HIST1H2BN/SUV39H1/CDK7
R12B/CACNA1F/CACNA1C/MYLK3/ADCY5/AVPR1B/ADCY8/MRVI1/CALML3/CYP4A22/ADRA1B
RKCD/SLC9A3R1/MAP2K1/PTPN11/YWHAG/ABI1/PRKCA/S1PR1/ARHGAP35/MAPK9/MAPK10/
107/NUP210/POLR2G/HIST1H4H/MYBL1/HIST2H3A/NUP35/NUP85/NUP54/POLR2I/NUP205/N
P5/RUVBL1/ING4/HAT1/ELP6/HIST2H2BE/RUVBL2/TAF12/HIST1H2BN/HIST2H2BF
UNC5C/PPP3CB/SEMA4D/EPHA7/ABLIM2/UNC5A/SEMA3G/UNC5D/SEMA4A/PAK1/CHP2/ABLI
H13/CLDN16/MYH1/PPP2R2C/PRKCB/MYH4/PRKCG
3/PKP2/FLG/EVPL/KRT6A/KRT79/SPRR2E/KLK8/DSG1/KRT3/KRT33B/KLK5/KRT31
PSMC3/PSMD9/PSMA3/PSMB6/LIN37/CCNH/PSMB2/PSMC6/PSMD3/CUL1/PSMB10/PSMD12/
4C/HIST2H2BE/TMED2
GLV3-1/IGLV1-40/IGHV1-2/MAPK1/PIK3CB/PLCG2/IGHV3-23/BAIAP2/PRKCD/IGHV2-5/ABI1/
/FGF12/HIPK2/KCNK17/CACNB2/CACNA1F/CACNA1C/CACNA2D2/FXYD4/CACNB1/ATP1B1/K
GAP8/OBSCN/ARHGAP35/ARHGEF4/ARHGEF16/ARHGAP26/TIAM1/ITSN1/ARHGAP32/ARHGE

A/WNT8A/BTRC/MAPK9/FZD9/MAPK10/WNT4/WNT8B/PPP2R5A/WNT2B/WNT2/PLCB4/PPP3
/SH3GL3/SLC18A3/REPS2/SNAP91/DNAJC6/ARRB1/KIAA0319/SH3GL2/DNM1/SYT2/CHRM2/S

2B/GRIN2A
9/KNG1/NPY4R/MC4R/PRLHR/RXFP3/SST/NPFFR1/CCK/OPRK1/RXFP1/NPFFR2/HCRTR2/CCKB
TPR2/PLN/GNA14/ATP2B1/TACR2/ADCY7/CHRM5/EDNRB/P2RX5/TRHR/PTK2B/CALM3/HTR7/P
3/GNB5/JAK3/PARD3/MAPK1/PIK3CB/CXCL5/PRKACB/CCR1/PIK3CD/PRKCD/MAP2K1/DOCK2

PIP5K1B/IQSEC2/ERBB4/PSD3/PSD/ADRB3/SH3GL3/RAB11FIP1/PRKCZ/RAB11FIP4/DNAJC6/EPN
N/ATP6V1A/UNC80/STOM/ATP7B/BEST1/ANO4/ATP6V0A1/ATP8B4/CLCA2/ATP6V1C1/TTYH2/

GALT1/DCTN1/LMAN1L/ANK1/GBF1/COL7A1/SPTBN1/DYNC1H1/B4GALT6/ANK2/ST8SIA6/FUT
NPP4B/PTEN/MGLL/PI4K2A/PLA2G2D/INPP4A/LPGAT1/CDS1/HADHB/ARF3/ENPP6/PLA2G4C/G

/TP53/HIST1H2AC/HIST1H2BD/HES1/FLT4/PSME2/HIST1H4H/HIST2H3A/PSMA7/PSMD8/HDAC

NB2/CALM1/GABRA3/ARHGEF9/PRKCA/RPS6KA6/PRKAG2/ADCY7/HTR3C/GABRB1/MAPT/GNAI

N/TTN/ATP2B1/KCND1/ATP1A2/TNNT3/SCN11A/ABCC9/SCN9A/AHCYL1/CACNB4/CASQ2/CA

/ARHGEF12/SCIN/MYH10/MRAS/GSN/MAPK1/PIK3CB/BDKRB1/ITGAM/INSRR/LIMK2/PIK3CD/P

/PRKCD/GHSR/GPR143/HCRTR1/PRKCA/ITPR2/MLNR/PRKCQ/GNA14/TACR2/TAC1/GRP/CHRM

KRT17/PCSK6/KRT83/KRT6B/TCHH/KRT86/KRT81/KRT82/KRT33A/DSG3/PKP2/FLG/EVPL/KRT6A/
1/SLC17A6/SLC30A2/SLC13A4/CALM1/SLC25A22/SLC25A18/SLC27A6/SLC30A10/SLC16A7/HEP
MRPL39/MTFMT/MRPL2/MRPL27/MRPL17/MRPL51/MRPL37/MRPL42/MRPS34/MRPL38/MRPS35
/RPS27A/INO80B/PSMC4/PSMD5/ACTR5/PSMB7/PSMC3/PSMD9/PSMA3/MCRS1/PSMB6/ACTB

PS1/GLE1/POM121/NUP155/SRSF1/NUP62/NUP188/DDX39B/SEC13/THOC7/ZC3H11A
H4H/CBX2/MDM2/HIST2H3A

PS8/SUMO1/COPS4/COPS7B/GPS1/RAD23A/ERCC3/COPS5

MPL/GP1BA/RASGRP1/HABP4/RAPGEF3/MAPK1/PIK3CB/PLCG2/DGKZ/ITIH3/SELP/PRKCD/PTPN
MC1

B10/PSMD12/PSMC1

/CACNA1H/PRKACB/MAP2K4/CACNA2D1/CACNA1D/FGF22/FGF2/MAP2K1/DUSP7/PRKX/FGF1
LRA4/NPTN/SLC6A1/CHRNA4/GRIA2/GAD1/MAOA/PRKX/DLG3/CHRNB2/CALM1/GABRA3/CAC

TAAR5/AVPR1B/GABBR1/NPY5R/ADRA2C/PRSS3/ADCYAP1R1/MC3R/GPR50/HTR4/ADRA1B/NP

/JAK1/NF1/PPP2R5C/RASGRP4/GAB2/SYNGAP1/CSF2RB/IL17RD/JAK3/ERBB3/PEBP1/RASGRP1/

CTN2/KSR1/DAB2IP/CDC42EP2/RASAL2/MAPK3/TNRC6B/JAK1/NF1/PPP2R5C/TNRC6C/RASGRI
PK3/TNRC6B/RRAGD/STRN/PPP2R5C/TNRC6C/RICTOR/ERBB3/CHD3/TNKS2/PHLPP2/MAPK1/F

GEF/RASGRF2

5/PENK/PTGDR/CCL21/TAAR5/AVPR1B/NPY5R/GPR68/PDYN/ADRA2C/MC3R/CCL19/HTR4/XK/
CACNB1/CNTN2/GFRA2/GFRA4/SPTB/CACNA1

57

3-15/IGHV4-59/IGKV1D-16/IGHV3-48/PAK1/IGHV3-11/IGLV6-57
V3-48/IGHV3-11/IGLV6-57
/MAG/RTN4R/PLEKHG5/MCF2/KALRN/NGEF/RASGRF2

2C/GDI1/TBC1D24/DENND4B/TBC1D10A/RAB3GAP1/ULK1/TSC1/SBF2/DENND5B/AKT3/RIN2/R

F/IFNA2/BMP15/CRNN/MEGF8/WNT11/S100A7/NGF/TNFSF15/CXCL5/IFNA8/FGF22/GDF11/FG

OR6T1/OR51Q1/OR2AK2/OR6A2/OR2D2/OR6J1/OR2K2/OR2M4/OR2C1/OR2H2/OR5AK2/OR3A

2AF

ARHGEF4/ARHGEF16/TIAM1/ITSN1/ARHGEF37/ARHGEF35/ABR/NET1/ARHGEF5/MAG/RTN4R/P

R10H5/CALM3/CLCA4/OR6B3/OR52W1/OR2T8/OR2A7/OR52N4/OR52N2/OR8S1/OR2T4/GNAL

LIPE/PDE3B/G6PC/PRKX/HK1/CALM1/PRKAG2/GYS2/MAPK9/MAPK10/PRKAR1B/CALM3/SORBS

D/QKI/BAG4/MIB1/CBL/HDAC4/PIK3AP1/VAV1/RASAL3/IRS1/CHUK/AKAP9/SEL1L/HDAC5/TTR/
R2/IGKV2D-40/IGLV2-11/PRKCQ/IGKV1-16/BTRC/MAPK9/IGHV1-46/MAPK10/AHCYL1/IGKV3-1

/AHCYL1/CASR/CX3CR1/PRKAR1B/DHRS9/GNAO1/RGS9/TAS2R4/PPP1R1B/PLCB4/CXCL12/LRP
GABRR3/CHRNA3/AP2B1/GNB5/GRIP2/SLC1A7/CACNA1A/KCNK16/RTN3/HOMER1/HCN4/GLUL

CC1/ACTR5/RBX1/MCRS1/ACTB/UBE2N/RFC5/CCNH/UBE2I/INO80E/RPA1/TCEA1/COPS8/POLF

E/HDAC1/MOV10/HIST1H2BN

B2/RHO/SSTR5/PENK/PTGDR/WNT7B/CCL21/TAAR5/WNT3A/AVPR1B/GABBR1/NPY5R/GPR68/
/CYBB/VAV1/IRS1/NTRK3/PTK2/ROCK1/COL3A1/ADORA2A/CLTC/ATP6V0C/BDNF/IGF2/DUSP3

-57
/PDE8A/PDE3B/GPR27/OR2C1/OR2H2/OR5AK2/OR3A3/OR10H2/OR13A1/OR13J1/PDE11A/CA
-11/IGLV6-57
PRKAG1/RFC5/RAD9A/TOP3A/TP53RK

TNF
/PSMD2/PSME3

L/AAAS/NUP43/TRMT61B/ADAT3/TRMT13/THADA/TPRKB/WDR4/NUP88/TRMT6/CDKAL1/TSEN

PSMD2/PSME3

G/PRMT1/HIST1H4H/FOXA1/CBFB/HIST2H3A/POLR2I/H2AFJ/NR5A2/HIST2H2BE/GTF2A2/HDAC
B/PRKCG/CAMK2A

E/POLR2E/COPS4/ISY1/COPS7B/GPS1/ERCC3/COPS5

GEF/RASGRF2
PR2/IGKV2D-40/IGLV2-11/IGKV1-16/BTRC/IGHV1-46/AHCYL1/IGKV3-15/IGHV4-59/IGKV1D-1

GF16

/PRKAG2/AKR1C3/THEM5/ELOVL7/FAAH/PON1/ACSL6/CYP1B1/PTGS2/CYP4A22/SCD/ALOXE3/
22/CYP1A1/CYP2A7/CYP46A1/CYP4A11/CYP26B1

I1

5/FGF16
3MX/PPIL6/SNRPA1/PRPF31/USP39/SF3B5/LSM6/SRSF2/SF3A2/POLR2C/SF3A3/SRRT/TRA2B/PC

.RC1/STAB2/ENO2/PFKP/PFKFB2/PGM2L1/FUT5/CSGALNACT1/CHST9/HS3ST2/FUT1/CHST15/F
CG/ITPR1/CAMK2A

F1/EIF4A3/SRSF7/SRSF6/DHX15/BCAS2/THOC1/DDX23/TRA2A/THOC3/U2AF2/SNRPA/SRSF10/

PSMA3/TUBB4B/TUBB2B/PSMB6

SEC13/WRN/NSMCE4A/NUP93/XRCC4/RAD52

4-59/IGKV1D-16/CR2/IGHV3-48/IGHV3-11/C6/IGLV6-57

T1H4H/C8A/HIST2H3A/SNRPD1/H2AFJ/HLA-DRA/C8G/HIST2H2BE/ACTN1/HIST1H2BN/HIST2H

CKL1/DUT/NME3/NME5/CTPS1/PNP/NT5C/POLR2C/TYMP/NT5M/POLR2D/UMPS/NME7/POLR1

/TP73/RBL1/CASP2/TP53/POLR2J/LAMTOR4/POLR2H/BAX/RAD9B/PRELID1/LAMTOR5/POLR2G

0A1/MEF2C/SFTPA1/DNM1

V2-5/IGKV2D-40/IGLV2-11/IGKV1-16/NCR3LG1/IGHV1-46/IGKV3-15/IGHV4-59/CD300LG/SIG

D12/PSMC1

)3

:1/OTOG/LRG1/THBS1/COL6A6/FBLN2/MEPE/COL7A1/EYS/IGSF10/NTNG2/ACAN/NELL2/COL5

SMA4/UBB/AP1S1/PSMA5/RPS27A/PSMC4/AAAS/NUP43/PSMD5/PSMB7/NPM1/NUP88/RBX1/

IIST1H2AC/HIST1H2BD/MMP2/ZNF217/POLR2J/CAV2/POLR2H/POLR2G/PRMT1/HIST1H4H/FO)

1/AURKA/NCAPH/CENPF/TK1/ORC1/GINS1/BUB1B/CENPM/CDC6/ESPL1/CDK6/TUBA3C/POLE2
'CENPI/HIST1H2AD/HIST1H4C/HIST1H4A/HIST1H2BJ/FBXO5/HIST1H2BL/HAUS1/CENPL/HIST1H
N1/RAD18/MAD2L2/ACTL6A/BRCA1/ISG15/PPP4C/HIST1H2BK/BARD1/LIG1/H2AFZ/TP53/HIST1
EIF3I/TRMT112/RPLP0/RPS15A/RPN2/RPL24/EIF3B/RPL17/MRPS33/MRPL14/MRPL4/MRPS7/RPN
H4C/HIST1H4A/RFC2/HIST1H2BJ/RPA3/HIST1H2BL/CENPL/ZWINT/ITGB3BP/CENPN/DSN1/CDC
'UBA52/RPS11/RPL32/RPL29/RPL35A/RPL36A/PSME1/PSMA4/RPL10A/UBB/RPL27A/PSMA5/RP
CENPO/PSME2/ANAPC11/CENPP/PSMA7/PSMD8/NDE1/BANF1/NUP85/LMNA/VRK1/NUP160/C
LD2/POLE/PSMD14/DNA2/CDC26/POLE3/PSMC2/PSMA1/PSMB4/PSMD13/PSMB1/UBA52/AKT2
C11/HIST1H4H/CBX2/IGFBP7/MDM2/HIST2H3A/POT1
N2/CEP152/NUP107/CENPO/HAUS7/CEP135/CENPP/NDE1/NUP85/TUBB/SSNA1/NCAPD2/NUP
P21/RPL36A/RRP9/RPL10A/RPL27A/RPLP2/NOP56/C1D/RPL12/RPL13/IMP3/RPS16/RPS13/RPS
RAN/RPL23A/RPS28/UBA52/RPS11/RPL32/RPL29/RAE1/RPL35A/RPL36A/RPL10A/RPL27A/RPLP2
3/RPA2/CCNE1/POLE/PSMD14/POLE3/PSMC2/PSMA1/E2F6/PSMB4/PSMD13/PSMB1/UBA52/HI
EN1/XRCC3/RPA2/POLD2/POLE/DNA2/HUS1/RHNO1/POLE3/PAXIP1/HIST2H2BE/RMI1/UBA52/
M5/MCM3/RAD9B/ORC5/PSME2/HIST1H4H/PSMA7/PSMD8/RPA2/PSMD14/DNA2/HUS1/RHNO

2/RPA2/H2AFJ/KCNU1/SUN1/CATSPERD/HIST2H2BE/CATSPER3/SYCP1/MLH1
3/RPS27/RPLP1/EIF2B1/RPS9/RPL28/RPL38/EIF4A1/RPS4X/RPL37A/RPL10/RPS8/EIF3G/FAU/RPL

5/POLE3/PSMC2/PSMA1/PSMB4/PSMD13/PSMB1/UBA52/PSME1/PSMA4/ANAPC7/UBB/PSMA5

DNA2/HUS1/RHNO1/POLE3/HIST2H2BE/RMI1/UBA52/HIST1H2BN/SUMO2/UBB/UIMC1/RAD1/E
A/RPS3/MAT1A/RPS27/RPLP1/RPS9/RPL28/RPL38/RPS4X/SCLY/RPL37A/RPL10/RPS8/CTH
PL12/RPL13/RPS16/RPS13/RPS27A/RPS3/SRP9/SPCS2/SSR3/RPS27/RPLP1/RPS9/RPL28/RPL38/R
'RPS9/EIF4A3/RPL28/RPL38/RPS4X/RPL37A/RPL10/RPS8/FAU/PNRC2/RNPS1/SMG9/RPL7A/RPL
NUP205/NUP160/H2AFJ/SMC2

'RPS4X/RPL37A/RPL10/RPS8

2AFV/POLR1D/HIST2H3D

RAE1/HIST1H2BN/POLR2C/POLR2D/AAAS/HIST1H2BC/NUP43/H2AFV/HIST2H3D/NUP88

S9/RPL28/RPL38/RPS4X/RPL37A/RPL10/RPS8
HIST2H3D
C5/PSMB10/PSMD12/PSMC1

SMB1/UBA52/HIST1H2BN/PSME1/CDK7/PSMA4/UBB/PSMA5/RPS27A/PSMC4/HIST1H2BC/PSM
/CDC16/CDC23/ANAPC15/PSMB2/ANAPC4/PSMC6/PSMD3/ANAPC5/CUL1/PSMB10/PSMD12/
MC1

OLE4/CENPP/POT1/MIS18A/FEN1/RPA2/POLD2/H2AFJ/POLE/CENPQ/DNA2/POLE3/RUVBL1
SMD9/PSMA3/ORC2/PSMB6
B2/ANAPC4/PSMC6/PSMD3/ANAPC5/CUL1/PSMB10/PSMD12/PSMC1

SLBP/PRPF4/U2AF1L4/PPIL1/RBMX/PPIL6/SNRPA1/RAE1/PRPF31/USP39/SF3B5/LSM6/SRSF2/SF

C2/PSMA1/CETN2/PSMB4/PSMD13/NEDD1/PSMB1/UBA52/PSME1/CDK7/PSMA4/UBB/PSMA5/

PS11/RPL32/RPL29/RPL35A/RPP21/RPL36A/RRP9/RPL10A/RPL27A/RPLP2/NOP56/C1D/RPL12/R

TRK4/STX1A/GRM1/SHANK1/SYT10/NRXN3/SYT2/IL1RAPL2/GRIN1/DLGAP2/GRIN2B/SYT1/GRI[illegible]
[illegible]3/KCNQ5/KCNA1/KCNH3/KCNAB2/KCNA4/KCNC2/KCNJ4/KCNJ3/KCNG3/KCNB2/KCNS1/KCN[illegible]

CENPL/HIST1H3D/ZWINT/ITGB3BP/CENPN/DSN1/TUBA3E/KNTC1/H2AFX/NUP37/ZWILCH/HIS

PIK3C2G
13

2B/RASGRF1/GRIN2A/CAMK2A
EF4/STXBP1/KCNB1/AKAP5/ADCY1/ADRA2A/SYT5/PRKACG/GNG3/STX1A/ITPR1/KCNC2/SNAP

32A1/SLC6A20/SLC8A2/SLC5A8/SLC6A15/SLC17A7/SLC12A

WASF1/IGHV4-59/IGKV1D-16/MYO5A/WASF3/CYFIP2/IGHV3-48/PAK1/IGHV3-11/IGLV6-57
/CAMK4/NTF3/IRS4/CAMK2A
/DEK/EED/RBBP4/HIST1H2BC/TWISTNB/MBD3/H2AFV/POLR1D/HIST2H3D
/PLCB1/ADCY2/PRKCE/ADRA1A/MYH11/ADCY1/PRKACG/CYP4A11/ITPR1/PRKCB/PRKCG
YWHAH/CYFIP2/PAK1/MYOCD/PRKCE
UP160/H2AFX

M3/SLIT2/NGEF/PAK6/EPHB6/EPHA8/SLIT3/EPHA6

PSMC1

TPR2/DOCK1/IGKV2D-40/IGLV2-11/IGKV1-16/IGHV1-46/PLD1/AHCYL1/IGKV3-15/WASF1/IGH
CNK12/CAMK2G/KCNK4/CAMK2B/SCN1B/KCNIP2/KCNK1/CACNG8/SCN8A/RYR1/SCN2A/KCNK
F37/OPHN1/ARHGEF35/ABR/NET1/ARHGEF5/PLEKHG5/ARHGAP28/SYDE2/MCF2/KALRN/ARHG

R2/PRICKLE1/PPP3CA/WNT9B/PPP3CB/WNT7B/WNT3A/CAMK2G/SFRP1/CAMK2B/PLCB1/CHP2
YT1/PACSIN1

R/OPRM1/SSTR4/PROKR2/NPBWR2/MCHR2/NPBWR1
LCB4/PPP3R2/CACNA1S/PPP3CA/PPP3CB/PLCZ1/CACNA1F/PTGFR/ADRB2/CACNA1C/MYLK3/
/CXCL2/PRKX/CX3CL1/ADCY7/GNAI1/TIAM1/CCL8/PTK2B/CX3CR1/PLCB4/CXCL12/CCL11/CCL2

N3/IQSEC3/ARRB1/SH3GL2/DNM1/RET
/FXYD7/CALM1/PLN/ATP2B1/ATP1A2/TRPV5/TRPM2/SGK2/CLCA4/ATP9A/CASQ2/FXYD4/ATP8

T3/MGAT3/NSF/GRIA1/CGA/SPTBN4/ST8SIA3/ANK3/SPTBN2/TUBA8/TUBB4A/MGAT4C/SPTB/D
GPAT2/GDPD5/PIK3CB/AGPAT3/MTMR9/DDHD2/PIK3CD/SLC44A1/PITPNM1/SBF1/MTMR8/BM

C3/DLL4/H2AFJ/PSMD14/POGLUT1/YBX1/PSMC2/POFUT1/PSMA1/MIR34C/PSMB4/PSMD13/HI

I1/KIF17/PRKAR1B/APBA1/GABRB3/DLG4/NSF/GRIA1/GLRA3/EPB41L1/GRIN3A/GNAL/ADCY5/G

ACNA1S/FGF12/HIPK2/KCNK17/MYL3/LMOD1/SORBS1/CACNB2/CACNA1F/CACNA1C/CACNA2

FGF22/APC/FGF2/BAIAP2/MAP2K1/MYH14/MYL2/FGF17/MYLK/DOCK1/ARHGAP35/FGFR2/ARH

5/DGKI/EDNRB/TRHR/CASR/QRFPR/PLCB4/NTSR2/UTS2R/PTGFR/AVPR1B/DGKB/GPR68/KALRN

/KRT79/SPRR2E/KLK8/DSG1/KRT3/KRT33B/KLK5/KRT31
PH/SLC16A8/SLC38A1/SLCO3A1/LCN12/SLC6A5/SLC9A5/SLC9A6/SLC22A2/SLC7A8/SLC1A4/SL
5/MRPS36/MRPS10/MRPL9/DAP3/MRPL48/MRPS23/MRPL20/MRPS18C/MRPL24/MRPL40/MRPS
/INO80E/PSMB2/PSMC6/PSMD3/PSMB10/ADRM1/PSMD12/PSMC1

N11/SYTL4/CALM1/CLEC3B/THBS1/PRKCA/ITPR2/PRKCQ/IGF1/TTN/GNA14/PLG/DGKI/GNAI1/S

17/PPP3R1/CACNB3/PRKCA/RPS6KA6/MAPK8IP3/FGFR2/NR4A1/MAPK9/MAPK10/RAPGEF2/MA
CNB3/ARHGEF9/PRKCA/RPS6KA6/SLC38A1/PRKAG2/VAMP2/ADCY7/HTR3C/GABRB1/MAPT/GN

PY1R/ADRB3/TSHR/CRHR1/OPRD1/TSHB/THRB/GRIN2C/CHRM4/CHRM3/VIPR1/RXFP2/TACR3/

/NCAM1/MAPK1/PIK3CB/SPTAN1/FGF22/FGF2/MAP2K1/DUSP7/PTPN11/PDE3B/DLG3/FGF17/K

P4/SYNGAP1/CSF2RB/IL17RD/JAK3/ERBB3/PEBP1/RASGRP1/NCAM1/IL6R/MAPK1/PRKACB/SPT/
PIK3CB/PRKACB/PIK3CD/FGF22/FGF2/PRKCD/PTPN11/PRKX/FGF17/KLB/CALM1/PRKCA/ITPR2/N

ADRA1B/NPY1R/P2RY12/ADRB3/TSHR/OPRD1/TSHB/FFAR1/CHRM4/CHRM3/RXFP2/TACR3/OX

ABEP1/RABGAP1/DENND5A/TBC1D14/RAB18/RAB3IL1/TRAPPC10/SBF1/MADD/OPTN/RAB27B

F2/ANGPTL5/GDF6/CBLN1/CXCL2/EDA/CFC1/FGF17/WNT5B/IL17D/IFNW1/VWC2L/NRG4/IGF1

3/OR10H2/OR13A1/OR13J1/OR4A47/OR2L2/OR10H5/OR6B3/OR52W1/OR2T8/OR2A7/OR52N

LEKHG5/MCF2/KALRN/NGEF/TNF/RASGRF2

/OR2L13/OR9A2/PRKG2/CAMK2G/CALML3/CAMK2B/OR3A1/OR1E1/OR1E2/OR2B11/OR1F1/O

31/PKLR/CALML3/PRKCZ/SLC2A4/PRKACG/EIF4E1B/IRS4

PDPK1/KL/VCL/BCR/MYC/KSR1/DAB2IP/CASP9/TCF7L2/CTBP2/EP300/RASAL2/AKT3/PTEN/SMA
15/IGHV4-59/IGKV1D-16/PPP3CA/PPP3CB/IGHV3-48/PAK1/IGHV3-11/IGLV6-57/ITPR1

2/PPP3CA/P2RY13/PPP3CB/SSTR1/RHO/SSTR5/PENK/GNAL/CCL21/ADCY5/GABBR1/NPY5R/PD
L/LRRTM4/DNAJC5/KCNJ10/MAPK1/KCND2/PRKACB/KCNG2/PRKAA2/SLC6A12/GLRA4/NPTN/S

R2K/SUMO1/PPIE/POLR2E/COPS4/ISY1/COPS7B/GPS1/RAD23A/ERCC3/COPS5

PDYN/ADRA2C/ADCYAP1R1/MC3R/CCL19/HTR4/ADCYAP1/XK/ADRA1B/NPY1R/P2RY12/ADRB
3/PDPK1/KL/RBFOX2/MST1R/ELMO2/RAPGEF1/COL4A5/ELMO1/SPARC/AKT3/WWP1/STAT6/NC

LCRL/PDE8B/OR4A47/GIP/INSL3/ADCY7/OR2L2/GPR20/GNAI1/OR10H5/GLP1R/HTR7/PTGER2/

N54/PUS1/OSGEP/PUS3/TP53RK/POM121/FTSJ1/NUP155/NUP62/NUP188/GTPBP3

1/MOV10/USF1

6/PPP3CA/CD22/PPP3CB/IGHV3-48/IGHV3-11/IGLV6-57/ITPR1/PRKCB

/CYP1A1/PTGDS/ACBD7/CYP4A11/SLC27A2

)LR2D/SRSF9/LSM3/SNRNP25/U2AF1/EIF4A3/SRSF7/SRSF6/DHX15/BCAS2/DDX23/HNRNPL/U2

UT3/B3GNT4/HS3ST5/IDS/B3GAT2/GOT1/PKLR/CHST1/FUT9/HAS1/OGN/OMD/PRKACG/LYVE1

HSPA1B/RBM22/CCDC12/SF3B4/SRSF1/HNRNPA1L2/PPIE/DDX39B/EFTUD2/ISY1/HNRNPC/CW

I2BF/H2AFY/HLA-DMB/HLA-DPA1/FCGR2C/IFNG/CD80/C5/HIST1H2BC/H2AFV/HIST2H3D/HLA

.D/DHODH/NME4/POLR3K/PNPT1/CDA

/PRMT1/RHEB/TP53AIP1/COX7B/TP63/NDUFA4/GTF2H2/MDM2/TP53I3/DDB2/POU4F2/LAMTC

;LEC6/IGKV1D-16/SH2D1B/CD22/SFTPD/IGHV3-48/IGHV3-11/IGLV6-57/LILRB5/ICAM5/CLEC4C

A1/FBN2/SMOC1/SPARCL1/DPT/NTN4/SPON1/SNED1/TSPEAR/SPOCK1/RSPO1/THSD4/SPOC

PSMC3/PSMD9/PSMA3/PSMB6/SLC25A5/RAC1/HMGA1/PSMB2/POM121/CD8B/PSMC6/PSMD

A1/CBFB/GNGT1/CAV1/HIST2H3

2/HIST2H2AC/PLK1/MAD2L1/PKMYT1/ORC6/CDCA5/E2F2/SMC4/MCM2/CENPH/PLK4/CDK2/PC
H3D/ZWINT/ITGB3BP/CENPN/HAUS8/DSN1/TUBA3E/PSMB8/KNTC1/PSMB9/H2AFX/NUP37/PSM
H2AC/HIST1H2BD/POLR2J/POLD1/SLX1A/POLR2H/FANCG/RAD9B/POLD3/TIPIN/SLX1B/POLR2
N1/MRPL34/MRPS6/RPS27L/RARS2/MRPL12/CARS2/AIMP2/SEC61A1/RPL23A/RPS28/MRPL15/U
25A/RFC4/PSMB8/KNTC1/RFC3/RBBP8/PSMB9/H2AFX/MCM7/NUP37/CHEK2/MCM4/MCM6/PS
LP2/RPL12/RPL13/RPS16/RPS13/RPS27A/RPS3/ROBO3/PSMC4/RPS27/PSMD5/RPLP1/RPS9/EIF4
CENPQ/PSMD14/CDC26/PSMC2/PSMA1/PMF1/MIS12/PSMB4/PSMD1
2/PSME1/CDK7/PSMA4/ANAPC7/UBB/PSMA5/RBBP4/RPS27A/E2F5/PSMC4/CCNE2/PSMD5/PSM

P160/HAUS5/ODF2/CENPQ/SMC2/TUBG1/PMF1/CETN2/MIS12/NEDD
27A/RPS3/UTP6/RRP36/UTP18/RPS27/NHP2/RPLP1/DCAF13/EMG1/RPS9/WDR46/RPL28/RPL38
2/RPL12/RPL13/POLR2C/RPS16/RPS13/RPS27A/RPS3/POLR2D/AAAS/RPS27/NUP43/RPLP1/RPS9
DAC1/AKT2/PSME1/CDK7/PSMA4/UBB/PSMA5/RBBP4/RPS27A/E2F5/PSMC4/CCNE2/PSMD5/RP
/POLM/HIST1H2BN/SUMO2/UBB/UIMC1/RAD1/BABAM1/TDP1/POLH/RPS27A
D1/PSMC2/PSMA1/PSMB4/PSMD13/HIST2H2BE/PSMB1/RMI1/UBA52/HIST1H2BN/PSME1/PSM

7A/RPL18/EIF3M/RPL13A/EIF3C/RPL8/EIF2B4

/RPS27A/PSMC4/CCNE2/PSMD5/RPA4/PSMB7/RBX1/ANAPC10/PSMC3/PSMD9/PSMA3/ORC2

BABAM1/POLH/RPS27A

RPS4X/RPL37A/RPL10/RPS8
18

D5/H2AFV/PSMB7/HIST2H3D/PSMC3/PSMD9/PSMA3/TCF3/PSMB
PSMC

F3A2/POLR2C/SF3A3/SRRT/TRA2B/POLR2D/SRSF9/LSM3/SNRNP25/AAAS/NUP43/U2AF1/EIF4A

/AJUBA/FKBPL/TUBA1B/RBBP4/CEP76/RPS27A/DCTN3/PSMC4/PSMD5/NME7/CEP41/PSMB7/RE

PL13/IMP3/RPS16/RPS13/RPS27A/RPS3/UTP6/RRP36/UTP18/RPS27/NHP2/RPLP1/DCAF13/EMC

N2A
J12/KCNH5/KCNS2/HCN1/KCNV

T1H2BK/TUBA1A/H3F3A/H2AFZ/PKN3/MYL6/TUBB6/RHOC/B9D2/DIAPH3/HIST1H2AC/HIST1H2

25/GNG13

IV4-59/IGKV1D-16/MYO5A/WASF3/CYFIP2/IGHV3-48/PAK1/IGHV3-11/PRKCE/IGLV6-57/ITPR1
K18/ATP1A3/SCN5A/ATP2B2/FGF13/SCN3B/SCN7A/CACNA2D3/KCNK9/ITPR1/SCN2B/KCNJ4/S
AP36/ARHGDIG/CHN1/RHOV/NGEF/ARHGAP44/RASGRF2

/WNT1/DAAM2/PRKACG/DKK2/SFRP2/PRKCB/WNT10B/WIF1/PRKCG/CAMK2A

GNAL/AVPR1B/ITPKA/ERBB4/CACNA1E/ADCY8/CD38/CAMK2G/HTR4/CALML3/CAMK2B/ADRA
21/ADCY5/ADCY8/CCL19/PAK1/PLCB1/PRKCZ/ADCY2/CCR9/ARRB1/ADCY1/PRKACG/GNG3/GN

3A1/TRPM3/ATP4A/ATP1B1/ATP6V1G2/CLCN4/NALCN/CAMK2G/CAMK2B/TRPM6/TRPA1/RYR1

YNC1I1/TUBA4A/CAPZA3/NAPB/F[illegible]
X/CDS2/PLEKHA6/GPAM/LPCAT4/PITPNM2/TNFAIP8L3/SYNJ2/PIP4K2A/PLD1/LPIN1/PLEKHA1/

ST2H2BE/TMED2/PSMB1/UBA52/HDAC9/HDAC1/STAT1/MOV10/HIST1H2BN/PSME1/PSMA4/A

GABBR1/ERBB4/KCNJ6/GRIP1/CAMKK2/ADCY8/CAMK2G/TUBA8/CAMK2B/GRIN2C/KCNJ9/CACN

D2/TMOD2/FXYD4/CACNB1/ATP1B1/KCNK12/CAMK2G/KCNK4/CAMK2B/SCN1B/KCNIP2/KCN

GEF4/ITGA8/CHRM5/PIP4K2A/TIAM1/WASF1/FGF9/PAK3/FGF8/FGF3/FGF12/CYFIP2/PIP5K1B/M

N/ADRA1B/FFAR1/PLCB1/CHRM3/TACR3/GRM5/PRKCE/KNG1/ADRA1A/OPN4/DGKE/GNG3/HT

C5A11/SLC13A1/SLC26A9/APOD/SLC30A8/SLC1A6/SLC1A2/SLC5A12/SLC22A6/SLC15A2/SLC6
S11/MRRF/MRPL16/MRPL46/MRPL44/ERAL1/MRPL18/MRPL1

CG3/TF/PHACTR2/RAB27B/DGKB/ADRA2C/P2RY12/PCDH7/PRKCZ/RAPGEF4/PRKCE/KNG1/ARF

APK13/RPS6KA5/MAPT/HSPA2/IL1B/CACNB4/FGF9/FGF8/FLNB/PPP3R2/FGF3/CACNA1S/FGF12
NAI1/SLC22A2/KIF17/ALDH5A1/CACNB4/PRKAR1B/APBA1/GLS/GABRB3/DLG4/NSF/GRIA1/GLR

GABRG2/GABBR2/GRM5/NPY4R/ADRA1A/HTR6/FSHR/MC4R/HRH3/GRM3/ADRA2A/GABRA2/P

KLB/CALM1/NRG4/SPTBN1/FGFR2/PEA15/VAMP2/IL34/RAPGEF2/PPP2R5A/CSF1R/FGF9/FGF8/F

AN1/FGF22/FGF2/MAP2K1/DUSP7/PTPN11/DLG3/FGF17/KLB/CALM1/NRG4/SPTBN1/FGFR2/PE
NRG4/FGFR2/FOXO4/NR4A1/ADCY7/PIP4K2A/IER3/PPP2R5A/FRK/AHCYL1/PRKAR1B/FGF9/FGF8

KGR1/CCR9/KNG1/NPY4R/ADRA1A/HTR6/FSHR/MC4R/HRH3/ADRA2A/OPN4/PRLHR/RXFP3/SS

/RAB6B/RAB3A

/WNT8A/SCUBE3/CX3CL1/IL34/WNT4/IFNA13/INSL3/HHIP/WNT8B/WNT2B/IL1B/CCL8/FGF9/F

4/OR52N2/OR8S1/OR2L5/OR2T4/GNAL/OR2L13/OR2AJ1/OR9A2/RTP1/OR3A1/OR1E1/OR1E2/

R2AG2/OR52E2/OR2W3/PRKACG/GUCA1C/OR52E8/CAMK2A/OR14I1/CNGB1

AD3/MAPK3/TGFBR2/STRN/NF1/PPP2R5C/RICTOR/GAB2/SYNGAP1/CLCN6/ERBB3/PEBP1/TNKS

DYN/ADRA2C/CAMKK2/ADCY8/CAMK2G/CCL19/RGS7/CAMK2B/NPY1R/P2RY12/OPRD1/CLPS/(
SLC6A1/KCNH6/KCNS3/CHRNA4/GRIA2/GAD1/MAOA/PRKX/KCNN2/DLG3/KCNH4/CHRNB2/K(

3/TSHR/CRHR1/OPRD1/TSHB/PTH/FFAR1/CHRM4/CHRM3/VIPR1/RXFP2/TACR3/CRHBP/OXGR
CKAP1L/TEC/MAPK3/AXL/RPS6KA2/HPN/FLT1/PTPRK/WWOX/AP2B1/TNS4/RICTOR/ATP6V1H/(

/OR6B3/CGA/OR52W1/CALCR/OR2T8/OR2A7/OR52N4/ADRB2/OR52N2/OR8S1/OR2L5/OR2T4/

AF2/DNAJC8/PCBP2/CSTF1/SNRPA/RNPS1/HNRNPA2B1

/NDST3/HS3ST4/HS6ST3/HPSE

C15/CRNKL1/TXNL4A/HSPA

-DPB1/CD40LG/C1

OR2/POLR2I/RPA2/CCNE1/TAF7/DNA2/PIP4K2C/HUS1/RHNO1/FAS/CYCS/COX5B/COX6B1/RM

G/SELI

2/HMCN2/COL19A1/COL11A1/PRG4/ADIPOQ/DMBT1/RSPO2/TINAG/EDIL3/CHAD/SPOCK3/L

3/NUP155/CD247/NUP62/NUP188/PSMB10/PSMD12/PSMC1/SEC1

CNA/LMNB1/DBF4/LPIN3/CENPI/HIST1H2AD/MCM10/CDT1/CDKN2A/E2F1/HIST1H4C/HIST1H4
MA2/NCAPG2/ZWILCH/TMPO/HIST1H2BK/TUBA1A/H3F3A/NEK6/H2AFZ/TUBB6/B9D2/HIST1H2
2G/HIST1H4H/GTF2H2/POLE4/POT1/DDB2/FEN1/XRCC3/POLR2I/RPA2/POLD2/H2AFJ/POLE/M
BA52/MRPL21/RPS11/SEC61B/RPL32/MRPL49/RPL29/MRPS26/MRPL33/RPL35A/SARS2/MRPS1
SMA2/ZWILCH/MCM8/BRCA1/HIST1H2BK/BARD1/TP53/CDC7/B9D2/HIST1H2BD/MCM5/MCM
4A3/LHX9/PSMB7/RPL28/RPL38/RPS4X/RBX1/RPL37A/RPL10/RPS8/PSMC3/PSMD9/PSMA3/PS

MB7/E2F4/RBX1/ANAPC10/PSMC3/PSMD9/PSMA3/ORC2/PSMB6/CDC16/RFC5/LIN37/CDC23/

3/NOP2/RPS4X/RPL37A/RPL10/DKC1/RPS8/EXOSC2/UTP20/NOC4L/TEX10/RIOK3/FAU/NOL12/
9/RPL28/RPL38/RPS4X/NUP88/RPL37A/RPL10/RPS
PA4/PSMB7/E2F

A4/UBB/PSMA5/UIMC1/RAD1/BABAM1/RPS27

/PSMB6/CDC16/RFC5/CDC23/ANAPC15/POLA1/GMNN/PSMB2/ANAPC4/RPA1/PSMC6/PSMD

3/SRSF7/SRSF6/DHX15/BCAS2/NUP88/THOC1/DDX23/THOC3/SARNP/HNRNPL/U2AF2/DNAJC

3X1/HAUS2/PSMC3/PSMD9/PSMA3/TUBB4B/MZT1/TUBB2B/PSMB6/PPP2R3

G1/RPS9/WDR46/RPL28/RPL38/NOP2/RPS4X/RPL37A/RPL10/DKC1/RPS8/EXOSC2/UTP20/NOC4

2BD/NUP107/MYL12B/CENPO/RHOD/HIST1H4H/IQGAP1/WIPF3/RHPN2/CENPP/HIST2H3A/ND

LC8A2/NOS1/ATP2B3/KCNJ12/RYR2/CACNG3/CAMK2

1B/ADRB3/GRIN2C/CAMK4/PLCB1/CHRM3/ADCY2/CHP2/TACR3/RYR1/GRM5/ATP2B2/ADRA1/
NG13/PRKC

l/ATP1A3/ATP2B2/ASIC2/WNK2/ATP8A2/TRPV6/TRPC5/ATP2B3/ANO3/RYR2/CAMK2/

'PLA1A/SYNJ1/PLA2G4D/PLA2G3/PIP5K1B/PLEKHA5/PI4KA/INPP5J/INPP5F/MTMR7/AWAT2/ETI

.TP2A1/UBB/PSMA5/APH1A/MIR200B/DTX2/RPS27A/NOTCH4/PSMC4/HIST1H2BC/PSMD5/H2/

NG8/CAMK4/PLCB1/TUBB4A/ADCY2/GABRG2/DLG2/GABBR2/AKAP5/LRRC7/ADCY1/GABRA2/P

K1/PAK1/CACNG8/SCN8A/RYR1/SCN2A/KCNK18/ATP1A3/SCN5A/ATP2B2/FGF13/MYH8/SCN3

1YLK3/ITGA9/FGF18/MOS/CHRM4/PAK1/CHRM3/FGF5/FGF13/PAK6/CHRM1/CHRM2/FGF1

R2A/NPFFR1/GRM1/ITPR1/CHRM1/CCK/GNG13/HTR2C/NPFFR2/HCRTR2/CCKBR/LPAR3/RGS4/

A13/SLC2A12/SLC45A3/SLC6A3/SLC13A2/SLC36A2/SLC7A2/SLC9A2/SLC6A18/SLC24A4/SLCO1

RB1/ADRA2A/TUBA4A/DGKE/GNG3/GP9/ITPR1/GNG13/PRKCB/F5/PRKC(

/MAPK8IP2/PPP3CA/MAP3K5/PLA2G3/DUSP8/PPP3CB/CACNB2/CACNA1F/CACNA1C/CACNA2
A3/CACNB2/SLC1A6/SYN3/ALDH2/SLC1A2/PPFIA3/EPB41L1/PPFIA2/GRIN3A/CACNA2D2/GNA

'TH2R/GABRD/PRLHR/GRM2/HTR1A/GRM7/GRM4/FSHB/HTR2A/NPFFR1/GRM1/DRD1/PTGER3/

GF3/DLG4/IFNL1/KIT/SPTBN4/DUSP8/FLT3/ERBB4/SPTBN2/CSF2/CAMK2G/FGF18/CNKSR2/CAM

A15/RAPGEF2/PPP2R5A/FGF9/PAK3/FGF8/FGF3/DLG4/KIT/SPTBN4/DUSP8/FLT3/ERBB4/SPTBN
3/FGF3/KIT/IL1RL1/CBX6/PIP5K1B/ADCY5/ERBB4/CAMKK2/ADCY8/CAMK2G/FGF18/CAMK2B/C

T/HTR1A/FSHB/HTR2A/NPFFR1/DRD1/PTGER3/CHRM1/CCK/OPRK1/HTR2C/HRH2/CHRM2/HT

LG2/IFNA21/FGF8/WNT2/FGF3/FGF12/CXCL12/S100A7A/WNT9B/CCL11/HRNR/WNT7B/CCL21/

/OR2B11/OR1F1/OR2AG2/OR52E2/OR2W3/OR52E8/OR14I1

32/KDM7A/MAPK1/PIK3CB/TRAK1/PIK3CD/JAG2/POLR2F/FGF22/APC/FGF2/MAP2K1/KIAA1549/

CHRM4/AKR1C1/CAMK4/PLCB1/ADCY2/OXGR1/GABBR2/PPEF1/AWAT2/CCR9/KNG1/TAS2R3/N
CNMB4/CALM1/GABRA3/CACNB3/ARHGEF9/PRKCA/RPS6KA6/KCNG1/SYT12/SLC38A1/PRKAG2

1/GABBR2/GRM5/CCR9/KNG1/WNT1/TAS2R3/NPY4R/ADRA1A/HTR6/FSHR/MC4R/HRH3/GRM3
GAB2/NGF/COL24A1/ERBB3/ATP6V1B2/MAPK1/PIK3CB/FLRT1/MEF2A/PRKACB/STAM/LAMA3/A

/PTGDR/GNAL/OR2L13/TAAR5/ADCY5/OR2AJ1/OR9A2/RTP1/ADCY8/ADCYAP1R1/MC3R/HTR4

I1/UBA52/HDAC1/MOV10/MLH1/ZNF420/TAF12/COX8A/TNFRSF10B/AKT2/COX6A1/CDK7/MD

GI3/VWA5B2/NELL1/OTOL1/ABI3BP/OGN/VWA7/COL10A1/OMD/SLIT2/COL26A1/SLIT3/EPYC/

4A/RFC2/HIST1H2BJ/RPA3/CKS1B/FBXO5/HIST1H2BL/HAUS1/CENPL/HIST1H3D/ZWINT/ITGB3B
2AC/HIST1H2BD/DCTN2/CEP152/NDC1/NUP107/CENPO/HAUS7/NUP210/PSME2/ANAPC11/HI
UTYH/INO80C/UNG/DNA2/HUS1/RHNO1/POLE3/USP1/RUVBL1/COPS6/PAXIP1/CETN2/COPS3
18A/RPL36A/MRPL52/EIF2B3/AURKAIP1/MRPL55/RPL10A/PPA2/RPL27A/MRPL39/RARS/RPLP2/N
3/NUP107/RAD9B/CENPO/ORC5/PSME2/ANAPC11/HIST1H4H/MDM2/CENPP/PSMA7/PSMD8/N
MB6/FAU

ANAPC15/POLA1/CCN

RBM28/FBL/BYSL/RPL7A/EXOSC3/RPL18/EXOSC4/RPL13A/NOP14

:8/PCBP2/CSTF1/SNRPA/RNPS1/HNRNPA2B1/GLE1/SRSF10/RBM22/POM121/SF3B4/NUP155/H

4L/TEX10/RIOK3/FAU/NOL12/RBM28/FBL/BYSL/RPL7A/EXOSC3/RPL18/EXOSC4/RPL13A/NOP1

[illegible]E1/NUP85/PFN1/NCF1/PPP1CB/NUP160/H2AFJ/CENP[illegible]

[illegible]A/HTR6/ADCY1/PRKACG/CACNA1I/PDE1B/HTR2A/GRM1/DRD1/PDE1A/ITPR1/PTGER3/CHRM1[illegible]

\NPPL/CPNE7/PITPNM3/CPNE6/PIK3C2(

AFV/PSMB7/HIST2H3D/RBX1/PSEN2/PSMC3/PSENEN/PSMD9/PSMA3/RFNG/NCSTN/PSMB

'RKACG/TUBA4A/HTR3A/GNG3/CAMKK1/GABRA4/KCNJ4/GNG13/RASGRF2/KCNJ3/PRKCB/CHF

B/MYH11/TRIM72/SCN7A/CACNA2D3/KCNK9/ITPR1/SCN2B/KCNJ4/SLC8A2/NOS1/TNNT2/ATF

'PROKR2/MCHR2

A2/LCN1/SLC38A4/SLC5A7/SLC5A5/SLCO4C1/LCN15/SLC24A2/SLC4A10/SLC2A4/SLC14A1/SL(

2D2/CACNB1/CACNA1E/FGF18/MEF2C/NTRK2/MOS/DUSP2/PAK1/IL1R1/CACNG8/CHP2/FGF5/
L/SLC6A13/ADCY5/GABBR1/ERBB4/CPLX1/CACNB1/KCNJ6/GRIP1/CAMKK2/PPFIA4/CACNA1E/

/GPR83/GABRA4/CRHR2/CHRM1/GLP2R/OPRK1/HTR2C/HRH2/CHRM2/HTR1B/GABRG1/RXFP1

MK2B/DUSP2/FGF5/DLG2/GFRA2/RASGEF1A/GFRA4/SPTB/LRRC7/ARRB1/NRG3/KSR2/RASAL1/S

2/CSF2/CAMK2G/KALRN/FGF18/CNKSR2/CAMK2B/DUSP2/PAK1/FGF5/DLG2/GFRA2/RASGEF1A
AMK4/ADCY2/FGF5/PRKCE/ADCY1/PRKACG/NRG3/PDE1B/CAMKK1/PDE1A/ITPR1/FGF16/PRKC

R1B/RXFP1/NPFFR2/HCRTR2/CCKBR/HTR1E/LPAR3/HTR5A/DRD5/OPRM1/SSTR4/PROKR2/NPBW

/WNT3A/CBLN4/TDGF1/GDF2/CSF2/TNFSF9/TPO/S100A1/SFRP1/CCL19/FGF18/CRLF1/VWC2/T

/PTPN11/MPRIP/ZFYVE9/HEY2/FGF17/KLB/CALM1/MYO18A/NRG4/RNF43/FGFR2/FOXO4/NR4/

NPY4R/HRH3/GRM3/ADCY1/ADRA2A/PRKACG/PDE1B/GRM2/RXFP3/SST/GNG3/CAMKK1/RBP4

2/KCND1/VAMP2/SLITRK1/NRXN1/SLITRK5/ADCY7/HTR3C/HCN2/KCNA5/ABCC9/GABRB1/SYT

3/ADRA2A/OPN4/PTH2R/PRLHR/GRM2/RXFP3/SST/GNG3/HTR1A/GRM7/GRM4/FSHB/HTR2A/N

ATP6V1A/POLR2F/FGF22/FGF2/BAIAP2/PRKCD/DUSP7/ATP6V0A1/PTPN11/PDE3B/ABI1/ATP6V

/ADCYAP1/GPR150/ADRB3/TSHR/CRHR1/TSHB/PTH/OR3A1/OR1E1/OR1E2/VIPR1/ADCY2/RXF

M4/TXN/UBB/TAF7L/RBBP4/POLR2C/NELFE/RAD1/SUPT4H1/RPS27A/POLR2D/MSH2/CCNE2/T

RSPO3/RELN

P/CENPN/HAUS8/DSN1/TUBA3E/CDC25A/RFC4/PSMB8/KNTC1/RFC3/PSMB9/H2AFX/MCM7/N
ST1H4H/CEP135/CENPP/HIST2H3A/PSMA7/PSMD8/NDE1/NUP35/BANF1/NUP85/TUBB/NUP54
/HIST2H2BE/PNKP/RMI1/FANCF/UBA52/TDG/MLH1/FANCE/POLM/ADPRHL2/HIST1H2BN/NTH
MTFMT/MRPL2/MRPL27/MRPL17/RPL12/RPL13/MRPL51/EIF3K/FARS2/RPS16/RPS13/RPS27A/R
NDE1/NUP85/NUP160/RPA2/CCNE1/CENPQ/PSMD14/DNA2/CDC26/HUS1/RHNO1/PSMC2/PS

NRNPD/SRSF1/NUP62/POLR2K/NUP188/CSTF3/PPIE/DDX39B/SEC13/THOC7/ZC3H11A/POLR2

/HTR2C/HRH2/SLC8A2/CHRM2/NOS1/PRKCB/GRIN1/CACNA1B/ATP2B3/CCKBR/HTR5A/GRIN2[illegible]

RNA2/GRIN1/NRGN/KCNJ12/GABRG3/GRIN2B/RASGRF1/GRIN2A/GABRA5/GABRA1/CHRNB3/C

2B3/KCNJ12/RYR2/CACNG3/CAMK2

C32A1/SLC6A20/SLC8A2/SLC5A8/SLC6A15/SLC17A7/SLC30A3/SLC22A8/SLC12A5/SLC6

NTF3/FGF13/ARRB1/PRKACG/CACNA2D3/CACNA1I/PTPN5/PTPRR/TNF/RASGRF2/PRKCB/FGF1
ADCY8/CAMK2G/SLC6A3/TUBA8/CAMK2B/SLC18A3/GRIN2C/KCNJ9/CACNG8/CAMK4/PLCB1/

/CHRNA2/NPFFR2/HCRTR2/GRIN1/CCKBR/GABRG3/HTR1E/LPAR3/GRIN2B/HTR5A/GRIN2A/GA

STX1A/RET/SNAP25/RASGRF2/FGF16/GRIN1/GRIN2B/RASGRF1/CAMK2/

/GFRA4/SPTB/LRRC7/ARRB1/PRKACG/NRG3/KSR2/RASAL1/RET/RASGRF2/FGF16/GRIN1/GRIN2
G/CAMK2/

WR2/MCHR2/NPBWR1

CHH/CRHBP/FGF5/NTF3/INHBA/BMP3/WNT1/FGF13/FLG/TNFSF18/IL9/NRG3/GDF7/GDF10/SC

A1/HDAC11/FBXW7/PPP2R5A/ESRP1/FGF9/FGF8/FGF3/KIT/WNT3A/ERBB4/CAMK2G/FGF18/CN

/HTR1A/GRM7/GRM4/PDE1A/ITPR1/PTGER3/OPRK1/GNG13/CHRM2/HTR1B/GUCA1C/HTR1E/L
9/MAPT/EPB41L2/GNAI1/SLC22A2/KIF17/SHANK3/KCNJ1/ALDH5A1/KCNAB1/CACNB4/PRKAR1

NPFFR1/GRM1/DRD1/PTGER3/CRHR2/CHRM1/CCK/GLP2R/OPRK1/GNG13/HTR2C/HRH2/CHRM
1C1/EPS15/FGF17/KLB/CALM1/APOE/THBS1/COL6A6/PRKCA/ITPR2/LRIG1/DOCK1/CDK5R1/NR

P2/OR2B11/OR1F1/PDE2A/OR2AG2/OR52E2/HTR6/FSHR/ARRB1/MC4R/OR2W3/ADCY1/PTH2F

AF11/NELFCD/MBD3/TAF13/SURF1/E2F4/NPM1/ING2/DAXX/PLK3/COX14/CDK5/CSNK2B/COX

UP37/GINS4/CCND2/MCM4/MCM6/PSMA2/NCAPG2/BORA/ZWILCH/MCM8/PRIM1/TMPO/HIS
4/SSNA1/LMNA/VRK1/NCAPD2/NUP205/NUP160/HAUS5/ODF2/H2AFJ/CENPQ/PSMD14/SMC2
L1/FANCL/ALKBH2/DCLRE1B/CDK7/SUMO2/ERCC8/UBB/HMGN1/POLR2C/UIMC1/RAD1/BABAI
PS3/SRP9/WARS2/MRPL37/MRPL42/SPCS2/EEF1A1/SSR3/RPS27/MRPS34/RPLP1/EIF2B1/RPS9/N
MA1/PMF1/MIS12/PSMB4/PSMD13/HIST2H2BE/PSMB1/NSL1/RMI1/UBA5

E/EFTUD2/ELAVL

A/DRD5/RYR2/PRKCG/CAMK2.

ACNG3/GABRA6/GABRB2/PRKCG/CAMK2A/HTR3

6/CACNA1B/RASGRF1/CACNG3/PRKC
TUBB4A/ADCY2/GABRG2/DLG2/GABBR2/SLC5A7/STXBP1/SYN1/RIMS1/AKAP5/LRRC7/ADCY1/

BRA5/DRD5/GABRA1/OPRM1/CHRNB3/SSTR4/GABRA6/GABRB2/MAS1/NPBWR2/MCHR2/NPB

2B/RASGRF1/CAMK2/

CUBE1/SFRP2/TNF/FGF16/WNT10B/WIF1/CBLN2/CCBE

KSR2/CAMK2B/FGF5/ARRB1/NRG3/KSR2/RBP4/RASAL1/DKK2/NEURL1/FGF16/CAMK2

LPAR3/HTR5A/RGS4/OPRM1/SSTR4/PRKCG/CAMK2A/NPBWR2/MCHR2/CNGB1/NPBWR
.B/GJD2/ABCC8/KCNC4/APBA1/KCNMA1/GLS/GABRB3/DLG4/PTPRD/NSF/IL1RAPL1/GRIA1/KCI

/12/HTR1B/CRH/RXFP1/NPFFR2/HCRTR2/VIP/WNT10B/CCKBR/HTR1E/LPAR3/HTR5A/DRD5/OPR
G4/IGF1/FGFR2/COL5A1/GFAP/MAPK13/ESRP1/RPS6KA5/PLG/GABRB1/TIAM1/AHCYL1/TNS3/

/PDE1B/GNG3/FSHB/DRD1/PDE1A/GPR83/CRHR2/GLP2R/GNG13/HRH2/CRH/RXFP1/VIP/DRD

[illegible]16/BBC3/YWHAQ/PRDX2/TAF10/SFN/PRKAB1/PRKAG1/RFC5/TACO1/CNOT11/CCNH/RAD9A/[illegible]

ST1H2BK/TUBA1A/H3F3A/NEK6/LIG1/H2AFZ/PRIM2/RBL1/POLA2/TUBB6/LIN9/TP53/CDC7/B9D
/CDC26/TUBG1/PSMC2/PSMA1/NUPL2/PMF1/CETN2/MIS12/PSMB4/PSMD13/NEDD1/HIST2H2
M1/TDP1/POLH/RPS27A/POLR2D/MSH2/MSH6/INO80
MRPL38/FARSB/EEF1G/RPL28/RPL38/EIF4A1/MRPS35/RPS4X/MRPS36/DARS2/PARS2/MRPS10/F

WR1

GABRA2/PRKACG/TUBA4A/CACNA2D3/HTR3A/GNG3/CAMKK1/GLS2/STX1A/SLC32A1/RAB3A/(

WR1

NK17/GLRA3/CACNB2/SLC1A6/SYN3/ALDH2/SLC1A2/PPFIA3/EPB41L1/PPFIA2/KCNA3/GRIN3A/

RM1/SSTR4/PROKR2/NPBWR2/MCHR2/NPBWR1
PTK2B/WASF1/FGF9/PAK3/FGF8/GABRB3/FGF3/DLG4/CXCL12/KIT/DNM3/WASF3/FLT3/CYFIP2/

5/OR52E8/OR14I1

TOP3A/TNFRSF10C/RBBP7/CNOT10/TP53RK/LAMTOR3/LAMTOR1/CCNG1/COX5A/RPA1/PRDX

[illegible]2/HIST1H2AC/HIST1H2BD/DCTN2/DHFR/MCM5/CEP152/POLD1/MCM3/NDC1/SKP2/NUP107/[illegible]
[illegible]

[illegible]RPL37A/RPL10/RPS8/EIF3G/MRPL9/DAP3/MRPL48/MRPS23/MRPL20/MRPS18C/FAU/SPCS1/MR[illegible]

GABRA4/GAD2/SYN2/SNAP25/KCNJ4/GNG13/RASGRF2/KCNJ3/PRKCB/SLC17A7/CHRNA2/GRIN

/CACNA2D2/GNAL/SLC6A13/ADCY5/BEGAIN/GABBR1/ERBB4/CPLX1/CACNB1/KCNJ6/KCNC1/[illegible]

/COL11A1/SPINT2/PTPRU/ERBB4/PTPN3/ATP6V1G2/ADAP1/ADCYAP1R1/DOCK3/FGF18/ADCY[illegible]

[illegible]1/STK11/TCEA1/TT[illegible]

POLD3/CENPO/HAUS7/NUP210/ORC5/PSME2/ANAPC11/HIST1H4H/CEP135/CDC25B/POLE4/C

PL24/MRPL40/WARS/RPL7A/RPL18/MRPS11/EIF3M/MRRF/RPL13A/EEF1B2/MRPL16/EIF3C/MRP

J1/CACNA1B/NRGN/KCNJ12/GABRG3/GRIN2B/RASGRF1/SYT1/GRIN2A/GABRA5/GABRA1/CHR

;RIP1/CAMKK2/PPFIA4/CACNA1E/ADCY8/CAMK2G/DLGAP1/SLC6A3/KCNN3/KCNK4/TUBA8/K[illegible]

AP1/SH3GL3/MEF2C/PCSK6/NTRK2/PAK1/PRKCZ/FGF5/GABRG2/FLRT2/PRKCE/NTF3/PGR/PRK[illegible]

:ENPP/HIST2H3A/PSMA7/PSMD8/NDE1/NUP35/BANF1/NUP85/FEN1

'L46/GSPT2/SEC11A/RPL8/

NB3/CACNG3/GABRA6/GABRB2/PRKCG/CAMK2A/HTR

CNC3/KCNA2/CAMK2B/EPB41L3/SLC18A3/PANX2/GRIN2C/KCNJ9/KCN

ACG/NRG3/SH3GL2/DNM1/MATK/ITPR1/PRKC
