# Supplementary material for: LncRNA PANTR1 is Associated with Poor Prognostic and Suppresses Apoptosis in Glioma
Source: J Oncol. 2023 Feb 20;2023:8537036. doi: 10.1155/2023/8537036 (PMC9970703; doi:10.1155/2023/8537036)
Supplement: Supplementary Materials — Table 1: Differential expression analysis of PANTR1 in GBM/LGG. Table 2: Gene ontology enrichment analysis of PANTR1 using the clusterProfiler package. Table 3: Pathway enrichment analysis of PANTR1. Table 4: Protein-protein interaction network of PANTR1. Table 5: The association of PANTR1 expression level with clinical parameters of gliomas using the Chi-squared test or Fisher's exact test for analysis. Student's t-test or Wilcoxon rank sum test revealed that age was significantly (p < 0.001) associated with PANTR1 expression. Table 6: The association of PANTR1 expression level with pathological parameters of gliomas using logistics regression. PANTR1 expression was significantly correlated with these variables including WHO grade (p < 0.001), IDH status (p < 0.001), primary therapy outcome (p = 0.016), and EGFR status (p < 0.001). Table 7: Uni- and multivariate Cox regression analysis showed the prognostic value of PANTR1 in overall survival. We observed IDH status (p < 0.001), primary therapy outcome (p < 0.001), age (p = 0.022), and PANTR1 (p = 0.045) are independent prognostic factors in progression-free interval (p < 0.05) of gliomas. Table 8: Uni- and multivariate Cox regression analysis showed the prognostic value of PANTR1 in progression-free survival. Table 9: Uni- and multivariate Cox regression analysis showed the prognostic value of PANTR1 in disease-specific survival. Supplement 10: Relative PANTR1 expression. PCR showed that all 15 glioma samples' PANTR1 expression outweighs normal adjacent tissues, whereas grade II and III glioma tend to have a higher expression rather than GBM compared with NAT. [file 8537036.f1.zip › Supplement table 5.docx]

**Table 5.** The association of PANTR1 expression level with clinical parameters of gliomas using Chi-squared test or Fisher’s exact test for analysis.

| Characters | level | Low expression of PANTR1 | High expression of PANTR1 | p | test |
| --- | --- | --- | --- | --- | --- |
| n |  | 335 | 335 |  |  |
| WHO grade (%) | G2 | 148(48.7%) | 68(22.0%) | <0.001 |  |
|  | G3 | 111(36.5%) | 126(40.8%) |  |  |
|  | G4 | 45(14.8%) | 115(37.2%) |  |  |
| IDH status (%) | Mut | 259(78.0%) | 165(50.2%) | <0.001 |  |
|  | WT | 73(22.0%) | 164(49.8%) |  |  |
| 1p/19q codeletion (%) | codel | 89(26.6%) | 79(23.9%) | 0.476 |  |
|  | non-codel | 245(73.4%) | 251(76.1%) |  |  |
| Primary therapy outcome (%) | CR | 90(34.9%) | 45(24.2%) | 0.020 |  |
|  | PD | 48(18.6%) | 55(29.6%) |  |  |
|  | PR | 35(13.6%) | 27(14.5%) |  |  |
|  | SD | 85(32.9%) | 59(31.7%) |  |  |
| Gender (%) | Female | 127(37.9%) | 157(46.9%) | 0.023 |  |
|  | Male | 208(62.1%) | 178(53.1%) |  |  |
| Race (%) | Asian | 6(1.8%) | 7(2.1%) | 0.751 |  |
|  | Black or African American | 18(5.5%) | 14(4.3%) |  |  |
|  | White | 306(92.7%) | 307(93.6%) |  |  |
| Histological type (%) | Astrocytoma | 107(31.9%) | 85(25.4%) | <0.001 |  |
|  | Glioblastoma | 45(13.4%) | 115(34.3%) |  |  |
|  | Oligoastrocytoma | 78(23.3%) | 50(14.9%) |  |  |
|  | Oligodendroglioma | 105(31.3%) | 85(25.4%) |  |  |
| EGFR status (%) | Mut | 16(4.9%) | 57(17.2%) | <0.001 |  |
|  | WT | 309(95.1%) | 274(82.8%) |  |  |
| PIK3CA status (%) | Mut | 26(8.0%) | 23(6.9%) | 0.716 |  |
|  | WT | 299(92.0%) | 308(93.1%) |  |  |
| Age (median [IQR]) |  | 39.00[31.00,52.00] | 52.00[40.50,62.00] | <0.001 | nonnorm |
