# Supplementary material for: LncRNA PANTR1 is Associated with Poor Prognostic and Suppresses Apoptosis in Glioma
Source: J Oncol. 2023 Feb 20;2023:8537036. doi: 10.1155/2023/8537036 (PMC9970703; doi:10.1155/2023/8537036)
Supplement: Supplementary Materials — Table 1: Differential expression analysis of PANTR1 in GBM/LGG. Table 2: Gene ontology enrichment analysis of PANTR1 using the clusterProfiler package. Table 3: Pathway enrichment analysis of PANTR1. Table 4: Protein-protein interaction network of PANTR1. Table 5: The association of PANTR1 expression level with clinical parameters of gliomas using the Chi-squared test or Fisher's exact test for analysis. Student's t-test or Wilcoxon rank sum test revealed that age was significantly (p < 0.001) associated with PANTR1 expression. Table 6: The association of PANTR1 expression level with pathological parameters of gliomas using logistics regression. PANTR1 expression was significantly correlated with these variables including WHO grade (p < 0.001), IDH status (p < 0.001), primary therapy outcome (p = 0.016), and EGFR status (p < 0.001). Table 7: Uni- and multivariate Cox regression analysis showed the prognostic value of PANTR1 in overall survival. We observed IDH status (p < 0.001), primary therapy outcome (p < 0.001), age (p = 0.022), and PANTR1 (p = 0.045) are independent prognostic factors in progression-free interval (p < 0.05) of gliomas. Table 8: Uni- and multivariate Cox regression analysis showed the prognostic value of PANTR1 in progression-free survival. Table 9: Uni- and multivariate Cox regression analysis showed the prognostic value of PANTR1 in disease-specific survival. Supplement 10: Relative PANTR1 expression. PCR showed that all 15 glioma samples' PANTR1 expression outweighs normal adjacent tissues, whereas grade II and III glioma tend to have a higher expression rather than GBM compared with NAT. [file 8537036.f1.zip › Supplement table4.pdf]

node1	node2	node1_ext	node2_ext	neighborh	gene_fusic	phylogene	coexpressi	experimer
GABRA1	GABRB2	ENSP0000	ENSP0000	0	0	0	0.348	0.809
GABRA5	GABRB2	ENSP0000	ENSP0000	0	0	0	0.231	0.743
GRIN2B	CAMK2A	ENSP0000	ENSP0000	0	0	0	0.231	0.521
HOXA7	HOXA6	ENSP0000	ENSP0000	0	0	0	0.39	0.96
HOXA6	HOXA5	ENSP0000	ENSP0000	0	0	0	0.363	0.96
HOXA7	HOXA5	ENSP0000	ENSP0000	0	0	0	0.356	0.96
GABRB2	GABRA6	ENSP0000	ENSP0000	0	0	0	0.458	0.544
CAMK2A	GRIN2A	ENSP0000	ENSP0000	0	0	0	0.243	0.454
HOXB4	HOXA5	ENSP0000	ENSP0000	0	0	0	0.238	0.96
GRIN2B	RASGRF1	ENSP0000	ENSP0000	0	0	0	0.129	0
HOXB4	HOXA7	ENSP0000	ENSP0000	0	0	0	0.143	0.96
HOXB4	HOXA6	ENSP0000	ENSP0000	0	0	0	0.12	0.96
KISS1	PROKR2	ENSP0000	ENSP0000	0	0	0	0	0.064
GRIN2B	RELN	ENSP0000	ENSP0000	0	0	0	0.095	0
RASGRF1	GRIN2A	ENSP0000	ENSP0000	0	0	0	0.173	0
RELN	GRIN2A	ENSP0000	ENSP0000	0	0	0	0.116	0
GABRA1	GABRA6	ENSP0000	ENSP0000	0	0	0	0.476	0.181
CAMK2A	CACNG3	ENSP0000	ENSP0000	0	0	0	0.535	0
HTR1E	HTR5A	ENSP0000	ENSP0000	0	0	0	0.395	0
GABRG3	GABRB2	ENSP0000	ENSP0000	0	0	0	0.088	0.333
GABRA1	GABRA5	ENSP0000	ENSP0000	0	0	0	0.231	0.26
GRIN2B	GRIN2A	ENSP0000	ENSP0000	0	0	0	0.175	0.268
PACSIN1	SYT1	ENSP0000	ENSP0000	0	0	0	0.23	0
HTR5A	SSTR4	ENSP0000	ENSP0000	0	0	0	0.352	0
GABRA5	GABRA6	ENSP0000	ENSP0000	0	0	0	0.183	0.181
RASGRF1	CAMK2A	ENSP0000	ENSP0000	0	0	0	0.242	0
LPAR3	EGFR	ENSP0000	ENSP0000	0	0	0	0	0.084
NPBWR2	MCHR2	ENSP0000	ENSP0000	0	0	0	0.108	0
HTR1E	SSTR4	ENSP0000	ENSP0000	0	0	0	0.223	0
NPBWR2	SSTR4	ENSP0000	ENSP0000	0	0	0	0.162	0
NPBWR2	HTR1E	ENSP0000	ENSP0000	0	0	0	0.161	0
EGFR	SYT1	ENSP0000	ENSP0000	0	0	0	0.061	0
NPBWR2	HTR5A	ENSP0000	ENSP0000	0	0	0	0.214	0
HTR1E	MCHR2	ENSP0000	ENSP0000	0	0	0	0.111	0
LPAR3	EDN2	ENSP0000	ENSP0000	0	0	0	0	0.157
OPRM1	HTR5A	ENSP0000	ENSP0000	0	0	0	0.061	0
EDN2	PROKR2	ENSP0000	ENSP0000	0	0	0	0	0.157
PACSIN1	EGFR	ENSP0000	ENSP0000	0	0	0	0.051	0
MCHR2	SSTR4	ENSP0000	ENSP0000	0	0	0	0.116	0
HTR5A	MCHR2	ENSP0000	ENSP0000	0	0	0	0.184	0
NPBWR1	MCHR2	ENSP0000	ENSP0000	0	0	0	0.063	0
GABRG3	GABRA5	ENSP0000	ENSP0000	0	0	0	0.088	0
GABRG3	GABRA1	ENSP0000	ENSP0000	0	0	0	0.109	0
EDN2	MCHR2	ENSP0000	ENSP0000	0	0	0	0	0.157
OPRM1	HTR1E	ENSP0000	ENSP0000	0	0	0	0	0
NPBWR1	SSTR4	ENSP0000	ENSP0000	0	0	0	0.052	0
KISS1	MCHR2	ENSP0000	ENSP0000	0	0	0	0.062	0.064
CHRNA9	CHRNA1	ENSP0000	ENSP0000	0	0	0	0	0
CHRNA9	CHRNB3	ENSP0000	ENSP0000	0	0	0	0	0
NPBWR1	HTR1E	ENSP0000	ENSP0000	0	0	0	0.062	0
GABRG3	GABRA6	ENSP0000	ENSP0000	0	0	0	0.089	0
MCHR2	PROKR2	ENSP0000	ENSP0000	0	0	0	0	0
OPRM1	SSTR4	ENSP0000	ENSP0000	0	0	0	0	0
OPRM1	NPBWR1	ENSP0000	ENSP0000	0	0	0	0	0
OPRM1	NPBWR2	ENSP0000	ENSP0000	0	0	0	0	0
LPAR3	OPRM1	ENSP0000	ENSP0000	0	0	0	0	0.077
CHRNB3	CHRNA1	ENSP0000	ENSP0000	0	0	0	0	0
NPBWR1	HTR5A	ENSP0000	ENSP0000	0	0	0	0	0

NPBWR2	NPBWR1	ENSP0000	ENSP0000	0	0	0	0	0
OPRM1	MCHR2	ENSP0000	ENSP0000	0	0	0	0	0
LPAR3	SSTR4	ENSP0000	ENSP0000	0	0	0	0	0
LPAR3	KISS1	ENSP0000	ENSP0000	0	0	0	0	0.064
LPAR3	NPBWR2	ENSP0000	ENSP0000	0	0	0	0	0
KRT33B	KRT31	ENSP0000	ENSP0000	0	0	0	0	0
LPAR3	NPBWR1	ENSP0000	ENSP0000	0	0	0	0	0
EDN2	KISS1	ENSP0000	ENSP0000	0	0	0	0	0
LPAR3	PROKR2	ENSP0000	ENSP0000	0	0	0	0	0
LPAR3	MCHR2	ENSP0000	ENSP0000	0	0	0	0	0
LPAR3	HTR5A	ENSP0000	ENSP0000	0	0	0	0	0
LPAR3	HTR1E	ENSP0000	ENSP0000	0	0	0	0	0
SOCS2	FBXO40	ENSP0000	ENSP0000	0	0	0	0	0
H2BFS	HOXA2	ENSP0000	ENSP0000	0	0	0	0	0
H2BFS	HOXB4	ENSP0000	ENSP0000	0	0	0	0	0
H2BFS	HOXB2	ENSP0000	ENSP0000	0	0	0	0	0
H2BFS	HOXA4	ENSP0000	ENSP0000	0	0	0	0	0
H2BFS	HOXB3	ENSP0000	ENSP0000	0	0	0	0	0
HOXA3	H2BFS	ENSP0000	ENSP0000	0	0	0	0	0
HOXB3	HOXB4	ENSP0000	ENSP0000	0	0	0	0.475	0.751
CAMK2A	RYR2	ENSP0000	ENSP0000	0	0	0	0.159	0.097
SV2B	SYT1	ENSP0000	ENSP0000	0	0	0	0.194	0.404
HOXA3	HOXA5	ENSP0000	ENSP0000	0	0	0	0.414	0.746
HOXB3	HOXA5	ENSP0000	ENSP0000	0	0	0	0.169	0.809
HOXA9	HOXA10	ENSP0000	ENSP0000	0	0	0	0.821	0
HOXB3	HOXA6	ENSP0000	ENSP0000	0	0	0	0.143	0.809
HOXA3	HOXA6	ENSP0000	ENSP0000	0	0	0	0.35	0.736
H2BFS	HIST2H2A	ENSP0000	ENSP0000	0	0	0	0.257	0.715
OTX2	SIX6	ENSP0000	ENSP0000	0	0	0	0.108	0.179
HOXB4	HOXB8	ENSP0000	ENSP0000	0	0	0	0.302	0.731
HOXD11	HOXD10	ENSP0000	ENSP0000	0	0	0	0.764	0
RELN	TBR1	ENSP0000	ENSP0000	0	0	0	0.092	0
GRIN2B	CACNG3	ENSP0000	ENSP0000	0	0	0	0.231	0.162
HOXA3	HOXB4	ENSP0000	ENSP0000	0	0	0	0.213	0.732
GRIN2A	CACNG3	ENSP0000	ENSP0000	0	0	0	0.23	0.162
TBR1	EMX1	ENSP0000	ENSP0000	0	0	0	0.123	0.097
HOXB8	HOXA5	ENSP0000	ENSP0000	0	0	0	0.19	0.731
FGF19	EGFR	ENSP0000	ENSP0000	0	0	0	0	0
HOXA7	HOXB8	ENSP0000	ENSP0000	0	0	0	0.174	0.731
HOXB3	HOXA7	ENSP0000	ENSP0000	0	0	0	0.156	0.731
MPPED1	SULT4A1	ENSP0000	ENSP0000	0	0	0	0.359	0
HOXB8	HOXA6	ENSP0000	ENSP0000	0	0	0	0.149	0.731
UGT2B17	DRD5	ENSP0000	ENSP0000	0	0	0	0	0
HTR1E	HTR3B	ENSP0000	ENSP0000	0	0	0	0	0.047
SP8	GSX2	ENSP0000	ENSP0000	0	0	0	0.061	0.059
HTR5A	HTR3B	ENSP0000	ENSP0000	0	0	0	0	0.047
GRIN2B	TBR1	ENSP0000	ENSP0000	0	0	0	0.173	0
SULT4A1	GABRA6	ENSP0000	ENSP0000	0	0	0	0.334	0
RBFOX3	TBR1	ENSP0000	ENSP0000	0	0	0	0.23	0
HOXA9	HOXA5	ENSP0000	ENSP0000	0	0	0	0.54	0.185
TBR1	GSX2	ENSP0000	ENSP0000	0	0	0	0	0.193
HIST2H2A	HIST1H1D	ENSP0000	ENSP0000	0	0.001	0	0.104	0.282
TBR1	NEUROD6	ENSP0000	ENSP0000	0	0	0	0.353	0.05
KLK7	KLK5	ENSP0000	ENSP0000	0	0	0.03382	0.663	0
FOXB1	OTP	ENSP0000	ENSP0000	0	0	0	0.064	0.106
RASGRF1	CACNG3	ENSP0000	ENSP0000	0	0	0	0.232	0
HOXA9	HOXA7	ENSP0000	ENSP0000	0	0	0	0.51	0.185
C1QL3	NEUROD6	ENSP0000	ENSP0000	0	0	0	0.146	0
HOXA3	HOXA7	ENSP0000	ENSP0000	0	0	0	0.356	0.414

CAMK2A	KCNH5	ENSP0000	ENSP0000	0	0	0	0.099	0.587
GABRA1	SLC12A5	ENSP0000	ENSP0000	0	0	0	0.345	0.065
GRIN2B	DRD5	ENSP0000	ENSP0000	0	0	0	0.118	0.157
HTR5A	GABRB2	ENSP0000	ENSP0000	0	0	0	0.551	0
GRIN2B	HTR3B	ENSP0000	ENSP0000	0	0	0	0	0.124
FOXB1	NPBWR1	ENSP0000	ENSP0000	0	0	0	0.643	0.052
GABRA1	SYT1	ENSP0000	ENSP0000	0	0	0	0.348	0
GRIN2A	GABRB2	ENSP0000	ENSP0000	0	0	0	0.26	0
HOXC11	HOXC10	ENSP0000	ENSP0000	0	0	0	0.532	0
CAMK2A	NEFM	ENSP0000	ENSP0000	0	0	0	0.293	0
HOXC11	HOXC13	ENSP0000	ENSP0000	0	0	0	0.524	0
KISS1	CARTPT	ENSP0000	ENSP0000	0	0	0	0	0
HOXA5	HOXA2	ENSP0000	ENSP0000	0	0	0	0.231	0.414
EGFR	IGFBP2	ENSP0000	ENSP0000	0	0	0	0	0.379
GRIN2A	HTR3B	ENSP0000	ENSP0000	0	0	0	0	0.124
HOXA4	HOXA5	ENSP0000	ENSP0000	0	0	0	0.569	0
EGFR	SOX4	ENSP0000	ENSP0000	0	0	0	0.05	0.108
GRIN2B	RBFOX3	ENSP0000	ENSP0000	0	0	0	0.198	0
DDN	CAMK2A	ENSP0000	ENSP0000	0	0	0	0.56	0
GABRA1	GRIN2A	ENSP0000	ENSP0000	0	0	0	0.232	0
TBR1	OTX1	ENSP0000	ENSP0000	0	0	0	0.062	0.051
PRKCG	CREB3L3	ENSP0000	ENSP0000	0	0	0	0.062	0
OTX2	HOXB4	ENSP0000	ENSP0000	0	0	0	0.052	0
CAMK2A	CREB3L3	ENSP0000	ENSP0000	0	0	0	0	0
RASGRF1	ANKRD34	ENSP0000	ENSP0000	0	0	0	0.201	0
TBR1	OTP	ENSP0000	ENSP0000	0	0	0	0.062	0.157
VAV3	EGFR	ENSP0000	ENSP0000	0	0	0	0	0.419
GRIN2A	DRD5	ENSP0000	ENSP0000	0	0	0	0.122	0.157
GRIN2B	PRKCG	ENSP0000	ENSP0000	0	0	0	0.164	0.336
SCNN1B	CALHM1	ENSP0000	ENSP0000	0	0	0	0.061	0
GRIN2B	GABRB2	ENSP0000	ENSP0000	0	0	0	0.232	0
HOXA3	HOXA10	ENSP0000	ENSP0000	0	0	0	0.352	0.185
HOXD13	HOXD11	ENSP0000	ENSP0000	0	0	0	0.377	0
RBFOX1	NEUROD6	ENSP0000	ENSP0000	0	0	0	0.485	0
TBR1	OTX2	ENSP0000	ENSP0000	0	0	0	0.061	0.051
GRIN2B	GABRA1	ENSP0000	ENSP0000	0	0	0	0.231	0
OLFM3	CACNG3	ENSP0000	ENSP0000	0	0	0	0.373	0
RBFOX3	RELN	ENSP0000	ENSP0000	0	0	0	0.095	0
NEUROD6	GABRA6	ENSP0000	ENSP0000	0	0	0	0.376	0
HTR5A	SLC6A7	ENSP0000	ENSP0000	0	0	0	0.557	0.073
SOCS2	EGFR	ENSP0000	ENSP0000	0	0	0	0	0.064
OLFM3	GABRB2	ENSP0000	ENSP0000	0	0	0	0.374	0
HOXA3	HOXA4	ENSP0000	ENSP0000	0	0	0	0.457	0
HOXA10	HOXA7	ENSP0000	ENSP0000	0	0	0	0.367	0.185
HOXB2	HOXA5	ENSP0000	ENSP0000	0	0	0	0.099	0.414
SLC12A5	GABRB2	ENSP0000	ENSP0000	0	0	0	0.315	0.171
GABRA1	CACNG3	ENSP0000	ENSP0000	0	0	0	0.431	0
HOXC10	HOXC13	ENSP0000	ENSP0000	0	0	0	0.423	0
OLFM3	NEUROD6	ENSP0000	ENSP0000	0	0	0	0.377	0
GABRA1	PRKCG	ENSP0000	ENSP0000	0	0	0	0.309	0.379
KRT31	HOXC13	ENSP0000	ENSP0000	0	0	0	0	0
HOXA4	HOXA10	ENSP0000	ENSP0000	0	0	0	0.332	0.185
EGFR	HOXA7	ENSP0000	ENSP0000	0	0	0	0.06	0.086
HOXA10	HOXA5	ENSP0000	ENSP0000	0	0	0	0.332	0.185
HOXA10	HOXA6	ENSP0000	ENSP0000	0	0	0	0.375	0.185
CAMK2A	RBFOX1	ENSP0000	ENSP0000	0	0	0	0.467	0
HOXB2	HOXB4	ENSP0000	ENSP0000	0	0	0	0.352	0.175
HOXD13	HOXD10	ENSP0000	ENSP0000	0	0	0	0.352	0
HOXA4	HOXA9	ENSP0000	ENSP0000	0	0	0	0.331	0.185

HTR5A	GABRA6	ENSP0000	ENSP0000	0	0	0	0.556	0
HOXB3	HOXB2	ENSP0000	ENSP0000	0	0	0	0.425	0
RYR2	NKX2-5	ENSP0000	ENSP0000	0	0	0	0.064	0
RBFOX3	CAMK2A	ENSP0000	ENSP0000	0	0	0	0.284	0
HOXA3	HOXA9	ENSP0000	ENSP0000	0	0	0	0.35	0.185
HOXA9	HOXA6	ENSP0000	ENSP0000	0	0	0	0.353	0.185
SEC61G	14-Sep	ENSP0000	ENSP0000	0	0	0	0	0
DDN	CACNG3	ENSP0000	ENSP0000	0	0	0	0.55	0
CAMK2A	HTR5A	ENSP0000	ENSP0000	0	0	0	0.507	0.074
GRIN2B	SYT1	ENSP0000	ENSP0000	0	0	0	0.23	0.159
NEUROD6	HTR5A	ENSP0000	ENSP0000	0	0	0	0.532	0.065
RBFOX1	OTP	ENSP0000	ENSP0000	0	0	0	0	0
GABRG3	DLGAP2	ENSP0000	ENSP0000	0	0	0	0.087	0
HTR5A	CACNG3	ENSP0000	ENSP0000	0	0	0	0.543	0
FEZF1	OTX2	ENSP0000	ENSP0000	0	0	0	0.171	0.064
RBFOX3	GRIN2A	ENSP0000	ENSP0000	0	0	0	0.159	0
HTR3B	CACNG3	ENSP0000	ENSP0000	0	0	0	0	0
NEUROD6	EMX1	ENSP0000	ENSP0000	0	0	0	0.229	0.05
RBFOX3	NEFM	ENSP0000	ENSP0000	0	0	0	0.195	0
SLC12A5	GRIN2A	ENSP0000	ENSP0000	0	0	0	0.172	0
GABRA1	HTR5A	ENSP0000	ENSP0000	0	0	0	0.532	0
CTXN3	NEUROD6	ENSP0000	ENSP0000	0	0	0	0.372	0
HTR5A	GPR26	ENSP0000	ENSP0000	0	0	0	0.298	0
GABRA6	SLC6A7	ENSP0000	ENSP0000	0	0	0	0.522	0
CTXN3	HTR5A	ENSP0000	ENSP0000	0	0	0	0.533	0
FEZF1	OTP	ENSP0000	ENSP0000	0	0	0	0.145	0.077
HOXA9	SOX4	ENSP0000	ENSP0000	0	0	0	0	0.078
GRIN2B	OPRM1	ENSP0000	ENSP0000	0	0	0	0.061	0.064
GABRA1	NEUROD6	ENSP0000	ENSP0000	0	0	0	0.379	0
RBFOX1	HTR5A	ENSP0000	ENSP0000	0	0	0	0.528	0
OLFM3	MAB21L2	ENSP0000	ENSP0000	0	0	0	0	0
OTX2	HOXA5	ENSP0000	ENSP0000	0	0	0	0	0
HOXA3	HOXA2	ENSP0000	ENSP0000	0	0	0	0.372	0
SIX6	OTP	ENSP0000	ENSP0000	0	0	0	0.064	0
RBFOX3	SLC12A5	ENSP0000	ENSP0000	0	0	0	0.23	0
GRIN2B	SLC12A5	ENSP0000	ENSP0000	0	0	0	0.171	0
RELN	EMX1	ENSP0000	ENSP0000	0	0	0	0.065	0
CLRN1	CNGB1	ENSP0000	ENSP0000	0	0	0	0	0
SLC12A5	SYT1	ENSP0000	ENSP0000	0	0	0	0.231	0
RGS4	OPRM1	ENSP0000	ENSP0000	0	0	0	0	0.05
RBFOX3	GABRA6	ENSP0000	ENSP0000	0	0	0	0.333	0
HOXC12	HOXC13	ENSP0000	ENSP0000	0	0	0	0.346	0
OPRM1	HTR3B	ENSP0000	ENSP0000	0	0	0	0.065	0.047
OTX2	GDF3	ENSP0000	ENSP0000	0	0	0	0.142	0
CALHM1	HTR5A	ENSP0000	ENSP0000	0	0	0	0.511	0
VSNL1	CAMK2A	ENSP0000	ENSP0000	0	0	0	0.291	0.261
HOXD13	SHOX2	ENSP0000	ENSP0000	0	0	0	0.076	0.083
MPPED1	HTR5A	ENSP0000	ENSP0000	0	0	0	0.51	0
RASGRF1	RBFOX1	ENSP0000	ENSP0000	0	0	0	0.201	0.185
CARTPT	HTR5A	ENSP0000	ENSP0000	0	0	0	0.508	0
GABRA6	CACNG3	ENSP0000	ENSP0000	0	0	0	0.442	0
OLFM3	SULT4A1	ENSP0000	ENSP0000	0	0	0	0.347	0
GRIN2A	RBFOX1	ENSP0000	ENSP0000	0	0	0	0.339	0
GRIN2A	GABRA5	ENSP0000	ENSP0000	0	0	0	0.2	0
CARTPT	EGFR	ENSP0000	ENSP0000	0	0	0	0	0
OLFM3	HTR5A	ENSP0000	ENSP0000	0	0	0	0.503	0
SLC12A5	GABRA6	ENSP0000	ENSP0000	0	0	0	0.341	0.065
TBR1	HOXC10	ENSP0000	ENSP0000	0	0	0	0	0.098
PACSIN1	GRIN2B	ENSP0000	ENSP0000	0	0	0	0.143	0

RBFOX1	GABRA6	ENSP0000	ENSP0000	0	0	0	0.394	0
HOXA4	HOXA2	ENSP0000	ENSP0000	0	0	0	0.341	0
FEZF1	TBR1	ENSP0000	ENSP0000	0	0	0	0.063	0.063
CAMK2A	EGFR	ENSP0000	ENSP0000	0	0	0	0.061	0.379
DLGAP2	GRIN2A	ENSP0000	ENSP0000	0	0	0	0.202	0.064
SLC6A7	CACNG3	ENSP0000	ENSP0000	0	0	0	0.493	0
KRT33B	HOXC13	ENSP0000	ENSP0000	0	0	0	0	0
MPPED1	NEUROD6	ENSP0000	ENSP0000	0	0	0	0.489	0
FEZF1	PROKR2	ENSP0000	ENSP0000	0	0	0	0	0.05
CAMK2A	SV2B	ENSP0000	ENSP0000	0	0	0	0.348	0
DRD5	HTR5A	ENSP0000	ENSP0000	0	0	0	0.435	0
HOXB4	SOX4	ENSP0000	ENSP0000	0	0	0	0	0.08
GRIN2A	OLFM3	ENSP0000	ENSP0000	0	0	0	0.231	0
GRIN2B	OLFM3	ENSP0000	ENSP0000	0	0	0	0.23	0
GRIN2A	SYT1	ENSP0000	ENSP0000	0	0	0	0.191	0.086
NEUROD6	CACNG3	ENSP0000	ENSP0000	0	0	0	0.423	0
CAMK2A	GABRB2	ENSP0000	ENSP0000	0	0	0	0.365	0
RBFOX3	NEUROD6	ENSP0000	ENSP0000	0	0	0	0.306	0
TBR1	NKX2-5	ENSP0000	ENSP0000	0	0	0	0	0.33
HOXC11	HOXC12	ENSP0000	ENSP0000	0	0	0	0.308	0
FRMPD4	SULT4A1	ENSP0000	ENSP0000	0	0	0	0.342	0
RASGRF1	EGFR	ENSP0000	ENSP0000	0	0	0	0	0.416
FOXB1	OTX2	ENSP0000	ENSP0000	0	0	0	0.077	0
CAMK2A	TBR1	ENSP0000	ENSP0000	0	0	0	0.227	0
GRIN2B	DLGAP2	ENSP0000	ENSP0000	0	0	0	0.178	0.064
MAGEA3	CARTPT	ENSP0000	ENSP0000	0	0	0	0	0
FRMPD4	NEUROD6	ENSP0000	ENSP0000	0	0	0	0.326	0
VSNL1	SYT1	ENSP0000	ENSP0000	0	0	0	0.332	0
GRIN2A	HTR5A	ENSP0000	ENSP0000	0	0	0	0.347	0.064
GABRA1	CAMK2A	ENSP0000	ENSP0000	0	0	0	0.371	0
DLGAP2	RBFOX1	ENSP0000	ENSP0000	0	0	0	0.345	0
FGF19	SLC30A3	ENSP0000	ENSP0000	0	0	0	0	0
CAMK2A	SYT1	ENSP0000	ENSP0000	0	0	0	0.297	0
SV2B	GABRB2	ENSP0000	ENSP0000	0	0	0	0.323	0
SLC12A5	SLC30A3	ENSP0000	ENSP0000	0	0	0	0.106	0
SLC12A5	SV2B	ENSP0000	ENSP0000	0	0	0	0.231	0.131
DRD5	GABRB2	ENSP0000	ENSP0000	0	0	0	0.235	0
RGS4	EGFR	ENSP0000	ENSP0000	0	0	0	0.075	0.393
RGS4	DRD5	ENSP0000	ENSP0000	0	0	0	0.096	0.05
OLFM3	HTR3B	ENSP0000	ENSP0000	0	0	0	0	0
RBFOX3	SP8	ENSP0000	ENSP0000	0	0	0	0.062	0.06
HOXA4	HOXA7	ENSP0000	ENSP0000	0	0	0	0.378	0
TBR1	HOXB4	ENSP0000	ENSP0000	0	0	0	0	0.193
GRIN2B	HTR5A	ENSP0000	ENSP0000	0	0	0	0.329	0.064
RBFOX1	GABRB2	ENSP0000	ENSP0000	0	0	0	0.442	0
SP8	TBR1	ENSP0000	ENSP0000	0	0	0	0.062	0.063
SYT1	NEFM	ENSP0000	ENSP0000	0	0	0	0.365	0.111
FOXB1	UNCX	ENSP0000	ENSP0000	0	0	0	0.061	0.106
SP8	EMX1	ENSP0000	ENSP0000	0	0	0	0.061	0.064
HOXD12	HOXD13	ENSP0000	ENSP0000	0	0	0	0.232	0
HOXC11	TBR1	ENSP0000	ENSP0000	0	0	0	0	0.098
EGFR	CHD5	ENSP0000	ENSP0000	0	0	0	0	0.417
OPRM1	PRKCG	ENSP0000	ENSP0000	0	0	0	0	0.056
RBFOX3	EMX1	ENSP0000	ENSP0000	0	0	0	0.109	0
RBFOX3	GABRA1	ENSP0000	ENSP0000	0	0	0	0.35	0
SIX6	MAB21L2	ENSP0000	ENSP0000	0	0	0	0.054	0
HOXA9	HOXA2	ENSP0000	ENSP0000	0	0	0	0.2	0.185
OPRM1	NOS2	ENSP0000	ENSP0000	0	0	0	0	0
SLC12A5	NEUROD6	ENSP0000	ENSP0000	0	0	0	0.345	0

DLGAP2	MPPED1	ENSP0000	ENSP0000	0	0	0	0.348	0
SHOX2	HOXA5	ENSP0000	ENSP0000	0	0	0	0	0.081
PRKCG	CACNG3	ENSP0000	ENSP0000	0	0	0	0.345	0
SIX6	PITX2	ENSP0000	ENSP0000	0	0	0	0.063	0
RELN	GSX2	ENSP0000	ENSP0000	0	0	0	0	0
HOXA10	HOXA2	ENSP0000	ENSP0000	0	0	0	0.148	0.185
DDN	GDA	ENSP0000	ENSP0000	0	0	0	0.088	0
HOXB3	HOXB8	ENSP0000	ENSP0000	0	0	0	0.313	0
OTX2	OTP	ENSP0000	ENSP0000	0	0	0	0.064	0.262
OPRM1	GRIN2A	ENSP0000	ENSP0000	0	0	0	0.061	0.064
CAMK2A	SLC6A7	ENSP0000	ENSP0000	0	0	0	0.429	0
GABRB2	SYT1	ENSP0000	ENSP0000	0	0	0	0.308	0
NEUROD6	SLC6A7	ENSP0000	ENSP0000	0	0	0	0.421	0
OTX2	PROKR2	ENSP0000	ENSP0000	0	0	0	0.065	0
GABRA1	OLFM3	ENSP0000	ENSP0000	0	0	0	0.369	0
RGS4	CAMK2A	ENSP0000	ENSP0000	0	0	0	0.191	0
GABRA6	KRT31	ENSP0000	ENSP0000	0	0	0	0.427	0
OTX2	MAB21L2	ENSP0000	ENSP0000	0	0	0	0.062	0
SLC12A5	HTR5A	ENSP0000	ENSP0000	0	0	0	0.422	0.056
DLGAP2	CAMK2A	ENSP0000	ENSP0000	0	0	0	0.27	0.106
RGS4	GABRB2	ENSP0000	ENSP0000	0	0	0	0.156	0
SLC12A5	CAMK2A	ENSP0000	ENSP0000	0	0	0	0.345	0
FEZF1	OTX1	ENSP0000	ENSP0000	0	0	0	0.064	0.064
HOXA9	HOXB8	ENSP0000	ENSP0000	0	0	0	0.154	0.185
SIX6	OTX1	ENSP0000	ENSP0000	0	0	0	0.062	0.082
SV2B	RBFOX1	ENSP0000	ENSP0000	0	0	0	0.347	0
HOXB3	HOXA10	ENSP0000	ENSP0000	0	0	0	0.155	0.185
OLFM3	RBFOX1	ENSP0000	ENSP0000	0	0	0	0.411	0
DDN	FRMPD4	ENSP0000	ENSP0000	0	0	0	0.23	0
CAMK2A	GABRA6	ENSP0000	ENSP0000	0	0	0	0.377	0
HOXA9	HOXB4	ENSP0000	ENSP0000	0	0	0	0.131	0.185
GABRA1	RBFOX1	ENSP0000	ENSP0000	0	0	0	0.381	0
GABRA1	SV2B	ENSP0000	ENSP0000	0	0	0	0.351	0
H2BFS	C1orf189	ENSP0000	ENSP0000	0	0	0	0	0
OTX2	FGF19	ENSP0000	ENSP0000	0	0	0	0.107	0.074
CARTPT	SLC6A7	ENSP0000	ENSP0000	0	0	0	0.418	0
GABRA1	VSNL1	ENSP0000	ENSP0000	0	0	0	0.348	0
RBFOX3	CARTPT	ENSP0000	ENSP0000	0	0	0	0.224	0
HOXD12	HOXD11	ENSP0000	ENSP0000	0	0	0	0.202	0
SULT4A1	RBFOX1	ENSP0000	ENSP0000	0	0	0	0.354	0
OR14I1	HTR5A	ENSP0000	ENSP0000	0	0	0	0.417	0
CTXN3	CACNG3	ENSP0000	ENSP0000	0	0	0	0.416	0
GRIN2B	GABRA5	ENSP0000	ENSP0000	0	0	0	0.191	0
GABRB2	SLC6A7	ENSP0000	ENSP0000	0	0	0	0.414	0
HOXB3	HOXA9	ENSP0000	ENSP0000	0	0	0	0.159	0.185
DDN	CTXN3	ENSP0000	ENSP0000	0	0	0	0.352	0
EGFR	WNT16	ENSP0000	ENSP0000	0	0	0	0.061	0.195
SIX6	EMX1	ENSP0000	ENSP0000	0	0	0	0.054	0.129
HOXB4	HOXA2	ENSP0000	ENSP0000	0	0	0	0.13	0.175
GABRA6	SYT1	ENSP0000	ENSP0000	0	0	0	0.312	0
GRIN2A	PRKCG	ENSP0000	ENSP0000	0	0	0	0.17	0.053
PACSIN1	SLC12A5	ENSP0000	ENSP0000	0	0	0	0.292	0
SYT1	C6orf15	ENSP0000	ENSP0000	0	0	0	0	0.294
RBFOX1	SLC6A7	ENSP0000	ENSP0000	0	0	0	0.408	0
PACSIN1	SULT4A1	ENSP0000	ENSP0000	0	0	0	0.313	0
VSNL1	NEFM	ENSP0000	ENSP0000	0	0	0	0.301	0
GRIN2B	RBFOX1	ENSP0000	ENSP0000	0	0	0	0.295	0
GABRA1	SLC6A7	ENSP0000	ENSP0000	0	0	0	0.406	0
RBFOX3	SLC30A3	ENSP0000	ENSP0000	0	0	0	0.117	0

SEC61G	EGFR	ENSP0000	ENSP0000	0	0	0	0.152	0
HOXA4	HOXA6	ENSP0000	ENSP0000	0	0	0	0.315	0
GABRA1	SULT4A1	ENSP0000	ENSP0000	0	0	0	0.342	0
PACSIN1	MPPED1	ENSP0000	ENSP0000	0	0	0	0.349	0
MPPED1	CACNG3	ENSP0000	ENSP0000	0	0	0	0.401	0
HOXB3	HOXD10	ENSP0000	ENSP0000	0	0	0	0.089	0.185

database_	automate(	combined_score
0.9	0.128426	0.988
0.9	0.117401	0.98
0.9	0.527	0.98
0	0.13532	0.978
0	0.122766	0.976
0	0.123384	0.976
0.9	0.131544	0.976
0.9	0.479	0.975
0	0.122772	0.972
0.9	0.7	0.971
0	0.114304	0.968
0	0.111926	0.967
0.9	0.633	0.962
0.9	0.578	0.958
0.9	0.501	0.955
0.9	0.526	0.954
0.9	0.034362	0.954
0.9	0.072	0.953
0.9	0.12969	0.945
0.9	0.105456	0.94
0.9	0.019656	0.939
0.9	0.052855	0.937
0.9	0.258	0.937
0.9	0.070348	0.936
0.9	0.02772	0.929
0.9	0.112	0.926
0.9	0.249	0.925
0.9	0.191646	0.924
0.9	0.041552	0.921
0.9	0.098736	0.921
0.9	0.100614	0.92
0.9	0.212	0.919
0.9	0.0336	0.919
0.9	0.133464	0.918
0.9	0.106	0.918
0.9	0.156238	0.916
0.9	0.074	0.915
0.9	0.185	0.915
0.9	0.074327	0.914
0.9	0	0.914
0.9	0.127332	0.914
0.9	0.088592	0.913
0.9	0.066429	0.913
0.9	0	0.912
0.9	0.13615	0.912
0.9	0.107568	0.911
0.9	0.106	0.911
0.9	0.092378	0.909
0.9	0.092072	0.909
0.9	0.086284	0.909
0.9	0.049392	0.909
0.9	0.076632	0.906
0.9	0.063875	0.906
0.9	0.068166	0.906
0.9	0.066124	0.906
0.9	0.067	0.906
0.9	0.0564	0.905
0.9	0.061686	0.904

0.9	0.0324	0.903
0.9	0.037668	0.902
0.9	0.039592	0.902
0.9	0.044	0.902
0.9	0.061	0.902
0.9	0.013515	0.901
0.9	0.058	0.901
0.9	0.046	0.9
0.9	0	0.9
0.9	0	0.9
0.9	0	0.9
0.9	0	0.9
0.9	0	0.9
0.9	0	0.9
0.9	0	0.9
0.9	0	0.9
0.9	0	0.9
0.9	0	0.9
0.9	0	0.9
0	0.203616	0.891
0.8	0.366	0.89
0	0.776	0.883
0	0.18352	0.873
0	0.16817	0.861
0	0.189873	0.855
0	0.132145	0.851
0	0.16456	0.85
0	0.329	0.845
0	0.791	0.834
0	0.152208	0.833
0	0.280082	0.83
0	0.811	0.821
0.72	0.126	0.821
0	0.139941	0.81
0.72	0.064	0.808
0	0.774	0.805
0	0.13653	0.803
0.6	0.519	0.799
0	0.118248	0.795
0	0.14152	0.795
0	0.689	0.792
0	0.115024	0.788
0	0.753	0.753
0	0.742	0.743
0	0.729	0.739
0	0.735	0.737
0	0.671	0.717
0	0.588	0.714
0	0.636	0.708
0	0.238336	0.701
0	0.642	0.699
0	0.569	0.698
0	0.541	0.693
0	0.060572	0.693
0	0.658	0.689
0.6	0	0.679
0	0.228228	0.678
0	0.636	0.676
0	0.16335	0.669

0	0.168	0.663
0	0.492	0.662
0	0.571	0.654
0	0.249	0.649
0.54	0.198	0.648
0	0	0.647
0	0.479	0.646
0	0.539	0.644
0	0.238788	0.643
0	0.514	0.642
0	0.252384	0.642
0	0.641	0.641
0	0.232343	0.638
0	0.441	0.638
0.54	0.168	0.635
0	0.130204	0.624
0	0.59	0.622
0	0.543	0.618
0	0.162	0.615
0	0.516	0.612
0	0.596	0.609
0.6	0	0.608
0	0.603	0.608
0.6	0.059	0.607
0	0.524	0.604
0	0.537	0.602
0	0.331	0.595
0	0.495	0.594
0	0.322	0.591
0	0.581	0.59
0	0.485	0.588
0	0.257282	0.588
0	0.336906	0.586
0	0.22	0.582
0	0.569	0.582
0	0.479	0.582
0.36	0	0.581
0	0.555	0.581
0	0.355	0.58
0	0.056	0.579
0	0.567	0.578
0	0.349	0.575
0	0.2184	0.574
0	0.209754	0.573
0	0.22099	0.569
0	0.3	0.568
0	0.272	0.568
0	0.254656	0.567
0	0.328	0.564
0	0.065	0.564
0	0.561	0.561
0	0.231768	0.561
0	0.529	0.56
0	0.22794	0.56
0	0.174504	0.56
0	0.209	0.56
0	0.212265	0.559
0	0.322176	0.559
0	0.22555	0.557

0	0	0.556
0	0.230622	0.556
0	0.543	0.554
0	0.402	0.554
0	0.197496	0.554
0	0.1862	0.551
0	0.55	0.55
0	0	0.55
0	0.087	0.547
0	0.356	0.546
0	0	0.544
0	0.544	0.544
0	0.52	0.544
0	0	0.543
0	0.456	0.541
0	0.477	0.541
0.54	0	0.54
0	0.422	0.54
0	0.452	0.54
0	0.466	0.539
0	0.052	0.538
0	0.294	0.537
0	0.34038	0.536
0	0.065	0.533
0	0	0.533
0	0.454	0.532
0	0.512	0.531
0	0.51	0.531
0	0.272	0.529
0	0	0.528
0	0.527	0.527
0	0.525	0.525
0	0.246792	0.525
0	0.512	0.524
0	0.405	0.522
0	0.447	0.522
0	0.509	0.521
0	0.518	0.518
0	0.398	0.517
0	0.512	0.516
0	0.304	0.516
0	0.25917	0.514
0	0.499	0.514
0	0.455	0.512
0	0	0.511
0	0.141	0.511
0	0.469	0.51
0	0	0.51
0	0.307	0.509
0	0	0.508
0	0.152	0.507
0	0.273	0.506
0	0.284	0.506
0	0.407	0.505
0	0.504	0.504
0	0	0.503
0	0.254	0.5
0	0.468	0.499
0	0.438	0.498

0	0.203	0.496
0	0.239364	0.496
0	0.472	0.496
0	0.203	0.495
0	0.378	0.495
0	0.043	0.494
0	0.494	0.494
0	0	0.489
0	0.484	0.489
0	0.246	0.488
0	0.095824	0.486
0	0.464	0.486
0.36	0	0.486
0.36	0	0.486
0	0.358	0.485
0	0.142	0.484
0	0.219	0.483
0	0.285	0.483
0	0.258	0.481
0	0.252539	0.481
0	0.243	0.48
0	0.146	0.48
0	0.459	0.479
0	0.354	0.479
0	0.376	0.478
0	0.477	0.477
0	0.251	0.473
0	0.243	0.473
0	0.205	0.472
0	0.194	0.471
0	0.225	0.471
0	0.47	0.47
0	0.276	0.469
0	0.246	0.468
0	0.429	0.468
0	0.265	0.466
0	0.33	0.465
0	0.123	0.464
0	0.426	0.464
0.36	0.195	0.463
0	0.44	0.463
0	0.136896	0.462
0	0.356	0.458
0	0.205	0.457
0	0.065	0.456
0	0.43	0.455
0	0.111	0.454
0	0.402	0.454
0	0.428	0.454
0	0.284589	0.45
0	0.416	0.45
0	0.093	0.449
0	0.441	0.449
0	0.407	0.449
0	0.187	0.449
0	0.44	0.448
0	0.193848	0.448
0	0.448	0.448
0	0.189	0.447

0	0.186	0.447
0	0.422	0.446
0	0.187	0.445
0	0.43	0.443
0	0.443	0.443
0	0.23474	0.441
0	0.412	0.441
0	0.188399	0.44
0	0.225308	0.439
0	0.414	0.439
0	0.056	0.438
0	0.219	0.437
0	0.061	0.433
0	0.419	0.433
0	0.139	0.433
0	0.328	0.433
0	0.05	0.432
0	0.42	0.432
0	0	0.431
0	0.198	0.431
0	0.353	0.43
0	0.163	0.429
0	0.401	0.429
0	0.207064	0.428
0	0.388	0.427
0	0.157	0.427
0	0.208921	0.426
0	0.064	0.425
0	0.283	0.424
0	0.112	0.423
0	0.220206	0.421
0	0.102	0.421
0	0.143	0.42
0	0.421	0.42
0	0.354	0.419
0	0	0.418
0	0.144	0.418
0	0.28	0.418
0	0.27048	0.417
0	0.132	0.416
0	0	0.416
0	0	0.416
0	0.307	0.416
0	0	0.414
0	0.18603	0.414
0	0.132	0.413
0	0.284	0.411
0	0.343	0.411
0	0.216795	0.411
0	0.177	0.41
0	0.309	0.41
0	0.201	0.41
0	0.198	0.409
0	0	0.408
0	0.174	0.408
0	0.187	0.407
0	0.194	0.407
0	0	0.406
0	0.355	0.406

0	0.328	0.405
0	0.131425	0.403
0	0.128	0.402
0	0.119	0.402
0	0	0.401
0	0.231633	0.4
