# Supplementary material for: LncRNA PANTR1 is Associated with Poor Prognostic and Suppresses Apoptosis in Glioma
Source: J Oncol. 2023 Feb 20;2023:8537036. doi: 10.1155/2023/8537036 (PMC9970703; doi:10.1155/2023/8537036)
Supplement: Supplementary Materials — Table 1: Differential expression analysis of PANTR1 in GBM/LGG. Table 2: Gene ontology enrichment analysis of PANTR1 using the clusterProfiler package. Table 3: Pathway enrichment analysis of PANTR1. Table 4: Protein-protein interaction network of PANTR1. Table 5: The association of PANTR1 expression level with clinical parameters of gliomas using the Chi-squared test or Fisher's exact test for analysis. Student's t-test or Wilcoxon rank sum test revealed that age was significantly (p < 0.001) associated with PANTR1 expression. Table 6: The association of PANTR1 expression level with pathological parameters of gliomas using logistics regression. PANTR1 expression was significantly correlated with these variables including WHO grade (p < 0.001), IDH status (p < 0.001), primary therapy outcome (p = 0.016), and EGFR status (p < 0.001). Table 7: Uni- and multivariate Cox regression analysis showed the prognostic value of PANTR1 in overall survival. We observed IDH status (p < 0.001), primary therapy outcome (p < 0.001), age (p = 0.022), and PANTR1 (p = 0.045) are independent prognostic factors in progression-free interval (p < 0.05) of gliomas. Table 8: Uni- and multivariate Cox regression analysis showed the prognostic value of PANTR1 in progression-free survival. Table 9: Uni- and multivariate Cox regression analysis showed the prognostic value of PANTR1 in disease-specific survival. Supplement 10: Relative PANTR1 expression. PCR showed that all 15 glioma samples' PANTR1 expression outweighs normal adjacent tissues, whereas grade II and III glioma tend to have a higher expression rather than GBM compared with NAT. [file 8537036.f1.zip › Supplement table6.docx]

Table.6 The association of PANTR1 expression level with pathological parameters of gliomas using logistics regression.

| Characteristics | Odds Ratio in PANTR1 expression | Odds Ratio(OR) | P value |
| --- | --- | --- | --- |
| WHO grade (G4 vs. G2&G3) | 613 | 3.41(2.32-5.09) | <0.001 |
| IDH status (WT vs. Mut) | 661 | 3.53(2.52-4.96) | <0.001 |
| 1p/19q codeletion (codel vs. non-codel) | 664 | 0.87(0.61-1.23) | 0.423 |
| Primary therapy outcome (CR vs. PD&SD&PR) | 444 | 0.60(0.39-0.90) | 0.016 |
| EGFR status (Mut vs. WT) | 656 | 4.02(2.31-7.38) | <0.001 |
| PIK3CA status (Mut vs. WT) | 656 | 0.86(0.48-1.54) | 0.609 |
