# Supplementary material for: LncRNA PANTR1 is Associated with Poor Prognostic and Suppresses Apoptosis in Glioma
Source: J Oncol. 2023 Feb 20;2023:8537036. doi: 10.1155/2023/8537036 (PMC9970703; doi:10.1155/2023/8537036)
Supplement: Supplementary Materials — Table 1: Differential expression analysis of PANTR1 in GBM/LGG. Table 2: Gene ontology enrichment analysis of PANTR1 using the clusterProfiler package. Table 3: Pathway enrichment analysis of PANTR1. Table 4: Protein-protein interaction network of PANTR1. Table 5: The association of PANTR1 expression level with clinical parameters of gliomas using the Chi-squared test or Fisher's exact test for analysis. Student's t-test or Wilcoxon rank sum test revealed that age was significantly (p < 0.001) associated with PANTR1 expression. Table 6: The association of PANTR1 expression level with pathological parameters of gliomas using logistics regression. PANTR1 expression was significantly correlated with these variables including WHO grade (p < 0.001), IDH status (p < 0.001), primary therapy outcome (p = 0.016), and EGFR status (p < 0.001). Table 7: Uni- and multivariate Cox regression analysis showed the prognostic value of PANTR1 in overall survival. We observed IDH status (p < 0.001), primary therapy outcome (p < 0.001), age (p = 0.022), and PANTR1 (p = 0.045) are independent prognostic factors in progression-free interval (p < 0.05) of gliomas. Table 8: Uni- and multivariate Cox regression analysis showed the prognostic value of PANTR1 in progression-free survival. Table 9: Uni- and multivariate Cox regression analysis showed the prognostic value of PANTR1 in disease-specific survival. Supplement 10: Relative PANTR1 expression. PCR showed that all 15 glioma samples' PANTR1 expression outweighs normal adjacent tissues, whereas grade II and III glioma tend to have a higher expression rather than GBM compared with NAT. [file 8537036.f1.zip › Supplement table7.docx]

**Table 7.** Uni- and multi-variate Cox regression analysis showed prognostic value of PANTR1 in overall survival.

| Characteristics | Total(N) | HR(95% CI) Univariate analysis | P value Univariate analysis | HR(95% CI) Multivariate analysis | P value Multivariate analysis |
| --- | --- | --- | --- | --- | --- |
| WHO grade (G4 vs. G2&G3) | 612 | 9.504(7.162-12.611) | <0.001 | 3.516(1.013-12.206) | 0.048 |
| IDH status (WT vs. Mut) | 660 | 9.850(7.428-13.061) | <0.001 | 3.911(2.220-6.888) | <0.001 |
| 1p/19q codeletion (codel vs. non-codel) | 663 | 0.216(0.138-0.338) | <0.001 | 0.630(0.355-1.119) | 0.115 |
| Primary therapy outcome (CR vs. PD&SD&PR) | 443 | 0.238(0.115-0.489) | <0.001 | 0.327(0.149-0.719) | 0.005 |
| Gender (Male vs. Female) | 669 | 1.230(0.955-1.585) | 0.109 |  |  |
| Age (>60 vs. <=60) | 669 | 4.716(3.609-6.161) | <0.001 | 3.654(2.195-6.082) | <0.001 |
| Race (White vs. Asian&Black or African American) | 657 | 0.806(0.492-1.321) | 0.393 |  |  |
| EGFR status (Mut vs. WT) | 655 | 3.628(2.672-4.927) | <0.001 | 1.019(0.481-2.160) | 0.961 |
| PIK3CA status (Mut vs. WT) | 655 | 1.011(0.625-1.635) | 0.966 |  |  |
| PANTR1 (High vs. Low) | 669 | 2.268(1.755-2.931) | <0.001 | 1.581(1.013-2.469) | 0.044 |
