# Supplementary material for: LncRNA PANTR1 is Associated with Poor Prognostic and Suppresses Apoptosis in Glioma
Source: J Oncol. 2023 Feb 20;2023:8537036. doi: 10.1155/2023/8537036 (PMC9970703; doi:10.1155/2023/8537036)
Supplement: Supplementary Materials — Table 1: Differential expression analysis of PANTR1 in GBM/LGG. Table 2: Gene ontology enrichment analysis of PANTR1 using the clusterProfiler package. Table 3: Pathway enrichment analysis of PANTR1. Table 4: Protein-protein interaction network of PANTR1. Table 5: The association of PANTR1 expression level with clinical parameters of gliomas using the Chi-squared test or Fisher's exact test for analysis. Student's t-test or Wilcoxon rank sum test revealed that age was significantly (p < 0.001) associated with PANTR1 expression. Table 6: The association of PANTR1 expression level with pathological parameters of gliomas using logistics regression. PANTR1 expression was significantly correlated with these variables including WHO grade (p < 0.001), IDH status (p < 0.001), primary therapy outcome (p = 0.016), and EGFR status (p < 0.001). Table 7: Uni- and multivariate Cox regression analysis showed the prognostic value of PANTR1 in overall survival. We observed IDH status (p < 0.001), primary therapy outcome (p < 0.001), age (p = 0.022), and PANTR1 (p = 0.045) are independent prognostic factors in progression-free interval (p < 0.05) of gliomas. Table 8: Uni- and multivariate Cox regression analysis showed the prognostic value of PANTR1 in progression-free survival. Table 9: Uni- and multivariate Cox regression analysis showed the prognostic value of PANTR1 in disease-specific survival. Supplement 10: Relative PANTR1 expression. PCR showed that all 15 glioma samples' PANTR1 expression outweighs normal adjacent tissues, whereas grade II and III glioma tend to have a higher expression rather than GBM compared with NAT. [file 8537036.f1.zip › Supplement table8.docx]

Table.8 Uni- and multi-variate Cox regression analysis showed prognostic value of PANTR1 in progression-free survival.

| Characteristics | Total(N) | HR(95% CI) Univariate analysis | P value Univariate analysis | HR(95% CI) Multivariate analysis | P value Multivariate analysis |
| --- | --- | --- | --- | --- | --- |
| WHO grade (G4 vs. G2&G3) | 592 | 9.499(7.058-12.783) | <0.001 | 3.390(0.976-11.768) | 0.055 |
| IDH status (WT vs. Mut) | 639 | 10.413(7.716-14.054) | <0.001 | 4.228(2.360-7.575) | <0.001 |
| 1p/19q codeletion (codel vs. non-codel) | 643 | 0.200(0.123-0.324) | <0.001 | 0.599(0.325-1.104) | 0.101 |
| Primary therapy outcome (CR vs. PD&SD&PR) | 439 | 0.218(0.101-0.470) | <0.001 | 0.299(0.128-0.697) | 0.005 |
| Gender (Male vs. Female) | 648 | 1.229(0.940-1.607) | 0.132 |  |  |
| Age (>60 vs. <=60) | 648 | 4.611(3.468-6.131) | <0.001 | 3.334(1.967-5.652) | <0.001 |
| Race (White vs. Asian&Black or African American) | 637 | 0.788(0.473-1.312) | 0.360 |  |  |
| EGFR status (Mut vs. WT) | 634 | 3.584(2.593-4.955) | <0.001 | 1.014(0.475-2.163) | 0.972 |
| PIK3CA status (Mut vs. WT) | 634 | 1.035(0.630-1.701) | 0.891 |  |  |
| PANTR1 (High vs. Low) | 648 | 2.465(1.875-3.240) | <0.001 | 1.651(1.032-2.644) | 0.037 |
