# Supplementary figures and images for: LncRNA PANTR1 is Associated with Poor Prognostic and Suppresses Apoptosis in Glioma
Source: J Oncol. 2023 Feb 20;2023:8537036. doi: 10.1155/2023/8537036 (PMC9970703; doi:10.1155/2023/8537036)

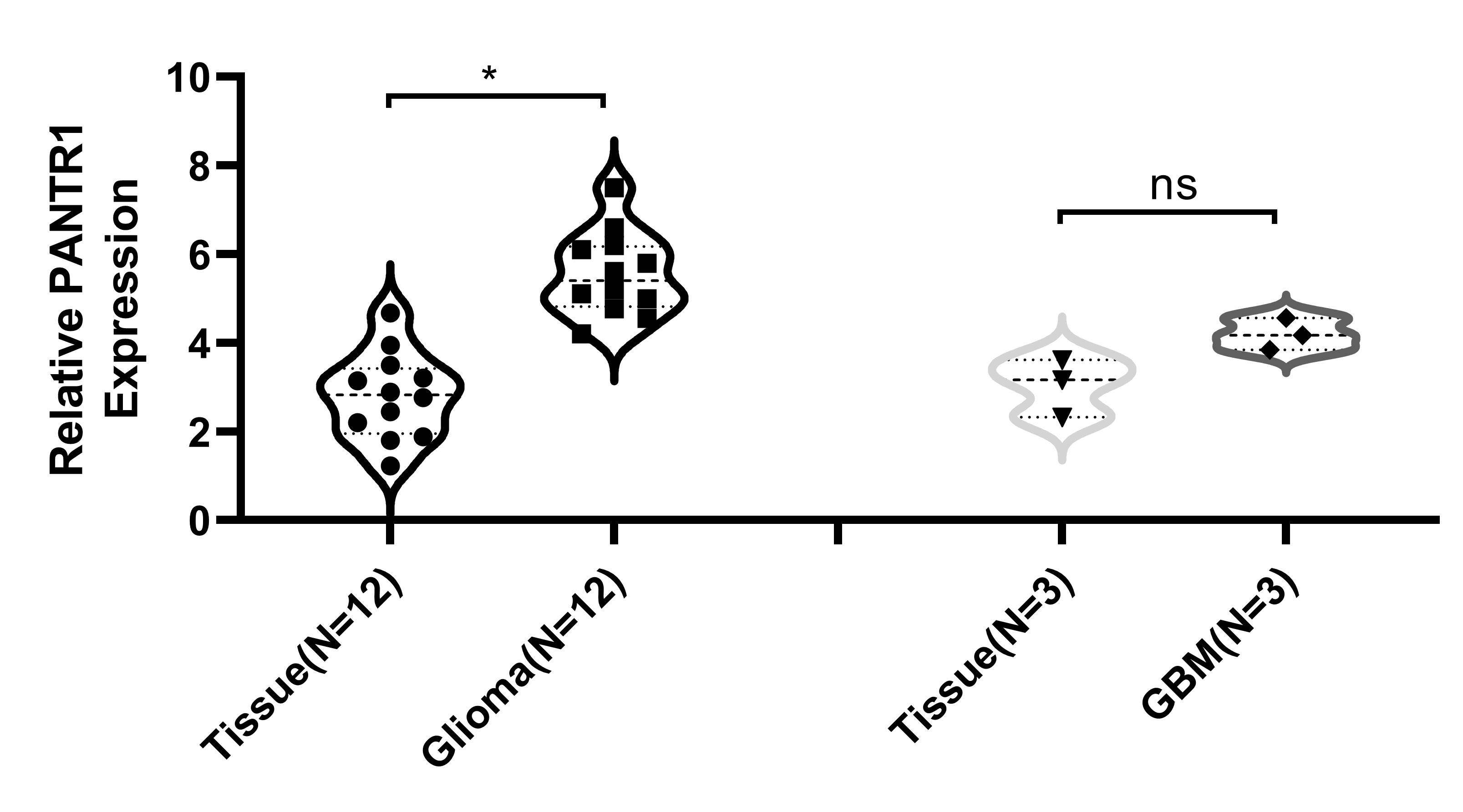

Supplement: Supplementary Materials — Table 1: Differential expression analysis of PANTR1 in GBM/LGG. Table 2: Gene ontology enrichment analysis of PANTR1 using the clusterProfiler package. Table 3: Pathway enrichment analysis of PANTR1. Table 4: Protein-protein interaction network of PANTR1. Table 5: The association of PANTR1 expression level with clinical parameters of gliomas using the Chi-squared test or Fisher's exact test for analysis. Student's t-test or Wilcoxon rank sum test revealed that age was significantly (p < 0.001) associated with PANTR1 expression. Table 6: The association of PANTR1 expression level with pathological parameters of gliomas using logistics regression. PANTR1 expression was significantly correlated with these variables including WHO grade (p < 0.001), IDH status (p < 0.001), primary therapy outcome (p = 0.016), and EGFR status (p < 0.001). Table 7: Uni- and multivariate Cox regression analysis showed the prognostic value of PANTR1 in overall survival. We observed IDH status (p < 0.001), primary therapy outcome (p < 0.001), age (p = 0.022), and PANTR1 (p = 0.045) are independent prognostic factors in progression-free interval (p < 0.05) of gliomas. Table 8: Uni- and multivariate Cox regression analysis showed the prognostic value of PANTR1 in progression-free survival. Table 9: Uni- and multivariate Cox regression analysis showed the prognostic value of PANTR1 in disease-specific survival. Supplement 10: Relative PANTR1 expression. PCR showed that all 15 glioma samples' PANTR1 expression outweighs normal adjacent tissues, whereas grade II and III glioma tend to have a higher expression rather than GBM compared with NAT. [file 8537036.f1.zip › Supplement10.jpg]
