# Supplementary material for: LncRNA PANTR1 is Associated with Poor Prognostic and Suppresses Apoptosis in Glioma
Source: J Oncol. 2023 Feb 20;2023:8537036. doi: 10.1155/2023/8537036 (PMC9970703; doi:10.1155/2023/8537036)
Supplement: Supplementary Materials — Table 1: Differential expression analysis of PANTR1 in GBM/LGG. Table 2: Gene ontology enrichment analysis of PANTR1 using the clusterProfiler package. Table 3: Pathway enrichment analysis of PANTR1. Table 4: Protein-protein interaction network of PANTR1. Table 5: The association of PANTR1 expression level with clinical parameters of gliomas using the Chi-squared test or Fisher's exact test for analysis. Student's t-test or Wilcoxon rank sum test revealed that age was significantly (p < 0.001) associated with PANTR1 expression. Table 6: The association of PANTR1 expression level with pathological parameters of gliomas using logistics regression. PANTR1 expression was significantly correlated with these variables including WHO grade (p < 0.001), IDH status (p < 0.001), primary therapy outcome (p = 0.016), and EGFR status (p < 0.001). Table 7: Uni- and multivariate Cox regression analysis showed the prognostic value of PANTR1 in overall survival. We observed IDH status (p < 0.001), primary therapy outcome (p < 0.001), age (p = 0.022), and PANTR1 (p = 0.045) are independent prognostic factors in progression-free interval (p < 0.05) of gliomas. Table 8: Uni- and multivariate Cox regression analysis showed the prognostic value of PANTR1 in progression-free survival. Table 9: Uni- and multivariate Cox regression analysis showed the prognostic value of PANTR1 in disease-specific survival. Supplement 10: Relative PANTR1 expression. PCR showed that all 15 glioma samples' PANTR1 expression outweighs normal adjacent tissues, whereas grade II and III glioma tend to have a higher expression rather than GBM compared with NAT. [file Supplement Table 1.pdf › Supplement Table 1.pdf]

gene_id	baseMean	log2FoldC	lfcSE	stat	pvalue	padj	gene_nam	gene_bioty
ENSG0000	1030532	-0.5015	0.0898	-5.58466	2.34E-08	1.23E-07	GFAP	protein_cc
ENSG0000	778122.6	-0.64351	0.05776	-11.141	7.92E-29	1.48E-26	MT-CO1	protein_cc
ENSG0000	728722.1	-0.76751	0.069665	-11.0171	3.16E-28	4.96E-26	MT-RNR2	Mt_rRNA
ENSG0000	634309	-0.66963	0.06286	-10.6527	1.69E-26	2.01E-24	MT-ND4	protein_cc
ENSG0000	434074.7	0.029953	0.076844	0.389784	0.696696	0.767812	CLU	protein_cc
ENSG0000	337579.9	-0.52019	0.05994	-8.67848	4.01E-18	1.02E-16	MT-CO3	protein_cc
ENSG0000	312924.5	-0.56974	0.059974	-9.49989	2.10E-21	9.21E-20	MT-CO2	protein_cc
ENSG0000	281412.6	-0.68845	0.065838	-10.4567	1.36E-25	1.35E-23	MT-CYB	protein_cc
ENSG0000	249340.7	-0.28341	0.075836	-3.73712	0.000186	0.000519	SPARC	protein_cc
ENSG0000	242753.8	0.151664	0.043488	3.487465	0.000488	0.00126	ACTB	protein_cc
ENSG0000	237159.2	-0.79271	0.062437	-12.6962	6.21E-37	5.67E-34	MT-ND5	protein_cc
ENSG0000	234316.6	-0.61884	0.060488	-10.2308	1.44E-24	1.16E-22	MT-ATP6	protein_cc
ENSG0000	224678.2	0.193053	0.050361	3.833417	0.000126	0.000363	EEF1A1	protein_cc
ENSG0000	218856.2	-0.64074	0.066528	-9.63107	5.91E-22	2.81E-20	MT-ND2	protein_cc
ENSG0000	216603.3	-0.66089	0.067274	-9.82385	8.89E-23	4.95E-21	MT-ND1	protein_cc
ENSG0000	168447.9	-0.02828	0.08685	-0.32557	0.744748	0.807398	BCAN	protein_cc
ENSG0000	161957.3	-0.50532	0.068495	-7.37741	1.61E-13	1.90E-12	SPARCL1	protein_cc
ENSG0000	140971.9	0.294868	0.053202	5.542404	2.98E-08	1.54E-07	GAPDH	protein_cc
ENSG0000	134930.9	-0.44276	0.076043	-5.82257	5.79E-09	3.33E-08	APOE	protein_cc
ENSG0000	130452	-0.79771	0.07331	-10.8813	1.41E-27	2.00E-25	MT-RNR1	Mt_rRNA
ENSG0000	130318.9	0.09199	0.038157	2.410828	0.015916	0.030369	ACTG1	protein_cc
ENSG0000	124314.5	-0.14366	0.045917	-3.12868	0.001756	0.004096	EEF2	protein_cc
ENSG0000	112979	-0.26882	0.038489	-6.98441	2.86E-12	2.77E-11	PSAP	protein_cc
ENSG0000	112524.5	-0.49364	0.053966	-9.14717	5.84E-20	1.99E-18	PEA15	protein_cc
ENSG0000	111338.4	-0.25905	0.096528	-2.68365	0.007282	0.015001	CRYAB	protein_cc
ENSG0000	111170.5	-0.74752	0.143819	-5.19763	2.02E-07	9.11E-07	MBP	protein_cc
ENSG0000	109228	-0.71218	0.109512	-6.50325	7.86E-11	6.08E-10	PLP1	protein_cc
ENSG0000	101083.3	-0.78792	0.072943	-10.8019	3.37E-27	4.50E-25	MT-ND6	protein_cc
ENSG0000	100143.9	0.195374	0.083802	2.331389	0.019733	0.036769	FTL	protein_cc
ENSG0000	97913.93	-0.34427	0.061771	-5.5734	2.50E-08	1.30E-07	GLUL	protein_cc
ENSG0000	97598.69	0.425033	0.066369	6.404061	1.51E-10	1.12E-09	PTPRZ1	protein_cc
ENSG0000	93178.62	0.461186	0.06081	7.584001	3.35E-14	4.41E-13	TUBA1A	protein_cc
ENSG0000	87953.71	0.051293	0.031484	1.629186	0.103274	0.15888	GNAS	protein_cc
ENSG0000	87281.46	-0.78991	0.087238	-9.05459	1.37E-19	4.38E-18	NTRK2	protein_cc
ENSG0000	85209.31	-0.4754	0.063405	-7.49775	6.49E-14	8.19E-13	MT-ND3	protein_cc
ENSG0000	81987.37	-0.11449	0.050499	-2.26717	0.02338	0.042862	RPL3	protein_cc
ENSG0000	80872.66	-0.13238	0.03908	-3.38736	0.000706	0.001769	APP	protein_cc
ENSG0000	80642.42	-0.66512	0.080793	-8.23241	1.83E-16	3.57E-15	NDRG2	protein_cc
ENSG0000	80085.48	0.15926	0.050832	3.133084	0.00173	0.00404	TPT1	protein_cc
ENSG0000	77733.36	-0.34426	0.048597	-7.08396	1.40E-12	1.42E-11	GPM6B	protein_cc
ENSG0000	77670.25	0.354916	0.073987	4.797	1.61E-06	6.30E-06	B2M	protein_cc
ENSG0000	76285.95	0.545309	0.138923	3.925265	8.66E-05	0.000256	SPP1	protein_cc
ENSG0000	72834.12	0.626292	0.112786	5.552937	2.81E-08	1.45E-07	VIM	protein_cc
ENSG0000	72680.17	1.234038	0.228644	5.39721	6.77E-08	3.29E-07	CHI3L1	protein_cc
ENSG0000	70270.94	-0.60891	0.073563	-8.27744	1.26E-16	2.52E-15	CPE	protein_cc
ENSG0000	67517.47	-0.04768	0.092386	-0.51612	0.60577	0.689884	AQP4	protein_cc
ENSG0000	64702.78	-0.7789	0.084372	-9.23179	2.66E-20	9.47E-19	SCD	protein_cc
ENSG0000	64679.14	0.679737	0.082641	8.225144	1.95E-16	3.77E-15	TMSB4X	protein_cc
ENSG0000	62828.95	0.11213	0.109415	1.024812	0.305452	0.398408	CD74	protein_cc
ENSG0000	61729.39	0.394816	0.08992	4.390749	1.13E-05	3.87E-05	CST3	protein_cc
ENSG0000	61646.41	-0.11231	0.051379	-2.18593	0.02882	0.051763	RPL4	protein_cc
ENSG0000	61498.45	-0.00874	0.0342	-0.2557	0.798183	0.85086	HSPA8	protein_cc
ENSG0000	59332.66	0.135723	0.054445	2.492868	0.012672	0.024712	RPL13A	protein_cc
ENSG0000	59094.83	-0.06441	0.06103	-1.05542	0.291235	0.382755	ITM2C	protein_cc
ENSG0000	57655	0.056383	0.049595	1.136855	0.255599	0.343269	PKM	protein_cc
ENSG0000	57577.55	0.073095	0.066803	1.094178	0.273877	0.363837	C1orf61	protein_cc
ENSG0000	57499.01	0.092421	0.048987	1.886633	0.05921	0.097926	ENO1	protein_cc
ENSG0000	57451.06	0.287767	0.058898	4.885865	1.03E-06	4.15E-06	RPLP0	protein_cc

ENSG0000	57288.08	-0.53631	0.085487	-6.27352	3.53E-10	2.47E-09	FAM107A	protein_co
ENSG0000	57034.69	0.042073	0.027217	1.545824	0.122147	0.183437	YWHAE	protein_co
ENSG0000	56764.31	-0.49772	0.099705	-4.99192	5.98E-07	2.50E-06	ATP1A2	protein_co
ENSG0000	55965.88	-0.11661	0.042376	-2.75177	0.005927	0.012431	ALDOA	protein_co
ENSG0000	55956.62	-0.03503	0.063995	-0.54744	0.584077	0.670488	SLC1A3	protein_co
ENSG0000	55192.72	-0.19397	0.061896	-3.13383	0.001725	0.00403	SCD5	protein_co
ENSG0000	55121.02	0.068773	0.044578	1.542769	0.122887	0.184371	DDR1	protein_co
ENSG0000	55095.31	-0.19717	0.037906	-5.20141	1.98E-07	8.94E-07	HSP90AA1	protein_co
ENSG0000	54575.01	-0.18065	0.05395	-3.34843	0.000813	0.002014	GPM6A	protein_co
ENSG0000	54357.12	-0.43914	0.053629	-8.18838	2.65E-16	5.03E-15	CALM1	protein_co
ENSG0000	53925.11	-0.33185	0.070909	-4.68001	2.87E-06	1.08E-05	PMP2	protein_co
ENSG0000	53599.47	-0.06804	0.037273	-1.82553	0.067921	0.11035	HSP90AB1	protein_co
ENSG0000	52184.49	-0.13083	0.061433	-2.12954	0.033209	0.058767	CKB	protein_co
ENSG0000	51809.66	-0.22765	0.041196	-5.52602	3.28E-08	1.68E-07	QKI	protein_co
ENSG0000	51148.91	0.11554	0.031833	3.629616	0.000284	0.000767	TMBIM6	protein_co
ENSG0000	50416.88	-0.08309	0.050894	-1.63262	0.102549	0.157895	LRP1	protein_co
ENSG0000	50238.5	-0.64264	0.100397	-6.40097	1.54E-10	1.14E-09	SLC1A2	protein_co
ENSG0000	49682.43	-0.47514	0.091288	-5.20485	1.94E-07	8.79E-07	ALDOC	protein_co
ENSG0000	49594.64	0.089711	0.054094	1.658428	0.097231	0.150757	RPS6	protein_co
ENSG0000	48915.16	1.683948	0.119692	14.06905	5.89E-45	1.34E-41	EGFR	protein_co
ENSG0000	47777.67	-0.07033	0.04485	-1.56818	0.116838	0.176701	CD81	protein_co
ENSG0000	47351.66	-0.16532	0.049367	-3.34884	0.000812	0.002011	DPYSL3	protein_co
ENSG0000	46792.08	0.088291	0.029503	2.992663	0.002766	0.006202	CFL1	protein_co
ENSG0000	45371	-0.39207	0.045048	-8.70354	3.22E-18	8.32E-17	SPTAN1	protein_co
ENSG0000	44137.99	0.004176	0.069472	0.060105	0.952072	0.966901	CTSB	protein_co
ENSG0000	44125.1	0.040357	0.031859	1.266727	0.205253	0.285452	CANX	protein_co
ENSG0000	44109.14	0.122601	0.051464	2.382241	0.017208	0.032525	RPL8	protein_co
ENSG0000	44080.65	-0.07845	0.038771	-2.02333	0.04304	0.074005	RPL15	protein_co
ENSG0000	43179.38	0.168291	0.046403	3.626719	0.000287	0.000775	RPL10	protein_co
ENSG0000	42923.28	0.201255	0.055613	3.618884	0.000296	0.000797	SDC3	protein_co
ENSG0000	42173.46	-0.35682	0.059778	-5.96909	2.39E-09	1.46E-08	NCAM1	protein_co
ENSG0000	42108.61	-0.22125	0.043306	-5.109	3.24E-07	1.41E-06	LDHB	protein_co
ENSG0000	42036.02	0.239463	0.05372	4.4576	8.29E-06	2.90E-05	RPS11	protein_co
ENSG0000	41342.28	0.145569	0.049626	2.933346	0.003353	0.007404	RPL7A	protein_co
ENSG0000	41267.49	0.173285	0.048805	3.55053	0.000384	0.001014	RPS4X	protein_co
ENSG0000	41221.8	0.188429	0.046391	4.061743	4.87E-05	0.00015	GNB2L1	protein_co
ENSG0000	40703.34	0.010298	0.030473	0.337949	0.735402	0.799732	2-Sep	protein_co
ENSG0000	40626.5	-0.23092	0.041044	-5.62623	1.84E-08	9.81E-08	SYT11	protein_co
ENSG0000	40187.47	0.382497	0.062403	6.129511	8.81E-10	5.80E-09	RPS18	protein_co
ENSG0000	39870.29	0.150237	0.02588	5.805251	6.43E-09	3.68E-08	HNRNPA2	protein_co
ENSG0000	39849.2	-0.12635	0.070109	-1.80226	0.071505	0.115416	A2M	protein_co
ENSG0000	39708.53	-0.47502	0.043186	-10.9994	3.85E-28	5.97E-26	SPTBN1	protein_co
ENSG0000	39682.75	-0.59069	0.0643	-9.1864	4.06E-20	1.41E-18	MT-ND4L	protein_co
ENSG0000	39408.94	0.091126	0.030733	2.965038	0.003026	0.006738	EIF4G2	protein_co
ENSG0000	39406.96	-0.73296	0.088572	-8.27524	1.28E-16	2.56E-15	ADCYAP1I	protein_co
ENSG0000	39112.49	-0.04447	0.060423	-0.7359	0.461789	0.557924	ATP1B2	protein_co
ENSG0000	38611.18	-0.34188	0.041121	-8.31404	9.25E-17	1.89E-15	MAP4	protein_co
ENSG0000	38508.92	0.074137	0.040907	1.812308	0.069939	0.113243	RPL19	protein_co
ENSG0000	38377.88	-0.03236	0.035362	-0.91517	0.360101	0.455977	H3F3B	protein_co
ENSG0000	38324.76	0.420997	0.086528	4.865449	1.14E-06	4.57E-06	HLA-B	protein_co
ENSG0000	37794.86	0.208433	0.050608	4.118562	3.81E-05	0.00012	RPL13	protein_co
ENSG0000	37618.13	0.185642	0.048869	3.798793	0.000145	0.000413	RPLP1	protein_co
ENSG0000	37615.2	0.255215	0.095336	2.677019	0.007428	0.015273	NCAN	protein_co
ENSG0000	37536.28	0.143627	0.05203	2.760475	0.005772	0.012136	GPR56	protein_co
ENSG0000	37515.71	0.198675	0.05702	3.484319	0.000493	0.001274	RPS3	protein_co
ENSG0000	37301.71	-0.40473	0.057428	-7.04767	1.82E-12	1.82E-11	MAP1B	protein_co
ENSG0000	36890.87	-0.55112	0.073299	-7.51884	5.53E-14	7.03E-13	AHCYL1	protein_co
ENSG0000	36867.14	-0.08514	0.108279	-0.7863	0.431693	0.528692	C3	protein_co
ENSG0000	36518.5	-0.11996	0.039619	-3.02773	0.002464	0.005593	ITM2B	protein_co

ENSG0000(	36445.76	-0.33462	0.073074	-4.57917	4.67E-06	1.70E-05	MAP2	protein_cc
ENSG0000(	36368.18	-0.33847	0.04459	-7.59067	3.18E-14	4.21E-13	RTN3	protein_cc
ENSG0000(	36281.05	-0.08818	0.035053	-2.51577	0.011877	0.023331	TSPAN3	protein_cc
ENSG0000(	36023.38	-0.36571	0.125831	-2.9064	0.003656	0.008011	TNR	protein_cc
ENSG0000(	36011.55	-0.32557	0.061649	-5.28097	1.28E-07	5.98E-07	FADS2	protein_cc
ENSG0000(	35859.16	-0.24278	0.065258	-3.72031	0.000199	0.000552	RHOB	protein_cc
ENSG0000(	35837.07	-0.0739	0.111551	-0.66249	0.507659	0.601235	FN1	protein_cc
ENSG0000(	35251.76	-0.17981	0.036868	-4.87717	1.08E-06	4.33E-06	DPYSL2	protein_cc
ENSG0000(	35153.25	0.16573	0.054851	3.021457	0.002516	0.005701	RPS8	protein_cc
ENSG0000(	35061.88	-0.1766	0.076295	-2.31467	0.020631	0.038288	AGT	protein_cc
ENSG0000(	34969.2	0.078598	0.041404	1.89833	0.057653	0.095717	CALM2	protein_cc
ENSG0000(	34715.99	-0.84326	0.076413	-11.0355	2.58E-28	4.26E-26	KIF1A	protein_cc
ENSG0000(	34689.46	-0.00768	0.105181	-0.07299	0.941818	0.959359	SOX8	protein_cc
ENSG0000(	34502.81	-0.02069	0.031693	-0.65272	0.513935	0.606806	UBC	protein_cc
ENSG0000(	34488.18	0.402774	0.074282	5.422241	5.89E-08	2.89E-07	NES	protein_cc
ENSG0000(	34139.75	-0.15436	0.073371	-2.10385	0.035391	0.062241	FXYD6	protein_cc
ENSG0000(	34127.87	-0.48417	0.046224	-10.4745	1.13E-25	1.15E-23	DYNC1H1	protein_cc
ENSG0000(	34106.01	0.519085	0.078294	6.629941	3.36E-11	2.75E-10	HLA-A	protein_cc
ENSG0000(	33890.8	-0.44425	0.071603	-6.20443	5.49E-10	3.72E-09	CNP	protein_cc
ENSG0000(	33863.87	0.369044	0.116061	3.179728	0.001474	0.003487	SOD2	protein_cc
ENSG0000(	33497.67	-0.54936	0.057927	-9.4836	2.46E-21	1.06E-19	GPRC5B	protein_cc
ENSG0000(	33366.74	0.04414	0.051586	0.855651	0.392191	0.4888	RPL5	protein_cc
ENSG0000(	33182.15	0.938871	0.138725	6.767857	1.31E-11	1.14E-10	AQP1	protein_cc
ENSG0000(	33058.87	-0.23108	0.03257	-7.09496	1.29E-12	1.32E-11	GDI1	protein_cc
ENSG0000(	32595.21	-0.35163	0.045619	-7.70801	1.28E-14	1.82E-13	PEBP1	protein_cc
ENSG0000(	32438.17	-0.57573	0.066831	-8.61469	7.01E-18	1.72E-16	MTATP6P:	pseudoge
ENSG0000(	32002.41	0.168713	0.049316	3.421027	0.000624	0.00158	RPL37A	protein_cc
ENSG0000(	31957.98	-0.08318	0.036019	-2.30943	0.02092	0.038772	APLP2	protein_cc
ENSG0000(	31492.44	-0.45224	0.068989	-6.55528	5.55E-11	4.40E-10	LRIG1	protein_cc
ENSG0000(	31383.39	-0.2153	0.041974	-5.12941	2.91E-07	1.28E-06	SRRM2	protein_cc
ENSG0000(	31252.83	0.304127	0.047685	6.377823	1.80E-10	1.31E-09	CALR	protein_cc
ENSG0000(	31225.91	-0.0375	0.054332	-0.69024	0.490045	0.584517	RPS24	protein_cc
ENSG0000(	31176.04	-0.18821	0.050921	-3.69611	0.000219	0.000603	PABPC1	protein_cc
ENSG0000(	31138.93	0.155994	0.037157	4.198278	2.69E-05	8.65E-05	BSG	protein_cc
ENSG0000(	31035.04	0.236472	0.051507	4.591076	4.41E-06	1.61E-05	RPL32	protein_cc
ENSG0000(	30824.35	-0.06447	0.055352	-1.16469	0.244146	0.330261	MARCKSL:	protein_cc
ENSG0000(	30767.89	-0.57833	0.073659	-7.85138	4.11E-15	6.42E-14	PLEKHB1	protein_cc
ENSG0000(	30756.48	0.032155	0.036856	0.872438	0.38297	0.479604	HNRNPA1	protein_cc
ENSG0000(	30449.46	-0.07567	0.04192	-1.80517	0.071048	0.114741	TIMP2	protein_cc
ENSG0000(	30417.79	0.339993	0.10756	3.160963	0.001572	0.0037	IGFBP5	protein_cc
ENSG0000(	30397.41	-0.14804	0.075587	-1.95856	0.050164	0.084737	MLC1	protein_cc
ENSG0000(	30272.37	0.348511	0.057668	6.043428	1.51E-09	9.55E-09	RPSA	protein_cc
ENSG0000(	30225.88	0.400448	0.079583	5.03185	4.86E-07	2.06E-06	HLA-C	protein_cc
ENSG0000(	30124.5	0.316581	0.049603	6.382327	1.74E-10	1.28E-09	TUBB	protein_cc
ENSG0000(	29993.12	-0.66252	0.067823	-9.76823	1.54E-22	8.23E-21	MT-ATP8	protein_cc
ENSG0000(	29919.43	-0.79679	0.084449	-9.43516	3.90E-21	1.62E-19	MTURN	protein_cc
ENSG0000(	29642.47	0.06117	0.018176	3.365476	0.000764	0.001902	HNRNPK	protein_cc
ENSG0000(	29496.65	-0.18166	0.054671	-3.32286	0.000891	0.002191	TSC22D1	protein_cc
ENSG0000(	29300.86	-0.14181	0.046875	-3.02537	0.002483	0.005633	NFIX	protein_cc
ENSG0000(	29252.66	-0.50318	0.130669	-3.85076	0.000118	0.00034	SMOC1	protein_cc
ENSG0000(	29141.93	0.556543	0.070931	7.846286	4.29E-15	6.66E-14	CD63	protein_cc
ENSG0000(	29131.65	-0.2156	0.099562	-2.16551	0.030349	0.054205	GJA1	protein_cc
ENSG0000(	28996.13	0.435501	0.058384	7.459278	8.70E-14	1.07E-12	MYL6	protein_cc
ENSG0000(	28968.62	0.054137	0.034443	1.571759	0.116006	0.175663	ATP5B	protein_cc
ENSG0000(	28967.49	0.335129	0.056548	5.926408	3.10E-09	1.86E-08	RPS2	protein_cc
ENSG0000(	28947.39	0.215697	0.05818	3.707405	0.000209	0.000578	UBB	protein_cc
ENSG0000(	28861.84	0.049693	0.028378	1.75112	0.079925	0.12716	EIF1	protein_cc
ENSG0000(	28783.88	0.054044	0.056144	0.962591	0.335753	0.430545	BAALC	protein_cc
ENSG0000(	28699.82	0.327733	0.05879	5.574675	2.48E-08	1.29E-07	RPS19	protein_cc

ENSG0000(	28675.92	-0.56975	0.058936	-9.66742	4.15E-22	2.04E-20	CALM3	protein_cc
ENSG0000(	28651.71	0.114003	0.059658	1.910958	0.05601	0.093295	HTRA1	protein_cc
ENSG0000(	28525.27	-0.5049	0.063532	-7.94718	1.91E-15	3.15E-14	DST	protein_cc
ENSG0000(	28514.87	0.017312	0.026917	0.643161	0.52012	0.612496	DDX5	protein_cc
ENSG0000(	28480.24	0.004475	0.057199	0.078238	0.937638	0.956478	TSC22D4	protein_cc
ENSG0000(	28097.95	-0.82989	0.101261	-8.19554	2.49E-16	4.75E-15	PTGDS	protein_cc
ENSG0000(	28039.54	-0.01826	0.089709	-0.20357	0.838687	0.882621	OLIG1	protein_cc
ENSG0000(	28028.48	-0.61237	0.068653	-8.91973	4.67E-19	1.37E-17	HIPK2	protein_cc
ENSG0000(	27896.84	-0.05124	0.030819	-1.66249	0.096415	0.149667	NCL	protein_cc
ENSG0000(	27648.58	-0.13829	0.069098	-2.00143	0.045346	0.077484	TTYH1	protein_cc
ENSG0000(	27409.73	-0.3143	0.034705	-9.05642	1.35E-19	4.32E-18	LINC0065	lincRNA
ENSG0000(	27397.76	-0.01598	0.039368	-0.40603	0.684723	0.757847	DDX17	protein_cc
ENSG0000(	27304.12	-0.56298	0.102599	-5.48724	4.08E-08	2.06E-07	SCG3	protein_cc
ENSG0000(	27252.43	0.759286	0.07267	10.44837	1.49E-25	1.45E-23	PTN	protein_cc
ENSG0000(	27140.02	0.184894	0.049071	3.767858	0.000165	0.000463	RPS9	protein_cc
ENSG0000(	27056.15	0.054865	0.046608	1.177144	0.239138	0.324467	RPS20	protein_cc
ENSG0000(	27033.89	-0.11295	0.029019	-3.89212	9.94E-05	0.00029	MRFAP1	protein_cc
ENSG0000(	26876.66	-0.14581	0.031511	-4.62745	3.70E-06	1.37E-05	GNB1	protein_cc
ENSG0000(	26795.31	-0.31714	0.034918	-9.08238	1.06E-19	3.46E-18	AP2B1	protein_cc
ENSG0000(	26512.92	-0.21743	0.034345	-6.33088	2.44E-10	1.75E-09	RTN4	protein_cc
ENSG0000(	26455.38	0.471802	0.067761	6.962699	3.34E-12	3.21E-11	CNN3	protein_cc
ENSG0000(	26322.14	0.225229	0.056015	4.020856	5.80E-05	0.000177	RAB31	protein_cc
ENSG0000(	26280.03	-0.10219	0.028192	-3.62497	0.000289	0.00078	TMEM66	protein_cc
ENSG0000(	26250.42	-0.03749	0.032917	-1.13886	0.254763	0.342348	GNAI2	protein_cc
ENSG0000(	26242.61	-0.09726	0.052252	-1.86132	0.0627	0.102953	FTH1	protein_cc
ENSG0000(	26073.77	0.054255	0.054483	0.995821	0.319337	0.413047	RPS12	protein_cc
ENSG0000(	25970.63	-0.38336	0.046269	-8.28535	1.18E-16	2.36E-15	CLSTN1	protein_cc
ENSG0000(	25924.95	-0.00626	0.047369	-0.13211	0.894896	0.925332	CIRBP	protein_cc
ENSG0000(	25862.57	0.010344	0.031011	0.333575	0.738701	0.802452	ATP5A1	protein_cc
ENSG0000(	25838.23	0.303764	0.052687	5.765401	8.15E-09	4.58E-08	RPS14	protein_cc
ENSG0000(	25826.36	-0.39835	0.042029	-9.47801	2.59E-21	1.11E-19	SERINC1	protein_cc
ENSG0000(	25756.23	-0.24994	0.02878	-8.68423	3.81E-18	9.73E-17	CLTC	protein_cc
ENSG0000(	25742.35	-0.1784	0.065392	-2.72823	0.006368	0.01327	ABAT	protein_cc
ENSG0000(	25677.12	-0.05125	0.042645	-1.20176	0.229456	0.313368	PTTG1IP	protein_cc
ENSG0000(	25671.96	0.040661	0.030159	1.348233	0.177584	0.252444	RHOA	protein_cc
ENSG0000(	25563.79	0.022023	0.057136	0.385446	0.699907	0.770567	NRCAM	protein_cc
ENSG0000(	25512.66	0.159432	0.079964	1.993809	0.046173	0.07875	FLNA	protein_cc
ENSG0000(	25454.84	-0.03655	0.062988	-0.58023	0.561762	0.650483	HLA-E	protein_cc
ENSG0000(	25432.64	0.317242	0.081826	3.877019	0.000106	0.000308	S100B	protein_cc
ENSG0000(	25400.41	0.494631	0.099934	4.949561	7.44E-07	3.07E-06	MT3	protein_cc
ENSG0000(	25389.9	0.084334	0.038241	2.205305	0.027433	0.049482	HSP90B1	protein_cc
ENSG0000(	25295.25	0.006481	0.043486	0.149026	0.881533	0.915366	RPS23	protein_cc
ENSG0000(	25230.13	-0.68057	0.092624	-7.34764	2.02E-13	2.32E-12	GABBR1	protein_cc
ENSG0000(	25229.76	-0.53721	0.069329	-7.7486	9.29E-15	1.36E-13	MAPT	protein_cc
ENSG0000(	25184.91	-0.02651	0.03888	-0.68183	0.495345	0.589563	NACA	protein_cc
ENSG0000(	25183.98	-0.55919	0.056521	-9.89353	4.44E-23	2.65E-21	PLEC	protein_cc
ENSG0000(	24987.99	-0.84079	0.070744	-11.885	1.42E-32	5.34E-30	MAP1A	protein_cc
ENSG0000(	24921.2	0.615058	0.088801	6.926251	4.32E-12	4.07E-11	TMSB10	protein_cc
ENSG0000(	24844.92	-0.60509	0.060009	-10.0834	6.54E-24	4.67E-22	GLUD1	protein_cc
ENSG0000(	24819.81	-0.23218	0.030347	-7.65101	1.99E-14	2.74E-13	NUCKS1	protein_cc
ENSG0000(	24802.24	0.21543	0.051727	4.164789	3.12E-05	9.92E-05	RPL27A	protein_cc
ENSG0000(	24743.85	-0.12717	0.036999	-3.43713	0.000588	0.001498	YWHAZ	protein_cc
ENSG0000(	24612.5	-0.62829	0.067112	-9.36182	7.84E-21	3.06E-19	SPOCK2	protein_cc
ENSG0000(	24407.92	0.140395	0.055153	2.545549	0.010911	0.021618	RPL18	protein_cc
ENSG0000(	24407.91	-0.33461	0.060542	-5.5269	3.26E-08	1.67E-07	THRA	protein_cc
ENSG0000(	24324.83	0.042297	0.043414	0.974275	0.32992	0.42432	RPL6	protein_cc
ENSG0000(	24302.1	0.043188	0.032617	1.324073	0.185479	0.261855	MAGED1	protein_cc
ENSG0000(	24291.15	0.213105	0.051971	4.100491	4.12E-05	0.000129	RPLP2	protein_cc
ENSG0000(	24288.67	0.185703	0.037385	4.967358	6.79E-07	2.82E-06	7-Sep	protein_cc

ENSG0000(	24260.79	0.044829	0.048515	0.924023	0.355474	0.451106	RPL7	protein_cc
ENSG0000(	23906.08	0.001363	0.047413	0.028758	0.977058	0.983823	RPL30	protein_cc
ENSG0000(	23838.47	-0.09615	0.038105	-2.52335	0.011624	0.022886	NAP1L1	protein_cc
ENSG0000(	23634.14	0.201446	0.056191	3.58501	0.000337	0.000899	RPS16	protein_cc
ENSG0000(	23594.41	0.024578	0.04638	0.529917	0.596169	0.681403	RPL23	protein_cc
ENSG0000(	23573.74	-0.25319	0.028811	-8.78788	1.52E-18	4.12E-17	ADD1	protein_cc
ENSG0000(	23529.9	0.001461	0.072075	0.020271	0.983827	0.988901	RASSF2	protein_cc
ENSG0000(	23506.5	0.293416	0.124763	2.35178	0.018684	0.035019	HLA-DRA	protein_cc
ENSG0000(	23285.35	0.254541	0.056141	4.533936	5.79E-06	2.08E-05	TPI1	protein_cc
ENSG0000(	23264.8	-0.10266	0.029025	-3.53679	0.000405	0.001064	AP2M1	protein_cc
ENSG0000(	23172.54	0.023236	0.050771	0.457668	0.647191	0.726313	RPL31	protein_cc
ENSG0000(	23093.51	-0.80427	0.076857	-10.4645	1.26E-25	1.26E-23	AHNAK	protein_cc
ENSG0000(	23092.34	0.436377	0.055683	7.836851	4.62E-15	7.15E-14	DBI	protein_cc
ENSG0000(	23060.21	-0.14332	0.046221	-3.10084	0.00193	0.004463	FEZ1	protein_cc
ENSG0000(	22956.33	-0.11245	0.043906	-2.56121	0.010431	0.020756	RPL37	protein_cc
ENSG0000(	22947.67	-0.39712	0.050644	-7.84143	4.45E-15	6.90E-14	CTNND2	protein_cc
ENSG0000(	22903.38	-0.17953	0.062789	-2.85919	0.004247	0.009187	DNER	protein_cc
ENSG0000(	22866.47	-0.15874	0.055688	-2.85059	0.004364	0.009412	ATP1A1	protein_cc
ENSG0000(	22825.44	-0.03681	0.048414	-0.76032	0.447065	0.543857	RPL11	protein_cc
ENSG0000(	22760.07	-0.34511	0.067465	-5.11534	3.13E-07	1.37E-06	GSN	protein_cc
ENSG0000(	22530.44	0.17774	0.05068	3.507068	0.000453	0.001179	RPL28	protein_cc
ENSG0000(	22434.05	0.009484	0.050983	0.186018	0.852431	0.893312	SOX2	protein_cc
ENSG0000(	22422.44	-0.17814	0.033792	-5.27166	1.35E-07	6.27E-07	PRRC2A	protein_cc
ENSG0000(	22409.11	0.502837	0.063424	7.928193	2.22E-15	3.63E-14	RPS5	protein_cc
ENSG0000(	22387.4	-0.05586	0.069594	-0.80271	0.422143	0.519145	TCF12	protein_cc
ENSG0000(	22266.54	-0.20759	0.057402	-3.61647	0.000299	0.000804	DTNA	protein_cc
ENSG0000(	22227.22	0.155255	0.090195	1.721327	0.085192	0.13439	CD99	protein_cc
ENSG0000(	22099.45	-0.36542	0.070249	-5.20181	1.97E-07	8.92E-07	VCAN	protein_cc
ENSG0000(	22072.29	0.420616	0.087478	4.808263	1.52E-06	5.98E-06	MT2A	protein_cc
ENSG0000(	22051.58	-0.18001	0.039592	-4.54661	5.45E-06	1.96E-05	ATN1	protein_cc
ENSG0000(	22035.87	-0.5969	0.0726	-8.2218	2.00E-16	3.88E-15	NDRG4	protein_cc
ENSG0000(	21919.15	-0.49164	0.055557	-8.8494	8.80E-19	2.46E-17	KIF1B	protein_cc
ENSG0000(	21878.45	-0.07541	0.042155	-1.78899	0.073616	0.118415	FHL1	protein_cc
ENSG0000(	21784.74	0.132036	0.050054	2.637872	0.008343	0.016975	HSPA5	protein_cc
ENSG0000(	21575.06	0.340521	0.089808	3.791664	0.00015	0.000424	IGFBP7	protein_cc
ENSG0000(	21542.02	-0.00101	0.056519	-0.01792	0.985699	0.990218	CTSD	protein_cc
ENSG0000(	21509.51	-0.32789	0.041401	-7.91989	2.38E-15	3.85E-14	MAP4K4	protein_cc
ENSG0000(	21382.44	0.199321	0.046395	4.296138	1.74E-05	5.78E-05	RPS27A	protein_cc
ENSG0000(	21349.26	0.099007	0.027372	3.617132	0.000298	0.000802	HNRNPC	protein_cc
ENSG0000(	21258	0.140884	0.026261	5.364737	8.11E-08	3.89E-07	RAC1	protein_cc
ENSG0000(	21189.13	-0.3525	0.058139	-6.06309	1.34E-09	8.53E-09	TTC3	protein_cc
ENSG0000(	21182.99	-0.17718	0.062735	-2.82421	0.00474	0.010139	APC2	protein_cc
ENSG0000(	21162.77	-0.03527	0.025541	-1.38108	0.167253	0.239984	HNRNPU	protein_cc
ENSG0000(	21007.09	-0.25065	0.039792	-6.29899	3.00E-10	2.12E-09	ATP2A2	protein_cc
ENSG0000(	20963.22	-0.66603	0.077307	-8.61541	6.97E-18	1.72E-16	IDS	protein_cc
ENSG0000(	20896.22	-0.24708	0.050752	-4.8684	1.13E-06	4.51E-06	PFN2	protein_cc
ENSG0000(	20822.9	0.634213	0.072612	8.734225	2.45E-18	6.45E-17	RPS3A	protein_cc
ENSG0000(	20623.38	0.306934	0.052786	5.814646	6.08E-09	3.49E-08	RPL27	protein_cc
ENSG0000(	20606.17	-0.20526	0.03793	-5.4116	6.25E-08	3.06E-07	FKBP8	protein_cc
ENSG0000(	20604.39	0.283665	0.055176	5.141065	2.73E-07	1.21E-06	YBX1	protein_cc
ENSG0000(	20585.29	0.296659	0.040984	7.238408	4.54E-13	4.96E-12	SRI	protein_cc
ENSG0000(	20507.37	0.006294	0.048541	0.129662	0.896834	0.926855	RPS17L	protein_cc
ENSG0000(	20484.03	0.061072	0.04319	1.414045	0.157349	0.227813	SLC25A6	protein_cc
ENSG0000(	20385.29	-0.18132	0.033319	-5.44186	5.27E-08	2.61E-07	CSDE1	protein_cc
ENSG0000(	20367.9	-0.15411	0.038553	-3.99725	6.41E-05	0.000194	EIF4A2	protein_cc
ENSG0000(	20338.15	0.073909	0.033649	2.196438	0.028061	0.050493	SKP1	protein_cc
ENSG0000(	20297.97	-0.11719	0.032249	-3.63396	0.000279	0.000755	SCARB2	protein_cc
ENSG0000(	20192.36	-0.3141	0.04744	-6.62086	3.57E-11	2.92E-10	MACF1	protein_cc
ENSG0000(	20116.94	-0.29081	0.050061	-5.80916	6.28E-09	3.60E-08	PYGB	protein_cc

ENSG0000(	20116.24	0.36208	0.064656	5.600111	2.14E-08	1.13E-07	PGK1	protein_cc
ENSG0000(	20112.18	0.008123	0.023635	0.34367	0.731094	0.7961	UBA1	protein_cc
ENSG0000(	20096.64	0.655995	0.061862	10.60416	2.85E-26	3.21E-24	PPIA	protein_cc
ENSG0000(	20071.26	0.005552	0.036575	0.151803	0.879343	0.913799	SNRNP20(	protein_cc
ENSG0000(	19972.94	0.037418	0.031161	1.200801	0.229829	0.313783	FUS	protein_cc
ENSG0000(	19959.52	-0.01493	0.030588	-0.48825	0.625373	0.707447	CTNNB1	protein_cc
ENSG0000(	19926.29	-0.3134	0.041447	-7.56158	3.98E-14	5.18E-13	WBP2	protein_cc
ENSG0000(	19924	-0.26603	0.062211	-4.27632	1.90E-05	6.28E-05	TSPAN7	protein_cc
ENSG0000(	19848.87	0.190472	0.049496	3.848202	0.000119	0.000343	RPS27	protein_cc
ENSG0000(	19838.21	0.225266	0.052638	4.279548	1.87E-05	6.19E-05	PTMS	protein_cc
ENSG0000(	19797.35	0.041335	0.047102	0.877573	0.380176	0.476715	RPS25	protein_cc
ENSG0000(	19791.92	-0.11844	0.040057	-2.95682	0.003108	0.006906	FYN	protein_cc
ENSG0000(	19725.03	-0.18591	0.032842	-5.66073	1.51E-08	8.13E-08	YWHAB	protein_cc
ENSG0000(	19683.12	-0.3555	0.048465	-7.33517	2.21E-13	2.54E-12	FAM168A	protein_cc
ENSG0000(	19673.29	0.284182	0.050091	5.673289	1.40E-08	7.60E-08	RPL24	protein_cc
ENSG0000(	19647.21	0.246107	0.0395	6.230485	4.65E-10	3.19E-09	UBA52	protein_cc
ENSG0000(	19604.75	0.212524	0.059273	3.585521	0.000336	0.000897	TUBA1B	protein_cc
ENSG0000(	19602.23	0.716799	0.073077	9.808819	1.03E-22	5.69E-21	ANXA5	protein_cc
ENSG0000(	19458.9	0.437318	0.06607	6.619019	3.62E-11	2.95E-10	RPL9	protein_cc
ENSG0000(	19430.8	0.16007	0.032419	4.937539	7.91E-07	3.25E-06	YWHAQ	protein_cc
ENSG0000(	19421.16	0.17693	0.043784	4.041021	5.32E-05	0.000163	OAZ1	protein_cc
ENSG0000(	19409.64	-0.78698	0.081154	-9.69737	3.09E-22	1.56E-20	FAIM2	protein_cc
ENSG0000(	19375.66	-0.25234	0.044151	-5.7153	1.10E-08	6.04E-08	GOLM1	protein_cc
ENSG0000(	19295.37	-0.09998	0.048228	-2.07313	0.038161	0.066573	SCRN1	protein_cc
ENSG0000(	19282.26	-0.36655	0.047971	-7.64099	2.16E-14	2.94E-13	CLIP3	protein_cc
ENSG0000(	19244.88	0.234797	0.052178	4.499888	6.80E-06	2.41E-05	RPL29	protein_cc
ENSG0000(	19242.95	-0.32896	0.067596	-4.8666	1.14E-06	4.55E-06	LRRC8A	protein_cc
ENSG0000(	19092.18	0.158889	0.052672	3.016591	0.002556	0.005784	TUBB2B	protein_cc
ENSG0000(	19082.54	0.123884	0.031942	3.878427	0.000105	0.000306	NONO	protein_cc
ENSG0000(	19034.12	-0.04278	0.032372	-1.32144	0.186354	0.262921	PPP2R1A	protein_cc
ENSG0000(	19000.24	-0.87036	0.108868	-7.9946	1.30E-15	2.21E-14	KIF5A	protein_cc
ENSG0000(	18909.72	0.501396	0.106727	4.697932	2.63E-06	9.94E-06	TNC	protein_cc
ENSG0000(	18832.32	0.019131	0.04456	0.429317	0.667693	0.74356	CLIP2	protein_cc
ENSG0000(	18829.66	-0.57849	0.068233	-8.47813	2.29E-17	5.19E-16	ATP9A	protein_cc
ENSG0000(	18816.68	-0.02027	0.029419	-0.68901	0.49082	0.585244	CCNI	protein_cc
ENSG0000(	18797	-0.24954	0.054375	-4.58931	4.45E-06	1.62E-05	SLC22A17	protein_cc
ENSG0000(	18762.4	-0.06497	0.055363	-1.17345	0.240616	0.326013	TXNIP	protein_cc
ENSG0000(	18658.33	-0.38951	0.050244	-7.75237	9.02E-15	1.33E-13	ARNT2	protein_cc
ENSG0000(	18614.47	-0.10693	0.072324	-1.47852	0.139268	0.205215	PCDHGC3	protein_cc
ENSG0000(	18596.13	-0.15323	0.030725	-4.98708	6.13E-07	2.56E-06	DAZAP2	protein_cc
ENSG0000(	18582.68	0.217335	0.063339	3.431268	0.000601	0.001527	CLIC4	protein_cc
ENSG0000(	18551.92	0.27013	0.049955	5.407502	6.39E-08	3.12E-07	PTMA	protein_cc
ENSG0000(	18503.42	-0.09458	0.026483	-3.57148	0.000355	0.000943	HDLBP	protein_cc
ENSG0000(	18411.61	-0.96991	0.107489	-9.02334	1.82E-19	5.73E-18	DAAM2	protein_cc
ENSG0000(	18376.18	-0.42364	0.057646	-7.34901	2.00E-13	2.30E-12	YWHAG	protein_cc
ENSG0000(	18375.05	-0.08656	0.05527	-1.56611	0.117324	0.177305	STMN1	protein_cc
ENSG0000(	18353.59	-0.09722	0.087684	-1.10871	0.267554	0.356622	CSRP1	protein_cc
ENSG0000(	18316.12	-0.10894	0.023364	-4.66271	3.12E-06	1.17E-05	RAB7A	protein_cc
ENSG0000(	18297.43	-0.60023	0.109436	-5.48477	4.14E-08	2.09E-07	TF	protein_cc
ENSG0000(	18258.11	0.075621	0.039174	1.93037	0.053561	0.08975	RPL14	protein_cc
ENSG0000(	18198.53	0.008368	0.063011	0.132799	0.894353	0.924957	RGMA	protein_cc
ENSG0000(	18192.46	-0.83552	0.100565	-8.30832	9.71E-17	1.98E-15	TUBB4A	protein_cc
ENSG0000(	18154.04	-0.34551	0.037371	-9.24517	2.35E-20	8.46E-19	ARF3	protein_cc
ENSG0000(	18096.42	-0.49708	0.076202	-6.52318	6.88E-11	5.38E-10	ABCA2	protein_cc
ENSG0000(	18052.62	-0.32713	0.075436	-4.33645	1.45E-05	4.87E-05	ID4	protein_cc
ENSG0000(	17950.64	-0.19896	0.027224	-7.30831	2.71E-13	3.06E-12	DDB1	protein_cc
ENSG0000(	17928.45	0.226018	0.107536	2.101793	0.035571	0.062525	CD44	protein_cc
ENSG0000(	17925.39	0.209569	0.049874	4.202004	2.65E-05	8.52E-05	RPL12	protein_cc
ENSG0000(	17886.18	0.155849	0.045355	3.436219	0.00059	0.001502	FAU	protein_cc

ENSG0000	17867.54	-0.02622	0.027184	-0.96445	0.334821	0.429535	ARF1	protein_cc
ENSG0000	17807.13	-0.5391	0.091022	-5.92273	3.17E-09	1.90E-08	EDNRB	protein_cc
ENSG0000	17761.3	-0.12204	0.031683	-3.85192	0.000117	0.000338	ADAR	protein_cc
ENSG0000	17739.63	0.154888	0.032671	4.740791	2.13E-06	8.17E-06	ARL6IP1	protein_cc
ENSG0000	17718.88	-0.47037	0.074978	-6.27349	3.53E-10	2.47E-09	APLP1	protein_cc
ENSG0000	17568.11	0.216248	0.050018	4.323408	1.54E-05	5.14E-05	RPL10A	protein_cc
ENSG0000	17567.3	0.139556	0.063029	2.214168	0.026817	0.048516	PON2	protein_cc
ENSG0000	17522.51	-0.17861	0.041937	-4.25895	2.05E-05	6.75E-05	USP11	protein_cc
ENSG0000	17510.48	-0.7009	0.062631	-11.1909	4.52E-29	8.64E-27	SORL1	protein_cc
ENSG0000	17481.87	-0.26285	0.037296	-7.04772	1.82E-12	1.82E-11	GRINA	protein_cc
ENSG0000	17470.91	0.720065	0.073214	9.835062	7.95E-23	4.49E-21	OS9	protein_cc
ENSG0000	17467.7	-0.04682	0.031517	-1.48552	0.137405	0.202801	SF3B2	protein_cc
ENSG0000	17459.02	-0.05577	0.032931	-1.69366	0.09033	0.141471	PRPF8	protein_cc
ENSG0000	17303.63	-0.47477	0.047793	-9.93388	2.97E-23	1.83E-21	PRNP	protein_cc
ENSG0000	17270.28	1.003006	0.078416	12.7908	1.85E-37	1.85E-34	CHCHD2	protein_cc
ENSG0000	17224.54	-0.12178	0.035584	-3.42238	0.000621	0.001573	ST13	protein_cc
ENSG0000	17183.71	-0.6712	0.090933	-7.3812	1.57E-13	1.85E-12	RTN1	protein_cc
ENSG0000	17176.74	0.106299	0.073767	1.441	0.149585	0.218254	PHLDA1	protein_cc
ENSG0000	17060.48	0.201126	0.030686	6.554292	5.59E-11	4.42E-10	GANAB	protein_cc
ENSG0000	17002.58	-0.08951	0.043751	-2.04588	0.040768	0.070568	ATP6V0E2	protein_cc
ENSG0000	16963.45	-0.03773	0.02782	-1.35633	0.174994	0.24941	HSPA9	protein_cc
ENSG0000	16936.79	-0.09803	0.085888	-1.1414	0.253704	0.341136	TNK2	protein_cc
ENSG0000	16936.45	0.22224	0.045576	4.876264	1.08E-06	4.34E-06	9-Sep	protein_cc
ENSG0000	16927.28	-0.15981	0.075952	-2.10416	0.035365	0.062202	PLTP	protein_cc
ENSG0000	16899.85	-0.26043	0.055022	-4.73313	2.21E-06	8.47E-06	DCLK2	protein_cc
ENSG0000	16882.19	-0.00668	0.089312	-0.07479	0.940378	0.958417	OLIG2	protein_cc
ENSG0000	16862.39	0.099564	0.115195	0.864313	0.387416	0.484096	SEZ6L	protein_cc
ENSG0000	16857.33	-0.32593	0.043472	-7.49758	6.50E-14	8.20E-13	DENND5A	protein_cc
ENSG0000	16770.12	-0.36026	0.072587	-4.9631	6.94E-07	2.88E-06	KCNJ10	protein_cc
ENSG0000	16766.62	0.23095	0.046853	4.929217	8.26E-07	3.38E-06	P4HB	protein_cc
ENSG0000	16747.12	0.001333	0.031678	0.042089	0.966428	0.97687	NDFIP1	protein_cc
ENSG0000	16709.54	0.091709	0.036032	2.545188	0.010922	0.021638	CTNNA1	protein_cc
ENSG0000	16704.68	-0.24031	0.031974	-7.5157	5.66E-14	7.18E-13	MTCH1	protein_cc
ENSG0000	16680.16	0.24745	0.03888	6.364402	1.96E-10	1.42E-09	SEC61A1	protein_cc
ENSG0000	16620.07	0.077545	0.061691	1.256986	0.208759	0.289554	METTL7A	protein_cc
ENSG0000	16586.3	-0.7617	0.088616	-8.59549	8.29E-18	2.01E-16	CNTN1	protein_cc
ENSG0000	16537.25	0.300577	0.041236	7.289226	3.12E-13	3.50E-12	PPP1CB	protein_cc
ENSG0000	16513.21	0.04256	0.027126	1.569004	0.116647	0.17647	ILF3	protein_cc
ENSG0000	16480.83	-0.26948	0.041589	-6.47961	9.20E-11	7.05E-10	SPAG9	protein_cc
ENSG0000	16470.47	0.042916	0.025781	1.664619	0.095989	0.149123	CNBP	protein_cc
ENSG0000	16457.76	-0.18289	0.068911	-2.65399	0.007955	0.016262	PBXIP1	protein_cc
ENSG0000	16432.47	0.014891	0.026083	0.570931	0.568047	0.656042	HNRNPDL	protein_cc
ENSG0000	16372.55	-0.32298	0.039694	-8.13684	4.06E-16	7.48E-15	HUWE1	protein_cc
ENSG0000	16351.04	-0.33557	0.072219	-4.64657	3.38E-06	1.25E-05	DKK3	protein_cc
ENSG0000	16333.7	0.446752	0.055469	8.054153	8.00E-16	1.40E-14	RPL35	protein_cc
ENSG0000	16329.7	0.412989	0.056909	7.25699	3.96E-13	4.37E-12	RPS7	protein_cc
ENSG0000	16327.58	0.246944	0.043107	5.728557	1.01E-08	5.61E-08	RPL23A	protein_cc
ENSG0000	16307.78	-0.5135	0.064282	-7.98832	1.37E-15	2.32E-14	ENO2	protein_cc
ENSG0000	16229.74	-0.60743	0.047879	-12.6868	7.00E-37	6.25E-34	ABR	protein_cc
ENSG0000	16198.53	-1.24979	0.139794	-8.94027	3.88E-19	1.15E-17	SNAP25	protein_cc
ENSG0000	16182.19	-0.1663	0.030053	-5.53366	3.14E-08	1.61E-07	USP22	protein_cc
ENSG0000	16180.97	0.047356	0.026658	1.776405	0.075666	0.121266	SLC25A3	protein_cc
ENSG0000	16171.6	-0.18321	0.058116	-3.1525	0.001619	0.003801	SERPINE2	protein_cc
ENSG0000	16137.58	-0.40943	0.044218	-9.25923	2.06E-20	7.48E-19	PTPN11	protein_cc
ENSG0000	16111.07	0.293759	0.083382	3.523033	0.000427	0.001116	ETV1	protein_cc
ENSG0000	16099.96	0.198981	0.046247	4.302577	1.69E-05	5.62E-05	AES	protein_cc
ENSG0000	16088.77	0.038464	0.039741	0.967886	0.333101	0.427623	COX4I1	protein_cc
ENSG0000	16073.47	-0.20903	0.052825	-3.95708	7.59E-05	0.000226	NUMA1	protein_cc
ENSG0000	16071.73	-0.20673	0.039385	-5.24907	1.53E-07	7.03E-07	TLN1	protein_cc

ENSG0000(	16011.61	0.199624	0.049879	4.002128	6.28E-05	0.00019	RPS13	protein_cc
ENSG0000(	16003.08	0.305742	0.052043	5.874781	4.23E-09	2.49E-08	PFN1	protein_cc
ENSG0000(	16000.91	-0.37142	0.044167	-8.40936	4.12E-17	8.93E-16	WNK1	protein_cc
ENSG0000(	15995.2	-0.41353	0.052598	-7.86208	3.78E-15	5.94E-14	SNN	protein_cc
ENSG0000(	15973.29	-0.097	0.043578	-2.22593	0.026019	0.047213	CADM4	protein_cc
ENSG0000(	15970.27	0.005202	0.031485	0.165227	0.868766	0.905768	MLF2	protein_cc
ENSG0000(	15958.72	-0.35045	0.074135	-4.72721	2.28E-06	8.70E-06	SULF2	protein_cc
ENSG0000(	15917.15	-0.17535	0.047158	-3.71833	0.000201	0.000556	NCOR2	protein_cc
ENSG0000(	15751.77	-0.33323	0.037931	-8.78514	1.56E-18	4.21E-17	NFE2L1	protein_cc
ENSG0000(	15709.84	-0.2717	0.062116	-4.37401	1.22E-05	4.15E-05	EZR	protein_cc
ENSG0000(	15694.04	-0.18599	0.06327	-2.93965	0.003286	0.007267	PIK3R1	protein_cc
ENSG0000(	15671.43	-0.31693	0.071198	-4.45134	8.53E-06	2.98E-05	RND2	protein_cc
ENSG0000(	15601.33	0.100622	0.049935	2.015053	0.043899	0.075309	RPL34	protein_cc
ENSG0000(	15591.54	-0.12239	0.035715	-3.42678	0.000611	0.00155	CHD4	protein_cc
ENSG0000(	15581.97	0.014102	0.118528	0.118973	0.905297	0.932952	PDGFRA	protein_cc
ENSG0000(	15575.48	-0.62592	0.075373	-8.30429	1.00E-16	2.04E-15	3-Sep	protein_cc
ENSG0000(	15561.34	-0.27593	0.035203	-7.83834	4.57E-15	7.07E-14	AARS	protein_cc
ENSG0000(	15545.44	0.034607	0.03327	1.040184	0.298255	0.390544	MTPN	protein_cc
ENSG0000(	15522.98	0.143654	0.026295	5.463145	4.68E-08	2.34E-07	SET	protein_cc
ENSG0000(	15471.22	-0.18132	0.051046	-3.55212	0.000382	0.001009	MYH9	protein_cc
ENSG0000(	15436.64	0.123962	0.037724	3.286001	0.001016	0.002473	TMED10	protein_cc
ENSG0000(	15377.08	0.399508	0.096633	4.134256	3.56E-05	0.000112	GAP43	protein_cc
ENSG0000(	15373.25	0.182983	0.084981	2.153234	0.0313	0.055718	CSPG5	protein_cc
ENSG0000(	15361.65	-0.20121	0.03435	-5.8578	4.69E-09	2.74E-08	ARPP19	protein_cc
ENSG0000(	15291.43	-0.41696	0.038753	-10.7596	5.34E-27	6.93E-25	MPRIP	protein_cc
ENSG0000(	15278.96	0.178535	0.040876	4.367662	1.26E-05	4.27E-05	NPM1	protein_cc
ENSG0000(	15187.46	-0.3831	0.080098	-4.78287	1.73E-06	6.73E-06	SLC4A4	protein_cc
ENSG0000(	15162.27	-0.04468	0.053816	-0.83029	0.406375	0.503124	SOX2-OT	sense_ove
ENSG0000(	15151.61	-0.5019	0.056708	-8.85051	8.71E-19	2.44E-17	ANK2	protein_cc
ENSG0000(	15143.98	-0.35867	0.064035	-5.60119	2.13E-08	1.12E-07	MTSS1L	protein_cc
ENSG0000(	15110.75	-0.88807	0.104012	-8.53818	1.36E-17	3.20E-16	ATP1A3	protein_cc
ENSG0000(	15090.69	-0.15763	0.048037	-3.2815	0.001033	0.002509	TPP1	protein_cc
ENSG0000(	15086	-0.14842	0.044147	-3.36197	0.000774	0.001925	EIF4B	protein_cc
ENSG0000(	15051.67	-0.20256	0.066463	-3.04775	0.002306	0.005258	RASSF4	protein_cc
ENSG0000(	15043.8	0.174097	0.028873	6.029761	1.64E-09	1.03E-08	HMGB1	protein_cc
ENSG0000(	14987.81	-0.13094	0.048792	-2.68355	0.007285	0.015005	CD59	protein_cc
ENSG0000(	14985.98	-0.1342	0.038697	-3.46783	0.000525	0.001348	SLC44A2	protein_cc
ENSG0000(	14964.72	0.073767	0.08428	0.875259	0.381433	0.478044	GPR37L1	protein_cc
ENSG0000(	14934.68	-0.2423	0.053107	-4.56248	5.06E-06	1.83E-05	DGCR2	protein_cc
ENSG0000(	14907.36	0.286044	0.054149	5.282538	1.27E-07	5.94E-07	RPS15A	protein_cc
ENSG0000(	14898.87	0.038775	0.032696	1.185924	0.235652	0.320424	SFPQ	protein_cc
ENSG0000(	14873.99	-0.29876	0.061302	-4.87361	1.10E-06	4.40E-06	FASN	protein_cc
ENSG0000(	14801.4	-0.25117	0.037965	-6.61581	3.70E-11	3.01E-10	TMEM30A	protein_cc
ENSG0000(	14796.15	-0.05928	0.027593	-2.1484	0.031682	0.056329	EIF4G1	protein_cc
ENSG0000(	14791.19	0.124673	0.034534	3.61011	0.000306	0.000822	BTF3	protein_cc
ENSG0000(	14783.18	0.158445	0.036104	4.388556	1.14E-05	3.90E-05	PRDX2	protein_cc
ENSG0000(	14740.2	-0.36331	0.057276	-6.34324	2.25E-10	1.62E-09	SIRT2	protein_cc
ENSG0000(	14707.92	-0.06437	0.036843	-1.74703	0.080633	0.128113	CBX5	protein_cc
ENSG0000(	14691	-0.22629	0.049855	-4.53893	5.65E-06	2.03E-05	SORT1	protein_cc
ENSG0000(	14689.99	0.073352	0.028083	2.612019	0.009001	0.018166	C6orf62	protein_cc
ENSG0000(	14637.97	0.657161	0.098745	6.65515	2.83E-11	2.35E-10	S100A6	protein_cc
ENSG0000(	14627.78	-0.02794	0.039661	-0.70444	0.481156	0.576479	MAT2A	protein_cc
ENSG0000(	14604.82	-0.01906	0.059405	-0.32088	0.748303	0.810357	WLS	protein_cc
ENSG0000(	14596.43	0.058019	0.032571	1.781283	0.074866	0.120161	CCT3	protein_cc
ENSG0000(	14592.45	-0.63133	0.103033	-6.1275	8.93E-10	5.87E-09	APOD	protein_cc
ENSG0000(	14591.64	-0.00646	0.089716	-0.07206	0.942557	0.959828	LAPTM5	protein_cc
ENSG0000(	14567.91	0.038317	0.03435	1.115461	0.264653	0.353398	MORF4L1	protein_cc
ENSG0000(	14564.34	-0.00758	0.045697	-0.16588	0.868254	0.90551	LTBP3	protein_cc
ENSG0000(	14564.07	0.262595	0.068588	3.828571	0.000129	0.000369	LGALS3BP	protein_cc

ENSG0000(	14547.91	0.090032	0.056094	1.605003	0.108493	0.165725	SEPW1	protein_cc
ENSG0000(	14528.55	-0.00996	0.045445	-0.21906	0.826603	0.872875	MCL1	protein_cc
ENSG0000(	14526.16	-0.495	0.06016	-8.22808	1.90E-16	3.69E-15	VAMP2	protein_cc
ENSG0000(	14524.16	0.027972	0.045144	0.619608	0.535516	0.626709	TCF4	protein_cc
ENSG0000(	14523.21	-0.02945	0.103969	-0.28321	0.777014	0.833523	C1QB	protein_cc
ENSG0000(	14509.96	-0.10672	0.030394	-3.5113	0.000446	0.001163	SON	protein_cc
ENSG0000(	14507.96	-0.32036	0.053775	-5.95745	2.56E-09	1.56E-08	NSMF	protein_cc
ENSG0000(	14505.75	0.23111	0.049072	4.70957	2.48E-06	9.43E-06	RPL35A	protein_cc
ENSG0000(	14505.53	0.145524	0.037779	3.851994	0.000117	0.000338	SLC3A2	protein_cc
ENSG0000(	14502.27	-0.11432	0.035554	-3.21548	0.001302	0.003108	NGFRAP1	protein_cc
ENSG0000(	14500.89	0.523722	0.070038	7.477657	7.57E-14	9.41E-13	CCND2	protein_cc
ENSG0000(	14481.07	-0.57335	0.066044	-8.68133	3.91E-18	9.96E-17	GNAO1	protein_cc
ENSG0000(	14472.76	-0.64197	0.066703	-9.62426	6.32E-22	2.99E-20	ALDH2	protein_cc
ENSG0000(	14467.32	0.24613	0.052685	4.671758	2.99E-06	1.12E-05	SERF2	protein_cc
ENSG0000(	14451.88	0.419857	0.063441	6.618112	3.64E-11	2.97E-10	RPL18A	protein_cc
ENSG0000(	14428.59	-0.04343	0.031729	-1.36886	0.171041	0.244607	DDX3X	protein_cc
ENSG0000(	14419.66	-0.2652	0.058507	-4.53271	5.82E-06	2.09E-05	ZMIZ1	protein_cc
ENSG0000(	14412.58	-0.09846	0.038293	-2.57114	0.010136	0.020215	LAMP1	protein_cc
ENSG0000(	14406.92	0.017693	0.027822	0.635938	0.524817	0.616733	EID1	protein_cc
ENSG0000(	14390.11	-0.13976	0.035539	-3.93267	8.40E-05	0.000249	TOMM20	protein_cc
ENSG0000(	14367.39	-0.21069	0.041306	-5.10078	3.38E-07	1.47E-06	PJA2	protein_cc
ENSG0000(	14337.36	-0.01817	0.033527	-0.54188	0.587904	0.674148	HNRNPUL	protein_cc
ENSG0000(	14334.16	0.158354	0.026227	6.037855	1.56E-09	9.87E-09	PCBP2	protein_cc
ENSG0000(	14325.44	-0.11217	0.050994	-2.19961	0.027835	0.050135	NLRP1	protein_cc
ENSG0000(	14322.37	-0.18425	0.052454	-3.51257	0.000444	0.001158	H1F0	protein_cc
ENSG0000(	14313.55	0.558887	0.097289	5.744579	9.21E-09	5.13E-08	MSN	protein_cc
ENSG0000(	14313.33	0.237724	0.037014	6.422516	1.34E-10	1.00E-09	EIF5A	protein_cc
ENSG0000(	14300.31	-0.13546	0.054132	-2.50247	0.012333	0.02412	PREX1	protein_cc
ENSG0000(	14274.47	-0.36565	0.035976	-10.1637	2.88E-24	2.17E-22	MAPK1	protein_cc
ENSG0000(	14261.9	-0.63287	0.059665	-10.6071	2.76E-26	3.12E-24	SORBS1	protein_cc
ENSG0000(	14254.11	0.146972	0.027133	5.416804	6.07E-08	2.98E-07	PTGES3	protein_cc
ENSG0000(	14217.25	0.362636	0.053361	6.795846	1.08E-11	9.50E-11	RPL36	protein_cc
ENSG0000(	14161.62	0.06314	0.089676	0.70409	0.481377	0.576676	HES6	protein_cc
ENSG0000(	14149.67	-0.08891	0.023744	-3.7444	0.000181	0.000505	VCP	protein_cc
ENSG0000(	14114.56	0.005768	0.082565	0.069858	0.944307	0.961039	LRP4	protein_cc
ENSG0000(	14113.85	-0.02791	0.024477	-1.14007	0.254259	0.341804	SF3B1	protein_cc
ENSG0000(	14103.11	-0.01561	0.053389	-0.29233	0.770035	0.827677	GPI	protein_cc
ENSG0000(	14100.16	-0.07826	0.053809	-1.45434	0.145853	0.213529	MYO10	protein_cc
ENSG0000(	14066.05	-0.04446	0.033504	-1.32701	0.184505	0.260634	PARP1	protein_cc
ENSG0000(	14037.55	-0.05925	0.025009	-2.36894	0.017839	0.033575	KPNB1	protein_cc
ENSG0000(	13962.07	0.449308	0.054696	8.214672	2.13E-16	4.09E-15	RPS15	protein_cc
ENSG0000(	13961.01	-0.05035	0.061857	-0.81395	0.415674	0.512677	ATP2B4	protein_cc
ENSG0000(	13930.4	-0.11388	0.043022	-2.64715	0.008117	0.016558	LAMP2	protein_cc
ENSG0000(	13930.19	-0.24889	0.046719	-5.3273	9.97E-08	4.73E-07	8-Sep	protein_cc
ENSG0000(	13905.95	0.039896	0.02592	1.539211	0.123753	0.185514	EIF4H	protein_cc
ENSG0000(	13903.22	-0.26355	0.069918	-3.76945	0.000164	0.000461	NRXN2	protein_cc
ENSG0000(	13876.02	-0.05179	0.052478	-0.98687	0.323706	0.41787	CRMP1	protein_cc
ENSG0000(	13851.52	0.087396	0.049808	1.754639	0.079321	0.126336	ITGAV	protein_cc
ENSG0000(	13841.51	0.065264	0.089537	0.728912	0.466056	0.56189	COL6A1	protein_cc
ENSG0000(	13803.39	-0.03432	0.024322	-1.41115	0.1582	0.228772	SF1	protein_cc
ENSG0000(	13789.22	-0.26208	0.031193	-8.40189	4.39E-17	9.47E-16	RAB5B	protein_cc
ENSG0000(	13777.74	0.178676	0.032287	5.534029	3.13E-08	1.61E-07	PRKCSH	protein_cc
ENSG0000(	13767.74	-0.26905	0.056218	-4.78584	1.70E-06	6.64E-06	PSAT1	protein_cc
ENSG0000(	13763.72	-0.27289	0.033657	-8.10788	5.15E-16	9.33E-15	PAFAH1B1	protein_cc
ENSG0000(	13724.94	-0.34906	0.03781	-9.23193	2.66E-20	9.46E-19	DYNLL2	protein_cc
ENSG0000(	13700.43	-0.39141	0.05577	-7.01822	2.25E-12	2.21E-11	SLC44A1	protein_cc
ENSG0000(	13683.11	0.568803	0.097604	5.827642	5.62E-09	3.24E-08	LDHA	protein_cc
ENSG0000(	13648.46	0.054603	0.053023	1.02979	0.303108	0.395816	TRIM9	protein_cc
ENSG0000(	13647.74	0.018705	0.032904	0.568474	0.569713	0.657565	DSTN	protein_cc

ENSG0000(	13626.19	-0.00567	0.0228	-0.24869	0.803602	0.85504	STARD7	protein_cc
ENSG0000(	13620.83	-0.06461	0.030334	-2.12999	0.033172	0.05871	GLG1	protein_cc
ENSG0000(	13611.14	-0.23025	0.111113	-2.07218	0.038249	0.066712	FOS	protein_cc
ENSG0000(	13584.9	-0.62608	0.0629	-9.95353	2.43E-23	1.55E-21	ABLIM1	protein_cc
ENSG0000(	13569.49	0.216486	0.06384	3.391071	0.000696	0.001746	FSCN1	protein_cc
ENSG0000(	13549.64	0.072235	0.053738	1.344203	0.178883	0.253975	HIF1A	protein_cc
ENSG0000(	13540.3	0.311283	0.039461	7.888306	3.06E-15	4.88E-14	GNB2	protein_cc
ENSG0000(	13537.6	0.007102	0.029729	0.238878	0.8112	0.861209	MAGED2	protein_cc
ENSG0000(	13529.3	-0.00513	0.052247	-0.09825	0.921732	0.944834	CDC42EP4	protein_cc
ENSG0000(	13492.27	-0.25834	0.06314	-4.0916	4.28E-05	0.000133	ZCCHC24	protein_cc
ENSG0000(	13489.65	-0.61365	0.076854	-7.98464	1.41E-15	2.38E-14	OGFRL1	protein_cc
ENSG0000(	13487.16	-0.02476	0.047902	-0.51697	0.605175	0.689358	PTPRS	protein_cc
ENSG0000(	13485.64	-0.73738	0.079122	-9.31946	1.17E-20	4.44E-19	KIF5C	protein_cc
ENSG0000(	13443.87	-0.28382	0.053635	-5.2918	1.21E-07	5.67E-07	PLXNB1	protein_cc
ENSG0000(	13375.97	-0.40081	0.039532	-10.1388	3.71E-24	2.74E-22	DCTN1	protein_cc
ENSG0000(	13367.79	-0.01328	0.026434	-0.50255	0.615281	0.698421	COPA	protein_cc
ENSG0000(	13363.02	0.012886	0.02618	0.492212	0.622569	0.704974	HNRNPA3	protein_cc
ENSG0000(	13359.86	0.018039	0.03381	0.533545	0.593657	0.679078	PUF60	protein_cc
ENSG0000(	13349.67	0.259533	0.030228	8.58571	9.03E-18	2.18E-16	HNRNPH1	protein_cc
ENSG0000(	13308.87	-0.64889	0.058889	-11.0189	3.10E-28	4.88E-26	ADD3	protein_cc
ENSG0000(	13292.93	0.339669	0.054249	6.261321	3.82E-10	2.65E-09	NDUFA4	protein_cc
ENSG0000(	13285.67	-0.32844	0.061709	-5.32238	1.02E-07	4.85E-07	PHGDH	protein_cc
ENSG0000(	13280.35	0.175755	0.046475	3.781724	0.000156	0.00044	RPL38	protein_cc
ENSG0000(	13270.22	-0.17571	0.034743	-5.0575	4.25E-07	1.82E-06	PRKAR1A	protein_cc
ENSG0000(	13261.42	0.015648	0.02581	0.606289	0.544323	0.634848	HSPD1	protein_cc
ENSG0000(	13259.01	-0.09169	0.031195	-2.93933	0.003289	0.007273	RAD21	protein_cc
ENSG0000(	13253.84	-0.09708	0.051263	-1.89377	0.058255	0.096569	SLC7A5	protein_cc
ENSG0000(	13252.58	-0.36262	0.053278	-6.80627	1.00E-11	8.89E-11	CLASP2	protein_cc
ENSG0000(	13222.25	-0.37967	0.127462	-2.97866	0.002895	0.006473	GPX3	protein_cc
ENSG0000(	13221.04	0.301946	0.067944	4.444044	8.83E-06	3.07E-05	GPX1	protein_cc
ENSG0000(	13220.06	0.019696	0.028937	0.680661	0.496086	0.590222	CAPRIN1	protein_cc
ENSG0000(	13201.65	-0.4195	0.056884	-7.37469	1.65E-13	1.93E-12	LSAMP	protein_cc
ENSG0000(	13182.78	0.11238	0.039277	2.861215	0.00422	0.009138	PPT1	protein_cc
ENSG0000(	13175.56	-0.42591	0.083807	-5.08211	3.73E-07	1.61E-06	PHYHIPL	protein_cc
ENSG0000(	13166.03	0.208246	0.063172	3.296473	0.000979	0.002388	OLFM2	protein_cc
ENSG0000(	13158.95	-0.08044	0.067036	-1.19989	0.230183	0.314163	SCARA3	protein_cc
ENSG0000(	13151.69	-0.19194	0.117536	-1.63305	0.102458	0.157772	EGR1	protein_cc
ENSG0000(	13148.54	0.069821	0.027033	2.582842	0.009799	0.019614	XRCC6	protein_cc
ENSG0000(	13147.79	-0.32331	0.048452	-6.67282	2.51E-11	2.10E-10	SIRPA	protein_cc
ENSG0000(	13129.4	-0.11875	0.109708	-1.08245	0.279052	0.369458	RP11-161	lincRNA
ENSG0000(	13117.54	-0.34574	0.06735	-5.1335	2.84E-07	1.25E-06	ITPKB	protein_cc
ENSG0000(	13069.81	0.547829	0.135469	4.043952	5.26E-05	0.000161	AEBP1	protein_cc
ENSG0000(	13057.16	-0.10392	0.039076	-2.65954	0.007825	0.016017	ZFAND5	protein_cc
ENSG0000(	13039.58	0.139671	0.036193	3.859072	0.000114	0.000329	CDK2AP1	protein_cc
ENSG0000(	12996.08	0.082577	0.025744	3.207661	0.001338	0.003189	XRCC5	protein_cc
ENSG0000(	12990.52	0.033544	0.040851	0.821123	0.411576	0.50846	WDR1	protein_cc
ENSG0000(	12962.19	0.094638	0.08798	1.075681	0.28207	0.37269	ID3	protein_cc
ENSG0000(	12958	-0.0071	0.04618	-0.15367	0.877873	0.912649	CORO1C	protein_cc
ENSG0000(	12957.64	0.16678	0.048111	3.466594	0.000527	0.001354	CAPNS1	protein_cc
ENSG0000(	12946.92	-0.03	0.030495	-0.9836	0.325313	0.419498	EIF5	protein_cc
ENSG0000(	12936.86	-0.6768	0.067018	-10.0988	5.59E-24	4.03E-22	TMOD2	protein_cc
ENSG0000(	12896.42	0.401681	0.065864	6.098626	1.07E-09	6.94E-09	GPC1	protein_cc
ENSG0000(	12889.84	-0.08892	0.026949	-3.29949	0.000969	0.002365	FGD5-AS1	antisense
ENSG0000(	12889.6	-0.05684	0.026354	-2.15675	0.031025	0.055284	EWSR1	protein_cc
ENSG0000(	12864.77	0.12497	0.054783	2.281175	0.022538	0.041489	FAM211A-	processed
ENSG0000(	12769.91	-0.43604	0.057055	-7.64238	2.13E-14	2.91E-13	ITPK1	protein_cc
ENSG0000(	12766.58	0.030783	0.041658	0.738936	0.459946	0.556187	CAP1	protein_cc
ENSG0000(	12744.45	0.221076	0.039949	5.533998	3.13E-08	1.61E-07	TRIM28	protein_cc
ENSG0000(	12743.46	-0.16411	0.031978	-5.13204	2.87E-07	1.26E-06	PTPRA	protein_cc

ENSG0000(	12743.14	-0.46461	0.099043	-4.69097	2.72E-06	1.03E-05	HSPB8		protein_cc
ENSG0000(	12734.8	-0.28898	0.05606	-5.15476	2.54E-07	1.13E-06	SREBF2		protein_cc
ENSG0000(	12734.36	-0.09667	0.049632	-1.94769	0.051452	0.086649	CSNK1E		protein_cc
ENSG0000(	12724.39	-0.06643	0.037101	-1.7906	0.073358	0.118055	IL6ST		protein_cc
ENSG0000(	12710.93	0.312326	0.05553	5.624498	1.86E-08	9.90E-08	RPS21		protein_cc
ENSG0000(	12697.03	0.003777	0.029996	0.125912	0.899802	0.928848	KHSRP		protein_cc
ENSG0000(	12678.55	-0.21869	0.039075	-5.59663	2.19E-08	1.15E-07	POLR2A		protein_cc
ENSG0000(	12656.62	-0.1754	0.031774	-5.52039	3.38E-08	1.73E-07	NCKAP1		protein_cc
ENSG0000(	12622.56	-0.2325	0.056146	-4.14104	3.46E-05	0.000109	PLD3		protein_cc
ENSG0000(	12612.33	-0.36775	0.053495	-6.87443	6.22E-12	5.71E-11	PRRC2B		protein_cc
ENSG0000(	12609.64	-0.11742	0.029404	-3.99339	6.51E-05	0.000197	HADHA		protein_cc
ENSG0000(	12603.11	-0.26223	0.070558	-3.71651	0.000202	0.000559	THY1		protein_cc
ENSG0000(	12601.17	0.295353	0.049914	5.917265	3.27E-09	1.96E-08	PFDN5		protein_cc
ENSG0000(	12583.36	-0.23678	0.033768	-7.01196	2.35E-12	2.31E-11	CYB5R3		protein_cc
ENSG0000(	12578.78	-0.22529	0.082743	-2.72276	0.006474	0.013475	UCHL1		protein_cc
ENSG0000(	12574.59	0.143006	0.046938	3.046699	0.002314	0.005275	ACTN4		protein_cc
ENSG0000(	12564.5	-0.2097	0.063399	-3.30767	0.000941	0.002303	CLDND1		protein_cc
ENSG0000(	12545.49	0.140009	0.026416	5.30017	1.16E-07	5.43E-07	CCT7		protein_cc
ENSG0000(	12538.82	-0.19922	0.056613	-3.51903	0.000433	0.001132	EPAS1		protein_cc
ENSG0000(	12527.33	-0.45376	0.082708	-5.4863	4.10E-08	2.07E-07	PDZD4		protein_cc
ENSG0000(	12496.51	0.125726	0.067744	1.855891	0.063469	0.104071	PRDX1		protein_cc
ENSG0000(	12494.75	-0.14947	0.081953	-1.82384	0.068176	0.110707	NDRG1		protein_cc
ENSG0000(	12491.39	0.106948	0.034713	3.080878	0.002064	0.004752	ATF4		protein_cc
ENSG0000(	12491.39	-0.04964	0.033745	-1.47098	0.141296	0.207768	UBE2H		protein_cc
ENSG0000(	12489.71	-0.19011	0.048952	-3.88351	0.000103	0.0003	RHOBTB3		protein_cc
ENSG0000(	12477.8	-0.17942	0.039195	-4.57746	4.71E-06	1.71E-05	ASAH1		protein_cc
ENSG0000(	12472.43	-0.09681	0.097354	-0.99436	0.320047	0.413835	C1QC		protein_cc
ENSG0000(	12448.92	0.221964	0.056672	3.916647	8.98E-05	0.000265	SEPN1		protein_cc
ENSG0000(	12447.29	-0.03543	0.057902	-0.61194	0.540577	0.631377	DPP6		protein_cc
ENSG0000(	12432.63	-0.2808	0.050424	-5.56865	2.57E-08	1.34E-07	NLGN2		protein_cc
ENSG0000(	12414.51	-0.2794	0.048856	-5.71888	1.07E-08	5.92E-08	MAPK8IP1		protein_cc
ENSG0000(	12404.13	0.075328	0.087073	0.865115	0.386976	0.483708	SOX4		protein_cc
ENSG0000(	12403.42	0.418189	0.080497	5.195094	2.05E-07	9.22E-07	GATM		protein_cc
ENSG0000(	12391.72	-0.01769	0.059865	-0.29544	0.767655	0.82568	NOTCH1		protein_cc
ENSG0000(	12377.25	-0.14153	0.05116	-2.76646	0.005667	0.011936	STMN3		protein_cc
ENSG0000(	12360.53	-0.1333	0.032827	-4.06065	4.89E-05	0.000151	RNF187		protein_cc
ENSG0000(	12332.68	-0.27852	0.038719	-7.19352	6.31E-13	6.76E-12	ANXA6		protein_cc
ENSG0000(	12332.08	0.181059	0.026468	6.840794	7.88E-12	7.10E-11	H2AFV		protein_cc
ENSG0000(	12301.05	-0.45888	0.05952	-7.70978	1.26E-14	1.79E-13	PFKFB3		protein_cc
ENSG0000(	12299.28	-0.21477	0.104402	-2.05718	0.039669	0.068886	LPL		protein_cc
ENSG0000(	12287.07	0.044554	0.093006	0.479048	0.631904	0.71313	HIP1R		protein_cc
ENSG0000(	12279.16	0.090116	0.074301	1.212841	0.225191	0.308514	JUN		protein_cc
ENSG0000(	12278.5	0.076456	0.054107	1.413049	0.157641	0.228147	AGRN		protein_cc
ENSG0000(	12251.36	-0.14457	0.036403	-3.97122	7.15E-05	0.000214	TOP2B		protein_cc
ENSG0000(	12240.38	-0.02984	0.024934	-1.19684	0.231369	0.315446	BRD2		protein_cc
ENSG0000(	12239.3	-0.03998	0.024942	-1.60296	0.108943	0.166314	BAG6		protein_cc
ENSG0000(	12205.32	-0.71505	0.075435	-9.47898	2.57E-21	1.10E-19	C14orf132		protein_cc
ENSG0000(	12202.09	0.285833	0.040804	7.005089	2.47E-12	2.42E-11	RPN2		protein_cc
ENSG0000(	12182.96	-0.07527	0.034385	-2.18912	0.028588	0.051367	SOD1		protein_cc
ENSG0000(	12171.09	-0.46643	0.060873	-7.66231	1.83E-14	2.52E-13	PDE4DIP		protein_cc
ENSG0000(	12114.19	-0.06452	0.1065	-0.6058	0.544646	0.635134	SLIT1		protein_cc
ENSG0000(	12107.31	-0.27914	0.058585	-4.76479	1.89E-06	7.32E-06	ZFP36L1		protein_cc
ENSG0000(	12086.96	0.134328	0.053185	2.525688	0.011547	0.022743	METRN		protein_cc
ENSG0000(	12070.97	-0.9163	0.084959	-10.7853	4.04E-27	5.34E-25	TJP2		protein_cc
ENSG0000(	12051.18	-0.22636	0.036458	-6.20877	5.34E-10	3.62E-09	REEP5		protein_cc
ENSG0000(	12019.09	-0.272	0.045517	-5.97582	2.29E-09	1.41E-08	ENAH		protein_cc
ENSG0000(	12006.53	-0.12003	0.061337	-1.95686	0.050364	0.085037	ZEB1		protein_cc
ENSG0000(	12005.48	-0.57108	0.071614	-7.97443	1.53E-15	2.56E-14	YWHAH		protein_cc
ENSG0000(	11988.28	-0.28037	0.060501	-4.63404	3.59E-06	1.33E-05	PPAP2B		protein_cc

ENSG0000(	11986.79	-0.11823	0.037566	-3.14712	0.001649	0.003864	CAPZB	protein_cc
ENSG0000(	11981.46	-0.08079	0.028543	-2.83037	0.004649	0.009963	PPP2CB	protein_cc
ENSG0000(	11980.06	0.078824	0.033812	2.331221	0.019742	0.036784	SRP14	protein_cc
ENSG0000(	11973.62	0.087743	0.025363	3.4595	0.000541	0.001387	PSMD2	protein_cc
ENSG0000(	11945.71	-0.19217	0.031323	-6.13506	8.51E-10	5.61E-09	AZIN1	protein_cc
ENSG0000(	11926.53	-0.26314	0.043174	-6.09495	1.09E-09	7.10E-09	FMNL2	protein_cc
ENSG0000(	11877.94	0.043488	0.059594	0.729738	0.465551	0.561479	SNRNP70	protein_cc
ENSG0000(	11870.22	-0.30113	0.034639	-8.69346	3.52E-18	9.02E-17	FAM168B	protein_cc
ENSG0000(	11845.87	0.103226	0.02724	3.789442	0.000151	0.000428	TUFM	protein_cc
ENSG0000(	11839.47	-0.15896	0.029841	-5.32691	9.99E-08	4.74E-07	TNPO2	protein_cc
ENSG0000(	11835.07	-0.2804	0.047047	-5.96004	2.52E-09	1.54E-08	ANTXR1	protein_cc
ENSG0000(	11826.42	0.779443	0.065459	11.9074	1.08E-32	4.20E-30	CCT6A	protein_cc
ENSG0000(	11824.44	-0.14094	0.043816	-3.21655	0.001297	0.003098	RPL22	protein_cc
ENSG0000(	11817.27	0.07251	0.038003	1.90802	0.056389	0.093862	GNA12	protein_cc
ENSG0000(	11785.79	-0.06403	0.039501	-1.62094	0.10503	0.161153	MSI2	protein_cc
ENSG0000(	11784.73	0.217795	0.039327	5.537985	3.06E-08	1.57E-07	SNX3	protein_cc
ENSG0000(	11784.29	0.126799	0.042747	2.966238	0.003015	0.006714	LAPTM4A	protein_cc
ENSG0000(	11774.98	-0.44911	0.056722	-7.91773	2.42E-15	3.91E-14	PRKCA	protein_cc
ENSG0000(	11717.83	0.130798	0.029288	4.465904	7.97E-06	2.80E-05	HDGF	protein_cc
ENSG0000(	11713.8	-0.34492	0.051577	-6.68741	2.27E-11	1.91E-10	CELSR2	protein_cc
ENSG0000(	11693.72	0.60798	0.067771	8.971055	2.94E-19	8.89E-18	TTYH3	protein_cc
ENSG0000(	11686.57	-0.37316	0.065406	-5.70538	1.16E-08	6.38E-08	RAP2A	protein_cc
ENSG0000(	11678.33	0.337988	0.062321	5.423336	5.85E-08	2.88E-07	CD9	protein_cc
ENSG0000(	11669.06	0.107284	0.041581	2.58011	0.009877	0.019757	DAG1	protein_cc
ENSG0000(	11649.14	-0.10433	0.03757	-2.7768	0.00549	0.011599	WDR6	protein_cc
ENSG0000(	11641.1	0.122858	0.039618	3.101084	0.001928	0.00446	FLOT1	protein_cc
ENSG0000(	11623.46	-0.16415	0.026623	-6.16592	7.01E-10	4.68E-09	ACTR2	protein_cc
ENSG0000(	11618.81	0.382551	0.061522	6.218156	5.03E-10	3.43E-09	CTDSP2	protein_cc
ENSG0000(	11594.15	0.245128	0.058028	4.224303	2.40E-05	7.77E-05	SOX9	protein_cc
ENSG0000(	11576.32	0.323357	0.03681	8.784422	1.57E-18	4.24E-17	SUB1	protein_cc
ENSG0000(	11566.17	0.108604	0.037537	2.893222	0.003813	0.008325	ATP5G2	protein_cc
ENSG0000(	11515.9	-0.30743	0.043126	-7.12853	1.01E-12	1.05E-11	PNMA1	protein_cc
ENSG0000(	11505.68	0.276633	0.073199	3.779165	0.000157	0.000444	SAT1	protein_cc
ENSG0000(	11504.23	-0.26463	0.070602	-3.74819	0.000178	0.000498	LIFR	protein_cc
ENSG0000(	11486.82	0.317461	0.06125	5.183025	2.18E-07	9.80E-07	RPL21	protein_cc
ENSG0000(	11484.29	-0.17017	0.035657	-4.77233	1.82E-06	7.07E-06	TKT	protein_cc
ENSG0000(	11471.61	0.24838	0.034785	7.140374	9.31E-13	9.73E-12	RAN	protein_cc
ENSG0000(	11469.8	0.22754	0.044139	5.155119	2.53E-07	1.13E-06	GSTP1	protein_cc
ENSG0000(	11441.54	0.18664	0.045571	4.095571	4.21E-05	0.000131	PDIA3	protein_cc
ENSG0000(	11435.46	-0.23776	0.035957	-6.61219	3.79E-11	3.08E-10	NBR1	protein_cc
ENSG0000(	11418.69	0.18097	0.04755	3.805853	0.000141	0.000402	NUCB1	protein_cc
ENSG0000(	11403.18	-0.55355	0.068849	-8.04001	8.98E-16	1.56E-14	DCLK1	protein_cc
ENSG0000(	11399.83	-0.42957	0.066286	-6.48058	9.14E-11	7.01E-10	SCAMP5	protein_cc
ENSG0000(	11385.65	0.002122	0.042073	0.050428	0.959782	0.972486	RDX	protein_cc
ENSG0000(	11370.28	0.135631	0.051374	2.640043	0.00829	0.016877	EEF1B2	protein_cc
ENSG0000(	11366.21	0.267359	0.035506	7.529908	5.08E-14	6.51E-13	RPN1	protein_cc
ENSG0000(	11334.74	-0.22476	0.037504	-5.99302	2.06E-09	1.28E-08	ABI2	protein_cc
ENSG0000(	11320.99	-0.06557	0.031522	-2.08014	0.037513	0.06555	FAM127A	protein_cc
ENSG0000(	11312.12	-0.38139	0.04935	-7.72827	1.09E-14	1.58E-13	DYNC1LI2	protein_cc
ENSG0000(	11306.1	-0.11337	0.034176	-3.31715	0.000909	0.002232	ZNF664	protein_cc
ENSG0000(	11305.75	-0.43315	0.042206	-10.2626	1.04E-24	8.60E-23	CDS2	protein_cc
ENSG0000(	11302.01	-0.02936	0.038435	-0.7639	0.444928	0.541738	GABARAP	protein_cc
ENSG0000(	11273.34	0.068818	0.037118	1.854048	0.063732	0.104432	PKN1	protein_cc
ENSG0000(	11267.27	0.076663	0.039137	1.958843	0.050131	0.084698	PARK7	protein_cc
ENSG0000(	11202.52	-0.02374	0.027518	-0.86281	0.388241	0.484935	RAB10	protein_cc
ENSG0000(	11175.83	-0.2245	0.04142	-5.41993	5.96E-08	2.93E-07	CLCN3	protein_cc
ENSG0000(	11168.61	-0.31681	0.043621	-7.26284	3.79E-13	4.20E-12	ARHGAP2	protein_cc
ENSG0000(	11160.78	0.155844	0.039973	3.898759	9.67E-05	0.000283	GPX4	protein_cc
ENSG0000(	11159.54	-0.01327	0.032221	-0.41182	0.680472	0.754014	HP1BP3	protein_cc

ENSG0000(	11154.06	-0.04828	0.031209	-1.54687	0.121893	0.183101	PFKL	protein_cc
ENSG0000(	11152.36	-0.23389	0.058072	-4.02747	5.64E-05	0.000172	ASTN1	protein_cc
ENSG0000(	11146.37	-0.29108	0.030719	-9.47559	2.65E-21	1.13E-19	LARP1	protein_cc
ENSG0000(	11144.33	0.410804	0.067373	6.097477	1.08E-09	6.99E-09	ITGB8	protein_cc
ENSG0000(	11132.12	0.122097	0.040246	3.033755	0.002415	0.005492	TMEM59	protein_cc
ENSG0000(	11113.01	0.154318	0.045229	3.411891	0.000645	0.00163	SLC25A5	protein_cc
ENSG0000(	11110.74	0.196839	0.108388	1.816058	0.069361	0.112433	EFEMP1	protein_cc
ENSG0000(	11098.35	0.082132	0.02684	3.060013	0.002213	0.005064	UBE2D3	protein_cc
ENSG0000(	11064.35	-0.10661	0.031731	-3.35984	0.00078	0.001939	FAM134A	protein_cc
ENSG0000(	11060.58	0.249869	0.026509	9.425681	4.27E-21	1.76E-19	SRSF3	protein_cc
ENSG0000(	11031.11	-0.21803	0.125287	-1.74025	0.081814	0.129665	DLL3	protein_cc
ENSG0000(	11025.61	-0.19555	0.030043	-6.50895	7.57E-11	5.88E-10	RAB6A	protein_cc
ENSG0000(	11014.48	-0.54006	0.044113	-12.2428	1.84E-34	1.01E-31	EPB41L2	protein_cc
ENSG0000(	11012.42	0.04595	0.043403	1.05868	0.289746	0.38115	DYNLL1	protein_cc
ENSG0000(	11008.25	0.039486	0.023703	1.665872	0.095739	0.148786	DHX9	protein_cc
ENSG0000(	10978.65	-0.29685	0.048132	-6.16753	6.94E-10	4.64E-09	ABCA3	protein_cc
ENSG0000(	10963.03	-0.20841	0.053798	-3.87386	0.000107	0.000311	GOLIM4	protein_cc
ENSG0000(	10959.06	0.061925	0.041213	1.502542	0.132957	0.197178	PRDX5	protein_cc
ENSG0000(	10958.24	0.047873	0.035039	1.366285	0.17185	0.245592	SERBP1	protein_cc
ENSG0000(	10946.16	-0.31303	0.116343	-2.69063	0.007132	0.014708	ATCAY	protein_cc
ENSG0000(	10941.11	-0.46362	0.071218	-6.50993	7.52E-11	5.84E-10	NFASC	protein_cc
ENSG0000(	10935.84	-0.32883	0.055213	-5.95561	2.59E-09	1.58E-08	NACC2	protein_cc
ENSG0000(	10925.12	-0.75569	0.065396	-11.5555	6.92E-31	1.94E-28	RAB6B	protein_cc
ENSG0000(	10916.6	0.045149	0.029464	1.532317	0.125444	0.187714	ATP6AP1	protein_cc
ENSG0000(	10911.17	-0.39387	0.058473	-6.73586	1.63E-11	1.40E-10	DDAH1	protein_cc
ENSG0000(	10908.51	-0.02136	0.039157	-0.54554	0.58538	0.671703	PTP4A2	protein_cc
ENSG0000(	10906.6	-0.25845	0.099323	-2.6021	0.009265	0.018651	MT1X	protein_cc
ENSG0000(	10900.51	0.075723	0.02969	2.55048	0.010757	0.021341	HNRNPM	protein_cc
ENSG0000(	10878.15	-0.2661	0.068505	-3.88432	0.000103	0.000299	BCR	protein_cc
ENSG0000(	10861.64	-0.01509	0.034696	-0.43493	0.663617	0.740184	CAPN2	protein_cc
ENSG0000(	10845.53	0.323058	0.055028	5.870844	4.34E-09	2.55E-08	PRCP	protein_cc
ENSG0000(	10845.11	0.190528	0.043542	4.375708	1.21E-05	4.12E-05	PHC2	protein_cc
ENSG0000(	10842.54	-0.04575	0.042616	-1.0736	0.283002	0.373677	SQSTM1	protein_cc
ENSG0000(	10840.43	0.406629	0.055244	7.360632	1.83E-13	2.13E-12	DCTN2	protein_cc
ENSG0000(	10831.37	0.009051	0.03203	0.282579	0.777499	0.833826	MORF4L2	protein_cc
ENSG0000(	10826.24	-0.20031	0.033828	-5.92143	3.19E-09	1.92E-08	TGOLN2	protein_cc
ENSG0000(	10824.44	-0.07309	0.033103	-2.20796	0.027247	0.049177	CNDP2	protein_cc
ENSG0000(	10797.89	0.246711	0.048966	5.03845	4.69E-07	2.00E-06	RPS28	protein_cc
ENSG0000(	10795.27	-0.0259	0.032674	-0.79274	0.427932	0.525024	CTNND1	protein_cc
ENSG0000(	10772.73	-0.40213	0.083109	-4.83854	1.31E-06	5.19E-06	SLC6A1	protein_cc
ENSG0000(	10756.05	0.138319	0.040016	3.456647	0.000547	0.001401	EMC10	protein_cc
ENSG0000(	10744.32	-0.33507	0.055675	-6.01835	1.76E-09	1.10E-08	PNMA2	protein_cc
ENSG0000(	10724.98	-0.35896	0.037091	-9.67774	3.75E-22	1.86E-20	NDST1	protein_cc
ENSG0000(	10711.71	-0.17421	0.031386	-5.55057	2.85E-08	1.47E-07	DNAJA1	protein_cc
ENSG0000(	10707.44	-0.25324	0.048247	-5.2488	1.53E-07	7.04E-07	RUFY3	protein_cc
ENSG0000(	10699.37	-0.37403	0.052454	-7.13062	9.99E-13	1.04E-11	APBB1	protein_cc
ENSG0000(	10673.48	-0.28538	0.067961	-4.19922	2.68E-05	8.62E-05	PRUNE2	protein_cc
ENSG0000(	10664.43	0.200254	0.034128	5.867728	4.42E-09	2.59E-08	MDH2	protein_cc
ENSG0000(	10662.5	-0.40897	0.04395	-9.30525	1.34E-20	5.02E-19	ATP6V0A1	protein_cc
ENSG0000(	10660.82	-0.07572	0.087296	-0.86738	0.385733	0.482404	ANGPTL2	protein_cc
ENSG0000(	10651.06	-0.33291	0.048213	-6.90501	5.02E-12	4.66E-11	ZEB2	protein_cc
ENSG0000(	10643.52	-0.14829	0.024042	-6.1682	6.91E-10	4.62E-09	GRB2	protein_cc
ENSG0000(	10632.88	0.335463	0.070432	4.762957	1.91E-06	7.38E-06	TPM4	protein_cc
ENSG0000(	10611.95	-0.30587	0.043711	-6.99765	2.60E-12	2.54E-11	PFKM	protein_cc
ENSG0000(	10580.39	-0.49856	0.049548	-10.0621	8.12E-24	5.73E-22	KIF1C	protein_cc
ENSG0000(	10578.84	-0.35134	0.045773	-7.6756	1.65E-14	2.29E-13	ATP6V1B2	protein_cc
ENSG0000(	10577.74	0.697123	0.129596	5.379204	7.48E-08	3.62E-07	COL4A2	protein_cc
ENSG0000(	10540.21	0.065508	0.068233	0.960052	0.337029	0.431869	ABHD2	protein_cc
ENSG0000(	10533.71	-0.30158	0.064103	-4.70459	2.54E-06	9.65E-06	ALCAM	protein_cc

ENSG0000(	10522.57	-0.34381	0.038401	-8.95311	3.46E-19	1.03E-17	HADHB	protein_cc
ENSG0000(	10522.03	0.143799	0.119416	1.204184	0.228518	0.312344	HLA-DRB1	protein_cc
ENSG0000(	10504.5	0.308015	0.058155	5.296459	1.18E-07	5.54E-07	RPS29	protein_cc
ENSG0000(	10502.39	2.431878	0.14703	16.54005	1.89E-61	3.88E-57	SEC61G	protein_cc
ENSG0000(	10494.41	0.07857	0.036199	2.170499	0.029969	0.053606	SDCBP	protein_cc
ENSG0000(	10494.04	-0.27517	0.030744	-8.95059	3.54E-19	1.05E-17	AP1B1	protein_cc
ENSG0000(	10485.14	-0.15591	0.083472	-1.86783	0.061785	0.101606	FGF1	protein_cc
ENSG0000(	10479.3	-0.21238	0.093378	-2.27436	0.022944	0.042159	NKAIN4	protein_cc
ENSG0000(	10459.3	-0.22329	0.028888	-7.72945	1.08E-14	1.56E-13	SEC62	protein_cc
ENSG0000(	10457.07	0.423833	0.067467	6.282037	3.34E-10	2.35E-09	RHOC	protein_cc
ENSG0000(	10453	0.182585	0.048001	3.803787	0.000143	0.000405	RHBDD2	protein_cc
ENSG0000(	10450.49	-0.54231	0.082032	-6.61094	3.82E-11	3.10E-10	CSF1R	protein_cc
ENSG0000(	10422.64	-0.11719	0.053094	-2.2072	0.0273	0.049262	NFIB	protein_cc
ENSG0000(	10419.96	0.021231	0.038016	0.558468	0.576525	0.663783	ACADVL	protein_cc
ENSG0000(	10419.23	-0.41285	0.034063	-12.1202	8.26E-34	4.09E-31	DDX24	protein_cc
ENSG0000(	10414.63	-0.3104	0.040318	-7.69865	1.38E-14	1.94E-13	PDXK	protein_cc
ENSG0000(	10410.05	-0.07605	0.030249	-2.51415	0.011932	0.023422	LASP1	protein_cc
ENSG0000(	10406.5	-0.27114	0.062193	-4.35963	1.30E-05	4.41E-05	NAV1	protein_cc
ENSG0000(	10405.67	-0.18141	0.040735	-4.45348	8.45E-06	2.95E-05	UQCRB	protein_cc
ENSG0000(	10403.46	-0.1654	0.04132	-4.00285	6.26E-05	0.00019	PSIP1	protein_cc
ENSG0000(	10398.53	-0.23596	0.035005	-6.7407	1.58E-11	1.36E-10	PTPLAD1	protein_cc
ENSG0000(	10377.98	-0.27725	0.043113	-6.43074	1.27E-10	9.54E-10	DNAJB2	protein_cc
ENSG0000(	10367.71	0.045359	0.035178	1.2894	0.197259	0.276005	ACLY	protein_cc
ENSG0000(	10348.22	-0.56033	0.056806	-9.86393	5.97E-23	3.48E-21	TNS3	protein_cc
ENSG0000(	10345.66	0.559048	0.074074	7.547163	4.45E-14	5.74E-13	ZYX	protein_cc
ENSG0000(	10333.94	-0.66799	0.082652	-8.08198	6.37E-16	1.13E-14	GAS7	protein_cc
ENSG0000(	10325.34	0.098173	0.045001	2.18156	0.029142	0.052285	GUK1	protein_cc
ENSG0000(	10315.35	-0.82375	0.118459	-6.95386	3.55E-12	3.39E-11	ENC1	protein_cc
ENSG0000(	10311.09	-0.74849	0.086952	-8.60815	7.43E-18	1.82E-16	KCNN3	protein_cc
ENSG0000(	10295.48	-0.07502	0.082261	-0.91197	0.361785	0.457631	PEG10	protein_cc
ENSG0000(	10293.64	-0.10786	0.039499	-2.7308	0.006318	0.013176	SRSF5	protein_cc
ENSG0000(	10287.61	-0.27644	0.0618	-4.47322	7.71E-06	2.71E-05	B3GAT1	protein_cc
ENSG0000(	10281.85	-0.08384	0.039344	-2.13092	0.033096	0.05859	PRKDC	protein_cc
ENSG0000(	10277.22	-0.0943	0.040213	-2.34496	0.019029	0.035602	RALGDS	protein_cc
ENSG0000(	10271.02	-0.26796	0.106748	-2.51016	0.012068	0.023662	C1QL1	protein_cc
ENSG0000(	10270.1	-0.02912	0.047297	-0.61574	0.538068	0.629158	EIF3E	protein_cc
ENSG0000(	10259.58	-0.05964	0.053524	-1.11432	0.265144	0.353961	GLTSCR2	protein_cc
ENSG0000(	10257.82	-0.40022	0.04729	-8.46305	2.60E-17	5.86E-16	SBF1	protein_cc
ENSG0000(	10249.37	0.020391	0.03733	0.546236	0.584904	0.67131	STAT3	protein_cc
ENSG0000(	10237.37	-0.61668	0.097769	-6.3075	2.84E-10	2.01E-09	OLFM1	protein_cc
ENSG0000(	10226.11	-0.21483	0.059278	-3.62404	0.00029	0.000783	SFXN5	protein_cc
ENSG0000(	10223.4	-0.16191	0.035522	-4.55811	5.16E-06	1.87E-05	DCAF7	protein_cc
ENSG0000(	10216.7	-0.01633	0.032058	-0.50925	0.610574	0.694259	ARHGDIA	protein_cc
ENSG0000(	10210.31	-0.57173	0.088771	-6.44051	1.19E-10	8.98E-10	NPTXR	protein_cc
ENSG0000(	10193.22	-0.24405	0.037964	-6.4283	1.29E-10	9.68E-10	KTN1	protein_cc
ENSG0000(	10188.42	-0.08561	0.065255	-1.31191	0.18955	0.266762	PRDX6	protein_cc
ENSG0000(	10182.69	0.045066	0.025152	1.791734	0.073176	0.117829	HNRNPA0	protein_cc
ENSG0000(	10159.89	-0.08675	0.043181	-2.00898	0.044539	0.076265	SLC6A8	protein_cc
ENSG0000(	10156.34	0.010162	0.026121	0.389057	0.697234	0.768179	VAPA	protein_cc
ENSG0000(	10148.6	-0.06265	0.039275	-1.59522	0.110664	0.168622	SMARCA4	protein_cc
ENSG0000(	10145.92	-0.01501	0.02837	-0.52908	0.596747	0.681964	AP3D1	protein_cc
ENSG0000(	10141.04	-0.31317	0.040016	-7.82598	5.04E-15	7.74E-14	KIF5B	protein_cc
ENSG0000(	10138.56	-0.58318	0.083839	-6.95596	3.50E-12	3.35E-11	CADM2	protein_cc
ENSG0000(	10131.73	-0.28086	0.040083	-7.00701	2.43E-12	2.39E-11	TJP1	protein_cc
ENSG0000(	10131.35	-0.22325	0.036935	-6.04448	1.50E-09	9.49E-09	TERF2IP	protein_cc
ENSG0000(	10128.61	-0.3189	0.052501	-6.07408	1.25E-09	8.01E-09	MEGF8	protein_cc
ENSG0000(	10122.79	-0.56371	0.075355	-7.48067	7.39E-14	9.22E-13	SLC1A4	protein_cc
ENSG0000(	10121.08	0.161648	0.052832	3.059637	0.002216	0.005069	TUBB4B	protein_cc
ENSG0000(	10114.5	-0.2386	0.02986	-7.9905	1.34E-15	2.28E-14	SERINC3	protein_cc

ENSG0000(	10110.23	0.251297	0.142825	1.75948	0.078496	0.125206	HBB	protein_cc
ENSG0000(	10107.29	-0.89257	0.089039	-10.0245	1.19E-23	8.04E-22	STXBP1	protein_cc
ENSG0000(	10092.09	-0.18146	0.033633	-5.39531	6.84E-08	3.33E-07	RAB2A	protein_cc
ENSG0000(	10081.24	-0.30146	0.041646	-7.23857	4.53E-13	4.95E-12	ARHGEF1z	protein_cc
ENSG0000(	10063.11	0.020974	0.041103	0.510263	0.609867	0.693567	DBN1	protein_cc
ENSG0000(	10045.82	0.153291	0.032754	4.679998	2.87E-06	1.08E-05	C1orf43	protein_cc
ENSG0000(	10028.46	-0.60911	0.076651	-7.94645	1.92E-15	3.17E-14	CAMK2N1	protein_cc
ENSG0000(	10025.35	-0.14279	0.039253	-3.63782	0.000275	0.000745	IPO7	protein_cc
ENSG0000(	10021.57	-0.28828	0.039158	-7.36187	1.81E-13	2.11E-12	MAP7D1	protein_cc
ENSG0000(	10014.86	-0.22482	0.027488	-8.1788	2.87E-16	5.40E-15	TBC1D9B	protein_cc
ENSG0000(	10010.61	0.139625	0.036861	3.787871	0.000152	0.00043	EDF1	protein_cc
ENSG0000(	9996.436	-1.06295	0.149608	-7.1049	1.20E-12	1.24E-11	CPLX2	protein_cc
ENSG0000(	9987.787	0.358363	0.040641	8.817746	1.17E-18	3.21E-17	ERGIC3	protein_cc
ENSG0000(	9983.838	0.079238	0.034428	2.301566	0.02136	0.039503	MLEC	protein_cc
ENSG0000(	9980.898	0.011178	0.028615	0.390621	0.696078	0.767254	MAPRE1	protein_cc
ENSG0000(	9979.874	-0.37966	0.045613	-8.32354	8.54E-17	1.75E-15	EIF3A	protein_cc
ENSG0000(	9979.196	-0.07529	0.037796	-1.99194	0.046377	0.079043	CRTAP	protein_cc
ENSG0000(	9955.69	-0.15443	0.034716	-4.44837	8.65E-06	3.02E-05	CKAP5	protein_cc
ENSG0000(	9943.232	0.113946	0.026242	4.342064	1.41E-05	4.76E-05	SSRP1	protein_cc
ENSG0000(	9937.751	-0.31371	0.058758	-5.33893	9.35E-08	4.45E-07	NOTCH2	protein_cc
ENSG0000(	9930.326	-0.23153	0.052475	-4.41218	1.02E-05	3.53E-05	PHLPP1	protein_cc
ENSG0000(	9928.967	0.197506	0.037466	5.271661	1.35E-07	6.27E-07	SRP9	protein_cc
ENSG0000(	9925.332	0.209819	0.035753	5.868551	4.40E-09	2.58E-08	RBBP4	protein_cc
ENSG0000(	9921.87	0.210049	0.118174	1.777461	0.075492	0.121021	HLA-DPA:	protein_cc
ENSG0000(	9909.775	0.210642	0.028735	7.330585	2.29E-13	2.62E-12	APH1A	protein_cc
ENSG0000(	9899.921	-0.08013	0.055539	-1.44271	0.149101	0.217659	TMEM132	protein_cc
ENSG0000(	9888.432	-0.12039	0.034279	-3.51216	0.000444	0.001159	GDI2	protein_cc
ENSG0000(	9887.257	-0.19711	0.042916	-4.59293	4.37E-06	1.60E-05	KIDINS22(	protein_cc
ENSG0000(	9886.465	0.032134	0.046998	0.683725	0.494149	0.588531	RPL26	protein_cc
ENSG0000(	9886.35	0.22262	0.044463	5.006807	5.53E-07	2.33E-06	ATP5E	protein_cc
ENSG0000(	9885.925	-0.1582	0.058335	-2.71195	0.006689	0.013881	SOGA1	protein_cc
ENSG0000(	9884.832	0.819801	0.142043	5.771499	7.86E-09	4.43E-08	COL4A1	protein_cc
ENSG0000(	9865.926	-0.06796	0.073977	-0.91868	0.358261	0.454054	CCDC80	protein_cc
ENSG0000(	9860.679	0.233606	0.060062	3.889402	0.0001	0.000294	COTL1	protein_cc
ENSG0000(	9855.412	-0.36143	0.04406	-8.20316	2.34E-16	4.49E-15	SLC25A23	protein_cc
ENSG0000(	9853.507	0.931838	0.114733	8.12177	4.59E-16	8.39E-15	TAGLN2	protein_cc
ENSG0000(	9850.03	-0.31585	0.051346	-6.15146	7.68E-10	5.10E-09	LPHN1	protein_cc
ENSG0000(	9849.251	-0.70146	0.088112	-7.96094	1.71E-15	2.84E-14	MTND2P2	pseudoge
ENSG0000(	9824.977	-0.43507	0.049832	-8.73073	2.53E-18	6.64E-17	TRIM2	protein_cc
ENSG0000(	9813.96	-0.50941	0.095473	-5.33567	9.52E-08	4.52E-07	SPON1	protein_cc
ENSG0000(	9813.603	-0.10967	0.028054	-3.9094	9.25E-05	0.000272	SUPT5H	protein_cc
ENSG0000(	9798.355	-0.4532	0.090176	-5.02571	5.02E-07	2.13E-06	AMER2	protein_cc
ENSG0000(	9795.023	-0.37601	0.047092	-7.98472	1.41E-15	2.38E-14	PRKACB	protein_cc
ENSG0000(	9786.039	-0.06408	0.031758	-2.01771	0.043621	0.074885	PPP2R4	protein_cc
ENSG0000(	9781.289	0.147006	0.024394	6.026228	1.68E-09	1.06E-08	EIF2AK1	protein_cc
ENSG0000(	9776.969	-0.07263	0.034054	-2.13284	0.032938	0.058343	CASC4	protein_cc
ENSG0000(	9758.178	-0.53146	0.067943	-7.82208	5.20E-15	7.97E-14	NAT8L	protein_cc
ENSG0000(	9754.421	-0.24758	0.055786	-4.438	9.08E-06	3.15E-05	PTPRF	protein_cc
ENSG0000(	9748.966	-0.30001	0.044074	-6.80691	9.97E-12	8.85E-11	MGEA5	protein_cc
ENSG0000(	9740.179	-0.13407	0.048025	-2.79159	0.005245	0.011135	SETD5	protein_cc
ENSG0000(	9738.527	-0.35698	0.036536	-9.77056	1.51E-22	8.05E-21	DNAJC5	protein_cc
ENSG0000(	9729.917	-0.40895	0.093122	-4.3916	1.13E-05	3.85E-05	GRIA2	protein_cc
ENSG0000(	9725.201	-0.07544	0.019063	-3.95728	7.58E-05	0.000226	CSNK1D	protein_cc
ENSG0000(	9723.808	-0.41857	0.058535	-7.15085	8.62E-13	9.07E-12	BIN1	protein_cc
ENSG0000(	9718.953	-0.00171	0.043011	-0.03984	0.968224	0.978071	PLXNB2	protein_cc
ENSG0000(	9717.434	-0.20093	0.043144	-4.65734	3.20E-06	1.19E-05	CCDC88A	protein_cc
ENSG0000(	9709.498	-0.34115	0.050026	-6.8193	9.15E-12	8.16E-11	FADS1	protein_cc
ENSG0000(	9709.331	-0.14412	0.038511	-3.74238	0.000182	0.000509	KLHDC3	protein_cc
ENSG0000(	9709.17	-0.01165	0.038271	-0.30436	0.760855	0.820297	NDUFV1	protein_cc

ENSG0000(	9705.819	0.375609	0.053974	6.959036	3.43E-12	3.29E-11	RPL41	protein_cc
ENSG0000(	9693.073	-0.01104	0.059618	-0.18525	0.853029	0.893757	HEY1	protein_cc
ENSG0000(	9688.819	0.035901	0.039561	0.907469	0.364159	0.460124	TPM3	protein_cc
ENSG0000(	9679.848	-0.3041	0.042293	-7.1902	6.47E-13	6.92E-12	ZC3H7B	protein_cc
ENSG0000(	9673.981	-0.42882	0.132967	-3.22503	0.00126	0.003014	NSG2	protein_cc
ENSG0000(	9670.42	0.551846	0.107883	5.115241	3.13E-07	1.37E-06	LGALS1	protein_cc
ENSG0000(	9659.389	-0.25478	0.057642	-4.42011	9.87E-06	3.41E-05	PDLIM5	protein_cc
ENSG0000(	9655.918	0.146944	0.057663	2.548318	0.010824	0.021464	GRN	protein_cc
ENSG0000(	9653.044	-0.16479	0.033168	-4.96836	6.75E-07	2.81E-06	DDX6	protein_cc
ENSG0000(	9650.958	0.257381	0.051033	5.043399	4.57E-07	1.95E-06	CDC42	protein_cc
ENSG0000(	9650.796	0.064449	0.063787	1.010369	0.312318	0.405614	NLGN3	protein_cc
ENSG0000(	9641.351	0.254218	0.032833	7.742807	9.72E-15	1.42E-13	ILF2	protein_cc
ENSG0000(	9637.836	-0.07567	0.033345	-2.26915	0.023259	0.042673	CLPTM1	protein_cc
ENSG0000(	9637.265	-1.00476	0.092708	-10.8379	2.28E-27	3.11E-25	NCDN	protein_cc
ENSG0000(	9630.936	0.027031	0.042132	0.641563	0.521157	0.613396	PRPF6	protein_cc
ENSG0000(	9628.28	0.220437	0.036418	6.052963	1.42E-09	9.04E-09	HSBP1	protein_cc
ENSG0000(	9627.763	-0.63984	0.065284	-9.80087	1.12E-22	6.12E-21	CYFIP2	protein_cc
ENSG0000(	9624.389	-0.49284	0.119794	-4.11404	3.89E-05	0.000122	APLNR	protein_cc
ENSG0000(	9621.931	-0.70478	0.084339	-8.35649	6.46E-17	1.36E-15	ATP1B1	protein_cc
ENSG0000(	9593.617	0.215048	0.058905	3.650762	0.000261	0.000711	LAMB2	protein_cc
ENSG0000(	9589.226	-0.09379	0.030186	-3.10725	0.001888	0.004377	NUDCD3	protein_cc
ENSG0000(	9573.14	-0.12906	0.033126	-3.89605	9.78E-05	0.000286	CASC3	protein_cc
ENSG0000(	9555.536	0.086289	0.031449	2.74375	0.006074	0.01271	ATP6AP2	protein_cc
ENSG0000(	9554.726	-0.46356	0.043559	-10.6419	1.90E-26	2.23E-24	ACO2	protein_cc
ENSG0000(	9540.034	0.046302	0.045435	1.019081	0.308164	0.401246	VAT1	protein_cc
ENSG0000(	9539.635	0.176594	0.065942	2.678022	0.007406	0.015229	WSB1	protein_cc
ENSG0000(	9534.545	-0.56851	0.095452	-5.95602	2.58E-09	1.58E-08	SPOCK1	protein_cc
ENSG0000(	9530.269	-0.00452	0.029493	-0.15331	0.878155	0.912873	IPO5	protein_cc
ENSG0000(	9525.455	0.265416	0.035203	7.539597	4.71E-14	6.06E-13	PSMB4	protein_cc
ENSG0000(	9517.71	0.513727	0.093927	5.469448	4.51E-08	2.26E-07	HSPB1	protein_cc
ENSG0000(	9511.231	0.660174	0.119187	5.538989	3.04E-08	1.56E-07	IFI6	protein_cc
ENSG0000(	9502.91	-0.11782	0.056397	-2.0892	0.03669	0.06428	PAM	protein_cc
ENSG0000(	9499.912	-0.506	0.056598	-8.94029	3.88E-19	1.15E-17	C1orf198	protein_cc
ENSG0000(	9489.734	0.013804	0.032421	0.42576	0.670283	0.745491	MAP1LC3I	protein_cc
ENSG0000(	9482.987	0.281789	0.032949	8.552345	1.21E-17	2.85E-16	EIF3B	protein_cc
ENSG0000(	9458.203	-0.09598	0.025914	-3.70396	0.000212	0.000586	WDR82	protein_cc
ENSG0000(	9452.616	-0.09387	0.038658	-2.42832	0.015169	0.029074	EIF3H	protein_cc
ENSG0000(	9449.592	-0.43708	0.038257	-11.4248	3.14E-30	7.54E-28	MAPRE2	protein_cc
ENSG0000(	9426.071	0.032048	0.031669	1.011943	0.311565	0.404854	TAX1BP1	protein_cc
ENSG0000(	9416.334	-0.06072	0.049783	-1.21972	0.222572	0.305489	ARHGEF6	protein_cc
ENSG0000(	9412.244	0.433339	0.035282	12.28231	1.13E-34	6.53E-32	CBX3	protein_cc
ENSG0000(	9412.016	0.099283	0.028384	3.497911	0.000469	0.001216	RBM39	protein_cc
ENSG0000(	9407.266	-0.39289	0.050249	-7.81885	5.33E-15	8.16E-14	APC	protein_cc
ENSG0000(	9403.048	-0.39837	0.03968	-10.0395	1.02E-23	7.03E-22	GHITM	protein_cc
ENSG0000(	9401.976	-0.45114	0.055598	-8.11426	4.89E-16	8.89E-15	MAPK8IP3	protein_cc
ENSG0000(	9401.9	-0.2936	0.036977	-7.94006	2.02E-15	3.32E-14	NCOA4	protein_cc
ENSG0000(	9401.878	-0.84316	0.080644	-10.4553	1.39E-25	1.36E-23	ADCY2	protein_cc
ENSG0000(	9401	-0.04841	0.02788	-1.73621	0.082527	0.130673	AMFR	protein_cc
ENSG0000(	9390.373	0.349924	0.099081	3.531707	0.000413	0.001083	IFITM3	protein_cc
ENSG0000(	9388.755	0.176267	0.03457	5.098831	3.42E-07	1.49E-06	BCAP31	protein_cc
ENSG0000(	9384.528	-0.31154	0.045646	-6.82511	8.79E-12	7.86E-11	JAK1	protein_cc
ENSG0000(	9379.494	-0.34207	0.056838	-6.01829	1.76E-09	1.10E-08	SV2A	protein_cc
ENSG0000(	9342.775	-0.22813	0.039473	-5.77948	7.49E-09	4.24E-08	UBL3	protein_cc
ENSG0000(	9340.239	-0.42037	0.062703	-6.70425	2.02E-11	1.71E-10	TMEM59L	protein_cc
ENSG0000(	9337.674	0.089598	0.04729	1.894642	0.05814	0.096399	ID2	protein_cc
ENSG0000(	9332.082	-0.267	0.029783	-8.96491	3.11E-19	9.34E-18	C6orf89	protein_cc
ENSG0000(	9318.938	1.637261	0.166003	9.862836	6.03E-23	3.51E-21	IGFBP2	protein_cc
ENSG0000(	9316.647	-0.22075	0.031013	-7.11794	1.10E-12	1.13E-11	PLEKHB2	protein_cc
ENSG0000(	9316.096	0.279214	0.096526	2.892641	0.00382	0.00834	CACNG4	protein_cc

ENSG0000(	9300.011	-0.03044	0.02377	-1.28047	0.20038	0.279726	PPP2CA	protein_cc
ENSG0000(	9290.461	-0.46745	0.0471	-9.9247	3.25E-23	1.98E-21	ARHGAP3	protein_cc
ENSG0000(	9280.278	-0.10319	0.032837	-3.14252	0.001675	0.003921	SND1	protein_cc
ENSG0000(	9273.113	0.249459	0.0578	4.315919	1.59E-05	5.31E-05	TRIO	protein_cc
ENSG0000(	9267.001	0.005715	0.030456	0.187634	0.851163	0.892362	BNIP3L	protein_cc
ENSG0000(	9263.778	0.121296	0.027165	4.465237	8.00E-06	2.80E-05	HNRNPD	protein_cc
ENSG0000(	9263.506	-0.22815	0.036865	-6.18885	6.06E-10	4.08E-09	ATXN7L3E	protein_cc
ENSG0000(	9253.175	0.123985	0.061209	2.025603	0.042805	0.073651	AIF1L	protein_cc
ENSG0000(	9239.755	-0.15352	0.037765	-4.0652	4.80E-05	0.000148	KIAA0195	protein_cc
ENSG0000(	9237.871	-0.15917	0.047239	-3.36951	0.000753	0.001877	APBA2	protein_cc
ENSG0000(	9232.126	-0.25918	0.05143	-5.03952	4.67E-07	1.99E-06	GPT2	protein_cc
ENSG0000(	9231.558	0.052633	0.030994	1.698162	0.089477	0.140339	RAB11B	protein_cc
ENSG0000(	9221.991	-0.81648	0.081274	-10.046	9.57E-24	6.65E-22	NCS1	protein_cc
ENSG0000(	9211.393	-0.0906	0.102435	-0.88444	0.376456	0.472918	C1QA	protein_cc
ENSG0000(	9188.708	-0.07703	0.038067	-2.02356	0.043015	0.073972	NFIC	protein_cc
ENSG0000(	9181.44	-0.15747	0.045574	-3.45531	0.00055	0.001407	LRP10	protein_cc
ENSG0000(	9173.925	-0.28286	0.033814	-8.36513	6.00E-17	1.27E-15	ACTR1A	protein_cc
ENSG0000(	9157.223	0.148883	0.035747	4.164874	3.12E-05	9.92E-05	SURF4	protein_cc
ENSG0000(	9148.797	-0.26319	0.064764	-4.06388	4.83E-05	0.000149	KCTD12	protein_cc
ENSG0000(	9141.529	0.108124	0.03541	3.05351	0.002262	0.005168	ARPC2	protein_cc
ENSG0000(	9141.358	-0.23287	0.038947	-5.97933	2.24E-09	1.38E-08	ANKFY1	protein_cc
ENSG0000(	9137.071	0.709474	0.120046	5.910023	3.42E-09	2.05E-08	NAMPT	protein_cc
ENSG0000(	9136.246	-0.21586	0.033938	-6.36052	2.01E-10	1.46E-09	6-Mar	protein_cc
ENSG0000(	9136.038	-0.26519	0.044459	-5.96483	2.45E-09	1.50E-08	SMARCC2	protein_cc
ENSG0000(	9130.354	-0.30159	0.037141	-8.11999	4.66E-16	8.51E-15	CD99L2	protein_cc
ENSG0000(	9128.552	-0.3925	0.070605	-5.55913	2.71E-08	1.41E-07	STOM	protein_cc
ENSG0000(	9122.724	-0.02994	0.030437	-0.98358	0.325321	0.419498	VDAC1	protein_cc
ENSG0000(	9117.445	-0.39516	0.038183	-10.3491	4.23E-25	3.80E-23	GNL1	protein_cc
ENSG0000(	9099.801	0.234899	0.028658	8.196553	2.47E-16	4.72E-15	RBMX	protein_cc
ENSG0000(	9084.243	-0.2146	0.054053	-3.9703	7.18E-05	0.000215	EIF3L	protein_cc
ENSG0000(	9082.324	-0.57983	0.081542	-7.11084	1.15E-12	1.19E-11	PAQR6	protein_cc
ENSG0000(	9058.525	-0.35571	0.044852	-7.93082	2.18E-15	3.56E-14	B3GNT1	protein_cc
ENSG0000(	9048.159	-0.35926	0.045635	-7.87247	3.48E-15	5.50E-14	PACS2	protein_cc
ENSG0000(	9043.616	-0.47257	0.038352	-12.3221	6.89E-35	4.29E-32	MINK1	protein_cc
ENSG0000(	9037.018	0.161722	0.028218	5.73117	9.97E-09	5.53E-08	U2AF2	protein_cc
ENSG0000(	9036.544	-0.11492	0.092182	-1.24671	0.212502	0.293884	PSD2	protein_cc
ENSG0000(	9026.91	-0.05667	0.029821	-1.90017	0.057411	0.095363	SH3BGRL	protein_cc
ENSG0000(	9015.786	-0.12361	0.029491	-4.19157	2.77E-05	8.90E-05	PICALM	protein_cc
ENSG0000(	9012.286	0.036065	0.027901	1.29264	0.196136	0.274695	SF3B3	protein_cc
ENSG0000(	9004.297	-0.0534	0.031813	-1.67871	0.093208	0.145325	SARS	protein_cc
ENSG0000(	8998.206	0.291781	0.028994	10.06343	8.02E-24	5.66E-22	TAF7	protein_cc
ENSG0000(	8992.389	0.196094	0.039716	4.937419	7.92E-07	3.25E-06	PHB2	protein_cc
ENSG0000(	8992.249	-0.26834	0.042735	-6.27917	3.40E-10	2.38E-09	TAOK1	protein_cc
ENSG0000(	8985.756	-0.02826	0.064567	-0.43763	0.661656	0.738498	BTG2	protein_cc
ENSG0000(	8985.439	0.287231	0.058719	4.891663	1.00E-06	4.04E-06	ZFP36L2	protein_cc
ENSG0000(	8983.017	-0.18916	0.035238	-5.36803	7.96E-08	3.83E-07	CRKL	protein_cc
ENSG0000(	8978.36	-0.25833	0.060833	-4.24655	2.17E-05	7.10E-05	TNFRSF21	protein_cc
ENSG0000(	8970.277	-0.28742	0.089632	-3.20671	0.001343	0.003198	KCNQ2	protein_cc
ENSG0000(	8969.533	0.03057	0.037587	0.813321	0.416034	0.513025	COX7C	protein_cc
ENSG0000(	8967.282	0.071092	0.038808	1.831892	0.066968	0.109008	PTOV1	protein_cc
ENSG0000(	8966.212	-0.5993	0.071184	-8.41901	3.80E-17	8.29E-16	FAM213A	protein_cc
ENSG0000(	8926.198	-0.11231	0.052685	-2.13179	0.033024	0.058479	BAI2	protein_cc
ENSG0000(	8918.31	-0.88447	0.096153	-9.1986	3.63E-20	1.27E-18	SYP	protein_cc
ENSG0000(	8913.634	-0.09902	0.05104	-1.94007	0.052371	0.087996	PHLDB1	protein_cc
ENSG0000(	8896.311	0.29723	0.112216	2.64873	0.008079	0.016489	EMP1	protein_cc
ENSG0000(	8889.909	0.582691	0.072953	7.987163	1.38E-15	2.33E-14	DTX3	protein_cc
ENSG0000(	8888.034	-0.2872	0.056514	-5.08196	3.74E-07	1.62E-06	SESN3	protein_cc
ENSG0000(	8885.182	0.162704	0.022269	7.306157	2.75E-13	3.11E-12	HNRNPL	protein_cc
ENSG0000(	8875.18	-0.37139	0.060302	-6.15889	7.33E-10	4.88E-09	SLCO2B1	protein_cc

ENSG0000(	8870.401	-0.92815	0.107112	-8.66527	4.50E-18	1.14E-16	TPPP	protein_cc
ENSG0000(	8869.441	-0.35992	0.034724	-10.3653	3.57E-25	3.23E-23	NISCH	protein_cc
ENSG0000(	8867.463	-0.2856	0.035157	-8.12359	4.53E-16	8.27E-15	TM9SF3	protein_cc
ENSG0000(	8865.587	-0.02528	0.045397	-0.55695	0.57756	0.664677	JAM2	protein_cc
ENSG0000(	8865.493	-0.29553	0.050142	-5.89393	3.77E-09	2.24E-08	SUN2	protein_cc
ENSG0000(	8858.75	-0.308	0.090333	-3.4096	0.000651	0.001643	PADI2	protein_cc
ENSG0000(	8844.791	-0.28336	0.095437	-2.96907	0.002987	0.00666	GRIA4	protein_cc
ENSG0000(	8829.62	0.675531	0.073453	9.196727	3.69E-20	1.29E-18	WSCD1	protein_cc
ENSG0000(	8813.124	-1.78535	0.165549	-10.7844	4.08E-27	5.37E-25	CAMK2A	protein_cc
ENSG0000(	8812.421	-0.11342	0.025835	-4.39011	1.13E-05	3.88E-05	NARS	protein_cc
ENSG0000(	8812.145	0.095596	0.035449	2.69676	0.007002	0.014466	UQCRC1	protein_cc
ENSG0000(	8808.297	-0.13716	0.042212	-3.24919	0.001157	0.002785	11-Sep	protein_cc
ENSG0000(	8797.313	-0.05652	0.023596	-2.39525	0.016609	0.031542	RAB14	protein_cc
ENSG0000(	8794.74	-0.36333	0.040545	-8.96106	3.22E-19	9.65E-18	UBR4	protein_cc
ENSG0000(	8793.757	-0.07693	0.032027	-2.40201	0.016305	0.03104	UQCRC2	protein_cc
ENSG0000(	8787.413	0.213269	0.03909	5.455921	4.87E-08	2.43E-07	PLCG1	protein_cc
ENSG0000(	8778.007	-0.0333	0.02722	-1.22355	0.22112	0.303811	ZFR	protein_cc
ENSG0000(	8771.135	-0.01024	0.048312	-0.21201	0.832098	0.877504	LUC7L3	protein_cc
ENSG0000(	8748.653	0.224114	0.026178	8.561147	1.12E-17	2.65E-16	H2AFY	protein_cc
ENSG0000(	8744.703	0.32596	0.054393	5.992662	2.06E-09	1.28E-08	CAPZA2	protein_cc
ENSG0000(	8744.653	0.729995	0.17393	4.197066	2.70E-05	8.69E-05	TIMP1	protein_cc
ENSG0000(	8716.008	0.100881	0.028348	3.558688	0.000373	0.000986	NAP1L4	protein_cc
ENSG0000(	8712.924	-0.08188	0.04755	-1.72209	0.085053	0.134207	SEC14L1	protein_cc
ENSG0000(	8697.264	0.078772	0.035804	2.20012	0.027798	0.050087	REPIN1	protein_cc
ENSG0000(	8694.121	0.075496	0.02439	3.095423	0.001965	0.00454	XPO1	protein_cc
ENSG0000(	8686.322	0.039287	0.067635	0.580869	0.561329	0.650113	FAM181B	protein_cc
ENSG0000(	8675.159	0.277603	0.041271	6.726324	1.74E-11	1.49E-10	TCEB2	protein_cc
ENSG0000(	8664.659	-0.33623	0.040122	-8.38006	5.29E-17	1.13E-15	WAC	protein_cc
ENSG0000(	8664.583	-0.157	0.033779	-4.64787	3.35E-06	1.25E-05	SF3A1	protein_cc
ENSG0000(	8655.753	0.158287	0.033201	4.767524	1.87E-06	7.22E-06	YKT6	protein_cc
ENSG0000(	8652.174	0.059723	0.06466	0.923656	0.355666	0.451279	LENG8	protein_cc
ENSG0000(	8620.71	0.753495	0.130938	5.754615	8.68E-09	4.86E-08	ANXA2	protein_cc
ENSG0000(	8620.042	-0.08103	0.036842	-2.19953	0.02784	0.050143	USF2	protein_cc
ENSG0000(	8619.775	-0.16953	0.044001	-3.85296	0.000117	0.000337	ZHX3	protein_cc
ENSG0000(	8606.423	-0.04218	0.067055	-0.62901	0.529343	0.621005	ACAP3	protein_cc
ENSG0000(	8596.29	-0.01737	0.036515	-0.47562	0.634347	0.715219	COMT	protein_cc
ENSG0000(	8592.818	-0.47639	0.091138	-5.22709	1.72E-07	7.86E-07	SLC38A1	protein_cc
ENSG0000(	8589.308	-0.39056	0.04689	-8.32924	8.14E-17	1.68E-15	SLC23A2	protein_cc
ENSG0000(	8574.435	0.140451	0.034483	4.073062	4.64E-05	0.000144	CENPB	protein_cc
ENSG0000(	8554.304	0.204302	0.046205	4.421615	9.80E-06	3.39E-05	MIDN	protein_cc
ENSG0000(	8553.916	0.081976	0.024596	3.332928	0.000859	0.00212	RAB1B	protein_cc
ENSG0000(	8543.8	-0.52249	0.069077	-7.5638	3.91E-14	5.10E-13	SERINC5	protein_cc
ENSG0000(	8543.617	-0.33637	0.053593	-6.27634	3.47E-10	2.43E-09	SNRPN	protein_cc
ENSG0000(	8539.951	-0.06519	0.02827	-2.30607	0.021107	0.03909	ATXN10	protein_cc
ENSG0000(	8533.761	-0.02041	0.034276	-0.59539	0.55158	0.641384	OGDH	protein_cc
ENSG0000(	8531.818	-0.01619	0.039371	-0.4113	0.680852	0.754313	ARL6IP5	protein_cc
ENSG0000(	8531.274	0.110782	0.056899	1.947003	0.051534	0.086765	HIP1	protein_cc
ENSG0000(	8522.938	-0.18695	0.049214	-3.79874	0.000145	0.000413	TNS1	protein_cc
ENSG0000(	8521.584	0.127001	0.07476	1.698794	0.089358	0.140183	DNAJB1	protein_cc
ENSG0000(	8496.164	0.10856	0.043987	2.46798	0.013588	0.026343	LAPTM4B	protein_cc
ENSG0000(	8495.645	0.512768	0.054255	9.451014	3.36E-21	1.42E-19	HMGN2	protein_cc
ENSG0000(	8483.854	0.068098	0.059346	1.147483	0.251182	0.338409	SCRG1	protein_cc
ENSG0000(	8481.756	0.031743	0.018736	1.694223	0.090223	0.141335	IST1	protein_cc
ENSG0000(	8481.646	-0.11338	0.041224	-2.75022	0.005956	0.012482	TUG1	antisense
ENSG0000(	8466.822	-0.65395	0.059565	-10.9786	4.84E-28	7.34E-26	LIMCH1	protein_cc
ENSG0000(	8437.02	-0.4752	0.042135	-11.2782	1.68E-29	3.46E-27	CDC42BPI	protein_cc
ENSG0000(	8435.478	-0.03663	0.029685	-1.23392	0.217234	0.299371	PSMC5	protein_cc
ENSG0000(	8427.738	0.180962	0.036635	4.939571	7.83E-07	3.22E-06	SRSF6	protein_cc
ENSG0000(	8427.407	0.282744	0.04815	5.872184	4.30E-09	2.53E-08	SSFA2	protein_cc

ENSG0000(	8422.688	-0.70711	0.068853	-10.2699	9.64E-25	8.04E-23	VPS13D	protein_cc
ENSG0000(	8421.078	-0.56962	0.082981	-6.86442	6.68E-12	6.10E-11	GALNT15	protein_cc
ENSG0000(	8419.546	-0.22538	0.035998	-6.26077	3.83E-10	2.66E-09	ALDH9A1	protein_cc
ENSG0000(	8407.375	-0.18579	0.032944	-5.63936	1.71E-08	9.13E-08	DDX42	protein_cc
ENSG0000(	8401.9	0.236747	0.039944	5.926906	3.09E-09	1.86E-08	TOMM7	protein_cc
ENSG0000(	8390.828	-0.35303	0.056177	-6.28419	3.30E-10	2.32E-09	CHD3	protein_cc
ENSG0000(	8389.773	-0.01634	0.030043	-0.54376	0.586608	0.672887	TCP1	protein_cc
ENSG0000(	8379.943	-0.07039	0.030844	-2.28197	0.022491	0.041419	UBTF	protein_cc
ENSG0000(	8377.584	-0.04942	0.025593	-1.93108	0.053473	0.089624	CHMP4B	protein_cc
ENSG0000(	8367.658	0.026045	0.07272	0.358151	0.72023	0.787084	SLAIN1	protein_cc
ENSG0000(	8360.172	-0.02437	0.026513	-0.91932	0.357928	0.453716	LARS	protein_cc
ENSG0000(	8359.887	-0.35842	0.042686	-8.39679	4.59E-17	9.86E-16	LLGL1	protein_cc
ENSG0000(	8359.463	-0.00457	0.025203	-0.18144	0.856019	0.896	APMAP	protein_cc
ENSG0000(	8351.887	-0.60053	0.090924	-6.6048	3.98E-11	3.23E-10	4-Sep	protein_cc
ENSG0000(	8350.639	0.261031	0.033916	7.696435	1.40E-14	1.97E-13	CCT2	protein_cc
ENSG0000(	8346.053	-0.0972	0.031086	-3.1267	0.001768	0.004121	CUX1	protein_cc
ENSG0000(	8334.675	0.149857	0.032129	4.664302	3.10E-06	1.16E-05	APEX1	protein_cc
ENSG0000(	8325.805	0.276396	0.04565	6.054702	1.41E-09	8.96E-09	FKBP1A	protein_cc
ENSG0000(	8324.215	-0.28127	0.036911	-7.62024	2.53E-14	3.41E-13	EFCAB14	protein_cc
ENSG0000(	8322.852	-0.27294	0.03588	-7.60683	2.81E-14	3.74E-13	EIF4EBP2	protein_cc
ENSG0000(	8317.254	0.010061	0.035959	0.279778	0.779648	0.835585	EIF3F	protein_cc
ENSG0000(	8312.095	-0.60531	0.068436	-8.84487	9.16E-19	2.55E-17	FAT1	protein_cc
ENSG0000(	8307.321	0.367571	0.044748	8.21415	2.14E-16	4.11E-15	SSR2	protein_cc
ENSG0000(	8303.835	0.339815	0.046542	7.301219	2.85E-13	3.22E-12	PAICS	protein_cc
ENSG0000(	8302.443	-0.0715	0.025239	-2.8328	0.004614	0.009894	UBAP2L	protein_cc
ENSG0000(	8295.419	-0.06943	0.026984	-2.57294	0.010084	0.020121	CS	protein_cc
ENSG0000(	8293.351	-0.37417	0.036072	-10.3729	3.29E-25	3.00E-23	AGPAT3	protein_cc
ENSG0000(	8292.162	0.122656	0.051705	2.372234	0.017681	0.033314	NOVA1	protein_cc
ENSG0000(	8286.299	-0.61176	0.103274	-5.92364	3.15E-09	1.89E-08	GRIA1	protein_cc
ENSG0000(	8279.307	0.34128	0.105882	3.223227	0.001268	0.003032	BGN	protein_cc
ENSG0000(	8276.112	0.055596	0.025514	2.179061	0.029327	0.05258	SSR1	protein_cc
ENSG0000(	8263.733	0.218071	0.043837	4.974615	6.54E-07	2.72E-06	ARF4	protein_cc
ENSG0000(	8250.426	-0.35952	0.055921	-6.42907	1.28E-10	9.63E-10	PLCD3	protein_cc
ENSG0000(	8247.321	0.234365	0.108714	2.155785	0.0311	0.055393	SCG2	protein_cc
ENSG0000(	8245.857	0.020056	0.028199	0.711249	0.47693	0.572591	IPO9	protein_cc
ENSG0000(	8245.235	-0.1181	0.029423	-4.01399	5.97E-05	0.000181	TCF25	protein_cc
ENSG0000(	8240.533	-0.23679	0.041378	-5.7226	1.05E-08	5.80E-08	EIF5B	protein_cc
ENSG0000(	8234.069	0.273321	0.044242	6.177829	6.50E-10	4.36E-09	HINT1	protein_cc
ENSG0000(	8230.157	-0.62893	0.066825	-9.41158	4.89E-21	1.99E-19	WASF3	protein_cc
ENSG0000(	8219.487	-0.1223	0.034487	-3.54629	0.000391	0.00103	SRCAP	protein_cc
ENSG0000(	8219.317	-0.20456	0.039026	-5.24149	1.59E-07	7.30E-07	HNRNPH3	protein_cc
ENSG0000(	8217.835	1.302157	0.104906	12.41257	2.23E-35	1.53E-32	CDK4	protein_cc
ENSG0000(	8206.553	-0.70216	0.089945	-7.80656	5.88E-15	8.91E-14	CADM3	protein_cc
ENSG0000(	8203.25	-0.01777	0.026642	-0.66702	0.504762	0.598588	HNRNPR	protein_cc
ENSG0000(	8202.174	-0.94975	0.100874	-9.41521	4.72E-21	1.93E-19	CHN1	protein_cc
ENSG0000(	8200.61	-0.31095	0.050839	-6.11642	9.57E-10	6.26E-09	MDH1	protein_cc
ENSG0000(	8187.851	0.242327	0.074978	3.231987	0.001229	0.002948	STAT1	protein_cc
ENSG0000(	8181.097	0.333741	0.068895	4.844214	1.27E-06	5.06E-06	CALD1	protein_cc
ENSG0000(	8177.032	-0.31617	0.046306	-6.82787	8.62E-12	7.72E-11	MYH10	protein_cc
ENSG0000(	8170.272	1.440559	0.116239	12.3931	2.85E-35	1.86E-32	FABP7	protein_cc
ENSG0000(	8161.574	-0.22909	0.105332	-2.17494	0.029635	0.053068	BAI1	protein_cc
ENSG0000(	8148.99	-0.47197	0.048068	-9.81879	9.35E-23	5.19E-21	CELF2	protein_cc
ENSG0000(	8141.257	0.095555	0.029885	3.197359	0.001387	0.003297	SGTA	protein_cc
ENSG0000(	8138.098	-0.12352	0.050741	-2.43439	0.014917	0.028644	ERBB2IP	protein_cc
ENSG0000(	8135.04	-0.32115	0.049611	-6.47332	9.59E-11	7.33E-10	CDIP1	protein_cc
ENSG0000(	8134.144	-0.29769	0.064539	-4.61261	3.98E-06	1.46E-05	TP53INP2	protein_cc
ENSG0000(	8130.092	-0.05004	0.036655	-1.36527	0.172167	0.245951	IDH2	protein_cc
ENSG0000(	8116.089	0.267914	0.133064	2.013419	0.044071	0.075556	MAOB	protein_cc
ENSG0000(	8104.737	-0.05712	0.048708	-1.17262	0.240946	0.326405	IMPDH2	protein_cc

ENSG0000	8099.229	0.053315	0.027968	1.90629	0.056613	0.094165	DCTN4	protein_cc
ENSG0000	8096.327	0.073079	0.081036	0.901819	0.367153	0.463281	SEMA5A	protein_cc
ENSG0000	8094.934	0.061315	0.03647	1.681237	0.092717	0.144685	INTS1	protein_cc
ENSG0000	8092.471	0.079732	0.039655	2.010633	0.044364	0.075995	OBSL1	protein_cc
ENSG0000	8087.573	-0.39186	0.04748	-8.2532	1.54E-16	3.04E-15	OIP5-AS1	processed
ENSG0000	8083.718	-0.14678	0.07166	-2.04828	0.040533	0.070208	MALAT1	lincRNA
ENSG0000	8074.088	-0.42275	0.050756	-8.32906	8.15E-17	1.68E-15	TOM1L2	protein_cc
ENSG0000	8071.066	0.056266	0.028961	1.942847	0.052035	0.087524	SBDS	protein_cc
ENSG0000	8070.165	-0.50379	0.091313	-5.51721	3.44E-08	1.76E-07	NRXN1	protein_cc
ENSG0000	8064.336	-1.43482	0.153797	-9.32928	1.07E-20	4.09E-19	NRGN	protein_cc
ENSG0000	8057.751	-0.38811	0.072044	-5.3871	7.16E-08	3.47E-07	KIF3C	protein_cc
ENSG0000	8048.282	0.166133	0.038148	4.354967	1.33E-05	4.50E-05	CXXC5	protein_cc
ENSG0000	8044.686	-0.029	0.022477	-1.29008	0.197022	0.275721	WDR45B	protein_cc
ENSG0000	8042.649	0.902813	0.13638	6.619811	3.60E-11	2.94E-10	VEGFA	protein_cc
ENSG0000	8042.499	-0.05048	0.021302	-2.3697	0.017803	0.033511	RNF10	protein_cc
ENSG0000	8026.764	0.12902	0.062787	2.054887	0.03989	0.069222	EPN2	protein_cc
ENSG0000	8024.761	-0.03294	0.031699	-1.03921	0.298708	0.390949	USP5	protein_cc
ENSG0000	8018.532	-0.1127	0.04532	-2.48677	0.012891	0.025106	WASF2	protein_cc
ENSG0000	8013.403	0.231969	0.037972	6.10893	1.00E-09	6.54E-09	COPE	protein_cc
ENSG0000	8001.474	-0.2612	0.035955	-7.26473	3.74E-13	4.14E-12	GMFB	protein_cc
ENSG0000	8000.438	-0.08243	0.034164	-2.4128	0.015831	0.03022	TAF15	protein_cc
ENSG0000	7995.555	-0.21979	0.043142	-5.09463	3.49E-07	1.52E-06	AGAP3	protein_cc
ENSG0000	7994.668	-0.05503	0.038335	-1.43553	0.151136	0.220221	EPN1	protein_cc
ENSG0000	7990.585	0.001514	0.025989	0.058246	0.953553	0.967886	OCIAD1	protein_cc
ENSG0000	7989.032	-0.23414	0.033628	-6.96263	3.34E-12	3.21E-11	WSB2	protein_cc
ENSG0000	7987.077	0.473532	0.076196	6.214661	5.14E-10	3.50E-09	CALU	protein_cc
ENSG0000	7984.728	-0.07645	0.027373	-2.79298	0.005222	0.01109	SERP1	protein_cc
ENSG0000	7983.329	-0.31369	0.031925	-9.8258	8.72E-23	4.88E-21	ZBTB4	protein_cc
ENSG0000	7969.636	0.37458	0.05069	7.389576	1.47E-13	1.74E-12	MYL12B	protein_cc
ENSG0000	7956.7	-0.14882	0.028451	-5.2307	1.69E-07	7.72E-07	KIAA0100	protein_cc
ENSG0000	7950.438	0.333223	0.042612	7.819869	5.29E-15	8.10E-14	CDH2	protein_cc
ENSG0000	7949.615	-0.0562	0.022345	-2.51518	0.011897	0.023367	RTFDC1	protein_cc
ENSG0000	7949.371	-0.48258	0.06402	-7.53797	4.77E-14	6.13E-13	ARHGEF4	protein_cc
ENSG0000	7939.821	-0.24687	0.043869	-5.62748	1.83E-08	9.75E-08	HTATSF1	protein_cc
ENSG0000	7928.152	-0.13412	0.043485	-3.08428	0.00204	0.004701	PBX1	protein_cc
ENSG0000	7924.457	-0.00508	0.022671	-0.22397	0.822783	0.870203	RAB1A	protein_cc
ENSG0000	7922.767	-0.09429	0.037205	-2.53431	0.011267	0.022252	ABL1	protein_cc
ENSG0000	7922.424	0.199229	0.029912	6.660529	2.73E-11	2.27E-10	CCT4	protein_cc
ENSG0000	7918.606	0.365671	0.040692	8.986365	2.56E-19	7.81E-18	PTBP1	protein_cc
ENSG0000	7913.401	0.121098	0.095711	1.265254	0.20578	0.286099	CHL1	protein_cc
ENSG0000	7910.242	-0.49097	0.052268	-9.39329	5.82E-21	2.32E-19	ZMYND11	protein_cc
ENSG0000	7909.095	-0.11827	0.069498	-1.70183	0.088788	0.139385	SGK1	protein_cc
ENSG0000	7907.553	-0.19502	0.072338	-2.69594	0.007019	0.014497	BEX1	protein_cc
ENSG0000	7906.95	-0.21343	0.036455	-5.85464	4.78E-09	2.79E-08	PRRC2C	protein_cc
ENSG0000	7901.909	1.135334	0.097065	11.69661	1.33E-31	4.01E-29	LANCL2	protein_cc
ENSG0000	7899.168	-0.04341	0.035667	-1.21716	0.223545	0.30664	RNH1	protein_cc
ENSG0000	7896.725	0.069071	0.038016	1.816899	0.069233	0.112246	ELOVL5	protein_cc
ENSG0000	7883.521	-0.51669	0.058115	-8.89093	6.06E-19	1.74E-17	PFKP	protein_cc
ENSG0000	7883.455	-0.23854	0.053892	-4.42628	9.59E-06	3.32E-05	ADIPOR2	protein_cc
ENSG0000	7881.138	0.031236	0.033699	0.926896	0.35398	0.449584	EIF3D	protein_cc
ENSG0000	7877.371	0.052569	0.031584	1.664441	0.096024	0.149167	BTBD2	protein_cc
ENSG0000	7870.213	-0.04593	0.035532	-1.29252	0.196176	0.274733	HYOU1	protein_cc
ENSG0000	7861.313	-0.20606	0.031818	-6.47636	9.40E-11	7.19E-10	USP9X	protein_cc
ENSG0000	7845.253	0.294834	0.043798	6.731702	1.68E-11	1.44E-10	EIF3I	protein_cc
ENSG0000	7838.28	-0.03409	0.034287	-0.99433	0.320064	0.413844	ACIN1	protein_cc
ENSG0000	7837.855	0.057191	0.038376	1.490284	0.13615	0.20123	TMEM123	protein_cc
ENSG0000	7835.887	-0.08736	0.028062	-3.11319	0.001851	0.004296	CTTN	protein_cc
ENSG0000	7833.392	-1.54105	0.15133	-10.1834	2.35E-24	1.81E-22	SYT1	protein_cc
ENSG0000	7832.018	-0.03386	0.029699	-1.14017	0.254216	0.341777	DVL3	protein_cc

ENSG0000(	7829.068	0.093066	0.024015	3.8753	0.000106	0.00031	KHDRBS1	protein_cc
ENSG0000(	7828.997	0.167841	0.124749	1.345425	0.178488	0.253547	C1R	protein_cc
ENSG0000(	7828.213	-0.05377	0.039973	-1.34522	0.178555	0.253624	SNX1	protein_cc
ENSG0000(	7825.885	0.139441	0.061566	2.264909	0.023518	0.043088	LINC0046:	lincRNA
ENSG0000(	7825.522	-0.43792	0.061978	-7.0657	1.60E-12	1.61E-11	TTYH2	protein_cc
ENSG0000(	7821.336	-0.13359	0.050397	-2.65068	0.008033	0.016401	SEMA6A	protein_cc
ENSG0000(	7820.186	-1.05524	0.105452	-10.0068	1.42E-23	9.53E-22	FBXL16	protein_cc
ENSG0000(	7806.215	-0.07707	0.047693	-1.61601	0.106091	0.16253	CTSF	protein_cc
ENSG0000(	7804.771	-0.09508	0.026295	-3.61583	0.000299	0.000806	PRPF19	protein_cc
ENSG0000(	7798.504	-0.01658	0.036095	-0.45932	0.646002	0.725286	PGRMC1	protein_cc
ENSG0000(	7796.452	-1.08353	0.116384	-9.30999	1.28E-20	4.82E-19	DNM1	protein_cc
ENSG0000(	7789.754	-0.3753	0.048388	-7.75601	8.76E-15	1.29E-13	LANCL1	protein_cc
ENSG0000(	7785.121	-0.15612	0.026238	-5.94994	2.68E-09	1.63E-08	PITPNA	protein_cc
ENSG0000(	7784.146	-0.24439	0.030629	-7.9791	1.47E-15	2.47E-14	PTK2	protein_cc
ENSG0000(	7781.147	-0.02904	0.024218	-1.19902	0.230521	0.314509	CTBP1	protein_cc
ENSG0000(	7780.056	0.1388	0.082141	1.689771	0.091072	0.142442	VWF	protein_cc
ENSG0000(	7778.545	0.1508	0.032313	4.666915	3.06E-06	1.14E-05	BRK1	protein_cc
ENSG0000(	7775.199	0.050551	0.06457	0.782884	0.433695	0.530712	PER1	protein_cc
ENSG0000(	7773.302	-0.14912	0.025479	-5.85238	4.85E-09	2.82E-08	SUPT6H	protein_cc
ENSG0000(	7770.958	-0.34032	0.039742	-8.56324	1.10E-17	2.61E-16	PPP1R9B	protein_cc
ENSG0000(	7770.568	-0.59305	0.106149	-5.58694	2.31E-08	1.21E-07	EEF1A2	protein_cc
ENSG0000(	7760.868	0.322658	0.058892	5.478849	4.28E-08	2.15E-07	PDE4B	protein_cc
ENSG0000(	7755.582	0.174243	0.077365	2.25223	0.024308	0.044408	GRIK3	protein_cc
ENSG0000(	7741.106	-0.08509	0.02553	-3.33301	0.000859	0.00212	ZFAND3	protein_cc
ENSG0000(	7739.017	0.049232	0.028028	1.756541	0.078996	0.125897	PSMD4	protein_cc
ENSG0000(	7727.487	-0.02093	0.035758	-0.58525	0.558382	0.647499	SLC38A2	protein_cc
ENSG0000(	7724.053	-0.08622	0.032289	-2.67017	0.007581	0.015562	C4orf3	protein_cc
ENSG0000(	7721.385	0.186544	0.053295	3.500218	0.000465	0.001207	NPDC1	protein_cc
ENSG0000(	7719.168	0.220161	0.027995	7.864288	3.71E-15	5.84E-14	SUMO2	protein_cc
ENSG0000(	7718.034	0.164572	0.046536	3.536409	0.000406	0.001066	CRIP2	protein_cc
ENSG0000(	7718.027	0.031092	0.025732	1.208304	0.22693	0.310455	CCDC47	protein_cc
ENSG0000(	7707.807	0.220303	0.070591	3.120831	0.001803	0.004197	PMP22	protein_cc
ENSG0000(	7697.835	0.836671	0.070722	11.83039	2.72E-32	9.56E-30	GBAS	protein_cc
ENSG0000(	7687.342	0.378731	0.086664	4.37011	1.24E-05	4.22E-05	CSPG4	protein_cc
ENSG0000(	7685.155	-0.0152	0.055799	-0.27242	0.785303	0.8402	SLC2A1	protein_cc
ENSG0000(	7685.071	-0.30023	0.038001	-7.9006	2.78E-15	4.47E-14	RERE	protein_cc
ENSG0000(	7680.207	0.417176	0.054105	7.71051	1.25E-14	1.78E-13	KDELR2	protein_cc
ENSG0000(	7679.464	0.135385	0.028715	4.714863	2.42E-06	9.20E-06	RBBP7	protein_cc
ENSG0000(	7676.148	-0.3144	0.030475	-10.3164	5.94E-25	5.13E-23	PPP2R5C	protein_cc
ENSG0000(	7671.008	1.247993	0.120737	10.33645	4.82E-25	4.28E-23	HOPX	protein_cc
ENSG0000(	7661.051	-0.02883	0.059589	-0.48377	0.628551	0.710027	NFIA	protein_cc
ENSG0000(	7659.256	1.243115	0.144761	8.58734	8.90E-18	2.16E-16	ANXA1	protein_cc
ENSG0000(	7650.098	0.407237	0.052976	7.687129	1.50E-14	2.11E-13	ODC1	protein_cc
ENSG0000(	7645.7	-0.73325	0.117332	-6.24939	4.12E-10	2.85E-09	BCAS1	protein_cc
ENSG0000(	7641.344	-0.25048	0.040887	-6.12607	9.01E-10	5.92E-09	GIT1	protein_cc
ENSG0000(	7633.295	-0.11737	0.025199	-4.65753	3.20E-06	1.19E-05	TRIP12	protein_cc
ENSG0000(	7631.624	-0.03473	0.049746	-0.6982	0.485054	0.580068	PKD1	protein_cc
ENSG0000(	7627.975	-0.02311	0.080239	-0.288	0.773348	0.830522	ASIC1	protein_cc
ENSG0000(	7627.508	-0.05533	0.040313	-1.3726	0.169877	0.243296	INPPL1	protein_cc
ENSG0000(	7621.404	0.210571	0.050375	4.18005	2.91E-05	9.32E-05	SHISA5	protein_cc
ENSG0000(	7621.167	0.573438	0.072855	7.871005	3.52E-15	5.56E-14	SUMF2	protein_cc
ENSG0000(	7618.57	-0.11862	0.035977	-3.29708	0.000977	0.002384	IVNS1ABP	protein_cc
ENSG0000(	7617.703	-0.60791	0.058996	-10.3043	6.74E-25	5.75E-23	ALDH6A1	protein_cc
ENSG0000(	7608.139	-0.39459	0.05573	-7.08034	1.44E-12	1.46E-11	SALL2	protein_cc
ENSG0000(	7605.998	-0.19698	0.087512	-2.25085	0.024395	0.04455	LINGO1	protein_cc
ENSG0000(	7602.806	0.061512	0.091671	0.67101	0.502214	0.596117	NEAT1	lincRNA
ENSG0000(	7598.223	-0.44848	0.055232	-8.11986	4.67E-16	8.51E-15	FAM171B	protein_cc
ENSG0000(	7597.562	0.257506	0.039015	6.600151	4.11E-11	3.32E-10	TMED2	protein_cc
ENSG0000(	7593.194	0.152395	0.037344	4.080832	4.49E-05	0.000139	ACTR3	protein_cc

ENSG0000(	7590.389	-0.24873	0.032601	-7.62948	2.36E-14	3.19E-13	MFN2	protein_cc
ENSG0000(	7583.678	-0.48137	0.093755	-5.13432	2.83E-07	1.25E-06	hsa-mir-6	pseudoge
ENSG0000(	7570.076	-0.01055	0.023571	-0.44745	0.65455	0.732494	RAD23B	protein_cc
ENSG0000(	7568.74	-0.15859	0.033517	-4.73165	2.23E-06	8.52E-06	SAP18	protein_cc
ENSG0000(	7567.003	-0.13916	0.031742	-4.38409	1.16E-05	3.98E-05	PCM1	protein_cc
ENSG0000(	7560.523	0.25562	0.103435	2.471308	0.013462	0.026116	ACTN1	protein_cc
ENSG0000(	7553.728	-0.09099	0.031388	-2.89902	0.003743	0.008183	GTF3C1	protein_cc
ENSG0000(	7549.284	0.50698	0.083622	6.062782	1.34E-09	8.55E-09	RCAN1	protein_cc
ENSG0000(	7538.484	-0.00133	0.039934	-0.03334	0.973404	0.981398	ARHGEF2	protein_cc
ENSG0000(	7534.38	-0.20965	0.040037	-5.23629	1.64E-07	7.50E-07	ASPH	protein_cc
ENSG0000(	7531.848	0.201847	0.035042	5.760127	8.41E-09	4.72E-08	ERGIC1	protein_cc
ENSG0000(	7528.722	-0.2708	0.065511	-4.13358	3.57E-05	0.000113	QDPR	protein_cc
ENSG0000(	7526.356	0.258728	0.036022	7.182562	6.84E-13	7.30E-12	TMEM248	protein_cc
ENSG0000(	7518.664	-0.29203	0.040491	-7.21229	5.50E-13	5.94E-12	NCOA1	protein_cc
ENSG0000(	7518.434	0.146681	0.0353	4.155218	3.25E-05	0.000103	TMEM259	protein_cc
ENSG0000(	7517.817	-0.19701	0.03399	-5.79598	6.79E-09	3.87E-08	AP2A1	protein_cc
ENSG0000(	7509.46	-0.49232	0.043759	-11.2509	2.29E-29	4.60E-27	TACC1	protein_cc
ENSG0000(	7491.388	0.032059	0.034323	0.934021	0.350293	0.445741	TECR	protein_cc
ENSG0000(	7487.029	0.304439	0.045671	6.665918	2.63E-11	2.19E-10	PPIB	protein_cc
ENSG0000(	7483.745	-0.0101	0.032343	-0.3122	0.754886	0.815315	CAND1	protein_cc
ENSG0000(	7481.755	0.252882	0.039958	6.328754	2.47E-10	1.77E-09	RBM3	protein_cc
ENSG0000(	7479.835	0.032541	0.04562	0.713299	0.475661	0.571493	JUND	protein_cc
ENSG0000(	7476.051	-0.26402	0.037984	-6.95074	3.63E-12	3.46E-11	G3BP2	protein_cc
ENSG0000(	7471.726	0.053843	0.028891	1.863649	0.062371	0.102458	STIP1	protein_cc
ENSG0000(	7466.94	-1.40199	0.171552	-8.17241	3.02E-16	5.68E-15	SLC17A7	protein_cc
ENSG0000(	7461.962	-0.29494	0.059502	-4.95681	7.17E-07	2.96E-06	MGLL	protein_cc
ENSG0000(	7455.284	-0.10664	0.023779	-4.48472	7.30E-06	2.58E-05	OAZ2	protein_cc
ENSG0000(	7453.901	-0.02358	0.031006	-0.76047	0.446976	0.543813	ARHGAP1	protein_cc
ENSG0000(	7453.328	0.039909	0.026012	1.534235	0.124972	0.187089	STRAP	protein_cc
ENSG0000(	7449.567	-0.32652	0.042884	-7.61395	2.66E-14	3.56E-13	SGSM2	protein_cc
ENSG0000(	7448.958	0.072545	0.040397	1.795805	0.072525	0.116907	TAPBP	protein_cc
ENSG0000(	7447.02	0.024165	0.031329	0.771317	0.440519	0.537468	UBXN6	protein_cc
ENSG0000(	7446.422	-0.61815	0.07374	-8.38279	5.17E-17	1.10E-15	PAQR8	protein_cc
ENSG0000(	7440.22	-0.00648	0.033066	-0.19583	0.84474	0.887351	ENSA	protein_cc
ENSG0000(	7440.167	-0.62712	0.053532	-11.7149	1.07E-31	3.33E-29	CBX6	protein_cc
ENSG0000(	7438.073	1.203809	0.244191	4.929793	8.23E-07	3.37E-06	LTF	protein_cc
ENSG0000(	7435.145	-0.2712	0.032217	-8.41784	3.84E-17	8.37E-16	RAPGEF1	protein_cc
ENSG0000(	7426.87	-0.10641	0.058342	-1.82398	0.068155	0.110677	PCBP4	protein_cc
ENSG0000(	7426.744	-0.08703	0.029275	-2.97278	0.002951	0.006588	PUM2	protein_cc
ENSG0000(	7424.404	0.372009	0.058335	6.377111	1.80E-10	1.32E-09	RPS10	protein_cc
ENSG0000(	7420.292	0.128725	0.034192	3.764749	0.000167	0.000469	FBXL5	protein_cc
ENSG0000(	7418.853	-0.04478	0.024589	-1.82111	0.06859	0.111305	DNM2	protein_cc
ENSG0000(	7415.346	-0.11696	0.035951	-3.25337	0.00114	0.00275	POLDIP3	protein_cc
ENSG0000(	7413.982	-0.00805	0.027328	-0.29472	0.768206	0.826121	SMARCA5	protein_cc
ENSG0000(	7409.552	0.158558	0.029933	5.297028	1.18E-07	5.52E-07	SNX17	protein_cc
ENSG0000(	7407.639	0.166209	0.034502	4.817382	1.45E-06	5.74E-06	AKR1B1	protein_cc
ENSG0000(	7403.627	0.122697	0.034217	3.58587	0.000336	0.000896	TNPO1	protein_cc
ENSG0000(	7402.391	0.723901	0.06953	10.41127	2.20E-25	2.05E-23	TRIB2	protein_cc
ENSG0000(	7401.41	0.233758	0.041484	5.634896	1.75E-08	9.36E-08	ATP5F1	protein_cc
ENSG0000(	7396.843	-0.18468	0.047092	-3.92181	8.79E-05	0.00026	ARHGEF7	protein_cc
ENSG0000(	7388.493	-0.53632	0.0635	-8.44598	3.02E-17	6.69E-16	SYNE1	protein_cc
ENSG0000(	7383.725	-0.24432	0.072664	-3.36227	0.000773	0.001923	HMGCS1	protein_cc
ENSG0000(	7383.194	0.063239	0.028264	2.237472	0.025255	0.045952	SYNCRIP	protein_cc
ENSG0000(	7383.073	-0.12303	0.035913	-3.42591	0.000613	0.001554	NHP2L1	protein_cc
ENSG0000(	7380.338	-0.77657	0.15389	-5.04624	4.51E-07	1.92E-06	MOBP	protein_cc
ENSG0000(	7377.765	-0.13734	0.043929	-3.12646	0.001769	0.004123	RBPJ	protein_cc
ENSG0000(	7373.9	-0.31319	0.053368	-5.86853	4.40E-09	2.58E-08	NF1	protein_cc
ENSG0000(	7369.381	-0.45385	0.053265	-8.52063	1.59E-17	3.67E-16	PREPL	protein_cc
ENSG0000(	7365.442	-0.25909	0.043491	-5.95743	2.56E-09	1.56E-08	FAM102A	protein_cc

ENSG0000(	7361.796	-0.72855	0.127245	-5.72558	1.03E-08	5.70E-08	CNTN2	protein_cc
ENSG0000(	7358.998	-0.22909	0.041587	-5.50863	3.62E-08	1.84E-07	ATP6V1E1	protein_cc
ENSG0000(	7352.205	-0.23902	0.034505	-6.92724	4.29E-12	4.05E-11	GNAQ	protein_cc
ENSG0000(	7347.582	0.057769	0.024748	2.334323	0.019579	0.036515	PRMT2	protein_cc
ENSG0000(	7345.154	0.106666	0.026851	3.972455	7.11E-05	0.000213	POLR2E	protein_cc
ENSG0000(	7333.591	0.209431	0.059051	3.546616	0.00039	0.001029	CHPF	protein_cc
ENSG0000(	7333.409	0.095159	0.039353	2.418111	0.015601	0.029818	FAM195B	protein_cc
ENSG0000(	7329.895	-0.43521	0.047254	-9.21006	3.26E-20	1.15E-18	KIAA0930	protein_cc
ENSG0000(	7322.35	-0.01505	0.03626	-0.41517	0.678019	0.752168	STAG2	protein_cc
ENSG0000(	7318.182	0.160036	0.02865	5.585928	2.32E-08	1.22E-07	PPP1CC	protein_cc
ENSG0000(	7317.956	-0.11219	0.031758	-3.53274	0.000411	0.001079	FAM192A	protein_cc
ENSG0000(	7313.935	-0.10292	0.032446	-3.17201	0.001514	0.003573	LRPPRC	protein_cc
ENSG0000(	7307.222	0.06549	0.040455	1.618825	0.105485	0.161744	OGT	protein_cc
ENSG0000(	7297.001	-0.03139	0.034648	-0.90604	0.364915	0.460966	ZNF24	protein_cc
ENSG0000(	7296.654	-0.01787	0.046837	-0.38143	0.702882	0.773035	ANKIB1	protein_cc
ENSG0000(	7294.197	0.349716	0.049458	7.070958	1.54E-12	1.56E-11	DDOST	protein_cc
ENSG0000(	7291.925	-0.21438	0.028178	-7.60817	2.78E-14	3.71E-13	HSD17B4	protein_cc
ENSG0000(	7291.633	-0.05605	0.022447	-2.49714	0.01252	0.024449	GSPT1	protein_cc
ENSG0000(	7281.222	-0.19242	0.024947	-7.71343	1.22E-14	1.75E-13	NMT1	protein_cc
ENSG0000(	7277.628	0.031809	0.025481	1.248342	0.211906	0.293227	COPG1	protein_cc
ENSG0000(	7258.86	-0.07097	0.032447	-2.18724	0.028725	0.051603	ZMIZ2	protein_cc
ENSG0000(	7256.517	-0.01376	0.057613	-0.23881	0.811252	0.861242	TSC22D3	protein_cc
ENSG0000(	7255.829	-0.12491	0.043358	-2.88084	0.003966	0.008634	FDFT1	protein_cc
ENSG0000(	7253.31	-0.12423	0.026968	-4.60647	4.10E-06	1.50E-05	UBQLN1	protein_cc
ENSG0000(	7251.898	0.125176	0.028782	4.349026	1.37E-05	4.62E-05	GLO1	protein_cc
ENSG0000(	7247.316	0.088163	0.02884	3.05697	0.002236	0.005112	TM9SF2	protein_cc
ENSG0000(	7246.884	-0.60935	0.069915	-8.71554	2.89E-18	7.53E-17	MYO5A	protein_cc
ENSG0000(	7240.914	0.543302	0.050816	10.69155	1.11E-26	1.36E-24	MCM7	protein_cc
ENSG0000(	7235.294	-0.21253	0.048929	-4.34364	1.40E-05	4.73E-05	KMT2C	protein_cc
ENSG0000(	7233.913	-0.05753	0.037854	-1.51981	0.128558	0.191622	SUPT16H	protein_cc
ENSG0000(	7227.655	-0.67234	0.059398	-11.3192	1.05E-29	2.20E-27	PI4KA	protein_cc
ENSG0000(	7225.826	0.064147	0.021368	3.002029	0.002682	0.006037	PSMF1	protein_cc
ENSG0000(	7222.873	-0.08194	0.034689	-2.36212	0.018171	0.034164	MXD4	protein_cc
ENSG0000(	7222.511	-0.05725	0.040247	-1.42238	0.154917	0.224942	SORBS3	protein_cc
ENSG0000(	7221.612	-0.69552	0.082239	-8.45721	2.74E-17	6.14E-16	BASP1	protein_cc
ENSG0000(	7217.693	0.554599	0.081391	6.813992	9.49E-12	8.46E-11	S100A16	protein_cc
ENSG0000(	7216.466	0.207183	0.043649	4.746548	2.07E-06	7.95E-06	SELT	protein_cc
ENSG0000(	7211.809	0.327222	0.042623	7.677127	1.63E-14	2.27E-13	PSMA7	protein_cc
ENSG0000(	7211.109	-0.381	0.076556	-4.97679	6.46E-07	2.69E-06	USP54	protein_cc
ENSG0000(	7206.036	-0.12836	0.034389	-3.73257	0.00019	0.000528	ARFGAP2	protein_cc
ENSG0000(	7205.114	0.062544	0.038117	1.640834	0.100832	0.155642	SRSF11	protein_cc
ENSG0000(	7203.663	-0.01516	0.025043	-0.60529	0.544983	0.635474	PAPOLA	protein_cc
ENSG0000(	7200.967	-0.13393	0.031433	-4.26069	2.04E-05	6.70E-05	ATP6V0D1	protein_cc
ENSG0000(	7195.351	-0.15695	0.028683	-5.47167	4.46E-08	2.23E-07	CDV3	protein_cc
ENSG0000(	7191.828	0.141655	0.048194	2.939288	0.00329	0.007274	SGCB	protein_cc
ENSG0000(	7191.586	-0.04466	0.049912	-0.89479	0.370902	0.467294	UBXN1	protein_cc
ENSG0000(	7188.075	0.24769	0.063454	3.903456	9.48E-05	0.000278	RAMP1	protein_cc
ENSG0000(	7187.784	-0.17726	0.042202	-4.20035	2.67E-05	8.58E-05	ZKSCAN1	protein_cc
ENSG0000(	7186.465	0.015414	0.022413	0.687707	0.491638	0.586117	KIAA1191	protein_cc
ENSG0000(	7186.149	0.100078	0.031613	3.165662	0.001547	0.003646	BAZ1B	protein_cc
ENSG0000(	7186.027	-0.43125	0.094572	-4.56007	5.11E-06	1.85E-05	ENHO	protein_cc
ENSG0000(	7179.517	0.115569	0.05719	2.020776	0.043303	0.074398	BMP7	protein_cc
ENSG0000(	7174.637	0.02164	0.022174	0.975897	0.329115	0.42355	RBM8A	protein_cc
ENSG0000(	7174.095	0.341078	0.14011	2.434361	0.014918	0.028645	COL1A2	protein_cc
ENSG0000(	7167.056	-0.064	0.027356	-2.33952	0.019308	0.03607	TMEM167	protein_cc
ENSG0000(	7161.272	-0.02327	0.029649	-0.78498	0.432466	0.529433	MAVS	protein_cc
ENSG0000(	7157.109	0.227867	0.043068	5.290844	1.22E-07	5.69E-07	FIS1	protein_cc
ENSG0000(	7151.433	-0.09481	0.064796	-1.46314	0.14343	0.21047	COLGALT:	protein_cc
ENSG0000(	7150.577	-0.1943	0.0603	-3.22216	0.001272	0.003043	MLLT11	protein_cc

ENSG0000(	7150.33	0.121847	0.021359	5.70479	1.16E-08	6.40E-08	PSMD3	protein_co
ENSG0000(	7145.739	0.006803	0.022547	0.301727	0.76286	0.821963	AAMP	protein_co
ENSG0000(	7145.243	-0.1918	0.037704	-5.08696	3.64E-07	1.58E-06	KDM3B	protein_co
ENSG0000(	7137.886	-0.20021	0.04006	-4.99787	5.80E-07	2.43E-06	RNF11	protein_co
ENSG0000(	7134.095	0.113099	0.03326	3.400433	0.000673	0.001694	TMEM9	protein_co
ENSG0000(	7128.944	0.246722	0.030583	8.06732	7.19E-16	1.27E-14	COPZ1	protein_co
ENSG0000(	7126.29	-0.12301	0.032411	-3.79526	0.000147	0.000418	RNF130	protein_co
ENSG0000(	7125.2	-0.10521	0.027316	-3.85156	0.000117	0.000339	EPRS	protein_co
ENSG0000(	7122.113	-0.64916	0.06168	-10.5246	6.66E-26	6.98E-24	EPB41L1	protein_co
ENSG0000(	7119.738	-0.25807	0.041858	-6.16533	7.03E-10	4.70E-09	BACE1	protein_co
ENSG0000(	7117.262	0.222407	0.032508	6.841681	7.83E-12	7.06E-11	TMX2	protein_co
ENSG0000(	7112.066	-0.2423	0.068386	-3.54314	0.000395	0.001041	ZNF385A	protein_co
ENSG0000(	7111.135	-0.31304	0.062468	-5.01124	5.41E-07	2.28E-06	LPHN3	protein_co
ENSG0000(	7104.188	-0.24466	0.182291	-1.34215	0.179547	0.254707	COL3A1	protein_co
ENSG0000(	7101.967	0.128722	0.050253	2.561485	0.010423	0.020741	ACSL3	protein_co
ENSG0000(	7100.557	0.505079	0.099277	5.087555	3.63E-07	1.57E-06	FSTL1	protein_co
ENSG0000(	7096.774	-0.33228	0.043858	-7.57626	3.56E-14	4.67E-13	RNF141	protein_co
ENSG0000(	7087.953	0.176959	0.043995	4.022266	5.76E-05	0.000176	MYO9B	protein_co
ENSG0000(	7085.984	0.582267	0.066306	8.781488	1.61E-18	4.34E-17	PDIA4	protein_co
ENSG0000(	7084.687	-0.10544	0.044226	-2.38414	0.017119	0.032387	SCRIB	protein_co
ENSG0000(	7083.887	-0.22597	0.048153	-4.69274	2.70E-06	1.02E-05	CAMSAP2	protein_co
ENSG0000(	7080.063	0.17425	0.041362	4.212792	2.52E-05	8.14E-05	ARPC3	protein_co
ENSG0000(	7079.706	1.167801	0.138517	8.430755	3.43E-17	7.54E-16	IGFBP3	protein_co
ENSG0000(	7076.693	-0.14222	0.046708	-3.04481	0.002328	0.005305	REEP2	protein_co
ENSG0000(	7076.253	-0.07101	0.023598	-3.00911	0.00262	0.005912	RAB11A	protein_co
ENSG0000(	7074.78	0.028556	0.0537	0.531775	0.594882	0.680158	LMO4	protein_co
ENSG0000(	7071.065	-0.20888	0.057497	-3.63285	0.00028	0.000758	AHCYL2	protein_co
ENSG0000(	7067.508	-0.73277	0.06467	-11.3309	9.23E-30	1.95E-27	CAMK2G	protein_co
ENSG0000(	7062.974	0.266131	0.062934	4.228737	2.35E-05	7.63E-05	GNS	protein_co
ENSG0000(	7061.165	-0.09695	0.023418	-4.14006	3.47E-05	0.00011	PAFAH1B2	protein_co
ENSG0000(	7051.631	-0.43114	0.048342	-8.91848	4.73E-19	1.38E-17	ARHGAP5	protein_co
ENSG0000(	7045.185	-0.0784	0.026853	-2.91974	0.003503	0.007707	ARCN1	protein_co
ENSG0000(	7044.678	0.17178	0.11474	1.497125	0.134361	0.198943	HLA-DPB1	protein_co
ENSG0000(	7037.629	-0.28843	0.03735	-7.72225	1.14E-14	1.64E-13	PLEKHM2	protein_co
ENSG0000(	7037.056	-0.33334	0.051219	-6.50818	7.61E-11	5.91E-10	EPHX1	protein_co
ENSG0000(	7036.21	-0.11533	0.044829	-2.57276	0.010089	0.02013	CREBBP	protein_co
ENSG0000(	7029.593	-0.15588	0.033764	-4.6169	3.90E-06	1.44E-05	DARS	protein_co
ENSG0000(	7025.672	0.367417	0.044224	8.308131	9.72E-17	1.98E-15	POU3F3	protein_co
ENSG0000(	7024.231	-0.06832	0.082128	-0.83189	0.405471	0.502353	MASP1	protein_co
ENSG0000(	7021.83	0.49282	0.058537	8.419006	3.80E-17	8.29E-16	15-Sep	protein_co
ENSG0000(	7021.253	-0.37742	0.047437	-7.95628	1.77E-15	2.94E-14	SMARCA2	protein_co
ENSG0000(	7020.371	-0.26292	0.04107	-6.40168	1.54E-10	1.13E-09	EVL	protein_co
ENSG0000(	7019.89	-0.01646	0.035832	-0.45928	0.646037	0.725286	ZNF532	protein_co
ENSG0000(	7017.595	-0.14849	0.053195	-2.79137	0.005249	0.011142	BCL6	protein_co
ENSG0000(	7006.223	0.07417	0.041465	1.788763	0.073653	0.118456	TNRC18	protein_co
ENSG0000(	7000.938	0.120871	0.021557	5.607105	2.06E-08	1.09E-07	SRSF1	protein_co
ENSG0000(	6998.893	-0.18625	0.034663	-5.37316	7.74E-08	3.73E-07	KCTD20	protein_co
ENSG0000(	6994.148	-0.42546	0.050029	-8.50442	1.83E-17	4.19E-16	HK1	protein_co
ENSG0000(	6985.787	-0.44671	0.052445	-8.51757	1.63E-17	3.76E-16	WDFY3	protein_co
ENSG0000(	6984.691	-0.39832	0.049522	-8.04315	8.76E-16	1.52E-14	SECISBP2L	protein_co
ENSG0000(	6982.795	-0.10078	0.035623	-2.82908	0.004668	0.01	MED29	protein_co
ENSG0000(	6978.781	0.271608	0.032578	8.337151	7.61E-17	1.58E-15	COPS6	protein_co
ENSG0000(	6973.196	0.009979	0.022675	0.440058	0.659895	0.736932	STAU1	protein_co
ENSG0000(	6964.384	-0.0779	0.08579	-0.90803	0.363861	0.459833	RASD1	protein_co
ENSG0000(	6958.708	-0.08381	0.027677	-3.02823	0.00246	0.005584	ANP32A	protein_co
ENSG0000(	6958.182	0.09421	0.02953	3.190287	0.001421	0.003371	DBNL	protein_co
ENSG0000(	6957.661	-0.08773	0.02703	-3.24585	0.001171	0.002816	VPS26B	protein_co
ENSG0000(	6957.36	-0.13356	0.03553	-3.75921	0.00017	0.000478	TPR	protein_co
ENSG0000(	6951.241	-0.03426	0.038537	-0.88895	0.374028	0.470585	BBS2	protein_co

ENSG0000(	6950.459	-0.0692	0.026017	-2.65977	0.007819	0.016008	GPR107	protein_cc
ENSG0000(	6948.69	-0.16925	0.035943	-4.70877	2.49E-06	9.46E-06	TCEAL4	protein_cc
ENSG0000(	6942.401	0.21543	0.030226	7.127408	1.02E-12	1.06E-11	RALY	protein_cc
ENSG0000(	6933.992	-0.34293	0.049167	-6.97474	3.06E-12	2.96E-11	TOB2	protein_cc
ENSG0000(	6933.339	0.089034	0.043201	2.060903	0.039312	0.068321	CPNE2	protein_cc
ENSG0000(	6929.491	-0.3453	0.046825	-7.37441	1.65E-13	1.94E-12	TRIM8	protein_cc
ENSG0000(	6925.175	-0.07316	0.035491	-2.06129	0.039276	0.068269	XPOT	protein_cc
ENSG0000(	6924.811	0.224742	0.048529	4.631123	3.64E-06	1.35E-05	ATP6V0E1	protein_cc
ENSG0000(	6919.172	-0.69526	0.081184	-8.56393	1.09E-17	2.59E-16	NEBL	protein_cc
ENSG0000(	6917.677	0.292208	0.116268	2.513227	0.011963	0.023477	FGFR3	protein_cc
ENSG0000(	6914.829	-0.40459	0.052818	-7.65997	1.86E-14	2.57E-13	NR2F1	protein_cc
ENSG0000(	6910.883	-0.14606	0.05174	-2.823	0.004758	0.010175	VANGL2	protein_cc
ENSG0000(	6906.902	-0.30583	0.051085	-5.98657	2.14E-09	1.33E-08	SLC7A1	protein_cc
ENSG0000(	6906.684	-0.66695	0.089019	-7.49227	6.77E-14	8.50E-13	KIF21B	protein_cc
ENSG0000(	6905.848	-0.33701	0.06766	-4.98096	6.33E-07	2.64E-06	SREBF1	protein_cc
ENSG0000(	6905	-0.03354	0.023126	-1.45041	0.146945	0.214914	RAB5C	protein_cc
ENSG0000(	6897.553	-0.04608	0.070954	-0.64943	0.51606	0.608896	ALDH7A1	protein_cc
ENSG0000(	6892.253	-0.28817	0.060929	-4.72964	2.25E-06	8.60E-06	FCHSD2	protein_cc
ENSG0000(	6889.554	-0.12218	0.035275	-3.46361	0.000533	0.001368	ATG13	protein_cc
ENSG0000(	6884.546	-0.40142	0.04864	-8.25283	1.55E-16	3.05E-15	TOLLIP	protein_cc
ENSG0000(	6877.988	-0.18675	0.038477	-4.8536	1.21E-06	4.84E-06	USP47	protein_cc
ENSG0000(	6872.633	0.07986	0.027961	2.856092	0.004289	0.009268	CDC37	protein_cc
ENSG0000(	6869.258	0.027222	0.024907	1.09292	0.274429	0.364441	G3BP1	protein_cc
ENSG0000(	6867.604	-0.01344	0.035282	-0.38103	0.70318	0.77333	LRPAP1	protein_cc
ENSG0000(	6864.134	0.269932	0.047373	5.698022	1.21E-08	6.64E-08	CYCS	protein_cc
ENSG0000(	6863.215	0.192046	0.046847	4.099429	4.14E-05	0.000129	SSR3	protein_cc
ENSG0000(	6859.62	0.044535	0.024323	1.830993	0.067102	0.109196	MKRN1	protein_cc
ENSG0000(	6857.535	0.193938	0.055167	3.515472	0.000439	0.001146	CD164	protein_cc
ENSG0000(	6855.716	-0.01295	0.066519	-0.19462	0.845687	0.888124	PODXL2	protein_cc
ENSG0000(	6855.54	-0.09402	0.056633	-1.66021	0.096873	0.150301	SMOX	protein_cc
ENSG0000(	6854.336	0.231237	0.041106	5.625416	1.85E-08	9.85E-08	HM13	protein_cc
ENSG0000(	6852.983	-0.12795	0.024063	-5.31724	1.05E-07	4.98E-07	GLYR1	protein_cc
ENSG0000(	6850.476	0.284466	0.038209	7.445042	9.69E-14	1.18E-12	ARF5	protein_cc
ENSG0000(	6848.839	-0.04126	0.035802	-1.15238	0.249167	0.336025	SPECC1	protein_cc
ENSG0000(	6840.507	-0.03431	0.025917	-1.32369	0.185607	0.261999	DHX30	protein_cc
ENSG0000(	6835.146	-0.06482	0.022853	-2.83634	0.004563	0.009797	PCBP1	protein_cc
ENSG0000(	6833.328	-0.22337	0.049927	-4.47382	7.68E-06	2.70E-05	SLC20A2	protein_cc
ENSG0000(	6828.852	0.106692	0.080307	1.328547	0.183998	0.260042	NPC2	protein_cc
ENSG0000(	6828.462	-0.19368	0.028865	-6.70977	1.95E-11	1.65E-10	IMPAD1	protein_cc
ENSG0000(	6825.783	-0.23736	0.045456	-5.22165	1.77E-07	8.07E-07	RNF213	protein_cc
ENSG0000(	6824.95	-0.10671	0.028093	-3.79853	0.000146	0.000413	DCTN5	protein_cc
ENSG0000(	6824.214	0.019574	0.027462	0.712784	0.475979	0.571709	FAM120A	protein_cc
ENSG0000(	6822.948	-0.29629	0.059922	-4.94466	7.63E-07	3.14E-06	KMT2A	protein_cc
ENSG0000(	6816.586	-0.11302	0.027715	-4.07782	4.55E-05	0.000141	CBX1	protein_cc
ENSG0000(	6816.455	0.045213	0.031206	1.448838	0.147383	0.215493	AKT1	protein_cc
ENSG0000(	6815.223	0.096864	0.025581	3.786613	0.000153	0.000432	GPS1	protein_cc
ENSG0000(	6812.294	0.138935	0.029506	4.708678	2.49E-06	9.47E-06	C11orf58	protein_cc
ENSG0000(	6811.586	0.119307	0.038056	3.135	0.001719	0.004016	PGD	protein_cc
ENSG0000(	6809.173	0.165286	0.03408	4.849869	1.24E-06	4.92E-06	PSMC3	protein_cc
ENSG0000(	6803.789	-0.28079	0.066898	-4.19727	2.70E-05	8.69E-05	ELMO1	protein_cc
ENSG0000(	6797.335	0.050621	0.036071	1.403359	0.16051	0.231672	IRAK1	protein_cc
ENSG0000(	6797.179	-0.17126	0.038604	-4.43638	9.15E-06	3.18E-05	AFF4	protein_cc
ENSG0000(	6796.2	-0.30744	0.049718	-6.18375	6.26E-10	4.21E-09	SPEN	protein_cc
ENSG0000(	6788.409	-0.1542	0.030675	-5.02694	4.98E-07	2.11E-06	THRAP3	protein_cc
ENSG0000(	6786.379	-0.13512	0.043028	-3.14026	0.001688	0.003949	C16orf45	protein_cc
ENSG0000(	6785.597	-0.53983	0.119956	-4.50024	6.79E-06	2.41E-05	GALNT13	protein_cc
ENSG0000(	6782.025	0.158254	0.044314	3.571188	0.000355	0.000944	C6orf48	protein_cc
ENSG0000(	6782.008	0.044899	0.07515	0.597455	0.550203	0.640127	ROBO1	protein_cc
ENSG0000(	6781.287	0.087881	0.036751	2.391236	0.016792	0.031849	VPS28	protein_cc

ENSG0000(	6779.195	0.295274	0.036496	8.090561	5.94E-16	1.06E-14	POLD2	protein_cc
ENSG0000(	6779.116	-0.01591	0.025886	-0.61476	0.538713	0.629698	NCBP2	protein_cc
ENSG0000(	6770.757	0.136675	0.028897	4.729787	2.25E-06	8.60E-06	PCNP	protein_cc
ENSG0000(	6767.332	-0.0806	0.030179	-2.67084	0.007566	0.015536	BAP1	protein_cc
ENSG0000(	6767.201	-0.12188	0.049398	-2.46726	0.013615	0.026386	KAT2B	protein_cc
ENSG0000(	6764.804	-0.15638	0.03237	-4.83099	1.36E-06	5.38E-06	SAMD4B	protein_cc
ENSG0000(	6764.317	-0.0426	0.025171	-1.69253	0.090545	0.141726	CPSF7	protein_cc
ENSG0000(	6763.823	-0.20599	0.043801	-4.703	2.56E-06	9.71E-06	LDOC1L	protein_cc
ENSG0000(	6762.197	0.243873	0.06272	3.88825	0.000101	0.000295	ADAM9	protein_cc
ENSG0000(	6761.857	-0.06237	0.039364	-1.58457	0.113063	0.171763	EHMT2	protein_cc
ENSG0000(	6761.056	0.282362	0.0628	4.496205	6.92E-06	2.45E-05	LPCAT1	protein_cc
ENSG0000(	6758.826	-0.51826	0.052884	-9.80002	1.13E-22	6.16E-21	TSPYL4	protein_cc
ENSG0000(	6758.002	-0.14951	0.02399	-6.23216	4.60E-10	3.16E-09	VPS39	protein_cc
ENSG0000(	6747.331	0.058864	0.022115	2.661762	0.007773	0.015923	RAF1	protein_cc
ENSG0000(	6741.735	0.037423	0.076257	0.490743	0.623608	0.705956	LRRN1	protein_cc
ENSG0000(	6738.375	0.135404	0.040965	3.3054	0.000948	0.00232	TBCB	protein_cc
ENSG0000(	6733.261	-0.02472	0.036186	-0.68318	0.49449	0.588835	ARHGEF4(	protein_cc
ENSG0000(	6729.389	-0.17917	0.024165	-7.41427	1.22E-13	1.47E-12	NPEPPS	protein_cc
ENSG0000(	6726.307	0.061293	0.036797	1.665707	0.095772	0.148816	CPNE3	protein_cc
ENSG0000(	6726.136	-0.18566	0.029521	-6.28902	3.19E-10	2.25E-09	ARL8B	protein_cc
ENSG0000(	6721.476	0.319653	0.059388	5.382478	7.35E-08	3.55E-07	LIMA1	protein_cc
ENSG0000(	6713.155	0.227765	0.041594	5.475894	4.35E-08	2.19E-07	COX8A	protein_cc
ENSG0000(	6711.985	-0.2367	0.030717	-7.7058	1.30E-14	1.84E-13	NDUFS1	protein_cc
ENSG0000(	6710.821	-0.66868	0.057405	-11.6485	2.34E-31	6.86E-29	GABARAP	protein_cc
ENSG0000(	6704.934	0.158461	0.045408	3.489715	0.000484	0.001251	CDK5RAP:	protein_cc
ENSG0000(	6703.423	-0.29956	0.039989	-7.49104	6.83E-14	8.58E-13	TBC1D5	protein_cc
ENSG0000(	6700.854	-0.35316	0.044924	-7.86128	3.80E-15	5.98E-14	PPP2R2B	protein_cc
ENSG0000(	6697.045	0.633795	0.075671	8.375632	5.49E-17	1.17E-15	CD151	protein_cc
ENSG0000(	6696.239	0.213439	0.029487	7.238367	4.54E-13	4.96E-12	PDAP1	protein_cc
ENSG0000(	6694.942	0.124048	0.06312	1.965285	0.049381	0.083574	FCGRT	protein_cc
ENSG0000(	6691.99	-0.15453	0.177428	-0.87092	0.383797	0.480474	COL1A1	protein_cc
ENSG0000(	6690.836	0.120818	0.024824	4.866943	1.13E-06	4.54E-06	ANAPC5	protein_cc
ENSG0000(	6690.558	-0.03154	0.033415	-0.94392	0.345211	0.440631	PTP4A1	protein_cc
ENSG0000(	6683.265	-0.15639	0.026471	-5.90802	3.46E-09	2.07E-08	USP7	protein_cc
ENSG0000(	6681.505	-0.03749	0.03433	-1.092	0.274835	0.364897	SKI	protein_cc
ENSG0000(	6678.699	0.133191	0.042138	3.160873	0.001573	0.003701	SH3GLB1	protein_cc
ENSG0000(	6677.693	0.044957	0.027364	1.642915	0.1004	0.155063	COPB2	protein_cc
ENSG0000(	6676.966	0.12001	0.039017	3.075844	0.002099	0.004823	SMARCD3	protein_cc
ENSG0000(	6671.909	-0.43982	0.048643	-9.04168	1.54E-19	4.90E-18	BEX4	protein_cc
ENSG0000(	6670.052	-0.15723	0.043394	-3.6233	0.000291	0.000785	NEO1	protein_cc
ENSG0000(	6667.131	0.256693	0.054114	4.743572	2.10E-06	8.07E-06	ARPC5	protein_cc
ENSG0000(	6666.508	-0.32094	0.040011	-8.02118	1.05E-15	1.80E-14	RABGAP1	protein_cc
ENSG0000(	6665.085	-0.56389	0.084262	-6.69213	2.20E-11	1.85E-10	PRKAR1B	protein_cc
ENSG0000(	6660.339	-0.18893	0.035792	-5.2785	1.30E-07	6.06E-07	CD47	protein_cc
ENSG0000(	6658.341	-0.00644	0.038109	-0.16891	0.865864	0.903652	TALDO1	protein_cc
ENSG0000(	6657.006	-0.40944	0.045069	-9.08466	1.04E-19	3.40E-18	KLF13	protein_cc
ENSG0000(	6651.759	-0.068	0.028087	-2.42098	0.015479	0.029614	UPF1	protein_cc
ENSG0000(	6648.351	-0.06488	0.025718	-2.52266	0.011647	0.022924	VPS35	protein_cc
ENSG0000(	6646.6	-0.38553	0.047586	-8.10184	5.41E-16	9.76E-15	ATP6V1A	protein_cc
ENSG0000(	6644.897	-0.64844	0.145223	-4.46513	8.00E-06	2.80E-05	MAG	protein_cc
ENSG0000(	6639.929	-0.27013	0.041687	-6.47982	9.18E-11	7.04E-10	RANGAP1	protein_cc
ENSG0000(	6637.687	0.320777	0.050908	6.301153	2.95E-10	2.09E-09	MARS	protein_cc
ENSG0000(	6633.322	-0.84514	0.078601	-10.7523	5.78E-27	7.43E-25	SYNGR1	protein_cc
ENSG0000(	6631.491	-0.06061	0.106839	-0.56731	0.570503	0.658266	CDKN1A	protein_cc
ENSG0000(	6631.436	0.16442	0.033134	4.962275	6.97E-07	2.89E-06	EIF3G	protein_cc
ENSG0000(	6629.452	0.129683	0.034631	3.744686	0.000181	0.000505	CUTA	protein_cc
ENSG0000(	6629.356	-0.06882	0.033114	-2.07834	0.037678	0.065807	HCFC1	protein_cc
ENSG0000(	6627.833	0.125904	0.032551	3.867895	0.00011	0.000318	MAP2K2	protein_cc
ENSG0000(	6627.415	-0.28733	0.037545	-7.65289	1.97E-14	2.70E-13	CABIN1	protein_cc

ENSG0000(	6622.938	-0.05628	0.064719	-0.86961	0.384515	0.481189	RGS5	protein_cc
ENSG0000(	6619.794	-0.02859	0.029745	-0.96126	0.33642	0.431277	PAK2	protein_cc
ENSG0000(	6617.467	0.266954	0.046066	5.795086	6.83E-09	3.89E-08	ATP6V1F	protein_cc
ENSG0000(	6615.758	0.192689	0.021245	9.069857	1.19E-19	3.85E-18	ZNF207	protein_cc
ENSG0000(	6614.128	-0.03013	0.029088	-1.03594	0.300229	0.39268	ATXN2L	protein_cc
ENSG0000(	6611.863	-0.20117	0.039602	-5.0798	3.78E-07	1.63E-06	ARID1A	protein_cc
ENSG0000(	6611.391	-0.15405	0.061514	-2.50435	0.012268	0.024009	ARHGAP3	protein_cc
ENSG0000(	6609.895	-0.5268	0.077593	-6.78932	1.13E-11	9.90E-11	TUB	protein_cc
ENSG0000(	6607.906	-0.09805	0.026713	-3.67052	0.000242	0.000662	WDR26	protein_cc
ENSG0000(	6596.649	0.450792	0.046561	9.681808	3.60E-22	1.80E-20	H2AFZ	protein_cc
ENSG0000(	6594.688	0.179377	0.028095	6.384701	1.72E-10	1.26E-09	PSMB7	protein_cc
ENSG0000(	6593.141	-0.13732	0.041373	-3.31902	0.000903	0.00222	MUM1	protein_cc
ENSG0000(	6592.299	0.130933	0.027656	4.734399	2.20E-06	8.42E-06	METTL9	protein_cc
ENSG0000(	6590.882	-0.13556	0.027238	-4.97671	6.47E-07	2.70E-06	ATP2C1	protein_cc
ENSG0000(	6588.683	-0.06105	0.026439	-2.30901	0.020943	0.038812	CCAR2	protein_cc
ENSG0000(	6586.682	-0.30984	0.063134	-4.90772	9.21E-07	3.74E-06	SASH1	protein_cc
ENSG0000(	6583.02	-0.2306	0.056762	-4.06251	4.85E-05	0.00015	APCDD1	protein_cc
ENSG0000(	6582.953	-0.11926	0.026746	-4.45884	8.24E-06	2.88E-05	STX12	protein_cc
ENSG0000(	6582.688	-0.11963	0.034671	-3.45055	0.000559	0.00143	BLCAP	protein_cc
ENSG0000(	6581.058	0.249864	0.088933	2.809564	0.004961	0.010579	ALDH1L1	protein_cc
ENSG0000(	6580.381	0.086133	0.043891	1.962407	0.049715	0.084076	NDUFB9	protein_cc
ENSG0000(	6578.766	0.033791	0.031777	1.06339	0.287605	0.378855	CLNS1A	protein_cc
ENSG0000(	6578.386	-0.59871	0.066523	-8.99997	2.26E-19	6.95E-18	DLG4	protein_cc
ENSG0000(	6575.787	0.17017	0.026672	6.3802	1.77E-10	1.29E-09	DDX23	protein_cc
ENSG0000(	6575.18	0.180976	0.033563	5.392159	6.96E-08	3.38E-07	ADH5	protein_cc
ENSG0000(	6574.818	0.03771	0.026827	1.405662	0.159824	0.230861	DDX1	protein_cc
ENSG0000(	6571.894	0.069181	0.03399	2.03531	0.04182	0.072175	UBE2E1	protein_cc
ENSG0000(	6571.216	0.155758	0.025187	6.183943	6.25E-10	4.21E-09	YIPF3	protein_cc
ENSG0000(	6568.348	0.04193	0.033962	1.234638	0.216965	0.299051	DYNLRB1	protein_cc
ENSG0000(	6566.002	-0.5548	0.064491	-8.60282	7.78E-18	1.90E-16	ALDH5A1	protein_cc
ENSG0000(	6565.234	0.00131	0.047104	0.027819	0.977807	0.984293	TNKS	protein_cc
ENSG0000(	6562.937	-0.04178	0.021332	-1.9585	0.050172	0.084744	XPO6	protein_cc
ENSG0000(	6561.593	-0.24354	0.032077	-7.59219	3.15E-14	4.17E-13	SPIN1	protein_cc
ENSG0000(	6560.146	0.112056	0.032687	3.428171	0.000608	0.001543	CCT5	protein_cc
ENSG0000(	6557.909	0.274228	0.04213	6.50908	7.56E-11	5.87E-10	TMED9	protein_cc
ENSG0000(	6550.067	-0.31281	0.081022	-3.86081	0.000113	0.000327	DHCR24	protein_cc
ENSG0000(	6548.974	-0.19934	0.043337	-4.59975	4.23E-06	1.55E-05	CHD2	protein_cc
ENSG0000(	6547.922	-0.4936	0.055223	-8.93828	3.95E-19	1.17E-17	AKAP11	protein_cc
ENSG0000(	6544.798	-0.13359	0.065866	-2.02824	0.042536	0.073255	CTNNA2	protein_cc
ENSG0000(	6542.326	-0.09421	0.054737	-1.72115	0.085224	0.134427	NFKBIA	protein_cc
ENSG0000(	6539.014	-0.1535	0.032548	-4.71612	2.40E-06	9.15E-06	CALCOCC	protein_cc
ENSG0000(	6538.762	0.158217	0.030944	5.113055	3.17E-07	1.39E-06	NCSTN	protein_cc
ENSG0000(	6530.447	-0.1714	0.062664	-2.73528	0.006233	0.013014	RNF19A	protein_cc
ENSG0000(	6529.39	-0.20341	0.048719	-4.17508	2.98E-05	9.51E-05	DIP2B	protein_cc
ENSG0000(	6522.77	-0.24144	0.062991	-3.83294	0.000127	0.000363	ABCA1	protein_cc
ENSG0000(	6522.757	-0.28244	0.041478	-6.80935	9.80E-12	8.72E-11	BMPR2	protein_cc
ENSG0000(	6521.524	-0.18954	0.067425	-2.81111	0.004937	0.010533	C8orf46	protein_cc
ENSG0000(	6521.098	-0.28275	0.071087	-3.97755	6.96E-05	0.000209	ADAM22	protein_cc
ENSG0000(	6518.462	0.257927	0.049061	5.257226	1.46E-07	6.74E-07	COX6B1	protein_cc
ENSG0000(	6516.426	0.091317	0.02557	3.571225	0.000355	0.000944	CTDNEP1	protein_cc
ENSG0000(	6511.943	0.190153	0.048781	3.898065	9.70E-05	0.000284	CADM1	protein_cc
ENSG0000(	6509.422	-0.65431	0.070937	-9.22392	2.86E-20	1.01E-18	AKAP6	protein_cc
ENSG0000(	6503.861	-0.11445	0.049487	-2.31277	0.020735	0.038466	ATF7IP	protein_cc
ENSG0000(	6502.444	-0.03829	0.027918	-1.37137	0.170259	0.243665	MFF	protein_cc
ENSG0000(	6501.296	-0.06539	0.036185	-1.807	0.070763	0.114357	RABL6	protein_cc
ENSG0000(	6498.641	-0.43037	0.054511	-7.89505	2.90E-15	4.64E-14	ARHGEF17	protein_cc
ENSG0000(	6495.168	0.058331	0.027551	2.117168	0.034246	0.060427	PA2G4	protein_cc
ENSG0000(	6493.694	-0.21388	0.030405	-7.03459	2.00E-12	1.99E-11	AP2A2	protein_cc
ENSG0000(	6492.272	-0.34018	0.052829	-6.4393	1.20E-10	9.05E-10	KIAA1147	protein_cc

ENSG0000(	6491.783	0.106359	0.034908	3.046861	0.002312	0.005273	EFTUD2	protein_cc
ENSG0000(	6490.006	0.060333	0.018971	3.18027	0.001471	0.003481	PI4KB	protein_cc
ENSG0000(	6488.456	0.071647	0.026591	2.694426	0.007051	0.014557	RSL1D1	protein_cc
ENSG0000(	6487.513	0.019266	0.022933	0.840067	0.400871	0.497688	RPL7L1	protein_cc
ENSG0000(	6486.044	0.656183	0.086797	7.559969	4.03E-14	5.24E-13	ITGA7	protein_cc
ENSG0000(	6484.659	-0.21024	0.049284	-4.26592	1.99E-05	6.55E-05	CASK	protein_cc
ENSG0000(	6482.284	-0.54126	0.081192	-6.66637	2.62E-11	2.19E-10	EFHD1	protein_cc
ENSG0000(	6481.734	0.129592	0.035408	3.659984	0.000252	0.000687	LAMTOR1	protein_cc
ENSG0000(	6481.17	-1.04788	0.135377	-7.74043	9.91E-15	1.44E-13	ETNPPL	protein_cc
ENSG0000(	6479.625	-0.56546	0.053381	-10.5929	3.21E-26	3.57E-24	TSPYL1	protein_cc
ENSG0000(	6478.17	-0.01935	0.036583	-0.52894	0.596846	0.682024	ATP5C1	protein_cc
ENSG0000(	6475.925	0.174188	0.036604	4.758655	1.95E-06	7.52E-06	FLOT2	protein_cc
ENSG0000(	6472.333	0.378681	0.040608	9.325172	1.11E-20	4.23E-19	AHCY	protein_cc
ENSG0000(	6471.874	-0.11674	0.027491	-4.24653	2.17E-05	7.10E-05	CELF1	protein_cc
ENSG0000(	6469.158	0.036571	0.025106	1.456682	0.145204	0.21273	UBXN4	protein_cc
ENSG0000(	6468.754	-0.13415	0.058264	-2.30249	0.021308	0.039414	KANK1	protein_cc
ENSG0000(	6464.94	-0.02622	0.034954	-0.75026	0.4531	0.549719	KDM5C	protein_cc
ENSG0000(	6461.898	0.025457	0.02996	0.849687	0.395499	0.492066	GTF2F1	protein_cc
ENSG0000(	6460.196	0.182278	0.03829	4.760387	1.93E-06	7.47E-06	MBD3	protein_cc
ENSG0000(	6460.178	-0.34477	0.043517	-7.92273	2.32E-15	3.78E-14	MRAS	protein_cc
ENSG0000(	6458.354	-0.30904	0.036222	-8.53189	1.44E-17	3.36E-16	HTT	protein_cc
ENSG0000(	6453.454	0.416864	0.046642	8.937475	3.98E-19	1.18E-17	UBL5	protein_cc
ENSG0000(	6451.095	0.102556	0.051826	1.97885	0.047833	0.0812	CORO2B	protein_cc
ENSG0000(	6450.565	0.151916	0.023261	6.530998	6.53E-11	5.12E-10	RNPS1	protein_cc
ENSG0000(	6449.351	-0.16328	0.073656	-2.21684	0.026634	0.048227	RGCC	protein_cc
ENSG0000(	6447.551	0.311986	0.054944	5.678236	1.36E-08	7.39E-08	ATP6V0B	protein_cc
ENSG0000(	6445.686	0.221229	0.045263	4.887653	1.02E-06	4.12E-06	COX6A1	protein_cc
ENSG0000(	6442.281	-0.00959	0.025761	-0.37226	0.709697	0.778552	STK25	protein_cc
ENSG0000(	6441.072	0.321915	0.0361	8.917292	4.78E-19	1.39E-17	BANF1	protein_cc
ENSG0000(	6440.452	0.325341	0.049432	6.581601	4.65E-11	3.73E-10	PSMD8	protein_cc
ENSG0000(	6439.817	-0.02873	0.041021	-0.70044	0.48365	0.578725	ARFGAP1	protein_cc
ENSG0000(	6437.915	-0.02207	0.035172	-0.62751	0.530322	0.62203	NUDC	protein_cc
ENSG0000(	6434.516	0.189594	0.048344	3.921765	8.79E-05	0.00026	SLC16A1	protein_cc
ENSG0000(	6426.349	-0.012	0.05594	-0.21456	0.830112	0.87586	ZMAT3	protein_cc
ENSG0000(	6425.919	-0.2742	0.031152	-8.80203	1.34E-18	3.67E-17	FBXW11	protein_cc
ENSG0000(	6425.191	-0.03494	0.033105	-1.05537	0.291254	0.382755	NUDT21	protein_cc
ENSG0000(	6424.334	-0.18505	0.039146	-4.72721	2.28E-06	8.70E-06	CLASP1	protein_cc
ENSG0000(	6422.024	0.02294	0.01895	1.210598	0.226049	0.309477	TRPC4AP	protein_cc
ENSG0000(	6421.461	0.017579	0.027552	0.638042	0.523446	0.615371	MLLT1	protein_cc
ENSG0000(	6414.807	-0.05488	0.027319	-2.0088	0.044558	0.07629	BCLAF1	protein_cc
ENSG0000(	6408.48	0.034731	0.025724	1.350105	0.176982	0.251659	ADNP	protein_cc
ENSG0000(	6404.848	0.083999	0.038943	2.15698	0.031007	0.055254	TMEM106	protein_cc
ENSG0000(	6403.818	-0.09169	0.045236	-2.02684	0.042679	0.073477	VPS51	protein_cc
ENSG0000(	6402.287	0.176882	0.032654	5.416855	6.07E-08	2.98E-07	SH3GL1	protein_cc
ENSG0000(	6400.327	-0.15724	0.098743	-1.59239	0.111298	0.169475	SEZ6L2	protein_cc
ENSG0000(	6398.782	-0.12615	0.025439	-4.95904	7.08E-07	2.93E-06	HSPA4	protein_cc
ENSG0000(	6396.437	0.178657	0.028918	6.178053	6.49E-10	4.36E-09	TADA3	protein_cc
ENSG0000(	6395.806	0.212189	0.027879	7.611042	2.72E-14	3.64E-13	NOP56	protein_cc
ENSG0000(	6395.447	-0.0788	0.115176	-0.68414	0.493884	0.588283	SHC3	protein_cc
ENSG0000(	6393.749	0.058802	0.032028	1.835951	0.066365	0.108168	LONP1	protein_cc
ENSG0000(	6388.655	-0.25813	0.036515	-7.06911	1.56E-12	1.57E-11	ATRN	protein_cc
ENSG0000(	6386.888	-0.25217	0.111106	-2.26967	0.023228	0.042625	ZDHHC22	protein_cc
ENSG0000(	6386.49	-0.05245	0.025584	-2.05006	0.040359	0.069945	DYNC1I2	protein_cc
ENSG0000(	6383.713	0.576027	0.079968	7.203241	5.88E-13	6.33E-12	FKBP10	protein_cc
ENSG0000(	6382.603	0.210425	0.052033	4.044073	5.25E-05	0.000161	ATP5G3	protein_cc
ENSG0000(	6382.231	-0.06241	0.048486	-1.28716	0.198039	0.276899	MPZL1	protein_cc
ENSG0000(	6379.204	0.296713	0.047291	6.274163	3.52E-10	2.46E-09	SSR4	protein_cc
ENSG0000(	6370.759	-0.30782	0.04363	-7.05525	1.72E-12	1.73E-11	YLPM1	protein_cc
ENSG0000(	6360.168	-0.02632	0.041768	-0.63006	0.528653	0.620301	GAA	protein_cc

ENSG0000(	6357.831	0.303167	0.048902	6.199432	5.67E-10	3.83E-09	PDIA6	protein_cc
ENSG0000(	6356.239	0.204179	0.026216	7.788384	6.79E-15	1.02E-13	PPM1G	protein_cc
ENSG0000(	6355.056	0.154728	0.038104	4.060641	4.89E-05	0.000151	DAD1	protein_cc
ENSG0000(	6354.812	-0.20406	0.036781	-5.54785	2.89E-08	1.49E-07	CIC	protein_cc
ENSG0000(	6353.873	-0.52022	0.095707	-5.4356	5.46E-08	2.70E-07	RUNDC3A	protein_cc
ENSG0000(	6348.97	0.014543	0.039169	0.371302	0.710413	0.779055	ANP32B	protein_cc
ENSG0000(	6348.258	-0.27392	0.032604	-8.40126	4.42E-17	9.52E-16	FTO	protein_cc
ENSG0000(	6342.546	0.046908	0.062864	0.74618	0.455559	0.5521	RTKN	protein_cc
ENSG0000(	6342.519	0.264495	0.042465	6.22856	4.71E-10	3.23E-09	NASP	protein_cc
ENSG0000(	6340.581	-0.66216	0.081498	-8.12486	4.48E-16	8.20E-15	5-Sep	protein_cc
ENSG0000(	6339.952	-0.0405	0.027849	-1.45442	0.14583	0.213512	SCAP	protein_cc
ENSG0000(	6337.318	0.010024	0.030264	0.331222	0.740477	0.803958	RDH11	protein_cc
ENSG0000(	6333.045	-0.22826	0.048262	-4.72951	2.25E-06	8.61E-06	MBOAT2	protein_cc
ENSG0000(	6330.722	-0.43538	0.085713	-5.0795	3.78E-07	1.64E-06	AC005013	pseudoge
ENSG0000(	6328.868	0.012603	0.023267	0.541663	0.58805	0.67424	CD2BP2	protein_cc
ENSG0000(	6308.843	-0.19879	0.038871	-5.11404	3.15E-07	1.38E-06	GOLGB1	protein_cc
ENSG0000(	6306.81	-0.20821	0.105731	-1.96924	0.048926	0.082899	ITGB4	protein_cc
ENSG0000(	6305.876	0.033478	0.041908	0.798861	0.424371	0.521464	HSD17B12	protein_cc
ENSG0000(	6301.374	0.090779	0.020525	4.422953	9.74E-06	3.37E-05	POLDIP2	protein_cc
ENSG0000(	6296.674	-0.03358	0.045078	-0.74485	0.456365	0.552833	FERMT2	protein_cc
ENSG0000(	6290.32	0.21488	0.04046	5.310923	1.09E-07	5.14E-07	EXTL3	protein_cc
ENSG0000(	6287.591	-0.06768	0.035236	-1.92067	0.054774	0.091521	SUGP2	protein_cc
ENSG0000(	6286.71	-0.39485	0.047972	-8.23089	1.86E-16	3.62E-15	ASH1L	protein_cc
ENSG0000(	6284.248	-0.41335	0.054624	-7.56723	3.81E-14	4.98E-13	MXI1	protein_cc
ENSG0000(	6283.685	-0.05041	0.027751	-1.8166	0.069278	0.112315	CIZ1	protein_cc
ENSG0000(	6282.073	-0.00316	0.025348	-0.12484	0.900646	0.929486	CSNK2A1	protein_cc
ENSG0000(	6278.046	0.650534	0.178087	3.652891	0.000259	0.000705	CHI3L2	protein_cc
ENSG0000(	6276.623	0.035218	0.080056	0.439918	0.659997	0.737005	MATN2	protein_cc
ENSG0000(	6272.635	0.086662	0.028227	3.070211	0.002139	0.004907	SNX5	protein_cc
ENSG0000(	6270.837	0.020366	0.030042	0.677943	0.497808	0.591833	IRF2BP2	protein_cc
ENSG0000(	6270.49	-0.21729	0.039882	-5.4482	5.09E-08	2.53E-07	UBR5	protein_cc
ENSG0000(	6269.694	0.086193	0.022605	3.81303	0.000137	0.000391	WIPI2	protein_cc
ENSG0000(	6269.628	0.058732	0.036203	1.622291	0.104741	0.160789	CLTA	protein_cc
ENSG0000(	6265.267	-0.3665	0.040851	-8.9717	2.92E-19	8.85E-18	RAB18	protein_cc
ENSG0000(	6262.978	-0.10447	0.054144	-1.92955	0.053663	0.089887	PNISR	protein_cc
ENSG0000(	6262.505	-0.01375	0.047472	-0.28957	0.772147	0.829492	LRRC4B	protein_cc
ENSG0000(	6257.77	-0.36897	0.107545	-3.43083	0.000602	0.001529	GPR98	protein_cc
ENSG0000(	6256.183	0.055706	0.030892	1.803265	0.071347	0.115197	NFE2L2	protein_cc
ENSG0000(	6244.085	0.146633	0.046171	3.175862	0.001494	0.00353	B4GALT5	protein_cc
ENSG0000(	6243.681	0.046431	0.034602	1.341861	0.179641	0.254831	GPAA1	protein_cc
ENSG0000(	6241.618	1.014649	0.088567	11.45629	2.19E-30	5.55E-28	VOPP1	protein_cc
ENSG0000(	6235.844	0.481373	0.077039	6.248412	4.15E-10	2.87E-09	RPS26	protein_cc
ENSG0000(	6234.763	-0.09715	0.030122	-3.22516	0.001259	0.003013	IVD	protein_cc
ENSG0000(	6230.433	0.102384	0.030653	3.340044	0.000838	0.002071	SLC39A7	protein_cc
ENSG0000(	6223.654	-0.11043	0.035424	-3.11739	0.001825	0.00424	TSC2	protein_cc
ENSG0000(	6222.531	0.335877	0.089991	3.732364	0.00019	0.000528	APOC1	protein_cc
ENSG0000(	6221.37	-0.21974	0.033272	-6.6042	4.00E-11	3.24E-10	SDHA	protein_cc
ENSG0000(	6220.909	0.354338	0.041381	8.562846	1.10E-17	2.61E-16	RHEB	protein_cc
ENSG0000(	6218.692	-0.19072	0.029722	-6.41664	1.39E-10	1.04E-09	ARMCX3	protein_cc
ENSG0000(	6216.635	-0.22643	0.067107	-3.37422	0.00074	0.001847	GRIA3	protein_cc
ENSG0000(	6214.047	-0.18052	0.02587	-6.978	2.99E-12	2.90E-11	HNRNPH2	protein_cc
ENSG0000(	6212.502	-0.44975	0.079035	-5.69045	1.27E-08	6.91E-08	TSPYL2	protein_cc
ENSG0000(	6211.517	-0.36458	0.063307	-5.75892	8.47E-09	4.75E-08	PALM	protein_cc
ENSG0000(	6210.403	0.054737	0.042183	1.297608	0.194422	0.272565	SLC39A6	protein_cc
ENSG0000(	6202.581	0.210682	0.065886	3.197652	0.001386	0.003294	PLOD1	protein_cc
ENSG0000(	6195.417	0.130711	0.029218	4.473644	7.69E-06	2.70E-05	CCT8	protein_cc
ENSG0000(	6184.218	0.109899	0.025676	4.280206	1.87E-05	6.18E-05	RIC8A	protein_cc
ENSG0000(	6183.879	0.356777	0.048334	7.38157	1.56E-13	1.85E-12	PRMT1	protein_cc
ENSG0000(	6181.017	-0.38187	0.045924	-8.31525	9.16E-17	1.87E-15	NDRG3	protein_cc

ENSG0000	6177.569	-0.231	0.029795	-7.75297	8.98E-15	1.32E-13	SNX19	protein_cc
ENSG0000	6177.16	-0.37171	0.044131	-8.42279	3.68E-17	8.04E-16	HERC2	protein_cc
ENSG0000	6173.184	0.097283	0.05512	1.764939	0.077574	0.123962	PODXL	protein_cc
ENSG0000	6167.857	0.014365	0.044514	0.322698	0.746924	0.809305	UBA2	protein_cc
ENSG0000	6162.754	0.022322	0.036286	0.615169	0.538443	0.629491	CDKN1B	protein_cc
ENSG0000	6161.589	-0.21653	0.067352	-3.21484	0.001305	0.003115	PTPRN2	protein_cc
ENSG0000	6159.85	0.289403	0.044089	6.564132	5.23E-11	4.16E-10	SDF4	protein_cc
ENSG0000	6159.44	-0.12827	0.066167	-1.93851	0.052561	0.088279	MTSS1	protein_cc
ENSG0000	6157.074	0.122999	0.064334	1.911875	0.055892	0.093133	KAT2A	protein_cc
ENSG0000	6156.531	-0.03977	0.061812	-0.64337	0.519984	0.612401	HEPACAM	protein_cc
ENSG0000	6154.623	0.065816	0.053004	1.241722	0.214339	0.296046	RRBP1	protein_cc
ENSG0000	6149.832	-0.01407	0.019855	-0.7085	0.478633	0.574058	GPBP1	protein_cc
ENSG0000	6147.673	-0.07442	0.028063	-2.65182	0.008006	0.016354	SMG5	protein_cc
ENSG0000	6145.064	0.017714	0.036569	0.484392	0.628108	0.709643	RBM5	protein_cc
ENSG0000	6144.715	0.151451	0.03945	3.839034	0.000124	0.000355	CYC1	protein_cc
ENSG0000	6142.309	0.144959	0.057581	2.517472	0.01182	0.023229	SH3BGRL3	protein_cc
ENSG0000	6140.404	-0.05059	0.031514	-1.60518	0.108454	0.165683	WDR13	protein_cc
ENSG0000	6138.604	-0.41104	0.054027	-7.60799	2.78E-14	3.72E-13	MADD	protein_cc
ENSG0000	6138.483	-0.86706	0.112483	-7.70836	1.27E-14	1.81E-13	GABBR2	protein_cc
ENSG0000	6137.323	0.085888	0.032781	2.620035	0.008792	0.017787	HNRNPF	protein_cc
ENSG0000	6136.79	-0.1966	0.045815	-4.29112	1.78E-05	5.90E-05	SLC12A4	protein_cc
ENSG0000	6135.062	-0.03624	0.023148	-1.56566	0.117428	0.17743	NDUFS2	protein_cc
ENSG0000	6133.872	0.203919	0.042386	4.810983	1.50E-06	5.91E-06	EIF3K	protein_cc
ENSG0000	6130.457	0.437602	0.119914	3.649296	0.000263	0.000714	SERPING1	protein_cc
ENSG0000	6122.064	-0.07273	0.085529	-0.85033	0.395144	0.491706	MAPK4	protein_cc
ENSG0000	6117.901	0.274278	0.035368	7.75497	8.84E-15	1.30E-13	ARPC1A	protein_cc
ENSG0000	6117.419	-0.49768	0.055974	-8.89127	6.04E-19	1.74E-17	CX3CL1	protein_cc
ENSG0000	6113.653	0.309949	0.05274	5.876936	4.18E-09	2.47E-08	NREP	protein_cc
ENSG0000	6112.86	-0.04962	0.028719	-1.72794	0.083999	0.132722	MED24	protein_cc
ENSG0000	6109.886	0.277493	0.057461	4.829275	1.37E-06	5.42E-06	LAP3	protein_cc
ENSG0000	6109.215	-0.27236	0.041542	-6.55636	5.51E-11	4.36E-10	DAB2IP	protein_cc
ENSG0000	6108.017	-0.08971	0.097338	-0.92162	0.356726	0.452415	DUSP1	protein_cc
ENSG0000	6101.013	-0.05142	0.06113	-0.84114	0.400271	0.497078	RHOU	protein_cc
ENSG0000	6096.006	-0.29821	0.049446	-6.03099	1.63E-09	1.03E-08	IGSF8	protein_cc
ENSG0000	6093.125	0.106683	0.056909	1.874607	0.060847	0.100267	ZC3H11A	protein_cc
ENSG0000	6087.886	-0.61314	0.055417	-11.0642	1.87E-28	3.21E-26	PPP3CA	protein_cc
ENSG0000	6083.83	0.190436	0.046323	4.111039	3.94E-05	0.000123	H1FX	protein_cc
ENSG0000	6078.33	-0.30072	0.105191	-2.85875	0.004253	0.009198	NMB	protein_cc
ENSG0000	6077.208	-0.07847	0.031714	-2.47415	0.013355	0.025933	OTUB1	protein_cc
ENSG0000	6068.303	-0.01396	0.033412	-0.4177	0.676168	0.750535	SEC31A	protein_cc
ENSG0000	6068.071	-0.07578	0.033711	-2.24803	0.024574	0.044842	PPP1R10	protein_cc
ENSG0000	6066.083	-0.76614	0.063662	-12.0345	2.34E-33	1.09E-30	FAM171A1	protein_cc
ENSG0000	6065.226	-0.05122	0.086344	-0.59322	0.553034	0.642733	CCND1	protein_cc
ENSG0000	6065.124	-0.01365	0.02958	-0.4613	0.644582	0.724158	EXOC4	protein_cc
ENSG0000	6061.803	-0.05339	0.037801	-1.41248	0.15781	0.22831	SAFB	protein_cc
ENSG0000	6061.058	-0.0061	0.06464	-0.09434	0.924842	0.946995	GNG7	protein_cc
ENSG0000	6058.199	0.260878	0.049687	5.250426	1.52E-07	6.98E-07	NDUFS5	protein_cc
ENSG0000	6055.717	-0.1162	0.072658	-1.5993	0.109753	0.167426	DDIT4	protein_cc
ENSG0000	6054.205	-0.51624	0.056095	-9.20312	3.48E-20	1.22E-18	ZBTB47	protein_cc
ENSG0000	6037.787	-0.26709	0.039976	-6.68114	2.37E-11	1.99E-10	DCAKD	protein_cc
ENSG0000	6033.924	0.018834	0.033874	0.556009	0.578205	0.665289	TMEM9B	protein_cc
ENSG0000	6032.711	-0.02129	0.030978	-0.68729	0.491899	0.586396	GRHPR	protein_cc
ENSG0000	6024.058	0.203545	0.031003	6.565317	5.19E-11	4.13E-10	SRRT	protein_cc
ENSG0000	6021.885	0.118368	0.117135	1.010522	0.312245	0.405558	C1S	protein_cc
ENSG0000	6021.22	-0.25091	0.04485	-5.59449	2.21E-08	1.16E-07	HIPK1	protein_cc
ENSG0000	6020.589	-0.54071	0.061916	-8.73291	2.48E-18	6.52E-17	PPIF	protein_cc
ENSG0000	6019.615	-0.51918	0.07461	-6.95858	3.44E-12	3.29E-11	PIP4K2A	protein_cc
ENSG0000	6017.508	-0.04222	0.032468	-1.30028	0.193505	0.271445	KCTD3	protein_cc
ENSG0000	6016.792	-0.14252	0.032416	-4.39667	1.10E-05	3.77E-05	NCOR1	protein_cc

ENSG0000(	6015.859	0.314605	0.062708	5.017021	5.25E-07	2.22E-06	LMNA	protein_cc
ENSG0000(	6013.516	0.2485	0.064473	3.854303	0.000116	0.000335	ECE1	protein_cc
ENSG0000(	6012.741	0.11716	0.02505	4.677029	2.91E-06	1.09E-05	KARS	protein_cc
ENSG0000(	6006.513	0.025841	0.034698	0.744725	0.456438	0.552905	DLGAP4	protein_cc
ENSG0000(	6004.38	-0.24684	0.088266	-2.79651	0.005166	0.010977	FAM84B	protein_cc
ENSG0000(	6001.611	-0.30906	0.02883	-10.72	8.20E-27	1.03E-24	RABEP1	protein_cc
ENSG0000(	6000.994	-0.05675	0.023333	-2.43221	0.015007	0.028798	AGPAT1	protein_cc
ENSG0000(	5999.601	0.021115	0.042804	0.493286	0.621811	0.70427	EEF1D	protein_cc
ENSG0000(	5999.396	-0.04842	0.025314	-1.91293	0.055757	0.092933	TBL1XR1	protein_cc
ENSG0000(	5998.744	-0.24537	0.044544	-5.50856	3.62E-08	1.84E-07	ZNF106	protein_cc
ENSG0000(	5997.66	0.199615	0.039708	5.027075	4.98E-07	2.11E-06	ATP5L	protein_cc
ENSG0000(	5995.288	-0.08746	0.030312	-2.88542	0.003909	0.008521	RRAGA	protein_cc
ENSG0000(	5994.817	-0.11738	0.025527	-4.59819	4.26E-06	1.56E-05	BSDC1	protein_cc
ENSG0000(	5991.34	0.222919	0.039891	5.588251	2.29E-08	1.20E-07	AKT2	protein_cc
ENSG0000(	5976.808	-0.12263	0.028378	-4.32108	1.55E-05	5.19E-05	MSL1	protein_cc
ENSG0000(	5975.915	0.224409	0.06396	3.508585	0.00045	0.001174	CCNL2	protein_cc
ENSG0000(	5974.518	-0.30539	0.039955	-7.64352	2.11E-14	2.89E-13	HECTD1	protein_cc
ENSG0000(	5974.06	0.620086	0.086788	7.144848	9.01E-13	9.44E-12	ELN	protein_cc
ENSG0000(	5972.014	-0.0839	0.02801	-2.99534	0.002741	0.006154	ARL8A	protein_cc
ENSG0000(	5970.278	-0.09175	0.034492	-2.66008	0.007812	0.015995	FKBP4	protein_cc
ENSG0000(	5966.624	0.166175	0.044222	3.757736	0.000171	0.000481	UBE2L6	protein_cc
ENSG0000(	5963.867	-0.30815	0.07529	-4.09275	4.26E-05	0.000133	AMOTL2	protein_cc
ENSG0000(	5951.521	-0.04454	0.04424	-1.00686	0.314003	0.407403	LSS	protein_cc
ENSG0000(	5947.232	-0.25519	0.040477	-6.30456	2.89E-10	2.05E-09	EIF4G3	protein_cc
ENSG0000(	5945.967	-0.10229	0.050611	-2.02115	0.043264	0.074338	TEAD1	protein_cc
ENSG0000(	5943.568	0.389952	0.109209	3.570675	0.000356	0.000946	MMP14	protein_cc
ENSG0000(	5940.884	-0.1068	0.026551	-4.02258	5.76E-05	0.000175	ATG9A	protein_cc
ENSG0000(	5936.028	0.298523	0.040513	7.36853	1.73E-13	2.02E-12	PCMTD2	protein_cc
ENSG0000(	5935.66	-0.41854	0.078367	-5.34079	9.25E-08	4.41E-07	SYNM	protein_cc
ENSG0000(	5934.894	-0.17732	0.034206	-5.18406	2.17E-07	9.74E-07	FBXO21	protein_cc
ENSG0000(	5932.634	-0.04924	0.064596	-0.76223	0.445921	0.542691	NTN1	protein_cc
ENSG0000(	5932.328	-0.29608	0.113132	-2.61711	0.008868	0.017926	AGAP2	protein_cc
ENSG0000(	5929.725	-0.53409	0.05555	-9.61461	6.94E-22	3.27E-20	HECTD4	protein_cc
ENSG0000(	5922.471	-0.08467	0.028865	-2.93338	0.003353	0.007403	SEC16A	protein_cc
ENSG0000(	5920.848	-0.04091	0.038537	-1.06169	0.288375	0.379674	SCP2	protein_cc
ENSG0000(	5915.938	-0.03261	0.034741	-0.93879	0.347839	0.443386	CAPN1	protein_cc
ENSG0000(	5915.384	-0.13668	0.034375	-3.97607	7.01E-05	0.00021	ARAP1	protein_cc
ENSG0000(	5913.49	0.049587	0.036224	1.368898	0.171031	0.244601	TXN2	protein_cc
ENSG0000(	5912.122	-0.48497	0.051481	-9.42033	4.50E-21	1.85E-19	SAMD8	protein_cc
ENSG0000(	5909.906	0.115155	0.031213	3.689303	0.000225	0.000618	SCAMP4	protein_cc
ENSG0000(	5907.234	-0.08538	0.051041	-1.67277	0.094374	0.14693	LIPA	protein_cc
ENSG0000(	5904.514	0.079609	0.056908	1.398916	0.161838	0.233303	PALLD	protein_cc
ENSG0000(	5904.308	0.289795	0.031061	9.329847	1.06E-20	4.07E-19	AUP1	protein_cc
ENSG0000(	5902.656	0.139248	0.033244	4.188721	2.81E-05	9.00E-05	EIF3M	protein_cc
ENSG0000(	5899.989	0.097864	0.049784	1.965778	0.049324	0.083487	CTSA	protein_cc
ENSG0000(	5897.323	-0.3189	0.053512	-5.95942	2.53E-09	1.55E-08	MED13L	protein_cc
ENSG0000(	5897.174	0.009551	0.029641	0.322215	0.747289	0.809598	NRD1	protein_cc
ENSG0000(	5897.152	-0.1621	0.032275	-5.0226	5.10E-07	2.16E-06	TAOK2	protein_cc
ENSG0000(	5896.625	-0.45697	0.056763	-8.05045	8.25E-16	1.44E-14	PKP4	protein_cc
ENSG0000(	5895.141	-0.12351	0.036536	-3.38043	0.000724	0.00181	NRSN2	protein_cc
ENSG0000(	5893.443	0.086419	0.02244	3.851111	0.000118	0.000339	PSME3	protein_cc
ENSG0000(	5887.738	-0.7621	0.120792	-6.30917	2.81E-10	1.99E-09	LUZP2	protein_cc
ENSG0000(	5880.179	-0.32623	0.044026	-7.40999	1.26E-13	1.51E-12	GAB2	protein_cc
ENSG0000(	5878.575	0.000343	0.023336	0.014683	0.988285	0.991976	IK	protein_cc
ENSG0000(	5878.329	-0.22371	0.058378	-3.83217	0.000127	0.000364	PCDH10	protein_cc
ENSG0000(	5877.179	-0.08284	0.031836	-2.6019	0.009271	0.018657	ABHD12	protein_cc
ENSG0000(	5876.456	-0.13522	0.020546	-6.58147	4.66E-11	3.73E-10	YY1	protein_cc
ENSG0000(	5875.392	-0.26449	0.057053	-4.6358	3.56E-06	1.32E-05	TYRO3	protein_cc
ENSG0000(	5872.744	-0.60672	0.099737	-6.08322	1.18E-09	7.60E-09	JPH4	protein_cc

ENSG0000(	5869.894	-0.59587	0.055036	-10.827	2.56E-27	3.47E-25	SPRYD3	protein_cc
ENSG0000(	5869.007	0.114053	0.023535	4.846182	1.26E-06	5.01E-06	RNF167	protein_cc
ENSG0000(	5863.908	-0.08964	0.058452	-1.5335	0.125153	0.187312	PRRX1	protein_cc
ENSG0000(	5863.408	0.252687	0.040805	6.192508	5.92E-10	4.00E-09	PSMB1	protein_cc
ENSG0000(	5862.466	0.367395	0.031141	11.79798	4.00E-32	1.36E-29	TMED4	protein_cc
ENSG0000(	5858.717	-0.29368	0.057294	-5.12586	2.96E-07	1.30E-06	ARHGEF1(	protein_cc
ENSG0000(	5855.211	0.058204	0.037886	1.536285	0.124468	0.186431	MCFD2	protein_cc
ENSG0000(	5851.961	0.202825	0.039217	5.17188	2.32E-07	1.04E-06	ARPC4	protein_cc
ENSG0000(	5850.054	0.206295	0.038201	5.400213	6.66E-08	3.24E-07	C8orf33	protein_cc
ENSG0000(	5848.162	-0.10516	0.0278	-3.78278	0.000155	0.000438	MGRN1	protein_cc
ENSG0000(	5846.955	0.034932	0.033872	1.031285	0.302407	0.395139	CDK16	protein_cc
ENSG0000(	5846.509	0.092686	0.025391	3.650284	0.000262	0.000712	KEAP1	protein_cc
ENSG0000(	5842.481	-0.25249	0.041365	-6.10403	1.03E-09	6.74E-09	HDAC5	protein_cc
ENSG0000(	5842.418	-0.2429	0.129077	-1.88183	0.059859	0.098865	CHGB	protein_cc
ENSG0000(	5839.612	-0.205	0.057362	-3.57384	0.000352	0.000936	CACNG7	protein_cc
ENSG0000(	5831.829	-0.37714	0.048165	-7.83026	4.87E-15	7.50E-14	SIK3	protein_cc
ENSG0000(	5829.371	-0.04163	0.041107	-1.01265	0.311226	0.40449	TMX4	protein_cc
ENSG0000(	5823.106	-0.24699	0.052367	-4.71653	2.40E-06	9.13E-06	MID1IP1	protein_cc
ENSG0000(	5822.935	0.053423	0.032039	1.667457	0.095424	0.148363	MAPKAP1	protein_cc
ENSG0000(	5822.882	0.094161	0.037409	2.517076	0.011833	0.023252	MGAT1	protein_cc
ENSG0000(	5815.529	0.080168	0.028384	2.824399	0.004737	0.010134	QARS	protein_cc
ENSG0000(	5811.322	0.204916	0.055034	3.723464	0.000197	0.000546	DPP7	protein_cc
ENSG0000(	5807.361	-0.01766	0.084226	-0.20968	0.833918	0.878952	RAB3IP	protein_cc
ENSG0000(	5805.268	0.215626	0.036559	5.898063	3.68E-09	2.19E-08	HMGN1	protein_cc
ENSG0000(	5804.796	-0.15925	0.04563	-3.49009	0.000483	0.00125	ZDHHC9	protein_cc
ENSG0000(	5802.469	-0.09592	0.032637	-2.93901	0.003293	0.00728	GCN1L1	protein_cc
ENSG0000(	5798.988	0.057235	0.034712	1.648874	0.099173	0.153422	AGPAT5	protein_cc
ENSG0000(	5795.668	0.216115	0.025739	8.396391	4.60E-17	9.89E-16	SRSF2	protein_cc
ENSG0000(	5793.876	0.271177	0.050971	5.320279	1.04E-07	4.90E-07	CAMK2D	protein_cc
ENSG0000(	5790.07	-0.07672	0.031512	-2.4347	0.014904	0.028624	RPRD1A	protein_cc
ENSG0000(	5789.337	0.127932	0.027267	4.691877	2.71E-06	1.02E-05	RPA1	protein_cc
ENSG0000(	5788.853	-0.00814	0.039823	-0.20431	0.838113	0.882245	PDDC1	protein_cc
ENSG0000(	5787.336	-0.53728	0.095784	-5.60922	2.03E-08	1.08E-07	HSPA2	protein_cc
ENSG0000(	5787.044	-0.36997	0.061161	-6.04908	1.46E-09	9.25E-09	GRAMD3	protein_cc
ENSG0000(	5786.59	0.171684	0.044102	3.892899	9.91E-05	0.00029	RCC2	protein_cc
ENSG0000(	5784.967	-0.23611	0.061134	-3.86224	0.000112	0.000325	FAM129B	protein_cc
ENSG0000(	5784.66	0.007431	0.061898	0.120057	0.904438	0.9323	SAMHD1	protein_cc
ENSG0000(	5780.34	-0.24204	0.058196	-4.15905	3.20E-05	0.000102	NTRK3	protein_cc
ENSG0000(	5776.658	-0.05793	0.025523	-2.26954	0.023235	0.042635	FXR1	protein_cc
ENSG0000(	5775.833	-0.09753	0.061068	-1.597	0.110265	0.168076	RFTN2	protein_cc
ENSG0000(	5775.664	-0.26719	0.02919	-9.15364	5.50E-20	1.88E-18	ELMO2	protein_cc
ENSG0000(	5774.298	-0.25134	0.03813	-6.59166	4.35E-11	3.50E-10	SEL1L	protein_cc
ENSG0000(	5772.477	0.019715	0.028911	0.68191	0.495296	0.589544	CDC42SE1	protein_cc
ENSG0000(	5771.549	0.200475	0.021949	9.133813	6.61E-20	2.23E-18	TRA2B	protein_cc
ENSG0000(	5761.665	-0.17779	0.036756	-4.83708	1.32E-06	5.22E-06	ANKRD17	protein_cc
ENSG0000(	5760.128	-0.25787	0.039148	-6.58703	4.49E-11	3.61E-10	FAM199X	protein_cc
ENSG0000(	5759.197	0.320704	0.053285	6.01865	1.76E-09	1.10E-08	TRAM1	protein_cc
ENSG0000(	5757.487	-0.07483	0.047333	-1.58093	0.113893	0.172844	APBB2	protein_cc
ENSG0000(	5757.48	-0.60292	0.064182	-9.39387	5.78E-21	2.31E-19	CMTM4	protein_cc
ENSG0000(	5756.285	0.031179	0.040064	0.77822	0.43644	0.533456	RNF13	protein_cc
ENSG0000(	5754.707	-0.23746	0.054511	-4.35613	1.32E-05	4.48E-05	AGAP1	protein_cc
ENSG0000(	5753.143	-0.11588	0.115902	-0.99983	0.317391	0.411112	LIMS2	protein_cc
ENSG0000(	5747.745	0.063934	0.035378	1.807146	0.07074	0.114324	LSM14A	protein_cc
ENSG0000(	5746.887	0.021156	0.050245	0.421062	0.67371	0.748357	GALNT2	protein_cc
ENSG0000(	5744.014	-0.1545	0.074469	-2.0747	0.038014	0.066329	CHD7	protein_cc
ENSG0000(	5733.929	-0.03588	0.037045	-0.96844	0.332825	0.427375	CCDC50	protein_cc
ENSG0000(	5733.285	0.148402	0.069145	2.146258	0.031852	0.056595	WARS	protein_cc
ENSG0000(	5717.285	-0.11333	0.049977	-2.2677	0.023348	0.042815	BCAR1	protein_cc
ENSG0000(	5711.311	-0.02752	0.037647	-0.73104	0.464754	0.560807	DKFZP761	protein_cc

ENSG0000(	5709.874	0.010714	0.030209	0.354657	0.722847	0.789125	GNA11	protein_cc
ENSG0000(	5708.384	-0.69134	0.093933	-7.3599	1.84E-13	2.14E-12	SPTBN2	protein_cc
ENSG0000(	5705.691	-0.19993	0.031635	-6.31984	2.62E-10	1.87E-09	TFE3	protein_cc
ENSG0000(	5705.606	-0.12868	0.103946	-1.23799	0.21572	0.297714	ZFP36	protein_cc
ENSG0000(	5703.487	-0.46535	0.100616	-4.62503	3.75E-06	1.38E-05	RNASE1	protein_cc
ENSG0000(	5697.661	-0.08036	0.028882	-2.78243	0.005395	0.011422	PACS1	protein_cc
ENSG0000(	5697.096	-0.17404	0.032253	-5.39598	6.82E-08	3.31E-07	PUM1	protein_cc
ENSG0000(	5694.428	0.158778	0.037081	4.28192	1.85E-05	6.13E-05	DNAJC8	protein_cc
ENSG0000(	5693.412	0.057314	0.024144	2.373845	0.017604	0.033181	CHMP1A	protein_cc
ENSG0000(	5692.778	-0.16444	0.038196	-4.30524	1.67E-05	5.56E-05	SRPK2	protein_cc
ENSG0000(	5691.153	0.19557	0.042491	4.602634	4.17E-06	1.53E-05	RCN1	protein_cc
ENSG0000(	5688.861	-0.23898	0.070218	-3.40334	0.000666	0.001677	TUBB2A	protein_cc
ENSG0000(	5685.798	0.266991	0.04134	6.458415	1.06E-10	8.04E-10	CHMP2A	protein_cc
ENSG0000(	5685.094	-0.17199	0.048542	-3.54323	0.000395	0.001041	GATS	pseudoge
ENSG0000(	5682.64	-0.28288	0.032974	-8.57881	9.59E-18	2.31E-16	ANKRD40	protein_cc
ENSG0000(	5674.399	-0.73121	0.140081	-5.2199	1.79E-07	8.15E-07	ERMN	protein_cc
ENSG0000(	5672.789	-0.03933	0.034634	-1.13554	0.256149	0.343896	GNPTG	protein_cc
ENSG0000(	5667.929	-0.19461	0.063614	-3.05923	0.002219	0.005076	KLF9	protein_cc
ENSG0000(	5662.431	-0.03861	0.02492	-1.54919	0.121335	0.182362	GRSF1	protein_cc
ENSG0000(	5662.132	-0.12207	0.053856	-2.2666	0.023415	0.042914	RNF157	protein_cc
ENSG0000(	5658.769	-0.14814	0.026248	-5.64382	1.66E-08	8.91E-08	SDHC	protein_cc
ENSG0000(	5655.99	-0.00234	0.046619	-0.05022	0.959946	0.972551	VAMP3	protein_cc
ENSG0000(	5651.432	0.264852	0.057387	4.615208	3.93E-06	1.45E-05	SERPINB6	protein_cc
ENSG0000(	5650.406	-0.28194	0.05328	-5.29167	1.21E-07	5.67E-07	PEAK1	protein_cc
ENSG0000(	5649.247	0.049748	0.027677	1.79746	0.072263	0.116515	ENOPH1	protein_cc
ENSG0000(	5645.866	-0.18124	0.036219	-5.00396	5.62E-07	2.36E-06	PCMT1	protein_cc
ENSG0000(	5644.921	-0.31492	0.05614	-5.60965	2.03E-08	1.07E-07	HSPH1	protein_cc
ENSG0000(	5643.413	0.045557	0.019131	2.381325	0.01725	0.032594	TARDBP	protein_cc
ENSG0000(	5642.061	0.142767	0.099924	1.428763	0.153072	0.222633	TIMP4	protein_cc
ENSG0000(	5641.007	-0.10878	0.036545	-2.97664	0.002914	0.006513	POGZ	protein_cc
ENSG0000(	5640.691	0.642539	0.082016	7.83427	4.72E-15	7.28E-14	LFNG	protein_cc
ENSG0000(	5638.532	-0.31776	0.055275	-5.74869	8.99E-09	5.02E-08	SNHG14	processed
ENSG0000(	5634.93	-0.0107	0.03043	-0.3515	0.725213	0.790994	UBE2G2	protein_cc
ENSG0000(	5634.663	0.014049	0.026062	0.53905	0.589852	0.675779	TM9SF4	protein_cc
ENSG0000(	5631.541	-0.01012	0.038083	-0.26578	0.790411	0.844457	COX6C	protein_cc
ENSG0000(	5630.373	0.199297	0.053192	3.746779	0.000179	0.000501	SHMT2	protein_cc
ENSG0000(	5629.449	0.087186	0.033576	2.596715	0.009412	0.01891	IARS	protein_cc
ENSG0000(	5623.571	-0.16605	0.148043	-1.12162	0.262026	0.350504	GPR17	protein_cc
ENSG0000(	5621.487	-0.04183	0.159296	-0.2626	0.792858	0.846523	COL6A2	protein_cc
ENSG0000(	5618.736	-0.18133	0.037704	-4.80927	1.51E-06	5.95E-06	ZNF638	protein_cc
ENSG0000(	5618.6	-0.43734	0.054465	-8.02974	9.77E-16	1.68E-14	PNMAL1	protein_cc
ENSG0000(	5618.499	-0.25331	0.038276	-6.61798	3.64E-11	2.97E-10	GM2A	protein_cc
ENSG0000(	5614.647	-0.29893	0.033014	-9.05482	1.37E-19	4.38E-18	MAPK3	protein_cc
ENSG0000(	5613.295	-0.44936	0.050097	-8.96985	2.97E-19	8.98E-18	MYO18A	protein_cc
ENSG0000(	5611.848	-0.70116	0.10109	-6.93599	4.03E-12	3.82E-11	CTSH	protein_cc
ENSG0000(	5608.273	-0.09512	0.030254	-3.14388	0.001667	0.003904	RAB11FIP3	protein_cc
ENSG0000(	5604.222	0.349085	0.06498	5.372164	7.78E-08	3.75E-07	TRIM47	protein_cc
ENSG0000(	5603.348	-0.01532	0.036742	-0.41687	0.676777	0.751073	ALDH3A2	protein_cc
ENSG0000(	5598.479	-0.04413	0.046018	-0.95896	0.337579	0.432439	IFNGR1	protein_cc
ENSG0000(	5598.329	0.297412	0.038216	7.782433	7.11E-15	1.07E-13	BZW1	protein_cc
ENSG0000(	5597.786	-0.13681	0.064083	-2.13486	0.032773	0.058072	GNG2	protein_cc
ENSG0000(	5597.185	-0.00705	0.031913	-0.22086	0.825204	0.871823	SMARCB1	protein_cc
ENSG0000(	5595.953	-0.29324	0.037921	-7.733	1.05E-14	1.52E-13	ZER1	protein_cc
ENSG0000(	5592.987	-0.08569	0.026206	-3.26982	0.001076	0.002607	PSMD1	protein_cc
ENSG0000(	5589.231	-0.12312	0.10881	-1.13147	0.257856	0.345769	CD14	protein_cc
ENSG0000(	5588.312	-0.18973	0.036676	-5.17328	2.30E-07	1.03E-06	GNA13	protein_cc
ENSG0000(	5588.114	-0.01944	0.028118	-0.69147	0.48927	0.583853	PAPSS1	protein_cc
ENSG0000(	5587.092	0.363116	0.036651	9.907486	3.86E-23	2.33E-21	ASNA1	protein_cc
ENSG0000(	5584.015	0.142235	0.063548	2.238226	0.025206	0.045877	CTSL	protein_cc

ENSG0000(	5583.625	0.365405	0.104477	3.497463	0.00047	0.001218	SEZ6	protein_cc
ENSG0000(	5579.862	-0.6553	0.147821	-4.43306	9.29E-06	3.22E-05	STMN2	protein_cc
ENSG0000(	5579.542	-0.01124	0.043319	-0.25947	0.795273	0.848374	SMC1A	protein_cc
ENSG0000(	5578.744	-0.25957	0.026421	-9.82439	8.84E-23	4.93E-21	SAR1A	protein_cc
ENSG0000(	5576.291	-0.15529	0.121632	-1.27672	0.2017	0.28133	SHD	protein_cc
ENSG0000(	5575.537	0.552088	0.112308	4.915844	8.84E-07	3.60E-06	S100A10	protein_cc
ENSG0000(	5575.465	-0.35185	0.042785	-8.22356	1.98E-16	3.82E-15	FAM219A	protein_cc
ENSG0000(	5563.751	-0.07963	0.026666	-2.98636	0.002823	0.00632	UBE2R2	protein_cc
ENSG0000(	5562.783	-0.06125	0.026003	-2.35556	0.018495	0.034708	BTBD1	protein_cc
ENSG0000(	5561.743	-0.48082	0.090068	-5.33838	9.38E-08	4.46E-07	CALCRL	protein_cc
ENSG0000(	5555.698	-0.49511	0.056498	-8.76326	1.90E-18	5.06E-17	KIAA1109	protein_cc
ENSG0000(	5552.583	0.148053	0.036795	4.023758	5.73E-05	0.000175	GIPC1	protein_cc
ENSG0000(	5550.774	0.258617	0.048667	5.314075	1.07E-07	5.06E-07	COX5B	protein_cc
ENSG0000(	5550.687	-0.40752	0.064259	-6.34189	2.27E-10	1.63E-09	ASTN2	protein_cc
ENSG0000(	5550.222	-0.29839	0.073404	-4.06511	4.80E-05	0.000148	PCDH9	protein_cc
ENSG0000(	5549.741	0.358026	0.054478	6.571978	4.97E-11	3.96E-10	KDELR1	protein_cc
ENSG0000(	5549.395	-0.11495	0.092358	-1.24457	0.21329	0.294806	ITGB2	protein_cc
ENSG0000(	5546.742	-0.2612	0.080113	-3.26041	0.001113	0.002687	SOX6	protein_cc
ENSG0000(	5539.869	0.000911	0.09195	0.009909	0.992094	0.994634	CTGF	protein_cc
ENSG0000(	5538.477	-0.13956	0.029051	-4.80391	1.56E-06	6.10E-06	IP6K1	protein_cc
ENSG0000(	5536.605	0.834836	0.101153	8.253224	1.54E-16	3.04E-15	MEST	protein_cc
ENSG0000(	5536.468	0.006488	0.032822	0.197664	0.843308	0.886259	PSMB5	protein_cc
ENSG0000(	5535.678	0.566851	0.099726	5.684066	1.32E-08	7.16E-08	MRC2	protein_cc
ENSG0000(	5532.975	0.569147	0.059866	9.507017	1.96E-21	8.66E-20	SNRPD2	protein_cc
ENSG0000(	5529.99	-0.30148	0.030018	-10.0433	9.84E-24	6.80E-22	VAPB	protein_cc
ENSG0000(	5529.893	0.004118	0.036406	0.113107	0.909946	0.936355	WHSC1	protein_cc
ENSG0000(	5527.759	0.002924	0.036404	0.08032	0.935983	0.95531	SUMO3	protein_cc
ENSG0000(	5525.259	-0.28017	0.041285	-6.78619	1.15E-11	1.01E-10	KIAA0430	protein_cc
ENSG0000(	5520.161	0.113734	0.027947	4.069562	4.71E-05	0.000146	TPD52L2	protein_cc
ENSG0000(	5515.652	-0.0672	0.025112	-2.67607	0.007449	0.015314	RAB5A	protein_cc
ENSG0000(	5515.471	0.083259	0.028482	2.923264	0.003464	0.007625	EIF2S3	protein_cc
ENSG0000(	5514.286	-0.13758	0.039639	-3.47089	0.000519	0.001334	KMT2E	protein_cc
ENSG0000(	5508.125	-0.21592	0.037592	-5.74378	9.26E-09	5.16E-08	WHSC1L1	protein_cc
ENSG0000(	5507.994	-0.26468	0.039359	-6.72472	1.76E-11	1.50E-10	TMEM245	protein_cc
ENSG0000(	5506.528	-0.22553	0.054728	-4.12095	3.77E-05	0.000118	ENDOD1	protein_cc
ENSG0000(	5504.555	-0.20001	0.093684	-2.13496	0.032764	0.05806	LMO3	protein_cc
ENSG0000(	5502.334	-0.17545	0.057933	-3.02852	0.002458	0.00558	NAV2	protein_cc
ENSG0000(	5499.841	-0.12697	0.025901	-4.90229	9.47E-07	3.84E-06	SMG7	protein_cc
ENSG0000(	5498.941	0.106922	0.026869	3.979378	6.91E-05	0.000208	NELFB	protein_cc
ENSG0000(	5498.099	0.149237	0.030811	4.843582	1.28E-06	5.07E-06	UBE2N	protein_cc
ENSG0000(	5496.27	0.558173	0.103863	5.37411	7.70E-08	3.71E-07	F3	protein_cc
ENSG0000(	5495.013	-0.27502	0.045657	-6.02354	1.71E-09	1.07E-08	ZFYVE20	protein_cc
ENSG0000(	5490.86	0.367784	0.095564	3.84856	0.000119	0.000343	PTRF	protein_cc
ENSG0000(	5490.041	-0.60455	0.075075	-8.05262	8.10E-16	1.41E-14	PTPRD	protein_cc
ENSG0000(	5486.545	-0.11297	0.090726	-1.24514	0.21308	0.294554	FBXO32	protein_cc
ENSG0000(	5482.238	-0.4043	0.046744	-8.6493	5.18E-18	1.30E-16	SMAP2	protein_cc
ENSG0000(	5479.784	-0.07425	0.03405	-2.18047	0.029223	0.052412	TOR1AIP1	protein_cc
ENSG0000(	5479.534	-0.0469	0.08869	-0.52878	0.596961	0.682099	AKAP12	protein_cc
ENSG0000(	5478.797	-0.05166	0.031793	-1.62473	0.104221	0.160062	YPEL5	protein_cc
ENSG0000(	5475.756	-0.71188	0.071222	-9.99526	1.60E-23	1.06E-21	PSD3	protein_cc
ENSG0000(	5475.364	-0.4277	0.042737	-10.0076	1.41E-23	9.47E-22	ABI1	protein_cc
ENSG0000(	5469.215	0.100734	0.019037	5.291616	1.21E-07	5.67E-07	TSN	protein_cc
ENSG0000(	5465.555	-0.26463	0.042347	-6.24901	4.13E-10	2.86E-09	TP53BP2	protein_cc
ENSG0000(	5465.16	-0.14509	0.030117	-4.81763	1.45E-06	5.73E-06	SEC63	protein_cc
ENSG0000(	5464.005	-0.10529	0.024994	-4.21279	2.52E-05	8.14E-05	UBE2L3	protein_cc
ENSG0000(	5458.061	0.060393	0.024788	2.436374	0.014835	0.028507	METAP2	protein_cc
ENSG0000(	5457.69	0.132325	0.032542	4.066312	4.78E-05	0.000147	PSMB2	protein_cc
ENSG0000(	5455.992	0.190936	0.034101	5.599139	2.15E-08	1.14E-07	POMGNT1	protein_cc
ENSG0000(	5454.99	0.017369	0.037381	0.464651	0.642182	0.721962	VARS	protein_cc

ENSG0000(	5453.404	-0.00304	0.036759	-0.08282	0.933992	0.953941	PRDX3	protein_cc
ENSG0000(	5450.08	-0.04733	0.043122	-1.09761	0.272374	0.362191	SAFB2	protein_cc
ENSG0000(	5449.83	-0.3043	0.057407	-5.30072	1.15E-07	5.42E-07	ST6GAL1	protein_cc
ENSG0000(	5449.713	-0.06378	0.030116	-2.11786	0.034187	0.060329	MTDH	protein_cc
ENSG0000(	5445.98	0.115809	0.031477	3.679177	0.000234	0.000641	RCN2	protein_cc
ENSG0000(	5445.759	-0.37755	0.036912	-10.2284	1.48E-24	1.19E-22	TRAK1	protein_cc
ENSG0000(	5443.668	-0.04938	0.033945	-1.45468	0.145757	0.213428	CMPK1	protein_cc
ENSG0000(	5439.809	0.086602	0.029768	2.909174	0.003624	0.007947	EIF1AX	protein_cc
ENSG0000(	5438.167	-0.21447	0.032974	-6.50421	7.81E-11	6.05E-10	SLC11A2	protein_cc
ENSG0000(	5434.289	0.032832	0.030987	1.059557	0.289346	0.380705	PURB	protein_cc
ENSG0000(	5433.369	-0.06325	0.039186	-1.61395	0.106537	0.163138	CNOT1	protein_cc
ENSG0000(	5432.133	-0.28742	0.054126	-5.31019	1.10E-07	5.16E-07	AKT3	protein_cc
ENSG0000(	5426.988	-0.60294	0.083289	-7.23915	4.52E-13	4.93E-12	MAPK8IP2	protein_cc
ENSG0000(	5424.843	-0.12465	0.027023	-4.61275	3.97E-06	1.46E-05	XPO7	protein_cc
ENSG0000(	5423.77	0.22573	0.047329	4.769352	1.85E-06	7.17E-06	PIGT	protein_cc
ENSG0000(	5421.181	0.644414	0.063026	10.22454	1.54E-24	1.23E-22	DENND2A	protein_cc
ENSG0000(	5420.392	-0.05431	0.026627	-2.0398	0.04137	0.071474	VPS41	protein_cc
ENSG0000(	5416.441	-0.20035	0.035027	-5.71982	1.07E-08	5.89E-08	IPO13	protein_cc
ENSG0000(	5416.379	0.187176	0.029156	6.419765	1.36E-10	1.02E-09	TEX261	protein_cc
ENSG0000(	5414.894	-0.11244	0.063656	-1.76631	0.077345	0.123643	RP11-553	lincRNA
ENSG0000(	5414.293	0.142786	0.060855	2.346352	0.018958	0.035477	MFGE8	protein_cc
ENSG0000(	5413.535	-0.38326	0.072423	-5.29192	1.21E-07	5.66E-07	TMEM47	protein_cc
ENSG0000(	5406.051	-0.03297	0.069086	-0.47728	0.633161	0.714141	NRP2	protein_cc
ENSG0000(	5403.965	-0.23808	0.036362	-6.54732	5.86E-11	4.62E-10	ANKRD11	protein_cc
ENSG0000(	5403.848	-0.34037	0.034181	-9.95768	2.33E-23	1.50E-21	CCNY	protein_cc
ENSG0000(	5403.557	-0.46606	0.056397	-8.2638	1.41E-16	2.80E-15	CTIF	protein_cc
ENSG0000(	5401.422	1.375403	0.164353	8.36861	5.83E-17	1.24E-15	PDPN	protein_cc
ENSG0000(	5400.586	0.044994	0.046835	0.960688	0.336709	0.431531	KDM1A	protein_cc
ENSG0000(	5396.584	0.263807	0.036489	7.229869	4.83E-13	5.26E-12	PSMD13	protein_cc
ENSG0000(	5396.295	-0.0913	0.030957	-2.94924	0.003186	0.007061	TBCD	protein_cc
ENSG0000(	5391.46	-0.0763	0.030009	-2.54238	0.01101	0.021792	POGK	protein_cc
ENSG0000(	5391.3	-0.09342	0.098391	-0.94944	0.342398	0.437631	JUNB	protein_cc
ENSG0000(	5390.099	-0.26835	0.04576	-5.86425	4.51E-09	2.64E-08	SETD7	protein_cc
ENSG0000(	5388.491	-0.20925	0.092492	-2.26233	0.023677	0.04335	STAB1	protein_cc
ENSG0000(	5386.448	-0.04864	0.023113	-2.10459	0.035327	0.062146	PDCD6IP	protein_cc
ENSG0000(	5384.16	-0.75752	0.107859	-7.0232	2.17E-12	2.14E-11	PSD	protein_cc
ENSG0000(	5383.883	-0.13852	0.037604	-3.68354	0.00023	0.000631	PELP1	protein_cc
ENSG0000(	5383.725	0.180904	0.063321	2.856951	0.004277	0.009246	GAS5	processed
ENSG0000(	5380.697	-0.24644	0.041037	-6.00539	1.91E-09	1.19E-08	RXRA	protein_cc
ENSG0000(	5378.702	0.207683	0.044148	4.704186	2.55E-06	9.66E-06	JAM3	protein_cc
ENSG0000(	5378.361	-0.1453	0.046548	-3.12152	0.001799	0.004188	BPTF	protein_cc
ENSG0000(	5377.792	0.184761	0.042563	4.340854	1.42E-05	4.78E-05	SZRD1	protein_cc
ENSG0000(	5377.747	0.265494	0.031699	8.375343	5.51E-17	1.17E-15	SUN1	protein_cc
ENSG0000(	5377.632	-0.0165	0.023577	-0.69974	0.484089	0.579183	KPNA4	protein_cc
ENSG0000(	5373.955	-0.07869	0.035048	-2.24532	0.024748	0.045118	YARS	protein_cc
ENSG0000(	5369.137	-0.43416	0.048154	-9.01617	1.95E-19	6.09E-18	EPS15	protein_cc
ENSG0000(	5368.442	-0.22361	0.04991	-4.48034	7.45E-06	2.63E-05	TANC2	protein_cc
ENSG0000(	5367.288	0.116	0.037093	3.127261	0.001764	0.004113	GARS	protein_cc
ENSG0000(	5364.565	-0.02523	0.020445	-1.23382	0.217271	0.299402	NPLOC4	protein_cc
ENSG0000(	5364.522	-0.61475	0.110384	-5.56923	2.56E-08	1.33E-07	SYNPO	protein_cc
ENSG0000(	5364.133	-0.20071	0.025409	-7.8991	2.81E-15	4.51E-14	CAB39	protein_cc
ENSG0000(	5362.726	-0.44687	0.055651	-8.02995	9.75E-16	1.68E-14	ARHGEF9	protein_cc
ENSG0000(	5362.578	-0.05459	0.036115	-1.5116	0.130636	0.19419	PNPLA8	protein_cc
ENSG0000(	5362.174	-0.0292	0.033212	-0.87918	0.379303	0.475853	WASL	protein_cc
ENSG0000(	5360.532	-0.25029	0.050469	-4.95932	7.07E-07	2.93E-06	DEAF1	protein_cc
ENSG0000(	5359.293	-0.09805	0.024546	-3.99453	6.48E-05	0.000196	AP1G1	protein_cc
ENSG0000(	5355.415	-0.00632	0.058057	-0.10886	0.913317	0.938911	CD82	protein_cc
ENSG0000(	5354.914	0.486515	0.091711	5.304853	1.13E-07	5.31E-07	FAM20C	protein_cc
ENSG0000(	5352.597	-0.06499	0.032399	-2.00579	0.044879	0.076781	PRKACA	protein_cc

ENSG0000(	5351.191	0.353144	0.084793	4.164783	3.12E-05	9.92E-05	TMBIM1	protein_cc
ENSG0000(	5350.29	-0.28079	0.065652	-4.27692	1.89E-05	6.26E-05	ADD2	protein_cc
ENSG0000(	5350.033	5.73E-05	0.039729	0.001442	0.998849	0.99917	BCL2L1	protein_cc
ENSG0000(	5347.851	0.270509	0.029589	9.142234	6.12E-20	2.07E-18	TMCO1	protein_cc
ENSG0000(	5346.265	-0.00014	0.025437	-0.00559	0.99554	0.997091	TMEM127	protein_cc
ENSG0000(	5344.857	-0.01648	0.029321	-0.56214	0.574019	0.661305	BRD4	protein_cc
ENSG0000(	5343.145	0.01419	0.032609	0.435153	0.663451	0.740049	COPS7A	protein_cc
ENSG0000(	5343.057	0.245088	0.05445	4.501143	6.76E-06	2.40E-05	IRS2	protein_cc
ENSG0000(	5341.37	-0.38469	0.065199	-5.90016	3.63E-09	2.17E-08	DGKZ	protein_cc
ENSG0000(	5333.495	-0.51952	0.043708	-11.8861	1.40E-32	5.32E-30	OAT	protein_cc
ENSG0000(	5331.302	-0.70685	0.088285	-8.00644	1.18E-15	2.01E-14	ATP6V1G2	protein_cc
ENSG0000(	5330.791	-0.41126	0.072078	-5.70571	1.16E-08	6.36E-08	NCAM2	protein_cc
ENSG0000(	5329.704	0.004953	0.048991	0.101094	0.919476	0.943187	RBM6	protein_cc
ENSG0000(	5328.448	-0.29284	0.049984	-5.8588	4.66E-09	2.72E-08	SLC48A1	protein_cc
ENSG0000(	5327.662	0.184221	0.072533	2.539815	0.011091	0.021939	DPYSL5	protein_cc
ENSG0000(	5327.611	0.243116	0.055986	4.342453	1.41E-05	4.75E-05	PPDPF	protein_cc
ENSG0000(	5327.389	0.168558	0.038414	4.387964	1.14E-05	3.91E-05	NME4	protein_cc
ENSG0000(	5324.942	0.085491	0.023323	3.665514	0.000247	0.000674	UBE2D2	protein_cc
ENSG0000(	5322.442	0.054348	0.022133	2.455512	0.014068	0.02718	CGGBP1	protein_cc
ENSG0000(	5317.966	-0.23096	0.03813	-6.05735	1.38E-09	8.83E-09	TAB2	protein_cc
ENSG0000(	5317.685	-0.31876	0.106049	-3.00581	0.002649	0.005971	LINC0092!	lincRNA
ENSG0000(	5313.357	-0.09409	0.027271	-3.45024	0.00056	0.001432	ZMAT2	protein_cc
ENSG0000(	5312.651	-0.58679	0.082883	-7.0798	1.44E-12	1.46E-11	OPHN1	protein_cc
ENSG0000(	5310.982	-0.05693	0.034193	-1.66501	0.09591	0.149024	RNF40	protein_cc
ENSG0000(	5309.321	-0.10498	0.038684	-2.71363	0.006655	0.013819	TAOK3	protein_cc
ENSG0000(	5307.72	-0.11888	0.025946	-4.58177	4.61E-06	1.68E-05	EGLN1	protein_cc
ENSG0000(	5307.173	-0.12064	0.029256	-4.1237	3.73E-05	0.000117	ACOX1	protein_cc
ENSG0000(	5306.45	-0.23904	0.053272	-4.48719	7.22E-06	2.55E-05	TENC1	protein_cc
ENSG0000(	5304.984	0.305335	0.0417	7.322186	2.44E-13	2.78E-12	BTG1	protein_cc
ENSG0000(	5302.16	-0.06959	0.040205	-1.73097	0.083457	0.131948	RGS12	protein_cc
ENSG0000(	5301.644	-0.20196	0.03828	-5.27596	1.32E-07	6.14E-07	PPA1	protein_cc
ENSG0000(	5298.72	-0.40616	0.053654	-7.56993	3.73E-14	4.88E-13	MPDZ	protein_cc
ENSG0000(	5298.367	-0.33236	0.03291	-10.0991	5.58E-24	4.02E-22	ANXA7	protein_cc
ENSG0000(	5295.035	-0.05762	0.024708	-2.33211	0.019695	0.036709	MRFAP1L1	protein_cc
ENSG0000(	5294.211	0.048384	0.066626	0.726208	0.467712	0.563556	ITGB1	protein_cc
ENSG0000(	5293.431	-0.15915	0.034889	-4.5615	5.08E-06	1.84E-05	PEX5	protein_cc
ENSG0000(	5292.575	-0.35119	0.037004	-9.49068	2.30E-21	9.95E-20	TRIM44	protein_cc
ENSG0000(	5291.333	-0.04338	0.017872	-2.42706	0.015222	0.029166	CIAO1	protein_cc
ENSG0000(	5290.033	0.075652	0.061529	1.22954	0.218869	0.301251	RFX4	protein_cc
ENSG0000(	5288.199	0.138679	0.023573	5.88306	4.03E-09	2.38E-08	EI24	protein_cc
ENSG0000(	5285.778	-0.65516	0.077634	-8.43905	3.20E-17	7.06E-16	KBTBD11	protein_cc
ENSG0000(	5284.703	-0.15221	0.061009	-2.4949	0.012599	0.024585	PLXND1	protein_cc
ENSG0000(	5284.307	-0.25341	0.056391	-4.4938	7.00E-06	2.48E-05	ZBTB18	protein_cc
ENSG0000(	5281.971	0.334179	0.039533	8.453147	2.84E-17	6.34E-16	C19orf43	protein_cc
ENSG0000(	5279.287	0.110913	0.040804	2.718214	0.006564	0.013638	FNDC3A	protein_cc
ENSG0000(	5279.101	0.273938	0.123569	2.216881	0.026631	0.048225	ASIC4	protein_cc
ENSG0000(	5271.898	-0.16488	0.031933	-5.16328	2.43E-07	1.08E-06	FBXO7	protein_cc
ENSG0000(	5270.591	-0.16749	0.03377	-4.95984	7.06E-07	2.92E-06	PTPN23	protein_cc
ENSG0000(	5269.89	0.037837	0.064425	0.587296	0.557005	0.646248	FGFR1	protein_cc
ENSG0000(	5267.053	0.109909	0.037052	2.966371	0.003013	0.006712	STRN4	protein_cc
ENSG0000(	5265.378	-0.27871	0.049339	-5.64888	1.61E-08	8.67E-08	EP300	protein_cc
ENSG0000(	5262.717	-0.00073	0.027	-0.02692	0.978525	0.984904	MTA2	protein_cc
ENSG0000(	5261.778	0.185712	0.05604	3.313919	0.00092	0.002256	CHST11	protein_cc
ENSG0000(	5259.319	-0.19432	0.038285	-5.07546	3.87E-07	1.67E-06	CHD8	protein_cc
ENSG0000(	5254.656	0.090769	0.0353	2.57139	0.010129	0.020202	SSU72	protein_cc
ENSG0000(	5254.305	0.140793	0.029228	4.817121	1.46E-06	5.74E-06	GOLGA7	protein_cc
ENSG0000(	5253.883	0.047223	0.046003	1.026515	0.304649	0.397525	GIGYF1	protein_cc
ENSG0000(	5252.657	0.240032	0.037365	6.424016	1.33E-10	9.92E-10	ERH	protein_cc
ENSG0000(	5252.475	-0.1769	0.042098	-4.2021	2.64E-05	8.52E-05	AKAP13	protein_cc

ENSG0000(	5252.358	-0.09337	0.028769	-3.24563	0.001172	0.002818	STAT5B	protein_cc
ENSG0000(	5252.282	0.2613	0.033145	7.883455	3.18E-15	5.07E-14	JTB	protein_cc
ENSG0000(	5251.485	0.158829	0.043761	3.629423	0.000284	0.000768	TCF3	protein_cc
ENSG0000(	5250.272	0.026506	0.034506	0.768159	0.442393	0.539226	SAP30BP	protein_cc
ENSG0000(	5247.263	-0.01669	0.045764	-0.36464	0.715383	0.783244	CERS2	protein_cc
ENSG0000(	5242.082	-0.06901	0.038293	-1.80204	0.07154	0.115467	EIF2AK2	protein_cc
ENSG0000(	5241.861	0.135816	0.019838	6.846327	7.58E-12	6.86E-11	UBE2I	protein_cc
ENSG0000(	5231.624	-0.91877	0.122056	-7.52748	5.17E-14	6.61E-13	SYN1	protein_cc
ENSG0000(	5229.282	-0.24181	0.055372	-4.36708	1.26E-05	4.28E-05	NRBP2	protein_cc
ENSG0000(	5228.294	-0.91667	0.094221	-9.72894	2.27E-22	1.17E-20	ATP2B2	protein_cc
ENSG0000(	5228.178	-0.16319	0.037224	-4.38394	1.17E-05	3.98E-05	RBM25	protein_cc
ENSG0000(	5224.47	-0.10013	0.026925	-3.71891	0.0002	0.000555	NACC1	protein_cc
ENSG0000(	5223.75	-0.14144	0.054259	-2.60685	0.009138	0.018411	KIF21A	protein_cc
ENSG0000(	5222.9	-0.0595	0.055394	-1.07406	0.282795	0.373464	GPSM1	protein_cc
ENSG0000(	5221.869	0.081965	0.035097	2.335348	0.019525	0.036432	CTDSP1	protein_cc
ENSG0000(	5217.97	-0.24024	0.032734	-7.33916	2.15E-13	2.47E-12	ADRBK1	protein_cc
ENSG0000(	5212.813	0.137158	0.028706	4.778075	1.77E-06	6.88E-06	UFC1	protein_cc
ENSG0000(	5212.675	-0.0524	0.05058	-1.03588	0.300257	0.392703	PCDH17	protein_cc
ENSG0000(	5211.651	0.272228	0.048091	5.660721	1.51E-08	8.13E-08	SLC39A1	protein_cc
ENSG0000(	5207.523	-0.08812	0.038796	-2.27129	0.023129	0.042465	ECH1	protein_cc
ENSG0000(	5207.118	-0.52862	0.103185	-5.12303	3.01E-07	1.32E-06	UGT8	protein_cc
ENSG0000(	5203.746	0.363445	0.107749	3.373085	0.000743	0.001854	DCN	protein_cc
ENSG0000(	5203.009	-0.63563	0.09225	-6.89032	5.57E-12	5.14E-11	LIX1	protein_cc
ENSG0000(	5199.031	-0.17947	0.033476	-5.36104	8.27E-08	3.97E-07	KCTD2	protein_cc
ENSG0000(	5197.204	-0.06324	0.064309	-0.98342	0.325401	0.419578	EFS	protein_cc
ENSG0000(	5197.097	0.274234	0.041939	6.53888	6.20E-11	4.87E-10	NDUFB2	protein_cc
ENSG0000(	5193.893	0.065245	0.044234	1.474981	0.140218	0.206382	VEGFB	protein_cc
ENSG0000(	5188.368	0.313403	0.04226	7.416046	1.21E-13	1.45E-12	PPP1CA	protein_cc
ENSG0000(	5187.244	-0.13346	0.02635	-5.065	4.08E-07	1.76E-06	SLC30A9	protein_cc
ENSG0000(	5185.604	0.017042	0.032289	0.527796	0.597641	0.682724	FLII	protein_cc
ENSG0000(	5185.603	-0.00788	0.025344	-0.3111	0.755724	0.816006	NDUFA10	protein_cc
ENSG0000(	5185.294	0.563935	0.054141	10.41596	2.10E-25	1.97E-23	ATP5J2	protein_cc
ENSG0000(	5183.697	-0.30626	0.073132	-4.18779	2.82E-05	9.03E-05	SATB1	protein_cc
ENSG0000(	5182.854	-0.05346	0.037538	-1.42426	0.154371	0.224308	SCAF1	protein_cc
ENSG0000(	5182.073	-0.51344	0.069593	-7.37781	1.61E-13	1.89E-12	NTM	protein_cc
ENSG0000(	5181.728	-0.16426	0.03248	-5.05718	4.26E-07	1.82E-06	DNM1L	protein_cc
ENSG0000(	5178.404	-0.08295	0.032618	-2.54304	0.010989	0.021755	SLC38A10	protein_cc
ENSG0000(	5176.658	0.379017	0.047525	7.975167	1.52E-15	2.55E-14	TMEM230	protein_cc
ENSG0000(	5173.358	-0.18931	0.062316	-3.03791	0.002382	0.005421	MAML2	protein_cc
ENSG0000(	5170.817	0.203812	0.056815	3.587294	0.000334	0.000892	SALL1	protein_cc
ENSG0000(	5166.799	-0.00829	0.034556	-0.23991	0.8104	0.860493	NDUFA5	protein_cc
ENSG0000(	5163.531	0.063785	0.141183	0.451793	0.651418	0.729904	COL20A1	protein_cc
ENSG0000(	5162.174	-0.06734	0.029792	-2.26042	0.023795	0.043551	PNPLA6	protein_cc
ENSG0000(	5158.923	-0.16311	0.143249	-1.13864	0.254852	0.342434	TGFBI	protein_cc
ENSG0000(	5158.851	0.261234	0.052496	4.976224	6.48E-07	2.70E-06	RABAC1	protein_cc
ENSG0000(	5158.28	-0.154	0.114872	-1.34065	0.180036	0.255312	VSIG4	protein_cc
ENSG0000(	5158.218	0.858542	0.110823	7.746957	9.41E-15	1.38E-13	CLIC1	protein_cc
ENSG0000(	5157.52	-0.15369	0.039174	-3.92323	8.74E-05	0.000258	HNRNPUL	protein_cc
ENSG0000(	5157.12	0.219587	0.036064	6.088868	1.14E-09	7.35E-09	HNRNPAE	protein_cc
ENSG0000(	5156.188	-0.01312	0.02474	-0.53041	0.595831	0.68111	MBTPS1	protein_cc
ENSG0000(	5153.956	-0.31273	0.050975	-6.13486	8.52E-10	5.62E-09	MVB12B	protein_cc
ENSG0000(	5146.941	0.03228	0.025843	1.249092	0.211631	0.292956	RELA	protein_cc
ENSG0000(	5145.022	-0.16159	0.044898	-3.59917	0.000319	0.000855	SLC25A4	protein_cc
ENSG0000(	5142.952	0.365624	0.089644	4.078605	4.53E-05	0.00014	CSRP2	protein_cc
ENSG0000(	5142.844	-0.11365	0.032002	-3.55126	0.000383	0.001011	FUBP3	protein_cc
ENSG0000(	5141.775	-0.3138	0.042344	-7.41066	1.26E-13	1.50E-12	ATP13A2	protein_cc
ENSG0000(	5137.56	-0.62372	0.09642	-6.46879	9.88E-11	7.53E-10	PEG3	protein_cc
ENSG0000(	5135.973	6.52E-05	0.02633	0.002476	0.998024	0.998823	STT3B	protein_cc
ENSG0000(	5135.845	-0.01181	0.040655	-0.29052	0.77142	0.828884	AMD1	protein_cc

ENSG0000(	5135.808	-0.09576	0.216266	-0.44279	0.657921	0.735235	RPS4Y1	protein_cc
ENSG0000(	5134.949	-0.16355	0.039073	-4.18586	2.84E-05	9.10E-05	CALCOCC	protein_cc
ENSG0000(	5133.939	-0.39545	0.047207	-8.37685	5.44E-17	1.16E-15	ANKH	protein_cc
ENSG0000(	5133.195	0.121712	0.033536	3.629307	0.000284	0.000768	SRP72	protein_cc
ENSG0000(	5132.784	0.396237	0.049605	7.987896	1.37E-15	2.32E-14	LAMTOR4	protein_cc
ENSG0000(	5132.695	-0.44729	0.039241	-11.3985	4.26E-30	9.67E-28	GBF1	protein_cc
ENSG0000(	5132.135	-0.26209	0.065151	-4.02288	5.75E-05	0.000175	CSF1	protein_cc
ENSG0000(	5131.828	-0.84281	0.077193	-10.9182	9.43E-28	1.38E-25	LYNX1	protein_cc
ENSG0000(	5130.099	-0.0048	0.044365	-0.10825	0.913801	0.93922	RPL36AL	protein_cc
ENSG0000(	5128.582	-0.4606	0.063255	-7.28155	3.30E-13	3.69E-12	C10orf54	protein_cc
ENSG0000(	5126.816	-0.2875	0.049498	-5.80832	6.31E-09	3.61E-08	ARHGAP1	protein_cc
ENSG0000(	5126.01	-0.44268	0.080109	-5.52605	3.28E-08	1.68E-07	SLC25A18	protein_cc
ENSG0000(	5125.94	0.053926	0.020823	2.589724	0.009605	0.019264	CRK	protein_cc
ENSG0000(	5122.784	-0.03281	0.040804	-0.8041	0.421338	0.518303	TRRAP	protein_cc
ENSG0000(	5119.489	-0.18154	0.073681	-2.46383	0.013746	0.026619	PLXNB3	protein_cc
ENSG0000(	5118.564	-1.17409	0.106359	-11.0389	2.48E-28	4.13E-26	NAPB	protein_cc
ENSG0000(	5118.431	-0.18806	0.061161	-3.07476	0.002107	0.004839	SEC14L2	protein_cc
ENSG0000(	5117.796	0.071965	0.033268	2.163215	0.030525	0.054489	MAF1	protein_cc
ENSG0000(	5117.607	0.18577	0.095507	1.9451	0.051763	0.087113	WWTR1	protein_cc
ENSG0000(	5116.043	0.612981	0.094577	6.481278	9.09E-11	6.98E-10	ETV5	protein_cc
ENSG0000(	5111.23	0.376711	0.043237	8.712619	2.97E-18	7.70E-17	LMAN2	protein_cc
ENSG0000(	5110.814	-0.26427	0.053938	-4.8995	9.61E-07	3.89E-06	RBFOX2	protein_cc
ENSG0000(	5107.853	-0.34286	0.035492	-9.66003	4.46E-22	2.17E-20	KIF3B	protein_cc
ENSG0000(	5106.703	0.35177	0.077073	4.564143	5.02E-06	1.82E-05	IQGAP1	protein_cc
ENSG0000(	5104.08	0.243566	0.037578	6.481592	9.08E-11	6.97E-10	AC013394	processed
ENSG0000(	5098.201	0.00343	0.030371	0.112951	0.910069	0.936395	TOP1	protein_cc
ENSG0000(	5097.042	-0.35343	0.043661	-8.09491	5.73E-16	1.03E-14	ST6GALN/	protein_cc
ENSG0000(	5096.854	-0.4862	0.056209	-8.64989	5.15E-18	1.29E-16	CCSER2	protein_cc
ENSG0000(	5095.621	0.100164	0.041479	2.414821	0.015743	0.030067	PEPD	protein_cc
ENSG0000(	5091.393	-0.34831	0.111117	-3.13459	0.001721	0.004021	MEG3	lincRNA
ENSG0000(	5090.44	-0.00249	0.043463	-0.0574	0.954229	0.968295	ABHD4	protein_cc
ENSG0000(	5088.998	-0.01668	0.028954	-0.57623	0.564457	0.652768	SPG7	protein_cc
ENSG0000(	5084.714	0.003227	0.023145	0.139417	0.88912	0.920943	SSB	protein_cc
ENSG0000(	5078.241	0.096133	0.025336	3.794409	0.000148	0.00042	RAD23A	protein_cc
ENSG0000(	5077.583	-0.16207	0.029145	-5.56077	2.69E-08	1.39E-07	FEM1B	protein_cc
ENSG0000(	5076.069	-0.03683	0.065959	-0.5584	0.576572	0.663809	SLC39A14	protein_cc
ENSG0000(	5075.338	-0.09121	0.031161	-2.92712	0.003421	0.007541	EXOC7	protein_cc
ENSG0000(	5073.443	-0.06109	0.04042	-1.5114	0.130686	0.19425	TNKS1BP1	protein_cc
ENSG0000(	5072.674	0.069511	0.018034	3.854459	0.000116	0.000335	CHTOP	protein_cc
ENSG0000(	5071.829	0.205107	0.053365	3.843472	0.000121	0.000349	HN1	protein_cc
ENSG0000(	5070.501	-0.68913	0.104236	-6.61119	3.81E-11	3.10E-10	UNC13A	protein_cc
ENSG0000(	5069.307	-0.7695	0.078233	-9.83596	7.88E-23	4.46E-21	AMOT	protein_cc
ENSG0000(	5063.454	-0.33014	0.044035	-7.4971	6.52E-14	8.22E-13	POLR3H	protein_cc
ENSG0000(	5058.305	-0.25063	0.033523	-7.47653	7.63E-14	9.48E-13	TMEM184	protein_cc
ENSG0000(	5055.401	0.116212	0.037878	3.068038	0.002155	0.004941	DRAP1	protein_cc
ENSG0000(	5055.222	-0.1095	0.034257	-3.19647	0.001391	0.003306	SYMPK	protein_cc
ENSG0000(	5055.205	-0.26742	0.044228	-6.0464	1.48E-09	9.38E-09	PIP5K1C	protein_cc
ENSG0000(	5054.151	0.108877	0.126105	0.863386	0.387925	0.484674	FCGR3A	protein_cc
ENSG0000(	5052.753	0.470672	0.050138	9.387499	6.14E-21	2.44E-19	IDH1	protein_cc
ENSG0000(	5050.76	-0.17622	0.081205	-2.17004	0.030004	0.053658	MTHFD2	protein_cc
ENSG0000(	5049.943	-0.0752	0.022753	-3.30484	0.00095	0.002324	WBP11	protein_cc
ENSG0000(	5048.553	-0.30152	0.032387	-9.31005	1.28E-20	4.82E-19	NOLC1	protein_cc
ENSG0000(	5048.35	-1.62143	0.167975	-9.65282	4.78E-22	2.32E-20	VSNL1	protein_cc
ENSG0000(	5044.775	-0.23478	0.03366	-6.97515	3.06E-12	2.95E-11	KIFAP3	protein_cc
ENSG0000(	5040.574	-0.09158	0.044721	-2.04791	0.040569	0.070268	C1orf21	protein_cc
ENSG0000(	5038.855	-0.08775	0.050086	-1.75195	0.079782	0.126962	NPAS3	protein_cc
ENSG0000(	5037.63	0.285686	0.044468	6.424591	1.32E-10	9.89E-10	CPNE1	protein_cc
ENSG0000(	5035.418	-0.07389	0.029359	-2.51678	0.011843	0.023269	NUFIP2	protein_cc
ENSG0000(	5034.612	-0.23447	0.035369	-6.62915	3.38E-11	2.77E-10	APPL2	protein_cc

ENSG0000(	5034.167	0.116338	0.032585	3.570292	0.000357	0.000947	NUTF2	protein_cc
ENSG0000(	5032.914	-0.24026	0.026599	-9.03283	1.67E-19	5.28E-18	OTUD5	protein_cc
ENSG0000(	5032.085	-0.06189	0.030417	-2.03462	0.041889	0.072275	DCAF8	protein_cc
ENSG0000(	5030.961	0.239084	0.051719	4.622716	3.79E-06	1.40E-05	ANKRD10	protein_cc
ENSG0000(	5030.285	-0.43062	0.042343	-10.1699	2.70E-24	2.05E-22	ATP6V1C1	protein_cc
ENSG0000(	5027.604	-0.48268	0.05566	-8.67196	4.25E-18	1.07E-16	PC	protein_cc
ENSG0000(	5026.794	-0.08728	0.036128	-2.41584	0.015699	0.029989	PABPC4	protein_cc
ENSG0000(	5025.733	0.102359	0.026256	3.898479	9.68E-05	0.000284	RNF216	protein_cc
ENSG0000(	5021.6	-0.28518	0.039347	-7.24769	4.24E-13	4.66E-12	RBM17	protein_cc
ENSG0000(	5021.233	-0.1434	0.040935	-3.50313	0.00046	0.001195	GSK3B	protein_cc
ENSG0000(	5019.743	0.048754	0.029574	1.648534	0.099243	0.153524	VDAC3	protein_cc
ENSG0000(	5017.022	-0.44392	0.047552	-9.33551	1.01E-20	3.87E-19	ACBD5	protein_cc
ENSG0000(	5015.604	0.212349	0.03671	5.784535	7.27E-09	4.12E-08	G6PC3	protein_cc
ENSG0000(	5011.145	0.280186	0.037848	7.402926	1.33E-13	1.59E-12	PSMC2	protein_cc
ENSG0000(	5008.904	0.382378	0.046395	8.241848	1.70E-16	3.32E-15	C7orf50	protein_cc
ENSG0000(	5007.649	-0.06735	0.039019	-1.72596	0.084355	0.133219	SCOC	protein_cc
ENSG0000(	5006.914	0.209683	0.028095	7.463327	8.44E-14	1.04E-12	VMA21	protein_cc
ENSG0000(	5006.614	-0.00991	0.029493	-0.33615	0.73676	0.800785	ABCF1	protein_cc
ENSG0000(	5005.668	-0.05275	0.034398	-1.53361	0.125125	0.187284	GABARAP	protein_cc
ENSG0000(	5005.511	-0.63326	0.063674	-9.94533	2.64E-23	1.66E-21	MYCBP2	protein_cc
ENSG0000(	5005.105	-0.4404	0.054624	-8.06238	7.48E-16	1.32E-14	TNIK	protein_cc
ENSG0000(	5003.659	-0.57114	0.066645	-8.56993	1.04E-17	2.48E-16	IQSEC1	protein_cc
ENSG0000(	4999.333	0.212242	0.048472	4.378653	1.19E-05	4.07E-05	DAP	protein_cc
ENSG0000(	4994.514	-0.17	0.029669	-5.72976	1.01E-08	5.57E-08	KIAA0319l	protein_cc
ENSG0000(	4992.727	-0.03048	0.024216	-1.25879	0.208104	0.288803	PEX19	protein_cc
ENSG0000(	4990.111	-0.1249	0.064222	-1.94476	0.051804	0.087172	FAM131B	protein_cc
ENSG0000(	4989.56	-0.25096	0.053842	-4.66105	3.15E-06	1.17E-05	MEGF9	protein_cc
ENSG0000(	4987.353	-0.21589	0.029063	-7.42824	1.10E-13	1.33E-12	TSPAN14	protein_cc
ENSG0000(	4987.203	0.043594	0.023579	1.848854	0.064479	0.105487	ATF6B	protein_cc
ENSG0000(	4981.128	0.056887	0.037824	1.503986	0.132585	0.196739	ZBED1	protein_cc
ENSG0000(	4980.945	-0.13554	0.040006	-3.38788	0.000704	0.001766	MED13	protein_cc
ENSG0000(	4980.924	0.269076	0.048306	5.570193	2.54E-08	1.33E-07	COX7A2	protein_cc
ENSG0000(	4980.821	-0.30883	0.049161	-6.28192	3.34E-10	2.35E-09	RIN2	protein_cc
ENSG0000(	4980.068	0.152503	0.066519	2.292621	0.02187	0.040365	LITAF	protein_cc
ENSG0000(	4979.125	-0.33944	0.068562	-4.95092	7.39E-07	3.05E-06	DLG5	protein_cc
ENSG0000(	4978.935	-0.04282	0.038113	-1.12352	0.261215	0.349581	MAPKAPK	protein_cc
ENSG0000(	4972.755	-0.21705	0.038651	-5.6156	1.96E-08	1.04E-07	MYO6	protein_cc
ENSG0000(	4971.047	0.177155	0.031433	5.635896	1.74E-08	9.31E-08	LRRC59	protein_cc
ENSG0000(	4962.118	-0.23928	0.044009	-5.43706	5.42E-08	2.68E-07	SMG1	protein_cc
ENSG0000(	4961.058	-0.08114	0.024394	-3.32623	0.00088	0.002167	AGFG1	protein_cc
ENSG0000(	4955.798	-0.11971	0.082006	-1.45982	0.144341	0.211602	PID1	protein_cc
ENSG0000(	4952.995	-0.27211	0.04345	-6.26261	3.79E-10	2.63E-09	MAN2A2	protein_cc
ENSG0000(	4952.792	-0.05965	0.040506	-1.47268	0.140838	0.207191	PNN	protein_cc
ENSG0000(	4949.204	-1.04549	0.085552	-12.2205	2.42E-34	1.31E-31	KIAA0513	protein_cc
ENSG0000(	4949.039	0.00278	0.04474	0.062134	0.950456	0.965816	PABPN1	protein_cc
ENSG0000(	4948.939	-0.66007	0.08376	-7.88048	3.26E-15	5.17E-14	SLC15A2	protein_cc
ENSG0000(	4948.827	0.21818	0.037318	5.846558	5.02E-09	2.92E-08	RSL24D1	protein_cc
ENSG0000(	4947.176	-0.45087	0.066422	-6.78804	1.14E-11	9.98E-11	S1PR1	protein_cc
ENSG0000(	4947.07	-0.02017	0.051673	-0.39043	0.696219	0.76739	ATAT1	protein_cc
ENSG0000(	4946.069	-0.0263	0.030644	-0.85814	0.390817	0.487399	HDAC6	protein_cc
ENSG0000(	4943.992	0.013519	0.025312	0.534113	0.593263	0.678723	ETF1	protein_cc
ENSG0000(	4943.494	-0.29035	0.031183	-9.31118	1.26E-20	4.78E-19	WIPF2	protein_cc
ENSG0000(	4942.792	-0.29702	0.068607	-4.32937	1.50E-05	5.02E-05	SYBU	protein_cc
ENSG0000(	4937.666	-0.14895	0.039152	-3.80456	0.000142	0.000404	PHF2	protein_cc
ENSG0000(	4937.344	-0.28762	0.073944	-3.88976	0.0001	0.000293	COBL	protein_cc
ENSG0000(	4935.092	0.177112	0.026764	6.61765	3.65E-11	2.98E-10	DHX15	protein_cc
ENSG0000(	4934.316	0.145908	0.048392	3.015159	0.002568	0.005808	ARGLU1	protein_cc
ENSG0000(	4933.796	-0.25153	0.077884	-3.22958	0.00124	0.002971	KAL1	protein_cc
ENSG0000(	4931.019	-0.0341	0.042579	-0.80088	0.423203	0.520278	SART1	protein_cc

ENSG0000(	4930.097	0.004627	0.031171	0.148444	0.881992	0.915713	ESD	protein_cc
ENSG0000(	4929.087	0.095079	0.040573	2.343392	0.019109	0.035737	TMEM219	protein_cc
ENSG0000(	4928.074	-0.02955	0.050868	-0.58086	0.561332	0.650113	SH3BP2	protein_cc
ENSG0000(	4926.595	-0.00888	0.027811	-0.31928	0.749515	0.811368	MAPK1IP1	protein_cc
ENSG0000(	4925.847	-0.20384	0.052318	-3.89622	9.77E-05	0.000286	CRYL1	protein_cc
ENSG0000(	4924.572	0.066967	0.036622	1.828584	0.067462	0.109721	KAT7	protein_cc
ENSG0000(	4924.316	-0.02361	0.024872	-0.94911	0.342564	0.437776	VPS4A	protein_cc
ENSG0000(	4924.073	-0.28705	0.07129	-4.02658	5.66E-05	0.000173	ELAVL3	protein_cc
ENSG0000(	4922.885	-0.06408	0.026	-2.46476	0.01371	0.026558	FEZ2	protein_cc
ENSG0000(	4922.542	-0.45857	0.090019	-5.09412	3.50E-07	1.52E-06	STMN4	protein_cc
ENSG0000(	4922.508	-1.23955	0.166865	-7.42847	1.10E-13	1.33E-12	SFRP2	protein_cc
ENSG0000(	4922.107	-0.36168	0.051786	-6.98421	2.86E-12	2.78E-11	SSBP3	protein_cc
ENSG0000(	4915.598	-0.02308	0.035042	-0.65852	0.510207	0.603462	TXNRD1	protein_cc
ENSG0000(	4912.973	-0.03377	0.039681	-0.85113	0.3947	0.491257	HERPUD1	protein_cc
ENSG0000(	4907.535	0.080763	0.065848	1.226508	0.220008	0.302545	NOTCH3	protein_cc
ENSG0000(	4905.798	0.249064	0.106394	2.340958	0.019234	0.035948	CD24	protein_cc
ENSG0000(	4905.166	-0.01302	0.036655	-0.35521	0.722433	0.788779	KDM3A	protein_cc
ENSG0000(	4904.078	-0.08957	0.038471	-2.32829	0.019897	0.037048	GOT2	protein_cc
ENSG0000(	4902.198	-0.33567	0.042125	-7.96852	1.61E-15	2.68E-14	MGAT5	protein_cc
ENSG0000(	4900.254	-0.01366	0.02305	-0.59254	0.553489	0.643167	SRP68	protein_cc
ENSG0000(	4891.395	-0.17027	0.029591	-5.75402	8.71E-09	4.88E-08	UBAP1	protein_cc
ENSG0000(	4890.976	0.132804	0.105436	1.259563	0.207827	0.288505	FKBP5	protein_cc
ENSG0000(	4890.771	0.035249	0.026093	1.350915	0.176723	0.251351	SEC23B	protein_cc
ENSG0000(	4889.063	-0.09444	0.05683	-1.66173	0.096568	0.149887	CDC42EP1	protein_cc
ENSG0000(	4887.831	-0.60191	0.120445	-4.99736	5.81E-07	2.44E-06	MYRF	protein_cc
ENSG0000(	4886.649	-0.20997	0.032801	-6.40148	1.54E-10	1.14E-09	NGRN	protein_cc
ENSG0000(	4885.588	-0.73357	0.09463	-7.75196	9.05E-15	1.33E-13	DLGAP1	protein_cc
ENSG0000(	4885.25	-0.39883	0.053291	-7.48396	7.21E-14	9.02E-13	NPTN	protein_cc
ENSG0000(	4880.167	0.000733	0.023083	0.031768	0.974657	0.982228	NSFL1C	protein_cc
ENSG0000(	4879.751	-0.18242	0.026872	-6.78838	1.13E-11	9.96E-11	LONP2	protein_cc
ENSG0000(	4878.511	0.107509	0.027437	3.918399	8.91E-05	0.000263	NRBP1	protein_cc
ENSG0000(	4878.083	-0.00855	0.04573	-0.18687	0.851765	0.892819	CPSF1	protein_cc
ENSG0000(	4877.228	0.37097	0.083189	4.459381	8.22E-06	2.87E-05	TNFRSF1A	protein_cc
ENSG0000(	4876.022	0.225195	0.033857	6.651353	2.90E-11	2.40E-10	COPB1	protein_cc
ENSG0000(	4874.883	-0.15893	0.032676	-4.86384	1.15E-06	4.61E-06	PHF10	protein_cc
ENSG0000(	4871.757	-0.17459	0.048425	-3.60545	0.000312	0.000836	CPEB4	protein_cc
ENSG0000(	4870.697	-0.22943	0.041371	-5.5457	2.93E-08	1.51E-07	BBX	protein_cc
ENSG0000(	4867.339	1.167187	0.094092	12.40473	2.46E-35	1.63E-32	TSPAN31	protein_cc
ENSG0000(	4862.98	-0.06606	0.023142	-2.8545	0.00431	0.009309	ALKBH5	protein_cc
ENSG0000(	4862.47	-0.00198	0.026538	-0.07443	0.940667	0.958616	PTCD3	protein_cc
ENSG0000(	4860.367	-0.3487	0.074326	-4.69142	2.71E-06	1.02E-05	GSTM3	protein_cc
ENSG0000(	4859.341	-0.32376	0.033127	-9.77339	1.46E-22	7.86E-21	C6orf106	protein_cc
ENSG0000(	4859.164	0.075297	0.040691	1.850469	0.064246	0.105148	HDGFRP2	protein_cc
ENSG0000(	4857.782	0.583303	0.095829	6.086945	1.15E-09	7.44E-09	CA2	protein_cc
ENSG0000(	4856.877	-1.33127	0.132428	-10.0528	8.93E-24	6.22E-22	PHYHIP	protein_cc
ENSG0000(	4856.114	0.197804	0.046552	4.249137	2.15E-05	7.02E-05	UQCRH	protein_cc
ENSG0000(	4855.11	-0.2777	0.056664	-4.90084	9.54E-07	3.87E-06	IDI1	protein_cc
ENSG0000(	4854.374	-0.34239	0.07069	-4.84355	1.28E-06	5.07E-06	BHLHE41	protein_cc
ENSG0000(	4852.446	-0.18827	0.033496	-5.62059	1.90E-08	1.01E-07	HDGFRP3	protein_cc
ENSG0000(	4851.067	-0.09571	0.031909	-2.99942	0.002705	0.006082	PSEN1	protein_cc
ENSG0000(	4850.653	-0.17343	0.088129	-1.96786	0.049084	0.083132	CERCAM	protein_cc
ENSG0000(	4843.675	-0.23098	0.04089	-5.64886	1.62E-08	8.67E-08	ENTPD6	protein_cc
ENSG0000(	4843.524	0.120022	0.030879	3.886835	0.000102	0.000296	COPS8	protein_cc
ENSG0000(	4843.356	0.216619	0.031906	6.789225	1.13E-11	9.90E-11	POR	protein_cc
ENSG0000(	4839.158	-0.08691	0.089221	-0.9741	0.330007	0.424385	LTBP4	protein_cc
ENSG0000(	4838.964	-0.4907	0.054462	-9.00987	2.06E-19	6.41E-18	HERC1	protein_cc
ENSG0000(	4838.896	-0.3338	0.039464	-8.45847	2.71E-17	6.07E-16	KIAA0232	protein_cc
ENSG0000(	4836.818	-0.88557	0.078106	-11.338	8.50E-30	1.82E-27	DNAJC6	protein_cc
ENSG0000(	4833.122	-0.26682	0.046356	-5.75579	8.62E-09	4.83E-08	ZC3H13	protein_cc

ENSG0000	4832.654	-0.28785	0.083704	-3.43887	0.000584	0.001489	SNTA1	protein_co
ENSG0000	4831.914	-0.27611	0.033519	-8.23733	1.76E-16	3.44E-15	RANBP2	protein_co
ENSG0000	4831.696	0.270658	0.046712	5.794174	6.87E-09	3.91E-08	WTAP	protein_co
ENSG0000	4826.638	0.146589	0.025863	5.667872	1.45E-08	7.82E-08	PDCD6	protein_co
ENSG0000	4824.541	0.205446	0.039938	5.144126	2.69E-07	1.19E-06	SAE1	protein_co
ENSG0000	4823.626	0.027369	0.024627	1.111339	0.266423	0.35538	TBC1D10E	protein_co
ENSG0000	4822.93	-0.00262	0.020772	-0.12611	0.899643	0.928777	TNFAIP1	protein_co
ENSG0000	4821.539	0.037489	0.04266	0.878789	0.379515	0.47609	DPY19L3	protein_co
ENSG0000	4821.124	0.186968	0.044658	4.186647	2.83E-05	9.07E-05	GNAI3	protein_co
ENSG0000	4814.857	-0.21658	0.081284	-2.66447	0.007711	0.015807	MMP16	protein_co
ENSG0000	4814.303	-0.21294	0.080305	-2.65164	0.00801	0.016361	FAM110B	protein_co
ENSG0000	4814.11	-0.61021	0.068939	-8.85155	8.63E-19	2.42E-17	NSF	protein_co
ENSG0000	4813.985	0.159412	0.03512	4.539062	5.65E-06	2.03E-05	ERP29	protein_co
ENSG0000	4813.53	-0.25885	0.049618	-5.21693	1.82E-07	8.26E-07	SH3GLB2	protein_co
ENSG0000	4813.402	-0.69439	0.088883	-7.81237	5.61E-15	8.54E-14	ATP8A1	protein_co
ENSG0000	4811.239	0.069401	0.031303	2.217098	0.026616	0.048202	DEGS1	protein_co
ENSG0000	4809.918	0.039125	0.029942	1.306709	0.191312	0.268864	POLR2B	protein_co
ENSG0000	4809.851	-0.0028	0.029295	-0.09551	0.923909	0.94649	CDIPT	protein_co
ENSG0000	4809.363	-0.09473	0.0295	-3.21132	0.001321	0.003151	MCM3AP	protein_co
ENSG0000	4803.734	-0.05263	0.085684	-0.61425	0.539048	0.630001	CTSZ	protein_co
ENSG0000	4800.986	0.093832	0.03445	2.723688	0.006456	0.013438	TMED7	protein_co
ENSG0000	4799.96	-0.17783	0.032812	-5.41971	5.97E-08	2.93E-07	UBQLN4	protein_co
ENSG0000	4798.963	-0.00451	0.028307	-0.15944	0.873324	0.909136	IFNAR1	protein_co
ENSG0000	4797.146	0.08592	0.024785	3.466629	0.000527	0.001354	AP1M1	protein_co
ENSG0000	4795.556	0.130074	0.03554	3.659933	0.000252	0.000687	STT3A	protein_co
ENSG0000	4793.414	-0.12228	0.056877	-2.14999	0.031556	0.056129	GALNT10	protein_co
ENSG0000	4791.36	0.021846	0.038851	0.562291	0.573918	0.661245	CUEDC1	protein_co
ENSG0000	4790.227	-0.50569	0.058005	-8.71804	2.83E-18	7.37E-17	MAPK10	protein_co
ENSG0000	4789.954	-0.14346	0.029339	-4.88974	1.01E-06	4.08E-06	AHSA1	protein_co
ENSG0000	4789.145	-0.25525	0.056684	-4.50299	6.70E-06	2.38E-05	ZHX2	protein_co
ENSG0000	4789.083	-0.71285	0.074022	-9.63028	5.96E-22	2.83E-20	CLSTN3	protein_co
ENSG0000	4786.652	0.292319	0.070562	4.142723	3.43E-05	0.000108	CLDN5	protein_co
ENSG0000	4786.456	-0.23023	0.074937	-3.07234	0.002124	0.004875	APOLD1	protein_co
ENSG0000	4786.444	-0.08975	0.027372	-3.27899	0.001042	0.002529	ZFP91	protein_co
ENSG0000	4784.631	0.154228	0.033059	4.665199	3.08E-06	1.15E-05	SPCS1	protein_co
ENSG0000	4780.607	-0.1109	0.033803	-3.28087	0.001035	0.002514	ISCU	protein_co
ENSG0000	4778.868	-0.18087	0.029964	-6.03621	1.58E-09	9.96E-09	YME1L1	protein_co
ENSG0000	4778.073	-0.28774	0.034689	-8.29495	1.09E-16	2.19E-15	MECP2	protein_co
ENSG0000	4777.611	-0.09919	0.03561	-2.78546	0.005345	0.011326	PPFIA1	protein_co
ENSG0000	4775.917	0.274427	0.036248	7.57082	3.71E-14	4.85E-13	C1QBP	protein_co
ENSG0000	4774.093	-0.14669	0.033823	-4.33687	1.45E-05	4.86E-05	CLUH	protein_co
ENSG0000	4773.831	-0.35426	0.050271	-7.04699	1.83E-12	1.83E-11	KMT2D	protein_co
ENSG0000	4772.947	-0.42955	0.043652	-9.84047	7.54E-23	4.27E-21	ALAD	protein_co
ENSG0000	4770.748	-0.02047	0.048131	-0.42534	0.670586	0.745713	KDM4B	protein_co
ENSG0000	4770.343	-0.40203	0.060054	-6.69439	2.17E-11	1.83E-10	PPM1K	protein_co
ENSG0000	4767.582	0.216627	0.035238	6.147478	7.87E-10	5.22E-09	DEK	protein_co
ENSG0000	4767.134	0.414637	0.047222	8.780595	1.63E-18	4.37E-17	ZNHIT1	protein_co
ENSG0000	4764.222	0.010821	0.024082	0.44936	0.653172	0.731331	GID8	protein_co
ENSG0000	4763.562	-0.7651	0.099268	-7.70742	1.28E-14	1.82E-13	EDIL3	protein_co
ENSG0000	4762.96	-0.03502	0.060678	-0.57721	0.563796	0.652193	ITM2A	protein_co
ENSG0000	4762.226	-0.02861	0.038449	-0.74405	0.456849	0.553288	ADIPOR1	protein_co
ENSG0000	4761.276	-0.45858	0.087602	-5.23478	1.65E-07	7.56E-07	CDK5R1	protein_co
ENSG0000	4760.548	0.116652	0.047837	2.438498	0.014748	0.02837	STAT2	protein_co
ENSG0000	4756.935	0.01963	0.052341	0.375028	0.707639	0.777	LIMK1	protein_co
ENSG0000	4754.893	0.174734	0.036728	4.757532	1.96E-06	7.56E-06	ATP5H	protein_co
ENSG0000	4753.948	0.079263	0.032941	2.406226	0.016118	0.030726	CPSF3L	protein_co
ENSG0000	4748.608	-0.11405	0.041513	-2.74735	0.006008	0.012581	CUEDC2	protein_co
ENSG0000	4748.558	0.320909	0.035007	9.167051	4.86E-20	1.67E-18	PHB	protein_co
ENSG0000	4745.047	-0.02749	0.043687	-0.62921	0.529212	0.620886	CCND3	protein_co

ENSG0000(	4744.904	0.08685	0.034975	2.48321	0.01302	0.025331	TMEM50A	protein_cc
ENSG0000(	4742.221	-0.59344	0.068503	-8.66306	4.59E-18	1.16E-16	CRY2	protein_cc
ENSG0000(	4742.048	0.163656	0.023652	6.91922	4.54E-12	4.26E-11	COG4	protein_cc
ENSG0000(	4740.818	-0.63492	0.071924	-8.82773	1.07E-18	2.95E-17	NAP1L3	protein_cc
ENSG0000(	4740.551	-0.25234	0.043457	-5.80682	6.37E-09	3.64E-08	SMARCA1	protein_cc
ENSG0000(	4740.454	0.159399	0.023373	6.819819	9.12E-12	8.13E-11	RNF114	protein_cc
ENSG0000(	4739.473	-0.20264	0.034963	-5.79565	6.81E-09	3.88E-08	SPG20	protein_cc
ENSG0000(	4739.095	-0.37184	0.05307	-7.00658	2.44E-12	2.39E-11	SEMA4B	protein_cc
ENSG0000(	4737.667	-0.54863	0.078432	-6.995	2.65E-12	2.59E-11	TCEAL2	protein_cc
ENSG0000(	4737.263	-0.14732	0.037625	-3.91563	9.02E-05	0.000266	TSPAN5	protein_cc
ENSG0000(	4734.69	-0.19354	0.03916	-4.94233	7.72E-07	3.17E-06	GTPBP1	protein_cc
ENSG0000(	4732.253	0.110294	0.031102	3.546159	0.000391	0.00103	SEC13	protein_cc
ENSG0000(	4731.908	-0.13084	0.064653	-2.02369	0.043002	0.073953	GPR162	protein_cc
ENSG0000(	4731.851	0.142361	0.133773	1.064203	0.287237	0.37843	FLNC	protein_cc
ENSG0000(	4722.296	-0.10507	0.046708	-2.24952	0.024479	0.04469	EHD1	protein_cc
ENSG0000(	4721.253	-0.01091	0.043503	-0.25076	0.801998	0.853707	VASH1	protein_cc
ENSG0000(	4716.377	0.208771	0.050958	4.096949	4.19E-05	0.00013	SYPL1	protein_cc
ENSG0000(	4715.662	0.085961	0.052509	1.637076	0.101615	0.156668	RP11-166	sense_ove
ENSG0000(	4715.655	0.434878	0.051673	8.415973	3.90E-17	8.48E-16	UQCRQ	protein_cc
ENSG0000(	4715.063	-0.0823	0.043005	-1.9138	0.055646	0.09277	SHISA4	protein_cc
ENSG0000(	4714.853	0.01096	0.031319	0.349944	0.72638	0.791848	LRRC41	protein_cc
ENSG0000(	4714.523	0.070577	0.029413	2.39948	0.016418	0.031225	RBM10	protein_cc
ENSG0000(	4713.265	-0.0398	0.027233	-1.46166	0.143835	0.21095	TXNL1	protein_cc
ENSG0000(	4710.088	-0.0461	0.024585	-1.87513	0.060775	0.100169	SPPL3	protein_cc
ENSG0000(	4709.91	0.023203	0.035083	0.661376	0.508371	0.601861	SRSF4	protein_cc
ENSG0000(	4705.856	0.014039	0.024297	0.577805	0.563396	0.651934	API5	protein_cc
ENSG0000(	4705.835	-0.0143	0.032419	-0.44102	0.659196	0.736324	ERLIN2	protein_cc
ENSG0000(	4705.414	0.007784	0.073726	0.105577	0.915918	0.940785	CYBRD1	protein_cc
ENSG0000(	4704.413	0.165292	0.038114	4.336757	1.45E-05	4.86E-05	NDUFS3	protein_cc
ENSG0000(	4704.373	-0.55072	0.061142	-9.00717	2.11E-19	6.55E-18	ARHGAP3	protein_cc
ENSG0000(	4703.011	-0.26117	0.053678	-4.86551	1.14E-06	4.57E-06	ZNF275	protein_cc
ENSG0000(	4701.073	-0.08839	0.062593	-1.41218	0.157897	0.228409	MSMO1	protein_cc
ENSG0000(	4699.102	0.074055	0.03563	2.078462	0.037667	0.065792	RNF145	protein_cc
ENSG0000(	4698.253	0.525653	0.05329	9.864049	5.96E-23	3.48E-21	SNRPB	protein_cc
ENSG0000(	4696.935	-0.03511	0.059521	-0.58983	0.555306	0.644805	SHC2	protein_cc
ENSG0000(	4696.526	0.041333	0.044805	0.922524	0.356255	0.451901	GAB1	protein_cc
ENSG0000(	4694.265	-0.08486	0.031996	-2.65232	0.007994	0.016333	SCAMP2	protein_cc
ENSG0000(	4689.08	-0.30215	0.05325	-5.67416	1.39E-08	7.56E-08	RPS6KA2	protein_cc
ENSG0000(	4687.484	-0.23872	0.029968	-7.96601	1.64E-15	2.73E-14	DCAF11	protein_cc
ENSG0000(	4686.857	-0.15792	0.035943	-4.39348	1.12E-05	3.82E-05	PPP6R2	protein_cc
ENSG0000(	4684.144	-0.23381	0.048222	-4.8486	1.24E-06	4.95E-06	MIB1	protein_cc
ENSG0000(	4682.063	-0.11436	0.038876	-2.94175	0.003264	0.007221	ASAP1	protein_cc
ENSG0000(	4679.843	-0.20444	0.039333	-5.19774	2.02E-07	9.10E-07	TBC1D16	protein_cc
ENSG0000(	4677.737	-0.041	0.026797	-1.52989	0.126043	0.18848	PAIP2	protein_cc
ENSG0000(	4675.448	0.157292	0.030708	5.122087	3.02E-07	1.33E-06	PSMB6	protein_cc
ENSG0000(	4671.709	-0.21531	0.02749	-7.83219	4.79E-15	7.40E-14	SNX27	protein_cc
ENSG0000(	4671.239	-0.30098	0.031816	-9.45992	3.08E-21	1.30E-19	UBQLN2	protein_cc
ENSG0000(	4669.723	0.190259	0.02384	7.980631	1.46E-15	2.45E-14	NELFCD	protein_cc
ENSG0000(	4668.342	-0.15905	0.038774	-4.102	4.10E-05	0.000128	ATPAF1	protein_cc
ENSG0000(	4668.323	-0.41689	0.05801	-7.18653	6.65E-13	7.10E-12	USP34	protein_cc
ENSG0000(	4668.146	-0.16637	0.052091	-3.19388	0.001404	0.003333	NKTR	protein_cc
ENSG0000(	4667.397	-0.24571	0.047552	-5.16725	2.38E-07	1.06E-06	IGF2R	protein_cc
ENSG0000(	4666.177	-0.18367	0.038351	-4.78906	1.68E-06	6.54E-06	FOXK1	protein_cc
ENSG0000(	4665.05	0.153539	0.038096	4.030305	5.57E-05	0.00017	ERI3	protein_cc
ENSG0000(	4662.89	-0.11736	0.029213	-4.01757	5.88E-05	0.000179	HGS	protein_cc
ENSG0000(	4662.633	-0.4502	0.061428	-7.32901	2.32E-13	2.65E-12	ITPR2	protein_cc
ENSG0000(	4662.5	-0.4014	0.052022	-7.71604	1.20E-14	1.72E-13	FMN2	protein_cc
ENSG0000(	4660.162	0.143662	0.029828	4.816387	1.46E-06	5.76E-06	MEPCE	protein_cc
ENSG0000(	4656.834	0.063392	0.112831	0.561835	0.574228	0.661509	SNX22	protein_cc

ENSG0000(	4656.549	0.072275	0.027065	2.670404	0.007576	0.015552	AMZ2	protein_cc
ENSG0000(	4655.691	-0.0309	0.031661	-0.97582	0.329155	0.423564	CRAT	protein_cc
ENSG0000(	4653.878	0.118892	0.022714	5.234369	1.66E-07	7.57E-07	TFG	protein_cc
ENSG0000(	4652.574	0.202699	0.033435	6.062488	1.34E-09	8.56E-09	SMARCD1	protein_cc
ENSG0000(	4645.714	0.058819	0.030284	1.94227	0.052104	0.087633	DLD	protein_cc
ENSG0000(	4645.504	0.077868	0.021851	3.56351	0.000366	0.00097	YTHDF1	protein_cc
ENSG0000(	4644.044	0.041705	0.042773	0.975034	0.329543	0.423955	SMARCC1	protein_cc
ENSG0000(	4642.732	0.410968	0.06058	6.783895	1.17E-11	1.02E-10	SPRED1	protein_cc
ENSG0000(	4641.699	-0.05138	0.024303	-2.11409	0.034507	0.060847	SCYL1	protein_cc
ENSG0000(	4636.741	-0.2236	0.049401	-4.52615	6.01E-06	2.15E-05	SLC25A1	protein_cc
ENSG0000(	4635.777	0.097134	0.077083	1.260125	0.207624	0.288273	ARHGDIB	protein_cc
ENSG0000(	4632.88	-0.58064	0.056357	-10.303	6.83E-25	5.82E-23	FARP1	protein_cc
ENSG0000(	4632.845	0.051373	0.039925	1.28672	0.198192	0.277066	PES1	protein_cc
ENSG0000(	4630.996	0.203122	0.028927	7.02198	2.19E-12	2.16E-11	ATP6V1G1	protein_cc
ENSG0000(	4630.913	-0.13971	0.071378	-1.9573	0.050312	0.084964	THBS2	protein_cc
ENSG0000(	4627.857	0.5965	0.065769	9.069615	1.19E-19	3.85E-18	TRAF4	protein_cc
ENSG0000(	4616.232	-0.03709	0.048046	-0.77201	0.440106	0.537028	DDAH2	protein_cc
ENSG0000(	4615.988	-0.42809	0.056486	-7.57874	3.49E-14	4.58E-13	SPIRE1	protein_cc
ENSG0000(	4613.595	0.124042	0.037823	3.279575	0.00104	0.002524	SEC11A	protein_cc
ENSG0000(	4612.583	-0.25644	0.03627	-7.07032	1.55E-12	1.56E-11	PCYOX1	protein_cc
ENSG0000(	4612.505	-0.05784	0.035275	-1.63964	0.101079	0.155977	PAF1	protein_cc
ENSG0000(	4611.786	-0.26441	0.03282	-8.05644	7.86E-16	1.38E-14	KIAA0368	protein_cc
ENSG0000(	4611.316	-0.46569	0.057565	-8.08981	5.98E-16	1.07E-14	RUSC2	protein_cc
ENSG0000(	4609.761	0.129439	0.032275	4.010549	6.06E-05	0.000184	TMEM129	protein_cc
ENSG0000(	4609.29	-0.53588	0.0479	-11.1875	4.69E-29	8.94E-27	PINK1	protein_cc
ENSG0000(	4608.692	0.369772	0.091869	4.02498	5.70E-05	0.000174	LAMC1	protein_cc
ENSG0000(	4608.473	-0.01276	0.073735	-0.17311	0.862565	0.900958	TSPAN11	protein_cc
ENSG0000(	4606.571	-0.24617	0.062173	-3.95947	7.51E-05	0.000224	KIF13A	protein_cc
ENSG0000(	4605.462	0.221093	0.045686	4.83939	1.30E-06	5.17E-06	PSME1	protein_cc
ENSG0000(	4602.662	-0.07281	0.032885	-2.21415	0.026818	0.048516	YIPF6	protein_cc
ENSG0000(	4596.232	0.061912	0.048651	1.272574	0.203169	0.283052	CHD9	protein_cc
ENSG0000(	4596.088	0.059379	0.033741	1.759843	0.078434	0.125132	CSNK1A1	protein_cc
ENSG0000(	4594.997	-0.00589	0.050977	-0.11548	0.908065	0.934867	KHDRBS3	protein_cc
ENSG0000(	4594.05	-0.10604	0.028224	-3.75702	0.000172	0.000482	YTHDC1	protein_cc
ENSG0000(	4593.702	0.242855	0.043018	5.645402	1.65E-08	8.84E-08	DNMT1	protein_cc
ENSG0000(	4592.475	0.548048	0.051982	10.54309	5.47E-26	5.81E-24	ATRAID	protein_cc
ENSG0000(	4591.782	0.251378	0.044496	5.649504	1.61E-08	8.64E-08	TMA7	protein_cc
ENSG0000(	4591.198	0.106067	0.029119	3.642575	0.00027	0.000732	VEZF1	protein_cc
ENSG0000(	4588.2	-0.25419	0.031264	-8.13066	4.27E-16	7.85E-15	CHP1	protein_cc
ENSG0000(	4586.678	-0.62792	0.059715	-10.5154	7.33E-26	7.66E-24	LZTS3	protein_cc
ENSG0000(	4586.529	0.023212	0.063886	0.363328	0.71636	0.784	SYNE2	protein_cc
ENSG0000(	4586.204	-0.05889	0.025241	-2.33308	0.019644	0.036624	C9orf78	protein_cc
ENSG0000(	4584.384	0.032274	0.026479	1.218877	0.222891	0.305838	CNPPD1	protein_cc
ENSG0000(	4583.996	0.291451	0.060525	4.815394	1.47E-06	5.79E-06	VMP1	protein_cc
ENSG0000(	4582.629	0.10402	0.021293	4.885175	1.03E-06	4.17E-06	ELAVL1	protein_cc
ENSG0000(	4582.265	0.07588	0.034262	2.214688	0.026781	0.048463	TCEA2	protein_cc
ENSG0000(	4581.728	-0.0305	0.061921	-0.49262	0.62228	0.704743	HMGCR	protein_cc
ENSG0000(	4581.448	0.334163	0.043318	7.714222	1.22E-14	1.74E-13	CDC25B	protein_cc
ENSG0000(	4581.355	-0.52512	0.059749	-8.78876	1.51E-18	4.09E-17	PPP2R5A	protein_cc
ENSG0000(	4580.638	-0.17036	0.046574	-3.65772	0.000254	0.000693	RSF1	protein_cc
ENSG0000(	4578.639	0.181072	0.027277	6.63818	3.18E-11	2.61E-10	SRSF7	protein_cc
ENSG0000(	4576.848	-0.44935	0.070735	-6.35262	2.12E-10	1.53E-09	UST	protein_cc
ENSG0000(	4575.462	-0.11647	0.027735	-4.1993	2.68E-05	8.62E-05	RMDN3	protein_cc
ENSG0000(	4573.485	0.335993	0.081562	4.119473	3.80E-05	0.000119	MCAM	protein_cc
ENSG0000(	4573.438	0.095337	0.039117	2.4372	0.014801	0.028452	SPCS3	protein_cc
ENSG0000(	4572.046	-0.18257	0.04022	-4.53919	5.65E-06	2.03E-05	SLC41A1	protein_cc
ENSG0000(	4571.643	0.219069	0.026769	8.18375	2.75E-16	5.20E-15	FAM32A	protein_cc
ENSG0000(	4571.632	-0.01533	0.119822	-0.12795	0.898189	0.927836	IFI44L	protein_cc
ENSG0000(	4570.662	-0.18519	0.040115	-4.61656	3.90E-06	1.44E-05	BAZ2A	protein_cc

ENSG0000(	4568.672	-0.1418	0.036481	-3.88686	0.000102	0.000296	ECHS1	protein_cc
ENSG0000(	4568.098	0.262963	0.028191	9.327945	1.08E-20	4.14E-19	IP6K2	protein_cc
ENSG0000(	4566.299	0.183243	0.029493	6.212991	5.20E-10	3.54E-09	DNAJB6	protein_cc
ENSG0000(	4566.016	0.045866	0.048159	0.952371	0.340909	0.436012	ZNF358	protein_cc
ENSG0000(	4563.998	0.350336	0.045162	7.757266	8.68E-15	1.28E-13	APOA1BP	protein_cc
ENSG0000(	4563.769	0.259976	0.03863	6.729866	1.70E-11	1.45E-10	BCAP29	protein_cc
ENSG0000(	4563.623	0.097487	0.064211	1.518233	0.128956	0.192074	B4GALNT4	protein_cc
ENSG0000(	4563.073	-0.21021	0.074871	-2.80771	0.00499	0.010634	GAREML	protein_cc
ENSG0000(	4560.121	0.041141	0.026255	1.56698	0.117119	0.177048	RBM23	protein_cc
ENSG0000(	4558.468	0.105197	0.025728	4.088817	4.34E-05	0.000135	IDH3B	protein_cc
ENSG0000(	4556.909	-0.40995	0.095945	-4.27279	1.93E-05	6.37E-05	EPHB1	protein_cc
ENSG0000(	4556.727	0.134816	0.028118	4.794608	1.63E-06	6.37E-06	CSE1L	protein_cc
ENSG0000(	4554.705	-0.1084	0.044677	-2.42628	0.015254	0.029223	PITPNC1	protein_cc
ENSG0000(	4554.182	0.198681	0.028902	6.874345	6.23E-12	5.71E-11	SLC35A4	protein_cc
ENSG0000(	4554.035	-0.22497	0.046815	-4.80554	1.54E-06	6.06E-06	CHD6	protein_cc
ENSG0000(	4553.185	0.381607	0.044963	8.487199	2.12E-17	4.82E-16	GSTK1	protein_cc
ENSG0000(	4551.141	0.331523	0.035127	9.437808	3.81E-21	1.58E-19	WDR83OS	protein_cc
ENSG0000(	4550.64	-0.02637	0.033005	-0.79882	0.424393	0.521475	SH2B1	protein_cc
ENSG0000(	4550.354	-0.19894	0.045373	-4.38449	1.16E-05	3.97E-05	TLE3	protein_cc
ENSG0000(	4549.287	-0.21203	0.031618	-6.70593	2.00E-11	1.69E-10	SETX	protein_cc
ENSG0000(	4548.454	-0.00448	0.062438	-0.07171	0.942836	0.960042	SIPA1L1	protein_cc
ENSG0000(	4546.601	-0.61024	0.088999	-6.85677	7.04E-12	6.41E-11	BZRAP1	protein_cc
ENSG0000(	4546.014	0.249058	0.04087	6.093958	1.10E-09	7.14E-09	NDUFB11	protein_cc
ENSG0000(	4545.646	-0.25559	0.045939	-5.56365	2.64E-08	1.37E-07	BIRC6	protein_cc
ENSG0000(	4542.963	-0.06133	0.030969	-1.98032	0.047667	0.080952	SLC39A9	protein_cc
ENSG0000(	4542.261	-0.09845	0.036702	-2.68226	0.007313	0.01506	NEK9	protein_cc
ENSG0000(	4541.92	-0.04251	0.024362	-1.74506	0.080975	0.128579	UBE2Z	protein_cc
ENSG0000(	4540.723	-0.05301	0.029392	-1.80347	0.071315	0.115155	PHKB	protein_cc
ENSG0000(	4539.57	-0.13441	0.037353	-3.59827	0.00032	0.000858	ARRB2	protein_cc
ENSG0000(	4538.66	-0.06108	0.024388	-2.5045	0.012263	0.024001	SMU1	protein_cc
ENSG0000(	4538.158	-0.32123	0.03456	-9.29485	1.47E-20	5.50E-19	DESI1	protein_cc
ENSG0000(	4534.364	-0.02828	0.020453	-1.38251	0.166815	0.239478	GORASP2	protein_cc
ENSG0000(	4531.27	0.696413	0.070676	9.853588	6.61E-23	3.83E-21	DPY19L1	protein_cc
ENSG0000(	4529.827	-0.36206	0.063821	-5.67299	1.40E-08	7.61E-08	INF2	protein_cc
ENSG0000(	4529.617	0.147357	0.034173	4.312095	1.62E-05	5.40E-05	ATP13A1	protein_cc
ENSG0000(	4529.005	0.079144	0.059397	1.33246	0.182709	0.25847	RAB13	protein_cc
ENSG0000(	4527.743	0.09302	0.026924	3.454958	0.00055	0.001409	DDX41	protein_cc
ENSG0000(	4525.86	0.02493	0.029019	0.859114	0.390277	0.486873	APEH	protein_cc
ENSG0000(	4524.981	0.130048	0.032592	3.990115	6.60E-05	0.000199	TYK2	protein_cc
ENSG0000(	4522.629	0.013283	0.047899	0.277307	0.781545	0.837116	QSOX1	protein_cc
ENSG0000(	4521.85	0.135796	0.057185	2.374666	0.017565	0.033117	SLC4A2	protein_cc
ENSG0000(	4520.668	0.105037	0.094236	1.114618	0.265014	0.3538	SRGN	protein_cc
ENSG0000(	4518.575	0.218358	0.068377	3.193442	0.001406	0.003337	MXRA7	protein_cc
ENSG0000(	4514.93	-0.0621	0.029495	-2.10545	0.035252	0.06203	BRD7	protein_cc
ENSG0000(	4512.365	-0.05243	0.033777	-1.55217	0.120622	0.181483	NCOA6	protein_cc
ENSG0000(	4510.121	0.080494	0.024861	3.237782	0.001205	0.002892	DPF2	protein_cc
ENSG0000(	4506.121	0.071418	0.039459	1.809926	0.070307	0.113746	HSPA13	protein_cc
ENSG0000(	4505.654	-0.05744	0.040251	-1.42696	0.15359	0.223307	FMNL3	protein_cc
ENSG0000(	4505.59	0.22087	0.039789	5.551058	2.84E-08	1.47E-07	ATP1B3	protein_cc
ENSG0000(	4504.016	-0.00279	0.034638	-0.08069	0.935688	0.95513	CFL2	protein_cc
ENSG0000(	4502.055	-0.45062	0.058015	-7.76725	8.02E-15	1.19E-13	WFS1	protein_cc
ENSG0000(	4501.639	-0.10836	0.036248	-2.98926	0.002797	0.006266	SIN3B	protein_cc
ENSG0000(	4501.334	-0.21905	0.036729	-5.96388	2.46E-09	1.51E-08	GIGYF2	protein_cc
ENSG0000(	4499.754	0.052441	0.037306	1.405681	0.159819	0.230861	MYL6B	protein_cc
ENSG0000(	4498.803	0.072119	0.031788	2.268751	0.023283	0.04271	TMEM87A	protein_cc
ENSG0000(	4495.304	0.287986	0.039053	7.374194	1.65E-13	1.94E-12	TRMT112	protein_cc
ENSG0000(	4492.576	-0.43455	0.057979	-7.49502	6.63E-14	8.34E-13	DOCK10	protein_cc
ENSG0000(	4491.279	-0.34469	0.070242	-4.90718	9.24E-07	3.75E-06	PIANP	protein_cc
ENSG0000(	4490.93	0.007417	0.022111	0.335444	0.73729	0.801256	DNAJA2	protein_cc

ENSG0000(	4490.756	-0.16266	0.044518	-3.6539	0.000258	0.000703	FOXO3	protein_cc
ENSG0000(	4490.1	0.008495	0.020728	0.409828	0.681932	0.755159	RANBP3	protein_cc
ENSG0000(	4488.779	0.247381	0.040968	6.038453	1.56E-09	9.83E-09	TMEM14C	protein_cc
ENSG0000(	4485.979	-0.20389	0.056188	-3.62861	0.000285	0.00077	AFAP1	protein_cc
ENSG0000(	4485.432	-0.23906	0.074936	-3.19023	0.001422	0.003371	NCALD	protein_cc
ENSG0000(	4485.283	0.205745	0.034873	5.899874	3.64E-09	2.17E-08	NELFE	protein_cc
ENSG0000(	4484.67	0.028462	0.026479	1.074881	0.282428	0.373075	PRPF40A	protein_cc
ENSG0000(	4484.042	0.195857	0.050404	3.885726	0.000102	0.000298	LDLRAD3	protein_cc
ENSG0000(	4480.719	0.246999	0.04065	6.076269	1.23E-09	7.91E-09	FUNDC2	protein_cc
ENSG0000(	4478.397	-0.09706	0.027741	-3.49874	0.000467	0.001213	GAK	protein_cc
ENSG0000(	4477.911	-0.36356	0.064214	-5.66166	1.50E-08	8.09E-08	TENM4	protein_cc
ENSG0000(	4477.579	-0.08617	0.028256	-3.04962	0.002291	0.005228	AGPAT6	protein_cc
ENSG0000(	4476.005	0.357598	0.040313	8.87048	7.28E-19	2.07E-17	POLR2G	protein_cc
ENSG0000(	4475.623	-0.03327	0.050659	-0.65674	0.511349	0.6045	MAGI2	protein_cc
ENSG0000(	4474.507	-0.17046	0.051663	-3.29942	0.000969	0.002366	BNIP3	protein_cc
ENSG0000(	4474.381	0.252641	0.036855	6.855085	7.13E-12	6.48E-11	RBCK1	protein_cc
ENSG0000(	4474.049	0.067833	0.050446	1.344664	0.178734	0.253824	SEC11C	protein_cc
ENSG0000(	4473.046	-0.00589	0.023503	-0.25074	0.802016	0.853707	USP10	protein_cc
ENSG0000(	4471.915	-0.45792	0.049408	-9.26821	1.89E-20	6.91E-19	DOCK1	protein_cc
ENSG0000(	4470.671	-0.47768	0.075459	-6.33033	2.45E-10	1.75E-09	ATP2B1	protein_cc
ENSG0000(	4470.537	-0.2228	0.049797	-4.47425	7.67E-06	2.70E-05	TPCN1	protein_cc
ENSG0000(	4470.094	-0.84236	0.092748	-9.08223	1.06E-19	3.46E-18	CA11	protein_cc
ENSG0000(	4469.572	0.074741	0.039148	1.909198	0.056237	0.09363	TIMM50	protein_cc
ENSG0000(	4468.934	0.108056	0.044158	2.44704	0.014403	0.027782	MOB1A	protein_cc
ENSG0000(	4467.883	-0.14805	0.031032	-4.77069	1.84E-06	7.12E-06	MBNL1	protein_cc
ENSG0000(	4465.594	-0.01532	0.064378	-0.23795	0.81192	0.861795	IGF1R	protein_cc
ENSG0000(	4464.179	-0.03963	0.026196	-1.51272	0.130351	0.193815	RTCB	protein_cc
ENSG0000(	4463.591	-0.04401	0.039531	-1.11333	0.265565	0.35442	CARKD	protein_cc
ENSG0000(	4463.29	-0.30176	0.052395	-5.75939	8.44E-09	4.73E-08	RRAGD	protein_cc
ENSG0000(	4462.754	-0.58813	0.051301	-11.4644	1.99E-30	5.12E-28	FLNB	protein_cc
ENSG0000(	4462.51	-0.22536	0.047535	-4.74106	2.13E-06	8.16E-06	KAT6A	protein_cc
ENSG0000(	4462.395	0.116899	0.025093	4.658691	3.18E-06	1.19E-05	RALA	protein_cc
ENSG0000(	4462.212	-0.08633	0.046522	-1.85576	0.063488	0.104099	PLXNA1	protein_cc
ENSG0000(	4461.628	-0.09278	0.043187	-2.14844	0.031679	0.056326	SCARB1	protein_cc
ENSG0000(	4461.2	-0.05868	0.029655	-1.97873	0.047847	0.081221	U2SURP	protein_cc
ENSG0000(	4458.34	-0.50133	0.055271	-9.07037	1.19E-19	3.84E-18	MOAP1	protein_cc
ENSG0000(	4457.981	0.135352	0.028102	4.816465	1.46E-06	5.76E-06	ABCF2	protein_cc
ENSG0000(	4456.838	-0.51173	0.051689	-9.90019	4.15E-23	2.49E-21	RAPGEF2	protein_cc
ENSG0000(	4455.782	0.110896	0.046542	2.382703	0.017186	0.032493	DDX39B	protein_cc
ENSG0000(	4454.422	0.19342	0.036667	5.275088	1.33E-07	6.16E-07	PSMC4	protein_cc
ENSG0000(	4453.49	0.394469	0.050007	7.888331	3.06E-15	4.88E-14	PHPT1	protein_cc
ENSG0000(	4452.245	-0.0541	0.036944	-1.46452	0.143051	0.210017	ST5	protein_cc
ENSG0000(	4451.998	-0.09077	0.045564	-1.99225	0.046343	0.078996	CDC42BP/	protein_cc
ENSG0000(	4451.371	-0.16342	0.111627	-1.46395	0.143208	0.210197	TMEM100	protein_cc
ENSG0000(	4450.912	-0.23977	0.060104	-3.98932	6.63E-05	0.0002	TMEM178	protein_cc
ENSG0000(	4449.631	-0.61731	0.11924	-5.17704	2.25E-07	1.01E-06	CLDN11	protein_cc
ENSG0000(	4447.628	0.051016	0.034606	1.474209	0.140425	0.206665	TNIP1	protein_cc
ENSG0000(	4447.235	0.018482	0.037438	0.493658	0.621548	0.70405	AKIRIN1	protein_cc
ENSG0000(	4444.387	-0.25299	0.0507	-4.98998	6.04E-07	2.53E-06	CASKIN2	protein_cc
ENSG0000(	4442.849	-0.20615	0.028746	-7.17143	7.42E-13	7.87E-12	FAM65A	protein_cc
ENSG0000(	4439.948	0.116245	0.036925	3.148152	0.001643	0.003852	SAT2	protein_cc
ENSG0000(	4437.562	-0.28655	0.03516	-8.15008	3.64E-16	6.75E-15	OPA1	protein_cc
ENSG0000(	4435.464	-0.21573	0.035149	-6.13753	8.38E-10	5.53E-09	RNF220	protein_cc
ENSG0000(	4434.228	0.080836	0.032517	2.485951	0.012921	0.025156	MTHFD1	protein_cc
ENSG0000(	4432.433	-0.95665	0.121183	-7.89426	2.92E-15	4.67E-14	SCN3B	protein_cc
ENSG0000(	4430.798	-0.10751	0.046184	-2.32797	0.019914	0.037078	MTA1	protein_cc
ENSG0000(	4430.614	-0.58209	0.075784	-7.6809	1.58E-14	2.21E-13	LONRF2	protein_cc
ENSG0000(	4429.929	-0.00404	0.02521	-0.16036	0.872596	0.908528	CUL4B	protein_cc
ENSG0000(	4428.187	-0.16085	0.045865	-3.50699	0.000453	0.00118	PATZ1	protein_cc

ENSG0000(	4427.03	0.143326	0.063911	2.242601	0.024923	0.045413	BCHE	protein_cc
ENSG0000(	4426.23	0.078913	0.023674	3.333322	0.000858	0.002118	NXF1	protein_cc
ENSG0000(	4425.119	-0.04555	0.0296	-1.53877	0.123861	0.185663	FAM208A	protein_cc
ENSG0000(	4423.832	0.026303	0.086318	0.304723	0.760577	0.820083	SCG5	protein_cc
ENSG0000(	4422.566	-0.69567	0.127712	-5.44722	5.12E-08	2.54E-07	PTPRN	protein_cc
ENSG0000(	4421.64	0.262656	0.042712	6.149464	7.77E-10	5.16E-09	NDUFB5	protein_cc
ENSG0000(	4421.455	-0.17537	0.046655	-3.7588	0.000171	0.000479	GPR125	protein_cc
ENSG0000(	4420.482	0.24063	0.040731	5.907853	3.47E-09	2.07E-08	RUVBL2	protein_cc
ENSG0000(	4418.751	0.016787	0.024274	0.691572	0.489206	0.583793	PDS5A	protein_cc
ENSG0000(	4418.098	0.049056	0.02967	1.653404	0.098249	0.152151	MTCH2	protein_cc
ENSG0000(	4415.884	-0.51985	0.070562	-7.36733	1.74E-13	2.03E-12	FRY	protein_cc
ENSG0000(	4415.396	0.035178	0.022606	1.55609	0.119687	0.180267	ARIH2	protein_cc
ENSG0000(	4415.315	0.125199	0.028852	4.339333	1.43E-05	4.81E-05	MAN1B1	protein_cc
ENSG0000(	4414.437	-0.03436	0.062626	-0.54858	0.583296	0.669816	ATRX	protein_cc
ENSG0000(	4412.176	0.252234	0.16859	1.496133	0.134619	0.19924	SERPINE1	protein_cc
ENSG0000(	4411.825	-0.08513	0.026655	-3.19386	0.001404	0.003333	CLCN7	protein_cc
ENSG0000(	4409.5	-0.06532	0.039816	-1.64061	0.100879	0.155702	LPIN2	protein_cc
ENSG0000(	4408.026	-0.43965	0.148604	-2.95855	0.003091	0.006871	L1CAM	protein_cc
ENSG0000(	4407.725	-0.17894	0.058933	-3.03643	0.002394	0.005446	LGMN	protein_cc
ENSG0000(	4406.594	0.125337	0.03449	3.63403	0.000279	0.000755	ATP5J	protein_cc
ENSG0000(	4406.097	-0.03624	0.065293	-0.55503	0.578877	0.665894	ZNF704	protein_cc
ENSG0000(	4405.521	0.356698	0.04563	7.817183	5.40E-15	8.25E-14	ANAPC11	protein_cc
ENSG0000(	4405.298	0.206321	0.06647	3.103974	0.001909	0.004421	PPP1R15B	protein_cc
ENSG0000(	4403.905	-0.23452	0.057359	-4.0887	4.34E-05	0.000135	JMJD1C	protein_cc
ENSG0000(	4403.874	-0.15045	0.050214	-2.99607	0.002735	0.00614	NADK2	protein_cc
ENSG0000(	4403.137	-0.19188	0.067057	-2.86137	0.004218	0.009134	ACSS1	protein_cc
ENSG0000(	4402.722	-0.11485	0.121025	-0.94898	0.342632	0.43783	SDC4	protein_cc
ENSG0000(	4401.803	-0.00755	0.029056	-0.25974	0.795061	0.848224	FARSA	protein_cc
ENSG0000(	4400.936	-0.31612	0.034475	-9.16939	4.76E-20	1.64E-18	BRWD1	protein_cc
ENSG0000(	4399.341	-0.18859	0.03225	-5.84754	4.99E-09	2.90E-08	SGPL1	protein_cc
ENSG0000(	4399.077	0.118986	0.041635	2.857811	0.004266	0.009223	CSTB	protein_cc
ENSG0000(	4397.915	0.016957	0.038934	0.435534	0.663175	0.739906	LEPROT	protein_cc
ENSG0000(	4396.248	-0.61207	0.137944	-4.43709	9.12E-06	3.17E-05	MOG	protein_cc
ENSG0000(	4396.07	-0.02458	0.027862	-0.88226	0.377637	0.474168	ADAM10	protein_cc
ENSG0000(	4393.601	-0.37739	0.119618	-3.15501	0.001605	0.00377	VAT1L	protein_cc
ENSG0000(	4393.276	0.087701	0.032281	2.716779	0.006592	0.013692	SLC25A11	protein_cc
ENSG0000(	4391.244	-1.42545	0.159912	-8.91394	4.93E-19	1.43E-17	GRIN1	protein_cc
ENSG0000(	4387.933	-0.16591	0.028123	-5.89965	3.64E-09	2.17E-08	ATMIN	protein_cc
ENSG0000(	4387.814	0.01848	0.054748	0.337549	0.735703	0.799915	C2orf72	protein_cc
ENSG0000(	4386.05	-0.17524	0.036431	-4.81017	1.51E-06	5.93E-06	NR3C1	protein_cc
ENSG0000(	4383.766	-0.60206	0.087468	-6.88316	5.85E-12	5.39E-11	PLEKHH1	protein_cc
ENSG0000(	4383.087	0.342875	0.05219	6.56975	5.04E-11	4.02E-10	OST4	protein_cc
ENSG0000(	4381.777	-0.04101	0.029816	-1.37533	0.169028	0.242198	SFXN1	protein_cc
ENSG0000(	4378.96	0.138776	0.037623	3.688568	0.000226	0.000619	CNIH1	protein_cc
ENSG0000(	4378.891	-0.19404	0.03241	-5.98709	2.14E-09	1.32E-08	EXOC5	protein_cc
ENSG0000(	4378.777	-0.29467	0.038089	-7.7362	1.02E-14	1.49E-13	FNBP1	protein_cc
ENSG0000(	4373.895	-0.12931	0.030139	-4.29027	1.78E-05	5.92E-05	RTF1	protein_cc
ENSG0000(	4370.956	-0.6056	0.058751	-10.3079	6.49E-25	5.57E-23	TLN2	protein_cc
ENSG0000(	4368.635	0.09751	0.033608	2.901408	0.003715	0.008125	FBXW5	protein_cc
ENSG0000(	4367.98	-0.09481	0.029878	-3.17311	0.001508	0.003561	MED15	protein_cc
ENSG0000(	4367.758	0.443084	0.050061	8.85081	8.69E-19	2.44E-17	NDUFB7	protein_cc
ENSG0000(	4365.077	-0.18921	0.040068	-4.72213	2.33E-06	8.90E-06	NPC1	protein_cc
ENSG0000(	4364.285	-0.38125	0.08039	-4.74244	2.11E-06	8.11E-06	EEPD1	protein_cc
ENSG0000(	4363.136	0.533315	0.073622	7.244007	4.36E-13	4.78E-12	TFRC	protein_cc
ENSG0000(	4360.953	-0.17625	0.055086	-3.19953	0.001377	0.003274	SACS	protein_cc
ENSG0000(	4360.175	-1.10491	0.159011	-6.94862	3.69E-12	3.51E-11	SLC14A1	protein_cc
ENSG0000(	4359.003	0.009669	0.024349	0.397096	0.691296	0.763355	NAA50	protein_cc
ENSG0000(	4358.575	0.032723	0.035252	0.928263	0.353271	0.44883	GALNT1	protein_cc
ENSG0000(	4355.638	-0.08393	0.031501	-2.66438	0.007713	0.01581	GBA2	protein_cc

ENSG0000(	4355.341	0.050679	0.110354	0.459238	0.646063	0.725286	LPPR1	protein_cc
ENSG0000(	4352.632	-0.44451	0.044855	-9.90997	3.77E-23	2.28E-21	KLHL5	protein_cc
ENSG0000(	4350.858	0.042267	0.032236	1.311177	0.189798	0.267065	CHMP5	protein_cc
ENSG0000(	4345.591	-0.13274	0.040825	-3.25158	0.001148	0.002765	AC243547	protein_cc
ENSG0000(	4343.65	-0.09231	0.045173	-2.04349	0.041004	0.070935	OSBPL8	protein_cc
ENSG0000(	4343.125	0.038819	0.040808	0.951261	0.341472	0.436637	ORAI2	protein_cc
ENSG0000(	4341.843	-0.37008	0.056653	-6.53244	6.47E-11	5.08E-10	MTUS1	protein_cc
ENSG0000(	4340.129	-0.13154	0.031706	-4.14883	3.34E-05	0.000106	SLTM	protein_cc
ENSG0000(	4339.402	0.346608	0.052568	6.593552	4.29E-11	3.46E-10	COX7B	protein_cc
ENSG0000(	4338.412	0.103828	0.031492	3.296972	0.000977	0.002384	SUCLG1	protein_cc
ENSG0000(	4337.357	-0.74126	0.084256	-8.7976	1.40E-18	3.81E-17	MRVI1	protein_cc
ENSG0000(	4337.218	-0.78196	0.073848	-10.5888	3.36E-26	3.70E-24	MEF2C	protein_cc
ENSG0000(	4337.042	-0.839	0.06785	-12.3655	4.02E-35	2.58E-32	CAMK1D	protein_cc
ENSG0000(	4336.838	0.017321	0.021737	0.796846	0.425541	0.522604	SPOP	protein_cc
ENSG0000(	4335.537	0.093603	0.038917	2.405174	0.016165	0.030812	HDAC2	protein_cc
ENSG0000(	4334.001	0.039409	0.029042	1.356926	0.174805	0.249191	GATAD2A	protein_cc
ENSG0000(	4331.392	0.029803	0.028993	1.027903	0.303995	0.39681	ASXL1	protein_cc
ENSG0000(	4328.571	-0.24135	0.042282	-5.70814	1.14E-08	6.28E-08	ARHGEF1:	protein_cc
ENSG0000(	4327.118	-0.55521	0.082728	-6.71128	1.93E-11	1.64E-10	FAM167A	protein_cc
ENSG0000(	4326.744	-0.33703	0.052216	-6.45448	1.09E-10	8.23E-10	LDLRAD4	protein_cc
ENSG0000(	4325.337	1.140193	0.131803	8.650761	5.12E-18	1.28E-16	LGALS3	protein_cc
ENSG0000(	4325.08	0.259749	0.05053	5.140494	2.74E-07	1.21E-06	MAGT1	protein_cc
ENSG0000(	4324.856	-0.21931	0.038064	-5.76153	8.34E-09	4.68E-08	MTOR	protein_cc
ENSG0000(	4324.563	-0.06417	0.024799	-2.58771	0.009662	0.019365	CNOT7	protein_cc
ENSG0000(	4321.066	-0.7101	0.093539	-7.59147	3.16E-14	4.19E-13	PDK4	protein_cc
ENSG0000(	4320.368	0.050115	0.032234	1.554712	0.120015	0.180681	DNAJC10	protein_cc
ENSG0000(	4319.921	-0.06767	0.023563	-2.87183	0.004081	0.00886	C16orf62	protein_cc
ENSG0000(	4318.935	-0.84587	0.120001	-7.0489	1.80E-12	1.81E-11	SNAP91	protein_cc
ENSG0000(	4316.126	-0.00233	0.163959	-0.0142	0.988669	0.992182	CD163	protein_cc
ENSG0000(	4315.975	0.044503	0.019192	2.318851	0.020403	0.037901	PSMD11	protein_cc
ENSG0000(	4315.78	0.016908	0.033305	0.507667	0.611687	0.695235	CPSF6	protein_cc
ENSG0000(	4312.453	-0.13358	0.030171	-4.4275	9.53E-06	3.30E-05	RNF20	protein_cc
ENSG0000(	4311.858	-0.12936	0.037646	-3.43615	0.00059	0.001502	AK3	protein_cc
ENSG0000(	4310.467	-0.10351	0.039687	-2.60808	0.009105	0.018357	LRRC58	protein_cc
ENSG0000(	4310.184	-0.21402	0.029858	-7.16798	7.61E-13	8.07E-12	MIA3	protein_cc
ENSG0000(	4310.1	-0.16136	0.031707	-5.08919	3.60E-07	1.56E-06	CDC42SE2	protein_cc
ENSG0000(	4308.841	-1.25312	0.146697	-8.54224	1.32E-17	3.10E-16	SELL	protein_cc
ENSG0000(	4307.866	-0.09659	0.100105	-0.96493	0.334582	0.429268	SCN3A	protein_cc
ENSG0000(	4307.481	0.354883	0.037894	9.36509	7.60E-21	2.97E-19	BRAT1	protein_cc
ENSG0000(	4306.706	0.50763	0.074986	6.769694	1.29E-11	1.13E-10	TAP1	protein_cc
ENSG0000(	4306.514	-0.06875	0.043598	-1.57698	0.1148	0.174067	GRIK5	protein_cc
ENSG0000(	4306.186	-0.01727	0.036911	-0.46777	0.639946	0.720032	PRAF2	protein_cc
ENSG0000(	4303.104	-0.46371	0.093783	-4.94455	7.63E-07	3.14E-06	KIAA1244	protein_cc
ENSG0000(	4303.051	-0.75428	0.070983	-10.6262	2.25E-26	2.59E-24	PPM1L	protein_cc
ENSG0000(	4302.914	-0.11382	0.034346	-3.31387	0.00092	0.002256	DHX38	protein_cc
ENSG0000(	4302.701	0.263145	0.060584	4.343494	1.40E-05	4.73E-05	SPATS2L	protein_cc
ENSG0000(	4301.94	-0.124	0.053454	-2.31976	0.020354	0.037822	NUDT4	protein_cc
ENSG0000(	4301.396	-0.65363	0.090145	-7.25092	4.14E-13	4.55E-12	MRO	protein_cc
ENSG0000(	4301.246	-0.12719	0.090955	-1.3984	0.161993	0.233502	PTCH1	protein_cc
ENSG0000(	4300.893	-0.25279	0.043977	-5.74812	9.02E-09	5.04E-08	VPS13C	protein_cc
ENSG0000(	4299.499	-0.09107	0.02911	-3.12855	0.001757	0.004097	PPARD	protein_cc
ENSG0000(	4299.092	0.258264	0.079687	3.240983	0.001191	0.002862	SLC6A9	protein_cc
ENSG0000(	4298.591	-0.3968	0.098082	-4.04565	5.22E-05	0.00016	BAIAP3	protein_cc
ENSG0000(	4297.588	0.080489	0.034339	2.343953	0.019081	0.035688	UNC119B	protein_cc
ENSG0000(	4295.698	-0.33906	0.057313	-5.91587	3.30E-09	1.98E-08	SRGAP3	protein_cc
ENSG0000(	4293.647	-0.21077	0.081685	-2.58026	0.009873	0.019751	SEPP1	protein_cc
ENSG0000(	4292.652	-0.25645	0.04058	-6.31969	2.62E-10	1.87E-09	GRIPAP1	protein_cc
ENSG0000(	4292.123	-0.29865	0.040517	-7.37113	1.69E-13	1.98E-12	RB1CC1	protein_cc
ENSG0000(	4291.409	-0.10572	0.02105	-5.02237	5.10E-07	2.16E-06	PPP6C	protein_cc

ENSG0000(	4290.129	-0.30303	0.052304	-5.79353	6.89E-09	3.92E-08	CYTH1	protein_co
ENSG0000(	4283.996	0.103953	0.054814	1.896484	0.057896	0.096059	SH3PXD2E	protein_co
ENSG0000(	4282.209	0.126021	0.035735	3.526553	0.000421	0.001103	FDPS	protein_co
ENSG0000(	4282.153	-0.46255	0.076974	-6.00911	1.87E-09	1.16E-08	KIAA1161	protein_co
ENSG0000(	4280.356	0.046617	0.036484	1.277749	0.201338	0.2809	TNPO3	protein_co
ENSG0000(	4279.108	0.00242	0.057638	0.041988	0.966508	0.976889	CDH11	protein_co
ENSG0000(	4278.931	-0.03812	0.046431	-0.82102	0.411633	0.508501	CDK14	protein_co
ENSG0000(	4277.324	-0.34604	0.032296	-10.7147	8.69E-27	1.09E-24	GORASP1	protein_co
ENSG0000(	4276.268	-0.08527	0.035187	-2.42329	0.015381	0.029443	GGA1	protein_co
ENSG0000(	4272.824	-0.25231	0.031751	-7.94657	1.92E-15	3.16E-14	STX7	protein_co
ENSG0000(	4271.069	-0.08062	0.025129	-3.2081	0.001336	0.003184	DNAJC7	protein_co
ENSG0000(	4270.279	-0.05968	0.038882	-1.53501	0.124782	0.186825	FAM49B	protein_co
ENSG0000(	4269.314	-0.02505	0.039553	-0.63342	0.526461	0.618242	RALBP1	protein_co
ENSG0000(	4269.274	0.081095	0.088538	0.915929	0.359704	0.455531	APLN	protein_co
ENSG0000(	4269.218	-0.13907	0.037232	-3.7351	0.000188	0.000523	EP400	protein_co
ENSG0000(	4268.786	0.201426	0.044052	4.572516	4.82E-06	1.75E-05	PGM1	protein_co
ENSG0000(	4268.694	0.231023	0.043261	5.340266	9.28E-08	4.42E-07	TIA1	protein_co
ENSG0000(	4267.539	0.021791	0.049205	0.442857	0.657869	0.735217	HIGD1A	protein_co
ENSG0000(	4267.315	-0.17333	0.037361	-4.63933	3.50E-06	1.30E-05	ZHX1	protein_co
ENSG0000(	4267.003	-0.05153	0.037433	-1.37661	0.168633	0.241692	PHF1	protein_co
ENSG0000(	4265.903	-0.00519	0.059997	-0.08656	0.931022	0.951711	KLF6	protein_co
ENSG0000(	4265.664	-0.11848	0.033747	-3.51073	0.000447	0.001165	ANP32E	protein_co
ENSG0000(	4263.494	0.091502	0.024666	3.709586	0.000208	0.000574	PRCC	protein_co
ENSG0000(	4262.789	-0.05823	0.030347	-1.91897	0.054989	0.091843	GDE1	protein_co
ENSG0000(	4262.137	-0.10152	0.036399	-2.78919	0.005284	0.011211	ESYT2	protein_co
ENSG0000(	4259.012	-0.11572	0.041386	-2.79606	0.005173	0.010992	FOXRED2	protein_co
ENSG0000(	4258.297	-0.30633	0.058995	-5.19236	2.08E-07	9.35E-07	MCF2L	protein_co
ENSG0000(	4254.83	-0.15805	0.036898	-4.28341	1.84E-05	6.10E-05	ZMYM2	protein_co
ENSG0000(	4254.665	-0.10899	0.02827	-3.85537	0.000116	0.000334	ACTR1B	protein_co
ENSG0000(	4253.414	0.011398	0.027849	0.409271	0.682341	0.755474	DLST	protein_co
ENSG0000(	4253.312	-0.27161	0.035703	-7.60749	2.79E-14	3.73E-13	AMPD2	protein_co
ENSG0000(	4252.921	-0.00086	0.116541	-0.00739	0.994106	0.996141	DCX	protein_co
ENSG0000(	4252.101	0.266357	0.037161	7.167645	7.63E-13	8.08E-12	FASTK	protein_co
ENSG0000(	4249.562	-1.2694	0.121887	-10.4146	2.13E-25	1.99E-23	HPCAL4	protein_co
ENSG0000(	4248.431	-0.06819	0.024457	-2.78801	0.005303	0.011246	SMAD2	protein_co
ENSG0000(	4247.708	0.082313	0.021492	3.829858	0.000128	0.000368	IMMT	protein_co
ENSG0000(	4246.699	-0.17685	0.045971	-3.84697	0.00012	0.000345	NECAP1	protein_co
ENSG0000(	4245.892	-0.43898	0.082957	-5.29163	1.21E-07	5.67E-07	IL17D	protein_co
ENSG0000(	4242.282	-0.12778	0.054908	-2.32714	0.019958	0.037154	MKLN1	protein_co
ENSG0000(	4239.595	-0.05204	0.034708	-1.49942	0.133764	0.198174	LMBRD1	protein_co
ENSG0000(	4239.521	0.05509	0.037197	1.481008	0.138604	0.204381	CNPY3	protein_co
ENSG0000(	4239.23	0.406509	0.094694	4.292853	1.76E-05	5.86E-05	MMP2	protein_co
ENSG0000(	4238.88	0.378156	0.058639	6.448927	1.13E-10	8.52E-10	PLOD3	protein_co
ENSG0000(	4238.63	-0.17291	0.049329	-3.50518	0.000456	0.001186	PIK3C2A	protein_co
ENSG0000(	4238.338	-1.3613	0.111012	-12.2626	1.44E-34	8.10E-32	PRKCB	protein_co
ENSG0000(	4237.893	-0.3172	0.041214	-7.69634	1.40E-14	1.97E-13	PLBD2	protein_co
ENSG0000(	4237.834	-0.24038	0.040879	-5.88027	4.10E-09	2.42E-08	PTPRG	protein_co
ENSG0000(	4237.703	-0.33151	0.053586	-6.18647	6.15E-10	4.14E-09	CNRIP1	protein_co
ENSG0000(	4237.017	-0.49726	0.071974	-6.90888	4.88E-12	4.55E-11	OSBPL11	protein_co
ENSG0000(	4235.086	0.38695	0.07786	4.969827	6.70E-07	2.79E-06	CAPN5	protein_co
ENSG0000(	4235.07	0.018289	0.032327	0.565741	0.57157	0.659093	EMC1	protein_co
ENSG0000(	4234.075	-0.028	0.038247	-0.73197	0.464186	0.560228	PXN	protein_co
ENSG0000(	4233.594	0.110014	0.02746	4.00638	6.17E-05	0.000187	BRD8	protein_co
ENSG0000(	4233.044	0.279864	0.037224	7.518405	5.54E-14	7.05E-13	POFUT1	protein_co
ENSG0000(	4232.982	-0.21675	0.083089	-2.60868	0.009089	0.018329	LHFPL4	protein_co
ENSG0000(	4231.886	-0.54834	0.046825	-11.7104	1.13E-31	3.46E-29	ITSN1	protein_co
ENSG0000(	4231.665	-0.05393	0.025166	-2.14303	0.032111	0.057018	USP19	protein_co
ENSG0000(	4231.472	0.225551	0.042785	5.271744	1.35E-07	6.27E-07	UROD	protein_co
ENSG0000(	4228.962	-0.11069	0.032155	-3.44256	0.000576	0.00147	SETD2	protein_co

ENSG0000(	4228.38	0.08515	0.034791	2.447493	0.014385	0.027753	TMEM43	protein_cc
ENSG0000(	4224.755	-0.17252	0.030765	-5.60764	2.05E-08	1.08E-07	GOLGA3	protein_cc
ENSG0000(	4224.551	-1.15328	0.098107	-11.7553	6.63E-32	2.18E-29	KCNAB2	protein_cc
ENSG0000(	4223.993	-0.09054	0.029249	-3.09562	0.001964	0.004537	TOMM70/	protein_cc
ENSG0000(	4221.575	0.30797	0.098387	3.1302	0.001747	0.004076	CBR1	protein_cc
ENSG0000(	4220.033	0.019505	0.023061	0.845787	0.397672	0.494238	PPP6R3	protein_cc
ENSG0000(	4219.924	-0.48179	0.079133	-6.0884	1.14E-09	7.37E-09	PDE8B	protein_cc
ENSG0000(	4218.42	-0.01583	0.038262	-0.41372	0.679077	0.752993	NAPA	protein_cc
ENSG0000(	4216.47	-0.115	0.069097	-1.66429	0.096055	0.14921	PKIA	protein_cc
ENSG0000(	4215.431	-1.04535	0.109095	-9.58202	9.52E-22	4.38E-20	SH3GL2	protein_cc
ENSG0000(	4213.694	-0.0168	0.030421	-0.55229	0.580752	0.667649	NUP214	protein_cc
ENSG0000(	4212.106	-0.2027	0.038075	-5.32362	1.02E-07	4.82E-07	DVL1	protein_cc
ENSG0000(	4210.228	-0.35414	0.042663	-8.30079	1.03E-16	2.10E-15	TNKS2	protein_cc
ENSG0000(	4208.854	-0.4932	0.052362	-9.41917	4.55E-21	1.86E-19	EFR3B	protein_cc
ENSG0000(	4207.697	0.224156	0.061076	3.670081	0.000242	0.000663	LHFP	protein_cc
ENSG0000(	4203.896	-0.47565	0.1241	-3.83284	0.000127	0.000364	ELFN2	protein_cc
ENSG0000(	4202.122	0.366199	0.044676	8.196789	2.47E-16	4.71E-15	C7orf73	protein_cc
ENSG0000(	4200.864	-0.25913	0.056335	-4.59985	4.23E-06	1.55E-05	MAGEH1	protein_cc
ENSG0000(	4200.539	0.067198	0.028784	2.334526	0.019568	0.036499	MAU2	protein_cc
ENSG0000(	4200.352	-0.60402	0.092634	-6.52047	7.01E-11	5.47E-10	ACSL6	protein_cc
ENSG0000(	4200.02	0.118262	0.028921	4.089184	4.33E-05	0.000135	EMC4	protein_cc
ENSG0000(	4199.056	-0.15646	0.087884	-1.78033	0.075023	0.120366	PDZD2	protein_cc
ENSG0000(	4197.96	-0.15003	0.069601	-2.15558	0.031116	0.055414	ITGB5	protein_cc
ENSG0000(	4197.404	-0.56315	0.104429	-5.39269	6.94E-08	3.37E-07	CX3CR1	protein_cc
ENSG0000(	4193.377	0.239361	0.037052	6.460194	1.05E-10	7.95E-10	SLC25A39	protein_cc
ENSG0000(	4193.078	-0.01954	0.034167	-0.57176	0.567483	0.655465	ACAT1	protein_cc
ENSG0000(	4192.555	-0.01669	0.074892	-0.22291	0.823605	0.870746	GNG4	protein_cc
ENSG0000(	4191.284	-0.01586	0.037562	-0.42211	0.672948	0.747772	MYEF2	protein_cc
ENSG0000(	4189.735	-0.40049	0.043837	-9.13585	6.49E-20	2.19E-18	MAP2K1	protein_cc
ENSG0000(	4189.076	0.177213	0.043636	4.061166	4.88E-05	0.00015	TRIM69	protein_cc
ENSG0000(	4186.667	-0.05862	0.026127	-2.24357	0.02486	0.045304	UBE3A	protein_cc
ENSG0000(	4186.178	-0.10019	0.050462	-1.98543	0.047096	0.080099	YPEL3	protein_cc
ENSG0000(	4185.896	0.166742	0.02858	5.834258	5.40E-09	3.12E-08	VBP1	protein_cc
ENSG0000(	4184.938	0.19217	0.037267	5.156597	2.51E-07	1.12E-06	CHID1	protein_cc
ENSG0000(	4184.249	-0.37654	0.09953	-3.78316	0.000155	0.000438	TMEM132	protein_cc
ENSG0000(	4182.953	0.082822	0.024384	3.396556	0.000682	0.001715	GOLPH3	protein_cc
ENSG0000(	4182.176	-0.31885	0.051028	-6.2486	4.14E-10	2.86E-09	TPM1	protein_cc
ENSG0000(	4177.422	0.136138	0.066247	2.054999	0.039879	0.069207	HMGA1	protein_cc
ENSG0000(	4177.046	-0.11925	0.03307	-3.60611	0.000311	0.000834	NNT	protein_cc
ENSG0000(	4176.12	0.059883	0.031573	1.896645	0.057875	0.096028	C14orf166	protein_cc
ENSG0000(	4175.124	0.009521	0.1012	0.09408	0.925045	0.947132	DLL1	protein_cc
ENSG0000(	4175.092	0.271891	0.042495	6.398121	1.57E-10	1.16E-09	GTF3A	protein_cc
ENSG0000(	4174.894	-0.08973	0.034858	-2.57404	0.010052	0.020062	PPM1B	protein_cc
ENSG0000(	4170.225	0.122235	0.033064	3.696915	0.000218	0.000601	OLA1	protein_cc
ENSG0000(	4169.853	0.121476	0.038218	3.178533	0.00148	0.0035	ABCD3	protein_cc
ENSG0000(	4168.901	0.384569	0.030704	12.52512	5.44E-36	4.48E-33	DDX56	protein_cc
ENSG0000(	4165.578	-0.85779	0.095549	-8.97751	2.77E-19	8.42E-18	STX1B	protein_cc
ENSG0000(	4165.208	-0.11525	0.026498	-4.34939	1.37E-05	4.61E-05	VPS52	protein_cc
ENSG0000(	4164.712	0.215793	0.031352	6.883017	5.86E-12	5.39E-11	URM1	protein_cc
ENSG0000(	4163.825	-0.04192	0.050745	-0.82615	0.40872	0.505525	SIPA1L2	protein_cc
ENSG0000(	4162.203	0.042486	0.026935	1.57733	0.11472	0.173964	SCCPDH	protein_cc
ENSG0000(	4161.53	0.211098	0.043943	4.803937	1.56E-06	6.10E-06	SF3A2	protein_cc
ENSG0000(	4160.772	0.156144	0.037027	4.217008	2.48E-05	8.00E-05	POMP	protein_cc
ENSG0000(	4160.724	-0.09515	0.033225	-2.86369	0.004187	0.009074	PYCR2	protein_cc
ENSG0000(	4159.24	0.304866	0.03384	9.008993	2.08E-19	6.44E-18	DHPS	protein_cc
ENSG0000(	4153.728	0.073938	0.052248	1.415144	0.157026	0.227451	CAST	protein_cc
ENSG0000(	4153.609	-0.624	0.052709	-11.8387	2.46E-32	8.81E-30	OPTN	protein_cc
ENSG0000(	4153.169	0.111051	0.048332	2.297665	0.021581	0.039881	CLK1	protein_cc
ENSG0000(	4151.36	0.196086	0.069157	2.835391	0.004577	0.009822	IFI16	protein_cc

ENSG0000	4151.168	-0.50237	0.072662	-6.91376	4.72E-12	4.41E-11	PLXDC2	protein_co
ENSG0000	4149.861	-0.35782	0.068613	-5.21509	1.84E-07	8.34E-07	ALDH4A1	protein_co
ENSG0000	4149.232	-0.10463	0.035433	-2.95283	0.003149	0.006988	TNRC6A	protein_co
ENSG0000	4148.916	-0.85372	0.097525	-8.75386	2.06E-18	5.48E-17	RAPGEF4	protein_co
ENSG0000	4147.713	-0.06026	0.053854	-1.11899	0.263146	0.351763	WWP2	protein_co
ENSG0000	4145.599	0.124283	0.030827	4.031581	5.54E-05	0.000169	TCEA1	protein_co
ENSG0000	4145.062	-0.11727	0.032804	-3.57473	0.000351	0.000933	PPP6R1	protein_co
ENSG0000	4141.811	0.11284	0.052349	2.155549	0.031119	0.055414	ARHGEF26	protein_co
ENSG0000	4140.358	-0.3637	0.039381	-9.23524	2.58E-20	9.22E-19	CIPC	protein_co
ENSG0000	4139.95	-0.40064	0.077713	-5.15534	2.53E-07	1.13E-06	OMG	protein_co
ENSG0000	4138.066	0.15355	0.031209	4.920004	8.65E-07	3.53E-06	RGL2	protein_co
ENSG0000	4137.123	-0.01791	0.025309	-0.70785	0.479039	0.5744	MARK3	protein_co
ENSG0000	4134.16	0.242961	0.05531	4.392676	1.12E-05	3.84E-05	ZFAS1	antisense
ENSG0000	4128.65	0.112955	0.02946	3.83416	0.000126	0.000362	ADRM1	protein_co
ENSG0000	4126.994	-0.27425	0.046179	-5.93879	2.87E-09	1.74E-08	ERC1	protein_co
ENSG0000	4125.886	-0.10754	0.038497	-2.79341	0.005216	0.011077	ZNFX1	protein_co
ENSG0000	4124.674	0.004385	0.047949	0.091451	0.927134	0.948704	RASA3	protein_co
ENSG0000	4123.02	0.041966	0.030469	1.377326	0.168411	0.241433	MAGEF1	protein_co
ENSG0000	4121.545	-0.02152	0.033777	-0.63708	0.524076	0.616056	PACSIN2	protein_co
ENSG0000	4115.62	0.043449	0.037875	1.147153	0.251318	0.33856	TFDP1	protein_co
ENSG0000	4112.154	0.009428	0.030041	0.313836	0.753645	0.814429	ZRANB2	protein_co
ENSG0000	4111.795	0.204914	0.039639	5.169532	2.35E-07	1.05E-06	MRPL51	protein_co
ENSG0000	4110.974	-0.23879	0.041713	-5.72446	1.04E-08	5.74E-08	GOLGA4	protein_co
ENSG0000	4109.851	0.057032	0.049667	1.148283	0.250852	0.338031	PPP1R18	protein_co
ENSG0000	4109.42	0.041892	0.033676	1.243968	0.213511	0.295072	C12orf49	protein_co
ENSG0000	4109.366	-0.35667	0.06644	-5.36823	7.95E-08	3.83E-07	FAM19A5	protein_co
ENSG0000	4109.359	-0.05546	0.032472	-1.7079	0.087655	0.137837	SLC25A36	protein_co
ENSG0000	4108.095	-0.09153	0.032287	-2.83483	0.004585	0.009838	AASDHPPT	protein_co
ENSG0000	4107.656	-0.31242	0.040984	-7.62304	2.48E-14	3.34E-13	STIM1	protein_co
ENSG0000	4107.039	-0.03197	0.028963	-1.10387	0.26965	0.358998	KLHDC10	protein_co
ENSG0000	4106.543	-0.21154	0.037523	-5.63754	1.72E-08	9.22E-08	ARID1B	protein_co
ENSG0000	4106.183	-0.09059	0.048695	-1.86032	0.06284	0.103153	GAS2L1	protein_co
ENSG0000	4105.699	-0.12454	0.024108	-5.1659	2.39E-07	1.07E-06	VPS11	protein_co
ENSG0000	4105.528	-0.07393	0.032649	-2.26442	0.023548	0.043138	GATAD2B	protein_co
ENSG0000	4104.514	-0.04046	0.084201	-0.48051	0.630867	0.712155	PCSK1N	protein_co
ENSG0000	4102.218	-0.40071	0.049804	-8.04567	8.58E-16	1.49E-14	ZNF609	protein_co
ENSG0000	4101.922	-0.61775	0.062697	-9.85295	6.66E-23	3.84E-21	CNTNAP1	protein_co
ENSG0000	4101.859	-0.38328	0.065717	-5.83229	5.47E-09	3.16E-08	SAMD4A	protein_co
ENSG0000	4101.75	-0.04758	0.052021	-0.91463	0.360386	0.456287	UGP2	protein_co
ENSG0000	4099.323	-0.30493	0.041356	-7.37326	1.67E-13	1.95E-12	MICU1	protein_co
ENSG0000	4096.758	-0.44899	0.05496	-8.16945	3.10E-16	5.80E-15	GCLC	protein_co
ENSG0000	4096.463	0.056248	0.030617	1.837145	0.066188	0.107906	RING1	protein_co
ENSG0000	4096.26	-0.08755	0.036958	-2.36881	0.017846	0.033586	PRPS1	protein_co
ENSG0000	4095.243	-0.24633	0.067655	-3.64091	0.000272	0.000737	FAM107B	protein_co
ENSG0000	4093.658	0.4624	0.045276	10.213	1.73E-24	1.37E-22	TRIM24	protein_co
ENSG0000	4093.453	-0.11268	0.026365	-4.27373	1.92E-05	6.34E-05	KDM2A	protein_co
ENSG0000	4093.021	0.131222	0.026163	5.015574	5.29E-07	2.23E-06	SP3	protein_co
ENSG0000	4092.664	-0.0706	0.056819	-1.24252	0.214043	0.295747	BOP1	protein_co
ENSG0000	4092.25	-0.58619	0.12299	-4.7662	1.88E-06	7.27E-06	PPP1R1B	protein_co
ENSG0000	4091.381	-0.23876	0.032833	-7.27216	3.54E-13	3.94E-12	WBP1L	protein_co
ENSG0000	4089.996	-0.40483	0.040107	-10.0937	5.89E-24	4.23E-22	TEX2	protein_co
ENSG0000	4087.956	0.109164	0.043668	2.499842	0.012425	0.024288	MPC1	protein_co
ENSG0000	4085.403	0.022165	0.042364	0.5232	0.600835	0.685516	MGST3	protein_co
ENSG0000	4082.895	0.237999	0.112382	2.117767	0.034195	0.06034	HMOX1	protein_co
ENSG0000	4082.383	0.032937	0.027471	1.198989	0.230532	0.314514	STX16	protein_co
ENSG0000	4082.294	-0.1948	0.083382	-2.33626	0.019478	0.036356	IFIT3	protein_co
ENSG0000	4081.509	-0.22649	0.045725	-4.95333	7.30E-07	3.01E-06	MTMR10	protein_co
ENSG0000	4079.02	-0.20842	0.074991	-2.77922	0.005449	0.011522	PLA2G16	protein_co
ENSG0000	4074.512	-0.1188	0.042892	-2.76981	0.005609	0.011825	CLTB	protein_co

ENSG0000(	4074.103	0.086598	0.059651	1.451752	0.146571	0.214451	LAMA5	protein_cc
ENSG0000(	4074.102	-0.19275	0.030053	-6.41371	1.42E-10	1.06E-09	WDR48	protein_cc
ENSG0000(	4072.584	-0.7617	0.108924	-6.99293	2.69E-12	2.62E-11	CAMK2B	protein_cc
ENSG0000(	4072.395	-0.1078	0.035189	-3.06341	0.002188	0.005011	SRGAP2	protein_cc
ENSG0000(	4071.748	-0.41006	0.045154	-9.0813	1.07E-19	3.49E-18	TRAK2	protein_cc
ENSG0000(	4071.054	0.048284	0.026263	1.838508	0.065988	0.107609	FZR1	protein_cc
ENSG0000(	4070.177	-0.35855	0.041538	-8.63181	6.04E-18	1.50E-16	TRIM37	protein_cc
ENSG0000(	4067.076	-0.3988	0.084844	-4.70041	2.60E-06	9.82E-06	CEND1	protein_cc
ENSG0000(	4066.025	0.205589	0.033427	6.150401	7.73E-10	5.13E-09	SF3A3	protein_cc
ENSG0000(	4061.306	0.431105	0.043341	9.946707	2.61E-23	1.65E-21	LSM4	protein_cc
ENSG0000(	4059.388	-0.43598	0.099711	-4.37242	1.23E-05	4.18E-05	CDK18	protein_cc
ENSG0000(	4058.399	-0.12941	0.029457	-4.39335	1.12E-05	3.83E-05	KDSR	protein_cc
ENSG0000(	4056.696	0.393095	0.086835	4.526943	5.98E-06	2.14E-05	NID1	protein_cc
ENSG0000(	4055.683	-0.3817	0.063468	-6.0141	1.81E-09	1.13E-08	KIF3A	protein_cc
ENSG0000(	4054.124	-0.02983	0.035555	-0.83901	0.401466	0.498246	PRPF38A	protein_cc
ENSG0000(	4053.48	0.037287	0.038089	0.978934	0.327613	0.421933	LMAN1	protein_cc
ENSG0000(	4053.116	-0.25009	0.042267	-5.91699	3.28E-09	1.97E-08	HSDL2	protein_cc
ENSG0000(	4053.002	-0.12764	0.027797	-4.59175	4.40E-06	1.61E-05	UBE2K	protein_cc
ENSG0000(	4051.499	0.254573	0.048521	5.246705	1.55E-07	7.11E-07	ATP5D	protein_cc
ENSG0000(	4050.88	-0.57068	0.064491	-8.84906	8.83E-19	2.47E-17	OSGIN2	protein_cc
ENSG0000(	4048.109	0.172814	0.060512	2.855885	0.004292	0.009273	PLIN3	protein_cc
ENSG0000(	4047.939	-0.1196	0.035805	-3.34023	0.000837	0.00207	GSK3A	protein_cc
ENSG0000(	4047.91	0.19918	0.041337	4.81849	1.45E-06	5.71E-06	DCTN3	protein_cc
ENSG0000(	4047.627	0.065467	0.070721	0.925711	0.354596	0.4502	ENG	protein_cc
ENSG0000(	4047.234	-0.13019	0.038764	-3.35844	0.000784	0.001949	TBC1D17	protein_cc
ENSG0000(	4046.197	-0.25001	0.0408	-6.12752	8.93E-10	5.87E-09	DOCK4	protein_cc
ENSG0000(	4045.012	0.259399	0.11938	2.172892	0.029788	0.05331	RGS1	protein_cc
ENSG0000(	4042.702	-0.24001	0.047395	-5.06397	4.11E-07	1.76E-06	EVI5L	protein_cc
ENSG0000(	4038.899	-0.13202	0.036758	-3.5915	0.000329	0.000879	PHF21A	protein_cc
ENSG0000(	4038.484	0.112489	0.049963	2.251432	0.024358	0.044487	TMED5	protein_cc
ENSG0000(	4038.467	0.573415	0.073913	7.75796	8.63E-15	1.28E-13	SPRY2	protein_cc
ENSG0000(	4038.23	0.058568	0.029635	1.976323	0.048118	0.081624	OXA1L	protein_cc
ENSG0000(	4038.137	0.019433	0.038644	0.502872	0.615055	0.698233	CYHR1	protein_cc
ENSG0000(	4037.937	0.150935	0.050366	2.996729	0.002729	0.006129	FBL	protein_cc
ENSG0000(	4037.573	-0.05575	0.054736	-1.01845	0.308463	0.401563	ITGA6	protein_cc
ENSG0000(	4033.837	-0.72782	0.116049	-6.27169	3.57E-10	2.49E-09	ENPP2	protein_cc
ENSG0000(	4033.251	-0.15861	0.039409	-4.02481	5.70E-05	0.000174	ZZEF1	protein_cc
ENSG0000(	4031.228	-0.07525	0.024849	-3.02819	0.00246	0.005584	OSBP	protein_cc
ENSG0000(	4030.994	-0.09878	0.025237	-3.91407	9.08E-05	0.000267	TOX4	protein_cc
ENSG0000(	4030.517	-0.69225	0.096105	-7.20308	5.89E-13	6.34E-12	AATK	protein_cc
ENSG0000(	4028.534	-0.75745	0.143181	-5.29018	1.22E-07	5.71E-07	CARNS1	protein_cc
ENSG0000(	4028.416	-0.1033	0.033082	-3.12253	0.001793	0.004176	TIAL1	protein_cc
ENSG0000(	4027.413	-0.12707	0.026369	-4.81869	1.45E-06	5.70E-06	EIF3J	protein_cc
ENSG0000(	4025.813	-1.23983	0.132154	-9.38176	6.49E-21	2.57E-19	SYN2	processed
ENSG0000(	4024.776	-0.02685	0.020358	-1.31879	0.187239	0.263979	ELAC2	protein_cc
ENSG0000(	4020.915	-0.11036	0.028806	-3.8312	0.000128	0.000366	RAB21	protein_cc
ENSG0000(	4019.77	-0.23804	0.037282	-6.3848	1.72E-10	1.26E-09	UHMK1	protein_cc
ENSG0000(	4019.319	0.099624	0.033556	2.968866	0.002989	0.006663	MED16	protein_cc
ENSG0000(	4018.926	-0.63541	0.057652	-11.0214	3.01E-28	4.82E-26	PPP3CB	protein_cc
ENSG0000(	4018.81	-0.45463	0.040991	-11.0908	1.39E-28	2.46E-26	ATXN7L3	protein_cc
ENSG0000(	4018.709	-0.52801	0.049489	-10.6692	1.42E-26	1.71E-24	SLC9A6	protein_cc
ENSG0000(	4018.206	-0.03866	0.029101	-1.32833	0.184068	0.260106	PPME1	protein_cc
ENSG0000(	4017.773	0.059352	0.037424	1.585931	0.112755	0.171358	B4GALT2	protein_cc
ENSG0000(	4017.459	-0.84191	0.107753	-7.81339	5.57E-15	8.49E-14	NMNAT2	protein_cc
ENSG0000(	4017.397	0.080251	0.039396	2.037014	0.041649	0.071919	AKR1A1	protein_cc
ENSG0000(	4016.372	0.070803	0.03631	1.949971	0.05118	0.086262	XAB2	protein_cc
ENSG0000(	4014.699	0.107502	0.02749	3.91056	9.21E-05	0.000271	SS18	protein_cc
ENSG0000(	4013.712	-0.09636	0.048538	-1.98516	0.047126	0.080137	PDPR	protein_cc
ENSG0000(	4013.606	-0.16376	0.034285	-4.77631	1.79E-06	6.94E-06	GPATCH8	protein_cc

ENSG0000(	4012.495	0.163171	0.052571	3.103828	0.00191	0.004422	NECAP2	protein_cc
ENSG0000(	4012.26	-0.62218	0.084147	-7.39393	1.43E-13	1.69E-12	DNM3	protein_cc
ENSG0000(	4012.106	0.389878	0.055993	6.962937	3.33E-12	3.20E-11	AK2	protein_cc
ENSG0000(	4011.794	0.09147	0.028793	3.176807	0.001489	0.00352	ATG4B	protein_cc
ENSG0000(	4010.579	-0.17019	0.035365	-4.81239	1.49E-06	5.87E-06	TUBGCP2	protein_cc
ENSG0000(	4010.397	-0.04336	0.063622	-0.68155	0.495522	0.589722	ANO6	protein_cc
ENSG0000(	4008.132	-0.12675	0.050283	-2.52077	0.01171	0.023032	HEG1	protein_cc
ENSG0000(	4007.989	-0.26648	0.04691	-5.68073	1.34E-08	7.29E-08	ARL3	protein_cc
ENSG0000(	4007.302	0.116612	0.037641	3.097996	0.001948	0.004503	NUP188	protein_cc
ENSG0000(	4006.657	-0.83368	0.081804	-10.1912	2.17E-24	1.69E-22	PLCB1	protein_cc
ENSG0000(	4006.535	0.115631	0.027372	4.224475	2.39E-05	7.76E-05	CUL1	protein_cc
ENSG0000(	4006.321	-0.15512	0.050603	-3.0654	0.002174	0.00498	CPD	protein_cc
ENSG0000(	4006.041	0.22833	0.053945	4.23265	2.31E-05	7.50E-05	CAPZA1	protein_cc
ENSG0000(	4005.605	-0.06818	0.033688	-2.02375	0.042996	0.073945	PJA1	protein_cc
ENSG0000(	4004.315	0.021039	0.02759	0.762553	0.44573	0.54249	UFM1	protein_cc
ENSG0000(	4003.213	0.170023	0.028781	5.90742	3.48E-09	2.08E-08	MANBAL	protein_cc
ENSG0000(	4003.142	-0.06088	0.031868	-1.9104	0.056081	0.093394	CACYBP	protein_cc
ENSG0000(	4002.022	-0.44145	0.052935	-8.33937	7.47E-17	1.55E-15	PPP3R1	protein_cc
ENSG0000(	4000.575	-0.34417	0.096811	-3.55501	0.000378	0.000999	ANLN	protein_cc
ENSG0000(	3999.917	-0.4505	0.069731	-6.46055	1.04E-10	7.93E-10	8-Mar	protein_cc
ENSG0000(	3998.495	0.027662	0.024548	1.12684	0.25981	0.34797	KIAA0141	protein_cc
ENSG0000(	3997.029	-0.02826	0.019766	-1.42985	0.152761	0.222281	QRICH1	protein_cc
ENSG0000(	3996.76	0.053767	0.026682	2.015104	0.043894	0.075303	SMAD4	protein_cc
ENSG0000(	3996.488	0.140183	0.036576	3.832606	0.000127	0.000364	CCDC124	protein_cc
ENSG0000(	3996.318	0.056294	0.030076	1.87171	0.061247	0.100821	CORO1B	protein_cc
ENSG0000(	3996.213	-0.02266	0.030415	-0.74511	0.456208	0.552707	BLOC1S6	protein_cc
ENSG0000(	3995.676	-0.25582	0.045488	-5.624	1.87E-08	9.93E-08	BTBD3	protein_cc
ENSG0000(	3995.318	-0.11385	0.042248	-2.69477	0.007044	0.014544	PPP1R12C	protein_cc
ENSG0000(	3994.969	0.257091	0.032188	7.987225	1.38E-15	2.33E-14	TXNDC15	protein_cc
ENSG0000(	3994.948	0.14315	0.038562	3.712203	0.000205	0.000568	PPP5C	protein_cc
ENSG0000(	3994.732	0.222367	0.041123	5.407429	6.39E-08	3.13E-07	NDUFS8	protein_cc
ENSG0000(	3991.96	-0.03762	0.059061	-0.63695	0.524157	0.616099	EFHD2	protein_cc
ENSG0000(	3991.856	0.049563	0.084346	0.587614	0.556791	0.646073	CNTFR	protein_cc
ENSG0000(	3991.83	0.208602	0.051737	4.031943	5.53E-05	0.000169	LUC7L	protein_cc
ENSG0000(	3990.49	-0.14872	0.027028	-5.50239	3.75E-08	1.90E-07	CHMP3	protein_cc
ENSG0000(	3990.021	-0.27662	0.040399	-6.84722	7.53E-12	6.83E-11	PHF3	protein_cc
ENSG0000(	3989.872	-0.12808	0.025549	-5.01306	5.36E-07	2.26E-06	DEF8	protein_cc
ENSG0000(	3988.979	-0.04841	0.027315	-1.77232	0.076341	0.122219	RSRC2	protein_cc
ENSG0000(	3988.409	-0.37066	0.05183	-7.15149	8.58E-13	9.03E-12	CRTC1	protein_cc
ENSG0000(	3987.182	-0.08419	0.086273	-0.97586	0.329132	0.423558	FIBIN	protein_cc
ENSG0000(	3986.224	-0.56667	0.072146	-7.85449	4.01E-15	6.27E-14	WASF1	protein_cc
ENSG0000(	3985.35	-0.09567	0.024951	-3.83439	0.000126	0.000362	XIAP	protein_cc
ENSG0000(	3984.385	-0.11838	0.04299	-2.75358	0.005895	0.012371	GLTP	protein_cc
ENSG0000(	3983.885	-0.71358	0.083057	-8.59146	8.59E-18	2.08E-16	CADPS	protein_cc
ENSG0000(	3983.408	-0.05589	0.025795	-2.16693	0.03024	0.054025	ORMDL3	protein_cc
ENSG0000(	3981.828	-0.53415	0.054819	-9.74388	1.96E-22	1.03E-20	PURA	protein_cc
ENSG0000(	3979.766	0.330674	0.030703	10.77003	4.77E-27	6.23E-25	WBSCR22	protein_cc
ENSG0000(	3979.134	-0.10478	0.039119	-2.67841	0.007397	0.015215	USP24	protein_cc
ENSG0000(	3979.053	0.739471	0.084075	8.795408	1.43E-18	3.88E-17	CRISPLD1	protein_cc
ENSG0000(	3978.127	-0.09081	0.110851	-0.81924	0.412647	0.509555	LHFPL3	protein_cc
ENSG0000(	3976.619	-0.22419	0.035647	-6.2892	3.19E-10	2.25E-09	ATP6V1D	protein_cc
ENSG0000(	3975.626	0.021984	0.029653	0.741364	0.458473	0.554863	SAR1B	protein_cc
ENSG0000(	3974.863	-0.22482	0.042296	-5.31535	1.06E-07	5.03E-07	CAMSAP1	protein_cc
ENSG0000(	3971.128	0.087748	0.025773	3.404599	0.000663	0.00167	SCAMP3	protein_cc
ENSG0000(	3968.576	0.548182	0.100065	5.478241	4.30E-08	2.16E-07	GNG12	protein_cc
ENSG0000(	3967.568	0.109517	0.031113	3.519952	0.000432	0.001128	AC242988	protein_cc
ENSG0000(	3967.092	-0.06442	0.069727	-0.92384	0.355568	0.451182	MVP	protein_cc
ENSG0000(	3966.41	0.12614	0.043334	2.910854	0.003604	0.007908	ATP5I	protein_cc
ENSG0000(	3965.181	0.097694	0.044615	2.1897	0.028546	0.051297	RB1	protein_cc

ENSG0000(	3964.405	-0.08511	0.027275	-3.12052	0.001805	0.004201	LRRC47	protein_cc
ENSG0000(	3964.228	-0.35337	0.065062	-5.43127	5.60E-08	2.76E-07	OSBPL1A	protein_cc
ENSG0000(	3963.649	-0.61353	0.059031	-10.3933	2.66E-25	2.45E-23	MAP3K5	protein_cc
ENSG0000(	3963.032	0.15041	0.057408	2.620024	0.008792	0.017787	ST3GAL5	protein_cc
ENSG0000(	3962.77	0.047773	0.032085	1.488963	0.136497	0.201664	SMAD5	protein_cc
ENSG0000(	3962.489	0.03375	0.047764	0.706603	0.479813	0.575189	CEP170	protein_cc
ENSG0000(	3961.903	-0.32713	0.054492	-6.00331	1.93E-09	1.20E-08	FAXDC2	protein_cc
ENSG0000(	3960.705	-0.1675	0.058927	-2.84252	0.004476	0.009631	CHST3	protein_cc
ENSG0000(	3957.694	-0.26082	0.045215	-5.76842	8.00E-09	4.51E-08	TP53BP1	protein_cc
ENSG0000(	3957.44	-0.34679	0.040493	-8.5641	1.09E-17	2.59E-16	CLCN6	protein_cc
ENSG0000(	3957.399	-0.025	0.026334	-0.94937	0.342431	0.437659	CMTR1	protein_cc
ENSG0000(	3955.921	0.193399	0.040794	4.740851	2.13E-06	8.17E-06	ATP5O	protein_cc
ENSG0000(	3955.235	-0.6741	0.065697	-10.2608	1.06E-24	8.72E-23	BAG3	protein_cc
ENSG0000(	3953.204	-0.15624	0.034578	-4.51855	6.23E-06	2.22E-05	FXR2	protein_cc
ENSG0000(	3953.173	-0.11091	0.039964	-2.77519	0.005517	0.011651	RBL2	protein_cc
ENSG0000(	3952.326	-0.78619	0.079363	-9.9062	3.91E-23	2.35E-21	EPB41L3	protein_cc
ENSG0000(	3951.9	0.157201	0.0391	4.020457	5.81E-05	0.000177	XPR1	protein_cc
ENSG0000(	3950.82	0.027403	0.069216	0.39591	0.692172	0.764055	MICAL1	protein_cc
ENSG0000(	3950.803	0.093684	0.031828	2.943428	0.003246	0.007185	SKA2	protein_cc
ENSG0000(	3950.251	0.183553	0.028091	6.534273	6.39E-11	5.02E-10	EIF4A3	protein_cc
ENSG0000(	3948.556	0.296855	0.036414	8.152202	3.57E-16	6.64E-15	XRN2	protein_cc
ENSG0000(	3948.309	0.046703	0.039742	1.175174	0.239925	0.325257	SELK	protein_cc
ENSG0000(	3946.503	-0.07527	0.030151	-2.49647	0.012544	0.024492	SCAF11	protein_cc
ENSG0000(	3944.944	-0.00076	0.037246	-0.02045	0.983685	0.988806	FAM50A	protein_cc
ENSG0000(	3943.946	0.023	0.040316	0.570479	0.568353	0.656304	ZNF146	protein_cc
ENSG0000(	3941.205	0.358803	0.079259	4.526973	5.98E-06	2.14E-05	COL16A1	protein_cc
ENSG0000(	3940.996	0.069757	0.035916	1.942204	0.052112	0.08764	TPGS2	protein_cc
ENSG0000(	3940.132	-0.05692	0.060641	-0.9386	0.347934	0.443493	DOCK7	protein_cc
ENSG0000(	3938.321	0.333248	0.052271	6.375406	1.82E-10	1.33E-09	MDM2	protein_cc
ENSG0000(	3938.248	0.045785	0.032701	1.400092	0.161486	0.232882	WIZ	protein_cc
ENSG0000(	3937.848	0.266785	0.054168	4.925091	8.43E-07	3.45E-06	CETN2	protein_cc
ENSG0000(	3936.985	-0.13408	0.037352	-3.58959	0.000331	0.000885	ITFG1	protein_cc
ENSG0000(	3935.88	-0.0542	0.024333	-2.22748	0.025915	0.047044	FBRS	protein_cc
ENSG0000(	3935.075	-0.17754	0.062381	-2.84605	0.004427	0.009535	GSE1	protein_cc
ENSG0000(	3934.728	-0.02162	0.024075	-0.89798	0.369199	0.46552	HARS	protein_cc
ENSG0000(	3932.925	-0.30265	0.035598	-8.50198	1.86E-17	4.27E-16	ZNF592	protein_cc
ENSG0000(	3932.157	0.226286	0.058009	3.900899	9.58E-05	0.000281	GLIPR2	protein_cc
ENSG0000(	3931.545	0.124381	0.033616	3.700051	0.000216	0.000594	PTDSS1	protein_cc
ENSG0000(	3930.01	-0.28387	0.037232	-7.6243	2.45E-14	3.31E-13	RGL1	protein_cc
ENSG0000(	3929.205	-0.43113	0.085052	-5.06908	4.00E-07	1.72E-06	SYTL4	protein_cc
ENSG0000(	3928.281	0.020284	0.028848	0.703121	0.48198	0.577162	UBFD1	protein_cc
ENSG0000(	3926.394	0.240156	0.03239	7.41452	1.22E-13	1.46E-12	UBE2A	protein_cc
ENSG0000(	3925.385	-0.2222	0.039912	-5.56717	2.59E-08	1.35E-07	CLIP1	protein_cc
ENSG0000(	3925.137	-0.10368	0.033121	-3.13038	0.001746	0.004074	RBBP6	protein_cc
ENSG0000(	3924.291	0.011951	0.040023	0.298611	0.765237	0.823803	SRPR	protein_cc
ENSG0000(	3924.024	0.099007	0.03152	3.141064	0.001683	0.003939	CYTH2	protein_cc
ENSG0000(	3920.814	0.009285	0.037655	0.246566	0.805244	0.8565	ZNF853	protein_cc
ENSG0000(	3920.4	0.062044	0.034284	1.809694	0.070343	0.113791	C11orf49	protein_cc
ENSG0000(	3920.316	-0.08078	0.024517	-3.29495	0.000984	0.0024	ZNF706	protein_cc
ENSG0000(	3919.907	-0.23902	0.029173	-8.1932	2.54E-16	4.84E-15	RAB3GAP:	protein_cc
ENSG0000(	3919.734	-0.06235	0.061818	-1.00865	0.313144	0.406559	PDGFRB	protein_cc
ENSG0000(	3919.54	-0.06144	0.049624	-1.2381	0.215678	0.297666	TRO	protein_cc
ENSG0000(	3918.691	-0.36611	0.044803	-8.17166	3.04E-16	5.71E-15	PCGF5	protein_cc
ENSG0000(	3917.469	0.396136	0.047019	8.424986	3.61E-17	7.90E-16	UQCR11	protein_cc
ENSG0000(	3916.569	-0.64708	0.076099	-8.50308	1.85E-17	4.23E-16	SEMA4D	protein_cc
ENSG0000(	3915.413	0.381617	0.084248	4.529692	5.91E-06	2.12E-05	ADORA1	protein_cc
ENSG0000(	3913.601	0.777121	0.060437	12.85835	7.72E-38	8.14E-35	PCNA	protein_cc
ENSG0000(	3912.562	-0.00589	0.037357	-0.15769	0.874703	0.910136	RAP1A	protein_cc
ENSG0000(	3912.164	-0.01643	0.032972	-0.49835	0.618237	0.701053	HSF1	protein_cc

ENSG0000(	3912.047	-0.2378	0.127042	-1.87179	0.061236	0.100811	CYR61	protein_cc
ENSG0000(	3911.69	-0.25748	0.042776	-6.01931	1.75E-09	1.10E-08	DUSP3	protein_cc
ENSG0000(	3910.526	-0.23444	0.042715	-5.48844	4.06E-08	2.05E-07	CBL	protein_cc
ENSG0000(	3909.845	-0.09423	0.044024	-2.14034	0.032327	0.057362	AHDC1	protein_cc
ENSG0000(	3909.436	-0.04942	0.048118	-1.02709	0.304376	0.397244	HS6ST1	protein_cc
ENSG0000(	3909.373	-0.03789	0.033386	-1.13482	0.256449	0.344254	INTS3	protein_cc
ENSG0000(	3908.586	0.186883	0.034061	5.486694	4.10E-08	2.07E-07	HAX1	protein_cc
ENSG0000(	3908.366	-0.60939	0.05162	-11.8053	3.66E-32	1.26E-29	USP46	protein_cc
ENSG0000(	3907.724	-0.25378	0.048716	-5.20926	1.90E-07	8.59E-07	SBF2	protein_cc
ENSG0000(	3906.918	-0.23527	0.039593	-5.94219	2.81E-09	1.71E-08	PITHD1	protein_cc
ENSG0000(	3905.848	0.35891	0.036829	9.74528	1.93E-22	1.02E-20	TBCA	protein_cc
ENSG0000(	3905.23	0.085474	0.083576	1.022702	0.306449	0.399467	FAM198B	protein_cc
ENSG0000(	3905.212	0.06749	0.030159	2.237818	0.025233	0.045919	SPG21	protein_cc
ENSG0000(	3904.696	0.247712	0.03852	6.430782	1.27E-10	9.54E-10	FAM96B	protein_cc
ENSG0000(	3904.048	-0.53984	0.052285	-10.325	5.43E-25	4.75E-23	LPIN1	protein_cc
ENSG0000(	3901.315	0.114158	0.026127	4.369416	1.25E-05	4.23E-05	SUMO1	protein_cc
ENSG0000(	3900.347	0.618436	0.060725	10.18425	2.33E-24	1.81E-22	HMGB2	protein_cc
ENSG0000(	3898.023	-0.13241	0.025233	-5.24756	1.54E-07	7.08E-07	DYRK1A	protein_cc
ENSG0000(	3897.911	-0.14282	0.029006	-4.92372	8.49E-07	3.47E-06	BCL2L13	protein_cc
ENSG0000(	3897.757	-0.48877	0.143313	-3.41051	0.000648	0.001638	INA	protein_cc
ENSG0000(	3896.614	-0.35731	0.122844	-2.90862	0.00363	0.007958	CSDC2	protein_cc
ENSG0000(	3896.437	0.503676	0.111984	4.497731	6.87E-06	2.43E-05	SERPINH1	protein_cc
ENSG0000(	3894.974	-0.23574	0.029591	-7.96681	1.63E-15	2.72E-14	DENND4B	protein_cc
ENSG0000(	3894.392	-0.95167	0.103324	-9.21052	3.25E-20	1.14E-18	BSN	protein_cc
ENSG0000(	3892.78	-0.40394	0.047347	-8.53146	1.45E-17	3.37E-16	CMIP	protein_cc
ENSG0000(	3892.14	-0.07542	0.034482	-2.18731	0.02872	0.051596	SRRM1	protein_cc
ENSG0000(	3891.628	0.519517	0.070007	7.420915	1.16E-13	1.40E-12	RPL39	protein_cc
ENSG0000(	3889.994	0.001101	0.036905	0.029825	0.976207	0.98326	NOC2L	protein_cc
ENSG0000(	3889.727	-0.06576	0.051842	-1.26852	0.204611	0.284723	MBOAT7	protein_cc
ENSG0000(	3889.565	-0.08891	0.027685	-3.2113	0.001321	0.003151	TTC37	protein_cc
ENSG0000(	3888.869	-0.05031	0.029395	-1.71163	0.086964	0.136892	RBM12	protein_cc
ENSG0000(	3887.247	-0.24845	0.039104	-6.35348	2.11E-10	1.52E-09	ZSWIM8	protein_cc
ENSG0000(	3886.394	1.494629	0.10332	14.46599	1.99E-47	5.84E-44	KLHDC8A	protein_cc
ENSG0000(	3886.354	0.268455	0.034003	7.894957	2.90E-15	4.65E-14	EIF6	protein_cc
ENSG0000(	3886.223	0.421226	0.072153	5.837991	5.28E-09	3.06E-08	CD276	protein_cc
ENSG0000(	3886.103	-0.03276	0.025621	-1.27864	0.201025	0.280512	IARS2	protein_cc
ENSG0000(	3884.957	0.017916	0.031577	0.567386	0.570452	0.658247	ZMYM4	protein_cc
ENSG0000(	3884.82	-0.93341	0.099329	-9.39711	5.61E-21	2.25E-19	KCNB1	protein_cc
ENSG0000(	3884.085	0.148852	0.029814	4.992729	5.95E-07	2.49E-06	CLPTM1L	protein_cc
ENSG0000(	3883.189	-0.53458	0.05624	-9.50532	1.99E-21	8.79E-20	UTRN	protein_cc
ENSG0000(	3882.614	0.280396	0.035853	7.820699	5.25E-15	8.05E-14	POLE3	protein_cc
ENSG0000(	3882.329	-0.52758	0.047413	-11.1275	9.22E-29	1.69E-26	C20orf194	protein_cc
ENSG0000(	3882.058	-0.02974	0.021532	-1.38109	0.167251	0.239984	UBE2Q1	protein_cc
ENSG0000(	3881.965	0.074652	0.028082	2.65836	0.007852	0.016067	DDX18	protein_cc
ENSG0000(	3878.232	0.286761	0.137614	2.083809	0.037178	0.065034	GPNMB	protein_cc
ENSG0000(	3877.989	0.274144	0.034157	8.026045	1.01E-15	1.73E-14	FIBP	protein_cc
ENSG0000(	3875.516	0.673137	0.097897	6.876007	6.16E-12	5.65E-11	FKBP9	protein_cc
ENSG0000(	3874.276	-0.07933	0.043402	-1.82784	0.067574	0.10989	LRP3	protein_cc
ENSG0000(	3873.156	0.18827	0.025025	7.523194	5.35E-14	6.82E-13	KXD1	protein_cc
ENSG0000(	3872.903	0.010958	0.039511	0.277336	0.781522	0.837114	CDK9	protein_cc
ENSG0000(	3872.639	-0.30105	0.101422	-2.9683	0.002994	0.006675	KCNIP3	protein_cc
ENSG0000(	3871.633	-0.15594	0.037271	-4.18396	2.86E-05	9.17E-05	SEPHS1	protein_cc
ENSG0000(	3869.251	-0.07419	0.073901	-1.00394	0.315409	0.408969	LY6E	protein_cc
ENSG0000(	3868.921	0.463791	0.04428	10.47406	1.14E-25	1.15E-23	PPP4C	protein_cc
ENSG0000(	3868.099	-0.10589	0.027593	-3.8375	0.000124	0.000357	APPBP2	protein_cc
ENSG0000(	3867.302	0.601209	0.115784	5.192515	2.07E-07	9.34E-07	MX1	protein_cc
ENSG0000(	3867.094	-0.14204	0.050083	-2.83606	0.004567	0.009805	SMC3	protein_cc
ENSG0000(	3866.25	-0.04581	0.024249	-1.88916	0.05887	0.097444	NUP133	protein_cc
ENSG0000(	3865.035	-0.57035	0.069302	-8.23003	1.87E-16	3.63E-15	PVRL1	protein_cc

ENSG0000(	3864.92	0.17723	0.055944	3.167984	0.001535	0.003619	DMPK	protein_cc
ENSG0000(	3864.846	0.236014	0.037982	6.213856	5.17E-10	3.52E-09	PTPN12	protein_cc
ENSG0000(	3863.754	0.104243	0.032275	3.229863	0.001238	0.002969	MLST8	protein_cc
ENSG0000(	3863.344	-0.21485	0.035278	-6.09012	1.13E-09	7.30E-09	SIK2	protein_cc
ENSG0000(	3862.249	0.38403	0.039009	9.844739	7.22E-23	4.13E-21	STARD3NI	protein_cc
ENSG0000(	3861.193	0.519583	0.055477	9.365796	7.55E-21	2.96E-19	MCM4	protein_cc
ENSG0000(	3860.538	0.66272	0.07749	8.552341	1.21E-17	2.85E-16	JAG1	protein_cc
ENSG0000(	3860.056	-0.33598	0.050549	-6.64662	3.00E-11	2.47E-10	SYNGAP1	protein_cc
ENSG0000(	3858.594	0.009373	0.026858	0.348983	0.727102	0.792509	DIDO1	protein_cc
ENSG0000(	3858.102	-0.0526	0.032611	-1.61309	0.106725	0.163401	MOCS2	protein_cc
ENSG0000(	3857.523	-0.85803	0.089633	-9.57267	1.04E-21	4.77E-20	RAB11FIP4	protein_cc
ENSG0000(	3857.201	-0.071	0.052118	-1.36234	0.173091	0.247022	CTSO	protein_cc
ENSG0000(	3855.928	0.216277	0.046473	4.65381	3.26E-06	1.21E-05	SRM	protein_cc
ENSG0000(	3854.512	-0.00051	0.043388	-0.01171	0.990658	0.99363	RIMKLB	protein_cc
ENSG0000(	3852.598	0.321509	0.072845	4.413623	1.02E-05	3.51E-05	F2R	protein_cc
ENSG0000(	3852.024	0.25043	0.050656	4.943714	7.66E-07	3.15E-06	ERF	protein_cc
ENSG0000(	3850.034	-0.4136	0.083467	-4.95529	7.22E-07	2.98E-06	CDH20	protein_cc
ENSG0000(	3849.395	0.324069	0.043397	7.467615	8.17E-14	1.01E-12	C19orf53	protein_cc
ENSG0000(	3848.321	-0.02944	0.024795	-1.18719	0.235153	0.319863	RXRB	protein_cc
ENSG0000(	3847.469	-0.10866	0.043455	-2.50062	0.012398	0.024239	ZSCAN18	protein_cc
ENSG0000(	3845.474	-0.08206	0.023399	-3.50683	0.000453	0.00118	SYVN1	protein_cc
ENSG0000(	3845.442	-0.07702	0.030023	-2.56537	0.010307	0.020524	SENP6	protein_cc
ENSG0000(	3844.755	-0.29341	0.072794	-4.0307	5.56E-05	0.00017	P2RX7	protein_cc
ENSG0000(	3844.416	-0.29811	0.051535	-5.78459	7.27E-09	4.12E-08	PIK3IP1	protein_cc
ENSG0000(	3840.981	-0.15558	0.051658	-3.01162	0.002599	0.00587	GNB4	protein_cc
ENSG0000(	3838.828	0.283343	0.083991	3.373496	0.000742	0.001852	SLC35F1	protein_cc
ENSG0000(	3837.214	-1.69678	0.165438	-10.2563	1.11E-24	9.07E-23	PACSIN1	protein_cc
ENSG0000(	3836.844	-0.40444	0.052754	-7.66654	1.77E-14	2.44E-13	MAPRE3	protein_cc
ENSG0000(	3836.723	-0.11527	0.053146	-2.16885	0.030094	0.053794	ZFYVE21	protein_cc
ENSG0000(	3836.295	-0.09861	0.037629	-2.62067	0.008776	0.017759	NSD1	protein_cc
ENSG0000(	3835.195	-0.07366	0.034339	-2.14498	0.031954	0.056762	DROSHA	protein_cc
ENSG0000(	3834.066	-0.34553	0.040896	-8.44901	2.94E-17	6.54E-16	DPP8	protein_cc
ENSG0000(	3833.752	-0.20424	0.033196	-6.15249	7.63E-10	5.07E-09	APPL1	protein_cc
ENSG0000(	3832.799	0.106633	0.038461	2.77251	0.005563	0.011736	PEF1	protein_cc
ENSG0000(	3832.779	-0.13212	0.030915	-4.27377	1.92E-05	6.34E-05	SMIM14	protein_cc
ENSG0000(	3831.493	0.220233	0.034667	6.352796	2.11E-10	1.53E-09	PSMA4	protein_cc
ENSG0000(	3831.054	0.056825	0.060404	0.940755	0.34683	0.442293	CXCL16	protein_cc
ENSG0000(	3830.833	0.117625	0.066371	1.772229	0.076356	0.122235	ETS1	protein_cc
ENSG0000(	3830.616	-0.00681	0.024418	-0.27886	0.780351	0.836077	ELP2	protein_cc
ENSG0000(	3829.034	-0.03562	0.032618	-1.09214	0.274772	0.364837	USP33	protein_cc
ENSG0000(	3828.001	-0.19185	0.047654	-4.02588	5.68E-05	0.000173	SLC39A10	protein_cc
ENSG0000(	3827.954	0.162636	0.046324	3.51083	0.000447	0.001165	TXLNA	protein_cc
ENSG0000(	3824.75	-0.13733	0.084072	-1.63347	0.102369	0.157678	BHLHE40	protein_cc
ENSG0000(	3823.949	-0.16634	0.038802	-4.28701	1.81E-05	6.00E-05	SCAMP1	protein_cc
ENSG0000(	3823.784	0.089183	0.059734	1.492989	0.13544	0.200304	DHCR7	protein_cc
ENSG0000(	3822.321	0.140747	0.02905	4.844981	1.27E-06	5.04E-06	SRSF10	protein_cc
ENSG0000(	3821.814	0.246863	0.0809	3.051455	0.002277	0.0052	ASCL1	protein_cc
ENSG0000(	3817.954	-0.05617	0.022977	-2.44437	0.01451	0.027965	TBC1D20	protein_cc
ENSG0000(	3816.849	0.420264	0.097428	4.313608	1.61E-05	5.36E-05	ARL4C	protein_cc
ENSG0000(	3815.742	-0.08849	0.038796	-2.28101	0.022548	0.041505	PPP1R12A	protein_cc
ENSG0000(	3815.39	-0.53517	0.045405	-11.7864	4.59E-32	1.53E-29	CAMTA2	protein_cc
ENSG0000(	3814.812	-0.14952	0.03353	-4.45937	8.22E-06	2.87E-05	KPNA6	protein_cc
ENSG0000(	3814.793	-0.00341	0.023316	-0.14632	0.883672	0.917032	MRPS27	protein_cc
ENSG0000(	3811.108	-0.09973	0.026313	-3.79018	0.000151	0.000426	PLRG1	protein_cc
ENSG0000(	3810.424	-0.43083	0.050912	-8.4622	2.62E-17	5.90E-16	FBXO44	protein_cc
ENSG0000(	3809.282	-0.1545	0.052343	-2.95162	0.003161	0.007012	GPR137B	protein_cc
ENSG0000(	3805.26	0.071309	0.03223	2.212505	0.026932	0.048677	THOP1	protein_cc
ENSG0000(	3805.186	0.259772	0.105568	2.460702	0.013867	0.026824	TAGLN	protein_cc
ENSG0000(	3804.862	-0.22589	0.053017	-4.26058	2.04E-05	6.70E-05	PALD1	protein_cc

ENSG0000(	3801.958	0.371918	0.053194	6.991683	2.72E-12	2.64E-11	DYNLT1	protein_cc
ENSG0000(	3800.245	-0.35778	0.043471	-8.23038	1.87E-16	3.62E-15	OXCT1	protein_cc
ENSG0000(	3799.684	0.15973	0.035748	4.468254	7.89E-06	2.77E-05	NDUFB10	protein_cc
ENSG0000(	3799.633	-0.31634	0.060063	-5.26682	1.39E-07	6.42E-07	NUDT16	protein_cc
ENSG0000(	3799.516	-0.3296	0.120982	-2.7244	0.006442	0.013412	FERMT1	protein_cc
ENSG0000(	3799.313	-0.26183	0.028727	-9.11443	7.91E-20	2.62E-18	VTI1B	protein_cc
ENSG0000(	3798.714	0.379131	0.040012	9.475418	2.66E-21	1.13E-19	HSD17B1(	protein_cc
ENSG0000(	3796.669	-0.16977	0.025835	-6.5712	4.99E-11	3.98E-10	ZMYM3	protein_cc
ENSG0000(	3796.079	-0.59237	0.072504	-8.17021	3.08E-16	5.77E-15	CIT	protein_cc
ENSG0000(	3795.286	-0.06586	0.02566	-2.5668	0.010264	0.020447	PDHB	protein_cc
ENSG0000(	3792.545	0.006412	0.030253	0.211958	0.83214	0.877504	STX6	protein_cc
ENSG0000(	3792.298	-0.15609	0.039595	-3.94205	8.08E-05	0.00024	NIPBL	protein_cc
ENSG0000(	3788.439	0.02178	0.025255	0.862425	0.388454	0.485142	FYTTD1	protein_cc
ENSG0000(	3785.79	0.454599	0.07562	6.011646	1.84E-09	1.15E-08	NEK6	protein_cc
ENSG0000(	3785.541	-0.32273	0.041768	-7.72678	1.10E-14	1.59E-13	TCEAL3	protein_cc
ENSG0000(	3782.619	-0.32125	0.034871	-9.21244	3.19E-20	1.12E-18	PPM1A	protein_cc
ENSG0000(	3782.094	0.150695	0.029025	5.191979	2.08E-07	9.36E-07	GART	protein_cc
ENSG0000(	3781.327	-0.64939	0.055796	-11.6386	2.62E-31	7.65E-29	BCL2L2	protein_cc
ENSG0000(	3779.345	-0.07107	0.052274	-1.35962	0.17395	0.248093	TUBGCP6	protein_cc
ENSG0000(	3779.157	0.028622	0.042126	0.679435	0.496862	0.591004	PIN1	protein_cc
ENSG0000(	3778.327	0.318788	0.036041	8.845061	9.15E-19	2.55E-17	SLC35B2	protein_cc
ENSG0000(	3778.204	-0.67186	0.098366	-6.83021	8.48E-12	7.61E-11	CLSTN2	protein_cc
ENSG0000(	3778.136	-0.23543	0.029989	-7.85033	4.15E-15	6.47E-14	DCAF5	protein_cc
ENSG0000(	3778.015	-0.03894	0.057479	-0.67738	0.498167	0.592151	HNMT	protein_cc
ENSG0000(	3778.012	-0.09576	0.058243	-1.64418	0.100138	0.154721	NACAD	protein_cc
ENSG0000(	3777.952	-0.32198	0.041801	-7.70282	1.33E-14	1.88E-13	ATP6V1H	protein_cc
ENSG0000(	3777.616	-0.15365	0.025728	-5.9721	2.34E-09	1.44E-08	EXOC3	protein_cc
ENSG0000(	3776.86	0.095815	0.033445	2.864839	0.004172	0.009043	LMBR1	protein_cc
ENSG0000(	3776.059	-0.04445	0.034308	-1.29574	0.195065	0.2734	POMGNT2	protein_cc
ENSG0000(	3775.476	0.228073	0.043353	5.260903	1.43E-07	6.62E-07	RBM42	protein_cc
ENSG0000(	3774.129	-0.72013	0.191187	-3.76662	0.000165	0.000466	TPTEP1	lincRNA
ENSG0000(	3771.673	-0.16459	0.050623	-3.25121	0.001149	0.002768	BID	protein_cc
ENSG0000(	3771.234	-0.00162	0.081078	-0.02001	0.984038	0.989041	BRINP2	protein_cc
ENSG0000(	3770.906	0.429341	0.101808	4.217163	2.47E-05	8.00E-05	SRPX	protein_cc
ENSG0000(	3770.332	0.140636	0.026805	5.246553	1.55E-07	7.12E-07	PPP4R1	protein_cc
ENSG0000(	3769.692	0.075665	0.026415	2.864455	0.004177	0.009053	NUB1	protein_cc
ENSG0000(	3769.677	-0.12749	0.038582	-3.30425	0.000952	0.002328	RSU1	protein_cc
ENSG0000(	3769.215	-0.45199	0.049412	-9.14737	5.83E-20	1.99E-18	AMOTL1	protein_cc
ENSG0000(	3768.449	-0.46465	0.063743	-7.2895	3.11E-13	3.49E-12	DAPK1	protein_cc
ENSG0000(	3768.169	-0.15708	0.042325	-3.71134	0.000206	0.00057	FRYL	protein_cc
ENSG0000(	3767.796	0.790128	0.109201	7.235552	4.64E-13	5.06E-12	MDK	protein_cc
ENSG0000(	3767.672	0.128104	0.026734	4.791791	1.65E-06	6.46E-06	SF3B4	protein_cc
ENSG0000(	3767.353	-0.49668	0.044771	-11.0936	1.35E-28	2.40E-26	MEF2D	protein_cc
ENSG0000(	3765.78	0.241078	0.032443	7.43078	1.08E-13	1.31E-12	DHX40	protein_cc
ENSG0000(	3765.576	0.045804	0.049042	0.933985	0.350312	0.445751	PKIG	protein_cc
ENSG0000(	3763.61	-0.23625	0.067444	-3.50296	0.00046	0.001196	HDAC4	protein_cc
ENSG0000(	3763.606	0.061737	0.029886	2.065773	0.03885	0.067609	ERLEC1	protein_cc
ENSG0000(	3763.248	-0.38575	0.081006	-4.76207	1.92E-06	7.41E-06	RAP1GAP	protein_cc
ENSG0000(	3763.049	0.107332	0.047125	2.277593	0.022751	0.041834	MLLT4	protein_cc
ENSG0000(	3761.627	0.007931	0.075618	0.10488	0.916471	0.941157	C3orf70	protein_cc
ENSG0000(	3761.149	-0.67929	0.073995	-9.18022	4.30E-20	1.49E-18	TEF	protein_cc
ENSG0000(	3760.976	0.045141	0.056762	0.795273	0.426455	0.523586	PMEPA1	protein_cc
ENSG0000(	3760.333	-0.43454	0.093756	-4.63475	3.57E-06	1.32E-05	PHACTR3	protein_cc
ENSG0000(	3759.617	0.039464	0.031778	1.241857	0.214289	0.296007	NAGK	protein_cc
ENSG0000(	3759.197	-0.07409	0.037784	-1.96097	0.049882	0.084334	BAG1	protein_cc
ENSG0000(	3758.432	0.078208	0.030724	2.545553	0.01091	0.021618	EIF2S1	protein_cc
ENSG0000(	3757.6	0.465184	0.048088	9.67353	3.91E-22	1.93E-20	TMPO	protein_cc
ENSG0000(	3756.484	-0.0229	0.022431	-1.02088	0.307312	0.400339	YIPF4	protein_cc
ENSG0000(	3756.232	-0.26606	0.064216	-4.14319	3.43E-05	0.000108	VCL	protein_cc

ENSG0000(	3754.649	-0.29257	0.034785	-8.41087	4.07E-17	8.82E-16	PTEN	protein_cc
ENSG0000(	3754.272	0.355166	0.061763	5.750505	8.90E-09	4.97E-08	NT5DC2	protein_cc
ENSG0000(	3753.663	-0.05536	0.027867	-1.98642	0.046987	0.079939	CTCF	protein_cc
ENSG0000(	3752.008	-0.44152	0.05407	-8.16573	3.20E-16	5.97E-15	DIP2C	protein_cc
ENSG0000(	3750.022	-0.16908	0.092065	-1.83647	0.066288	0.108051	CPNE5	protein_cc
ENSG0000(	3749.905	-0.49195	0.118032	-4.16796	3.07E-05	9.80E-05	TMEM144	protein_cc
ENSG0000(	3749.807	-0.10139	0.023864	-4.2487	2.15E-05	7.03E-05	SEC22C	protein_cc
ENSG0000(	3748.256	-0.27515	0.042425	-6.48557	8.84E-11	6.80E-10	LZTS2	protein_cc
ENSG0000(	3746.849	0.308751	0.038987	7.919354	2.39E-15	3.87E-14	HSPE1	protein_cc
ENSG0000(	3746.32	0.090785	0.02737	3.317018	0.00091	0.002233	EIF2S2	protein_cc
ENSG0000(	3744.089	0.000323	0.021465	0.015048	0.987994	0.991749	RNF4	protein_cc
ENSG0000(	3743.617	-0.04251	0.030546	-1.39167	0.164022	0.236096	PCNXL4	protein_cc
ENSG0000(	3742.987	-0.29663	0.040771	-7.27555	3.45E-13	3.85E-12	AKAP1	protein_cc
ENSG0000(	3742.937	-0.03744	0.022225	-1.68451	0.092083	0.143805	CUL3	protein_cc
ENSG0000(	3741.316	-0.11298	0.02849	-3.96541	7.33E-05	0.000219	RALGAPB	protein_cc
ENSG0000(	3740.709	0.11027	0.042568	2.590424	0.009586	0.019231	NDUFA1	protein_cc
ENSG0000(	3740.173	0.145235	0.034175	4.249729	2.14E-05	7.01E-05	C17orf62	protein_cc
ENSG0000(	3739.243	0.060393	0.029166	2.070646	0.038392	0.066925	PCNXL3	protein_cc
ENSG0000(	3738.907	-0.18793	0.035081	-5.35704	8.46E-08	4.05E-07	ZDHHC17	protein_cc
ENSG0000(	3737.864	0.048433	0.028548	1.696581	0.089776	0.140715	CYB5B	protein_cc
ENSG0000(	3736.957	0.015833	0.037084	0.426944	0.66942	0.744798	NDUFS7	protein_cc
ENSG0000(	3735.948	0.215632	0.048035	4.489054	7.15E-06	2.53E-05	AP1S1	protein_cc
ENSG0000(	3731.075	0.077592	0.032508	2.386878	0.016992	0.032184	POMT1	protein_cc
ENSG0000(	3726.83	-0.65843	0.079906	-8.2401	1.72E-16	3.37E-15	KIAA1598	protein_cc
ENSG0000(	3726.144	0.756243	0.082615	9.153794	5.50E-20	1.88E-18	PTGFRN	protein_cc
ENSG0000(	3725.42	0.2083	0.025048	8.316066	9.09E-17	1.86E-15	POLR2C	protein_cc
ENSG0000(	3724.286	-0.26255	0.045683	-5.74727	9.07E-09	5.06E-08	KIAA0247	protein_cc
ENSG0000(	3723.881	-0.13111	0.081413	-1.61048	0.107294	0.164168	COL18A1	protein_cc
ENSG0000(	3718.014	-0.42442	0.046263	-9.17409	4.55E-20	1.57E-18	SPTLC2	protein_cc
ENSG0000(	3717.596	-0.22356	0.02989	-7.47949	7.46E-14	9.29E-13	OSBPL2	protein_cc
ENSG0000(	3717.008	0.018528	0.038876	0.476588	0.633656	0.714652	CAMLG	protein_cc
ENSG0000(	3715.684	0.014919	0.038681	0.38569	0.699726	0.77045	COX7A2L	protein_cc
ENSG0000(	3714.502	-0.57302	0.147791	-3.87725	0.000106	0.000307	MTND4P1	pseudoge
ENSG0000(	3713.748	-0.20396	0.030018	-6.79457	1.09E-11	9.57E-11	KIAA0226	protein_cc
ENSG0000(	3712.811	-0.04421	0.025512	-1.73294	0.083106	0.131433	UBE2J1	protein_cc
ENSG0000(	3712.634	0.255706	0.039426	6.485775	8.83E-11	6.79E-10	STOML2	protein_cc
ENSG0000(	3710.334	0.18875	0.067954	2.777595	0.005476	0.011573	SOX13	protein_cc
ENSG0000(	3709.876	-0.48091	0.118811	-4.04768	5.17E-05	0.000159	NELL2	protein_cc
ENSG0000(	3709.634	-0.19573	0.098214	-1.99293	0.046269	0.078885	GADD45B	protein_cc
ENSG0000(	3709.109	0.102024	0.028557	3.572641	0.000353	0.00094	TMEM131	protein_cc
ENSG0000(	3709.032	0.28019	0.038028	7.367921	1.73E-13	2.02E-12	CDC34	protein_cc
ENSG0000(	3708.998	0.077895	0.100345	0.776271	0.437589	0.534576	IGFBP4	protein_cc
ENSG0000(	3708.687	0.399404	0.058537	6.823153	8.91E-12	7.96E-11	AP1S2	protein_cc
ENSG0000(	3708.323	-0.07979	0.023178	-3.44243	0.000577	0.001471	PPP1R11	protein_cc
ENSG0000(	3708.187	0.031972	0.030758	1.039476	0.298583	0.390879	WRB	protein_cc
ENSG0000(	3706.368	-0.11774	0.036703	-3.20785	0.001337	0.003187	DLG1	protein_cc
ENSG0000(	3705.139	0.119064	0.065867	1.807633	0.070664	0.114228	PECAM1	protein_cc
ENSG0000(	3704.087	-0.03801	0.059157	-0.64252	0.520535	0.612839	PTPN13	protein_cc
ENSG0000(	3702.845	0.179084	0.039919	4.486163	7.25E-06	2.56E-05	DMTF1	protein_cc
ENSG0000(	3702.621	-0.36293	0.059313	-6.11892	9.42E-10	6.17E-09	FAM49A	protein_cc
ENSG0000(	3702.271	-0.19428	0.039253	-4.94938	7.44E-07	3.07E-06	PANK3	protein_cc
ENSG0000(	3700.507	-0.12076	0.030887	-3.90971	9.24E-05	0.000272	MIEF1	protein_cc
ENSG0000(	3699.655	0.085514	0.037049	2.308096	0.020994	0.038898	GTPBP6	protein_cc
ENSG0000(	3698.953	0.032962	0.031003	1.063166	0.287707	0.378952	C5orf24	protein_cc
ENSG0000(	3698.766	-0.07823	0.039022	-2.00463	0.045003	0.076952	RETSAT	protein_cc
ENSG0000(	3697.943	0.078522	0.035512	2.211103	0.027029	0.048826	EIF2A	protein_cc
ENSG0000(	3696.135	-0.21306	0.032923	-6.47133	9.71E-11	7.42E-10	WDTC1	protein_cc
ENSG0000(	3695.724	-0.89188	0.104452	-8.53871	1.36E-17	3.18E-16	PDE2A	protein_cc
ENSG0000(	3695.202	-0.70878	0.067794	-10.455	1.39E-25	1.36E-23	CAMKK2	protein_cc

ENSG0000(	3695.116	-0.0426	0.028488	-1.49553	0.134775	0.199457	SIN3A	protein_cc
ENSG0000(	3694.679	-0.26851	0.049802	-5.39148	6.99E-08	3.39E-07	NIN	protein_cc
ENSG0000(	3693.837	0.124758	0.035726	3.492078	0.000479	0.001241	EMC7	protein_cc
ENSG0000(	3693.721	-0.07434	0.050933	-1.45963	0.14439	0.211667	ARHGAP3	protein_cc
ENSG0000(	3693.628	-0.2969	0.075856	-3.91394	9.08E-05	0.000267	SLC7A11	protein_cc
ENSG0000(	3692.577	0.011162	0.029161	0.38277	0.70189	0.772173	IDH3G	protein_cc
ENSG0000(	3692.019	-0.15108	0.036736	-4.11259	3.91E-05	0.000122	RNF38	protein_cc
ENSG0000(	3691.627	0.584683	0.102968	5.67831	1.36E-08	7.39E-08	CAPG	protein_cc
ENSG0000(	3690.696	0.164292	0.02825	5.815596	6.04E-09	3.47E-08	DAP3	protein_cc
ENSG0000(	3690.059	-0.34064	0.042738	-7.97043	1.58E-15	2.65E-14	DICER1	protein_cc
ENSG0000(	3689.3	0.030441	0.026433	1.151631	0.249473	0.336382	PCIF1	protein_cc
ENSG0000(	3688.217	-0.10704	0.047517	-2.25269	0.024279	0.044363	KDM5B	protein_cc
ENSG0000(	3686.458	-0.0477	0.026833	-1.77754	0.07548	0.121015	PPP2R5D	protein_cc
ENSG0000(	3684.041	-0.05203	0.02582	-2.01503	0.043901	0.07531	SART3	protein_cc
ENSG0000(	3682.135	0.31201	0.049895	6.253366	4.02E-10	2.78E-09	SPPL2B	processed
ENSG0000(	3680.738	-0.09772	0.059128	-1.65268	0.098396	0.152363	NINJ1	protein_cc
ENSG0000(	3680.311	0.057402	0.039789	1.442654	0.149118	0.217674	CREBZF	protein_cc
ENSG0000(	3678.927	-0.20389	0.037796	-5.39454	6.87E-08	3.34E-07	RC3H2	protein_cc
ENSG0000(	3678.67	-0.15049	0.027314	-5.50973	3.59E-08	1.83E-07	SLC41A3	protein_cc
ENSG0000(	3677.81	0.256241	0.037301	6.869522	6.44E-12	5.89E-11	EXT2	protein_cc
ENSG0000(	3677.435	-0.03155	0.033995	-0.92811	0.353353	0.448908	N4BP2L2	protein_cc
ENSG0000(	3675.314	0.105658	0.043009	2.456648	0.014024	0.027103	ADI1	protein_cc
ENSG0000(	3673.461	-0.04172	0.02814	-1.48265	0.138169	0.203789	DFFA	protein_cc
ENSG0000(	3673.317	0.059743	0.03375	1.770147	0.076703	0.122708	CARS	protein_cc
ENSG0000(	3672.593	0.162886	0.062136	2.621462	0.008755	0.017721	SLC38A5	protein_cc
ENSG0000(	3672.202	-0.28704	0.033781	-8.49731	1.94E-17	4.43E-16	TSR2	protein_cc
ENSG0000(	3671.483	-0.03608	0.021794	-1.65561	0.0978	0.151537	TMEM33	protein_cc
ENSG0000(	3670.193	-0.19939	0.078046	-2.55478	0.010626	0.0211	ID1	protein_cc
ENSG0000(	3669.335	-0.25802	0.038013	-6.78759	1.14E-11	1.00E-10	UBE2O	protein_cc
ENSG0000(	3669.249	0.092817	0.04708	1.971482	0.048669	0.082493	TANC1	protein_cc
ENSG0000(	3664.327	-0.0259	0.020665	-1.25342	0.210053	0.291055	MBD1	protein_cc
ENSG0000(	3663.241	0.030704	0.050595	0.606859	0.543945	0.634524	TAF1C	protein_cc
ENSG0000(	3663.076	0.054339	0.027641	1.9659	0.04931	0.08347	TARS	protein_cc
ENSG0000(	3662.859	0.292787	0.030701	9.536789	1.47E-21	6.62E-20	PSMD14	protein_cc
ENSG0000(	3662.641	-0.22458	0.030508	-7.36119	1.82E-13	2.12E-12	RLIM	protein_cc
ENSG0000(	3662.213	-0.092	0.030274	-3.03894	0.002374	0.005405	FAR1	protein_cc
ENSG0000(	3662.201	0.047135	0.031361	1.503006	0.132838	0.197028	BUB3	protein_cc
ENSG0000(	3659.044	0.218857	0.039022	5.608519	2.04E-08	1.08E-07	ECI2	protein_cc
ENSG0000(	3658.549	-0.63739	0.056092	-11.3633	6.37E-30	1.41E-27	PPP1R12B	protein_cc
ENSG0000(	3658.353	0.079115	0.039943	1.980707	0.047624	0.080896	ILVBL	protein_cc
ENSG0000(	3657.501	-0.01512	0.022101	-0.6841	0.493912	0.5883	CSNK2A2	protein_cc
ENSG0000(	3656.957	0.028634	0.047368	0.604498	0.545513	0.636001	SNHG6	processed
ENSG0000(	3655.717	-0.12855	0.048886	-2.62958	0.008549	0.01735	GYG1	protein_cc
ENSG0000(	3653.335	-0.0275	0.069182	-0.39756	0.690951	0.763117	ELOVL1	protein_cc
ENSG0000(	3652.355	0.121803	0.038349	3.176178	0.001492	0.003527	ADAM15	protein_cc
ENSG0000(	3652.23	-0.03656	0.025458	-1.436	0.151002	0.220081	TTC19	protein_cc
ENSG0000(	3651.573	-0.13598	0.04618	-2.94455	0.003234	0.007161	ZCCHC14	protein_cc
ENSG0000(	3650.807	-0.37303	0.043719	-8.53226	1.44E-17	3.35E-16	FOXN3	protein_cc
ENSG0000(	3650.575	0.531655	0.065222	8.151518	3.59E-16	6.68E-15	KPNA2	protein_cc
ENSG0000(	3649.151	0.468924	0.05574	8.412716	4.01E-17	8.69E-16	C12orf57	protein_cc
ENSG0000(	3649.127	0.003324	0.029109	0.114193	0.909085	0.935683	PRPF4B	protein_cc
ENSG0000(	3648.66	0.182305	0.033961	5.367998	7.96E-08	3.83E-07	MEA1	protein_cc
ENSG0000(	3647.788	1.277693	0.253817	5.033911	4.81E-07	2.04E-06	POSTN	protein_cc
ENSG0000(	3647.192	0.155496	0.0387	4.017947	5.87E-05	0.000179	RHOT2	protein_cc
ENSG0000(	3645.265	-0.16194	0.036686	-4.41412	1.01E-05	3.50E-05	ANKRD52	protein_cc
ENSG0000(	3641.947	-0.16298	0.043864	-3.71553	0.000203	0.000561	ATM	protein_cc
ENSG0000(	3640.471	-1.20757	0.12473	-9.68146	3.62E-22	1.80E-20	PPP2R2C	protein_cc
ENSG0000(	3639.48	-0.13408	0.091707	-1.46205	0.143727	0.210845	DSCAM	protein_cc
ENSG0000(	3638.971	0.051725	0.032347	1.599069	0.109805	0.167481	DECR1	protein_cc

ENSG0000(	3635.74	-0.10556	0.031059	-3.39878	0.000677	0.001703	USP8	protein_cc
ENSG0000(	3635.711	0.238515	0.026854	8.881978	6.57E-19	1.88E-17	USF1	protein_cc
ENSG0000(	3634.199	-0.04695	0.048286	-0.97235	0.330877	0.425311	MAN2C1	protein_cc
ENSG0000(	3634.064	0.415509	0.052235	7.954583	1.80E-15	2.98E-14	POU3F2	protein_cc
ENSG0000(	3633.632	0.370552	0.050754	7.300921	2.86E-13	3.22E-12	TSPAN6	protein_cc
ENSG0000(	3633.29	0.213384	0.067955	3.140097	0.001689	0.003951	TMOD1	protein_cc
ENSG0000(	3631.84	0.067433	0.02075	3.249753	0.001155	0.00278	GLOD4	protein_cc
ENSG0000(	3630.773	-1.16062	0.139814	-8.30118	1.03E-16	2.09E-15	NPTX1	protein_cc
ENSG0000(	3627.964	-0.56709	0.080819	-7.01686	2.27E-12	2.23E-11	SEMA6B	protein_cc
ENSG0000(	3626.573	0.107344	0.032662	3.286477	0.001014	0.002469	GTF3C5	protein_cc
ENSG0000(	3626.102	0.102699	0.043318	2.370817	0.017749	0.033422	UBE2M	protein_cc
ENSG0000(	3626.049	0.123294	0.04545	2.712733	0.006673	0.013852	ZNF428	protein_cc
ENSG0000(	3626.042	-0.52481	0.069504	-7.55083	4.32E-14	5.59E-13	PFKFB2	protein_cc
ENSG0000(	3625.852	0.152691	0.152956	0.998266	0.31815	0.41181	MGP	protein_cc
ENSG0000(	3625.445	-0.05641	0.07267	-0.7763	0.43757	0.534568	IL13RA1	protein_cc
ENSG0000(	3622.59	-0.07728	0.031504	-2.453	0.014167	0.02736	SPG11	protein_cc
ENSG0000(	3620.614	0.04839	0.032956	1.468334	0.142014	0.208659	UBE3C	protein_cc
ENSG0000(	3619.97	0.267461	0.058977	4.535017	5.76E-06	2.07E-05	COLGALT:	protein_cc
ENSG0000(	3619.753	0.131211	0.0203	6.463663	1.02E-10	7.78E-10	RBM22	protein_cc
ENSG0000(	3618.784	0.085563	0.03653	2.342309	0.019165	0.035829	ETFA	protein_cc
ENSG0000(	3618.623	-0.48562	0.08037	-6.04229	1.52E-09	9.61E-09	GRAMD1E	protein_cc
ENSG0000(	3617.417	-0.0459	0.031305	-1.46617	0.142602	0.209434	CSK	protein_cc
ENSG0000(	3616.765	-0.34488	0.058145	-5.9314	3.00E-09	1.81E-08	IL17RD	protein_cc
ENSG0000(	3616.65	0.240353	0.039323	6.112325	9.82E-10	6.41E-09	TMEM205	protein_cc
ENSG0000(	3613.916	0.224238	0.044167	5.077022	3.83E-07	1.66E-06	AURKAIP1	protein_cc
ENSG0000(	3612.129	-0.54356	0.074361	-7.30974	2.68E-13	3.04E-12	LHPP	protein_cc
ENSG0000(	3612.073	-0.00435	0.029147	-0.14924	0.881362	0.915212	MCCC2	protein_cc
ENSG0000(	3609.978	-0.12049	0.036541	-3.29729	0.000976	0.002382	KLHL42	protein_cc
ENSG0000(	3609.158	0.004114	0.03043	0.135205	0.89245	0.923461	IPO8	protein_cc
ENSG0000(	3609.094	0.277712	0.031695	8.761926	1.92E-18	5.12E-17	UBE2E3	protein_cc
ENSG0000(	3607.194	0.147042	0.034453	4.26788	1.97E-05	6.50E-05	NDUFB4	protein_cc
ENSG0000(	3606.274	-0.1883	0.035704	-5.27395	1.34E-07	6.20E-07	EHBP1	protein_cc
ENSG0000(	3603.568	0.389062	0.047644	8.166105	3.19E-16	5.95E-15	PHF19	protein_cc
ENSG0000(	3601.228	0.044222	0.049223	0.898398	0.368974	0.465336	ARHGEF1	protein_cc
ENSG0000(	3601.096	-0.57445	0.10179	-5.64347	1.67E-08	8.93E-08	TAGLN3	protein_cc
ENSG0000(	3600.589	0.128118	0.031732	4.03751	5.40E-05	0.000165	VPS72	protein_cc
ENSG0000(	3599.981	-0.01215	0.032031	-0.37924	0.704513	0.774435	POLR1A	protein_cc
ENSG0000(	3598.882	0.242368	0.039761	6.095631	1.09E-09	7.07E-09	RER1	protein_cc
ENSG0000(	3598.408	-0.06491	0.034915	-1.859	0.063027	0.103424	NR1H2	protein_cc
ENSG0000(	3595.344	-0.12366	0.036142	-3.42164	0.000622	0.001577	YEATS2	protein_cc
ENSG0000(	3593.689	-0.34592	0.13825	-2.5021	0.012346	0.024144	RPH3A	protein_cc
ENSG0000(	3592.935	-0.07497	0.03346	-2.24065	0.025049	0.045618	TRAP1	protein_cc
ENSG0000(	3592.073	-0.07813	0.026423	-2.95678	0.003109	0.006906	SLC25A46	protein_cc
ENSG0000(	3590.612	0.034986	0.027667	1.264539	0.206037	0.286426	UCK1	protein_cc
ENSG0000(	3589.227	-0.02913	0.021441	-1.35847	0.174313	0.248577	SNX12	protein_cc
ENSG0000(	3588.887	0.043333	0.025574	1.694452	0.090179	0.141272	PFDN1	protein_cc
ENSG0000(	3588.106	0.028843	0.033373	0.864256	0.387447	0.484121	NPRL3	protein_cc
ENSG0000(	3588.015	-0.14054	0.030084	-4.67162	2.99E-06	1.12E-05	PDHA1	protein_cc
ENSG0000(	3587.282	-1.06392	0.110112	-9.66219	4.36E-22	2.14E-20	DMTN	protein_cc
ENSG0000(	3587.189	-0.41377	0.044293	-9.34164	9.49E-21	3.67E-19	TECPR2	protein_cc
ENSG0000(	3586.02	-0.1154	0.026517	-4.35184	1.35E-05	4.56E-05	PCYT1A	protein_cc
ENSG0000(	3585.288	0.003191	0.035873	0.088949	0.929123	0.950195	P4HTM	protein_cc
ENSG0000(	3583.075	0.144167	0.036686	3.929787	8.50E-05	0.000252	BTF3L4	protein_cc
ENSG0000(	3582.531	-0.1026	0.030453	-3.3691	0.000754	0.001879	PITPNB	protein_cc
ENSG0000(	3582.499	0.129437	0.040401	3.203785	0.001356	0.003228	COX5A	protein_cc
ENSG0000(	3579.196	0.280934	0.044108	6.369168	1.90E-10	1.38E-09	TXNDC12	protein_cc
ENSG0000(	3578.923	0.135982	0.043923	3.095902	0.001962	0.004533	CKAP4	protein_cc
ENSG0000(	3576.921	-0.00434	0.020648	-0.21004	0.833636	0.878744	IMP4	protein_cc
ENSG0000(	3574.766	-0.50593	0.056725	-8.91894	4.71E-19	1.38E-17	WWC1	protein_cc

ENSG0000(	3573.538	-0.25772	0.043564	-5.91579	3.30E-09	1.98E-08	ASB1	protein_cc
ENSG0000(	3573.386	-0.23562	0.04301	-5.47839	4.29E-08	2.16E-07	NR2C2	protein_cc
ENSG0000(	3573.323	-0.08242	0.03814	-2.16107	0.03069	0.05475	CHURC1	protein_cc
ENSG0000(	3573.303	0.115478	0.032048	3.603252	0.000314	0.000843	MTFR1L	protein_cc
ENSG0000(	3572.414	-0.29662	0.051165	-5.79727	6.74E-09	3.85E-08	SMAD3	protein_cc
ENSG0000(	3572.029	0.064534	0.059321	1.087881	0.276648	0.366759	FAM212B	protein_cc
ENSG0000(	3571.122	0.042361	0.03915	1.081995	0.279255	0.369691	SAMD1	protein_cc
ENSG0000(	3570.622	0.19796	0.030956	6.394989	1.61E-10	1.18E-09	SRSF9	protein_cc
ENSG0000(	3569.255	0.415145	0.121083	3.428588	0.000607	0.001541	ARC	protein_cc
ENSG0000(	3565.709	-0.18975	0.040163	-4.72453	2.31E-06	8.81E-06	CREBL2	protein_cc
ENSG0000(	3565.029	-0.12768	0.057796	-2.20915	0.027164	0.049051	PCDH1	protein_cc
ENSG0000(	3564.402	0.20622	0.079801	2.584184	0.009761	0.019546	UBE2Q2P(	pseudoge
ENSG0000(	3558.441	-0.1567	0.029347	-5.33965	9.31E-08	4.43E-07	SLC25A44	protein_cc
ENSG0000(	3557.833	-0.17469	0.029456	-5.9308	3.01E-09	1.82E-08	MON1B	protein_cc
ENSG0000(	3552.981	0.05771	0.03282	1.758365	0.078685	0.12546	SREK1	protein_cc
ENSG0000(	3552.459	0.279481	0.034617	8.073496	6.83E-16	1.21E-14	MPDU1	protein_cc
ENSG0000(	3551.87	0.447747	0.114736	3.902418	9.52E-05	0.000279	S100A11	protein_cc
ENSG0000(	3549.855	-0.29549	0.037488	-7.88224	3.22E-15	5.11E-14	EZH1	protein_cc
ENSG0000(	3545.904	0.081195	0.025479	3.186768	0.001439	0.003408	TRIM27	protein_cc
ENSG0000(	3545.844	-0.21661	0.032627	-6.63906	3.16E-11	2.60E-10	NF2	protein_cc
ENSG0000(	3545.435	-0.22377	0.0429	-5.21605	1.83E-07	8.30E-07	RCBTB1	protein_cc
ENSG0000(	3545.322	-0.10753	0.031293	-3.43628	0.00059	0.001502	MYBBP1A	protein_cc
ENSG0000(	3543.796	0.060935	0.036614	1.664267	0.096059	0.14921	ATPIF1	protein_cc
ENSG0000(	3542.865	-0.07359	0.027744	-2.65261	0.007987	0.01632	RFC1	protein_cc
ENSG0000(	3542.561	0.111669	0.043132	2.589038	0.009624	0.019299	UQCR10	protein_cc
ENSG0000(	3542.366	-0.25972	0.07418	-3.50122	0.000463	0.001203	C15orf59	protein_cc
ENSG0000(	3540.344	-0.19507	0.036058	-5.40985	6.31E-08	3.09E-07	RPRD2	protein_cc
ENSG0000(	3539.493	-0.43713	0.070127	-6.23342	4.56E-10	3.14E-09	KCNMB4	protein_cc
ENSG0000(	3536.03	0.043299	0.025331	1.709311	0.087393	0.137463	CNOT8	protein_cc
ENSG0000(	3535.944	-0.05826	0.041736	-1.39588	0.162749	0.234452	FUBP1	protein_cc
ENSG0000(	3534.877	-0.07555	0.030846	-2.44916	0.014319	0.027634	FAM160B2	protein_cc
ENSG0000(	3534.341	0.002643	0.033711	0.078387	0.93752	0.956428	ATIC	protein_cc
ENSG0000(	3530.294	-0.28704	0.038984	-7.36309	1.80E-13	2.09E-12	CCDC92	protein_cc
ENSG0000(	3528.23	-0.42653	0.055514	-7.68325	1.55E-14	2.17E-13	MDN1	protein_cc
ENSG0000(	3527.497	0.058409	0.046575	1.254093	0.209808	0.290765	DCXR	protein_cc
ENSG0000(	3525.281	-0.14586	0.043117	-3.383	0.000717	0.001795	SLC35E2B	protein_cc
ENSG0000(	3525.091	-0.04278	0.044167	-0.96863	0.33273	0.427291	NIPSNAP1	protein_cc
ENSG0000(	3525.09	-0.34779	0.042851	-8.11618	4.81E-16	8.76E-15	CAMTA1	protein_cc
ENSG0000(	3525.037	0.169346	0.036748	4.608328	4.06E-06	1.49E-05	YIF1A	protein_cc
ENSG0000(	3524.72	0.09089	0.026948	3.372787	0.000744	0.001856	LEPROTL1	protein_cc
ENSG0000(	3524.13	-0.40012	0.049958	-8.00911	1.16E-15	1.97E-14	BAIAP2	protein_cc
ENSG0000(	3523.415	0.392248	0.043889	8.937302	3.99E-19	1.18E-17	MCM3	protein_cc
ENSG0000(	3523.226	0.097995	0.024119	4.062925	4.85E-05	0.000149	FAF2	protein_cc
ENSG0000(	3522.251	0.044989	0.031815	1.414065	0.157343	0.227813	CHERP	protein_cc
ENSG0000(	3521.25	0.017938	0.020975	0.855211	0.392434	0.489059	KANSL3	protein_cc
ENSG0000(	3521.027	-0.10364	0.037352	-2.77478	0.005524	0.011663	PNKD	protein_cc
ENSG0000(	3518.984	-0.00738	0.077823	-0.09479	0.924478	0.946787	KLF15	protein_cc
ENSG0000(	3516.899	0.000355	0.032774	0.010843	0.991349	0.99407	GRAMD1A	protein_cc
ENSG0000(	3516.169	0.091152	0.02683	3.397314	0.000681	0.001711	MRPL3	protein_cc
ENSG0000(	3514.988	0.203738	0.028338	7.189575	6.50E-13	6.95E-12	KBTBD2	protein_cc
ENSG0000(	3514.303	-0.48817	0.064641	-7.55205	4.28E-14	5.54E-13	BRSK1	protein_cc
ENSG0000(	3513.856	-0.0733	0.043715	-1.67668	0.093604	0.145865	YES1	protein_cc
ENSG0000(	3512.858	-0.10203	0.064406	-1.58422	0.113144	0.171878	APOL2	protein_cc
ENSG0000(	3511.431	-0.2064	0.066069	-3.12408	0.001784	0.004156	ARAP2	protein_cc
ENSG0000(	3509.507	-0.43455	0.095476	-4.55142	5.33E-06	1.92E-05	PLEKHA6	protein_cc
ENSG0000(	3508.153	-0.11146	0.027635	-4.0332	5.50E-05	0.000168	UBE2V2	protein_cc
ENSG0000(	3507.335	0.023648	0.086316	0.273974	0.784105	0.83929	PXDN	protein_cc
ENSG0000(	3506.149	0.217512	0.049959	4.353825	1.34E-05	4.52E-05	TXN	protein_cc
ENSG0000(	3505.389	0.311575	0.097517	3.195077	0.001398	0.00332	CPXM1	protein_cc

ENSG0000(	3505.364	-0.24597	0.042307	-5.81399	6.10E-09	3.50E-08	TSC1	protein_cc
ENSG0000(	3504.336	-0.2905	0.073366	-3.95966	7.51E-05	0.000224	DIRAS1	protein_cc
ENSG0000(	3503.086	0.201905	0.031201	6.4711	9.73E-11	7.43E-10	SUPT4H1	protein_cc
ENSG0000(	3502.387	-0.11665	0.032238	-3.61822	0.000297	0.000799	LZTR1	protein_cc
ENSG0000(	3500.196	-0.49934	0.04369	-11.4292	2.99E-30	7.23E-28	MAPK9	protein_cc
ENSG0000(	3497.828	0.096085	0.02782	3.45377	0.000553	0.001414	TSG101	protein_cc
ENSG0000(	3496.712	0.00399	0.049772	0.080172	0.936101	0.955359	H6PD	protein_cc
ENSG0000(	3496.083	-0.15391	0.026675	-5.77001	7.93E-09	4.47E-08	ATF6	protein_cc
ENSG0000(	3495.73	-0.0013	0.02864	-0.04524	0.963915	0.975347	URI1	protein_cc
ENSG0000(	3493.85	-0.28925	0.045183	-6.40172	1.54E-10	1.13E-09	ANKRD28	protein_cc
ENSG0000(	3493.419	-0.30015	0.05603	-5.35689	8.47E-08	4.05E-07	AXL	protein_cc
ENSG0000(	3492.904	0.112545	0.034403	3.271397	0.00107	0.002594	PCCB	protein_cc
ENSG0000(	3492.32	-0.13395	0.093662	-1.4301	0.15269	0.22221	IFIT1	protein_cc
ENSG0000(	3492.161	-0.28277	0.042867	-6.5965	4.21E-11	3.40E-10	C14orf159	protein_cc
ENSG0000(	3492.101	0.261277	0.038294	6.822851	8.93E-12	7.98E-11	ZFAND6	protein_cc
ENSG0000(	3490.704	0.107196	0.046148	2.322889	0.020185	0.037537	PLXNA3	protein_cc
ENSG0000(	3490.22	-0.05282	0.041988	-1.25793	0.208418	0.289159	PMVK	protein_cc
ENSG0000(	3489.285	-0.23262	0.049916	-4.66022	3.16E-06	1.18E-05	KIF2A	protein_cc
ENSG0000(	3489.018	0.280339	0.073961	3.790353	0.00015	0.000426	GLCCI1	protein_cc
ENSG0000(	3488.847	-0.477	0.082993	-5.74751	9.06E-09	5.05E-08	FGFR2	protein_cc
ENSG0000(	3488.693	-0.56246	0.077184	-7.28725	3.16E-13	3.54E-12	PTK2B	protein_cc
ENSG0000(	3487.064	0.130168	0.026522	4.907887	9.21E-07	3.74E-06	PMPCA	protein_cc
ENSG0000(	3486.888	-0.1202	0.030178	-3.9831	6.80E-05	0.000205	SLU7	protein_cc
ENSG0000(	3486.438	-0.08619	0.107748	-0.79989	0.423774	0.52084	MEGF11	protein_cc
ENSG0000(	3484.884	-0.08367	0.031448	-2.66061	0.0078	0.015974	SP1	protein_cc
ENSG0000(	3484.779	-0.27179	0.036496	-7.44706	9.54E-14	1.17E-12	ATXN1	protein_cc
ENSG0000(	3484.365	0.173933	0.055326	3.143761	0.001668	0.003906	MFSD2A	protein_cc
ENSG0000(	3483.717	0.005529	0.034978	0.158083	0.874392	0.909858	MRPL28	protein_cc
ENSG0000(	3478.754	-0.35239	0.054948	-6.41312	1.43E-10	1.06E-09	OXR1	protein_cc
ENSG0000(	3478.408	0.073899	0.029163	2.53401	0.011277	0.022266	UBR7	protein_cc
ENSG0000(	3475.76	-0.41628	0.057576	-7.23012	4.83E-13	5.25E-12	FBXW4	protein_cc
ENSG0000(	3475.389	0.003473	0.028999	0.119747	0.904684	0.932483	RBM15B	protein_cc
ENSG0000(	3475.178	-0.19577	0.036106	-5.42194	5.90E-08	2.90E-07	HOOK3	protein_cc
ENSG0000(	3474.889	-0.10644	0.027127	-3.9238	8.72E-05	0.000258	LSM14B	protein_cc
ENSG0000(	3473.719	0.268042	0.032789	8.174772	2.96E-16	5.57E-15	KLHL7	protein_cc
ENSG0000(	3473.201	-0.34184	0.074004	-4.61927	3.85E-06	1.42E-05	DTX4	protein_cc
ENSG0000(	3472.312	-0.29194	0.046245	-6.31291	2.74E-10	1.95E-09	ZFYVE16	protein_cc
ENSG0000(	3472.236	0.158201	0.094456	1.674868	0.09396	0.146381	TYROBP	protein_cc
ENSG0000(	3471.783	-0.00294	0.025506	-0.11516	0.908319	0.935062	YTHDF3	protein_cc
ENSG0000(	3471.531	-0.35771	0.0433	-8.26121	1.44E-16	2.85E-15	HABP4	protein_cc
ENSG0000(	3471.03	-0.12353	0.036672	-3.3685	0.000756	0.001883	FLYWCH1	protein_cc
ENSG0000(	3468.697	0.127531	0.033467	3.810669	0.000139	0.000395	TMEM222	protein_cc
ENSG0000(	3468.493	-0.16244	0.030547	-5.3176	1.05E-07	4.97E-07	MTMR4	protein_cc
ENSG0000(	3466.717	0.296473	0.040273	7.361623	1.82E-13	2.12E-12	IFRD1	protein_cc
ENSG0000(	3465.949	-0.46596	0.064883	-7.18151	6.89E-13	7.35E-12	MKL2	protein_cc
ENSG0000(	3464.675	0.082035	0.049171	1.668346	0.095247	0.148122	CHPT1	protein_cc
ENSG0000(	3464.554	-0.25935	0.047589	-5.44982	5.04E-08	2.50E-07	CEP350	protein_cc
ENSG0000(	3464.12	0.052255	0.04178	1.250725	0.211035	0.292218	MKNK2	protein_cc
ENSG0000(	3461.482	-0.42259	0.075178	-5.62125	1.90E-08	1.01E-07	MAFB	protein_cc
ENSG0000(	3461.397	0.076769	0.026446	2.902912	0.003697	0.008091	TIMMDC1	protein_cc
ENSG0000(	3461.163	-0.43676	0.055065	-7.93179	2.16E-15	3.53E-14	RAB30	protein_cc
ENSG0000(	3458.785	0.057342	0.040316	1.422334	0.154929	0.224952	DFNA5	protein_cc
ENSG0000(	3457.726	0.119458	0.043564	2.742108	0.006105	0.012767	PLEKHO1	protein_cc
ENSG0000(	3457.233	0.036213	0.029466	1.228987	0.219077	0.301456	NUP98	protein_cc
ENSG0000(	3454.709	0.270145	0.03534	7.644146	2.10E-14	2.88E-13	RANBP1	protein_cc
ENSG0000(	3454.085	-0.15356	0.031596	-4.85997	1.17E-06	4.69E-06	GOLGA2	protein_cc
ENSG0000(	3452.415	0.004048	0.052797	0.076672	0.938884	0.957345	GTF2I	protein_cc
ENSG0000(	3451.167	-0.0465	0.03878	-1.19908	0.230496	0.314496	SFT2D2	protein_cc
ENSG0000(	3449.506	-0.36815	0.031229	-11.7885	4.47E-32	1.51E-29	SETD3	protein_cc

ENSG0000	3447.027	0.180924	0.038413	4.709925	2.48E-06	9.42E-06	ERCC1	protein_cc
ENSG0000	3445.726	0.048407	0.034832	1.38975	0.164605	0.236802	PIGQ	protein_cc
ENSG0000	3445.644	-0.07206	0.035301	-2.04119	0.041232	0.071269	TECPR1	protein_cc
ENSG0000	3445.517	0.549177	0.046008	11.93649	7.64E-33	3.14E-30	DDX39A	protein_cc
ENSG0000	3445.097	-0.18142	0.032944	-5.50699	3.65E-08	1.86E-07	PEX26	protein_cc
ENSG0000	3445.009	0.042613	0.052231	0.815848	0.414587	0.511581	SH3BP4	protein_cc
ENSG0000	3443.507	0.36336	0.050785	7.154882	8.37E-13	8.81E-12	LAMTOR5	protein_cc
ENSG0000	3442.731	0.11865	0.037609	3.154861	0.001606	0.003772	NUP62	protein_cc
ENSG0000	3442.545	0.012101	0.030397	0.398111	0.690548	0.762775	UGGT1	protein_cc
ENSG0000	3441.749	0.385004	0.05085	7.571317	3.69E-14	4.84E-13	LMNB2	protein_cc
ENSG0000	3441.322	0.420138	0.055668	7.547236	4.45E-14	5.74E-13	TP53	protein_cc
ENSG0000	3441.011	-0.23522	0.093916	-2.50453	0.012261	0.024	CYBB	protein_cc
ENSG0000	3440.502	0.028788	0.025663	1.121778	0.261957	0.350456	MED28	protein_cc
ENSG0000	3440.403	-0.14441	0.031096	-4.64413	3.42E-06	1.27E-05	FAM8A1	protein_cc
ENSG0000	3440.352	-0.1716	0.040057	-4.28388	1.84E-05	6.08E-05	HMOX2	protein_cc
ENSG0000	3439.81	0.005686	0.043646	0.130285	0.896341	0.926415	TMEM181	protein_cc
ENSG0000	3439.641	0.249719	0.055473	4.501623	6.74E-06	2.39E-05	NENF	protein_cc
ENSG0000	3439.511	-0.26759	0.065694	-4.07328	4.64E-05	0.000143	DPYSL4	protein_cc
ENSG0000	3439.258	0.008352	0.063934	0.130629	0.896069	0.926204	INSIG1	protein_cc
ENSG0000	3438.628	0.088698	0.01859	4.771288	1.83E-06	7.10E-06	VPS45	protein_cc
ENSG0000	3437.405	0.299762	0.072673	4.124823	3.71E-05	0.000117	ARHGEF25	protein_cc
ENSG0000	3437.115	-0.05643	0.03449	-1.63607	0.101825	0.156957	ANAPC16	protein_cc
ENSG0000	3436.286	-0.03035	0.045875	-0.66155	0.508257	0.601761	DNAJB5	protein_cc
ENSG0000	3435.504	-0.06744	0.041765	-1.61482	0.10635	0.162894	HDDC2	protein_cc
ENSG0000	3435.006	-0.0651	0.032156	-2.02438	0.042931	0.073846	ACAA1	protein_cc
ENSG0000	3434.995	-0.24566	0.078522	-3.12853	0.001757	0.004097	NUPR1	protein_cc
ENSG0000	3432.928	0.543807	0.055549	9.789679	1.25E-22	6.79E-21	H2AFX	protein_cc
ENSG0000	3432.546	0.881986	0.092034	9.583246	9.40E-22	4.34E-20	CDK6	protein_cc
ENSG0000	3432.438	0.049013	0.036912	1.327817	0.184239	0.260311	ZNF84	protein_cc
ENSG0000	3431.321	-0.00505	0.035209	-0.14348	0.88591	0.918752	DCAF12	protein_cc
ENSG0000	3430.166	0.15208	0.03586	4.240876	2.23E-05	7.26E-05	MRPL24	protein_cc
ENSG0000	3429.59	-0.05658	0.027342	-2.06936	0.038513	0.067116	RP11-735.	pseudoge
ENSG0000	3429.163	-0.18558	0.04043	-4.58999	4.43E-06	1.62E-05	ZBTB38	protein_cc
ENSG0000	3428.548	0.047601	0.038591	1.233478	0.217397	0.299559	PQBP1	protein_cc
ENSG0000	3427.778	-0.08238	0.037755	-2.18187	0.029119	0.052251	KLHL24	protein_cc
ENSG0000	3427.456	0.960225	0.090953	10.55734	4.70E-26	5.04E-24	PSRC1	protein_cc
ENSG0000	3427.065	-0.0394	0.026915	-1.46397	0.143203	0.210197	ITCH	protein_cc
ENSG0000	3424.707	0.005791	0.029087	0.199093	0.84219	0.885378	RANBP9	protein_cc
ENSG0000	3424.672	0.113271	0.021571	5.250962	1.51E-07	6.96E-07	ERAL1	protein_cc
ENSG0000	3424.663	-0.38426	0.035512	-10.8205	2.75E-27	3.69E-25	DDHD2	protein_cc
ENSG0000	3424.15	0.138582	0.036833	3.762414	0.000168	0.000473	TRAPPC1	protein_cc
ENSG0000	3424.141	-0.59538	0.066034	-9.01633	1.95E-19	6.08E-18	GLS	protein_cc
ENSG0000	3423.555	-0.43796	0.072443	-6.04556	1.49E-09	9.43E-09	FRMD5	protein_cc
ENSG0000	3421.587	-0.21824	0.040039	-5.45067	5.02E-08	2.49E-07	RAP1GDS1	protein_cc
ENSG0000	3418.465	-0.02272	0.107042	-0.21228	0.83189	0.877353	ATOH8	protein_cc
ENSG0000	3416.527	0.124479	0.031391	3.965513	7.32E-05	0.000219	STK11	protein_cc
ENSG0000	3416.317	0.075933	0.03958	1.918458	0.055053	0.091935	FAM210B	protein_cc
ENSG0000	3414.747	-0.25317	0.046055	-5.49721	3.86E-08	1.95E-07	BOD1L1	protein_cc
ENSG0000	3414.563	0.557021	0.080857	6.888971	5.62E-12	5.18E-11	TRIP6	protein_cc
ENSG0000	3414.021	-0.16543	0.05308	-3.11665	0.001829	0.00425	PTPRE	protein_cc
ENSG0000	3413.371	0.103479	0.037381	2.768208	0.005637	0.011877	PGPEP1	protein_cc
ENSG0000	3413.158	-0.99811	0.140049	-7.12684	1.03E-12	1.06E-11	SNCB	protein_cc
ENSG0000	3412.835	0.036862	0.058343	0.631821	0.527504	0.619271	REV3L	protein_cc
ENSG0000	3412.797	-0.21392	0.045117	-4.74148	2.12E-06	8.15E-06	KLC1	protein_cc
ENSG0000	3412.112	0.072733	0.041127	1.768508	0.076976	0.123106	VARS2	protein_cc
ENSG0000	3412.065	0.128608	0.036752	3.499373	0.000466	0.001211	LMF2	protein_cc
ENSG0000	3411.021	-0.03646	0.043403	-0.83995	0.400935	0.497746	SLC43A2	protein_cc
ENSG0000	3410.152	0.300621	0.049706	6.047945	1.47E-09	9.30E-09	NCAPD2	protein_cc
ENSG0000	3409.737	0.064908	0.036426	1.781917	0.074763	0.12002	PNRC1	protein_cc

ENSG0000(	3408.547	0.183068	0.040548	4.514807	6.34E-06	2.26E-05	MGAT4B	protein_co
ENSG0000(	3408.484	0.272906	0.062504	4.366203	1.26E-05	4.29E-05	FJX1	protein_co
ENSG0000(	3407.397	-0.401	0.07313	-5.48344	4.17E-08	2.10E-07	SMAD9	protein_co
ENSG0000(	3407.396	-0.03223	0.041303	-0.7803	0.435213	0.532273	IQCE	protein_co
ENSG0000(	3407.283	0.502024	0.054161	9.269029	1.88E-20	6.87E-19	NME1	protein_co
ENSG0000(	3407.2	-0.26983	0.057624	-4.6825	2.83E-06	1.07E-05	ELL2	protein_co
ENSG0000(	3406.272	0.059465	0.040186	1.479745	0.138941	0.204796	AIP	protein_co
ENSG0000(	3406.199	-0.06173	0.04708	-1.31114	0.189812	0.267067	FBXO31	protein_co
ENSG0000(	3405.227	0.319242	0.063196	5.051596	4.38E-07	1.87E-06	TPST1	protein_co
ENSG0000(	3404.49	-0.04887	0.024596	-1.98687	0.046937	0.079864	CDC5L	protein_co
ENSG0000(	3404.313	-0.01574	0.070575	-0.22307	0.823484	0.870654	RNASET2	protein_co
ENSG0000(	3403.384	-0.59576	0.12189	-4.88769	1.02E-06	4.12E-06	GABRB3	protein_co
ENSG0000(	3403.187	-0.15505	0.037047	-4.18526	2.85E-05	9.12E-05	MLXIP	protein_co
ENSG0000(	3402.958	-0.03459	0.029645	-1.16685	0.243272	0.32922	DHX36	protein_co
ENSG0000(	3402.45	-0.04305	0.033926	-1.26902	0.204434	0.284525	VDAC2	protein_co
ENSG0000(	3401.767	-0.23761	0.043537	-5.45781	4.82E-08	2.40E-07	WIPF1	protein_co
ENSG0000(	3401.612	0.159533	0.025436	6.271996	3.56E-10	2.49E-09	MCRS1	protein_co
ENSG0000(	3401.466	-0.31652	0.056529	-5.59933	2.15E-08	1.13E-07	TNRC6C	protein_co
ENSG0000(	3400.954	0.220588	0.036263	6.083024	1.18E-09	7.61E-09	SLC12A9	protein_co
ENSG0000(	3400.626	-0.16532	0.060619	-2.72724	0.006387	0.013306	CREG1	protein_co
ENSG0000(	3399.615	-0.2501	0.05555	-4.50217	6.73E-06	2.39E-05	CRIM1	protein_co
ENSG0000(	3398.489	0.169652	0.027524	6.163871	7.10E-10	4.74E-09	TRA2A	protein_co
ENSG0000(	3397.388	0.470135	0.087802	5.35447	8.58E-08	4.11E-07	MYL12A	protein_co
ENSG0000(	3396.101	-0.08066	0.027083	-2.97838	0.002898	0.006478	LETM1	protein_co
ENSG0000(	3396.012	-0.07464	0.022128	-3.37317	0.000743	0.001854	RAB22A	protein_co
ENSG0000(	3395.106	0.111548	0.030201	3.693562	0.000221	0.000608	FNTA	protein_co
ENSG0000(	3393.363	0.058522	0.05333	1.097351	0.272488	0.362319	TGFBR1	protein_co
ENSG0000(	3392.58	0.212089	0.042052	5.04353	4.57E-07	1.95E-06	NRAS	protein_co
ENSG0000(	3391.314	-0.34077	0.05843	-5.83207	5.47E-09	3.16E-08	TBC1D14	protein_co
ENSG0000(	3391.279	-0.57162	0.059125	-9.66811	4.12E-22	2.03E-20	ANXA11	protein_co
ENSG0000(	3389.507	-0.57506	0.071274	-8.06823	7.13E-16	1.26E-14	EHD3	protein_co
ENSG0000(	3388.762	0.036865	0.035826	1.028988	0.303485	0.396245	ARL1	protein_co
ENSG0000(	3388.066	-0.01559	0.021525	-0.72436	0.468847	0.564692	YY1AP1	protein_co
ENSG0000(	3387.935	-0.12741	0.024708	-5.15652	2.52E-07	1.12E-06	PDXDC1	protein_co
ENSG0000(	3387.743	0.140215	0.102157	1.372539	0.169896	0.243307	HSPA1B	protein_co
ENSG0000(	3387.721	-0.11461	0.028177	-4.06751	4.75E-05	0.000147	PPIG	protein_co
ENSG0000(	3385.825	-0.13461	0.081117	-1.65944	0.097027	0.150491	CTTNBP2	protein_co
ENSG0000(	3384.927	0.138927	0.026443	5.253828	1.49E-07	6.86E-07	ANAPC13	protein_co
ENSG0000(	3384.871	0.022871	0.034245	0.667857	0.504225	0.598054	ZMYND8	protein_co
ENSG0000(	3384.549	-0.62768	0.077723	-8.07582	6.70E-16	1.19E-14	NET1	protein_co
ENSG0000(	3384.287	0.085535	0.037213	2.29852	0.021532	0.039801	NEDD8	protein_co
ENSG0000(	3384.141	0.196243	0.054824	3.57949	0.000344	0.000917	ARRDC3	protein_co
ENSG0000(	3383.339	0.116782	0.127841	0.913496	0.360982	0.456815	HLA-DRB5	protein_co
ENSG0000(	3382.767	-0.1655	0.037371	-4.42854	9.49E-06	3.29E-05	RMND5A	protein_co
ENSG0000(	3382.634	-0.41833	0.052768	-7.9277	2.23E-15	3.64E-14	NR1D2	protein_co
ENSG0000(	3382.064	0.115173	0.031401	3.667789	0.000245	0.000668	SUCO	protein_co
ENSG0000(	3381.918	-0.11967	0.07653	-1.56372	0.117884	0.178015	SEMA3B	processed
ENSG0000(	3380.809	-0.09067	0.024448	-3.70867	0.000208	0.000575	MAX	protein_co
ENSG0000(	3378.717	-0.02658	0.031281	-0.84968	0.395505	0.492066	KLF3	protein_co
ENSG0000(	3378.562	0.349739	0.050276	6.956316	3.49E-12	3.34E-11	TMEM147	protein_co
ENSG0000(	3378.479	-0.19814	0.03799	-5.21567	1.83E-07	8.32E-07	CERK	protein_co
ENSG0000(	3377.038	-0.03506	0.023739	-1.4768	0.139729	0.205765	ABCF3	protein_co
ENSG0000(	3375.75	-0.18611	0.044572	-4.17549	2.97E-05	9.49E-05	PIKFYVE	protein_co
ENSG0000(	3374.123	-0.17097	0.02653	-6.44435	1.16E-10	8.77E-10	ARIH1	protein_co
ENSG0000(	3373.289	0.22095	0.030814	7.170395	7.48E-13	7.93E-12	MPV17	protein_co
ENSG0000(	3372.872	-0.03159	0.038571	-0.81904	0.412766	0.50964	CAT	protein_co
ENSG0000(	3372.599	-0.08433	0.033214	-2.5391	0.011114	0.021977	FOXJ3	protein_co
ENSG0000(	3372.447	-0.19193	0.028916	-6.63758	3.19E-11	2.62E-10	PHF20	protein_co
ENSG0000(	3370.075	-0.40032	0.05045	-7.93497	2.11E-15	3.45E-14	SLC9A3R1	protein_co

ENSG0000(	3368.624	-0.07756	0.03909	-1.98406	0.047249	0.080339	EML3	protein_cc
ENSG0000(	3368.579	-0.09332	0.033366	-2.79677	0.005162	0.010971	RNF41	protein_cc
ENSG0000(	3367.93	-0.13847	0.040098	-3.45337	0.000554	0.001416	URB1	protein_cc
ENSG0000(	3367.579	-0.0448	0.029523	-1.51752	0.129135	0.192314	PIGS	protein_cc
ENSG0000(	3367.487	-0.36971	0.096291	-3.83952	0.000123	0.000355	KCND2	protein_cc
ENSG0000(	3366.788	0.220765	0.041558	5.312216	1.08E-07	5.11E-07	SF3B5	protein_cc
ENSG0000(	3366.209	0.073372	0.022159	3.31113	0.000929	0.002277	FAM134C	protein_cc
ENSG0000(	3363.598	-0.11396	0.037826	-3.01277	0.002589	0.00585	MED12	protein_cc
ENSG0000(	3362.186	-0.13183	0.088832	-1.48405	0.137796	0.203312	MGST1	protein_cc
ENSG0000(	3361.437	-0.07979	0.030314	-2.6321	0.008486	0.017239	KPNA3	protein_cc
ENSG0000(	3361.401	0.168884	0.025637	6.587499	4.47E-11	3.60E-10	DAXX	protein_cc
ENSG0000(	3359.756	0.068477	0.048248	1.419281	0.155817	0.225938	ATL3	protein_cc
ENSG0000(	3359.455	-0.90961	0.098764	-9.21001	3.26E-20	1.15E-18	C1orf95	protein_cc
ENSG0000(	3359.174	0.296222	0.036215	8.179614	2.85E-16	5.37E-15	RPA2	protein_cc
ENSG0000(	3357.493	0.029713	0.027042	1.098747	0.271878	0.361613	CUL4A	protein_cc
ENSG0000(	3356.476	0.12241	0.042853	2.856505	0.004283	0.009258	DIAPH1	protein_cc
ENSG0000(	3356.202	-0.21092	0.053459	-3.94552	7.96E-05	0.000237	TSHZ1	protein_cc
ENSG0000(	3356.187	0.073997	0.029208	2.533415	0.011296	0.0223	ABCE1	protein_cc
ENSG0000(	3355.16	-0.29402	0.03798	-7.74152	9.82E-15	1.43E-13	MTA3	protein_cc
ENSG0000(	3355.157	0.636531	0.070951	8.97147	2.93E-19	8.86E-18	PRDX4	protein_cc
ENSG0000(	3353.148	-0.14867	0.063673	-2.33485	0.019551	0.036471	CORO1A	protein_cc
ENSG0000(	3353.066	0.053225	0.027679	1.92297	0.054484	0.091114	PSMD10	protein_cc
ENSG0000(	3352.202	0.30621	0.044263	6.91796	4.58E-12	4.29E-11	SHFM1	protein_cc
ENSG0000(	3351.455	-0.06646	0.026528	-2.50528	0.012236	0.023956	BROX	protein_cc
ENSG0000(	3350.397	0.021805	0.033116	0.658452	0.510247	0.603492	LINC0009	antisense
ENSG0000(	3349.773	0.363935	0.050195	7.250382	4.16E-13	4.57E-12	PSME2	protein_cc
ENSG0000(	3349.475	-0.08712	0.037449	-2.32638	0.019998	0.037217	ZCRB1	protein_cc
ENSG0000(	3349.313	0.037177	0.037641	0.987688	0.323305	0.417444	PRMT5	protein_cc
ENSG0000(	3348.954	0.187438	0.03099	6.048287	1.46E-09	9.29E-09	COQ9	protein_cc
ENSG0000(	3348.263	-0.0594	0.03399	-1.74747	0.080556	0.128001	HLTF	protein_cc
ENSG0000(	3346.996	-0.03585	0.032269	-1.11096	0.266587	0.355554	KANSL1	protein_cc
ENSG0000(	3346.644	-0.0814	0.040358	-2.01686	0.04371	0.075028	FURIN	protein_cc
ENSG0000(	3346.473	0.046295	0.033076	1.399633	0.161623	0.233034	MAST2	protein_cc
ENSG0000(	3346.091	-0.14377	0.111355	-1.29107	0.19668	0.275298	NEU4	protein_cc
ENSG0000(	3344.833	0.019409	0.025803	0.752198	0.451932	0.548529	FBXW2	protein_cc
ENSG0000(	3344.528	-0.42781	0.047901	-8.93115	4.22E-19	1.24E-17	MBNL2	protein_cc
ENSG0000(	3342.188	-0.2473	0.035628	-6.9412	3.89E-12	3.69E-11	HIPK3	protein_cc
ENSG0000(	3340.841	0.015179	0.029342	0.517324	0.60493	0.689155	GUCD1	protein_cc
ENSG0000(	3340.545	0.063923	0.041831	1.528107	0.126486	0.189046	DNASE2	protein_cc
ENSG0000(	3340.037	-0.13568	0.023807	-5.69912	1.20E-08	6.60E-08	FAM53C	protein_cc
ENSG0000(	3338.53	0.155197	0.052437	2.959676	0.00308	0.006847	C1orf122	protein_cc
ENSG0000(	3338.464	-0.09978	0.034889	-2.85984	0.004239	0.009171	PPP1R7	protein_cc
ENSG0000(	3338.37	0.26952	0.034787	7.747674	9.36E-15	1.37E-13	MRPS7	protein_cc
ENSG0000(	3338.313	0.221219	0.026219	8.437366	3.25E-17	7.15E-16	PMPCB	protein_cc
ENSG0000(	3337.908	-0.12879	0.029737	-4.33095	1.48E-05	4.98E-05	FMR1	protein_cc
ENSG0000(	3334.484	-0.60933	0.078684	-7.74403	9.63E-15	1.41E-13	LRP1B	protein_cc
ENSG0000(	3331.997	0.016059	0.079521	0.201949	0.839957	0.883688	ARPC1B	protein_cc
ENSG0000(	3331.675	-0.21891	0.076383	-2.86591	0.004158	0.009016	CD4	protein_cc
ENSG0000(	3330.677	-0.15876	0.023375	-6.79196	1.11E-11	9.73E-11	ZDHHC3	protein_cc
ENSG0000(	3329.903	0.293558	0.110436	2.658165	0.007857	0.016075	TMEM176	protein_cc
ENSG0000(	3329.331	-0.1482	0.036894	-4.01703	5.89E-05	0.000179	AVL9	protein_cc
ENSG0000(	3327.045	0.000152	0.018214	0.008328	0.993355	0.995631	TOR1AIP2	protein_cc
ENSG0000(	3326.085	-0.17131	0.046836	-3.65756	0.000255	0.000693	UBXN7	protein_cc
ENSG0000(	3325.33	0.074795	0.026377	2.835603	0.004574	0.009817	HIATL1	protein_cc
ENSG0000(	3323.168	0.038151	0.034463	1.107034	0.268279	0.357485	PARP6	protein_cc
ENSG0000(	3322.674	0.219495	0.027706	7.922272	2.33E-15	3.79E-14	SMARCE1	protein_cc
ENSG0000(	3321.926	-0.19248	0.037206	-5.17332	2.30E-07	1.03E-06	EXD2	protein_cc
ENSG0000(	3321.876	0.409046	0.090351	4.527318	5.97E-06	2.14E-05	SOX11	protein_cc
ENSG0000(	3320.015	-0.04841	0.032529	-1.48812	0.136719	0.201934	NBN	protein_cc

ENSG0000(	3319.984	0.029132	0.04519	0.644667	0.519143	0.611761	TFDP2	protein_cc
ENSG0000(	3319.685	-0.25555	0.037416	-6.82996	8.49E-12	7.62E-11	ZYG11B	protein_cc
ENSG0000(	3318.11	-0.14311	0.083788	-1.70803	0.08763	0.137804	FOSL2	protein_cc
ENSG0000(	3317.97	-0.60606	0.047448	-12.7732	2.31E-37	2.27E-34	ETS2	protein_cc
ENSG0000(	3317.824	0.191334	0.039058	4.898754	9.64E-07	3.91E-06	TOMM22	protein_cc
ENSG0000(	3317.686	-0.00514	0.023637	-0.21724	0.82802	0.874057	STAMBP	protein_cc
ENSG0000(	3314.956	-0.20813	0.039378	-5.28543	1.25E-07	5.85E-07	SOS2	protein_cc
ENSG0000(	3314.362	0.091667	0.038816	2.361561	0.018198	0.034207	EXOSC10	protein_cc
ENSG0000(	3313.852	0.101735	0.03047	3.338832	0.000841	0.002079	CNOT2	protein_cc
ENSG0000(	3313.744	-0.12686	0.0351	-3.6144	0.000301	0.00081	CDC123	protein_cc
ENSG0000(	3312.916	-0.61382	0.094885	-6.46911	9.86E-11	7.52E-10	DDR2	protein_cc
ENSG0000(	3311.48	-0.43813	0.062595	-6.99938	2.57E-12	2.51E-11	MFSD6	protein_cc
ENSG0000(	3311.374	0.126268	0.03705	3.408049	0.000654	0.001651	MAZ	protein_cc
ENSG0000(	3311.044	-0.08644	0.07649	-1.13008	0.258444	0.346456	DHRS3	protein_cc
ENSG0000(	3310.152	-0.03151	0.040449	-0.77899	0.435988	0.533015	SMCHD1	protein_cc
ENSG0000(	3310.033	-0.4147	0.107859	-3.84479	0.000121	0.000348	GAD1	protein_cc
ENSG0000(	3308.298	0.20727	0.035955	5.764632	8.18E-09	4.60E-08	NCLN	protein_cc
ENSG0000(	3307.74	-0.07358	0.028089	-2.61944	0.008807	0.017816	TERF2	protein_cc
ENSG0000(	3307.348	-0.214	0.039729	-5.38648	7.18E-08	3.48E-07	TAPT1	protein_cc
ENSG0000(	3305.965	-0.06143	0.06499	-0.9453	0.344508	0.439822	TIPARP	protein_cc
ENSG0000(	3305.688	0.034624	0.025882	1.337788	0.180966	0.256413	STARD3	protein_cc
ENSG0000(	3303.971	0.220576	0.12401	1.778699	0.075289	0.120752	TPPP3	protein_cc
ENSG0000(	3302.194	-0.01993	0.030836	-0.64642	0.518009	0.610687	PCF11	protein_cc
ENSG0000(	3301.488	0.300943	0.047815	6.29389	3.10E-10	2.19E-09	GADD45G	protein_cc
ENSG0000(	3299.056	0.090066	0.028694	3.138857	0.001696	0.003966	INTS10	protein_cc
ENSG0000(	3298.817	-0.25629	0.072798	-3.52063	0.000431	0.001126	SLITRK2	protein_cc
ENSG0000(	3298.467	-0.83936	0.110687	-7.5832	3.37E-14	4.44E-13	SLCO1A2	protein_cc
ENSG0000(	3297.581	0.29923	0.039597	7.556972	4.13E-14	5.36E-13	NUP205	protein_cc
ENSG0000(	3297.572	-0.02932	0.034747	-0.84382	0.398767	0.495465	SUV420H1	protein_cc
ENSG0000(	3295.463	0.108448	0.037393	2.900232	0.003729	0.008154	RNPEPL1	protein_cc
ENSG0000(	3293.831	0.002928	0.037485	0.078109	0.937741	0.956559	CCM2	protein_cc
ENSG0000(	3293.629	-0.39532	0.047944	-8.24536	1.65E-16	3.23E-15	GDF11	protein_cc
ENSG0000(	3293.581	0.070657	0.023356	3.02528	0.002484	0.005634	7-Mar	protein_cc
ENSG0000(	3291.581	0.725059	0.100137	7.240669	4.46E-13	4.88E-12	BST2	protein_cc
ENSG0000(	3291.235	-0.40589	0.075696	-5.36215	8.22E-08	3.95E-07	MYH14	protein_cc
ENSG0000(	3290.199	-0.10819	0.046976	-2.30309	0.021274	0.03936	SC5D	protein_cc
ENSG0000(	3286.524	-0.24441	0.047572	-5.13769	2.78E-07	1.23E-06	ANKRD46	protein_cc
ENSG0000(	3285.875	-0.11486	0.034527	-3.32676	0.000879	0.002164	TAB1	protein_cc
ENSG0000(	3284.684	0.072593	0.042555	1.705856	0.088035	0.138387	IRF2BPL	protein_cc
ENSG0000(	3284.159	-1.36109	0.104505	-13.0243	8.91E-39	1.05E-35	RIMS3	protein_cc
ENSG0000(	3281.235	-0.15898	0.031927	-4.97942	6.38E-07	2.66E-06	TRIM41	protein_cc
ENSG0000(	3281.219	0.003941	0.031578	0.124804	0.900679	0.929486	C11orf68	protein_cc
ENSG0000(	3280.879	0.052546	0.047783	1.09969	0.271467	0.361137	CIB1	protein_cc
ENSG0000(	3280.067	-0.1704	0.037432	-4.55233	5.31E-06	1.91E-05	USP20	protein_cc
ENSG0000(	3278.47	0.098063	0.026711	3.6713	0.000241	0.00066	PHF12	protein_cc
ENSG0000(	3278.283	0.238392	0.051989	4.585452	4.53E-06	1.65E-05	HS2ST1	protein_cc
ENSG0000(	3278.128	-0.12472	0.032529	-3.83419	0.000126	0.000362	EPM2AIP1	protein_cc
ENSG0000(	3278.037	-0.01196	0.039853	-0.30015	0.764059	0.822953	GSTA4	protein_cc
ENSG0000(	3277.088	0.142	0.042707	3.324983	0.000884	0.002176	TM7SF3	protein_cc
ENSG0000(	3276.648	0.349399	0.041064	8.508657	1.76E-17	4.05E-16	SRPRB	protein_cc
ENSG0000(	3275.577	-0.12917	0.029806	-4.33365	1.47E-05	4.93E-05	EIF1B	protein_cc
ENSG0000(	3275.262	0.049117	0.026788	1.833583	0.066716	0.108654	USP4	protein_cc
ENSG0000(	3275.103	0.200981	0.026193	7.673062	1.68E-14	2.33E-13	GLT8D1	protein_cc
ENSG0000(	3274.778	-0.03253	0.035047	-0.92809	0.35336	0.448908	TRAPPC12	protein_cc
ENSG0000(	3274.319	-0.1018	0.03068	-3.3181	0.000906	0.002226	HGSNAT	protein_cc
ENSG0000(	3274.146	0.095027	0.019032	4.992936	5.95E-07	2.49E-06	ERCC3	protein_cc
ENSG0000(	3272.578	0.100801	0.052645	1.914735	0.055526	0.092617	SLC27A1	protein_cc
ENSG0000(	3271.842	0.240331	0.154348	1.557071	0.119454	0.180001	VGF	protein_cc
ENSG0000(	3270.854	-0.21477	0.04725	-4.5454	5.48E-06	1.97E-05	BRD3	protein_cc

ENSG0000(	3269.111	-0.10146	0.024076	-4.2142	2.51E-05	8.09E-05	UBP1	protein_cc
ENSG0000(	3265.984	0.309122	0.059357	5.207894	1.91E-07	8.65E-07	AP2S1	protein_cc
ENSG0000(	3264.6	-0.36146	0.031713	-11.3976	4.30E-30	9.71E-28	VPS53	protein_cc
ENSG0000(	3264.287	-0.28328	0.043515	-6.51006	7.51E-11	5.84E-10	PCNX	protein_cc
ENSG0000(	3261.916	-0.03908	0.034486	-1.1333	0.257086	0.344951	GPBP1L1	protein_cc
ENSG0000(	3261.85	-0.02481	0.030313	-0.81841	0.41312	0.509986	RNF24	protein_cc
ENSG0000(	3261.576	0.08075	0.031606	2.554878	0.010623	0.021097	KIAA1715	protein_cc
ENSG0000(	3261.329	-0.37768	0.052019	-7.26041	3.86E-13	4.27E-12	DHTKD1	protein_cc
ENSG0000(	3260.946	-0.04392	0.033953	-1.29357	0.195814	0.27431	GALNT11	protein_cc
ENSG0000(	3260.451	-0.23599	0.042495	-5.55331	2.80E-08	1.45E-07	NOVA2	protein_cc
ENSG0000(	3259.564	0.04947	0.025804	1.91715	0.055219	0.092175	UBAC1	protein_cc
ENSG0000(	3259.008	-0.48104	0.075132	-6.40258	1.53E-10	1.13E-09	FAT3	protein_cc
ENSG0000(	3258.267	-0.11518	0.024799	-4.64449	3.41E-06	1.27E-05	GAPVD1	protein_cc
ENSG0000(	3258.24	-0.2143	0.032877	-6.51818	7.12E-11	5.55E-10	TOM1	protein_cc
ENSG0000(	3257.237	-0.05628	0.025559	-2.202	0.027665	0.049862	ATF2	protein_cc
ENSG0000(	3256.93	-0.29209	0.032952	-8.86413	7.71E-19	2.18E-17	MMS19	protein_cc
ENSG0000(	3256.014	-0.01459	0.03126	-0.46671	0.640705	0.720629	FTSJ3	protein_cc
ENSG0000(	3255.91	0.264031	0.035013	7.541051	4.66E-14	6.00E-13	GNPDA1	protein_cc
ENSG0000(	3255.867	-0.04445	0.035963	-1.23588	0.216505	0.298576	CFDP1	protein_cc
ENSG0000(	3255.819	-0.04869	0.046688	-1.04286	0.297011	0.389127	AUTS2	protein_cc
ENSG0000(	3255.804	-0.18613	0.030732	-6.05637	1.39E-09	8.88E-09	RPTOR	protein_cc
ENSG0000(	3254.93	0.012361	0.037157	0.332674	0.739381	0.802997	MAT2B	protein_cc
ENSG0000(	3254.591	0.248531	0.066428	3.741379	0.000183	0.000511	ILDR2	protein_cc
ENSG0000(	3254.566	-0.28644	0.037868	-7.56435	3.90E-14	5.09E-13	SESTD1	protein_cc
ENSG0000(	3253.683	-0.5953	0.05989	-9.93978	2.79E-23	1.75E-21	CHST15	protein_cc
ENSG0000(	3253.584	0.328559	0.040508	8.111011	5.02E-16	9.10E-15	TMEM50B	protein_cc
ENSG0000(	3253.236	-0.13591	0.032608	-4.16788	3.07E-05	9.80E-05	SUGT1	protein_cc
ENSG0000(	3253.17	-0.00308	0.099456	-0.031	0.97527	0.982651	MAN1C1	protein_cc
ENSG0000(	3252.567	0.198775	0.057529	3.455211	0.00055	0.001407	SLC38A3	processed
ENSG0000(	3251.394	0.219005	0.041606	5.263843	1.41E-07	6.52E-07	ATP5G1	protein_cc
ENSG0000(	3250.19	0.037308	0.035222	1.059217	0.289501	0.380889	ETNK1	protein_cc
ENSG0000(	3248.387	-0.01531	0.049784	-0.30748	0.758477	0.818398	SPEG	protein_cc
ENSG0000(	3247.422	-0.12336	0.094485	-1.30556	0.191703	0.26934	IFITM10	protein_cc
ENSG0000(	3246.508	-0.75436	0.072099	-10.4629	1.28E-25	1.28E-23	LINC0064:	processed
ENSG0000(	3246.006	0.079789	0.032339	2.467258	0.013615	0.026386	C12orf23	protein_cc
ENSG0000(	3243.105	-0.14294	0.032519	-4.39541	1.11E-05	3.79E-05	DNAJC18	protein_cc
ENSG0000(	3242.98	0.092961	0.021834	4.257525	2.07E-05	6.79E-05	DNAJA3	protein_cc
ENSG0000(	3242.364	0.133448	0.026913	4.958575	7.10E-07	2.94E-06	ACP1	protein_cc
ENSG0000(	3242.11	0.18485	0.066801	2.767194	0.005654	0.011911	DCHS1	protein_cc
ENSG0000(	3240.379	0.370517	0.059671	6.209309	5.32E-10	3.61E-09	IFNGR2	protein_cc
ENSG0000(	3239.941	0.304571	0.034936	8.717927	2.83E-18	7.37E-17	NUBP2	protein_cc
ENSG0000(	3239.808	-0.18373	0.0336	-5.46815	4.55E-08	2.28E-07	RNF146	protein_cc
ENSG0000(	3239.109	0.08967	0.03384	2.649836	0.008053	0.01644	RNF5	protein_cc
ENSG0000(	3238.984	0.001496	0.032737	0.045687	0.96356	0.975107	DDX54	protein_cc
ENSG0000(	3238.883	0.073409	0.03607	2.035157	0.041835	0.072199	ANAPC2	protein_cc
ENSG0000(	3238.633	0.306952	0.050284	6.104308	1.03E-09	6.72E-09	BRI3	protein_cc
ENSG0000(	3236.573	-0.06658	0.048512	-1.37237	0.169947	0.243346	PTPRJ	protein_cc
ENSG0000(	3236.085	0.122146	0.029814	4.096989	4.19E-05	0.00013	SKIV2L	protein_cc
ENSG0000(	3236.037	0.027024	0.030127	0.897	0.369719	0.466047	HMGN3	protein_cc
ENSG0000(	3235.051	-0.31731	0.046748	-6.78768	1.14E-11	1.00E-10	ATAD1	protein_cc
ENSG0000(	3234.62	-0.01451	0.040164	-0.36133	0.717853	0.785112	ZNF512B	protein_cc
ENSG0000(	3234.488	-0.42441	0.080064	-5.30097	1.15E-07	5.41E-07	NIPAL3	protein_cc
ENSG0000(	3234.378	0.081858	0.038754	2.112249	0.034665	0.061091	SMPD1	protein_cc
ENSG0000(	3234.35	-0.21439	0.035951	-5.96323	2.47E-09	1.51E-08	MKL1	protein_cc
ENSG0000(	3232.877	0.122703	0.032182	3.812829	0.000137	0.000392	SNX13	protein_cc
ENSG0000(	3232.794	-0.11528	0.061638	-1.8702	0.061456	0.101121	ASAP3	protein_cc
ENSG0000(	3232.616	0.214807	0.041594	5.164374	2.41E-07	1.08E-06	PSMA5	protein_cc
ENSG0000(	3231.661	0.032217	0.03741	0.861193	0.389132	0.485797	IGBP1	protein_cc
ENSG0000(	3231.436	-0.1162	0.023642	-4.91519	8.87E-07	3.61E-06	KPNA1	protein_cc

ENSG0000(	3230.581	-0.09217	0.044565	-2.0682	0.038621	0.067276	PGAM1	protein_cc
ENSG0000(	3229.329	0.323555	0.096803	3.342392	0.000831	0.002055	TREM2	protein_cc
ENSG0000(	3227.141	-0.0526	0.034157	-1.53986	0.123594	0.18531	AKAP8L	protein_cc
ENSG0000(	3226.944	-0.33316	0.040849	-8.15589	3.47E-16	6.45E-15	UBR3	protein_cc
ENSG0000(	3226.221	-0.17103	0.047547	-3.59704	0.000322	0.000861	ATP11A	protein_cc
ENSG0000(	3226.087	-0.46396	0.04839	-9.58795	8.99E-22	4.15E-20	BAIAP2-A	lincRNA
ENSG0000(	3225.474	-0.02176	0.028395	-0.76627	0.443518	0.54031	USP14	protein_cc
ENSG0000(	3225.43	-0.10499	0.085908	-1.22209	0.221674	0.304429	HCLS1	protein_cc
ENSG0000(	3224.58	0.094503	0.033294	2.838437	0.004534	0.009738	DTD1	protein_cc
ENSG0000(	3220.777	-0.07124	0.03354	-2.1241	0.033661	0.059473	GPD2	protein_cc
ENSG0000(	3220.166	0.056875	0.029954	1.898759	0.057596	0.095635	COPG2	protein_cc
ENSG0000(	3219.645	0.018842	0.026847	0.701828	0.482786	0.577826	URGCP	protein_cc
ENSG0000(	3219.274	0.186674	0.043569	4.284558	1.83E-05	6.07E-05	UQCRFS1	protein_cc
ENSG0000(	3219.209	-0.13063	0.075944	-1.72013	0.085409	0.134687	PLXNA4	protein_cc
ENSG0000(	3217.53	0.033064	0.019796	1.670245	0.094871	0.147587	ANKLE2	protein_cc
ENSG0000(	3216.985	-0.18585	0.028146	-6.60304	4.03E-11	3.26E-10	SIDT2	protein_cc
ENSG0000(	3216.975	0.009743	0.064636	0.150734	0.880186	0.914336	SPATA20	protein_cc
ENSG0000(	3215.23	0.001257	0.019272	0.065243	0.947981	0.964043	WDR33	protein_cc
ENSG0000(	3213.084	-0.09224	0.026142	-3.52832	0.000418	0.001096	GMPR2	protein_cc
ENSG0000(	3212.016	-0.23236	0.042218	-5.50372	3.72E-08	1.89E-07	TMCC1	protein_cc
ENSG0000(	3211.299	-0.03592	0.055806	-0.64371	0.519762	0.612262	PIEZO1	protein_cc
ENSG0000(	3210.718	-0.1245	0.033629	-3.7023	0.000214	0.000589	SOS1	protein_cc
ENSG0000(	3210.549	0.133464	0.035026	3.810384	0.000139	0.000395	XPO5	protein_cc
ENSG0000(	3210.086	-0.31963	0.036233	-8.8216	1.13E-18	3.11E-17	GNPTAB	protein_cc
ENSG0000(	3208.952	-0.19108	0.029894	-6.39185	1.64E-10	1.20E-09	CPSF2	protein_cc
ENSG0000(	3208.524	0.143489	0.03814	3.762214	0.000168	0.000473	PCED1A	protein_cc
ENSG0000(	3208.208	-0.00897	0.038718	-0.23176	0.816724	0.865554	ICMT	protein_cc
ENSG0000(	3206.618	-0.87112	0.081731	-10.6583	1.59E-26	1.90E-24	KIAA1549l	protein_cc
ENSG0000(	3206.416	0.156686	0.036774	4.260815	2.04E-05	6.69E-05	CRLS1	protein_cc
ENSG0000(	3206.322	0.108609	0.03667	2.961764	0.003059	0.006805	NCAPH2	protein_cc
ENSG0000(	3205.726	-0.8261	0.072181	-11.4449	2.50E-30	6.14E-28	INPP5F	protein_cc
ENSG0000(	3204.98	0.075842	0.032647	2.323093	0.020174	0.037518	CREB3	protein_cc
ENSG0000(	3204.852	0.109834	0.059448	1.84758	0.064663	0.105734	TRIM56	protein_cc
ENSG0000(	3204.834	0.030067	0.029835	1.007779	0.31356	0.406983	MEAF6	protein_cc
ENSG0000(	3202.743	0.035775	0.02738	1.306602	0.191348	0.268906	SLC35E1	protein_cc
ENSG0000(	3201.577	0.289652	0.105912	2.734841	0.006241	0.013031	ITGA3	protein_cc
ENSG0000(	3201.085	0.109049	0.047346	2.303225	0.021266	0.03935	FZD3	protein_cc
ENSG0000(	3200.611	0.025702	0.038798	0.662475	0.507667	0.601235	CD46	protein_cc
ENSG0000(	3200.083	0.116964	0.037555	3.114484	0.001843	0.004279	DAPK3	protein_cc
ENSG0000(	3199.916	-0.50578	0.06438	-7.85629	3.96E-15	6.19E-14	HDAC11	protein_cc
ENSG0000(	3199.342	-0.41963	0.126523	-3.31664	0.000911	0.002236	CRTAC1	protein_cc
ENSG0000(	3198.939	0.146219	0.065967	2.216552	0.026654	0.04826	CDO1	protein_cc
ENSG0000(	3198.691	-0.12678	0.048023	-2.63994	0.008292	0.016881	ADCK3	protein_cc
ENSG0000(	3198.569	0.08904	0.05846	1.523099	0.127734	0.190636	TLE1	protein_cc
ENSG0000(	3198.325	-0.00256	0.032229	-0.0793	0.936791	0.955863	NTPCR	protein_cc
ENSG0000(	3197.155	-0.20941	0.043313	-4.83481	1.33E-06	5.28E-06	TUBG2	protein_cc
ENSG0000(	3194.722	-0.30245	0.035224	-8.58653	8.96E-18	2.17E-16	EXOC6B	protein_cc
ENSG0000(	3194.258	0.208269	0.043505	4.787247	1.69E-06	6.59E-06	TCTA	protein_cc
ENSG0000(	3191.938	0.070171	0.071122	0.986634	0.323822	0.417967	KANK2	protein_cc
ENSG0000(	3191.781	-0.23036	0.040674	-5.6636	1.48E-08	8.00E-08	CEP68	protein_cc
ENSG0000(	3191.61	-0.72921	0.099696	-7.3143	2.59E-13	2.94E-12	CD38	protein_cc
ENSG0000(	3191.223	-0.85601	0.079377	-10.7841	4.09E-27	5.37E-25	ABLIM3	protein_cc
ENSG0000(	3191.012	0.06809	0.034647	1.96524	0.049387	0.083579	NSA2	protein_cc
ENSG0000(	3190.627	-0.25124	0.053523	-4.69402	2.68E-06	1.01E-05	AKAP9	protein_cc
ENSG0000(	3190.516	-0.85416	0.147299	-5.79885	6.68E-09	3.81E-08	CHGA	protein_cc
ENSG0000(	3189.686	0.476179	0.057075	8.343036	7.24E-17	1.51E-15	PPP1R14B	protein_cc
ENSG0000(	3189.565	0.464865	0.12982	3.580843	0.000342	0.000912	ISG15	protein_cc
ENSG0000(	3188.728	-0.44783	0.047413	-9.44547	3.54E-21	1.48E-19	EFR3A	protein_cc
ENSG0000(	3188.606	0.161429	0.05872	2.749147	0.005975	0.012519	SNHG1	processed

ENSG0000(	3188.137	0.145508	0.028758	5.059812	4.20E-07	1.80E-06	PRKAG1	protein_cc
ENSG0000(	3187.098	0.093715	0.027469	3.411661	0.000646	0.001631	CWC15	protein_cc
ENSG0000(	3186.688	-0.37407	0.040008	-9.3499	8.77E-21	3.41E-19	MEF2A	protein_cc
ENSG0000(	3186.161	-0.01927	0.029206	-0.6599	0.509316	0.602702	MESDC2	protein_cc
ENSG0000(	3185.461	1.143181	0.13105	8.723212	2.70E-18	7.05E-17	TOP2A	protein_cc
ENSG0000(	3183.03	0.089483	0.035323	2.533272	0.0113	0.022308	SLC35A5	protein_cc
ENSG0000(	3182.396	0.110587	0.063449	1.742929	0.081346	0.129052	SEMA6D	protein_cc
ENSG0000(	3180.628	-0.24705	0.039093	-6.31962	2.62E-10	1.87E-09	HELZ	protein_cc
ENSG0000(	3180.498	-0.1065	0.039381	-2.70427	0.006846	0.014176	FAM120B	protein_cc
ENSG0000(	3180.323	0.113458	0.034837	3.256866	0.001126	0.002719	YTHDF2	protein_cc
ENSG0000(	3180.054	-0.17554	0.038664	-4.54007	5.62E-06	2.02E-05	ROCK2	protein_cc
ENSG0000(	3180.009	-0.17241	0.1085	-1.58903	0.112053	0.170423	MIAT	lincRNA
ENSG0000(	3179.401	0.753292	0.103496	7.278446	3.38E-13	3.77E-12	C1QTNF1	protein_cc
ENSG0000(	3179.036	-0.05565	0.045073	-1.23471	0.216939	0.299034	KCTD15	protein_cc
ENSG0000(	3179.016	0.030964	0.021252	1.457016	0.145112	0.212619	CHTF8	protein_cc
ENSG0000(	3177.875	0.169049	0.029179	5.793573	6.89E-09	3.92E-08	ZNF655	protein_cc
ENSG0000(	3177.486	0.023051	0.030492	0.755951	0.449678	0.546536	TROVE2	protein_cc
ENSG0000(	3177.142	0.064515	0.0328	1.966914	0.049193	0.0833	TTL	protein_cc
ENSG0000(	3177.081	-0.10092	0.028502	-3.54101	0.000399	0.001049	MRPS16	protein_cc
ENSG0000(	3176.546	0.084307	0.083728	1.006919	0.313974	0.407389	SDC2	protein_cc
ENSG0000(	3175.813	0.375	0.064234	5.838058	5.28E-09	3.06E-08	SMS	protein_cc
ENSG0000(	3175.422	-0.31328	0.034573	-9.06124	1.29E-19	4.14E-18	HINT3	protein_cc
ENSG0000(	3174.306	0.146171	0.037611	3.886434	0.000102	0.000297	NSMCE1	protein_cc
ENSG0000(	3174.253	-0.0322	0.022594	-1.42517	0.154109	0.223975	VAC14	protein_cc
ENSG0000(	3174.013	-0.22727	0.059852	-3.79725	0.000146	0.000415	RNF144A	protein_cc
ENSG0000(	3172.424	0.107231	0.0905	1.184879	0.236065	0.320903	VWA1	protein_cc
ENSG0000(	3172.338	-0.39555	0.050828	-7.78219	7.13E-15	1.07E-13	DMXL2	protein_cc
ENSG0000(	3172.229	0.249474	0.041032	6.080018	1.20E-09	7.74E-09	TWF1	protein_cc
ENSG0000(	3170.706	-0.14131	0.035266	-4.00695	6.15E-05	0.000187	SLC16A2	protein_cc
ENSG0000(	3169.467	0.06324	0.030171	2.096068	0.036076	0.063331	MRPS18B	protein_cc
ENSG0000(	3168.754	0.636839	0.087651	7.265578	3.71E-13	4.12E-12	FOXG1	protein_cc
ENSG0000(	3167.782	0.288735	0.033584	8.597353	8.16E-18	1.98E-16	DDX49	protein_cc
ENSG0000(	3167.671	0.143609	0.035748	4.017286	5.89E-05	0.000179	B3GAT3	protein_cc
ENSG0000(	3167.426	0.036259	0.028409	1.276321	0.201842	0.281489	EXOC1	protein_cc
ENSG0000(	3166.75	-0.8193	0.093264	-8.78474	1.57E-18	4.23E-17	PAK1	protein_cc
ENSG0000(	3165.654	0.039197	0.029263	1.339471	0.180417	0.255734	DERL1	protein_cc
ENSG0000(	3163.334	0.017423	0.025024	0.696279	0.486254	0.581147	ZC3H15	protein_cc
ENSG0000(	3162.898	-0.19507	0.094878	-2.05595	0.039787	0.069056	RHBDL3	protein_cc
ENSG0000(	3161.798	0.333192	0.059514	5.598499	2.16E-08	1.14E-07	GLDC	protein_cc
ENSG0000(	3161.718	-0.05546	0.091171	-0.60835	0.542957	0.633686	HSPA1A	protein_cc
ENSG0000(	3160.342	-0.00136	0.027886	-0.04885	0.961042	0.973362	TTC17	protein_cc
ENSG0000(	3160.166	-0.22295	0.091286	-2.44234	0.014592	0.028106	FAM222A	protein_cc
ENSG0000(	3159.87	-0.12431	0.029892	-4.15881	3.20E-05	0.000102	TK2	protein_cc
ENSG0000(	3159.731	-0.01132	0.02749	-0.41195	0.680375	0.753984	NAT10	protein_cc
ENSG0000(	3159.493	-0.24171	0.037723	-6.40763	1.48E-10	1.09E-09	ULK1	protein_cc
ENSG0000(	3159.308	0.37039	0.035775	10.35338	4.04E-25	3.64E-23	TBRG4	protein_cc
ENSG0000(	3158.63	0.091041	0.024353	3.738472	0.000185	0.000516	COPS5	protein_cc
ENSG0000(	3158.467	-0.00284	0.027079	-0.10479	0.91654	0.941166	CRTC3	protein_cc
ENSG0000(	3158.174	-0.77528	0.072819	-10.6467	1.81E-26	2.14E-24	CBX7	protein_cc
ENSG0000(	3157.685	0.07566	0.043011	1.7591	0.078561	0.12529	MFSD1	protein_cc
ENSG0000(	3156.074	0.27457	0.038837	7.069778	1.55E-12	1.57E-11	TIMM13	protein_cc
ENSG0000(	3155.715	0.384417	0.037255	10.31855	5.81E-25	5.04E-23	SSBP1	protein_cc
ENSG0000(	3155.703	-0.12779	0.048314	-2.64497	0.00817	0.016656	MAP6	protein_cc
ENSG0000(	3154.508	-0.0406	0.026148	-1.5527	0.120494	0.181316	FKBP15	protein_cc
ENSG0000(	3153.81	-0.03373	0.028502	-1.18335	0.236671	0.32164	RBM26	protein_cc
ENSG0000(	3153.694	-0.2002	0.07124	-2.81026	0.00495	0.01056	BCL7A	protein_cc
ENSG0000(	3152.743	0.1372	0.04301	3.189964	0.001423	0.003374	ECSIT	protein_cc
ENSG0000(	3152.311	-0.08358	0.041458	-2.0161	0.04379	0.075156	AGO2	protein_cc
ENSG0000(	3151.406	-0.23625	0.03782	-6.24659	4.20E-10	2.90E-09	MAN1A2	protein_cc

ENSG0000(	3150.095	-0.13268	0.039061	-3.39675	0.000682	0.001714	JAKMIP2	protein_cc
ENSG0000(	3150.039	-0.00325	0.038974	-0.08343	0.933506	0.953681	CAD	protein_cc
ENSG0000(	3149.951	0.349595	0.121603	2.874898	0.004042	0.008783	SLC6A11	protein_cc
ENSG0000(	3145.262	-0.02761	0.030107	-0.91702	0.359132	0.454919	COPS2	protein_cc
ENSG0000(	3143.948	-0.16847	0.038578	-4.36698	1.26E-05	4.28E-05	PTPLB	protein_cc
ENSG0000(	3142.258	-0.12345	0.035844	-3.44411	0.000573	0.001462	MAP1S	protein_cc
ENSG0000(	3141.416	0.040869	0.037126	1.100819	0.270975	0.360541	DHRS7	protein_cc
ENSG0000(	3140.506	0.04799	0.021688	2.212803	0.026911	0.048646	GNPAT	protein_cc
ENSG0000(	3139.162	0.32114	0.083511	3.845489	0.00012	0.000347	AK4	protein_cc
ENSG0000(	3137.161	0.520614	0.068036	7.652002	1.98E-14	2.72E-13	SPRED2	protein_cc
ENSG0000(	3135.831	0.157743	0.040769	3.869155	0.000109	0.000317	MRPL20	protein_cc
ENSG0000(	3134.561	-0.2171	0.05559	-3.90546	9.40E-05	0.000276	GSTM2	protein_cc
ENSG0000(	3133.421	0.208028	0.053336	3.900294	9.61E-05	0.000282	PGAP1	protein_cc
ENSG0000(	3132.345	0.056869	0.02561	2.220589	0.026379	0.047816	UBA3	protein_cc
ENSG0000(	3131.496	-0.00629	0.019533	-0.32225	0.747261	0.80959	SUPT7L	protein_cc
ENSG0000(	3130.779	0.011066	0.040353	0.274241	0.7839	0.839093	MEX3C	protein_cc
ENSG0000(	3130.758	0.016981	0.036908	0.460098	0.645446	0.72489	MAPK6	protein_cc
ENSG0000(	3130.688	-0.62059	0.051841	-11.9711	5.04E-33	2.23E-30	AAK1	protein_cc
ENSG0000(	3130.197	0.110384	0.030957	3.56575	0.000363	0.000962	VKORC1L1	protein_cc
ENSG0000(	3128.396	-0.05165	0.036479	-1.41593	0.156795	0.22718	TMEM167	protein_cc
ENSG0000(	3127.927	0.061443	0.034846	1.763266	0.077856	0.124359	SNX6	protein_cc
ENSG0000(	3127.914	-0.05928	0.037693	-1.5726	0.115811	0.175393	SGSM3	protein_cc
ENSG0000(	3127.202	0.045638	0.029516	1.546218	0.122052	0.183319	ARF6	protein_cc
ENSG0000(	3126.496	-0.17608	0.03271	-5.38311	7.32E-08	3.54E-07	PPP2R5B	protein_cc
ENSG0000(	3125.754	0.155292	0.057558	2.697993	0.006976	0.014417	LRRN3	protein_cc
ENSG0000(	3125.507	-0.40809	0.063158	-6.46139	1.04E-10	7.89E-10	KIAA1549	protein_cc
ENSG0000(	3123.623	-0.11127	0.027003	-4.12063	3.78E-05	0.000119	TBC1D13	protein_cc
ENSG0000(	3123.515	-1.3829	0.14566	-9.49409	2.22E-21	9.67E-20	TMEM130	protein_cc
ENSG0000(	3122.769	-0.1886	0.047362	-3.98201	6.83E-05	0.000206	CPT1A	protein_cc
ENSG0000(	3121.002	0.228921	0.105907	2.161527	0.030655	0.054696	SAPCD2	protein_cc
ENSG0000(	3120.721	-0.66791	0.096798	-6.89999	5.20E-12	4.82E-11	RAPGEF5	protein_cc
ENSG0000(	3120.605	0.633599	0.100678	6.293316	3.11E-10	2.19E-09	C10orf10	protein_cc
ENSG0000(	3120.028	-0.08756	0.028776	-3.04267	0.002345	0.005341	TM2D3	protein_cc
ENSG0000(	3118.113	0.098966	0.027208	3.637408	0.000275	0.000746	AKIRIN2	protein_cc
ENSG0000(	3116.079	-0.12792	0.026695	-4.7918	1.65E-06	6.46E-06	JOSD1	protein_cc
ENSG0000(	3115.148	-0.21334	0.03803	-5.60987	2.02E-08	1.07E-07	PQLC1	protein_cc
ENSG0000(	3114.761	-0.36889	0.036254	-10.1749	2.57E-24	1.95E-22	CCDC6	protein_cc
ENSG0000(	3114.49	-0.22031	0.048092	-4.58108	4.63E-06	1.69E-05	MAP4K5	protein_cc
ENSG0000(	3114.48	0.448668	0.062535	7.174682	7.25E-13	7.71E-12	MBD6	protein_cc
ENSG0000(	3113.412	0.150512	0.025714	5.853276	4.82E-09	2.81E-08	CDC16	protein_cc
ENSG0000(	3113.104	-0.10035	0.027337	-3.67074	0.000242	0.000661	ENTPD1	protein_cc
ENSG0000(	3112.465	0.159323	0.038685	4.118527	3.81E-05	0.00012	COA3	protein_cc
ENSG0000(	3112.071	-0.45537	0.076125	-5.9819	2.21E-09	1.36E-08	MOB3B	protein_cc
ENSG0000(	3111.309	-0.81384	0.095976	-8.47967	2.26E-17	5.13E-16	FUT9	protein_cc
ENSG0000(	3109.932	-0.40351	0.065418	-6.16812	6.91E-10	4.63E-09	CAB39L	protein_cc
ENSG0000(	3109.916	-0.10484	0.036003	-2.91202	0.003591	0.007882	BRF1	protein_cc
ENSG0000(	3108.356	0.022905	0.048849	0.468901	0.63914	0.719243	FAM162A	protein_cc
ENSG0000(	3107.544	-0.441	0.082858	-5.32232	1.02E-07	4.85E-07	MYLK	protein_cc
ENSG0000(	3106.114	0.294512	0.04778	6.163887	7.10E-10	4.74E-09	VPS37B	protein_cc
ENSG0000(	3104.336	0.104481	0.026737	3.907796	9.31E-05	0.000274	TINF2	protein_cc
ENSG0000(	3103.555	0.451923	0.074645	6.054269	1.41E-09	8.98E-09	LMO2	protein_cc
ENSG0000(	3103.266	0.052996	0.036778	1.440984	0.149589	0.218254	NDUFC2	protein_cc
ENSG0000(	3102.188	0.44465	0.061211	7.264206	3.75E-13	4.15E-12	TMEM165	protein_cc
ENSG0000(	3102.005	-0.03146	0.039187	-0.80287	0.422052	0.519065	KMT2B	protein_cc
ENSG0000(	3101.899	-0.16234	0.099237	-1.63589	0.101864	0.156999	LPAR1	protein_cc
ENSG0000(	3100.941	-0.09991	0.035215	-2.83714	0.004552	0.009774	MAP3K11	protein_cc
ENSG0000(	3100.677	0.119731	0.028147	4.253849	2.10E-05	6.89E-05	KCTD5	protein_cc
ENSG0000(	3100.198	0.258296	0.055873	4.622938	3.78E-06	1.40E-05	LINC0051	lincRNA
ENSG0000(	3098.853	-0.26849	0.074841	-3.58752	0.000334	0.000891	MYC	protein_cc

ENSG0000(	3098.71	0.141314	0.03905	3.618777	0.000296	0.000797	NDUFA6	protein_cc
ENSG0000(	3098.512	0.144669	0.077748	1.860743	0.06278	0.103073	SLC24A3	protein_cc
ENSG0000(	3098.179	0.004312	0.061646	0.069942	0.944239	0.960994	EBF4	protein_cc
ENSG0000(	3093.647	-0.26807	0.050597	-5.29806	1.17E-07	5.49E-07	KIF13B	protein_cc
ENSG0000(	3093.083	0.274321	0.081095	3.382705	0.000718	0.001797	EHD2	protein_cc
ENSG0000(	3091.717	-0.32056	0.058953	-5.43759	5.40E-08	2.67E-07	UNC5B	protein_cc
ENSG0000(	3091.544	-0.20364	0.054099	-3.76424	0.000167	0.00047	ARID5B	protein_cc
ENSG0000(	3089.669	-0.14081	0.049886	-2.82258	0.004764	0.010188	ABCC5	protein_cc
ENSG0000(	3088.924	-0.2488	0.042921	-5.79661	6.77E-09	3.86E-08	CLEC16A	protein_cc
ENSG0000(	3088.78	0.196556	0.040984	4.795937	1.62E-06	6.33E-06	TMX1	protein_cc
ENSG0000(	3088.681	0.033769	0.106116	0.318223	0.750316	0.811914	GPR37	protein_cc
ENSG0000(	3088.561	-0.14958	0.028423	-5.26253	1.42E-07	6.56E-07	PIK3C3	protein_cc
ENSG0000(	3087.98	-0.54299	0.049269	-11.021	3.03E-28	4.82E-26	TIAM1	protein_cc
ENSG0000(	3087.374	0.455207	0.054667	8.326901	8.30E-17	1.71E-15	CMTM3	protein_cc
ENSG0000(	3087.319	0.163191	0.04733	3.447918	0.000565	0.001443	SOX12	protein_cc
ENSG0000(	3084.564	-0.88265	0.077115	-11.4459	2.47E-30	6.11E-28	MAST3	protein_cc
ENSG0000(	3083.591	-0.02575	0.027681	-0.93037	0.352177	0.447737	USO1	protein_cc
ENSG0000(	3083.561	-0.06217	0.054231	-1.14643	0.251618	0.338842	IFI27L2	protein_cc
ENSG0000(	3082.29	0.226924	0.029484	7.696616	1.40E-14	1.97E-13	CHST10	protein_cc
ENSG0000(	3081.976	-0.17564	0.035483	-4.95013	7.42E-07	3.06E-06	C15orf38	protein_cc
ENSG0000(	3081.753	-0.0396	0.033315	-1.18867	0.23457	0.319166	RBM33	protein_cc
ENSG0000(	3081.541	-0.14911	0.044809	-3.32777	0.000875	0.002157	KLHL21	protein_cc
ENSG0000(	3080.896	-0.1112	0.061587	-1.80555	0.070988	0.114663	ZNF711	protein_cc
ENSG0000(	3080.685	-0.11983	0.024615	-4.86836	1.13E-06	4.51E-06	SENP2	protein_cc
ENSG0000(	3079.467	0.056526	0.026528	2.130797	0.033106	0.058605	SNRPD3	protein_cc
ENSG0000(	3078.693	-0.75963	0.178419	-4.25755	2.07E-05	6.79E-05	LINC0068(	processed
ENSG0000(	3078.443	0.347425	0.081608	4.257253	2.07E-05	6.79E-05	COL5A3	protein_cc
ENSG0000(	3078.082	0.081697	0.031324	2.608161	0.009103	0.018353	TTC1	protein_cc
ENSG0000(	3076.687	-0.44569	0.051369	-8.67616	4.09E-18	1.04E-16	KLC2	protein_cc
ENSG0000(	3076.62	0.133197	0.029418	4.527678	5.96E-06	2.14E-05	LAMTOR3	protein_cc
ENSG0000(	3076.559	0.038885	0.028144	1.381638	0.167083	0.239804	TAF9B	protein_cc
ENSG0000(	3076.369	0.266755	0.055837	4.777394	1.78E-06	6.90E-06	NLGN4X	protein_cc
ENSG0000(	3076.309	-0.51531	0.0581	-8.86939	7.36E-19	2.09E-17	PDZD8	protein_cc
ENSG0000(	3074.117	0.267968	0.038675	6.928688	4.25E-12	4.01E-11	ING4	protein_cc
ENSG0000(	3073.624	-0.38731	0.038212	-10.1358	3.83E-24	2.83E-22	PITRM1	protein_cc
ENSG0000(	3073.505	-0.07136	0.044801	-1.59288	0.111188	0.169327	QSER1	protein_cc
ENSG0000(	3072.387	0.126098	0.034907	3.612385	0.000303	0.000815	DDRGK1	protein_cc
ENSG0000(	3071.706	-0.18057	0.037827	-4.77348	1.81E-06	7.03E-06	NOP9	protein_cc
ENSG0000(	3071.39	-0.19562	0.061763	-3.16733	0.001538	0.003626	KLHL25	protein_cc
ENSG0000(	3070.471	0.128947	0.028171	4.577356	4.71E-06	1.71E-05	COA5	protein_cc
ENSG0000(	3070.459	-0.12134	0.02745	-4.42045	9.85E-06	3.40E-05	MPI	protein_cc
ENSG0000(	3070.217	0.169076	0.042644	3.964847	7.34E-05	0.00022	RBX1	protein_cc
ENSG0000(	3069.183	-0.02093	0.031728	-0.65951	0.509569	0.602863	ME2	protein_cc
ENSG0000(	3069.012	0.202535	0.0584	3.468072	0.000524	0.001348	GSTO1	protein_cc
ENSG0000(	3065.724	0.528435	0.062974	8.39131	4.81E-17	1.03E-15	POLR2L	protein_cc
ENSG0000(	3065.696	0.00963	0.026239	0.367019	0.713605	0.781609	LTA4H	protein_cc
ENSG0000(	3065.62	0.235183	0.033769	6.964437	3.30E-12	3.17E-11	MRPL49	protein_cc
ENSG0000(	3064.567	-0.18397	0.055683	-3.30391	0.000953	0.002331	JADE1	protein_cc
ENSG0000(	3064.024	-0.07706	0.052519	-1.46724	0.142312	0.20906	GPR173	protein_cc
ENSG0000(	3063.221	-0.38339	0.075684	-5.06563	4.07E-07	1.75E-06	BAI3	protein_cc
ENSG0000(	3062.357	-0.48119	0.067131	-7.16802	7.61E-13	8.07E-12	SSPN	protein_cc
ENSG0000(	3061.225	-0.37424	0.039775	-9.40879	5.02E-21	2.03E-19	KIAA1279	protein_cc
ENSG0000(	3061.068	0.084046	0.032335	2.599176	0.009345	0.01879	HADH	protein_cc
ENSG0000(	3060.896	0.094051	0.02417	3.891211	9.97E-05	0.000291	SLC35C2	protein_cc
ENSG0000(	3058.872	0.014653	0.038358	0.382005	0.702458	0.772672	FNBP4	protein_cc
ENSG0000(	3058.567	0.040196	0.044995	0.89334	0.371675	0.468097	RHOQ	protein_cc
ENSG0000(	3058.369	-0.02982	0.02861	-1.04236	0.297244	0.389357	ARL5A	protein_cc
ENSG0000(	3057.718	-0.05407	0.049516	-1.09188	0.274885	0.36492	SLC25A29	protein_cc
ENSG0000(	3057.549	0.131281	0.038529	3.407362	0.000656	0.001654	ZNF316	pseudoge

ENSG0000(	3057.277	-0.27839	0.034412	-8.08978	5.98E-16	1.07E-14	MPHOSPH	protein_cc
ENSG0000(	3054.74	-0.98	0.104819	-9.34943	8.81E-21	3.42E-19	FBXO2	protein_cc
ENSG0000(	3053.47	-0.04764	0.025001	-1.90562	0.0567	0.094299	SNX2	protein_cc
ENSG0000(	3053.249	0.112173	0.031977	3.50792	0.000452	0.001176	TRAF7	protein_cc
ENSG0000(	3050.483	0.179592	0.033688	5.330959	9.77E-08	4.64E-07	RNF14	protein_cc
ENSG0000(	3050.154	-0.08305	0.03752	-2.21336	0.026873	0.048589	ALDH18A1	protein_cc
ENSG0000(	3049.624	0.5263	0.051386	10.24206	1.28E-24	1.04E-22	CARHSP1	protein_cc
ENSG0000(	3048.705	0.101163	0.038424	2.632773	0.008469	0.017206	ZMPSTE24	protein_cc
ENSG0000(	3048.503	0.214609	0.078412	2.736944	0.006201	0.012955	YBX3	protein_cc
ENSG0000(	3048.411	0.281527	0.083237	3.382236	0.000719	0.0018	LRRN2	protein_cc
ENSG0000(	3048.013	-0.0059	0.06364	-0.09274	0.926113	0.948084	IER2	protein_cc
ENSG0000(	3048.002	0.183737	0.026474	6.940202	3.92E-12	3.72E-11	FARSB	protein_cc
ENSG0000(	3047.819	-0.10194	0.088459	-1.15245	0.249137	0.335996	PLLP	protein_cc
ENSG0000(	3046.345	0.383824	0.072358	5.304528	1.13E-07	5.31E-07	GRB10	protein_cc
ENSG0000(	3046.303	-0.06181	0.028931	-2.13641	0.032646	0.057878	NBAS	protein_cc
ENSG0000(	3045.057	-0.19626	0.030934	-6.3445	2.23E-10	1.61E-09	SAP30L	protein_cc
ENSG0000(	3044.109	-0.04275	0.02276	-1.87817	0.060358	0.09959	IWS1	protein_cc
ENSG0000(	3041.588	0.091214	0.034532	2.64144	0.008255	0.016818	TXNL4A	protein_cc
ENSG0000(	3041.437	0.301173	0.040018	7.526014	5.23E-14	6.69E-13	RRM1	protein_cc
ENSG0000(	3039.13	0.199068	0.03653	5.449382	5.05E-08	2.51E-07	AC211429	protein_cc
ENSG0000(	3038.444	-0.01381	0.093879	-0.14712	0.883036	0.916533	GRIK2	protein_cc
ENSG0000(	3037.543	-0.06877	0.033069	-2.07942	0.037579	0.065652	RAD50	protein_cc
ENSG0000(	3037.138	-0.19792	0.034757	-5.69433	1.24E-08	6.77E-08	TRAPPC9	protein_cc
ENSG0000(	3037.006	0.014613	0.031269	0.467341	0.640256	0.72026	CCDC104	protein_cc
ENSG0000(	3036.469	-0.36133	0.038532	-9.37728	6.77E-21	2.67E-19	CYLD	protein_cc
ENSG0000(	3036.329	0.10051	0.034937	2.876862	0.004017	0.008735	SIGMAR1	protein_cc
ENSG0000(	3036.228	-0.02081	0.031468	-0.6613	0.508418	0.601882	ACTR10	protein_cc
ENSG0000(	3036.148	0.029519	0.025869	1.141101	0.253828	0.341292	PGRMC2	protein_cc
ENSG0000(	3035.793	0.047755	0.06193	0.771116	0.440638	0.537566	TPD52	protein_cc
ENSG0000(	3034.459	0.034582	0.027124	1.27498	0.202316	0.282055	CLINT1	protein_cc
ENSG0000(	3034.299	0.03598	0.040092	0.897438	0.369485	0.465838	ZCCHC17	protein_cc
ENSG0000(	3033.255	-0.11679	0.033319	-3.50534	0.000456	0.001186	LRRFIP2	protein_cc
ENSG0000(	3033.064	0.071795	0.03277	2.190873	0.028461	0.051157	GNL3	protein_cc
ENSG0000(	3032.134	-0.23673	0.054184	-4.36891	1.25E-05	4.24E-05	PLXNA2	protein_cc
ENSG0000(	3032.085	-0.85938	0.126367	-6.80066	1.04E-11	9.21E-11	JPH3	protein_cc
ENSG0000(	3031.415	-1.00832	0.118951	-8.47682	2.31E-17	5.24E-16	ADCY1	protein_cc
ENSG0000(	3030.013	-0.70601	0.07388	-9.5562	1.22E-21	5.55E-20	GOT1	protein_cc
ENSG0000(	3029.422	-0.02419	0.051887	-0.46619	0.641077	0.720988	MAGI1	protein_cc
ENSG0000(	3028.94	-0.71386	0.085048	-8.39366	4.71E-17	1.01E-15	PWAR6	lincRNA
ENSG0000(	3028.683	-0.21843	0.042629	-5.12403	2.99E-07	1.31E-06	FRS2	protein_cc
ENSG0000(	3028.139	0.013001	0.03255	0.399413	0.689589	0.762023	NMD3	protein_cc
ENSG0000(	3027.514	0.074668	0.021563	3.462713	0.000535	0.001372	PSMD6	protein_cc
ENSG0000(	3026.531	-0.34735	0.081204	-4.27757	1.89E-05	6.25E-05	NDN	protein_cc
ENSG0000(	3024.287	0.339853	0.11799	2.880352	0.003972	0.008646	CAV1	protein_cc
ENSG0000(	3024.083	0.000214	0.02756	0.007779	0.993793	0.995876	SMEK1	protein_cc
ENSG0000(	3024.029	-0.58953	0.074309	-7.93346	2.13E-15	3.49E-14	KCNMA1	protein_cc
ENSG0000(	3023.211	0.136263	0.029025	4.69469	2.67E-06	1.01E-05	PREB	protein_cc
ENSG0000(	3022.592	-0.22694	0.034295	-6.61748	3.65E-11	2.98E-10	UBE4B	protein_cc
ENSG0000(	3022.398	-0.0686	0.031906	-2.15008	0.031549	0.05612	DIMT1	protein_cc
ENSG0000(	3020.243	0.067637	0.028583	2.366336	0.017965	0.033797	ARFIP2	protein_cc
ENSG0000(	3019.492	-0.72453	0.097278	-7.44801	9.48E-14	1.16E-12	ADCY8	protein_cc
ENSG0000(	3019.064	-0.48549	0.13618	-3.56505	0.000364	0.000964	PCDH15	protein_cc
ENSG0000(	3018.967	0.107638	0.038006	2.832108	0.004624	0.009913	SDHB	protein_cc
ENSG0000(	3017.488	-0.02268	0.072371	-0.31341	0.753968	0.81458	SYNGR2	protein_cc
ENSG0000(	3016.374	0.116155	0.0309	3.759022	0.000171	0.000479	PPP1R8	protein_cc
ENSG0000(	3015.42	-0.11876	0.036313	-3.27057	0.001073	0.002601	HPS4	protein_cc
ENSG0000(	3015.128	-0.10517	0.060267	-1.74514	0.080961	0.128566	ANO8	protein_cc
ENSG0000(	3014.906	-0.06428	0.026053	-2.4673	0.013614	0.026385	FAM193A	protein_cc
ENSG0000(	3014.633	0.024081	0.100991	0.238448	0.811534	0.861497	NRN1	protein_cc

ENSG0000(	3014.435	-0.13102	0.050402	-2.59959	0.009333	0.018769	TTLL12	protein_cc
ENSG0000(	3013.172	-0.3197	0.064158	-4.983	6.26E-07	2.61E-06	KAT6B	protein_cc
ENSG0000(	3013.131	0.06934	0.040528	1.710933	0.087094	0.13707	RPS19BP1	protein_cc
ENSG0000(	3012.636	-0.01086	0.075789	-0.14329	0.886063	0.91884	FAM69C	protein_cc
ENSG0000(	3012.393	-0.03696	0.0296	-1.2486	0.211813	0.293127	VTA1	protein_cc
ENSG0000(	3010.336	0.05869	0.032973	1.779949	0.075084	0.120447	NOMO1	protein_cc
ENSG0000(	3008.712	-0.38655	0.09605	-4.0244	5.71E-05	0.000174	UNC80	protein_cc
ENSG0000(	3008.348	0.148204	0.024575	6.030664	1.63E-09	1.03E-08	PWP1	protein_cc
ENSG0000(	3008.239	-0.42515	0.092922	-4.57529	4.76E-06	1.73E-05	GPR158	protein_cc
ENSG0000(	3007.456	-0.5567	0.060998	-9.12659	7.07E-20	2.36E-18	TPRG1L	protein_cc
ENSG0000(	3006.922	0.085786	0.026284	3.263857	0.001099	0.002659	TRAFD1	protein_cc
ENSG0000(	3006.298	0.406602	0.041565	9.782276	1.34E-22	7.24E-21	SNRPC	protein_cc
ENSG0000(	3005.975	-0.10137	0.032971	-3.07451	0.002108	0.004842	SEMA4C	protein_cc
ENSG0000(	3005.655	-0.0989	0.035205	-2.80933	0.004964	0.010586	KIAA0947	protein_cc
ENSG0000(	3004.911	0.031816	0.070926	0.44858	0.653735	0.731702	PARP14	protein_cc
ENSG0000(	3004.302	-0.0851	0.031379	-2.71194	0.006689	0.013881	TMEM63B	protein_cc
ENSG0000(	3004.286	0.018008	0.034411	0.523314	0.600756	0.685463	CFLAR	protein_cc
ENSG0000(	3003.728	0.2145	0.059254	3.620008	0.000295	0.000794	STK40	protein_cc
ENSG0000(	3003.667	0.011882	0.032289	0.367993	0.712879	0.780959	ACER3	protein_cc
ENSG0000(	3002.602	0.061929	0.036742	1.685542	0.091884	0.143527	TSR3	protein_cc
ENSG0000(	3001.464	0.099752	0.026914	3.706342	0.00021	0.00058	NCOA5	protein_cc
ENSG0000(	3000.621	-0.01616	0.042681	-0.37867	0.704935	0.774734	ATP13A3	protein_cc
ENSG0000(	3000.518	-0.86533	0.090461	-9.56578	1.11E-21	5.09E-20	DLG2	protein_cc
ENSG0000(	3000.264	-0.01006	0.031648	-0.31779	0.750644	0.812205	TFCP2	protein_cc
ENSG0000(	2999.848	-0.30977	0.087825	-3.52714	0.00042	0.001101	SLC2A5	protein_cc
ENSG0000(	2999.417	0.311876	0.034632	9.005363	2.15E-19	6.64E-18	BCL7B	protein_cc
ENSG0000(	2998.874	-0.05422	0.031047	-1.74647	0.08073	0.128252	CRBN	protein_cc
ENSG0000(	2998.547	-0.3196	0.065038	-4.91406	8.92E-07	3.63E-06	RICTOR	protein_cc
ENSG0000(	2997.892	-0.04626	0.024934	-1.85532	0.063551	0.104188	FOXK2	protein_cc
ENSG0000(	2997.623	0.108708	0.031072	3.498602	0.000468	0.001214	R3HCC1	protein_cc
ENSG0000(	2997.604	-0.27565	0.073786	-3.73574	0.000187	0.000521	HRSP12	protein_cc
ENSG0000(	2996.721	0.105557	0.040614	2.599012	0.009349	0.018798	HEXA	protein_cc
ENSG0000(	2996.707	-0.09354	0.036157	-2.58709	0.009679	0.019395	MAN2B2	protein_cc
ENSG0000(	2996.613	0.24164	0.037646	6.418713	1.37E-10	1.03E-09	RNASEH2(	protein_cc
ENSG0000(	2996.556	-0.05061	0.098114	-0.51587	0.605944	0.689986	CELSR3	protein_cc
ENSG0000(	2996.494	-0.05206	0.029313	-1.77617	0.075705	0.121319	DDX46	protein_cc
ENSG0000(	2996.162	-0.00729	0.03475	-0.20981	0.83382	0.87887	SLC35B4	protein_cc
ENSG0000(	2994.672	0.006481	0.065531	0.0989	0.921218	0.944538	KIAA0895I	protein_cc
ENSG0000(	2994.2	0.034274	0.082313	0.41639	0.677124	0.751337	LAMA4	protein_cc
ENSG0000(	2993.352	-0.04701	0.035831	-1.31203	0.189511	0.266717	CDK5RAP2	protein_cc
ENSG0000(	2993.046	-0.07528	0.036342	-2.0714	0.038322	0.066825	SURF6	protein_cc
ENSG0000(	2991.637	-0.02732	0.070165	-0.38935	0.697019	0.76803	ARRDC4	protein_cc
ENSG0000(	2991.249	-0.08267	0.026575	-3.111	0.001865	0.004325	FN3KRP	protein_cc
ENSG0000(	2990.074	-0.28201	0.041505	-6.79463	1.09E-11	9.57E-11	UNC13B	protein_cc
ENSG0000(	2989.938	-0.05111	0.035947	-1.42183	0.155077	0.225066	PRKAR2A	protein_cc
ENSG0000(	2984.925	-0.40667	0.04131	-9.84414	7.27E-23	4.14E-21	LDB1	protein_cc
ENSG0000(	2984.7	0.146901	0.035305	4.160902	3.17E-05	0.000101	EMC3	protein_cc
ENSG0000(	2984.668	0.039032	0.030718	1.270624	0.203862	0.283835	SLC35F6	protein_cc
ENSG0000(	2983.938	-0.60002	0.091263	-6.57462	4.88E-11	3.90E-10	FGF12	protein_cc
ENSG0000(	2983.538	0.115083	0.052148	2.206862	0.027324	0.049298	FNDC4	protein_cc
ENSG0000(	2982.203	-0.07184	0.030944	-2.32157	0.020256	0.037657	NAB1	protein_cc
ENSG0000(	2981.92	-0.23992	0.04201	-5.71095	1.12E-08	6.18E-08	DDX21	protein_cc
ENSG0000(	2981.082	-0.15497	0.03344	-4.63415	3.58E-06	1.33E-05	RNF44	protein_cc
ENSG0000(	2979.735	0.978745	0.110359	8.868698	7.40E-19	2.10E-17	ELOVL2	protein_cc
ENSG0000(	2977.402	0.141265	0.031457	4.490697	7.10E-06	2.51E-05	R3HDM4	protein_cc
ENSG0000(	2977.18	-0.14563	0.081159	-1.79439	0.072751	0.117229	COL9A2	protein_cc
ENSG0000(	2974.992	-0.0589	0.037703	-1.5621	0.118265	0.178517	PHRF1	protein_cc
ENSG0000(	2974.838	0.161267	0.031429	5.131138	2.88E-07	1.27E-06	PIP5K1A	protein_cc
ENSG0000(	2974.464	-0.19476	0.054835	-3.55168	0.000383	0.00101	GAS6	protein_cc

ENSG0000(	2974.427	-0.01594	0.036879	-0.43209	0.665673	0.741974	NDUFAF3	protein_cc
ENSG0000(	2973.966	-0.0718	0.041276	-1.73942	0.081961	0.129876	TCERG1	protein_cc
ENSG0000(	2971.293	-0.30859	0.092024	-3.35338	0.000798	0.001981	SCN4B	protein_cc
ENSG0000(	2971.272	0.095431	0.03284	2.905903	0.003662	0.008022	POLRMT	protein_cc
ENSG0000(	2970.82	0.076817	0.033209	2.313172	0.020713	0.038427	TRUB2	protein_cc
ENSG0000(	2970.004	-0.04084	0.025251	-1.61735	0.105803	0.162165	COASY	protein_cc
ENSG0000(	2969.739	-0.29942	0.046037	-6.50376	7.83E-11	6.07E-10	TNRC6B	protein_cc
ENSG0000(	2969.427	0.0017	0.032924	0.051636	0.958819	0.971864	EHMT1	protein_cc
ENSG0000(	2969.27	0.293966	0.02552	11.51881	1.06E-30	2.87E-28	CRCP	protein_cc
ENSG0000(	2969.239	0.246103	0.034524	7.128455	1.02E-12	1.05E-11	NT5C3B	protein_cc
ENSG0000(	2967.773	-0.04188	0.057326	-0.73055	0.465052	0.561042	TM7SF2	protein_cc
ENSG0000(	2967.76	0.113178	0.08599	1.316175	0.188115	0.265024	XYLT1	protein_cc
ENSG0000(	2967.092	-0.24268	0.033695	-7.20226	5.92E-13	6.37E-12	CDK17	protein_cc
ENSG0000(	2964.982	-0.28502	0.111593	-2.55413	0.010645	0.021134	DSCAML1	protein_cc
ENSG0000(	2964.181	0.254718	0.039168	6.503177	7.86E-11	6.08E-10	UNC45A	protein_cc
ENSG0000(	2963.731	-0.1533	0.025857	-5.92889	3.05E-09	1.84E-08	PHAX	protein_cc
ENSG0000(	2963.477	0.002582	0.025775	0.100192	0.920192	0.943739	PSMD7	protein_cc
ENSG0000(	2963.427	-0.08961	0.03339	-2.68363	0.007283	0.015002	ZC3H4	protein_cc
ENSG0000(	2961.875	-0.16097	0.110533	-1.45628	0.145316	0.212842	BMP2	protein_cc
ENSG0000(	2961.485	0.39086	0.073963	5.284564	1.26E-07	5.88E-07	NT5E	protein_cc
ENSG0000(	2960.674	0.04687	0.024485	1.914197	0.055595	0.092706	MAEA	protein_cc
ENSG0000(	2960.409	0.297286	0.08011	3.710978	0.000206	0.000571	PLOD2	protein_cc
ENSG0000(	2960.103	-0.53451	0.086904	-6.1506	7.72E-10	5.13E-09	PGM2L1	protein_cc
ENSG0000(	2959.422	0.150421	0.034277	4.388344	1.14E-05	3.91E-05	TLK1	protein_cc
ENSG0000(	2958.909	0.187091	0.03407	5.491311	3.99E-08	2.02E-07	FH	protein_cc
ENSG0000(	2958.286	-0.03224	0.029185	-1.10467	0.2693	0.35859	LYRM2	protein_cc
ENSG0000(	2957.541	-0.06148	0.032122	-1.91404	0.055615	0.092734	KIAA1033	protein_cc
ENSG0000(	2957.295	-0.05562	0.037236	-1.49365	0.135267	0.200105	GTF3C4	protein_cc
ENSG0000(	2955.936	-0.01651	0.070067	-0.23564	0.813713	0.863141	TGFB1	protein_cc
ENSG0000(	2955.68	0.61282	0.153787	3.984848	6.75E-05	0.000203	CXCL14	protein_cc
ENSG0000(	2955.5	0.224172	0.024234	9.250349	2.24E-20	8.11E-19	USP39	protein_cc
ENSG0000(	2955.08	0.121907	0.027819	4.382121	1.18E-05	4.01E-05	MKKS	protein_cc
ENSG0000(	2954.302	0.178427	0.04032	4.425299	9.63E-06	3.33E-05	PTAR1	protein_cc
ENSG0000(	2954.061	-0.0803	0.02731	-2.94045	0.003277	0.007249	GFM1	protein_cc
ENSG0000(	2953.44	0.33972	0.254248	1.336179	0.181491	0.257065	XIST	lincRNA
ENSG0000(	2952.195	0.128442	0.028137	4.564841	5.00E-06	1.81E-05	UBAC2	protein_cc
ENSG0000(	2952.062	-0.24805	0.043607	-5.68833	1.28E-08	6.99E-08	GPATCH2I	protein_cc
ENSG0000(	2951.632	0.09517	0.025567	3.722388	0.000197	0.000548	SNF8	protein_cc
ENSG0000(	2950.296	-0.38609	0.055889	-6.9081	4.91E-12	4.57E-11	LDOC1	protein_cc
ENSG0000(	2949.622	-0.08934	0.024536	-3.6413	0.000271	0.000736	CLPX	protein_cc
ENSG0000(	2948.276	0.041865	0.03843	1.089376	0.275988	0.366132	PRKD3	protein_cc
ENSG0000(	2948.252	0.086566	0.031967	2.707945	0.00677	0.014033	ACP2	protein_cc
ENSG0000(	2947.726	-0.30743	0.037038	-8.30037	1.04E-16	2.10E-15	LARP4B	protein_cc
ENSG0000(	2947.594	0.153258	0.055371	2.767845	0.005643	0.01189	DANCR	processed
ENSG0000(	2946.233	-0.05867	0.044843	-1.30831	0.190767	0.268209	ETFB	protein_cc
ENSG0000(	2944.702	-0.17855	0.039381	-4.53399	5.79E-06	2.08E-05	SRC	protein_cc
ENSG0000(	2943.594	-0.05442	0.074952	-0.72608	0.46779	0.563634	BBOX1	protein_cc
ENSG0000(	2942.454	-0.09058	0.022367	-4.0498	5.13E-05	0.000158	CDC27	protein_cc
ENSG0000(	2940.013	0.345868	0.041359	8.362657	6.13E-17	1.29E-15	ALYREF	protein_cc
ENSG0000(	2939.097	0.152096	0.043049	3.53304	0.000411	0.001078	SPTSSA	protein_cc
ENSG0000(	2938.487	-0.03816	0.0533	-0.71587	0.474071	0.569933	NLGN1	protein_cc
ENSG0000(	2938.375	0.23569	0.029897	7.883449	3.18E-15	5.07E-14	ELOF1	protein_cc
ENSG0000(	2937.434	-0.44062	0.064478	-6.83367	8.28E-12	7.44E-11	SLC25A22	protein_cc
ENSG0000(	2937.176	0.114897	0.025655	4.478491	7.52E-06	2.65E-05	BIRC2	protein_cc
ENSG0000(	2937.014	0.636307	0.075992	8.373345	5.60E-17	1.19E-15	GNG5	protein_cc
ENSG0000(	2936.086	-0.04882	0.052124	-0.93656	0.348983	0.444403	RHOG	protein_cc
ENSG0000(	2935.741	-0.00595	0.02964	-0.20076	0.840888	0.884326	IKBKAP	protein_cc
ENSG0000(	2934.416	0.029045	0.032975	0.880806	0.378423	0.474952	DAZAP1	protein_cc
ENSG0000(	2934.026	0.10617	0.030571	3.472868	0.000515	0.001326	CXXC1	protein_cc

ENSG0000(	2932.836	0.059266	0.032625	1.816579	0.069282	0.112317	RWDD1	protein_cc
ENSG0000(	2932.757	0.078048	0.034302	2.275277	0.022889	0.042066	ZNF302	protein_cc
ENSG0000(	2932.584	0.036682	0.031761	1.154912	0.248126	0.33483	SPTLC1	protein_cc
ENSG0000(	2931.543	-0.74074	0.106737	-6.93983	3.93E-12	3.73E-11	S100A1	protein_cc
ENSG0000(	2931.256	-0.05686	0.036886	-1.54144	0.12321	0.18478	INSR	protein_cc
ENSG0000(	2930.559	-0.12584	0.051684	-2.43479	0.0149	0.028618	ZNF292	protein_cc
ENSG0000(	2930.029	-0.20224	0.062488	-3.2364	0.00121	0.002906	ABCG2	protein_cc
ENSG0000(	2929.855	-0.34405	0.05091	-6.75801	1.40E-11	1.21E-10	DSTYK	protein_cc
ENSG0000(	2929.499	0.148633	0.04643	3.201209	0.001369	0.003256	MZT2B	protein_cc
ENSG0000(	2929.452	-0.06274	0.043803	-1.4323	0.152058	0.221401	ESYT1	protein_cc
ENSG0000(	2929.104	-0.06222	0.030476	-2.04166	0.041185	0.07121	MUT	protein_cc
ENSG0000(	2929.079	0.441583	0.089131	4.954307	7.26E-07	3.00E-06	KCNIP1	protein_cc
ENSG0000(	2929.003	-0.00768	0.032522	-0.23604	0.8134	0.862953	COMMD9	protein_cc
ENSG0000(	2927.264	-0.18169	0.0447	-4.06456	4.81E-05	0.000148	CDK19	protein_cc
ENSG0000(	2926.911	-0.08579	0.069357	-1.23688	0.216133	0.298174	LHFPL2	protein_cc
ENSG0000(	2926.518	-0.3445	0.055838	-6.16951	6.85E-10	4.59E-09	MAF	protein_cc
ENSG0000(	2925.483	0.071939	0.035519	2.025395	0.042827	0.073685	DMAP1	protein_cc
ENSG0000(	2925.155	-0.31032	0.05129	-6.05025	1.45E-09	9.19E-09	KAZN	protein_cc
ENSG0000(	2924.653	-0.09113	0.023673	-3.84943	0.000118	0.000342	CCDC25	protein_cc
ENSG0000(	2923.871	-0.33323	0.057717	-5.77342	7.77E-09	4.38E-08	GNAZ	protein_cc
ENSG0000(	2923.372	0.122333	0.028811	4.24603	2.18E-05	7.11E-05	PSMC6	protein_cc
ENSG0000(	2923.352	-0.15146	0.029983	-5.05165	4.38E-07	1.87E-06	MCUR1	protein_cc
ENSG0000(	2923.123	-0.58406	0.059985	-9.73667	2.10E-22	1.09E-20	SYNJ1	protein_cc
ENSG0000(	2922.49	0.145729	0.025634	5.685046	1.31E-08	7.12E-08	DENR	protein_cc
ENSG0000(	2922.079	0.391761	0.046383	8.446312	3.01E-17	6.68E-16	CD320	protein_cc
ENSG0000(	2921.757	-0.01415	0.038306	-0.36951	0.71175	0.78018	SFSWAP	protein_cc
ENSG0000(	2921.481	-0.0426	0.031432	-1.35537	0.175299	0.249723	THOC2	protein_cc
ENSG0000(	2918.822	-0.34103	0.043649	-7.81299	5.58E-15	8.51E-14	NEK7	protein_cc
ENSG0000(	2917.996	0.09832	0.047915	2.051981	0.040171	0.069661	GPSM2	protein_cc
ENSG0000(	2917.892	-0.11176	0.057359	-1.94837	0.05137	0.086534	AGFG2	protein_cc
ENSG0000(	2915.867	-0.12668	0.029631	-4.27531	1.91E-05	6.30E-05	HDHD2	protein_cc
ENSG0000(	2915.756	0.104349	0.03801	2.745274	0.006046	0.012656	PLEKHA2	protein_cc
ENSG0000(	2915.173	-0.32101	0.042088	-7.62708	2.40E-14	3.24E-13	INPP4A	protein_cc
ENSG0000(	2914.815	-0.02437	0.030591	-0.79661	0.425678	0.522742	ZNF629	protein_cc
ENSG0000(	2913.572	-0.17755	0.0456	-3.89356	9.88E-05	0.000289	CCSAP	protein_cc
ENSG0000(	2913.291	0.32755	0.050942	6.429852	1.28E-10	9.59E-10	QTRT1	protein_cc
ENSG0000(	2912.415	-0.11281	0.04627	-2.4381	0.014765	0.028395	PAG1	protein_cc
ENSG0000(	2911.413	0.141224	0.023252	6.073493	1.25E-09	8.04E-09	ATG12	protein_cc
ENSG0000(	2911.223	-0.3691	0.045906	-8.04048	8.95E-16	1.55E-14	NCOA2	protein_cc
ENSG0000(	2910.864	-0.10503	0.037513	-2.79992	0.005112	0.010875	GPCPD1	protein_cc
ENSG0000(	2910.494	-0.18146	0.03296	-5.50549	3.68E-08	1.87E-07	RNMT	protein_cc
ENSG0000(	2910.076	0.326912	0.047554	6.874539	6.22E-12	5.70E-11	NDUFA2	protein_cc
ENSG0000(	2909.827	-0.16308	0.03532	-4.6173	3.89E-06	1.43E-05	RAB4A	protein_cc
ENSG0000(	2909.504	0.185203	0.020538	9.017787	1.92E-19	6.02E-18	EIF2B1	protein_cc
ENSG0000(	2909.109	-0.00395	0.028648	-0.1379	0.890316	0.921925	PPP2R2A	protein_cc
ENSG0000(	2908.885	0.022402	0.042268	0.529994	0.596116	0.68138	KIAA2013	protein_cc
ENSG0000(	2908.422	0.089895	0.07078	1.270065	0.204061	0.284074	CERS1	protein_cc
ENSG0000(	2905.91	-0.14497	0.051437	-2.81846	0.004825	0.010308	STK38L	protein_cc
ENSG0000(	2905.21	-0.27831	0.034632	-8.03609	9.28E-16	1.60E-14	CTBP2	protein_cc
ENSG0000(	2904.847	-0.60524	0.0682	-8.87445	7.03E-19	2.00E-17	MGAT3	protein_cc
ENSG0000(	2903.916	0.297901	0.033923	8.781778	1.61E-18	4.33E-17	GPR108	protein_cc
ENSG0000(	2901.502	-0.6508	0.106764	-6.0957	1.09E-09	7.07E-09	OPCML	protein_cc
ENSG0000(	2901.216	-0.08722	0.03093	-2.82	0.004802	0.010263	ZNF687	protein_cc
ENSG0000(	2900.98	0.253194	0.050508	5.012913	5.36E-07	2.26E-06	PXDC1	protein_cc
ENSG0000(	2900.68	-0.37337	0.058917	-6.33724	2.34E-10	1.68E-09	GDAP1	protein_cc
ENSG0000(	2900.529	-0.0917	0.033251	-2.75782	0.005819	0.012225	AP3M1	protein_cc
ENSG0000(	2900.433	-0.05619	0.03309	-1.69808	0.089492	0.140356	RIF1	protein_cc
ENSG0000(	2900.381	-0.82287	0.115504	-7.12415	1.05E-12	1.08E-11	LGI3	protein_cc
ENSG0000(	2899.978	-0.22314	0.051258	-4.35325	1.34E-05	4.53E-05	NDFIP2	protein_cc

ENSG0000(	2899.442	0.064241	0.023282	2.759252	0.005793	0.012177	GTF3C2	protein_cc
ENSG0000(	2898.396	0.262752	0.0333	7.890504	3.01E-15	4.80E-14	COPS3	protein_cc
ENSG0000(	2898.186	0.110814	0.103314	1.072595	0.283453	0.37408	GADD45G	protein_cc
ENSG0000(	2897.818	-0.08627	0.039318	-2.19421	0.028221	0.050759	M6PR	protein_cc
ENSG0000(	2897.195	-0.06259	0.020269	-3.08797	0.002015	0.004647	RNF115	protein_cc
ENSG0000(	2896.507	-0.01825	0.029344	-0.62205	0.53391	0.625328	FBXL3	protein_cc
ENSG0000(	2896.494	-0.12373	0.024586	-5.03247	4.84E-07	2.06E-06	ATXN2	protein_cc
ENSG0000(	2895.102	0.045312	0.031882	1.42125	0.155244	0.225266	GPR137	protein_cc
ENSG0000(	2894.176	-0.09091	0.060249	-1.50885	0.131337	0.195113	KLF12	protein_cc
ENSG0000(	2893.969	0.219483	0.026343	8.331901	7.96E-17	1.64E-15	PAIP1	protein_cc
ENSG0000(	2893.71	0.279113	0.047398	5.888658	3.89E-09	2.31E-08	SLMO2	protein_cc
ENSG0000(	2891.02	-0.10267	0.024571	-4.17857	2.93E-05	9.37E-05	CHMP1B	protein_cc
ENSG0000(	2890.49	-0.35435	0.049917	-7.09885	1.26E-12	1.29E-11	C1orf216	protein_cc
ENSG0000(	2890.426	0.132193	0.028481	4.641367	3.46E-06	1.28E-05	RSPRY1	protein_cc
ENSG0000(	2889.82	-0.038	0.117473	-0.32346	0.746345	0.808838	VCAM1	protein_cc
ENSG0000(	2887.839	-0.31435	0.050258	-6.25471	3.98E-10	2.76E-09	PTPRK	protein_cc
ENSG0000(	2887.271	-0.21046	0.039817	-5.28575	1.25E-07	5.84E-07	ASMTL	protein_cc
ENSG0000(	2887.183	0.2103	0.036552	5.753442	8.74E-09	4.89E-08	TEX264	protein_cc
ENSG0000(	2886.597	-0.07216	0.026793	-2.69327	0.007075	0.014604	CES2	protein_cc
ENSG0000(	2886.435	0.107144	0.031222	3.431631	0.0006	0.001525	FAM3A	protein_cc
ENSG0000(	2885	-0.17577	0.038861	-4.52301	6.10E-06	2.18E-05	DIP2A	protein_cc
ENSG0000(	2884.777	-0.00779	0.022299	-0.34926	0.726892	0.792321	SNX14	protein_cc
ENSG0000(	2883.158	-0.02435	0.076385	-0.31878	0.74989	0.811603	CTXN1	protein_cc
ENSG0000(	2883.116	0.060741	0.039295	1.545763	0.122162	0.183443	PNPLA2	protein_cc
ENSG0000(	2882.495	-0.1588	0.070275	-2.25963	0.023844	0.043638	KCND3	protein_cc
ENSG0000(	2882.231	-0.14048	0.031554	-4.452	8.51E-06	2.97E-05	ACO1	protein_cc
ENSG0000(	2882.103	-0.73961	0.07575	-9.76392	1.61E-22	8.57E-21	KALRN	protein_cc
ENSG0000(	2881.443	0.287167	0.044514	6.451104	1.11E-10	8.40E-10	HMG20B	protein_cc
ENSG0000(	2881.058	-0.15515	0.040867	-3.79655	0.000147	0.000416	FAM178A	protein_cc
ENSG0000(	2878.108	0.373766	0.065193	5.73322	9.85E-09	5.47E-08	HEXB	protein_cc
ENSG0000(	2876.171	0.031831	0.054686	0.582069	0.56052	0.649447	MAN2B1	protein_cc
ENSG0000(	2875.342	-0.15967	0.035996	-4.43576	9.17E-06	3.18E-05	HAGH	protein_cc
ENSG0000(	2875.169	0.187675	0.058808	3.191313	0.001416	0.003359	DOT1L	protein_cc
ENSG0000(	2875.086	0.18567	0.033291	5.577201	2.44E-08	1.28E-07	NHP2	protein_cc
ENSG0000(	2875.01	0.063065	0.035287	1.787195	0.073906	0.11882	RRN3	protein_cc
ENSG0000(	2874.587	0.137744	0.031937	4.312936	1.61E-05	5.38E-05	MOB3A	protein_cc
ENSG0000(	2873.836	-0.48816	0.068129	-7.16525	7.76E-13	8.21E-12	DOCK9	protein_cc
ENSG0000(	2873.206	0.358417	0.058947	6.080335	1.20E-09	7.72E-09	MYEOV2	protein_cc
ENSG0000(	2872.88	-0.77016	0.089048	-8.64879	5.20E-18	1.30E-16	CHST1	protein_cc
ENSG0000(	2872.874	0.418492	0.051058	8.196353	2.48E-16	4.72E-15	C19orf10	protein_cc
ENSG0000(	2872.447	-0.22678	0.032713	-6.93242	4.14E-12	3.91E-11	GTF2A1	protein_cc
ENSG0000(	2871.512	-0.46861	0.080253	-5.83914	5.25E-09	3.04E-08	TMEM63A	protein_cc
ENSG0000(	2871.004	-0.54182	0.070357	-7.70098	1.35E-14	1.91E-13	GNAI1	protein_cc
ENSG0000(	2870.36	-0.94611	0.1043	-9.07104	1.18E-19	3.82E-18	PPP1R16B	protein_cc
ENSG0000(	2868.279	0.212661	0.033883	6.276344	3.47E-10	2.43E-09	THYN1	protein_cc
ENSG0000(	2867.51	0.080194	0.035261	2.274289	0.022949	0.042165	OGFR	protein_cc
ENSG0000(	2867.458	-0.16729	0.054308	-3.08044	0.002067	0.004758	SLC4A8	protein_cc
ENSG0000(	2867.351	0.193828	0.027701	6.99706	2.61E-12	2.55E-11	SPCS2	protein_cc
ENSG0000(	2867.311	-0.00038	0.027183	-0.01381	0.988984	0.992382	ZC3H18	protein_cc
ENSG0000(	2867.023	0.49298	0.055353	8.906165	5.28E-19	1.53E-17	UBALD2	protein_cc
ENSG0000(	2865.817	0.14017	0.059365	2.361169	0.018217	0.034235	SNHG5	processed
ENSG0000(	2865.551	0.231333	0.043057	5.372707	7.76E-08	3.74E-07	HSPBP1	protein_cc
ENSG0000(	2863.68	-0.38441	0.05139	-7.48029	7.42E-14	9.24E-13	LIMK2	protein_cc
ENSG0000(	2862.619	-0.12785	0.028576	-4.47401	7.68E-06	2.70E-05	C5orf51	protein_cc
ENSG0000(	2861.127	0.082574	0.033723	2.448603	0.014341	0.02767	TMEM214	protein_cc
ENSG0000(	2860.947	-0.10702	0.029357	-3.64537	0.000267	0.000725	STRIP1	protein_cc
ENSG0000(	2860.904	0.109166	0.023983	4.551803	5.32E-06	1.92E-05	ASNSD1	protein_cc
ENSG0000(	2860.779	0.044047	0.034298	1.284227	0.199063	0.278122	CCDC93	protein_cc
ENSG0000(	2860.647	-0.30534	0.054656	-5.58663	2.32E-08	1.21E-07	CSRNP1	protein_cc

ENSG0000(	2860.36	0.013077	0.02567	0.509422	0.610457	0.694159	GMPS	protein_cc
ENSG0000(	2859.23	-0.09484	0.033309	-2.84729	0.004409	0.009501	GGA2	protein_cc
ENSG0000(	2857.806	0.06085	0.045377	1.340984	0.179926	0.255182	C14orf2	protein_cc
ENSG0000(	2857.088	0.047424	0.025727	1.843384	0.065273	0.106595	TRIM26	protein_cc
ENSG0000(	2857.06	-0.01501	0.02204	-0.68093	0.495915	0.590105	SNW1	protein_cc
ENSG0000(	2857.041	0.035015	0.043446	0.805938	0.420279	0.517224	CC2D1A	protein_cc
ENSG0000(	2856.283	-0.18889	0.037593	-5.02461	5.04E-07	2.14E-06	FBXL17	protein_cc
ENSG0000(	2855.359	-0.11965	0.038649	-3.09578	0.001963	0.004535	PBRM1	protein_cc
ENSG0000(	2854.709	0.32068	0.058572	5.474939	4.38E-08	2.20E-07	SGCE	protein_cc
ENSG0000(	2854.019	0.033171	0.026088	1.271487	0.203555	0.283501	INPP5K	protein_cc
ENSG0000(	2853.591	0.001866	0.028647	0.065121	0.948078	0.96409	BAG5	protein_cc
ENSG0000(	2851.523	0.036689	0.055393	0.662336	0.507756	0.601306	ACPL2	protein_cc
ENSG0000(	2851.121	0.260374	0.029794	8.739252	2.35E-18	6.20E-17	CIAPIN1	protein_cc
ENSG0000(	2850.78	0.330892	0.032907	10.05548	8.69E-24	6.07E-22	ZDHHC4	protein_cc
ENSG0000(	2850.255	-0.14606	0.04121	-3.54437	0.000394	0.001037	DGKD	protein_cc
ENSG0000(	2849.741	-0.20818	0.054406	-3.82635	0.00013	0.000372	LRP6	protein_cc
ENSG0000(	2849.161	0.062994	0.036431	1.729159	0.083781	0.132413	PEX6	protein_cc
ENSG0000(	2848.591	-0.09259	0.032064	-2.88761	0.003882	0.008465	DENND1A	protein_cc
ENSG0000(	2848.5	-0.13017	0.034835	-3.73692	0.000186	0.000519	CTNNBIP1	protein_cc
ENSG0000(	2847.508	0.069739	0.023431	2.976366	0.002917	0.006518	EIF2B5	protein_cc
ENSG0000(	2847.368	-0.21655	0.118595	-1.82597	0.067855	0.110273	GBP2	protein_cc
ENSG0000(	2846.345	-0.06917	0.054257	-1.27485	0.202362	0.2821	ZNF462	protein_cc
ENSG0000(	2846.302	-0.17114	0.043599	-3.92523	8.66E-05	0.000256	REEP3	protein_cc
ENSG0000(	2846.134	-0.02156	0.030435	-0.70829	0.478764	0.574182	UBL7	protein_cc
ENSG0000(	2845.239	-0.03161	0.022436	-1.40903	0.158826	0.229565	RAB35	protein_cc
ENSG0000(	2844.528	0.21935	0.025263	8.682669	3.87E-18	9.86E-17	ANAPC7	protein_cc
ENSG0000(	2843.811	-0.41117	0.067792	-6.06516	1.32E-09	8.44E-09	LRRC4	protein_cc
ENSG0000(	2843.447	-0.22146	0.03933	-5.63074	1.79E-08	9.58E-08	CEP250	protein_cc
ENSG0000(	2842.658	1.139145	0.147	7.74927	9.24E-15	1.36E-13	RBP1	protein_cc
ENSG0000(	2842.143	0.152474	0.042938	3.550978	0.000384	0.001012	BAD	protein_cc
ENSG0000(	2841.283	0.059481	0.04928	1.207002	0.227431	0.311068	SPATA13	protein_cc
ENSG0000(	2841.223	0.156185	0.042434	3.680624	0.000233	0.000638	VAV2	protein_cc
ENSG0000(	2840.879	-0.16053	0.036903	-4.34992	1.36E-05	4.60E-05	FAM120C	protein_cc
ENSG0000(	2840.638	-0.03904	0.04074	-0.95815	0.337985	0.432865	TAF1D	protein_cc
ENSG0000(	2839.901	-0.05162	0.0223	-2.31479	0.020625	0.038278	CHMP7	protein_cc
ENSG0000(	2839.143	0.030471	0.023961	1.271676	0.203488	0.283429	AGGF1	protein_cc
ENSG0000(	2839.098	-0.67736	0.107178	-6.31993	2.62E-10	1.87E-09	B3GAT2	protein_cc
ENSG0000(	2838.147	-0.27511	0.053653	-5.12755	2.94E-07	1.29E-06	FAM208B	protein_cc
ENSG0000(	2837.401	-0.01109	0.043017	-0.25771	0.796634	0.849517	RRM2B	protein_cc
ENSG0000(	2837.339	0.367871	0.032914	11.17659	5.31E-29	1.00E-26	CERS5	protein_cc
ENSG0000(	2836.088	-0.0431	0.042306	-1.0188	0.308298	0.401394	ZKSCAN8	protein_cc
ENSG0000(	2835.749	-0.11875	0.028336	-4.19069	2.78E-05	8.93E-05	KIAA0196	protein_cc
ENSG0000(	2835.264	-0.11734	0.043569	-2.69312	0.007079	0.01461	RAB7L1	protein_cc
ENSG0000(	2834.053	0.001373	0.021464	0.063977	0.948988	0.964753	KCMF1	protein_cc
ENSG0000(	2833.879	-0.27914	0.055408	-5.0379	4.71E-07	2.00E-06	DENND5B	protein_cc
ENSG0000(	2833.599	0.120156	0.037306	3.220849	0.001278	0.003055	ABCD4	protein_cc
ENSG0000(	2832.975	-0.75324	0.077069	-9.77358	1.46E-22	7.86E-21	DOCK3	protein_cc
ENSG0000(	2832.621	0.232645	0.036551	6.364915	1.95E-10	1.42E-09	MRPS26	protein_cc
ENSG0000(	2832.494	0.038685	0.021073	1.835794	0.066388	0.108202	RBM14	protein_cc
ENSG0000(	2830.998	0.451932	0.052223	8.653947	4.97E-18	1.25E-16	FAM3C	protein_cc
ENSG0000(	2829.936	-0.30364	0.04405	-6.89312	5.46E-12	5.04E-11	VPS13B	protein_cc
ENSG0000(	2828.78	0.042443	0.035393	1.199193	0.230453	0.314448	LAS1L	protein_cc
ENSG0000(	2828.015	0.151023	0.07841	1.92608	0.054094	0.090526	SLC40A1	protein_cc
ENSG0000(	2827.899	0.313877	0.130938	2.397137	0.016524	0.031397	COL5A2	protein_cc
ENSG0000(	2827.26	-0.3879	0.052692	-7.36151	1.82E-13	2.12E-12	RHOBTB2	protein_cc
ENSG0000(	2826.451	-0.33397	0.049158	-6.79372	1.09E-11	9.62E-11	TMEM170	protein_cc
ENSG0000(	2825.931	-0.13249	0.063502	-2.08644	0.036939	0.064653	MEIS3	protein_cc
ENSG0000(	2825.171	-0.17924	0.05001	-3.58418	0.000338	0.000902	BRPF3	protein_cc
ENSG0000(	2824.848	0.023568	0.071508	0.329581	0.741716	0.805002	GABPB1-/	processed

ENSG0000(	2824.678	-0.04003	0.040572	-0.98672	0.323778	0.417924	CPT1C	protein_cc
ENSG0000(	2824.555	-0.26983	0.030914	-8.72835	2.58E-18	6.77E-17	FAM20B	protein_cc
ENSG0000(	2823.982	-0.03439	0.033898	-1.01458	0.310307	0.40355	MDC1	protein_cc
ENSG0000(	2823.543	0.105914	0.037056	2.858193	0.004261	0.009213	PIH1D1	protein_cc
ENSG0000(	2822.632	-0.0329	0.030754	-1.0697	0.284754	0.375532	FBXO9	protein_cc
ENSG0000(	2822.356	-0.25356	0.041976	-6.04055	1.54E-09	9.71E-09	TMEM8B	protein_cc
ENSG0000(	2822.254	0.006112	0.027869	0.219293	0.826422	0.872773	UBXN2B	protein_cc
ENSG0000(	2821.449	-0.27715	0.037163	-7.45755	8.81E-14	1.08E-12	ACVR1B	protein_cc
ENSG0000(	2821.251	0.043784	0.025437	1.721277	0.085201	0.134399	ATG3	protein_cc
ENSG0000(	2821.075	-0.04844	0.028	-1.73006	0.083619	0.132183	NOL6	protein_cc
ENSG0000(	2820.886	0.37643	0.040701	9.248711	2.27E-20	8.20E-19	COMMD7	protein_cc
ENSG0000(	2820.725	0.229737	0.030702	7.48281	7.28E-14	9.09E-13	SMIM7	protein_cc
ENSG0000(	2818.679	0.470137	0.042391	11.09036	1.40E-28	2.47E-26	BUD31	protein_cc
ENSG0000(	2818.195	0.065379	0.02745	2.381769	0.01723	0.032559	IKBKB	protein_cc
ENSG0000(	2817.605	0.071185	0.037806	1.882888	0.059716	0.098665	ULK3	protein_cc
ENSG0000(	2817.086	-0.03521	0.027711	-1.27073	0.203824	0.283792	KIAA1429	protein_cc
ENSG0000(	2816.913	0.149691	0.034123	4.386786	1.15E-05	3.93E-05	AKT1S1	protein_cc
ENSG0000(	2816.081	-0.12821	0.036299	-3.53203	0.000412	0.001082	MTMR1	protein_cc
ENSG0000(	2816.025	0.124439	0.046589	2.67101	0.007562	0.01553	GYS1	protein_cc
ENSG0000(	2814.328	0.122224	0.028248	4.326897	1.51E-05	5.07E-05	HMGXB3	protein_cc
ENSG0000(	2814.046	-0.07294	0.025367	-2.87522	0.004037	0.008776	RNF185	protein_cc
ENSG0000(	2812.605	0.156453	0.040227	3.889273	0.000101	0.000294	CEBPG	protein_cc
ENSG0000(	2812.466	-0.14832	0.042273	-3.50855	0.000451	0.001174	PCNT	protein_cc
ENSG0000(	2812.036	0.07398	0.095465	0.774949	0.43837	0.5353	MDFI	protein_cc
ENSG0000(	2810.866	0.051254	0.042098	1.217484	0.22342	0.306489	VGLL4	protein_cc
ENSG0000(	2810.168	0.104326	0.037147	2.808473	0.004978	0.010611	PTDSS2	protein_cc
ENSG0000(	2810.081	-0.11002	0.052996	-2.07602	0.037892	0.066144	ARVCF	protein_cc
ENSG0000(	2810.018	-1.31185	0.132688	-9.88676	4.75E-23	2.82E-21	AK5	protein_cc
ENSG0000(	2809.69	-0.39793	0.052928	-7.51826	5.55E-14	7.05E-13	FGF2	protein_cc
ENSG0000(	2806.526	-0.01203	0.028207	-0.42656	0.669703	0.745073	ZNF148	protein_cc
ENSG0000(	2806.403	-0.07589	0.040643	-1.86737	0.06185	0.101697	SH3KBP1	protein_cc
ENSG0000(	2806.288	-0.2439	0.045665	-5.34114	9.24E-08	4.40E-07	SOBP	protein_cc
ENSG0000(	2804.225	-0.13957	0.036789	-3.79385	0.000148	0.00042	ACAP2	protein_cc
ENSG0000(	2803.31	-0.19074	0.055314	-3.44839	0.000564	0.001441	SESN1	protein_cc
ENSG0000(	2803.105	0.30815	0.116935	2.635215	0.008408	0.017092	KCNJ16	protein_cc
ENSG0000(	2802.958	-0.01418	0.021878	-0.64797	0.517007	0.60975	PROSC	protein_cc
ENSG0000(	2802.594	0.223544	0.040702	5.492231	3.97E-08	2.01E-07	CECR5	protein_cc
ENSG0000(	2800.973	0.029557	0.086791	0.340551	0.733442	0.798022	TPM2	protein_cc
ENSG0000(	2799.524	-0.27303	0.050582	-5.39784	6.74E-08	3.28E-07	FAM131A	protein_cc
ENSG0000(	2799.163	-0.41184	0.085418	-4.82148	1.42E-06	5.63E-06	GFPT2	protein_cc
ENSG0000(	2798.537	0.295992	0.083401	3.549034	0.000387	0.00102	TRIM22	protein_cc
ENSG0000(	2796.88	0.151226	0.034151	4.428148	9.50E-06	3.29E-05	NARF	protein_cc
ENSG0000(	2796.786	-0.92939	0.126822	-7.32826	2.33E-13	2.66E-12	PTPRT	protein_cc
ENSG0000(	2796.37	-1.17551	0.11387	-10.3233	5.53E-25	4.82E-23	RAB3A	protein_cc
ENSG0000(	2796.145	-0.1479	0.039892	-3.70744	0.000209	0.000578	FBRSL1	protein_cc
ENSG0000(	2795.563	-0.03199	0.043279	-0.73912	0.459832	0.556098	FKBP2	protein_cc
ENSG0000(	2795.228	0.254039	0.043019	5.905294	3.52E-09	2.10E-08	ITFG3	protein_cc
ENSG0000(	2793.536	-0.01194	0.021115	-0.56528	0.571883	0.659362	ASH2L	protein_cc
ENSG0000(	2792.866	-0.17008	0.087021	-1.95447	0.050646	0.085454	CMTM5	protein_cc
ENSG0000(	2792.428	0.115173	0.029804	3.864369	0.000111	0.000323	TMX3	protein_cc
ENSG0000(	2792.294	-0.02139	0.022319	-0.95857	0.337776	0.432651	PPP4R2	protein_cc
ENSG0000(	2791.957	-0.20089	0.036302	-5.53369	3.14E-08	1.61E-07	GFPT1	protein_cc
ENSG0000(	2791.341	0.060584	0.059278	1.022023	0.30677	0.399835	NUMBL	protein_cc
ENSG0000(	2790.692	0.02013	0.031197	0.64525	0.518765	0.611404	ASCC2	protein_cc
ENSG0000(	2790.41	-0.01862	0.060197	-0.30935	0.757052	0.817182	EPDR1	protein_cc
ENSG0000(	2789.932	-0.27828	0.049876	-5.57952	2.41E-08	1.26E-07	AGPAT4	protein_cc
ENSG0000(	2789.705	-0.05575	0.033358	-1.6712	0.094683	0.147344	NCK2	protein_cc
ENSG0000(	2788.893	-0.02022	0.029213	-0.69215	0.488845	0.583498	RSAD1	protein_cc
ENSG0000(	2788.398	-0.02464	0.039869	-0.61791	0.536636	0.62777	EPB41L5	protein_cc

ENSG0000(	2788.257	0.259881	0.033764	7.696927	1.39E-14	1.96E-13	CHCHD3	protein_cc
ENSG0000(	2788.117	-0.14846	0.023693	-6.26577	3.71E-10	2.58E-09	UBE3B	protein_cc
ENSG0000(	2787.028	0.112181	0.030839	3.637674	0.000275	0.000745	UBE2B	protein_cc
ENSG0000(	2786.915	-0.19021	0.045121	-4.21546	2.49E-05	8.05E-05	ANKRD12	protein_cc
ENSG0000(	2786.811	0.179861	0.04506	3.991633	6.56E-05	0.000198	TRAPPC3	protein_cc
ENSG0000(	2786.009	0.158346	0.040899	3.871632	0.000108	0.000314	NADK	protein_cc
ENSG0000(	2785.738	-0.01206	0.069267	-0.17407	0.861813	0.900437	SBK1	protein_cc
ENSG0000(	2785.221	-0.09554	0.031926	-2.99259	0.002766	0.006203	ATG2A	protein_cc
ENSG0000(	2784.189	0.146278	0.076892	1.902379	0.057122	0.09492	SHC1	protein_cc
ENSG0000(	2784.072	0.026198	0.034737	0.754195	0.450732	0.547477	MAGI2-AS	processed
ENSG0000(	2783.715	-0.02966	0.02531	-1.17173	0.241307	0.326851	SKIV2L2	protein_cc
ENSG0000(	2783.076	0.130327	0.040999	3.178795	0.001479	0.003497	COMMD6	protein_cc
ENSG0000(	2782.415	0.157337	0.03733	4.214793	2.50E-05	8.07E-05	TAF10	protein_cc
ENSG0000(	2781.99	0.152806	0.055124	2.77203	0.005571	0.011752	ZNF219	protein_cc
ENSG0000(	2780.491	-0.3032	0.06599	-4.59462	4.34E-06	1.59E-05	TGFBR2	protein_cc
ENSG0000(	2780.065	-0.0655	0.054941	-1.19216	0.233199	0.31752	PLCD1	protein_cc
ENSG0000(	2779.812	-0.01635	0.027438	-0.59572	0.551365	0.641206	SYAP1	protein_cc
ENSG0000(	2779.585	-0.05326	0.116665	-0.45651	0.648024	0.727072	LAMB1	protein_cc
ENSG0000(	2779.47	0.455614	0.101064	4.508155	6.54E-06	2.33E-05	MT1E	protein_cc
ENSG0000(	2779.286	0.00808	0.033188	0.243455	0.807653	0.858362	POMT2	protein_cc
ENSG0000(	2778.639	0.489265	0.096923	5.047978	4.47E-07	1.91E-06	GADD45A	protein_cc
ENSG0000(	2778.245	1.121477	0.119528	9.382555	6.44E-21	2.55E-19	BCAT1	protein_cc
ENSG0000(	2776.616	-0.18259	0.032175	-5.67475	1.39E-08	7.54E-08	AKTIP	protein_cc
ENSG0000(	2776.593	0.513119	0.111736	4.592232	4.39E-06	1.60E-05	THBS4	protein_cc
ENSG0000(	2776.116	0.042513	0.037944	1.120407	0.26254	0.351117	ACADM	protein_cc
ENSG0000(	2773.553	0.026966	0.033179	0.812747	0.416363	0.513311	C9orf114	protein_cc
ENSG0000(	2773.53	0.045555	0.034905	1.305121	0.191852	0.269512	KIAA1430	protein_cc
ENSG0000(	2771.342	0.045546	0.023492	1.938815	0.052524	0.08823	DDA1	protein_cc
ENSG0000(	2771.258	0.019281	0.021958	0.878075	0.379903	0.476417	EIF4E2	protein_cc
ENSG0000(	2770.932	-0.05325	0.035849	-1.48543	0.137429	0.202829	DNAJC3	protein_cc
ENSG0000(	2769.877	0.237384	0.031545	7.525223	5.26E-14	6.72E-13	CCDC90B	protein_cc
ENSG0000(	2769.668	0.252224	0.042371	5.95274	2.64E-09	1.61E-08	NEU1	protein_cc
ENSG0000(	2769.667	-0.27588	0.052739	-5.23106	1.69E-07	7.71E-07	MFAP3L	protein_cc
ENSG0000(	2768.846	-0.16748	0.035451	-4.72436	2.31E-06	8.82E-06	TRIM33	protein_cc
ENSG0000(	2767.879	-0.45949	0.064593	-7.11362	1.13E-12	1.17E-11	RP11-617	antisense
ENSG0000(	2767.263	-0.0171	0.034181	-0.50041	0.616784	0.69978	PRPF38B	protein_cc
ENSG0000(	2767.163	-0.3959	0.084829	-4.66703	3.06E-06	1.14E-05	FAM189A:	protein_cc
ENSG0000(	2767.109	-0.17275	0.034786	-4.96612	6.83E-07	2.83E-06	ATXN1L	protein_cc
ENSG0000(	2766.691	-0.14534	0.036728	-3.95721	7.58E-05	0.000226	GOPC	protein_cc
ENSG0000(	2766.372	0.243549	0.053349	4.565225	4.99E-06	1.81E-05	HDAC1	protein_cc
ENSG0000(	2765.25	0.136056	0.027692	4.913191	8.96E-07	3.65E-06	C7orf55-L	protein_cc
ENSG0000(	2763.647	0.175138	0.048083	3.642386	0.00027	0.000733	ASNS	protein_cc
ENSG0000(	2761.724	0.066926	0.040631	1.647168	0.099524	0.153923	SMDT1	protein_cc
ENSG0000(	2761.184	-0.3876	0.095914	-4.04108	5.32E-05	0.000163	NKD1	protein_cc
ENSG0000(	2761.046	-0.5044	0.058053	-8.68865	3.67E-18	9.39E-17	NIPA1	protein_cc
ENSG0000(	2760.782	-0.32494	0.04739	-6.85673	7.05E-12	6.41E-11	DZIP3	protein_cc
ENSG0000(	2760.658	-0.06898	0.036808	-1.87408	0.06092	0.100367	PGM3	protein_cc
ENSG0000(	2760.358	-0.02405	0.029164	-0.8246	0.409598	0.506473	CTR9	protein_cc
ENSG0000(	2760.277	-0.36226	0.062725	-5.77539	7.68E-09	4.34E-08	ACOT7	protein_cc
ENSG0000(	2758.846	0.291185	0.042201	6.899945	5.20E-12	4.82E-11	TMEM258	protein_cc
ENSG0000(	2758.352	0.10191	0.130338	0.78189	0.434279	0.531305	HLA-DQB	protein_cc
ENSG0000(	2756.44	-0.03853	0.03405	-1.13171	0.257756	0.345692	ZNF362	protein_cc
ENSG0000(	2755.687	-0.08698	0.037572	-2.31507	0.020609	0.038251	LETMD1	protein_cc
ENSG0000(	2755.384	-0.3333	0.044782	-7.44276	9.86E-14	1.20E-12	PDCD4	protein_cc
ENSG0000(	2755.354	-0.14132	0.032697	-4.32228	1.54E-05	5.17E-05	REPS1	protein_cc
ENSG0000(	2754.783	-0.04154	0.094343	-0.44031	0.659712	0.736782	RASL12	protein_cc
ENSG0000(	2754.715	-0.0724	0.02818	-2.56925	0.010192	0.020315	CCAR1	protein_cc
ENSG0000(	2754.443	-0.12681	0.048512	-2.61408	0.008947	0.01807	ZNF827	protein_cc
ENSG0000(	2754.001	0.403339	0.047422	8.505223	1.81E-17	4.16E-16	POLR2J	protein_cc

ENSG0000(	2753.769	-0.10545	0.049354	-2.13659	0.032631	0.057857	BAZ2B	protein_cc
ENSG0000(	2752.659	0.36527	0.045279	8.067116	7.20E-16	1.27E-14	C11orf31	protein_cc
ENSG0000(	2751.572	-0.02066	0.035271	-0.58583	0.55799	0.647136	STUB1	protein_cc
ENSG0000(	2751.521	0.23681	0.037097	6.383611	1.73E-10	1.27E-09	PPIL1	protein_cc
ENSG0000(	2751.507	0.118066	0.045782	2.578888	0.009912	0.019821	C6orf1	protein_cc
ENSG0000(	2750.63	-0.0226	0.033243	-0.6798	0.496631	0.590768	SECISBP2	protein_cc
ENSG0000(	2749.979	-0.04015	0.022408	-1.79171	0.07318	0.117829	GOSR1	protein_cc
ENSG0000(	2749.255	0.026305	0.026762	0.982924	0.325645	0.419806	KAT8	protein_cc
ENSG0000(	2749.244	-0.08263	0.047645	-1.73429	0.082867	0.1311	ARL2	protein_cc
ENSG0000(	2749.071	0.125507	0.032924	3.812028	0.000138	0.000393	PRR14	protein_cc
ENSG0000(	2748.014	0.032156	0.034816	0.92361	0.35569	0.451295	SHARPIN	protein_cc
ENSG0000(	2747.995	0.574576	0.074692	7.692608	1.44E-14	2.03E-13	PSMB8	protein_cc
ENSG0000(	2747.8	-0.1547	0.032104	-4.81871	1.44E-06	5.70E-06	MON2	protein_cc
ENSG0000(	2745.879	-0.05047	0.032388	-1.55819	0.119188	0.179707	HSDL1	protein_cc
ENSG0000(	2745.398	0.069399	0.031189	2.225117	0.026073	0.047294	SPAG7	protein_cc
ENSG0000(	2745.313	1.576598	0.161324	9.772886	1.47E-22	7.89E-21	METTL7B	protein_cc
ENSG0000(	2744.832	-0.11031	0.036804	-2.99736	0.002723	0.006117	STK39	protein_cc
ENSG0000(	2744.25	0.126684	0.030099	4.208832	2.57E-05	8.28E-05	TM2D2	protein_cc
ENSG0000(	2742.422	0.479748	0.076853	6.242391	4.31E-10	2.97E-09	TMEM255	protein_cc
ENSG0000(	2739.775	-0.38113	0.060197	-6.33139	2.43E-10	1.74E-09	ROGDI	protein_cc
ENSG0000(	2739.739	-0.25827	0.04062	-6.35814	2.04E-10	1.48E-09	BLOC1S2	protein_cc
ENSG0000(	2739.049	-0.12417	0.027059	-4.58897	4.45E-06	1.63E-05	EPS15L1	protein_cc
ENSG0000(	2738.715	-0.0737	0.031765	-2.32	0.020341	0.037802	AQR	protein_cc
ENSG0000(	2738.59	0.187873	0.034812	5.396768	6.79E-08	3.30E-07	MRPS34	protein_cc
ENSG0000(	2737.985	0.218586	0.065658	3.329171	0.000871	0.002147	MDM4	protein_cc
ENSG0000(	2737.501	0.059903	0.051942	1.15326	0.248804	0.335612	PLK1S1	processed
ENSG0000(	2736.924	-0.08051	0.039109	-2.0585	0.039543	0.068686	MRPL43	protein_cc
ENSG0000(	2736.843	0.271247	0.065468	4.143198	3.42E-05	0.000108	CYB5R1	protein_cc
ENSG0000(	2736.694	0.044208	0.035414	1.248318	0.211915	0.293229	UPF3A	protein_cc
ENSG0000(	2736.676	0.228854	0.039125	5.849347	4.94E-09	2.87E-08	SDHD	protein_cc
ENSG0000(	2736.041	-0.16897	0.036728	-4.6006	4.21E-06	1.54E-05	MPP5	protein_cc
ENSG0000(	2735.59	0.023199	0.036053	0.643478	0.519914	0.612354	STXBP3	protein_cc
ENSG0000(	2735.025	-0.25783	0.036611	-7.04242	1.89E-12	1.89E-11	CSTF2T	protein_cc
ENSG0000(	2734.56	-0.00212	0.100413	-0.02115	0.98313	0.98849	CTSC	protein_cc
ENSG0000(	2734.312	0.144987	0.09727	1.490573	0.136074	0.201125	HLA-DMA	protein_cc
ENSG0000(	2732.709	0.133981	0.024265	5.521507	3.36E-08	1.72E-07	NAE1	protein_cc
ENSG0000(	2732.132	0.116475	0.03389	3.436907	0.000588	0.001499	GTF3C6	protein_cc
ENSG0000(	2731.106	-0.25831	0.045647	-5.65887	1.52E-08	8.21E-08	POU6F1	protein_cc
ENSG0000(	2730.955	-0.16707	0.027277	-6.1249	9.07E-10	5.96E-09	XPNPEP1	protein_cc
ENSG0000(	2730.873	-0.07009	0.036272	-1.93229	0.053324	0.089403	CUL9	protein_cc
ENSG0000(	2730.598	-0.34408	0.053319	-6.45334	1.09E-10	8.29E-10	WDR47	protein_cc
ENSG0000(	2729.777	0.005703	0.034253	0.166489	0.867772	0.905192	CDC14B	protein_cc
ENSG0000(	2729.714	-0.0938	0.04031	-2.32691	0.01997	0.037174	LRRC8D	protein_cc
ENSG0000(	2728.994	0.007514	0.029077	0.258423	0.796081	0.848982	PSME4	protein_cc
ENSG0000(	2726.744	0.081186	0.036967	2.196179	0.028079	0.050522	CCNL1	protein_cc
ENSG0000(	2726.37	0.165822	0.027904	5.942563	2.81E-09	1.70E-08	DKC1	protein_cc
ENSG0000(	2726.057	0.64093	0.101602	6.308223	2.82E-10	2.00E-09	OAS3	protein_cc
ENSG0000(	2725.245	0.005771	0.037067	0.155694	0.876275	0.911356	AIG1	protein_cc
ENSG0000(	2725.209	-0.00844	0.022108	-0.38161	0.702753	0.772935	ZC3H7A	protein_cc
ENSG0000(	2724.641	-0.34596	0.048564	-7.12373	1.05E-12	1.09E-11	FAM53B	protein_cc
ENSG0000(	2723.364	-0.28906	0.031699	-9.11895	7.59E-20	2.52E-18	BICD2	protein_cc
ENSG0000(	2722.182	-0.04034	0.030096	-1.3404	0.180115	0.255415	WDR59	protein_cc
ENSG0000(	2721.196	-0.5515	0.073019	-7.55283	4.26E-14	5.51E-13	SHANK3	protein_cc
ENSG0000(	2720.728	0.26241	0.046182	5.682114	1.33E-08	7.24E-08	C19orf70	protein_cc
ENSG0000(	2720.553	0.110962	0.023365	4.749088	2.04E-06	7.86E-06	PSMD12	protein_cc
ENSG0000(	2720.157	0.11018	0.024449	4.506511	6.59E-06	2.34E-05	CDK13	protein_cc
ENSG0000(	2719.65	-0.18452	0.049297	-3.743	0.000182	0.000508	PLEKHO2	protein_cc
ENSG0000(	2719.584	0.064469	0.026687	2.415726	0.015704	0.029997	DHX8	protein_cc
ENSG0000(	2719.486	-0.3606	0.048509	-7.43374	1.06E-13	1.28E-12	DACT3	protein_cc

ENSG0000(	2719.413	0.105694	0.065125	1.622938	0.104603	0.1606	SLC20A1	protein_co
ENSG0000(	2719.142	-0.09547	0.029559	-3.22985	0.001239	0.002969	PPP1R2	protein_co
ENSG0000(	2719.077	-0.72124	0.072814	-9.90511	3.96E-23	2.37E-21	SH3PXD2/	protein_co
ENSG0000(	2718.807	0.054639	0.041714	1.309855	0.190245	0.267557	COPRS	protein_co
ENSG0000(	2718.603	-0.35229	0.037096	-9.49677	2.16E-21	9.44E-20	ANKS1A	protein_co
ENSG0000(	2718.495	-0.15027	0.031065	-4.83741	1.32E-06	5.22E-06	HEXIM1	protein_co
ENSG0000(	2716.128	0.093302	0.050104	1.862169	0.062579	0.102772	ERAP1	protein_co
ENSG0000(	2716.088	0.126821	0.042286	2.999106	0.002708	0.006087	PAXBP1	protein_co
ENSG0000(	2715.876	0.011805	0.05895	0.200262	0.841276	0.884643	MVD	protein_co
ENSG0000(	2715.142	-0.20732	0.035526	-5.83587	5.35E-09	3.10E-08	AGO1	protein_co
ENSG0000(	2714.569	-0.16676	0.044435	-3.753	0.000175	0.00049	TPP2	protein_co
ENSG0000(	2714.037	-1.05424	0.142798	-7.3827	1.55E-13	1.83E-12	CALN1	protein_co
ENSG0000(	2712.602	0.011998	0.055812	0.214977	0.829785	0.875605	SLC18B1	protein_co
ENSG0000(	2712	-0.81699	0.125942	-6.48704	8.75E-11	6.74E-10	KCNIP2	protein_co
ENSG0000(	2711.103	0.098514	0.04496	2.191146	0.028441	0.051129	ZC3HAV1	protein_co
ENSG0000(	2710.248	-0.07825	0.029856	-2.62102	0.008767	0.017742	CYTH3	protein_co
ENSG0000(	2710.142	-0.06108	0.024691	-2.47391	0.013364	0.025949	ELP3	protein_co
ENSG0000(	2709.854	0.04999	0.029725	1.681766	0.092614	0.144541	FKBP3	protein_co
ENSG0000(	2709.838	-0.12832	0.055712	-2.30327	0.021264	0.039347	KLHL9	protein_co
ENSG0000(	2709.824	0.417653	0.074962	5.571508	2.53E-08	1.32E-07	PARP9	protein_co
ENSG0000(	2709.672	0.478888	0.058153	8.234913	1.80E-16	3.50E-15	MAD2L2	protein_co
ENSG0000(	2707.788	-0.01144	0.097544	-0.11729	0.906631	0.933858	CTSS	protein_co
ENSG0000(	2707.687	0.249338	0.048669	5.123181	3.00E-07	1.32E-06	FEM1C	protein_co
ENSG0000(	2705.48	-0.09254	0.047538	-1.94661	0.051581	0.086829	OSBPL9	protein_co
ENSG0000(	2704.088	0.275483	0.033211	8.294873	1.09E-16	2.19E-15	RUVBL1	protein_co
ENSG0000(	2703.878	0.159153	0.031362	5.074629	3.88E-07	1.67E-06	MRPS23	protein_co
ENSG0000(	2703.836	-0.23317	0.049609	-4.70003	2.60E-06	9.84E-06	SLC12A6	protein_co
ENSG0000(	2703.508	0.130578	0.049462	2.639998	0.008291	0.016878	LIMS1	protein_co
ENSG0000(	2703.469	-0.26615	0.069375	-3.83642	0.000125	0.000359	ACSL1	protein_co
ENSG0000(	2701.557	0.196999	0.031876	6.180144	6.40E-10	4.30E-09	MSH6	protein_co
ENSG0000(	2701.519	0.13687	0.05766	2.37373	0.017609	0.03319	FAM127B	protein_co
ENSG0000(	2701.357	0.194887	0.027748	7.023407	2.17E-12	2.14E-11	MRPL42	protein_co
ENSG0000(	2701.228	0.055526	0.025162	2.206691	0.027336	0.049318	ACAD8	protein_co
ENSG0000(	2701.226	-0.00689	0.028737	-0.23975	0.810524	0.860602	MED22	protein_co
ENSG0000(	2701	0.135968	0.037701	3.606464	0.00031	0.000833	C20orf27	protein_co
ENSG0000(	2700.799	-0.06877	0.073568	-0.93475	0.349916	0.445357	CRB1	protein_co
ENSG0000(	2700.791	0.503794	0.064468	7.814685	5.51E-15	8.41E-14	TWSG1	protein_co
ENSG0000(	2700.438	-0.07767	0.026664	-2.91283	0.003582	0.007862	MAP3K2	protein_co
ENSG0000(	2700.379	0.021248	0.030747	0.691068	0.489523	0.584036	ECHDC1	protein_co
ENSG0000(	2699.619	0.115251	0.022426	5.139148	2.76E-07	1.22E-06	C11orf57	protein_co
ENSG0000(	2699.37	0.27736	0.037042	7.487672	7.01E-14	8.79E-13	PDZD11	protein_co
ENSG0000(	2699.276	0.0254	0.030502	0.832723	0.405001	0.501876	SAMM50	protein_co
ENSG0000(	2698.825	-0.0593	0.02685	-2.20849	0.02721	0.04912	RMND5B	protein_co
ENSG0000(	2698.651	0.015357	0.02675	0.574091	0.565906	0.653938	SH3BP5L	protein_co
ENSG0000(	2698.531	0.820447	0.075105	10.92398	8.85E-28	1.30E-25	TMEM106	protein_co
ENSG0000(	2698.267	0.066454	0.022977	2.892173	0.003826	0.008351	SMPD4	protein_co
ENSG0000(	2697.299	0.26049	0.02537	10.26785	9.84E-25	8.19E-23	RQCD1	protein_co
ENSG0000(	2696.605	-0.16767	0.034703	-4.83159	1.35E-06	5.36E-06	MARK4	protein_co
ENSG0000(	2696.193	-0.12055	0.028142	-4.28382	1.84E-05	6.09E-05	MCMBP	protein_co
ENSG0000(	2696.161	-0.24454	0.043876	-5.57332	2.50E-08	1.30E-07	ROCK1	protein_co
ENSG0000(	2695.253	-0.41119	0.046859	-8.77514	1.71E-18	4.58E-17	PRDM2	protein_co
ENSG0000(	2695.019	0.13346	0.032279	4.134613	3.56E-05	0.000112	UNK	protein_co
ENSG0000(	2693.7	0.214153	0.038487	5.564249	2.63E-08	1.37E-07	ABHD17A	protein_co
ENSG0000(	2693.676	-0.21607	0.09777	-2.20996	0.027108	0.048959	RCOR2	protein_co
ENSG0000(	2692.82	0.154195	0.099546	1.548985	0.121385	0.182417	S1PR3	protein_co
ENSG0000(	2692.745	0.114469	0.044179	2.591	0.00957	0.019205	TMEM175	protein_co
ENSG0000(	2692.73	0.114781	0.028914	3.969788	7.19E-05	0.000216	TMEM115	protein_co
ENSG0000(	2692.461	0.237877	0.038209	6.225686	4.79E-10	3.28E-09	SEC61B	protein_co
ENSG0000(	2691.914	-0.21311	0.039408	-5.4079	6.38E-08	3.12E-07	PRR12	protein_co

ENSG0000(	2691.005	-0.50736	0.076205	-6.65773	2.78E-11	2.31E-10	FRMPD3	protein_cc
ENSG0000(	2689.105	0.005547	0.037231	0.148981	0.881569	0.91538	KDM4A	protein_cc
ENSG0000(	2688.737	-0.30947	0.050196	-6.16531	7.03E-10	4.70E-09	ULK2	protein_cc
ENSG0000(	2688.187	-0.21535	0.035299	-6.10078	1.06E-09	6.86E-09	LNPEP	protein_cc
ENSG0000(	2687.314	-0.25137	0.037426	-6.71638	1.86E-11	1.59E-10	ATP6V0C	protein_cc
ENSG0000(	2686.992	0.241432	0.040333	5.985888	2.15E-09	1.33E-08	SPPL2A	protein_cc
ENSG0000(	2686.877	-0.34886	0.109477	-3.18663	0.001439	0.003409	ERBB3	protein_cc
ENSG0000(	2686.501	-0.03131	0.107359	-0.29162	0.770581	0.828155	GDAP1L1	protein_cc
ENSG0000(	2686.063	-0.17171	0.02989	-5.7448	9.20E-09	5.13E-08	DNAJC13	protein_cc
ENSG0000(	2685.802	0.038968	0.025846	1.507693	0.131633	0.195503	PYGO2	protein_cc
ENSG0000(	2684.904	0.094488	0.029594	3.192836	0.001409	0.003343	EMD	protein_cc
ENSG0000(	2684.107	0.63779	0.069758	9.142905	6.08E-20	2.06E-18	SNRPE	protein_cc
ENSG0000(	2683.732	-0.01727	0.089635	-0.19262	0.847256	0.889385	CD53	protein_cc
ENSG0000(	2683.068	-0.08528	0.053513	-1.59355	0.111036	0.169121	P4HA1	protein_cc
ENSG0000(	2682.262	-0.02173	0.027254	-0.79732	0.425267	0.522362	TBC1D15	protein_cc
ENSG0000(	2681.603	0.345171	0.037135	9.295115	1.47E-20	5.49E-19	CBFB	protein_cc
ENSG0000(	2681.502	-0.04677	0.0261	-1.79191	0.073148	0.117795	CHD1L	protein_cc
ENSG0000(	2680.551	-0.29124	0.079023	-3.68545	0.000228	0.000626	KIAA1755	protein_cc
ENSG0000(	2677.844	-0.13039	0.051062	-2.55365	0.01066	0.021161	RAI1	protein_cc
ENSG0000(	2676.349	-0.08107	0.043256	-1.87411	0.060915	0.100364	PELI1	protein_cc
ENSG0000(	2675.842	0.325278	0.036874	8.821358	1.13E-18	3.11E-17	COMMD4	protein_cc
ENSG0000(	2675.351	0.147077	0.033916	4.336495	1.45E-05	4.87E-05	C1orf123	protein_cc
ENSG0000(	2674.432	-0.24569	0.03625	-6.77788	1.22E-11	1.07E-10	FBXO18	protein_cc
ENSG0000(	2672.966	0.111971	0.049499	2.262068	0.023693	0.043376	LRP5	protein_cc
ENSG0000(	2672.039	0.04129	0.041844	0.986761	0.32376	0.417915	PML	protein_cc
ENSG0000(	2671.931	0.221331	0.03345	6.616836	3.67E-11	2.99E-10	PLEKHJ1	protein_cc
ENSG0000(	2671.15	-0.61087	0.062629	-9.75384	1.78E-22	9.39E-21	HIVEP2	protein_cc
ENSG0000(	2671.069	-0.14522	0.03441	-4.22028	2.44E-05	7.90E-05	EDEM3	protein_cc
ENSG0000(	2669.868	-0.42445	0.070049	-6.05933	1.37E-09	8.73E-09	PRKX	protein_cc
ENSG0000(	2669.625	0.195746	0.041031	4.770671	1.84E-06	7.12E-06	MRPL37	protein_cc
ENSG0000(	2669.489	-0.03431	0.046485	-0.73817	0.460411	0.556618	TMEM109	protein_cc
ENSG0000(	2668.865	0.089473	0.031296	2.858896	0.004251	0.009195	BCKDK	protein_cc
ENSG0000(	2668.863	0.030118	0.041648	0.723164	0.469579	0.565475	ZNF608	protein_cc
ENSG0000(	2667.755	-0.01463	0.032758	-0.44661	0.655158	0.733023	BMI1	protein_cc
ENSG0000(	2666.882	0.117154	0.027194	4.308062	1.65E-05	5.49E-05	LEMD2	protein_cc
ENSG0000(	2666.56	-0.01628	0.033763	-0.4821	0.629733	0.711128	PATL1	protein_cc
ENSG0000(	2665.858	-0.01475	0.021973	-0.67111	0.50215	0.596058	DSCR3	protein_cc
ENSG0000(	2665.55	-0.46705	0.069819	-6.68943	2.24E-11	1.89E-10	GRID1	protein_cc
ENSG0000(	2664.581	0.562763	0.125567	4.481765	7.40E-06	2.61E-05	GBP1	protein_cc
ENSG0000(	2663.234	-0.01281	0.035614	-0.35957	0.719172	0.78622	RAB8B	protein_cc
ENSG0000(	2662.321	0.008096	0.02561	0.316137	0.751898	0.813177	MRPL10	protein_cc
ENSG0000(	2661.682	-0.66939	0.106856	-6.26443	3.74E-10	2.61E-09	ANK3	protein_cc
ENSG0000(	2659.55	-0.20443	0.071387	-2.86365	0.004188	0.009074	PLSCR4	protein_cc
ENSG0000(	2659.339	-0.04377	0.053296	-0.82121	0.411528	0.508446	NHSL1	protein_cc
ENSG0000(	2659.012	0.20294	0.050738	3.99974	6.34E-05	0.000192	CMTM6	protein_cc
ENSG0000(	2658.459	-0.15715	0.052393	-2.99943	0.002705	0.006082	BAHCC1	protein_cc
ENSG0000(	2657.487	0.146827	0.041713	3.519913	0.000432	0.001129	PAN2	protein_cc
ENSG0000(	2657.171	-0.02375	0.030985	-0.76657	0.443336	0.540121	IREB2	protein_cc
ENSG0000(	2656.729	-0.01328	0.02879	-0.46137	0.644532	0.72414	VHL	protein_cc
ENSG0000(	2656.39	-0.12579	0.03636	-3.45959	0.000541	0.001387	GRAMD4	protein_cc
ENSG0000(	2656.055	-0.00593	0.022156	-0.26779	0.788864	0.843113	NOL7	protein_cc
ENSG0000(	2655.834	-0.80736	0.108318	-7.45362	9.08E-14	1.11E-12	KCNJ9	protein_cc
ENSG0000(	2655.237	0.71689	0.110045	6.514511	7.29E-11	5.68E-10	EFEMP2	protein_cc
ENSG0000(	2653.923	-0.04114	0.05797	-0.70962	0.477941	0.573396	DTX1	protein_cc
ENSG0000(	2653.348	-0.32368	0.069295	-4.67108	3.00E-06	1.12E-05	TGM2	protein_cc
ENSG0000(	2652.853	0.228899	0.02976	7.691563	1.45E-14	2.04E-13	SLC35B1	protein_cc
ENSG0000(	2652.723	0.278581	0.033856	8.228314	1.90E-16	3.68E-15	MMADHC	protein_cc
ENSG0000(	2651.9	0.097518	0.036873	2.64472	0.008176	0.016668	CERS4	protein_cc
ENSG0000(	2651.337	0.681173	0.062511	10.89678	1.19E-27	1.71E-25	STK17A	protein_cc

ENSG0000(	2650.543	0.085322	0.040242	2.120239	0.033986	0.060002	PTPN18	protein_cc
ENSG0000(	2650.052	0.861953	0.126326	6.823229	8.90E-12	7.96E-11	PLAT	protein_cc
ENSG0000(	2649.705	-0.05359	0.078988	-0.67849	0.497462	0.591517	YAP1	protein_cc
ENSG0000(	2649.413	-0.10128	0.035665	-2.83984	0.004514	0.009699	IRGQ	protein_cc
ENSG0000(	2648.765	-0.0202	0.027049	-0.74684	0.45516	0.551795	HMCES	protein_cc
ENSG0000(	2648.68	0.07286	0.028029	2.599439	0.009338	0.018777	CTNNBL1	protein_cc
ENSG0000(	2648.016	-0.01467	0.040311	-0.36391	0.715925	0.783691	SYT17	protein_cc
ENSG0000(	2647.704	0.230605	0.037727	6.112483	9.81E-10	6.41E-09	ABCB8	protein_cc
ENSG0000(	2647.546	0.001817	0.026276	0.069153	0.944868	0.961539	MRPS14	protein_cc
ENSG0000(	2646.546	0.304336	0.05222	5.827925	5.61E-09	3.24E-08	CHPF2	protein_cc
ENSG0000(	2646.4	-0.22807	0.035625	-6.40183	1.54E-10	1.13E-09	SFXN3	protein_cc
ENSG0000(	2644.44	-0.06924	0.03971	-1.74358	0.081232	0.128916	PCYT2	protein_cc
ENSG0000(	2644.42	-0.16325	0.030228	-5.40066	6.64E-08	3.24E-07	ARFGEF1	protein_cc
ENSG0000(	2644.213	0.060674	0.049364	1.229103	0.219033	0.301416	C9orf16	protein_cc
ENSG0000(	2643.865	-0.0951	0.024947	-3.81194	0.000138	0.000393	GGA3	protein_cc
ENSG0000(	2643.502	0.058221	0.031457	1.850843	0.064192	0.105081	ZNF768	protein_cc
ENSG0000(	2643.302	-0.0055	0.025755	-0.2137	0.830779	0.876429	BNIP2	protein_cc
ENSG0000(	2643.201	0.241246	0.036627	6.586618	4.50E-11	3.62E-10	VPS29	protein_cc
ENSG0000(	2642.9	-0.21878	0.040862	-5.35418	8.59E-08	4.11E-07	UBE4A	protein_cc
ENSG0000(	2641.406	-0.19776	0.029567	-6.68839	2.26E-11	1.90E-10	AP5M1	protein_cc
ENSG0000(	2640.827	-0.52591	0.058145	-9.04482	1.50E-19	4.77E-18	IGIP	protein_cc
ENSG0000(	2640.004	-0.21976	0.032654	-6.73015	1.69E-11	1.45E-10	RMDN1	protein_cc
ENSG0000(	2639.964	0.254754	0.051349	4.961249	7.00E-07	2.90E-06	RPS27L	protein_cc
ENSG0000(	2639.401	-0.01371	0.024287	-0.56435	0.572516	0.659925	MAP3K7	protein_cc
ENSG0000(	2638.985	0.052449	0.030217	1.735756	0.082607	0.13077	KLHL12	protein_cc
ENSG0000(	2638.977	0.137399	0.02916	4.711851	2.45E-06	9.33E-06	TRAPPC4	protein_cc
ENSG0000(	2638.884	0.033438	0.031616	1.057643	0.290218	0.3816	PTPN1	protein_cc
ENSG0000(	2638.875	0.009925	0.02742	0.361965	0.717378	0.784718	RFX5	protein_cc
ENSG0000(	2638.565	0.177784	0.0313	5.679914	1.35E-08	7.33E-08	NABP2	protein_cc
ENSG0000(	2637.936	0.231661	0.040435	5.729265	1.01E-08	5.59E-08	PRPF31	protein_cc
ENSG0000(	2637.88	0.178784	0.040022	4.467146	7.93E-06	2.78E-05	HIGD2A	protein_cc
ENSG0000(	2637.854	0.699678	0.089631	7.806187	5.89E-15	8.93E-14	DUSP6	protein_cc
ENSG0000(	2636.692	0.003523	0.041485	0.084919	0.932325	0.952783	DR1	protein_cc
ENSG0000(	2636.019	-0.10654	0.0295	-3.6116	0.000304	0.000818	RNF103	protein_cc
ENSG0000(	2635.628	-0.0654	0.034825	-1.87801	0.060379	0.099601	ZNF621	protein_cc
ENSG0000(	2635.568	-0.05856	0.020274	-2.88842	0.003872	0.008445	UBE2G1	protein_cc
ENSG0000(	2634.97	0.131073	0.041143	3.185751	0.001444	0.003419	POM121	protein_cc
ENSG0000(	2634.726	-0.10047	0.023145	-4.34105	1.42E-05	4.78E-05	CCDC115	protein_cc
ENSG0000(	2634.638	-0.05307	0.0324	-1.63799	0.101425	0.156433	USP36	protein_cc
ENSG0000(	2633.023	-0.30471	0.034376	-8.86407	7.71E-19	2.18E-17	NUDT3	protein_cc
ENSG0000(	2632.313	0.329684	0.035971	9.165384	4.94E-20	1.70E-18	WDR61	protein_cc
ENSG0000(	2632.233	0.272806	0.032028	8.51762	1.63E-17	3.76E-16	MRPL4	protein_cc
ENSG0000(	2631.824	0.283952	0.076007	3.735868	0.000187	0.000521	PIP4K2C	protein_cc
ENSG0000(	2631.315	0.051441	0.031634	1.626139	0.10392	0.159678	HECTD3	protein_cc
ENSG0000(	2631.305	0.422828	0.055172	7.663805	1.81E-14	2.50E-13	DSEL	protein_cc
ENSG0000(	2630.95	-0.07584	0.036534	-2.07583	0.03791	0.066168	PAAF1	protein_cc
ENSG0000(	2629.98	-0.37432	0.04664	-8.0257	1.01E-15	1.74E-14	RABGAP1I	protein_cc
ENSG0000(	2629.771	0.104825	0.040082	2.615259	0.008916	0.018013	TRIM25	protein_cc
ENSG0000(	2629.381	0.009742	0.020854	0.467146	0.640395	0.720359	DDX19A	protein_cc
ENSG0000(	2629.153	-0.1878	0.039509	-4.75343	2.00E-06	7.71E-06	EPT1	protein_cc
ENSG0000(	2629.128	-0.00613	0.028179	-0.21769	0.827669	0.873836	PPIL2	protein_cc
ENSG0000(	2628.236	-0.05522	0.03721	-1.48389	0.137837	0.203366	NNT-AS1	antisense
ENSG0000(	2627.937	0.211798	0.088879	2.382988	0.017173	0.032474	CYBA	protein_cc
ENSG0000(	2627.745	-0.18321	0.036065	-5.08005	3.77E-07	1.63E-06	ADO	protein_cc
ENSG0000(	2627.295	0.003424	0.029478	0.116168	0.90752	0.934488	TCEB3	protein_cc
ENSG0000(	2626.493	0.127792	0.033287	3.839137	0.000123	0.000355	EBNA1BP2	protein_cc
ENSG0000(	2626.107	0.030338	0.041389	0.733007	0.463554	0.559678	2-Mar	protein_cc
ENSG0000(	2623.364	-0.28846	0.092612	-3.11471	0.001841	0.004276	AFAP1L2	protein_cc
ENSG0000(	2622.678	0.060746	0.028566	2.12652	0.03346	0.059168	ZNF512	protein_cc

ENSG0000	2622.61	-0.12466	0.029816	-4.18094	2.90E-05	9.28E-05	PHF20L1	protein_co
ENSG0000	2622.13	0.608135	0.060747	10.01099	1.36E-23	9.16E-22	ROMO1	protein_co
ENSG0000	2622.104	-0.83612	0.0933	-8.96167	3.20E-19	9.61E-18	KNDC1	protein_co
ENSG0000	2621.575	-0.30863	0.074245	-4.15695	3.23E-05	0.000102	AP3B2	protein_co
ENSG0000	2620.165	-0.02216	0.033975	-0.65215	0.514302	0.607187	NUP153	protein_co
ENSG0000	2619.834	-0.48627	0.047959	-10.1393	3.70E-24	2.74E-22	BTRC	protein_co
ENSG0000	2619.546	-0.19998	0.13582	-1.47235	0.140926	0.207298	GFRA1	protein_co
ENSG0000	2619.23	-0.44053	0.055651	-7.91598	2.45E-15	3.97E-14	MPP2	protein_co
ENSG0000	2618.985	0.020527	0.032011	0.641236	0.521369	0.613542	PSKH1	protein_co
ENSG0000	2617.206	0.125291	0.034755	3.604918	0.000312	0.000838	CSNK1G2	protein_co
ENSG0000	2617.084	-0.01946	0.024017	-0.81034	0.417744	0.51472	WRNIP1	protein_co
ENSG0000	2616.926	-0.22501	0.04134	-5.44294	5.24E-08	2.59E-07	SMIM13	protein_co
ENSG0000	2615.815	-0.75632	0.076931	-9.83107	8.27E-23	4.66E-21	MT-TP	Mt_tRNA
ENSG0000	2615.789	-0.20558	0.031663	-6.4928	8.43E-11	6.50E-10	DNAJB12	protein_co
ENSG0000	2615.641	-0.27831	0.06985	-3.98438	6.77E-05	0.000204	PPP1R9A	protein_co
ENSG0000	2615.417	-0.24915	0.041801	-5.96032	2.52E-09	1.54E-08	ABL2	protein_co
ENSG0000	2615.086	-0.21173	0.03354	-6.31264	2.74E-10	1.95E-09	WDR11	protein_co
ENSG0000	2614.594	0.027528	0.022318	1.233473	0.217399	0.299559	C16orf72	protein_co
ENSG0000	2614.432	0.178299	0.047286	3.77067	0.000163	0.000459	DNAJB9	protein_co
ENSG0000	2614.22	-0.30666	0.062726	-4.88895	1.01E-06	4.09E-06	ADAM23	protein_co
ENSG0000	2613.72	0.191637	0.063786	3.004358	0.002661	0.005996	PARVA	protein_co
ENSG0000	2611.107	-0.44642	0.076287	-5.85188	4.86E-09	2.83E-08	TMCC2	protein_co
ENSG0000	2610.868	-0.07642	0.037627	-2.03094	0.042261	0.072827	PTGES2	protein_co
ENSG0000	2610.816	-0.13374	0.030183	-4.43106	9.38E-06	3.25E-05	DIS3L	protein_co
ENSG0000	2610.495	0.227303	0.030884	7.359951	1.84E-13	2.14E-12	SF3B14	protein_co
ENSG0000	2609.669	-0.33845	0.030402	-11.1324	8.72E-29	1.62E-26	HIF1AN	protein_co
ENSG0000	2609.061	-0.15145	0.070013	-2.16314	0.03053	0.054493	ZMAT1	protein_co
ENSG0000	2609.048	-0.02542	0.031739	-0.8009	0.42319	0.520277	MAP3K13	protein_co
ENSG0000	2608.809	0.090444	0.027724	3.262307	0.001105	0.002672	NUP93	protein_co
ENSG0000	2608.642	0.152239	0.038605	3.943501	8.03E-05	0.000239	C17orf70	protein_co
ENSG0000	2608.368	-0.05549	0.029648	-1.87175	0.061241	0.100815	PPP2R5E	protein_co
ENSG0000	2607.664	0.248771	0.042715	5.823914	5.75E-09	3.31E-08	C5orf15	protein_co
ENSG0000	2606.779	0.107635	0.042998	2.50326	0.012306	0.024074	CEBPZ-AS	protein_co
ENSG0000	2606.313	0.067212	0.034934	1.923963	0.054359	0.090913	DRG1	protein_co
ENSG0000	2606.303	1.0014	0.126768	7.899494	2.80E-15	4.50E-14	SPRY4	protein_co
ENSG0000	2605.452	-0.11467	0.033077	-3.46676	0.000527	0.001354	OCRL	protein_co
ENSG0000	2605.335	-0.64521	0.097441	-6.62152	3.56E-11	2.91E-10	ARPP21	protein_co
ENSG0000	2605.226	-0.24091	0.036482	-6.60353	4.01E-11	3.25E-10	MAFG	protein_co
ENSG0000	2605.113	-0.08812	0.044899	-1.9627	0.049681	0.084029	KDM5A	protein_co
ENSG0000	2604.736	-0.08826	0.022838	-3.86458	0.000111	0.000323	VPS8	protein_co
ENSG0000	2604.613	0.151723	0.040693	3.728464	0.000193	0.000536	NDUFS6	protein_co
ENSG0000	2604.468	-0.06995	0.036248	-1.92967	0.053647	0.089869	DMWD	protein_co
ENSG0000	2603.502	0.034061	0.025087	1.357701	0.174559	0.248875	NSUN2	protein_co
ENSG0000	2603.033	-0.01309	0.027916	-0.46903	0.639048	0.719163	TCEAL8	protein_co
ENSG0000	2602.024	-0.01398	0.020944	-0.66769	0.504332	0.598147	ARMC8	protein_co
ENSG0000	2601.869	0.388321	0.042395	9.159648	5.21E-20	1.79E-18	CLPP	protein_co
ENSG0000	2601.51	-0.07928	0.032601	-2.43201	0.015015	0.028813	FAM98B	protein_co
ENSG0000	2601.464	0.086559	0.096492	0.897059	0.369688	0.466047	DGKG	protein_co
ENSG0000	2599.74	0.329088	0.047038	6.996154	2.63E-12	2.57E-11	PTPN9	protein_co
ENSG0000	2599.581	0.110618	0.028955	3.820345	0.000133	0.000381	TELO2	protein_co
ENSG0000	2599.263	-0.05764	0.02987	-1.92987	0.053623	0.089836	CSRNP2	protein_co
ENSG0000	2599.248	-0.15726	0.03766	-4.17585	2.97E-05	9.48E-05	SUCLA2	protein_co
ENSG0000	2598.902	-0.01874	0.023992	-0.78125	0.434653	0.531715	USP16	protein_co
ENSG0000	2598.412	0.085657	0.078736	1.087893	0.276642	0.366759	MEX3A	protein_co
ENSG0000	2598.387	0.196072	0.030125	6.508629	7.58E-11	5.89E-10	ARAF	protein_co
ENSG0000	2596.991	-0.76933	0.097182	-7.91642	2.44E-15	3.95E-14	P2RY12	protein_co
ENSG0000	2596.823	0.062813	0.02907	2.160729	0.030716	0.054787	GSR	protein_co
ENSG0000	2596.686	-0.17558	0.075949	-2.31181	0.020788	0.03855	EFNB3	protein_co
ENSG0000	2594.754	-0.12495	0.04388	-2.84757	0.004405	0.009494	ZDHHC20	protein_co

ENSG0000(	2593.427	0.292134	0.048712	5.997137	2.01E-09	1.25E-08	MFSD10	protein_cc
ENSG0000(	2593.255	-0.33832	0.054515	-6.20604	5.43E-10	3.68E-09	SLC12A2	protein_cc
ENSG0000(	2593.072	-0.15873	0.045037	-3.52444	0.000424	0.001111	ANGEL1	protein_cc
ENSG0000(	2592.577	0.594652	0.053377	11.14062	7.96E-29	1.48E-26	WDR34	protein_cc
ENSG0000(	2592.534	0.455454	0.088544	5.143819	2.69E-07	1.19E-06	IFI27	protein_cc
ENSG0000(	2591.764	-0.97285	0.110406	-8.81153	1.23E-18	3.38E-17	SLC24A2	protein_cc
ENSG0000(	2591.665	-0.13639	0.038404	-3.55139	0.000383	0.001011	BCCIP	protein_cc
ENSG0000(	2591.632	0.173226	0.112065	1.545772	0.12216	0.183443	HSPG2	protein_cc
ENSG0000(	2589.594	0.035303	0.032808	1.076057	0.281902	0.372536	TBL3	protein_cc
ENSG0000(	2588.431	-0.16602	0.047853	-3.46947	0.000521	0.001341	LPP	protein_cc
ENSG0000(	2588.367	0.053875	0.027761	1.940681	0.052297	0.087897	NCBP1	protein_cc
ENSG0000(	2588.29	0.471211	0.066741	7.060329	1.66E-12	1.67E-11	CREB5	protein_cc
ENSG0000(	2587.706	-0.13178	0.10693	-1.23237	0.217813	0.300028	NXPH1	protein_cc
ENSG0000(	2585.013	0.051762	0.036278	1.426816	0.153633	0.223353	GGT7	protein_cc
ENSG0000(	2584.594	0.300824	0.046241	6.505586	7.74E-11	6.00E-10	NDUFA11	protein_cc
ENSG0000(	2584.511	0.264032	0.035654	7.405312	1.31E-13	1.56E-12	PEX2	protein_cc
ENSG0000(	2584.163	-0.83248	0.092473	-9.00249	2.21E-19	6.81E-18	PCDH7	protein_cc
ENSG0000(	2582.758	-0.02553	0.031262	-0.81665	0.414127	0.511091	SETD1A	protein_cc
ENSG0000(	2582.503	-0.32698	0.044421	-7.36107	1.82E-13	2.12E-12	ACSL4	protein_cc
ENSG0000(	2582.421	0.097167	0.027435	3.541717	0.000398	0.001046	RUFY1	protein_cc
ENSG0000(	2582.322	-0.05183	0.04044	-1.2816	0.199981	0.279258	IFT140	protein_cc
ENSG0000(	2582.27	0.331448	0.048772	6.795878	1.08E-11	9.50E-11	SCAND1	protein_cc
ENSG0000(	2582.055	-0.13489	0.029165	-4.62512	3.74E-06	1.38E-05	VPS18	protein_cc
ENSG0000(	2581.592	0.086816	0.027508	3.155979	0.0016	0.003759	WDR5	protein_cc
ENSG0000(	2581.097	0.162045	0.033846	4.787726	1.69E-06	6.58E-06	CSNK2B	protein_cc
ENSG0000(	2580.909	-0.15167	0.028785	-5.26923	1.37E-07	6.35E-07	VPS26A	protein_cc
ENSG0000(	2580.859	0.071088	0.042397	1.67671	0.093599	0.145865	HDAC7	protein_cc
ENSG0000(	2579.748	0.204514	0.037941	5.390339	7.03E-08	3.41E-07	TOMM40	protein_cc
ENSG0000(	2578.39	0.264048	0.054079	4.882629	1.05E-06	4.22E-06	NDUFA3	protein_cc
ENSG0000(	2577.461	0.390634	0.069919	5.586938	2.31E-08	1.21E-07	TSPAN13	protein_cc
ENSG0000(	2577.23	0.120585	0.028781	4.189803	2.79E-05	8.96E-05	ZBED5	protein_cc
ENSG0000(	2577.152	-0.52414	0.066977	-7.82559	5.05E-15	7.76E-14	MICAL3	protein_cc
ENSG0000(	2575.945	0.359335	0.101796	3.529945	0.000416	0.001089	CNR1	protein_cc
ENSG0000(	2575.66	0.029957	0.051561	0.580989	0.561248	0.650086	SQLE	protein_cc
ENSG0000(	2575.325	-0.11935	0.032117	-3.71594	0.000202	0.00056	KCTD7	protein_cc
ENSG0000(	2574.742	-0.31044	0.05304	-5.8529	4.83E-09	2.82E-08	FLT1	protein_cc
ENSG0000(	2574.48	0.055044	0.029943	1.838257	0.066025	0.107665	TAF6	protein_cc
ENSG0000(	2572.334	0.086978	0.034785	2.500465	0.012403	0.024247	NDUFA8	protein_cc
ENSG0000(	2572.054	-0.03431	0.047273	-0.72579	0.467966	0.563814	SRGAP1	protein_cc
ENSG0000(	2571.737	0.067537	0.03338	2.023306	0.043042	0.074005	MED14	protein_cc
ENSG0000(	2571.016	-0.0344	0.031516	-1.0915	0.275054	0.365118	GINM1	protein_cc
ENSG0000(	2570.866	0.116878	0.035103	3.3296	0.00087	0.002144	FAF1	protein_cc
ENSG0000(	2570.717	-0.00409	0.038785	-0.10545	0.916016	0.940838	RAB3GAP:	protein_cc
ENSG0000(	2570.339	0.097112	0.03978	2.441204	0.014638	0.028186	LINC0096:	processed
ENSG0000(	2570.238	-0.42417	0.057884	-7.32793	2.34E-13	2.67E-12	NAP1L5	protein_cc
ENSG0000(	2569.258	0.002769	0.03355	0.082537	0.93422	0.954055	PCGF3	protein_cc
ENSG0000(	2568.662	0.378242	0.054618	6.925166	4.35E-12	4.10E-11	HYAL2	protein_cc
ENSG0000(	2567.843	-0.40577	0.037063	-10.9482	6.78E-28	1.01E-25	SNX18	protein_cc
ENSG0000(	2567.739	0.208861	0.036337	5.747937	9.03E-09	5.04E-08	DNAJB11	protein_cc
ENSG0000(	2567.693	0.093904	0.051437	1.825616	0.067908	0.11035	KIFC3	protein_cc
ENSG0000(	2567.447	0.202799	0.031985	6.340532	2.29E-10	1.65E-09	BABAM1	protein_cc
ENSG0000(	2566.691	-0.07276	0.089463	-0.8133	0.416046	0.513025	ADORA3	protein_cc
ENSG0000(	2565.814	0.259878	0.03532	7.357877	1.87E-13	2.17E-12	DPM2	protein_cc
ENSG0000(	2565.529	0.105015	0.03726	2.818415	0.004826	0.010309	MPC2	protein_cc
ENSG0000(	2564.809	-0.20782	0.057193	-3.63358	0.00028	0.000756	GPD1L	protein_cc
ENSG0000(	2564.353	0.265496	0.026711	9.939469	2.80E-23	1.75E-21	TMEM203	protein_cc
ENSG0000(	2563.916	-0.0056	0.024565	-0.22812	0.819555	0.867739	VEZT	protein_cc
ENSG0000(	2563.89	0.162738	0.031837	5.111615	3.19E-07	1.40E-06	PSMA3	protein_cc
ENSG0000(	2562.79	-0.51413	0.1453	-3.5384	0.000403	0.001058	CNDP1	protein_cc

ENSG0000(	2562.102	0.298122	0.036203	8.234723	1.80E-16	3.51E-15	NUP160	protein_cc
ENSG0000(	2561.964	-0.31872	0.055611	-5.73133	9.96E-09	5.53E-08	FN3K	protein_cc
ENSG0000(	2561.413	-0.20715	0.037264	-5.55913	2.71E-08	1.41E-07	R3HDM2	protein_cc
ENSG0000(	2560.662	0.00109	0.045826	0.023787	0.981023	0.986709	HIBADH	protein_cc
ENSG0000(	2560.174	-0.44021	0.079048	-5.56893	2.56E-08	1.33E-07	SELPLG	protein_cc
ENSG0000(	2560.008	0.173446	0.030451	5.695908	1.23E-08	6.71E-08	MRPS35	protein_cc
ENSG0000(	2559.137	0.104945	0.029731	3.529771	0.000416	0.00109	CEBPZ	protein_cc
ENSG0000(	2557.665	-0.31329	0.046025	-6.80699	9.97E-12	8.85E-11	R3HDM1	protein_cc
ENSG0000(	2557.436	-0.22437	0.046815	-4.79276	1.65E-06	6.43E-06	ZBED3	protein_cc
ENSG0000(	2555.58	-0.12246	0.037502	-3.26547	0.001093	0.002644	C12orf76	protein_cc
ENSG0000(	2555.236	-0.18351	0.041012	-4.47456	7.66E-06	2.70E-05	AHCTF1	protein_cc
ENSG0000(	2555.233	0.462469	0.111564	4.14531	3.39E-05	0.000107	PLEKHA4	protein_cc
ENSG0000(	2553.623	-0.05551	0.040265	-1.37867	0.167995	0.240946	CASD1	protein_cc
ENSG0000(	2553.539	-0.94391	0.122012	-7.73618	1.02E-14	1.49E-13	CNTNAP2	protein_cc
ENSG0000(	2552.997	-0.0649	0.034325	-1.89081	0.058649	0.097133	NEIL2	protein_cc
ENSG0000(	2552.937	-0.56349	0.057224	-9.84701	7.06E-23	4.06E-21	CSGALNA	protein_cc
ENSG0000(	2552.206	-0.2904	0.067544	-4.2994	1.71E-05	5.70E-05	SLC9A7	protein_cc
ENSG0000(	2551.923	0.297417	0.033464	8.887613	6.24E-19	1.79E-17	SNRPD1	protein_cc
ENSG0000(	2550.847	0.192961	0.038014	5.07612	3.85E-07	1.66E-06	SNRNP25	protein_cc
ENSG0000(	2550.58	-0.05193	0.023943	-2.16897	0.030085	0.05378	ZFP90	protein_cc
ENSG0000(	2549.894	-0.04425	0.11315	-0.39103	0.695774	0.767022	CDHR1	protein_cc
ENSG0000(	2549.695	0.112769	0.031053	3.631552	0.000282	0.000762	DVL2	protein_cc
ENSG0000(	2549.306	-0.21446	0.081495	-2.63158	0.008499	0.017261	IGSF21	protein_cc
ENSG0000(	2549.032	0.245236	0.031312	7.831989	4.80E-15	7.40E-14	UBE2D4	protein_cc
ENSG0000(	2549.014	-0.0291	0.031686	-0.91841	0.358404	0.454211	TMEM170	protein_cc
ENSG0000(	2548.747	0.156106	0.034874	4.476325	7.59E-06	2.67E-05	SNRPA	protein_cc
ENSG0000(	2548.739	0.022985	0.024827	0.925803	0.354548	0.450153	ZNF259	protein_cc
ENSG0000(	2548.426	0.027785	0.026022	1.067727	0.285644	0.376543	OGFOD1	protein_cc
ENSG0000(	2547.19	-0.56611	0.077644	-7.29107	3.07E-13	3.46E-12	PHACTR1	protein_cc
ENSG0000(	2546.788	-0.51701	0.077125	-6.70356	2.03E-11	1.72E-10	HCN2	protein_cc
ENSG0000(	2546.746	0.06685	0.025492	2.622415	0.008731	0.017676	STX5	protein_cc
ENSG0000(	2545.837	0.010928	0.024752	0.441508	0.658845	0.73604	ZNF271	pseudoge
ENSG0000(	2545.032	0.053802	0.044741	1.202505	0.229168	0.313069	FIP1L1	protein_cc
ENSG0000(	2544.851	0.166257	0.039736	4.184002	2.86E-05	9.17E-05	WBP5	protein_cc
ENSG0000(	2544.737	-0.08228	0.021187	-3.8836	0.000103	0.0003	ZDHHC7	protein_cc
ENSG0000(	2543.574	-0.13064	0.032804	-3.98248	6.82E-05	0.000205	ARID4B	protein_cc
ENSG0000(	2543.282	-0.22021	0.06353	-3.46627	0.000528	0.001356	GALC	protein_cc
ENSG0000(	2542.757	-0.35718	0.057558	-6.20551	5.45E-10	3.69E-09	PARD3	protein_cc
ENSG0000(	2542.228	0.084457	0.031851	2.65166	0.00801	0.016361	METTL16	protein_cc
ENSG0000(	2541.957	0.29616	0.031556	9.385163	6.28E-21	2.50E-19	SNRPB2	protein_cc
ENSG0000(	2541.682	-0.12907	0.041751	-3.09146	0.001992	0.004597	C2CD5	protein_cc
ENSG0000(	2540.842	0.003567	0.039623	0.090011	0.928278	0.949521	TMCO3	protein_cc
ENSG0000(	2540.566	0.052778	0.029421	1.793863	0.072835	0.117351	PRPSAP2	protein_cc
ENSG0000(	2540.475	-0.4239	0.074848	-5.66342	1.48E-08	8.01E-08	MAOA	protein_cc
ENSG0000(	2540.46	-0.20274	0.037965	-5.34015	9.29E-08	4.42E-07	AREL1	protein_cc
ENSG0000(	2540.172	-0.18756	0.034757	-5.39631	6.80E-08	3.31E-07	SLC30A1	protein_cc
ENSG0000(	2539.956	-0.78981	0.066087	-11.951	6.41E-33	2.72E-30	KIAA1671	protein_cc
ENSG0000(	2539.947	-0.15472	0.030163	-5.12936	2.91E-07	1.28E-06	EXOC2	protein_cc
ENSG0000(	2539.537	-0.07853	0.050259	-1.56244	0.118185	0.178417	SLC9A3R2	protein_cc
ENSG0000(	2539.233	0.273986	0.045238	6.056589	1.39E-09	8.87E-09	TMED3	protein_cc
ENSG0000(	2539.012	-0.50133	0.072088	-6.9544	3.54E-12	3.38E-11	TTLL7	protein_cc
ENSG0000(	2537.634	-0.37565	0.099865	-3.7616	0.000169	0.000474	RP11-401	lincRNA
ENSG0000(	2537.371	-0.31077	0.073102	-4.25117	2.13E-05	6.97E-05	IFIT2	protein_cc
ENSG0000(	2536.923	-0.00055	0.030296	-0.01827	0.985427	0.990082	FBXO3	protein_cc
ENSG0000(	2535.733	-0.10415	0.022908	-4.54649	5.45E-06	1.97E-05	SNAP29	protein_cc
ENSG0000(	2535.42	-0.23156	0.053176	-4.35467	1.33E-05	4.51E-05	SLC8A1	protein_cc
ENSG0000(	2535.339	-0.02105	0.028726	-0.73271	0.463733	0.559861	USP48	protein_cc
ENSG0000(	2534.323	-0.62431	0.081392	-7.67043	1.71E-14	2.37E-13	TLR4	protein_cc
ENSG0000(	2533.446	0.217418	0.034533	6.295939	3.06E-10	2.16E-09	UFD1L	protein_cc

ENSG0000(	2531.944	0.103227	0.033054	3.12298	0.00179	0.00417	COPS4	protein_cc
ENSG0000(	2530.93	0.10006	0.033758	2.964084	0.003036	0.006756	UBE2Q2	protein_cc
ENSG0000(	2530.101	-0.0499	0.043273	-1.15304	0.248894	0.335723	ASCC3	protein_cc
ENSG0000(	2529.591	-0.10007	0.046714	-2.14211	0.032185	0.057132	AFF1	protein_cc
ENSG0000(	2528.833	-0.15919	0.033668	-4.72813	2.27E-06	8.67E-06	GPKOW	protein_cc
ENSG0000(	2528.788	-0.96644	0.106552	-9.07012	1.19E-19	3.84E-18	DIRAS2	protein_cc
ENSG0000(	2528.428	0.333669	0.039177	8.516957	1.64E-17	3.78E-16	SNAPIN	protein_cc
ENSG0000(	2528.268	-0.05393	0.034124	-1.58044	0.114006	0.172984	MTMR2	protein_cc
ENSG0000(	2527.509	-0.13646	0.031993	-4.26526	2.00E-05	6.57E-05	IBTK	protein_cc
ENSG0000(	2527.501	0.26729	0.047674	5.606641	2.06E-08	1.09E-07	SSBP4	protein_cc
ENSG0000(	2526.104	-0.11426	0.026029	-4.38966	1.14E-05	3.89E-05	AGPS	protein_cc
ENSG0000(	2525.765	0.126385	0.049704	2.542758	0.010998	0.021772	ILF3-AS1	lincRNA
ENSG0000(	2525.585	-1.0287	0.106416	-9.66685	4.17E-22	2.05E-20	TUBA4A	protein_cc
ENSG0000(	2525.061	0.212238	0.1637	1.296502	0.194802	0.273061	NNAT	protein_cc
ENSG0000(	2523.602	-0.16005	0.024813	-6.4504	1.12E-10	8.44E-10	MARK2	protein_cc
ENSG0000(	2523.427	0.129558	0.042128	3.075342	0.002103	0.004831	CCNG1	protein_cc
ENSG0000(	2523.124	-0.27754	0.086559	-3.20638	0.001344	0.003201	CTD-3193	protein_cc
ENSG0000(	2522.289	-0.10181	0.031272	-3.25566	0.001131	0.00273	LRRC14	protein_cc
ENSG0000(	2520.463	-0.09692	0.03366	-2.87948	0.003983	0.008668	ZDHHC8	protein_cc
ENSG0000(	2520.289	-0.0635	0.086993	-0.72996	0.465415	0.561381	UCP2	protein_cc
ENSG0000(	2520.089	0.2614	0.044878	5.824626	5.72E-09	3.30E-08	RNF181	protein_cc
ENSG0000(	2520.03	0.19264	0.044073	4.370874	1.24E-05	4.21E-05	TMEM8A	protein_cc
ENSG0000(	2519.7	0.054508	0.044065	1.23699	0.216091	0.298143	SLC25A25	protein_cc
ENSG0000(	2519.386	0.036157	0.052904	0.683444	0.494327	0.588691	SSBP2	protein_cc
ENSG0000(	2518.846	-0.64653	0.136767	-4.72722	2.28E-06	8.70E-06	ST8SIA3	protein_cc
ENSG0000(	2518.639	-0.07627	0.027347	-2.78906	0.005286	0.011215	MRPL35	protein_cc
ENSG0000(	2518.349	-0.24135	0.051361	-4.69901	2.61E-06	9.89E-06	KDM6B	protein_cc
ENSG0000(	2517.596	0.289247	0.030046	9.626661	6.17E-22	2.92E-20	ELP5	protein_cc
ENSG0000(	2517.526	-0.02959	0.040499	-0.73074	0.46494	0.560924	PHF6	protein_cc
ENSG0000(	2516.772	-0.44083	0.058824	-7.49404	6.68E-14	8.40E-13	LMTK2	protein_cc
ENSG0000(	2516.468	0.099793	0.038133	2.616934	0.008872	0.017934	LBR	protein_cc
ENSG0000(	2516.361	-0.17582	0.034746	-5.06008	4.19E-07	1.80E-06	KRAS	protein_cc
ENSG0000(	2515.945	0.067817	0.048	1.412868	0.157695	0.228193	MPST	protein_cc
ENSG0000(	2515.317	0.069818	0.028225	2.473649	0.013374	0.025965	ZNF496	protein_cc
ENSG0000(	2515.151	-0.09437	0.03883	-2.43021	0.01509	0.028936	MED1	protein_cc
ENSG0000(	2513.895	-0.23655	0.18419	-1.28428	0.199045	0.278107	TRIM67	protein_cc
ENSG0000(	2513.275	-0.56357	0.114669	-4.91479	8.89E-07	3.62E-06	CHST9	protein_cc
ENSG0000(	2513.232	0.169467	0.038009	4.458588	8.25E-06	2.88E-05	ITGB1BP1	protein_cc
ENSG0000(	2513.147	-0.22207	0.043795	-5.07068	3.96E-07	1.71E-06	PPP1R26	protein_cc
ENSG0000(	2512.643	0.098303	0.027521	3.571918	0.000354	0.000942	DDX27	protein_cc
ENSG0000(	2509.24	0.074289	0.027057	2.745674	0.006039	0.012641	DNTTIP2	protein_cc
ENSG0000(	2508.428	-0.40942	0.080731	-5.07141	3.95E-07	1.70E-06	BRSK2	protein_cc
ENSG0000(	2508.183	-0.06106	0.029463	-2.07238	0.03823	0.066683	AFG3L2	protein_cc
ENSG0000(	2507.277	0.337652	0.076693	4.402676	1.07E-05	3.68E-05	BTN3A2	protein_cc
ENSG0000(	2507.232	-0.07669	0.030242	-2.53583	0.011218	0.022163	VAMP7	protein_cc
ENSG0000(	2505.536	-0.42013	0.045235	-9.28755	1.58E-20	5.83E-19	TMED8	protein_cc
ENSG0000(	2505.374	0.182314	0.025178	7.240933	4.46E-13	4.88E-12	NIFK	protein_cc
ENSG0000(	2505.249	-0.18115	0.149593	-1.21098	0.225902	0.309326	PCDHGB7	protein_cc
ENSG0000(	2505.223	0.122837	0.117563	1.044861	0.296087	0.388114	MYT1	protein_cc
ENSG0000(	2504.132	-0.13578	0.032906	-4.12626	3.69E-05	0.000116	RABGGTB	protein_cc
ENSG0000(	2503.963	0.036459	0.036071	1.01076	0.312131	0.405448	MED25	protein_cc
ENSG0000(	2503.913	0.335262	0.043864	7.643275	2.12E-14	2.89E-13	PGLS	protein_cc
ENSG0000(	2503.713	-0.05636	0.071004	-0.79372	0.42736	0.52451	FNBP1L	protein_cc
ENSG0000(	2502.874	-0.23296	0.034538	-6.7451	1.53E-11	1.32E-10	WAPAL	protein_cc
ENSG0000(	2502.052	0.272656	0.034046	8.0084	1.16E-15	1.98E-14	DUT	protein_cc
ENSG0000(	2501.984	0.179946	0.041613	4.324261	1.53E-05	5.13E-05	SURF1	protein_cc
ENSG0000(	2500.822	0.231243	0.039421	5.866034	4.46E-09	2.62E-08	NDUFAB1	protein_cc
ENSG0000(	2499.317	-0.17699	0.041049	-4.3118	1.62E-05	5.40E-05	ZBTB44	protein_cc
ENSG0000(	2499.142	0.021703	0.028648	0.757547	0.448722	0.545519	ZDHHC5	protein_cc

ENSG0000(	2499.122	0.091627	0.036597	2.50367	0.012291	0.024049	MFSD12	protein_cc
ENSG0000(	2498.731	0.033007	0.044211	0.746585	0.455314	0.551901	MIB2	protein_cc
ENSG0000(	2498.153	0.041604	0.038825	1.071561	0.283917	0.374609	LRRFIP1	protein_cc
ENSG0000(	2497.906	0.576826	0.060513	9.532239	1.54E-21	6.89E-20	GUSB	protein_cc
ENSG0000(	2497.762	0.239936	0.038126	6.293176	3.11E-10	2.20E-09	NKIRAS2	protein_cc
ENSG0000(	2496.538	0.248646	0.030367	8.18813	2.65E-16	5.04E-15	PRKRIP1	protein_cc
ENSG0000(	2496.44	-0.03713	0.025871	-1.43523	0.151223	0.22033	RHOT1	protein_cc
ENSG0000(	2496.107	-0.12615	0.022143	-5.69712	1.22E-08	6.67E-08	BECN1	protein_cc
ENSG0000(	2495.903	-0.26915	0.048469	-5.55304	2.81E-08	1.45E-07	KSR1	protein_cc
ENSG0000(	2495.741	0.187587	0.080326	2.335326	0.019526	0.036432	NRP1	protein_cc
ENSG0000(	2495.572	-0.12773	0.032452	-3.93611	8.28E-05	0.000246	KCTD10	protein_cc
ENSG0000(	2494.979	-0.72113	0.082312	-8.76091	1.94E-18	5.16E-17	NALCN	protein_cc
ENSG0000(	2493.649	-0.13238	0.031299	-4.22942	2.34E-05	7.61E-05	ANKRD13,	protein_cc
ENSG0000(	2493.164	-0.30308	0.03584	-8.45656	2.75E-17	6.17E-16	KLHL36	protein_cc
ENSG0000(	2493.05	0.12799	0.024581	5.206872	1.92E-07	8.70E-07	VPS16	protein_cc
ENSG0000(	2492.49	-0.32786	0.041888	-7.82696	5.00E-15	7.69E-14	ARFGEF2	protein_cc
ENSG0000(	2492.008	-0.21853	0.033001	-6.62199	3.54E-11	2.90E-10	SBNO1	protein_cc
ENSG0000(	2490.238	-0.31562	0.039558	-7.97866	1.48E-15	2.48E-14	NCKIPSD	protein_cc
ENSG0000(	2490.023	0.055879	0.041697	1.340114	0.180208	0.25553	PARP4	protein_cc
ENSG0000(	2489.618	0.352627	0.047883	7.364408	1.78E-13	2.07E-12	LSMD1	protein_cc
ENSG0000(	2489.416	-0.7089	0.072763	-9.74259	1.98E-22	1.04E-20	CLCN4	protein_cc
ENSG0000(	2489.069	0.110395	0.023549	4.687881	2.76E-06	1.04E-05	AIMP1	protein_cc
ENSG0000(	2488.61	0.32086	0.041168	7.793992	6.49E-15	9.79E-14	CANT1	protein_cc
ENSG0000(	2487.981	-0.02065	0.073864	-0.27963	0.77976	0.835662	RGMB	protein_cc
ENSG0000(	2486.909	-0.28066	0.122627	-2.28872	0.022095	0.040748	ZNF488	protein_cc
ENSG0000(	2486.351	-0.0643	0.097773	-0.65766	0.510759	0.603941	PHKG1	protein_cc
ENSG0000(	2486.212	-0.0862	0.034914	-2.46903	0.013548	0.026271	SRF	protein_cc
ENSG0000(	2486.181	0.31574	0.031734	9.949478	2.54E-23	1.61E-21	HDAC3	protein_cc
ENSG0000(	2486.153	-0.35957	0.053822	-6.68066	2.38E-11	1.99E-10	ASRGL1	protein_cc
ENSG0000(	2486.101	0.1842	0.06117	3.011269	0.002602	0.005876	MCC	protein_cc
ENSG0000(	2485.739	0.416828	0.03946	10.56321	4.41E-26	4.76E-24	CLDN12	protein_cc
ENSG0000(	2485.348	0.063825	0.097139	0.65705	0.511149	0.604349	EMILIN1	protein_cc
ENSG0000(	2484.852	-0.01614	0.022086	-0.73074	0.46494	0.560924	SRP54	protein_cc
ENSG0000(	2484.16	-0.19626	0.03568	-5.50057	3.79E-08	1.92E-07	C22orf39	protein_cc
ENSG0000(	2483.705	-0.58507	0.070351	-8.31643	9.06E-17	1.85E-15	KIAA1462	protein_cc
ENSG0000(	2483.322	0.540472	0.050886	10.62116	2.38E-26	2.72E-24	LSM7	protein_cc
ENSG0000(	2483.27	0.078396	0.024104	3.252375	0.001144	0.002758	UQCC1	protein_cc
ENSG0000(	2483.237	-0.05911	0.052955	-1.11618	0.264346	0.353103	ARHGEF3	protein_cc
ENSG0000(	2482.299	-0.16186	0.031111	-5.20286	1.96E-07	8.88E-07	FGFR1OP2	protein_cc
ENSG0000(	2482.253	0.450166	0.12797	3.517748	0.000435	0.001137	CA12	protein_cc
ENSG0000(	2481.887	-0.14149	0.029317	-4.82632	1.39E-06	5.50E-06	AP3S2	protein_cc
ENSG0000(	2481.559	-0.77436	0.086809	-8.92031	4.65E-19	1.36E-17	FBXO41	protein_cc
ENSG0000(	2479.756	0.029312	0.028786	1.018269	0.30855	0.401647	MAPK14	protein_cc
ENSG0000(	2479.661	-0.12152	0.037644	-3.22807	0.001246	0.002985	TAF1	protein_cc
ENSG0000(	2478.573	0.038917	0.027226	1.429416	0.152885	0.222407	RAB12	protein_cc
ENSG0000(	2478.319	0.38625	0.039878	9.685797	3.46E-22	1.74E-20	CPSF4	protein_cc
ENSG0000(	2478.292	0.019702	0.037655	0.523207	0.600831	0.685516	ADCY6	protein_cc
ENSG0000(	2478.161	0.276258	0.038953	7.092152	1.32E-12	1.35E-11	USP1	protein_cc
ENSG0000(	2478.108	-0.00458	0.034928	-0.13118	0.895631	0.92589	ZBTB33	protein_cc
ENSG0000(	2477.13	0.14327	0.037413	3.829416	0.000128	0.000368	UQCC2	protein_cc
ENSG0000(	2477.07	-0.31554	0.049141	-6.4211	1.35E-10	1.01E-09	HEATR5A	protein_cc
ENSG0000(	2476.178	-0.72127	0.072462	-9.95377	2.43E-23	1.55E-21	SNPH	protein_cc
ENSG0000(	2475.883	-0.15377	0.039851	-3.85871	0.000114	0.00033	ZNF652	protein_cc
ENSG0000(	2475.618	0.26339	0.092202	2.856665	0.004281	0.009254	B4GALNT1	protein_cc
ENSG0000(	2473.959	0.179758	0.039565	4.543368	5.54E-06	1.99E-05	COMMD2	protein_cc
ENSG0000(	2473.38	-0.2716	0.030582	-8.88093	6.63E-19	1.89E-17	CACUL1	protein_cc
ENSG0000(	2473.091	0.135403	0.024364	5.557484	2.74E-08	1.42E-07	AAR2	protein_cc
ENSG0000(	2472.896	1.036617	0.074414	13.93035	4.14E-44	8.11E-41	PSPH	protein_cc
ENSG0000(	2472.498	0.099753	0.05517	1.808108	0.07059	0.114122	PAQR4	protein_cc

ENSG0000(	2472.233	1.336127	0.098837	13.51856	1.22E-41	2.00E-38	CDKN2C	protein_cc
ENSG0000(	2471.322	-0.19079	0.040172	-4.74938	2.04E-06	7.85E-06	ELMSAN1	protein_cc
ENSG0000(	2470.761	0.339238	0.043135	7.864641	3.70E-15	5.83E-14	ECI1	protein_cc
ENSG0000(	2470.365	-0.0663	0.030806	-2.15214	0.031387	0.055852	ALAS1	protein_cc
ENSG0000(	2470.344	0.033572	0.027226	1.233098	0.217539	0.299721	USB1	protein_cc
ENSG0000(	2467.705	-0.15166	0.033461	-4.53259	5.83E-06	2.09E-05	WDR81	protein_cc
ENSG0000(	2466.754	-0.50917	0.056109	-9.07471	1.14E-19	3.70E-18	LRRC8B	protein_cc
ENSG0000(	2466.583	-0.49081	0.078141	-6.28112	3.36E-10	2.36E-09	BEX2	protein_cc
ENSG0000(	2466.451	-0.03462	0.025276	-1.36965	0.170795	0.244302	SACM1L	protein_cc
ENSG0000(	2464.854	-0.05617	0.045087	-1.24572	0.212869	0.294302	PLD2	protein_cc
ENSG0000(	2464.108	0.203967	0.076121	2.6795	0.007373	0.015174	SLC4A3	protein_cc
ENSG0000(	2464.102	-1.57251	0.15157	-10.3748	3.23E-25	2.94E-23	RGS4	protein_cc
ENSG0000(	2463.189	0.012067	0.026514	0.455112	0.649029	0.727742	ARFGAP3	protein_cc
ENSG0000(	2462.56	-0.36407	0.062859	-5.7919	6.96E-09	3.96E-08	GPR155	protein_cc
ENSG0000(	2461.382	0.056705	0.03091	1.834514	0.066578	0.108463	RNF139	protein_cc
ENSG0000(	2461.37	-0.13162	0.031693	-4.1529	3.28E-05	0.000104	ZC3H14	protein_cc
ENSG0000(	2460.182	-1.19321	0.112409	-10.6148	2.54E-26	2.89E-24	SERPINI1	protein_cc
ENSG0000(	2460.168	-0.30833	0.076459	-4.03266	5.51E-05	0.000169	GUCY1A2	protein_cc
ENSG0000(	2459.714	-0.15598	0.071843	-2.17107	0.029926	0.053535	INPP5D	protein_cc
ENSG0000(	2459.665	-0.00112	0.025557	-0.0438	0.965062	0.976099	RCOR3	protein_cc
ENSG0000(	2459.41	0.087407	0.033097	2.640924	0.008268	0.016841	SMARCAD	protein_cc
ENSG0000(	2458.93	0.079579	0.090206	0.882192	0.377673	0.474199	LTBP1	protein_cc
ENSG0000(	2458.566	0.382306	0.064542	5.923364	3.15E-09	1.90E-08	ERBB2	protein_cc
ENSG0000(	2458.294	-0.06964	0.034436	-2.02241	0.043134	0.074147	EXOSC6	protein_cc
ENSG0000(	2458.134	-0.07002	0.051082	-1.37082	0.17043	0.243885	GLIS2	protein_cc
ENSG0000(	2456.775	0.012403	0.026587	0.466496	0.640861	0.720771	CRTC2	protein_cc
ENSG0000(	2456.674	0.248435	0.035378	7.022307	2.18E-12	2.16E-11	PRPF3	protein_cc
ENSG0000(	2456.212	0.020269	0.080315	0.252366	0.800758	0.852756	LGALS9	protein_cc
ENSG0000(	2455.733	-0.32543	0.087606	-3.71475	0.000203	0.000563	ACSBG1	protein_cc
ENSG0000(	2455.551	0.03243	0.029512	1.098855	0.271831	0.361563	CDC73	protein_cc
ENSG0000(	2455.024	-0.1059	0.077326	-1.3695	0.170843	0.244349	REC8	protein_cc
ENSG0000(	2454.259	-0.22441	0.049196	-4.56143	5.08E-06	1.84E-05	POU2F1	protein_cc
ENSG0000(	2454.251	-0.02805	0.031608	-0.88754	0.37479	0.471385	LRCH3	protein_cc
ENSG0000(	2454.14	0.090087	0.042819	2.103901	0.035387	0.062236	GBA	protein_cc
ENSG0000(	2454.132	-0.07471	0.117566	-0.63545	0.525136	0.617005	FBLN1	protein_cc
ENSG0000(	2453.369	-0.5005	0.060648	-8.25253	1.55E-16	3.06E-15	HPCAL1	protein_cc
ENSG0000(	2453	0.284168	0.036365	7.814401	5.52E-15	8.43E-14	COA4	protein_cc
ENSG0000(	2452.421	-0.30508	0.056927	-5.35913	8.36E-08	4.01E-07	JADE2	protein_cc
ENSG0000(	2452.263	0.179162	0.024175	7.411107	1.25E-13	1.50E-12	E2F4	protein_cc
ENSG0000(	2451.588	0.010915	0.025601	0.426363	0.669843	0.745181	CREB1	protein_cc
ENSG0000(	2451.577	-0.0701	0.036296	-1.93126	0.053451	0.089591	RRP7A	protein_cc
ENSG0000(	2451.364	0.110114	0.035378	3.112523	0.001855	0.004304	SYS1	protein_cc
ENSG0000(	2451.349	0.017043	0.044057	0.386841	0.698874	0.769676	DCAF16	protein_cc
ENSG0000(	2450.422	-0.31394	0.054173	-5.79514	6.83E-09	3.89E-08	ZDHHC2	protein_cc
ENSG0000(	2450.366	0.081015	0.036678	2.208811	0.027188	0.049081	FAM91A1	protein_cc
ENSG0000(	2449.987	0.022365	0.02964	0.754546	0.450521	0.547318	ARMC1	protein_cc
ENSG0000(	2449.491	-0.09522	0.03369	-2.82633	0.004709	0.010078	KATNB1	protein_cc
ENSG0000(	2449.051	-0.31196	0.128724	-2.42348	0.015372	0.029431	SULF1	protein_cc
ENSG0000(	2448.623	0.288774	0.038474	7.505699	6.11E-14	7.72E-13	TRIM4	protein_cc
ENSG0000(	2448.523	0.162146	0.025066	6.468666	9.89E-11	7.54E-10	ORMDL1	protein_cc
ENSG0000(	2448.394	-0.51239	0.070057	-7.31394	2.59E-13	2.95E-12	PLEKHG3	protein_cc
ENSG0000(	2448.2	-0.44499	0.042117	-10.5655	4.31E-26	4.66E-24	SLK	protein_cc
ENSG0000(	2448.086	-0.10422	0.030436	-3.42421	0.000617	0.001563	FAM177A:	protein_cc
ENSG0000(	2447.167	-0.03538	0.042649	-0.82962	0.406755	0.503548	DZIP1	protein_cc
ENSG0000(	2445.007	0.347722	0.046058	7.549734	4.36E-14	5.63E-13	GATAD1	protein_cc
ENSG0000(	2443.242	-0.19824	0.029485	-6.72338	1.78E-11	1.51E-10	5-Mar	protein_cc
ENSG0000(	2442.809	0.217566	0.042027	5.176796	2.26E-07	1.01E-06	MRTO4	protein_cc
ENSG0000(	2441.816	0.04346	0.065546	0.663044	0.507302	0.600933	TMEM98	protein_cc
ENSG0000(	2441.787	0.263848	0.060531	4.358869	1.31E-05	4.43E-05	C1orf63	protein_cc

ENSG0000(	2441.604	0.165784	0.061263	2.706098	0.006808	0.014106	ACKR3	protein_cc
ENSG0000(	2440.908	-0.30556	0.051864	-5.89159	3.82E-09	2.27E-08	SEC24C	protein_cc
ENSG0000(	2440.806	0.156863	0.030059	5.218447	1.80E-07	8.20E-07	RIOK3	protein_cc
ENSG0000(	2440.419	-0.65357	0.078113	-8.36705	5.91E-17	1.25E-15	IQSEC2	protein_cc
ENSG0000(	2440.104	0.012634	0.030412	0.415422	0.677833	0.752002	SNX8	protein_cc
ENSG0000(	2439.161	-0.04656	0.030309	-1.5363	0.124464	0.18643	DCTN6	protein_cc
ENSG0000(	2438.86	0.204775	0.041258	4.963249	6.93E-07	2.87E-06	CENPT	protein_cc
ENSG0000(	2438.5	-0.01936	0.03552	-0.54503	0.585733	0.672014	ZNF644	protein_cc
ENSG0000(	2438.269	-0.21939	0.062575	-3.50607	0.000455	0.001183	GRIK4	protein_cc
ENSG0000(	2437.666	-0.97512	0.113138	-8.61892	6.76E-18	1.67E-16	BRINP1	protein_cc
ENSG0000(	2437.423	-0.47959	0.042474	-11.2915	1.45E-29	2.99E-27	CLN8	protein_cc
ENSG0000(	2437.409	-0.07181	0.038513	-1.86443	0.062261	0.10229	MORC2	protein_cc
ENSG0000(	2437.254	0.512911	0.065857	7.788284	6.79E-15	1.02E-13	SMO	protein_cc
ENSG0000(	2437.065	-0.84569	0.076511	-11.0532	2.11E-28	3.58E-26	CEP170B	protein_cc
ENSG0000(	2436.793	-0.07906	0.078956	-1.00128	0.316689	0.410332	SEMA5B	protein_cc
ENSG0000(	2436.775	0.17365	0.037795	4.594559	4.34E-06	1.59E-05	TCEB1	protein_cc
ENSG0000(	2436.606	0.233426	0.035064	6.657077	2.79E-11	2.32E-10	RNF7	protein_cc
ENSG0000(	2436.429	-0.18735	0.078644	-2.38224	0.017208	0.032525	LRRTM2	protein_cc
ENSG0000(	2436.205	0.159952	0.060647	2.637455	0.008353	0.016992	TBC1D1	protein_cc
ENSG0000(	2434.884	0.153227	0.030885	4.961231	7.00E-07	2.90E-06	TIPRL	protein_cc
ENSG0000(	2434.059	-0.23741	0.03779	-6.28231	3.34E-10	2.34E-09	STAU2	protein_cc
ENSG0000(	2433.063	0.335719	0.074189	4.525197	6.03E-06	2.16E-05	DCTD	protein_cc
ENSG0000(	2432.958	-0.12688	0.03938	-3.22203	0.001273	0.003044	SEC23A	protein_cc
ENSG0000(	2432.748	0.248159	0.033322	7.447213	9.53E-14	1.16E-12	NSL1	protein_cc
ENSG0000(	2432.737	0.180668	0.049315	3.663558	0.000249	0.000678	CCDC14	protein_cc
ENSG0000(	2432.58	-0.98879	0.086441	-11.439	2.67E-30	6.53E-28	HSPA12A	protein_cc
ENSG0000(	2431.706	-1.57734	0.13807	-11.4242	3.16E-30	7.54E-28	SLC12A5	protein_cc
ENSG0000(	2431.455	0.017839	0.041736	0.427432	0.669065	0.744503	HIRIP3	protein_cc
ENSG0000(	2431.362	0.213599	0.03275	6.522178	6.93E-11	5.42E-10	ARMC6	protein_cc
ENSG0000(	2431.198	0.015456	0.031551	0.489875	0.624222	0.70646	TUSC2	protein_cc
ENSG0000(	2430.768	0.379254	0.049787	7.617536	2.59E-14	3.47E-13	BAX	protein_cc
ENSG0000(	2430.663	0.15248	0.031848	4.78774	1.69E-06	6.58E-06	DPP9	protein_cc
ENSG0000(	2429.686	-0.3654	0.098969	-3.69206	0.000222	0.000611	ALDH1A1	protein_cc
ENSG0000(	2429.654	-0.33222	0.052625	-6.31298	2.74E-10	1.95E-09	ABHD6	protein_cc
ENSG0000(	2429.593	0.01564	0.031858	0.49091	0.62349	0.705881	CCDC97	protein_cc
ENSG0000(	2429.199	0.099981	0.061799	1.617844	0.105696	0.162019	KCNH2	protein_cc
ENSG0000(	2429.098	0.03838	0.02274	1.687762	0.091457	0.142969	PARN	protein_cc
ENSG0000(	2428.453	-0.00782	0.031833	-0.24578	0.805851	0.85688	ISCA1	protein_cc
ENSG0000(	2427.944	-0.24833	0.078556	-3.16114	0.001572	0.003698	ARRDC2	protein_cc
ENSG0000(	2427.916	0.190853	0.029272	6.519994	7.03E-11	5.49E-10	AAAS	protein_cc
ENSG0000(	2427.158	0.264975	0.030458	8.699628	3.33E-18	8.59E-17	TIMM44	protein_cc
ENSG0000(	2426.872	0.003437	0.033322	0.103137	0.917854	0.942068	MRPS25	protein_cc
ENSG0000(	2426.835	0.178608	0.025864	6.905576	5.00E-12	4.65E-11	MEN1	protein_cc
ENSG0000(	2426.502	0.072345	0.021808	3.317362	0.000909	0.002231	PRKRA	protein_cc
ENSG0000(	2425.911	-0.10363	0.037756	-2.74469	0.006057	0.012675	ATP5S	protein_cc
ENSG0000(	2425.635	-0.24356	0.037833	-6.43792	1.21E-10	9.12E-10	SNX29	protein_cc
ENSG0000(	2425.035	0.042344	0.032564	1.300359	0.193478	0.271436	TIMM17A	protein_cc
ENSG0000(	2423.958	-1.77546	0.176803	-10.042	9.96E-24	6.87E-22	NEFM	protein_cc
ENSG0000(	2423.095	-0.13031	0.027802	-4.68692	2.77E-06	1.05E-05	NFX1	protein_cc
ENSG0000(	2422.052	0.306966	0.077936	3.938699	8.19E-05	0.000243	LYPD1	protein_cc
ENSG0000(	2422.036	0.087963	0.01795	4.900556	9.56E-07	3.87E-06	TMUB2	protein_cc
ENSG0000(	2421.723	-0.53689	0.054094	-9.92513	3.24E-23	1.98E-21	LARGE	protein_cc
ENSG0000(	2421.058	0.093551	0.050317	1.859236	0.062994	0.103378	TCN2	protein_cc
ENSG0000(	2421.056	0.14656	0.022865	6.409706	1.46E-10	1.08E-09	PRKAB1	protein_cc
ENSG0000(	2420.656	0.224622	0.079075	2.840613	0.004503	0.009679	PHLDA3	protein_cc
ENSG0000(	2417.568	-0.14432	0.042488	-3.39662	0.000682	0.001715	RP11-566	lincRNA
ENSG0000(	2416.876	-0.17497	0.038309	-4.56741	4.94E-06	1.79E-05	ZFYVE26	protein_cc
ENSG0000(	2415.813	-0.07004	0.046885	-1.49388	0.135206	0.200022	PRTFDC1	protein_cc
ENSG0000(	2412.997	0.213226	0.043626	4.887592	1.02E-06	4.12E-06	UBL4A	protein_cc

ENSG0000(	2412.94	-0.08765	0.068921	-1.27172	0.203473	0.283418	RNF112	protein_cc
ENSG0000(	2412.909	0.132367	0.123619	1.070764	0.284275	0.374967	MS4A6A	protein_cc
ENSG0000(	2412.286	-1.00732	0.106186	-9.48631	2.39E-21	1.03E-19	RCAN2	protein_cc
ENSG0000(	2412.005	-0.13132	0.039543	-3.32098	0.000897	0.002205	PMM1	protein_cc
ENSG0000(	2411.329	0.103876	0.031214	3.327908	0.000875	0.002156	DPY19L4	protein_cc
ENSG0000(	2411.242	0.222695	0.035103	6.344119	2.24E-10	1.61E-09	AP5Z1	protein_cc
ENSG0000(	2409.84	0.259901	0.047592	5.461025	4.73E-08	2.36E-07	NOSIP	protein_cc
ENSG0000(	2408.841	0.17547	0.047901	3.663164	0.000249	0.000679	HMGB3	protein_cc
ENSG0000(	2408.81	-0.00663	0.034922	-0.1898	0.849465	0.891249	NUPL1	protein_cc
ENSG0000(	2406.603	-0.29482	0.071479	-4.1245	3.72E-05	0.000117	ZNF423	protein_cc
ENSG0000(	2406.296	0.026935	0.030282	0.889476	0.373748	0.470347	AP3B1	protein_cc
ENSG0000(	2405.942	-0.39618	0.113017	-3.50547	0.000456	0.001186	CELF3	protein_cc
ENSG0000(	2405.845	-0.2845	0.047983	-5.9293	3.04E-09	1.84E-08	NLK	protein_cc
ENSG0000(	2405.434	0.146477	0.029713	4.929758	8.23E-07	3.37E-06	PARL	protein_cc
ENSG0000(	2405.37	-0.42387	0.069709	-6.08053	1.20E-09	7.72E-09	SLC16A9	protein_cc
ENSG0000(	2405.28	0.167395	0.043565	3.842448	0.000122	0.000351	CDK2AP2	protein_cc
ENSG0000(	2405.215	0.197456	0.033156	5.955333	2.60E-09	1.58E-08	LSM3	protein_cc
ENSG0000(	2405.128	-0.2758	0.069017	-3.99607	6.44E-05	0.000195	PREX2	protein_cc
ENSG0000(	2403.753	-0.11411	0.028302	-4.03197	5.53E-05	0.000169	WDFY1	protein_cc
ENSG0000(	2403.16	-0.09577	0.033873	-2.82719	0.004696	0.010054	NCKAP5L	protein_cc
ENSG0000(	2402.977	0.430499	0.08715	4.939745	7.82E-07	3.21E-06	SALL3	protein_cc
ENSG0000(	2402.608	-0.27754	0.088341	-3.14168	0.00168	0.003932	MAST1	protein_cc
ENSG0000(	2400.691	-0.11421	0.025494	-4.47998	7.46E-06	2.63E-05	STK4	protein_cc
ENSG0000(	2400.485	-0.34718	0.045574	-7.61789	2.58E-14	3.47E-13	MYO9A	protein_cc
ENSG0000(	2399.354	0.201973	0.034995	5.771549	7.85E-09	4.43E-08	NAA20	protein_cc
ENSG0000(	2398.675	0.183802	0.032948	5.578467	2.43E-08	1.27E-07	DNPEP	protein_cc
ENSG0000(	2398.66	0.092639	0.043878	2.111266	0.034749	0.061227	ZNF395	protein_cc
ENSG0000(	2398.455	-0.14876	0.059217	-2.51208	0.012002	0.023551	PAX6	protein_cc
ENSG0000(	2398.337	0.230876	0.038175	6.047889	1.47E-09	9.31E-09	TMEM120	polymorpl
ENSG0000(	2397.914	0.257779	0.093713	2.750735	0.005946	0.012465	IGSF3	protein_cc
ENSG0000(	2397.894	-0.53021	0.059595	-8.89682	5.75E-19	1.66E-17	ARHGAP2	protein_cc
ENSG0000(	2397.82	-0.49374	0.066583	-7.41543	1.21E-13	1.45E-12	DAB2	protein_cc
ENSG0000(	2397.076	0.137268	0.08826	1.555259	0.119884	0.180511	OLFML3	protein_cc
ENSG0000(	2396.707	0.447115	0.035888	12.45848	1.26E-35	9.76E-33	BOD1	protein_cc
ENSG0000(	2395.541	1.347201	0.089224	15.09909	1.64E-51	6.14E-48	9-Mar	protein_cc
ENSG0000(	2395.111	-0.12312	0.031761	-3.87638	0.000106	0.000308	XPC	protein_cc
ENSG0000(	2394.933	0.084558	0.030775	2.747629	0.006003	0.012571	SETDB1	protein_cc
ENSG0000(	2394.831	-0.10268	0.037295	-2.75317	0.005902	0.012384	EDC4	protein_cc
ENSG0000(	2394.34	1.003099	0.093875	10.68546	1.19E-26	1.45E-24	PDGFA	protein_cc
ENSG0000(	2394.164	0.05516	0.027131	2.03307	0.042045	0.072507	LARP7	protein_cc
ENSG0000(	2393.752	-0.0042	0.029081	-0.14446	0.885141	0.918116	TMEM184	protein_cc
ENSG0000(	2391.328	-0.25078	0.061069	-4.10644	4.02E-05	0.000126	CHDH	protein_cc
ENSG0000(	2390.326	0.24011	0.035571	6.750147	1.48E-11	1.28E-10	VIMP	protein_cc
ENSG0000(	2389.841	0.026097	0.022749	1.147189	0.251303	0.338551	UBA5	protein_cc
ENSG0000(	2389.654	0.044868	0.024373	1.840917	0.065634	0.107114	TSR1	protein_cc
ENSG0000(	2388.76	-0.06329	0.106875	-0.59219	0.553727	0.643389	ACTA2	protein_cc
ENSG0000(	2388.655	0.133211	0.063216	2.107229	0.035098	0.061796	AHSA2	protein_cc
ENSG0000(	2388.478	-0.14152	0.034731	-4.07463	4.61E-05	0.000143	PRKAB2	protein_cc
ENSG0000(	2387.791	-0.11053	0.03541	-3.12151	0.001799	0.004188	SNRK	protein_cc
ENSG0000(	2387.717	-0.69056	0.094484	-7.30871	2.70E-13	3.06E-12	ERBB4	protein_cc
ENSG0000(	2387.53	0.167793	0.036503	4.596722	4.29E-06	1.57E-05	TRAPPC2L	protein_cc
ENSG0000(	2387.518	-0.11786	0.030629	-3.84803	0.000119	0.000343	THUMPD1	protein_cc
ENSG0000(	2387.262	0.278378	0.035254	7.896361	2.87E-15	4.60E-14	PSMA1	protein_cc
ENSG0000(	2386.981	0.022614	0.029357	0.770311	0.441115	0.538032	NELFA	protein_cc
ENSG0000(	2384.557	-0.12056	0.041736	-2.88856	0.00387	0.008441	ZBTB10	protein_cc
ENSG0000(	2384.526	-0.13578	0.028915	-4.69562	2.66E-06	1.00E-05	COG1	protein_cc
ENSG0000(	2384.426	0.185843	0.024851	7.478188	7.54E-14	9.37E-13	LSG1	protein_cc
ENSG0000(	2384.075	-0.58722	0.069577	-8.43988	3.18E-17	7.02E-16	APBA1	protein_cc
ENSG0000(	2382.394	-0.47558	0.047874	-9.93402	2.96E-23	1.83E-21	WDR7	protein_cc

ENSG0000(	2382.211	0.02969	0.040335	0.736098	0.461671	0.557814	G6PD	protein_cc
ENSG0000(	2381.688	-0.05339	0.029949	-1.78265	0.074643	0.119846	NUP50	protein_cc
ENSG0000(	2381.28	-0.19556	0.035287	-5.54213	2.99E-08	1.54E-07	SSH1	protein_cc
ENSG0000(	2381.108	-0.20109	0.034902	-5.76135	8.34E-09	4.68E-08	COL4A3BI	protein_cc
ENSG0000(	2380.51	-0.06591	0.031143	-2.11626	0.034322	0.06056	SKIL	protein_cc
ENSG0000(	2380.388	-0.31827	0.05868	-5.42382	5.83E-08	2.87E-07	MERTK	protein_cc
ENSG0000(	2380.073	-0.37847	0.048987	-7.72593	1.11E-14	1.60E-13	MAP2K4	protein_cc
ENSG0000(	2379.955	-0.1909	0.040472	-4.71668	2.40E-06	9.13E-06	NEURL4	protein_cc
ENSG0000(	2379.275	-0.21334	0.043334	-4.92319	8.51E-07	3.48E-06	FAM63B	protein_cc
ENSG0000(	2377.679	-0.29684	0.040437	-7.34096	2.12E-13	2.44E-12	AP3M2	protein_cc
ENSG0000(	2377.464	-0.0886	0.056915	-1.55678	0.119524	0.180087	ST8SIA1	protein_cc
ENSG0000(	2377.351	0.19841	0.023063	8.602854	7.78E-18	1.90E-16	POLR2D	protein_cc
ENSG0000(	2376.728	-0.07981	0.04791	-1.66582	0.095749	0.148796	SOCS6	protein_cc
ENSG0000(	2376.176	-0.25911	0.039054	-6.63469	3.25E-11	2.67E-10	ASCC1	protein_cc
ENSG0000(	2376.134	0.187737	0.057699	3.253728	0.001139	0.002746	PVRL2	protein_cc
ENSG0000(	2376.068	-0.0395	0.030229	-1.30681	0.191278	0.268826	SNAP47	protein_cc
ENSG0000(	2376.059	0.286469	0.056776	5.045578	4.52E-07	1.93E-06	BZW2	protein_cc
ENSG0000(	2374.962	-0.12198	0.027762	-4.3938	1.11E-05	3.82E-05	UBR2	protein_cc
ENSG0000(	2374.658	0.114625	0.027017	4.24268	2.21E-05	7.21E-05	NDUFV3	protein_cc
ENSG0000(	2374.354	-0.06278	0.027239	-2.30493	0.021171	0.039196	BRD9	protein_cc
ENSG0000(	2374.263	-0.04608	0.028383	-1.62346	0.104492	0.160436	ACBD3	protein_cc
ENSG0000(	2372.046	-0.14102	0.071003	-1.98617	0.047014	0.079983	MYADM	protein_cc
ENSG0000(	2371.945	-0.84562	0.123766	-6.83241	8.35E-12	7.50E-11	TMEM151	protein_cc
ENSG0000(	2371.367	0.027467	0.059028	0.465327	0.641697	0.721567	MIIP	protein_cc
ENSG0000(	2371.331	0.257244	0.04794	5.36594	8.05E-08	3.87E-07	CDK10	protein_cc
ENSG0000(	2370.946	-0.01361	0.036255	-0.37543	0.707338	0.776751	DIS3	protein_cc
ENSG0000(	2370.371	-0.14213	0.031142	-4.56404	5.02E-06	1.82E-05	HBS1L	protein_cc
ENSG0000(	2370.214	-0.05029	0.031472	-1.59783	0.11008	0.167851	SPAST	protein_cc
ENSG0000(	2369.049	0.044557	0.027267	1.634124	0.102233	0.157479	NFYC	protein_cc
ENSG0000(	2368.932	0.280828	0.035799	7.844586	4.34E-15	6.74E-14	MRPS33	protein_cc
ENSG0000(	2368.294	0.183285	0.033522	5.467601	4.56E-08	2.28E-07	ERP44	protein_cc
ENSG0000(	2366.892	0.155196	0.039252	3.953882	7.69E-05	0.000229	PNRC2	protein_cc
ENSG0000(	2366.592	-0.82526	0.103009	-8.01152	1.13E-15	1.94E-14	REPS2	protein_cc
ENSG0000(	2365.908	-0.20576	0.047374	-4.34334	1.40E-05	4.73E-05	ZDHHC14	protein_cc
ENSG0000(	2365.67	-0.11174	0.057968	-1.92758	0.053907	0.090246	LRP8	protein_cc
ENSG0000(	2365.466	-0.26964	0.051563	-5.22935	1.70E-07	7.77E-07	MAPK8	protein_cc
ENSG0000(	2365.302	-0.95949	0.090649	-10.5847	3.51E-26	3.86E-24	HLF	protein_cc
ENSG0000(	2365.284	0.106233	0.08595	1.235987	0.216463	0.298529	CYP27A1	protein_cc
ENSG0000(	2364.808	-0.06322	0.023934	-2.6416	0.008252	0.01681	CUL5	protein_cc
ENSG0000(	2364.787	0.061671	0.052507	1.174545	0.240177	0.325502	IGSF11	protein_cc
ENSG0000(	2364.596	0.793431	0.10363	7.656385	1.91E-14	2.63E-13	RPS2P5	pseudoge
ENSG0000(	2364.316	-0.32323	0.036464	-8.86433	7.70E-19	2.18E-17	EMC2	protein_cc
ENSG0000(	2362.447	-0.02217	0.044169	-0.502	0.615669	0.698757	HCFC1R1	protein_cc
ENSG0000(	2362.402	0.006691	0.039571	0.169084	0.865731	0.903589	DUSP16	protein_cc
ENSG0000(	2362.247	0.458522	0.079434	5.772395	7.82E-09	4.41E-08	DNALI1	protein_cc
ENSG0000(	2361.829	-0.12393	0.024218	-5.1173	3.10E-07	1.36E-06	FAM98A	protein_cc
ENSG0000(	2361.705	0.193199	0.030677	6.297922	3.02E-10	2.13E-09	PHF14	protein_cc
ENSG0000(	2361.149	-0.71609	0.08785	-8.15129	3.60E-16	6.68E-15	GPRASP1	protein_cc
ENSG0000(	2360.498	0.062251	0.062402	0.99757	0.318488	0.412169	ZNF521	protein_cc
ENSG0000(	2358.535	-0.01179	0.036259	-0.32504	0.745147	0.807733	CNOT6	protein_cc
ENSG0000(	2357.992	0.402824	0.054125	7.442477	9.88E-14	1.20E-12	MCM5	protein_cc
ENSG0000(	2357.784	-0.00282	0.031759	-0.08872	0.929308	0.950337	KDM2B	protein_cc
ENSG0000(	2356.821	0.01487	0.033314	0.446374	0.655327	0.733124	EIF2AK4	protein_cc
ENSG0000(	2356.695	-0.20256	0.083765	-2.41821	0.015597	0.029813	LCP1	protein_cc
ENSG0000(	2355.337	-0.35484	0.13497	-2.62901	0.008563	0.017377	CA10	protein_cc
ENSG0000(	2355.234	-0.09313	0.040278	-2.31228	0.020762	0.038511	KCTD17	protein_cc
ENSG0000(	2355.111	-0.18253	0.035669	-5.11746	3.10E-07	1.36E-06	RGP1	protein_cc
ENSG0000(	2353.938	0.037723	0.025	1.508917	0.13132	0.195101	PHF23	protein_cc
ENSG0000(	2353.76	0.295086	0.080703	3.656461	0.000256	0.000696	TSPO	protein_cc

ENSG0000(	2353.252	0.006148	0.02684	0.229055	0.818826	0.867179	MMGT1	protein_cc
ENSG0000(	2353.044	-0.56715	0.05421	-10.4622	1.29E-25	1.28E-23	PTPRM	protein_cc
ENSG0000(	2352.147	0.047189	0.026783	1.761905	0.078085	0.124687	THAP4	protein_cc
ENSG0000(	2352.028	0.212483	0.104968	2.024253	0.042944	0.073865	HLA-DMB	protein_cc
ENSG0000(	2351.844	-0.04526	0.02594	-1.74464	0.081048	0.128663	OXSR1	protein_cc
ENSG0000(	2351.594	0.276202	0.030052	9.190816	3.90E-20	1.36E-18	COA1	protein_cc
ENSG0000(	2351.574	-0.13846	0.048953	-2.82836	0.004679	0.01002	LYST	protein_cc
ENSG0000(	2351.573	0.182468	0.069328	2.631956	0.008489	0.017245	BOC	protein_cc
ENSG0000(	2351.403	-0.06685	0.049857	-1.34073	0.180007	0.25528	FAM214A	protein_cc
ENSG0000(	2351.332	0.251036	0.041465	6.054212	1.41E-09	8.98E-09	ADSL	protein_cc
ENSG0000(	2350.698	-0.23239	0.039253	-5.92033	3.21E-09	1.93E-08	SZT2	protein_cc
ENSG0000(	2350.621	0.074351	0.020892	3.558878	0.000372	0.000985	C3orf17	protein_cc
ENSG0000(	2349.74	-0.33114	0.095094	-3.48227	0.000497	0.001283	DUSP26	protein_cc
ENSG0000(	2349.646	0.277808	0.090256	3.078015	0.002084	0.004794	RARRES3	protein_cc
ENSG0000(	2349.265	0.051551	0.03337	1.544852	0.122382	0.183707	SLC39A13	protein_cc
ENSG0000(	2349.096	-0.24907	0.042097	-5.9165	3.29E-09	1.97E-08	SMG6	protein_cc
ENSG0000(	2348.516	-0.08686	0.030585	-2.83993	0.004512	0.009697	TGFBRAP1	protein_cc
ENSG0000(	2347.594	-0.00688	0.02375	-0.28977	0.771993	0.829391	KAT5	protein_cc
ENSG0000(	2347.41	-0.19335	0.051621	-3.74549	0.00018	0.000503	TBKBP1	protein_cc
ENSG0000(	2347.072	0.188204	0.039237	4.796536	1.61E-06	6.32E-06	NDUFB6	protein_cc
ENSG0000(	2346.328	-0.06741	0.025948	-2.59806	0.009375	0.018845	L3MBTL2	protein_cc
ENSG0000(	2345.662	0.151082	0.035913	4.206877	2.59E-05	8.35E-05	RBM28	protein_cc
ENSG0000(	2344.311	-0.08896	0.022369	-3.97684	6.98E-05	0.00021	N4BP1	protein_cc
ENSG0000(	2344.073	-0.01588	0.04237	-0.37476	0.707839	0.777136	GCDH	protein_cc
ENSG0000(	2343.427	0.111757	0.037659	2.967648	0.003001	0.006688	NDUFA9	protein_cc
ENSG0000(	2343.315	-0.46785	0.058288	-8.02659	1.00E-15	1.73E-14	C22orf46	protein_cc
ENSG0000(	2343.3	-0.21783	0.035825	-6.08035	1.20E-09	7.72E-09	MTR	protein_cc
ENSG0000(	2343.042	-0.13581	0.074304	-1.8278	0.067579	0.10989	RHPN1	protein_cc
ENSG0000(	2342.414	-0.13511	0.061718	-2.18914	0.028586	0.051367	ABCG1	protein_cc
ENSG0000(	2342.186	-0.42618	0.089884	-4.74143	2.12E-06	8.15E-06	EPHA4	protein_cc
ENSG0000(	2342.088	0.110327	0.03898	2.830353	0.00465	0.009963	IFFO1	protein_cc
ENSG0000(	2341.264	-0.17944	0.035714	-5.02434	5.05E-07	2.14E-06	ZADH2	protein_cc
ENSG0000(	2339.94	0.25228	0.054375	4.639602	3.49E-06	1.29E-05	PTK7	protein_cc
ENSG0000(	2339.121	-0.04246	0.030899	-1.37422	0.169374	0.242669	ZNF740	protein_cc
ENSG0000(	2338.968	0.05306	0.02616	2.028273	0.042532	0.073252	PIGG	protein_cc
ENSG0000(	2338.016	-0.33066	0.090563	-3.65115	0.000261	0.00071	CACNA1A	protein_cc
ENSG0000(	2337.498	-1.15131	0.110354	-10.4329	1.75E-25	1.67E-23	WNK2	protein_cc
ENSG0000(	2337.412	-0.25631	0.034786	-7.36819	1.73E-13	2.02E-12	NAPG	protein_cc
ENSG0000(	2337.221	-0.20824	0.072382	-2.877	0.004015	0.008732	LCAT	protein_cc
ENSG0000(	2336.032	0.027884	0.052988	0.52624	0.598721	0.683692	FADS3	protein_cc
ENSG0000(	2335.693	0.075666	0.035638	2.123152	0.033741	0.059609	ARFRP1	protein_cc
ENSG0000(	2333.81	0.028391	0.032056	0.885665	0.375798	0.472349	WDR45	protein_cc
ENSG0000(	2333.351	-0.14614	0.030305	-4.82225	1.42E-06	5.61E-06	PPM1F	protein_cc
ENSG0000(	2333.273	0.159583	0.038917	4.100582	4.12E-05	0.000129	SLC31A1	protein_cc
ENSG0000(	2333.059	0.129757	0.029947	4.332875	1.47E-05	4.94E-05	NOP58	protein_cc
ENSG0000(	2332.628	0.077878	0.024367	3.195997	0.001393	0.003311	PRPSAP1	protein_cc
ENSG0000(	2331.934	0.144998	0.030121	4.813896	1.48E-06	5.83E-06	MRP63	protein_cc
ENSG0000(	2331.523	0.037797	0.033706	1.121395	0.26212	0.350619	NLN	protein_cc
ENSG0000(	2330.999	-0.31814	0.071692	-4.43755	9.10E-06	3.16E-05	BMPR1B	protein_cc
ENSG0000(	2330.617	-0.24175	0.056944	-4.24543	2.18E-05	7.12E-05	FAM134B	protein_cc
ENSG0000(	2330.248	-0.08882	0.025653	-3.46226	0.000536	0.001374	DYM	protein_cc
ENSG0000(	2329.924	0.001972	0.046179	0.042704	0.965938	0.976576	BTBD6	protein_cc
ENSG0000(	2329.261	-0.02893	0.044507	-0.65002	0.515682	0.608537	USMG5	protein_cc
ENSG0000(	2329.097	0.068326	0.029636	2.30551	0.021138	0.039144	MICU2	protein_cc
ENSG0000(	2328.326	0.192089	0.030691	6.258784	3.88E-10	2.69E-09	FPGS	protein_cc
ENSG0000(	2327.789	0.101543	0.028863	3.518105	0.000435	0.001136	BLMH	protein_cc
ENSG0000(	2327.404	0.032658	0.030897	1.05699	0.290516	0.381931	MAML1	protein_cc
ENSG0000(	2326.385	-0.02509	0.02833	-0.88555	0.375858	0.472381	SEC24B	protein_cc
ENSG0000(	2326.129	0.231104	0.152508	1.515355	0.129683	0.192982	SOCS3	protein_cc

ENSG0000(	2326.127	-0.06089	0.027915	-2.18136	0.029156	0.052305	PBX2	protein_co
ENSG0000(	2325.66	0.16507	0.028321	5.82846	5.59E-09	3.23E-08	ENY2	protein_co
ENSG0000(	2325.309	0.215535	0.034387	6.267958	3.66E-10	2.55E-09	COQ4	protein_co
ENSG0000(	2324.534	-0.07417	0.079028	-0.93852	0.347976	0.443529	RFTN1	protein_co
ENSG0000(	2324.231	-0.69137	0.092017	-7.51354	5.75E-14	7.30E-13	GGTA1P	pseudoge
ENSG0000(	2323.722	-0.28806	0.053352	-5.39916	6.70E-08	3.26E-07	ASB13	protein_co
ENSG0000(	2323.406	-0.25824	0.044615	-5.78805	7.12E-09	4.04E-08	LMBRD2	protein_co
ENSG0000(	2322.985	-0.45726	0.060251	-7.58924	3.22E-14	4.25E-13	LMTK3	protein_co
ENSG0000(	2322.271	-0.01751	0.051732	-0.33854	0.734953	0.799316	ARMCX2	protein_co
ENSG0000(	2321.826	-0.13065	0.038073	-3.4316	0.0006	0.001525	NUDT5	protein_co
ENSG0000(	2321.056	0.193598	0.037989	5.096224	3.46E-07	1.51E-06	TRMT1	protein_co
ENSG0000(	2320.942	-0.19107	0.04251	-4.49459	6.97E-06	2.47E-05	FBXO11	protein_co
ENSG0000(	2319.894	-0.23363	0.056613	-4.12683	3.68E-05	0.000116	DNAJC15	protein_co
ENSG0000(	2319.728	-0.01799	0.039998	-0.44982	0.652842	0.731121	PRR13	protein_co
ENSG0000(	2318.814	-0.07309	0.028509	-2.56354	0.010361	0.020628	POLG	protein_co
ENSG0000(	2318.372	0.207754	0.035405	5.867918	4.41E-09	2.59E-08	BLVRA	protein_co
ENSG0000(	2317.836	-0.22534	0.030442	-7.40237	1.34E-13	1.59E-12	EIF4ENIF1	protein_co
ENSG0000(	2317.37	0.162243	0.030806	5.266649	1.39E-07	6.43E-07	ZNF282	protein_co
ENSG0000(	2317.318	0.194508	0.030736	6.328265	2.48E-10	1.78E-09	FAM172A	protein_co
ENSG0000(	2315.901	0.160694	0.03617	4.442783	8.88E-06	3.09E-05	RFNG	protein_co
ENSG0000(	2315.468	0.136668	0.033764	4.047692	5.17E-05	0.000159	SCYL2	protein_co
ENSG0000(	2315.361	0.003611	0.031462	0.114775	0.908624	0.935255	USP40	protein_co
ENSG0000(	2314.955	-1.14274	0.124869	-9.15147	5.62E-20	1.92E-18	NGEF	protein_co
ENSG0000(	2314.355	0.081631	0.037553	2.17375	0.029724	0.053209	ST7	protein_co
ENSG0000(	2313.967	-0.37257	0.051234	-7.27197	3.54E-13	3.94E-12	KIAA2026	protein_co
ENSG0000(	2313.719	-0.19961	0.042072	-4.74438	2.09E-06	8.04E-06	OTUD7B	protein_co
ENSG0000(	2313.718	0.111424	0.03031	3.676134	0.000237	0.000648	MRPL18	protein_co
ENSG0000(	2313.501	-0.14343	0.028043	-5.11468	3.14E-07	1.37E-06	AMBRA1	protein_co
ENSG0000(	2313.482	0.080332	0.032766	2.451705	0.014218	0.027455	EPC2	protein_co
ENSG0000(	2312.724	-0.10645	0.038392	-2.77258	0.005561	0.011734	PDS5B	protein_co
ENSG0000(	2310.834	-0.34286	0.050212	-6.8282	8.60E-12	7.71E-11	LARP6	protein_co
ENSG0000(	2310.567	0.132519	0.025846	5.127197	2.94E-07	1.29E-06	INO80E	protein_co
ENSG0000(	2309.658	0.146323	0.067535	2.166622	0.030264	0.054062	SLC29A4	protein_co
ENSG0000(	2309.355	0.060548	0.034872	1.736306	0.08251	0.130651	TOPBP1	protein_co
ENSG0000(	2308.681	-0.60652	0.076187	-7.96091	1.71E-15	2.84E-14	PGBD5	protein_co
ENSG0000(	2308.595	0.252177	0.036003	7.00442	2.48E-12	2.43E-11	IFT46	protein_co
ENSG0000(	2307.876	0.354952	0.074296	4.777514	1.77E-06	6.90E-06	PLS3	protein_co
ENSG0000(	2307.82	0.03946	0.037744	1.045465	0.295808	0.387772	PHACTR4	protein_co
ENSG0000(	2307.037	-0.12112	0.039667	-3.05339	0.002263	0.00517	FAM63A	protein_co
ENSG0000(	2306.571	-0.47961	0.070688	-6.78494	1.16E-11	1.02E-10	STON2	protein_co
ENSG0000(	2304.087	-0.37077	0.046254	-8.01609	1.09E-15	1.87E-14	USP32	protein_co
ENSG0000(	2304.01	-0.1258	0.036705	-3.42736	0.000609	0.001547	VAMP4	protein_co
ENSG0000(	2303.037	-0.5617	0.094388	-5.95101	2.66E-09	1.62E-08	ANKS1B	protein_co
ENSG0000(	2302.772	0.059271	0.024277	2.4415	0.014626	0.028168	MRPS5	protein_co
ENSG0000(	2302.756	0.002704	0.029858	0.090556	0.927846	0.949219	FLCN	protein_co
ENSG0000(	2302.666	-0.35562	0.049381	-7.20148	5.96E-13	6.41E-12	SNX30	protein_co
ENSG0000(	2300.895	-0.04598	0.047519	-0.96761	0.333241	0.427789	ARL10	protein_co
ENSG0000(	2300.752	-0.26325	0.048435	-5.43514	5.48E-08	2.70E-07	FAM217B	protein_co
ENSG0000(	2300.482	0.103053	0.042004	2.453448	0.014149	0.027329	AKR7A2	protein_co
ENSG0000(	2300.226	-0.05517	0.036058	-1.53	0.126016	0.188454	TMEM164	protein_co
ENSG0000(	2299.172	-0.80142	0.138739	-5.77645	7.63E-09	4.32E-08	LINC0108(	antisense
ENSG0000(	2298.805	-0.01024	0.02245	-0.45599	0.648396	0.727251	METTL13	protein_co
ENSG0000(	2297.61	0.003247	0.063386	0.051223	0.959148	0.972077	SOX21	protein_co
ENSG0000(	2296.734	0.129022	0.023462	5.49911	3.82E-08	1.93E-07	HNRNPLL	protein_co
ENSG0000(	2295.864	0.242364	0.044264	5.475444	4.36E-08	2.19E-07	TMEM183	protein_co
ENSG0000(	2295.065	0.063523	0.034469	1.842886	0.065346	0.106693	METTL3	protein_co
ENSG0000(	2295.042	-0.0307	0.054241	-0.56598	0.571409	0.659024	MEIS2	protein_co
ENSG0000(	2294.616	0.107473	0.03568	3.01213	0.002594	0.005861	ZNF862	protein_co
ENSG0000(	2294.424	0.05662	0.039826	1.421705	0.155112	0.225098	PKN2	protein_co

ENSG0000(	2294.268	0.024822	0.029124	0.852269	0.394065	0.490615	PRRC1	protein_cc
ENSG0000(	2293.204	0.201295	0.027577	7.299397	2.89E-13	3.26E-12	MOGS	protein_cc
ENSG0000(	2292.985	0.021767	0.023725	0.917484	0.358889	0.454667	SMYD5	protein_cc
ENSG0000(	2292.861	-0.87803	0.103097	-8.51652	1.64E-17	3.79E-16	SCN2A	protein_cc
ENSG0000(	2292.466	0.028309	0.14117	0.200535	0.841063	0.884464	HLA-DQA	protein_cc
ENSG0000(	2291.321	-0.10531	0.043995	-2.39366	0.016681	0.031667	EEA1	protein_cc
ENSG0000(	2290.25	0.073136	0.033061	2.21213	0.026958	0.048719	C12orf10	protein_cc
ENSG0000(	2289.953	0.066831	0.022238	3.005247	0.002654	0.005981	SNRNP27	protein_cc
ENSG0000(	2289.044	0.124853	0.047524	2.627162	0.00861	0.017459	RARA	protein_cc
ENSG0000(	2288.824	0.062901	0.040368	1.55819	0.119188	0.179707	DUS1L	protein_cc
ENSG0000(	2288.462	-0.19225	0.047004	-4.09011	4.31E-05	0.000134	CREB3L2	protein_cc
ENSG0000(	2288.395	-0.59572	0.078314	-7.60692	2.81E-14	3.74E-13	NT5DC3	protein_cc
ENSG0000(	2288.249	0.055137	0.028912	1.907033	0.056516	0.094032	BET1L	protein_cc
ENSG0000(	2285.35	-0.12821	0.046254	-2.77194	0.005572	0.011755	KBTBD6	protein_cc
ENSG0000(	2284.849	0.25216	0.049044	5.141534	2.73E-07	1.20E-06	GLB1	protein_cc
ENSG0000(	2284.596	-0.04079	0.042797	-0.95302	0.34058	0.435687	GTF2F2	protein_cc
ENSG0000(	2284.423	-0.05187	0.030879	-1.67977	0.093002	0.145075	CIR1	protein_cc
ENSG0000(	2283.653	0.074753	0.05102	1.465172	0.142874	0.209789	HOMER3	protein_cc
ENSG0000(	2283.615	0.114069	0.046692	2.443044	0.014564	0.028058	FAM193B	protein_cc
ENSG0000(	2282.799	-0.10078	0.036136	-2.78882	0.00529	0.011222	MOB1B	protein_cc
ENSG0000(	2282.563	0.329988	0.039873	8.27594	1.27E-16	2.55E-15	NUDT16L1	protein_cc
ENSG0000(	2280.339	-0.6673	0.080433	-8.29632	1.07E-16	2.17E-15	ADCY5	protein_cc
ENSG0000(	2280.272	-1.77213	0.165429	-10.7124	8.90E-27	1.11E-24	SV2B	protein_cc
ENSG0000(	2280.112	-0.24362	0.039637	-6.14613	7.94E-10	5.26E-09	MGAT4A	protein_cc
ENSG0000(	2279.9	0.292554	0.031478	9.293945	1.49E-20	5.53E-19	ZNF3	protein_cc
ENSG0000(	2278.671	0.212783	0.031226	6.814371	9.47E-12	8.44E-11	GSS	protein_cc
ENSG0000(	2278.318	0.096339	0.041259	2.335007	0.019543	0.036458	STIM2	protein_cc
ENSG0000(	2277.95	0.246872	0.037861	6.520519	7.01E-11	5.47E-10	UBE2J2	protein_cc
ENSG0000(	2276.307	0.314759	0.040422	7.786898	6.87E-15	1.03E-13	SSNA1	protein_cc
ENSG0000(	2275.207	0.159313	0.029644	5.37415	7.69E-08	3.71E-07	KIAA0907	protein_cc
ENSG0000(	2275.008	0.082001	0.031489	2.604162	0.00921	0.018547	COG2	protein_cc
ENSG0000(	2274.09	-0.01945	0.031928	-0.60922	0.542379	0.63314	RRP1B	protein_cc
ENSG0000(	2272.623	0.112963	0.041469	2.724006	0.00645	0.013427	YIF1B	protein_cc
ENSG0000(	2272.135	0.229507	0.068351	3.357787	0.000786	0.001953	PARP10	protein_cc
ENSG0000(	2271.504	0.122293	0.024576	4.976067	6.49E-07	2.70E-06	EIF2B4	protein_cc
ENSG0000(	2271.491	-0.22467	0.053656	-4.18722	2.82E-05	9.05E-05	ICK	protein_cc
ENSG0000(	2271.033	0.146078	0.037596	3.885439	0.000102	0.000298	SMG9	protein_cc
ENSG0000(	2270.898	-0.2345	0.076059	-3.0831	0.002049	0.004719	TNFRSF1B	protein_cc
ENSG0000(	2270.414	0.126809	0.034927	3.630702	0.000283	0.000764	RPAIN	protein_cc
ENSG0000(	2269.718	-0.01967	0.032098	-0.6128	0.540006	0.630886	LINC0066	lincRNA
ENSG0000(	2268.555	0.006167	0.02397	0.257293	0.796952	0.849768	SCFD1	protein_cc
ENSG0000(	2267.294	-0.40793	0.055463	-7.355	1.91E-13	2.21E-12	KIAA1467	protein_cc
ENSG0000(	2267.203	-0.09658	0.052613	-1.83569	0.066403	0.108222	SIAE	protein_cc
ENSG0000(	2266.855	-0.32016	0.041949	-7.63212	2.31E-14	3.13E-13	DMXL1	protein_cc
ENSG0000(	2266.581	-0.52705	0.054437	-9.6818	3.60E-22	1.80E-20	GAN	protein_cc
ENSG0000(	2266.213	-0.18439	0.058325	-3.16142	0.00157	0.003695	ZNF703	protein_cc
ENSG0000(	2266.159	0.214759	0.029715	7.227421	4.92E-13	5.34E-12	WDR41	protein_cc
ENSG0000(	2265.806	0.237927	0.068926	3.451918	0.000557	0.001423	NEDD9	protein_cc
ENSG0000(	2265.559	-0.07396	0.029958	-2.46864	0.013563	0.026297	TRAPPC8	protein_cc
ENSG0000(	2265.433	0.181066	0.026804	6.755206	1.43E-11	1.23E-10	TOR1A	protein_cc
ENSG0000(	2265.368	-0.11238	0.037336	-3.00985	0.002614	0.005901	SLC27A4	protein_cc
ENSG0000(	2264.938	0.021085	0.051942	0.405926	0.684797	0.757888	VWA5A	protein_cc
ENSG0000(	2264.506	-0.00453	0.075436	-0.06	0.952154	0.966921	STK32A	protein_cc
ENSG0000(	2264.048	-0.0644	0.021417	-3.00717	0.002637	0.005947	ASB8	protein_cc
ENSG0000(	2263.702	-0.08817	0.037485	-2.35201	0.018672	0.035003	MTMR6	protein_cc
ENSG0000(	2263.66	-0.86732	0.092476	-9.37885	6.67E-21	2.63E-19	RYR1	protein_cc
ENSG0000(	2262.911	-0.52126	0.092227	-5.65193	1.59E-08	8.53E-08	CDH13	protein_cc
ENSG0000(	2262.159	-0.05727	0.059479	-0.9628	0.335648	0.430462	SIPA1	protein_cc
ENSG0000(	2261.716	0.145633	0.035375	4.116816	3.84E-05	0.00012	SEPHS2	protein_cc

ENSG0000(	2261.663	-0.46233	0.046054	-10.0387	1.03E-23	7.07E-22	TTC7B	protein_cc
ENSG0000(	2261.5	-0.21714	0.043095	-5.0386	4.69E-07	2.00E-06	INPP1	protein_cc
ENSG0000(	2261.395	-0.08142	0.030393	-2.67887	0.007387	0.015199	GON4L	protein_cc
ENSG0000(	2261.28	-0.17429	0.032394	-5.38037	7.43E-08	3.59E-07	NUMB	protein_cc
ENSG0000(	2261.019	0.400061	0.042616	9.38766	6.13E-21	2.44E-19	KIF22	protein_cc
ENSG0000(	2260.072	-0.18665	0.04496	-4.15156	3.30E-05	0.000105	ARHGEF1(	protein_cc
ENSG0000(	2260.052	-0.39596	0.052006	-7.61374	2.66E-14	3.57E-13	PITPNM1	protein_cc
ENSG0000(	2260.021	-0.31569	0.046639	-6.76884	1.30E-11	1.13E-10	DCAF6	protein_cc
ENSG0000(	2259.989	0.30959	0.037834	8.182802	2.77E-16	5.23E-15	ITPA	protein_cc
ENSG0000(	2259.553	0.089539	0.047616	1.880442	0.060048	0.099125	EPS8	protein_cc
ENSG0000(	2259.43	0.11181	0.030185	3.704194	0.000212	0.000585	PRKRIR	protein_cc
ENSG0000(	2259.218	-0.2791	0.08663	-3.22175	0.001274	0.003047	GPR123	protein_cc
ENSG0000(	2259.204	0.270988	0.04125	6.569343	5.05E-11	4.03E-10	TMEM14B	protein_cc
ENSG0000(	2259.112	-0.1765	0.041657	-4.23696	2.27E-05	7.37E-05	TTC28	protein_cc
ENSG0000(	2258.969	-0.00715	0.038696	-0.18477	0.853408	0.893903	TJAP1	protein_cc
ENSG0000(	2257.483	-0.00769	0.028097	-0.27373	0.784295	0.83939	CLK3	protein_cc
ENSG0000(	2257.41	-0.00856	0.029198	-0.29332	0.769277	0.827014	KIAA1143	protein_cc
ENSG0000(	2257.149	0.289399	0.034282	8.441766	3.13E-17	6.91E-16	HMGN4	protein_cc
ENSG0000(	2256.296	0.095052	0.039929	2.380505	0.017289	0.032662	CACFD1	protein_cc
ENSG0000(	2254.652	-0.25305	0.056062	-4.51371	6.37E-06	2.27E-05	BCL9L	protein_cc
ENSG0000(	2254.604	-0.10515	0.063983	-1.6434	0.100299	0.154925	EGLN3	protein_cc
ENSG0000(	2252.762	-0.31784	0.04384	-7.24993	4.17E-13	4.59E-12	FNIP2	protein_cc
ENSG0000(	2252.329	0.04281	0.086964	0.492266	0.622532	0.704951	RGS2	protein_cc
ENSG0000(	2251.076	0.142742	0.030889	4.621114	3.82E-06	1.41E-05	C16orf80	protein_cc
ENSG0000(	2250.801	-0.43021	0.040074	-10.7353	6.95E-27	8.79E-25	TRAF3	protein_cc
ENSG0000(	2250.453	0.171361	0.038615	4.437647	9.09E-06	3.16E-05	ADCK2	protein_cc
ENSG0000(	2249.706	-0.0177	0.024349	-0.72691	0.467278	0.563149	C17orf85	protein_cc
ENSG0000(	2248.805	0.277557	0.043792	6.338061	2.33E-10	1.67E-09	MSI1	protein_cc
ENSG0000(	2248.659	0.023912	0.0247	0.96812	0.332985	0.427526	UBA6	protein_cc
ENSG0000(	2247.488	-0.13263	0.035008	-3.78841	0.000152	0.000429	SNAPC3	protein_cc
ENSG0000(	2247.023	0.369926	0.036117	10.2424	1.28E-24	1.04E-22	STX10	protein_cc
ENSG0000(	2246.985	-0.081	0.063785	-1.26984	0.204143	0.284178	TMEM108	protein_cc
ENSG0000(	2244.894	0.009825	0.070871	0.13863	0.889743	0.921471	GGT5	protein_cc
ENSG0000(	2244.812	-0.19488	0.038547	-5.05564	4.29E-07	1.84E-06	TBL1X	protein_cc
ENSG0000(	2244.555	-0.23639	0.032634	-7.24367	4.37E-13	4.79E-12	COX15	protein_cc
ENSG0000(	2244.29	-0.72695	0.073231	-9.92671	3.19E-23	1.95E-21	PER3	protein_cc
ENSG0000(	2244.004	0.31917	0.08182	3.900867	9.58E-05	0.000281	IGDCC4	protein_cc
ENSG0000(	2243.318	-0.20419	0.037037	-5.51319	3.52E-08	1.79E-07	LRSAM1	protein_cc
ENSG0000(	2243.076	-0.08168	0.026455	-3.08746	0.002019	0.004654	GOLGA5	protein_cc
ENSG0000(	2242.922	0.095657	0.0507	1.886744	0.059195	0.09791	TTC14	protein_cc
ENSG0000(	2240.41	-0.08758	0.025725	-3.40429	0.000663	0.001672	VPS4B	protein_cc
ENSG0000(	2238.551	-0.07817	0.022876	-3.41717	0.000633	0.001601	MKRN2	protein_cc
ENSG0000(	2238.137	0.186688	0.025711	7.260994	3.84E-13	4.25E-12	BFAR	protein_cc
ENSG0000(	2236.822	0.038297	0.037784	1.013567	0.310789	0.40405	CHKA	protein_cc
ENSG0000(	2235.698	-0.00736	0.031237	-0.23546	0.813852	0.863262	SYF2	protein_cc
ENSG0000(	2234.322	0.684421	0.110595	6.188545	6.07E-10	4.09E-09	EEF1A1P5	pseudoge
ENSG0000(	2232.606	0.05032	0.043883	1.146684	0.251512	0.338732	HRAS	protein_cc
ENSG0000(	2232.508	-0.01341	0.031963	-0.41969	0.67471	0.749345	TSSC1	protein_cc
ENSG0000(	2232.411	0.164754	0.04368	3.771844	0.000162	0.000457	RCBTB2	protein_cc
ENSG0000(	2231.729	-0.32553	0.072695	-4.47811	7.53E-06	2.65E-05	SUSD4	protein_cc
ENSG0000(	2231.109	-0.02353	0.026624	-0.88385	0.376777	0.473234	DLAT	protein_cc
ENSG0000(	2230.464	-0.31107	0.063281	-4.91564	8.85E-07	3.60E-06	DMD	protein_cc
ENSG0000(	2229.383	-0.00763	0.058867	-0.12962	0.896867	0.926866	IMPACT	protein_cc
ENSG0000(	2229.199	-0.04675	0.042489	-1.10035	0.27118	0.36079	RALB	protein_cc
ENSG0000(	2228.975	0.083031	0.045154	1.838867	0.065935	0.107527	ATOX1	protein_cc
ENSG0000(	2228.447	0.123186	0.044227	2.785331	0.005347	0.01133	SOAT1	protein_cc
ENSG0000(	2228.098	-0.02138	0.027294	-0.78338	0.433405	0.530441	ARHGAP1	protein_cc
ENSG0000(	2227.81	-0.38056	0.043139	-8.82177	1.13E-18	3.11E-17	MTMR9	protein_cc
ENSG0000(	2227.708	-0.02043	0.039759	-0.51376	0.607422	0.691248	SUCLG2	protein_cc

ENSG0000(	2227.591	-0.04646	0.025611	-1.81422	0.069644	0.112832	FBXO28	protein_cc
ENSG0000(	2227.014	-0.19607	0.039917	-4.91185	9.02E-07	3.67E-06	BMPR1A	protein_cc
ENSG0000(	2226.758	0.065621	0.032759	2.003172	0.045159	0.0772	AXIN1	protein_cc
ENSG0000(	2225.988	0.210669	0.042232	4.988413	6.09E-07	2.55E-06	ADCK4	protein_cc
ENSG0000(	2225.554	0.347073	0.041074	8.449999	2.91E-17	6.50E-16	RFXANK	protein_cc
ENSG0000(	2224.287	-0.10703	0.146714	-0.72949	0.465704	0.561614	CCL2	protein_cc
ENSG0000(	2224.075	0.130197	0.030064	4.330588	1.49E-05	4.99E-05	SPIDR	protein_cc
ENSG0000(	2223.784	-0.60482	0.151293	-3.99766	6.40E-05	0.000193	USH1C	protein_cc
ENSG0000(	2223.166	0.473771	0.101196	4.681703	2.85E-06	1.07E-05	CEBPD	protein_cc
ENSG0000(	2222.677	-0.2121	0.043314	-4.89674	9.74E-07	3.94E-06	ZSWIM6	protein_cc
ENSG0000(	2222.471	0.196051	0.040726	4.813867	1.48E-06	5.83E-06	MIEN1	protein_cc
ENSG0000(	2222.457	-0.16968	0.081024	-2.09416	0.036246	0.063585	GPRIN1	protein_cc
ENSG0000(	2221.088	0.16135	0.080793	1.997093	0.045815	0.078201	CHST2	protein_cc
ENSG0000(	2220.939	-0.10665	0.087687	-1.21624	0.223893	0.307015	LPPR4	protein_cc
ENSG0000(	2219.802	-0.12526	0.032599	-3.84239	0.000122	0.000351	GTF2H5	protein_cc
ENSG0000(	2219.768	-0.27088	0.03924	-6.903	5.09E-12	4.73E-11	ZFYVE27	protein_cc
ENSG0000(	2219.254	0.115222	0.034522	3.337658	0.000845	0.002087	CNOT3	protein_cc
ENSG0000(	2219.105	-0.00727	0.030153	-0.24102	0.809539	0.859779	STX4	protein_cc
ENSG0000(	2218.759	-0.09513	0.035903	-2.64965	0.008057	0.016447	BCL9	protein_cc
ENSG0000(	2218.516	0.127557	0.04221	3.021952	0.002512	0.005692	ZNF32	protein_cc
ENSG0000(	2217.896	-0.24105	0.045072	-5.34806	8.89E-08	4.25E-07	PDCD11	protein_cc
ENSG0000(	2217.602	-0.31827	0.046197	-6.88934	5.61E-12	5.17E-11	SLC22A23	protein_cc
ENSG0000(	2215.488	-0.32308	0.046472	-6.95218	3.60E-12	3.43E-11	LPGAT1	protein_cc
ENSG0000(	2215.244	-0.12012	0.088308	-1.36026	0.173748	0.247831	AIF1	protein_cc
ENSG0000(	2214.935	-0.40937	0.048977	-8.35846	6.35E-17	1.34E-15	TULP4	protein_cc
ENSG0000(	2214.836	-0.19624	0.057062	-3.43905	0.000584	0.001488	CACHD1	protein_cc
ENSG0000(	2213.987	-0.05721	0.050717	-1.12802	0.259312	0.347394	RFX7	protein_cc
ENSG0000(	2213.446	-0.05245	0.037903	-1.3839	0.16639	0.238977	PHIP	protein_cc
ENSG0000(	2212.797	-0.03743	0.027367	-1.36782	0.17137	0.245017	JKAMP	protein_cc
ENSG0000(	2212.669	-0.02388	0.035543	-0.67175	0.501744	0.595696	LYRM7	protein_cc
ENSG0000(	2212.488	-0.07653	0.092823	-0.82446	0.40968	0.50653	SLC2A3	protein_cc
ENSG0000(	2212.171	-0.06064	0.031653	-1.91578	0.055394	0.092433	RPAP1	protein_cc
ENSG0000(	2212.149	0.056485	0.025059	2.254077	0.024191	0.044222	MFAP1	protein_cc
ENSG0000(	2212.103	-0.14194	0.035196	-4.03299	5.51E-05	0.000168	LARS2	protein_cc
ENSG0000(	2211.349	0.093534	0.030159	3.101308	0.001927	0.004457	MRPS2	protein_cc
ENSG0000(	2210.994	-0.40406	0.050129	-8.06039	7.61E-16	1.34E-14	DIXDC1	protein_cc
ENSG0000(	2210.844	-0.11349	0.034891	-3.2526	0.001144	0.002756	NCOA3	protein_cc
ENSG0000(	2210.209	-0.0878	0.038472	-2.28217	0.022479	0.041404	DDX51	protein_cc
ENSG0000(	2209.501	-0.38325	0.084917	-4.5132	6.39E-06	2.27E-05	TOX	protein_cc
ENSG0000(	2208.272	-0.06018	0.030761	-1.95636	0.050423	0.085123	KLHL22	protein_cc
ENSG0000(	2207.975	0.400615	0.050511	7.931298	2.17E-15	3.54E-14	TCTN1	protein_cc
ENSG0000(	2207.839	0.065298	0.045997	1.419629	0.155716	0.225831	SMAD1	protein_cc
ENSG0000(	2207.705	0.128427	0.027619	4.649894	3.32E-06	1.24E-05	MUS81	protein_cc
ENSG0000(	2206.445	0.587256	0.106422	5.518178	3.43E-08	1.75E-07	KCNF1	protein_cc
ENSG0000(	2204.085	0.008838	0.038388	0.230225	0.817917	0.866505	VPS9D1	protein_cc
ENSG0000(	2204.003	0.017262	0.043639	0.395551	0.692436	0.764286	STRADB	protein_cc
ENSG0000(	2203.192	-0.02313	0.036244	-0.63827	0.523296	0.615298	PFDN2	protein_cc
ENSG0000(	2199.924	0.152345	0.032356	4.708399	2.50E-06	9.48E-06	PRMT7	protein_cc
ENSG0000(	2199.697	-0.06199	0.07042	-0.8803	0.378696	0.475265	REEP1	protein_cc
ENSG0000(	2198.648	0.286687	0.03658	7.837278	4.60E-15	7.12E-14	UNG	protein_cc
ENSG0000(	2197.499	-0.1184	0.054606	-2.16826	0.030139	0.053865	ANKRD13I	protein_cc
ENSG0000(	2196.068	0.195111	0.060928	3.202329	0.001363	0.003244	ACAT2	protein_cc
ENSG0000(	2193.989	-0.30061	0.045797	-6.56398	5.24E-11	4.16E-10	JMY	protein_cc
ENSG0000(	2192.825	0.175911	0.062826	2.799973	0.005111	0.010874	ZNF436	protein_cc
ENSG0000(	2192.204	0.336598	0.04055	8.300776	1.03E-16	2.10E-15	SRA1	protein_cc
ENSG0000(	2192.103	-0.8825	0.078263	-11.2761	1.72E-29	3.52E-27	PRKCE	protein_cc
ENSG0000(	2191.986	-0.25636	0.032767	-7.82371	5.13E-15	7.87E-14	GTDC1	protein_cc
ENSG0000(	2191.955	-0.35992	0.041613	-8.6492	5.19E-18	1.30E-16	ATG2B	protein_cc
ENSG0000(	2191.65	-0.01665	0.029877	-0.55727	0.577341	0.664518	ABHD10	protein_cc

ENSG0000(	2191.252	0.048138	0.028672	1.678915	0.093169	0.145269	CCNT2	protein_cc
ENSG0000(	2190.291	0.091393	0.027761	3.292152	0.000994	0.002422	SUGP1	protein_cc
ENSG0000(	2188.462	-0.44598	0.046393	-9.61318	7.03E-22	3.31E-20	PRICKLE2	protein_cc
ENSG0000(	2186.913	0.196959	0.045685	4.311199	1.62E-05	5.42E-05	GOLT1B	protein_cc
ENSG0000(	2186.782	-0.15045	0.040766	-3.69049	0.000224	0.000615	ZNF445	protein_cc
ENSG0000(	2185.436	-0.21988	0.098892	-2.22348	0.026183	0.047487	RIMS4	protein_cc
ENSG0000(	2185.206	-0.06498	0.02996	-2.16873	0.030103	0.053809	EAF1	protein_cc
ENSG0000(	2184.424	-0.17924	0.028293	-6.3354	2.37E-10	1.70E-09	PLA2G12A	protein_cc
ENSG0000(	2184.354	-0.26708	0.14509	-1.84078	0.065653	0.10714	VIPR2	protein_cc
ENSG0000(	2184.328	0.803784	0.080781	9.950203	2.52E-23	1.60E-21	MCM2	protein_cc
ENSG0000(	2183.901	-0.09436	0.045621	-2.06837	0.038605	0.067254	EPC1	protein_cc
ENSG0000(	2183.586	0.18382	0.02927	6.280139	3.38E-10	2.37E-09	WDR46	protein_cc
ENSG0000(	2181.665	-0.21498	0.039451	-5.44945	5.05E-08	2.51E-07	GTPBP4	protein_cc
ENSG0000(	2181.63	0.204684	0.034718	5.895694	3.73E-09	2.22E-08	IMP3	protein_cc
ENSG0000(	2181.328	-0.25468	0.050036	-5.08986	3.58E-07	1.55E-06	AKNA	protein_cc
ENSG0000(	2180.704	-1.1163	0.125835	-8.8711	7.24E-19	2.06E-17	SYT7	protein_cc
ENSG0000(	2180.17	-0.21629	0.044599	-4.84957	1.24E-06	4.93E-06	UROS	protein_cc
ENSG0000(	2179.521	0.168359	0.037109	4.536892	5.71E-06	2.05E-05	C19orf25	protein_cc
ENSG0000(	2178.791	-0.11003	0.030637	-3.59134	0.000329	0.000879	KLHDC2	protein_cc
ENSG0000(	2177.886	0.717127	0.076531	9.370384	7.23E-21	2.83E-19	TSFM	protein_cc
ENSG0000(	2177.416	-0.41859	0.043048	-9.72384	2.39E-22	1.23E-20	ZFYVE9	protein_cc
ENSG0000(	2177.286	0.052377	0.026839	1.951528	0.050994	0.085996	ZNF317	protein_cc
ENSG0000(	2177.043	-0.32909	0.066334	-4.96116	7.01E-07	2.90E-06	NR1D1	protein_cc
ENSG0000(	2176.754	-0.69257	0.113893	-6.08087	1.20E-09	7.71E-09	CPLX1	protein_cc
ENSG0000(	2175.764	0.609001	0.091825	6.632158	3.31E-11	2.71E-10	PDLIM3	protein_cc
ENSG0000(	2175.215	-0.46508	0.059093	-7.87039	3.54E-15	5.58E-14	FAM69A	protein_cc
ENSG0000(	2174.909	0.193515	0.103894	1.862617	0.062516	0.102676	SNX10	protein_cc
ENSG0000(	2174.873	0.290251	0.038837	7.473583	7.80E-14	9.68E-13	TSEN34	protein_cc
ENSG0000(	2174.462	0.118344	0.041185	2.873498	0.00406	0.008818	NAGLU	protein_cc
ENSG0000(	2174.181	0.117532	0.031718	3.705502	0.000211	0.000582	POLR2K	protein_cc
ENSG0000(	2173.869	-0.07111	0.057761	-1.2311	0.218285	0.300548	ROM1	protein_cc
ENSG0000(	2173.473	-0.24541	0.0363	-6.76054	1.37E-11	1.19E-10	HECA	protein_cc
ENSG0000(	2172.952	0.31655	0.087238	3.628599	0.000285	0.00077	PROS1	protein_cc
ENSG0000(	2172.759	-0.09961	0.037636	-2.64678	0.008126	0.016576	DCUN1D4	protein_cc
ENSG0000(	2171.178	0.332779	0.174071	1.911741	0.055909	0.093157	SPOCD1	protein_cc
ENSG0000(	2171.159	0.101682	0.021271	4.780309	1.75E-06	6.81E-06	COPS7B	protein_cc
ENSG0000(	2171.023	0.250984	0.030819	8.143741	3.83E-16	7.08E-15	CIRH1A	protein_cc
ENSG0000(	2170.911	0.136433	0.05167	2.64046	0.008279	0.016859	CCNB1IP1	protein_cc
ENSG0000(	2170.705	0.219099	0.068009	3.221639	0.001275	0.003048	GAL3ST4	protein_cc
ENSG0000(	2169.904	0.396223	0.03576	11.08003	1.57E-28	2.73E-26	DGUOK	protein_cc
ENSG0000(	2169.705	-0.10228	0.027898	-3.6661	0.000246	0.000672	CHMP2B	protein_cc
ENSG0000(	2169.406	0.099533	0.022666	4.391341	1.13E-05	3.86E-05	THUMPD3	protein_cc
ENSG0000(	2168.64	-0.18595	0.035907	-5.17876	2.23E-07	1.00E-06	HEATR5B	protein_cc
ENSG0000(	2167.795	-0.04832	0.028567	-1.69152	0.090737	0.141983	ATF7	protein_cc
ENSG0000(	2166.198	0.131821	0.03993	3.301334	0.000962	0.002351	TSSC4	protein_cc
ENSG0000(	2165.871	-0.25011	0.110964	-2.25401	0.024196	0.044227	UNC79	protein_cc
ENSG0000(	2165.65	-1.47519	0.150225	-9.81987	9.25E-23	5.14E-21	SLC6A17	protein_cc
ENSG0000(	2165.092	0.001861	0.028824	0.064568	0.948518	0.964394	MAP4K3	protein_cc
ENSG0000(	2164.546	-0.22683	0.03589	-6.32019	2.61E-10	1.87E-09	PIAS1	protein_cc
ENSG0000(	2163.787	-0.16071	0.027659	-5.81044	6.23E-09	3.57E-08	GZF1	protein_cc
ENSG0000(	2163.321	-0.18075	0.036578	-4.94145	7.75E-07	3.19E-06	DNAJB14	protein_cc
ENSG0000(	2162.829	-0.11921	0.063627	-1.87362	0.060982	0.10045	USP2	protein_cc
ENSG0000(	2162.372	0.180924	0.03628	4.986833	6.14E-07	2.57E-06	MUL1	protein_cc
ENSG0000(	2161.111	-0.37513	0.074358	-5.04489	4.54E-07	1.94E-06	CCDC136	protein_cc
ENSG0000(	2159.369	-0.13141	0.027598	-4.76168	1.92E-06	7.42E-06	ALS2	protein_cc
ENSG0000(	2158.383	0.141225	0.033919	4.163549	3.13E-05	9.97E-05	KRI1	protein_cc
ENSG0000(	2158.133	-0.3204	0.036644	-8.74373	2.26E-18	5.98E-17	PDK2	protein_cc
ENSG0000(	2157.281	0.025687	0.050028	0.513443	0.607642	0.691421	MAPKAPK	protein_cc
ENSG0000(	2157.094	-0.09809	0.035657	-2.75102	0.005941	0.012456	MAP3K12	protein_cc

ENSG0000(	2157.015	0.262872	0.028765	9.138511	6.33E-20	2.14E-18	METTL2B	protein_cc
ENSG0000(	2156.59	0.173572	0.038281	4.534207	5.78E-06	2.08E-05	NDUFA12	protein_cc
ENSG0000(	2156.236	0.032197	0.041415	0.777412	0.436916	0.533959	CYP2U1	protein_cc
ENSG0000(	2155.614	-0.02199	0.033113	-0.66413	0.506609	0.600276	APH1B	protein_cc
ENSG0000(	2154.551	0.134448	0.028056	4.792166	1.65E-06	6.44E-06	EIF2D	protein_cc
ENSG0000(	2154.376	0.07147	0.032058	2.229386	0.025788	0.046838	CEP57	protein_cc
ENSG0000(	2153.645	-0.32579	0.056388	-5.77763	7.58E-09	4.29E-08	DAGLA	protein_cc
ENSG0000(	2152.75	-0.18816	0.03045	-6.17943	6.43E-10	4.32E-09	BCAS3	protein_cc
ENSG0000(	2151.889	-0.10472	0.029744	-3.52061	0.000431	0.001126	SPECC1L	protein_cc
ENSG0000(	2151.888	-0.00683	0.050611	-0.13497	0.892635	0.923594	PYCR1	protein_cc
ENSG0000(	2151.171	0.073671	0.027259	2.702675	0.006878	0.014237	SNX4	protein_cc
ENSG0000(	2150.424	-0.08507	0.03015	-2.82163	0.004778	0.010215	TCOF1	protein_cc
ENSG0000(	2150.112	0.069656	0.023873	2.917735	0.003526	0.00775	COX11	protein_cc
ENSG0000(	2149.856	-0.19105	0.067032	-2.85017	0.00437	0.009423	NUDT11	protein_cc
ENSG0000(	2149.23	-0.07548	0.036838	-2.04882	0.040479	0.07013	HIAT1	protein_cc
ENSG0000(	2147.791	0.420246	0.083782	5.015947	5.28E-07	2.23E-06	PIK3C2B	protein_cc
ENSG0000(	2146.266	-0.00015	0.035082	-0.00437	0.996513	0.997774	C14orf1	protein_cc
ENSG0000(	2145.364	0.071137	0.032187	2.210124	0.027097	0.04894	NADSYN1	protein_cc
ENSG0000(	2143.867	-0.79684	0.117036	-6.80852	9.86E-12	8.77E-11	SPOCK3	protein_cc
ENSG0000(	2143.384	-0.01367	0.030383	-0.45002	0.652698	0.731037	ARMCX1	protein_cc
ENSG0000(	2143.232	0.053194	0.047421	1.121739	0.261973	0.350457	RFX3	protein_cc
ENSG0000(	2143.196	0.443301	0.047411	9.350155	8.75E-21	3.40E-19	LSM2	protein_cc
ENSG0000(	2142.934	-0.04093	0.024989	-1.63785	0.101453	0.156466	SAP130	protein_cc
ENSG0000(	2142.477	0.27403	0.041513	6.601058	4.08E-11	3.30E-10	APRT	protein_cc
ENSG0000(	2141.148	0.126256	0.037231	3.391154	0.000696	0.001746	DGCR6L	protein_cc
ENSG0000(	2139.486	-0.00863	0.108032	-0.07985	0.936357	0.955526	LRRC55	protein_cc
ENSG0000(	2139.393	-0.05589	0.032466	-1.72141	0.085177	0.134378	GLRX5	protein_cc
ENSG0000(	2138.372	-0.66217	0.123202	-5.37471	7.67E-08	3.70E-07	SLC7A14	protein_cc
ENSG0000(	2138.008	-0.24548	0.03913	-6.27326	3.54E-10	2.47E-09	WDFY2	protein_cc
ENSG0000(	2137.612	-0.30895	0.043743	-7.06287	1.63E-12	1.64E-11	STRN	protein_cc
ENSG0000(	2137.073	0.326823	0.067531	4.839584	1.30E-06	5.17E-06	DTX3L	protein_cc
ENSG0000(	2136.938	-0.01651	0.061828	-0.26711	0.789386	0.843603	TACC2	protein_cc
ENSG0000(	2136.914	-0.22245	0.0362	-6.14493	8.00E-10	5.29E-09	FBXO25	protein_cc
ENSG0000(	2136.768	0.241778	0.033639	7.187419	6.60E-13	7.06E-12	TSEN15	protein_cc
ENSG0000(	2136.096	-0.36669	0.055671	-6.58671	4.50E-11	3.61E-10	ZNF25	protein_cc
ENSG0000(	2135.711	-0.00745	0.050425	-0.14765	0.882622	0.916196	CHSY1	protein_cc
ENSG0000(	2135.126	-0.3669	0.036414	-10.0759	7.06E-24	5.01E-22	PIK3CB	protein_cc
ENSG0000(	2135.072	0.288424	0.029876	9.654126	4.72E-22	2.30E-20	C7orf26	protein_cc
ENSG0000(	2134.547	1.092092	0.121635	8.978457	2.75E-19	8.36E-18	TUBA1C	protein_cc
ENSG0000(	2134.247	-0.05111	0.032651	-1.56525	0.117524	0.177561	SFT2D1	protein_cc
ENSG0000(	2134.216	0.144042	0.026238	5.489828	4.02E-08	2.03E-07	PPHLN1	protein_cc
ENSG0000(	2133.912	-0.13374	0.029368	-4.554	5.26E-06	1.90E-05	SUOX	protein_cc
ENSG0000(	2133.901	-1.71553	0.15754	-10.8895	1.29E-27	1.84E-25	DDN	protein_cc
ENSG0000(	2133.77	0.165983	0.025578	6.489305	8.62E-11	6.64E-10	HERPUD2	protein_cc
ENSG0000(	2133.281	0.1365	0.101011	1.351328	0.17659	0.251197	FCER1G	protein_cc
ENSG0000(	2132.696	0.018142	0.025653	0.707222	0.479428	0.574811	ZNF76	protein_cc
ENSG0000(	2131.969	-0.12195	0.0909	-1.34162	0.17972	0.254934	CHADL	protein_cc
ENSG0000(	2131.009	-0.13479	0.103429	-1.30318	0.192514	0.27035	MYL9	protein_cc
ENSG0000(	2129.963	0.242154	0.026518	9.131765	6.74E-20	2.26E-18	UNC50	protein_cc
ENSG0000(	2128.599	0.276997	0.048846	5.670822	1.42E-08	7.70E-08	NDUFB1	protein_cc
ENSG0000(	2127.483	-0.0024	0.046494	-0.0517	0.958771	0.971863	CD27-AS1	antisense
ENSG0000(	2127.054	-0.1047	0.02963	-3.53357	0.00041	0.001076	SMURF2	protein_cc
ENSG0000(	2126.487	-0.28442	0.081031	-3.50999	0.000448	0.001168	C2orf88	protein_cc
ENSG0000(	2126.431	0.29496	0.035191	8.381672	5.22E-17	1.11E-15	ODF2	protein_cc
ENSG0000(	2125.8	0.260634	0.13392	1.946195	0.051631	0.086909	HBA2	protein_cc
ENSG0000(	2125.401	-0.21071	0.047273	-4.45731	8.30E-06	2.90E-05	TRPS1	protein_cc
ENSG0000(	2124.087	-0.27748	0.082355	-3.36931	0.000754	0.001878	COL4A5	protein_cc
ENSG0000(	2123.276	0.045426	0.028023	1.621008	0.105016	0.161139	EDC3	protein_cc
ENSG0000(	2122.122	0.359081	0.051056	7.033016	2.02E-12	2.01E-11	NDUFA13	protein_cc

ENSG0000(	2121.63	0.057255	0.040039	1.429957	0.152729	0.222252	AKAP17A	protein_cc
ENSG0000(	2121.326	-0.14334	0.036219	-3.95757	7.57E-05	0.000226	UFL1	protein_cc
ENSG0000(	2120.238	0.097306	0.052682	1.847042	0.064741	0.105836	FBXL7	protein_cc
ENSG0000(	2120.134	0.058053	0.029019	2.000514	0.045445	0.077637	AGK	protein_cc
ENSG0000(	2119.82	0.1155	0.043331	2.665526	0.007687	0.015762	WDR91	protein_cc
ENSG0000(	2118.615	-0.12445	0.03727	-3.33923	0.00084	0.002077	SNX33	protein_cc
ENSG0000(	2118.572	-0.02113	0.044775	-0.47202	0.636909	0.717282	CCNG2	protein_cc
ENSG0000(	2118.371	1.194548	0.156885	7.614167	2.65E-14	3.56E-13	EMP3	protein_cc
ENSG0000(	2117.626	0.062875	0.035148	1.788864	0.073637	0.118434	SUPT20H	protein_cc
ENSG0000(	2117.594	-0.05344	0.030087	-1.77615	0.075709	0.12132	DHX33	protein_cc
ENSG0000(	2117.561	0.022514	0.019413	1.159707	0.246168	0.33257	ACAD9	protein_cc
ENSG0000(	2117.508	-0.49145	0.094585	-5.19587	2.04E-07	9.19E-07	NR4A1	protein_cc
ENSG0000(	2117.4	0.079957	0.031339	2.551394	0.010729	0.02129	TCTN3	protein_cc
ENSG0000(	2117.024	0.12055	0.029106	4.141748	3.45E-05	0.000109	NARFL	protein_cc
ENSG0000(	2116.881	0.215862	0.036547	5.906401	3.50E-09	2.09E-08	HBP1	protein_cc
ENSG0000(	2116.711	-0.03015	0.028321	-1.06471	0.287008	0.378165	SCAF4	protein_cc
ENSG0000(	2116.577	0.405578	0.054651	7.421223	1.16E-13	1.40E-12	NOP10	protein_cc
ENSG0000(	2116.362	0.014273	0.026327	0.542154	0.587713	0.673966	C16orf58	protein_cc
ENSG0000(	2115.975	-0.03616	0.026107	-1.38518	0.165997	0.23852	CNOT4	protein_cc
ENSG0000(	2115.562	-0.35065	0.046745	-7.50122	6.32E-14	7.99E-13	TMEM65	protein_cc
ENSG0000(	2114.528	0.036999	0.079246	0.466884	0.640583	0.720511	C11orf96	protein_cc
ENSG0000(	2114.074	0.061194	0.030644	1.996904	0.045836	0.078226	USP38	protein_cc
ENSG0000(	2113.779	0.000932	0.037079	0.025136	0.979946	0.98594	CYB5D2	protein_cc
ENSG0000(	2111.985	0.147919	0.024629	6.005887	1.90E-09	1.19E-08	GLE1	protein_cc
ENSG0000(	2111.715	0.03938	0.046126	0.853751	0.393243	0.489859	MANEAL	protein_cc
ENSG0000(	2110.766	0.032879	0.030663	1.07229	0.28359	0.37423	CCDC82	protein_cc
ENSG0000(	2110.577	0.152741	0.073137	2.088413	0.036761	0.064384	DLC1	protein_cc
ENSG0000(	2110.465	-0.29086	0.040895	-7.11221	1.14E-12	1.18E-11	SSH2	protein_cc
ENSG0000(	2110.348	-0.05732	0.045011	-1.27342	0.202868	0.28269	C11orf95	lincRNA
ENSG0000(	2109.925	-0.1471	0.025714	-5.72067	1.06E-08	5.86E-08	TADA2B	protein_cc
ENSG0000(	2109.237	-0.22034	0.073724	-2.98867	0.002802	0.006277	IL33	protein_cc
ENSG0000(	2108.872	0.137708	0.038569	3.570475	0.000356	0.000946	RNPEP	protein_cc
ENSG0000(	2107.614	-0.21335	0.045238	-4.71611	2.40E-06	9.15E-06	ASXL2	protein_cc
ENSG0000(	2107.126	0.334082	0.033653	9.927197	3.17E-23	1.95E-21	SDCCAG3	protein_cc
ENSG0000(	2107.07	-0.79252	0.114434	-6.92558	4.34E-12	4.09E-11	FRRS1L	protein_cc
ENSG0000(	2106.994	0.063957	0.028148	2.272197	0.023075	0.042383	ST3GAL2	protein_cc
ENSG0000(	2106.281	0.185372	0.037984	4.880274	1.06E-06	4.26E-06	NDUFC1	protein_cc
ENSG0000(	2105.8	-0.84156	0.098529	-8.54124	1.33E-17	3.12E-16	ATRNL1	protein_cc
ENSG0000(	2105.735	0.063793	0.03344	1.907659	0.056435	0.093923	TRMT2A	protein_cc
ENSG0000(	2105.325	-0.35074	0.098398	-3.56453	0.000365	0.000966	TMEM132	protein_cc
ENSG0000(	2105.201	0.100129	0.042851	2.336667	0.019456	0.03632	ACYP2	protein_cc
ENSG0000(	2104.551	-0.0933	0.037465	-2.49031	0.012763	0.024873	CRELD1	protein_cc
ENSG0000(	2104.538	0.240961	0.024751	9.735409	2.13E-22	1.10E-20	MLH1	protein_cc
ENSG0000(	2104.321	-0.00727	0.040781	-0.17835	0.858447	0.897763	FAM229B	protein_cc
ENSG0000(	2104.046	-0.12436	0.034437	-3.61125	0.000305	0.000819	ZNF318	protein_cc
ENSG0000(	2103.811	0.246892	0.030674	8.048923	8.35E-16	1.46E-14	SLBP	protein_cc
ENSG0000(	2103.044	-0.03254	0.067251	-0.4839	0.628455	0.709938	STK17B	protein_cc
ENSG0000(	2101.274	0.317613	0.052064	6.100423	1.06E-09	6.87E-09	PAFAH1B3	protein_cc
ENSG0000(	2101.162	-0.01413	0.027351	-0.51646	0.605535	0.689689	PSPC1	protein_cc
ENSG0000(	2100.707	0.115803	0.028022	4.132517	3.59E-05	0.000113	SMIM15	protein_cc
ENSG0000(	2100.707	0.6283	0.082126	7.65044	2.00E-14	2.75E-13	ZIC1	protein_cc
ENSG0000(	2100.694	-0.06821	0.087452	-0.77995	0.435422	0.532448	HK2	protein_cc
ENSG0000(	2099.124	0.06533	0.028121	2.323201	0.020168	0.037511	ORC4	protein_cc
ENSG0000(	2097.809	0.266508	0.031977	8.334468	7.78E-17	1.61E-15	HAT1	protein_cc
ENSG0000(	2097.323	0.049169	0.039503	1.244706	0.21324	0.294746	WDR3	protein_cc
ENSG0000(	2097.182	-0.17029	0.040012	-4.2559	2.08E-05	6.83E-05	MAPKBP1	protein_cc
ENSG0000(	2096.171	0.269452	0.044305	6.081695	1.19E-09	7.67E-09	STK36	protein_cc
ENSG0000(	2095.962	-0.20804	0.030683	-6.78049	1.20E-11	1.05E-10	GUF1	protein_cc
ENSG0000(	2095.422	0.500916	0.053909	9.29193	1.52E-20	5.63E-19	MCM6	protein_cc

ENSG0000(	2094.298	0.030627	0.037115	0.825196	0.409261	0.506118	DNMT3A	protein_cc
ENSG0000(	2093.356	0.036282	0.023884	1.519113	0.128734	0.191814	STAM2	protein_cc
ENSG0000(	2092.587	0.232123	0.065753	3.530226	0.000415	0.001089	BCAM	protein_cc
ENSG0000(	2092.056	-0.26143	0.091719	-2.85031	0.004368	0.009419	SLC1A1	protein_cc
ENSG0000(	2091.532	0.317698	0.037261	8.526193	1.51E-17	3.51E-16	LRWD1	protein_cc
ENSG0000(	2090.218	-0.68665	0.084965	-8.08162	6.39E-16	1.14E-14	MAP7	protein_cc
ENSG0000(	2089.738	-1.14743	0.133189	-8.61504	6.99E-18	1.72E-16	RAB3C	protein_cc
ENSG0000(	2088.554	0.042172	0.026667	1.581434	0.113779	0.172703	ZNF672	protein_cc
ENSG0000(	2087.849	0.082781	0.026969	3.069539	0.002144	0.004917	NAA15	protein_cc
ENSG0000(	2086.938	-0.40364	0.048689	-8.29014	1.13E-16	2.28E-15	UPF2	protein_cc
ENSG0000(	2086.328	-0.16881	0.050367	-3.35162	0.000803	0.001993	KLHL23	protein_cc
ENSG0000(	2084.804	0.078741	0.028647	2.748677	0.005984	0.012535	PRKAA1	protein_cc
ENSG0000(	2084.739	-0.41285	0.069808	-5.91403	3.34E-09	2.00E-08	PDE8A	protein_cc
ENSG0000(	2084.657	0.014319	0.030366	0.471552	0.637247	0.717544	DCP2	protein_cc
ENSG0000(	2084.523	-0.04142	0.083457	-0.49635	0.619644	0.702327	BRINP3	protein_cc
ENSG0000(	2082.839	0.088435	0.043446	2.035497	0.041801	0.072146	ZNF22	protein_cc
ENSG0000(	2082.333	-0.05243	0.027398	-1.91372	0.055656	0.092784	TRAPPC11	protein_cc
ENSG0000(	2081.805	-0.12332	0.027976	-4.40799	1.04E-05	3.59E-05	ARNT	protein_cc
ENSG0000(	2081.704	-0.28573	0.043517	-6.56601	5.17E-11	4.11E-10	PXK	protein_cc
ENSG0000(	2081.67	-0.29801	0.037383	-7.97181	1.56E-15	2.62E-14	GS1-251I9	antisense
ENSG0000(	2081.557	-0.11249	0.028034	-4.01255	6.01E-05	0.000183	NFYB	protein_cc
ENSG0000(	2081.309	0.328977	0.039627	8.301785	1.03E-16	2.08E-15	GPN1	protein_cc
ENSG0000(	2080.837	-0.08377	0.043943	-1.90634	0.056606	0.094164	FAM115A	protein_cc
ENSG0000(	2080.506	-0.91022	0.07799	-11.671	1.79E-31	5.38E-29	NUAK1	protein_cc
ENSG0000(	2080.457	-0.05093	0.060716	-0.83881	0.401578	0.498356	BDH2	protein_cc
ENSG0000(	2080.176	0.407735	0.040041	10.18283	2.37E-24	1.82E-22	TBL2	protein_cc
ENSG0000(	2079.559	0.343592	0.045535	7.545648	4.50E-14	5.81E-13	LINC00998	protein_cc
ENSG0000(	2079.406	0.076741	0.025559	3.002493	0.002678	0.00603	CBLL1	protein_cc
ENSG0000(	2079.083	0.19313	0.064038	3.01586	0.002563	0.005797	PLEKHG2	protein_cc
ENSG0000(	2078.809	1.43752	0.178402	8.057759	7.77E-16	1.36E-14	FMOD	protein_cc
ENSG0000(	2078.335	0.047793	0.035482	1.346952	0.177996	0.25296	DGAT1	protein_cc
ENSG0000(	2078.137	0.179113	0.039767	4.504117	6.66E-06	2.37E-05	C16orf13	protein_cc
ENSG0000(	2077.716	0.051527	0.041622	1.237965	0.215729	0.297716	SEC22B	protein_cc
ENSG0000(	2077.273	-1.09623	0.110863	-9.88816	4.69E-23	2.78E-21	SNCA	protein_cc
ENSG0000(	2075.4	0.65932	0.07125	9.253573	2.17E-20	7.87E-19	ARL4A	protein_cc
ENSG0000(	2074.58	-0.15213	0.04601	-3.30658	0.000944	0.002311	FAM213B	protein_cc
ENSG0000(	2074.4	0.52444	0.093702	5.596881	2.18E-08	1.15E-07	PYGL	protein_cc
ENSG0000(	2073.345	-0.2605	0.045258	-5.7559	8.62E-09	4.83E-08	GPHN	protein_cc
ENSG0000(	2073.248	0.05356	0.02589	2.06874	0.03857	0.067205	NFATC2IP	protein_cc
ENSG0000(	2073.164	0.134475	0.048328	2.782566	0.005393	0.011419	GAMT	protein_cc
ENSG0000(	2072.185	-0.21209	0.065228	-3.25146	0.001148	0.002766	DENND3	protein_cc
ENSG0000(	2072.096	0.142239	0.02648	5.371504	7.81E-08	3.76E-07	RP5-1180	antisense
ENSG0000(	2071.828	-0.07057	0.02744	-2.5719	0.010114	0.020175	UBN1	protein_cc
ENSG0000(	2071.402	-0.21107	0.087053	-2.42461	0.015325	0.029348	GPR34	protein_cc
ENSG0000(	2071.321	0.231708	0.027172	8.527512	1.50E-17	3.48E-16	RAE1	protein_cc
ENSG0000(	2071.307	-0.4704	0.074615	-6.30442	2.89E-10	2.05E-09	NBEA	protein_cc
ENSG0000(	2070.671	-0.27096	0.035668	-7.59666	3.04E-14	4.03E-13	SPATA2	protein_cc
ENSG0000(	2069.943	0.228083	0.053847	4.235795	2.28E-05	7.41E-05	RPL36A	protein_cc
ENSG0000(	2069.454	0.319372	0.092229	3.462799	0.000535	0.001372	CXCR4	protein_cc
ENSG0000(	2069.055	0.208785	0.029403	7.100725	1.24E-12	1.27E-11	SDF2	protein_cc
ENSG0000(	2068.839	-0.03345	0.047131	-0.70974	0.477863	0.573368	ELK1	protein_cc
ENSG0000(	2068.262	-0.18759	0.080302	-2.33601	0.019491	0.036376	SYTL2	protein_cc
ENSG0000(	2068.208	-0.31703	0.07007	-4.52449	6.05E-06	2.17E-05	NEDD4L	protein_cc
ENSG0000(	2066.383	0.083329	0.073567	1.132693	0.257343	0.345229	SNCAIP	protein_cc
ENSG0000(	2065.639	0.280578	0.03279	8.556703	1.16E-17	2.75E-16	VPS25	protein_cc
ENSG0000(	2065.101	0.34815	0.061663	5.645966	1.64E-08	8.81E-08	ESAM	protein_cc
ENSG0000(	2064.187	0.689261	0.07994	8.62228	6.56E-18	1.62E-16	DDIT3	protein_cc
ENSG0000(	2063.519	-0.14337	0.038123	-3.7606	0.00017	0.000476	WWC3	protein_cc
ENSG0000(	2063.055	0.735838	0.06442	11.42244	3.23E-30	7.64E-28	LYPLA1	protein_cc

ENSG0000	2063.011	0.071931	0.069776	1.030896	0.302589	0.395341	PPP1R15A	protein_co
ENSG0000	2062.915	0.187071	0.024669	7.583306	3.37E-14	4.43E-13	VWA9	protein_co
ENSG0000	2062.619	-0.01216	0.038563	-0.31534	0.752507	0.813622	IFT172	protein_co
ENSG0000	2062.51	0.113931	0.04431	2.571216	0.010134	0.020211	TTC7A	protein_co
ENSG0000	2062.445	-0.04169	0.025793	-1.61643	0.106002	0.162409	UBLCP1	protein_co
ENSG0000	2062.089	-0.29137	0.063487	-4.58942	4.44E-06	1.62E-05	TMC6	protein_co
ENSG0000	2062.021	-0.15617	0.047436	-3.29217	0.000994	0.002422	MAMLD1	protein_co
ENSG0000	2061.957	0.365859	0.074866	4.88687	1.02E-06	4.13E-06	NUP210	protein_co
ENSG0000	2061.423	0.368047	0.054824	6.713293	1.90E-11	1.62E-10	ZNF83	protein_co
ENSG0000	2061.257	-0.48373	0.061447	-7.87228	3.48E-15	5.50E-14	FOXO4	protein_co
ENSG0000	2060.589	-0.47748	0.054294	-8.79433	1.44E-18	3.91E-17	PRKAG2	protein_co
ENSG0000	2059.959	-0.47175	0.094622	-4.98567	6.17E-07	2.58E-06	CAP2	protein_co
ENSG0000	2059.764	-0.08357	0.029892	-2.79563	0.00518	0.011003	NSRP1	protein_co
ENSG0000	2059.518	0.317951	0.078544	4.048046	5.16E-05	0.000159	LBH	protein_co
ENSG0000	2059.439	-0.21759	0.03562	-6.10871	1.00E-09	6.55E-09	RANBP6	protein_co
ENSG0000	2059.416	0.428763	0.043387	9.882277	4.97E-23	2.93E-21	BCL7C	protein_co
ENSG0000	2059.03	-0.16356	0.041312	-3.95905	7.52E-05	0.000225	MGA	protein_co
ENSG0000	2058.886	0.191218	0.026594	7.190233	6.47E-13	6.92E-12	ZNF639	protein_co
ENSG0000	2058.819	-0.11457	0.036896	-3.10523	0.001901	0.004405	CBX4	protein_co
ENSG0000	2057.305	-0.02821	0.025792	-1.09367	0.274098	0.364095	ANGEL2	protein_co
ENSG0000	2056.978	-0.32138	0.040563	-7.92295	2.32E-15	3.77E-14	UHRF1BP1	protein_co
ENSG0000	2056.719	0.268033	0.111489	2.404118	0.016212	0.030883	CRB2	protein_co
ENSG0000	2056.571	-0.06675	0.089228	-0.74813	0.454382	0.550998	GLIS3	protein_co
ENSG0000	2055.774	-0.25634	0.115477	-2.21983	0.02643	0.047901	IGF2	protein_co
ENSG0000	2055.702	-0.47755	0.106975	-4.46415	8.04E-06	2.82E-05	SHISA7	protein_co
ENSG0000	2055.256	0.052392	0.051784	1.011744	0.31166	0.404926	PIK3R3	protein_co
ENSG0000	2054.491	-0.00749	0.026828	-0.27932	0.779997	0.835829	MAP2K7	protein_co
ENSG0000	2054.156	0.251442	0.035058	7.172214	7.38E-13	7.83E-12	GTF2A2	protein_co
ENSG0000	2053.462	-0.07762	0.028433	-2.73011	0.006331	0.013202	TESK1	protein_co
ENSG0000	2052.837	0.104716	0.061374	1.706181	0.087974	0.138303	FOXO1	protein_co
ENSG0000	2051.42	-0.03734	0.037082	-1.0069	0.313982	0.407389	ZNF397	protein_co
ENSG0000	2051.263	-0.23011	0.030314	-7.59106	3.17E-14	4.20E-13	BAHD1	protein_co
ENSG0000	2051.184	0.348128	0.061972	5.617549	1.94E-08	1.03E-07	TMEM97	protein_co
ENSG0000	2050.341	0.063004	0.031418	2.00533	0.044928	0.076843	KLC4	protein_co
ENSG0000	2050.059	-0.27148	0.040612	-6.68471	2.31E-11	1.94E-10	PRR14L	protein_co
ENSG0000	2049.899	-0.08759	0.03248	-2.69682	0.007001	0.014464	SIRT3	protein_co
ENSG0000	2049.456	-0.08673	0.030592	-2.83507	0.004582	0.009831	PIGX	protein_co
ENSG0000	2048.641	-0.10556	0.042299	-2.49562	0.012574	0.024544	CEP78	protein_co
ENSG0000	2048.555	0.022469	0.025084	0.895735	0.370394	0.466755	PEX11B	protein_co
ENSG0000	2047.552	0.881813	0.131167	6.722818	1.78E-11	1.52E-10	STEAP3	protein_co
ENSG0000	2047.27	0.220023	0.105305	2.089389	0.036673	0.064255	ITGA5	protein_co
ENSG0000	2046.338	-0.09821	0.026148	-3.75577	0.000173	0.000485	TMEM55B	protein_co
ENSG0000	2046.311	-0.42914	0.04702	-9.12668	7.06E-20	2.36E-18	NXPE3	protein_co
ENSG0000	2046.305	0.102131	0.026325	3.879562	0.000105	0.000305	RBM18	protein_co
ENSG0000	2045.974	-0.45442	0.048083	-9.45077	3.36E-21	1.42E-19	WDR37	protein_co
ENSG0000	2045.149	-0.27252	0.09177	-2.96964	0.002981	0.006648	PTCHD2	protein_co
ENSG0000	2045.078	-0.07326	0.024626	-2.97468	0.002933	0.006551	DYNC1LI1	protein_co
ENSG0000	2044.912	-0.05787	0.038063	-1.52031	0.128434	0.191464	KIAA0355	protein_co
ENSG0000	2043.646	0.346001	0.044605	7.757011	8.70E-15	1.28E-13	UXT	protein_co
ENSG0000	2043.625	-0.09567	0.033081	-2.89209	0.003827	0.008353	RP11-357	processed
ENSG0000	2042.953	-0.3873	0.086154	-4.4955	6.94E-06	2.46E-05	SLC2A13	protein_co
ENSG0000	2041.983	-0.77809	0.150049	-5.18559	2.15E-07	9.67E-07	CRLF1	protein_co
ENSG0000	2041.75	0.096902	0.027807	3.484748	0.000493	0.001272	MCCC1	protein_co
ENSG0000	2041.201	-0.10842	0.091632	-1.18322	0.236722	0.321689	GOLGA8A	protein_co
ENSG0000	2040.91	0.290072	0.034386	8.435797	3.29E-17	7.23E-16	TOR3A	protein_co
ENSG0000	2040.785	0.047048	0.042045	1.118998	0.263141	0.351763	ADAM17	protein_co
ENSG0000	2040.552	0.151257	0.034689	4.360346	1.30E-05	4.40E-05	NDUFS4	protein_co
ENSG0000	2040.478	0.234705	0.038913	6.031493	1.62E-09	1.02E-08	FRMD8	protein_co
ENSG0000	2040.354	0.043232	0.090939	0.475393	0.634507	0.71538	CPM	protein_co

ENSG0000(	2039.747	-0.39627	0.111458	-3.55533	0.000378	0.000998	NRSN1	protein_cc
ENSG0000(	2039.728	0.211214	0.038049	5.551038	2.84E-08	1.47E-07	MRPL27	protein_cc
ENSG0000(	2039.117	0.027957	0.062426	0.447843	0.654266	0.732197	ZFHX4	protein_cc
ENSG0000(	2038.281	0.266786	0.041909	6.365878	1.94E-10	1.41E-09	WDR18	protein_cc
ENSG0000(	2037.102	0.121856	0.033698	3.616065	0.000299	0.000805	YIPF5	protein_cc
ENSG0000(	2036.417	-0.0609	0.058597	-1.03935	0.298644	0.390906	TCF7L1	protein_cc
ENSG0000(	2036.351	0.083633	0.058811	1.422058	0.155009	0.225028	SCPEP1	protein_cc
ENSG0000(	2036.27	0.142102	0.036594	3.883166	0.000103	0.0003	OSGEP	protein_cc
ENSG0000(	2036.044	-0.79842	0.089517	-8.91928	4.69E-19	1.37E-17	PRRT2	protein_cc
ENSG0000(	2035.893	-0.19804	0.035521	-5.5754	2.47E-08	1.29E-07	TMEM25	protein_cc
ENSG0000(	2035.396	0.070991	0.032273	2.199728	0.027826	0.050124	NAA40	protein_cc
ENSG0000(	2034.946	0.17816	0.037955	4.693954	2.68E-06	1.01E-05	KRIT1	protein_cc
ENSG0000(	2034.943	0.173063	0.023728	7.29355	3.02E-13	3.40E-12	DERL2	protein_cc
ENSG0000(	2034.509	0.009388	0.031579	0.297298	0.766239	0.824566	LARP4	protein_cc
ENSG0000(	2034.298	0.766045	0.193638	3.956076	7.62E-05	0.000227	NNMT	protein_cc
ENSG0000(	2034.135	0.213868	0.028742	7.441018	9.99E-14	1.21E-12	PSMG2	protein_cc
ENSG0000(	2033.874	-0.03106	0.040327	-0.77026	0.441148	0.538044	SMARCD2	protein_cc
ENSG0000(	2033.42	-0.23304	0.042143	-5.52981	3.21E-08	1.64E-07	TRIM23	protein_cc
ENSG0000(	2033.22	0.316556	0.055736	5.67955	1.35E-08	7.34E-08	BLOC1S1	protein_cc
ENSG0000(	2032.546	0.409569	0.055738	7.348148	2.01E-13	2.31E-12	IER5	protein_cc
ENSG0000(	2032.364	0.065178	0.041547	1.568778	0.1167	0.176517	ING5	protein_cc
ENSG0000(	2031.635	-0.26757	0.054602	-4.90045	9.56E-07	3.87E-06	BTAF1	protein_cc
ENSG0000(	2031.541	0.050969	0.054796	0.93015	0.352294	0.447852	TSPAN33	protein_cc
ENSG0000(	2031.421	-0.01109	0.03049	-0.36369	0.716088	0.7838	POLR2M	protein_cc
ENSG0000(	2031.353	0.035776	0.020446	1.749787	0.080155	0.127475	TARS2	protein_cc
ENSG0000(	2030.735	-0.10644	0.025409	-4.18919	2.80E-05	8.98E-05	SENP5	protein_cc
ENSG0000(	2030.249	-0.53607	0.06154	-8.71094	3.01E-18	7.80E-17	MAP3K10	protein_cc
ENSG0000(	2029.888	-0.05162	0.034089	-1.51442	0.129919	0.193258	WDR35	protein_cc
ENSG0000(	2029.817	-0.26474	0.054705	-4.83936	1.30E-06	5.17E-06	TMCC3	protein_cc
ENSG0000(	2029.745	0.025192	0.026197	0.961631	0.336235	0.431053	GOLGA1	protein_cc
ENSG0000(	2028.888	0.317106	0.047664	6.652981	2.87E-11	2.38E-10	ALKBH7	protein_cc
ENSG0000(	2028.315	-0.15036	0.024739	-6.0778	1.22E-09	7.84E-09	SLC4A1AF	protein_cc
ENSG0000(	2028.272	-0.74365	0.067462	-11.0231	2.96E-28	4.75E-26	PDP1	protein_cc
ENSG0000(	2028.234	0.075535	0.045984	1.642641	0.100457	0.155122	FUT8	protein_cc
ENSG0000(	2028.206	0.14188	0.039171	3.622095	0.000292	0.000788	TMEM261	protein_cc
ENSG0000(	2026.834	-0.18021	0.037458	-4.81092	1.50E-06	5.91E-06	KLF7	protein_cc
ENSG0000(	2026.184	-0.13231	0.036389	-3.63596	0.000277	0.00075	VPS36	protein_cc
ENSG0000(	2025.895	0.185522	0.034846	5.324068	1.01E-07	4.81E-07	DCAF13	protein_cc
ENSG0000(	2025.543	0.407612	0.084062	4.848922	1.24E-06	4.95E-06	MICALL2	protein_cc
ENSG0000(	2024.517	-0.63163	0.108679	-5.81182	6.18E-09	3.54E-08	CELF5	protein_cc
ENSG0000(	2024.001	-0.16588	0.038164	-4.34641	1.38E-05	4.67E-05	ZBTB40	protein_cc
ENSG0000(	2023.142	-0.16551	0.035074	-4.71877	2.37E-06	9.04E-06	RASA1	protein_cc
ENSG0000(	2022.596	-0.08464	0.044019	-1.92288	0.054496	0.09113	TFEB	protein_cc
ENSG0000(	2021.949	-0.44279	0.049028	-9.03142	1.69E-19	5.34E-18	SCAPER	protein_cc
ENSG0000(	2021.686	-1.18071	0.100391	-11.7611	6.20E-32	2.05E-29	ITPR1	protein_cc
ENSG0000(	2020.518	0.112572	0.031417	3.58317	0.000339	0.000905	PPIE	protein_cc
ENSG0000(	2019.954	-0.22003	0.045692	-4.81536	1.47E-06	5.79E-06	DENND4C	protein_cc
ENSG0000(	2019.917	-0.33121	0.035901	-9.22562	2.82E-20	9.98E-19	SHOC2	protein_cc
ENSG0000(	2019.883	-0.03082	0.048254	-0.63864	0.523056	0.615079	CCS	protein_cc
ENSG0000(	2018.995	-0.0536	0.053237	-1.00684	0.314013	0.407403	TMEM206	protein_cc
ENSG0000(	2018.46	0.014555	0.03539	0.411259	0.680883	0.754327	PROSER1	protein_cc
ENSG0000(	2017.965	0.202002	0.025346	7.969698	1.59E-15	2.66E-14	NUDCD2	protein_cc
ENSG0000(	2017.91	0.129655	0.024485	5.295254	1.19E-07	5.57E-07	THAP11	protein_cc
ENSG0000(	2017.74	0.248408	0.056056	4.431421	9.36E-06	3.25E-05	BMP1	protein_cc
ENSG0000(	2017.376	0.195764	0.033775	5.796167	6.78E-09	3.87E-08	PGAM5	protein_cc
ENSG0000(	2016.11	-0.20189	0.075745	-2.66543	0.007689	0.015765	ATP13A4	protein_cc
ENSG0000(	2015.247	0.247795	0.109497	2.263035	0.023634	0.04328	CD93	protein_cc
ENSG0000(	2014.752	-0.24339	0.058978	-4.12681	3.68E-05	0.000116	PCYT1B	protein_cc
ENSG0000(	2014.635	0.477554	0.097007	4.922902	8.53E-07	3.48E-06	FGFRL1	protein_cc

ENSG0000(	2014.583	-0.18858	0.040151	-4.69672	2.64E-06	9.99E-06	UBAP2	protein_cc
ENSG0000(	2013.74	-0.17475	0.041231	-4.23828	2.25E-05	7.33E-05	NRIP1	protein_cc
ENSG0000(	2013.662	0.027083	0.032775	0.826348	0.408607	0.50543	COG5	protein_cc
ENSG0000(	2013.268	-0.10245	0.055962	-1.83074	0.067139	0.109253	SH3D19	protein_cc
ENSG0000(	2012.951	0.018381	0.033113	0.555094	0.57883	0.66586	FOXRED1	protein_cc
ENSG0000(	2012.845	0.541635	0.086687	6.248163	4.15E-10	2.87E-09	CDH4	protein_cc
ENSG0000(	2012.223	-0.44142	0.044929	-9.82478	8.81E-23	4.91E-21	ZBTB7A	protein_cc
ENSG0000(	2012.002	0.278608	0.12908	2.158414	0.030896	0.055069	SERPINA1	protein_cc
ENSG0000(	2011.71	0.000149	0.043255	0.003435	0.997259	0.998279	ZCCHC11	protein_cc
ENSG0000(	2011.641	0.227724	0.042869	5.312059	1.08E-07	5.11E-07	MRPL52	protein_cc
ENSG0000(	2011.577	-0.00243	0.039382	-0.0618	0.95072	0.965988	PFAS	protein_cc
ENSG0000(	2011.327	0.025824	0.028557	0.904306	0.365833	0.461942	FAM122B	protein_cc
ENSG0000(	2011.217	0.261836	0.058514	4.474747	7.65E-06	2.69E-05	RNF180	protein_cc
ENSG0000(	2011.175	-0.50591	0.06134	-8.24763	1.62E-16	3.18E-15	PDZRN3	protein_cc
ENSG0000(	2010.19	0.312658	0.057423	5.444801	5.19E-08	2.57E-07	HN1L	protein_cc
ENSG0000(	2010.155	-0.26861	0.068691	-3.9105	9.21E-05	0.000271	ACTN2	protein_cc
ENSG0000(	2009.407	0.108347	0.103007	1.051839	0.292874	0.38449	RP11-742	pseudoge
ENSG0000(	2008.798	-0.09763	0.05603	-1.74243	0.081433	0.129155	GPR161	protein_cc
ENSG0000(	2008.645	0.503842	0.100839	4.996513	5.84E-07	2.45E-06	CPVL	protein_cc
ENSG0000(	2008.604	-0.15509	0.064679	-2.39782	0.016493	0.031345	TP53I11	protein_cc
ENSG0000(	2008.02	-0.23149	0.032939	-7.02791	2.10E-12	2.08E-11	PCMTD1	protein_cc
ENSG0000(	2007.877	-0.29884	0.082186	-3.6361	0.000277	0.000749	NCKAP1L	protein_cc
ENSG0000(	2007.223	0.096235	0.039174	2.456583	0.014027	0.027107	NUCB2	protein_cc
ENSG0000(	2007.12	-0.13849	0.086754	-1.59639	0.110402	0.168242	LILRB4	protein_cc
ENSG0000(	2006.074	-0.28388	0.102036	-2.78215	0.0054	0.011431	NSG1	protein_cc
ENSG0000(	2005.155	-0.1129	0.036363	-3.10467	0.001905	0.004412	BRD1	protein_cc
ENSG0000(	2004.612	0.16307	0.038086	4.281668	1.85E-05	6.14E-05	GTPBP2	protein_cc
ENSG0000(	2004.097	0.062713	0.030212	2.075763	0.037916	0.066172	UXS1	protein_cc
ENSG0000(	2004.003	0.236335	0.075001	3.151111	0.001627	0.003817	NDP	protein_cc
ENSG0000(	2003.972	0.160644	0.028635	5.610143	2.02E-08	1.07E-07	THAP5	protein_cc
ENSG0000(	2003.683	-0.03969	0.033494	-1.18507	0.235991	0.320812	TTLL4	protein_cc
ENSG0000(	2003.291	-0.00649	0.035054	-0.18519	0.85308	0.893765	ARPC5L	protein_cc
ENSG0000(	2002.479	-0.14084	0.029889	-4.71209	2.45E-06	9.32E-06	TMEM57	protein_cc
ENSG0000(	2001.69	-0.08089	0.028134	-2.87521	0.004038	0.008776	GPATCH1:	protein_cc
ENSG0000(	2001.548	0.017319	0.030888	0.560718	0.57499	0.662219	TMEM41B	protein_cc
ENSG0000(	2001.355	-0.05159	0.058245	-0.88574	0.37576	0.472344	RASSF8	protein_cc
ENSG0000(	2001.122	-0.2381	0.062635	-3.80141	0.000144	0.000409	TMEM229	protein_cc
ENSG0000(	2001.021	-0.26023	0.035402	-7.35083	1.97E-13	2.27E-12	PDPK1	protein_cc
ENSG0000(	2001.007	-0.15634	0.029431	-5.31213	1.08E-07	5.11E-07	ZFYVE1	protein_cc
ENSG0000(	2000.373	0.039867	0.040772	0.977798	0.328174	0.422577	LYRM5	protein_cc
ENSG0000(	2000.371	-0.11582	0.034531	-3.35412	0.000796	0.001977	SETD1B	protein_cc
ENSG0000(	1999.944	0.07334	0.042844	1.71178	0.086937	0.136861	ZNF444	protein_cc
ENSG0000(	1999.336	-0.47809	0.047454	-10.0748	7.14E-24	5.06E-22	CCDC85C	protein_cc
ENSG0000(	1998.702	0.029618	0.028214	1.049747	0.293834	0.385587	RNF26	protein_cc
ENSG0000(	1998.531	-0.00145	0.043028	-0.03375	0.97308	0.981168	KIAA0556	protein_cc
ENSG0000(	1998.447	-0.22589	0.027578	-8.19097	2.59E-16	4.92E-15	RPS6KC1	protein_cc
ENSG0000(	1997.991	0.291203	0.048677	5.9824	2.20E-09	1.36E-08	C1orf86	protein_cc
ENSG0000(	1997.878	0.216344	0.034745	6.226585	4.77E-10	3.26E-09	BRMS1	protein_cc
ENSG0000(	1997.783	-0.31726	0.045874	-6.91586	4.65E-12	4.35E-11	FAM13A	protein_cc
ENSG0000(	1997.388	-0.43964	0.074714	-5.88427	4.00E-09	2.37E-08	GALNT16	protein_cc
ENSG0000(	1997.33	-0.0031	0.035491	-0.08722	0.930495	0.951267	CARM1	protein_cc
ENSG0000(	1997.329	0.098395	0.049457	1.989508	0.046645	0.079437	ANKZF1	protein_cc
ENSG0000(	1996.994	-0.1631	0.028807	-5.66162	1.50E-08	8.09E-08	SYNJ2BP	protein_cc
ENSG0000(	1996.92	0.157968	0.030113	5.24575	1.56E-07	7.15E-07	HKR1	protein_cc
ENSG0000(	1996.793	0.160435	0.032494	4.937369	7.92E-07	3.25E-06	MSANTD4	protein_cc
ENSG0000(	1996.464	0.036131	0.030738	1.175424	0.239825	0.325165	DGCR8	protein_cc
ENSG0000(	1995.798	0.248733	0.038137	6.5221	6.93E-11	5.42E-10	GMPPA	protein_cc
ENSG0000(	1995.555	-0.20706	0.052381	-3.95298	7.72E-05	0.00023	RUSC1	protein_cc
ENSG0000(	1995.261	-0.96847	0.10787	-8.97813	2.75E-19	8.38E-18	NEGR1	protein_cc

ENSG0000(	1994.872	0.266682	0.027627	9.652953	4.78E-22	2.32E-20	CPSF3	protein_cc
ENSG0000(	1994.865	-0.11635	0.034866	-3.33708	0.000847	0.002091	NAPEPLD	protein_cc
ENSG0000(	1994.398	0.110582	0.022516	4.911319	9.05E-07	3.68E-06	RFWD2	protein_cc
ENSG0000(	1994.319	-0.11541	0.034033	-3.39117	0.000696	0.001746	FOXJ2	protein_cc
ENSG0000(	1994.085	-0.35601	0.051493	-6.91363	4.72E-12	4.41E-11	RAB11FIP5	protein_cc
ENSG0000(	1993.983	-0.54817	0.105558	-5.19309	2.07E-07	9.32E-07	TMEFF2	protein_cc
ENSG0000(	1993.269	-0.34973	0.062041	-5.63707	1.73E-08	9.25E-08	NFAT5	protein_cc
ENSG0000(	1992.828	0.104908	0.043105	2.433767	0.014943	0.028684	GHDC	protein_cc
ENSG0000(	1992.003	0.046372	0.028351	1.635649	0.101913	0.157064	C9orf69	protein_cc
ENSG0000(	1991.786	1.073521	0.110468	9.717895	2.53E-22	1.30E-20	IL1RAP	protein_cc
ENSG0000(	1991.71	-0.17951	0.030864	-5.81614	6.02E-09	3.46E-08	PLEKHM1	protein_cc
ENSG0000(	1991.705	-0.15483	0.042131	-3.67496	0.000238	0.000651	SLC25A12	protein_cc
ENSG0000(	1991.269	0.172202	0.050372	3.418635	0.000629	0.001593	SNHG7	antisense
ENSG0000(	1990.254	-0.39665	0.09574	-4.14292	3.43E-05	0.000108	RGS11	protein_cc
ENSG0000(	1990.032	-0.23192	0.04323	-5.36467	8.11E-08	3.89E-07	NEK1	protein_cc
ENSG0000(	1990.004	-0.18238	0.04445	-4.10306	4.08E-05	0.000127	HMG20A	protein_cc
ENSG0000(	1989.936	-0.08755	0.067697	-1.29328	0.195913	0.274412	MMP15	protein_cc
ENSG0000(	1989.714	-0.23994	0.03892	-6.165	7.05E-10	4.71E-09	MPP1	protein_cc
ENSG0000(	1989.612	0.009535	0.030059	0.317218	0.751078	0.812419	PHF8	protein_cc
ENSG0000(	1989.406	0.243583	0.040551	6.006786	1.89E-09	1.18E-08	SLC52A2	protein_cc
ENSG0000(	1988.66	0.14019	0.037549	3.733486	0.000189	0.000526	POFUT2	protein_cc
ENSG0000(	1987.983	-0.20091	0.050248	-3.99831	6.38E-05	0.000193	CCP110	protein_cc
ENSG0000(	1987.248	-0.00325	0.0999	-0.03251	0.974068	0.981875	HAP1	protein_cc
ENSG0000(	1986.943	-0.14394	0.072284	-1.99136	0.046442	0.079129	GPC4	protein_cc
ENSG0000(	1986.38	-0.06535	0.027779	-2.35237	0.018654	0.034975	DCUN1D1	protein_cc
ENSG0000(	1986.262	0.102972	0.03341	3.082045	0.002056	0.004735	CNTROB	protein_cc
ENSG0000(	1986.156	-1.50868	0.141218	-10.6833	1.22E-26	1.48E-24	CHD5	protein_cc
ENSG0000(	1986.047	-0.08452	0.032603	-2.5923	0.009534	0.01914	SH3YL1	protein_cc
ENSG0000(	1985.77	0.057023	0.044771	1.273669	0.202781	0.282597	TOB1	protein_cc
ENSG0000(	1985.755	0.0876	0.024859	3.523905	0.000425	0.001113	USP21	protein_cc
ENSG0000(	1985.498	-0.34543	0.058394	-5.91561	3.31E-09	1.98E-08	1-Mar	protein_cc
ENSG0000(	1985.209	0.013347	0.02856	0.467319	0.640272	0.72026	ERGIC2	protein_cc
ENSG0000(	1985.186	-0.12916	0.039063	-3.30654	0.000945	0.002312	PPP1R37	protein_cc
ENSG0000(	1984.414	-0.17684	0.059799	-2.95726	0.003104	0.006896	MAN2A1	protein_cc
ENSG0000(	1984.326	0.080299	0.01887	4.255295	2.09E-05	6.85E-05	DEDD	protein_cc
ENSG0000(	1983.55	0.01628	0.030343	0.536526	0.591595	0.677209	CINP	protein_cc
ENSG0000(	1982.89	-0.16942	0.034207	-4.95268	7.32E-07	3.02E-06	FAM214B	protein_cc
ENSG0000(	1982.242	0.122552	0.021895	5.597182	2.18E-08	1.15E-07	GGPS1	protein_cc
ENSG0000(	1979.236	-0.01147	0.023505	-0.48796	0.625578	0.707601	ZNF451	protein_cc
ENSG0000(	1978.272	0.292285	0.041209	7.09278	1.31E-12	1.34E-11	PDCD5	protein_cc
ENSG0000(	1977.227	-0.26423	0.058206	-4.53967	5.63E-06	2.03E-05	GPR116	protein_cc
ENSG0000(	1977.102	0.472511	0.087895	5.375851	7.62E-08	3.68E-07	FAM84A	protein_cc
ENSG0000(	1976.637	-0.06815	0.030525	-2.2327	0.025569	0.046466	TCAIM	protein_cc
ENSG0000(	1976.369	0.191679	0.0424	4.520753	6.16E-06	2.20E-05	ADPGK	protein_cc
ENSG0000(	1975.358	0.051577	0.025365	2.033356	0.042017	0.072472	ASB6	protein_cc
ENSG0000(	1975.09	0.555584	0.043664	12.72405	4.35E-37	4.16E-34	LSM5	protein_cc
ENSG0000(	1974.401	-0.24709	0.056972	-4.33703	1.44E-05	4.86E-05	DOPEY1	protein_cc
ENSG0000(	1973.845	0.248464	0.089364	2.780358	0.00543	0.01149	ROBO2	protein_cc
ENSG0000(	1973.201	-0.04955	0.032201	-1.53862	0.123896	0.185689	TIMM23B	protein_cc
ENSG0000(	1972.434	-0.03829	0.033801	-1.13267	0.257353	0.34523	FBXO42	protein_cc
ENSG0000(	1972.05	0.063456	0.03399	1.866923	0.061912	0.10177	EIF4E	protein_cc
ENSG0000(	1971.807	-0.03476	0.055935	-0.62147	0.534293	0.625664	NAGA	protein_cc
ENSG0000(	1970.549	0.065884	0.03897	1.690645	0.090905	0.142213	YAF2	protein_cc
ENSG0000(	1970.476	-0.22507	0.043104	-5.22143	1.78E-07	8.08E-07	CLOCK	protein_cc
ENSG0000(	1970.365	0.043508	0.017969	2.421297	0.015465	0.029592	MAPKAPK	protein_cc
ENSG0000(	1970.358	0.071265	0.064034	1.112919	0.265743	0.354583	UBA7	protein_cc
ENSG0000(	1970.136	0.278837	0.045923	6.071846	1.26E-09	8.12E-09	RBM38	protein_cc
ENSG0000(	1969.893	0.240292	0.040535	5.928038	3.07E-09	1.85E-08	ZNF667-A	lincRNA
ENSG0000(	1969.69	0.186859	0.034691	5.38644	7.19E-08	3.48E-07	CTDSPL2	protein_cc

ENSG0000(	1969.468	0.120288	0.027357	4.397023	1.10E-05	3.77E-05	TBC1D23	protein_cc
ENSG0000(	1968.922	-0.23572	0.043874	-5.37255	7.76E-08	3.74E-07	TBC1D24	protein_cc
ENSG0000(	1967.976	0.092633	0.026262	3.527311	0.00042	0.0011	CRNKL1	protein_cc
ENSG0000(	1967.252	0.159227	0.027527	5.784352	7.28E-09	4.13E-08	PNPT1	protein_cc
ENSG0000(	1966.747	0.084966	0.039971	2.125679	0.03353	0.059276	ASPSCR1	protein_cc
ENSG0000(	1963.494	0.31654	0.04577	6.915887	4.65E-12	4.35E-11	C9orf142	protein_cc
ENSG0000(	1963.383	-0.05369	0.030672	-1.75055	0.080024	0.127307	ZNF326	protein_cc
ENSG0000(	1963.17	-0.07698	0.031246	-2.46385	0.013745	0.026619	SCAF8	protein_cc
ENSG0000(	1962.634	0.070646	0.02497	2.829219	0.004666	0.009996	GTF2H1	protein_cc
ENSG0000(	1962.444	-0.09051	0.041602	-2.17562	0.029583	0.05298	CBR4	protein_cc
ENSG0000(	1961.893	-0.10123	0.031524	-3.21123	0.001322	0.003152	ELK4	protein_cc
ENSG0000(	1961.784	0.026316	0.048443	0.543241	0.586964	0.673257	RGAG4	protein_cc
ENSG0000(	1961.738	-0.1008	0.03031	-3.32566	0.000882	0.002171	INO80	protein_cc
ENSG0000(	1959.966	0.078785	0.079842	0.986759	0.323761	0.417915	MXRA8	protein_cc
ENSG0000(	1959.256	0.012935	0.029928	0.432203	0.665594	0.741906	POLR3GL	protein_cc
ENSG0000(	1958.007	-0.50934	0.070975	-7.17632	7.16E-13	7.62E-12	FBXW7	protein_cc
ENSG0000(	1956.683	-0.02964	0.036367	-0.81502	0.415061	0.512074	TMEM189	protein_cc
ENSG0000(	1954.783	0.02402	0.068933	0.348455	0.727498	0.792856	CYSTM1	protein_cc
ENSG0000(	1954.675	0.060715	0.028835	2.105633	0.035236	0.06201	TSNAX	protein_cc
ENSG0000(	1954.557	-0.11403	0.034224	-3.33183	0.000863	0.002128	RAD54L2	protein_cc
ENSG0000(	1953.816	-0.07884	0.042577	-1.85177	0.064059	0.104896	PLCB3	protein_cc
ENSG0000(	1952.288	-0.10613	0.035119	-3.02202	0.002511	0.005691	ATP8B2	protein_cc
ENSG0000(	1952.265	0.259557	0.048417	5.360848	8.28E-08	3.97E-07	MRPS6	protein_cc
ENSG0000(	1952.216	0.262027	0.112821	2.322508	0.020206	0.037573	TMEM176	protein_cc
ENSG0000(	1951.733	0.063566	0.035486	1.791305	0.073244	0.117904	RRP1	protein_cc
ENSG0000(	1951.012	-0.08618	0.024889	-3.46236	0.000535	0.001374	SP2	protein_cc
ENSG0000(	1950.829	0.372476	0.03616	10.30091	6.98E-25	5.93E-23	IFT52	protein_cc
ENSG0000(	1950.814	0.037672	0.045116	0.835008	0.403713	0.500567	CLASRP	protein_cc
ENSG0000(	1950.356	-0.02225	0.029778	-0.74734	0.454861	0.551514	DAGLB	protein_cc
ENSG0000(	1949.743	0.059664	0.030008	1.988292	0.046779	0.079639	EAPP	protein_cc
ENSG0000(	1947.636	0.510916	0.051845	9.854652	6.54E-23	3.80E-21	DESI2	protein_cc
ENSG0000(	1946.564	0.273505	0.050233	5.444747	5.19E-08	2.57E-07	NAT14	protein_cc
ENSG0000(	1946.419	0.181515	0.104041	1.744653	0.081045	0.128663	BEST3	protein_cc
ENSG0000(	1946.103	-0.03672	0.039209	-0.93663	0.348951	0.444377	ASF1A	protein_cc
ENSG0000(	1945.683	0.245238	0.029319	8.364559	6.03E-17	1.27E-15	RYK	protein_cc
ENSG0000(	1945.369	-0.28655	0.050249	-5.70258	1.18E-08	6.48E-08	RASAL2	protein_cc
ENSG0000(	1945.244	-0.05034	0.029352	-1.71497	0.08635	0.136024	MSL2	protein_cc
ENSG0000(	1945.213	0.071361	0.024016	2.971393	0.002965	0.006616	PAPD4	protein_cc
ENSG0000(	1945.109	-0.2628	0.048827	-5.38222	7.36E-08	3.56E-07	MMD	protein_cc
ENSG0000(	1944.997	-0.25803	0.053318	-4.83948	1.30E-06	5.17E-06	TLE4	protein_cc
ENSG0000(	1944.937	-0.00581	0.069222	-0.084	0.933053	0.953289	CNIH2	protein_cc
ENSG0000(	1944.918	0.568867	0.054528	10.43264	1.76E-25	1.67E-23	CASP3	protein_cc
ENSG0000(	1944.254	-0.58396	0.141534	-4.12598	3.69E-05	0.000116	SHISA6	protein_cc
ENSG0000(	1943.487	-0.0252	0.025651	-0.9824	0.325902	0.420076	ZBTB22	protein_cc
ENSG0000(	1943.21	0.113226	0.027915	4.056067	4.99E-05	0.000154	GGCX	protein_cc
ENSG0000(	1942.85	-0.08974	0.03441	-2.60804	0.009106	0.018357	CDK12	protein_cc
ENSG0000(	1942.815	0.174494	0.057429	3.038426	0.002378	0.005413	B3GALNT:	protein_cc
ENSG0000(	1942.121	-0.18546	0.039723	-4.66882	3.03E-06	1.13E-05	RAB40C	protein_cc
ENSG0000(	1941.692	0.414341	0.060781	6.816962	9.30E-12	8.29E-11	TMEM54	protein_cc
ENSG0000(	1941.669	-0.18001	0.03965	-4.54008	5.62E-06	2.02E-05	SLC25A28	protein_cc
ENSG0000(	1941.512	-0.45477	0.05297	-8.58534	9.06E-18	2.19E-16	KLHL2	protein_cc
ENSG0000(	1941.071	-0.0338	0.038468	-0.87862	0.379609	0.476171	TOMM34	protein_cc
ENSG0000(	1940.473	0.038561	0.031467	1.225464	0.220401	0.303024	PEX10	protein_cc
ENSG0000(	1940.141	-0.12937	0.07915	-1.63453	0.102148	0.157366	CXXC4	protein_cc
ENSG0000(	1938.051	-1.53845	0.123969	-12.4099	2.31E-35	1.56E-32	RASGRF1	protein_cc
ENSG0000(	1937.952	0.011401	0.035611	0.320165	0.748843	0.810833	ZZZ3	protein_cc
ENSG0000(	1937.869	-0.02896	0.082743	-0.34994	0.726381	0.791848	CECR1	protein_cc
ENSG0000(	1936.843	-0.05924	0.045873	-1.29134	0.196584	0.275212	ERO1L	protein_cc
ENSG0000(	1935.643	-0.35259	0.039936	-8.82882	1.06E-18	2.93E-17	ENTPD4	protein_cc

ENSG0000(	1934.996	-0.27477	0.046171	-5.9512	2.66E-09	1.62E-08	PTBP2	protein_cc
ENSG0000(	1934.728	-0.08021	0.026937	-2.97768	0.002904	0.006492	MRPL19	protein_cc
ENSG0000(	1934.198	-0.0842	0.046031	-1.82912	0.067382	0.109612	UBN2	protein_cc
ENSG0000(	1934.184	0.236117	0.041965	5.626489	1.84E-08	9.80E-08	ADPRHL2	protein_cc
ENSG0000(	1932.409	0.051664	0.025952	1.990795	0.046503	0.079225	WDR75	protein_cc
ENSG0000(	1932.289	0.594142	0.125427	4.736941	2.17E-06	8.32E-06	COL9A3	protein_cc
ENSG0000(	1932.169	0.173756	0.049555	3.50634	0.000454	0.001182	NAB2	protein_cc
ENSG0000(	1932.167	0.52891	0.080596	6.562491	5.29E-11	4.20E-10	TLE2	protein_cc
ENSG0000(	1932.024	0.227445	0.033897	6.709814	1.95E-11	1.65E-10	PDCD2	protein_cc
ENSG0000(	1932.002	0.097803	0.033518	2.917931	0.003524	0.007746	CLK2	protein_cc
ENSG0000(	1931.635	0.145552	0.063783	2.281984	0.02249	0.041419	NEURL1B	protein_cc
ENSG0000(	1931.353	0.174338	0.036635	4.758729	1.95E-06	7.52E-06	BCAS2	protein_cc
ENSG0000(	1931.155	-0.4513	0.049008	-9.20868	3.30E-20	1.16E-18	BTBD9	protein_cc
ENSG0000(	1930.865	-0.35118	0.053548	-6.55833	5.44E-11	4.31E-10	TTBK1	protein_cc
ENSG0000(	1930.674	0.006958	0.047614	0.146134	0.883816	0.917105	PPAP2A	protein_cc
ENSG0000(	1930.329	0.593506	0.07416	8.003078	1.21E-15	2.07E-14	TSPAN12	protein_cc
ENSG0000(	1930.094	0.364075	0.047898	7.601056	2.94E-14	3.90E-13	SIVA1	protein_cc
ENSG0000(	1930.009	-0.03488	0.032347	-1.07821	0.28094	0.371528	OTUD4	protein_cc
ENSG0000(	1929.889	0.110307	0.02735	4.033128	5.50E-05	0.000168	SUZ12	protein_cc
ENSG0000(	1928.904	0.003491	0.030908	0.112947	0.910072	0.936395	REXO4	protein_cc
ENSG0000(	1928.311	-0.47457	0.085203	-5.56996	2.55E-08	1.33E-07	NTNG2	protein_cc
ENSG0000(	1928.056	0.343689	0.051561	6.665642	2.64E-11	2.20E-10	NME1-NM	protein_cc
ENSG0000(	1927.744	-0.045	0.031586	-1.42453	0.154292	0.2242	C2CD3	protein_cc
ENSG0000(	1927.599	0.545029	0.056482	9.649692	4.93E-22	2.39E-20	TIMM8B	protein_cc
ENSG0000(	1927.587	0.334122	0.039532	8.451873	2.87E-17	6.40E-16	TMEM208	protein_cc
ENSG0000(	1927.422	-0.633	0.052211	-12.1238	7.90E-34	3.96E-31	PPARA	protein_cc
ENSG0000(	1926.414	0.201471	0.033455	6.022151	1.72E-09	1.08E-08	KRCC1	protein_cc
ENSG0000(	1926.196	-0.22196	0.084007	-2.64215	0.008238	0.016786	HR	protein_cc
ENSG0000(	1926.18	-0.44809	0.041371	-10.8311	2.45E-27	3.33E-25	GCC2	protein_cc
ENSG0000(	1926.153	-0.08402	0.043526	-1.93043	0.053553	0.089741	TMOD3	protein_cc
ENSG0000(	1925.6	0.002394	0.059246	0.040411	0.967765	0.977751	TCEAL7	protein_cc
ENSG0000(	1925.392	0.032125	0.040796	0.787455	0.431015	0.528051	CARD8	protein_cc
ENSG0000(	1925.271	-0.02249	0.051975	-0.43265	0.665268	0.741703	MYO1C	protein_cc
ENSG0000(	1924.895	-0.29072	0.077885	-3.73267	0.000189	0.000527	RHBDF2	protein_cc
ENSG0000(	1924.824	0.138049	0.036102	3.823895	0.000131	0.000376	AAGAB	protein_cc
ENSG0000(	1924.193	-0.34044	0.045398	-7.49911	6.43E-14	8.11E-13	TCF20	protein_cc
ENSG0000(	1924.087	-0.23258	0.038923	-5.97523	2.30E-09	1.41E-08	PPRC1	protein_cc
ENSG0000(	1923.6	0.156324	0.042294	3.696114	0.000219	0.000603	OSTM1	protein_cc
ENSG0000(	1923.32	0.036275	0.030598	1.185539	0.235804	0.32059	EIF2B2	protein_cc
ENSG0000(	1923.224	0.044925	0.022104	2.03245	0.042108	0.072603	DHX16	protein_cc
ENSG0000(	1923.007	-0.21939	0.081761	-2.68326	0.007291	0.015017	PRELP	protein_cc
ENSG0000(	1922.904	-0.01905	0.035718	-0.53332	0.593815	0.679165	UCK2	protein_cc
ENSG0000(	1922.728	0.246958	0.033316	7.41263	1.24E-13	1.48E-12	FAM120A(	protein_cc
ENSG0000(	1922.693	-0.10588	0.119233	-0.88803	0.374524	0.471108	HIF3A	protein_cc
ENSG0000(	1922.637	0.24658	0.037216	6.625703	3.46E-11	2.83E-10	MRPL15	protein_cc
ENSG0000(	1922.585	0.347184	0.054787	6.336939	2.34E-10	1.68E-09	TMEM231	protein_cc
ENSG0000(	1921.926	0.020087	0.110045	0.182532	0.855165	0.895333	FCGR2A	protein_cc
ENSG0000(	1921.769	0.143431	0.026881	5.335809	9.51E-08	4.52E-07	RSBN1L	protein_cc
ENSG0000(	1920.229	0.385974	0.044894	8.597531	8.14E-18	1.98E-16	HEATR2	protein_cc
ENSG0000(	1919.945	0.303107	0.035732	8.482758	2.20E-17	5.00E-16	OSER1	protein_cc
ENSG0000(	1919.746	0.055146	0.023051	2.392324	0.016742	0.031763	PIAS2	protein_cc
ENSG0000(	1919.381	-0.01258	0.021914	-0.57385	0.566071	0.654054	TLK2	protein_cc
ENSG0000(	1919.198	0.040789	0.026051	1.565751	0.117407	0.177405	TATDN2	protein_cc
ENSG0000(	1919.177	0.067569	0.034582	1.953896	0.050714	0.085557	ERCC2	protein_cc
ENSG0000(	1918.331	-0.52557	0.12041	-4.36479	1.27E-05	4.32E-05	SSTR2	protein_cc
ENSG0000(	1918.264	-0.15555	0.033231	-4.68097	2.86E-06	1.07E-05	CNOT6L	protein_cc
ENSG0000(	1917.964	0.099164	0.03689	2.688107	0.007186	0.014813	IRF2BP1	protein_cc
ENSG0000(	1917.935	-0.0593	0.10144	-0.58454	0.558859	0.647943	PRSS23	protein_cc
ENSG0000(	1917.31	-0.16553	0.033701	-4.91193	9.02E-07	3.67E-06	UBR1	protein_cc

ENSG0000(	1917.042	0.148295	0.034476	4.301372	1.70E-05	5.65E-05	RAB9A	protein_cc
ENSG0000(	1916.921	-1.38465	0.128782	-10.7519	5.80E-27	7.44E-25	CAMKV	protein_cc
ENSG0000(	1916.2	0.144615	0.02348	6.159133	7.31E-10	4.87E-09	CDC23	protein_cc
ENSG0000(	1916.022	-0.03295	0.044197	-0.7456	0.455907	0.552391	RPS6KA3	protein_cc
ENSG0000(	1915.954	0.031924	0.035224	0.906324	0.364764	0.460814	CDYL	protein_cc
ENSG0000(	1915.573	0.533019	0.123749	4.307271	1.65E-05	5.51E-05	TGFB2	protein_cc
ENSG0000(	1915.438	0.348597	0.067281	5.181219	2.20E-07	9.89E-07	PTP4A3	protein_cc
ENSG0000(	1915.343	-0.45011	0.054975	-8.18752	2.67E-16	5.06E-15	GPAM	protein_cc
ENSG0000(	1915.023	0.119987	0.08906	1.347254	0.177898	0.252839	SLITRK3	protein_cc
ENSG0000(	1913.956	-0.38036	0.09035	-4.20983	2.56E-05	8.25E-05	CACNA2D	protein_cc
ENSG0000(	1913.67	0.264871	0.057781	4.584093	4.56E-06	1.66E-05	NME3	protein_cc
ENSG0000(	1913.567	-0.0379	0.030867	-1.22779	0.219528	0.301975	CDK11B	protein_cc
ENSG0000(	1913.431	-0.09224	0.032235	-2.86151	0.004216	0.009131	NICN1	protein_cc
ENSG0000(	1911.643	-0.31939	0.057299	-5.57402	2.49E-08	1.30E-07	GNB5	protein_cc
ENSG0000(	1910.641	-0.12434	0.043392	-2.86558	0.004162	0.009024	FAM13B	protein_cc
ENSG0000(	1910.247	-0.09933	0.065084	-1.52614	0.126974	0.1897	FSD1	protein_cc
ENSG0000(	1910.2	0.526873	0.065598	8.031893	9.60E-16	1.66E-14	OSTC	protein_cc
ENSG0000(	1909.873	0.522754	0.090279	5.790453	7.02E-09	3.99E-08	VAMP5	protein_cc
ENSG0000(	1909.862	0.317931	0.053023	5.996045	2.02E-09	1.25E-08	BICD1	protein_cc
ENSG0000(	1909.5	-0.33482	0.111409	-3.0053	0.002653	0.00598	FAM155A	protein_cc
ENSG0000(	1908.839	0.250829	0.036074	6.953082	3.57E-12	3.41E-11	CRY1	protein_cc
ENSG0000(	1907.553	-0.43376	0.065465	-6.62582	3.45E-11	2.83E-10	DLG3	protein_cc
ENSG0000(	1907.096	-0.13719	0.045339	-3.02582	0.00248	0.005625	TET2	protein_cc
ENSG0000(	1906.75	-0.20001	0.038446	-5.20225	1.97E-07	8.90E-07	FER	protein_cc
ENSG0000(	1906.469	-0.24545	0.036567	-6.71215	1.92E-11	1.63E-10	RPS6KA4	protein_cc
ENSG0000(	1905.482	-0.7148	0.095537	-7.48196	7.32E-14	9.14E-13	AMPH	protein_cc
ENSG0000(	1904.962	-0.20066	0.04208	-4.76855	1.86E-06	7.19E-06	MICALL1	protein_cc
ENSG0000(	1904.393	0.002996	0.038843	0.077134	0.938517	0.957065	SPAG16	protein_cc
ENSG0000(	1904.388	0.081623	0.028546	2.85931	0.004246	0.009184	NARG2	protein_cc
ENSG0000(	1903.111	-0.15266	0.032824	-4.65097	3.30E-06	1.23E-05	BMS1	protein_cc
ENSG0000(	1903.083	-0.06851	0.028428	-2.40978	0.015962	0.03045	SLC9A8	protein_cc
ENSG0000(	1902.876	-1.10039	0.115553	-9.52283	1.69E-21	7.51E-20	PEX5L	protein_cc
ENSG0000(	1902.065	-0.19256	0.030126	-6.3919	1.64E-10	1.20E-09	DUSP22	protein_cc
ENSG0000(	1901.912	0.114542	0.039878	2.872326	0.004075	0.008849	PAK4	protein_cc
ENSG0000(	1901.721	-0.11853	0.040708	-2.91167	0.003595	0.007889	STOML1	protein_cc
ENSG0000(	1901.329	-0.17968	0.045274	-3.96874	7.23E-05	0.000216	UBE2E2	protein_cc
ENSG0000(	1901.279	-0.0754	0.028915	-2.60759	0.009118	0.018376	ZNF213	protein_cc
ENSG0000(	1901.249	0.187202	0.041484	4.512661	6.40E-06	2.28E-05	TSPAN17	protein_cc
ENSG0000(	1900.123	0.335058	0.053805	6.227317	4.74E-10	3.25E-09	MANF	protein_cc
ENSG0000(	1899.684	-0.09882	0.059125	-1.67147	0.09463	0.147285	TIE1	protein_cc
ENSG0000(	1899.333	0.075733	0.075874	0.998134	0.318214	0.41188	APOL6	protein_cc
ENSG0000(	1899.2	0.145696	0.040843	3.567197	0.000361	0.000957	DERA	protein_cc
ENSG0000(	1897.801	0.178034	0.039023	4.562279	5.06E-06	1.83E-05	ATP5SL	protein_cc
ENSG0000(	1897.541	-0.63196	0.071392	-8.852	8.60E-19	2.42E-17	SPTBN4	protein_cc
ENSG0000(	1897.027	0.615328	0.054371	11.31711	1.08E-29	2.24E-27	SNRPG	protein_cc
ENSG0000(	1896.789	0.215706	0.03843	5.61292	1.99E-08	1.05E-07	PPA2	protein_cc
ENSG0000(	1896.712	0.194611	0.026868	7.243325	4.38E-13	4.80E-12	UTP18	protein_cc
ENSG0000(	1895.924	-1.50157	0.157478	-9.53511	1.50E-21	6.72E-20	SULT4A1	protein_cc
ENSG0000(	1895.745	0.273818	0.036227	7.558397	4.08E-14	5.30E-13	NUPL2	protein_cc
ENSG0000(	1895.472	-0.29239	0.038843	-7.52752	5.17E-14	6.61E-13	DNAL1	protein_cc
ENSG0000(	1895.333	0.128621	0.118063	1.08942	0.275969	0.366118	MYH7	protein_cc
ENSG0000(	1895.068	-0.84063	0.088106	-9.54114	1.41E-21	6.38E-20	PRKCZ	protein_cc
ENSG0000(	1894.956	0.096025	0.040204	2.388481	0.016918	0.032058	GNL2	protein_cc
ENSG0000(	1893.988	-0.0256	0.052493	-0.48767	0.62578	0.707792	TST	protein_cc
ENSG0000(	1893.954	0.104039	0.110739	0.939493	0.347477	0.442967	PGF	protein_cc
ENSG0000(	1893.941	0.130091	0.034304	3.792299	0.000149	0.000423	NAT9	protein_cc
ENSG0000(	1893.097	0.031538	0.043328	0.727896	0.466678	0.562524	FOXN2	protein_cc
ENSG0000(	1892.243	-0.29961	0.09394	-3.18937	0.001426	0.00338	TMEM119	protein_cc
ENSG0000(	1891.853	0.451205	0.0623	7.242457	4.41E-13	4.83E-12	RAP1B	protein_cc

ENSG0000(	1891.501	-0.33844	0.039214	-8.63049	6.11E-18	1.52E-16	USP12	protein_cc
ENSG0000(	1890.913	-0.40958	0.096667	-4.23701	2.27E-05	7.37E-05	MN1	protein_cc
ENSG0000(	1890.475	0.112379	0.024109	4.661237	3.14E-06	1.17E-05	SLC25A38	protein_cc
ENSG0000(	1890.117	0.397981	0.078428	5.074448	3.89E-07	1.68E-06	ACAA2	protein_cc
ENSG0000(	1889.32	-0.44185	0.045208	-9.77371	1.46E-22	7.86E-21	NEMF	protein_cc
ENSG0000(	1889.308	-0.05688	0.040566	-1.40206	0.160898	0.23215	JAZF1	protein_cc
ENSG0000(	1889.134	-0.34546	0.084868	-4.0706	4.69E-05	0.000145	SCN1A	protein_cc
ENSG0000(	1889.023	-0.29855	0.043335	-6.88933	5.61E-12	5.17E-11	CLIP4	protein_cc
ENSG0000(	1888.31	0.128146	0.037706	3.398522	0.000678	0.001704	CCDC12	protein_cc
ENSG0000(	1887.534	-0.0687	0.026716	-2.5716	0.010123	0.020192	FCF1	protein_cc
ENSG0000(	1887.13	0.190569	0.031576	6.035162	1.59E-09	1.00E-08	B4GALT3	protein_cc
ENSG0000(	1886.943	-0.35176	0.119915	-2.93343	0.003352	0.007403	DIO2	protein_cc
ENSG0000(	1886.269	-0.03585	0.025959	-1.38108	0.167255	0.239984	RPS6KB1	protein_cc
ENSG0000(	1886.169	-0.08814	0.027084	-3.25432	0.001137	0.002742	TFIP11	protein_cc
ENSG0000(	1886.077	-0.22752	0.108261	-2.10161	0.035588	0.062551	ST6GAL2	protein_cc
ENSG0000(	1885.923	0.412921	0.052415	7.877984	3.33E-15	5.27E-14	RABL5	protein_cc
ENSG0000(	1885.765	0.516021	0.111011	4.648362	3.35E-06	1.24E-05	OSMR	protein_cc
ENSG0000(	1885.174	0.147985	0.044284	3.341763	0.000832	0.002059	KCTD13	protein_cc
ENSG0000(	1885.172	0.339901	0.037372	9.095082	9.45E-20	3.10E-18	CCDC130	protein_cc
ENSG0000(	1885.06	-0.22016	0.045907	-4.79585	1.62E-06	6.34E-06	BDP1	protein_cc
ENSG0000(	1884.792	-0.18925	0.039701	-4.76686	1.87E-06	7.25E-06	IFIT5	protein_cc
ENSG0000(	1884.547	-0.22517	0.044927	-5.01194	5.39E-07	2.27E-06	C20orf112	protein_cc
ENSG0000(	1883.036	-0.75598	0.094969	-7.96025	1.72E-15	2.85E-14	CABLES1	protein_cc
ENSG0000(	1882.589	0.032553	0.031344	1.038582	0.298999	0.39127	XRCC1	protein_cc
ENSG0000(	1882.398	-0.2824	0.069358	-4.07158	4.67E-05	0.000144	LRRC4C	protein_cc
ENSG0000(	1882.382	-0.18526	0.039969	-4.63507	3.57E-06	1.32E-05	CMAS	protein_cc
ENSG0000(	1881.65	0.252673	0.111089	2.274521	0.022935	0.042145	LZTS1	protein_cc
ENSG0000(	1881.575	-0.21856	0.064741	-3.37589	0.000736	0.001837	SLC5A3	protein_cc
ENSG0000(	1881.485	0.060782	0.044459	1.367141	0.171581	0.245259	CCDC106	protein_cc
ENSG0000(	1880.778	0.263306	0.041133	6.401364	1.54E-10	1.14E-09	NAA10	protein_cc
ENSG0000(	1880.704	-0.11918	0.037372	-3.18915	0.001427	0.003383	LATS1	protein_cc
ENSG0000(	1880.66	0.208756	0.032371	6.448929	1.13E-10	8.52E-10	TANK	protein_cc
ENSG0000(	1880.219	-0.05006	0.031833	-1.57276	0.115775	0.175354	CBFA2T2	protein_cc
ENSG0000(	1880.083	-0.21239	0.045434	-4.67475	2.94E-06	1.10E-05	TET3	protein_cc
ENSG0000(	1879.797	0.071093	0.038268	1.857751	0.063204	0.103678	WDR19	protein_cc
ENSG0000(	1879.706	0.093312	0.032078	2.908894	0.003627	0.007953	ZNF598	protein_cc
ENSG0000(	1879.404	0.027398	0.081822	0.334842	0.737744	0.801601	MAP1LC3/	protein_cc
ENSG0000(	1879.401	-0.41619	0.069876	-5.95621	2.58E-09	1.57E-08	ENPP4	protein_cc
ENSG0000(	1879.303	1.078424	0.096241	11.20547	3.83E-29	7.51E-27	PIPOX	protein_cc
ENSG0000(	1879.248	0.154014	0.047234	3.260662	0.001112	0.002685	TWF2	protein_cc
ENSG0000(	1879.213	-0.09051	0.023438	-3.86156	0.000113	0.000326	BRE	protein_cc
ENSG0000(	1879.003	-0.01082	0.036624	-0.29531	0.767756	0.825721	CC2D2A	protein_cc
ENSG0000(	1878.987	0.144933	0.029859	4.853859	1.21E-06	4.83E-06	DPP3	protein_cc
ENSG0000(	1878.936	-0.73868	0.09038	-8.17297	3.01E-16	5.65E-15	CASKIN1	protein_cc
ENSG0000(	1878.231	0.188787	0.035591	5.30439	1.13E-07	5.32E-07	TMEM126	protein_cc
ENSG0000(	1878.146	-0.06451	0.029067	-2.21937	0.026462	0.047945	SMURF1	protein_cc
ENSG0000(	1877.589	0.002533	0.043988	0.057594	0.954072	0.968186	CTTNBP2N	protein_cc
ENSG0000(	1877.223	0.242935	0.026958	9.011712	2.03E-19	6.31E-18	PRPF4	protein_cc
ENSG0000(	1876.261	-0.19759	0.032891	-6.00731	1.89E-09	1.18E-08	RYBP	protein_cc
ENSG0000(	1876.239	0.274615	0.029296	9.37389	6.99E-21	2.75E-19	NOL11	protein_cc
ENSG0000(	1874.782	-0.03817	0.036276	-1.0521	0.292755	0.384369	PARP2	protein_cc
ENSG0000(	1874.112	-0.04134	0.028155	-1.46834	0.142011	0.208659	SEH1L	protein_cc
ENSG0000(	1873.361	-0.52936	0.068086	-7.77479	7.56E-15	1.13E-13	RAB40B	protein_cc
ENSG0000(	1873.077	-0.78884	0.116141	-6.79204	1.11E-11	9.73E-11	PCSK6	protein_cc
ENSG0000(	1871.689	0.053707	0.047197	1.137942	0.255145	0.342738	TP53INP1	protein_cc
ENSG0000(	1871.051	-0.3193	0.058494	-5.45866	4.80E-08	2.39E-07	PKNOX2	protein_cc
ENSG0000(	1870.764	-0.07615	0.103607	-0.735	0.462341	0.55846	OLR1	protein_cc
ENSG0000(	1870.532	-0.23613	0.049089	-4.81036	1.51E-06	5.92E-06	TMEM136	protein_cc
ENSG0000(	1870.365	0.080944	0.033684	2.403065	0.016258	0.030961	MBD2	protein_cc

ENSG0000(	1869.798	0.195396	0.039109	4.996238	5.85E-07	2.45E-06	TSTA3	protein_cc
ENSG0000(	1869.057	0.082886	0.025496	3.250961	0.00115	0.00277	ZNF746	protein_cc
ENSG0000(	1868.6	0.16377	0.051365	3.188353	0.001431	0.003391	PSENEN	protein_cc
ENSG0000(	1868.101	0.581239	0.097255	5.97641	2.28E-09	1.41E-08	S100A13	protein_cc
ENSG0000(	1867.752	-0.06982	0.037055	-1.88436	0.059516	0.098353	GPATCH4	protein_cc
ENSG0000(	1867.385	-0.40606	0.064657	-6.28023	3.38E-10	2.37E-09	SYNDIG1	protein_cc
ENSG0000(	1867.097	0.023754	0.040619	0.58481	0.558676	0.647766	ADSS	protein_cc
ENSG0000(	1866.696	0.033694	0.030069	1.120528	0.262489	0.351078	DAK	protein_cc
ENSG0000(	1866.31	-0.3192	0.10205	-3.12791	0.001761	0.004105	ELAVL4	protein_cc
ENSG0000(	1865.742	-0.07032	0.028473	-2.46987	0.013516	0.026213	C2orf49	protein_cc
ENSG0000(	1865.241	0.195893	0.02495	7.851333	4.12E-15	6.42E-14	UTP6	protein_cc
ENSG0000(	1864.765	0.05944	0.035013	1.697646	0.089575	0.140456	ZFX	protein_cc
ENSG0000(	1864.257	0.019615	0.024199	0.810571	0.417612	0.514588	FBXO38	protein_cc
ENSG0000(	1864.196	0.071327	0.023877	2.987338	0.002814	0.006302	RPRD1B	protein_cc
ENSG0000(	1864.184	0.217032	0.036826	5.893473	3.78E-09	2.25E-08	JMJD8	protein_cc
ENSG0000(	1864.151	-0.20237	0.027852	-7.26586	3.71E-13	4.11E-12	SLMAP	protein_cc
ENSG0000(	1864.022	0.208429	0.03721	5.601377	2.13E-08	1.12E-07	BPGM	protein_cc
ENSG0000(	1863.986	-0.25006	0.05306	-4.7128	2.44E-06	9.29E-06	LRRC20	protein_cc
ENSG0000(	1863.725	-0.25036	0.049261	-5.08232	3.73E-07	1.61E-06	ERMP1	protein_cc
ENSG0000(	1862.875	0.43526	0.113817	3.824201	0.000131	0.000375	RDH10	protein_cc
ENSG0000(	1861.901	-0.23435	0.04801	-4.88124	1.05E-06	4.24E-06	FBXL19	protein_cc
ENSG0000(	1861.843	-0.27246	0.073646	-3.69967	0.000216	0.000595	CDH10	protein_cc
ENSG0000(	1861.77	-0.16648	0.062312	-2.67174	0.007546	0.015499	SETBP1	protein_cc
ENSG0000(	1861.693	-0.06066	0.048297	-1.25594	0.209139	0.290023	PIK3CA	protein_cc
ENSG0000(	1860.548	-0.18189	0.030836	-5.89851	3.67E-09	2.19E-08	PIK3R4	protein_cc
ENSG0000(	1859.984	0.088803	0.124815	0.711477	0.476789	0.572464	SLC11A1	protein_cc
ENSG0000(	1859.869	-0.11724	0.024069	-4.87113	1.11E-06	4.45E-06	VPS37A	protein_cc
ENSG0000(	1859.622	0.160797	0.034833	4.616213	3.91E-06	1.44E-05	RBBP9	protein_cc
ENSG0000(	1859.427	0.137503	0.041833	3.286917	0.001013	0.002465	HSD17B11	protein_cc
ENSG0000(	1858.99	0.119785	0.068793	1.741239	0.081642	0.129436	CD34	protein_cc
ENSG0000(	1858.937	-0.21087	0.046013	-4.58289	4.59E-06	1.67E-05	ZDHHC21	protein_cc
ENSG0000(	1858.021	-0.37567	0.103556	-3.62771	0.000286	0.000772	LINC0084	lincRNA
ENSG0000(	1857.998	-0.0751	0.108708	-0.69085	0.489658	0.584164	BTBD17	protein_cc
ENSG0000(	1857.919	0.205216	0.047835	4.290059	1.79E-05	5.93E-05	ISOC2	protein_cc
ENSG0000(	1857.765	0.144548	0.025081	5.763317	8.25E-09	4.63E-08	MFN1	protein_cc
ENSG0000(	1857.645	-0.269	0.072658	-3.70232	0.000214	0.000589	SEMA7A	protein_cc
ENSG0000(	1857.639	-0.07814	0.032051	-2.43813	0.014764	0.028395	ZNF623	protein_cc
ENSG0000(	1856.622	-0.28674	0.044108	-6.50095	7.98E-11	6.17E-10	JARID2	protein_cc
ENSG0000(	1856.249	-0.18866	0.05123	-3.68261	0.000231	0.000633	IL17RA	protein_cc
ENSG0000(	1856.159	-0.58031	0.127378	-4.55582	5.22E-06	1.88E-05	ABCC8	protein_cc
ENSG0000(	1856.056	0.146171	0.037449	3.903168	9.49E-05	0.000279	NDUFB3	protein_cc
ENSG0000(	1855.554	0.181825	0.034076	5.33583	9.51E-08	4.52E-07	SIKE1	protein_cc
ENSG0000(	1854.896	0.286167	0.04266	6.708114	1.97E-11	1.67E-10	C14orf119	protein_cc
ENSG0000(	1854.697	0.119695	0.035327	3.388156	0.000704	0.001764	TIMM17B	protein_cc
ENSG0000(	1853.547	-0.0654	0.036378	-1.79785	0.072201	0.116429	CC2D1B	protein_cc
ENSG0000(	1853.3	0.21091	0.071274	2.959132	0.003085	0.006859	SWAP70	protein_cc
ENSG0000(	1853.151	0.101321	0.026423	3.834596	0.000126	0.000361	MTG2	protein_cc
ENSG0000(	1852.961	0.174323	0.038263	4.55588	5.22E-06	1.88E-05	NOP2	protein_cc
ENSG0000(	1852.89	0.098668	0.039874	2.474507	0.013342	0.025909	OSBPL6	protein_cc
ENSG0000(	1852.032	-0.24166	0.047039	-5.13743	2.79E-07	1.23E-06	C10orf32	protein_cc
ENSG0000(	1851.951	-0.10848	0.149702	-0.72465	0.468668	0.564543	NPTX2	protein_cc
ENSG0000(	1851.174	0.026234	0.028636	0.916107	0.359611	0.455427	ISG20L2	protein_cc
ENSG0000(	1851.101	-0.05714	0.033659	-1.69762	0.08958	0.140456	IFT27	protein_cc
ENSG0000(	1851.071	-0.53512	0.070087	-7.63497	2.26E-14	3.06E-13	KIFC2	protein_cc
ENSG0000(	1850.521	0.036374	0.030953	1.175145	0.239937	0.325262	SUDS3	protein_cc
ENSG0000(	1850.276	0.144754	0.049148	2.945245	0.003227	0.007146	IRF3	protein_cc
ENSG0000(	1850.022	-0.03588	0.033924	-1.05773	0.290178	0.381572	ANKRD13I	protein_cc
ENSG0000(	1849.94	-0.27704	0.056178	-4.93151	8.16E-07	3.34E-06	CASP9	protein_cc
ENSG0000(	1849.822	-0.19465	0.031262	-6.22627	4.78E-10	3.27E-09	PISD	protein_cc

ENSG0000(	1849.369	-0.47599	0.050432	-9.43825	3.79E-21	1.57E-19	SGTB	protein_cc
ENSG0000(	1848.955	0.154941	0.023932	6.474336	9.52E-11	7.28E-10	C17orf80	protein_cc
ENSG0000(	1848.814	0.195028	0.042731	4.564036	5.02E-06	1.82E-05	NOTCH4	protein_cc
ENSG0000(	1848.595	-0.11266	0.027714	-4.06506	4.80E-05	0.000148	FNIP1	protein_cc
ENSG0000(	1848.191	-0.06293	0.021815	-2.88457	0.003919	0.008542	TERF1	protein_cc
ENSG0000(	1847.897	0.131671	0.066826	1.970372	0.048796	0.082692	CMBL	protein_cc
ENSG0000(	1847.745	0.387391	0.060416	6.412062	1.44E-10	1.07E-09	UGCG	protein_cc
ENSG0000(	1847.705	0.408607	0.120704	3.385187	0.000711	0.001782	ALOX5AP	protein_cc
ENSG0000(	1846.729	0.025818	0.029102	0.887174	0.374985	0.471558	NSMAF	protein_cc
ENSG0000(	1846.378	-0.18508	0.050097	-3.6944	0.00022	0.000606	SNX9	protein_cc
ENSG0000(	1845.616	0.285259	0.055203	5.167489	2.37E-07	1.06E-06	BTN3A1	protein_cc
ENSG0000(	1844.978	-0.48779	0.062991	-7.74386	9.64E-15	1.41E-13	STOX2	protein_cc
ENSG0000(	1844.565	-0.06374	0.045962	-1.38688	0.165478	0.237891	ABHD17C	protein_cc
ENSG0000(	1844.373	-0.18191	0.028222	-6.44552	1.15E-10	8.71E-10	UCHL5	protein_cc
ENSG0000(	1844.26	0.032971	0.117692	0.280148	0.779364	0.835368	CHRDL1	protein_cc
ENSG0000(	1843.698	-0.00252	0.034353	-0.07328	0.941583	0.959241	AP3S1	protein_cc
ENSG0000(	1842.735	-0.01672	0.043885	-0.38092	0.703263	0.773371	SCRN2	protein_cc
ENSG0000(	1842.638	0.4291	0.109538	3.917369	8.95E-05	0.000264	TUBB6	protein_cc
ENSG0000(	1842.638	-0.05812	0.038088	-1.52588	0.12704	0.189776	FGD1	protein_cc
ENSG0000(	1842.435	-0.04528	0.037099	-1.2205	0.222274	0.305184	EEF2K	protein_cc
ENSG0000(	1842.216	0.060853	0.025547	2.381978	0.01722	0.032544	TDP2	protein_cc
ENSG0000(	1841.925	-0.04986	0.032816	-1.51948	0.128643	0.19172	ZNF252P	pseudoge
ENSG0000(	1840.789	0.199032	0.03798	5.240433	1.60E-07	7.34E-07	STX8	protein_cc
ENSG0000(	1840.628	0.055648	0.030557	1.821111	0.06859	0.111305	TMEM104	protein_cc
ENSG0000(	1840.496	-0.05646	0.034154	-1.6531	0.09831	0.15224	SPHK2	protein_cc
ENSG0000(	1840.435	0.070679	0.025266	2.797365	0.005152	0.010954	OGG1	protein_cc
ENSG0000(	1839.831	0.059181	0.035241	1.679321	0.09309	0.14519	QSOX2	protein_cc
ENSG0000(	1838.739	-0.12593	0.032997	-3.81635	0.000135	0.000387	MTX3	protein_cc
ENSG0000(	1838.499	-0.33569	0.042784	-7.84633	4.28E-15	6.66E-14	MROH1	protein_cc
ENSG0000(	1838.381	-0.19622	0.039662	-4.94738	7.52E-07	3.10E-06	AGO4	protein_cc
ENSG0000(	1837.274	0.090694	0.030969	2.928521	0.003406	0.007509	RBM4B	protein_cc
ENSG0000(	1837.144	0.000619	0.035987	0.017188	0.986287	0.990647	ZBTB17	protein_cc
ENSG0000(	1836.483	0.06015	0.022721	2.647389	0.008112	0.016548	GTF2H3	protein_cc
ENSG0000(	1836.452	-0.19822	0.044108	-4.49385	6.99E-06	2.48E-05	ARID4A	protein_cc
ENSG0000(	1836.258	-0.27195	0.040369	-6.73656	1.62E-11	1.40E-10	CREBRF	protein_cc
ENSG0000(	1835.353	0.037785	0.032615	1.158503	0.246659	0.333082	HS1BP3	protein_cc
ENSG0000(	1835.237	-0.97991	0.073993	-13.2433	4.93E-40	6.76E-37	ARRB1	protein_cc
ENSG0000(	1835.237	0.017608	0.035071	0.502087	0.615606	0.698744	DCK	protein_cc
ENSG0000(	1835.171	0.322621	0.039251	8.219356	2.05E-16	3.95E-15	ZNF266	protein_cc
ENSG0000(	1833.852	0.389744	0.127651	3.053189	0.002264	0.005173	MSR1	protein_cc
ENSG0000(	1833.617	-0.28557	0.043938	-6.49952	8.06E-11	6.23E-10	TBC1D2B	protein_cc
ENSG0000(	1832.547	0.046245	0.030587	1.511895	0.130561	0.194085	ACSS2	protein_cc
ENSG0000(	1832.491	-0.02725	0.030536	-0.89226	0.372253	0.468667	TUBGCP3	protein_cc
ENSG0000(	1832.476	-0.21002	0.034147	-6.15046	7.73E-10	5.13E-09	EPG5	protein_cc
ENSG0000(	1832.338	-0.05834	0.031966	-1.82494	0.06801	0.11046	NFRKB	protein_cc
ENSG0000(	1832.194	0.010527	0.096577	0.109006	0.913197	0.938835	MEGF10	protein_cc
ENSG0000(	1831.465	-0.17537	0.044425	-3.94748	7.90E-05	0.000235	HMBOX1	protein_cc
ENSG0000(	1831.387	-0.20217	0.037267	-5.42484	5.80E-08	2.85E-07	BRAF	protein_cc
ENSG0000(	1831.015	0.011585	0.036622	0.316327	0.751754	0.813043	TMEM198	pseudoge
ENSG0000(	1831.003	-0.31223	0.120058	-2.6007	0.009303	0.018714	SCIN	protein_cc
ENSG0000(	1830.874	-0.10681	0.039085	-2.7328	0.00628	0.013104	GNE	protein_cc
ENSG0000(	1830.821	0.084827	0.038671	2.193556	0.028267	0.050838	DPH3	protein_cc
ENSG0000(	1830.754	0.145514	0.033344	4.364062	1.28E-05	4.33E-05	SLC37A3	protein_cc
ENSG0000(	1829.737	-0.06068	0.044463	-1.36467	0.172357	0.246171	IMPA1	protein_cc
ENSG0000(	1829.441	0.012255	0.028833	0.425022	0.670821	0.745892	PIGO	protein_cc
ENSG0000(	1828.549	0.183091	0.039279	4.661293	3.14E-06	1.17E-05	ZNF260	protein_cc
ENSG0000(	1827.524	-0.21211	0.031159	-6.80726	9.95E-12	8.84E-11	CUL2	protein_cc
ENSG0000(	1827.091	0.108423	0.034221	3.168362	0.001533	0.003615	PFDN6	protein_cc
ENSG0000(	1826.519	-0.22572	0.077354	-2.91797	0.003523	0.007746	MPP6	protein_cc

ENSG0000(	1826.222	-0.3963	0.040987	-9.66911	4.08E-22	2.01E-20	SUFU	protein_cc
ENSG0000(	1826.134	-0.06654	0.042459	-1.56723	0.117061	0.176992	USP3	protein_cc
ENSG0000(	1825.406	-0.13918	0.040029	-3.47713	0.000507	0.001306	PHC3	protein_cc
ENSG0000(	1825.168	0.14796	0.058844	2.514433	0.011922	0.023408	ROBO4	protein_cc
ENSG0000(	1824.984	0.363878	0.043162	8.430499	3.44E-17	7.55E-16	PRELID1	protein_cc
ENSG0000(	1824.591	0.075125	0.044971	1.670536	0.094813	0.14752	DRAM2	protein_cc
ENSG0000(	1824.133	1.405423	0.183921	7.641435	2.15E-14	2.93E-13	MOXD1	protein_cc
ENSG0000(	1823.848	0.087967	0.073052	1.204171	0.228524	0.312344	DCBLD2	protein_cc
ENSG0000(	1823.137	0.24135	0.056198	4.294659	1.75E-05	5.82E-05	MOV10	protein_cc
ENSG0000(	1822.375	0.329937	0.036463	9.048465	1.45E-19	4.63E-18	POT1	protein_cc
ENSG0000(	1822.246	0.306765	0.041347	7.419276	1.18E-13	1.42E-12	MVB12A	protein_cc
ENSG0000(	1821.525	-0.02073	0.030988	-0.66907	0.503452	0.597293	RBM19	protein_cc
ENSG0000(	1821.448	-0.17527	0.031329	-5.59442	2.21E-08	1.16E-07	FAM204A	protein_cc
ENSG0000(	1821.438	-0.18908	0.142367	-1.32815	0.184129	0.260175	SUSD5	protein_cc
ENSG0000(	1821.258	0.195658	0.025948	7.540474	4.68E-14	6.02E-13	RRP36	protein_cc
ENSG0000(	1820.736	-0.11235	0.036423	-3.08461	0.002038	0.004697	PET112	protein_cc
ENSG0000(	1820.555	0.139326	0.026068	5.344705	9.06E-08	4.32E-07	MRPS11	protein_cc
ENSG0000(	1820.549	-0.09839	0.039419	-2.49605	0.012559	0.024516	UTP14C	protein_cc
ENSG0000(	1820.433	0.166252	0.052737	3.152495	0.001619	0.003801	SLC29A1	protein_cc
ENSG0000(	1820.244	-0.66529	0.072851	-9.13209	6.72E-20	2.26E-18	PLEKHA5	protein_cc
ENSG0000(	1820.067	-0.19756	0.059434	-3.32408	0.000887	0.002183	C15orf52	protein_cc
ENSG0000(	1818.934	0.09839	0.035917	2.7394	0.006155	0.012865	THAP7	protein_cc
ENSG0000(	1818.055	-0.21639	0.034608	-6.25267	4.03E-10	2.79E-09	DENND4A	protein_cc
ENSG0000(	1816.978	0.214183	0.057996	3.69307	0.000222	0.000609	VASP	protein_cc
ENSG0000(	1816.687	0.143621	0.04165	3.448247	0.000564	0.001442	CCDC94	protein_cc
ENSG0000(	1816.388	0.289249	0.047939	6.033711	1.60E-09	1.01E-08	SMC2	protein_cc
ENSG0000(	1815.532	-0.12535	0.040046	-3.1302	0.001747	0.004076	ADCY3	protein_cc
ENSG0000(	1814.015	-0.20589	0.038741	-5.31456	1.07E-07	5.05E-07	NDEL1	protein_cc
ENSG0000(	1814.013	0.017994	0.027977	0.643154	0.520124	0.612496	GABPA	protein_cc
ENSG0000(	1813.72	-0.78793	0.06327	-12.4535	1.34E-35	9.97E-33	ADRBK2	protein_cc
ENSG0000(	1813.702	0.121697	0.023183	5.249371	1.53E-07	7.02E-07	HTRA2	protein_cc
ENSG0000(	1813.221	0.150869	0.037845	3.986436	6.71E-05	0.000202	MRPL40	protein_cc
ENSG0000(	1812.869	-0.01773	0.0343	-0.517	0.605158	0.689358	PDE6D	protein_cc
ENSG0000(	1812.81	0.003495	0.089275	0.039145	0.968775	0.978386	HAVCR2	protein_cc
ENSG0000(	1811.877	0.237206	0.02697	8.795087	1.43E-18	3.89E-17	EMC8	protein_cc
ENSG0000(	1811.406	0.075778	0.037985	1.994933	0.04605	0.078563	RP11-349.	antisense
ENSG0000(	1810.397	0.248339	0.03648	6.807577	9.93E-12	8.82E-11	MLX	protein_cc
ENSG0000(	1810.154	-0.06735	0.022868	-2.9451	0.003228	0.007148	PDE12	protein_cc
ENSG0000(	1810.039	0.252224	0.028636	8.808034	1.27E-18	3.49E-17	TPRA1	protein_cc
ENSG0000(	1809.733	0.164312	0.023281	7.057717	1.69E-12	1.70E-11	MRPL9	protein_cc
ENSG0000(	1809.562	-0.03071	0.027299	-1.12497	0.260603	0.348897	USP15	protein_cc
ENSG0000(	1809.499	0.038321	0.024447	1.567523	0.116992	0.176908	POLR3E	protein_cc
ENSG0000(	1809.372	0.00445	0.034405	0.129352	0.897079	0.927061	SDCCAG8	protein_cc
ENSG0000(	1809.034	-0.07044	0.055518	-1.26882	0.204505	0.284587	TARBP1	protein_cc
ENSG0000(	1808.92	-0.28447	0.059287	-4.79819	1.60E-06	6.27E-06	CD200	protein_cc
ENSG0000(	1808.111	-0.20898	0.037007	-5.64705	1.63E-08	8.76E-08	SMCR8	protein_cc
ENSG0000(	1807.345	-0.05092	0.034427	-1.47905	0.139126	0.20504	ESRRA	protein_cc
ENSG0000(	1806.024	0.840325	0.087468	9.60725	7.45E-22	3.49E-20	TNFRSF19	protein_cc
ENSG0000(	1804.631	-0.04397	0.142968	-0.30758	0.758399	0.818357	CP	protein_cc
ENSG0000(	1804.458	-0.32168	0.084019	-3.82867	0.000129	0.000369	EVI2A	protein_cc
ENSG0000(	1804.327	-0.24981	0.143912	-1.73586	0.082589	0.130746	PCSK2	protein_cc
ENSG0000(	1804.197	-0.68961	0.092134	-7.4848	7.17E-14	8.97E-13	DGKB	protein_cc
ENSG0000(	1803.834	-0.5478	0.057706	-9.49306	2.24E-21	9.73E-20	TBC1D9	protein_cc
ENSG0000(	1803.686	-0.23321	0.031889	-7.31302	2.61E-13	2.97E-12	FAM21A	protein_cc
ENSG0000(	1803.209	-0.07554	0.056701	-1.33227	0.182771	0.25854	SH2B3	protein_cc
ENSG0000(	1802.918	0.081262	0.042392	1.916946	0.055245	0.09221	PARP3	protein_cc
ENSG0000(	1802.871	0.22801	0.032685	6.975899	3.04E-12	2.93E-11	RABEPK	protein_cc
ENSG0000(	1801.254	-0.00965	0.022664	-0.42576	0.670282	0.745491	GRPEL1	protein_cc
ENSG0000(	1800.623	0.086531	0.032786	2.639248	0.008309	0.016911	DHDDS	protein_cc

ENSG0000	1800.458	0.121028	0.026599	4.550126	5.36E-06	1.93E-05	GABPB1	protein_co
ENSG0000	1800.056	0.16936	0.034264	4.942799	7.70E-07	3.17E-06	ANKMY2	protein_co
ENSG0000	1799.625	-0.00266	0.026963	-0.0985	0.921538	0.944717	ACTR6	protein_co
ENSG0000	1798.972	0.133815	0.035547	3.764513	0.000167	0.000469	GLRX3	protein_co
ENSG0000	1798.684	-0.33173	0.067889	-4.88633	1.03E-06	4.14E-06	AMIGO1	protein_co
ENSG0000	1798.444	0.096423	0.044624	2.160809	0.03071	0.054779	SIPA1L3	protein_co
ENSG0000	1798.409	0.168995	0.025423	6.647424	2.98E-11	2.46E-10	ZNF263	protein_co
ENSG0000	1796.678	-0.01449	0.030679	-0.47243	0.63662	0.717015	CAPN7	protein_co
ENSG0000	1796.196	-0.02992	0.022515	-1.32889	0.183884	0.259899	DHX29	protein_co
ENSG0000	1795.666	-0.11279	0.035334	-3.19202	0.001413	0.003352	NEK4	protein_co
ENSG0000	1794.951	-0.11468	0.071234	-1.60998	0.107402	0.164292	NPL	protein_co
ENSG0000	1794.485	-0.36929	0.039834	-9.2707	1.85E-20	6.77E-19	POLR3A	protein_co
ENSG0000	1794.072	-0.5082	0.140082	-3.62788	0.000286	0.000772	CSMD3	protein_co
ENSG0000	1793.575	-0.09841	0.036117	-2.72466	0.006437	0.013403	REXO1	protein_co
ENSG0000	1790.747	0.477695	0.048504	9.848613	6.95E-23	4.00E-21	ACTL6A	protein_co
ENSG0000	1789.409	0.128076	0.027228	4.703847	2.55E-06	9.67E-06	MRPL16	protein_co
ENSG0000	1789.257	-0.52795	0.108265	-4.87647	1.08E-06	4.34E-06	RP11-731.	sense_ove
ENSG0000	1788.355	0.382259	0.038273	9.987591	1.73E-23	1.13E-21	POLR2H	protein_co
ENSG0000	1788.336	-0.02797	0.043917	-0.63697	0.524144	0.616099	STK10	protein_co
ENSG0000	1788.319	0.371984	0.03556	10.46061	1.31E-25	1.30E-23	TMEM237	protein_co
ENSG0000	1788.094	-0.29635	0.11786	-2.51441	0.011923	0.023408	TMEM179	protein_co
ENSG0000	1787.991	0.280416	0.043588	6.433261	1.25E-10	9.40E-10	TUBG1	protein_co
ENSG0000	1787.833	-0.41162	0.076366	-5.39008	7.04E-08	3.42E-07	AFF3	protein_co
ENSG0000	1787.149	-0.07373	0.030692	-2.40228	0.016293	0.031022	STK35	protein_co
ENSG0000	1787.091	0.28164	0.053374	5.276711	1.32E-07	6.12E-07	RPL17	protein_co
ENSG0000	1787.043	-0.18383	0.034034	-5.40132	6.62E-08	3.23E-07	NAA30	protein_co
ENSG0000	1786.506	-0.14796	0.03491	-4.23842	2.25E-05	7.33E-05	RRP12	protein_co
ENSG0000	1786.475	0.203141	0.065059	3.12244	0.001794	0.004177	TRIM14	protein_co
ENSG0000	1785.973	0.914006	0.104732	8.727115	2.61E-18	6.84E-17	SMIM3	protein_co
ENSG0000	1784.665	0.10144	0.044859	2.261299	0.023741	0.043459	LYPLA2	protein_co
ENSG0000	1784.417	-0.17638	0.06388	-2.76117	0.005759	0.012112	TMEM178	protein_co
ENSG0000	1784.204	-0.05007	0.045382	-1.10328	0.269904	0.359302	BCKDHB	protein_co
ENSG0000	1784.177	0.043084	0.025105	1.716125	0.086139	0.135718	MPHOSPH	protein_co
ENSG0000	1783.91	-0.66631	0.100097	-6.65664	2.80E-11	2.33E-10	GOLGA7B	protein_co
ENSG0000	1783.433	-0.2442	0.033223	-7.35042	1.98E-13	2.28E-12	FBXL20	protein_co
ENSG0000	1782.708	0.09912	0.02361	4.198239	2.69E-05	8.65E-05	ZNF131	protein_co
ENSG0000	1782.468	0.189603	0.028683	6.610299	3.84E-11	3.12E-10	NUP43	protein_co
ENSG0000	1782.064	0.307668	0.045911	6.701454	2.06E-11	1.74E-10	LAMTOR2	protein_co
ENSG0000	1781.957	-0.57952	0.10527	-5.50503	3.69E-08	1.88E-07	PAK3	protein_co
ENSG0000	1781.936	0.057681	0.087612	0.658364	0.510304	0.603524	IFITM2	protein_co
ENSG0000	1781.202	-0.00857	0.036236	-0.23641	0.813115	0.86273	C19orf12	protein_co
ENSG0000	1781.094	0.701809	0.052208	13.44244	3.41E-41	5.01E-38	RFC2	protein_co
ENSG0000	1780.967	0.297556	0.032417	9.178994	4.35E-20	1.51E-18	SLC30A5	protein_co
ENSG0000	1780.672	-0.33861	0.082459	-4.10639	4.02E-05	0.000126	TUSC3	protein_co
ENSG0000	1780.571	-0.14563	0.090421	-1.61062	0.107262	0.164126	SLA	protein_co
ENSG0000	1780.568	0.177733	0.050485	3.52052	0.000431	0.001126	LRFN4	protein_co
ENSG0000	1779.73	-0.17388	0.093122	-1.86728	0.061863	0.101709	ASPHD1	protein_co
ENSG0000	1779.518	-0.23768	0.092407	-2.57204	0.01011	0.020169	MS4A7	protein_co
ENSG0000	1778.945	0.167506	0.060873	2.751728	0.005928	0.012432	GBE1	protein_co
ENSG0000	1778.796	-0.17778	0.0511	-3.47916	0.000503	0.001297	SRD5A1	protein_co
ENSG0000	1778.775	-0.57888	0.080405	-7.19956	6.04E-13	6.49E-12	RAB15	protein_co
ENSG0000	1778.723	0.226024	0.068723	3.288925	0.001006	0.002449	TNFRSF10	protein_co
ENSG0000	1778.088	0.207166	0.0421	4.92078	8.62E-07	3.52E-06	SMC5	protein_co
ENSG0000	1778.007	-0.01034	0.035409	-0.292	0.77029	0.827908	SRPK1	protein_co
ENSG0000	1777.902	-0.14029	0.032393	-4.33107	1.48E-05	4.98E-05	NFS1	protein_co
ENSG0000	1777.661	-0.50872	0.097108	-5.2387	1.62E-07	7.41E-07	SYNJ2	protein_co
ENSG0000	1777.043	-1.03092	0.134633	-7.65728	1.90E-14	2.62E-13	CELF4	protein_co
ENSG0000	1776.652	-0.40083	0.063431	-6.3192	2.63E-10	1.88E-09	PDE4A	protein_co
ENSG0000	1776.091	0.094583	0.048049	1.968483	0.049013	0.083021	DNPH1	protein_co

ENSG0000(	1775.25	-0.0272	0.023883	-1.13887	0.254756	0.342348	BRAP	protein_cc
ENSG0000(	1775.175	-0.07902	0.041275	-1.91446	0.055561	0.09266	FBXO30	protein_cc
ENSG0000(	1774.266	-1.52318	0.16038	-9.49733	2.15E-21	9.40E-20	CREG2	protein_cc
ENSG0000(	1773.684	-0.05912	0.028936	-2.04304	0.041048	0.070993	GFOD2	protein_cc
ENSG0000(	1773.406	-0.08657	0.038864	-2.22762	0.025906	0.047031	BACH1	protein_cc
ENSG0000(	1773.325	0.03931	0.081465	0.482531	0.629429	0.710862	THSD7A	protein_cc
ENSG0000(	1773.125	-0.24159	0.046559	-5.18886	2.12E-07	9.52E-07	AGO3	protein_cc
ENSG0000(	1772.985	0.09826	0.048401	2.03012	0.042344	0.072952	MMAB	protein_cc
ENSG0000(	1772.559	0.060519	0.028991	2.087485	0.036844	0.064509	NAA60	protein_cc
ENSG0000(	1772.503	-0.1071	0.043328	-2.47174	0.013446	0.02609	ELF1	protein_cc
ENSG0000(	1772.466	0.277818	0.031123	8.926382	4.40E-19	1.30E-17	PIGU	protein_cc
ENSG0000(	1772.233	0.101327	0.031941	3.172372	0.001512	0.003569	NGDN	protein_cc
ENSG0000(	1771.135	0.020457	0.027777	0.73647	0.461445	0.557622	LCMT1	protein_cc
ENSG0000(	1770.948	-0.48554	0.053156	-9.1342	6.59E-20	2.22E-18	LUZP1	protein_cc
ENSG0000(	1770.835	-0.1309	0.039417	-3.32085	0.000897	0.002206	SS18L1	protein_cc
ENSG0000(	1769.844	-0.14068	0.032166	-4.37349	1.22E-05	4.16E-05	CLUAP1	protein_cc
ENSG0000(	1769.548	0.264664	0.061343	4.314471	1.60E-05	5.34E-05	SBNO2	protein_cc
ENSG0000(	1769.093	-0.31918	0.047031	-6.78665	1.15E-11	1.01E-10	ATE1	protein_cc
ENSG0000(	1768.355	0.167149	0.045173	3.700188	0.000215	0.000594	RAVER1	protein_cc
ENSG0000(	1768.346	0.079656	0.019074	4.176247	2.96E-05	9.46E-05	RNF34	protein_cc
ENSG0000(	1768.218	-1.1301	0.110355	-10.2406	1.30E-24	1.06E-22	STX1A	protein_cc
ENSG0000(	1767.784	-0.19785	0.085123	-2.32432	0.020109	0.037408	APBB1IP	protein_cc
ENSG0000(	1766.792	-0.03921	0.018721	-2.09421	0.036241	0.06358	SCO1	protein_cc
ENSG0000(	1766.231	0.540647	0.059148	9.140613	6.21E-20	2.10E-18	CKAP2	protein_cc
ENSG0000(	1766.068	-0.11609	0.030275	-3.83454	0.000126	0.000361	FBXO45	protein_cc
ENSG0000(	1765.788	-0.07572	0.029895	-2.53287	0.011313	0.022331	TBCK	protein_cc
ENSG0000(	1765.575	-0.10177	0.042606	-2.38856	0.016914	0.032052	C5orf42	protein_cc
ENSG0000(	1765.271	-0.41962	0.077095	-5.44288	5.24E-08	2.59E-07	CSRNP3	protein_cc
ENSG0000(	1765.058	0.083111	0.040727	2.04068	0.041283	0.071341	EML4	protein_cc
ENSG0000(	1764.872	-0.00307	0.028307	-0.10854	0.913569	0.939029	KRR1	protein_cc
ENSG0000(	1764.828	-0.61278	0.096268	-6.36537	1.95E-10	1.42E-09	TMEM246	protein_cc
ENSG0000(	1764.506	-0.10649	0.049979	-2.13072	0.033112	0.058613	ANKRD6	protein_cc
ENSG0000(	1764.341	-0.34211	0.057221	-5.97881	2.25E-09	1.39E-08	ARMCX4	protein_cc
ENSG0000(	1764.027	0.19674	0.040582	4.84798	1.25E-06	4.97E-06	PRMT3	protein_cc
ENSG0000(	1763.71	-0.91253	0.103868	-8.78544	1.56E-18	4.21E-17	ABI3BP	protein_cc
ENSG0000(	1763.436	0.051012	0.02362	2.159699	0.030796	0.05491	AMMECR1	protein_cc
ENSG0000(	1762.056	0.079975	0.033149	2.412612	0.015839	0.030233	NIPA2	protein_cc
ENSG0000(	1761.659	0.000128	0.041201	0.003107	0.997521	0.998444	ICA1L	protein_cc
ENSG0000(	1761.558	0.423279	0.047176	8.972285	2.90E-19	8.81E-18	CASP2	protein_cc
ENSG0000(	1760.969	0.026246	0.03693	0.710698	0.477271	0.572809	RECQL5	protein_cc
ENSG0000(	1760.92	-0.28975	0.054714	-5.29568	1.19E-07	5.56E-07	BCL2	protein_cc
ENSG0000(	1760.575	-0.04904	0.046328	-1.05854	0.289809	0.381208	STAG1	protein_cc
ENSG0000(	1759.992	-0.08613	0.04589	-1.87688	0.060535	0.099816	CTDSPL	protein_cc
ENSG0000(	1759.938	-0.14673	0.030873	-4.75286	2.01E-06	7.73E-06	TSC22D2	protein_cc
ENSG0000(	1759.575	0.222663	0.057	3.906378	9.37E-05	0.000275	PNP	protein_cc
ENSG0000(	1759.285	-0.27797	0.045114	-6.16148	7.21E-10	4.80E-09	TCF7L2	protein_cc
ENSG0000(	1758.542	-0.71408	0.134805	-5.29713	1.18E-07	5.52E-07	MAL	protein_cc
ENSG0000(	1758.33	0.775393	0.081595	9.503007	2.04E-21	8.96E-20	LMNB1	protein_cc
ENSG0000(	1758.185	-0.21634	0.033054	-6.54491	5.95E-11	4.69E-10	PPTC7	protein_cc
ENSG0000(	1758.114	0.130992	0.024899	5.261014	1.43E-07	6.61E-07	FTSJ1	protein_cc
ENSG0000(	1758.083	-0.0188	0.028067	-0.66965	0.503079	0.596953	COG8	protein_cc
ENSG0000(	1757.553	-0.10037	0.05297	-1.89485	0.058113	0.096368	CPEB2	protein_cc
ENSG0000(	1756.963	0.180208	0.043567	4.136334	3.53E-05	0.000111	POLR1D	protein_cc
ENSG0000(	1756.79	0.011377	0.032487	0.350207	0.726184	0.791767	ORC3	protein_cc
ENSG0000(	1756.542	-0.09386	0.036963	-2.53933	0.011107	0.021964	LTN1	protein_cc
ENSG0000(	1756.541	-0.22471	0.045586	-4.92936	8.25E-07	3.38E-06	TPST2	protein_cc
ENSG0000(	1756.235	-0.61993	0.065186	-9.5102	1.90E-21	8.41E-20	ATL1	protein_cc
ENSG0000(	1755.315	-0.01991	0.05051	-0.39419	0.693439	0.765125	MAPK12	protein_cc
ENSG0000(	1754.964	-0.27258	0.138115	-1.97357	0.048431	0.082114	PI16	protein_cc

ENSG0000(	1754.858	0.022977	0.032126	0.715219	0.474474	0.570317	RP11-785	pseudoge
ENSG0000(	1754.627	0.085803	0.03649	2.351372	0.018704	0.03505	IRF2	protein_cc
ENSG0000(	1754.454	0.028	0.02522	1.110216	0.266906	0.355909	REV1	protein_cc
ENSG0000(	1754.269	0.16795	0.032806	5.119515	3.06E-07	1.34E-06	UBTD2	protein_cc
ENSG0000(	1753.834	-0.03756	0.03131	-1.19963	0.230282	0.314256	CCDC132	protein_cc
ENSG0000(	1753.536	-0.44633	0.151844	-2.93944	0.003288	0.007271	THBS1	protein_cc
ENSG0000(	1753.426	0.06078	0.030458	1.995529	0.045985	0.078465	RRNAD1	protein_cc
ENSG0000(	1753.335	-0.28689	0.071418	-4.01713	5.89E-05	0.000179	CGNL1	protein_cc
ENSG0000(	1752.634	0.937496	0.114774	8.168159	3.13E-16	5.86E-15	CENPF	protein_cc
ENSG0000(	1750.848	0.438891	0.037131	11.8199	3.08E-32	1.07E-29	KANSL2	protein_cc
ENSG0000(	1750.807	0.033377	0.024016	1.389766	0.1646	0.236802	MTMR14	protein_cc
ENSG0000(	1750.799	-0.04689	0.047591	-0.98527	0.324491	0.418673	TRMT5	protein_cc
ENSG0000(	1750.263	0.231295	0.038227	6.050622	1.44E-09	9.17E-09	USE1	protein_cc
ENSG0000(	1749.107	-0.40772	0.044363	-9.19053	3.91E-20	1.36E-18	DUSP7	protein_cc
ENSG0000(	1748.42	-0.27671	0.040432	-6.84387	7.71E-12	6.97E-11	RUFY2	protein_cc
ENSG0000(	1747.743	0.130844	0.046382	2.821007	0.004787	0.010233	SIL1	protein_cc
ENSG0000(	1747.55	-0.20786	0.034057	-6.10336	1.04E-09	6.76E-09	ZNF142	protein_cc
ENSG0000(	1747.464	0.07357	0.040841	1.80137	0.071645	0.115623	PRKD1	protein_cc
ENSG0000(	1747.407	-0.40896	0.06707	-6.09748	1.08E-09	6.99E-09	TSPAN15	protein_cc
ENSG0000(	1747.212	-0.08264	0.034113	-2.42262	0.015409	0.029493	ZNF280D	protein_cc
ENSG0000(	1747.136	-0.47388	0.051329	-9.2323	2.65E-20	9.44E-19	PITPNM2	protein_cc
ENSG0000(	1747.051	-0.18942	0.029492	-6.42278	1.34E-10	1.00E-09	RNF170	protein_cc
ENSG0000(	1746.339	-0.01611	0.043218	-0.37282	0.709281	0.778221	RP11-345	antisense
ENSG0000(	1746.16	0.149595	0.047643	3.139917	0.00169	0.003953	CTC-444N	lincRNA
ENSG0000(	1745.959	-0.07351	0.036901	-1.99203	0.046367	0.079032	BTBD10	protein_cc
ENSG0000(	1745.823	-0.00921	0.0288	-0.31978	0.749133	0.811082	GPN2	protein_cc
ENSG0000(	1745.814	-0.11915	0.027481	-4.3356	1.45E-05	4.88E-05	RC3H1	protein_cc
ENSG0000(	1744.143	-0.49455	0.0808	-6.12065	9.32E-10	6.11E-09	FAM13C	protein_cc
ENSG0000(	1744.074	-0.04871	0.027933	-1.74375	0.081203	0.12888	MRPS30	protein_cc
ENSG0000(	1743.987	-0.0682	0.054403	-1.25369	0.209956	0.29093	ZNF276	protein_cc
ENSG0000(	1743.606	0.006729	0.027546	0.24427	0.807021	0.857902	MED9	protein_cc
ENSG0000(	1743.335	0.083937	0.027142	3.092539	0.001985	0.004581	NOL8	protein_cc
ENSG0000(	1743.093	0.233355	0.03745	6.231048	4.63E-10	3.18E-09	ARL16	protein_cc
ENSG0000(	1742.749	0.05571	0.028929	1.925779	0.054132	0.090572	SLC33A1	protein_cc
ENSG0000(	1742.205	-0.09364	0.034508	-2.71347	0.006658	0.013824	MOCS1	protein_cc
ENSG0000(	1742.181	-0.13467	0.039639	-3.39754	0.00068	0.00171	STK24	protein_cc
ENSG0000(	1741.296	0.034234	0.025728	1.330625	0.183312	0.259199	MED4	protein_cc
ENSG0000(	1740.625	0.041753	0.026096	1.599988	0.109601	0.167214	INTS5	protein_cc
ENSG0000(	1740.591	-0.05963	0.041643	-1.43189	0.152175	0.221556	POLR3D	protein_cc
ENSG0000(	1740.571	0.423373	0.095446	4.435753	9.18E-06	3.18E-05	SKAP2	protein_cc
ENSG0000(	1739.765	-0.31643	0.033579	-9.42341	4.37E-21	1.80E-19	PI4K2A	protein_cc
ENSG0000(	1739.498	-0.10548	0.165822	-0.6361	0.52471	0.616688	F13A1	protein_cc
ENSG0000(	1738.703	-0.42429	0.083885	-5.05803	4.24E-07	1.81E-06	LINC0109	lincRNA
ENSG0000(	1738.538	-1.00308	0.081797	-12.2631	1.43E-34	8.10E-32	SYNPO2	protein_cc
ENSG0000(	1738.152	-0.20445	0.055805	-3.66364	0.000249	0.000678	HMHA1	protein_cc
ENSG0000(	1737.928	-0.00205	0.034235	-0.05981	0.952309	0.967001	TRIM13	protein_cc
ENSG0000(	1737.861	-0.07333	0.036394	-2.01488	0.043917	0.075333	DGCR14	protein_cc
ENSG0000(	1737.466	0.180085	0.023796	7.567772	3.80E-14	4.96E-13	THADA	protein_cc
ENSG0000(	1737.378	0.081178	0.033839	2.398948	0.016442	0.031261	MIF4GD	protein_cc
ENSG0000(	1737.335	0.169148	0.030278	5.586457	2.32E-08	1.21E-07	MRPS10	protein_cc
ENSG0000(	1736.995	0.252027	0.037536	6.714297	1.89E-11	1.61E-10	SCNM1	protein_cc
ENSG0000(	1736.63	0.317773	0.032464	9.788493	1.26E-22	6.85E-21	NUP85	protein_cc
ENSG0000(	1736.463	-0.32697	0.058641	-5.57575	2.46E-08	1.29E-07	ZNF770	protein_cc
ENSG0000(	1736.032	-0.54268	0.052934	-10.2521	1.16E-24	9.45E-23	SFMBT2	protein_cc
ENSG0000(	1735.988	0.170613	0.06618	2.577989	0.009938	0.019867	DOK5	protein_cc
ENSG0000(	1734.904	-0.10047	0.029554	-3.39973	0.000675	0.001697	UBE2W	protein_cc
ENSG0000(	1734.524	0.281123	0.03497	8.038908	9.06E-16	1.57E-14	JAGN1	protein_cc
ENSG0000(	1734.517	-0.03631	0.040724	-0.89169	0.37256	0.469011	ZNF516	protein_cc
ENSG0000(	1734.287	0.030525	0.075843	0.402478	0.687332	0.759998	LYN	protein_cc

ENSG0000(	1734.228	-0.25959	0.057629	-4.50459	6.65E-06	2.36E-05	EML2	protein_cc
ENSG0000(	1733.299	0.014319	0.029903	0.478851	0.632045	0.713268	ZSCAN30	protein_cc
ENSG0000(	1733.112	0.162339	0.047268	3.434468	0.000594	0.001511	CDK5	protein_cc
ENSG0000(	1733.098	-0.01504	0.045265	-0.33227	0.739686	0.803223	SLC25A37	protein_cc
ENSG0000(	1731.714	-0.11509	0.030629	-3.75764	0.000172	0.000481	TMF1	protein_cc
ENSG0000(	1731.284	-0.043	0.031472	-1.36627	0.171853	0.245592	FAM73B	protein_cc
ENSG0000(	1730.518	-0.76523	0.108324	-7.06429	1.61E-12	1.63E-11	DPP10	protein_cc
ENSG0000(	1730.249	1.35138	0.048846	27.666	#######	#######	LINC0115(	antisense
ENSG0000(	1730.187	0.267768	0.032414	8.260917	1.45E-16	2.86E-15	LMAN2L	protein_cc
ENSG0000(	1729.752	-0.94346	0.088875	-10.6156	2.52E-26	2.87E-24	KCNQ3	protein_cc
ENSG0000(	1729.333	-0.22112	0.041532	-5.32419	1.01E-07	4.81E-07	DDHD1	protein_cc
ENSG0000(	1728.971	-0.00191	0.03815	-0.04996	0.960158	0.97267	MBTPS2	protein_cc
ENSG0000(	1728.781	0.295843	0.030686	9.641007	5.37E-22	2.58E-20	FTSJ2	protein_cc
ENSG0000(	1728.564	0.214185	0.030829	6.947589	3.72E-12	3.53E-11	RARS	protein_cc
ENSG0000(	1728.458	0.099004	0.039173	2.527345	0.011493	0.02265	PRR3	protein_cc
ENSG0000(	1728.21	-0.64417	0.072474	-8.88824	6.21E-19	1.79E-17	PPFIA3	protein_cc
ENSG0000(	1727.684	-0.15916	0.094828	-1.67841	0.093267	0.145389	LAIR1	protein_cc
ENSG0000(	1727.589	-0.2259	0.040398	-5.59183	2.25E-08	1.18E-07	TRAPPC6E	protein_cc
ENSG0000(	1727.351	-0.26757	0.080316	-3.33142	0.000864	0.002131	KIAA1211	protein_cc
ENSG0000(	1726.95	-0.46044	0.055259	-8.33237	7.92E-17	1.64E-15	TTBK2	protein_cc
ENSG0000(	1726.924	0.642035	0.094179	6.817182	9.28E-12	8.28E-11	PLSCR1	protein_cc
ENSG0000(	1726.865	0.043365	0.032813	1.321567	0.186312	0.26288	TRIM65	protein_cc
ENSG0000(	1726.273	-0.35953	0.03309	-10.8653	1.69E-27	2.36E-25	FAM149B1	protein_cc
ENSG0000(	1726.092	-0.06336	0.084168	-0.75276	0.451597	0.548203	SPI1	protein_cc
ENSG0000(	1725.939	-0.09646	0.035806	-2.69397	0.007061	0.014574	ACVR1	protein_cc
ENSG0000(	1725.833	-0.11021	0.040778	-2.70262	0.00688	0.014238	ZC3H12C	protein_cc
ENSG0000(	1725.423	0.112054	0.039114	2.864799	0.004173	0.009044	IFT122	protein_cc
ENSG0000(	1724.675	-0.0138	0.027015	-0.51083	0.60947	0.693157	FAM200B	protein_cc
ENSG0000(	1724.241	-0.30836	0.048123	-6.40781	1.48E-10	1.09E-09	SH3RF3	protein_cc
ENSG0000(	1723.03	0.255912	0.070641	3.622723	0.000292	0.000786	KLF10	protein_cc
ENSG0000(	1722.913	-0.29343	0.050418	-5.81985	5.89E-09	3.39E-08	RALGPS1	protein_cc
ENSG0000(	1722.148	-1.36605	0.144586	-9.44799	3.45E-21	1.45E-19	GABRG1	protein_cc
ENSG0000(	1722.122	0.016063	0.022356	0.7185	0.472449	0.568315	DHX57	protein_cc
ENSG0000(	1721.625	-0.24123	0.05147	-4.68689	2.77E-06	1.05E-05	DAAM1	protein_cc
ENSG0000(	1721.617	0.11353	0.033026	3.437548	0.000587	0.001496	GTF2IRD1	protein_cc
ENSG0000(	1720.984	0.115721	0.04105	2.819001	0.004817	0.010292	CAPRIN2	protein_cc
ENSG0000(	1720.976	-0.00893	0.024194	-0.369	0.712131	0.780456	NUDT9	protein_cc
ENSG0000(	1720.325	-0.34113	0.047859	-7.12784	1.02E-12	1.06E-11	SLC9A9	protein_cc
ENSG0000(	1719.629	0.061827	0.057879	1.068221	0.285421	0.376279	PDGFC	protein_cc
ENSG0000(	1719.385	-0.45534	0.051399	-8.85901	8.07E-19	2.28E-17	NT5C2	protein_cc
ENSG0000(	1719.306	-0.00281	0.032138	-0.08733	0.930406	0.951223	SOCS4	protein_cc
ENSG0000(	1719.192	0.180194	0.04946	3.643224	0.000269	0.00073	TAF13	protein_cc
ENSG0000(	1718.803	-0.30992	0.072185	-4.29346	1.76E-05	5.84E-05	CSMD2	protein_cc
ENSG0000(	1718.605	-0.03936	0.031166	-1.26294	0.20661	0.287039	YTHDC2	protein_cc
ENSG0000(	1718.411	-0.01131	0.03263	-0.34655	0.728928	0.794225	TCEAL1	protein_cc
ENSG0000(	1718.373	0.109684	0.03434	3.194095	0.001403	0.003331	MAD1L1	protein_cc
ENSG0000(	1717.539	-0.1386	0.117679	-1.1778	0.238876	0.324156	ANGPTL4	protein_cc
ENSG0000(	1716.859	-0.07594	0.02173	-3.49463	0.000475	0.00123	AKAP10	protein_cc
ENSG0000(	1715.578	-0.24948	0.039131	-6.37545	1.82E-10	1.33E-09	TRIP11	protein_cc
ENSG0000(	1715.374	0.142784	0.042431	3.365089	0.000765	0.001905	IFT57	protein_cc
ENSG0000(	1713.948	-0.15775	0.059506	-2.65106	0.008024	0.016386	SPSB1	protein_cc
ENSG0000(	1713.924	0.165762	0.041728	3.972441	7.11E-05	0.000213	CRELD2	protein_cc
ENSG0000(	1713.796	0.095692	0.046727	2.047877	0.040572	0.07027	NFKBIL1	protein_cc
ENSG0000(	1713.552	-0.35826	0.120771	-2.96645	0.003013	0.006711	MMD2	protein_cc
ENSG0000(	1713.475	-0.23168	0.12536	-1.84812	0.064585	0.105635	HAPLN2	protein_cc
ENSG0000(	1713.185	0.190046	0.037238	5.103592	3.33E-07	1.45E-06	RIT1	protein_cc
ENSG0000(	1712.434	-0.18693	0.025975	-7.19648	6.18E-13	6.62E-12	PPP1R21	protein_cc
ENSG0000(	1711.523	-0.37763	0.093251	-4.04957	5.13E-05	0.000158	FAM57B	protein_cc
ENSG0000(	1711.005	-0.71489	0.092932	-7.69265	1.44E-14	2.03E-13	PAIP2B	protein_cc

ENSG0000(	1710.688	-0.38894	0.058971	-6.59541	4.24E-11	3.42E-10	GPRASP2	protein_cc
ENSG0000(	1710.641	0.027837	0.043624	0.638128	0.52339	0.615355	C21orf2	protein_cc
ENSG0000(	1709.654	-1.02823	0.14384	-7.14846	8.78E-13	9.21E-12	HPCA	protein_cc
ENSG0000(	1709.552	-0.06422	0.02578	-2.49112	0.012734	0.024825	PRDM4	protein_cc
ENSG0000(	1709.281	-0.14711	0.055446	-2.65313	0.007975	0.016298	TPRN	protein_cc
ENSG0000(	1708.98	0.343688	0.08675	3.961811	7.44E-05	0.000222	RND3	protein_cc
ENSG0000(	1708.802	-0.21135	0.10289	-2.0541	0.039966	0.069342	SERPINF1	protein_cc
ENSG0000(	1708.683	-0.20843	0.055375	-3.76387	0.000167	0.000471	MAST4	protein_cc
ENSG0000(	1707.202	-0.26032	0.03383	-7.69501	1.41E-14	1.99E-13	TBRG1	protein_cc
ENSG0000(	1706.801	-0.13282	0.039945	-3.32496	0.000884	0.002176	LRBA	protein_cc
ENSG0000(	1706.67	0.042742	0.055351	0.772207	0.439992	0.53692	SATB2	protein_cc
ENSG0000(	1706.426	-0.07174	0.024357	-2.94516	0.003228	0.007148	USP30	protein_cc
ENSG0000(	1706.31	0.368891	0.078539	4.696918	2.64E-06	9.99E-06	FUCA2	protein_cc
ENSG0000(	1705.902	0.065429	0.028721	2.278113	0.02272	0.041784	MTERFD2	protein_cc
ENSG0000(	1705.831	-0.69065	0.062753	-11.0058	3.58E-28	5.58E-26	NCOA7	protein_cc
ENSG0000(	1705.72	-0.21352	0.054396	-3.92526	8.66E-05	0.000256	RORA	protein_cc
ENSG0000(	1705.476	-0.08497	0.033424	-2.54212	0.011018	0.021808	TFAM	protein_cc
ENSG0000(	1704.706	-0.26439	0.068127	-3.88086	0.000104	0.000303	GLRB	protein_cc
ENSG0000(	1704.698	0.03715	0.02853	1.302109	0.192879	0.270789	WDR12	protein_cc
ENSG0000(	1704.478	-0.06693	0.090407	-0.7403	0.459116	0.555379	MYOF	protein_cc
ENSG0000(	1704.283	0.045295	0.04769	0.949778	0.342225	0.437464	IMPDH1	protein_cc
ENSG0000(	1703.572	-0.08197	0.020426	-4.0129	6.00E-05	0.000182	PGS1	protein_cc
ENSG0000(	1703.237	0.171894	0.040459	4.24857	2.15E-05	7.04E-05	PEX1	protein_cc
ENSG0000(	1703.097	-0.00493	0.027448	-0.17947	0.857569	0.897051	PGGT1B	protein_cc
ENSG0000(	1702.862	-0.08632	0.042777	-2.0179	0.043601	0.074858	UNC119	protein_cc
ENSG0000(	1702.311	0.040295	0.036436	1.105915	0.268763	0.358015	MIER1	protein_cc
ENSG0000(	1701.919	0.125047	0.028102	4.449833	8.59E-06	3.00E-05	E4F1	protein_cc
ENSG0000(	1701.7	-0.16559	0.058324	-2.83922	0.004522	0.009716	PHKA1	protein_cc
ENSG0000(	1701.365	0.029396	0.048881	0.601373	0.547591	0.637864	PDE9A	protein_cc
ENSG0000(	1701.301	0.078562	0.030066	2.613008	0.008975	0.01812	PPP2R3C	protein_cc
ENSG0000(	1701.292	-0.51712	0.064614	-8.00333	1.21E-15	2.06E-14	PARD3B	protein_cc
ENSG0000(	1701.074	0.086756	0.088159	0.984081	0.325075	0.419283	DBNDD1	protein_cc
ENSG0000(	1700.991	0.027199	0.028704	0.947577	0.343345	0.438541	RAB2B	protein_cc
ENSG0000(	1700.82	-0.55346	0.071856	-7.70235	1.34E-14	1.89E-13	RNF150	protein_cc
ENSG0000(	1700.306	-0.26268	0.088823	-2.95729	0.003104	0.006896	C3AR1	protein_cc
ENSG0000(	1700.248	0.163492	0.030923	5.287014	1.24E-07	5.80E-07	ILKAP	protein_cc
ENSG0000(	1700.097	0.298146	0.046206	6.452528	1.10E-10	8.33E-10	TMBIM4	protein_cc
ENSG0000(	1699.955	-0.48217	0.161269	-2.98982	0.002791	0.006258	COL5A1	protein_cc
ENSG0000(	1699.579	-0.49788	0.051354	-9.69496	3.17E-22	1.60E-20	ACADSB	protein_cc
ENSG0000(	1699.409	-0.46257	0.052592	-8.79533	1.43E-18	3.88E-17	FRMD4A	protein_cc
ENSG0000(	1699.031	0.450793	0.049121	9.177243	4.42E-20	1.53E-18	MRPL54	protein_cc
ENSG0000(	1699.014	0.062372	0.105039	0.593797	0.552648	0.642353	PAMR1	protein_cc
ENSG0000(	1698.791	0.112077	0.02234	5.016835	5.25E-07	2.22E-06	AKAP8	protein_cc
ENSG0000(	1698.652	-0.50012	0.097208	-5.14488	2.68E-07	1.18E-06	SDPR	protein_cc
ENSG0000(	1698.647	0.269409	0.030743	8.763399	1.89E-18	5.06E-17	FLAD1	protein_cc
ENSG0000(	1698.316	-0.10362	0.030913	-3.35198	0.000802	0.001991	MORC3	protein_cc
ENSG0000(	1697.419	0.035873	0.02097	1.710678	0.087141	0.137133	MRPS22	protein_cc
ENSG0000(	1697.351	-0.08555	0.040086	-2.1342	0.032826	0.058157	ENGASE	protein_cc
ENSG0000(	1696.638	0.247204	0.039179	6.309582	2.80E-10	1.99E-09	ARMC10	protein_cc
ENSG0000(	1696.322	-0.78151	0.155335	-5.03109	4.88E-07	2.07E-06	SVOP	protein_cc
ENSG0000(	1695.217	-0.01383	0.034923	-0.39615	0.691995	0.763881	FKTN	protein_cc
ENSG0000(	1695.119	0.216966	0.038999	5.563416	2.65E-08	1.37E-07	MRPL55	protein_cc
ENSG0000(	1695.112	0.116163	0.023492	4.944854	7.62E-07	3.14E-06	CSTF3	protein_cc
ENSG0000(	1694.98	-0.08001	0.032236	-2.48213	0.01306	0.025402	VPRBP	protein_cc
ENSG0000(	1694.677	0.237237	0.070819	3.349921	0.000808	0.002004	ARID5A	protein_cc
ENSG0000(	1694.543	0.776225	0.124756	6.221961	4.91E-10	3.35E-09	PLP2	protein_cc
ENSG0000(	1694.339	0.214679	0.030879	6.952275	3.59E-12	3.43E-11	C11orf73	protein_cc
ENSG0000(	1693.75	-0.12572	0.032063	-3.921	8.82E-05	0.00026	RNF111	protein_cc
ENSG0000(	1693.662	-0.26585	0.050747	-5.23871	1.62E-07	7.41E-07	MAP9	protein_cc

ENSG0000(	1693.587	0.097308	0.025352	3.838314	0.000124	0.000356	AGBL5	protein_cc
ENSG0000(	1692.716	0.108492	0.069469	1.561743	0.118349	0.178637	ELK3	protein_cc
ENSG0000(	1692.239	0.087219	0.03483	2.504155	0.012274	0.024019	CNNM3	protein_cc
ENSG0000(	1691.883	0.057313	0.025834	2.218476	0.026522	0.048045	INIP	protein_cc
ENSG0000(	1691.805	-0.21784	0.032943	-6.61264	3.78E-11	3.07E-10	MTHFR	protein_cc
ENSG0000(	1691.503	-0.00934	0.044298	-0.21083	0.833023	0.878208	GSTM4	protein_cc
ENSG0000(	1691.046	-1.12156	0.113412	-9.88922	4.64E-23	2.76E-21	MICAL2	protein_cc
ENSG0000(	1690.483	-0.02015	0.028656	-0.70308	0.482007	0.577162	ARL14EP	protein_cc
ENSG0000(	1690.333	0.335806	0.068559	4.898064	9.68E-07	3.92E-06	NMRAL1	protein_cc
ENSG0000(	1689.742	-0.01183	0.063455	-0.18641	0.852125	0.893071	PLXNC1	protein_cc
ENSG0000(	1689.546	-0.21343	0.029911	-7.13543	9.65E-13	1.01E-11	PDHX	protein_cc
ENSG0000(	1689.488	-0.17627	0.032059	-5.49833	3.83E-08	1.94E-07	PNPO	protein_cc
ENSG0000(	1689.463	-0.21363	0.04447	-4.80393	1.56E-06	6.10E-06	RAVER2	protein_cc
ENSG0000(	1689.399	0.525287	0.056193	9.347899	8.94E-21	3.46E-19	BTG3	protein_cc
ENSG0000(	1688.134	0.055349	0.098354	0.562757	0.573601	0.660934	GYG2	protein_cc
ENSG0000(	1688.029	0.287195	0.030913	9.290458	1.54E-20	5.70E-19	DNTTIP1	protein_cc
ENSG0000(	1687.904	0.016735	0.043708	0.382881	0.701808	0.772143	RFFL	protein_cc
ENSG0000(	1687.839	0.134391	0.030445	4.41427	1.01E-05	3.50E-05	ARFIP1	protein_cc
ENSG0000(	1687.645	0.204645	0.039957	5.121656	3.03E-07	1.33E-06	NCAPD3	protein_cc
ENSG0000(	1687.265	-0.21496	0.045797	-4.69375	2.68E-06	1.01E-05	ARHGAP3	protein_cc
ENSG0000(	1687.137	-0.51562	0.066524	-7.75083	9.13E-15	1.34E-13	HID1	protein_cc
ENSG0000(	1687.128	-0.12655	0.047587	-2.6593	0.00783	0.016026	MMP24-A	antisense
ENSG0000(	1686.821	0.121837	0.030064	4.052653	5.06E-05	0.000156	NUP155	protein_cc
ENSG0000(	1686.663	-0.0248	0.028476	-0.871	0.383757	0.480474	FAM222B	protein_cc
ENSG0000(	1686.255	0.330537	0.047945	6.894081	5.42E-12	5.01E-11	TMUB1	protein_cc
ENSG0000(	1685.96	0.018292	0.041711	0.438534	0.660999	0.737845	ZNF384	protein_cc
ENSG0000(	1685.832	0.157311	0.031206	5.041025	4.63E-07	1.97E-06	TEX10	protein_cc
ENSG0000(	1685.811	-0.09255	0.035492	-2.60752	0.00912	0.018378	ZNF189	protein_cc
ENSG0000(	1685.395	-0.20862	0.099577	-2.09508	0.036164	0.063459	IFITM1	protein_cc
ENSG0000(	1685.37	0.191549	0.080192	2.388641	0.016911	0.032048	EPHB2	protein_cc
ENSG0000(	1684.481	0.052092	0.02206	2.361374	0.018207	0.034221	STX18	protein_cc
ENSG0000(	1684.159	0.203163	0.036689	5.537485	3.07E-08	1.58E-07	TMEM19	protein_cc
ENSG0000(	1683.7	0.058735	0.03917	1.499471	0.133751	0.198162	TMEM201	protein_cc
ENSG0000(	1682.547	0.170634	0.022929	7.441881	9.93E-14	1.21E-12	ZNF562	protein_cc
ENSG0000(	1682.265	0.089934	0.02649	3.394979	0.000686	0.001724	MRPL50	protein_cc
ENSG0000(	1682.251	0.066454	0.027548	2.412278	0.015853	0.030258	RCHY1	protein_cc
ENSG0000(	1681.852	-0.34547	0.081593	-4.23402	2.30E-05	7.46E-05	GALNT8	protein_cc
ENSG0000(	1681.837	-0.00495	0.025088	-0.19725	0.843634	0.886429	TXNDC11	protein_cc
ENSG0000(	1681.827	0.19815	0.035414	5.595327	2.20E-08	1.16E-07	MSH2	protein_cc
ENSG0000(	1681.818	-0.82646	0.108662	-7.6058	2.83E-14	3.77E-13	CACNG8	protein_cc
ENSG0000(	1681.381	-0.24248	0.090857	-2.66877	0.007613	0.015623	TMEM163	protein_cc
ENSG0000(	1681.316	0.143157	0.034255	4.179109	2.93E-05	9.35E-05	CMC2	protein_cc
ENSG0000(	1681.077	-0.17006	0.033755	-5.03801	4.70E-07	2.00E-06	MFHAS1	protein_cc
ENSG0000(	1680.715	0.07963	0.029599	2.690282	0.007139	0.014722	MOB4	protein_cc
ENSG0000(	1680.101	0.183134	0.032784	5.586155	2.32E-08	1.22E-07	MAPK7	protein_cc
ENSG0000(	1679.918	-1.1989	0.125793	-9.53074	1.56E-21	6.98E-20	EPHB6	protein_cc
ENSG0000(	1679.289	-0.18593	0.039988	-4.64968	3.32E-06	1.24E-05	NR2F1-AS	processed
ENSG0000(	1679.264	0.456316	0.064946	7.026046	2.12E-12	2.10E-11	H3F3A	protein_cc
ENSG0000(	1679.093	0.201004	0.024743	8.123767	4.52E-16	8.26E-15	ZKSCAN5	protein_cc
ENSG0000(	1679.002	-0.8949	0.12716	-7.03755	1.96E-12	1.95E-11	CNTNAP4	protein_cc
ENSG0000(	1678.819	0.09189	0.044442	2.067636	0.038674	0.06734	SLC5A6	protein_cc
ENSG0000(	1678.758	0.180897	0.041076	4.403954	1.06E-05	3.65E-05	CEP41	protein_cc
ENSG0000(	1678.339	0.092699	0.025459	3.641094	0.000271	0.000736	RPE	protein_cc
ENSG0000(	1678.239	-0.97983	0.104075	-9.41467	4.75E-21	1.94E-19	NECAB1	protein_cc
ENSG0000(	1677.844	-0.0632	0.033176	-1.90501	0.056779	0.094411	LEMD3	protein_cc
ENSG0000(	1677.755	0.027851	0.045828	0.607724	0.54337	0.634043	TNIP2	protein_cc
ENSG0000(	1677.687	0.083809	0.057178	1.465755	0.142715	0.209585	MRPL41	protein_cc
ENSG0000(	1677.335	0.191919	0.038433	4.993625	5.93E-07	2.48E-06	FUZ	protein_cc
ENSG0000(	1677.067	-0.08583	0.022858	-3.75477	0.000173	0.000486	CCDC127	protein_cc

ENSG0000(	1675.541	0.265671	0.035933	7.39352	1.43E-13	1.70E-12	SNRPF	protein_cc
ENSG0000(	1675.152	0.105382	0.049145	2.144322	0.032007	0.056848	10-Sep	protein_cc
ENSG0000(	1675.114	0.132064	0.0331	3.989884	6.61E-05	0.000199	RNF126	protein_cc
ENSG0000(	1675.014	0.156132	0.05234	2.983037	0.002854	0.006386	UAP1L1	protein_cc
ENSG0000(	1674.855	0.024277	0.03773	0.643446	0.519934	0.61236	ARID2	protein_cc
ENSG0000(	1673.908	-0.18999	0.05416	-3.50795	0.000452	0.001176	PLA2G6	protein_cc
ENSG0000(	1673.793	-0.21745	0.045594	-4.76918	1.85E-06	7.17E-06	JRK	processed
ENSG0000(	1673.759	0.15604	0.035021	4.455627	8.36E-06	2.92E-05	LMBR1L	protein_cc
ENSG0000(	1673.703	0.054838	0.037302	1.470102	0.141534	0.208059	CEP164	protein_cc
ENSG0000(	1673.183	-0.07028	0.039263	-1.7901	0.073437	0.118164	PEX16	protein_cc
ENSG0000(	1673.12	-0.01247	0.036996	-0.33702	0.736102	0.800235	BBS10	protein_cc
ENSG0000(	1671.932	0.268441	0.031465	8.531532	1.44E-17	3.36E-16	ZNF12	protein_cc
ENSG0000(	1671.34	-0.25055	0.090825	-2.75862	0.005805	0.012198	RNF165	protein_cc
ENSG0000(	1670.254	0.111874	0.030881	3.622748	0.000291	0.000786	MCOLN1	protein_cc
ENSG0000(	1669.708	-0.73107	0.108414	-6.74336	1.55E-11	1.33E-10	UNC5A	protein_cc
ENSG0000(	1669.127	0.100261	0.043711	2.293741	0.021805	0.040261	RFK	protein_cc
ENSG0000(	1668.932	0.115077	0.030675	3.751441	0.000176	0.000492	C2orf68	protein_cc
ENSG0000(	1668.804	-0.24918	0.025118	-9.92059	3.39E-23	2.06E-21	C10orf76	protein_cc
ENSG0000(	1667.895	-0.04369	0.02206	-1.98047	0.04765	0.080929	HARS2	protein_cc
ENSG0000(	1667.069	-1.08152	0.107023	-10.1055	5.22E-24	3.80E-22	CAMKK1	protein_cc
ENSG0000(	1666.616	0.042995	0.034451	1.247998	0.212032	0.293352	TIMM10B	protein_cc
ENSG0000(	1666.544	0.153773	0.068799	2.235112	0.02541	0.046211	MLTK	protein_cc
ENSG0000(	1666.23	0.017394	0.033898	0.513128	0.607862	0.691633	KLHDC4	protein_cc
ENSG0000(	1665.656	-0.02699	0.045992	-0.58693	0.557248	0.646494	OSBPL5	protein_cc
ENSG0000(	1665.415	-0.5162	0.067008	-7.70366	1.32E-14	1.87E-13	PTPRB	protein_cc
ENSG0000(	1665.319	-0.46963	0.089876	-5.22538	1.74E-07	7.92E-07	HEPH	protein_cc
ENSG0000(	1664.614	0.544123	0.098605	5.518213	3.42E-08	1.75E-07	P2RY1	protein_cc
ENSG0000(	1664.396	-0.07305	0.043949	-1.66224	0.096464	0.149732	SH3RF1	protein_cc
ENSG0000(	1664.36	-0.55399	0.122587	-4.51914	6.21E-06	2.21E-05	FA2H	protein_cc
ENSG0000(	1664.243	-0.01645	0.029063	-0.56593	0.571444	0.659024	PIAS3	protein_cc
ENSG0000(	1663.813	0.060954	0.070205	0.868235	0.385266	0.481922	CCDC85B	protein_cc
ENSG0000(	1663.01	0.358116	0.069318	5.166301	2.39E-07	1.06E-06	LIMD1	protein_cc
ENSG0000(	1662.667	-0.31453	0.045666	-6.88762	5.67E-12	5.23E-11	WWOX	protein_cc
ENSG0000(	1662.262	0.273692	0.039378	6.950448	3.64E-12	3.47E-11	UCKL1	protein_cc
ENSG0000(	1662.136	0.228211	0.036326	6.282264	3.34E-10	2.34E-09	VRK3	protein_cc
ENSG0000(	1661.933	0.599326	0.065795	9.108973	8.32E-20	2.75E-18	FAM126A	protein_cc
ENSG0000(	1661.446	0.07992	0.058906	1.356728	0.174868	0.249264	TMTC4	protein_cc
ENSG0000(	1661.033	-0.31845	0.052068	-6.1161	9.59E-10	6.27E-09	SGMS1	protein_cc
ENSG0000(	1660.911	0.401878	0.045817	8.771387	1.76E-18	4.72E-17	SNRNP40	protein_cc
ENSG0000(	1660.692	-0.13043	0.03575	-3.64841	0.000264	0.000717	RRAGB	protein_cc
ENSG0000(	1660.622	0.129434	0.046689	2.772261	0.005567	0.011744	HMGCL	protein_cc
ENSG0000(	1660.442	-0.62077	0.140867	-4.40676	1.05E-05	3.61E-05	KLK6	protein_cc
ENSG0000(	1660.322	-0.37828	0.039028	-9.69265	3.24E-22	1.63E-20	STAM	protein_cc
ENSG0000(	1660.182	0.07367	0.031473	2.340767	0.019244	0.035964	CTPS2	protein_cc
ENSG0000(	1659.454	0.075203	0.072804	1.032964	0.301621	0.394261	ERRFI1	protein_cc
ENSG0000(	1659.433	-0.41539	0.044775	-9.27737	1.74E-20	6.38E-19	C2CD2L	protein_cc
ENSG0000(	1659.07	0.06619	0.038087	1.737874	0.082233	0.13026	ZNF775	protein_cc
ENSG0000(	1658.653	-0.25343	0.065678	-3.85868	0.000114	0.00033	PPFIBP1	protein_cc
ENSG0000(	1658.205	-0.69877	0.075339	-9.27501	1.78E-20	6.52E-19	CACNB1	protein_cc
ENSG0000(	1658.162	0.260231	0.032609	7.980408	1.46E-15	2.45E-14	ELP6	protein_cc
ENSG0000(	1657.593	-0.19512	0.102839	-1.89731	0.057787	0.095902	LINC0110!	processed
ENSG0000(	1657.552	0.243784	0.038965	6.256465	3.94E-10	2.73E-09	MRPL21	protein_cc
ENSG0000(	1657.155	0.045662	0.031529	1.448218	0.147556	0.215731	PPIP5K2	protein_cc
ENSG0000(	1656.701	0.174581	0.033169	5.263342	1.41E-07	6.54E-07	MFSD5	protein_cc
ENSG0000(	1656.624	-0.19058	0.029147	-6.53868	6.21E-11	4.88E-10	VCPIP1	protein_cc
ENSG0000(	1655.2	-0.41477	0.217025	-1.91118	0.055981	0.093265	DDX3Y	protein_cc
ENSG0000(	1655.048	-0.29357	0.062119	-4.72593	2.29E-06	8.75E-06	STAT6	protein_cc
ENSG0000(	1654.578	-0.1824	0.035997	-5.06691	4.04E-07	1.74E-06	BTBD7	protein_cc
ENSG0000(	1654.441	0.053297	0.027803	1.916976	0.055241	0.092208	WDR43	protein_cc

ENSG0000(	1654.161	0.034699	0.040909	0.848203	0.396325	0.492939	CEP192	protein_cc
ENSG0000(	1654.062	0.150214	0.083704	1.794587	0.07272	0.117188	ADAMTS9	protein_cc
ENSG0000(	1653.753	-0.29507	0.035415	-8.33174	7.97E-17	1.65E-15	PPP2R2D	protein_cc
ENSG0000(	1651.09	-0.06914	0.039885	-1.73355	0.082999	0.131279	MAP3K4	protein_cc
ENSG0000(	1650.12	0.052347	0.037033	1.413515	0.157504	0.228015	ADNP2	protein_cc
ENSG0000(	1649.961	-0.50488	0.091649	-5.50883	3.61E-08	1.84E-07	SLITRK5	protein_cc
ENSG0000(	1649.844	0.274367	0.068881	3.983219	6.80E-05	0.000205	AGTRAP	protein_cc
ENSG0000(	1649.6	0.952152	0.14114	6.74614	1.52E-11	1.31E-10	TNFRSF12	protein_cc
ENSG0000(	1648.968	-0.00738	0.053563	-0.13773	0.890452	0.921997	LGR4	protein_cc
ENSG0000(	1648.934	-0.01079	0.083644	-0.12903	0.89733	0.927228	ITGAX	protein_cc
ENSG0000(	1648.758	-0.21205	0.034054	-6.22694	4.76E-10	3.26E-09	DBT	protein_cc
ENSG0000(	1648.125	0.119725	0.037264	3.212861	0.001314	0.003136	CENPBD1I	pseudoge
ENSG0000(	1647.953	-0.05759	0.041619	-1.38377	0.166429	0.239016	PLA2G15	protein_cc
ENSG0000(	1646.931	-0.11158	0.057828	-1.92959	0.053657	0.089882	ITIH5	protein_cc
ENSG0000(	1646.6	0.269631	0.119962	2.247629	0.0246	0.044878	HSPB6	protein_cc
ENSG0000(	1646.205	-0.06217	0.023365	-2.66094	0.007792	0.015959	SCRN3	protein_cc
ENSG0000(	1645.979	0.180519	0.043376	4.1617	3.16E-05	0.0001	EXTL2	protein_cc
ENSG0000(	1645.885	0.054673	0.080523	0.678975	0.497154	0.591288	LDLR	protein_cc
ENSG0000(	1645.692	-0.17319	0.03817	-4.53733	5.70E-06	2.05E-05	EVI5	protein_cc
ENSG0000(	1645.608	0.037852	0.032299	1.171926	0.241227	0.326753	NOB1	protein_cc
ENSG0000(	1645.577	-0.02349	0.033749	-0.69611	0.48636	0.581207	SDE2	protein_cc
ENSG0000(	1645.474	-0.08151	0.026712	-3.05133	0.002278	0.005201	DDX50	protein_cc
ENSG0000(	1644.819	-0.08604	0.042901	-2.00562	0.044897	0.076796	ZFC3H1	protein_cc
ENSG0000(	1644.755	-0.00417	0.030845	-0.13521	0.892448	0.923461	C1orf27	protein_cc
ENSG0000(	1644.456	0.751301	0.05754	13.05711	5.79E-39	7.00E-36	DTYMK	protein_cc
ENSG0000(	1644.317	-0.131	0.068134	-1.92273	0.054515	0.091143	CECR2	protein_cc
ENSG0000(	1643.38	0.135144	0.024942	5.418263	6.02E-08	2.95E-07	NOP14	protein_cc
ENSG0000(	1642.812	0.098348	0.024081	4.084062	4.43E-05	0.000137	MED17	protein_cc
ENSG0000(	1641.445	0.103285	0.061366	1.683102	0.092355	0.144176	ZNF692	protein_cc
ENSG0000(	1641.064	0.654052	0.121612	5.378194	7.52E-08	3.63E-07	MKI67	protein_cc
ENSG0000(	1640.919	0.018038	0.025912	0.696121	0.486353	0.581207	LIN7C	protein_cc
ENSG0000(	1640.825	-0.20682	0.035047	-5.90124	3.61E-09	2.15E-08	FAM21C	protein_cc
ENSG0000(	1640.479	-0.35771	0.052589	-6.80195	1.03E-11	9.14E-11	TARSL2	protein_cc
ENSG0000(	1639.997	-0.17631	0.03447	-5.1148	3.14E-07	1.37E-06	KDM1B	protein_cc
ENSG0000(	1639.968	-0.5372	0.078587	-6.83569	8.16E-12	7.35E-11	DGKI	protein_cc
ENSG0000(	1639.497	-0.84569	0.079765	-10.6024	2.91E-26	3.26E-24	CYP46A1	protein_cc
ENSG0000(	1639.3	0.116403	0.043768	2.659507	0.007826	0.016018	B3GALT6	protein_cc
ENSG0000(	1638.396	0.479064	0.100872	4.749237	2.04E-06	7.86E-06	SCUBE2	protein_cc
ENSG0000(	1637.787	0.177602	0.060816	2.920335	0.003497	0.007693	BTN3A3	protein_cc
ENSG0000(	1637.686	0.038018	0.031583	1.203739	0.22869	0.31253	GCC1	protein_cc
ENSG0000(	1637.655	-0.00957	0.032064	-0.29856	0.765279	0.823814	FAM160A;	protein_cc
ENSG0000(	1637.396	0.30402	0.071507	4.25162	2.12E-05	6.95E-05	OSBPL3	protein_cc
ENSG0000(	1637.292	-0.04958	0.039998	-1.23963	0.21511	0.296972	FARP2	protein_cc
ENSG0000(	1637.24	0.319153	0.046006	6.937138	4.00E-12	3.79E-11	ZNF117	protein_cc
ENSG0000(	1637.192	0.314792	0.040491	7.774446	7.58E-15	1.13E-13	DUS3L	protein_cc
ENSG0000(	1636.891	-0.84872	0.092955	-9.13045	6.82E-20	2.29E-18	SCN8A	protein_cc
ENSG0000(	1636.416	0.659	0.063904	10.31237	6.20E-25	5.34E-23	RACGAP1	protein_cc
ENSG0000(	1636.305	-0.58371	0.060774	-9.60456	7.65E-22	3.57E-20	SH3BGRL2	protein_cc
ENSG0000(	1636.139	-0.06554	0.031191	-2.10114	0.035628	0.062609	ZBTB1	protein_cc
ENSG0000(	1634.581	0.286234	0.034367	8.328861	8.16E-17	1.68E-15	DPY30	protein_cc
ENSG0000(	1633.576	-0.09557	0.026613	-3.59109	0.000329	0.00088	SLC26A11	protein_cc
ENSG0000(	1632.899	0.352382	0.100798	3.495918	0.000472	0.001225	PLIN2	protein_cc
ENSG0000(	1631.999	0.161609	0.058144	2.77946	0.005445	0.011516	SNHG8	lincRNA
ENSG0000(	1630.989	-0.264	0.065465	-4.03266	5.51E-05	0.000169	SNTB1	protein_cc
ENSG0000(	1630.979	-0.08962	0.033845	-2.64792	0.008099	0.016526	TMEM192	protein_cc
ENSG0000(	1629.892	0.402038	0.054047	7.438736	1.02E-13	1.23E-12	LSM10	protein_cc
ENSG0000(	1629.262	0.035956	0.039416	0.912219	0.361653	0.457548	C1GALT1	protein_cc
ENSG0000(	1628.985	0.08533	0.046703	1.827087	0.067687	0.110043	TRAF3IP2	protein_cc
ENSG0000(	1628.858	-0.05042	0.071535	-0.70487	0.480893	0.576231	MDGA1	protein_cc

ENSG0000(	1628.595	0.251697	0.048845	5.152939	2.56E-07	1.14E-06	TMEM168	protein_cc
ENSG0000(	1628.29	0.287801	0.084641	3.400267	0.000673	0.001695	EFNB2	protein_cc
ENSG0000(	1627.927	0.249496	0.039952	6.244868	4.24E-10	2.93E-09	CARS2	protein_cc
ENSG0000(	1627.608	-0.06173	0.114466	-0.53927	0.589702	0.675625	MYBPC1	protein_cc
ENSG0000(	1627.534	-0.16626	0.05561	-2.98969	0.002793	0.00626	FOXP4	protein_cc
ENSG0000(	1627.469	0.351197	0.041791	8.403671	4.33E-17	9.35E-16	IAH1	protein_cc
ENSG0000(	1627.438	0.143478	0.029449	4.872085	1.10E-06	4.43E-06	CACTIN	protein_cc
ENSG0000(	1627.094	0.118292	0.027952	4.23194	2.32E-05	7.53E-05	ZXDC	protein_cc
ENSG0000(	1626.581	-0.04877	0.028116	-1.73473	0.082788	0.131021	TTPAL	protein_cc
ENSG0000(	1626.532	0.258544	0.091902	2.81327	0.004904	0.010467	CAPS	protein_cc
ENSG0000(	1626.423	0.120261	0.032154	3.740162	0.000184	0.000513	CCNC	protein_cc
ENSG0000(	1626.147	0.219294	0.03067	7.150193	8.67E-13	9.10E-12	COQ5	protein_cc
ENSG0000(	1626.125	-0.41334	0.050866	-8.12591	4.44E-16	8.14E-15	SEC61A2	protein_cc
ENSG0000(	1625.951	0.177711	0.034403	5.165506	2.40E-07	1.07E-06	OGFOD3	protein_cc
ENSG0000(	1625.914	0.242925	0.029308	8.28867	1.15E-16	2.30E-15	FOPNL	protein_cc
ENSG0000(	1625.476	0.046888	0.049821	0.941145	0.34663	0.442106	LINC0093(	lincRNA
ENSG0000(	1625.261	0.122867	0.033606	3.656078	0.000256	0.000697	UHRF2	protein_cc
ENSG0000(	1624.536	-0.34637	0.051843	-6.68111	2.37E-11	1.99E-10	PTPN4	protein_cc
ENSG0000(	1624.349	-0.06657	0.036593	-1.81917	0.068885	0.111744	TDRD7	protein_cc
ENSG0000(	1623.871	0.258686	0.067351	3.840839	0.000123	0.000353	CXADR	protein_cc
ENSG0000(	1623.481	-0.48049	0.074117	-6.48291	9.00E-11	6.91E-10	SLCO3A1	protein_cc
ENSG0000(	1622.76	-0.02937	0.029012	-1.01221	0.311439	0.404716	SLC38A7	protein_cc
ENSG0000(	1622.749	0.336885	0.046909	7.181736	6.88E-13	7.34E-12	EFHC1	protein_cc
ENSG0000(	1622.304	-0.90586	0.098029	-9.24074	2.45E-20	8.78E-19	RAP1GAP2	protein_cc
ENSG0000(	1622.156	-0.05625	0.068122	-0.82576	0.408938	0.505749	BLVRB	protein_cc
ENSG0000(	1621.835	-0.15771	0.038947	-4.04931	5.14E-05	0.000158	KATNAL1	protein_cc
ENSG0000(	1621.805	0.156324	0.032856	4.757799	1.96E-06	7.55E-06	SNX11	protein_cc
ENSG0000(	1621.357	0.140219	0.027475	5.103518	3.33E-07	1.45E-06	CCNH	protein_cc
ENSG0000(	1621.342	0.173071	0.026415	6.551964	5.68E-11	4.49E-10	NUP88	protein_cc
ENSG0000(	1621.291	-0.20382	0.05265	-3.8712	0.000108	0.000314	BCOR	protein_cc
ENSG0000(	1621.071	-0.40368	0.077798	-5.18875	2.12E-07	9.52E-07	BEST1	protein_cc
ENSG0000(	1620.886	-0.15968	0.028635	-5.57635	2.46E-08	1.28E-07	ZNF641	protein_cc
ENSG0000(	1620.678	-0.33424	0.082052	-4.07354	4.63E-05	0.000143	MAP6D1	protein_cc
ENSG0000(	1620.113	1.006833	0.118289	8.511658	1.71E-17	3.95E-16	RAB34	protein_cc
ENSG0000(	1619.89	0.024915	0.03733	0.667433	0.504496	0.598292	LONRF1	protein_cc
ENSG0000(	1619.833	0.523702	0.089186	5.872048	4.30E-09	2.53E-08	NETO2	protein_cc
ENSG0000(	1619.765	-0.12048	0.041114	-2.93048	0.003384	0.007465	ADK	protein_cc
ENSG0000(	1619.717	0.012946	0.042458	0.304906	0.760438	0.819976	PTBP3	protein_cc
ENSG0000(	1619.319	0.20684	0.027963	7.397029	1.39E-13	1.65E-12	STK16	protein_cc
ENSG0000(	1619.064	0.26561	0.029776	8.920233	4.65E-19	1.36E-17	TMEM128	protein_cc
ENSG0000(	1618.922	0.120662	0.0435	2.773835	0.00554	0.011694	TAZ	protein_cc
ENSG0000(	1618.637	-0.02697	0.024839	-1.08571	0.277606	0.367899	CWC27	protein_cc
ENSG0000(	1618.606	-0.70798	0.092713	-7.63629	2.24E-14	3.04E-13	MGAT5B	protein_cc
ENSG0000(	1618.445	0.20941	0.058964	3.551464	0.000383	0.001011	B3GALTL	protein_cc
ENSG0000(	1618.269	0.019933	0.044818	0.444744	0.656505	0.734063	PAQR3	protein_cc
ENSG0000(	1617.886	0.133084	0.03852	3.454922	0.00055	0.001409	WDR60	protein_cc
ENSG0000(	1617.25	0.00324	0.027885	0.116181	0.907509	0.934488	C18orf25	protein_cc
ENSG0000(	1616.814	0.05713	0.0373	1.531617	0.125617	0.187918	CHAMP1	protein_cc
ENSG0000(	1616.451	-0.03247	0.037096	-0.8753	0.381412	0.478032	ZNF646	protein_cc
ENSG0000(	1615.98	0.423562	0.058361	7.257647	3.94E-13	4.35E-12	MRPS15	protein_cc
ENSG0000(	1615.758	0.332926	0.07522	4.426024	9.60E-06	3.32E-05	RHPN2	protein_cc
ENSG0000(	1615.158	0.21753	0.046594	4.668635	3.03E-06	1.14E-05	PKD2	protein_cc
ENSG0000(	1614.823	0.45892	0.045412	10.10568	5.21E-24	3.80E-22	STRA13	protein_cc
ENSG0000(	1614.589	-1.2611	0.111035	-11.3577	6.79E-30	1.48E-27	SCN2B	protein_cc
ENSG0000(	1614.374	1.040071	0.107823	9.646074	5.11E-22	2.47E-20	TPX2	protein_cc
ENSG0000(	1613.776	-0.25934	0.031822	-8.14983	3.64E-16	6.76E-15	HERC4	protein_cc
ENSG0000(	1613.611	0.057894	0.069966	0.827462	0.407975	0.504801	KITLG	protein_cc
ENSG0000(	1613.112	0.042776	0.027573	1.551362	0.120815	0.181713	CCNT1	protein_cc
ENSG0000(	1612.977	0.121562	0.02196	5.535577	3.10E-08	1.59E-07	ZNF791	protein_cc

ENSG0000(	1612.799	-0.13353	0.045275	-2.94924	0.003186	0.007061	NDUFB8	protein_cc
ENSG0000(	1612.153	-0.48561	0.066507	-7.30164	2.84E-13	3.21E-12	NYAP1	protein_cc
ENSG0000(	1611.189	-1.2263	0.151453	-8.09689	5.64E-16	1.01E-14	SYT13	protein_cc
ENSG0000(	1611.054	-0.10262	0.031915	-3.21533	0.001303	0.00311	RBMS2	protein_cc
ENSG0000(	1610.744	0.348668	0.037638	9.263746	1.97E-20	7.20E-19	POP7	protein_cc
ENSG0000(	1610.655	-0.43393	0.056688	-7.65475	1.94E-14	2.66E-13	SPNS2	protein_cc
ENSG0000(	1610.221	-0.49873	0.088839	-5.61388	1.98E-08	1.05E-07	RP11-2E1	lincRNA
ENSG0000(	1609.23	-0.28977	0.034711	-8.34808	6.94E-17	1.45E-15	WWP1	protein_cc
ENSG0000(	1609.057	-0.15836	0.036617	-4.32464	1.53E-05	5.12E-05	UHRF1BP1	protein_cc
ENSG0000(	1609.043	0.115834	0.034586	3.349152	0.000811	0.002009	POLR1B	protein_cc
ENSG0000(	1608.87	0.175836	0.046108	3.813555	0.000137	0.000391	SLC35F5	protein_cc
ENSG0000(	1608.648	-0.28206	0.058972	-4.78302	1.73E-06	6.72E-06	ABHD8	protein_cc
ENSG0000(	1608.121	-0.36829	0.065021	-5.66418	1.48E-08	7.98E-08	STK32C	protein_cc
ENSG0000(	1607.894	-0.87488	0.186268	-4.69689	2.64E-06	9.99E-06	OPALIN	protein_cc
ENSG0000(	1607.622	0.163785	0.041306	3.965166	7.33E-05	0.00022	OSER1-AS	lincRNA
ENSG0000(	1607.55	0.000342	0.065227	0.005249	0.995812	0.997291	RFX2	protein_cc
ENSG0000(	1607.478	-0.05898	0.032558	-1.81151	0.070061	0.113403	ATG14	protein_cc
ENSG0000(	1607.407	0.204869	0.039533	5.182212	2.19E-07	9.84E-07	TRMU	protein_cc
ENSG0000(	1607.301	-0.048	0.03738	-1.28417	0.199082	0.27813	RNF215	protein_cc
ENSG0000(	1607.28	-0.07467	0.027266	-2.73866	0.006169	0.012892	PANK2	protein_cc
ENSG0000(	1607.248	-0.17678	0.08713	-2.02894	0.042465	0.073145	ABCA8	protein_cc
ENSG0000(	1607.046	-0.05213	0.037545	-1.38846	0.164998	0.237285	HAUS6	protein_cc
ENSG0000(	1607.044	-0.05481	0.031438	-1.74347	0.081252	0.128932	HIBCH	protein_cc
ENSG0000(	1605.744	0.927322	0.092776	9.99526	1.60E-23	1.06E-21	CD97	protein_cc
ENSG0000(	1605.353	0.398769	0.076815	5.191306	2.09E-07	9.40E-07	HELZ2	protein_cc
ENSG0000(	1605.265	-0.44811	0.049963	-8.96871	3.00E-19	9.07E-18	LRP11	protein_cc
ENSG0000(	1605.204	-0.02766	0.048126	-0.57485	0.565393	0.653523	TNFSF12	protein_cc
ENSG0000(	1605.145	0.036449	0.027139	1.343055	0.179254	0.254396	MRPL30	protein_cc
ENSG0000(	1603.594	1.476229	0.134398	10.984	4.56E-28	6.98E-26	NPNT	protein_cc
ENSG0000(	1603.341	-0.60064	0.137446	-4.37004	1.24E-05	4.22E-05	NTSR2	protein_cc
ENSG0000(	1603.161	-0.50109	0.104431	-4.79832	1.60E-06	6.27E-06	C4B	protein_cc
ENSG0000(	1603.097	-0.03339	0.077703	-0.42974	0.667385	0.743298	RPS6KA1	protein_cc
ENSG0000(	1602.425	0.193957	0.03788	5.120358	3.05E-07	1.34E-06	CRIPT	protein_cc
ENSG0000(	1601.996	0.18586	0.042301	4.393713	1.11E-05	3.82E-05	STK38	protein_cc
ENSG0000(	1601.78	0.028406	0.113186	0.250967	0.801839	0.853607	LGI4	protein_cc
ENSG0000(	1601.611	0.075439	0.022729	3.319127	0.000903	0.002219	NIT1	protein_cc
ENSG0000(	1601.536	0.443666	0.063688	6.96622	3.26E-12	3.13E-11	CROT	protein_cc
ENSG0000(	1601.399	-0.04267	0.058712	-0.72673	0.467393	0.563254	TSPAN9	protein_cc
ENSG0000(	1600.502	0.029714	0.026014	1.142238	0.253355	0.34079	GEMIN5	protein_cc
ENSG0000(	1600.387	0.281734	0.037647	7.48359	7.23E-14	9.04E-13	RHNO1	protein_cc
ENSG0000(	1600.347	0.444887	0.035716	12.45627	1.29E-35	9.84E-33	ZNF558	protein_cc
ENSG0000(	1599.086	-0.14198	0.034052	-4.16948	3.05E-05	9.74E-05	ZBTB41	protein_cc
ENSG0000(	1598.091	0.092599	0.045795	2.022044	0.043172	0.0742	RAP2B	protein_cc
ENSG0000(	1597.459	-0.4457	0.12039	-3.70212	0.000214	0.00059	GABRA3	protein_cc
ENSG0000(	1596.27	-0.06168	0.047885	-1.28814	0.197697	0.276527	KIAA1958	protein_cc
ENSG0000(	1596.16	0.316953	0.047534	6.667935	2.59E-11	2.16E-10	FEN1	protein_cc
ENSG0000(	1595.546	-0.95747	0.096315	-9.94106	2.76E-23	1.73E-21	NAP1L2	protein_cc
ENSG0000(	1595.511	-0.12872	0.037879	-3.39826	0.000678	0.001706	ZSCAN29	protein_cc
ENSG0000(	1595.463	-0.0171	0.029668	-0.57622	0.564466	0.652768	PAPD7	protein_cc
ENSG0000(	1594.781	-0.23231	0.033499	-6.93488	4.07E-12	3.85E-11	CEP104	protein_cc
ENSG0000(	1594.293	-1.11033	0.114042	-9.73615	2.11E-22	1.10E-20	EGR3	protein_cc
ENSG0000(	1594.094	-0.31845	0.045461	-7.00499	2.47E-12	2.42E-11	ZNF33A	protein_cc
ENSG0000(	1594.091	0.173696	0.036092	4.81256	1.49E-06	5.87E-06	CHD1	protein_cc
ENSG0000(	1593.842	0.095313	0.073422	1.298159	0.194233	0.272336	EHD4	protein_cc
ENSG0000(	1593.762	-0.98411	0.090171	-10.9138	9.90E-28	1.44E-25	PNMAL2	protein_cc
ENSG0000(	1593.433	0.012413	0.036237	0.342556	0.731932	0.796759	PIGK	protein_cc
ENSG0000(	1592.858	-0.29645	0.043534	-6.8096	9.79E-12	8.71E-11	IKZF5	protein_cc
ENSG0000(	1592.015	-0.18763	0.032131	-5.83951	5.24E-09	3.04E-08	RNF123	protein_cc
ENSG0000(	1591.472	0.276225	0.059231	4.663495	3.11E-06	1.16E-05	CITED2	protein_cc

ENSG0000(	1591.467	-0.15122	0.033409	-4.52643	6.00E-06	2.15E-05	HPS5	protein_cc
ENSG0000(	1591.28	-0.17846	0.032514	-5.48871	4.05E-08	2.04E-07	DNAJC11	protein_cc
ENSG0000(	1589.365	-0.81183	0.087659	-9.26124	2.02E-20	7.35E-19	SCN1B	protein_cc
ENSG0000(	1589.357	0.123453	0.030081	4.104007	4.06E-05	0.000127	RP11-622	processed
ENSG0000(	1589.029	0.114829	0.023423	4.902409	9.47E-07	3.84E-06	SENP3	protein_cc
ENSG0000(	1588.727	0.063017	0.050415	1.249965	0.211312	0.292563	TAP2	protein_cc
ENSG0000(	1588.66	0.38119	0.043398	8.783598	1.58E-18	4.26E-17	FAM96A	protein_cc
ENSG0000(	1588.465	0.57113	0.057754	9.889014	4.65E-23	2.76E-21	C19orf48	protein_cc
ENSG0000(	1588.351	0.052506	0.028611	1.835122	0.066488	0.108338	ZNF777	protein_cc
ENSG0000(	1587.918	-0.27176	0.045311	-5.99772	2.00E-09	1.24E-08	RALGAPA:	protein_cc
ENSG0000(	1587.507	0.020263	0.039235	0.516461	0.605532	0.689689	SNAP23	protein_cc
ENSG0000(	1587.366	-0.2907	0.074273	-3.9139	9.08E-05	0.000267	PLCB2	protein_cc
ENSG0000(	1586.651	1.019896	0.106713	9.557414	1.21E-21	5.50E-20	TMEM158	protein_cc
ENSG0000(	1586.583	-0.0605	0.077521	-0.78049	0.435105	0.532173	TMTC2	protein_cc
ENSG0000(	1586.323	-0.12926	0.052039	-2.48379	0.012999	0.025295	DNMBP	protein_cc
ENSG0000(	1585.707	-1.39438	0.140052	-9.95618	2.37E-23	1.52E-21	KIAA1045	protein_cc
ENSG0000(	1584.951	0.056377	0.065473	0.861066	0.389202	0.485859	MYO1E	protein_cc
ENSG0000(	1584.108	0.573201	0.048931	11.71441	1.08E-31	3.33E-29	PSMG3	protein_cc
ENSG0000(	1584.081	0.39676	0.098568	4.025232	5.69E-05	0.000174	FZD7	protein_cc
ENSG0000(	1583.955	0.015569	0.061301	0.253975	0.799515	0.851904	PLCE1	protein_cc
ENSG0000(	1583.85	0.39929	0.108152	3.691932	0.000223	0.000612	MFAP4	protein_cc
ENSG0000(	1583.466	0.415987	0.076632	5.428345	5.69E-08	2.80E-07	IFI35	protein_cc
ENSG0000(	1583.126	-0.28735	0.030814	-9.3254	1.11E-20	4.23E-19	MNT	protein_cc
ENSG0000(	1582.947	0.065798	0.029641	2.219856	0.026429	0.0479	ANKRD54	protein_cc
ENSG0000(	1582.769	-0.07388	0.059256	-1.24675	0.212489	0.293876	MAPK11	protein_cc
ENSG0000(	1582.696	0.199727	0.105672	1.890068	0.058749	0.097266	SLC16A3	protein_cc
ENSG0000(	1582.319	0.094127	0.025031	3.760345	0.00017	0.000477	GTF3C3	protein_cc
ENSG0000(	1582.293	0.330435	0.139123	2.375121	0.017543	0.033085	AZGP1	protein_cc
ENSG0000(	1582.128	-0.90629	0.08312	-10.9033	1.11E-27	1.60E-25	ZNF365	protein_cc
ENSG0000(	1581.98	-0.2944	0.094947	-3.10068	0.001931	0.004465	H2AFY2	protein_cc
ENSG0000(	1581.661	-0.08998	0.035409	-2.54115	0.011049	0.02186	XRN1	protein_cc
ENSG0000(	1581.512	-0.20722	0.04426	-4.68193	2.84E-06	1.07E-05	ANKRD44	protein_cc
ENSG0000(	1581.009	0.23033	0.03536	6.513837	7.33E-11	5.70E-10	METTL17	protein_cc
ENSG0000(	1580.898	0.057659	0.031647	1.821938	0.068464	0.111132	UEVLD	protein_cc
ENSG0000(	1580.363	-0.2849	0.051843	-5.49529	3.90E-08	1.97E-07	SAMD14	protein_cc
ENSG0000(	1580.241	-0.40724	0.055187	-7.3794	1.59E-13	1.87E-12	SLC39A11	protein_cc
ENSG0000(	1578.323	-0.03439	0.02622	-1.31166	0.189633	0.266861	CSRP2BP	protein_cc
ENSG0000(	1578.169	0.085647	0.045387	1.887063	0.059152	0.097852	CYP4V2	protein_cc
ENSG0000(	1577.779	0.266121	0.037511	7.094495	1.30E-12	1.33E-11	DPH7	protein_cc
ENSG0000(	1577.665	-0.16793	0.03626	-4.63122	3.64E-06	1.35E-05	NFYA	protein_cc
ENSG0000(	1577.399	-0.00721	0.033493	-0.2153	0.829535	0.875409	PAN3	protein_cc
ENSG0000(	1576.926	-0.18359	0.035507	-5.17053	2.33E-07	1.04E-06	C3orf18	protein_cc
ENSG0000(	1576.644	-0.20704	0.045851	-4.51545	6.32E-06	2.25E-05	DENND6B	protein_cc
ENSG0000(	1576.401	-0.16192	0.066339	-2.44083	0.014653	0.028211	EPB41	protein_cc
ENSG0000(	1576.234	-0.35554	0.055295	-6.42982	1.28E-10	9.59E-10	PLA2G4C	protein_cc
ENSG0000(	1575.978	-0.57796	0.072096	-8.01657	1.09E-15	1.86E-14	ARHGEF37	protein_cc
ENSG0000(	1574.795	-0.76832	0.097912	-7.84706	4.26E-15	6.62E-14	SLC7A2	protein_cc
ENSG0000(	1574.439	-0.05707	0.052901	-1.07884	0.280661	0.371244	ARSA	protein_cc
ENSG0000(	1574.029	-0.09186	0.028179	-3.2597	0.001115	0.002693	DCAF10	protein_cc
ENSG0000(	1574.023	-0.35733	0.045775	-7.80635	5.89E-15	8.92E-14	TBC1D12	protein_cc
ENSG0000(	1573.723	-0.18009	0.05172	-3.48195	0.000498	0.001285	PCNXL2	protein_cc
ENSG0000(	1573.68	0.157082	0.064207	2.446472	0.014426	0.02782	PPP2R3B	protein_cc
ENSG0000(	1573.639	0.095331	0.042993	2.217359	0.026599	0.048174	SPATS2	protein_cc
ENSG0000(	1573.446	-0.01985	0.038361	-0.51756	0.604762	0.689002	LZIC	protein_cc
ENSG0000(	1573.264	-0.05947	0.060965	-0.97551	0.329305	0.423728	EPB41L4A	lincRNA
ENSG0000(	1572.649	0.044696	0.031436	1.421805	0.155083	0.225066	RAP2C	protein_cc
ENSG0000(	1572.605	-0.15538	0.029993	-5.18048	2.21E-07	9.92E-07	ZNHIT6	protein_cc
ENSG0000(	1572.553	-0.00326	0.056143	-0.05812	0.953651	0.967955	SHKBP1	protein_cc
ENSG0000(	1572.32	-0.22618	0.044118	-5.12674	2.95E-07	1.30E-06	CISD1	protein_cc

ENSG0000(	1572.106	-0.02009	0.044639	-0.44999	0.652715	0.731037	SLC15A4	protein_cc
ENSG0000(	1571.646	-0.10941	0.062425	-1.75265	0.079662	0.126811	RIN3	protein_cc
ENSG0000(	1571.296	-0.64612	0.100888	-6.40433	1.51E-10	1.12E-09	FOLR2	protein_cc
ENSG0000(	1569.833	0.029322	0.039584	0.740762	0.458838	0.555198	NFKB1	protein_cc
ENSG0000(	1569.777	-0.04239	0.146905	-0.28853	0.772937	0.830167	PCDHGB2	protein_cc
ENSG0000(	1568.891	0.126108	0.028796	4.37937	1.19E-05	4.06E-05	GSPT2	protein_cc
ENSG0000(	1568.612	0.267549	0.039085	6.845368	7.63E-12	6.90E-11	RIPK1	protein_cc
ENSG0000(	1568.339	0.250435	0.123613	2.025963	0.042769	0.073603	CFI	protein_cc
ENSG0000(	1568.251	-0.36569	0.042023	-8.7022	3.26E-18	8.40E-17	FBXO34	protein_cc
ENSG0000(	1568.205	-1.16898	0.1381	-8.46474	2.57E-17	5.79E-16	WBSCR17	protein_cc
ENSG0000(	1567.928	0.306011	0.051883	5.898071	3.68E-09	2.19E-08	C19orf60	protein_cc
ENSG0000(	1567.865	0.006068	0.037224	0.163013	0.870508	0.906987	SEC24A	protein_cc
ENSG0000(	1566.794	0.090954	0.035107	2.590753	0.009577	0.019216	GPANK1	protein_cc
ENSG0000(	1566.716	0.234652	0.02652	8.848062	8.91E-19	2.49E-17	MIOS	protein_cc
ENSG0000(	1566.132	0.061173	0.03031	2.018224	0.043568	0.074806	CHFR	protein_cc
ENSG0000(	1565.459	0.178577	0.035856	4.980347	6.35E-07	2.65E-06	S100PBP	protein_cc
ENSG0000(	1565.342	0.048359	0.060754	0.79597	0.42605	0.523152	PROX1	protein_cc
ENSG0000(	1564.761	-0.04198	0.063182	-0.66449	0.506378	0.600089	CROCC	protein_cc
ENSG0000(	1564.516	-0.86089	0.160467	-5.36488	8.10E-08	3.89E-07	GABRG2	protein_cc
ENSG0000(	1563.573	0.251867	0.06471	3.892265	9.93E-05	0.00029	MIF	protein_cc
ENSG0000(	1563.144	-0.63388	0.073957	-8.57091	1.03E-17	2.46E-16	DUSP8	protein_cc
ENSG0000(	1562.556	-0.30259	0.038213	-7.91841	2.41E-15	3.90E-14	RNF6	protein_cc
ENSG0000(	1561.075	0.089501	0.035182	2.543941	0.010961	0.021706	USPL1	protein_cc
ENSG0000(	1560.845	0.107102	0.033992	3.15082	0.001628	0.00382	THOC7	protein_cc
ENSG0000(	1560.712	-0.52158	0.079271	-6.57979	4.71E-11	3.77E-10	STON1	protein_cc
ENSG0000(	1560.5	-0.18553	0.122818	-1.51057	0.130897	0.194543	GPD1	protein_cc
ENSG0000(	1560.387	-0.43939	0.056864	-7.72709	1.10E-14	1.59E-13	FRA10AC1	protein_cc
ENSG0000(	1559.8	0.245343	0.063145	3.885355	0.000102	0.000298	HDAC9	protein_cc
ENSG0000(	1559.082	-0.13791	0.056895	-2.42401	0.01535	0.029393	C15orf39	protein_cc
ENSG0000(	1558.981	0.122979	0.041136	2.989583	0.002794	0.006261	RRAGC	protein_cc
ENSG0000(	1558.743	0.16164	0.035369	4.57007	4.88E-06	1.77E-05	LYRM4	protein_cc
ENSG0000(	1558.741	-0.0806	0.029307	-2.75018	0.005956	0.012483	GNPDA2	protein_cc
ENSG0000(	1558.718	0.143167	0.061694	2.320607	0.020308	0.037749	IL17RB	protein_cc
ENSG0000(	1558.636	0.150077	0.054349	2.761366	0.005756	0.012105	ANXA4	protein_cc
ENSG0000(	1558.452	-0.14089	0.03666	-3.84306	0.000122	0.00035	HIRA	protein_cc
ENSG0000(	1557.471	-0.2445	0.120604	-2.02728	0.042634	0.073411	HAPLN1	protein_cc
ENSG0000(	1557.242	-0.75658	0.079818	-9.47877	2.57E-21	1.10E-19	CNKSR2	protein_cc
ENSG0000(	1556.973	0.063762	0.02412	2.64352	0.008205	0.016724	TIMM21	protein_cc
ENSG0000(	1556.934	-0.96123	0.149697	-6.42119	1.35E-10	1.01E-09	FOSB	protein_cc
ENSG0000(	1556.92	0.374616	0.038118	9.827687	8.56E-23	4.81E-21	NUP107	protein_cc
ENSG0000(	1556.789	-0.08777	0.059129	-1.48442	0.137698	0.203197	RIC3	protein_cc
ENSG0000(	1556.532	-0.01157	0.037884	-0.3055	0.759982	0.819528	ZBTB5	protein_cc
ENSG0000(	1556.307	-0.12774	0.03792	-3.36876	0.000755	0.001881	XPO4	protein_cc
ENSG0000(	1555.434	0.110161	0.033233	3.314789	0.000917	0.00225	FAM118B	protein_cc
ENSG0000(	1555.152	-0.00835	0.030032	-0.27819	0.78087	0.836546	DPH1	protein_cc
ENSG0000(	1555.128	-0.21776	0.059374	-3.66759	0.000245	0.000669	PELI2	protein_cc
ENSG0000(	1554.291	0.116344	0.033377	3.485771	0.000491	0.001268	ACD	protein_cc
ENSG0000(	1553.503	-1.29791	0.131696	-9.85534	6.50E-23	3.78E-21	SLC8A2	protein_cc
ENSG0000(	1553.285	-0.05571	0.12174	-0.45762	0.647228	0.726313	FPR1	protein_cc
ENSG0000(	1553.195	-0.08413	0.028363	-2.96611	0.003016	0.006716	CRYZL1	protein_cc
ENSG0000(	1552.43	0.194976	0.033475	5.824514	5.73E-09	3.30E-08	COMMD5	protein_cc
ENSG0000(	1551.908	0.333685	0.040814	8.175823	2.94E-16	5.53E-15	TMEM179	protein_cc
ENSG0000(	1551.043	0.625435	0.084532	7.39879	1.37E-13	1.63E-12	NDUFA4L2	protein_cc
ENSG0000(	1550.057	0.232237	0.039207	5.923343	3.15E-09	1.90E-08	MRPL33	protein_cc
ENSG0000(	1549.709	-0.04744	0.027003	-1.757	0.078917	0.12579	ACTR8	protein_cc
ENSG0000(	1548.594	-0.16085	0.037977	-4.23543	2.28E-05	7.42E-05	CTC1	protein_cc
ENSG0000(	1548.184	0.06994	0.029846	2.343352	0.019111	0.035739	C11orf54	protein_cc
ENSG0000(	1548.041	0.194042	0.056248	3.449728	0.000561	0.001434	ROBO3	protein_cc
ENSG0000(	1548.013	-0.4738	0.098153	-4.8272	1.38E-06	5.48E-06	ADAMTS4	protein_cc

ENSG0000(	1547.927	0.097085	0.026553	3.656258	0.000256	0.000696	CSNK1G3	protein_cc
ENSG0000(	1547.795	-0.00892	0.026843	-0.33241	0.739581	0.80313	RAB28	protein_cc
ENSG0000(	1547.556	0.074483	0.054927	1.356044	0.175085	0.249513	AC005154	processed
ENSG0000(	1547.327	0.207396	0.031631	6.556727	5.50E-11	4.36E-10	NIT2	protein_cc
ENSG0000(	1546.914	0.237623	0.062056	3.82917	0.000129	0.000368	TGFB3	protein_cc
ENSG0000(	1546.511	0.094853	0.066828	1.419356	0.155795	0.225914	C1orf226	protein_cc
ENSG0000(	1546.136	-0.66577	0.081189	-8.20024	2.40E-16	4.59E-15	GUCY1B3	protein_cc
ENSG0000(	1546.054	0.216788	0.105037	2.063921	0.039025	0.067885	TM4SF1	protein_cc
ENSG0000(	1545.443	0.412913	0.083488	4.945778	7.58E-07	3.12E-06	HIST1H2A	protein_cc
ENSG0000(	1544.392	-1.02843	0.151162	-6.80346	1.02E-11	9.05E-11	SYT4	protein_cc
ENSG0000(	1542.847	-0.64095	0.116907	-5.48258	4.19E-08	2.11E-07	LY6H	protein_cc
ENSG0000(	1541.883	0.163724	0.026811	6.106594	1.02E-09	6.64E-09	BPNT1	protein_cc
ENSG0000(	1541.735	0.554987	0.094277	5.886784	3.94E-09	2.33E-08	SAMD9L	protein_cc
ENSG0000(	1541.255	-0.76282	0.127681	-5.97439	2.31E-09	1.42E-08	ADARB2	protein_cc
ENSG0000(	1541.195	-0.02826	0.044471	-0.63542	0.525153	0.617005	UAP1	protein_cc
ENSG0000(	1541.087	0.094853	0.024378	3.890963	9.98E-05	0.000292	ALG13	protein_cc
ENSG0000(	1540.992	0.186438	0.024806	7.515848	5.65E-14	7.17E-13	CYP20A1	protein_cc
ENSG0000(	1540.899	-0.05229	0.054514	-0.95917	0.337472	0.432315	STARD10	protein_cc
ENSG0000(	1540.417	0.013053	0.045755	0.285268	0.775439	0.832311	MBTD1	protein_cc
ENSG0000(	1539.737	0.13755	0.027848	4.939304	7.84E-07	3.22E-06	TOP3A	protein_cc
ENSG0000(	1539.533	-0.85795	0.118259	-7.25483	4.02E-13	4.44E-12	SHANK2	protein_cc
ENSG0000(	1539.523	-0.16863	0.032882	-5.12834	2.92E-07	1.29E-06	ANO10	protein_cc
ENSG0000(	1539.479	-0.36149	0.073761	-4.9008	9.54E-07	3.87E-06	FBN1	protein_cc
ENSG0000(	1538.845	0.138824	0.055036	2.522406	0.011655	0.022938	HEXDC	protein_cc
ENSG0000(	1538.569	0.03336	0.02645	1.261266	0.207213	0.287789	AIFM1	protein_cc
ENSG0000(	1538.209	0.194034	0.032152	6.034835	1.59E-09	1.00E-08	MRS2	protein_cc
ENSG0000(	1538.125	-0.14718	0.043165	-3.40964	0.00065	0.001643	CEP97	protein_cc
ENSG0000(	1537.969	-0.23103	0.0354	-6.52628	6.74E-11	5.28E-10	USP25	protein_cc
ENSG0000(	1537.75	0.152697	0.029993	5.091063	3.56E-07	1.54E-06	B4GALT7	protein_cc
ENSG0000(	1537.547	-0.18592	0.034565	-5.379	7.49E-08	3.62E-07	NHLRC2	protein_cc
ENSG0000(	1536.396	0.053195	0.046168	1.152203	0.249238	0.336098	LRRCC1	protein_cc
ENSG0000(	1536.321	0.356744	0.097108	3.673677	0.000239	0.000654	LAMA2	protein_cc
ENSG0000(	1536.283	0.217252	0.040656	5.343599	9.11E-08	4.34E-07	ZNF680	protein_cc
ENSG0000(	1536.078	0.132438	0.030675	4.317383	1.58E-05	5.28E-05	ATG5	protein_cc
ENSG0000(	1536.066	0.20905	0.075099	2.783672	0.005375	0.011385	EMID1	protein_cc
ENSG0000(	1535.769	0.383412	0.053256	7.199441	6.05E-13	6.49E-12	LEPREL4	protein_cc
ENSG0000(	1535.733	-0.13846	0.074708	-1.85336	0.06383	0.104563	SSX2IP	protein_cc
ENSG0000(	1535.282	-0.0264	0.028519	-0.92562	0.354641	0.450229	AC112218	protein_cc
ENSG0000(	1535.279	-0.13356	0.095253	-1.40218	0.160861	0.232106	GLDN	protein_cc
ENSG0000(	1534.851	-0.35993	0.051897	-6.9354	4.05E-12	3.83E-11	KDM7A	protein_cc
ENSG0000(	1534.565	-0.38398	0.100529	-3.81957	0.000134	0.000382	COLEC12	protein_cc
ENSG0000(	1532.547	0.443196	0.093925	4.718603	2.37E-06	9.05E-06	LPHN2	protein_cc
ENSG0000(	1532.143	-0.96862	0.105843	-9.15149	5.62E-20	1.92E-18	DYNC1I1	protein_cc
ENSG0000(	1531.698	-0.50343	0.087021	-5.78519	7.24E-09	4.11E-08	TCEAL5	protein_cc
ENSG0000(	1531.682	0.081187	0.062783	1.29314	0.195963	0.274472	DOCK6	protein_cc
ENSG0000(	1531.476	0.148048	0.026204	5.649737	1.61E-08	8.63E-08	TTI1	protein_cc
ENSG0000(	1531.254	-1.04951	0.158075	-6.63928	3.15E-11	2.60E-10	MYT1L	protein_cc
ENSG0000(	1531.093	0.156599	0.021053	7.438293	1.02E-13	1.24E-12	CSTF1	protein_cc
ENSG0000(	1530.843	0.165444	0.045832	3.609808	0.000306	0.000823	SMIM12	protein_cc
ENSG0000(	1530.318	0.291837	0.028765	10.1456	3.47E-24	2.60E-22	ALG8	protein_cc
ENSG0000(	1529.082	-0.0611	0.040372	-1.51354	0.130144	0.193542	PARP8	protein_cc
ENSG0000(	1528.922	0.04723	0.028381	1.664156	0.096081	0.149216	GRK6	protein_cc
ENSG0000(	1528.63	0.042908	0.026233	1.635646	0.101914	0.157064	BTN2A1	protein_cc
ENSG0000(	1528.503	-0.11849	0.08752	-1.35384	0.175786	0.250335	SLCO1C1	protein_cc
ENSG0000(	1528.467	-0.36265	0.072328	-5.01393	5.33E-07	2.25E-06	RAPGEF3	protein_cc
ENSG0000(	1527.867	-0.10465	0.065498	-1.59781	0.110085	0.167851	SEMA6C	protein_cc
ENSG0000(	1527.197	-0.20275	0.090197	-2.24787	0.024584	0.044858	PLD4	protein_cc
ENSG0000(	1526.725	0.143359	0.024633	5.819746	5.89E-09	3.39E-08	CNOT11	protein_cc
ENSG0000(	1525.885	0.11829	0.030051	3.936367	8.27E-05	0.000245	ATP6V0A2	protein_cc

ENSG0000(	1525.871	0.056387	0.025041	2.251785	0.024336	0.044455	ZNF513	protein_cc
ENSG0000(	1525.564	-0.47839	0.151376	-3.16026	0.001576	0.003708	VSTM2A	protein_cc
ENSG0000(	1525.069	-0.02646	0.036512	-0.72459	0.468702	0.564553	C6orf120	protein_cc
ENSG0000(	1524.642	-0.36396	0.069187	-5.26049	1.44E-07	6.63E-07	SULT1C4	protein_cc
ENSG0000(	1524.278	0.187247	0.028422	6.588003	4.46E-11	3.59E-10	FAM220A	protein_cc
ENSG0000(	1523.928	-0.1699	0.032201	-5.27619	1.32E-07	6.13E-07	KIAA1468	protein_cc
ENSG0000(	1523.775	-0.10735	0.118852	-0.90318	0.36643	0.462497	HLA-DOA	protein_cc
ENSG0000(	1523.694	0.104471	0.067404	1.549918	0.121161	0.182114	SLC12A7	protein_cc
ENSG0000(	1523.286	0.073739	0.03649	2.020795	0.043301	0.074398	RHBDD3	protein_cc
ENSG0000(	1523.202	-0.0014	0.030508	-0.04582	0.963456	0.97505	FECH	protein_cc
ENSG0000(	1522.841	0.556811	0.084614	6.580576	4.69E-11	3.76E-10	PSMB9	protein_cc
ENSG0000(	1522.645	0.143706	0.034671	4.14488	3.40E-05	0.000108	SGSH	protein_cc
ENSG0000(	1522.08	-0.00189	0.033919	-0.05575	0.955542	0.969304	ZNF160	protein_cc
ENSG0000(	1521.687	0.309147	0.04614	6.700244	2.08E-11	1.76E-10	CNIH4	protein_cc
ENSG0000(	1520.299	0.005081	0.02826	0.179813	0.8573	0.896971	OARD1	protein_cc
ENSG0000(	1520.14	-0.06369	0.039526	-1.61135	0.107104	0.163927	ZNF91	protein_cc
ENSG0000(	1520.043	-0.13012	0.077816	-1.67219	0.094486	0.147078	MYO1F	protein_cc
ENSG0000(	1520.003	0.089575	0.041219	2.173142	0.02977	0.053284	ANKS6	protein_cc
ENSG0000(	1519.816	-0.02151	0.029065	-0.74018	0.45919	0.555452	IPO11	protein_cc
ENSG0000(	1518.364	0.038698	0.030929	1.251182	0.210868	0.292027	SLAIN2	protein_cc
ENSG0000(	1518.041	-0.11599	0.062735	-1.84887	0.064476	0.105487	TMEM145	protein_cc
ENSG0000(	1517.656	0.248477	0.032019	7.760289	8.47E-15	1.25E-13	PCID2	protein_cc
ENSG0000(	1517.401	0.288681	0.027526	10.48769	9.84E-26	1.01E-23	RNF25	protein_cc
ENSG0000(	1516.891	-0.13834	0.040339	-3.42939	0.000605	0.001536	MBD5	protein_cc
ENSG0000(	1516.404	0.299927	0.050737	5.911388	3.39E-09	2.03E-08	POLR2I	protein_cc
ENSG0000(	1515.982	-0.25667	0.029912	-8.58088	9.41E-18	2.27E-16	MAP3K3	protein_cc
ENSG0000(	1514.648	0.330634	0.034599	9.556088	1.22E-21	5.55E-20	TMEM161	protein_cc
ENSG0000(	1514.57	-0.01625	0.038543	-0.42172	0.673229	0.748004	PREP	protein_cc
ENSG0000(	1514.369	-0.29102	0.045764	-6.35913	2.03E-10	1.47E-09	IFFO2	protein_cc
ENSG0000(	1514.327	-0.06252	0.032246	-1.93876	0.052531	0.088236	ARRDC1	protein_cc
ENSG0000(	1514.026	-0.32858	0.046453	-7.07329	1.51E-12	1.53E-11	CNST	protein_cc
ENSG0000(	1514.011	-0.11207	0.077296	-1.44982	0.147109	0.215124	RGS10	protein_cc
ENSG0000(	1513.719	-0.28757	0.077325	-3.71896	0.0002	0.000555	TMEM35	protein_cc
ENSG0000(	1512.981	0.092289	0.044057	2.094769	0.036192	0.063504	ARMC5	protein_cc
ENSG0000(	1512.37	-0.19748	0.054421	-3.62873	0.000285	0.00077	PTPN21	protein_cc
ENSG0000(	1512.296	-0.215	0.0521	-4.12669	3.68E-05	0.000116	MKNK1	protein_cc
ENSG0000(	1512.259	0.161613	0.028142	5.742821	9.31E-09	5.18E-08	EXOSC2	protein_cc
ENSG0000(	1512.203	0.204743	0.041565	4.925809	8.40E-07	3.43E-06	PHYKPL	protein_cc
ENSG0000(	1511.926	0.363394	0.041989	8.654556	4.95E-18	1.24E-16	TMEM209	protein_cc
ENSG0000(	1511.827	-0.0853	0.058644	-1.45446	0.145819	0.213503	SLC9A1	protein_cc
ENSG0000(	1510.381	-0.00458	0.030361	-0.15088	0.880074	0.914267	MTRR	protein_cc
ENSG0000(	1510.173	0.168303	0.026455	6.361859	1.99E-10	1.45E-09	SMUG1	protein_cc
ENSG0000(	1509.501	0.09435	0.031356	3.009011	0.002621	0.005914	CHMP6	protein_cc
ENSG0000(	1508.923	0.330814	0.076414	4.329248	1.50E-05	5.02E-05	MYD88	protein_cc
ENSG0000(	1508.677	0.312501	0.041857	7.465925	8.27E-14	1.02E-12	CEP89	protein_cc
ENSG0000(	1508.364	-0.16238	0.053591	-3.02996	0.002446	0.005556	RASL10B	protein_cc
ENSG0000(	1507.985	0.028302	0.038041	0.743977	0.456891	0.553322	TXNRD2	protein_cc
ENSG0000(	1507.653	-0.27191	0.083341	-3.26266	0.001104	0.002669	LRRTM3	protein_cc
ENSG0000(	1505.615	-0.75969	0.077822	-9.76195	1.64E-22	8.72E-21	PRRT1	protein_cc
ENSG0000(	1505.123	0.007213	0.041931	0.172021	0.863421	0.901659	HEATR1	protein_cc
ENSG0000(	1504.675	-0.00118	0.030195	-0.039	0.968892	0.978444	PGAP3	protein_cc
ENSG0000(	1504.602	0.374053	0.056519	6.618141	3.64E-11	2.97E-10	ATAD2	protein_cc
ENSG0000(	1504.512	-0.106	0.052126	-2.03351	0.042001	0.072452	PYGO1	protein_cc
ENSG0000(	1504.324	-0.36341	0.065664	-5.53435	3.12E-08	1.60E-07	PPP1R3C	protein_cc
ENSG0000(	1503.405	-0.03394	0.033788	-1.00461	0.315086	0.40864	UBIAD1	protein_cc
ENSG0000(	1502.988	0.074104	0.0427	1.735443	0.082662	0.130842	C11orf24	protein_cc
ENSG0000(	1502.935	-0.01451	0.028748	-0.5048	0.6137	0.69708	XYLT2	protein_cc
ENSG0000(	1502.892	0.315167	0.062469	5.045154	4.53E-07	1.93E-06	EPHB3	protein_cc
ENSG0000(	1502.887	0.004569	0.043585	0.104833	0.916509	0.941157	TMEM14A	protein_cc

ENSG0000(	1502.598	0.019779	0.024571	0.804999	0.42082	0.517812	RIC8B	protein_cc
ENSG0000(	1502.085	-0.03426	0.030134	-1.13684	0.255607	0.343269	ACOT13	protein_cc
ENSG0000(	1501.923	0.137766	0.047122	2.923609	0.00346	0.007618	RP11-212	lincRNA
ENSG0000(	1501.282	0.210086	0.04262	4.929223	8.26E-07	3.38E-06	MRPL17	protein_cc
ENSG0000(	1501.13	0.097564	0.02984	3.269615	0.001077	0.002609	ACAD10	protein_cc
ENSG0000(	1501.015	0.158993	0.057576	2.761438	0.005755	0.012103	MID1	protein_cc
ENSG0000(	1501.007	0.07105	0.044336	1.602522	0.10904	0.166444	LMF1	protein_cc
ENSG0000(	1500.804	0.062487	0.036447	1.714449	0.086446	0.136166	ZNF195	protein_cc
ENSG0000(	1500.604	0.118344	0.034082	3.472354	0.000516	0.001328	FLYWCH2	protein_cc
ENSG0000(	1500.427	-0.10127	0.030163	-3.35744	0.000787	0.001955	ATG7	protein_cc
ENSG0000(	1499.899	0.049179	0.039015	1.260515	0.207484	0.288126	SCFD2	protein_cc
ENSG0000(	1499.631	-0.23257	0.042899	-5.42121	5.92E-08	2.91E-07	TRIQK	protein_cc
ENSG0000(	1499.537	-0.29884	0.066454	-4.49694	6.89E-06	2.44E-05	ACVR2B	protein_cc
ENSG0000(	1499.305	-0.0318	0.031921	-0.99635	0.319081	0.412806	EARS2	protein_cc
ENSG0000(	1499.026	0.259588	0.06949	3.735643	0.000187	0.000522	HIST2H2B	protein_cc
ENSG0000(	1498.539	0.472466	0.097287	4.856409	1.20E-06	4.77E-06	PTGS1	protein_cc
ENSG0000(	1497.561	0.137407	0.065481	2.098412	0.035869	0.063002	GMNN	protein_cc
ENSG0000(	1497.022	0.036077	0.035392	1.019343	0.30804	0.401122	TRMT61A	protein_cc
ENSG0000(	1496.921	-0.38274	0.048697	-7.85956	3.85E-15	6.06E-14	AGTPBP1	protein_cc
ENSG0000(	1496.076	0.045833	0.029141	1.572799	0.115765	0.175349	MSL3	protein_cc
ENSG0000(	1496.074	-0.23664	0.070074	-3.37696	0.000733	0.001831	TBC1D10A	protein_cc
ENSG0000(	1495.911	0.36557	0.04279	8.543387	1.30E-17	3.07E-16	PDRG1	protein_cc
ENSG0000(	1495.793	-0.23832	0.02972	-8.01886	1.07E-15	1.83E-14	FIG4	protein_cc
ENSG0000(	1495.368	-0.03924	0.030174	-1.30045	0.193446	0.271435	JMJD6	protein_cc
ENSG0000(	1495.044	-0.02348	0.051072	-0.45972	0.645717	0.725096	APBB3	protein_cc
ENSG0000(	1494.958	0.20886	0.031322	6.668144	2.59E-11	2.16E-10	CHST12	protein_cc
ENSG0000(	1494.181	0.130411	0.031339	4.161361	3.16E-05	0.000101	DPAGT1	protein_cc
ENSG0000(	1493.888	0.010847	0.047343	0.229122	0.818774	0.867145	SESN2	protein_cc
ENSG0000(	1493.717	0.451505	0.051922	8.695818	3.44E-18	8.86E-17	LIG1	protein_cc
ENSG0000(	1493.53	0.114614	0.026002	4.407957	1.04E-05	3.59E-05	MRPL44	protein_cc
ENSG0000(	1493.024	-0.00101	0.035335	-0.02847	0.977284	0.983956	TRPM7	protein_cc
ENSG0000(	1492.933	-0.35592	0.06264	-5.68194	1.33E-08	7.25E-08	ABCB1	protein_cc
ENSG0000(	1492.616	0.046998	0.090101	0.52161	0.601942	0.686436	GOLGA8B	protein_cc
ENSG0000(	1492.298	0.023931	0.031969	0.748581	0.45411	0.550684	TAF5L	protein_cc
ENSG0000(	1492.297	0.303096	0.040345	7.512642	5.79E-14	7.34E-13	MSRB1	protein_cc
ENSG0000(	1492.143	0.205323	0.032776	6.26451	3.74E-10	2.60E-09	TMEM101	protein_cc
ENSG0000(	1491.983	0.202134	0.069071	2.926471	0.003428	0.007555	ATF5	protein_cc
ENSG0000(	1491.931	0.636464	0.068597	9.278346	1.72E-20	6.34E-19	TIMELESS	protein_cc
ENSG0000(	1491.242	0.141891	0.024898	5.698976	1.21E-08	6.60E-08	ELF2	protein_cc
ENSG0000(	1491.053	0.143742	0.032337	4.445098	8.79E-06	3.06E-05	NFU1	protein_cc
ENSG0000(	1490.779	0.116532	0.069359	1.680141	0.09293	0.144979	ITPKC	protein_cc
ENSG0000(	1490.762	-0.26695	0.052439	-5.09061	3.57E-07	1.55E-06	FOCAD	protein_cc
ENSG0000(	1490.7	0.124726	0.026571	4.694078	2.68E-06	1.01E-05	AL034548	pseudoge
ENSG0000(	1490.139	0.032521	0.026201	1.241248	0.214514	0.296248	RANBP10	protein_cc
ENSG0000(	1489.783	0.029044	0.060989	0.476221	0.633917	0.714793	D2HGDH	protein_cc
ENSG0000(	1488.665	0.266979	0.047644	5.603663	2.10E-08	1.11E-07	MRPL34	protein_cc
ENSG0000(	1488.51	-0.78702	0.125657	-6.26325	3.77E-10	2.62E-09	SLC24A4	protein_cc
ENSG0000(	1488.086	0.185042	0.041254	4.485395	7.28E-06	2.57E-05	GRWD1	protein_cc
ENSG0000(	1486.645	-0.06767	0.034445	-1.96457	0.049464	0.083694	ZC3H3	protein_cc
ENSG0000(	1486.614	-0.00155	0.033708	-0.04597	0.963333	0.974974	ZNF264	protein_cc
ENSG0000(	1486.579	-0.7634	0.125165	-6.09916	1.07E-09	6.93E-09	IGSF1	protein_cc
ENSG0000(	1486.101	0.152412	0.030131	5.058406	4.23E-07	1.81E-06	CCDC137	protein_cc
ENSG0000(	1485.95	-0.04749	0.035444	-1.33987	0.180286	0.255588	MED23	protein_cc
ENSG0000(	1485.946	-0.23148	0.042296	-5.4729	4.43E-08	2.22E-07	ZFHX3	protein_cc
ENSG0000(	1485.5	0.013879	0.040277	0.344586	0.730406	0.795456	SENP7	protein_cc
ENSG0000(	1485.479	-0.21588	0.04776	-4.52023	6.18E-06	2.21E-05	SIRT1	protein_cc
ENSG0000(	1485.19	0.419646	0.042105	9.966558	2.14E-23	1.38E-21	MRPL32	protein_cc
ENSG0000(	1485.033	0.127186	0.026775	4.750176	2.03E-06	7.82E-06	C12orf65	protein_cc
ENSG0000(	1484.326	-0.01181	0.026863	-0.4395	0.660297	0.737281	PRKCI	protein_cc

ENSG0000(	1484.236	-0.00032	0.023598	-0.01374	0.989037	0.992391	ZDHHC6	protein_cc
ENSG0000(	1483.323	0.495636	0.088971	5.570741	2.54E-08	1.32E-07	SPATA6	protein_cc
ENSG0000(	1482.969	0.029353	0.029563	0.992884	0.320767	0.414583	DHX37	protein_cc
ENSG0000(	1482.884	-0.10398	0.037769	-2.75311	0.005903	0.012386	LRIG2	protein_cc
ENSG0000(	1482.733	0.32277	0.069907	4.617117	3.89E-06	1.43E-05	FNDC3B	protein_cc
ENSG0000(	1482.459	-0.20113	0.041333	-4.86612	1.14E-06	4.56E-06	MTM1	protein_cc
ENSG0000(	1482.273	-0.48658	0.049521	-9.82562	8.73E-23	4.88E-21	FAM73A	protein_cc
ENSG0000(	1481.21	0.064984	0.027565	2.357449	0.018401	0.034544	LINC0084	lincRNA
ENSG0000(	1480.716	-0.40063	0.070665	-5.66937	1.43E-08	7.76E-08	B4GALT1	protein_cc
ENSG0000(	1480.156	0.149435	0.072839	2.051577	0.040211	0.06972	KIRREL	protein_cc
ENSG0000(	1480.072	-0.12098	0.027238	-4.44157	8.93E-06	3.11E-05	AFTPH	protein_cc
ENSG0000(	1480.065	-0.17252	0.089322	-1.93149	0.053422	0.089547	SIGLEC10	protein_cc
ENSG0000(	1479.563	-0.37231	0.171992	-2.16469	0.030411	0.054307	PCDHGA1	protein_cc
ENSG0000(	1479.417	0.339952	0.04505	7.546123	4.48E-14	5.79E-13	PRADC1	protein_cc
ENSG0000(	1479.105	-0.02716	0.048059	-0.56513	0.571986	0.659462	ANKRD50	protein_cc
ENSG0000(	1478.823	0.009791	0.029917	0.327259	0.743472	0.806418	ERLIN1	protein_cc
ENSG0000(	1478.708	-0.99226	0.105057	-9.44502	3.55E-21	1.49E-19	PARM1	protein_cc
ENSG0000(	1478.451	0.059106	0.026675	2.21579	0.026706	0.048346	METAP1	protein_cc
ENSG0000(	1478.145	-0.19382	0.05219	-3.71379	0.000204	0.000565	VPS13A	protein_cc
ENSG0000(	1478.086	-0.27618	0.040804	-6.76845	1.30E-11	1.13E-10	PDP2	protein_cc
ENSG0000(	1477.659	-0.27033	0.045821	-5.89968	3.64E-09	2.17E-08	ADCY9	protein_cc
ENSG0000(	1477.55	-0.03432	0.034445	-0.99624	0.319133	0.412848	TAF2	protein_cc
ENSG0000(	1476.853	0.251155	0.032263	7.784531	7.00E-15	1.05E-13	IER3IP1	protein_cc
ENSG0000(	1476.648	0.108292	0.109624	0.987855	0.323224	0.417379	FBLN5	protein_cc
ENSG0000(	1476.514	-0.78925	0.079332	-9.94873	2.55E-23	1.62E-21	THRB	protein_cc
ENSG0000(	1476.32	-0.54821	0.05657	-9.69082	3.30E-22	1.66E-20	PLEKHA1	protein_cc
ENSG0000(	1476.275	-0.15465	0.066685	-2.31909	0.02039	0.037881	HPS1	protein_cc
ENSG0000(	1475.847	0.069587	0.034573	2.012738	0.044142	0.075659	FCHO2	protein_cc
ENSG0000(	1475.603	-0.9637	0.13645	-7.06266	1.63E-12	1.64E-11	IQSEC3	protein_cc
ENSG0000(	1475.299	0.00848	0.026695	0.317652	0.750749	0.812233	PDCL	protein_cc
ENSG0000(	1474.945	0.010816	0.043678	0.247642	0.804411	0.855759	PHC1	protein_cc
ENSG0000(	1474.594	0.155923	0.062233	2.505463	0.012229	0.023946	PIDD	protein_cc
ENSG0000(	1474.043	-0.46782	0.046498	-10.0611	8.21E-24	5.78E-22	INPP5A	protein_cc
ENSG0000(	1473.953	-0.15302	0.047768	-3.20347	0.001358	0.003231	ARL5B	protein_cc
ENSG0000(	1473.92	0.422978	0.049049	8.623649	6.49E-18	1.60E-16	ZFAND2A	protein_cc
ENSG0000(	1471.574	-0.13802	0.042457	-3.25083	0.001151	0.002771	HSD11B1L	protein_cc
ENSG0000(	1471.443	-0.16156	0.036572	-4.4176	9.98E-06	3.45E-05	FAN1	protein_cc
ENSG0000(	1471.083	-0.04533	0.026509	-1.71008	0.087251	0.13727	BRPF1	protein_cc
ENSG0000(	1470.981	-1.0204	0.138742	-7.35465	1.91E-13	2.22E-12	KIAA1644	protein_cc
ENSG0000(	1470.945	-0.12778	0.029201	-4.37586	1.21E-05	4.12E-05	SEC23IP	protein_cc
ENSG0000(	1470.667	-0.46616	0.058612	-7.95335	1.82E-15	3.01E-14	LPCAT4	protein_cc
ENSG0000(	1470.244	-0.2011	0.038195	-5.26501	1.40E-07	6.48E-07	PCCA	protein_cc
ENSG0000(	1470.097	-0.05406	0.031357	-1.72387	0.084732	0.133762	MTIF3	protein_cc
ENSG0000(	1469.888	0.083115	0.040261	2.064402	0.03898	0.067812	CAPN15	protein_cc
ENSG0000(	1469.854	0.263935	0.062224	4.241723	2.22E-05	7.23E-05	EFNA1	protein_cc
ENSG0000(	1469.679	-0.21213	0.044913	-4.7231	2.32E-06	8.86E-06	MBIP	protein_cc
ENSG0000(	1469.528	-0.22174	0.035319	-6.27823	3.42E-10	2.40E-09	RBMXL1	protein_cc
ENSG0000(	1468.676	-0.43346	0.081733	-5.30333	1.14E-07	5.34E-07	KCNN2	protein_cc
ENSG0000(	1468.577	0.075097	0.029425	2.552179	0.010705	0.021244	ACOT8	protein_cc
ENSG0000(	1468.053	-0.03625	0.033157	-1.09317	0.27432	0.36433	HCFC2	protein_cc
ENSG0000(	1467.79	0.243429	0.142986	1.702467	0.088668	0.139232	CXXC11	protein_cc
ENSG0000(	1466.984	-0.25313	0.042421	-5.96699	2.42E-09	1.48E-08	IDH3A	protein_cc
ENSG0000(	1466.354	0.032361	0.087553	0.369621	0.711665	0.780108	LRRK2	protein_cc
ENSG0000(	1465.892	0.724679	0.117897	6.146723	7.91E-10	5.24E-09	OAS1	protein_cc
ENSG0000(	1465.482	-0.88207	0.136378	-6.46782	9.94E-11	7.58E-10	BCYRN1	lincRNA
ENSG0000(	1465.259	0.030816	0.030012	1.026767	0.30453	0.397399	ZNF622	protein_cc
ENSG0000(	1465.13	-0.55806	0.071927	-7.75867	8.58E-15	1.27E-13	SLC7A8	protein_cc
ENSG0000(	1465.117	-0.44438	0.090368	-4.91746	8.77E-07	3.57E-06	ELMOD1	protein_cc
ENSG0000(	1464.952	0.123978	0.031867	3.890551	0.0001	0.000292	KATNBL1	protein_cc

ENSG0000(	1463.681	-0.20095	0.080227	-2.50481	0.012252	0.023985	WNT5A	protein_cc
ENSG0000(	1463.286	0.033733	0.038248	0.881967	0.377795	0.474319	ST3GAL3	protein_cc
ENSG0000(	1462.681	0.242277	0.064345	3.765285	0.000166	0.000468	UBXN11	protein_cc
ENSG0000(	1462.529	0.233676	0.035144	6.649057	2.95E-11	2.44E-10	YIPF2	protein_cc
ENSG0000(	1462.436	0.235114	0.09583	2.453447	0.014149	0.027329	TNFAIP2	protein_cc
ENSG0000(	1462.367	-0.12118	0.03402	-3.56208	0.000368	0.000975	PLAA	protein_cc
ENSG0000(	1462.362	-1.55986	0.138868	-11.2327	2.82E-29	5.55E-27	GRIN2A	protein_cc
ENSG0000(	1462.236	-0.43293	0.045511	-9.51261	1.86E-21	8.23E-20	ZNF710	protein_cc
ENSG0000(	1461.858	0.17061	0.10727	1.590469	0.111729	0.170044	PCDHGC4	protein_cc
ENSG0000(	1460.549	-0.43339	0.076687	-5.65136	1.59E-08	8.56E-08	RNF182	protein_cc
ENSG0000(	1460.432	0.753863	0.104985	7.180666	6.94E-13	7.40E-12	HILPDA	protein_cc
ENSG0000(	1459.72	0.112862	0.036531	3.089474	0.002005	0.004625	NSDHL	protein_cc
ENSG0000(	1459.603	0.29428	0.104086	2.827283	0.004694	0.010052	CHST6	protein_cc
ENSG0000(	1459.46	-0.41121	0.052227	-7.87351	3.45E-15	5.46E-14	C2CD2	protein_cc
ENSG0000(	1459.215	-0.06986	0.033577	-2.08059	0.037472	0.06549	ZNF721	protein_cc
ENSG0000(	1459.185	-0.42958	0.224234	-1.91578	0.055394	0.092433	KDM5D	protein_cc
ENSG0000(	1459.019	0.162532	0.048069	3.381219	0.000722	0.001806	COX14	protein_cc
ENSG0000(	1458.858	-0.05169	0.112621	-0.45898	0.64625	0.725477	SRRM3	protein_cc
ENSG0000(	1458.769	-0.04826	0.030368	-1.58912	0.112032	0.170398	ZNRF1	protein_cc
ENSG0000(	1456.149	0.218195	0.033953	6.426358	1.31E-10	9.79E-10	RPS6KB2	protein_cc
ENSG0000(	1456.07	0.076602	0.046938	1.632001	0.102679	0.158078	TRABD	protein_cc
ENSG0000(	1455.734	-0.68654	0.109525	-6.26839	3.65E-10	2.54E-09	TMEM151	protein_cc
ENSG0000(	1455.63	0.065823	0.047295	1.391762	0.163994	0.236064	NFKBIB	protein_cc
ENSG0000(	1455.577	-0.08337	0.036418	-2.28939	0.022057	0.040684	STRBP	protein_cc
ENSG0000(	1455.003	-0.03016	0.059782	-0.50452	0.613895	0.697205	DPF1	protein_cc
ENSG0000(	1454.961	-0.18984	0.10612	-1.78893	0.073625	0.118423	ZBTB16	protein_cc
ENSG0000(	1454.615	0.120939	0.038894	3.109489	0.001874	0.004346	LYRM1	protein_cc
ENSG0000(	1453.202	0.105069	0.031291	3.357814	0.000786	0.001952	EEFSEC	protein_cc
ENSG0000(	1453.159	-0.03592	0.037892	-0.94798	0.343141	0.438349	MANBA	protein_cc
ENSG0000(	1452.614	-0.02488	0.033672	-0.73901	0.459903	0.556151	RABL3	protein_cc
ENSG0000(	1452.421	0.32078	0.035787	8.963469	3.15E-19	9.46E-18	MRPL47	protein_cc
ENSG0000(	1452.276	0.136801	0.030952	4.419798	9.88E-06	3.41E-05	DOLK	protein_cc
ENSG0000(	1452.037	0.153582	0.03003	5.114361	3.15E-07	1.38E-06	DOLPP1	protein_cc
ENSG0000(	1452.007	-0.00081	0.043112	-0.01871	0.98507	0.989763	AGL	protein_cc
ENSG0000(	1451.956	-0.10659	0.026575	-4.01098	6.05E-05	0.000184	ANKRD13(	protein_cc
ENSG0000(	1451.679	-0.16038	0.042508	-3.77303	0.000161	0.000455	KLHDC8B	protein_cc
ENSG0000(	1451.13	0.137715	0.047249	2.914661	0.003561	0.007821	ETV6	protein_cc
ENSG0000(	1451.014	-0.03496	0.029245	-1.19539	0.231933	0.31609	COG3	protein_cc
ENSG0000(	1450.36	-0.30473	0.057009	-5.3452	9.03E-08	4.31E-07	NXN	protein_cc
ENSG0000(	1449.737	-0.20297	0.057699	-3.51772	0.000435	0.001137	CCBL1	protein_cc
ENSG0000(	1449.046	1.221901	0.085811	14.23942	5.22E-46	1.43E-42	GAS1	protein_cc
ENSG0000(	1448.677	-0.01088	0.038712	-0.28103	0.778684	0.834857	HMGXB4	protein_cc
ENSG0000(	1447.696	0.103791	0.02664	3.89612	9.77E-05	0.000286	UBXN2A	protein_cc
ENSG0000(	1447.157	-0.99005	0.109817	-9.01544	1.96E-19	6.11E-18	GRM3	protein_cc
ENSG0000(	1446.572	-0.66141	0.102415	-6.45808	1.06E-10	8.06E-10	GNAL	protein_cc
ENSG0000(	1446.539	0.004269	0.056068	0.076146	0.939303	0.957654	CORO7	protein_cc
ENSG0000(	1446.396	-0.65855	0.133405	-4.93643	7.96E-07	3.26E-06	COL11A1	protein_cc
ENSG0000(	1446.345	0.230286	0.037429	6.152565	7.62E-10	5.07E-09	ZNF121	protein_cc
ENSG0000(	1446.023	0.322984	0.054778	5.896286	3.72E-09	2.21E-08	NDE1	protein_cc
ENSG0000(	1445.51	-0.12035	0.040715	-2.95604	0.003116	0.006921	ABHD17B	protein_cc
ENSG0000(	1444.966	0.146115	0.042255	3.457963	0.000544	0.001395	RBBP5	protein_cc
ENSG0000(	1444.939	-0.35866	0.088374	-4.05844	4.94E-05	0.000152	FYB	protein_cc
ENSG0000(	1444.814	0.041104	0.033408	1.230375	0.218557	0.300881	GPALPP1	protein_cc
ENSG0000(	1444.794	-0.24818	0.05385	-4.60876	4.05E-06	1.49E-05	DYNC2H1	protein_cc
ENSG0000(	1444.319	0.127909	0.04708	2.716834	0.006591	0.013691	MZT2A	protein_cc
ENSG0000(	1443.973	0.962032	0.188278	5.10964	3.23E-07	1.41E-06	ABCC3	protein_cc
ENSG0000(	1443.598	-0.43584	0.103983	-4.1915	2.77E-05	8.90E-05	CHRNB2	protein_cc
ENSG0000(	1443.528	-0.27872	0.078243	-3.56224	0.000368	0.000974	MYO15B	protein_cc
ENSG0000(	1442.832	0.018149	0.018325	0.990424	0.321967	0.41599	MTIF2	protein_cc

ENSG0000(	1442.219	0.080532	0.034116	2.360553	0.018248	0.034286	INSIG2	protein_cc
ENSG0000(	1442.212	0.19418	0.032599	5.956556	2.58E-09	1.57E-08	ARL6IP6	protein_cc
ENSG0000(	1442.087	-0.07748	0.039574	-1.95771	0.050265	0.08489	THEM6	protein_cc
ENSG0000(	1441.599	0.449547	0.041101	10.93752	7.63E-28	1.13E-25	MRPL11	protein_cc
ENSG0000(	1441.583	0.512882	0.071043	7.219288	5.23E-13	5.66E-12	AEN	protein_cc
ENSG0000(	1441.145	0.407352	0.12755	3.193663	0.001405	0.003335	NGFR	protein_cc
ENSG0000(	1440.797	1.131126	0.112453	10.05865	8.41E-24	5.92E-22	FOXM1	protein_cc
ENSG0000(	1440.608	-0.08085	0.023808	-3.39589	0.000684	0.001719	CEP63	protein_cc
ENSG0000(	1440.527	0.205632	0.03303	6.22565	4.80E-10	3.28E-09	ZNF333	protein_cc
ENSG0000(	1440.078	0.052689	0.029913	1.761416	0.078168	0.124792	MIER3	protein_cc
ENSG0000(	1438.513	-0.18526	0.031503	-5.88072	4.08E-09	2.41E-08	ANKRD27	protein_cc
ENSG0000(	1437.362	-0.07761	0.043918	-1.76708	0.077216	0.123456	LZTFL1	protein_cc
ENSG0000(	1437.257	0.130715	0.038707	3.377063	0.000733	0.00183	SCMH1	protein_cc
ENSG0000(	1437.158	-0.0087	0.033932	-0.2563	0.797716	0.850474	MOB2	protein_cc
ENSG0000(	1436.722	0.077309	0.108762	0.710808	0.477203	0.572763	ATF3	protein_cc
ENSG0000(	1436.412	-0.13304	0.037433	-3.55412	0.000379	0.001002	APOOL	protein_cc
ENSG0000(	1436.391	-0.026	0.039621	-0.65628	0.511642	0.604759	SNAPC4	protein_cc
ENSG0000(	1435.928	-0.43414	0.074607	-5.81898	5.92E-09	3.40E-08	HEY2	protein_cc
ENSG0000(	1435.288	0.093507	0.036954	2.530352	0.011395	0.022468	NANS	protein_cc
ENSG0000(	1434.776	0.216452	0.039217	5.519356	3.40E-08	1.74E-07	PPAPDC1I	protein_cc
ENSG0000(	1434.51	0.051426	0.02487	2.067815	0.038657	0.067317	MBD4	protein_cc
ENSG0000(	1433.908	-0.25789	0.046215	-5.58029	2.40E-08	1.26E-07	WAC-AS1	antisense
ENSG0000(	1433.625	0.813928	0.084167	9.670396	4.03E-22	1.99E-20	PRC1	protein_cc
ENSG0000(	1433.149	-0.48202	0.053586	-8.99532	2.36E-19	7.24E-18	RAB11FIP2	protein_cc
ENSG0000(	1433.077	-0.19267	0.037099	-5.19346	2.06E-07	9.30E-07	GLTSCR1L	protein_cc
ENSG0000(	1432.876	-0.02312	0.032385	-0.71404	0.475202	0.571042	PANK4	protein_cc
ENSG0000(	1432.654	-0.11717	0.03512	-3.33636	0.000849	0.002096	ESF1	protein_cc
ENSG0000(	1431.869	0.289753	0.06953	4.167332	3.08E-05	9.82E-05	PLEKHG1	protein_cc
ENSG0000(	1431.355	0.223907	0.078067	2.868132	0.004129	0.008957	HLA-F	protein_cc
ENSG0000(	1431.261	-0.05357	0.037914	-1.41289	0.157687	0.228193	MTHFD1L	protein_cc
ENSG0000(	1431.14	-0.19486	0.035403	-5.50419	3.71E-08	1.88E-07	ZNF281	protein_cc
ENSG0000(	1430.747	0.355025	0.063569	5.584846	2.34E-08	1.23E-07	PBX3	protein_cc
ENSG0000(	1430.573	0.064014	0.030636	2.089498	0.036663	0.06424	SMIM19	protein_cc
ENSG0000(	1430.482	-0.23677	0.042453	-5.5772	2.44E-08	1.28E-07	MSRB2	protein_cc
ENSG0000(	1430.168	0.231691	0.08699	2.663432	0.007735	0.015851	MYO1B	protein_cc
ENSG0000(	1429.947	0.316195	0.030983	10.20538	1.88E-24	1.48E-22	NUP54	protein_cc
ENSG0000(	1429.837	-0.04774	0.033192	-1.43827	0.150356	0.219249	TPMT	protein_cc
ENSG0000(	1429.795	-0.58617	0.103179	-5.68115	1.34E-08	7.28E-08	TPD52L1	protein_cc
ENSG0000(	1429.746	0.247348	0.027652	8.945153	3.71E-19	1.10E-17	TOR1B	protein_cc
ENSG0000(	1429.46	-0.13936	0.077205	-1.80506	0.071066	0.114766	TBXAS1	protein_cc
ENSG0000(	1428.893	0.167456	0.034823	4.808834	1.52E-06	5.96E-06	UBE2F	protein_cc
ENSG0000(	1428.846	0.074805	0.070727	1.057653	0.290214	0.3816	TMEM2	protein_cc
ENSG0000(	1428.451	-0.12715	0.057234	-2.22162	0.026309	0.047696	PDGFB	protein_cc
ENSG0000(	1427.979	0.155293	0.032347	4.800855	1.58E-06	6.19E-06	UTP11L	protein_cc
ENSG0000(	1427.586	-0.17023	0.032314	-5.26802	1.38E-07	6.38E-07	MTMR12	protein_cc
ENSG0000(	1427.322	-0.16661	0.068351	-2.43754	0.014788	0.02843	ARHGAP4	protein_cc
ENSG0000(	1426.534	0.052191	0.022321	2.338205	0.019377	0.036182	PDCD7	protein_cc
ENSG0000(	1425.816	0.089707	0.040153	2.234144	0.025474	0.046306	PLEKHH3	protein_cc
ENSG0000(	1425.21	1.587069	0.116144	13.66461	1.65E-42	2.95E-39	SOCS2	protein_cc
ENSG0000(	1424.719	0.140097	0.065805	2.128965	0.033257	0.058835	CDH5	protein_cc
ENSG0000(	1424.291	-0.12423	0.024956	-4.97793	6.43E-07	2.68E-06	RUNDC1	protein_cc
ENSG0000(	1423.994	0.082264	0.031516	2.610233	0.009048	0.018253	UFSP2	protein_cc
ENSG0000(	1423.472	0.142475	0.035274	4.039128	5.37E-05	0.000164	PDCD10	protein_cc
ENSG0000(	1423.35	0.008501	0.047293	0.179748	0.857351	0.896982	SERTAD2	protein_cc
ENSG0000(	1423.026	-0.25073	0.031412	-7.98195	1.44E-15	2.43E-14	ITSN2	protein_cc
ENSG0000(	1422.171	0.335913	0.036767	9.136221	6.47E-20	2.18E-18	ZNF561	protein_cc
ENSG0000(	1421.707	-0.48125	0.09753	-4.93439	8.04E-07	3.29E-06	AKR1C3	protein_cc
ENSG0000(	1420.529	0.003535	0.069949	0.05053	0.9597	0.972469	PTPRO	protein_cc
ENSG0000(	1420.47	0.08972	0.025687	3.492767	0.000478	0.001238	CDKN2AIF	protein_cc

ENSG0000(	1420.245	-0.1249	0.051454	-2.4274	0.015207	0.029141	UGDH	protein_cc
ENSG0000(	1419.824	0.061572	0.026873	2.291241	0.021949	0.040499	WDR36	protein_cc
ENSG0000(	1419.424	-0.57884	0.114369	-5.06116	4.17E-07	1.79E-06	HHATL	protein_cc
ENSG0000(	1418.958	-0.14051	0.034631	-4.05751	4.96E-05	0.000153	TBCEL	protein_cc
ENSG0000(	1418.67	0.268197	0.036038	7.441992	9.92E-14	1.21E-12	TRPT1	protein_cc
ENSG0000(	1418.099	0.035315	0.059078	0.597766	0.549996	0.63994	AASS	protein_cc
ENSG0000(	1417.911	-0.09869	0.080271	-1.22952	0.218877	0.301252	QPRT	protein_cc
ENSG0000(	1417.368	0.027038	0.045857	0.589624	0.555443	0.644927	NQO2	protein_cc
ENSG0000(	1417.066	-0.09252	0.074226	-1.2464	0.212618	0.294004	PCDH19	protein_cc
ENSG0000(	1416.637	0.341354	0.074253	4.597145	4.28E-06	1.57E-05	CRYZ	protein_cc
ENSG0000(	1416.602	-0.04081	0.038953	-1.04774	0.294757	0.386555	CRAMP1L	protein_cc
ENSG0000(	1416.291	-0.24	0.064945	-3.69547	0.000219	0.000604	FAM171A:	protein_cc
ENSG0000(	1416.132	-0.19275	0.067816	-2.84222	0.00448	0.009639	ARHGEF26	processed
ENSG0000(	1415.536	-1.61901	0.158389	-10.2217	1.58E-24	1.27E-22	GDA	protein_cc
ENSG0000(	1415.528	0.02924	0.115889	0.252306	0.800804	0.852756	LOXL2	protein_cc
ENSG0000(	1415.462	-0.05037	0.033615	-1.49856	0.133988	0.19847	EXOSC7	protein_cc
ENSG0000(	1415.343	-0.2581	0.106786	-2.41702	0.015648	0.029899	SLC8A3	protein_cc
ENSG0000(	1414.901	0.215361	0.044092	4.884348	1.04E-06	4.18E-06	TULP3	protein_cc
ENSG0000(	1414.365	-0.31367	0.080314	-3.90549	9.40E-05	0.000276	SYK	protein_cc
ENSG0000(	1414.06	-0.0594	0.046221	-1.28505	0.198774	0.277757	ALMS1	protein_cc
ENSG0000(	1413.728	-0.01256	0.030382	-0.41348	0.679253	0.75309	LIG3	protein_cc
ENSG0000(	1413.207	0.061269	0.030568	2.004311	0.045037	0.077007	NUS1	protein_cc
ENSG0000(	1412.953	-0.15959	0.068121	-2.34279	0.01914	0.035788	USP49	protein_cc
ENSG0000(	1412.662	0.288007	0.06693	4.303105	1.68E-05	5.61E-05	MLF1	protein_cc
ENSG0000(	1412.352	-0.00892	0.039957	-0.22313	0.823435	0.870644	CCNDBP1	protein_cc
ENSG0000(	1412.172	0.083717	0.045663	1.83337	0.066748	0.108701	CROCCP2	lincRNA
ENSG0000(	1412.131	1.057949	0.093958	11.25981	2.07E-29	4.22E-27	TACC3	protein_cc
ENSG0000(	1411.325	0.080306	0.035359	2.271153	0.023138	0.042474	COG6	protein_cc
ENSG0000(	1410.617	-0.27134	0.058063	-4.67317	2.97E-06	1.11E-05	HSD17B14	protein_cc
ENSG0000(	1410.522	0.503702	0.051475	9.785294	1.30E-22	7.04E-21	DCTPP1	protein_cc
ENSG0000(	1410.262	-0.12375	0.032883	-3.76343	0.000168	0.000471	ETV3	protein_cc
ENSG0000(	1410.101	0.526584	0.062666	8.403017	4.35E-17	9.39E-16	FAM111A	protein_cc
ENSG0000(	1409.786	0.134015	0.130414	1.027611	0.304133	0.396952	ST8SIA5	protein_cc
ENSG0000(	1409.733	0.135509	0.079993	1.694008	0.090264	0.141394	PDLIM7	protein_cc
ENSG0000(	1408.875	1.037897	0.092847	11.17852	5.19E-29	9.84E-27	WEE1	protein_cc
ENSG0000(	1408.392	-0.1124	0.034068	-3.29936	0.000969	0.002366	ZNF197	protein_cc
ENSG0000(	1408.369	-0.13942	0.056802	-2.45441	0.014112	0.027258	EHBP1L1	protein_cc
ENSG0000(	1407.665	0.196201	0.045563	4.306149	1.66E-05	5.53E-05	PXMP4	protein_cc
ENSG0000(	1407.509	0.03762	0.045138	0.833437	0.404598	0.501483	CLK4	protein_cc
ENSG0000(	1406.775	-0.063	0.042345	-1.48768	0.136836	0.202084	ZNF274	protein_cc
ENSG0000(	1406.738	0.197833	0.041675	4.747036	2.06E-06	7.93E-06	SLC25A13	protein_cc
ENSG0000(	1405.81	-1.60666	0.173791	-9.24476	2.36E-20	8.48E-19	GABRA1	protein_cc
ENSG0000(	1405.619	-0.08369	0.037457	-2.2342	0.02547	0.046301	FAM135A	protein_cc
ENSG0000(	1405.555	-0.39243	0.078993	-4.96793	6.77E-07	2.81E-06	DOCK8	protein_cc
ENSG0000(	1404.983	-0.66281	0.078842	-8.40686	4.21E-17	9.11E-16	VAMP1	protein_cc
ENSG0000(	1404.872	-0.18077	0.047563	-3.80073	0.000144	0.00041	NECAB3	protein_cc
ENSG0000(	1404.825	0.417475	0.091419	4.566592	4.96E-06	1.80E-05	HRH1	protein_cc
ENSG0000(	1404.797	-0.50695	0.080065	-6.33168	2.43E-10	1.74E-09	DISP2	protein_cc
ENSG0000(	1404.454	-0.20952	0.029659	-7.06428	1.61E-12	1.63E-11	TBC1D22A	protein_cc
ENSG0000(	1404.435	-0.27631	0.039555	-6.98547	2.84E-12	2.75E-11	FAM188A	protein_cc
ENSG0000(	1404.26	0.432858	0.049971	8.662132	4.63E-18	1.17E-16	CNPY4	protein_cc
ENSG0000(	1404.232	0.218353	0.050048	4.36285	1.28E-05	4.35E-05	PRKD2	protein_cc
ENSG0000(	1404.103	1.664312	0.158913	10.47313	1.15E-25	1.16E-23	FABP5	protein_cc
ENSG0000(	1403.947	-0.6677	0.089975	-7.42103	1.16E-13	1.40E-12	RANBP3L	protein_cc
ENSG0000(	1403.544	0.415098	0.060538	6.856773	7.04E-12	6.41E-11	MAP3K1	protein_cc
ENSG0000(	1403.144	-0.00548	0.036441	-0.15048	0.880387	0.914407	CYB561A3	protein_cc
ENSG0000(	1402.967	0.372132	0.037768	9.853082	6.65E-23	3.84E-21	ZNF43	protein_cc
ENSG0000(	1402.218	-0.16205	0.029217	-5.54651	2.91E-08	1.50E-07	STRN3	protein_cc
ENSG0000(	1402.194	0.141257	0.034508	4.093494	4.25E-05	0.000132	INPP5E	protein_cc

ENSG0000(	1401.493	0.461066	0.04667	9.879196	5.12E-23	3.01E-21	CHST14	protein_cc
ENSG0000(	1401.273	-0.01878	0.037751	-0.49734	0.618947	0.701683	RSBN1	protein_cc
ENSG0000(	1401.062	-0.06858	0.050573	-1.35612	0.17506	0.249486	E2F3	protein_cc
ENSG0000(	1400.55	0.17408	0.101364	1.717369	0.085912	0.135396	AHR	protein_cc
ENSG0000(	1399.628	0.06453	0.041745	1.545818	0.122148	0.183437	RALGAPA2	protein_cc
ENSG0000(	1399.253	0.087253	0.031381	2.780394	0.005429	0.011489	LEO1	protein_cc
ENSG0000(	1399.251	1.161815	0.111782	10.39357	2.65E-25	2.45E-23	NUSAP1	protein_cc
ENSG0000(	1398.694	0.149114	0.084535	1.763941	0.077742	0.124191	HERC6	protein_cc
ENSG0000(	1398.312	0.173885	0.025891	6.716042	1.87E-11	1.59E-10	BCS1L	protein_cc
ENSG0000(	1398.091	0.06443	0.033094	1.946886	0.051548	0.086784	RNF166	protein_cc
ENSG0000(	1397.981	0.137803	0.031494	4.375594	1.21E-05	4.12E-05	TMEM18	protein_cc
ENSG0000(	1397.867	-0.26887	0.068285	-3.93742	8.24E-05	0.000245	TMEM38A	protein_cc
ENSG0000(	1397.68	0.878708	0.102393	8.581706	9.35E-18	2.26E-16	ACSS3	protein_cc
ENSG0000(	1397.394	0.024667	0.069086	0.357047	0.721057	0.787699	CTD-2228	processed
ENSG0000(	1397.391	0.259246	0.032398	8.002016	1.22E-15	2.08E-14	RNF138	protein_cc
ENSG0000(	1397.355	-0.47309	0.065096	-7.26759	3.66E-13	4.06E-12	ST3GAL6	protein_cc
ENSG0000(	1397.136	-0.18249	0.072946	-2.5017	0.01236	0.02417	NXPH3	protein_cc
ENSG0000(	1396.282	-0.29782	0.067719	-4.39781	1.09E-05	3.75E-05	ALPL	protein_cc
ENSG0000(	1395.718	-0.73166	0.071717	-10.2021	1.94E-24	1.52E-22	SGIP1	protein_cc
ENSG0000(	1394.699	0.122452	0.0407	3.008625	0.002624	0.00592	OFD1	protein_cc
ENSG0000(	1394.394	-0.02604	0.035088	-0.74205	0.458054	0.554495	KLHL20	protein_cc
ENSG0000(	1393.967	-0.42369	0.054241	-7.81123	5.66E-15	8.62E-14	PPP1R3F	protein_cc
ENSG0000(	1393.67	-0.65435	0.091352	-7.16296	7.90E-13	8.34E-12	KLHL32	protein_cc
ENSG0000(	1393.43	0.172877	0.032557	5.309939	1.10E-07	5.17E-07	NPRL2	protein_cc
ENSG0000(	1393.384	0.032584	0.041465	0.785816	0.431975	0.528927	PHF13	protein_cc
ENSG0000(	1392.532	-0.50604	0.083743	-6.04275	1.52E-09	9.59E-09	SORCS2	protein_cc
ENSG0000(	1392.131	0.51023	0.113332	4.502075	6.73E-06	2.39E-05	OAS2	protein_cc
ENSG0000(	1392.058	-0.32279	0.054682	-5.90306	3.57E-09	2.13E-08	SORD	protein_cc
ENSG0000(	1391.889	0.192789	0.029857	6.45706	1.07E-10	8.10E-10	ZNF268	protein_cc
ENSG0000(	1391.811	0.235567	0.030171	7.807659	5.83E-15	8.85E-14	C4orf27	protein_cc
ENSG0000(	1391.75	-0.01853	0.027702	-0.66887	0.503576	0.597405	MAP2K5	protein_cc
ENSG0000(	1391.605	-0.07201	0.098191	-0.73334	0.46335	0.559497	ADAMTS1	protein_cc
ENSG0000(	1391.524	0.102854	0.052925	1.943382	0.05197	0.087437	ST3GAL4	protein_cc
ENSG0000(	1391.498	-0.46051	0.043481	-10.591	3.28E-26	3.63E-24	HERC3	protein_cc
ENSG0000(	1391.4	0.095458	0.048736	1.958683	0.05015	0.084721	AKAP7	protein_cc
ENSG0000(	1391.03	0.140276	0.039113	3.586434	0.000335	0.000895	POLA1	protein_cc
ENSG0000(	1390.771	-0.08512	0.03167	-2.68766	0.007195	0.014829	SGPP1	protein_cc
ENSG0000(	1390.345	0.068509	0.037221	1.84059	0.065682	0.107181	CYB5A	protein_cc
ENSG0000(	1389.733	0.05088	0.034726	1.465184	0.142871	0.209789	TXLNG	protein_cc
ENSG0000(	1389.365	0.104035	0.030126	3.453326	0.000554	0.001416	CTNS	protein_cc
ENSG0000(	1388.97	0.052777	0.020672	2.553086	0.010677	0.021193	RAD17	protein_cc
ENSG0000(	1388.676	0.100063	0.023949	4.178199	2.94E-05	9.39E-05	VPS54	protein_cc
ENSG0000(	1388.409	0.279319	0.042323	6.599743	4.12E-11	3.33E-10	MRPL14	protein_cc
ENSG0000(	1388.082	0.113323	0.025811	4.390478	1.13E-05	3.87E-05	HINFP	protein_cc
ENSG0000(	1388.037	0.152719	0.039364	3.879633	0.000105	0.000305	GLTPD1	protein_cc
ENSG0000(	1387.916	-1.15019	0.11909	-9.65821	4.54E-22	2.21E-20	SHANK1	protein_cc
ENSG0000(	1387.783	0.156232	0.034929	4.472849	7.72E-06	2.71E-05	TMEM134	protein_cc
ENSG0000(	1387.77	0.266782	0.029646	8.998836	2.28E-19	7.02E-18	MIS12	protein_cc
ENSG0000(	1387.355	-0.02911	0.027498	-1.05848	0.289837	0.381221	ZNF330	protein_cc
ENSG0000(	1386.924	0.037417	0.031891	1.173268	0.240688	0.326098	CCDC53	protein_cc
ENSG0000(	1386.66	0.103755	0.038964	2.662875	0.007748	0.015876	NPR2	protein_cc
ENSG0000(	1386.297	-0.25666	0.073849	-3.4755	0.00051	0.001313	PRKAR2B	protein_cc
ENSG0000(	1386.179	-0.23878	0.045508	-5.24704	1.55E-07	7.10E-07	MORN4	protein_cc
ENSG0000(	1386.026	0.191946	0.026604	7.214923	5.40E-13	5.83E-12	TAF11	protein_cc
ENSG0000(	1386.012	-0.36585	0.041243	-8.87042	7.29E-19	2.07E-17	NKIRAS1	protein_cc
ENSG0000(	1385.709	0.125682	0.034552	3.637433	0.000275	0.000746	RAB8A	protein_cc
ENSG0000(	1385.004	0.039097	0.029206	1.338659	0.180682	0.25606	PHKG2	protein_cc
ENSG0000(	1384.924	0.524085	0.084049	6.23546	4.50E-10	3.10E-09	SMYD2	protein_cc
ENSG0000(	1384.707	0.232044	0.032477	7.144922	9.00E-13	9.43E-12	PEMT	protein_cc

ENSG0000(	1384.48	0.245531	0.074109	3.313098	0.000923	0.002262	RBMS1	protein_cc
ENSG0000(	1384.132	-0.07677	0.043225	-1.77601	0.075732	0.121348	TEP1	protein_cc
ENSG0000(	1383.485	0.292252	0.070813	4.127106	3.67E-05	0.000116	SHROOM:	protein_cc
ENSG0000(	1383.193	-0.04325	0.054614	-0.79187	0.428435	0.525531	SLC8B1	protein_cc
ENSG0000(	1383.159	0.549301	0.08743	6.282715	3.33E-10	2.34E-09	IFI44	protein_cc
ENSG0000(	1382.647	-0.05867	0.031809	-1.84439	0.065127	0.106386	PEX14	protein_cc
ENSG0000(	1382.551	0.050081	0.03604	1.389625	0.164643	0.236839	ZFAND2B	protein_cc
ENSG0000(	1382.357	0.168825	0.022281	7.577235	3.53E-14	4.63E-13	WDR70	protein_cc
ENSG0000(	1382.198	-0.57365	0.074146	-7.73679	1.02E-14	1.48E-13	RAPGEFL1	protein_cc
ENSG0000(	1381.448	0.258158	0.035815	7.208142	5.67E-13	6.12E-12	DCAF15	protein_cc
ENSG0000(	1380.843	0.102664	0.027432	3.742524	0.000182	0.000509	TRAPPC1:	protein_cc
ENSG0000(	1380.623	-0.25524	0.064111	-3.98118	6.86E-05	0.000206	PPM1E	protein_cc
ENSG0000(	1380.531	-0.79729	0.151183	-5.2737	1.34E-07	6.20E-07	SPHKAP	protein_cc
ENSG0000(	1380.318	0.084167	0.045492	1.850142	0.064293	0.105217	ABHD14B	protein_cc
ENSG0000(	1380.166	0.199737	0.040148	4.975022	6.52E-07	2.72E-06	STYXL1	protein_cc
ENSG0000(	1379.914	-0.71679	0.114673	-6.25075	4.08E-10	2.83E-09	CACNA1E	protein_cc
ENSG0000(	1379.747	-0.22191	0.059716	-3.71606	0.000202	0.00056	ABTB1	protein_cc
ENSG0000(	1379.619	0.302957	0.030502	9.932504	3.01E-23	1.86E-21	ZC3HC1	protein_cc
ENSG0000(	1378.992	-0.09515	0.021751	-4.37465	1.22E-05	4.14E-05	NKAP	protein_cc
ENSG0000(	1378.596	0.006848	0.034017	0.201299	0.840465	0.883994	TCHP	protein_cc
ENSG0000(	1378.546	-1.10371	0.090068	-12.2541	1.60E-34	8.88E-32	CDKL5	protein_cc
ENSG0000(	1377.219	-0.21431	0.067041	-3.19673	0.00139	0.003304	JUP	protein_cc
ENSG0000(	1377.073	-0.50139	0.108003	-4.6424	3.44E-06	1.28E-05	BOK	protein_cc
ENSG0000(	1376.949	-0.72466	0.089426	-8.10353	5.34E-16	9.63E-15	DNAJA4	protein_cc
ENSG0000(	1376.365	0.173926	0.024584	7.07474	1.50E-12	1.52E-11	NGLY1	protein_cc
ENSG0000(	1376.258	0.005298	0.035382	0.149741	0.880969	0.914896	POLR1E	protein_cc
ENSG0000(	1376.175	0.122496	0.041735	2.93508	0.003335	0.007366	CEP95	protein_cc
ENSG0000(	1375.763	0.027273	0.051	0.534767	0.592811	0.678299	PIM3	protein_cc
ENSG0000(	1375.679	-1.23711	0.112543	-10.9923	4.16E-28	6.41E-26	C1orf115	protein_cc
ENSG0000(	1375.301	0.241549	0.037701	6.406986	1.48E-10	1.10E-09	BBS4	protein_cc
ENSG0000(	1375.078	0.069938	0.08375	0.835073	0.403677	0.500536	FXYD5	protein_cc
ENSG0000(	1374.784	0.168345	0.030912	5.445985	5.15E-08	2.55E-07	HAUS2	protein_cc
ENSG0000(	1374.328	0.327794	0.043538	7.528984	5.11E-14	6.55E-13	FAM57A	protein_cc
ENSG0000(	1374.124	-0.37028	0.095284	-3.88608	0.000102	0.000297	FLRT1	protein_cc
ENSG0000(	1374.091	0.072647	0.058218	1.247858	0.212083	0.293403	TTLL3	protein_cc
ENSG0000(	1373.942	0.529055	0.049718	10.64109	1.92E-26	2.24E-24	KRT10	protein_cc
ENSG0000(	1373.71	0.120766	0.035802	3.373183	0.000743	0.001854	C5orf45	protein_cc
ENSG0000(	1373.123	-0.22947	0.03935	-5.83155	5.49E-09	3.17E-08	BAG4	protein_cc
ENSG0000(	1372.905	0.082179	0.036091	2.277014	0.022785	0.041888	CBY1	protein_cc
ENSG0000(	1372.721	0.172923	0.039313	4.398639	1.09E-05	3.74E-05	THOC1	protein_cc
ENSG0000(	1372.237	0.106237	0.037196	2.856119	0.004289	0.009268	WDR74	protein_cc
ENSG0000(	1372.23	0.031194	0.028663	1.088295	0.276465	0.366623	CLCC1	protein_cc
ENSG0000(	1371.921	-0.72958	0.103882	-7.02315	2.17E-12	2.14E-11	UBE2QL1	protein_cc
ENSG0000(	1371.635	0.124733	0.051681	2.413503	0.0158	0.030167	PLCXD1	protein_cc
ENSG0000(	1370.951	0.182158	0.029863	6.099778	1.06E-09	6.90E-09	DNAJC2	protein_cc
ENSG0000(	1370.056	0.313371	0.113526	2.760356	0.005774	0.01214	GBP3	protein_cc
ENSG0000(	1370.019	0.034974	0.038341	0.91218	0.361674	0.457548	SLIRP	protein_cc
ENSG0000(	1369.877	0.130047	0.023918	5.437179	5.41E-08	2.67E-07	ANAPC4	protein_cc
ENSG0000(	1369.086	-0.19866	0.050824	-3.90882	9.27E-05	0.000273	CCDC91	protein_cc
ENSG0000(	1368.671	-0.42525	0.101062	-4.2078	2.58E-05	8.32E-05	C4A	protein_cc
ENSG0000(	1368.109	-0.19097	0.049687	-3.84346	0.000121	0.000349	KPNA5	protein_cc
ENSG0000(	1367.422	0.132711	0.054438	2.43783	0.014776	0.028411	UACA	protein_cc
ENSG0000(	1366.835	0.326808	0.063849	5.118466	3.08E-07	1.35E-06	C19orf66	protein_cc
ENSG0000(	1366.561	-0.2895	0.047711	-6.0678	1.30E-09	8.31E-09	FOXP1	protein_cc
ENSG0000(	1366.075	0.090216	0.032766	2.753333	0.005899	0.012379	C1orf52	protein_cc
ENSG0000(	1365.816	0.293045	0.055234	5.305492	1.12E-07	5.29E-07	POLE	protein_cc
ENSG0000(	1365.683	-1.21467	0.114034	-10.6519	1.71E-26	2.03E-24	NRXN3	protein_cc
ENSG0000(	1365.411	-0.71149	0.086182	-8.2556	1.51E-16	2.98E-15	PPFIA4	protein_cc
ENSG0000(	1364.755	0.145031	0.028457	5.096459	3.46E-07	1.50E-06	RSRC1	protein_cc

ENSG0000(	1364.723	-0.05602	0.033868	-1.65402	0.098124	0.151998	NAA25	protein_cc
ENSG0000(	1364.316	-0.05106	0.032547	-1.56883	0.116688	0.176506	EXT1	protein_cc
ENSG0000(	1364.272	-1.53133	0.147059	-10.413	2.16E-25	2.02E-23	GRIN2B	protein_cc
ENSG0000(	1363.587	0.38596	0.070019	5.512227	3.54E-08	1.80E-07	EIF4EBP1	protein_cc
ENSG0000(	1362.179	0.035496	0.031307	1.133794	0.256881	0.344757	FAHD1	protein_cc
ENSG0000(	1362.016	-0.13187	0.037874	-3.48167	0.000498	0.001286	POLL	protein_cc
ENSG0000(	1361.523	0.057441	0.02901	1.980023	0.047701	0.081	POLI	protein_cc
ENSG0000(	1361.407	-0.23407	0.048856	-4.79103	1.66E-06	6.48E-06	ZNF37A	protein_cc
ENSG0000(	1361.241	0.088882	0.038075	2.334389	0.019575	0.03651	RTCA	protein_cc
ENSG0000(	1360.839	-0.12476	0.101267	-1.23203	0.217938	0.300171	GEM	protein_cc
ENSG0000(	1360.659	0.076126	0.04155	1.83214	0.066931	0.108961	BIVM	protein_cc
ENSG0000(	1360.621	-0.2966	0.050037	-5.92774	3.07E-09	1.85E-08	CERS6	protein_cc
ENSG0000(	1360.437	-0.31797	0.041059	-7.74436	9.61E-15	1.41E-13	TBC1D4	protein_cc
ENSG0000(	1360.274	-0.4099	0.056057	-7.3123	2.63E-13	2.98E-12	EIF4E3	protein_cc
ENSG0000(	1360.238	0.060764	0.028811	2.109062	0.034939	0.06154	SETD4	protein_cc
ENSG0000(	1359.912	-0.6503	0.074544	-8.72367	2.69E-18	7.03E-17	TGFBR3	protein_cc
ENSG0000(	1359.754	0.295474	0.038355	7.703757	1.32E-14	1.87E-13	POP5	protein_cc
ENSG0000(	1359.001	0.177622	0.047809	3.715256	0.000203	0.000562	PPP1R16A	protein_cc
ENSG0000(	1358.892	0.156587	0.035206	4.447712	8.68E-06	3.02E-05	CISD2	protein_cc
ENSG0000(	1358.797	-0.21502	0.028237	-7.61479	2.64E-14	3.54E-13	DNAJC21	protein_cc
ENSG0000(	1358.768	0.194432	0.024445	7.953798	1.81E-15	3.00E-14	UMPS	protein_cc
ENSG0000(	1358.754	-0.48439	0.046893	-10.3296	5.18E-25	4.55E-23	C9orf91	protein_cc
ENSG0000(	1358.258	-0.11013	0.061728	-1.78419	0.074393	0.1195	TRIB1	protein_cc
ENSG0000(	1357.95	-0.05558	0.030065	-1.84859	0.064516	0.105536	NAA35	protein_cc
ENSG0000(	1357.698	-0.01729	0.033575	-0.51497	0.606577	0.690496	TOMM40L	protein_cc
ENSG0000(	1357.635	-0.18011	0.093673	-1.92279	0.054507	0.091138	PODN	protein_cc
ENSG0000(	1357.291	-0.03507	0.037299	-0.94011	0.347161	0.442659	PTPMT1	protein_cc
ENSG0000(	1356.87	-0.10126	0.023757	-4.26226	2.02E-05	6.65E-05	UTP3	protein_cc
ENSG0000(	1356.167	-0.00662	0.032922	-0.20114	0.840588	0.8841	PEX12	protein_cc
ENSG0000(	1356.094	-0.14171	0.031535	-4.49389	6.99E-06	2.48E-05	USP6NL	protein_cc
ENSG0000(	1356.041	0.147239	0.044958	3.27506	0.001056	0.002563	TMEM141	protein_cc
ENSG0000(	1355.826	-0.87147	0.085625	-10.1778	2.49E-24	1.90E-22	GFOD1	protein_cc
ENSG0000(	1355.757	-0.02617	0.035726	-0.73253	0.463843	0.559962	ZFP62	protein_cc
ENSG0000(	1355.706	-0.07424	0.029262	-2.53719	0.011175	0.022085	VIPAS39	protein_cc
ENSG0000(	1355.278	-0.08672	0.033437	-2.59357	0.009499	0.019074	FITM2	protein_cc
ENSG0000(	1355.009	-0.50105	0.115857	-4.32476	1.53E-05	5.12E-05	RASL10A	protein_cc
ENSG0000(	1354.239	-0.35996	0.05201	-6.921	4.48E-12	4.21E-11	PHLPP2	protein_cc
ENSG0000(	1354.229	-0.10247	0.038922	-2.63279	0.008469	0.017206	HCG18	antisense
ENSG0000(	1353.187	0.049896	0.044779	1.114264	0.265166	0.353979	MARK1	protein_cc
ENSG0000(	1353.044	-0.23375	0.045499	-5.13756	2.78E-07	1.23E-06	ZNF518A	processed
ENSG0000(	1352.883	0.375893	0.032824	11.45175	2.30E-30	5.78E-28	NT5C3A	protein_cc
ENSG0000(	1351.993	0.17149	0.061064	2.808354	0.00498	0.010614	NFIL3	protein_cc
ENSG0000(	1351.781	-0.50336	0.060954	-8.25796	1.48E-16	2.93E-15	FSD1L	protein_cc
ENSG0000(	1351.743	0.062271	0.021997	2.830909	0.004642	0.009949	VPS33A	protein_cc
ENSG0000(	1351.735	0.811959	0.083606	9.711731	2.69E-22	1.37E-20	TGIF1	protein_cc
ENSG0000(	1350.537	0.003697	0.040506	0.09126	0.927286	0.948812	OSBPL7	protein_cc
ENSG0000(	1350.222	0.121329	0.041955	2.891899	0.003829	0.008358	DNAJC4	protein_cc
ENSG0000(	1349.968	0.422055	0.043558	9.689456	3.34E-22	1.68E-20	LINC0066!	lincRNA
ENSG0000(	1349.848	-0.01795	0.043442	-0.41315	0.679494	0.753255	NBPF1	protein_cc
ENSG0000(	1349.839	-0.29053	0.040065	-7.25152	4.12E-13	4.54E-12	FAM179B	protein_cc
ENSG0000(	1349.595	0.148506	0.030217	4.914677	8.89E-07	3.62E-06	SLC25A17	protein_cc
ENSG0000(	1349.507	-0.03264	0.03094	-1.055	0.291424	0.382927	PPIL4	protein_cc
ENSG0000(	1348.711	-1.19797	0.111391	-10.7547	5.63E-27	7.26E-25	PITPNM3	protein_cc
ENSG0000(	1348.521	0.41073	0.06755	6.080399	1.20E-09	7.72E-09	IL17RC	protein_cc
ENSG0000(	1348.33	0.274113	0.036493	7.511439	5.85E-14	7.41E-13	DNAJC19	protein_cc
ENSG0000(	1348.224	-0.05306	0.034366	-1.54398	0.122593	0.18399	CLPB	protein_cc
ENSG0000(	1348.216	0.226777	0.050252	4.512783	6.40E-06	2.28E-05	CTPS1	protein_cc
ENSG0000(	1347.873	-0.09526	0.035457	-2.68661	0.007218	0.014874	RCOR1	protein_cc
ENSG0000(	1347.372	0.173091	0.029302	5.907155	3.48E-09	2.08E-08	STX2	protein_cc

ENSG0000(	1346.833	0.285588	0.04064	7.02731	2.11E-12	2.09E-11	POP4	protein_cc
ENSG0000(	1346.741	0.045025	0.030971	1.453787	0.146005	0.213708	PIAS4	protein_cc
ENSG0000(	1346.708	-0.24757	0.039857	-6.21148	5.25E-10	3.57E-09	DNAJB4	protein_cc
ENSG0000(	1346.505	0.105133	0.027571	3.813196	0.000137	0.000391	INTS4	protein_cc
ENSG0000(	1346.416	0.432801	0.054904	7.882838	3.20E-15	5.09E-14	CCDC167	protein_cc
ENSG0000(	1346.376	0.156291	0.038355	4.074888	4.60E-05	0.000143	THAP9-AS	antisense
ENSG0000(	1346.309	0.045072	0.036372	1.23919	0.215275	0.29717	C12orf29	protein_cc
ENSG0000(	1346.236	0.447824	0.051268	8.73488	2.44E-18	6.42E-17	TXNDC17	protein_cc
ENSG0000(	1345.538	0.220308	0.028522	7.724243	1.13E-14	1.62E-13	INTS12	protein_cc
ENSG0000(	1345.48	0.070583	0.027532	2.563671	0.010357	0.020621	APTX	protein_cc
ENSG0000(	1345.392	0.32742	0.037931	8.632011	6.03E-18	1.50E-16	EXOSC8	protein_cc
ENSG0000(	1345.356	-0.08164	0.049598	-1.64599	0.099766	0.154211	ZNF74	protein_cc
ENSG0000(	1345.182	-0.12343	0.033015	-3.73852	0.000185	0.000516	TOPORS	protein_cc
ENSG0000(	1345.179	-0.41564	0.081502	-5.09974	3.40E-07	1.48E-06	SORBS2	protein_cc
ENSG0000(	1345.168	-0.19071	0.027426	-6.95365	3.56E-12	3.40E-11	SAV1	protein_cc
ENSG0000(	1344.962	-0.01583	0.032782	-0.48292	0.629149	0.710605	AEBP2	protein_cc
ENSG0000(	1344.58	0.123973	0.030069	4.122905	3.74E-05	0.000117	MED10	protein_cc
ENSG0000(	1344.466	-0.05795	0.035435	-1.63544	0.101957	0.157122	NOL9	protein_cc
ENSG0000(	1343.233	0.463687	0.048887	9.484827	2.43E-21	1.05E-19	TMEM194	protein_cc
ENSG0000(	1343.089	0.069907	0.047945	1.45806	0.144824	0.212258	RNF19B	protein_cc
ENSG0000(	1341.833	0.103292	0.022817	4.527062	5.98E-06	2.14E-05	CDK5RAP1	protein_cc
ENSG0000(	1341.595	0.953219	0.135749	7.021943	2.19E-12	2.16E-11	FOXJ1	protein_cc
ENSG0000(	1341.565	1.359015	0.136476	9.957906	2.33E-23	1.50E-21	BIRC5	protein_cc
ENSG0000(	1341.337	-0.07322	0.086367	-0.84777	0.396564	0.493205	THEMIS2	protein_cc
ENSG0000(	1340.613	-0.28506	0.051237	-5.56348	2.64E-08	1.37E-07	AACS	protein_cc
ENSG0000(	1340.602	0.098929	0.078879	1.254197	0.209771	0.290732	SLC15A3	protein_cc
ENSG0000(	1340.51	-0.08408	0.029912	-2.81085	0.004941	0.010542	CTBP1-AS	antisense
ENSG0000(	1340.446	0.038443	0.036248	1.060571	0.288885	0.380224	PRPF39	protein_cc
ENSG0000(	1340.321	0.032719	0.030203	1.083278	0.278685	0.369067	QRSL1	protein_cc
ENSG0000(	1340.319	-0.07726	0.035472	-2.17812	0.029397	0.052691	PCSK7	protein_cc
ENSG0000(	1340.084	0.049258	0.0486	1.013545	0.3108	0.404051	BAZ1A	protein_cc
ENSG0000(	1339.905	0.394511	0.069177	5.702881	1.18E-08	6.47E-08	SLC2A4RG	protein_cc
ENSG0000(	1339.857	-0.10967	0.033763	-3.24837	0.001161	0.002793	TMEM55A	protein_cc
ENSG0000(	1339.708	0.09679	0.023778	4.070497	4.69E-05	0.000145	DENND6A	protein_cc
ENSG0000(	1338.883	0.070996	0.031162	2.2783	0.022709	0.041767	FUK	protein_cc
ENSG0000(	1338.641	0.410646	0.037489	10.95382	6.37E-28	9.53E-26	AP4M1	protein_cc
ENSG0000(	1338.509	-1.45901	0.173037	-8.43178	3.40E-17	7.48E-16	F5	protein_cc
ENSG0000(	1338.507	0.211568	0.033293	6.354765	2.09E-10	1.51E-09	MRPL2	protein_cc
ENSG0000(	1338.419	-0.76996	0.118806	-6.48082	9.12E-11	7.00E-10	EGR2	protein_cc
ENSG0000(	1337.543	-0.21212	0.038567	-5.49996	3.80E-08	1.93E-07	SLC9B2	protein_cc
ENSG0000(	1337.531	0.168988	0.031829	5.309294	1.10E-07	5.18E-07	NCK1	protein_cc
ENSG0000(	1336.956	0.133745	0.046668	2.865905	0.004158	0.009016	ZNF580	protein_cc
ENSG0000(	1336.55	0.166347	0.096507	1.723681	0.084766	0.1338	FGFBP3	protein_cc
ENSG0000(	1336.103	-0.07393	0.036757	-2.01123	0.044301	0.075903	GIT2	protein_cc
ENSG0000(	1335.782	0.148461	0.050171	2.959088	0.003086	0.006859	CREM	protein_cc
ENSG0000(	1335.708	0.77386	0.062146	12.45238	1.36E-35	9.97E-33	RNASEH2A	protein_cc
ENSG0000(	1335.598	0.437463	0.066615	6.567087	5.13E-11	4.08E-10	SYDE1	protein_cc
ENSG0000(	1335.575	0.069798	0.034784	2.006583	0.044794	0.076652	POC1B	protein_cc
ENSG0000(	1335.413	0.012096	0.033306	0.363174	0.716475	0.784044	ZNF335	protein_cc
ENSG0000(	1334.244	-0.27476	0.040568	-6.77286	1.26E-11	1.10E-10	MAP4K2	protein_cc
ENSG0000(	1334.213	-0.19779	0.037394	-5.28927	1.23E-07	5.74E-07	TUBGCP4	protein_cc
ENSG0000(	1334.084	-0.28304	0.0557	-5.08159	3.74E-07	1.62E-06	SLC25A33	protein_cc
ENSG0000(	1333.82	0.127947	0.076699	1.668182	0.09528	0.148167	CASC15	lincRNA
ENSG0000(	1333.724	0.227551	0.043744	5.201876	1.97E-07	8.92E-07	HINT2	protein_cc
ENSG0000(	1333.577	-0.09331	0.035668	-2.61599	0.008897	0.01798	ENDOV	protein_cc
ENSG0000(	1333.546	-0.16654	0.095144	-1.75041	0.080048	0.127336	PTPRC	protein_cc
ENSG0000(	1332.993	-0.10461	0.048175	-2.17144	0.029898	0.053491	CDR2L	protein_cc
ENSG0000(	1332.866	0.120191	0.032412	3.70822	0.000209	0.000576	PRUNE	protein_cc
ENSG0000(	1332.68	-0.35442	0.091204	-3.88602	0.000102	0.000297	SIGLEC8	protein_cc

ENSG0000(	1332.648	-0.01144	0.025833	-0.44285	0.657876	0.735217	ZDHHC24	protein_cc
ENSG0000(	1332.453	-0.56047	0.072982	-7.67963	1.60E-14	2.23E-13	CACNB4	protein_cc
ENSG0000(	1331.779	0.087571	0.036492	2.399727	0.016407	0.031209	B4GALT4	protein_cc
ENSG0000(	1331.43	0.136841	0.038334	3.569748	0.000357	0.000949	TM2D1	protein_cc
ENSG0000(	1331.232	-0.05876	0.066489	-0.88371	0.376855	0.473301	RAC3	protein_cc
ENSG0000(	1330.581	0.033907	0.080481	0.421311	0.673528	0.748255	LAT2	protein_cc
ENSG0000(	1330.455	0.912777	0.084642	10.78399	4.10E-27	5.37E-25	UBE2S	protein_cc
ENSG0000(	1330.366	0.07677	0.047714	1.608979	0.107621	0.164559	PM20D2	protein_cc
ENSG0000(	1330.349	-0.06144	0.034403	-1.78604	0.074092	0.119063	ZNF507	protein_cc
ENSG0000(	1330.256	0.146706	0.028716	5.108849	3.24E-07	1.41E-06	METTL5	protein_cc
ENSG0000(	1330.005	0.112061	0.041365	2.709102	0.006747	0.013989	TMEM198	protein_cc
ENSG0000(	1329.514	-0.10862	0.029959	-3.62574	0.000288	0.000778	WBP4	protein_cc
ENSG0000(	1329.386	-0.19253	0.035028	-5.4964	3.88E-08	1.96E-07	CDC37L1	protein_cc
ENSG0000(	1329.223	0.420818	0.042987	9.789403	1.25E-22	6.80E-21	ZNF627	protein_cc
ENSG0000(	1329.177	-0.44741	0.059176	-7.56075	4.01E-14	5.21E-13	MAGI3	protein_cc
ENSG0000(	1327.547	-0.19676	0.031239	-6.2985	3.01E-10	2.13E-09	RILPL1	protein_cc
ENSG0000(	1327.531	0.026977	0.02328	1.158825	0.246528	0.332946	RNF121	protein_cc
ENSG0000(	1327.386	0.086022	0.084998	1.012047	0.311515	0.404802	LHX2	protein_cc
ENSG0000(	1326.781	0.399309	0.067699	5.898277	3.67E-09	2.19E-08	HES1	protein_cc
ENSG0000(	1326.61	0.088592	0.026415	3.353816	0.000797	0.001979	GATC	protein_cc
ENSG0000(	1326.415	-0.14132	0.049748	-2.84072	0.004501	0.009677	TDRP	protein_cc
ENSG0000(	1326.163	-0.18033	0.059541	-3.02869	0.002456	0.005577	ADAMTSL	protein_cc
ENSG0000(	1325.939	-0.27599	0.096427	-2.86211	0.004208	0.009115	KIF26B	protein_cc
ENSG0000(	1325.74	0.29898	0.040207	7.436055	1.04E-13	1.26E-12	RPF1	protein_cc
ENSG0000(	1325.664	0.402051	0.038869	10.34382	4.46E-25	3.99E-23	MOSPD3	protein_cc
ENSG0000(	1325.654	0.415807	0.076755	5.417306	6.05E-08	2.97E-07	RP11-849	lincRNA
ENSG0000(	1325.319	0.042243	0.022689	1.861796	0.062632	0.102846	WDR55	protein_cc
ENSG0000(	1325.016	0.334498	0.042574	7.856835	3.94E-15	6.17E-14	SLC39A3	protein_cc
ENSG0000(	1324.302	0.308003	0.043214	7.127383	1.02E-12	1.06E-11	ALG3	protein_cc
ENSG0000(	1323.884	-0.04357	0.029718	-1.46618	0.142599	0.209434	ZNF319	protein_cc
ENSG0000(	1323.317	-0.09293	0.054809	-1.69554	0.089972	0.140985	ATG16L2	protein_cc
ENSG0000(	1323.164	0.160738	0.032283	4.979087	6.39E-07	2.66E-06	UTP20	protein_cc
ENSG0000(	1323.094	0.127373	0.0364	3.499281	0.000467	0.001211	HEMK1	protein_cc
ENSG0000(	1323.041	-1.76473	0.161813	-10.906	1.08E-27	1.56E-25	GABRB2	protein_cc
ENSG0000(	1322.485	0.073878	0.061419	1.202861	0.22903	0.312911	LRRC23	protein_cc
ENSG0000(	1322.368	-0.00673	0.024412	-0.27548	0.782947	0.8384	TBK1	protein_cc
ENSG0000(	1322.359	0.056505	0.032257	1.751737	0.079819	0.127016	ATL2	protein_cc
ENSG0000(	1321.221	0.258853	0.03867	6.693848	2.17E-11	1.83E-10	WDR77	protein_cc
ENSG0000(	1321.104	-0.12007	0.052721	-2.27751	0.022756	0.041839	ABCC1	protein_cc
ENSG0000(	1320.881	-0.01627	0.028305	-0.57472	0.565478	0.653569	HEATR6	protein_cc
ENSG0000(	1320.792	0.006739	0.094447	0.071353	0.943116	0.960233	MMP24	protein_cc
ENSG0000(	1320.6	0.05857	0.070743	0.827926	0.407712	0.504552	PIM1	protein_cc
ENSG0000(	1320.557	0.525289	0.080811	6.500252	8.02E-11	6.20E-10	KDELC2	protein_cc
ENSG0000(	1320.211	-0.07327	0.036192	-2.02458	0.04291	0.073819	UBALD1	protein_cc
ENSG0000(	1320.171	-0.07395	0.029023	-2.54816	0.010829	0.021472	C6orf47	protein_cc
ENSG0000(	1320.076	-0.44769	0.088583	-5.05388	4.33E-07	1.85E-06	PLK2	protein_cc
ENSG0000(	1319.901	0.040121	0.029721	1.349905	0.177046	0.251741	ATG16L1	protein_cc
ENSG0000(	1319.776	-0.21599	0.047701	-4.52801	5.95E-06	2.13E-05	BEND6	protein_cc
ENSG0000(	1319.45	-0.03789	0.033569	-1.12856	0.259082	0.347136	ARSB	protein_cc
ENSG0000(	1318.789	0.230995	0.052153	4.429158	9.46E-06	3.28E-05	MZF1	protein_cc
ENSG0000(	1318.511	0.27656	0.110223	2.509082	0.012105	0.023729	ECM2	protein_cc
ENSG0000(	1318.379	-0.19373	0.049151	-3.9416	8.09E-05	0.000241	KLHL28	protein_cc
ENSG0000(	1318.208	0.329521	0.033958	9.703714	2.91E-22	1.48E-20	SBDSP1	pseudoge
ENSG0000(	1318.151	-0.02304	0.031962	-0.72086	0.470993	0.566921	AC093323	lincRNA
ENSG0000(	1317.959	-0.14431	0.031688	-4.554	5.26E-06	1.90E-05	TMEM242	protein_cc
ENSG0000(	1317.86	-0.14981	0.02947	-5.08363	3.70E-07	1.60E-06	ZBTB11	protein_cc
ENSG0000(	1317.386	-0.07128	0.040253	-1.77067	0.076615	0.122601	SERGEF	protein_cc
ENSG0000(	1316.802	0.06709	0.023854	2.812547	0.004915	0.010489	COG7	protein_cc
ENSG0000(	1316.417	-0.0008	0.078375	-0.01019	0.991872	0.994435	NQO1	protein_cc

ENSG0000(	1315.982	-0.03496	0.048011	-0.72807	0.466568	0.562426	RNFT2	protein_cc
ENSG0000(	1315.649	-0.15863	0.037903	-4.18523	2.85E-05	9.12E-05	EML1	protein_cc
ENSG0000(	1315.441	-0.63555	0.069374	-9.16121	5.13E-20	1.76E-18	NBL1	protein_cc
ENSG0000(	1315.269	0.266945	0.058958	4.527683	5.96E-06	2.14E-05	DRG2	protein_cc
ENSG0000(	1314.957	-0.37459	0.063654	-5.88474	3.99E-09	2.36E-08	DBP	protein_cc
ENSG0000(	1314.729	0.039434	0.063595	0.620092	0.535197	0.626408	RP11-263	antisense
ENSG0000(	1314.589	-0.194	0.046573	-4.16539	3.11E-05	9.90E-05	BRI3BP	protein_cc
ENSG0000(	1314.505	-0.30361	0.073397	-4.13645	3.53E-05	0.000111	CGREF1	protein_cc
ENSG0000(	1314.454	-0.34462	0.065923	-5.22767	1.72E-07	7.83E-07	JADE3	protein_cc
ENSG0000(	1313.68	-0.15416	0.034232	-4.50344	6.69E-06	2.37E-05	ECD	protein_cc
ENSG0000(	1313.632	-0.70557	0.094396	-7.4746	7.74E-14	9.61E-13	KCNC1	protein_cc
ENSG0000(	1313.414	0.144947	0.026512	5.467197	4.57E-08	2.29E-07	SREK1IP1	protein_cc
ENSG0000(	1313.133	-0.02979	0.036926	-0.80686	0.419746	0.516661	USP13	protein_cc
ENSG0000(	1312.366	-0.12679	0.059851	-2.11836	0.034145	0.06026	HSPA4L	protein_cc
ENSG0000(	1312.294	0.377746	0.039875	9.473182	2.71E-21	1.16E-19	TMEM218	protein_cc
ENSG0000(	1311.984	-0.06184	0.027935	-2.21385	0.026839	0.048546	DIS3L2	protein_cc
ENSG0000(	1311.949	1.073544	0.09043	11.87156	1.66E-32	6.07E-30	ARAP3	protein_cc
ENSG0000(	1310.492	-0.3056	0.035215	-8.67816	4.02E-18	1.02E-16	FAM160B1	protein_cc
ENSG0000(	1310.488	0.012422	0.04941	0.251406	0.8015	0.853334	AMZ2P1	pseudoge
ENSG0000(	1310.487	0.001479	0.067159	0.022029	0.982425	0.987863	1-Mar	protein_cc
ENSG0000(	1309.976	0.020538	0.035984	0.570759	0.568163	0.656121	RLF	protein_cc
ENSG0000(	1309.966	0.293787	0.041032	7.159997	8.07E-13	8.51E-12	TIMM10	protein_cc
ENSG0000(	1309.914	0.387972	0.109688	3.537056	0.000405	0.001063	ERAP2	protein_cc
ENSG0000(	1309.733	0.801834	0.113466	7.06674	1.59E-12	1.60E-11	KIAA0040	protein_cc
ENSG0000(	1309.729	-0.11848	0.050647	-2.33938	0.019316	0.036082	DHRS4-AS	processed
ENSG0000(	1309.439	0.216334	0.033811	6.398342	1.57E-10	1.16E-09	DHRS7B	protein_cc
ENSG0000(	1309.231	-0.03244	0.078795	-0.41173	0.680535	0.754064	SOX5	protein_cc
ENSG0000(	1309.121	-0.21976	0.099323	-2.21256	0.026928	0.048672	CNN2	protein_cc
ENSG0000(	1308.519	0.446939	0.057619	7.756747	8.71E-15	1.29E-13	TP53I13	protein_cc
ENSG0000(	1308.44	-1.03869	0.126909	-8.1845	2.73E-16	5.17E-15	GABRD	protein_cc
ENSG0000(	1308.333	-0.09808	0.06028	-1.62701	0.103736	0.159448	PPFIBP2	protein_cc
ENSG0000(	1308	0.215552	0.032683	6.595163	4.25E-11	3.43E-10	PIGC	protein_cc
ENSG0000(	1307.995	0.221675	0.046775	4.739203	2.15E-06	8.23E-06	ZNF677	protein_cc
ENSG0000(	1307.731	-0.73926	0.074238	-9.95792	2.33E-23	1.50E-21	PPM1H	protein_cc
ENSG0000(	1307.615	-0.33533	0.069826	-4.80232	1.57E-06	6.15E-06	FAXC	protein_cc
ENSG0000(	1307.153	0.050015	0.037524	1.332881	0.182571	0.258301	ATP11B	protein_cc
ENSG0000(	1306.852	-0.00373	0.036312	-0.10265	0.918241	0.942324	C11orf30	protein_cc
ENSG0000(	1306.363	0.070665	0.030414	2.323403	0.020158	0.037494	RPUSD4	protein_cc
ENSG0000(	1306.289	-0.60846	0.07705	-7.89691	2.86E-15	4.59E-14	CXCL12	protein_cc
ENSG0000(	1305.968	0.056315	0.047715	1.180248	0.237902	0.323057	GCAT	protein_cc
ENSG0000(	1305.599	0.191549	0.0406	4.717952	2.38E-06	9.07E-06	MED8	protein_cc
ENSG0000(	1305.591	0.307778	0.040908	7.523682	5.33E-14	6.80E-13	INTS7	protein_cc
ENSG0000(	1305.396	-0.20821	0.094718	-2.19824	0.027932	0.050283	PAPLN	protein_cc
ENSG0000(	1305.141	-0.14773	0.033026	-4.47295	7.71E-06	2.71E-05	DNAJC16	protein_cc
ENSG0000(	1304.938	0.017205	0.094414	0.182233	0.8554	0.895523	GLIPR1	protein_cc
ENSG0000(	1304.889	-0.13122	0.041898	-3.1319	0.001737	0.004055	TXNDC16	protein_cc
ENSG0000(	1304.515	-0.27818	0.048033	-5.79142	6.98E-09	3.97E-08	ZBTB37	protein_cc
ENSG0000(	1304.439	-0.50825	0.104265	-4.87463	1.09E-06	4.38E-06	NTN4	protein_cc
ENSG0000(	1304.063	0.067826	0.025823	2.626599	0.008624	0.017485	CCDC22	protein_cc
ENSG0000(	1303.529	0.248259	0.037301	6.655628	2.82E-11	2.34E-10	IFT81	protein_cc
ENSG0000(	1302.809	-0.42727	0.061401	-6.95866	3.44E-12	3.29E-11	C14orf37	protein_cc
ENSG0000(	1302.751	-0.42079	0.086558	-4.86135	1.17E-06	4.66E-06	LINC0063	pseudoge
ENSG0000(	1302.424	-0.07276	0.038229	-1.90318	0.057017	0.094765	ALDH1B1	protein_cc
ENSG0000(	1302.202	0.187769	0.043282	4.338267	1.44E-05	4.83E-05	PSMD5	protein_cc
ENSG0000(	1301.992	-0.15971	0.087317	-1.82904	0.067393	0.109627	CD37	protein_cc
ENSG0000(	1301.9	0.235091	0.035548	6.613391	3.76E-11	3.06E-10	ZSWIM7	protein_cc
ENSG0000(	1301.305	0.000338	0.10788	0.003132	0.997501	0.998444	RORB	protein_cc
ENSG0000(	1301.29	0.234798	0.03272	7.175889	7.18E-13	7.64E-12	RPUSD1	protein_cc
ENSG0000(	1300.793	0.343582	0.05331	6.445028	1.16E-10	8.73E-10	EFNB1	protein_cc

ENSG0000(	1300.509	-0.47082	0.051753	-9.09747	9.25E-20	3.03E-18	GAREM	protein_cc
ENSG0000(	1300.372	-0.83359	0.125439	-6.64538	3.02E-11	2.49E-10	CAMK4	protein_cc
ENSG0000(	1300.064	0.332405	0.051852	6.410636	1.45E-10	1.08E-09	GALK1	protein_cc
ENSG0000(	1299.532	0.111648	0.035953	3.105378	0.0019	0.004403	SMC6	protein_cc
ENSG0000(	1297.968	-0.52316	0.079257	-6.60083	4.09E-11	3.31E-10	PLCL1	protein_cc
ENSG0000(	1297.7	-0.14993	0.03907	-3.83736	0.000124	0.000357	CSGALNA	protein_cc
ENSG0000(	1297.685	-0.03193	0.018827	-1.6959	0.089904	0.140894	CCDC43	protein_cc
ENSG0000(	1297.321	1.065665	0.101856	10.46245	1.28E-25	1.28E-23	TYMS	protein_cc
ENSG0000(	1297.225	0.293341	0.038771	7.565908	3.85E-14	5.03E-13	HMBS	protein_cc
ENSG0000(	1297.028	0.173894	0.032228	5.395802	6.82E-08	3.32E-07	STK11IP	protein_cc
ENSG0000(	1296.033	-0.0342	0.027928	-1.22457	0.220738	0.303377	ATP9B	protein_cc
ENSG0000(	1295.899	0.217358	0.038495	5.646383	1.64E-08	8.79E-08	RRP9	protein_cc
ENSG0000(	1295.489	0.561447	0.058504	9.596756	8.25E-22	3.83E-20	RBBP8	protein_cc
ENSG0000(	1295.457	0.038165	0.035621	1.071406	0.283987	0.374689	LYPLAL1	protein_cc
ENSG0000(	1295.329	-0.13267	0.053931	-2.45992	0.013897	0.026879	CBLB	protein_cc
ENSG0000(	1294.976	-0.08216	0.024765	-3.31761	0.000908	0.002229	TRIM35	protein_cc
ENSG0000(	1294.875	-0.1165	0.023871	-4.88056	1.06E-06	4.26E-06	VPS37C	protein_cc
ENSG0000(	1294.771	-0.04731	0.043454	-1.08868	0.276297	0.366436	SARM1	protein_cc
ENSG0000(	1294.22	0.163312	0.037375	4.369508	1.25E-05	4.23E-05	GTF2B	protein_cc
ENSG0000(	1294.197	0.122586	0.029755	4.119761	3.79E-05	0.000119	GEMIN4	protein_cc
ENSG0000(	1293.897	-0.26319	0.08785	-2.99592	0.002736	0.006143	PHYHD1	protein_cc
ENSG0000(	1293.589	0.490613	0.057192	8.578372	9.62E-18	2.32E-16	NCAPG2	protein_cc
ENSG0000(	1293.526	-0.46648	0.072587	-6.42655	1.31E-10	9.78E-10	RND1	protein_cc
ENSG0000(	1293.511	0.117176	0.040883	2.866114	0.004155	0.009011	SLC35E3	protein_cc
ENSG0000(	1292.51	-0.37839	0.063557	-5.95351	2.62E-09	1.60E-08	FHOD3	protein_cc
ENSG0000(	1292.354	-0.37187	0.050553	-7.35607	1.89E-13	2.20E-12	RAB3IL1	protein_cc
ENSG0000(	1292.312	-0.02775	0.03024	-0.91764	0.358806	0.454605	C12orf43	protein_cc
ENSG0000(	1292.194	0.176691	0.038982	4.532585	5.83E-06	2.09E-05	MPG	protein_cc
ENSG0000(	1291.931	-0.53781	0.070897	-7.58577	3.31E-14	4.36E-13	FAM169A	protein_cc
ENSG0000(	1291.786	0.102992	0.062614	1.644881	0.099994	0.154529	DISC1	protein_cc
ENSG0000(	1291.714	-0.35138	0.05148	-6.8256	8.76E-12	7.84E-11	RIPK2	protein_cc
ENSG0000(	1291.682	-0.05815	0.058335	-0.99677	0.318877	0.412581	SMTN	protein_cc
ENSG0000(	1291.278	-0.95266	0.106476	-8.94722	3.65E-19	1.08E-17	STOX1	protein_cc
ENSG0000(	1291.083	0.195574	0.037096	5.272052	1.35E-07	6.26E-07	PHTF2	protein_cc
ENSG0000(	1290.706	0.164253	0.038086	4.312708	1.61E-05	5.38E-05	DPH5	protein_cc
ENSG0000(	1290.335	-0.02789	0.028008	-0.99592	0.319289	0.413011	GFM2	protein_cc
ENSG0000(	1290.314	-0.09945	0.034863	-2.85252	0.004337	0.009361	COA7	protein_cc
ENSG0000(	1290.219	-0.04457	0.026468	-1.68388	0.092204	0.143978	SMNDC1	protein_cc
ENSG0000(	1289.848	-1.59798	0.150814	-10.5957	3.12E-26	3.49E-24	RBFOX1	protein_cc
ENSG0000(	1289.685	-0.80782	0.079916	-10.1085	5.07E-24	3.71E-22	LINC0008(	lincRNA
ENSG0000(	1289.314	0.030963	0.036071	0.858379	0.390683	0.48729	COQ10B	protein_cc
ENSG0000(	1289.182	0.235574	0.033255	7.083958	1.40E-12	1.42E-11	C7orf49	protein_cc
ENSG0000(	1288.768	0.069672	0.092452	0.753596	0.451092	0.547816	NR2F2	protein_cc
ENSG0000(	1288.648	0.203908	0.045725	4.45948	8.22E-06	2.87E-05	ATAD3A	protein_cc
ENSG0000(	1288.631	0.443669	0.069714	6.364084	1.96E-10	1.43E-09	EPHB4	protein_cc
ENSG0000(	1288.269	0.042805	0.041894	1.021737	0.306905	0.399955	ZFP3	protein_cc
ENSG0000(	1288.088	-0.4692	0.075315	-6.22974	4.67E-10	3.21E-09	ADHFE1	protein_cc
ENSG0000(	1287.805	-0.284	0.10151	-2.79777	0.005146	0.010941	LAMP5	protein_cc
ENSG0000(	1287.7	0.090176	0.071617	1.259137	0.207981	0.288664	MR1	protein_cc
ENSG0000(	1287.489	0.149707	0.026573	5.63369	1.76E-08	9.42E-08	GFER	protein_cc
ENSG0000(	1287.045	0.162792	0.036182	4.499231	6.82E-06	2.42E-05	MED21	protein_cc
ENSG0000(	1286.923	0.031948	0.03011	1.061023	0.28868	0.380003	NOA1	protein_cc
ENSG0000(	1286.795	-0.18983	0.030476	-6.22891	4.70E-10	3.22E-09	TTLL5	protein_cc
ENSG0000(	1286.174	0.472629	0.122174	3.868485	0.00011	0.000318	SEL1L3	protein_cc
ENSG0000(	1285.204	0.310266	0.079949	3.88081	0.000104	0.000303	GLRX	protein_cc
ENSG0000(	1285.163	-0.39205	0.052712	-7.43756	1.03E-13	1.24E-12	VSTM4	protein_cc
ENSG0000(	1285.061	0.099444	0.031573	3.149664	0.001635	0.003834	SRR	protein_cc
ENSG0000(	1284.964	0.239661	0.026775	8.950881	3.53E-19	1.05E-17	ZNF426	protein_cc
ENSG0000(	1284.695	-0.31622	0.049351	-6.40756	1.48E-10	1.09E-09	C17orf51	protein_cc

ENSG0000	1283.872	0.152891	0.046995	3.253321	0.001141	0.00275	SMIM20	protein_cc
ENSG0000	1283.742	0.171657	0.028561	6.010205	1.85E-09	1.16E-08	TIMM22	protein_cc
ENSG0000	1283.4	-0.04962	0.087125	-0.56954	0.568989	0.65693	GYPC	protein_cc
ENSG0000	1283.287	0.095016	0.023563	4.032513	5.52E-05	0.000169	FAM122A	protein_cc
ENSG0000	1283.039	0.02982	0.026741	1.115128	0.264796	0.353542	CTC-228N	lincRNA
ENSG0000	1282.958	-0.2635	0.041382	-6.36759	1.92E-10	1.40E-09	DGKQ	protein_cc
ENSG0000	1282.175	0.168468	0.112867	1.492627	0.135535	0.200424	FAM129A	protein_cc
ENSG0000	1281.885	-0.07754	0.029801	-2.60186	0.009272	0.018658	INTS6	protein_cc
ENSG0000	1281.514	0.062501	0.034457	1.813904	0.069692	0.112889	FAM203A	protein_cc
ENSG0000	1280.575	-0.32876	0.077409	-4.24712	2.17E-05	7.08E-05	DRP2	protein_cc
ENSG0000	1280.356	-1.01958	0.139677	-7.29956	2.89E-13	3.26E-12	C12orf39	protein_cc
ENSG0000	1280.112	0.269736	0.03646	7.398225	1.38E-13	1.64E-12	PMF1	protein_cc
ENSG0000	1279.919	0.569041	0.097312	5.847617	4.99E-09	2.90E-08	UPP1	protein_cc
ENSG0000	1279.829	-0.16093	0.047879	-3.36122	0.000776	0.00193	ADC	protein_cc
ENSG0000	1279.828	0.089946	0.030944	2.906733	0.003652	0.008003	ZNF529	protein_cc
ENSG0000	1279.758	0.160844	0.034625	4.645262	3.40E-06	1.26E-05	COX16	protein_cc
ENSG0000	1279.678	-0.35927	0.122285	-2.93801	0.003303	0.007301	SHISA9	protein_cc
ENSG0000	1279.549	-0.22031	0.069623	-3.16425	0.001555	0.003661	GIMAP4	protein_cc
ENSG0000	1279.518	0.123433	0.033203	3.717519	0.000201	0.000557	C7orf43	protein_cc
ENSG0000	1279.405	0.271309	0.040468	6.704243	2.02E-11	1.71E-10	IFRD2	protein_cc
ENSG0000	1278.536	-0.02257	0.039311	-0.57405	0.565936	0.653954	ZNF510	protein_cc
ENSG0000	1278.283	-0.3143	0.063071	-4.98333	6.25E-07	2.61E-06	RPS6KL1	protein_cc
ENSG0000	1278.059	0.036482	0.024719	1.475829	0.13999	0.206106	CWC22	protein_cc
ENSG0000	1277.613	-0.30727	0.050623	-6.06974	1.28E-09	8.22E-09	FGD4	protein_cc
ENSG0000	1277.481	0.199049	0.034057	5.84461	5.08E-09	2.95E-08	RBAK	protein_cc
ENSG0000	1276.732	0.087191	0.031721	2.748718	0.005983	0.012534	CCDC86	protein_cc
ENSG0000	1276.04	0.010601	0.081234	0.130501	0.89617	0.926262	PSMD5-A	antisense
ENSG0000	1275.951	0.367645	0.08783	4.185872	2.84E-05	9.10E-05	IRF1	protein_cc
ENSG0000	1275.652	-0.28313	0.08612	-3.28757	0.001011	0.00246	EDNRA	protein_cc
ENSG0000	1275.098	0.333841	0.06499	5.13679	2.79E-07	1.23E-06	POLE4	protein_cc
ENSG0000	1274.997	0.200604	0.073371	2.734094	0.006255	0.013058	FRMD3	protein_cc
ENSG0000	1274.827	0.260755	0.131234	1.986938	0.046929	0.079854	PCOLCE	protein_cc
ENSG0000	1274.504	-0.15829	0.033335	-4.74842	2.05E-06	7.89E-06	TAB3	protein_cc
ENSG0000	1274.01	0.793627	0.168531	4.709076	2.49E-06	9.45E-06	RARRES2	protein_cc
ENSG0000	1273.809	0.057042	0.03632	1.570533	0.116291	0.175997	NIM1K	protein_cc
ENSG0000	1273.578	-0.10549	0.057838	-1.8238	0.068182	0.110712	ZC4H2	protein_cc
ENSG0000	1273.467	0.363449	0.080196	4.532025	5.84E-06	2.10E-05	ITGA1	protein_cc
ENSG0000	1273.223	-0.32797	0.09847	-3.33061	0.000867	0.002137	CSMD1	protein_cc
ENSG0000	1272.608	-0.17028	0.034414	-4.94787	7.50E-07	3.09E-06	CCDC149	protein_cc
ENSG0000	1272.438	-0.15783	0.082935	-1.90313	0.057024	0.094773	CSF3R	protein_cc
ENSG0000	1272.246	0.022258	0.026595	0.836926	0.402634	0.499425	FAHD2A	protein_cc
ENSG0000	1272.188	0.238815	0.068106	3.506536	0.000454	0.001181	RGS3	protein_cc
ENSG0000	1272.064	0.116023	0.02447	4.741447	2.12E-06	8.15E-06	NIF3L1	protein_cc
ENSG0000	1272.036	0.05841	0.033374	1.750152	0.080092	0.127397	ZFYVE19	protein_cc
ENSG0000	1271.931	-0.07439	0.047204	-1.57599	0.115028	0.174362	ABHD14A	protein_cc
ENSG0000	1271.003	0.008106	0.034086	0.237805	0.812033	0.86187	MSRA	protein_cc
ENSG0000	1270.974	0.028733	0.0305	0.942069	0.346157	0.441585	SLC2A8	protein_cc
ENSG0000	1270.721	0.34002	0.063164	5.383099	7.32E-08	3.54E-07	THBS3	protein_cc
ENSG0000	1270.466	0.664468	0.080586	8.245461	1.65E-16	3.23E-15	RP11-620.	lincRNA
ENSG0000	1270.455	0.222918	0.040252	5.538086	3.06E-08	1.57E-07	ALKBH2	protein_cc
ENSG0000	1270.401	-0.24088	0.038641	-6.2339	4.55E-10	3.13E-09	MTF1	protein_cc
ENSG0000	1269.975	-0.6091	0.140663	-4.33024	1.49E-05	5.00E-05	SCRT1	protein_cc
ENSG0000	1269.424	0.482489	0.049939	9.661589	4.39E-22	2.15E-20	C19orf24	protein_cc
ENSG0000	1269.374	0.048117	0.021812	2.206025	0.027382	0.049398	COQ7	protein_cc
ENSG0000	1269.351	0.241434	0.032781	7.365096	1.77E-13	2.07E-12	FAM136A	protein_cc
ENSG0000	1268.62	0.994959	0.107459	9.258949	2.06E-20	7.49E-19	KLHL4	protein_cc
ENSG0000	1268.616	0.203133	0.027799	7.307281	2.73E-13	3.09E-12	RAD1	protein_cc
ENSG0000	1268.599	-0.28337	0.101273	-2.79808	0.005141	0.010932	GRID2	protein_cc
ENSG0000	1268.235	0.137681	0.035451	3.883752	0.000103	0.0003	CMSS1	protein_cc

ENSG0000(	1267.98	0.045618	0.044065	1.035247	0.300553	0.393041	SELO	protein_cc
ENSG0000(	1266.476	0.198237	0.02648	7.486315	7.08E-14	8.87E-13	FASTKD2	protein_cc
ENSG0000(	1266.416	-0.00788	0.029146	-0.27032	0.786911	0.841543	DHX32	protein_cc
ENSG0000(	1265.223	0.124146	0.053032	2.340949	0.019235	0.035948	SNX7	protein_cc
ENSG0000(	1265.086	0.43615	0.044245	9.857722	6.35E-23	3.69E-21	ALG5	protein_cc
ENSG0000(	1265.058	0.219901	0.057649	3.81451	0.000136	0.000389	ACOT9	protein_cc
ENSG0000(	1265.046	0.00757	0.034894	0.216952	0.828246	0.874227	HSF2	protein_cc
ENSG0000(	1264.739	-0.1427	0.045102	-3.16381	0.001557	0.003667	ME3	protein_cc
ENSG0000(	1264.681	0.017424	0.027968	0.622996	0.533287	0.624693	ACSF3	protein_cc
ENSG0000(	1264.453	-0.23603	0.055026	-4.28945	1.79E-05	5.94E-05	CEP290	protein_cc
ENSG0000(	1263.852	-0.06934	0.116698	-0.59416	0.552402	0.642122	EYA1	protein_cc
ENSG0000(	1263.731	0.054442	0.041173	1.322262	0.186081	0.262581	SNX21	protein_cc
ENSG0000(	1263.66	0.287895	0.040661	7.080304	1.44E-12	1.46E-11	PHF5A	protein_cc
ENSG0000(	1263.633	0.400608	0.120747	3.317764	0.000907	0.002228	C8orf4	protein_cc
ENSG0000(	1263.587	-0.06785	0.110336	-0.61495	0.538588	0.629605	GMPR	protein_cc
ENSG0000(	1263.535	0.084245	0.046775	1.801057	0.071694	0.115685	FKBP14	protein_cc
ENSG0000(	1262.878	0.261145	0.039797	6.561842	5.31E-11	4.22E-10	CDKN2AIF	protein_cc
ENSG0000(	1262.489	-0.98636	0.126906	-7.77235	7.70E-15	1.15E-13	SLC4A10	protein_cc
ENSG0000(	1262.401	-0.52207	0.128343	-4.06779	4.75E-05	0.000147	C2orf82	protein_cc
ENSG0000(	1262.305	-0.24628	0.071922	-3.42432	0.000616	0.001563	ATP10A	protein_cc
ENSG0000(	1261.691	-0.00243	0.05449	-0.04466	0.964375	0.97562	LGALS8	protein_cc
ENSG0000(	1261.322	0.129586	0.042007	3.084882	0.002036	0.004693	FAM69B	protein_cc
ENSG0000(	1261.244	0.343101	0.046587	7.364663	1.78E-13	2.07E-12	MRPL13	protein_cc
ENSG0000(	1260.954	-0.2134	0.045105	-4.73129	2.23E-06	8.54E-06	VWA8	protein_cc
ENSG0000(	1260.857	0.10433	0.03457	3.017952	0.002545	0.005761	FBXO8	protein_cc
ENSG0000(	1260.855	-0.18506	0.12934	-1.43083	0.15248	0.22196	ACTL6B	protein_cc
ENSG0000(	1260.668	-0.06789	0.052488	-1.29336	0.195888	0.274386	ZNF331	protein_cc
ENSG0000(	1260.356	-0.09168	0.039625	-2.31378	0.02068	0.038369	NPAT	protein_cc
ENSG0000(	1260.25	-1.06946	0.143422	-7.45675	8.87E-14	1.09E-12	GNG3	protein_cc
ENSG0000(	1258.972	0.269097	0.087805	3.064699	0.002179	0.00499	CPQ	protein_cc
ENSG0000(	1258.933	0.15993	0.040043	3.993924	6.50E-05	0.000196	CLN6	protein_cc
ENSG0000(	1258.751	0.041442	0.041688	0.994092	0.320178	0.413966	AIDA	protein_cc
ENSG0000(	1258.684	0.246018	0.081736	3.0099	0.002613	0.0059	SERPINB1	protein_cc
ENSG0000(	1258.596	0.111514	0.049065	2.272795	0.023039	0.042325	MINOS1	protein_cc
ENSG0000(	1257.735	-0.08421	0.035151	-2.39571	0.016588	0.031512	EDEM1	protein_cc
ENSG0000(	1257.717	-0.00505	0.033103	-0.15271	0.878631	0.913229	NHLRC3	protein_cc
ENSG0000(	1257.309	-0.07946	0.060666	-1.30982	0.190258	0.267567	PHYH	protein_cc
ENSG0000(	1256.872	-0.42045	0.065936	-6.37658	1.81E-10	1.32E-09	GS1-358P	sense_ove
ENSG0000(	1256.529	0.251633	0.100859	2.494907	0.012599	0.024585	POU3F4	protein_cc
ENSG0000(	1256.381	-1.57875	0.166382	-9.48872	2.34E-21	1.01E-19	HPSE2	protein_cc
ENSG0000(	1256.171	0.012295	0.066848	0.183922	0.854075	0.894511	KDR	protein_cc
ENSG0000(	1256.162	0.08622	0.037617	2.29207	0.021902	0.040422	ZNF844	protein_cc
ENSG0000(	1255.924	0.068835	0.035954	1.91454	0.055551	0.092651	KDM6A	protein_cc
ENSG0000(	1255.908	0.000213	0.044208	0.004816	0.996157	0.997491	LRTOMT	protein_cc
ENSG0000(	1255.664	0.185945	0.066693	2.788058	0.005303	0.011245	ISYNA1	protein_cc
ENSG0000(	1255.318	-0.3374	0.110402	-3.05611	0.002242	0.005126	OLFML2B	protein_cc
ENSG0000(	1255.041	0.179347	0.038972	4.601915	4.19E-06	1.54E-05	GGCT	protein_cc
ENSG0000(	1254.97	0.070961	0.029759	2.384575	0.017099	0.032359	RP5-874C	pseudoge
ENSG0000(	1253.732	0.118621	0.055366	2.142497	0.032154	0.057086	KLHL13	protein_cc
ENSG0000(	1253.542	0.050941	0.035479	1.435804	0.151058	0.220147	OPA3	protein_cc
ENSG0000(	1253.4	0.175389	0.03861	4.542538	5.56E-06	2.00E-05	C12orf4	protein_cc
ENSG0000(	1253.167	-0.21288	0.059939	-3.55163	0.000383	0.00101	RNF208	protein_cc
ENSG0000(	1252.997	-0.00013	0.039551	-0.0033	0.997371	0.998357	ZBED4	protein_cc
ENSG0000(	1252.922	-0.38492	0.066746	-5.76702	8.07E-09	4.54E-08	KIAA1324l	protein_cc
ENSG0000(	1252.763	-0.05465	0.045152	-1.21027	0.226176	0.309609	ZNF605	protein_cc
ENSG0000(	1252.678	-0.55373	0.053148	-10.4187	2.04E-25	1.93E-23	ZRANB1	protein_cc
ENSG0000(	1252.625	0.229551	0.074173	3.094816	0.001969	0.004548	NLRC5	protein_cc
ENSG0000(	1252.401	0.20625	0.033318	6.19029	6.01E-10	4.05E-09	CNPY2	protein_cc
ENSG0000(	1252.284	0.057199	0.02961	1.931724	0.053394	0.089506	FAM92A1	protein_cc

ENSG0000(	1251.814	0.313987	0.052525	5.977906	2.26E-09	1.39E-08	REXO2	protein_cc
ENSG0000(	1251.644	0.152244	0.032383	4.701355	2.58E-06	9.78E-06	DPH2	protein_cc
ENSG0000(	1250.971	0.169737	0.034301	4.948511	7.48E-07	3.08E-06	SLC37A4	protein_cc
ENSG0000(	1250.884	0.001981	0.046897	0.042233	0.966313	0.97682	TTC28-AS	processed
ENSG0000(	1250.335	0.457291	0.055713	8.207915	2.25E-16	4.32E-15	ARNTL	protein_cc
ENSG0000(	1249.85	0.009273	0.040251	0.230385	0.817793	0.866441	FBXW8	protein_cc
ENSG0000(	1249.672	-0.34324	0.089343	-3.84184	0.000122	0.000351	SORCS3	protein_cc
ENSG0000(	1248.536	-0.52712	0.064431	-8.18112	2.81E-16	5.31E-15	WDFY3-A	processed
ENSG0000(	1248.53	0.41194	0.052049	7.914508	2.48E-15	4.01E-14	EDEM2	protein_cc
ENSG0000(	1248.489	-0.00137	0.039853	-0.03437	0.972582	0.980874	MRPS28	protein_cc
ENSG0000(	1247.567	-0.02438	0.028612	-0.85208	0.39417	0.490673	RNF8	protein_cc
ENSG0000(	1247.386	-0.30173	0.068941	-4.37655	1.21E-05	4.11E-05	FAM101B	protein_cc
ENSG0000(	1246.463	-0.08764	0.041622	-2.10564	0.035236	0.06201	CHM	protein_cc
ENSG0000(	1246.426	-0.10486	0.024617	-4.25962	2.05E-05	6.73E-05	VPS33B	protein_cc
ENSG0000(	1246.275	0.127938	0.045849	2.790404	0.005264	0.011172	ANKS3	protein_cc
ENSG0000(	1246.188	-0.24627	0.027695	-8.89241	5.98E-19	1.72E-17	CHUK	protein_cc
ENSG0000(	1245.858	-1.28576	0.124535	-10.3244	5.46E-25	4.77E-23	SNCG	protein_cc
ENSG0000(	1245.386	-0.34594	0.057903	-5.97449	2.31E-09	1.42E-08	GALNT18	protein_cc
ENSG0000(	1244.619	-0.09918	0.031949	-3.10426	0.001908	0.004418	SUPV3L1	protein_cc
ENSG0000(	1244.378	0.016389	0.030411	0.538912	0.589947	0.675818	PPP2R1B	protein_cc
ENSG0000(	1243.865	-0.10688	0.037458	-2.85339	0.004326	0.009337	RAB39B	protein_cc
ENSG0000(	1243.766	0.120702	0.057434	2.101584	0.03559	0.062552	PCBD1	protein_cc
ENSG0000(	1243.619	0.090592	0.022403	4.043688	5.26E-05	0.000161	NRF1	protein_cc
ENSG0000(	1243.574	0.313256	0.036426	8.599845	7.98E-18	1.94E-16	MED27	protein_cc
ENSG0000(	1243.417	0.528817	0.047796	11.06413	1.87E-28	3.21E-26	DHRSX	protein_cc
ENSG0000(	1242.836	0.135111	0.022809	5.923565	3.15E-09	1.90E-08	CNOT10	protein_cc
ENSG0000(	1242.771	0.012383	0.021408	0.578432	0.562972	0.651598	PKNOX1	protein_cc
ENSG0000(	1242.595	-0.21477	0.080661	-2.66261	0.007754	0.015887	LPCAT2	protein_cc
ENSG0000(	1242.56	0.095092	0.035981	2.642845	0.008221	0.016756	CAPN10	protein_cc
ENSG0000(	1242.371	0.115673	0.028088	4.118203	3.82E-05	0.00012	RFT1	protein_cc
ENSG0000(	1241.463	-0.11196	0.047701	-2.34703	0.018924	0.035417	SCHIP1	protein_cc
ENSG0000(	1241.427	0.199216	0.032535	6.12322	9.17E-10	6.01E-09	ALG2	protein_cc
ENSG0000(	1241.399	0.006932	0.046036	0.150569	0.880316	0.91438	C9orf72	protein_cc
ENSG0000(	1241.339	-0.19108	0.063357	-3.01592	0.002562	0.005796	EGFL7	protein_cc
ENSG0000(	1241.119	-0.11193	0.088309	-1.26745	0.204994	0.285121	IGLON5	protein_cc
ENSG0000(	1240.943	0.219088	0.034237	6.399241	1.56E-10	1.15E-09	RNF2	protein_cc
ENSG0000(	1240.317	0.177939	0.024482	7.268193	3.64E-13	4.04E-12	NOL10	protein_cc
ENSG0000(	1239.905	0.221344	0.075733	2.922674	0.00347	0.007639	IL11RA	protein_cc
ENSG0000(	1239.165	0.180918	0.034952	5.176137	2.27E-07	1.01E-06	RASSF1	protein_cc
ENSG0000(	1239.149	0.396646	0.062309	6.365774	1.94E-10	1.41E-09	RCC1	protein_cc
ENSG0000(	1238.687	0.136921	0.03162	4.330264	1.49E-05	5.00E-05	SETD8	protein_cc
ENSG0000(	1237.954	0.015176	0.023641	0.641959	0.520899	0.613198	TAF8	protein_cc
ENSG0000(	1237.393	-0.49958	0.116278	-4.29642	1.74E-05	5.77E-05	SLITRK1	protein_cc
ENSG0000(	1236.926	0.21973	0.033639	6.532021	6.49E-11	5.09E-10	CTU2	protein_cc
ENSG0000(	1236.893	0.072552	0.034296	2.1155	0.034387	0.060659	RP4-758J1	protein_cc
ENSG0000(	1235.661	-0.03541	0.079158	-0.44738	0.654604	0.732515	LCP2	protein_cc
ENSG0000(	1235.498	0.766524	0.082881	9.248453	2.28E-20	8.21E-19	RHOJ	protein_cc
ENSG0000(	1235.303	-0.16068	0.04804	-3.34478	0.000823	0.002038	UBTD1	protein_cc
ENSG0000(	1235.132	0.137312	0.048448	2.834205	0.004594	0.009854	RP2	protein_cc
ENSG0000(	1235.079	0.397571	0.046564	8.538169	1.36E-17	3.20E-16	ZNF528	protein_cc
ENSG0000(	1234.745	0.142293	0.034955	4.070718	4.69E-05	0.000145	ZNF134	protein_cc
ENSG0000(	1233.741	0.065272	0.042914	1.521007	0.128258	0.191243	NUDT12	protein_cc
ENSG0000(	1233.654	-0.05003	0.029941	-1.67097	0.094728	0.147393	MTX2	protein_cc
ENSG0000(	1232.965	0.484878	0.075612	6.41268	1.43E-10	1.06E-09	GNG11	protein_cc
ENSG0000(	1232.763	-0.24	0.064001	-3.7499	0.000177	0.000495	MTAP	protein_cc
ENSG0000(	1232.57	-0.86554	0.109151	-7.92969	2.20E-15	3.59E-14	EMX2OS	antisense
ENSG0000(	1232.012	-0.49775	0.044929	-11.0787	1.59E-28	2.75E-26	YPEL2	protein_cc
ENSG0000(	1231.884	-0.44591	0.075688	-5.89139	3.83E-09	2.27E-08	CACNB3	protein_cc
ENSG0000(	1231.864	0.257812	0.032501	7.932345	2.15E-15	3.52E-14	TRMT10C	protein_cc

ENSG0000(	1231.718	0.012821	0.026011	0.492922	0.622068	0.704522	CXorf38	protein_cc
ENSG0000(	1231.712	-0.10549	0.039095	-2.69835	0.006968	0.014403	DOPEY2	protein_cc
ENSG0000(	1231.199	-0.27665	0.087894	-3.14751	0.001647	0.00386	PTAFR	protein_cc
ENSG0000(	1231.182	0.241071	0.037506	6.427492	1.30E-10	9.73E-10	EIF2AK3	protein_cc
ENSG0000(	1231.058	-0.19669	0.073768	-2.6664	0.007667	0.015724	CCDC71L	protein_cc
ENSG0000(	1230.467	0.275209	0.06565	4.192053	2.76E-05	8.88E-05	FHL3	protein_cc
ENSG0000(	1229.475	0.158626	0.033276	4.766966	1.87E-06	7.24E-06	FRG1	protein_cc
ENSG0000(	1229.359	0.007247	0.029431	0.246229	0.805505	0.856667	CTDP1	protein_cc
ENSG0000(	1229.26	1.335655	0.144016	9.274378	1.79E-20	6.55E-19	OCIAD2	protein_cc
ENSG0000(	1229.16	0.345602	0.043944	7.864681	3.70E-15	5.83E-14	RFWD3	protein_cc
ENSG0000(	1228.946	0.383352	0.067482	5.680826	1.34E-08	7.29E-08	IQCG	protein_cc
ENSG0000(	1228.942	-0.10599	0.025744	-4.11724	3.83E-05	0.00012	TBC1D25	protein_cc
ENSG0000(	1228.346	0.103221	0.030075	3.432139	0.000599	0.001523	TAF4	protein_cc
ENSG0000(	1228.187	-0.04326	0.047669	-0.90749	0.36415	0.460124	PSMG3-A:	lincRNA
ENSG0000(	1228.079	-0.43133	0.096168	-4.48523	7.28E-06	2.57E-05	SELENBP1	protein_cc
ENSG0000(	1227.813	0.050087	0.030422	1.646401	0.099681	0.154115	NLE1	protein_cc
ENSG0000(	1227.366	-0.03697	0.078539	-0.47068	0.637869	0.718068	CEBPB	protein_cc
ENSG0000(	1226.884	0.041457	0.09227	0.449303	0.653213	0.731348	DYNLT3	protein_cc
ENSG0000(	1226.821	0.21004	0.059391	3.536536	0.000405	0.001065	TSKU	protein_cc
ENSG0000(	1226.61	0.323557	0.038183	8.473764	2.38E-17	5.37E-16	C3orf38	protein_cc
ENSG0000(	1226.594	0.096278	0.028884	3.33331	0.000858	0.002118	INTS8	protein_cc
ENSG0000(	1226.112	0.005514	0.071672	0.076931	0.938679	0.957183	HEBP1	protein_cc
ENSG0000(	1225.972	0.290388	0.081889	3.546113	0.000391	0.00103	UNC93B1	protein_cc
ENSG0000(	1225.58	0.11895	0.034726	3.425381	0.000614	0.001557	RNASEH2I	protein_cc
ENSG0000(	1225.204	-0.01477	0.03288	-0.4491	0.653356	0.731437	KLHL8	protein_cc
ENSG0000(	1224.923	0.156302	0.043668	3.579353	0.000344	0.000917	C17orf89	protein_cc
ENSG0000(	1224.711	0.065129	0.030318	2.148211	0.031697	0.056348	RPAP3	protein_cc
ENSG0000(	1224.457	0.37949	0.046186	8.216626	2.09E-16	4.03E-15	NDC1	protein_cc
ENSG0000(	1224.387	0.239075	0.034332	6.963646	3.32E-12	3.19E-11	SIRT6	protein_cc
ENSG0000(	1223.83	-0.14101	0.059295	-2.37815	0.0174	0.032856	OSTF1	protein_cc
ENSG0000(	1223.746	0.053523	0.045001	1.189369	0.234295	0.318833	TFPT	protein_cc
ENSG0000(	1223.398	0.00792	0.04303	0.184061	0.853966	0.894442	HPS3	protein_cc
ENSG0000(	1222.703	-0.51504	0.065942	-7.81057	5.69E-15	8.66E-14	PRRG1	protein_cc
ENSG0000(	1222.379	-0.04038	0.038532	-1.04791	0.294679	0.386499	ZBTB21	protein_cc
ENSG0000(	1221.943	0.151871	0.037805	4.017206	5.89E-05	0.000179	APOO	protein_cc
ENSG0000(	1221.611	0.231982	0.077381	2.997927	0.002718	0.006107	FZD1	protein_cc
ENSG0000(	1220.951	0.220966	0.024851	8.891657	6.02E-19	1.74E-17	GTPBP10	protein_cc
ENSG0000(	1220.823	-0.03804	0.041795	-0.91009	0.362776	0.458716	MOSPD2	protein_cc
ENSG0000(	1220.578	0.177583	0.028385	6.256302	3.94E-10	2.73E-09	METTL2A	protein_cc
ENSG0000(	1220.317	-0.09665	0.024926	-3.87766	0.000105	0.000307	WDR20	protein_cc
ENSG0000(	1219.501	-0.07903	0.034014	-2.32336	0.02016	0.037496	FAM189B	protein_cc
ENSG0000(	1219.158	-0.26566	0.049084	-5.41247	6.22E-08	3.05E-07	C5orf30	protein_cc
ENSG0000(	1218.972	-0.11137	0.03712	-3.0002	0.002698	0.006069	ZC3H6	protein_cc
ENSG0000(	1218.339	-0.25138	0.067611	-3.71807	0.000201	0.000556	USP53	protein_cc
ENSG0000(	1217.902	0.206676	0.022711	9.100369	9.00E-20	2.96E-18	UIMC1	protein_cc
ENSG0000(	1217.475	0.252144	0.037615	6.703241	2.04E-11	1.72E-10	RARS2	protein_cc
ENSG0000(	1217.393	0.056008	0.040836	1.371537	0.170208	0.243601	ZDHHC18	protein_cc
ENSG0000(	1215.97	0.147768	0.036547	4.043199	5.27E-05	0.000162	BBS9	protein_cc
ENSG0000(	1215.796	0.064453	0.054376	1.185315	0.235893	0.3207	RAI14	protein_cc
ENSG0000(	1215.273	0.652193	0.114617	5.690206	1.27E-08	6.92E-08	DPYD	protein_cc
ENSG0000(	1215.144	-0.41898	0.080521	-5.2033	1.96E-07	8.86E-07	DOCK2	protein_cc
ENSG0000(	1214.974	0.403585	0.055231	7.307239	2.73E-13	3.09E-12	DHFR	protein_cc
ENSG0000(	1214.398	-0.15201	0.108463	-1.40147	0.161074	0.232375	PPP1R14A	protein_cc
ENSG0000(	1214.379	-0.15073	0.072639	-2.07504	0.037983	0.066279	GPR176	protein_cc
ENSG0000(	1214.052	-0.31896	0.056115	-5.68405	1.32E-08	7.16E-08	ZNF248	protein_cc
ENSG0000(	1214.003	0.182178	0.032261	5.647076	1.63E-08	8.76E-08	AKIP1	protein_cc
ENSG0000(	1213.71	-0.0107	0.025385	-0.42141	0.673456	0.748216	LRRC57	protein_cc
ENSG0000(	1213.49	0.184146	0.029517	6.238698	4.41E-10	3.04E-09	SLC30A6	protein_cc
ENSG0000(	1212.922	-0.08739	0.029819	-2.93059	0.003383	0.007463	SOCS5	protein_cc

ENSG0000(	1212.8	-0.10178	0.04357	-2.33612	0.019485	0.036366	UNKL	protein_cc
ENSG0000(	1212.32	0.296139	0.042846	6.911705	4.79E-12	4.46E-11	EBP	protein_cc
ENSG0000(	1212.182	0.121418	0.032521	3.733485	0.000189	0.000526	ASUN	protein_cc
ENSG0000(	1212.114	0.145951	0.035518	4.109255	3.97E-05	0.000124	C7orf60	protein_cc
ENSG0000(	1211.605	0.177979	0.040615	4.382137	1.18E-05	4.01E-05	DYRK4	protein_cc
ENSG0000(	1211.075	0.424879	0.088326	4.810358	1.51E-06	5.92E-06	SP100	protein_cc
ENSG0000(	1211.067	0.127498	0.029405	4.335846	1.45E-05	4.88E-05	OTUD6B	protein_cc
ENSG0000(	1210.715	-0.21522	0.036917	-5.82987	5.55E-09	3.20E-08	TANGO2	protein_cc
ENSG0000(	1210.301	0.00625	0.099514	0.062808	0.949919	0.965365	MYO16	protein_cc
ENSG0000(	1208.429	-0.01529	0.045611	-0.33527	0.737423	0.801336	CHCHD6	protein_cc
ENSG0000(	1208.314	0.073559	0.03511	2.095073	0.036164	0.063459	WBP1	protein_cc
ENSG0000(	1207.876	-0.09533	0.092696	-1.02843	0.303748	0.396575	CCDC3	protein_cc
ENSG0000(	1207.8	-0.00627	0.030768	-0.20372	0.838574	0.882569	MECR	protein_cc
ENSG0000(	1207.74	-0.22647	0.057493	-3.93909	8.18E-05	0.000243	LRRC1	protein_cc
ENSG0000(	1207.633	0.120893	0.025592	4.723831	2.31E-06	8.84E-06	LLPH	protein_cc
ENSG0000(	1207.311	-0.87349	0.087351	-9.99978	1.53E-23	1.02E-21	FLRT2	protein_cc
ENSG0000(	1207.251	0.056725	0.028702	1.976326	0.048118	0.081624	MIER2	protein_cc
ENSG0000(	1207.057	0.114933	0.021016	5.468917	4.53E-08	2.27E-07	GOSR2	protein_cc
ENSG0000(	1206.975	0.167033	0.029823	5.600826	2.13E-08	1.13E-07	FAM114A:	protein_cc
ENSG0000(	1206.713	-0.02996	0.038899	-0.77032	0.441111	0.538032	DYRK1B	protein_cc
ENSG0000(	1205.906	-0.03312	0.027048	-1.22437	0.220814	0.303471	PPID	protein_cc
ENSG0000(	1205.849	-0.02411	0.024901	-0.96834	0.332875	0.427425	METTL14	protein_cc
ENSG0000(	1205.834	-0.02218	0.040997	-0.5409	0.588573	0.674671	CRHR1-IT	pseudoge
ENSG0000(	1205.794	0.211889	0.027637	7.666966	1.76E-14	2.44E-13	TBCE	protein_cc
ENSG0000(	1205.461	-0.30463	0.053842	-5.65782	1.53E-08	8.26E-08	FAM102B	protein_cc
ENSG0000(	1204.763	0.080235	0.041119	1.951289	0.051023	0.086026	TMEM80	protein_cc
ENSG0000(	1204.155	-0.0519	0.037272	-1.39237	0.16381	0.235839	NLRX1	protein_cc
ENSG0000(	1204.06	0.141893	0.040409	3.511456	0.000446	0.001162	ZNF431	protein_cc
ENSG0000(	1203.734	-0.26399	0.047397	-5.56982	2.55E-08	1.33E-07	FYCO1	protein_cc
ENSG0000(	1203.373	0.146282	0.111005	1.31779	0.187574	0.264379	RUNX1	protein_cc
ENSG0000(	1202.419	0.17575	0.035565	4.941639	7.75E-07	3.19E-06	C1orf35	protein_cc
ENSG0000(	1202.307	-0.66676	0.078741	-8.46777	2.50E-17	5.64E-16	NAV3	protein_cc
ENSG0000(	1202.103	-0.31977	0.066905	-4.77942	1.76E-06	6.84E-06	ANKRD9	protein_cc
ENSG0000(	1202.022	-0.2323	0.05152	-4.50894	6.52E-06	2.32E-05	PER2	protein_cc
ENSG0000(	1201.965	-0.14545	0.077111	-1.88623	0.059264	0.097989	CYB561	protein_cc
ENSG0000(	1201.878	0.282613	0.075998	3.718708	0.0002	0.000555	LPAR6	protein_cc
ENSG0000(	1201.507	-0.86287	0.115664	-7.46014	8.64E-14	1.06E-12	DOK6	protein_cc
ENSG0000(	1201.384	0.216868	0.073838	2.937076	0.003313	0.007321	SLC39A8	protein_cc
ENSG0000(	1201.085	-0.09907	0.037614	-2.63398	0.008439	0.017149	CHORDC1	protein_cc
ENSG0000(	1200.74	-0.22655	0.034495	-6.56763	5.11E-11	4.07E-10	DDI2	protein_cc
ENSG0000(	1200.623	0.021366	0.033196	0.643621	0.519821	0.61228	RPAP2	protein_cc
ENSG0000(	1200.099	0.176389	0.030192	5.842265	5.15E-09	2.99E-08	USP42	protein_cc
ENSG0000(	1198.453	-0.30535	0.052478	-5.81868	5.93E-09	3.41E-08	PRDM11	protein_cc
ENSG0000(	1198.362	0.248548	0.037746	6.584835	4.55E-11	3.66E-10	ZNF320	protein_cc
ENSG0000(	1198.178	-0.13758	0.103259	-1.33236	0.182743	0.258509	PDE1C	protein_cc
ENSG0000(	1197.882	-0.12185	0.076241	-1.5982	0.109998	0.167737	PTPN6	protein_cc
ENSG0000(	1197.364	0.445285	0.041575	10.71047	9.09E-27	1.12E-24	TARBP2	protein_cc
ENSG0000(	1197.243	-0.29833	0.115825	-2.57569	0.010004	0.01998	RPRM	protein_cc
ENSG0000(	1196.772	0.248194	0.030718	8.079846	6.48E-16	1.15E-14	AIMP2	protein_cc
ENSG0000(	1196.629	-0.00976	0.032551	-0.29993	0.764227	0.823112	HSPA14	protein_cc
ENSG0000(	1196.561	-0.27326	0.089131	-3.06579	0.002171	0.004974	IL10RA	protein_cc
ENSG0000(	1196.292	0.278753	0.095272	2.925875	0.003435	0.007568	VAMP8	protein_cc
ENSG0000(	1196.192	0.257284	0.06272	4.102129	4.09E-05	0.000128	CUL7	protein_cc
ENSG0000(	1196.026	0.028287	0.038377	0.737086	0.46107	0.557317	DIEXF	protein_cc
ENSG0000(	1195.863	-0.75502	0.123216	-6.12767	8.92E-10	5.86E-09	CDK5R2	protein_cc
ENSG0000(	1195.61	0.021401	0.031943	0.669991	0.502864	0.596767	NDUFAF1	protein_cc
ENSG0000(	1194.982	0.215736	0.03786	5.698321	1.21E-08	6.63E-08	WRAP73	protein_cc
ENSG0000(	1194.968	-0.09212	0.03863	-2.38455	0.0171	0.032359	CEP70	protein_cc
ENSG0000(	1194.869	0.295784	0.051689	5.722399	1.05E-08	5.80E-08	YIPF1	protein_cc

ENSG0000(	1194.46	-0.65939	0.132071	-4.99273	5.95E-07	2.49E-06	WNT7B	protein_cc
ENSG0000(	1194.258	-0.01265	0.099549	-0.12703	0.89892	0.92831	AR	protein_cc
ENSG0000(	1194.025	-0.04901	0.082082	-0.59711	0.550437	0.640362	LRRC16A	protein_cc
ENSG0000(	1193.85	-0.03098	0.034966	-0.88611	0.375558	0.472163	CPT2	protein_cc
ENSG0000(	1193.576	-0.1568	0.035963	-4.36006	1.30E-05	4.40E-05	ZBTB43	protein_cc
ENSG0000(	1193.167	-0.39509	0.050917	-7.7594	8.53E-15	1.26E-13	TRAPPC1(	protein_cc
ENSG0000(	1192.716	-0.02007	0.028936	-0.69361	0.487926	0.582655	DDX10	protein_cc
ENSG0000(	1192.638	-0.26379	0.124522	-2.11843	0.034138	0.060251	KCNJ11	protein_cc
ENSG0000(	1191.814	0.078562	0.032104	2.447134	0.0144	0.027777	GOLPH3L	protein_cc
ENSG0000(	1191.192	-0.21704	0.076342	-2.84301	0.004469	0.00962	SH3TC1	protein_cc
ENSG0000(	1191.058	0.117943	0.104319	1.130607	0.258221	0.346191	TOX3	protein_cc
ENSG0000(	1190.296	-0.00861	0.033885	-0.25404	0.799467	0.851875	ELP4	protein_cc
ENSG0000(	1190.122	-0.64499	0.074043	-8.71097	3.01E-18	7.80E-17	CACNA1C	protein_cc
ENSG0000(	1189.938	-0.1219	0.0373	-3.26804	0.001083	0.002622	INO80D	protein_cc
ENSG0000(	1189.796	0.353531	0.05163	6.847382	7.52E-12	6.82E-11	CTNNAL1	protein_cc
ENSG0000(	1189.438	0.014569	0.022803	0.638899	0.522888	0.614906	MCPH1	protein_cc
ENSG0000(	1188.854	0.014891	0.02022	0.736445	0.46146	0.557624	PLEKHA3	protein_cc
ENSG0000(	1188.789	0.24057	0.038388	6.266859	3.68E-10	2.57E-09	DALRD3	protein_cc
ENSG0000(	1188.775	-0.1939	0.024904	-7.78595	6.92E-15	1.04E-13	UVRAG	protein_cc
ENSG0000(	1188.013	-0.29992	0.030549	-9.81778	9.44E-23	5.23E-21	C2orf69	protein_cc
ENSG0000(	1187.589	0.561097	0.078291	7.166838	7.67E-13	8.13E-12	CDH6	protein_cc
ENSG0000(	1187.558	0.51514	0.134381	3.833418	0.000126	0.000363	SFRP4	protein_cc
ENSG0000(	1187.548	-0.12199	0.049039	-2.48763	0.01286	0.025053	BCL2L11	protein_cc
ENSG0000(	1186.325	-0.08321	0.038248	-2.17563	0.029583	0.05298	ZNF517	protein_cc
ENSG0000(	1186.199	1.411953	0.102633	13.75724	4.61E-43	8.61E-40	METTL1	protein_cc
ENSG0000(	1185.847	0.228405	0.03164	7.218907	5.24E-13	5.67E-12	WIBG	protein_cc
ENSG0000(	1185.832	0.315982	0.048617	6.499446	8.06E-11	6.23E-10	PGM2	protein_cc
ENSG0000(	1185.776	-0.01816	0.074721	-0.24298	0.808019	0.858674	GPSM3	protein_cc
ENSG0000(	1185.562	1.272205	0.153852	8.269001	1.35E-16	2.69E-15	PDLIM4	protein_cc
ENSG0000(	1185.458	0.002561	0.032098	0.079784	0.936409	0.955555	TANGO6	protein_cc
ENSG0000(	1185.338	0.308419	0.038823	7.944145	1.96E-15	3.22E-14	LINC0049:	lincRNA
ENSG0000(	1185.285	1.26206	0.135868	9.288872	1.56E-20	5.77E-19	RRM2	protein_cc
ENSG0000(	1185.211	0.165146	0.032954	5.011415	5.40E-07	2.28E-06	SLC25A40	protein_cc
ENSG0000(	1185.205	-0.18191	0.050207	-3.62319	0.000291	0.000785	KCTD1	protein_cc
ENSG0000(	1185	-0.39572	0.071986	-5.49721	3.86E-08	1.95E-07	HMGN5	protein_cc
ENSG0000(	1183.946	-0.88824	0.1176	-7.55306	4.25E-14	5.51E-13	ARHGDIG	protein_cc
ENSG0000(	1183.63	0.928745	0.107473	8.641622	5.54E-18	1.38E-16	SPRY1	protein_cc
ENSG0000(	1183.266	-0.23119	0.041354	-5.59043	2.27E-08	1.19E-07	MTPAP	protein_cc
ENSG0000(	1183.196	-0.38108	0.069966	-5.44664	5.13E-08	2.55E-07	FMNL1	protein_cc
ENSG0000(	1182.549	-1.30212	0.097423	-13.3656	9.60E-41	1.36E-37	RASGRF2	protein_cc
ENSG0000(	1182.248	-0.13412	0.113224	-1.18458	0.236183	0.32103	CDKN2B	protein_cc
ENSG0000(	1182.058	-0.97381	0.126918	-7.67274	1.68E-14	2.33E-13	GALNT9	protein_cc
ENSG0000(	1182.054	0.148893	0.046846	3.178382	0.001481	0.003502	ENKD1	protein_cc
ENSG0000(	1181.936	0.050582	0.051781	0.976856	0.32864	0.423084	AZI1	protein_cc
ENSG0000(	1181.811	-0.37724	0.050569	-7.45997	8.65E-14	1.06E-12	FAM126B	protein_cc
ENSG0000(	1181.799	-0.08521	0.028512	-2.9886	0.002803	0.006278	RHBDD1	protein_cc
ENSG0000(	1181.718	-0.25713	0.043933	-5.85277	4.83E-09	2.82E-08	HIVEP1	protein_cc
ENSG0000(	1181.55	0.048632	0.028607	1.699966	0.089137	0.13988	ZNF7	protein_cc
ENSG0000(	1181.329	-0.40352	0.05482	-7.36081	1.83E-13	2.13E-12	PPP1R13B	protein_cc
ENSG0000(	1180.822	0.325935	0.041982	7.763736	8.25E-15	1.22E-13	WDR54	protein_cc
ENSG0000(	1180.669	-1.38818	0.149013	-9.31589	1.21E-20	4.58E-19	CPNE6	protein_cc
ENSG0000(	1180.413	-0.04943	0.064105	-0.77112	0.440639	0.537566	ITPRIP	protein_cc
ENSG0000(	1180.346	0.196693	0.082769	2.376407	0.017482	0.032985	TCIRG1	protein_cc
ENSG0000(	1180.243	-0.12145	0.045302	-2.68085	0.007344	0.015119	N4BP2L1	protein_cc
ENSG0000(	1180.153	0.096282	0.021686	4.439858	9.00E-06	3.13E-05	CXorf56	protein_cc
ENSG0000(	1179.73	0.193999	0.029985	6.469859	9.81E-11	7.48E-10	TADA1	protein_cc
ENSG0000(	1179.32	-0.16381	0.091726	-1.78581	0.074129	0.11911	CADPS2	protein_cc
ENSG0000(	1179.253	0.302151	0.030592	9.876684	5.25E-23	3.09E-21	ELMOD2	protein_cc
ENSG0000(	1179.026	-0.21798	0.039523	-5.5153	3.48E-08	1.78E-07	C22orf29	protein_cc

ENSG0000(	1178.651	-0.2934	0.047267	-6.20726	5.39E-10	3.66E-09	PTGR2	protein_cc
ENSG0000(	1178.359	0.069752	0.053481	1.304252	0.192148	0.2699	ADAMTS1	protein_cc
ENSG0000(	1178.2	-0.06904	0.080768	-0.85479	0.392669	0.489278	PPARGC1/	protein_cc
ENSG0000(	1177.809	0.263379	0.031514	8.357399	6.41E-17	1.35E-15	DXO	protein_cc
ENSG0000(	1177.633	-0.20965	0.042761	-4.90283	9.45E-07	3.83E-06	ZNF778	protein_cc
ENSG0000(	1177.566	0.515177	0.071317	7.22374	5.06E-13	5.49E-12	RPL22L1	protein_cc
ENSG0000(	1177.453	0.096278	0.035494	2.712498	0.006678	0.013861	PSMA6	protein_cc
ENSG0000(	1177.064	0.045982	0.051262	0.897011	0.369713	0.466047	CCDC57	protein_cc
ENSG0000(	1176.814	0.19241	0.050561	3.805538	0.000141	0.000402	SUMF1	protein_cc
ENSG0000(	1176.328	0.063096	0.030991	2.035969	0.041753	0.072082	KRBA1	protein_cc
ENSG0000(	1176.228	0.177972	0.042053	4.232081	2.32E-05	7.52E-05	ZNF787	protein_cc
ENSG0000(	1175.276	0.821412	0.12027	6.829714	8.51E-12	7.63E-11	ANGPT2	protein_cc
ENSG0000(	1175.033	0.022866	0.035772	0.639231	0.522672	0.614722	PUS7L	protein_cc
ENSG0000(	1174.828	-0.02723	0.030318	-0.89803	0.369168	0.46551	DTWD1	protein_cc
ENSG0000(	1173.576	-1.14298	0.105169	-10.868	1.64E-27	2.30E-25	MFSD4	protein_cc
ENSG0000(	1173.533	0.059422	0.025	2.376922	0.017458	0.032948	GMEB2	protein_cc
ENSG0000(	1172.733	0.231751	0.032265	7.182731	6.83E-13	7.29E-12	SNRPA1	protein_cc
ENSG0000(	1172.14	-0.14282	0.051261	-2.78605	0.005335	0.011307	ZBTB7B	protein_cc
ENSG0000(	1172.129	0.284647	0.028924	9.841238	7.48E-23	4.24E-21	MPLKIP	protein_cc
ENSG0000(	1171.054	-0.00461	0.080413	-0.05737	0.95425	0.968295	FERMT3	protein_cc
ENSG0000(	1171.041	-0.3073	0.094218	-3.26159	0.001108	0.002677	SELM	protein_cc
ENSG0000(	1170.584	-0.05191	0.04477	-1.15945	0.246274	0.332658	FNTB	protein_cc
ENSG0000(	1170.194	-0.09941	0.06254	-1.58961	0.111922	0.170274	AK1	protein_cc
ENSG0000(	1169.389	0.285302	0.026802	10.64472	1.85E-26	2.17E-24	EXOSC9	protein_cc
ENSG0000(	1169.379	0.130367	0.03203	4.070146	4.70E-05	0.000145	ZNF133	protein_cc
ENSG0000(	1169.265	0.497088	0.054344	9.147011	5.85E-20	1.99E-18	DPM3	protein_cc
ENSG0000(	1169.157	-0.03825	0.033319	-1.14813	0.250914	0.338104	SDAD1	protein_cc
ENSG0000(	1168.494	0.036169	0.038337	0.943439	0.345457	0.440882	PDLIM2	protein_cc
ENSG0000(	1168.026	0.361451	0.103302	3.49899	0.000467	0.001212	PLVAP	protein_cc
ENSG0000(	1167.643	-0.57238	0.07928	-7.21976	5.21E-13	5.64E-12	LDB2	protein_cc
ENSG0000(	1167.346	-0.91211	0.127509	-7.15327	8.47E-13	8.92E-12	SLC25A48	protein_cc
ENSG0000(	1167.238	0.134624	0.027455	4.903354	9.42E-07	3.82E-06	ZNF212	protein_cc
ENSG0000(	1166.614	-0.06403	0.050476	-1.26847	0.204629	0.284739	FRMD6	protein_cc
ENSG0000(	1166.401	-0.74061	0.072148	-10.2651	1.01E-24	8.39E-23	TMTC1	protein_cc
ENSG0000(	1166.298	0.19231	0.048332	3.978965	6.92E-05	0.000208	ATP10D	protein_cc
ENSG0000(	1166.188	0.203568	0.036182	5.626174	1.84E-08	9.81E-08	MCTS1	protein_cc
ENSG0000(	1166.084	0.195198	0.03409	5.725946	1.03E-08	5.69E-08	PIGN	protein_cc
ENSG0000(	1166.006	0.260182	0.030944	8.408075	4.17E-17	9.02E-16	PPOX	protein_cc
ENSG0000(	1165.827	0.094579	0.023645	3.99999	6.33E-05	0.000192	PEX13	protein_cc
ENSG0000(	1165.442	0.262188	0.029267	8.958543	3.29E-19	9.85E-18	BRIX1	protein_cc
ENSG0000(	1165.331	-0.44979	0.052567	-8.55651	1.16E-17	2.75E-16	ST3GAL1	protein_cc
ENSG0000(	1165.221	-0.07644	0.031332	-2.43952	0.014707	0.028296	AARS2	protein_cc
ENSG0000(	1165.049	-0.39456	0.088954	-4.43561	9.18E-06	3.19E-05	CD84	protein_cc
ENSG0000(	1164.868	-0.23064	0.06285	-3.66965	0.000243	0.000664	RCSD1	protein_cc
ENSG0000(	1164.859	-0.0285	0.042855	-0.66509	0.50599	0.599681	DYRK2	protein_cc
ENSG0000(	1164.678	-0.08533	0.033313	-2.56155	0.010421	0.020739	SLC19A1	protein_cc
ENSG0000(	1164.642	0.004098	0.037508	0.109251	0.913003	0.938682	LRFN3	protein_cc
ENSG0000(	1164.229	-0.12489	0.043983	-2.83949	0.004519	0.009709	AC092171	lincRNA
ENSG0000(	1164.21	0.034066	0.03662	0.930271	0.352231	0.447791	CHCHD1	protein_cc
ENSG0000(	1163.462	0.388507	0.076771	5.060601	4.18E-07	1.79E-06	CNIH3	protein_cc
ENSG0000(	1162.939	-0.00684	0.034685	-0.19726	0.843624	0.886429	KCTD11	protein_cc
ENSG0000(	1162.351	-0.00832	0.03094	-0.26905	0.787894	0.842359	INPP5B	protein_cc
ENSG0000(	1162.349	-0.41175	0.077901	-5.2856	1.25E-07	5.84E-07	NCEH1	protein_cc
ENSG0000(	1161.945	0.147249	0.032787	4.491113	7.09E-06	2.51E-05	FIZ1	protein_cc
ENSG0000(	1161.272	-0.13458	0.040629	-3.3124	0.000925	0.002267	MXD1	protein_cc
ENSG0000(	1160.954	0.169945	0.02797	6.075965	1.23E-09	7.92E-09	NDNL2	protein_cc
ENSG0000(	1160.799	0.682288	0.086079	7.926272	2.26E-15	3.68E-14	GSDMD	protein_cc
ENSG0000(	1160.38	0.098256	0.021504	4.569102	4.90E-06	1.78E-05	SMARCAL	protein_cc
ENSG0000(	1160.29	0.199081	0.039654	5.020502	5.15E-07	2.18E-06	CCHCR1	protein_cc

ENSG0000(	1159.911	0.156876	0.046671	3.361306	0.000776	0.001929	ZNF865	protein_cc
ENSG0000(	1159.899	0.455683	0.045828	9.943243	2.70E-23	1.70E-21	TRIAP1	protein_cc
ENSG0000(	1159.546	-0.36254	0.076925	-4.71291	2.44E-06	9.29E-06	CD83	protein_cc
ENSG0000(	1159.173	0.178213	0.037854	4.707921	2.50E-06	9.50E-06	ING1	protein_cc
ENSG0000(	1159.137	0.036916	0.030428	1.2132	0.225054	0.308339	PCYOX1L	protein_cc
ENSG0000(	1159.068	0.063232	0.028304	2.234001	0.025483	0.046321	RPUSD3	protein_cc
ENSG0000(	1158.874	0.094467	0.028152	3.355642	0.000792	0.001967	RRP15	protein_cc
ENSG0000(	1158.867	0.244022	0.066127	3.690217	0.000224	0.000615	LOXL3	protein_cc
ENSG0000(	1158.839	0.067363	0.039143	1.720947	0.085261	0.134473	HLCS	protein_cc
ENSG0000(	1158.337	0.246698	0.063814	3.865875	0.000111	0.000321	PPM1M	protein_cc
ENSG0000(	1157.281	-0.77646	0.232947	-3.33319	0.000859	0.002118	MTRNR2L	protein_cc
ENSG0000(	1157.253	0.273854	0.037385	7.325271	2.38E-13	2.72E-12	PPIL3	protein_cc
ENSG0000(	1157.089	-0.28028	0.095559	-2.93304	0.003357	0.00741	SMPD3	protein_cc
ENSG0000(	1156.693	0.1396	0.047286	2.952263	0.003155	0.006998	EXOSC4	protein_cc
ENSG0000(	1154.951	0.145939	0.027343	5.337308	9.43E-08	4.48E-07	POLR3C	protein_cc
ENSG0000(	1154.936	-0.02539	0.032524	-0.78069	0.434982	0.532039	ABCC10	protein_cc
ENSG0000(	1154.613	0.277703	0.038013	7.305414	2.76E-13	3.12E-12	GALNS	protein_cc
ENSG0000(	1154.513	-0.08194	0.045456	-1.80257	0.071455	0.115354	MYSM1	protein_cc
ENSG0000(	1154.457	0.207389	0.124591	1.664564	0.096	0.149135	TYMP	protein_cc
ENSG0000(	1154.259	0.264918	0.077287	3.427728	0.000609	0.001545	TBX2	protein_cc
ENSG0000(	1153.836	0.020887	0.072497	0.288112	0.773261	0.83045	PDE4D	protein_cc
ENSG0000(	1153.46	0.246292	0.029581	8.326043	8.36E-17	1.72E-15	DPM1	protein_cc
ENSG0000(	1152.975	0.039814	0.031216	1.275441	0.202153	0.281876	ZNF48	protein_cc
ENSG0000(	1152.919	0.050789	0.031218	1.626916	0.103755	0.159465	C17orf75	protein_cc
ENSG0000(	1152.703	0.187891	0.071673	2.621489	0.008755	0.01772	FAM60A	protein_cc
ENSG0000(	1152.468	-0.02835	0.032614	-0.86916	0.38476	0.481407	TRIM11	protein_cc
ENSG0000(	1152.344	-0.00196	0.032363	-0.0605	0.951754	0.966729	ZFP1	protein_cc
ENSG0000(	1151.612	0.139183	0.036973	3.764469	0.000167	0.000469	FAM89B	protein_cc
ENSG0000(	1151.599	0.219843	0.030466	7.215997	5.35E-13	5.79E-12	MED20	protein_cc
ENSG0000(	1151.565	-0.72963	0.078699	-9.27105	1.84E-20	6.75E-19	OTUD7A	protein_cc
ENSG0000(	1151.511	0.338348	0.071604	4.725298	2.30E-06	8.78E-06	C1orf85	protein_cc
ENSG0000(	1151.341	0.096627	0.030938	3.12325	0.001789	0.004167	ARV1	protein_cc
ENSG0000(	1150.96	0.106516	0.044111	2.414716	0.015747	0.030073	ELOVL6	protein_cc
ENSG0000(	1150.912	0.258938	0.031209	8.297024	1.07E-16	2.16E-15	RNF216P1	pseudoge
ENSG0000(	1150.752	-0.06702	0.036065	-1.8584	0.063112	0.10354	INTS2	protein_cc
ENSG0000(	1150.514	-0.16683	0.050525	-3.30191	0.00096	0.002346	ABCC4	protein_cc
ENSG0000(	1150.301	-0.03717	0.027095	-1.3718	0.170127	0.243527	ZDHHC16	protein_cc
ENSG0000(	1150.163	-0.18189	0.069105	-2.63207	0.008487	0.01724	CECR6	protein_cc
ENSG0000(	1150.147	-0.3147	0.085621	-3.67549	0.000237	0.00065	SEC31B	protein_cc
ENSG0000(	1150.14	0.036598	0.073395	0.49864	0.618033	0.700937	HBEGF	protein_cc
ENSG0000(	1149.803	-0.80465	0.102468	-7.85264	4.07E-15	6.36E-14	GRIN2C	protein_cc
ENSG0000(	1148.677	0.15006	0.030631	4.898925	9.64E-07	3.90E-06	ABCB7	protein_cc
ENSG0000(	1148.56	0.225688	0.039152	5.764382	8.20E-09	4.61E-08	EIF2B3	protein_cc
ENSG0000(	1147.818	-1.12649	0.138868	-8.11195	4.98E-16	9.04E-15	PTPN5	protein_cc
ENSG0000(	1147.246	0.096062	0.023276	4.127159	3.67E-05	0.000116	RIOK2	protein_cc
ENSG0000(	1146.723	0.049362	0.028214	1.749556	0.080195	0.12752	EFTUD1	protein_cc
ENSG0000(	1146.58	0.138835	0.025355	5.475723	4.36E-08	2.19E-07	MRRF	protein_cc
ENSG0000(	1146.525	-0.05975	0.034397	-1.73713	0.082364	0.130444	TBCC	protein_cc
ENSG0000(	1146.246	-0.00259	0.091203	-0.02845	0.977304	0.983956	MT1F	protein_cc
ENSG0000(	1146.208	-0.34711	0.075838	-4.57693	4.72E-06	1.72E-05	ALDH1L2	protein_cc
ENSG0000(	1146.197	0.070865	0.036798	1.925786	0.054131	0.090572	CEPT1	protein_cc
ENSG0000(	1145.966	0.068065	0.043494	1.564918	0.117602	0.177654	TIGD5	protein_cc
ENSG0000(	1145.743	0.035308	0.031168	1.132824	0.257288	0.345184	RP11-156	sense_ove
ENSG0000(	1145.437	0.482414	0.048883	9.868789	5.68E-23	3.33E-21	COMMD1	protein_cc
ENSG0000(	1145.182	-0.22592	0.041837	-5.4	6.66E-08	3.25E-07	JDP2	protein_cc
ENSG0000(	1145.132	-0.16549	0.029185	-5.67036	1.42E-08	7.72E-08	PORCN	protein_cc
ENSG0000(	1144.838	-0.62205	0.096334	-6.45725	1.07E-10	8.10E-10	KIT	protein_cc
ENSG0000(	1144.667	-0.12425	0.033703	-3.68673	0.000227	0.000623	ATXN7	protein_cc
ENSG0000(	1144.538	-0.21415	0.040121	-5.33757	9.42E-08	4.48E-07	TRIM3	protein_cc

ENSG0000(	1144.339	0.130869	0.032888	3.979279	6.91E-05	0.000208	SIAH2	protein_cc
ENSG0000(	1144.186	-0.13163	0.088718	-1.48371	0.137886	0.203409	GSG1L	protein_cc
ENSG0000(	1143.832	-0.00106	0.048723	-0.02183	0.98258	0.987985	LINC0088(	pseudoge
ENSG0000(	1143.265	0.259486	0.068618	3.781615	0.000156	0.00044	LEPRE1	protein_cc
ENSG0000(	1143.203	-0.27618	0.037359	-7.39256	1.44E-13	1.71E-12	SMAP1	protein_cc
ENSG0000(	1143.041	-0.17782	0.060926	-2.91866	0.003515	0.007731	ZNF37BP	pseudoge
ENSG0000(	1142.76	-0.00506	0.04745	-0.10671	0.915016	0.940117	MANSC1	protein_cc
ENSG0000(	1142.569	-0.92399	0.114174	-8.09282	5.83E-16	1.05E-14	FGF13	protein_cc
ENSG0000(	1142.509	-0.01191	0.028459	-0.41839	0.675663	0.750121	MON1A	protein_cc
ENSG0000(	1141.66	-0.00856	0.029938	-0.28588	0.774972	0.831923	CWF19L1	protein_cc
ENSG0000(	1141.599	-0.34939	0.135683	-2.57503	0.010023	0.020012	STAC2	protein_cc
ENSG0000(	1141.468	-0.02903	0.022987	-1.26291	0.206623	0.287047	RPP14	protein_cc
ENSG0000(	1141.176	0.197588	0.033603	5.880069	4.10E-09	2.42E-08	ZMYND19	protein_cc
ENSG0000(	1140.996	-0.58032	0.099177	-5.85134	4.88E-09	2.84E-08	DBNDD2	protein_cc
ENSG0000(	1140.705	0.135062	0.033204	4.067711	4.75E-05	0.000147	SLC35A2	protein_cc
ENSG0000(	1140.454	0.275183	0.032155	8.557918	1.15E-17	2.72E-16	POLR1C	protein_cc
ENSG0000(	1140.391	-0.03364	0.038669	-0.87007	0.384262	0.48096	KCTD6	protein_cc
ENSG0000(	1140.112	0.033485	0.028382	1.179795	0.238082	0.323249	PPWD1	protein_cc
ENSG0000(	1139.936	-0.07083	0.027282	-2.59631	0.009423	0.018931	AZI2	protein_cc
ENSG0000(	1139.826	0.234814	0.028386	8.272051	1.32E-16	2.62E-15	ABT1	protein_cc
ENSG0000(	1139.283	-0.04166	0.042771	-0.97409	0.330013	0.424385	TSPY26P	pseudoge
ENSG0000(	1138.569	0.83044	0.123723	6.712069	1.92E-11	1.63E-10	C21orf62	protein_cc
ENSG0000(	1138.444	-0.1525	0.027925	-5.4612	4.73E-08	2.36E-07	KLHL18	protein_cc
ENSG0000(	1138.333	0.199783	0.041488	4.815411	1.47E-06	5.79E-06	ZNF226	protein_cc
ENSG0000(	1138.182	-0.3296	0.069989	-4.70922	2.49E-06	9.44E-06	6-Sep	protein_cc
ENSG0000(	1137.852	-0.16298	0.058636	-2.77953	0.005444	0.011515	SHF	protein_cc
ENSG0000(	1137.663	0.080031	0.021538	3.715827	0.000203	0.000561	MFSD11	protein_cc
ENSG0000(	1137.224	0.317539	0.051845	6.124753	9.08E-10	5.96E-09	LRRC42	protein_cc
ENSG0000(	1137.078	0.206928	0.028661	7.21982	5.21E-13	5.64E-12	ALKBH4	protein_cc
ENSG0000(	1136.78	-0.18608	0.080232	-2.31924	0.020382	0.037867	CDKN1C	protein_cc
ENSG0000(	1136.746	-0.5622	0.071961	-7.81251	5.61E-15	8.54E-14	NR3C2	protein_cc
ENSG0000(	1136.586	0.060922	0.047692	1.277401	0.201461	0.281043	PHKA2	protein_cc
ENSG0000(	1136.374	-0.31124	0.041538	-7.49291	6.74E-14	8.46E-13	KIAA2018	protein_cc
ENSG0000(	1136.349	0.078124	0.036056	2.16673	0.030255	0.05405	SLC35C1	protein_cc
ENSG0000(	1136.314	-0.09033	0.036018	-2.50805	0.01214	0.023789	DIRC2	protein_cc
ENSG0000(	1136.257	0.00907	0.033168	0.273449	0.784508	0.839524	RABGGTA	protein_cc
ENSG0000(	1136.153	-0.36661	0.083713	-4.37936	1.19E-05	4.06E-05	SLC25A27	protein_cc
ENSG0000(	1135.67	-0.53319	0.061387	-8.68557	3.77E-18	9.63E-17	ADARB1	protein_cc
ENSG0000(	1134.583	0.162769	0.028088	5.79489	6.84E-09	3.89E-08	LSM1	protein_cc
ENSG0000(	1134.491	0.253686	0.039667	6.39548	1.60E-10	1.18E-09	XXYLT1	protein_cc
ENSG0000(	1133.59	-0.03035	0.026094	-1.16313	0.244777	0.330972	C15orf40	protein_cc
ENSG0000(	1133.476	0.082982	0.036743	2.258455	0.023917	0.043755	ERMARD	protein_cc
ENSG0000(	1133.278	0.626858	0.121869	5.143711	2.69E-07	1.19E-06	TFPI	protein_cc
ENSG0000(	1132.888	-0.16463	0.065819	-2.50126	0.012375	0.024198	DSE	protein_cc
ENSG0000(	1132.69	-0.87173	0.085234	-10.2274	1.49E-24	1.20E-22	MAGEE1	protein_cc
ENSG0000(	1131.971	-0.10211	0.053713	-1.90106	0.057294	0.09518	BRWD3	protein_cc
ENSG0000(	1131.952	0.174919	0.02277	7.682054	1.57E-14	2.19E-13	EIF1AD	protein_cc
ENSG0000(	1131.785	0.871988	0.080973	10.76893	4.83E-27	6.28E-25	EZH2	protein_cc
ENSG0000(	1131.744	0.165663	0.036272	4.567291	4.94E-06	1.79E-05	VSIG10	protein_cc
ENSG0000(	1131.685	0.167083	0.068513	2.438697	0.01474	0.028355	TRAM2	protein_cc
ENSG0000(	1131.256	-0.00942	0.02283	-0.41253	0.679951	0.75364	RBM27	protein_cc
ENSG0000(	1131.181	0.201421	0.045684	4.408968	1.04E-05	3.58E-05	LAGE3	protein_cc
ENSG0000(	1130.998	-0.64402	0.101356	-6.35407	2.10E-10	1.52E-09	NECAB2	protein_cc
ENSG0000(	1130.938	0.921473	0.082789	11.13041	8.92E-29	1.65E-26	FANCI	protein_cc
ENSG0000(	1130.907	-0.14289	0.042665	-3.34914	0.000811	0.002009	TBPL1	protein_cc
ENSG0000(	1130.905	-0.1783	0.032895	-5.42013	5.96E-08	2.92E-07	TMEM254	protein_cc
ENSG0000(	1130.772	0.143846	0.030158	4.769718	1.84E-06	7.15E-06	TACO1	protein_cc
ENSG0000(	1130.465	-0.00458	0.026941	-0.17018	0.864869	0.90285	SMG8	protein_cc
ENSG0000(	1130.36	-0.01741	0.047225	-0.36858	0.712439	0.780644	MFSD3	protein_cc

ENSG0000(	1130.331	-0.13392	0.045367	-2.95189	0.003158	0.007006	CHCHD7	protein_co
ENSG0000(	1130.187	-0.00442	0.029275	-0.15098	0.879989	0.914202	ATR	protein_co
ENSG0000(	1129.606	-0.52426	0.085662	-6.12014	9.35E-10	6.13E-09	TMEM63C	protein_co
ENSG0000(	1129.385	-0.00971	0.027504	-0.35316	0.723966	0.789928	ACOX3	protein_co
ENSG0000(	1128.962	-0.10171	0.043797	-2.32225	0.020219	0.037596	SNTB2	protein_co
ENSG0000(	1128.684	-0.13259	0.032162	-4.1224	3.75E-05	0.000118	WDR44	protein_co
ENSG0000(	1128.669	0.191624	0.079538	2.409204	0.015987	0.030493	TNXB	protein_co
ENSG0000(	1127.969	-0.42279	0.13413	-3.15212	0.001621	0.003805	CLVS2	protein_co
ENSG0000(	1127.919	-0.02319	0.053107	-0.43673	0.66231	0.739067	SOX21-AS	lincRNA
ENSG0000(	1127.742	-0.18551	0.031563	-5.8775	4.17E-09	2.46E-08	FAM219B	protein_co
ENSG0000(	1127.704	0.043767	0.070566	0.620229	0.535107	0.626322	FUCA1	protein_co
ENSG0000(	1127.702	0.305425	0.051821	5.893878	3.77E-09	2.24E-08	IL10RB	protein_co
ENSG0000(	1127.302	0.143443	0.037556	3.819465	0.000134	0.000382	ZNF181	protein_co
ENSG0000(	1127.181	-0.04495	0.08851	-0.50787	0.611545	0.695132	FGF14	protein_co
ENSG0000(	1127.15	-0.32697	0.059963	-5.45278	4.96E-08	2.47E-07	STS	protein_co
ENSG0000(	1127.071	0.30564	0.066991	4.562372	5.06E-06	1.83E-05	TRIP10	protein_co
ENSG0000(	1126.913	-0.1285	0.036803	-3.4916	0.00048	0.001243	SLC17A5	protein_co
ENSG0000(	1126.884	-1.01444	0.126984	-7.98873	1.36E-15	2.31E-14	SYT5	protein_co
ENSG0000(	1126.008	0.265033	0.043893	6.038117	1.56E-09	9.85E-09	SAP30	protein_co
ENSG0000(	1125.481	-0.27125	0.043211	-6.27742	3.44E-10	2.41E-09	KCTD21	protein_co
ENSG0000(	1125.115	0.007134	0.06048	0.117962	0.906098	0.933432	FIGN	protein_co
ENSG0000(	1125.075	-0.10121	0.028917	-3.50006	0.000465	0.001208	IDE	protein_co
ENSG0000(	1124.744	1.167694	0.08663	13.47909	2.08E-41	3.28E-38	CDCA7L	protein_co
ENSG0000(	1124.54	0.074383	0.029077	2.558153	0.010523	0.020922	TATDN3	protein_co
ENSG0000(	1124.404	-0.07288	0.040229	-1.8116	0.070048	0.113389	APAF1	protein_co
ENSG0000(	1123.925	-0.681	0.133926	-5.08493	3.68E-07	1.59E-06	RIMS2	protein_co
ENSG0000(	1123.517	-0.40311	0.080521	-5.00632	5.55E-07	2.34E-06	LIPE	protein_co
ENSG0000(	1123.359	0.718919	0.143618	5.005776	5.56E-07	2.34E-06	CDKN2A	protein_co
ENSG0000(	1123.084	0.189205	0.072003	2.627744	0.008595	0.017432	PLXDC1	protein_co
ENSG0000(	1123.045	-0.01806	0.059426	-0.30385	0.761239	0.820646	EXD3	protein_co
ENSG0000(	1122.859	-0.24403	0.03614	-6.75235	1.45E-11	1.26E-10	C16orf52	protein_co
ENSG0000(	1122.519	-0.20621	0.044513	-4.63256	3.61E-06	1.34E-05	TRUB1	protein_co
ENSG0000(	1122.508	0.202525	0.027738	7.301473	2.85E-13	3.21E-12	RBMX2	protein_co
ENSG0000(	1122.48	-0.32329	0.056698	-5.70198	1.18E-08	6.50E-08	OSBP2	protein_co
ENSG0000(	1122.44	0.113028	0.043193	2.616799	0.008876	0.01794	NUDT2	protein_co
ENSG0000(	1122.081	0.523112	0.043017	12.16059	5.04E-34	2.59E-31	MALSU1	protein_co
ENSG0000(	1121.941	0.291004	0.078404	3.711597	0.000206	0.000569	RHBDF1	protein_co
ENSG0000(	1121.824	0.812549	0.073487	11.05706	2.03E-28	3.46E-26	GGH	protein_co
ENSG0000(	1121.395	-0.21049	0.083186	-2.53041	0.011393	0.022466	HCK	protein_co
ENSG0000(	1120.513	-0.20123	0.033674	-5.97568	2.29E-09	1.41E-08	HACE1	protein_co
ENSG0000(	1120.175	-0.05076	0.041336	-1.22786	0.219498	0.301945	ZNF251	protein_co
ENSG0000(	1120.072	0.003206	0.036109	0.088783	0.929254	0.950306	EYA3	protein_co
ENSG0000(	1120.028	0.160112	0.053184	3.010532	0.002608	0.005889	TMEM70	protein_co
ENSG0000(	1119.676	-0.00107	0.055899	-0.0192	0.98468	0.989517	MACROD1	protein_co
ENSG0000(	1119.321	0.333198	0.04071	8.184624	2.73E-16	5.16E-15	PNO1	protein_co
ENSG0000(	1118.998	-0.1394	0.059774	-2.33217	0.019692	0.036704	SERP2	protein_co
ENSG0000(	1118.796	-0.57168	0.061875	-9.23931	2.48E-20	8.88E-19	PELI3	protein_co
ENSG0000(	1117.935	-0.14592	0.054543	-2.67534	0.007465	0.015345	CCDC113	protein_co
ENSG0000(	1117.309	-0.80479	0.102795	-7.82906	4.92E-15	7.57E-14	SGSM1	protein_co
ENSG0000(	1117.132	1.192023	0.099606	11.96744	5.26E-33	2.30E-30	CCNB1	protein_co
ENSG0000(	1116.677	0.119882	0.029046	4.127374	3.67E-05	0.000115	ZNF398	protein_co
ENSG0000(	1116.497	-0.82327	0.122302	-6.73147	1.68E-11	1.44E-10	SYT16	protein_co
ENSG0000(	1116.467	0.277146	0.055978	4.950972	7.38E-07	3.05E-06	TSPAN4	protein_co
ENSG0000(	1116.39	0.209577	0.037792	5.545535	2.93E-08	1.51E-07	ZNF254	protein_co
ENSG0000(	1116.073	0.03689	0.035604	1.036114	0.300149	0.392587	LRRC40	protein_co
ENSG0000(	1116.048	0.047092	0.03524	1.336336	0.18144	0.257016	CDK20	protein_co
ENSG0000(	1115.87	0.289272	0.033566	8.618017	6.81E-18	1.68E-16	POGLUT1	protein_co
ENSG0000(	1115.64	0.010574	0.026263	0.402624	0.687225	0.759959	EBAG9	protein_co
ENSG0000(	1115.468	-0.14	0.027732	-5.04834	4.46E-07	1.90E-06	NOP14-AS	antisense

ENSG0000(	1115.392	0.15921	0.058555	2.718978	0.006548	0.01361	SLC4A7	protein_cc
ENSG0000(	1115.307	0.236627	0.051968	4.553303	5.28E-06	1.91E-05	SNAPC2	protein_cc
ENSG0000(	1114.864	-0.6524	0.097598	-6.68455	2.32E-11	1.94E-10	DOCK5	protein_cc
ENSG0000(	1114.741	0.216308	0.037818	5.719668	1.07E-08	5.89E-08	RP11-379	antisense
ENSG0000(	1114.655	0.135233	0.032311	4.185354	2.85E-05	9.12E-05	DDX55	protein_cc
ENSG0000(	1114.376	0.052974	0.028394	1.865644	0.062091	0.102044	MRPS9	protein_cc
ENSG0000(	1114.242	-0.67851	0.072772	-9.32381	1.12E-20	4.28E-19	SPINT2	protein_cc
ENSG0000(	1113.721	0.112006	0.032483	3.448107	0.000565	0.001443	CMTR2	protein_cc
ENSG0000(	1113.636	0.188987	0.034133	5.536769	3.08E-08	1.58E-07	PPM1D	protein_cc
ENSG0000(	1113.278	-0.1529	0.074285	-2.05834	0.039558	0.06871	RASGRP3	protein_cc
ENSG0000(	1113.174	-0.2065	0.066577	-3.10171	0.001924	0.004452	PRR5L	protein_cc
ENSG0000(	1113.007	0.005185	0.109201	0.047483	0.962128	0.974074	ZFPM2	protein_cc
ENSG0000(	1112.757	0.032373	0.031128	1.039996	0.298342	0.390621	ZNF506	protein_cc
ENSG0000(	1112.648	0.193935	0.03752	5.168835	2.36E-07	1.05E-06	FAM206A	protein_cc
ENSG0000(	1112.567	-0.68812	0.118592	-5.80242	6.54E-09	3.73E-08	PPP1R1A	protein_cc
ENSG0000(	1112.564	-0.51972	0.091661	-5.67003	1.43E-08	7.73E-08	DGCR6	protein_cc
ENSG0000(	1111.915	0.13741	0.04428	3.103206	0.001914	0.004431	ZNF783	protein_cc
ENSG0000(	1111.82	-0.0311	0.076705	-0.4054	0.685182	0.758192	ECE2	protein_cc
ENSG0000(	1111.616	0.081875	0.060904	1.344311	0.178848	0.253942	REST	protein_cc
ENSG0000(	1111.132	0.290557	0.041268	7.040765	1.91E-12	1.91E-11	ZNF92	protein_cc
ENSG0000(	1111.063	0.115973	0.038427	3.017995	0.002545	0.005761	PLAGL2	protein_cc
ENSG0000(	1110.676	0.473851	0.047691	9.93589	2.91E-23	1.81E-21	NUDT1	protein_cc
ENSG0000(	1110.378	-0.19712	0.02912	-6.7692	1.30E-11	1.13E-10	TRMT1L	protein_cc
ENSG0000(	1110.066	0.418411	0.138023	3.031454	0.002434	0.005531	COL28A1	protein_cc
ENSG0000(	1110.052	0.392934	0.058114	6.761434	1.37E-11	1.19E-10	HEBP2	protein_cc
ENSG0000(	1109.914	0.226592	0.03643	6.219873	4.98E-10	3.40E-09	OXLD1	protein_cc
ENSG0000(	1109.793	-0.60127	0.064631	-9.30316	1.36E-20	5.12E-19	OTUD1	protein_cc
ENSG0000(	1109.714	-0.13148	0.033966	-3.87093	0.000108	0.000315	DNAL4	protein_cc
ENSG0000(	1109.686	-0.45896	0.096791	-4.74175	2.12E-06	8.14E-06	LINC0067	lincRNA
ENSG0000(	1109.495	0.052354	0.026564	1.97088	0.048738	0.082599	METTL8	protein_cc
ENSG0000(	1109.289	0.494427	0.078709	6.281721	3.35E-10	2.35E-09	TGFB1I1	protein_cc
ENSG0000(	1108.347	-0.01127	0.038033	-0.2964	0.766924	0.825152	RRS1	protein_cc
ENSG0000(	1108.326	0.086635	0.025493	3.398412	0.000678	0.001705	TAF9	protein_cc
ENSG0000(	1108.199	-0.85746	0.125178	-6.84995	7.39E-12	6.71E-11	MGAT4C	protein_cc
ENSG0000(	1108.071	-1.07573	0.107326	-10.023	1.21E-23	8.14E-22	KCNQ5	protein_cc
ENSG0000(	1107.399	-0.99932	0.120464	-8.29561	1.08E-16	2.18E-15	VSTM2L	protein_cc
ENSG0000(	1106.981	0.086413	0.033226	2.60079	0.009301	0.01871	RITA1	protein_cc
ENSG0000(	1106.974	-0.17516	0.033806	-5.18138	2.20E-07	9.88E-07	SPOPL	protein_cc
ENSG0000(	1106.461	0.184482	0.039633	4.654742	3.24E-06	1.21E-05	IFT43	protein_cc
ENSG0000(	1106.279	-0.24717	0.044379	-5.56947	2.56E-08	1.33E-07	LRP12	protein_cc
ENSG0000(	1105.903	-0.23933	0.067629	-3.53883	0.000402	0.001057	IL4R	protein_cc
ENSG0000(	1105.615	-0.04005	0.042033	-0.95292	0.340632	0.435726	ERCC5	protein_cc
ENSG0000(	1105.606	0.853581	0.152587	5.59408	2.22E-08	1.17E-07	ADM	protein_cc
ENSG0000(	1105.077	0.166313	0.037669	4.415117	1.01E-05	3.48E-05	SLC36A4	protein_cc
ENSG0000(	1103.995	0.272935	0.040621	6.719099	1.83E-11	1.56E-10	FBXO22	protein_cc
ENSG0000(	1103.89	0.00183	0.030231	0.060542	0.951724	0.966724	RNGTT	protein_cc
ENSG0000(	1103.866	-0.18482	0.051226	-3.60792	0.000309	0.000829	DIAPH2	protein_cc
ENSG0000(	1103.685	0.19068	0.049238	3.872621	0.000108	0.000313	NXT2	protein_cc
ENSG0000(	1103.514	-0.14878	0.108358	-1.37305	0.169736	0.243137	ASS1	protein_cc
ENSG0000(	1103.064	-0.14705	0.055217	-2.66314	0.007742	0.015864	MLLT3	protein_cc
ENSG0000(	1102.359	0.73652	0.109069	6.752771	1.45E-11	1.25E-10	DRAXIN	protein_cc
ENSG0000(	1102.174	-0.03825	0.03127	-1.22336	0.221192	0.303875	UTP23	protein_cc
ENSG0000(	1101.561	0.413942	0.034338	12.05494	1.83E-33	8.84E-31	ING3	protein_cc
ENSG0000(	1101.469	-0.47863	0.138441	-3.45727	0.000546	0.001398	GJB1	protein_cc
ENSG0000(	1101.101	-0.17538	0.121688	-1.44121	0.149525	0.218183	PYGM	protein_cc
ENSG0000(	1101.09	-0.02564	0.067068	-0.3823	0.702235	0.772468	PTPN14	protein_cc
ENSG0000(	1101.067	0.297462	0.036901	8.06116	7.56E-16	1.33E-14	PGAP2	protein_cc
ENSG0000(	1100.954	0.265373	0.045481	5.834831	5.38E-09	3.11E-08	PRMT6	protein_cc
ENSG0000(	1100.79	0.221805	0.028239	7.854545	4.01E-15	6.27E-14	ELMOD3	protein_cc

ENSG0000(	1100.629	-0.1605	0.037368	-4.29506	1.75E-05	5.81E-05	BRMS1L	protein_cc
ENSG0000(	1099.782	0.076984	0.062801	1.225853	0.220254	0.302863	SDSL	protein_cc
ENSG0000(	1099.667	-0.01152	0.052108	-0.22115	0.824975	0.87167	AC093838	pseudoge
ENSG0000(	1099.271	-0.3475	0.082487	-4.21282	2.52E-05	8.14E-05	CMKLR1	protein_cc
ENSG0000(	1099.215	-0.40935	0.105435	-3.88244	0.000103	0.000301	PRR18	protein_cc
ENSG0000(	1098.97	0.171919	0.059322	2.898087	0.003754	0.008207	CSAD	protein_cc
ENSG0000(	1098.703	0.248245	0.091674	2.707897	0.006771	0.014034	MDFIC	protein_cc
ENSG0000(	1098.401	0.330051	0.049834	6.623012	3.52E-11	2.88E-10	MRPL23	protein_cc
ENSG0000(	1098.361	0.138697	0.042909	3.232357	0.001228	0.002945	CCBL2	protein_cc
ENSG0000(	1098.132	0.086468	0.049153	1.759166	0.078549	0.125277	MAP3K14	processed
ENSG0000(	1097.779	1.051099	0.147735	7.114758	1.12E-12	1.16E-11	APOL4	protein_cc
ENSG0000(	1097.547	-0.33878	0.053754	-6.30242	2.93E-10	2.08E-09	PLCL2	protein_cc
ENSG0000(	1097.515	0.515088	0.057954	8.8879	6.23E-19	1.79E-17	MRPS12	protein_cc
ENSG0000(	1097.337	0.261629	0.063073	4.148006	3.35E-05	0.000106	TTC38	protein_cc
ENSG0000(	1097.238	-0.11511	0.099267	-1.15965	0.246193	0.332592	NKAIN1	protein_cc
ENSG0000(	1097.176	0.229049	0.02849	8.039605	9.01E-16	1.56E-14	TMEM11	protein_cc
ENSG0000(	1097.072	-0.40014	0.068044	-5.88062	4.09E-09	2.41E-08	PRKCD	protein_cc
ENSG0000(	1097.048	0.009772	0.026686	0.366172	0.714237	0.782155	MRE11A	protein_cc
ENSG0000(	1096.617	-0.03651	0.069717	-0.52362	0.600541	0.685333	NAPRT1	protein_cc
ENSG0000(	1096.028	0.097664	0.022126	4.414061	1.01E-05	3.50E-05	COIL	protein_cc
ENSG0000(	1095.848	-0.41611	0.063634	-6.53902	6.19E-11	4.87E-10	MSRB3	protein_cc
ENSG0000(	1095.346	-0.15494	0.039991	-3.87438	0.000107	0.000311	KIAA1551	protein_cc
ENSG0000(	1095.335	0.164533	0.033313	4.938941	7.85E-07	3.23E-06	C11orf84	protein_cc
ENSG0000(	1095.042	0.02221	0.029784	0.745715	0.45584	0.552326	SLC25A26	protein_cc
ENSG0000(	1095.027	-0.3782	0.091091	-4.15192	3.30E-05	0.000104	DDX25	protein_cc
ENSG0000(	1094.894	-1.01551	0.166464	-6.1005	1.06E-09	6.87E-09	IGFN1	protein_cc
ENSG0000(	1094.747	-1.12338	0.126269	-8.89671	5.75E-19	1.66E-17	SYNGR3	protein_cc
ENSG0000(	1094.599	0.157645	0.033941	4.644724	3.41E-06	1.27E-05	TMEM69	protein_cc
ENSG0000(	1094.598	0.158882	0.037243	4.266134	1.99E-05	6.55E-05	NOC4L	protein_cc
ENSG0000(	1094.547	0.046791	0.102955	0.454481	0.649483	0.728171	FRZB	protein_cc
ENSG0000(	1093.513	-0.22769	0.06948	-3.27701	0.001049	0.002546	NOL4	protein_cc
ENSG0000(	1093.135	0.150103	0.069145	2.170841	0.029943	0.053564	B3GNT9	protein_cc
ENSG0000(	1092.848	-0.03216	0.032171	-0.99956	0.317523	0.411231	LRRC49	protein_cc
ENSG0000(	1092.263	0.059748	0.04135	1.444931	0.148477	0.216878	RP11-396	pseudoge
ENSG0000(	1092.231	0.174539	0.051628	3.380682	0.000723	0.001809	HOOK2	protein_cc
ENSG0000(	1090.735	-0.23635	0.081884	-2.8864	0.003897	0.008495	PIK3AP1	protein_cc
ENSG0000(	1090.474	0.213165	0.029907	7.12771	1.02E-12	1.06E-11	ZSCAN25	protein_cc
ENSG0000(	1090.277	-0.36903	0.046025	-8.01806	1.07E-15	1.84E-14	DTNB	protein_cc
ENSG0000(	1090.243	0.092	0.072946	1.261208	0.207234	0.287808	ARHGAP2	protein_cc
ENSG0000(	1090.046	0.182712	0.030666	5.958113	2.55E-09	1.56E-08	C2orf43	protein_cc
ENSG0000(	1089.903	-0.22157	0.027818	-7.96488	1.65E-15	2.76E-14	C16orf70	protein_cc
ENSG0000(	1089.719	0.102363	0.068548	1.493297	0.135359	0.20022	NEK11	protein_cc
ENSG0000(	1089.595	0.353427	0.049101	7.197939	6.11E-13	6.56E-12	AC004447	antisense
ENSG0000(	1089.001	0.009108	0.050094	0.181811	0.855731	0.895766	WNK3	protein_cc
ENSG0000(	1088.401	0.145971	0.038029	3.838435	0.000124	0.000356	CGRRF1	protein_cc
ENSG0000(	1088.228	0.74925	0.085878	8.724626	2.67E-18	6.97E-17	ARSD	protein_cc
ENSG0000(	1088.172	-0.35675	0.073752	-4.83716	1.32E-06	5.22E-06	GREB1	protein_cc
ENSG0000(	1088.148	-0.10398	0.031599	-3.29053	0.001	0.002435	SLC25A42	protein_cc
ENSG0000(	1088.124	0.371407	0.099886	3.718325	0.000201	0.000556	APOBEC3(	protein_cc
ENSG0000(	1087.978	0.365114	0.059758	6.109907	9.97E-10	6.50E-09	VKORC1	protein_cc
ENSG0000(	1087.829	0.120263	0.07483	1.607147	0.108022	0.165086	NKX2-2	protein_cc
ENSG0000(	1086.858	-0.03131	0.029943	-1.04561	0.295739	0.387719	CCDC174	protein_cc
ENSG0000(	1086.837	0.088619	0.033185	2.670475	0.007574	0.01555	C12orf44	protein_cc
ENSG0000(	1086.749	-0.35157	0.066731	-5.26847	1.38E-07	6.37E-07	RALGPS2	protein_cc
ENSG0000(	1086.653	-0.54051	0.077588	-6.96635	3.25E-12	3.13E-11	FAAH	protein_cc
ENSG0000(	1086.584	-0.04986	0.023315	-2.13842	0.032483	0.057615	ZBTB14	protein_cc
ENSG0000(	1086.428	0.094752	0.031535	3.004673	0.002659	0.005991	NSUN4	protein_cc
ENSG0000(	1086.253	0.077381	0.03092	2.502587	0.012329	0.024115	RIOK1	protein_cc
ENSG0000(	1086.052	-0.77288	0.076672	-10.0804	6.74E-24	4.81E-22	STXBP5	protein_cc

ENSG0000(	1085.585	0.014406	0.029735	0.484482	0.628044	0.70959	UPF3B	protein_cc
ENSG0000(	1085.525	-0.09796	0.047646	-2.05594	0.039789	0.069056	ERCC6L2	protein_cc
ENSG0000(	1084.939	0.267652	0.041688	6.420431	1.36E-10	1.01E-09	ZNF606	protein_cc
ENSG0000(	1084.559	0.16509	0.036115	4.571274	4.85E-06	1.76E-05	C1orf109	protein_cc
ENSG0000(	1084.152	-0.38166	0.108046	-3.53238	0.000412	0.00108	ENPP5	protein_cc
ENSG0000(	1084.011	0.572159	0.114576	4.993695	5.92E-07	2.48E-06	PIFO	protein_cc
ENSG0000(	1083.721	-1.21674	0.129488	-9.3966	5.64E-21	2.26E-19	CABP1	protein_cc
ENSG0000(	1083.049	0.026383	0.030739	0.858279	0.390738	0.487315	NR2C1	protein_cc
ENSG0000(	1082.511	-0.11318	0.027372	-4.1349	3.55E-05	0.000112	RPP30	protein_cc
ENSG0000(	1081.674	-0.24393	0.056805	-4.29426	1.75E-05	5.83E-05	TCFL5	protein_cc
ENSG0000(	1081.579	0.363202	0.043493	8.350838	6.78E-17	1.42E-15	ZNF776	protein_cc
ENSG0000(	1081.187	0.16879	0.049398	3.416961	0.000633	0.001602	MORN2	protein_cc
ENSG0000(	1081.137	0.434133	0.107212	4.049305	5.14E-05	0.000158	C1RL	protein_cc
ENSG0000(	1080.97	-0.01115	0.043947	-0.2537	0.799724	0.852044	INCENP	protein_cc
ENSG0000(	1080.8	0.04354	0.047911	0.908767	0.363473	0.45947	GALNT7	protein_cc
ENSG0000(	1080.723	-0.11508	0.068205	-1.68733	0.09154	0.143077	RNF149	protein_cc
ENSG0000(	1080.553	0.024703	0.042737	0.578016	0.563254	0.651825	SFXN4	protein_cc
ENSG0000(	1080.346	0.397704	0.064246	6.19037	6.00E-10	4.05E-09	WDR90	protein_cc
ENSG0000(	1079.953	-0.14941	0.030611	-4.88099	1.06E-06	4.25E-06	ATXN3	protein_cc
ENSG0000(	1079.86	0.007947	0.028323	0.280589	0.779025	0.835114	FAM104B	protein_cc
ENSG0000(	1079.778	0.096587	0.113114	0.853894	0.393164	0.489805	TSPAN18	protein_cc
ENSG0000(	1079.426	0.16862	0.044749	3.768147	0.000164	0.000463	PSEN2	protein_cc
ENSG0000(	1079.35	-0.62759	0.082225	-7.63262	2.30E-14	3.11E-13	LMOD1	protein_cc
ENSG0000(	1079.344	-0.11951	0.083938	-1.42377	0.154514	0.224476	KIAA1522	protein_cc
ENSG0000(	1079.073	0.263128	0.077742	3.384613	0.000713	0.001786	SH3BGR	protein_cc
ENSG0000(	1078.996	-0.01855	0.029627	-0.62597	0.531334	0.622932	POLK	protein_cc
ENSG0000(	1078.965	-0.0038	0.026867	-0.14147	0.887498	0.919911	ELL	protein_cc
ENSG0000(	1078.89	-0.2399	0.057099	-4.20139	2.65E-05	8.54E-05	IRS1	protein_cc
ENSG0000(	1078.878	-0.00435	0.029295	-0.14843	0.882002	0.915713	TTC13	protein_cc
ENSG0000(	1078.351	-0.41048	0.062914	-6.52435	6.83E-11	5.34E-10	ARHGAP2	protein_cc
ENSG0000(	1077.755	-0.19196	0.083158	-2.30839	0.020977	0.038871	CYTH4	protein_cc
ENSG0000(	1077.383	-0.04432	0.036222	-1.22368	0.221073	0.303756	MLLT10	protein_cc
ENSG0000(	1077.347	0.089295	0.035871	2.489321	0.012799	0.024938	GMPPB	protein_cc
ENSG0000(	1077.317	0.159327	0.0305	5.223838	1.75E-07	7.98E-07	APEX2	protein_cc
ENSG0000(	1076.086	0.083027	0.044892	1.849492	0.064387	0.105366	DNASE1	protein_cc
ENSG0000(	1075.785	-0.65963	0.081725	-8.07131	6.95E-16	1.23E-14	DGCR5	antisense
ENSG0000(	1075.64	-0.3545	0.086734	-4.08727	4.36E-05	0.000136	RASGRP1	protein_cc
ENSG0000(	1075.635	-0.5255	0.071904	-7.30844	2.70E-13	3.06E-12	TRPM2	protein_cc
ENSG0000(	1075.456	0.319003	0.037334	8.544652	1.29E-17	3.03E-16	ZNF559	protein_cc
ENSG0000(	1075.239	0.044423	0.022427	1.980745	0.04762	0.080893	RAD51D	protein_cc
ENSG0000(	1075.135	0.106664	0.032282	3.304112	0.000953	0.002329	RPF2	protein_cc
ENSG0000(	1074.778	-0.52563	0.089029	-5.90409	3.55E-09	2.12E-08	SYT6	protein_cc
ENSG0000(	1074.726	0.116206	0.078951	1.471867	0.141057	0.207439	SEC24D	protein_cc
ENSG0000(	1074.611	-0.3883	0.067336	-5.76654	8.09E-09	4.55E-08	SVIP	protein_cc
ENSG0000(	1074.399	0.025394	0.032072	0.791791	0.428482	0.525562	ZBTB6	protein_cc
ENSG0000(	1074.309	0.13226	0.053598	2.467616	0.013602	0.026365	RAMP2	protein_cc
ENSG0000(	1073.741	-0.12225	0.101904	-1.19969	0.23026	0.314241	TLR2	protein_cc
ENSG0000(	1073.578	0.029469	0.031435	0.937454	0.348525	0.443986	FAM35A	protein_cc
ENSG0000(	1073.456	0.037063	0.059947	0.618262	0.536403	0.627551	GCA	protein_cc
ENSG0000(	1073.422	0.210777	0.025349	8.315122	9.17E-17	1.87E-15	BUD13	protein_cc
ENSG0000(	1073.042	0.057706	0.031686	1.821215	0.068574	0.111288	FAM127C	protein_cc
ENSG0000(	1072.537	0.323273	0.054612	5.919489	3.23E-09	1.94E-08	DDB2	protein_cc
ENSG0000(	1072.33	-0.07532	0.0224	-3.36244	0.000773	0.001922	KCTD18	protein_cc
ENSG0000(	1072.091	0.130114	0.029157	4.462577	8.10E-06	2.83E-05	INTS9	protein_cc
ENSG0000(	1071.548	-0.25999	0.069697	-3.73021	0.000191	0.000532	ARHGAP2	protein_cc
ENSG0000(	1071.446	0.007199	0.032134	0.224031	0.822733	0.870173	TTC21B	protein_cc
ENSG0000(	1070.648	0.062422	0.030597	2.040125	0.041338	0.071428	SNAPC5	protein_cc
ENSG0000(	1070.587	0.01003	0.031438	0.319029	0.749704	0.811423	KNOP1	protein_cc
ENSG0000(	1070.401	-0.09574	0.039135	-2.44637	0.01443	0.027827	GK5	protein_cc

ENSG0000(	1070.245	0.296659	0.037614	7.887003	3.10E-15	4.93E-14	IFT20	protein_cc
ENSG0000(	1069.82	0.221105	0.02704	8.177018	2.91E-16	5.48E-15	NVL	protein_cc
ENSG0000(	1069.391	0.181456	0.042144	4.305657	1.66E-05	5.55E-05	FAM109A	protein_cc
ENSG0000(	1069.077	-1.29624	0.134975	-9.60357	7.72E-22	3.60E-20	NEFH	protein_cc
ENSG0000(	1068.746	0.117152	0.022801	5.138106	2.78E-07	1.22E-06	POLR3F	protein_cc
ENSG0000(	1068.213	-0.31807	0.053179	-5.98107	2.22E-09	1.37E-08	PPP1R3E	protein_cc
ENSG0000(	1067.718	-0.39771	0.054033	-7.36057	1.83E-13	2.13E-12	SMAD7	protein_cc
ENSG0000(	1067.639	-0.03031	0.097106	-0.31218	0.754906	0.815315	FGL2	protein_cc
ENSG0000(	1066.92	0.070904	0.053648	1.321654	0.186283	0.262849	SHMT1	protein_cc
ENSG0000(	1066.5	-0.09336	0.027724	-3.36764	0.000758	0.001889	PAPOLG	protein_cc
ENSG0000(	1066.471	-0.11138	0.071514	-1.55748	0.119356	0.179919	PARVB	protein_cc
ENSG0000(	1066.199	0.24191	0.142893	1.692948	0.090465	0.141645	PLAU	protein_cc
ENSG0000(	1066.184	0.112511	0.032211	3.49292	0.000478	0.001238	RRP8	protein_cc
ENSG0000(	1066.14	0.133784	0.035392	3.780086	0.000157	0.000443	ST6GALNA	protein_cc
ENSG0000(	1066.136	0.114174	0.028597	3.992464	6.54E-05	0.000197	PMS1	protein_cc
ENSG0000(	1065.932	0.356752	0.044414	8.032468	9.55E-16	1.65E-14	ZNF736	protein_cc
ENSG0000(	1065.908	0.232332	0.047916	4.848736	1.24E-06	4.95E-06	SLC35D1	protein_cc
ENSG0000(	1065.891	-0.08528	0.058894	-1.44796	0.147628	0.215814	ITPRIPL2	protein_cc
ENSG0000(	1065.82	-0.16947	0.072928	-2.32384	0.020134	0.037453	ACACB	protein_cc
ENSG0000(	1065.791	-0.18386	0.051107	-3.59756	0.000321	0.00086	FAM117B	protein_cc
ENSG0000(	1065.79	0.754312	0.129069	5.844262	5.09E-09	2.95E-08	FREM2	protein_cc
ENSG0000(	1065.746	0.003537	0.029154	0.121315	0.903441	0.931483	C5orf22	protein_cc
ENSG0000(	1065.659	-0.03907	0.043999	-0.88787	0.374609	0.471201	KIF7	protein_cc
ENSG0000(	1065.648	0.294737	0.060676	4.8576	1.19E-06	4.75E-06	CDH24	protein_cc
ENSG0000(	1065.574	-0.08319	0.047862	-1.73808	0.082196	0.13021	KLF16	protein_cc
ENSG0000(	1065.5	-0.14445	0.060311	-2.39505	0.016618	0.031559	ASAP2	protein_cc
ENSG0000(	1064.949	-0.12476	0.036779	-3.39212	0.000694	0.001741	ZCCHC6	protein_cc
ENSG0000(	1064.873	-0.36079	0.054426	-6.62893	3.38E-11	2.77E-10	SCAI	protein_cc
ENSG0000(	1064.743	-0.06449	0.045444	-1.41902	0.155892	0.226031	PANX1	protein_cc
ENSG0000(	1064	0.154914	0.033042	4.688352	2.75E-06	1.04E-05	TTC31	protein_cc
ENSG0000(	1063.861	-0.25622	0.060734	-4.21877	2.46E-05	7.94E-05	CHRD	protein_cc
ENSG0000(	1063.779	0.271628	0.04999	5.433708	5.52E-08	2.72E-07	EFCAB2	protein_cc
ENSG0000(	1063.712	0.106111	0.03466	3.061468	0.002203	0.005042	NTMT1	protein_cc
ENSG0000(	1063.103	-1.02895	0.097218	-10.584	3.54E-26	3.88E-24	NRG3	protein_cc
ENSG0000(	1062.55	0.222803	0.051314	4.341984	1.41E-05	4.76E-05	TMEM256	protein_cc
ENSG0000(	1062.433	-0.15694	0.054244	-2.89324	0.003813	0.008325	F8	protein_cc
ENSG0000(	1061.552	-0.46078	0.093101	-4.9492	7.45E-07	3.07E-06	COL7A1	protein_cc
ENSG0000(	1061.446	-0.0945	0.068178	-1.38604	0.165735	0.238202	ENKUR	protein_cc
ENSG0000(	1061.312	-0.0068	0.066048	-0.10297	0.917985	0.942155	MINA	protein_cc
ENSG0000(	1061.054	-0.37644	0.082175	-4.58091	4.63E-06	1.69E-05	ITGAM	protein_cc
ENSG0000(	1060.971	0.161971	0.035763	4.528984	5.93E-06	2.12E-05	MRPL48	protein_cc
ENSG0000(	1060.879	-0.16435	0.139058	-1.1819	0.237246	0.322263	MSTN	protein_cc
ENSG0000(	1060.523	-0.05708	0.116328	-0.4907	0.623639	0.705971	ICAM1	protein_cc
ENSG0000(	1059.082	0.014858	0.032865	0.452103	0.651194	0.729693	ZNF574	protein_cc
ENSG0000(	1058.964	0.208714	0.047129	4.428581	9.49E-06	3.29E-05	FAM195A	protein_cc
ENSG0000(	1058.716	-0.04412	0.086195	-0.51188	0.608735	0.692455	SLC1A5	protein_cc
ENSG0000(	1058.696	0.071244	0.02454	2.903198	0.003694	0.008084	GMCL1	protein_cc
ENSG0000(	1058.545	0.182449	0.044101	4.137104	3.52E-05	0.000111	GAS8	protein_cc
ENSG0000(	1058.518	0.487482	0.094652	5.150256	2.60E-07	1.15E-06	TP73-AS1	antisense
ENSG0000(	1058.426	0.051979	0.033335	1.559284	0.118929	0.179382	DTD2	protein_cc
ENSG0000(	1057.927	-0.03197	0.042493	-0.75244	0.451784	0.548381	CASP8AP2	processed
ENSG0000(	1057.701	0.777484	0.076496	10.16375	2.88E-24	2.17E-22	CDK2	protein_cc
ENSG0000(	1057.553	-0.22919	0.056997	-4.02102	5.79E-05	0.000176	3-Mar	protein_cc
ENSG0000(	1057.42	0.265181	0.035629	7.442941	9.85E-14	1.20E-12	MAPKAPK	lincRNA
ENSG0000(	1057.172	0.045969	0.072953	0.630126	0.528612	0.620271	TMPRSS5	protein_cc
ENSG0000(	1057.126	-0.07503	0.04358	-1.72175	0.085114	0.134295	CPOX	protein_cc
ENSG0000(	1056.86	0.240315	0.028505	8.430555	3.44E-17	7.55E-16	TYW1	protein_cc
ENSG0000(	1056.842	0.091567	0.032226	2.841434	0.004491	0.009659	PIGH	protein_cc
ENSG0000(	1056.49	-0.20526	0.036587	-5.61003	2.02E-08	1.07E-07	C6orf136	protein_cc

ENSG0000(	1056.414	-0.01739	0.053025	-0.32797	0.742936	0.806028	LINC0047(	lincRNA
ENSG0000(	1056.212	-0.16392	0.076409	-2.14536	0.031924	0.056716	ACHE	protein_cc
ENSG0000(	1056.073	0.25101	0.045637	5.500164	3.79E-08	1.92E-07	PNKP	protein_cc
ENSG0000(	1056.06	-0.2431	0.048136	-5.0502	4.41E-07	1.89E-06	C3orf58	protein_cc
ENSG0000(	1055.807	0.057927	0.022628	2.559992	0.010467	0.020827	ZNF346	protein_cc
ENSG0000(	1055.703	-1.23361	0.117938	-10.4598	1.32E-25	1.31E-23	AJAP1	protein_cc
ENSG0000(	1055.656	0.32584	0.042009	7.756425	8.74E-15	1.29E-13	PPIAP22	pseudoge
ENSG0000(	1055.622	0.203789	0.041167	4.95029	7.41E-07	3.06E-06	MALT1	protein_cc
ENSG0000(	1054.467	-0.03311	0.104489	-0.31688	0.751332	0.812672	HS3ST1	protein_cc
ENSG0000(	1054.41	1.170504	0.133904	8.741376	2.30E-18	6.09E-17	CNGA3	protein_cc
ENSG0000(	1054.346	0.135084	0.031816	4.245792	2.18E-05	7.11E-05	TP53RK	protein_cc
ENSG0000(	1054.335	0.121396	0.050036	2.426158	0.01526	0.029231	TMEM64	protein_cc
ENSG0000(	1053.675	0.046453	0.025676	1.809196	0.070421	0.113894	TMEM41A	protein_cc
ENSG0000(	1053.576	0.013778	0.03242	0.424982	0.67085	0.745904	SAYSD1	protein_cc
ENSG0000(	1052.542	0.475297	0.062997	7.54478	4.53E-14	5.84E-13	ZNF300	protein_cc
ENSG0000(	1052.452	-0.3771	0.048903	-7.71112	1.25E-14	1.78E-13	TAF3	protein_cc
ENSG0000(	1052.365	0.268092	0.051113	5.245041	1.56E-07	7.17E-07	MRI1	protein_cc
ENSG0000(	1052.246	0.241337	0.036108	6.683726	2.33E-11	1.96E-10	SP4	protein_cc
ENSG0000(	1052.112	0.159081	0.06442	2.469427	0.013533	0.026243	FDXR	protein_cc
ENSG0000(	1051.889	-0.32753	0.094645	-3.46064	0.000539	0.001382	ZNF469	protein_cc
ENSG0000(	1051.866	0.149915	0.037279	4.021402	5.79E-05	0.000176	BYSL	protein_cc
ENSG0000(	1051.739	-0.11582	0.033191	-3.48964	0.000484	0.001252	AP1AR	protein_cc
ENSG0000(	1050.859	-0.40824	0.124565	-3.27736	0.001048	0.002543	CHRNA4	protein_cc
ENSG0000(	1050.603	0.1731	0.037985	4.557051	5.19E-06	1.87E-05	RABL2B	protein_cc
ENSG0000(	1050.243	-0.04987	0.072216	-0.6905	0.489878	0.584375	PHF21B	protein_cc
ENSG0000(	1049.62	0.171957	0.033195	5.180248	2.22E-07	9.93E-07	FDX1	protein_cc
ENSG0000(	1049.599	0.092404	0.038637	2.391582	0.016776	0.031823	ISCA2	protein_cc
ENSG0000(	1049.348	0.458931	0.052759	8.698687	3.36E-18	8.66E-17	CTD-3018	processed
ENSG0000(	1049.252	0.145343	0.040325	3.604304	0.000313	0.00084	RFC5	protein_cc
ENSG0000(	1048.885	0.133544	0.04906	2.722062	0.006488	0.013498	FAM58A	protein_cc
ENSG0000(	1048.623	0.056684	0.04771	1.188079	0.234802	0.31946	SLC22A5	protein_cc
ENSG0000(	1048.603	0.23812	0.04822	4.93823	7.88E-07	3.24E-06	ZSWIM4	protein_cc
ENSG0000(	1048.573	0.281417	0.040015	7.032744	2.03E-12	2.01E-11	TMEM161	antisense
ENSG0000(	1048.525	0.080329	0.043138	1.86214	0.062583	0.102774	ATG4C	protein_cc
ENSG0000(	1048.307	0.210158	0.029524	7.118279	1.09E-12	1.13E-11	PAK1IP1	protein_cc
ENSG0000(	1046.709	-0.05277	0.067889	-0.77732	0.43697	0.534009	STAT5A	protein_cc
ENSG0000(	1046.482	-0.07177	0.02259	-3.1772	0.001487	0.003515	METTL6	protein_cc
ENSG0000(	1046.258	0.060986	0.082898	0.735673	0.46193	0.558045	EMP2	protein_cc
ENSG0000(	1045.377	0.104496	0.038292	2.728927	0.006354	0.013245	TMEM42	protein_cc
ENSG0000(	1044.675	0.297076	0.047591	6.242262	4.31E-10	2.97E-09	HAUS5	protein_cc
ENSG0000(	1044.625	-0.11635	0.0409	-2.84485	0.004443	0.009568	NRDE2	protein_cc
ENSG0000(	1044.275	0.058042	0.047604	1.219268	0.222743	0.305672	PVR	protein_cc
ENSG0000(	1043.658	-0.13494	0.052685	-2.56119	0.010431	0.020757	ACTR3B	protein_cc
ENSG0000(	1043.191	-0.39995	0.080568	-4.96416	6.90E-07	2.86E-06	IRF8	protein_cc
ENSG0000(	1043.086	0.255366	0.058489	4.36608	1.26E-05	4.29E-05	NOL3	protein_cc
ENSG0000(	1042.956	-0.6739	0.105296	-6.40005	1.55E-10	1.15E-09	CTNNA3	protein_cc
ENSG0000(	1042.606	-0.20006	0.091333	-2.19039	0.028496	0.051211	PLEK	protein_cc
ENSG0000(	1042.603	0.10092	0.031807	3.172918	0.001509	0.003563	NSMCE4A	protein_cc
ENSG0000(	1042.488	-0.21227	0.03893	-5.4526	4.96E-08	2.47E-07	CAND2	protein_cc
ENSG0000(	1042.256	0.02711	0.042957	0.631094	0.527979	0.619705	ZNF471	protein_cc
ENSG0000(	1041.805	0.028343	0.081585	0.3474	0.728291	0.793636	ATHL1	protein_cc
ENSG0000(	1041.733	0.811978	0.084218	9.64137	5.35E-22	2.57E-20	SMC4	protein_cc
ENSG0000(	1041.497	0.094909	0.050753	1.870018	0.061481	0.10115	CCDC101	protein_cc
ENSG0000(	1041.289	-0.2046	0.035952	-5.69098	1.26E-08	6.89E-08	CDC40	protein_cc
ENSG0000(	1041.214	-0.39918	0.080837	-4.93811	7.89E-07	3.24E-06	ABTB2	protein_cc
ENSG0000(	1040.799	0.189365	0.035011	5.408791	6.35E-08	3.10E-07	HEATR3	protein_cc
ENSG0000(	1040.624	0.370092	0.04569	8.100022	5.49E-16	9.90E-15	FANCG	protein_cc
ENSG0000(	1040.394	0.287038	0.044881	6.39552	1.60E-10	1.18E-09	ZNF542	pseudoge
ENSG0000(	1040.007	0.187338	0.042158	4.443737	8.84E-06	3.08E-05	ZNF614	protein_cc

ENSG0000(	1039.979	0.181551	0.034517	5.259835	1.44E-07	6.65E-07	TIMM9	protein_cc
ENSG0000(	1039.315	-0.09089	0.032242	-2.81901	0.004817	0.010292	IGHMBP2	protein_cc
ENSG0000(	1039.302	-0.46076	0.061162	-7.53341	4.94E-14	6.34E-13	RP11-295	protein_cc
ENSG0000(	1039.293	-0.30469	0.03915	-7.78253	7.11E-15	1.07E-13	DEPDC5	protein_cc
ENSG0000(	1039.291	-0.09357	0.044474	-2.10383	0.035393	0.062242	PGP	protein_cc
ENSG0000(	1038.784	-0.08448	0.031059	-2.7199	0.00653	0.013577	DNAJC14	protein_cc
ENSG0000(	1038.636	0.258775	0.051697	5.005612	5.57E-07	2.34E-06	PFKFB4	protein_cc
ENSG0000(	1038.106	-0.12716	0.056362	-2.25613	0.024063	0.044004	GDPD1	protein_cc
ENSG0000(	1037.803	0.293142	0.108534	2.700922	0.006915	0.014305	APOL1	protein_cc
ENSG0000(	1036.871	-0.42661	0.055955	-7.62424	2.45E-14	3.31E-13	CNNM2	protein_cc
ENSG0000(	1036.631	0.195516	0.057636	3.39229	0.000693	0.00174	CCDC84	protein_cc
ENSG0000(	1036.55	0.159452	0.041689	3.824782	0.000131	0.000375	TSEN54	protein_cc
ENSG0000(	1036.467	0.000715	0.089291	0.00801	0.993609	0.995764	SASH3	protein_cc
ENSG0000(	1036.426	0.174295	0.066758	2.610852	0.009032	0.018224	NIPSNAP3	protein_cc
ENSG0000(	1036.353	0.035732	0.05944	0.60115	0.54774	0.637947	FAM211B	protein_cc
ENSG0000(	1036.261	-0.44862	0.135989	-3.29893	0.000971	0.002369	PCSK1	protein_cc
ENSG0000(	1036.023	0.595751	0.098506	6.047863	1.47E-09	9.31E-09	ECHDC2	protein_cc
ENSG0000(	1035.711	-0.06968	0.043574	-1.59919	0.109779	0.167454	DTNBP1	protein_cc
ENSG0000(	1035.608	0.84189	0.08464	9.946696	2.61E-23	1.65E-21	SLC27A3	protein_cc
ENSG0000(	1035.423	-0.06217	0.033168	-1.87446	0.060867	0.100296	DCUN1D2	protein_cc
ENSG0000(	1035.327	-0.01391	0.029188	-0.47654	0.633693	0.714652	CEP120	protein_cc
ENSG0000(	1035.066	-0.03332	0.050015	-0.66623	0.505264	0.599045	MLH3	protein_cc
ENSG0000(	1034.904	0.252333	0.082571	3.055947	0.002244	0.005128	GPC2	protein_cc
ENSG0000(	1034.764	0.184938	0.036151	5.115777	3.12E-07	1.37E-06	TWISTNB	protein_cc
ENSG0000(	1034.654	-0.40254	0.041589	-9.67892	3.71E-22	1.84E-20	NMT2	protein_cc
ENSG0000(	1034.616	-0.08496	0.048156	-1.76432	0.077679	0.124105	GMIP	protein_cc
ENSG0000(	1034.234	0.279424	0.131072	2.131831	0.033021	0.058477	NAPSB	pseudoge
ENSG0000(	1033.902	-0.11592	0.03083	-3.75983	0.00017	0.000477	FUT11	protein_cc
ENSG0000(	1033.545	0.343148	0.035852	9.571216	1.06E-21	4.83E-20	C21orf59	protein_cc
ENSG0000(	1033.458	-0.12048	0.033915	-3.5524	0.000382	0.001008	TMEM135	protein_cc
ENSG0000(	1032.787	0.193574	0.02203	8.786736	1.54E-18	4.16E-17	TMEM199	protein_cc
ENSG0000(	1032.187	-0.1466	0.050253	-2.91726	0.003531	0.00776	C10orf2	protein_cc
ENSG0000(	1031.527	-0.06677	0.042394	-1.57508	0.115238	0.17464	AHI1	protein_cc
ENSG0000(	1031.219	0.196247	0.043799	4.480636	7.44E-06	2.63E-05	DHX34	protein_cc
ENSG0000(	1031.163	0.089822	0.032345	2.777025	0.005486	0.011593	ADAT1	protein_cc
ENSG0000(	1030.583	0.15557	0.027146	5.730935	9.99E-09	5.54E-08	LINC0090!	lincRNA
ENSG0000(	1030.341	0.220639	0.028528	7.734213	1.04E-14	1.51E-13	IQCB1	protein_cc
ENSG0000(	1029.892	0.132301	0.035589	3.717519	0.000201	0.000557	RPA3-AS1	protein_cc
ENSG0000(	1029.875	0.275261	0.075459	3.647833	0.000264	0.000718	PPCS	protein_cc
ENSG0000(	1029.754	-0.64014	0.092308	-6.93477	4.07E-12	3.85E-11	GUCY1A3	protein_cc
ENSG0000(	1029.675	0.030497	0.048084	0.634247	0.525919	0.617746	RAB4B	protein_cc
ENSG0000(	1029.674	0.090422	0.044627	2.026189	0.042745	0.073575	GSTZ1	protein_cc
ENSG0000(	1029.668	0.176631	0.050798	3.477139	0.000507	0.001306	ALDH16A1	protein_cc
ENSG0000(	1029.445	0.108701	0.03084	3.524726	0.000424	0.00111	ZNF800	protein_cc
ENSG0000(	1029.003	0.149099	0.03178	4.691576	2.71E-06	1.02E-05	CCDC71	protein_cc
ENSG0000(	1028.324	0.166157	0.033993	4.887997	1.02E-06	4.11E-06	TTC23	protein_cc
ENSG0000(	1027.388	-0.01228	0.050664	-0.24232	0.808531	0.859012	CNKSR3	protein_cc
ENSG0000(	1026.281	-0.37053	0.04392	-8.43653	3.27E-17	7.19E-16	FAM161B	protein_cc
ENSG0000(	1026.064	0.190993	0.03223	5.925954	3.10E-09	1.87E-08	NOM1	protein_cc
ENSG0000(	1026.041	-0.97236	0.100899	-9.63692	5.58E-22	2.68E-20	STXBP6	protein_cc
ENSG0000(	1026.018	0.160116	0.033612	4.763599	1.90E-06	7.36E-06	TMEM138	protein_cc
ENSG0000(	1025.882	0.249556	0.030368	8.217841	2.07E-16	3.99E-15	NAA38	protein_cc
ENSG0000(	1025.827	-0.22651	0.072277	-3.13388	0.001725	0.00403	LINC0026:	processed
ENSG0000(	1025.66	0.178709	0.05473	3.265258	0.001094	0.002646	DOK1	protein_cc
ENSG0000(	1025.533	-0.51751	0.084361	-6.1344	8.55E-10	5.63E-09	PPAPDC1/	protein_cc
ENSG0000(	1025.373	0.08358	0.047907	1.744643	0.081047	0.128663	CCDC28B	protein_cc
ENSG0000(	1024.779	-1.14803	0.117844	-9.74199	2.00E-22	1.04E-20	KCNH3	protein_cc
ENSG0000(	1024.731	0.086961	0.022809	3.812517	0.000138	0.000392	C18orf8	protein_cc
ENSG0000(	1023.998	-0.0088	0.092357	-0.09529	0.924082	0.946546	FILIP1L	protein_cc

ENSG0000(	1023.509	-0.09931	0.038251	-2.59621	0.009426	0.018935	ARHGAP1	protein_cc
ENSG0000(	1023.367	-0.16891	0.043838	-3.85295	0.000117	0.000337	TRIM62	protein_cc
ENSG0000(	1022.712	0.171096	0.038487	4.445584	8.77E-06	3.05E-05	PPAT	protein_cc
ENSG0000(	1022.681	-0.98716	0.100699	-9.80306	1.09E-22	6.00E-21	MCF2L2	protein_cc
ENSG0000(	1022.573	0.125289	0.036623	3.421036	0.000624	0.00158	NDOR1	protein_cc
ENSG0000(	1022.311	0.092345	0.031849	2.899496	0.003738	0.008172	DYNC2LI1	protein_cc
ENSG0000(	1022.162	0.150912	0.039773	3.794321	0.000148	0.00042	ZNF205	protein_cc
ENSG0000(	1021.743	-0.42892	0.088294	-4.85789	1.19E-06	4.74E-06	GPR153	protein_cc
ENSG0000(	1021.143	-0.01374	0.076925	-0.17863	0.858225	0.8976	PCDH18	protein_cc
ENSG0000(	1021.061	-0.3175	0.068912	-4.60734	4.08E-06	1.50E-05	FRMD4B	protein_cc
ENSG0000(	1020.691	-0.16166	0.046273	-3.49359	0.000477	0.001234	KBTBD7	protein_cc
ENSG0000(	1020.672	0.203071	0.062058	3.272268	0.001067	0.002586	PTGR1	protein_cc
ENSG0000(	1020.222	1.709769	0.168991	10.11752	4.62E-24	3.39E-22	EMILIN3	protein_cc
ENSG0000(	1019.497	0.063168	0.038288	1.649823	0.098979	0.153179	WDR24	protein_cc
ENSG0000(	1019.005	-0.05521	0.071994	-0.76687	0.443159	0.539938	NPAS2	protein_cc
ENSG0000(	1018.759	0.196061	0.071076	2.758462	0.005807	0.012203	RRAS	protein_cc
ENSG0000(	1018.674	0.460896	0.123683	3.726433	0.000194	0.00054	RSAD2	protein_cc
ENSG0000(	1018.329	-0.00223	0.073338	-0.03047	0.975692	0.982886	TTC3P1	pseudoge
ENSG0000(	1017.086	0.108224	0.041945	2.580163	0.009875	0.019755	NR1H3	protein_cc
ENSG0000(	1017.081	-0.19813	0.070478	-2.81129	0.004934	0.010528	TMEM173	protein_cc
ENSG0000(	1016.762	-0.04521	0.029448	-1.5353	0.12471	0.186731	XPNPEP3	protein_cc
ENSG0000(	1016.731	-0.41897	0.064821	-6.46351	1.02E-10	7.78E-10	NYNRIN	protein_cc
ENSG0000(	1016.436	-0.84409	0.084203	-10.0244	1.19E-23	8.04E-22	CPEB3	protein_cc
ENSG0000(	1016.31	-0.23558	0.080719	-2.91847	0.003518	0.007735	CSF2RA	protein_cc
ENSG0000(	1016.286	0.145288	0.030338	4.788986	1.68E-06	6.54E-06	USP28	protein_cc
ENSG0000(	1016.004	0.235859	0.061283	3.848675	0.000119	0.000343	MAP2K3	protein_cc
ENSG0000(	1015.881	-0.06546	0.034796	-1.88134	0.059926	0.098964	KIAA0586	protein_cc
ENSG0000(	1015.432	0.230673	0.036921	6.247731	4.16E-10	2.88E-09	RBM7	protein_cc
ENSG0000(	1015.401	-0.25659	0.048926	-5.24453	1.57E-07	7.19E-07	LRCH2	protein_cc
ENSG0000(	1015.306	0.053731	0.0379	1.417716	0.156274	0.226536	MTERFD3	protein_cc
ENSG0000(	1015.158	-0.18047	0.061023	-2.95746	0.003102	0.006893	GIMAP6	protein_cc
ENSG0000(	1014.625	-0.06967	0.039574	-1.76061	0.078304	0.124963	IFT88	protein_cc
ENSG0000(	1014.625	0.168482	0.072012	2.339657	0.019301	0.036062	AC084018	lincRNA
ENSG0000(	1014.404	0.167913	0.089266	1.881028	0.059968	0.099015	MYCN	protein_cc
ENSG0000(	1014.277	-0.26088	0.075585	-3.45146	0.000558	0.001426	SLC6A6	protein_cc
ENSG0000(	1014.243	-0.38303	0.04914	-7.79465	6.46E-15	9.75E-14	AXIN2	protein_cc
ENSG0000(	1014.007	0.016735	0.026444	0.632844	0.526835	0.618628	DCAF17	protein_cc
ENSG0000(	1013.863	-0.36976	0.075093	-4.92402	8.48E-07	3.46E-06	HPRT1	protein_cc
ENSG0000(	1013.624	0.215697	0.038567	5.592849	2.23E-08	1.17E-07	ZNF544	protein_cc
ENSG0000(	1013.385	-0.1535	0.066646	-2.3033	0.021262	0.039346	BCAR3	protein_cc
ENSG0000(	1013.303	0.311535	0.063134	4.934533	8.03E-07	3.29E-06	NSUN5	protein_cc
ENSG0000(	1013.267	0.177968	0.062587	2.843547	0.004461	0.009604	DDX58	protein_cc
ENSG0000(	1012.941	-0.33529	0.043621	-7.68646	1.51E-14	2.12E-13	LRRC8C	protein_cc
ENSG0000(	1012.707	0.172237	0.038049	4.526753	5.99E-06	2.14E-05	PQLC2	protein_cc
ENSG0000(	1012.231	0.175306	0.153503	1.142033	0.25344	0.340871	LYZ	protein_cc
ENSG0000(	1012.104	0.248494	0.050883	4.883604	1.04E-06	4.20E-06	AGA	protein_cc
ENSG0000(	1011.933	0.119612	0.029389	4.069977	4.70E-05	0.000145	ANKRA2	protein_cc
ENSG0000(	1011.917	0.24482	0.033216	7.370654	1.70E-13	1.99E-12	MAD2L1B	protein_cc
ENSG0000(	1011.261	0.015982	0.072428	0.220664	0.825354	0.871914	SLC26A2	protein_cc
ENSG0000(	1011.109	0.107029	0.037041	2.889461	0.003859	0.008419	PLEKHA8	protein_cc
ENSG0000(	1010.997	0.202489	0.057222	3.538666	0.000402	0.001057	JOSD2	protein_cc
ENSG0000(	1010.957	-0.14678	0.033241	-4.41555	1.01E-05	3.48E-05	TCEANC2	protein_cc
ENSG0000(	1010.71	0.08783	0.029717	2.955497	0.003122	0.006933	PELO	protein_cc
ENSG0000(	1010.295	-0.13584	0.059492	-2.28332	0.022412	0.041285	CCDC88B	protein_cc
ENSG0000(	1010.16	0.109812	0.030746	3.571635	0.000355	0.000943	TGS1	protein_cc
ENSG0000(	1009.897	0.337858	0.03448	9.798645	1.14E-22	6.23E-21	RINT1	protein_cc
ENSG0000(	1009.777	0.094275	0.055898	1.686569	0.091686	0.143273	LIMD2	protein_cc
ENSG0000(	1009.724	0.03616	0.024823	1.456712	0.145196	0.212727	ZNF830	protein_cc
ENSG0000(	1009.659	0.175668	0.032168	5.461016	4.73E-08	2.36E-07	CHRAC1	protein_cc

ENSG0000(	1009.558	-0.07104	0.035089	-2.02443	0.042926	0.07384	ZNF589	protein_cc
ENSG0000(	1009.401	0.597285	0.084035	7.107558	1.18E-12	1.22E-11	MREG	protein_cc
ENSG0000(	1009.022	-0.26448	0.071052	-3.72238	0.000197	0.000548	ARHGAP3	protein_cc
ENSG0000(	1008.29	-0.06523	0.044041	-1.48109	0.138582	0.204363	C9orf9	protein_cc
ENSG0000(	1008.217	-0.18885	0.026902	-7.01996	2.22E-12	2.19E-11	FAM175B	protein_cc
ENSG0000(	1007.819	0.240213	0.037058	6.482165	9.04E-11	6.94E-10	MSANTD3	protein_cc
ENSG0000(	1007.719	-0.0032	0.031971	-0.10019	0.920194	0.943739	THOC5	protein_cc
ENSG0000(	1007.632	-0.11018	0.06038	-1.8247	0.068046	0.110513	GCLM	protein_cc
ENSG0000(	1007.566	-0.10926	0.032771	-3.33407	0.000856	0.002112	DGKA	protein_cc
ENSG0000(	1007.53	0.065721	0.036229	1.814033	0.069673	0.112866	DHRS1	protein_cc
ENSG0000(	1007.258	0.49964	0.045511	10.97836	4.86E-28	7.34E-26	PSMA2	protein_cc
ENSG0000(	1006.956	-0.25462	0.049476	-5.14627	2.66E-07	1.18E-06	STARD13	protein_cc
ENSG0000(	1006.662	0.123167	0.067416	1.82696	0.067706	0.110066	DDX60	protein_cc
ENSG0000(	1006.478	0.180848	0.050714	3.566007	0.000362	0.000961	ERMAP	protein_cc
ENSG0000(	1006.223	-0.54282	0.072412	-7.4962	6.57E-14	8.27E-13	FAM135B	protein_cc
ENSG0000(	1005.971	0.31412	0.037165	8.452037	2.86E-17	6.39E-16	ICT1	protein_cc
ENSG0000(	1005.767	0.355715	0.047993	7.411818	1.25E-13	1.49E-12	SLC35D2	protein_cc
ENSG0000(	1005.424	0.150121	0.037121	4.044125	5.25E-05	0.000161	ZBTB48	protein_cc
ENSG0000(	1004.889	0.139891	0.055164	2.535909	0.011216	0.02216	ZNF649	protein_cc
ENSG0000(	1004.592	-0.22893	0.034594	-6.61748	3.65E-11	2.98E-10	KIAA1432	protein_cc
ENSG0000(	1004.018	-0.12421	0.033838	-3.67068	0.000242	0.000661	ETFDH	protein_cc
ENSG0000(	1003.742	-0.19866	0.045377	-4.37796	1.20E-05	4.08E-05	NPHP4	protein_cc
ENSG0000(	1003.732	-0.30956	0.054393	-5.69126	1.26E-08	6.88E-08	FLI1	protein_cc
ENSG0000(	1003.337	0.128626	0.03435	3.744517	0.000181	0.000505	YRDC	protein_cc
ENSG0000(	1003.071	-0.43804	0.047556	-9.21104	3.23E-20	1.14E-18	CHIC1	protein_cc
ENSG0000(	1003.064	0.528409	0.060868	8.681286	3.91E-18	9.96E-17	CHAF1A	protein_cc
ENSG0000(	1003.052	0.254164	0.052649	4.827555	1.38E-06	5.47E-06	SLC30A7	protein_cc
ENSG0000(	1002.931	0.158793	0.031374	5.06123	4.17E-07	1.79E-06	MAK16	protein_cc
ENSG0000(	1002.83	0.140752	0.086186	1.633119	0.102444	0.157757	SLC7A7	protein_cc
ENSG0000(	1002.532	0.220755	0.041973	5.259466	1.44E-07	6.66E-07	NT5C	protein_cc
ENSG0000(	1002.058	0.140103	0.038867	3.604646	0.000313	0.000839	SURF2	protein_cc
ENSG0000(	1002.009	0.108627	0.034364	3.161046	0.001572	0.003699	ITFG2	protein_cc
ENSG0000(	1001.524	-0.39257	0.065137	-6.02677	1.67E-09	1.05E-08	TTC9	protein_cc
ENSG0000(	1001.2	-0.08324	0.030585	-2.72155	0.006498	0.013518	ZBTB25	protein_cc
ENSG0000(	1000.552	0.072191	0.03238	2.229487	0.025781	0.04683	MFSD8	protein_cc
ENSG0000(	999.8557	-0.11413	0.070715	-1.61397	0.106535	0.163138	C6orf3	antisense
ENSG0000(	999.8294	0.342753	0.058655	5.843498	5.11E-09	2.97E-08	TCTN2	protein_cc
ENSG0000(	999.3962	0.116606	0.069988	1.666071	0.095699	0.148743	MFNG	protein_cc
ENSG0000(	999.202	0.197407	0.029658	6.656173	2.81E-11	2.33E-10	ZNF75A	protein_cc
ENSG0000(	999.1211	-0.65106	0.105048	-6.19771	5.73E-10	3.87E-09	PPFIA2	protein_cc
ENSG0000(	998.6025	0.050012	0.033882	1.476095	0.139918	0.206008	SNRNP35	protein_cc
ENSG0000(	998.2274	-0.00596	0.041427	-0.14388	0.885593	0.918492	ZNF708	protein_cc
ENSG0000(	998.2163	0.406248	0.048143	8.438366	3.22E-17	7.10E-16	PET100	protein_cc
ENSG0000(	998.1933	-0.14111	0.025106	-5.62065	1.90E-08	1.01E-07	SRRD	protein_cc
ENSG0000(	998.1221	-0.29939	0.083545	-3.58359	0.000339	0.000903	LPAR5	protein_cc
ENSG0000(	997.9945	-0.08441	0.055826	-1.51205	0.130522	0.194034	FAM149A	protein_cc
ENSG0000(	997.9363	0.128798	0.046183	2.788863	0.005289	0.011221	C8orf82	protein_cc
ENSG0000(	997.8508	-0.05191	0.057807	-0.89799	0.369191	0.46552	AFMID	protein_cc
ENSG0000(	997.4258	0.162999	0.027936	5.834712	5.39E-09	3.12E-08	ZCCHC8	protein_cc
ENSG0000(	997.0826	-0.04944	0.032187	-1.53595	0.12455	0.186533	SLC25A32	protein_cc
ENSG0000(	997.0324	0.195919	0.160993	1.216945	0.223625	0.306709	S100A9	protein_cc
ENSG0000(	996.9771	0.16203	0.037494	4.321508	1.55E-05	5.19E-05	FAM98C	protein_cc
ENSG0000(	996.958	0.272283	0.043931	6.197968	5.72E-10	3.87E-09	ABHD11	protein_cc
ENSG0000(	996.2044	-0.52963	0.083931	-6.31026	2.79E-10	1.98E-09	ABCC9	protein_cc
ENSG0000(	995.9481	-0.10619	0.047976	-2.2133	0.026877	0.048592	FZD4	protein_cc
ENSG0000(	995.948	0.121098	0.06034	2.006916	0.044759	0.076603	RGS19	protein_cc
ENSG0000(	995.8534	0.482666	0.080591	5.989066	2.11E-09	1.31E-08	PARP12	protein_cc
ENSG0000(	995.7354	-0.24256	0.128233	-1.89159	0.058546	0.096984	KCNK3	protein_cc
ENSG0000(	995.5093	0.410594	0.100511	4.085086	4.41E-05	0.000137	CFH	protein_cc

ENSG0000(	995.5037	0.014992	0.029607	0.50636	0.612604	0.696047	TTF1	protein_cc
ENSG0000(	995.3277	0.064849	0.045803	1.415813	0.15683	0.227223	DDT	protein_cc
ENSG0000(	995.1486	0.244015	0.093047	2.62249	0.008729	0.017673	GBP4	protein_cc
ENSG0000(	995.0341	-1.2399	0.16217	-7.64569	2.08E-14	2.85E-13	CCK	protein_cc
ENSG0000(	994.8098	-0.24756	0.049475	-5.0037	5.62E-07	2.36E-06	TSHZ3	protein_cc
ENSG0000(	993.8721	0.223064	0.028942	7.707268	1.29E-14	1.82E-13	MYNN	protein_cc
ENSG0000(	993.8654	-0.00764	0.03321	-0.23002	0.818078	0.86661	CDAN1	protein_cc
ENSG0000(	993.4916	0.116871	0.034438	3.393665	0.00069	0.001732	GPN3	protein_cc
ENSG0000(	993.0959	0.175976	0.031505	5.585754	2.33E-08	1.22E-07	TPRKB	protein_cc
ENSG0000(	993.001	-0.17748	0.042573	-4.16892	3.06E-05	9.76E-05	LRRC27	protein_cc
ENSG0000(	992.579	0.09414	0.028955	3.251227	0.001149	0.002768	TAF6L	protein_cc
ENSG0000(	992.5692	0.099685	0.071797	1.388428	0.165007	0.237289	METRNL	protein_cc
ENSG0000(	992.4551	0.105047	0.042365	2.47956	0.013154	0.025575	CCDC159	protein_cc
ENSG0000(	992.4098	-1.17493	0.162531	-7.229	4.87E-13	5.29E-12	CRYM	protein_cc
ENSG0000(	992.3544	-0.33175	0.062077	-5.34416	9.08E-08	4.33E-07	SHROOM4	protein_cc
ENSG0000(	992.3462	0.049202	0.037555	1.310138	0.190149	0.26744	C19orf54	protein_cc
ENSG0000(	992.0501	0.114087	0.070227	1.624541	0.10426	0.160117	PCDHB9	pseudoge
ENSG0000(	991.9357	0.231583	0.026908	8.606551	7.53E-18	1.84E-16	MKS1	protein_cc
ENSG0000(	991.6404	0.083351	0.043095	1.934116	0.053099	0.08907	SFI1	protein_cc
ENSG0000(	991.6247	-0.05068	0.054601	-0.92826	0.353271	0.44883	TMEM200	protein_cc
ENSG0000(	991.4662	0.017076	0.025568	0.667867	0.504218	0.598054	TRNT1	protein_cc
ENSG0000(	991.3565	-0.00446	0.04736	-0.09412	0.925016	0.947126	MVK	protein_cc
ENSG0000(	991.3139	-0.04795	0.034434	-1.39251	0.163767	0.235786	RNF31	protein_cc
ENSG0000(	991.3113	-0.65826	0.08806	-7.47509	7.71E-14	9.58E-13	MYO1D	protein_cc
ENSG0000(	991.2002	-0.29708	0.114481	-2.59504	0.009458	0.018995	LGI1	protein_cc
ENSG0000(	991.1812	0.303022	0.070116	4.321722	1.55E-05	5.18E-05	MAP3K6	protein_cc
ENSG0000(	991.0076	-0.07828	0.05177	-1.51211	0.130505	0.194017	DOHH	protein_cc
ENSG0000(	990.7332	0.178748	0.03508	5.095402	3.48E-07	1.51E-06	TXNDC9	protein_cc
ENSG0000(	990.7167	-0.78242	0.08148	-9.60255	7.80E-22	3.63E-20	MYRIP	protein_cc
ENSG0000(	990.658	0.335805	0.051047	6.578332	4.76E-11	3.81E-10	MEX3D	protein_cc
ENSG0000(	990.4974	0.179719	0.047873	3.754055	0.000174	0.000488	LRRC45	protein_cc
ENSG0000(	990.4634	0.107928	0.038463	2.806047	0.005015	0.010687	CHRNB1	protein_cc
ENSG0000(	990.3066	0.315642	0.046116	6.844596	7.67E-12	6.94E-11	C11orf83	protein_cc
ENSG0000(	990.002	-0.06885	0.043267	-1.59138	0.111525	0.16977	CDK8	protein_cc
ENSG0000(	989.9567	0.067526	0.060795	1.11071	0.266693	0.35566	IL18BP	protein_cc
ENSG0000(	989.87	-1.5628	0.150067	-10.414	2.14E-25	2.00E-23	RBFOX3	protein_cc
ENSG0000(	989.7621	0.185103	0.031788	5.823135	5.78E-09	3.32E-08	C9orf37	protein_cc
ENSG0000(	989.6883	0.174443	0.034773	5.016591	5.26E-07	2.22E-06	FAM76B	protein_cc
ENSG0000(	989.082	0.7336	0.090436	8.111853	4.99E-16	9.04E-15	BLM	protein_cc
ENSG0000(	989.0213	0.075854	0.026179	2.897571	0.003761	0.008219	RAD51C	protein_cc
ENSG0000(	988.8436	-0.24502	0.049911	-4.90913	9.15E-07	3.72E-06	SH3BP1	protein_cc
ENSG0000(	987.6145	0.094961	0.046105	2.059648	0.039432	0.068506	LRRC37B	protein_cc
ENSG0000(	986.5696	0.142485	0.021905	6.504711	7.78E-11	6.03E-10	DHX35	protein_cc
ENSG0000(	986.2894	0.120822	0.028483	4.241844	2.22E-05	7.23E-05	ESCO1	protein_cc
ENSG0000(	986.127	0.102779	0.030438	3.376638	0.000734	0.001833	ISY1	protein_cc
ENSG0000(	985.7136	0.09795	0.028578	3.427504	0.000609	0.001546	COX18	protein_cc
ENSG0000(	985.587	0.203313	0.02833	7.176636	7.14E-13	7.61E-12	TMEM68	protein_cc
ENSG0000(	985.3748	0.059708	0.043304	1.378794	0.167958	0.24091	TXNL4B	protein_cc
ENSG0000(	985.2103	0.621544	0.132016	4.708112	2.50E-06	9.49E-06	RN7SK	misc_RNA
ENSG0000(	984.8534	0.062878	0.034387	1.828504	0.067474	0.109736	TRAPPC2	protein_cc
ENSG0000(	984.3235	-0.20924	0.054831	-3.81603	0.000136	0.000387	BHLHB9	protein_cc
ENSG0000(	984.1442	0.200359	0.042944	4.66553	3.08E-06	1.15E-05	COX17	protein_cc
ENSG0000(	983.8309	0.068784	0.043782	1.571056	0.11617	0.17585	EXOSC5	protein_cc
ENSG0000(	983.7735	-0.17313	0.039602	-4.37162	1.23E-05	4.19E-05	NDUFAF4	protein_cc
ENSG0000(	983.7153	0.140582	0.046625	3.015143	0.002569	0.005808	CNTRL	protein_cc
ENSG0000(	983.3818	0.228379	0.042185	5.413714	6.17E-08	3.03E-07	NTHL1	protein_cc
ENSG0000(	983.1875	-0.55153	0.069738	-7.90853	2.60E-15	4.20E-14	FAM124A	protein_cc
ENSG0000(	982.9952	-0.67519	0.091398	-7.38732	1.50E-13	1.77E-12	PCDHGC5	protein_cc
ENSG0000(	982.7898	0.233493	0.044403	5.25854	1.45E-07	6.69E-07	C20orf96	protein_cc

ENSG0000(	982.5843	0.178717	0.023643	7.559038	4.06E-14	5.28E-13	DBR1	protein_cc
ENSG0000(	982.5288	0.214546	0.026469	8.105463	5.25E-16	9.49E-15	EED	protein_cc
ENSG0000(	982.461	0.168077	0.054456	3.086474	0.002025	0.004669	RECQL	protein_cc
ENSG0000(	982.417	0.196106	0.040681	4.820544	1.43E-06	5.65E-06	COMMD1	protein_cc
ENSG0000(	981.5541	-0.50678	0.064667	-7.83669	4.63E-15	7.15E-14	HECW2	protein_cc
ENSG0000(	981.2221	0.292208	0.054028	5.408451	6.36E-08	3.11E-07	TAPBPL	protein_cc
ENSG0000(	981.1697	-0.15562	0.072544	-2.14524	0.031933	0.056729	ADAP2	protein_cc
ENSG0000(	981.0332	0.094242	0.072074	1.307569	0.191019	0.268499	PCDH12	protein_cc
ENSG0000(	980.9134	-0.43553	0.075671	-5.75556	8.64E-09	4.84E-08	TMEM150	protein_cc
ENSG0000(	980.428	0.11603	0.032473	3.573068	0.000353	0.000938	FUNDC1	protein_cc
ENSG0000(	979.7733	-0.17201	0.044013	-3.9082	9.30E-05	0.000273	HECTD2	protein_cc
ENSG0000(	979.643	0.394841	0.057178	6.905509	5.00E-12	4.65E-11	POLD1	protein_cc
ENSG0000(	979.3282	-0.2839	0.040586	-6.9949	2.65E-12	2.59E-11	DHFRL1	protein_cc
ENSG0000(	978.5686	0.311913	0.035499	8.786563	1.54E-18	4.17E-17	NIP7	protein_cc
ENSG0000(	978.3198	-0.0519	0.074333	-0.69821	0.485049	0.580068	RENBP	protein_cc
ENSG0000(	978.008	-0.04361	0.037787	-1.15405	0.248479	0.335229	BTD	protein_cc
ENSG0000(	977.7945	1.303699	0.146112	8.922596	4.55E-19	1.34E-17	MYBL2	protein_cc
ENSG0000(	977.0763	0.181194	0.038638	4.689562	2.74E-06	1.03E-05	MTHFD2L	protein_cc
ENSG0000(	976.1815	0.220666	0.090433	2.440105	0.014683	0.028256	FAM114A1	protein_cc
ENSG0000(	976.0813	0.029699	0.025826	1.149975	0.250154	0.337202	PWWP2A	protein_cc
ENSG0000(	975.9448	-0.03633	0.035618	-1.02011	0.307678	0.400765	TDRD3	protein_cc
ENSG0000(	975.9117	0.101595	0.063257	1.606077	0.108257	0.165427	TMEM204	protein_cc
ENSG0000(	975.9103	0.256092	0.076261	3.358106	0.000785	0.001951	ANTXR2	protein_cc
ENSG0000(	975.5712	0.318727	0.066909	4.763583	1.90E-06	7.36E-06	DDX11	protein_cc
ENSG0000(	974.7674	0.017479	0.039231	0.445548	0.655924	0.733607	MTF2	protein_cc
ENSG0000(	974.6667	-0.13377	0.081901	-1.63336	0.102392	0.157696	GLB1L2	protein_cc
ENSG0000(	974.548	0.881148	0.073715	11.95342	6.23E-33	2.67E-30	MRPS17	protein_cc
ENSG0000(	974.4995	-0.87008	0.122792	-7.08581	1.38E-12	1.41E-11	NKAIN2	protein_cc
ENSG0000(	974.3678	0.313117	0.11018	2.841872	0.004485	0.009647	STC2	protein_cc
ENSG0000(	973.9408	0.297932	0.027873	10.68895	1.15E-26	1.40E-24	DUSP12	protein_cc
ENSG0000(	973.6229	-0.43357	0.102983	-4.21009	2.55E-05	8.24E-05	FHDC1	protein_cc
ENSG0000(	973.6031	-0.02858	0.076835	-0.37195	0.709927	0.778721	CHCHD10	protein_cc
ENSG0000(	973.5779	-0.00331	0.089402	-0.03707	0.970426	0.979501	LY86	protein_cc
ENSG0000(	973.3352	-0.53005	0.066264	-7.99909	1.25E-15	2.13E-14	RPS6KA5	protein_cc
ENSG0000(	973.0257	0.461627	0.052505	8.792025	1.47E-18	3.98E-17	ERI1	protein_cc
ENSG0000(	972.8592	-0.24872	0.052529	-4.73485	2.19E-06	8.40E-06	FAM118A	protein_cc
ENSG0000(	972.7643	-0.60014	0.102144	-5.87545	4.22E-09	2.49E-08	THSD4	protein_cc
ENSG0000(	971.5991	0.143894	0.036231	3.971579	7.14E-05	0.000214	ZNF585B	protein_cc
ENSG0000(	971.5	0.000188	0.169687	0.001107	0.999117	0.99936	PCDHGB6	protein_cc
ENSG0000(	971.224	0.035275	0.047539	0.742021	0.458075	0.554495	MDM1	protein_cc
ENSG0000(	970.658	0.40585	0.048728	8.32897	8.15E-17	1.68E-15	PPIH	protein_cc
ENSG0000(	970.5472	-0.16876	0.033393	-5.05363	4.33E-07	1.86E-06	PARG	protein_cc
ENSG0000(	970.5409	0.042203	0.024854	1.698047	0.089499	0.140356	FAM104A	protein_cc
ENSG0000(	970.5193	0.243396	0.037789	6.440948	1.19E-10	8.96E-10	APOPT1	protein_cc
ENSG0000(	970.3654	-1.05161	0.196481	-5.35219	8.69E-08	4.16E-07	PRLHR	protein_cc
ENSG0000(	970.2293	-0.71448	0.125511	-5.69257	1.25E-08	6.83E-08	OGDHL	protein_cc
ENSG0000(	969.772	-0.37804	0.069654	-5.42736	5.72E-08	2.82E-07	GPR124	protein_cc
ENSG0000(	969.7304	0.150015	0.0325	4.615785	3.92E-06	1.44E-05	AP5S1	protein_cc
ENSG0000(	969.5758	-0.24439	0.039683	-6.15859	7.34E-10	4.89E-09	BBIP1	protein_cc
ENSG0000(	969.463	0.048158	0.027827	1.730582	0.083526	0.132047	CSTF2	protein_cc
ENSG0000(	969.4419	0.247444	0.040531	6.105042	1.03E-09	6.70E-09	ZFP30	protein_cc
ENSG0000(	969.3807	0.149862	0.032231	4.649694	3.32E-06	1.24E-05	PBDC1	protein_cc
ENSG0000(	969.2831	0.195392	0.036371	5.372248	7.78E-08	3.75E-07	NUDT22	protein_cc
ENSG0000(	968.4414	-0.21773	0.078625	-2.76922	0.005619	0.011845	LRRC16B	protein_cc
ENSG0000(	968.324	-1.177	0.138848	-8.47686	2.31E-17	5.24E-16	ATP8A2	protein_cc
ENSG0000(	968.0813	0.12564	0.04815	2.609372	0.009071	0.018297	P2RX4	protein_cc
ENSG0000(	967.701	0.055893	0.086817	0.643801	0.519705	0.612234	MST1	protein_cc
ENSG0000(	967.6685	0.122788	0.025011	4.909323	9.14E-07	3.71E-06	DDX59	protein_cc
ENSG0000(	967.635	-0.29619	0.065431	-4.52672	5.99E-06	2.14E-05	SLFN5	protein_cc

ENSG0000(	967.1388	0.195553	0.033947	5.760624	8.38E-09	4.70E-08	ZNF227	protein_cc
ENSG0000(	967.0489	0.105411	0.031665	3.32898	0.000872	0.002148	NCBP2-AS	lincRNA
ENSG0000(	966.9739	0.154953	0.055313	2.801414	0.005088	0.010829	PLEKHF2	protein_cc
ENSG0000(	966.8761	-0.14259	0.036903	-3.86385	0.000112	0.000323	PIGM	protein_cc
ENSG0000(	966.5228	-0.73222	0.132362	-5.53195	3.17E-08	1.62E-07	AIFM3	protein_cc
ENSG0000(	966.437	0.02343	0.025602	0.915184	0.360095	0.455977	ZFP64	protein_cc
ENSG0000(	966.397	0.042374	0.027396	1.546746	0.121924	0.183141	TBC1D22E	protein_cc
ENSG0000(	966.1153	0.065736	0.031467	2.089071	0.036701	0.064294	GANC	protein_cc
ENSG0000(	965.2547	-0.38096	0.076232	-4.99742	5.81E-07	2.44E-06	MAN1A1	protein_cc
ENSG0000(	965.2096	0.185245	0.037014	5.004775	5.59E-07	2.35E-06	EMG1	protein_cc
ENSG0000(	965.0664	0.252662	0.037214	6.78949	1.13E-11	9.89E-11	TMEM126	protein_cc
ENSG0000(	964.6599	0.370659	0.046971	7.891265	2.99E-15	4.78E-14	MRPL36	protein_cc
ENSG0000(	964.5916	-0.5575	0.097541	-5.71557	1.09E-08	6.03E-08	KCNAB1	protein_cc
ENSG0000(	964.4318	-0.25136	0.038741	-6.4882	8.69E-11	6.69E-10	SPRYD7	protein_cc
ENSG0000(	964.43	-0.14441	0.070744	-2.04125	0.041226	0.071268	LMCD1	protein_cc
ENSG0000(	964.3995	-0.07917	0.026899	-2.94338	0.003246	0.007186	LIN54	protein_cc
ENSG0000(	964.3949	0.183891	0.038622	4.761311	1.92E-06	7.43E-06	TMEM150	protein_cc
ENSG0000(	964.3925	-0.3572	0.079313	-4.50365	6.68E-06	2.37E-05	ADAM28	protein_cc
ENSG0000(	963.9671	-0.37636	0.048012	-7.83888	4.55E-15	7.04E-14	USP31	protein_cc
ENSG0000(	963.8625	-0.07203	0.028599	-2.51874	0.011777	0.023154	FBXL4	protein_cc
ENSG0000(	963.8623	0.234821	0.04174	5.625845	1.85E-08	9.83E-08	TAF12	protein_cc
ENSG0000(	963.6484	-0.90783	0.131082	-6.92565	4.34E-12	4.09E-11	SEC14L5	protein_cc
ENSG0000(	963.3746	-0.18907	0.05521	-3.42461	0.000616	0.001561	MAFK	protein_cc
ENSG0000(	963.3589	-0.68231	0.085979	-7.93582	2.09E-15	3.43E-14	PTPRU	protein_cc
ENSG0000(	963.066	0.192945	0.055433	3.480684	0.0005	0.00129	TMEM140	protein_cc
ENSG0000(	962.9565	0.13544	0.084907	1.595163	0.110676	0.168634	ABI3	protein_cc
ENSG0000(	962.944	0.001841	0.032883	0.055975	0.955361	0.969184	TTC8	protein_cc
ENSG0000(	962.9082	-1.33244	0.129244	-10.3095	6.39E-25	5.49E-23	PCLO	protein_cc
ENSG0000(	962.8566	0.232797	0.111279	2.092013	0.036437	0.063883	RGS6	protein_cc
ENSG0000(	962.7309	-0.28938	0.092914	-3.11446	0.001843	0.004279	CPS1	protein_cc
ENSG0000(	962.5544	0.022783	0.028752	0.792402	0.428127	0.525216	PPP3CC	protein_cc
ENSG0000(	962.4655	-0.08198	0.035236	-2.32649	0.019992	0.037211	CWF19L2	protein_cc
ENSG0000(	962.175	0.069283	0.032937	2.103499	0.035422	0.062279	TRIOBP	protein_cc
ENSG0000(	961.7514	0.04891	0.034393	1.422096	0.154998	0.225021	TMTC3	protein_cc
ENSG0000(	961.289	-0.04885	0.053947	-0.90557	0.365165	0.461239	DOCK11	protein_cc
ENSG0000(	961.2386	0.237882	0.034644	6.866515	6.58E-12	6.01E-11	POLM	protein_cc
ENSG0000(	960.7973	0.405232	0.041724	9.712159	2.68E-22	1.37E-20	RPL26L1	protein_cc
ENSG0000(	960.7178	0.104365	0.030693	3.400265	0.000673	0.001695	PCBP1-AS	antisense
ENSG0000(	960.2951	0.049812	0.038456	1.295281	0.195223	0.273586	FAM216A	protein_cc
ENSG0000(	960.161	0.100855	0.036591	2.756265	0.005847	0.012277	UGGT2	protein_cc
ENSG0000(	960.1314	0.389195	0.038362	10.14523	3.48E-24	2.61E-22	YEATS4	protein_cc
ENSG0000(	959.9849	0.219966	0.040247	5.46535	4.62E-08	2.31E-07	ZNF414	protein_cc
ENSG0000(	959.9765	0.072157	0.025292	2.85293	0.004332	0.009349	MPHOSPH	protein_cc
ENSG0000(	959.8785	-0.01526	0.047192	-0.32343	0.746372	0.808838	TDRKH	protein_cc
ENSG0000(	959.776	-0.01098	0.057263	-0.19178	0.847913	0.889871	DNHD1	protein_cc
ENSG0000(	959.4395	0.112969	0.048291	2.339345	0.019318	0.036084	ABHD3	protein_cc
ENSG0000(	958.9886	-0.00689	0.029922	-0.23012	0.817999	0.866548	C18orf32	protein_cc
ENSG0000(	958.3184	0.457197	0.048852	9.358888	8.06E-21	3.14E-19	BET1	protein_cc
ENSG0000(	958.0328	-0.22965	0.036967	-6.21232	5.22E-10	3.55E-09	FICD	protein_cc
ENSG0000(	957.6249	-0.05785	0.065305	-0.88591	0.375664	0.472252	HIVEP3	protein_cc
ENSG0000(	957.5719	-0.40023	0.071162	-5.62424	1.86E-08	9.91E-08	VLDLR	protein_cc
ENSG0000(	957.3268	0.183173	0.029734	6.160349	7.26E-10	4.84E-09	RBM48	protein_cc
ENSG0000(	957.2019	0.090036	0.027485	3.275865	0.001053	0.002556	FASTKD1	protein_cc
ENSG0000(	956.9339	0.019733	0.04268	0.462334	0.643842	0.723464	FKRP	protein_cc
ENSG0000(	956.8826	0.382273	0.119481	3.199453	0.001377	0.003274	RGS16	protein_cc
ENSG0000(	956.7392	-0.33958	0.070115	-4.84323	1.28E-06	5.08E-06	HOMER1	protein_cc
ENSG0000(	956.6963	-0.04415	0.037397	-1.18051	0.237796	0.322936	MPND	protein_cc
ENSG0000(	956.598	0.02424	0.075481	0.321141	0.748103	0.810202	AC004158	lincRNA
ENSG0000(	955.5014	0.042097	0.07885	0.533885	0.593421	0.678866	BDH1	protein_cc

ENSG0000(	955.2456	0.115473	0.049085	2.352531	0.018646	0.034961	TTC30A	protein_cc
ENSG0000(	955.1608	0.020849	0.030921	0.674254	0.50015	0.594078	SRBD1	protein_cc
ENSG0000(	954.7243	-0.26046	0.056271	-4.62862	3.68E-06	1.36E-05	DNAH1	protein_cc
ENSG0000(	954.6729	0.139052	0.038144	3.645454	0.000267	0.000725	THAP3	protein_cc
ENSG0000(	954.4119	0.113934	0.035149	3.241471	0.001189	0.002857	ZNF785	protein_cc
ENSG0000(	954.2643	0.113817	0.044971	2.53088	0.011378	0.022437	TUT1	protein_cc
ENSG0000(	954.0907	-0.61932	0.059873	-10.3439	4.46E-25	3.99E-23	GARNL3	protein_cc
ENSG0000(	953.6323	-0.03267	0.031686	-1.03089	0.302591	0.395341	GPATCH3	protein_cc
ENSG0000(	953.5853	0.073045	0.039619	1.843685	0.065229	0.106536	PYCRL	protein_cc
ENSG0000(	953.518	-0.06285	0.036073	-1.74229	0.081458	0.129186	TRIM32	protein_cc
ENSG0000(	953.4244	0.350966	0.091176	3.84934	0.000118	0.000342	FAM46A	protein_cc
ENSG0000(	953.0249	0.227798	0.039344	5.789951	7.04E-09	4.00E-08	ZNF470	protein_cc
ENSG0000(	952.7975	0.159978	0.022492	7.112511	1.14E-12	1.17E-11	ORC2	protein_cc
ENSG0000(	952.634	0.001518	0.075738	0.020049	0.984005	0.989041	STARD4	protein_cc
ENSG0000(	952.1112	0.23629	0.029144	8.107567	5.16E-16	9.34E-15	METTL23	protein_cc
ENSG0000(	951.9535	-0.05717	0.109521	-0.52201	0.601664	0.686271	CIITA	protein_cc
ENSG0000(	951.8929	-0.57723	0.100781	-5.72758	1.02E-08	5.64E-08	RGS9	protein_cc
ENSG0000(	951.7579	-0.02162	0.046943	-0.46066	0.645046	0.72454	RP11-421	antisense
ENSG0000(	951.7186	-0.94876	0.100851	-9.40757	5.08E-21	2.05E-19	SGCD	protein_cc
ENSG0000(	951.6197	0.122114	0.116225	1.050665	0.293412	0.385123	AC004540	antisense
ENSG0000(	951.4497	0.22419	0.081665	2.745231	0.006047	0.012657	GLI3	protein_cc
ENSG0000(	951.4053	0.019882	0.048871	0.406836	0.684129	0.75725	MOB3C	protein_cc
ENSG0000(	951.3617	-0.07946	0.031731	-2.50423	0.012272	0.024014	TTLL1	protein_cc
ENSG0000(	951.342	0.262982	0.046307	5.679049	1.35E-08	7.36E-08	NEDD1	protein_cc
ENSG0000(	951.2867	0.075933	0.064538	1.176556	0.239373	0.324712	ACVRL1	protein_cc
ENSG0000(	951.2095	-0.01632	0.067348	-0.24226	0.808578	0.859012	DOK4	protein_cc
ENSG0000(	950.8603	-0.09212	0.052466	-1.75577	0.079127	0.126059	GLCE	protein_cc
ENSG0000(	950.5652	-0.45879	0.07	-6.55417	5.60E-11	4.42E-10	AMPD3	protein_cc
ENSG0000(	950.3692	0.246609	0.030324	8.132461	4.21E-16	7.75E-15	TMEM62	protein_cc
ENSG0000(	950.2424	0.445268	0.122964	3.621124	0.000293	0.000791	RYR3	protein_cc
ENSG0000(	948.9803	-0.1926	0.078189	-2.46322	0.01377	0.026661	C12orf68	protein_cc
ENSG0000(	948.7695	0.106385	0.029133	3.651711	0.00026	0.000708	NARS2	protein_cc
ENSG0000(	948.0654	0.036069	0.037682	0.957194	0.338469	0.433428	HAUS4	protein_cc
ENSG0000(	947.8521	-0.03671	0.034546	-1.06276	0.287891	0.379138	DNAJC9	protein_cc
ENSG0000(	947.8411	-0.13062	0.04567	-2.86015	0.004234	0.009164	ACBD4	protein_cc
ENSG0000(	947.4999	-0.68329	0.100526	-6.7971	1.07E-11	9.43E-11	ASPA	protein_cc
ENSG0000(	947.208	-0.1523	0.086466	-1.76139	0.078172	0.124792	COL11A2	protein_cc
ENSG0000(	947.1666	0.216977	0.054464	3.983821	6.78E-05	0.000204	FAM173A	protein_cc
ENSG0000(	947.1639	-0.37513	0.092737	-4.04513	5.23E-05	0.00016	KIAA1199	protein_cc
ENSG0000(	947.0928	0.049707	0.077182	0.644016	0.519565	0.612122	NFATC4	protein_cc
ENSG0000(	946.8645	0.197926	0.040671	4.866462	1.14E-06	4.55E-06	E2F5	protein_cc
ENSG0000(	946.8259	0.041164	0.058795	0.700135	0.483843	0.578923	SPIRE2	protein_cc
ENSG0000(	946.7532	0.219733	0.073439	2.992032	0.002771	0.006214	KHNYN	protein_cc
ENSG0000(	946.0897	0.097689	0.025429	3.841573	0.000122	0.000352	UTP14A	protein_cc
ENSG0000(	946.045	0.106872	0.136855	0.780911	0.434855	0.53193	PCDHB5	protein_cc
ENSG0000(	945.8483	0.066257	0.032867	2.01593	0.043807	0.075181	GEMIN8	protein_cc
ENSG0000(	945.719	0.407452	0.046037	8.850546	8.71E-19	2.44E-17	ZNF107	protein_cc
ENSG0000(	945.6652	0.104061	0.054175	1.920833	0.054753	0.091493	BCAT2	protein_cc
ENSG0000(	945.5935	-0.04033	0.032973	-1.223	0.22133	0.304027	RWDD2A	protein_cc
ENSG0000(	945.4938	-0.22971	0.059469	-3.86264	0.000112	0.000325	SYNE3	protein_cc
ENSG0000(	945.3523	-0.02895	0.046343	-0.62459	0.53224	0.623763	TSTD2	protein_cc
ENSG0000(	945.1854	0.369258	0.044675	8.265463	1.39E-16	2.76E-15	GRPEL2	protein_cc
ENSG0000(	944.8165	0.109558	0.042093	2.602725	0.009249	0.018619	PTPN2	protein_cc
ENSG0000(	944.6071	-0.044	0.032864	-1.33885	0.18062	0.255991	ZXDB	protein_cc
ENSG0000(	944.2101	-0.41075	0.078342	-5.24306	1.58E-07	7.24E-07	C21orf91	protein_cc
ENSG0000(	943.9595	0.121818	0.035697	3.412532	0.000644	0.001626	IFNAR2	protein_cc
ENSG0000(	943.7746	0.160748	0.031164	5.158116	2.49E-07	1.11E-06	CDKAL1	protein_cc
ENSG0000(	942.922	-0.16339	0.025404	-6.43143	1.26E-10	9.50E-10	GID4	protein_cc
ENSG0000(	942.8228	0.286449	0.035201	8.137457	4.04E-16	7.44E-15	MED19	protein_cc

ENSG0000(	942.7926	-0.2667	0.071173	-3.74729	0.000179	0.0005	BAMBI	protein_cc
ENSG0000(	942.4689	0.52583	0.108592	4.842256	1.28E-06	5.10E-06	CENPV	protein_cc
ENSG0000(	942.2262	0.036213	0.03589	1.008989	0.31298	0.406409	COQ10A	protein_cc
ENSG0000(	942.0656	-0.359	0.094471	-3.80015	0.000145	0.000411	CYP2J2	protein_cc
ENSG0000(	941.7998	0.192814	0.039058	4.936616	7.95E-07	3.26E-06	NR2F6	protein_cc
ENSG0000(	941.7686	-0.45863	0.074565	-6.15071	7.71E-10	5.12E-09	CLMN	protein_cc
ENSG0000(	941.3752	-0.13981	0.080298	-1.74108	0.081669	0.129475	C3orf14	protein_cc
ENSG0000(	941.1277	-0.14825	0.085916	-1.72556	0.084427	0.133323	SLFN11	protein_cc
ENSG0000(	940.7286	-0.66744	0.066781	-9.99448	1.61E-23	1.06E-21	PLEKHG5	protein_cc
ENSG0000(	940.3245	-0.17724	0.076294	-2.3231	0.020174	0.037518	OAF	protein_cc
ENSG0000(	940.2828	-0.09602	0.064501	-1.48861	0.13659	0.201765	ACSF2	protein_cc
ENSG0000(	940.0611	0.331472	0.043178	7.676908	1.63E-14	2.27E-13	CHCHD5	protein_cc
ENSG0000(	939.7436	0.005249	0.02805	0.18715	0.851543	0.892655	MIEF2	protein_cc
ENSG0000(	939.4888	0.086253	0.077293	1.115924	0.264455	0.35319	SCNN1D	protein_cc
ENSG0000(	939.3175	0.405052	0.043112	9.395344	5.70E-21	2.29E-19	ITGAE	protein_cc
ENSG0000(	939.2144	0.065017	0.028934	2.247089	0.024634	0.044933	KIAA0753	protein_cc
ENSG0000(	938.7453	0.136594	0.057837	2.361695	0.018192	0.034196	STK33	protein_cc
ENSG0000(	938.54	-0.03238	0.076877	-0.42113	0.673659	0.748322	RP3-525N	antisense
ENSG0000(	938.5309	0.023158	0.027924	0.829323	0.406922	0.503725	MTHFSD	protein_cc
ENSG0000(	938.2234	-0.01789	0.03468	-0.51595	0.605891	0.689945	TRIM68	protein_cc
ENSG0000(	938.1382	-0.42562	0.110643	-3.8468	0.00012	0.000345	GPR27	protein_cc
ENSG0000(	938.1378	-0.00333	0.030346	-0.10986	0.912524	0.9383	RNF168	protein_cc
ENSG0000(	938.0466	-0.3737	0.046599	-8.01962	1.06E-15	1.82E-14	RP11-890	lincRNA
ENSG0000(	938.0272	-0.19152	0.059314	-3.22883	0.001243	0.002978	KANSL1L	protein_cc
ENSG0000(	937.766	-0.27687	0.037502	-7.38294	1.55E-13	1.83E-12	LIG4	protein_cc
ENSG0000(	937.7262	0.364871	0.039729	9.18408	4.15E-20	1.44E-18	ORC5	protein_cc
ENSG0000(	937.5861	-0.54798	0.060796	-9.01337	2.00E-19	6.22E-18	HHIPL1	protein_cc
ENSG0000(	937.4949	-0.12081	0.030867	-3.914	9.08E-05	0.000267	HPS6	protein_cc
ENSG0000(	937.2479	0.210716	0.041694	5.053896	4.33E-07	1.85E-06	COMMD3	protein_cc
ENSG0000(	936.9876	-0.11834	0.055526	-2.13133	0.033062	0.058539	STAG3	protein_cc
ENSG0000(	936.9782	0.089254	0.02911	3.066083	0.002169	0.004971	SIAH1	protein_cc
ENSG0000(	936.9314	0.119278	0.032266	3.696694	0.000218	0.000601	HACL1	protein_cc
ENSG0000(	936.8331	0.115357	0.039767	2.900836	0.003722	0.008139	PARP11	protein_cc
ENSG0000(	936.8307	-0.21064	0.035397	-5.95087	2.67E-09	1.62E-08	CUTC	protein_cc
ENSG0000(	936.0644	-0.45424	0.077169	-5.88625	3.95E-09	2.34E-08	LDLRAP1	protein_cc
ENSG0000(	935.9975	0.075328	0.037458	2.010975	0.044328	0.075943	TMEM39A	protein_cc
ENSG0000(	935.5435	-0.14419	0.050725	-2.84263	0.004474	0.009629	ZSWIM5	protein_cc
ENSG0000(	935.2314	0.474074	0.157112	3.017429	0.002549	0.00577	LOX	protein_cc
ENSG0000(	935.1552	0.368109	0.042186	8.725745	2.64E-18	6.91E-17	CENPO	protein_cc
ENSG0000(	934.8469	0.761536	0.067071	11.3541	7.08E-30	1.53E-27	MEIS1	protein_cc
ENSG0000(	934.8458	0.131259	0.033612	3.905141	9.42E-05	0.000277	ZNF408	protein_cc
ENSG0000(	934.2087	0.027009	0.028658	0.942467	0.345954	0.441339	SNIP1	protein_cc
ENSG0000(	934.063	-0.14843	0.033915	-4.37649	1.21E-05	4.11E-05	SLC25A16	protein_cc
ENSG0000(	933.8946	-0.44084	0.07258	-6.0739	1.25E-09	8.02E-09	GRASP	protein_cc
ENSG0000(	933.1695	-0.10892	0.082678	-1.31736	0.187719	0.264556	RAB33A	protein_cc
ENSG0000(	932.8862	0.073801	0.033991	2.171234	0.029914	0.053516	ZMAT5	protein_cc
ENSG0000(	932.041	0.763715	0.109462	6.977012	3.02E-12	2.91E-11	IQGAP2	protein_cc
ENSG0000(	931.851	0.263404	0.04814	5.471654	4.46E-08	2.23E-07	ZNF767	pseudoge
ENSG0000(	931.5963	0.162942	0.031515	5.170297	2.34E-07	1.04E-06	PSMD9	protein_cc
ENSG0000(	931.3893	-0.47026	0.068303	-6.88487	5.78E-12	5.32E-11	CDKN2D	protein_cc
ENSG0000(	931.1706	-0.04044	0.058272	-0.69401	0.487677	0.582425	ARHGAP4	protein_cc
ENSG0000(	931.0168	0.160777	0.032391	4.963618	6.92E-07	2.87E-06	AP4B1	protein_cc
ENSG0000(	930.9887	0.093412	0.038355	2.435478	0.014872	0.028568	PDE7A	protein_cc
ENSG0000(	930.7058	0.190609	0.035874	5.313222	1.08E-07	5.08E-07	SWI5	protein_cc
ENSG0000(	930.5035	0.253737	0.069263	3.663365	0.000249	0.000679	DHX58	protein_cc
ENSG0000(	930.2095	0.106006	0.038376	2.762327	0.005739	0.012073	ABHD13	protein_cc
ENSG0000(	929.9391	0.158094	0.039303	4.022428	5.76E-05	0.000176	IFT80	protein_cc
ENSG0000(	929.7042	-0.11245	0.062547	-1.79789	0.072194	0.116423	PVRL3	protein_cc
ENSG0000(	929.3765	0.078202	0.024808	3.152338	0.00162	0.003803	ZNF500	protein_cc

ENSG000(	929.2409	0.143638	0.032034	4.483967	7.33E-06	2.59E-05	ZCCHC7	protein_cc
ENSG000(	929.0615	-0.16725	0.045542	-3.67243	0.00024	0.000657	CTD-2336	antisense
ENSG000(	929.0429	-0.02965	0.04412	-0.6721	0.501518	0.595462	DNAJC1	protein_cc
ENSG000(	928.9229	-0.09434	0.059328	-1.59007	0.11182	0.170143	COBLL1	protein_cc
ENSG000(	928.8641	0.065774	0.042191	1.55895	0.119008	0.179467	ZNF493	protein_cc
ENSG000(	928.7293	0.232805	0.029668	7.846912	4.26E-15	6.63E-14	CNEP1R1	protein_cc
ENSG000(	928.5378	0.126142	0.026704	4.723746	2.32E-06	8.84E-06	TTC9C	protein_cc
ENSG000(	928.3507	0.257039	0.031845	8.071466	6.95E-16	1.23E-14	TMEM5	protein_cc
ENSG000(	926.5505	-0.17497	0.073347	-2.38552	0.017055	0.032288	TIAM2	protein_cc
ENSG000(	926.2731	-0.26082	0.029615	-8.80697	1.29E-18	3.52E-17	EXOC8	protein_cc
ENSG000(	926.1843	0.143879	0.090411	1.591389	0.111522	0.16977	CA14	protein_cc
ENSG000(	926.167	0.127618	0.025612	4.982737	6.27E-07	2.62E-06	SMYD4	protein_cc
ENSG000(	925.7942	0.172253	0.03671	4.692239	2.70E-06	1.02E-05	DARS2	protein_cc
ENSG000(	925.781	0.113401	0.037574	3.018043	0.002544	0.00576	HDHD1	protein_cc
ENSG000(	925.5634	-0.28505	0.083179	-3.42695	0.00061	0.001549	FAM65B	protein_cc
ENSG000(	925.4266	0.149229	0.033427	4.46427	8.03E-06	2.81E-05	SIRT7	protein_cc
ENSG000(	925.3308	0.408377	0.067667	6.035107	1.59E-09	1.00E-08	CHTF18	protein_cc
ENSG000(	925.1401	0.198747	0.060103	3.306773	0.000944	0.00231	DPF3	protein_cc
ENSG000(	924.518	0.096246	0.024257	3.967841	7.25E-05	0.000217	ATPAF2	protein_cc
ENSG000(	924.3274	-1.05839	0.097663	-10.8372	2.29E-27	3.12E-25	PDE1B	protein_cc
ENSG000(	924.1007	0.36101	0.125591	2.874494	0.004047	0.008793	OXTR	protein_cc
ENSG000(	924.0934	-0.36075	0.061423	-5.87323	4.27E-09	2.51E-08	IL6R	protein_cc
ENSG000(	924.0572	-0.23018	0.03896	-5.90819	3.46E-09	2.07E-08	ERO1LB	protein_cc
ENSG000(	923.7681	-0.0747	0.073167	-1.0209	0.3073	0.400335	STRIP2	protein_cc
ENSG000(	923.1899	0.956907	0.150038	6.37777	1.80E-10	1.31E-09	ETV4	protein_cc
ENSG000(	923.0757	-0.61155	0.149264	-4.09713	4.18E-05	0.00013	CUX2	protein_cc
ENSG000(	923.0034	0.228371	0.042874	5.326577	1.00E-07	4.75E-07	MRPS18A	protein_cc
ENSG000(	922.6845	-0.18839	0.03416	-5.51476	3.49E-08	1.78E-07	TRAF3IP1	protein_cc
ENSG000(	922.5167	0.250236	0.032188	7.774259	7.59E-15	1.13E-13	CCDC59	protein_cc
ENSG000(	922.3473	-0.80049	0.080037	-10.0015	1.50E-23	1.00E-21	SEMA4A	protein_cc
ENSG000(	922.2416	0.40857	0.057581	7.095535	1.29E-12	1.32E-11	SNHG15	lincRNA
ENSG000(	922.2067	0.086098	0.044087	1.952926	0.050828	0.085744	RPP25L	protein_cc
ENSG000(	921.6662	-0.25437	0.043454	-5.85377	4.81E-09	2.80E-08	EPM2A	protein_cc
ENSG000(	921.3253	-0.50441	0.093209	-5.41165	6.24E-08	3.06E-07	KIAA1211I	protein_cc
ENSG000(	921.0817	-0.84567	0.107623	-7.85778	3.91E-15	6.13E-14	PLCH2	protein_cc
ENSG000(	920.9551	0.081172	0.03086	2.630383	0.008529	0.017314	SNX24	protein_cc
ENSG000(	920.8466	0.288534	0.045597	6.327869	2.49E-10	1.78E-09	IRAK4	protein_cc
ENSG000(	920.7579	0.029147	0.044536	0.654458	0.512817	0.605781	GLTSCR1	protein_cc
ENSG000(	920.6039	-1.77868	0.156819	-11.3422	8.11E-30	1.75E-27	PRKCG	protein_cc
ENSG000(	920.2281	-0.42056	0.063815	-6.5902	4.39E-11	3.54E-10	RTN2	protein_cc
ENSG000(	919.8609	-0.04221	0.026937	-1.56689	0.117141	0.177074	STRADA	protein_cc
ENSG000(	919.4678	0.028425	0.078371	0.362697	0.716831	0.784286	LTBR	protein_cc
ENSG000(	919.2387	-0.04852	0.039534	-1.22723	0.219738	0.302234	GXYLT1	protein_cc
ENSG000(	919.2058	0.069606	0.034358	2.025925	0.042772	0.073607	ZBTB45	protein_cc
ENSG000(	919.1809	0.354145	0.045991	7.700317	1.36E-14	1.92E-13	MAGOH	protein_cc
ENSG000(	919.1297	0.161707	0.029135	5.550278	2.85E-08	1.47E-07	TRMT6	protein_cc
ENSG000(	918.632	-0.21318	0.041789	-5.10136	3.37E-07	1.47E-06	AP4S1	protein_cc
ENSG000(	917.8677	0.213891	0.042311	5.055173	4.30E-07	1.84E-06	ZNF688	protein_cc
ENSG000(	917.1827	0.712843	0.084172	8.468905	2.48E-17	5.59E-16	E2F1	protein_cc
ENSG000(	916.5139	0.071842	0.044385	1.61861	0.105531	0.161804	EGLN2	protein_cc
ENSG000(	916.357	-0.1766	0.074449	-2.37203	0.017691	0.033328	MMP28	protein_cc
ENSG000(	916.1486	-0.00962	0.053306	-0.18046	0.856789	0.896623	TOP1MT	protein_cc
ENSG000(	915.5285	0.369018	0.032831	11.2401	2.59E-29	5.12E-27	POLD3	protein_cc
ENSG000(	915.4218	-0.03598	0.020609	-1.74594	0.080822	0.128379	MTO1	protein_cc
ENSG000(	915.2071	0.396619	0.102008	3.8881	0.000101	0.000295	CAV2	protein_cc
ENSG000(	914.8274	0.43984	0.090921	4.837606	1.31E-06	5.21E-06	SLC16A4	protein_cc
ENSG000(	914.7918	0.274139	0.0661	4.147349	3.36E-05	0.000106	MSX1	protein_cc
ENSG000(	914.273	-0.24601	0.091478	-2.68924	0.007161	0.014764	YPEL4	protein_cc
ENSG000(	913.5596	-0.21127	0.040302	-5.24219	1.59E-07	7.28E-07	FBXL14	protein_cc

ENSG0000(	913.0979	0.011941	0.037842	0.315541	0.752351	0.813517	EID2	protein_cc
ENSG0000(	913.0413	0.378487	0.046194	8.193408	2.54E-16	4.83E-15	SKP2	protein_cc
ENSG0000(	912.335	-0.2071	0.11412	-1.81476	0.069561	0.112707	MS4A4A	protein_cc
ENSG0000(	911.9279	0.000259	0.031494	0.008229	0.993434	0.995637	SLC35A3	protein_cc
ENSG0000(	911.8878	0.00186	0.039133	0.047535	0.962087	0.974074	ZNF780B	protein_cc
ENSG0000(	911.6145	0.262071	0.045834	5.717794	1.08E-08	5.95E-08	CLN5	protein_cc
ENSG0000(	911.551	0.091725	0.034386	2.667508	0.007642	0.015677	URB2	protein_cc
ENSG0000(	911.3657	0.165166	0.061993	2.664268	0.007716	0.015815	ZNF334	protein_cc
ENSG0000(	911.1434	-0.05818	0.026559	-2.1907	0.028474	0.051178	FASTKD5	protein_cc
ENSG0000(	911.0473	0.215029	0.03275	6.565794	5.18E-11	4.12E-10	MRPL39	protein_cc
ENSG0000(	910.8685	-0.10036	0.035741	-2.80783	0.004988	0.010631	SRGAP2C	pseudoge
ENSG0000(	910.5721	-0.01889	0.071879	-0.26278	0.792717	0.846437	PICK1	protein_cc
ENSG0000(	910.2002	-0.1113	0.033896	-3.28353	0.001025	0.002493	IKZF4	protein_cc
ENSG0000(	910.1528	0.122614	0.035863	3.418947	0.000629	0.001591	TTC5	protein_cc
ENSG0000(	909.9339	-0.0101	0.039845	-0.25347	0.799907	0.852121	ABHD5	protein_cc
ENSG0000(	909.5071	0.053315	0.067991	0.784135	0.432961	0.52996	RP11-159	lincRNA
ENSG0000(	909.3887	0.302386	0.035197	8.591191	8.61E-18	2.09E-16	DCUN1D5	protein_cc
ENSG0000(	909.3086	-0.17798	0.107921	-1.64917	0.099112	0.153358	KIF26A	protein_cc
ENSG0000(	909.2926	-0.01146	0.037325	-0.30691	0.758913	0.818718	SLX4	protein_cc
ENSG0000(	909.136	0.107879	0.02755	3.915697	9.01E-05	0.000266	DDX31	protein_cc
ENSG0000(	909.0919	-0.23949	0.09344	-2.56307	0.010375	0.020653	DCC	protein_cc
ENSG0000(	908.8399	0.129926	0.063313	2.052107	0.040159	0.069643	LINC0096(	lincRNA
ENSG0000(	908.7948	0.40228	0.079883	5.035861	4.76E-07	2.02E-06	TNFRSF14	protein_cc
ENSG0000(	908.662	-0.07788	0.0379	-2.05479	0.0399	0.069233	ZC3H8	protein_cc
ENSG0000(	908.6158	-0.15865	0.040684	-3.89966	9.63E-05	0.000282	PEX11A	protein_cc
ENSG0000(	908.512	-0.13406	0.08574	-1.56353	0.117928	0.178074	ZNF518B	protein_cc
ENSG0000(	908.0958	-0.43692	0.04939	-8.84615	9.06E-19	2.53E-17	MYLIP	protein_cc
ENSG0000(	908.0551	-0.34425	0.056865	-6.05377	1.41E-09	9.01E-09	SLC16A14	protein_cc
ENSG0000(	907.5172	0.023853	0.082635	0.288651	0.772849	0.830094	CEBPA	protein_cc
ENSG0000(	906.2629	0.307207	0.06249	4.916085	8.83E-07	3.60E-06	TGIF2	protein_cc
ENSG0000(	906.0509	-0.11663	0.046975	-2.48277	0.013037	0.025359	PCTP	protein_cc
ENSG0000(	905.9095	0.405062	0.070778	5.72297	1.05E-08	5.79E-08	ZNF217	protein_cc
ENSG0000(	905.7094	0.002066	0.083502	0.024739	0.980263	0.986187	PARVG	protein_cc
ENSG0000(	905.617	0.196334	0.043493	4.514179	6.36E-06	2.26E-05	MORC4	protein_cc
ENSG0000(	905.4873	0.249675	0.047164	5.293766	1.20E-07	5.61E-07	MRPL12	protein_cc
ENSG0000(	905.2264	-0.35669	0.105891	-3.36848	0.000756	0.001883	LRRTM4	protein_cc
ENSG0000(	905.2115	0.50124	0.103489	4.843393	1.28E-06	5.07E-06	EYA2	protein_cc
ENSG0000(	905.2028	0.157567	0.037571	4.193804	2.74E-05	8.81E-05	RFX1	protein_cc
ENSG0000(	905.1191	-0.88603	0.087841	-10.0867	6.33E-24	4.53E-22	KIAA1107	protein_cc
ENSG0000(	905.0924	-0.09897	0.059939	-1.65118	0.098702	0.152796	STARD9	protein_cc
ENSG0000(	904.8922	0.465903	0.04377	10.6443	1.85E-26	2.18E-24	C7orf55	protein_cc
ENSG0000(	904.8584	0.215485	0.028533	7.552044	4.28E-14	5.54E-13	DUSP11	protein_cc
ENSG0000(	904.6396	-0.04984	0.064306	-0.77507	0.438301	0.535255	NEDD4	protein_cc
ENSG0000(	904.5861	0.69279	0.060183	11.51133	1.16E-30	3.09E-28	RPA3	protein_cc
ENSG0000(	904.5459	0.042742	0.06015	0.71059	0.477338	0.572873	UNC13D	protein_cc
ENSG0000(	904.349	-0.52076	0.065198	-7.98729	1.38E-15	2.33E-14	NT5DC1	protein_cc
ENSG0000(	904.045	0.189532	0.024137	7.852182	4.09E-15	6.38E-14	DDX19B	protein_cc
ENSG0000(	903.3294	0.240343	0.076255	3.151829	0.001623	0.003808	SLC25A20	protein_cc
ENSG0000(	902.9063	0.142702	0.053643	2.660192	0.00781	0.015991	C19orf26	protein_cc
ENSG0000(	902.7139	-0.01957	0.037637	-0.52006	0.60302	0.687265	ZKSCAN2	protein_cc
ENSG0000(	902.7044	0.003372	0.044719	0.075411	0.939888	0.958107	ZFP41	protein_cc
ENSG0000(	902.3946	-0.16272	0.05312	-3.06336	0.002189	0.005012	LYRM9	protein_cc
ENSG0000(	902.1613	-0.2657	0.059403	-4.47288	7.72E-06	2.71E-05	PAPSS2	protein_cc
ENSG0000(	902.1263	0.027455	0.032416	0.846948	0.397024	0.493627	RBM4	protein_cc
ENSG0000(	901.8853	0.173612	0.03529	4.919554	8.67E-07	3.54E-06	PSMG1	protein_cc
ENSG0000(	901.7965	-0.49381	0.077575	-6.36565	1.94E-10	1.41E-09	FNDC5	protein_cc
ENSG0000(	901.391	0.545639	0.09019	6.049854	1.45E-09	9.20E-09	SAMD9	protein_cc
ENSG0000(	901.3323	-0.25862	0.048176	-5.36821	7.95E-08	3.83E-07	PTCHD3P1	antisense
ENSG0000(	901.0086	-0.43741	0.061779	-7.08026	1.44E-12	1.46E-11	WDR17	protein_cc

ENSG0000(	900.9837	0.366797	0.088809	4.130189	3.62E-05	0.000114	TRIB3	protein_cc
ENSG0000(	900.8424	0.24094	0.05427	4.439643	9.01E-06	3.13E-05	ETHE1	protein_cc
ENSG0000(	900.7829	0.049827	0.025859	1.926845	0.053999	0.090381	BRCC3	protein_cc
ENSG0000(	900.5056	-0.16324	0.041627	-3.92138	8.80E-05	0.00026	CYB561D1	protein_cc
ENSG0000(	900.4763	-0.03656	0.080133	-0.45625	0.648214	0.727185	HVCN1	protein_cc
ENSG0000(	900.458	0.036733	0.032717	1.122779	0.261532	0.349945	ZNF277	protein_cc
ENSG0000(	900.3662	-0.15456	0.051705	-2.98926	0.002797	0.006266	ZNF33B	protein_cc
ENSG0000(	900.3593	-0.00403	0.03254	-0.12394	0.901364	0.930041	APIP	protein_cc
ENSG0000(	899.9332	0.169521	0.06971	2.4318	0.015024	0.028827	SHROOM:	protein_cc
ENSG0000(	899.5371	-0.22502	0.041641	-5.40386	6.52E-08	3.18E-07	BCORL1	protein_cc
ENSG0000(	899.3031	-0.02844	0.025788	-1.10263	0.270189	0.359633	RNF214	protein_cc
ENSG0000(	898.6573	-0.09679	0.053495	-1.80938	0.070392	0.113861	NFKB2	protein_cc
ENSG0000(	898.6511	-0.85922	0.092598	-9.27904	1.71E-20	6.30E-19	KCNN1	protein_cc
ENSG0000(	898.2442	-0.20728	0.03927	-5.27842	1.30E-07	6.06E-07	JAK2	protein_cc
ENSG0000(	898.2232	0.163921	0.063087	2.598337	0.009368	0.018831	PCDHB10	protein_cc
ENSG0000(	898.029	-0.37994	0.069432	-5.47209	4.45E-08	2.23E-07	DNAJC12	protein_cc
ENSG0000(	898.0273	0.263088	0.043764	6.01148	1.84E-09	1.15E-08	C1orf174	protein_cc
ENSG0000(	897.823	0.09759	0.030043	3.248337	0.001161	0.002793	TBP	protein_cc
ENSG0000(	897.2559	0.129772	0.046753	2.775701	0.005508	0.011636	LGALSL	protein_cc
ENSG0000(	896.9449	-0.16458	0.026737	-6.15533	7.49E-10	4.99E-09	UBE2D1	protein_cc
ENSG0000(	896.7866	-0.26181	0.059562	-4.39567	1.10E-05	3.79E-05	SERPINB9	protein_cc
ENSG0000(	896.7534	-0.25503	0.079	-3.22816	0.001246	0.002984	DYSF	protein_cc
ENSG0000(	895.8479	-0.09529	0.063734	-1.49505	0.134902	0.199608	DFNB31	protein_cc
ENSG0000(	895.7682	0.067787	0.02471	2.7433	0.006083	0.012726	UBOX5	protein_cc
ENSG0000(	895.5737	-0.62124	0.117146	-5.30313	1.14E-07	5.35E-07	ELAVL2	protein_cc
ENSG0000(	895.4162	0.060862	0.036485	1.66813	0.09529	0.148178	ISOC1	protein_cc
ENSG0000(	895.2516	0.510297	0.042379	12.0412	2.16E-33	1.02E-30	NR2C2AP	protein_cc
ENSG0000(	894.7979	-0.17541	0.109302	-1.60487	0.108523	0.165759	ISLR	protein_cc
ENSG0000(	894.6017	-0.06913	0.036893	-1.87371	0.060971	0.100435	ZNF250	protein_cc
ENSG0000(	894.5663	0.01747	0.02318	0.753688	0.451037	0.547798	ASB7	protein_cc
ENSG0000(	894.3714	-0.14203	0.095582	-1.48595	0.137293	0.202651	ALOX5	protein_cc
ENSG0000(	894.3051	0.187534	0.032639	5.745725	9.15E-09	5.10E-08	PTRH2	protein_cc
ENSG0000(	894.1114	0.141114	0.035524	3.972357	7.12E-05	0.000214	TMEM161	protein_cc
ENSG0000(	893.7639	0.182714	0.025943	7.042998	1.88E-12	1.88E-11	CXorf40B	protein_cc
ENSG0000(	893.7626	0.350305	0.046376	7.553664	4.23E-14	5.48E-13	TRNAU1A	protein_cc
ENSG0000(	893.6628	0.271367	0.026296	10.31977	5.74E-25	4.99E-23	E2F6	protein_cc
ENSG0000(	893.5009	-0.9449	0.110811	-8.52718	1.50E-17	3.48E-16	PNMA3	protein_cc
ENSG0000(	893.1314	0.43629	0.044497	9.80502	1.07E-22	5.91E-21	POLA2	protein_cc
ENSG0000(	892.8999	-0.01124	0.047876	-0.23476	0.814393	0.863684	TYSND1	protein_cc
ENSG0000(	892.8831	-0.07285	0.025958	-2.8065	0.005008	0.010673	PTCD2	protein_cc
ENSG0000(	892.5756	0.795176	0.077921	10.20491	1.88E-24	1.48E-22	ECT2	protein_cc
ENSG0000(	892.2426	0.021134	0.090249	0.234171	0.814852	0.86406	MAFF	protein_cc
ENSG0000(	892.2026	0.016891	0.101101	0.167067	0.867317	0.904854	FCGR1A	protein_cc
ENSG0000(	892.1941	-0.16602	0.070472	-2.35581	0.018482	0.034687	WAS	protein_cc
ENSG0000(	891.9301	0.025639	0.028111	0.912084	0.361724	0.457583	SRP19	protein_cc
ENSG0000(	891.8588	0.108047	0.031006	3.484756	0.000493	0.001272	MRPL1	protein_cc
ENSG0000(	891.6281	0.151957	0.061501	2.470791	0.013481	0.026149	C9orf3	protein_cc
ENSG0000(	891.5432	0.038876	0.040558	0.958533	0.337794	0.432661	CSPP1	protein_cc
ENSG0000(	891.2717	0.687632	0.102999	6.676131	2.45E-11	2.05E-10	SLC2A10	protein_cc
ENSG0000(	890.4887	-0.26448	0.05211	-5.07538	3.87E-07	1.67E-06	C19orf68	protein_cc
ENSG0000(	890.4744	-0.09625	0.039622	-2.42922	0.015131	0.029011	RELT	protein_cc
ENSG0000(	890.2233	0.049668	0.036963	1.343723	0.179038	0.254151	PMS2	protein_cc
ENSG0000(	890.0072	0.044611	0.029876	1.493212	0.135382	0.200231	MRPS31	protein_cc
ENSG0000(	889.8533	-0.16571	0.051844	-3.19626	0.001392	0.003308	ARHGAP2	protein_cc
ENSG0000(	889.5648	0.054654	0.028528	1.915791	0.055392	0.092433	BLZF1	protein_cc
ENSG0000(	889.5182	0.15968	0.048318	3.304814	0.00095	0.002324	MZT1	protein_cc
ENSG0000(	889.0309	-0.44384	0.073777	-6.01597	1.79E-09	1.12E-08	LDHD	protein_cc
ENSG0000(	888.506	0.193651	0.060224	3.215498	0.001302	0.003108	IDUA	protein_cc
ENSG0000(	888.2448	-0.04275	0.035898	-1.19099	0.233657	0.318081	SDR39U1	protein_cc

ENSG0000(	888.0277	0.137384	0.034036	4.036472	5.43E-05	0.000166	ZNF576	protein_cc
ENSG0000(	887.5102	-0.26007	0.039477	-6.5877	4.47E-11	3.59E-10	MTMR3	protein_cc
ENSG0000(	887.2823	0.300889	0.044479	6.764674	1.34E-11	1.16E-10	ZNF814	protein_cc
ENSG0000(	887.1149	0.338004	0.037695	8.966781	3.05E-19	9.20E-18	APBA3	protein_cc
ENSG0000(	887.056	0.301593	0.067255	4.484296	7.32E-06	2.58E-05	LEF1	protein_cc
ENSG0000(	886.7661	0.190479	0.081809	2.32833	0.019895	0.037048	ETNK2	protein_cc
ENSG0000(	886.727	0.238647	0.039448	6.049693	1.45E-09	9.21E-09	C8orf59	protein_cc
ENSG0000(	885.4678	0.802874	0.092502	8.679538	3.97E-18	1.01E-16	PABPC1L	protein_cc
ENSG0000(	885.2687	0.042048	0.034774	1.209182	0.226593	0.310097	MINPP1	protein_cc
ENSG0000(	884.96	-0.12774	0.041057	-3.11125	0.001863	0.004321	TRPC1	protein_cc
ENSG0000(	884.8393	-0.21123	0.08636	-2.44598	0.014446	0.027855	MYCL	protein_cc
ENSG0000(	884.5866	-1.0607	0.106865	-9.92563	3.22E-23	1.97E-21	DLGAP3	protein_cc
ENSG0000(	884.4239	0.044103	0.022281	1.979369	0.047774	0.081111	HDAC8	protein_cc
ENSG0000(	884.2106	-0.25271	0.048937	-5.16403	2.42E-07	1.08E-06	WWC2	protein_cc
ENSG0000(	883.5625	0.055301	0.083146	0.665114	0.505978	0.599681	LST1	protein_cc
ENSG0000(	883.0511	0.33434	0.096725	3.456586	0.000547	0.001401	RPSAP58	protein_cc
ENSG0000(	883.0086	0.283489	0.030461	9.306739	1.32E-20	4.96E-19	GEMIN6	protein_cc
ENSG0000(	882.5691	-0.45686	0.088828	-5.14318	2.70E-07	1.19E-06	SYT12	protein_cc
ENSG0000(	882.224	0.186199	0.032276	5.769024	7.97E-09	4.49E-08	NME7	protein_cc
ENSG0000(	881.7265	0.143554	0.049986	2.871901	0.00408	0.008859	ABCD1	protein_cc
ENSG0000(	881.6423	0.833788	0.078577	10.61113	2.65E-26	3.00E-24	MAD2L1	protein_cc
ENSG0000(	881.5719	0.172539	0.038859	4.440186	8.99E-06	3.13E-05	NDUFAF6	protein_cc
ENSG0000(	881.3544	-0.05976	0.046205	-1.29342	0.195865	0.274363	ZC2HC1A	protein_cc
ENSG0000(	881.2374	0.368457	0.035557	10.36239	3.68E-25	3.32E-23	PAXIP1-A:	lincRNA
ENSG0000(	881.2194	0.058064	0.022079	2.629836	0.008543	0.017339	ZNF689	protein_cc
ENSG0000(	880.4811	0.059674	0.03961	1.506521	0.131933	0.195892	TICAM1	protein_cc
ENSG0000(	880.4348	-0.16381	0.052166	-3.14011	0.001689	0.003951	SLC36A1	protein_cc
ENSG0000(	880.1534	-0.13909	0.042375	-3.28234	0.001029	0.002503	CCPG1	protein_cc
ENSG0000(	879.753	-0.04177	0.035994	-1.16043	0.245872	0.332257	ZFP14	protein_cc
ENSG0000(	879.4743	0.000265	0.118391	0.002241	0.998212	0.998892	C5AR1	protein_cc
ENSG0000(	879.117	0.264175	0.086296	3.061259	0.002204	0.005045	BACE2	protein_cc
ENSG0000(	879.0091	0.369453	0.068586	5.386735	7.17E-08	3.48E-07	INHBB	protein_cc
ENSG0000(	877.9752	0.001143	0.03044	0.037559	0.97004	0.979183	NOC3L	protein_cc
ENSG0000(	877.8989	-0.65319	0.084365	-7.74235	9.76E-15	1.42E-13	MPEG1	protein_cc
ENSG0000(	877.4992	0.024443	0.046333	0.527553	0.59781	0.68286	ZNF678	protein_cc
ENSG0000(	877.2407	0.33036	0.04074	8.108904	5.11E-16	9.25E-15	ZNF626	protein_cc
ENSG0000(	877.2133	0.203038	0.059836	3.393224	0.000691	0.001735	ATAD3B	protein_cc
ENSG0000(	876.8737	0.830242	0.096883	8.569574	1.04E-17	2.48E-16	ADAM19	protein_cc
ENSG0000(	876.0135	0.048308	0.02508	1.926203	0.054079	0.090504	HAUS3	protein_cc
ENSG0000(	875.1908	0.106687	0.040497	2.634427	0.008428	0.017129	CYB5R4	protein_cc
ENSG0000(	875.1849	0.093906	0.028792	3.261583	0.001108	0.002677	LCMT2	protein_cc
ENSG0000(	874.8918	0.278292	0.159229	1.747749	0.080507	0.127938	PCDHGA9	protein_cc
ENSG0000(	874.665	0.368531	0.035043	10.51659	7.24E-26	7.58E-24	WRAP53	protein_cc
ENSG0000(	874.651	0.112175	0.03825	2.932701	0.00336	0.007417	METAP1D	protein_cc
ENSG0000(	874.3644	0.032232	0.046346	0.69545	0.486774	0.581617	C11orf71	protein_cc
ENSG0000(	874.078	-0.8179	0.11451	-7.14259	9.16E-13	9.58E-12	KCNK1	protein_cc
ENSG0000(	873.5856	-0.53295	0.220192	-2.4204	0.015503	0.029653	USP9Y	protein_cc
ENSG0000(	873.5286	0.18346	0.034189	5.366004	8.05E-08	3.87E-07	JPX	lincRNA
ENSG0000(	872.2372	0.407762	0.097833	4.167937	3.07E-05	9.80E-05	PROM1	protein_cc
ENSG0000(	872.2085	-0.17542	0.045111	-3.88868	0.000101	0.000294	RP11-283	sense_ove
ENSG0000(	871.9986	0.314528	0.148726	2.114808	0.034446	0.060755	COL22A1	protein_cc
ENSG0000(	871.9393	-0.12603	0.035392	-3.56105	0.000369	0.000978	NEU3	protein_cc
ENSG0000(	871.5872	-0.17859	0.058392	-3.05846	0.002225	0.005088	BEND7	protein_cc
ENSG0000(	871.37	-0.29702	0.202275	-1.46838	0.142001	0.208656	NLGN4Y	protein_cc
ENSG0000(	871.2065	0.050179	0.045398	1.105314	0.269024	0.358328	MPHOSPH	protein_cc
ENSG0000(	870.6371	0.381946	0.07359	5.190186	2.10E-07	9.45E-07	ELTD1	protein_cc
ENSG0000(	870.5067	-0.41659	0.075423	-5.5233	3.33E-08	1.70E-07	FABP3	protein_cc
ENSG0000(	870.4497	0.174589	0.038058	4.58739	4.49E-06	1.64E-05	BOLA1	protein_cc
ENSG0000(	870.0549	-0.03718	0.06434	-0.57785	0.563367	0.65192	TRANK1	protein_cc

ENSG0000(	870.0221	-1.58716	0.133095	-11.925	8.77E-33	3.47E-30	RELN	protein_cc
ENSG0000(	869.9542	0.102554	0.033884	3.026595	0.002473	0.005612	POLB	protein_cc
ENSG0000(	869.9204	0.081201	0.030316	2.678537	0.007394	0.015211	ZNF429	protein_cc
ENSG0000(	869.6277	-0.02758	0.047661	-0.57873	0.562771	0.651468	RILPL2	protein_cc
ENSG0000(	869.5345	0.225895	0.032796	6.887883	5.66E-12	5.22E-11	ATG4D	protein_cc
ENSG0000(	869.2327	-0.04315	0.0606	-0.71198	0.476479	0.572143	SOGA2	protein_cc
ENSG0000(	868.9072	0.197217	0.042899	4.597194	4.28E-06	1.57E-05	CCNYL1	protein_cc
ENSG0000(	868.5192	0.121529	0.052738	2.30442	0.021199	0.03924	SPR	protein_cc
ENSG0000(	868.5099	-1.14622	0.121486	-9.43503	3.91E-21	1.62E-19	RET	protein_cc
ENSG0000(	868.3721	-1.23219	0.143141	-8.60823	7.42E-18	1.82E-16	CHRM1	protein_cc
ENSG0000(	868.3112	0.343099	0.115284	2.976123	0.002919	0.006522	WIPF3	protein_cc
ENSG0000(	868.2711	0.277215	0.040494	6.84578	7.61E-12	6.89E-11	ZDHHC15	protein_cc
ENSG0000(	867.9509	-1.02305	0.138126	-7.40666	1.30E-13	1.55E-12	GABRA2	protein_cc
ENSG0000(	867.9357	-0.01367	0.07908	-0.1729	0.862731	0.901076	LIN7A	protein_cc
ENSG0000(	866.7211	0.05328	0.034321	1.552413	0.120563	0.181401	GCFC2	protein_cc
ENSG0000(	866.4381	0.071934	0.080358	0.895175	0.370694	0.467047	GPRC5C	protein_cc
ENSG0000(	866.4284	0.127657	0.028034	4.553718	5.27E-06	1.90E-05	PIN4	protein_cc
ENSG0000(	866.1115	0.792187	0.103489	7.654818	1.94E-14	2.66E-13	RPL13AP5	pseudoge
ENSG0000(	866.0627	-0.05543	0.070892	-0.78189	0.434278	0.531305	LEPREL2	polymorpl
ENSG0000(	865.9559	-0.33051	0.03617	-9.13785	6.37E-20	2.15E-18	ZCCHC2	protein_cc
ENSG0000(	865.9172	0.223227	0.030359	7.352894	1.94E-13	2.24E-12	FANCL	protein_cc
ENSG0000(	865.75	0.106928	0.041605	2.570058	0.010168	0.020271	NFKBIE	protein_cc
ENSG0000(	865.3914	0.291242	0.062743	4.641819	3.45E-06	1.28E-05	SLC35F2	protein_cc
ENSG0000(	864.9778	0.033989	0.035807	0.949213	0.342512	0.437736	AASDH	protein_cc
ENSG0000(	864.9723	0.589129	0.047953	12.28555	1.08E-34	6.36E-32	RFC4	protein_cc
ENSG0000(	864.1953	0.030411	0.031807	0.956129	0.339007	0.433998	SPTY2D1	protein_cc
ENSG0000(	864.1185	0.307807	0.078759	3.90821	9.30E-05	0.000273	IFIH1	protein_cc
ENSG0000(	863.8189	0.199342	0.064722	3.079949	0.00207	0.004765	DEDD2	protein_cc
ENSG0000(	863.8156	0.12244	0.037554	3.260324	0.001113	0.002688	RABIF	protein_cc
ENSG0000(	863.4681	0.0635	0.04469	1.420902	0.155345	0.225397	TUBE1	protein_cc
ENSG0000(	863.0953	0.283709	0.039619	7.16093	8.01E-13	8.45E-12	ERVK3-1	processed
ENSG0000(	863.0945	0.48776	0.105064	4.642519	3.44E-06	1.28E-05	CTSK	protein_cc
ENSG0000(	863.0165	-0.63944	0.063736	-10.0325	1.10E-23	7.49E-22	RP11-690	sense_ove
ENSG0000(	862.8106	-1.0624	0.147657	-7.19505	6.24E-13	6.69E-12	FAM163B	protein_cc
ENSG0000(	862.7703	-0.0398	0.034828	-1.14277	0.253132	0.340513	DCLRE1A	protein_cc
ENSG0000(	862.3541	-0.48091	0.074089	-6.49097	8.53E-11	6.57E-10	KCND1	protein_cc
ENSG0000(	862.3338	-0.02002	0.079239	-0.25262	0.800561	0.852643	CASQ1	protein_cc
ENSG0000(	861.5984	-0.77931	0.112763	-6.91107	4.81E-12	4.48E-11	SH3GL3	protein_cc
ENSG0000(	861.5773	0.051338	0.039477	1.300438	0.193451	0.271435	LRIF1	protein_cc
ENSG0000(	860.8168	0.014683	0.043914	0.334357	0.73811	0.801914	PAQR7	protein_cc
ENSG0000(	860.2567	-0.06651	0.042778	-1.55481	0.119991	0.180652	ARL15	protein_cc
ENSG0000(	859.9658	-0.58144	0.120407	-4.82892	1.37E-06	5.43E-06	NWD1	protein_cc
ENSG0000(	859.8748	0.270592	0.043285	6.251409	4.07E-10	2.81E-09	ZNF211	protein_cc
ENSG0000(	859.8358	-1.19468	0.106199	-11.2494	2.33E-29	4.65E-27	ARHGAP4	protein_cc
ENSG0000(	859.5798	1.187534	0.119608	9.928548	3.13E-23	1.93E-21	CCDC109I	protein_cc
ENSG0000(	859.0067	0.5905	0.077209	7.648043	2.04E-14	2.80E-13	SPAG5	protein_cc
ENSG0000(	858.7842	0.266438	0.047563	5.601778	2.12E-08	1.12E-07	MANEA	protein_cc
ENSG0000(	858.7547	0.110397	0.037613	2.935051	0.003335	0.007367	PSMC1	protein_cc
ENSG0000(	858.7421	-0.15853	0.039819	-3.98134	6.85E-05	0.000206	FBXO10	protein_cc
ENSG0000(	858.4295	0.108644	0.03776	2.877204	0.004012	0.008727	ZBTB26	protein_cc
ENSG0000(	858.388	-0.9267	0.123796	-7.48572	7.12E-14	8.91E-13	RIMS1	protein_cc
ENSG0000(	858.0316	0.48299	0.102808	4.697999	2.63E-06	9.94E-06	TENM1	protein_cc
ENSG0000(	857.3658	0.298121	0.086704	3.438356	0.000585	0.001491	ARHGAP1	protein_cc
ENSG0000(	857.2095	-0.26017	0.061274	-4.24608	2.18E-05	7.11E-05	NCKAP5	protein_cc
ENSG0000(	856.969	0.167796	0.043485	3.858713	0.000114	0.00033	RP11-101	antisense
ENSG0000(	856.6728	-0.693	0.086138	-8.04522	8.61E-16	1.50E-14	ABLIM2	protein_cc
ENSG0000(	856.1837	-0.28231	0.060999	-4.62808	3.69E-06	1.37E-05	GPR85	protein_cc
ENSG0000(	856.1069	0.214416	0.083938	2.554451	0.010636	0.021116	FAM198A	protein_cc
ENSG0000(	855.8583	0.075225	0.030356	2.478106	0.013208	0.025669	LYSMD1	protein_cc

ENSG0000(	855.2838	0.155808	0.033007	4.720523	2.35E-06	8.97E-06	BPHL	protein_cc
ENSG0000(	855.1252	0.402694	0.081328	4.951497	7.36E-07	3.04E-06	SERTAD1	protein_cc
ENSG0000(	854.9191	0.105245	0.04317	2.437939	0.014771	0.028404	SETD5-AS	antisense
ENSG0000(	854.7812	0.244469	0.048751	5.014684	5.31E-07	2.24E-06	TMEM38B	protein_cc
ENSG0000(	854.6588	-0.18359	0.042761	-4.29327	1.76E-05	5.85E-05	RBM12B	protein_cc
ENSG0000(	854.5848	0.3052	0.076292	4.000425	6.32E-05	0.000191	MPV17L	protein_cc
ENSG0000(	854.5211	-0.27557	0.04333	-6.35987	2.02E-10	1.46E-09	TMEM86A	protein_cc
ENSG0000(	854.4626	0.176571	0.041352	4.269912	1.96E-05	6.44E-05	GALE	protein_cc
ENSG0000(	853.9672	0.838132	0.119841	6.993694	2.68E-12	2.61E-11	DUSP4	protein_cc
ENSG0000(	853.9304	0.490632	0.067959	7.219519	5.22E-13	5.65E-12	RECQL4	protein_cc
ENSG0000(	853.8359	0.371422	0.03977	9.339348	9.69E-21	3.73E-19	MGME1	protein_cc
ENSG0000(	853.5089	-0.04501	0.086706	-0.51913	0.603667	0.687888	C1orf162	protein_cc
ENSG0000(	853.4733	0.38689	0.089153	4.339626	1.43E-05	4.81E-05	LAMC3	protein_cc
ENSG0000(	853.4018	0.453721	0.103093	4.401097	1.08E-05	3.70E-05	LRRC17	protein_cc
ENSG0000(	853.2367	0.250109	0.057502	4.349589	1.36E-05	4.61E-05	TAX1BP3	protein_cc
ENSG0000(	852.7131	0.447197	0.115067	3.886412	0.000102	0.000297	ENTPD2	protein_cc
ENSG0000(	852.6648	0.082745	0.04463	1.854028	0.063735	0.104432	TRIT1	protein_cc
ENSG0000(	852.6291	-0.43806	0.058226	-7.52332	5.34E-14	6.81E-13	ZFYVE28	protein_cc
ENSG0000(	852.3949	0.313283	0.041671	7.517938	5.56E-14	7.06E-13	THOC6	protein_cc
ENSG0000(	852.3828	0.152692	0.024658	6.192449	5.92E-10	4.00E-09	KRBOX4	protein_cc
ENSG0000(	852.1598	-0.05941	0.046083	-1.28921	0.197326	0.27609	GALT	protein_cc
ENSG0000(	852.1438	0.089153	0.043301	2.058911	0.039503	0.06862	LINC0066?	lincRNA
ENSG0000(	852.0311	0.433508	0.123794	3.501849	0.000462	0.001201	LSP1	protein_cc
ENSG0000(	852.0056	0.483787	0.068604	7.051843	1.77E-12	1.77E-11	NPEPL1	protein_cc
ENSG0000(	851.9861	-0.42276	0.052276	-8.08711	6.11E-16	1.09E-14	PPP2R3A	protein_cc
ENSG0000(	851.8832	-0.24526	0.035932	-6.82568	8.75E-12	7.84E-11	FBXO33	protein_cc
ENSG0000(	851.7485	0.367473	0.037649	9.760486	1.66E-22	8.84E-21	MRGBP	protein_cc
ENSG0000(	851.5297	-0.45041	0.089265	-5.04576	4.52E-07	1.93E-06	RASGEF1C	protein_cc
ENSG0000(	851.4102	0.118085	0.030394	3.885164	0.000102	0.000298	MED11	protein_cc
ENSG0000(	851.3986	0.203707	0.034002	5.991068	2.08E-09	1.29E-08	TMEM223	protein_cc
ENSG0000(	851.3127	-0.09906	0.035622	-2.78088	0.005421	0.011473	RHOBTB1	protein_cc
ENSG0000(	851.291	0.332124	0.070149	4.734578	2.20E-06	8.41E-06	TP53I3	protein_cc
ENSG0000(	851.1064	0.045349	0.034628	1.309597	0.190332	0.267661	TATDN1	protein_cc
ENSG0000(	850.842	0.185871	0.031341	5.930669	3.02E-09	1.82E-08	CETN3	protein_cc
ENSG0000(	850.5903	-0.05356	0.030664	-1.74676	0.08068	0.128177	LIN52	protein_cc
ENSG0000(	850.5843	-1.07284	0.108747	-9.86541	5.88E-23	3.44E-21	ADAM11	protein_cc
ENSG0000(	850.2571	-1.18816	0.103622	-11.4663	1.95E-30	5.04E-28	DOC2B	protein_cc
ENSG0000(	850.0072	-0.16424	0.039694	-4.13758	3.51E-05	0.000111	ADCK1	protein_cc
ENSG0000(	849.9525	0.068103	0.036064	1.888403	0.058972	0.097592	PFDN4	protein_cc
ENSG0000(	849.886	-0.01851	0.028252	-0.65531	0.512266	0.605305	XPA	protein_cc
ENSG0000(	849.6201	-0.34486	0.045601	-7.56249	3.95E-14	5.15E-13	LINC0112(	processed
ENSG0000(	849.4799	-0.28216	0.050798	-5.55459	2.78E-08	1.44E-07	STARD8	protein_cc
ENSG0000(	849.4676	-0.12469	0.036052	-3.45859	0.000543	0.001392	NRBF2	protein_cc
ENSG0000(	849.2592	-0.27899	0.037165	-7.50692	6.05E-14	7.66E-13	VTI1A	protein_cc
ENSG0000(	849.2541	0.371333	0.134385	2.763196	0.005724	0.012043	CD248	protein_cc
ENSG0000(	849.0755	-0.37849	0.138502	-2.73276	0.006281	0.013105	NKX6-2	protein_cc
ENSG0000(	849.0575	-0.15321	0.03635	-4.21483	2.50E-05	8.07E-05	BBS7	protein_cc
ENSG0000(	849.0033	-0.45183	0.10247	-4.40939	1.04E-05	3.57E-05	TMEM132	protein_cc
ENSG0000(	848.9423	0.331486	0.067104	4.939862	7.82E-07	3.21E-06	SNHG17	processed
ENSG0000(	848.9319	0.114327	0.049795	2.295967	0.021678	0.040041	PSMB10	protein_cc
ENSG0000(	848.898	0.18982	0.034996	5.424085	5.83E-08	2.87E-07	ARMCX6	protein_cc
ENSG0000(	848.7294	0.11529	0.187508	0.614857	0.538649	0.629659	GSTM1	protein_cc
ENSG0000(	848.5997	0.09795	0.039166	2.500899	0.012388	0.024222	NAA16	protein_cc
ENSG0000(	848.1789	0.013272	0.061171	0.216971	0.828231	0.874227	NEIL1	protein_cc
ENSG0000(	847.9982	0.095275	0.027346	3.48402	0.000494	0.001275	PPT2	protein_cc
ENSG0000(	847.7641	0.400729	0.076088	5.266632	1.39E-07	6.43E-07	MTMR11	protein_cc
ENSG0000(	847.3952	0.162746	0.043176	3.76935	0.000164	0.000461	ZNF253	protein_cc
ENSG0000(	847.3348	0.10285	0.049257	2.088016	0.036796	0.064436	N6AMT1	protein_cc
ENSG0000(	847.2928	-0.51784	0.076622	-6.75844	1.39E-11	1.21E-10	GPR75	protein_cc

ENSG0000(	847.291	-0.11423	0.041164	-2.77502	0.00552	0.011657	GPR180	protein_cc
ENSG0000(	846.1649	0.162758	0.042017	3.873649	0.000107	0.000311	RABEP2	protein_cc
ENSG0000(	846.0787	-0.18632	0.043567	-4.2766	1.90E-05	6.27E-05	SLC2A11	protein_cc
ENSG0000(	845.5195	0.106929	0.034285	3.118809	0.001816	0.004222	JMJD4	protein_cc
ENSG0000(	845.2918	0.172914	0.03531	4.896979	9.73E-07	3.94E-06	MRPS36	protein_cc
ENSG0000(	845.2117	-0.19963	0.087897	-2.27124	0.023132	0.042466	TGFA	protein_cc
ENSG0000(	845.1849	0.575328	0.09866	5.831408	5.50E-09	3.17E-08	GDPD2	protein_cc
ENSG0000(	845.14	0.0316	0.049679	0.636079	0.524725	0.616688	GSKIP	protein_cc
ENSG0000(	844.7669	-0.65398	0.069014	-9.47607	2.64E-21	1.13E-19	CDYL2	protein_cc
ENSG0000(	844.7078	0.014876	0.06855	0.217006	0.828204	0.874227	FAH	protein_cc
ENSG0000(	844.68	0.184055	0.040394	4.556492	5.20E-06	1.88E-05	DDX20	protein_cc
ENSG0000(	844.3057	-0.5233	0.058043	-9.01574	1.96E-19	6.11E-18	SLC45A4	protein_cc
ENSG0000(	843.6297	0.328134	0.029878	10.9825	4.64E-28	7.07E-26	ZNF26	protein_cc
ENSG0000(	843.3405	-0.24316	0.088666	-2.74238	0.0061	0.012757	LTBP2	protein_cc
ENSG0000(	843.2854	0.045857	0.036279	1.264027	0.20622	0.286613	GNPNAT1	protein_cc
ENSG0000(	842.5916	0.484744	0.180719	2.682309	0.007312	0.015058	COL8A1	protein_cc
ENSG0000(	842.2907	0.076496	0.061966	1.234488	0.217021	0.299097	FHOD1	protein_cc
ENSG0000(	842.2051	-0.22688	0.057576	-3.94048	8.13E-05	0.000242	RP11-210	protein_cc
ENSG0000(	841.9458	-0.76006	0.072709	-10.4535	1.41E-25	1.38E-23	KCNC3	protein_cc
ENSG0000(	841.8142	-0.09611	0.039792	-2.41528	0.015723	0.030031	ENTPD5	protein_cc
ENSG0000(	841.5635	-0.45638	0.082256	-5.54825	2.89E-08	1.49E-07	ZDBF2	protein_cc
ENSG0000(	841.5594	-0.06754	0.042797	-1.57819	0.114523	0.173698	CCDC9	protein_cc
ENSG0000(	840.8133	0.031795	0.031214	1.018622	0.308383	0.401479	CXorf23	protein_cc
ENSG0000(	840.5419	-0.70949	0.093908	-7.55518	4.18E-14	5.43E-13	ADAP1	protein_cc
ENSG0000(	840.008	-0.14828	0.041846	-3.54338	0.000395	0.00104	LYSMD2	protein_cc
ENSG0000(	839.9353	-0.06234	0.096576	-0.64555	0.518572	0.611246	CRISPLD2	protein_cc
ENSG0000(	839.924	0.116335	0.02486	4.679535	2.88E-06	1.08E-05	PDSS2	protein_cc
ENSG0000(	839.8967	0.471361	0.070379	6.697439	2.12E-11	1.79E-10	NSUN5P1	pseudoge
ENSG0000(	839.4846	-0.19212	0.048571	-3.95549	7.64E-05	0.000228	TSNARE1	protein_cc
ENSG0000(	839.1975	-0.09158	0.029516	-3.10267	0.001918	0.004438	POLR3B	protein_cc
ENSG0000(	838.5109	0.174223	0.030094	5.789342	7.07E-09	4.02E-08	ALKBH3	protein_cc
ENSG0000(	838.4893	0.223266	0.037437	5.963749	2.47E-09	1.51E-08	RBFA	protein_cc
ENSG0000(	838.3864	0.080068	0.042447	1.886289	0.059256	0.097979	ZNF415	protein_cc
ENSG0000(	837.8191	-0.29261	0.152725	-1.9159	0.055378	0.092418	VEPH1	protein_cc
ENSG0000(	837.6224	-0.14573	0.157088	-0.92772	0.353555	0.449117	COL6A3	protein_cc
ENSG0000(	837.4567	-0.05934	0.027961	-2.12238	0.033805	0.059712	CENPC	protein_cc
ENSG0000(	837.128	-0.00546	0.090768	-0.06017	0.952017	0.9669	CXorf57	protein_cc
ENSG0000(	836.8399	-0.15297	0.033203	-4.60723	4.08E-06	1.50E-05	SIRT5	protein_cc
ENSG0000(	836.716	-1.17974	0.126508	-9.32542	1.11E-20	4.23E-19	NEURL1	protein_cc
ENSG0000(	836.5671	-0.20144	0.043552	-4.62526	3.74E-06	1.38E-05	ZNF236	protein_cc
ENSG0000(	835.5851	-0.04203	0.02806	-1.4977	0.134211	0.198743	ERCC4	protein_cc
ENSG0000(	835.5174	-0.15826	0.072214	-2.19156	0.028411	0.05108	SLC29A2	protein_cc
ENSG0000(	834.5331	-0.02678	0.047094	-0.56865	0.569595	0.657502	TRAPPC6/	protein_cc
ENSG0000(	834.163	0.047954	0.036667	1.307812	0.190937	0.268411	TRAM2-A:	lincRNA
ENSG0000(	833.3809	0.171306	0.029675	5.772626	7.80E-09	4.40E-08	DHODH	protein_cc
ENSG0000(	833.2159	0.058288	0.02615	2.228956	0.025817	0.046884	PACRGL	protein_cc
ENSG0000(	832.8557	0.656062	0.054982	11.93228	8.03E-33	3.21E-30	HAUS1	protein_cc
ENSG0000(	832.766	-0.58599	0.099556	-5.88605	3.96E-09	2.34E-08	MMP17	protein_cc
ENSG0000(	832.5937	0.11064	0.024933	4.437522	9.10E-06	3.16E-05	ZSWIM1	protein_cc
ENSG0000(	832.593	-0.10803	0.045445	-2.37716	0.017447	0.03293	CCDC28A	protein_cc
ENSG0000(	832.4624	-0.06155	0.039524	-1.55726	0.119408	0.179966	LINC0098	antisense
ENSG0000(	832.0696	0.070176	0.035967	1.951108	0.051044	0.086059	NDUFAF5	protein_cc
ENSG0000(	831.9083	-0.65396	0.098179	-6.66089	2.72E-11	2.26E-10	FAM81A	protein_cc
ENSG0000(	831.5326	-0.21108	0.063599	-3.31889	0.000904	0.00222	REEP6	protein_cc
ENSG0000(	831.4442	0.132076	0.057068	2.314357	0.020648	0.038317	CTD-2368	protein_cc
ENSG0000(	831.2144	0.120906	0.032583	3.710673	0.000207	0.000571	GAR1	protein_cc
ENSG0000(	831.1075	-0.11082	0.061066	-1.81469	0.069572	0.112721	INADL	protein_cc
ENSG0000(	831.1029	0.183879	0.040008	4.596007	4.31E-06	1.58E-05	ZNF100	protein_cc
ENSG0000(	830.4907	0.097634	0.195427	0.499596	0.617359	0.700309	IL8	protein_cc

ENSG0000(	830.4817	0.020572	0.033513	0.613863	0.539306	0.630194	CSNK1G1	protein_cc
ENSG0000(	830.1737	0.278781	0.09854	2.829124	0.004668	0.009999	LAMA1	protein_cc
ENSG0000(	830.1641	-0.06369	0.042201	-1.50931	0.131221	0.194978	ZNF772	protein_cc
ENSG0000(	829.9267	-0.61428	0.216014	-2.84371	0.004459	0.0096	CTD-2339	lincRNA
ENSG0000(	829.643	0.65	0.100514	6.466767	1.00E-10	7.63E-10	NR2E1	protein_cc
ENSG0000(	829.6204	0.175454	0.026861	6.531864	6.50E-11	5.09E-10	MTX1	protein_cc
ENSG0000(	829.3637	0.435913	0.036521	11.93604	7.68E-33	3.14E-30	YAE1D1	protein_cc
ENSG0000(	829.2827	0.166277	0.036242	4.588009	4.47E-06	1.63E-05	TOMM5	protein_cc
ENSG0000(	828.9425	0.160321	0.062219	2.576725	0.009974	0.019929	CHST7	protein_cc
ENSG0000(	828.5266	-0.1845	0.099497	-1.85428	0.063699	0.10439	FBLL1	protein_cc
ENSG0000(	828.4059	-0.19097	0.07187	-2.65716	0.00788	0.01612	PLEKHA7	protein_cc
ENSG0000(	827.905	0.659173	0.061281	10.75654	5.52E-27	7.14E-25	NXT1	protein_cc
ENSG0000(	827.8005	-0.43794	0.138986	-3.15095	0.001627	0.003818	FXYD7	protein_cc
ENSG0000(	827.6726	0.143053	0.117423	1.218272	0.22312	0.306098	TNFSF13B	protein_cc
ENSG0000(	827.6443	0.235305	0.041485	5.672057	1.41E-08	7.65E-08	PLGRKT	protein_cc
ENSG0000(	827.5075	0.236472	0.044273	5.34126	9.23E-08	4.40E-07	EBPL	protein_cc
ENSG0000(	827.4258	-0.01009	0.029715	-0.33964	0.734125	0.798555	RMND1	protein_cc
ENSG0000(	827.1503	-1.04452	0.122506	-8.5263	1.51E-17	3.51E-16	CH25H	protein_cc
ENSG0000(	826.3801	0.01619	0.026129	0.619614	0.535512	0.626709	MED6	protein_cc
ENSG0000(	826.3651	0.102038	0.090265	1.130431	0.258295	0.346268	CD300A	protein_cc
ENSG0000(	826.2736	-0.00222	0.028413	-0.07803	0.937803	0.956596	ZBTB2	protein_cc
ENSG0000(	826.0233	0.138584	0.038691	3.581812	0.000341	0.000909	RASSF8-A	antisense
ENSG0000(	825.7114	0.120636	0.037358	3.229204	0.001241	0.002974	DNAJC30	protein_cc
ENSG0000(	825.2212	0.132268	0.031171	4.243323	2.20E-05	7.19E-05	TVP23B	protein_cc
ENSG0000(	825.2029	-0.38219	0.073542	-5.19692	2.03E-07	9.14E-07	CHN2	protein_cc
ENSG0000(	824.8007	-0.07461	0.053982	-1.38205	0.166956	0.239647	FAM184A	protein_cc
ENSG0000(	824.6665	0.32971	0.041966	7.856571	3.95E-15	6.18E-14	ALG1	protein_cc
ENSG0000(	824.3137	-0.03508	0.032353	-1.08418	0.278286	0.368694	ZFAND1	protein_cc
ENSG0000(	824.1369	0.40188	0.096994	4.143335	3.42E-05	0.000108	ITGA2	protein_cc
ENSG0000(	823.9032	0.020068	0.048055	0.417602	0.676238	0.750577	PARD6A	protein_cc
ENSG0000(	823.5534	-0.81938	0.097359	-8.41607	3.89E-17	8.48E-16	PNCK	protein_cc
ENSG0000(	823.4789	-0.07082	0.039463	-1.79461	0.072716	0.117186	EDRF1	protein_cc
ENSG0000(	823.1103	0.183978	0.042414	4.337691	1.44E-05	4.84E-05	RWDD2B	protein_cc
ENSG0000(	823.0391	0.074721	0.026852	2.782709	0.005391	0.011415	LRRC28	protein_cc
ENSG0000(	823.0245	0.098318	0.139937	0.702585	0.482315	0.577357	MXRA5	protein_cc
ENSG0000(	822.8449	-0.17056	0.041645	-4.09544	4.21E-05	0.000131	RSPH3	protein_cc
ENSG0000(	822.3684	0.207013	0.087624	2.362498	0.018152	0.03413	ST8SIA4	protein_cc
ENSG0000(	822.2384	-0.01267	0.04375	-0.28959	0.772132	0.829492	LRCH4	protein_cc
ENSG0000(	822.1352	-0.15151	0.038395	-3.94623	7.94E-05	0.000236	MLYCD	protein_cc
ENSG0000(	821.8156	0.409701	0.083942	4.880772	1.06E-06	4.25E-06	IRF7	protein_cc
ENSG0000(	821.622	-0.20172	0.060363	-3.34172	0.000833	0.002059	CHML	protein_cc
ENSG0000(	821.2807	0.091542	0.035343	2.590087	0.009595	0.019245	G2E3	protein_cc
ENSG0000(	820.6797	0.167162	0.040555	4.121836	3.76E-05	0.000118	HCCS	protein_cc
ENSG0000(	820.3971	0.005301	0.03859	0.137368	0.89074	0.922179	MNAT1	protein_cc
ENSG0000(	819.9777	-0.03524	0.041438	-0.85038	0.395111	0.49168	TMEM87B	protein_cc
ENSG0000(	819.9412	0.131108	0.035634	3.679334	0.000234	0.000641	PDIK1L	protein_cc
ENSG0000(	819.4814	-0.02085	0.084671	-0.24623	0.805505	0.856667	FAHD2CP	pseudoge
ENSG0000(	819.3999	0.129294	0.032955	3.923326	8.73E-05	0.000258	TRAF2	protein_cc
ENSG0000(	818.8045	0.778485	0.106439	7.313891	2.60E-13	2.95E-12	SLC43A3	protein_cc
ENSG0000(	817.7887	0.715977	0.107688	6.64865	2.96E-11	2.44E-10	DNAH9	protein_cc
ENSG0000(	817.6514	0.091975	0.047594	1.932477	0.053301	0.089368	SOWAHC	protein_cc
ENSG0000(	817.5917	-0.21413	0.028949	-7.39666	1.40E-13	1.66E-12	FAM45A	protein_cc
ENSG0000(	817.5693	0.815279	0.075974	10.73107	7.27E-27	9.18E-25	TCF19	protein_cc
ENSG0000(	817.4309	-1.04765	0.139298	-7.52094	5.44E-14	6.93E-13	KSR2	protein_cc
ENSG0000(	817.1657	-0.08678	0.086907	-0.99853	0.318021	0.411708	EVI2B	protein_cc
ENSG0000(	816.8718	0.681393	0.091257	7.466741	8.22E-14	1.02E-12	EVA1C	protein_cc
ENSG0000(	816.8704	0.228922	0.038916	5.882465	4.04E-09	2.39E-08	C1GALT1(	protein_cc
ENSG0000(	816.4082	-0.34736	0.035196	-9.86925	5.66E-23	3.32E-21	USP35	protein_cc
ENSG0000(	816.3969	-0.16721	0.035198	-4.75056	2.03E-06	7.81E-06	FAM174A	protein_cc

ENSG0000(	816.3935	-0.05885	0.029237	-2.01291	0.044124	0.075635	C6orf211	protein_cc
ENSG0000(	816.3561	0.211698	0.038198	5.542174	2.99E-08	1.54E-07	GTF2E2	protein_cc
ENSG0000(	816.2849	-0.3673	0.084169	-4.36377	1.28E-05	4.34E-05	CACNA1H	protein_cc
ENSG0000(	816.126	0.084396	0.042552	1.983343	0.047329	0.080458	GLI4	protein_cc
ENSG0000(	816.0579	-0.68878	0.099915	-6.89369	5.44E-12	5.02E-11	RAB27B	protein_cc
ENSG0000(	815.6884	0.30559	0.029303	10.42867	1.83E-25	1.74E-23	ZCCHC10	protein_cc
ENSG0000(	815.6453	0.068255	0.030355	2.248539	0.024542	0.044786	SSSCA1	protein_cc
ENSG0000(	815.6328	0.247507	0.041448	5.971547	2.35E-09	1.44E-08	RMI1	protein_cc
ENSG0000(	815.6074	0.107586	0.034508	3.117718	0.001823	0.004236	LYSMD3	protein_cc
ENSG0000(	815.4419	-0.14592	0.040644	-3.59026	0.00033	0.000883	GABPB2	protein_cc
ENSG0000(	815.395	0.233031	0.065498	3.557825	0.000374	0.000989	INTU	protein_cc
ENSG0000(	815.383	0.326035	0.032417	10.05755	8.51E-24	5.97E-22	TBCCD1	protein_cc
ENSG0000(	815.251	-0.15096	0.051933	-2.90676	0.003652	0.008003	SH2D3C	protein_cc
ENSG0000(	815.0121	-0.04584	0.028487	-1.60916	0.107581	0.164528	TRIM39	protein_cc
ENSG0000(	814.7403	0.08438	0.084578	0.997664	0.318442	0.412123	CA8	protein_cc
ENSG0000(	814.2567	0.300045	0.066202	4.532289	5.83E-06	2.09E-05	DRAM1	protein_cc
ENSG0000(	814.0934	0.209324	0.02568	8.151367	3.60E-16	6.68E-15	NUBP1	protein_cc
ENSG0000(	813.647	-0.09649	0.089086	-1.08312	0.278757	0.369126	CD86	protein_cc
ENSG0000(	813.5364	0.085823	0.045152	1.90074	0.057336	0.095245	ZNF354C	protein_cc
ENSG0000(	813.3077	0.108612	0.036244	2.996648	0.00273	0.006131	SS18L2	protein_cc
ENSG0000(	813.3051	-0.04578	0.047348	-0.96681	0.333639	0.428219	TPT1-AS1	antisense
ENSG0000(	813.1128	0.10236	0.037279	2.745767	0.006037	0.012638	ZNF304	protein_cc
ENSG0000(	812.3696	0.295728	0.030973	9.547936	1.32E-21	5.99E-20	FBXL12	protein_cc
ENSG0000(	812.0285	0.05348	0.029244	1.828762	0.067435	0.109682	FAM105B	protein_cc
ENSG0000(	812.0056	-0.29143	0.058876	-4.94991	7.42E-07	3.06E-06	NSUN6	protein_cc
ENSG0000(	811.9834	0.331379	0.04695	7.058192	1.69E-12	1.69E-11	PTRHD1	protein_cc
ENSG0000(	811.7657	0.234207	0.071362	3.281951	0.001031	0.002506	PHF11	protein_cc
ENSG0000(	811.5278	0.066684	0.019467	3.42547	0.000614	0.001557	C2orf42	protein_cc
ENSG0000(	811.0579	0.045008	0.055293	0.813982	0.415655	0.512669	RASGRP2	protein_cc
ENSG0000(	810.9668	0.403438	0.061176	6.594733	4.26E-11	3.44E-10	PCDHB14	protein_cc
ENSG0000(	810.5336	-0.01859	0.046811	-0.3972	0.69122	0.763312	SLC19A2	protein_cc
ENSG0000(	810.2513	-0.12075	0.033341	-3.62163	0.000293	0.000789	TYW3	protein_cc
ENSG0000(	810.0223	-0.26811	0.079839	-3.35817	0.000785	0.00195	PRIMA1	protein_cc
ENSG0000(	809.8623	0.209962	0.032666	6.427587	1.30E-10	9.73E-10	NOP16	protein_cc
ENSG0000(	809.8595	0.224035	0.078626	2.849387	0.00438	0.009445	C9orf89	protein_cc
ENSG0000(	809.8095	-0.17805	0.047275	-3.76637	0.000166	0.000466	TRERF1	protein_cc
ENSG0000(	809.4501	-0.08813	0.103096	-0.85487	0.392624	0.489236	SLC44A5	protein_cc
ENSG0000(	808.9774	0.26627	0.031544	8.441324	3.14E-17	6.94E-16	PDCL3	protein_cc
ENSG0000(	808.9297	0.369028	0.09395	3.927902	8.57E-05	0.000254	HERC5	protein_cc
ENSG0000(	808.5903	-0.37748	0.05518	-6.84088	7.87E-12	7.10E-11	BMP2K	protein_cc
ENSG0000(	808.31	-0.47065	0.079505	-5.91976	3.22E-09	1.94E-08	CRYBG3	protein_cc
ENSG0000(	808.2335	-0.24921	0.032946	-7.56422	3.90E-14	5.09E-13	NKRF	protein_cc
ENSG0000(	808.1926	-0.24144	0.035506	-6.80003	1.05E-11	9.25E-11	PAPD5	protein_cc
ENSG0000(	807.8448	0.113348	0.026147	4.335062	1.46E-05	4.90E-05	MPPE1	protein_cc
ENSG0000(	807.8162	1.243911	0.136672	9.101458	8.91E-20	2.93E-18	UBE2C	protein_cc
ENSG0000(	807.5902	0.178401	0.079729	2.237591	0.025248	0.04594	HLA-H	pseudoge
ENSG0000(	807.502	-0.56756	0.107272	-5.2909	1.22E-07	5.69E-07	CAMSAP3	protein_cc
ENSG0000(	807.1973	0.029513	0.027963	1.055442	0.291223	0.382755	MSH3	protein_cc
ENSG0000(	807.1369	0.819354	0.092021	8.904026	5.39E-19	1.56E-17	CDCA5	protein_cc
ENSG0000(	806.9547	0.200335	0.036523	5.485145	4.13E-08	2.08E-07	SNUPN	protein_cc
ENSG0000(	806.7845	-0.47224	0.096455	-4.89597	9.78E-07	3.96E-06	IGSF9B	protein_cc
ENSG0000(	806.5401	0.14536	0.032646	4.452581	8.48E-06	2.96E-05	SENP1	protein_cc
ENSG0000(	806.4386	-0.08205	0.056098	-1.46255	0.143592	0.210684	RREB1	protein_cc
ENSG0000(	806.287	-0.00371	0.039033	-0.09497	0.924337	0.946713	FBXO46	protein_cc
ENSG0000(	806.2831	0.180493	0.039961	4.516788	6.28E-06	2.24E-05	ZNF138	protein_cc
ENSG0000(	806.2133	-0.00318	0.05513	-0.05769	0.953994	0.968143	WDR27	protein_cc
ENSG0000(	806.1237	-1.43384	0.157945	-9.07807	1.11E-19	3.59E-18	SYNPR	protein_cc
ENSG0000(	805.9106	-0.01117	0.048689	-0.22942	0.818541	0.866943	LPXN	protein_cc
ENSG0000(	805.5041	0.120807	0.025104	4.812237	1.49E-06	5.87E-06	ZW10	protein_cc

ENSG0000(	805.0863	0.824095	0.087614	9.40594	5.16E-21	2.08E-19	GJC1	protein_cc
ENSG0000(	805.0822	0.459644	0.127164	3.614589	0.000301	0.000809	PDLIM1	protein_cc
ENSG0000(	804.7812	-0.16425	0.058865	-2.79019	0.005268	0.011178	MMRN2	protein_cc
ENSG0000(	804.5054	0.0591	0.029706	1.989535	0.046642	0.079437	STYX	protein_cc
ENSG0000(	804.4015	-0.69818	0.125199	-5.57654	2.45E-08	1.28E-07	TRPM3	protein_cc
ENSG0000(	804.3678	0.261197	0.116616	2.239815	0.025103	0.045709	RAMP3	protein_cc
ENSG0000(	804.2298	0.010165	0.025192	0.403482	0.686594	0.759406	ZNF143	protein_cc
ENSG0000(	804.063	0.038904	0.054783	0.710152	0.47761	0.573132	ATXN7L2	protein_cc
ENSG0000(	803.9736	0.473634	0.053312	8.884267	6.43E-19	1.84E-17	GLA	protein_cc
ENSG0000(	803.7141	0.36382	0.031474	11.55941	6.62E-31	1.86E-28	ZNF140	protein_cc
ENSG0000(	803.6917	0.221877	0.037883	5.856882	4.72E-09	2.75E-08	KRTCAP2	protein_cc
ENSG0000(	803.6738	0.026807	0.042865	0.625385	0.531719	0.623258	TRAF3IP2-	antisense
ENSG0000(	803.6318	0.288766	0.028579	10.10412	5.30E-24	3.84E-22	C2orf44	protein_cc
ENSG0000(	803.4286	-1.10612	0.15295	-7.23189	4.76E-13	5.18E-12	CALY	protein_cc
ENSG0000(	803.2666	0.120227	0.049095	2.448855	0.014331	0.027655	CLCN2	protein_cc
ENSG0000(	803.0771	-0.05549	0.034851	-1.59214	0.111352	0.169539	TMEM260	protein_cc
ENSG0000(	802.7927	-0.18701	0.038531	-4.8534	1.21E-06	4.84E-06	TTC33	protein_cc
ENSG0000(	802.6752	1.280241	0.111425	11.48969	1.49E-30	3.89E-28	PTTG1	protein_cc
ENSG0000(	802.2248	0.114247	0.039262	2.909867	0.003616	0.00793	GTPBP3	protein_cc
ENSG0000(	802.2071	-0.02466	0.038075	-0.64754	0.517283	0.609984	ACVR2A	protein_cc
ENSG0000(	801.8834	0.129618	0.049812	2.602143	0.009264	0.018649	NPM3	protein_cc
ENSG0000(	801.8172	0.228818	0.03584	6.384442	1.72E-10	1.26E-09	SDHAF2	protein_cc
ENSG0000(	801.6922	0.13982	0.032995	4.237658	2.26E-05	7.35E-05	CEP44	protein_cc
ENSG0000(	801.6666	-0.23125	0.045004	-5.13855	2.77E-07	1.22E-06	GRK5	protein_cc
ENSG0000(	801.1506	0.222656	0.05437	4.095189	4.22E-05	0.000131	SNHG3	sense_intr
ENSG0000(	799.8729	0.15188	0.042117	3.606142	0.000311	0.000834	C14orf79	protein_cc
ENSG0000(	799.7822	0.613989	0.068175	9.00605	2.14E-19	6.60E-18	NRM	protein_cc
ENSG0000(	799.3133	0.006853	0.056071	0.122227	0.902719	0.930948	ZBTB12	protein_cc
ENSG0000(	798.6967	-0.00195	0.090102	-0.02165	0.982729	0.988111	RNASE6	protein_cc
ENSG0000(	798.5676	0.270327	0.036757	7.354478	1.92E-13	2.22E-12	PAXIP1	protein_cc
ENSG0000(	798.3073	-0.1961	0.134133	-1.46199	0.143743	0.210861	RGR	protein_cc
ENSG0000(	798.1069	-0.49163	0.079983	-6.14665	7.91E-10	5.24E-09	B4GALT6	protein_cc
ENSG0000(	797.865	0.026415	0.038486	0.686341	0.492498	0.586939	ALG10B	protein_cc
ENSG0000(	797.7217	-0.56346	0.106519	-5.2897	1.23E-07	5.72E-07	KIRREL3	protein_cc
ENSG0000(	797.2931	-1.16223	0.106785	-10.8838	1.38E-27	1.95E-25	PDE1A	protein_cc
ENSG0000(	796.5708	0.074155	0.087084	0.851537	0.394471	0.490988	HIST3H2A	protein_cc
ENSG0000(	796.531	0.258711	0.039544	6.542372	6.06E-11	4.77E-10	PTS	protein_cc
ENSG0000(	796.523	-0.42641	0.100573	-4.23978	2.24E-05	7.29E-05	ANK1	protein_cc
ENSG0000(	796.4928	-0.01895	0.037435	-0.50626	0.612676	0.696109	RPGRIP1L	protein_cc
ENSG0000(	796.3212	0.208456	0.041022	5.081624	3.74E-07	1.62E-06	CYB561D2	protein_cc
ENSG0000(	796.0102	-0.21234	0.038497	-5.51591	3.47E-08	1.77E-07	ZNF407	protein_cc
ENSG0000(	795.7885	0.191035	0.035988	5.308252	1.11E-07	5.21E-07	TMEM39B	protein_cc
ENSG0000(	795.7486	-0.20436	0.036066	-5.66617	1.46E-08	7.89E-08	YOD1	protein_cc
ENSG0000(	795.2872	0.904719	0.109133	8.290054	1.13E-16	2.28E-15	INSM1	protein_cc
ENSG0000(	794.9678	0.031653	0.028811	1.09863	0.27193	0.36167	ORAOV1	protein_cc
ENSG0000(	794.551	0.031354	0.054322	0.577191	0.56381	0.652193	CXorf36	protein_cc
ENSG0000(	794.5153	0.076371	0.050131	1.523432	0.127651	0.190545	LINC-PINT	antisense
ENSG0000(	794.3805	-0.64872	0.12158	-5.33576	9.51E-08	4.52E-07	CYP1B1	protein_cc
ENSG0000(	794.102	-0.24651	0.052174	-4.72484	2.30E-06	8.80E-06	SLC2A6	protein_cc
ENSG0000(	794.0319	-0.13783	0.062976	-2.18868	0.02862	0.051423	TNFAIP8L1	protein_cc
ENSG0000(	793.9364	-1.00168	0.136524	-7.33701	2.18E-13	2.51E-12	SRRM4	protein_cc
ENSG0000(	793.834	0.164626	0.030988	5.312644	1.08E-07	5.10E-07	FAM210A	protein_cc
ENSG0000(	793.6403	0.205962	0.038942	5.288953	1.23E-07	5.75E-07	DECR2	protein_cc
ENSG0000(	793.6012	-0.22831	0.037694	-6.05682	1.39E-09	8.86E-09	TMEM180	protein_cc
ENSG0000(	793.4776	0.072382	0.072152	1.003196	0.315766	0.409381	LINC0034	lincRNA
ENSG0000(	793.346	0.23409	0.052142	4.489503	7.14E-06	2.53E-05	ACP6	protein_cc
ENSG0000(	793.2897	-1.02745	0.113344	-9.06486	1.25E-19	4.02E-18	JAKMIP1	protein_cc
ENSG0000(	792.8796	0.090703	0.08614	1.052976	0.292352	0.383903	SNTG1	protein_cc
ENSG0000(	792.7158	0.418491	0.048619	8.607481	7.47E-18	1.83E-16	ZNF432	protein_cc

ENSG0000(	792.5245	0.315601	0.054538	5.786808	7.17E-09	4.07E-08	PHTF1	protein_cc
ENSG0000(	792.0372	0.002202	0.04332	0.050832	0.959459	0.972298	ZNF329	protein_cc
ENSG0000(	790.5656	0.427626	0.080725	5.297305	1.18E-07	5.51E-07	SLC35G2	protein_cc
ENSG0000(	790.2686	0.566334	0.064362	8.799185	1.38E-18	3.76E-17	KNTC1	protein_cc
ENSG0000(	790.2084	-0.88922	0.119041	-7.46983	8.03E-14	9.93E-13	STXBP5L	protein_cc
ENSG0000(	789.6286	-0.14155	0.05915	-2.39308	0.016708	0.031712	FBXL15	protein_cc
ENSG0000(	789.5438	-0.03205	0.031031	-1.03285	0.301673	0.394317	CNNM4	protein_cc
ENSG0000(	789.5265	-0.03955	0.04801	-0.82384	0.410029	0.50687	RRAS2	protein_cc
ENSG0000(	789.4246	0.165067	0.097104	1.6999	0.08915	0.139894	RP3-428L:	antisense
ENSG0000(	789.0904	0.117233	0.032166	3.644595	0.000268	0.000727	C9orf156	protein_cc
ENSG0000(	788.9807	-0.24711	0.091826	-2.69109	0.007122	0.01469	ZDHHC11	protein_cc
ENSG0000(	788.5802	-1.23034	0.16007	-7.68626	1.52E-14	2.12E-13	GAD2	protein_cc
ENSG0000(	787.3596	0.267937	0.031061	8.626194	6.34E-18	1.57E-16	PIGF	protein_cc
ENSG0000(	787.3085	0.027393	0.021837	1.254433	0.209685	0.290643	ZNF747	protein_cc
ENSG0000(	787.2033	0.056561	0.066306	0.853036	0.393639	0.490205	NRARP	protein_cc
ENSG0000(	787.0329	-0.27998	0.108642	-2.57709	0.009964	0.019912	CACNA1G	protein_cc
ENSG0000(	786.8249	-0.23801	0.035379	-6.72745	1.73E-11	1.48E-10	ZBTB46	protein_cc
ENSG0000(	786.8203	0.481157	0.071123	6.765155	1.33E-11	1.16E-10	TRIM5	protein_cc
ENSG0000(	786.8129	-0.05014	0.100586	-0.49845	0.618166	0.701018	LNX1	protein_cc
ENSG0000(	786.7873	1.116311	0.111348	10.02541	1.18E-23	7.99E-22	CDK1	protein_cc
ENSG0000(	786.4628	0.311024	0.039385	7.89704	2.86E-15	4.58E-14	MRPL22	protein_cc
ENSG0000(	785.9125	0.202629	0.029921	6.772121	1.27E-11	1.11E-10	CLN3	protein_cc
ENSG0000(	785.8241	-0.16959	0.062237	-2.72489	0.006432	0.013395	KLF2	protein_cc
ENSG0000(	785.1997	0.380317	0.095476	3.983359	6.79E-05	0.000205	MARVELD	protein_cc
ENSG0000(	785.146	0.125634	0.032931	3.815101	0.000136	0.000388	CIDECP	pseudoge
ENSG0000(	784.8888	0.322447	0.041466	7.77621	7.47E-15	1.12E-13	ZNF789	protein_cc
ENSG0000(	784.8292	-0.20723	0.059834	-3.46351	0.000533	0.001369	SEMA4F	protein_cc
ENSG0000(	784.5311	0.535191	0.045554	11.7486	7.18E-32	2.31E-29	LINC0100:	lincRNA
ENSG0000(	784.3165	0.292042	0.039888	7.321566	2.45E-13	2.79E-12	MUTYH	protein_cc
ENSG0000(	784.3123	0.824232	0.114348	7.208106	5.67E-13	6.12E-12	MT1M	protein_cc
ENSG0000(	784.2258	-0.04617	0.063487	-0.72717	0.467122	0.56301	TUBB3	protein_cc
ENSG0000(	784.2185	-0.1932	0.045979	-4.20182	2.65E-05	8.53E-05	THNSL1	protein_cc
ENSG0000(	783.8299	0.61415	0.118445	5.185119	2.16E-07	9.70E-07	KCNE4	protein_cc
ENSG0000(	783.8179	0.087517	0.027059	3.234341	0.001219	0.002926	ABCB10	protein_cc
ENSG0000(	783.5586	-0.06585	0.048158	-1.36732	0.171526	0.245197	DISP1	protein_cc
ENSG0000(	783.4597	0.054428	0.09626	0.565427	0.571784	0.659266	KLHL26	protein_cc
ENSG0000(	783.2649	0.237711	0.040314	5.896537	3.71E-09	2.21E-08	ZNF45	protein_cc
ENSG0000(	783.1968	-0.29166	0.079867	-3.6518	0.00026	0.000708	C1QTNF3	protein_cc
ENSG0000(	783.0716	-0.53676	0.104013	-5.16046	2.46E-07	1.10E-06	SEMA3C	protein_cc
ENSG0000(	782.5349	-1.12044	0.127147	-8.81215	1.23E-18	3.37E-17	DOC2A	protein_cc
ENSG0000(	782.3197	0.15377	0.033019	4.65704	3.21E-06	1.20E-05	ZNF671	protein_cc
ENSG0000(	782.0587	0.354859	0.052543	6.753751	1.44E-11	1.25E-10	ASL	protein_cc
ENSG0000(	781.7212	0.078639	0.041799	1.88134	0.059926	0.098964	TRIM52-A	antisense
ENSG0000(	781.4934	-0.54826	0.064958	-8.44028	3.17E-17	7.00E-16	CPEB1	protein_cc
ENSG0000(	781.3654	0.089654	0.036031	2.488254	0.012837	0.02501	GCSH	protein_cc
ENSG0000(	781.0762	0.155937	0.027928	5.583576	2.36E-08	1.23E-07	ARMC7	protein_cc
ENSG0000(	780.776	-0.61365	0.098876	-6.20624	5.43E-10	3.68E-09	ME1	protein_cc
ENSG0000(	780.5917	0.395202	0.097101	4.07002	4.70E-05	0.000145	AC244517	protein_cc
ENSG0000(	780.5548	0.313339	0.040154	7.803373	6.03E-15	9.13E-14	TRMT2B	protein_cc
ENSG0000(	780.2121	-0.87522	0.118621	-7.37833	1.60E-13	1.89E-12	HECW1	protein_cc
ENSG0000(	780.2084	-0.82077	0.11531	-7.11798	1.10E-12	1.13E-11	IL1R1	protein_cc
ENSG0000(	779.9971	-0.21612	0.05445	-3.9691	7.21E-05	0.000216	FAM105A	protein_cc
ENSG0000(	779.1953	-1.44411	0.147152	-9.81377	9.82E-23	5.44E-21	ATP2B3	protein_cc
ENSG0000(	779.0968	0.052776	0.025233	2.091538	0.03648	0.06395	ZMYM6	protein_cc
ENSG0000(	779.0626	-0.2913	0.129888	-2.24268	0.024917	0.045405	SIGLEC1	protein_cc
ENSG0000(	778.9593	0.121645	0.047254	2.574292	0.010045	0.020049	N4BP2	protein_cc
ENSG0000(	778.6994	0.211005	0.023753	8.883374	6.49E-19	1.85E-17	C19orf52	protein_cc
ENSG0000(	778.5609	0.138964	0.028992	4.793234	1.64E-06	6.41E-06	SETD6	protein_cc
ENSG0000(	778.3699	0.029331	0.039534	0.741933	0.458128	0.554543	AMDHD2	protein_cc

ENSG0000(	778.3356	0.071334	0.024304	2.935021	0.003335	0.007367	GPATCH2	protein_cc
ENSG0000(	778.2965	-0.38218	0.095424	-4.00501	6.20E-05	0.000188	CCR1	protein_cc
ENSG0000(	778.2054	0.977979	0.209876	4.659807	3.17E-06	1.18E-05	MMP9	protein_cc
ENSG0000(	778.2043	0.238933	0.049725	4.805069	1.55E-06	6.07E-06	IFI27L1	protein_cc
ENSG0000(	778.175	-0.11819	0.056708	-2.08416	0.037146	0.064984	ZNF154	protein_cc
ENSG0000(	778.1524	0.092985	0.036215	2.567606	0.01024	0.020406	GORAB	protein_cc
ENSG0000(	777.8393	-0.02434	0.032914	-0.73953	0.459583	0.555813	RASA2	protein_cc
ENSG0000(	777.7337	-0.13835	0.045449	-3.04406	0.002334	0.005317	CEP85L	protein_cc
ENSG0000(	777.6588	1.196084	0.102538	11.66474	1.93E-31	5.75E-29	METTL21E	protein_cc
ENSG0000(	777.0924	0.316668	0.056015	5.653261	1.57E-08	8.47E-08	C12orf5	protein_cc
ENSG0000(	776.9403	0.251521	0.041366	6.08034	1.20E-09	7.72E-09	TBC1D7	protein_cc
ENSG0000(	776.8045	-0.02075	0.034312	-0.60484	0.545284	0.63577	ANAPC1	protein_cc
ENSG0000(	776.4691	0.241372	0.080591	2.995038	0.002744	0.006159	GMFG	protein_cc
ENSG0000(	776.3303	0.028882	0.030231	0.955364	0.339394	0.434426	ZNF175	protein_cc
ENSG0000(	776.226	0.035673	0.026087	1.36745	0.171484	0.245155	ZC3H10	protein_cc
ENSG0000(	775.9187	-0.0599	0.040998	-1.46093	0.144035	0.211213	LRCH1	protein_cc
ENSG0000(	775.8162	0.176036	0.061529	2.86102	0.004223	0.009143	EME2	protein_cc
ENSG0000(	775.606	-0.17531	0.039066	-4.48758	7.20E-06	2.55E-05	EXOC6	protein_cc
ENSG0000(	775.4883	0.320013	0.039028	8.199485	2.41E-16	4.61E-15	TMED1	protein_cc
ENSG0000(	775.4335	0.164715	0.025701	6.408946	1.47E-10	1.09E-09	LRRC37BP	pseudoge
ENSG0000(	774.9616	-0.33631	0.102134	-3.29287	0.000992	0.002416	TTC9B	protein_cc
ENSG0000(	774.5534	-0.04714	0.030841	-1.5284	0.126414	0.188966	GMEB1	protein_cc
ENSG0000(	774.3226	-0.3845	0.063901	-6.01712	1.78E-09	1.11E-08	PIK3CD	protein_cc
ENSG0000(	774.0242	0.28466	0.069802	4.078083	4.54E-05	0.000141	DDX60L	protein_cc
ENSG0000(	773.9242	0.10337	0.044138	2.34201	0.01918	0.035856	TRIM52	protein_cc
ENSG0000(	773.4582	0.07151	0.041901	1.706643	0.087888	0.138178	AAMDC	protein_cc
ENSG0000(	773.4395	-0.43504	0.094828	-4.58768	4.48E-06	1.64E-05	STEAP2	protein_cc
ENSG0000(	773.2719	-0.6341	0.080954	-7.83286	4.77E-15	7.36E-14	P2RY13	protein_cc
ENSG0000(	773.1661	0.090367	0.031004	2.914686	0.00356	0.00782	UPRT	protein_cc
ENSG0000(	773.125	-0.40197	0.119862	-3.35363	0.000798	0.00198	RPE65	protein_cc
ENSG0000(	772.6717	0.119531	0.035783	3.340477	0.000836	0.002068	IPP	protein_cc
ENSG0000(	772.4233	-0.21979	0.049828	-4.41085	1.03E-05	3.55E-05	FAM78A	protein_cc
ENSG0000(	772.3165	0.994444	0.112414	8.846246	9.05E-19	2.53E-17	KIFC1	protein_cc
ENSG0000(	772.0834	-0.14716	0.045236	-3.25312	0.001141	0.002751	JRKL	protein_cc
ENSG0000(	772.0618	-0.0259	0.085064	-0.30448	0.760765	0.820222	GPC6	protein_cc
ENSG0000(	771.9942	0.488707	0.042315	11.54915	7.46E-31	2.06E-28	TMEM60	protein_cc
ENSG0000(	771.8709	-0.41369	0.098129	-4.21574	2.49E-05	8.05E-05	XKR4	protein_cc
ENSG0000(	771.6664	0.090708	0.029416	3.083679	0.002045	0.00471	PEX3	protein_cc
ENSG0000(	771.2052	0.112613	0.033547	3.356821	0.000788	0.001959	ZNF766	protein_cc
ENSG0000(	771.1457	0.217484	0.026999	8.055354	7.92E-16	1.39E-14	RNMTL1	protein_cc
ENSG0000(	770.878	0.297325	0.03793	7.83885	4.55E-15	7.04E-14	C20orf24	protein_cc
ENSG0000(	770.2258	0.172699	0.069516	2.484318	0.01298	0.025262	AMT	protein_cc
ENSG0000(	770.1097	0.292462	0.046529	6.285524	3.27E-10	2.30E-09	DONSON	protein_cc
ENSG0000(	769.5819	-0.59116	0.068103	-8.68034	3.95E-18	1.00E-16	LRRK1	protein_cc
ENSG0000(	769.5544	-0.00036	0.029497	-0.01234	0.990157	0.993321	C3orf62	protein_cc
ENSG0000(	769.3718	0.047535	0.028443	1.67122	0.094678	0.147344	LTV1	protein_cc
ENSG0000(	769.3231	0.202348	0.028925	6.995543	2.64E-12	2.58E-11	FARS2	protein_cc
ENSG0000(	769.2886	0.43269	0.087682	4.934756	8.03E-07	3.29E-06	OLFML2A	protein_cc
ENSG0000(	769.2731	0.144599	0.037686	3.836983	0.000125	0.000358	CABLES2	protein_cc
ENSG0000(	768.8685	-0.0405	0.03241	-1.24952	0.211477	0.292761	DNAJC24	protein_cc
ENSG0000(	768.7561	0.138583	0.090124	1.53769	0.124124	0.186003	C2orf80	protein_cc
ENSG0000(	768.5241	0.176873	0.035145	5.032691	4.84E-07	2.06E-06	MPV17L2	protein_cc
ENSG0000(	768.1928	-0.24938	0.129479	-1.92602	0.054102	0.090534	COL14A1	protein_cc
ENSG0000(	767.964	0.327339	0.07736	4.231356	2.32E-05	7.54E-05	RP11-304	lincRNA
ENSG0000(	767.5515	-0.00911	0.060057	-0.15162	0.879489	0.91389	GINS3	protein_cc
ENSG0000(	767.4263	-0.04986	0.043747	-1.13979	0.254373	0.341914	TPCN2	protein_cc
ENSG0000(	767.2419	-0.03845	0.038288	-1.00427	0.315247	0.408786	THTPA	protein_cc
ENSG0000(	765.8046	0.099967	0.085282	1.172204	0.241115	0.326612	PLEKHH2	protein_cc
ENSG0000(	765.7908	-0.11404	0.034084	-3.34582	0.00082	0.002031	TRMT11	protein_cc

ENSG0000(	765.4529	0.022483	0.08772	0.256302	0.797717	0.850474	HES4	protein_cc
ENSG0000(	765.2137	-0.82483	0.126525	-6.51906	7.08E-11	5.52E-10	AKR1C1	protein_cc
ENSG0000(	765.007	-0.03498	0.025253	-1.38515	0.166005	0.238524	WHAMM	protein_cc
ENSG0000(	764.9708	0.221866	0.0448	4.952429	7.33E-07	3.03E-06	DCLRE1B	protein_cc
ENSG0000(	764.9562	0.17335	0.027417	6.322664	2.57E-10	1.84E-09	DDX28	protein_cc
ENSG0000(	764.9008	0.851137	0.091047	9.348344	8.90E-21	3.45E-19	PLK1	protein_cc
ENSG0000(	764.8567	0.17572	0.05098	3.44685	0.000567	0.001449	SRD5A3	protein_cc
ENSG0000(	764.687	0.329055	0.061259	5.37158	7.80E-08	3.76E-07	POMZP3	protein_cc
ENSG0000(	764.6692	0.05128	0.082141	0.624286	0.53244	0.623926	IGSF6	protein_cc
ENSG0000(	764.5353	0.069266	0.029698	2.332367	0.019681	0.036688	ALG9	protein_cc
ENSG0000(	764.4924	0.16265	0.036282	4.482984	7.36E-06	2.60E-05	ZNF566	protein_cc
ENSG0000(	764.4023	-0.75475	0.088126	-8.56448	1.09E-17	2.58E-16	SEMA3G	protein_cc
ENSG0000(	764.3368	0.248537	0.039672	6.264765	3.73E-10	2.60E-09	COA6	protein_cc
ENSG0000(	764.1309	0.249084	0.029923	8.324046	8.50E-17	1.75E-15	KATNA1	protein_cc
ENSG0000(	764.0025	0.040963	0.031624	1.295318	0.19521	0.273577	SETMAR	protein_cc
ENSG0000(	763.4649	-0.12624	0.051579	-2.44742	0.014388	0.027757	TRIM66	protein_cc
ENSG0000(	762.3376	0.147813	0.063376	2.3323	0.019685	0.036693	SH2B2	protein_cc
ENSG0000(	762.1056	0.171791	0.069411	2.474997	0.013324	0.025878	CTBS	protein_cc
ENSG0000(	761.7988	0.022978	0.032567	0.705554	0.480466	0.575803	FAM228B	protein_cc
ENSG0000(	761.3523	-0.33688	0.097035	-3.47171	0.000517	0.001331	PCBP3	protein_cc
ENSG0000(	761.3486	0.830937	0.087946	9.448298	3.44E-21	1.45E-19	AVIL	protein_cc
ENSG0000(	761.1463	0.173122	0.085961	2.013948	0.044015	0.075479	COPZ2	protein_cc
ENSG0000(	761.1347	0.16618	0.031039	5.353863	8.61E-08	4.12E-07	LCLAT1	protein_cc
ENSG0000(	761.1022	-0.219	0.06206	-3.52893	0.000417	0.001093	RAB9B	protein_cc
ENSG0000(	760.4421	0.247349	0.032505	7.609616	2.75E-14	3.68E-13	FANCF	protein_cc
ENSG0000(	759.9879	0.333483	0.03782	8.817568	1.17E-18	3.22E-17	ZNF480	protein_cc
ENSG0000(	759.9087	-0.21127	0.087577	-2.4124	0.015848	0.030249	P4HA2	protein_cc
ENSG0000(	759.7923	0.074914	0.029169	2.56826	0.010221	0.020369	C6orf203	protein_cc
ENSG0000(	759.5357	0.092195	0.081851	1.126387	0.260002	0.348182	ECM1	protein_cc
ENSG0000(	759.3691	0.162553	0.07161	2.269974	0.023209	0.042596	PLK3	protein_cc
ENSG0000(	759.1853	0.008851	0.03814	0.232061	0.816491	0.865374	NFATC3	protein_cc
ENSG0000(	758.5835	-0.13914	0.041863	-3.32361	0.000889	0.002186	C16orf87	protein_cc
ENSG0000(	758.3476	0.31489	0.03317	9.493318	2.24E-21	9.72E-20	MTERFD1	protein_cc
ENSG0000(	758.0121	-1.11817	0.131246	-8.51964	1.60E-17	3.70E-16	LCNL1	protein_cc
ENSG0000(	757.6902	0.114949	0.047321	2.429125	0.015135	0.029017	AFG3L1P	pseudoge
ENSG0000(	757.2965	0.001922	0.057144	0.033633	0.97317	0.981188	MFAP3	protein_cc
ENSG0000(	757.2212	0.419092	0.060775	6.895832	5.36E-12	4.95E-11	CDC7	protein_cc
ENSG0000(	757.2142	0.455681	0.057777	7.886853	3.10E-15	4.94E-14	BARD1	protein_cc
ENSG0000(	756.8311	0.143687	0.034652	4.146545	3.38E-05	0.000107	NUDCD1	protein_cc
ENSG0000(	756.6107	0.126039	0.046205	2.72784	0.006375	0.013283	RTTN	protein_cc
ENSG0000(	756.2409	-0.13615	0.071799	-1.89631	0.057918	0.096088	IRF5	protein_cc
ENSG0000(	756.1669	1.084758	0.092329	11.74886	7.16E-32	2.31E-29	CKS2	protein_cc
ENSG0000(	755.972	0.072479	0.034277	2.114484	0.034474	0.060794	ZNF75D	protein_cc
ENSG0000(	755.866	-1.04902	0.130121	-8.06188	7.51E-16	1.32E-14	LYVE1	protein_cc
ENSG0000(	755.8505	-0.1543	0.051312	-3.00704	0.002638	0.005949	GPR137C	protein_cc
ENSG0000(	755.1107	0.028448	0.028601	0.994635	0.319914	0.413681	DUS2	protein_cc
ENSG0000(	754.9712	0.049457	0.066629	0.742279	0.457918	0.554402	SCARF2	protein_cc
ENSG0000(	754.7374	-0.87858	0.087568	-10.0331	1.09E-23	7.46E-22	TMEM56	protein_cc
ENSG0000(	754.3538	-0.04409	0.037487	-1.17621	0.23951	0.324855	TRAF6	protein_cc
ENSG0000(	753.9952	-0.10306	0.039856	-2.58572	0.009718	0.019466	CARF	protein_cc
ENSG0000(	753.991	0.353594	0.080911	4.370137	1.24E-05	4.22E-05	PRR24	protein_cc
ENSG0000(	753.971	0.315205	0.06402	4.923509	8.50E-07	3.47E-06	MGST2	protein_cc
ENSG0000(	753.5566	0.215137	0.02568	8.377644	5.40E-17	1.15E-15	SEC22A	protein_cc
ENSG0000(	753.5512	1.29932	0.156898	8.281284	1.22E-16	2.44E-15	PLA2G5	protein_cc
ENSG0000(	753.5211	-0.05497	0.036996	-1.48585	0.137318	0.202681	PIGV	protein_cc
ENSG0000(	752.3038	-0.02087	0.029042	-0.71874	0.472299	0.568184	BLOC1S3	protein_cc
ENSG0000(	752.0118	0.30378	0.065996	4.602992	4.16E-06	1.53E-05	SHC4	protein_cc
ENSG0000(	751.7366	-0.38727	0.07594	-5.09964	3.40E-07	1.48E-06	FGD2	protein_cc
ENSG0000(	751.2565	-0.14439	0.073256	-1.97098	0.048726	0.082584	CPPED1	protein_cc

ENSG0000(	750.8881	-0.0351	0.056312	-0.62327	0.533108	0.624549	THEM4	protein_cc
ENSG0000(	750.7715	0.672515	0.101679	6.61409	3.74E-11	3.04E-10	TEAD2	protein_cc
ENSG0000(	750.7377	-0.21503	0.084232	-2.55288	0.010684	0.021204	TNFAIP3	protein_cc
ENSG0000(	750.6707	0.320902	0.055897	5.740972	9.41E-09	5.24E-08	LINC0011(	lincRNA
ENSG0000(	750.4523	-1.30126	0.10848	-11.9954	3.76E-33	1.70E-30	IPCEF1	protein_cc
ENSG0000(	750.4016	0.041984	0.02297	1.827778	0.067583	0.109892	METTL21A	protein_cc
ENSG0000(	750.178	-0.63572	0.156429	-4.06398	4.82E-05	0.000149	SSTR1	protein_cc
ENSG0000(	750.0773	0.192668	0.038854	4.958767	7.09E-07	2.94E-06	NDUFAF2	protein_cc
ENSG0000(	750.0494	-0.19878	0.136988	-1.45104	0.146769	0.214695	GSTM5	protein_cc
ENSG0000(	749.9489	0.320701	0.039464	8.126445	4.42E-16	8.11E-15	ZBTB8OS	protein_cc
ENSG0000(	749.673	-0.74197	0.120296	-6.16784	6.92E-10	4.63E-09	SFRP1	protein_cc
ENSG0000(	749.5819	0.064079	0.028318	2.262839	0.023646	0.043301	LARP1B	protein_cc
ENSG0000(	749.5221	-0.36591	0.063392	-5.7722	7.82E-09	4.41E-08	C9orf172	protein_cc
ENSG0000(	749.4563	0.24572	0.043679	5.625576	1.85E-08	9.84E-08	ZSCAN16-	antisense
ENSG0000(	748.4386	0.236982	0.043343	5.467629	4.56E-08	2.28E-07	ZNF419	protein_cc
ENSG0000(	748.3108	0.257161	0.04165	6.174354	6.64E-10	4.46E-09	FIGNL1	protein_cc
ENSG0000(	747.9353	-0.11611	0.040888	-2.83979	0.004514	0.0097	CD2AP	protein_cc
ENSG0000(	747.7425	-0.90498	0.101014	-8.95893	3.28E-19	9.82E-18	SORCS1	protein_cc
ENSG0000(	747.6787	0.205178	0.110759	1.852472	0.063958	0.104748	AMH	protein_cc
ENSG0000(	747.2185	-0.73404	0.093441	-7.85565	3.98E-15	6.22E-14	KIAA1217	protein_cc
ENSG0000(	746.998	0.120835	0.069089	1.748984	0.080294	0.127648	ACADS	protein_cc
ENSG0000(	746.9133	-0.51437	0.081684	-6.29707	3.03E-10	2.14E-09	RIMKLA	protein_cc
ENSG0000(	746.8706	0.110426	0.080483	1.372031	0.170054	0.24344	GRAMD1(	protein_cc
ENSG0000(	746.8551	-0.43363	0.084266	-5.14602	2.66E-07	1.18E-06	B3GALT2	protein_cc
ENSG0000(	746.7587	-0.24926	0.068092	-3.66068	0.000252	0.000686	LSR	protein_cc
ENSG0000(	746.6418	0.114054	0.041743	2.73227	0.00629	0.013122	FAM76A	protein_cc
ENSG0000(	746.4112	0.097632	0.071917	1.357568	0.174601	0.248926	CDC42EP3	protein_cc
ENSG0000(	746.076	-0.54612	0.076411	-7.14718	8.86E-13	9.29E-12	SNED1	protein_cc
ENSG0000(	745.927	-0.05065	0.075628	-0.66969	0.503058	0.596946	ENOSF1	protein_cc
ENSG0000(	745.7676	-1.56088	0.147365	-10.5919	3.25E-26	3.60E-24	HS6ST3	protein_cc
ENSG0000(	745.5111	-1.03365	0.113478	-9.10885	8.33E-20	2.75E-18	NRIP3	protein_cc
ENSG0000(	745.5058	0.106008	0.028282	3.748213	0.000178	0.000498	STK19	protein_cc
ENSG0000(	745.4831	0.766356	0.110144	6.957743	3.46E-12	3.31E-11	CITED1	protein_cc
ENSG0000(	745.2302	0.260388	0.061477	4.235507	2.28E-05	7.42E-05	REEP4	protein_cc
ENSG0000(	745.0347	0.183782	0.061879	2.970034	0.002978	0.006641	SUSD1	protein_cc
ENSG0000(	744.9351	0.333182	0.078586	4.239693	2.24E-05	7.29E-05	PPP1R3B	protein_cc
ENSG0000(	744.662	-0.31628	0.040985	-7.71695	1.19E-14	1.71E-13	PPIP5K1	protein_cc
ENSG0000(	744.4582	0.210325	0.12389	1.69768	0.089568	0.140454	PLAUR	protein_cc
ENSG0000(	743.8823	0.001425	0.040362	0.035298	0.971842	0.980497	RCCD1	protein_cc
ENSG0000(	743.678	-0.56506	0.080378	-7.02995	2.07E-12	2.05E-11	LMO7	protein_cc
ENSG0000(	743.5172	-0.42258	0.05913	-7.14667	8.89E-13	9.32E-12	SEMA4G	protein_cc
ENSG0000(	743.4753	0.055245	0.056872	0.971402	0.331348	0.425861	NBEAL2	protein_cc
ENSG0000(	743.1528	0.048146	0.033455	1.439112	0.150119	0.218964	ZNF10	protein_cc
ENSG0000(	743.1506	0.137832	0.040067	3.440055	0.000582	0.001483	RAD9A	protein_cc
ENSG0000(	742.9899	0.25805	0.092895	2.777869	0.005472	0.011566	PRPS2	protein_cc
ENSG0000(	742.9201	-0.00631	0.086701	-0.07275	0.942002	0.959454	EPHX2	protein_cc
ENSG0000(	742.6528	-0.24909	0.0467	-5.33369	9.62E-08	4.57E-07	SLC45A1	protein_cc
ENSG0000(	742.3574	0.221243	0.036305	6.093958	1.10E-09	7.14E-09	GSTCD	protein_cc
ENSG0000(	742.3343	-0.28751	0.060861	-4.72398	2.31E-06	8.83E-06	SLC25A53	protein_cc
ENSG0000(	742.2389	0.836048	0.087901	9.511232	1.88E-21	8.33E-20	AFAP1L1	protein_cc
ENSG0000(	742.0169	0.24828	0.039652	6.261545	3.81E-10	2.65E-09	CAAP1	protein_cc
ENSG0000(	741.8865	-0.21317	0.056991	-3.74034	0.000184	0.000513	RAB23	protein_cc
ENSG0000(	741.4488	0.141223	0.051128	2.762168	0.005742	0.012077	ZNF579	protein_cc
ENSG0000(	741.2239	-0.30893	0.053561	-5.76774	8.03E-09	4.52E-08	TBC1D8	protein_cc
ENSG0000(	741.1827	0.180374	0.042197	4.274564	1.92E-05	6.32E-05	KPTN	protein_cc
ENSG0000(	740.8013	-0.72714	0.117605	-6.18291	6.29E-10	4.23E-09	CDH22	protein_cc
ENSG0000(	740.3391	0.144892	0.031325	4.62544	3.74E-06	1.38E-05	DNAAF2	protein_cc
ENSG0000(	739.9761	0.596931	0.096572	6.181172	6.36E-10	4.28E-09	HIST1H1C	protein_cc
ENSG0000(	739.2531	0.115321	0.023163	4.978721	6.40E-07	2.67E-06	PALB2	protein_cc

ENSG0000(	739.2144	0.246839	0.026291	9.388554	6.08E-21	2.42E-19	TTC27	protein_cc
ENSG0000(	738.7974	-0.38703	0.055514	-6.97188	3.13E-12	3.01E-11	JAG2	protein_cc
ENSG0000(	738.7606	-0.63612	0.06732	-9.44918	3.41E-21	1.44E-19	MICU3	protein_cc
ENSG0000(	738.243	0.106317	0.038765	2.74257	0.006096	0.012751	SERAC1	protein_cc
ENSG0000(	738.0956	0.155374	0.037534	4.139552	3.48E-05	0.00011	QTRTD1	protein_cc
ENSG0000(	738.0225	0.070785	0.028232	2.507296	0.012166	0.023831	CCDC51	protein_cc
ENSG0000(	737.8063	0.132998	0.028781	4.621117	3.82E-06	1.41E-05	OSGEPL1	protein_cc
ENSG0000(	737.7505	-0.0745	0.068684	-1.08463	0.278087	0.368453	ADAMTS1	protein_cc
ENSG0000(	737.7312	0.186184	0.027651	6.733382	1.66E-11	1.42E-10	TRMT61B	protein_cc
ENSG0000(	737.6851	0.333676	0.097912	3.407918	0.000655	0.001652	CD109	protein_cc
ENSG0000(	737.5606	-0.09101	0.077491	-1.17449	0.240199	0.325522	LRP2BP	protein_cc
ENSG0000(	737.3186	0.138992	0.031866	4.361825	1.29E-05	4.37E-05	TOR2A	protein_cc
ENSG0000(	737.2689	0.161207	0.025188	6.40024	1.55E-10	1.14E-09	ZNF394	protein_cc
ENSG0000(	737.1801	-0.10979	0.04723	-2.32455	0.020096	0.037387	TRIM16L	protein_cc
ENSG0000(	737.1578	-0.02678	0.06632	-0.40376	0.686386	0.759217	TMEM169	protein_cc
ENSG0000(	737.1088	-0.26848	0.121351	-2.21244	0.026936	0.048682	COL12A1	protein_cc
ENSG0000(	737.0311	0.305584	0.045363	6.736352	1.62E-11	1.40E-10	XRCC3	protein_cc
ENSG0000(	736.9884	-0.15497	0.03599	-4.30581	1.66E-05	5.54E-05	SLC46A1	protein_cc
ENSG0000(	735.8816	-0.63625	0.08111	-7.84425	4.36E-15	6.75E-14	CACNB2	protein_cc
ENSG0000(	735.6092	-0.06176	0.044488	-1.38827	0.165056	0.23733	ZNF667	protein_cc
ENSG0000(	735.5212	0.135651	0.038298	3.542015	0.000397	0.001045	FLVCR1	protein_cc
ENSG0000(	735.519	0.118543	0.028485	4.161619	3.16E-05	0.000101	SLC35B3	protein_cc
ENSG0000(	735.4029	0.237256	0.037007	6.411048	1.45E-10	1.07E-09	ZNF420	protein_cc
ENSG0000(	735.3872	0.35722	0.063011	5.669131	1.44E-08	7.77E-08	TMEM51	protein_cc
ENSG0000(	735.2524	0.055799	0.029798	1.872535	0.061133	0.100678	EIF3J-AS1	lincRNA
ENSG0000(	735.196	0.19758	0.03489	5.662989	1.49E-08	8.03E-08	PAFAH2	protein_cc
ENSG0000(	735.061	0.024844	0.118916	0.208917	0.834513	0.879488	ADAMTS1	protein_cc
ENSG0000(	734.9877	0.112304	0.027957	4.016982	5.89E-05	0.000179	EXOG	protein_cc
ENSG0000(	734.9712	0.309118	0.11857	2.60705	0.009133	0.018401	FBLIM1	protein_cc
ENSG0000(	734.6378	-0.35792	0.070445	-5.08086	3.76E-07	1.62E-06	RP11-999	sense_ove
ENSG0000(	734.2577	-0.72966	0.132148	-5.52157	3.36E-08	1.72E-07	ST18	protein_cc
ENSG0000(	734.1692	-0.22764	0.05163	-4.40899	1.04E-05	3.58E-05	HOMER2	protein_cc
ENSG0000(	734.1449	0.079929	0.03248	2.460839	0.013861	0.026819	ZNF202	protein_cc
ENSG0000(	734.0016	0.043757	0.036936	1.184651	0.236155	0.321004	ZNF184	protein_cc
ENSG0000(	733.8617	-0.19265	0.064071	-3.00675	0.002641	0.005954	CCDC120	protein_cc
ENSG0000(	733.6057	-0.09757	0.060504	-1.6127	0.10681	0.163513	WNT3	protein_cc
ENSG0000(	733.4082	0.801804	0.08683	9.234178	2.60E-20	9.30E-19	CDCA7	protein_cc
ENSG0000(	733.342	0.240037	0.026362	9.105385	8.60E-20	2.83E-18	ZNF136	protein_cc
ENSG0000(	733.3022	0.09895	0.047884	2.066434	0.038787	0.067514	CEP85	protein_cc
ENSG0000(	732.9859	-0.04443	0.040346	-1.10118	0.270816	0.360371	ATAD2B	protein_cc
ENSG0000(	732.376	0.594179	0.072578	8.186749	2.68E-16	5.09E-15	ACN9	protein_cc
ENSG0000(	731.8531	0.060887	0.041195	1.478017	0.139403	0.205359	CBX8	protein_cc
ENSG0000(	731.7256	-0.59792	0.056553	-10.5728	3.98E-26	4.35E-24	RP11-57H	sense_ove
ENSG0000(	731.4959	-0.42255	0.08624	-4.89974	9.60E-07	3.89E-06	KIAA1377	protein_cc
ENSG0000(	731.4796	0.267309	0.046462	5.753274	8.75E-09	4.90E-08	FBXL6	protein_cc
ENSG0000(	731.211	-0.04045	0.066503	-0.60823	0.543036	0.633742	NUDT10	protein_cc
ENSG0000(	731.2061	-0.03464	0.074489	-0.46503	0.641912	0.721749	MKRN3	protein_cc
ENSG0000(	730.7327	0.163512	0.044054	3.711649	0.000206	0.000569	B9D1	protein_cc
ENSG0000(	730.7026	-0.01195	0.038191	-0.31284	0.754404	0.81488	ZBTB39	protein_cc
ENSG0000(	730.557	0.112747	0.043307	2.603405	0.00923	0.018585	RP1-39G2	antisense
ENSG0000(	730.4412	-0.01249	0.030116	-0.41461	0.678425	0.752557	ZNF41	protein_cc
ENSG0000(	730.0247	-0.3257	0.048626	-6.69814	2.11E-11	1.78E-10	LCOR	protein_cc
ENSG0000(	729.8756	0.131389	0.083061	1.581835	0.113687	0.172603	LPPR5	protein_cc
ENSG0000(	729.7157	-0.07045	0.068535	-1.02795	0.303974	0.396795	ALDH3B1	protein_cc
ENSG0000(	729.2869	-0.46617	0.087956	-5.30011	1.16E-07	5.43E-07	C1orf233	protein_cc
ENSG0000(	729.1599	0.209943	0.039005	5.382466	7.35E-08	3.55E-07	C1D	protein_cc
ENSG0000(	728.8844	0.086335	0.032045	2.694232	0.007055	0.014565	MCAT	protein_cc
ENSG0000(	728.8368	0.087179	0.045401	1.920218	0.05483	0.091605	RAD52	protein_cc
ENSG0000(	728.7703	-0.86924	0.098444	-8.82978	1.05E-18	2.90E-17	EXTL1	protein_cc

ENSG0000(	727.9636	-0.34965	0.100029	-3.49546	0.000473	0.001227	KCNIP4	protein_cc
ENSG0000(	727.6936	-0.22395	0.041615	-5.38163	7.38E-08	3.57E-07	ZNF438	protein_cc
ENSG0000(	727.5857	-0.19334	0.105084	-1.83989	0.065785	0.107308	AMZ1	protein_cc
ENSG0000(	727.5842	-0.41113	0.053802	-7.6415	2.15E-14	2.93E-13	CLTCL1	protein_cc
ENSG0000(	727.5762	0.0301	0.032371	0.929825	0.352462	0.447988	C19orf47	protein_cc
ENSG0000(	727.2215	-0.13009	0.117373	-1.10837	0.267701	0.356773	NOG	protein_cc
ENSG0000(	726.3602	0.052184	0.112188	0.465143	0.641829	0.721695	COL8A2	protein_cc
ENSG0000(	726.0269	-0.07898	0.136287	-0.57948	0.562267	0.651002	PIRT	protein_cc
ENSG0000(	725.7078	-0.27653	0.045096	-6.13195	8.68E-10	5.72E-09	TCP11L1	protein_cc
ENSG0000(	725.688	1.344028	0.129317	10.39327	2.66E-25	2.45E-23	FAM64A	protein_cc
ENSG0000(	725.5953	-0.01362	0.051925	-0.26224	0.793138	0.846733	DPCD	protein_cc
ENSG0000(	725.5354	0.327154	0.029649	11.03439	2.61E-28	4.29E-26	FAM200A	protein_cc
ENSG0000(	725.3845	0.069485	0.033535	2.072007	0.038265	0.066737	ZFP28	protein_cc
ENSG0000(	725.0748	0.143345	0.0461	3.109454	0.001874	0.004346	ARMC9	protein_cc
ENSG0000(	725.0237	0.055233	0.039411	1.401463	0.161076	0.232375	THAP2	protein_cc
ENSG0000(	724.8238	0.012998	0.034913	0.372303	0.709667	0.77854	CCDC66	protein_cc
ENSG0000(	724.8099	0.071155	0.041704	1.706212	0.087969	0.138299	FAIM	protein_cc
ENSG0000(	724.5929	0.567491	0.076557	7.412617	1.24E-13	1.48E-12	SPSB4	protein_cc
ENSG0000(	724.5154	0.111788	0.083014	1.346619	0.178103	0.25306	HEYL	protein_cc
ENSG0000(	724.3685	-0.19809	0.070841	-2.79633	0.005169	0.010983	MYO7A	protein_cc
ENSG0000(	724.3506	-0.26891	0.05227	-5.14472	2.68E-07	1.19E-06	IPMK	protein_cc
ENSG0000(	724.1507	0.961481	0.126295	7.612954	2.68E-14	3.59E-13	DIRAS3	protein_cc
ENSG0000(	723.803	-0.34638	0.159325	-2.17403	0.029703	0.053176	RP5-1119.	sense_ove
ENSG0000(	723.754	1.261662	0.128136	9.846257	7.11E-23	4.08E-21	CDC20	protein_cc
ENSG0000(	722.5326	0.414981	0.076296	5.439087	5.36E-08	2.65E-07	PDK1	protein_cc
ENSG0000(	722.4159	0.031056	0.042415	0.732203	0.464045	0.560106	C11orf1	protein_cc
ENSG0000(	722.3306	-0.0225	0.052127	-0.43172	0.665946	0.742178	TFAP4	protein_cc
ENSG0000(	722.2799	-0.00955	0.040046	-0.2385	0.811497	0.861479	TMEM120	protein_cc
ENSG0000(	721.7495	-0.08757	0.037532	-2.33323	0.019636	0.036611	SLC46A3	protein_cc
ENSG0000(	721.3713	0.200041	0.041662	4.801537	1.57E-06	6.17E-06	MEF2BNB	protein_cc
ENSG0000(	721.2667	0.050772	0.061445	0.826309	0.408629	0.505442	ZNF714	protein_cc
ENSG0000(	721.2003	-0.50846	0.076086	-6.68264	2.35E-11	1.97E-10	SVIL	protein_cc
ENSG0000(	720.7117	-0.40173	0.046994	-8.54852	1.25E-17	2.94E-16	C10orf118	protein_cc
ENSG0000(	720.5616	-0.02197	0.075779	-0.28997	0.771837	0.829267	CAMK2N2	protein_cc
ENSG0000(	720.3066	0.378977	0.152355	2.487467	0.012866	0.025062	ADAM12	protein_cc
ENSG0000(	720.2498	0.160246	0.077498	2.067736	0.038665	0.067327	RAB20	protein_cc
ENSG0000(	720.2307	0.188556	0.038363	4.91499	8.88E-07	3.62E-06	ZNF548	protein_cc
ENSG0000(	719.8826	0.315983	0.074098	4.264412	2.00E-05	6.59E-05	TRAF1	protein_cc
ENSG0000(	719.831	-0.11698	0.089956	-1.30037	0.193476	0.271436	SAMSN1	protein_cc
ENSG0000(	719.7306	0.076335	0.155685	0.490317	0.62391	0.706239	KLRC2	protein_cc
ENSG0000(	719.7221	0.227406	0.035041	6.489651	8.60E-11	6.63E-10	SUV39H1	protein_cc
ENSG0000(	719.497	0.001616	0.039416	0.040994	0.967301	0.977422	AADAT	protein_cc
ENSG0000(	719.1637	-0.45379	0.053684	-8.45296	2.84E-17	6.35E-16	PRRT3	protein_cc
ENSG0000(	719.1485	-0.47889	0.122128	-3.92124	8.81E-05	0.00026	AHNAK2	protein_cc
ENSG0000(	718.8918	0.245795	0.04835	5.083695	3.70E-07	1.60E-06	VPS37D	protein_cc
ENSG0000(	718.8484	-0.10841	0.043307	-2.50337	0.012302	0.024068	NAGPA	protein_cc
ENSG0000(	718.6818	-0.15622	0.033067	-4.72437	2.31E-06	8.82E-06	ZBTB34	protein_cc
ENSG0000(	718.5121	0.543287	0.083536	6.503605	7.84E-11	6.07E-10	KIF11	protein_cc
ENSG0000(	718.4931	-0.09654	0.048737	-1.98074	0.04762	0.080893	CAMK1	protein_cc
ENSG0000(	717.9015	-0.86923	0.116831	-7.44005	1.01E-13	1.22E-12	GFRA2	protein_cc
ENSG0000(	717.8419	0.14021	0.04757	2.947468	0.003204	0.007098	ATP6V0E2	antisense
ENSG0000(	717.7779	0.187934	0.121471	1.547148	0.121828	0.183015	PCDHB2	protein_cc
ENSG0000(	717.7693	0.126968	0.03057	4.153314	3.28E-05	0.000104	MRPL46	protein_cc
ENSG0000(	717.5457	0.282665	0.038297	7.380786	1.57E-13	1.85E-12	HUS1	protein_cc
ENSG0000(	717.082	-0.26569	0.065764	-4.04013	5.34E-05	0.000164	POU2F2	protein_cc
ENSG0000(	717.0404	-0.03415	0.031963	-1.06848	0.285306	0.376164	C15orf57	protein_cc
ENSG0000(	716.8915	-0.02185	0.032403	-0.6743	0.500122	0.594062	ZNF720	protein_cc
ENSG0000(	716.6028	0.115061	0.072379	1.589704	0.111901	0.170249	LAYN	protein_cc
ENSG0000(	716.5861	0.389165	0.050237	7.746599	9.44E-15	1.38E-13	KNSTRN	protein_cc

ENSG0000(	716.5019	0.393903	0.04845	8.130107	4.29E-16	7.88E-15	ZIK1	protein_cc
ENSG0000(	716.408	-0.84421	0.134369	-6.28275	3.33E-10	2.34E-09	ARHGAP3	protein_cc
ENSG0000(	716.3785	0.105405	0.042048	2.506747	0.012185	0.023866	BEND5	protein_cc
ENSG0000(	716.2645	0.120487	0.030807	3.911031	9.19E-05	0.00027	DNAJC17	protein_cc
ENSG0000(	716.2568	0.10366	0.034196	3.031316	0.002435	0.005533	RNF113A	protein_cc
ENSG0000(	715.8132	-0.11094	0.047061	-2.35742	0.018403	0.034545	BDNF-AS	antisense
ENSG0000(	715.7742	-0.63265	0.07157	-8.83958	9.61E-19	2.67E-17	FBXL2	protein_cc
ENSG0000(	715.7018	0.061828	0.043773	1.412462	0.157814	0.22831	ABCB6	protein_cc
ENSG0000(	715.6534	0.220255	0.02827	7.791227	6.64E-15	1.00E-13	CDK7	protein_cc
ENSG0000(	715.636	0.102363	0.041395	2.472803	0.013406	0.026017	WRN	protein_cc
ENSG0000(	715.4473	0.24412	0.040512	6.02583	1.68E-09	1.06E-08	TDG	protein_cc
ENSG0000(	715.229	-0.46282	0.068981	-6.70939	1.95E-11	1.66E-10	FCHO1	protein_cc
ENSG0000(	715.2178	-0.19417	0.075898	-2.55827	0.010519	0.020916	NFATC2	protein_cc
ENSG0000(	715.0683	0.129769	0.095025	1.365625	0.172057	0.24581	COL27A1	protein_cc
ENSG0000(	714.9191	-0.05568	0.032653	-1.70514	0.088168	0.138559	EXOSC1	protein_cc
ENSG0000(	714.6276	-0.20356	0.03723	-5.46763	4.56E-08	2.28E-07	SNX25	protein_cc
ENSG0000(	714.5753	-0.14781	0.061743	-2.39396	0.016667	0.031647	L3MBTL1	protein_cc
ENSG0000(	714.4648	-0.19334	0.034174	-5.65741	1.54E-08	8.28E-08	AP5B1	protein_cc
ENSG0000(	714.4071	-0.16004	0.089277	-1.79261	0.073036	0.117642	PRDM16	protein_cc
ENSG0000(	714.3079	0.062872	0.03517	1.78765	0.073832	0.118716	SUZ12P	pseudoge
ENSG0000(	714.2958	0.188114	0.049893	3.770353	0.000163	0.000459	ZNF738	protein_cc
ENSG0000(	714.1874	0.040331	0.03542	1.138649	0.25485	0.342434	WDR25	protein_cc
ENSG0000(	713.9984	0.610085	0.059756	10.20955	1.80E-24	1.42E-22	DSN1	protein_cc
ENSG0000(	713.8876	0.118626	0.095877	1.23728	0.215983	0.298017	HSF4	protein_cc
ENSG0000(	713.5675	-0.19912	0.101284	-1.9659	0.04931	0.08347	ANO5	protein_cc
ENSG0000(	713.5606	-0.12363	0.053091	-2.32855	0.019883	0.037028	HERC2P9	pseudoge
ENSG0000(	712.8079	0.253123	0.055042	4.598725	4.25E-06	1.56E-05	DCBLD1	protein_cc
ENSG0000(	712.6897	0.397669	0.044004	9.037033	1.61E-19	5.09E-18	PXMP2	protein_cc
ENSG0000(	712.441	0.116565	0.044342	2.628792	0.008569	0.017385	SNHG16	processed
ENSG0000(	712.2924	0.190686	0.033203	5.743091	9.30E-09	5.18E-08	ZNF568	protein_cc
ENSG0000(	712.2481	0.026351	0.036989	0.712406	0.476214	0.571924	ENOX2	protein_cc
ENSG0000(	711.9548	0.322486	0.033808	9.538716	1.45E-21	6.51E-20	NUP35	protein_cc
ENSG0000(	711.4766	0.674815	0.085924	7.853596	4.04E-15	6.32E-14	GSAP	protein_cc
ENSG0000(	711.4057	-0.02092	0.032352	-0.64668	0.517841	0.610507	TRMT10B	protein_cc
ENSG0000(	711.3035	0.079471	0.026503	2.998585	0.002712	0.006096	BANP	protein_cc
ENSG0000(	711.2431	-0.11599	0.08647	-1.34136	0.179802	0.255029	SLC37A2	protein_cc
ENSG0000(	711.0977	-0.00322	0.038471	-0.0838	0.933212	0.953405	COX20	protein_cc
ENSG0000(	710.9023	-0.20543	0.03642	-5.64054	1.70E-08	9.07E-08	LOH12CR1	protein_cc
ENSG0000(	710.4183	0.717222	0.109108	6.573529	4.91E-11	3.92E-10	RPL13P12	pseudoge
ENSG0000(	710.4055	-0.19351	0.042298	-4.57486	4.77E-06	1.73E-05	OTUD3	protein_cc
ENSG0000(	710.3385	-0.14173	0.062951	-2.25146	0.024356	0.044485	MYOM2	protein_cc
ENSG0000(	710.2732	0.168221	0.034852	4.82669	1.39E-06	5.49E-06	POLR3K	protein_cc
ENSG0000(	710.079	-0.15699	0.061638	-2.54697	0.010866	0.021541	APOL3	protein_cc
ENSG0000(	710.07	0.508538	0.096374	5.276718	1.32E-07	6.12E-07	CASP1	protein_cc
ENSG0000(	709.9788	0.198503	0.039657	5.005521	5.57E-07	2.34E-06	LINC0033(	processed
ENSG0000(	709.8084	0.240932	0.024199	9.956101	2.37E-23	1.52E-21	ETAA1	protein_cc
ENSG0000(	709.4624	-0.20687	0.137035	-1.50963	0.131137	0.194872	TENM3	protein_cc
ENSG0000(	709.2529	-0.18181	0.037927	-4.79361	1.64E-06	6.40E-06	STX17	protein_cc
ENSG0000(	708.9286	0.331834	0.034802	9.534786	1.50E-21	6.74E-20	DCPS	protein_cc
ENSG0000(	708.9086	0.393093	0.143277	2.743589	0.006077	0.012715	CPNE4	protein_cc
ENSG0000(	708.5345	0.148318	0.050571	2.932838	0.003359	0.007414	HHLA3	protein_cc
ENSG0000(	708.4439	0.012008	0.06267	0.1916	0.848055	0.889997	HCN3	protein_cc
ENSG0000(	708.0879	-0.39714	0.063436	-6.26049	3.84E-10	2.67E-09	RASGEF1B	protein_cc
ENSG0000(	706.5701	0.110737	0.04781	2.316182	0.020548	0.038152	CCDC23	protein_cc
ENSG0000(	706.4728	-0.40954	0.071605	-5.71947	1.07E-08	5.90E-08	WI2-1896	pseudoge
ENSG0000(	706.2071	-0.49235	0.07682	-6.40917	1.46E-10	1.08E-09	TNFAIP8L3	protein_cc
ENSG0000(	706.1705	-0.41963	0.080798	-5.19354	2.06E-07	9.30E-07	WDFY4	protein_cc
ENSG0000(	705.6756	-0.28862	0.101057	-2.85603	0.00429	0.00927	GAL3ST1	protein_cc
ENSG0000(	705.6614	0.046169	0.062671	0.736685	0.461314	0.55753	PCK2	protein_cc

ENSG0000(	705.6368	-0.78996	0.091531	-8.63058	6.10E-18	1.52E-16	TSPYL5	protein_cc
ENSG0000(	705.4886	0.108482	0.029031	3.73679	0.000186	0.000519	SEPSECS	protein_cc
ENSG0000(	705.3661	0.096585	0.029925	3.227519	0.001249	0.00299	ADAL	protein_cc
ENSG0000(	705.2584	-0.57287	0.07087	-8.08332	6.30E-16	1.12E-14	MIR600H(	sense_intr
ENSG0000(	704.9077	-1.27786	0.129783	-9.84609	7.13E-23	4.08E-21	RFPL1S	antisense
ENSG0000(	704.7944	0.267434	0.046796	5.714857	1.10E-08	6.05E-08	TP53TG1	lincRNA
ENSG0000(	704.6253	0.214521	0.037793	5.676176	1.38E-08	7.48E-08	WDR5B	protein_cc
ENSG0000(	704.0533	0.256935	0.049053	5.237929	1.62E-07	7.43E-07	TMEM107	protein_cc
ENSG0000(	704.0089	0.713913	0.153207	4.659789	3.17E-06	1.18E-05	SRPX2	protein_cc
ENSG0000(	703.7802	0.220677	0.04414	4.999435	5.75E-07	2.41E-06	BAK1	protein_cc
ENSG0000(	703.6946	0.394207	0.069173	5.698856	1.21E-08	6.61E-08	TRIM21	protein_cc
ENSG0000(	703.3557	0.136238	0.038851	3.506671	0.000454	0.001181	ARL13B	protein_cc
ENSG0000(	702.9775	0.250334	0.041649	6.010595	1.85E-09	1.15E-08	ZNF347	protein_cc
ENSG0000(	702.9471	0.91014	0.079068	11.51084	1.16E-30	3.09E-28	GINS1	protein_cc
ENSG0000(	702.6721	-0.19451	0.032803	-5.92977	3.03E-09	1.83E-08	SLC30A4	protein_cc
ENSG0000(	702.6482	0.319263	0.101181	3.155358	0.001603	0.003766	LIPG	protein_cc
ENSG0000(	702.4019	0.12833	0.061822	2.075799	0.037913	0.066171	CDR2	protein_cc
ENSG0000(	702.3638	0.080498	0.04102	1.962424	0.049713	0.084076	ZNF587	protein_cc
ENSG0000(	701.9677	-0.63222	0.063962	-9.88426	4.87E-23	2.88E-21	PHACTR2	protein_cc
ENSG0000(	701.5246	-0.1455	0.040262	-3.6138	0.000302	0.000811	ANKRD42	protein_cc
ENSG0000(	700.8072	0.120289	0.061437	1.957935	0.050238	0.084848	FAR2	protein_cc
ENSG0000(	700.3398	-1.68331	0.154232	-10.9141	9.87E-28	1.44E-25	RYR2	protein_cc
ENSG0000(	699.9925	0.474234	0.056064	8.458796	2.70E-17	6.06E-16	BRCA1	protein_cc
ENSG0000(	699.7902	-0.58224	0.1351	-4.30972	1.63E-05	5.45E-05	SEMA3D	protein_cc
ENSG0000(	699.7607	0.142126	0.031278	4.544003	5.52E-06	1.99E-05	PGBD1	protein_cc
ENSG0000(	699.6124	0.143527	0.036533	3.928672	8.54E-05	0.000253	PUS1	protein_cc
ENSG0000(	699.5631	-0.05682	0.056875	-0.99901	0.317789	0.411472	UVSSA	protein_cc
ENSG0000(	699.5588	-0.26656	0.096069	-2.77467	0.005526	0.011667	KIAA2022	protein_cc
ENSG0000(	699.3172	0.010758	0.041373	0.260034	0.794838	0.848085	GKAP1	protein_cc
ENSG0000(	699.1893	-0.00635	0.042	-0.15127	0.879759	0.914101	AC244394	protein_cc
ENSG0000(	699.071	-0.16291	0.029586	-5.50621	3.67E-08	1.86E-07	MCU	protein_cc
ENSG0000(	698.7637	0.359139	0.076324	4.705482	2.53E-06	9.61E-06	C1QTNF6	protein_cc
ENSG0000(	698.6007	0.138792	0.047059	2.949343	0.003185	0.00706	CRYBB2P1	pseudoge
ENSG0000(	698.2167	0.055487	0.045595	1.216953	0.223622	0.306709	CCDC41	protein_cc
ENSG0000(	697.8569	0.184989	0.04015	4.607458	4.08E-06	1.50E-05	RANGRF	protein_cc
ENSG0000(	697.5188	0.164464	0.049207	3.342306	0.000831	0.002055	L3HYPDH	protein_cc
ENSG0000(	697.5111	0.247848	0.063147	3.92492	8.68E-05	0.000257	BTN2A2	protein_cc
ENSG0000(	697.1025	0.481065	0.048176	9.985525	1.76E-23	1.15E-21	RAD18	protein_cc
ENSG0000(	696.9167	-0.12338	0.035573	-3.46835	0.000524	0.001346	ZNF780A	protein_cc
ENSG0000(	696.8674	0.249297	0.05103	4.885266	1.03E-06	4.16E-06	ZNF581	protein_cc
ENSG0000(	696.6976	-0.23498	0.079423	-2.95865	0.00309	0.006869	TEK	protein_cc
ENSG0000(	696.4501	0.291551	0.056788	5.133992	2.84E-07	1.25E-06	ZNF880	protein_cc
ENSG0000(	696.4206	0.088639	0.018891	4.692037	2.70E-06	1.02E-05	ZNF174	protein_cc
ENSG0000(	696.3767	0.334422	0.070592	4.737413	2.16E-06	8.30E-06	MPPED2	protein_cc
ENSG0000(	696.0658	-0.9419	0.113031	-8.33316	7.87E-17	1.63E-15	PRSS35	protein_cc
ENSG0000(	695.8373	-0.12086	0.027307	-4.42592	9.60E-06	3.32E-05	ERICH1	protein_cc
ENSG0000(	695.8295	0.061693	0.07055	0.874462	0.381867	0.478515	FSTL3	protein_cc
ENSG0000(	695.7263	-0.21191	0.042061	-5.03815	4.70E-07	2.00E-06	PRPF40B	protein_cc
ENSG0000(	695.1893	0.259078	0.048505	5.341298	9.23E-08	4.40E-07	HSPB11	protein_cc
ENSG0000(	695.0758	0.486998	0.088876	5.479518	4.26E-08	2.14E-07	TMEM45A	protein_cc
ENSG0000(	694.9451	0.164646	0.029625	5.557619	2.73E-08	1.42E-07	SLX4IP	protein_cc
ENSG0000(	694.9009	0.318935	0.047307	6.74187	1.56E-11	1.35E-10	PUS7	protein_cc
ENSG0000(	694.6432	0.096569	0.032184	3.000502	0.002695	0.006064	FGFR1OP	protein_cc
ENSG0000(	694.5078	-0.12049	0.042504	-2.83476	0.004586	0.009839	RP11-644	antisense
ENSG0000(	694.4933	0.223957	0.032109	6.974895	3.06E-12	2.95E-11	MITD1	protein_cc
ENSG0000(	694.2948	-0.5853	0.077373	-7.56461	3.89E-14	5.08E-13	KCNC4	protein_cc
ENSG0000(	694.0075	-0.92182	0.08829	-10.4409	1.61E-25	1.54E-23	ERC2	protein_cc
ENSG0000(	693.9848	-0.77645	0.092673	-8.3783	5.37E-17	1.14E-15	ACSM5	protein_cc
ENSG0000(	693.4222	-0.58482	0.083683	-6.98847	2.78E-12	2.70E-11	ANKRD24	protein_cc

ENSG0000(	693.2344	-0.36688	0.059021	-6.21607	5.10E-10	3.47E-09	ANKRD26	protein_cc
ENSG0000(	692.6819	-0.65984	0.145852	-4.52403	6.07E-06	2.17E-05	GRIN3A	protein_cc
ENSG0000(	692.6216	-1.28221	0.127888	-10.0261	1.17E-23	7.95E-22	RIMBP2	protein_cc
ENSG0000(	692.1027	-0.21342	0.071223	-2.99654	0.002731	0.006132	CCDC88C	protein_cc
ENSG0000(	691.0056	-0.03865	0.09733	-0.39715	0.691259	0.763334	BMPER	protein_cc
ENSG0000(	690.7689	-0.29754	0.081595	-3.64647	0.000266	0.000722	TRNP1	protein_cc
ENSG0000(	690.7275	0.519211	0.076298	6.805059	1.01E-11	8.96E-11	EEF1A1P6	pseudoge
ENSG0000(	690.6644	0.260444	0.030595	8.512574	1.70E-17	3.92E-16	BLOC1S4	protein_cc
ENSG0000(	690.1953	-0.18391	0.077624	-2.36922	0.017826	0.033553	RP11-196	sense_ove
ENSG0000(	689.7961	-0.04187	0.038643	-1.08345	0.278608	0.368995	AC024560	pseudoge
ENSG0000(	689.6096	-0.36173	0.054404	-6.64895	2.95E-11	2.44E-10	RP11-244	sense_ove
ENSG0000(	689.3179	-0.14882	0.089327	-1.66606	0.095701	0.148743	HS6ST2	protein_cc
ENSG0000(	689.0148	-0.0243	0.029766	-0.81646	0.414235	0.511208	ZNF654	protein_cc
ENSG0000(	688.9184	-0.15996	0.056814	-2.81546	0.004871	0.010399	RP4-717I2	processed
ENSG0000(	688.8732	0.368677	0.03232	11.40721	3.85E-30	8.94E-28	PRIMPOL	protein_cc
ENSG0000(	688.8582	0.024631	0.064495	0.381905	0.702532	0.772732	ABCB9	protein_cc
ENSG0000(	688.6285	-0.24946	0.044599	-5.59334	2.23E-08	1.17E-07	RP11-53C	lincRNA
ENSG0000(	688.6258	-0.39075	0.065344	-5.97984	2.23E-09	1.38E-08	ZBTB7C	protein_cc
ENSG0000(	688.5692	0.043664	0.030408	1.43591	0.151028	0.220111	DCP1B	protein_cc
ENSG0000(	688.256	0.178839	0.034155	5.236111	1.64E-07	7.51E-07	ZNF446	protein_cc
ENSG0000(	688.0214	0.038765	0.026101	1.485192	0.137493	0.202916	SPRTN	protein_cc
ENSG0000(	687.9738	-0.21452	0.129395	-1.6579	0.097337	0.150909	LINC0059	lincRNA
ENSG0000(	687.8009	-0.42036	0.124315	-3.38138	0.000721	0.001805	RP4-555D	lincRNA
ENSG0000(	687.7151	0.629059	0.081825	7.687827	1.50E-14	2.10E-13	ZWINT	protein_cc
ENSG0000(	687.5725	0.151069	0.08219	1.838048	0.066055	0.107702	SDK1	protein_cc
ENSG0000(	687.506	0.107526	0.055585	1.93445	0.053058	0.089016	MOK	protein_cc
ENSG0000(	687.3333	-0.67053	0.096241	-6.96719	3.23E-12	3.11E-11	MYOM1	protein_cc
ENSG0000(	687.2739	1.001241	0.127229	7.869623	3.56E-15	5.61E-14	RPLP0P6	pseudoge
ENSG0000(	687.1326	-0.0325	0.042149	-0.77115	0.440618	0.537566	ZNF514	protein_cc
ENSG0000(	687.0588	-1.42799	0.138038	-10.3449	4.41E-25	3.96E-23	CACNA1B	protein_cc
ENSG0000(	686.9581	0.227144	0.08195	2.771754	0.005576	0.011761	CCDC40	protein_cc
ENSG0000(	686.8152	-0.9555	0.113083	-8.44958	2.92E-17	6.52E-16	ACBD7	protein_cc
ENSG0000(	686.7791	-0.48801	0.086343	-5.65203	1.59E-08	8.53E-08	HERC2P3	pseudoge
ENSG0000(	686.2369	0.253599	0.026849	9.445223	3.55E-21	1.49E-19	UTP15	protein_cc
ENSG0000(	685.0917	0.660862	0.145047	4.556189	5.21E-06	1.88E-05	S100A4	protein_cc
ENSG0000(	684.6979	-0.7139	0.076828	-9.29215	1.51E-20	5.62E-19	RUNX1T1	protein_cc
ENSG0000(	684.3762	0.001806	0.035906	0.050285	0.959895	0.972526	NUFIP1	protein_cc
ENSG0000(	684.351	0.027591	0.036109	0.764099	0.444808	0.541625	LCORL	protein_cc
ENSG0000(	683.673	0.074536	0.086406	0.862617	0.388348	0.485055	CDON	protein_cc
ENSG0000(	683.6539	-1.72257	0.161389	-10.6734	1.36E-26	1.64E-24	MPPED1	protein_cc
ENSG0000(	683.5243	-0.51697	0.10109	-5.11396	3.15E-07	1.38E-06	SDS	protein_cc
ENSG0000(	683.3646	0.416719	0.078999	5.274996	1.33E-07	6.16E-07	ALPK3	protein_cc
ENSG0000(	683.2106	0.161483	0.06107	2.644251	0.008187	0.016689	ABC7-423	pseudoge
ENSG0000(	683.14	-0.18159	0.041922	-4.33166	1.48E-05	4.97E-05	FRS3	protein_cc
ENSG0000(	683.1373	-0.07074	0.070552	-1.00272	0.315994	0.409586	PALMD	protein_cc
ENSG0000(	682.9391	0.135646	0.033542	4.044081	5.25E-05	0.000161	CRLF3	protein_cc
ENSG0000(	682.6344	-0.16878	0.066732	-2.52915	0.011434	0.022541	MB21D2	protein_cc
ENSG0000(	682.4604	0.307308	0.035313	8.70234	3.25E-18	8.40E-17	C17orf58	protein_cc
ENSG0000(	682.2436	0.420332	0.07806	5.384721	7.26E-08	3.51E-07	C1orf54	protein_cc
ENSG0000(	682.045	0.320029	0.039413	8.119847	4.67E-16	8.51E-15	RP11-109	lincRNA
ENSG0000(	681.8476	0.063283	0.034666	1.82553	0.067921	0.11035	EP400NL	protein_cc
ENSG0000(	681.7796	-1.47434	0.151502	-9.73145	2.21E-22	1.14E-20	MAP7D2	protein_cc
ENSG0000(	681.3929	0.147814	0.030384	4.864933	1.14E-06	4.58E-06	DIABLO	protein_cc
ENSG0000(	681.1785	-0.66536	0.063072	-10.5492	5.12E-26	5.46E-24	DEPTOR	protein_cc
ENSG0000(	680.9814	-0.37563	0.100212	-3.74837	0.000178	0.000498	ATP10B	protein_cc
ENSG0000(	680.9139	0.390189	0.104538	3.732524	0.00019	0.000528	RGN	protein_cc
ENSG0000(	680.7461	0.013305	0.033322	0.399282	0.689686	0.762108	ZRSR2	protein_cc
ENSG0000(	680.5933	-0.30264	0.229494	-1.31874	0.187255	0.263992	TXLNG2P	pseudoge
ENSG0000(	680.3538	-0.54651	0.108376	-5.04268	4.59E-07	1.96E-06	BTBD11	protein_cc

ENSG0000(	679.9422	-0.2552	0.092744	-2.75167	0.005929	0.012433	EDN1	protein_cc
ENSG0000(	679.5484	0.033285	0.038983	0.853853	0.393186	0.489818	STXBP4	protein_cc
ENSG0000(	678.817	0.62178	0.081868	7.594867	3.08E-14	4.09E-13	GPX7	protein_cc
ENSG0000(	678.6159	-0.05274	0.050819	-1.03772	0.2994	0.391694	GPER1	protein_cc
ENSG0000(	678.3198	-0.16095	0.078892	-2.04016	0.041334	0.071424	BMF	protein_cc
ENSG0000(	677.9875	-0.14528	0.072592	-2.00134	0.045356	0.077498	PSD4	protein_cc
ENSG0000(	677.961	1.14896	0.135796	8.460909	2.65E-17	5.97E-16	MIR4435-	lincRNA
ENSG0000(	677.8826	0.61272	0.131646	4.654285	3.25E-06	1.21E-05	CREB3L1	protein_cc
ENSG0000(	677.4472	0.070072	0.077855	0.90003	0.368104	0.464353	PGM5	protein_cc
ENSG0000(	677.2494	0.237774	0.047615	4.993697	5.92E-07	2.48E-06	RBM43	protein_cc
ENSG0000(	677.1244	-1.07669	0.13382	-8.04582	8.57E-16	1.49E-14	ICAM5	protein_cc
ENSG0000(	676.7567	0.284865	0.052305	5.446235	5.14E-08	2.55E-07	ORMDL2	protein_cc
ENSG0000(	676.4899	-0.26982	0.061793	-4.36656	1.26E-05	4.28E-05	SHPRH	protein_cc
ENSG0000(	676.4648	-0.36109	0.084101	-4.29349	1.76E-05	5.84E-05	FAM89A	protein_cc
ENSG0000(	676.4141	0.445539	0.047398	9.399978	5.46E-21	2.20E-19	RBL1	protein_cc
ENSG0000(	676.1526	0.034452	0.020387	1.689908	0.091046	0.142407	SCYL3	protein_cc
ENSG0000(	675.9941	-0.01482	0.075703	-0.19572	0.844831	0.887406	NFATC1	protein_cc
ENSG0000(	675.8334	0.016848	0.028645	0.588157	0.556427	0.645815	WDR89	protein_cc
ENSG0000(	675.4917	1.281169	0.128103	10.00107	1.51E-23	1.00E-21	PBK	protein_cc
ENSG0000(	675.1008	0.347087	0.044955	7.720754	1.16E-14	1.66E-13	ZNRD1	protein_cc
ENSG0000(	674.9057	0.21474	0.034623	6.202213	5.57E-10	3.77E-09	SPDL1	protein_cc
ENSG0000(	674.469	-0.77003	0.096477	-7.98152	1.45E-15	2.43E-14	SIDT1	protein_cc
ENSG0000(	674.4647	0.076075	0.024299	3.130735	0.001744	0.00407	GTF2E1	protein_cc
ENSG0000(	673.9227	-0.29458	0.078931	-3.73219	0.00019	0.000528	TIMP3	protein_cc
ENSG0000(	673.88	-0.21846	0.037944	-5.75741	8.54E-09	4.79E-08	KLHL15	protein_cc
ENSG0000(	673.766	0.555202	0.099276	5.592531	2.24E-08	1.18E-07	KIF18B	protein_cc
ENSG0000(	673.4839	-0.22015	0.10754	-2.04718	0.04064	0.07037	PRODH	protein_cc
ENSG0000(	673.181	0.170545	0.039729	4.292743	1.76E-05	5.86E-05	SCO2	protein_cc
ENSG0000(	672.9373	0.346739	0.064153	5.404842	6.49E-08	3.17E-07	TRIP4	protein_cc
ENSG0000(	672.7363	-0.10571	0.042888	-2.46468	0.013713	0.026563	ZNF425	protein_cc
ENSG0000(	672.5539	-0.16133	0.227875	-0.70798	0.478959	0.574348	EIF1AY	protein_cc
ENSG0000(	672.1616	-0.13326	0.032918	-4.04816	5.16E-05	0.000158	ZBTB24	protein_cc
ENSG0000(	672.1524	-0.30849	0.08844	-3.48811	0.000486	0.001258	ASXL3	protein_cc
ENSG0000(	671.3205	-0.03581	0.085157	-0.42054	0.674091	0.748719	C2orf27A	protein_cc
ENSG0000(	670.6244	0.864264	0.119496	7.232575	4.74E-13	5.16E-12	SHROOM3	protein_cc
ENSG0000(	670.5318	0.114483	0.030709	3.728043	0.000193	0.000536	FAM173B	protein_cc
ENSG0000(	670.1824	-0.22778	0.080183	-2.84082	0.0045	0.009676	PIK3R5	protein_cc
ENSG0000(	669.7355	0.031685	0.06234	0.508252	0.611276	0.694921	HAGHL	protein_cc
ENSG0000(	669.4018	0.093088	0.080189	1.160865	0.245697	0.332064	IGDCC3	protein_cc
ENSG0000(	669.2473	0.194951	0.049089	3.971345	7.15E-05	0.000214	SLC27A5	protein_cc
ENSG0000(	669.1988	0.47181	0.094396	4.998212	5.79E-07	2.43E-06	2-Mar	protein_cc
ENSG0000(	669.0754	0.322661	0.060538	5.329892	9.83E-08	4.66E-07	CIB2	protein_cc
ENSG0000(	668.9804	0.157196	0.029517	5.325689	1.01E-07	4.77E-07	MRPS18C	protein_cc
ENSG0000(	668.6716	0.171977	0.034142	5.037178	4.72E-07	2.01E-06	SPRYD4	protein_cc
ENSG0000(	668.5626	0.255409	0.061386	4.160694	3.17E-05	0.000101	ERV3-1	protein_cc
ENSG0000(	668.1031	0.149946	0.031689	4.731744	2.23E-06	8.52E-06	TMA16	protein_cc
ENSG0000(	668.0714	0.027812	0.025638	1.084821	0.278001	0.368375	TSEN2	protein_cc
ENSG0000(	667.8558	0.282699	0.049932	5.661683	1.50E-08	8.09E-08	RP11-43F	pseudoge
ENSG0000(	667.6907	-0.03246	0.046538	-0.69758	0.485443	0.580325	RNPC3	protein_cc
ENSG0000(	667.5763	0.314493	0.06885	4.567824	4.93E-06	1.79E-05	CHIC2	protein_cc
ENSG0000(	667.5136	-0.38315	0.056617	-6.76741	1.31E-11	1.14E-10	MED12L	protein_cc
ENSG0000(	667.3527	0.04756	0.060415	0.787232	0.431146	0.528195	WIPI1	protein_cc
ENSG0000(	667.2944	-0.49168	0.057345	-8.57398	1.00E-17	2.40E-16	GIMAP8	protein_cc
ENSG0000(	667.2544	0.334897	0.043905	7.62776	2.39E-14	3.22E-13	NSMCE2	protein_cc
ENSG0000(	667.1503	-0.31478	0.078492	-4.01036	6.06E-05	0.000184	AOAH	protein_cc
ENSG0000(	667.0565	0.52069	0.05343	9.745205	1.93E-22	1.02E-20	ALG6	protein_cc
ENSG0000(	666.9758	-0.73573	0.107858	-6.82124	9.03E-12	8.06E-11	VSTM2B	protein_cc
ENSG0000(	666.9682	-0.00075	0.068363	-0.01104	0.991195	0.993999	ARHGAP2	protein_cc
ENSG0000(	666.3644	-0.15277	0.12882	-1.18589	0.235666	0.320424	LINC0064	lincRNA

ENSG0000(	666.2195	0.147655	0.06392	2.310008	0.020888	0.038716	KCNJ8	protein_cc
ENSG0000(	666.0445	0.015042	0.035602	0.422508	0.672654	0.747547	ZNF324	protein_cc
ENSG0000(	665.765	0.001123	0.04387	0.0256	0.979577	0.985665	STK3	protein_cc
ENSG0000(	665.3742	0.518282	0.13301	3.896557	9.76E-05	0.000286	VASN	protein_cc
ENSG0000(	665.2394	0.28112	0.079637	3.53001	0.000416	0.001089	RNF135	protein_cc
ENSG0000(	665.1067	-0.70152	0.18612	-3.76917	0.000164	0.000461	PDYN	protein_cc
ENSG0000(	664.9981	-0.05706	0.134032	-0.42574	0.670297	0.745491	HLA-DRB(	pseudoge
ENSG0000(	664.8067	-0.86625	0.120669	-7.17874	7.04E-13	7.50E-12	FAM19A2	protein_cc
ENSG0000(	664.7343	0.428134	0.047488	9.015569	1.96E-19	6.11E-18	FBXW9	protein_cc
ENSG0000(	664.2515	0.23028	0.029585	7.783693	7.04E-15	1.06E-13	METTL22	protein_cc
ENSG0000(	663.6654	-0.06183	0.058623	-1.05475	0.29154	0.383057	PPAPDC3	protein_cc
ENSG0000(	663.2726	-0.28497	0.115885	-2.4591	0.013929	0.026936	NETO1	protein_cc
ENSG0000(	663.1807	0.257086	0.051404	5.001294	5.69E-07	2.39E-06	KIAA0922	protein_cc
ENSG0000(	662.9934	0.228928	0.030818	7.428429	1.10E-13	1.33E-12	SLC35A1	protein_cc
ENSG0000(	662.8364	0.200416	0.03562	5.626429	1.84E-08	9.80E-08	TDP1	protein_cc
ENSG0000(	662.7163	-0.66127	0.099421	-6.65124	2.91E-11	2.40E-10	RTN4R	protein_cc
ENSG0000(	662.7052	-0.04117	0.043012	-0.95708	0.338527	0.433475	ATP11C	protein_cc
ENSG0000(	662.6319	0.225255	0.060521	3.721925	0.000198	0.000549	TMEM216	protein_cc
ENSG0000(	662.5547	0.141644	0.046352	3.055858	0.002244	0.005129	SLC50A1	protein_cc
ENSG0000(	662.5206	-0.40153	0.080993	-4.95756	7.14E-07	2.95E-06	IKZF1	protein_cc
ENSG0000(	662.4842	0.220708	0.034558	6.386574	1.70E-10	1.24E-09	LSM6	protein_cc
ENSG0000(	662.4149	-0.75664	0.110425	-6.85209	7.28E-12	6.61E-11	RGS7	protein_cc
ENSG0000(	662.1941	0.026154	0.033611	0.778152	0.436479	0.533473	TRIM16	protein_cc
ENSG0000(	661.8748	0.253454	0.039516	6.413978	1.42E-10	1.06E-09	ZNF584	protein_cc
ENSG0000(	661.6729	0.110879	0.039368	2.816517	0.004855	0.010367	CRADD	protein_cc
ENSG0000(	661.2126	-0.9867	0.122437	-8.05883	7.70E-16	1.35E-14	MRC1	protein_cc
ENSG0000(	661.152	-0.10849	0.036988	-2.93307	0.003356	0.00741	PRDM10	protein_cc
ENSG0000(	661.0396	0.209401	0.029843	7.016841	2.27E-12	2.23E-11	UBXN8	processed
ENSG0000(	660.7063	-0.33705	0.053858	-6.25799	3.90E-10	2.70E-09	RASSF3	protein_cc
ENSG0000(	660.4591	0.218768	0.045261	4.833453	1.34E-06	5.32E-06	RP11-15H	pseudoge
ENSG0000(	660.346	0.172444	0.02717	6.346928	2.20E-10	1.58E-09	C1orf131	protein_cc
ENSG0000(	660.1975	0.182932	0.03858	4.741655	2.12E-06	8.14E-06	TRMT13	protein_cc
ENSG0000(	659.757	-0.02521	0.092604	-0.27225	0.785432	0.840273	KCNK2	protein_cc
ENSG0000(	659.7513	0.157498	0.034407	4.577542	4.70E-06	1.71E-05	WDR83	protein_cc
ENSG0000(	659.7487	0.301912	0.113503	2.659951	0.007815	0.016	STK32B	protein_cc
ENSG0000(	659.4589	-0.15377	0.037122	-4.1422	3.44E-05	0.000109	RNASEH1	protein_cc
ENSG0000(	659.4018	-0.52275	0.082845	-6.31	2.79E-10	1.98E-09	SPRN	protein_cc
ENSG0000(	659.3279	-0.17682	0.049658	-3.56076	0.00037	0.000979	ANKDD1A	protein_cc
ENSG0000(	659.3102	0.075414	0.031723	2.377231	0.017443	0.032925	ERI2	protein_cc
ENSG0000(	658.9888	0.337336	0.037601	8.971524	2.92E-19	8.86E-18	BNIP1	protein_cc
ENSG0000(	658.864	-0.1605	0.052839	-3.0375	0.002385	0.005428	TRIM46	protein_cc
ENSG0000(	658.7945	-0.25191	0.106363	-2.36841	0.017865	0.03362	KCNH8	protein_cc
ENSG0000(	658.5797	0.20941	0.062624	3.34394	0.000826	0.002044	SPATC1L	protein_cc
ENSG0000(	658.5116	0.897525	0.166339	5.39577	6.82E-08	3.32E-07	PTX3	protein_cc
ENSG0000(	658.2553	0.39036	0.038144	10.23397	1.40E-24	1.13E-22	ZCCHC9	protein_cc
ENSG0000(	658.2262	-0.09217	0.038071	-2.42108	0.015475	0.029607	ZNF707	protein_cc
ENSG0000(	658.0282	-0.88284	0.10153	-8.69532	3.46E-18	8.89E-17	RASD2	protein_cc
ENSG0000(	657.909	-0.25395	0.059861	-4.24243	2.21E-05	7.21E-05	DGKH	protein_cc
ENSG0000(	657.8744	0.090318	0.037786	2.390272	0.016836	0.031924	ZNF441	protein_cc
ENSG0000(	657.4962	0.149996	0.040021	3.747917	0.000178	0.000499	KIAA0020	protein_cc
ENSG0000(	657.1712	0.295545	0.041808	7.069138	1.56E-12	1.57E-11	ZNF439	protein_cc
ENSG0000(	657.1675	-0.34917	0.105619	-3.30598	0.000946	0.002316	ENPP6	protein_cc
ENSG0000(	656.929	-0.20657	0.052929	-3.90273	9.51E-05	0.000279	NOMO2	protein_cc
ENSG0000(	656.8396	0.455787	0.124829	3.651303	0.000261	0.000709	EMILIN2	protein_cc
ENSG0000(	656.7678	0.215785	0.045755	4.716064	2.40E-06	9.15E-06	SDHAF1	protein_cc
ENSG0000(	656.6576	0.007534	0.033146	0.22729	0.820198	0.868117	ZNF430	protein_cc
ENSG0000(	655.908	0.080389	0.032221	2.494903	0.012599	0.024585	COX19	protein_cc
ENSG0000(	655.8186	0.199872	0.044797	4.461732	8.13E-06	2.85E-05	POLH	protein_cc
ENSG0000(	654.7669	0.160511	0.027376	5.863249	4.54E-09	2.66E-08	RPUSD2	protein_cc

ENSG0000(	654.4228	0.6058	0.103378	5.860068	4.63E-09	2.70E-08	RAB32	protein_cc
ENSG0000(	654.2849	-0.02727	0.045182	-0.60349	0.54618	0.636652	FBXO36	protein_cc
ENSG0000(	653.6081	1.694057	0.17933	9.446603	3.50E-21	1.47E-19	CA3	protein_cc
ENSG0000(	652.3901	0.178128	0.04028	4.422262	9.77E-06	3.38E-05	IMMP1L	protein_cc
ENSG0000(	652.324	-0.89615	0.082557	-10.8549	1.89E-27	2.63E-25	NOS1AP	protein_cc
ENSG0000(	651.9537	0.254538	0.051849	4.909179	9.15E-07	3.72E-06	ZNF28	protein_cc
ENSG0000(	651.9418	0.004474	0.027194	0.164522	0.86932	0.906156	FAM11A	protein_cc
ENSG0000(	651.674	0.068044	0.038266	1.778155	0.075378	0.120876	PTOV1-AS	antisense
ENSG0000(	651.4928	0.136851	0.030634	4.467323	7.92E-06	2.78E-05	ZSCAN9	protein_cc
ENSG0000(	651.4705	0.306591	0.033088	9.265902	1.93E-20	7.06E-19	C18orf21	protein_cc
ENSG0000(	651.4414	0.003163	0.029669	0.106595	0.915111	0.940181	NME6	protein_cc
ENSG0000(	651.1673	-0.2219	0.026244	-8.45519	2.79E-17	6.23E-16	NUBPL	protein_cc
ENSG0000(	650.9941	-1.23426	0.109769	-11.2442	2.47E-29	4.91E-27	MAP3K9	protein_cc
ENSG0000(	650.9909	0.342258	0.042316	8.088062	6.06E-16	1.08E-14	ZNF473	protein_cc
ENSG0000(	650.9294	-0.43232	0.115481	-3.74364	0.000181	0.000507	PCDH8	protein_cc
ENSG0000(	650.6537	0.362859	0.042284	8.581475	9.37E-18	2.26E-16	FAM133B	protein_cc
ENSG0000(	650.5756	-0.54039	0.136347	-3.96334	7.39E-05	0.000221	IL1B	protein_cc
ENSG0000(	650.4952	-0.1176	0.147864	-0.79536	0.426406	0.523543	4-Mar	protein_cc
ENSG0000(	650.4229	0.238664	0.051825	4.605155	4.12E-06	1.51E-05	ZNF883	lincRNA
ENSG0000(	650.3895	0.03107	0.028169	1.102969	0.27004	0.359471	SNRNP48	protein_cc
ENSG0000(	649.86	0.343618	0.077809	4.416197	1.00E-05	3.47E-05	RNF122	protein_cc
ENSG0000(	649.6147	-0.11708	0.053791	-2.17657	0.029513	0.052874	EML6	protein_cc
ENSG0000(	649.2895	-0.61284	0.079116	-7.74613	9.47E-15	1.39E-13	PRICKLE1	protein_cc
ENSG0000(	649.1309	0.980154	0.122238	8.01843	1.07E-15	1.84E-14	FBXO17	protein_cc
ENSG0000(	648.6841	0.265642	0.044256	6.002414	1.94E-09	1.21E-08	EMC9	protein_cc
ENSG0000(	648.2882	-0.07201	0.156327	-0.46066	0.645045	0.72454	LUM	protein_cc
ENSG0000(	648.0877	0.108214	0.023892	4.529229	5.92E-06	2.12E-05	MOCS3	protein_cc
ENSG0000(	647.548	0.249885	0.150588	1.659397	0.097036	0.1505	PAX8-AS1	processed
ENSG0000(	647.1927	-2.10218	0.190552	-11.032	2.68E-28	4.39E-26	TESPA1	protein_cc
ENSG0000(	646.686	1.985898	0.208934	9.504912	2.00E-21	8.82E-20	MEOX2	protein_cc
ENSG0000(	646.6571	0.066601	0.03368	1.977435	0.047992	0.081441	PIBF1	protein_cc
ENSG0000(	646.5571	0.144285	0.054985	2.624084	0.008688	0.0176	ACOT2	protein_cc
ENSG0000(	646.315	0.181344	0.091557	1.980673	0.047628	0.080899	TRIM36	protein_cc
ENSG0000(	646.1722	0.173147	0.067653	2.559351	0.010487	0.020857	TMEM185	protein_cc
ENSG0000(	645.9387	0.429876	0.080238	5.357497	8.44E-08	4.04E-07	ZIC2	protein_cc
ENSG0000(	645.8105	0.070425	0.075999	0.926661	0.354103	0.449698	TUSC1	protein_cc
ENSG0000(	645.7716	0.033045	0.046067	0.717316	0.473179	0.568977	POLD4	protein_cc
ENSG0000(	645.4862	0.101213	0.108053	0.936697	0.348914	0.444344	KIAA1456	protein_cc
ENSG0000(	645.3978	0.075755	0.0493	1.536611	0.124389	0.186347	ZNF503	protein_cc
ENSG0000(	645.2605	0.169463	0.091324	1.855628	0.063507	0.104124	IL18	protein_cc
ENSG0000(	645.1863	-0.17295	0.052784	-3.27652	0.001051	0.00255	LRFN1	protein_cc
ENSG0000(	645.0928	1.039883	0.151515	6.863229	6.73E-12	6.14E-11	TFCP2L1	protein_cc
ENSG0000(	644.3992	0.150334	0.042976	3.498092	0.000469	0.001216	OCEL1	protein_cc
ENSG0000(	644.1536	0.517892	0.104942	4.935024	8.01E-07	3.29E-06	CASP4	protein_cc
ENSG0000(	643.5618	-0.04021	0.034116	-1.17865	0.238538	0.323814	THAP8	protein_cc
ENSG0000(	643.4094	-0.64539	0.106379	-6.06686	1.30E-09	8.36E-09	PRDM8	protein_cc
ENSG0000(	643.0311	0.10401	0.094943	1.095505	0.273295	0.363193	LRRC32	protein_cc
ENSG0000(	643.0187	-1.03437	0.136773	-7.56269	3.95E-14	5.14E-13	CNTN3	protein_cc
ENSG0000(	642.9621	0.574733	0.05167	11.12314	9.68E-29	1.75E-26	PPP1R35	protein_cc
ENSG0000(	642.8097	0.06608	0.050042	1.320494	0.18667	0.263322	RP4-639F	antisense
ENSG0000(	642.4716	-0.216	0.077643	-2.78202	0.005402	0.011435	PACSIN3	protein_cc
ENSG0000(	642.177	-0.11033	0.02739	-4.02803	5.62E-05	0.000172	AP4E1	protein_cc
ENSG0000(	642.1463	-0.09963	0.041864	-2.37991	0.017317	0.032709	LMLN	protein_cc
ENSG0000(	642.0289	0.100254	0.030551	3.28156	0.001032	0.002509	ZNF570	protein_cc
ENSG0000(	641.9971	0.012396	0.051585	0.24031	0.81009	0.860252	SLC41A2	protein_cc
ENSG0000(	641.912	0.185066	0.045115	4.102056	4.09E-05	0.000128	MTFR1	protein_cc
ENSG0000(	641.6132	-0.00107	0.034161	-0.03143	0.974928	0.982407	RFXAP	protein_cc
ENSG0000(	641.3297	0.137626	0.033355	4.12614	3.69E-05	0.000116	PUS3	protein_cc
ENSG0000(	641.3034	-0.21418	0.042917	-4.99045	6.02E-07	2.52E-06	METTL10	protein_cc

ENSG0000(	640.9314	-1.5763	0.161992	-9.73069	2.23E-22	1.15E-20	GABRA5	protein_cc
ENSG0000(	640.7943	-0.36957	0.066189	-5.58356	2.36E-08	1.23E-07	PLCG2	protein_cc
ENSG0000(	640.2174	0.390412	0.056332	6.930576	4.19E-12	3.95E-11	CCDC34	protein_cc
ENSG0000(	640.1886	-0.13196	0.077975	-1.69234	0.090581	0.141769	FLVCR2	protein_cc
ENSG0000(	639.8939	0.434528	0.071738	6.057153	1.39E-09	8.84E-09	SNHG11	processed
ENSG0000(	639.815	-0.97987	0.120387	-8.13935	3.97E-16	7.33E-15	MYH11	protein_cc
ENSG0000(	639.8009	-0.3368	0.094289	-3.57197	0.000354	0.000942	TLR7	protein_cc
ENSG0000(	639.6481	-0.00549	0.032146	-0.17069	0.864468	0.902575	KBTBD4	protein_cc
ENSG0000(	639.2561	-0.00768	0.036383	-0.21119	0.832738	0.878	LACTB	protein_cc
ENSG0000(	639.1807	0.106945	0.025787	4.14732	3.36E-05	0.000106	ZNF44	protein_cc
ENSG0000(	639.0461	0.818323	0.12819	6.383677	1.73E-10	1.27E-09	GRIK1	protein_cc
ENSG0000(	638.9356	-0.10862	0.060444	-1.79711	0.072318	0.116596	ICA1	protein_cc
ENSG0000(	638.8574	-1.09538	0.120975	-9.05459	1.37E-19	4.38E-18	SOWAHA	protein_cc
ENSG0000(	638.402	0.05407	0.036772	1.470418	0.141448	0.207948	CCDC112	protein_cc
ENSG0000(	638.3595	-0.95867	0.13195	-7.26539	3.72E-13	4.13E-12	TENM2	protein_cc
ENSG0000(	638.1821	-0.08347	0.075818	-1.10089	0.270946	0.360513	KCNT2	protein_cc
ENSG0000(	637.9592	0.145133	0.040103	3.619019	0.000296	0.000797	YBEY	protein_cc
ENSG0000(	637.9333	-0.1844	0.046395	-3.97457	7.05E-05	0.000212	PRKCH	protein_cc
ENSG0000(	637.905	0.223437	0.032824	6.807207	9.95E-12	8.84E-11	RPIA	protein_cc
ENSG0000(	637.648	0.559219	0.057192	9.777984	1.40E-22	7.54E-21	STAG3L4	pseudoge
ENSG0000(	637.3803	-0.17921	0.030015	-5.97081	2.36E-09	1.45E-08	HOMEZ	protein_cc
ENSG0000(	637.3698	-0.08613	0.05037	-1.70988	0.087289	0.137324	ODF2L	protein_cc
ENSG0000(	637.304	0.190631	0.068136	2.797783	0.005145	0.010941	SERTAD3	protein_cc
ENSG0000(	636.7787	0.143221	0.032859	4.358624	1.31E-05	4.43E-05	ZNF383	protein_cc
ENSG0000(	636.7764	-0.35662	0.087603	-4.07082	4.68E-05	0.000145	MNDA	protein_cc
ENSG0000(	636.6645	0.205825	0.038223	5.384846	7.25E-08	3.51E-07	P2RY11	protein_cc
ENSG0000(	636.4595	1.126322	0.116076	9.703317	2.92E-22	1.48E-20	KIF2C	protein_cc
ENSG0000(	636.4539	-0.22255	0.051978	-4.28166	1.86E-05	6.14E-05	LRRC37A1	pseudoge
ENSG0000(	635.9929	0.760024	0.065692	11.56953	5.88E-31	1.67E-28	CHEK1	protein_cc
ENSG0000(	635.8166	0.141318	0.063835	2.21381	0.026842	0.048548	CLEC14A	protein_cc
ENSG0000(	635.6494	-0.53984	0.064822	-8.32812	8.21E-17	1.69E-15	OPRL1	protein_cc
ENSG0000(	635.6306	0.334912	0.044876	7.46297	8.46E-14	1.04E-12	AAED1	protein_cc
ENSG0000(	635.4699	-0.4375	0.079354	-5.51321	3.52E-08	1.79E-07	FAT4	protein_cc
ENSG0000(	635.1962	0.016396	0.061184	0.267973	0.78872	0.843027	IL3RA	protein_cc
ENSG0000(	635.0561	-0.01015	0.042008	-0.24151	0.809157	0.859462	KHK	protein_cc
ENSG0000(	634.9198	-0.53722	0.070134	-7.65995	1.86E-14	2.57E-13	ELOVL7	protein_cc
ENSG0000(	634.7761	-0.01783	0.08198	-0.21745	0.827855	0.873965	NCF2	protein_cc
ENSG0000(	634.2788	-0.12852	0.053957	-2.38196	0.017221	0.032544	RNF169	protein_cc
ENSG0000(	634.0835	0.14147	0.057195	2.473471	0.013381	0.025975	LRRC48	protein_cc
ENSG0000(	634.0654	0.099447	0.036561	2.720011	0.006528	0.013574	WDYHV1	protein_cc
ENSG0000(	633.9002	-0.31586	0.097636	-3.23502	0.001216	0.002919	SSPO	processed
ENSG0000(	633.6557	-0.43943	0.105702	-4.15728	3.22E-05	0.000102	EMX2	protein_cc
ENSG0000(	633.5373	0.139504	0.067086	2.079477	0.037573	0.065646	AGPAT2	protein_cc
ENSG0000(	633.4372	-1.46138	0.122818	-11.8988	1.20E-32	4.62E-30	PLCXD3	protein_cc
ENSG0000(	633.1299	0.139659	0.02812	4.966466	6.82E-07	2.83E-06	CTD-2228	protein_cc
ENSG0000(	632.8219	0.070598	0.041153	1.715512	0.086251	0.135885	RPL23AP8	pseudoge
ENSG0000(	632.7555	0.261616	0.044351	5.898718	3.66E-09	2.18E-08	COMMD8	protein_cc
ENSG0000(	632.7323	0.002216	0.058449	0.037918	0.969753	0.979014	CENPJ	protein_cc
ENSG0000(	632.6275	0.274646	0.042677	6.435455	1.23E-10	9.26E-10	MIPEP	protein_cc
ENSG0000(	632.2811	0.106168	0.057617	1.842656	0.065379	0.106739	RPGR	protein_cc
ENSG0000(	632.1577	-0.09839	0.10905	-0.90226	0.366918	0.463056	C2	protein_cc
ENSG0000(	631.9882	-0.07519	0.057366	-1.31072	0.189954	0.267239	AP1G2	protein_cc
ENSG0000(	631.7392	0.461713	0.064049	7.208797	5.64E-13	6.09E-12	TMEM67	protein_cc
ENSG0000(	631.4233	-0.83544	0.09102	-9.17864	4.37E-20	1.51E-18	MTMR7	protein_cc
ENSG0000(	631.3918	0.56612	0.055926	10.12264	4.38E-24	3.23E-22	RFC3	protein_cc
ENSG0000(	631.2901	-0.07786	0.04108	-1.89538	0.058042	0.096274	ZNF34	protein_cc
ENSG0000(	631.0144	0.237671	0.031205	7.616417	2.61E-14	3.50E-13	RCE1	protein_cc
ENSG0000(	630.9029	0.264278	0.036105	7.319623	2.49E-13	2.83E-12	MSANTD2	protein_cc
ENSG0000(	630.4704	-0.02115	0.035528	-0.59536	0.5516	0.641389	BBS5	protein_cc

ENSG0000(	630.3	-0.50863	0.06893	-7.37893	1.60E-13	1.88E-12	ADCY7	protein_cc
ENSG0000(	630.2918	-0.23914	0.08746	-2.73423	0.006253	0.013053	P2RX6	protein_cc
ENSG0000(	630.2518	0.22202	0.053229	4.171013	3.03E-05	9.67E-05	MORF4L1I	pseudoge
ENSG0000(	630.0624	0.099452	0.035017	2.840093	0.00451	0.009693	EFCAB7	protein_cc
ENSG0000(	629.8766	0.352662	0.044644	7.899506	2.80E-15	4.50E-14	SEPT7P2	pseudoge
ENSG0000(	629.8324	0.060328	0.084081	0.717495	0.473069	0.568861	PIR	protein_cc
ENSG0000(	629.4391	0.16573	0.039664	4.178363	2.94E-05	9.38E-05	TMEM187	protein_cc
ENSG0000(	629.3594	0.440229	0.07618	5.778796	7.52E-09	4.26E-08	F11R	protein_cc
ENSG0000(	629.0733	-0.27137	0.056026	-4.84365	1.27E-06	5.07E-06	SGK3	protein_cc
ENSG0000(	628.898	0.039136	0.039415	0.992911	0.320753	0.414579	ZNF615	protein_cc
ENSG0000(	628.5165	-0.14208	0.070489	-2.01559	0.043843	0.075229	PNPLA7	protein_cc
ENSG0000(	628.4568	-0.23892	0.046949	-5.08898	3.60E-07	1.56E-06	B3GNT2	protein_cc
ENSG0000(	628.1485	0.390269	0.041074	9.501588	2.07E-21	9.07E-20	MAGOHB	protein_cc
ENSG0000(	628.0714	0.025285	0.03562	0.709852	0.477796	0.573305	ZNF555	protein_cc
ENSG0000(	627.9685	0.024456	0.039972	0.611819	0.540657	0.63145	ILK	protein_cc
ENSG0000(	627.3862	0.326201	0.069716	4.678987	2.88E-06	1.08E-05	RPL3P4	pseudoge
ENSG0000(	627.252	0.196078	0.045896	4.272193	1.94E-05	6.38E-05	ZNF273	protein_cc
ENSG0000(	627.1831	0.298557	0.047566	6.276634	3.46E-10	2.42E-09	C6orf57	protein_cc
ENSG0000(	627.1629	-0.02473	0.031677	-0.78077	0.434939	0.532017	RAPGEF6	protein_cc
ENSG0000(	626.9449	0.152706	0.055472	2.752852	0.005908	0.012393	SOX15	protein_cc
ENSG0000(	626.7895	0.044825	0.042293	1.059852	0.289212	0.380618	ZNF793	protein_cc
ENSG0000(	626.4756	0.267133	0.032315	8.266522	1.38E-16	2.74E-15	SAAL1	protein_cc
ENSG0000(	626.0151	0.068606	0.033544	2.04523	0.040832	0.070671	ZNF585A	protein_cc
ENSG0000(	626.0013	-0.29933	0.098898	-3.02668	0.002473	0.005611	LRRTM1	protein_cc
ENSG0000(	625.8201	-0.74703	0.111056	-6.7266	1.74E-11	1.48E-10	ZNF536	protein_cc
ENSG0000(	625.4724	-0.02715	0.029911	-0.90768	0.364047	0.460039	MTRF1L	protein_cc
ENSG0000(	625.3933	0.217283	0.061476	3.534429	0.000409	0.001073	SDF2L1	protein_cc
ENSG0000(	625.1172	0.046287	0.033968	1.362655	0.172991	0.246923	C14orf93	protein_cc
ENSG0000(	625.1098	0.289894	0.032616	8.888164	6.21E-19	1.79E-17	C16orf91	protein_cc
ENSG0000(	625.0359	0.308928	0.036549	8.452433	2.85E-17	6.37E-16	TMEM116	protein_cc
ENSG0000(	624.8069	0.107843	0.070311	1.533806	0.125077	0.187227	FES	protein_cc
ENSG0000(	624.6609	-0.07438	0.046011	-1.61667	0.105949	0.162343	CLCN5	protein_cc
ENSG0000(	624.6052	0.124126	0.04138	2.999647	0.002703	0.006078	DBF4B	protein_cc
ENSG0000(	624.4326	0.267491	0.059722	4.478928	7.50E-06	2.64E-05	SNHG12	antisense
ENSG0000(	624.3143	-0.10634	0.036763	-2.89272	0.003819	0.008338	KIN	protein_cc
ENSG0000(	623.4443	0.068255	0.058862	1.159581	0.24622	0.332601	CALHM2	protein_cc
ENSG0000(	623.3398	-0.99401	0.118122	-8.41511	3.93E-17	8.54E-16	RGS7BP	protein_cc
ENSG0000(	623.2208	-0.21562	0.08945	-2.41054	0.015929	0.03039	RCN3	protein_cc
ENSG0000(	622.9818	0.594445	0.077579	7.662428	1.82E-14	2.52E-13	IKBIP	protein_cc
ENSG0000(	622.8495	-0.08574	0.063985	-1.33999	0.180249	0.255561	WDR52	protein_cc
ENSG0000(	622.8472	-0.17194	0.041747	-4.11871	3.81E-05	0.00012	RNASEL	protein_cc
ENSG0000(	621.8618	0.24619	0.061618	3.995428	6.46E-05	0.000195	S1PR2	protein_cc
ENSG0000(	621.8297	-0.23935	0.080616	-2.96903	0.002987	0.006661	VAV1	protein_cc
ENSG0000(	621.4457	0.26934	0.037047	7.27032	3.59E-13	3.99E-12	C5orf54	protein_cc
ENSG0000(	621.1652	-0.44307	0.116783	-3.794	0.000148	0.00042	FOLH1	protein_cc
ENSG0000(	621.0672	0.018032	0.024817	0.726622	0.467457	0.563316	CHCHD4	protein_cc
ENSG0000(	620.4712	-1.46226	0.145586	-10.0439	9.77E-24	6.76E-22	FSTL5	protein_cc
ENSG0000(	620.3872	0.352082	0.03316	10.61774	2.46E-26	2.82E-24	PCGF1	protein_cc
ENSG0000(	619.8179	0.292969	0.03797	7.71585	1.20E-14	1.72E-13	CENPQ	protein_cc
ENSG0000(	619.6492	1.135455	0.110667	10.26009	1.07E-24	8.77E-23	KIF4A	protein_cc
ENSG0000(	619.2372	0.272557	0.033252	8.196724	2.47E-16	4.71E-15	ZNF502	protein_cc
ENSG0000(	619.2186	0.376979	0.093968	4.011772	6.03E-05	0.000183	SPRED3	protein_cc
ENSG0000(	619.1545	0.291882	0.034193	8.536241	1.39E-17	3.24E-16	INO80C	protein_cc
ENSG0000(	619.0151	-0.40871	0.0764	-5.34958	8.82E-08	4.21E-07	ARHGAP6	protein_cc
ENSG0000(	618.6857	0.197761	0.045388	4.357097	1.32E-05	4.46E-05	SPATA2L	protein_cc
ENSG0000(	618.6854	0.364513	0.070785	5.149562	2.61E-07	1.16E-06	PCSK5	protein_cc
ENSG0000(	618.6518	0.031226	0.026944	1.158923	0.246487	0.332913	LIAS	protein_cc
ENSG0000(	618.5844	0.070648	0.030897	2.286588	0.02222	0.04096	RNF219	protein_cc
ENSG0000(	618.5281	0.179989	0.04017	4.480644	7.44E-06	2.63E-05	RABL2A	protein_cc

ENSG0000(	618.1199	-0.07651	0.049307	-1.55181	0.120708	0.181585	MESDC1	protein_cc
ENSG0000(	617.9446	-0.09327	0.079054	-1.17987	0.238052	0.323235	PPP1R13L	protein_cc
ENSG0000(	617.8959	-0.00827	0.04985	-0.16596	0.868186	0.905501	SLC37A1	protein_cc
ENSG0000(	617.6723	-0.6267	0.100173	-6.25618	3.95E-10	2.73E-09	UNC5C	protein_cc
ENSG0000(	617.4276	0.238231	0.032956	7.228702	4.88E-13	5.30E-12	BLOC1S5	protein_cc
ENSG0000(	616.9329	-0.48814	0.113038	-4.31834	1.57E-05	5.26E-05	FBN2	protein_cc
ENSG0000(	616.616	0.124836	0.035854	3.481825	0.000498	0.001285	DENND1B	protein_cc
ENSG0000(	616.5235	-0.05405	0.02833	-1.90795	0.056398	0.093868	C9orf85	protein_cc
ENSG0000(	615.8754	0.381565	0.096647	3.948029	7.88E-05	0.000235	BCL3	protein_cc
ENSG0000(	615.6335	-0.15021	0.026791	-5.60693	2.06E-08	1.09E-07	GALK2	protein_cc
ENSG0000(	615.158	0.136676	0.033246	4.111007	3.94E-05	0.000123	LYAR	protein_cc
ENSG0000(	615.0409	-0.24765	0.085252	-2.90491	0.003674	0.008044	PTCHD1	protein_cc
ENSG0000(	615.031	0.019799	0.035624	0.555769	0.578369	0.665422	SLC25A14	protein_cc
ENSG0000(	614.8068	0.052662	0.031788	1.65665	0.09759	0.151245	GPATCH1	protein_cc
ENSG0000(	614.7957	0.066754	0.025086	2.661062	0.007789	0.015954	TLDC1	protein_cc
ENSG0000(	614.7463	-0.38558	0.0717	-5.37761	7.55E-08	3.65E-07	GPRIN3	protein_cc
ENSG0000(	614.7378	0.189923	0.085641	2.217679	0.026577	0.048137	RBPMS	protein_cc
ENSG0000(	614.6086	0.259303	0.03275	7.917722	2.42E-15	3.91E-14	METTL4	protein_cc
ENSG0000(	614.5843	0.223416	0.074895	2.983071	0.002854	0.006386	MAMDC4	protein_cc
ENSG0000(	613.8179	0.000249	0.122366	0.002038	0.998374	0.998943	CLDN10	protein_cc
ENSG0000(	613.8079	1.284493	0.196263	6.54475	5.96E-11	4.69E-10	LGR6	protein_cc
ENSG0000(	613.7994	-0.06591	0.028719	-2.29506	0.02173	0.040126	PRMT10	protein_cc
ENSG0000(	613.1875	0.054868	0.029084	1.886564	0.059219	0.097938	MCM9	protein_cc
ENSG0000(	612.914	0.330493	0.067153	4.921527	8.59E-07	3.50E-06	TONSL	protein_cc
ENSG0000(	611.9976	0.240711	0.051812	4.645809	3.39E-06	1.26E-05	CLDN15	protein_cc
ENSG0000(	611.7366	0.121456	0.037155	3.268906	0.00108	0.002614	ZNF71	protein_cc
ENSG0000(	611.6115	-0.03612	0.039917	-0.90493	0.3655	0.461607	ZNF696	protein_cc
ENSG0000(	611.5892	0.065374	0.028453	2.297643	0.021582	0.039881	TRMT12	protein_cc
ENSG0000(	611.4574	0.054553	0.033739	1.616892	0.105902	0.162279	TAF1B	protein_cc
ENSG0000(	611.4507	-0.25495	0.060886	-4.18741	2.82E-05	9.05E-05	RNF144B	protein_cc
ENSG0000(	611.2512	0.003611	0.057461	0.062851	0.949886	0.965355	BACH2	protein_cc
ENSG0000(	610.309	-0.12901	0.084715	-1.52287	0.12779	0.190684	NFAM1	protein_cc
ENSG0000(	610.2926	0.110801	0.037259	2.973803	0.002941	0.006568	ZNF549	protein_cc
ENSG0000(	610.1612	-0.05211	0.039847	-1.30771	0.19097	0.268439	DHRS12	protein_cc
ENSG0000(	609.8037	-0.61014	0.136477	-4.47064	7.80E-06	2.74E-05	LRP2	protein_cc
ENSG0000(	609.3276	0.18387	0.046488	3.955192	7.65E-05	0.000228	C4orf33	protein_cc
ENSG0000(	608.923	-0.37232	0.062477	-5.95935	2.53E-09	1.55E-08	ST6GALN/	protein_cc
ENSG0000(	608.4943	0.208862	0.0479	4.360346	1.30E-05	4.40E-05	ALG14	protein_cc
ENSG0000(	608.4782	-1.28117	0.099587	-12.8648	7.10E-38	7.89E-35	SLIT3	protein_cc
ENSG0000(	607.8746	-0.16873	0.048378	-3.48785	0.000487	0.001259	IDNK	protein_cc
ENSG0000(	607.8316	-0.02453	0.033944	-0.72277	0.469821	0.5657	RCL1	protein_cc
ENSG0000(	607.8108	0.349241	0.032747	10.66471	1.49E-26	1.78E-24	RSBN1L-A	lincRNA
ENSG0000(	607.6393	0.061355	0.035939	1.707174	0.08779	0.138039	ZNF267	protein_cc
ENSG0000(	607.5257	-0.23597	0.103834	-2.27254	0.023054	0.042351	BMP8B	protein_cc
ENSG0000(	607.397	-0.08899	0.0523	-1.70145	0.088858	0.139478	PIGZ	protein_cc
ENSG0000(	607.3671	0.231598	0.026414	8.768054	1.82E-18	4.86E-17	ZNF354A	protein_cc
ENSG0000(	607.3302	-0.16713	0.031705	-5.27154	1.35E-07	6.27E-07	RECK	protein_cc
ENSG0000(	607.1589	0.248484	0.037882	6.559438	5.40E-11	4.28E-10	ZNF232	protein_cc
ENSG0000(	607.0649	0.129728	0.039282	3.302505	0.000958	0.002342	KIAA1841	protein_cc
ENSG0000(	607.019	-0.25954	0.044572	-5.82292	5.78E-09	3.33E-08	SLC29A3	protein_cc
ENSG0000(	606.6528	0.216098	0.035766	6.042051	1.52E-09	9.63E-09	TMEM251	protein_cc
ENSG0000(	606.4989	-0.20908	0.05425	-3.85399	0.000116	0.000336	ENOX1	protein_cc
ENSG0000(	606.4879	0.389252	0.072276	5.38562	7.22E-08	3.50E-07	FLT4	protein_cc
ENSG0000(	606.2928	0.503978	0.060691	8.304061	1.01E-16	2.05E-15	FPGT	protein_cc
ENSG0000(	606.1227	-1.22314	0.131164	-9.32525	1.11E-20	4.23E-19	PTER	protein_cc
ENSG0000(	605.7659	0.303614	0.045121	6.72892	1.71E-11	1.46E-10	ARL2BP	protein_cc
ENSG0000(	605.516	0.019829	0.054908	0.361129	0.718003	0.785249	NMRK1	protein_cc
ENSG0000(	605.2737	-0.25561	0.040694	-6.28134	3.36E-10	2.36E-09	L2HGDH	protein_cc
ENSG0000(	605.0202	-0.04274	0.03687	-1.15915	0.246397	0.332802	NAT6	protein_cc

ENSG0000	604.9204	0.050823	0.039618	1.282836	0.199549	0.278746	KIAA1586	protein_co
ENSG0000	604.8858	-0.51063	0.07039	-7.25431	4.04E-13	4.45E-12	SDK2	protein_co
ENSG0000	604.7854	-1.26283	0.134185	-9.41116	4.91E-21	1.99E-19	KCNJ4	protein_co
ENSG0000	604.7685	-0.37105	0.067636	-5.48599	4.11E-08	2.07E-07	ELOVL4	protein_co
ENSG0000	604.5437	0.085984	0.032602	2.63739	0.008355	0.016994	ATF1	protein_co
ENSG0000	604.1797	0.119087	0.049456	2.407929	0.016043	0.030589	SLC10A3	protein_co
ENSG0000	604.1712	0.169045	0.04618	3.660575	0.000252	0.000686	TIGD1	protein_co
ENSG0000	604.1291	-0.13266	0.090815	-1.46076	0.144081	0.21126	PSTPIP1	protein_co
ENSG0000	604.046	0.772493	0.062052	12.44915	1.41E-35	1.02E-32	DBF4	protein_co
ENSG0000	603.8389	-0.08882	0.094493	-0.94	0.34722	0.442707	ELFN1	protein_co
ENSG0000	603.6337	-0.08336	0.076448	-1.09043	0.275526	0.365625	NINL	protein_co
ENSG0000	603.5139	0.097189	0.022694	4.282505	1.85E-05	6.12E-05	C2orf47	protein_co
ENSG0000	603.3311	-0.12819	0.050494	-2.53882	0.011123	0.021994	PIM2	protein_co
ENSG0000	603.3195	-0.64946	0.116755	-5.56261	2.66E-08	1.38E-07	KIF6	protein_co
ENSG0000	602.9831	-0.00616	0.050343	-0.12236	0.902617	0.930866	KIAA1407	protein_co
ENSG0000	602.8658	-0.0726	0.07404	-0.98052	0.32683	0.42107	ASIC3	protein_co
ENSG0000	602.6831	-0.12224	0.031443	-3.88753	0.000101	0.000296	DCUN1D3	protein_co
ENSG0000	602.3759	0.042841	0.032822	1.305256	0.191806	0.269457	ALKBH8	protein_co
ENSG0000	602.3014	-0.2905	0.046706	-6.21976	4.98E-10	3.40E-09	LCA5	protein_co
ENSG0000	602.2906	-0.64607	0.127954	-5.04927	4.43E-07	1.90E-06	SLC39A12	protein_co
ENSG0000	602.0909	0.435355	0.090822	4.793478	1.64E-06	6.41E-06	IER5L	protein_co
ENSG0000	601.9108	0.558825	0.106246	5.259725	1.44E-07	6.66E-07	IL32	protein_co
ENSG0000	601.8855	-0.23927	0.06479	-3.69304	0.000222	0.000609	FGD3	protein_co
ENSG0000	601.7853	-0.0227	0.047936	-0.47361	0.635774	0.716334	MMACHC	protein_co
ENSG0000	601.6439	-1.3485	0.131281	-10.2718	9.44E-25	7.92E-23	KCNJ3	protein_co
ENSG0000	601.2922	0.294813	0.071418	4.127991	3.66E-05	0.000115	H2AFJ	protein_co
ENSG0000	601.2726	-0.22405	0.071327	-3.14124	0.001682	0.003937	GRIN2D	protein_co
ENSG0000	601.2017	-0.02206	0.031106	-0.70905	0.478294	0.573773	CDADC1	protein_co
ENSG0000	601.074	-1.25197	0.11691	-10.7088	9.25E-27	1.14E-24	KCTD16	protein_co
ENSG0000	601.0326	0.199008	0.048798	4.078213	4.54E-05	0.000141	NT5M	protein_co
ENSG0000	600.7827	0.571743	0.103043	5.548569	2.88E-08	1.49E-07	HCP5	sense_ove
ENSG0000	600.292	0.342959	0.114368	2.998735	0.002711	0.006094	RAB36	protein_co
ENSG0000	600.1631	-0.3041	0.069111	-4.40018	1.08E-05	3.72E-05	RP4-706A	pseudoge
ENSG0000	599.9307	-0.00274	0.023202	-0.11798	0.906085	0.933432	RDH14	protein_co
ENSG0000	599.9208	-0.05363	0.047938	-1.11875	0.263248	0.351864	DNASE1L1	protein_co
ENSG0000	599.4772	0.861768	0.122971	7.007906	2.42E-12	2.37E-11	CMYA5	protein_co
ENSG0000	599.0932	-0.40226	0.049885	-8.06374	7.40E-16	1.30E-14	PBLD	protein_co
ENSG0000	598.9361	0.351035	0.084082	4.174892	2.98E-05	9.52E-05	SPA17	protein_co
ENSG0000	598.7107	0.157277	0.076694	2.050694	0.040297	0.069849	SSH3	protein_co
ENSG0000	598.6427	-0.32998	0.058284	-5.66154	1.50E-08	8.09E-08	BSCL2	protein_co
ENSG0000	598.1611	0.381582	0.073715	5.176455	2.26E-07	1.01E-06	SERPINB8	protein_co
ENSG0000	598.1546	-0.6942	0.104595	-6.63696	3.20E-11	2.63E-10	ANKRD33I	protein_co
ENSG0000	598.0763	-0.05341	0.10538	-0.50685	0.612258	0.695731	C16orf89	protein_co
ENSG0000	597.9653	0.222854	0.042886	5.196421	2.03E-07	9.16E-07	TMEM243	protein_co
ENSG0000	597.6899	0.197573	0.104498	1.890689	0.058666	0.097148	ANKRD35	protein_co
ENSG0000	597.45	0.201637	0.054715	3.685215	0.000229	0.000627	ZNF577	protein_co
ENSG0000	596.9274	0.105974	0.040369	2.625152	0.008661	0.017553	CTC-429P	sense_intr
ENSG0000	596.9023	0.241745	0.076716	3.151151	0.001626	0.003816	TVP23A	protein_co
ENSG0000	596.6386	-0.24397	0.085506	-2.85326	0.004327	0.009341	HRK	protein_co
ENSG0000	596.3983	0.084246	0.037509	2.245994	0.024704	0.045049	SPATA7	protein_co
ENSG0000	596.3194	-0.01701	0.025347	-0.67114	0.502133	0.596054	COX10	protein_co
ENSG0000	596.0766	-0.699	0.111235	-6.28397	3.30E-10	2.32E-09	SLC45A3	protein_co
ENSG0000	595.8083	-0.10271	0.045252	-2.26966	0.023228	0.042625	RP4-773N	antisense
ENSG0000	595.7246	-0.40242	0.048434	-8.30877	9.67E-17	1.97E-15	LSM11	protein_co
ENSG0000	595.3899	0.278509	0.033487	8.317008	9.02E-17	1.85E-15	UBA6-AS1	antisense
ENSG0000	595.3392	-0.18398	0.075946	-2.42255	0.015412	0.029497	RASSF5	protein_co
ENSG0000	595.0742	0.158552	0.071832	2.20726	0.027296	0.049257	TMEM121	protein_co
ENSG0000	594.6024	0.157603	0.025004	6.303086	2.92E-10	2.07E-09	ZNF18	protein_co
ENSG0000	594.5614	0.565645	0.13345	4.238617	2.25E-05	7.33E-05	NPPA	protein_co

ENSG0000(	594.5608	-0.01667	0.043752	-0.38102	0.703188	0.77333	PLCD4	protein_cc
ENSG0000(	594.5036	0.023644	0.030379	0.778303	0.436391	0.533412	TYW5	protein_cc
ENSG0000(	594.4549	-0.26879	0.069011	-3.89494	9.82E-05	0.000287	TMC7	protein_cc
ENSG0000(	594.411	0.544349	0.102683	5.301261	1.15E-07	5.40E-07	ACE	protein_cc
ENSG0000(	594.4053	0.119023	0.023704	5.02128	5.13E-07	2.17E-06	TMEM186	protein_cc
ENSG0000(	594.3251	-0.25979	0.036776	-7.06428	1.61E-12	1.63E-11	RP11-67L:	lincRNA
ENSG0000(	594.1147	0.228478	0.043773	5.219663	1.79E-07	8.15E-07	ZFP37	protein_cc
ENSG0000(	594.0531	0.178065	0.111179	1.601604	0.109243	0.166717	AC114730	antisense
ENSG0000(	593.9437	0.2158	0.035114	6.145667	7.96E-10	5.27E-09	ZNF440	protein_cc
ENSG0000(	593.8717	-0.12646	0.031997	-3.95209	7.75E-05	0.000231	KDM4C	protein_cc
ENSG0000(	593.5891	-0.13203	0.043936	-3.00515	0.002654	0.005983	TBC1D2	protein_cc
ENSG0000(	593.3203	-0.18781	0.054755	-3.43004	0.000603	0.001533	LINC0020!	lincRNA
ENSG0000(	593.3123	0.693001	0.099419	6.970533	3.16E-12	3.04E-11	KIAA0226l	protein_cc
ENSG0000(	593.1446	-0.06078	0.038761	-1.56806	0.116868	0.176733	ERN1	protein_cc
ENSG0000(	593.0792	0.62366	0.115569	5.396429	6.80E-08	3.31E-07	FAM196A	protein_cc
ENSG0000(	593	0.405421	0.072832	5.566512	2.60E-08	1.35E-07	PRR11	protein_cc
ENSG0000(	592.8812	0.741587	0.087693	8.456607	2.75E-17	6.17E-16	CDT1	protein_cc
ENSG0000(	592.7682	0.144338	0.048867	2.953664	0.00314	0.006972	LPCAT3	protein_cc
ENSG0000(	592.464	0.0578	0.031445	1.838119	0.066045	0.10769	ZNF786	protein_cc
ENSG0000(	592.3003	0.026868	0.036888	0.728377	0.466383	0.562235	GMDS	protein_cc
ENSG0000(	592.0528	-1.08124	0.122357	-8.83678	9.85E-19	2.73E-17	RNF219-A	antisense
ENSG0000(	591.916	0.1049	0.053804	1.94966	0.051217	0.086303	HSD17B8	protein_cc
ENSG0000(	591.7198	0.115767	0.029299	3.951208	7.78E-05	0.000232	BIN3	protein_cc
ENSG0000(	591.6672	-0.89994	0.117309	-7.67154	1.70E-14	2.35E-13	BEX5	protein_cc
ENSG0000(	591.1265	0.351342	0.074621	4.708328	2.50E-06	9.48E-06	TM4SF18	protein_cc
ENSG0000(	591.0846	-0.56696	0.090427	-6.26981	3.61E-10	2.52E-09	DNAH17	protein_cc
ENSG0000(	591.0763	-0.06226	0.098798	-0.63021	0.528555	0.620222	KCNK10	protein_cc
ENSG0000(	591.003	-0.83987	0.145724	-5.76344	8.24E-09	4.63E-08	CHRM3	protein_cc
ENSG0000(	590.6068	-0.01884	0.076708	-0.24562	0.805976	0.856968	PDE7B	protein_cc
ENSG0000(	590.2673	-0.04461	0.054191	-0.82323	0.410376	0.507261	ZNF594	protein_cc
ENSG0000(	590.0914	0.05804	0.047861	1.212686	0.22525	0.308577	RAB24	protein_cc
ENSG0000(	590.0188	-0.12277	0.125717	-0.9766	0.328769	0.42321	LINC0083!	lincRNA
ENSG0000(	589.8007	0.274878	0.045235	6.076679	1.23E-09	7.90E-09	SAC3D1	protein_cc
ENSG0000(	589.6641	-0.05064	0.056449	-0.89704	0.369696	0.466047	RARG	protein_cc
ENSG0000(	589.0856	0.139153	0.033983	4.094778	4.23E-05	0.000132	ZNF569	protein_cc
ENSG0000(	588.9233	-0.50969	0.130017	-3.92014	8.85E-05	0.000261	HHIP	protein_cc
ENSG0000(	588.919	-0.10312	0.052619	-1.95971	0.05003	0.08455	LIFR-AS1	antisense
ENSG0000(	588.6392	-0.65989	0.088608	-7.44726	9.53E-14	1.16E-12	CACNA2D	protein_cc
ENSG0000(	588.5601	0.112364	0.072174	1.556855	0.119505	0.180065	PTCH2	protein_cc
ENSG0000(	588.1732	1.098154	0.239753	4.580356	4.64E-06	1.69E-05	H19	processed
ENSG0000(	588.0712	-0.07816	0.053704	-1.45533	0.145578	0.213211	ZNF519	protein_cc
ENSG0000(	587.8794	0.710396	0.068852	10.31776	5.86E-25	5.07E-23	WDR76	protein_cc
ENSG0000(	587.5993	0.512053	0.095788	5.345706	9.01E-08	4.30E-07	B3GNT5	protein_cc
ENSG0000(	587.0368	0.160582	0.046318	3.466909	0.000526	0.001353	CTB-89H1	processed
ENSG0000(	586.9582	0.027391	0.07575	0.361602	0.71765	0.78491	UBASH3B	protein_cc
ENSG0000(	586.6917	-0.20446	0.109653	-1.86466	0.06223	0.102247	USP43	protein_cc
ENSG0000(	586.6165	0.160605	0.083269	1.92874	0.053763	0.090023	CCDC146	protein_cc
ENSG0000(	586.1829	0.260348	0.045737	5.692283	1.25E-08	6.84E-08	LRRC37A3	protein_cc
ENSG0000(	586.029	-0.05104	0.045568	-1.11999	0.262716	0.35129	SCARF1	protein_cc
ENSG0000(	585.6167	0.35226	0.046243	7.617652	2.58E-14	3.47E-13	MED18	protein_cc
ENSG0000(	585.5179	-0.61704	0.073426	-8.40353	4.33E-17	9.36E-16	KB-1460A	lincRNA
ENSG0000(	585.0508	-0.33771	0.048058	-7.02724	2.11E-12	2.09E-11	RNF217	protein_cc
ENSG0000(	585.0392	0.211647	0.033776	6.266224	3.70E-10	2.58E-09	TOE1	protein_cc
ENSG0000(	584.9572	0.13192	0.049438	2.668405	0.007621	0.015638	SUV420H2	protein_cc
ENSG0000(	584.9181	0.586935	0.078913	7.437779	1.02E-13	1.24E-12	GCNT2	protein_cc
ENSG0000(	584.9054	-0.04383	0.125139	-0.35029	0.726124	0.791757	SOD3	protein_cc
ENSG0000(	584.769	0.0773	0.109516	0.705831	0.480294	0.575647	HAS2	protein_cc
ENSG0000(	584.2348	-1.19653	0.168191	-7.11412	1.13E-12	1.16E-11	KCNC2	protein_cc
ENSG0000(	584.1721	0.225517	0.03868	5.830405	5.53E-09	3.19E-08	ZNF691	protein_cc

ENSG0000(	583.9226	0.054677	0.046995	1.163442	0.24465	0.330845	CNTLN	protein_cc
ENSG0000(	583.6244	0.106646	0.058045	1.837292	0.066167	0.107875	ERVK13-1	processed
ENSG0000(	583.5868	-0.18885	0.077036	-2.45148	0.014227	0.027469	DEF6	protein_cc
ENSG0000(	583.4436	-0.47809	0.118541	-4.03315	5.50E-05	0.000168	MDGA2	protein_cc
ENSG0000(	583.4352	0.298614	0.095595	3.123738	0.001786	0.004161	ABCD2	protein_cc
ENSG0000(	583.3924	0.042824	0.052594	0.814238	0.415509	0.512503	ZCWPW1	protein_cc
ENSG0000(	583.285	-0.02399	0.061346	-0.39113	0.695697	0.766959	STX3	protein_cc
ENSG0000(	583.1923	0.713825	0.131478	5.429229	5.66E-08	2.79E-07	FAM181A	protein_cc
ENSG0000(	583.0923	-0.80102	0.114112	-7.01966	2.22E-12	2.19E-11	LDB3	protein_cc
ENSG0000(	582.6984	-0.5235	0.104501	-5.00949	5.46E-07	2.30E-06	IER3	protein_cc
ENSG0000(	582.6044	0.141994	0.037336	3.803091	0.000143	0.000406	ANAPC15	protein_cc
ENSG0000(	582.5107	-0.10141	0.090414	-1.12166	0.262008	0.350492	TNFSF10	protein_cc
ENSG0000(	582.288	0.518248	0.083058	6.239566	4.39E-10	3.02E-09	LINC0110:	lincRNA
ENSG0000(	582.266	-0.45677	0.132751	-3.44082	0.00058	0.001479	NPY	protein_cc
ENSG0000(	582.2065	-0.48714	0.142232	-3.42495	0.000615	0.00156	C1orf173	protein_cc
ENSG0000(	582.0172	-0.05348	0.03376	-1.58419	0.11315	0.171881	C11orf74	protein_cc
ENSG0000(	581.9961	-0.04548	0.029998	-1.51602	0.129514	0.192766	C9orf41	protein_cc
ENSG0000(	581.8963	-0.27767	0.071275	-3.89583	9.79E-05	0.000286	CDC42EP2	protein_cc
ENSG0000(	581.8497	0.14231	0.046758	3.043514	0.002338	0.005327	RP11-473	antisense
ENSG0000(	581.7254	-0.22955	0.038297	-5.99392	2.05E-09	1.27E-08	IBA57	protein_cc
ENSG0000(	581.6021	0.342235	0.086058	3.976784	6.99E-05	0.00021	CBX2	protein_cc
ENSG0000(	581.4144	-0.01847	0.047254	-0.3908	0.695942	0.76715	NEK3	protein_cc
ENSG0000(	581.3693	0.38646	0.080353	4.809547	1.51E-06	5.95E-06	ZDHHC1	protein_cc
ENSG0000(	580.7617	-0.26569	0.075441	-3.52176	0.000429	0.001121	BIN2	protein_cc
ENSG0000(	580.682	-0.01287	0.089627	-0.14358	0.885829	0.918704	BVES	protein_cc
ENSG0000(	580.6033	0.27724	0.029574	9.374553	6.95E-21	2.73E-19	MTERF	protein_cc
ENSG0000(	580.5003	0.09768	0.040733	2.398062	0.016482	0.031325	ZNF675	protein_cc
ENSG0000(	580.3725	-0.02662	0.0731	-0.36418	0.715726	0.783557	ZNF681	protein_cc
ENSG0000(	580.2752	0.459561	0.061535	7.468261	8.13E-14	1.00E-12	CA5B	protein_cc
ENSG0000(	580.224	0.131819	0.04006	3.29054	0.001	0.002435	ENTHD2	protein_cc
ENSG0000(	579.9301	-0.24596	0.062811	-3.91587	9.01E-05	0.000265	RP11-983	antisense
ENSG0000(	579.8243	-0.19128	0.070037	-2.73118	0.006311	0.013161	NUDT14	protein_cc
ENSG0000(	579.8138	0.163782	0.03064	5.345315	9.03E-08	4.31E-07	PSMG4	protein_cc
ENSG0000(	579.7794	-0.29291	0.038871	-7.53536	4.87E-14	6.25E-13	DNAJC27	protein_cc
ENSG0000(	579.6957	#######	0.062823	-0.00048	0.999618	0.999812	DICER1-A	antisense
ENSG0000(	579.6021	0.407701	0.05708	7.142676	9.15E-13	9.58E-12	CCNF	protein_cc
ENSG0000(	579.4798	0.585541	0.053654	10.91339	9.95E-28	1.44E-25	FANCC	protein_cc
ENSG0000(	579.0183	0.212172	0.050643	4.189554	2.80E-05	8.97E-05	GAL3ST3	protein_cc
ENSG0000(	578.9526	-0.62273	0.161978	-3.84457	0.000121	0.000348	GLRA3	protein_cc
ENSG0000(	578.9198	0.131057	0.03419	3.83315	0.000127	0.000363	NUDT15	protein_cc
ENSG0000(	578.7154	-0.14363	0.029873	-4.80792	1.53E-06	5.99E-06	R3HCC1L	protein_cc
ENSG0000(	578.3228	-0.0406	0.064377	-0.63067	0.528256	0.619942	SDCBP2	protein_cc
ENSG0000(	578.2701	0.183442	0.05076	3.613919	0.000302	0.000811	SCML1	protein_cc
ENSG0000(	578.2519	0.337991	0.068178	4.957507	7.14E-07	2.95E-06	THSD1	protein_cc
ENSG0000(	577.5211	0.202099	0.079466	2.543204	0.010984	0.021746	SPAG1	protein_cc
ENSG0000(	577.4389	0.457915	0.09896	4.62725	3.71E-06	1.37E-05	LEPREL1	protein_cc
ENSG0000(	577.2189	0.240021	0.045547	5.269743	1.37E-07	6.33E-07	CCDC102I	protein_cc
ENSG0000(	577.0774	-0.49511	0.093855	-5.27531	1.33E-07	6.16E-07	RTN4RL2	protein_cc
ENSG0000(	576.1362	-0.01803	0.054482	-0.33094	0.740687	0.804151	CRIPAK	protein_cc
ENSG0000(	576.0062	-0.44152	0.079377	-5.56234	2.66E-08	1.38E-07	EBF1	protein_cc
ENSG0000(	575.8292	-0.45877	0.098938	-4.63697	3.54E-06	1.31E-05	FBLN2	protein_cc
ENSG0000(	575.6794	-0.10626	0.030248	-3.51295	0.000443	0.001156	SETDB2	protein_cc
ENSG0000(	575.5423	0.538035	0.044918	11.97825	4.62E-33	2.07E-30	NUP37	protein_cc
ENSG0000(	575.126	0.190001	0.050845	3.736872	0.000186	0.000519	SLC26A6	protein_cc
ENSG0000(	574.9905	-0.98359	0.114916	-8.55922	1.14E-17	2.69E-16	KIAA0319	protein_cc
ENSG0000(	574.9403	0.11	0.118436	0.928772	0.353007	0.448556	DUSP5	protein_cc
ENSG0000(	574.8064	0.158699	0.085377	1.858811	0.063054	0.103459	PDE6B	protein_cc
ENSG0000(	574.6614	0.154852	0.042675	3.628611	0.000285	0.00077	NMNAT1	protein_cc
ENSG0000(	574.4891	0.894757	0.118013	7.581851	3.41E-14	4.48E-13	PCDHB7	protein_cc

ENSG0000(	574.4133	0.159928	0.041735	3.832002	0.000127	0.000365	ORAI3	protein_cc
ENSG0000(	573.3672	-0.31692	0.123211	-2.57214	0.010107	0.020164	PCDHB4	protein_cc
ENSG0000(	573.2543	-0.384	0.051822	-7.41012	1.26E-13	1.51E-12	PRKAA2	protein_cc
ENSG0000(	573.2541	0.211172	0.054541	3.871783	0.000108	0.000314	NTAN1	protein_cc
ENSG0000(	573.2396	0.003631	0.029437	0.123346	0.901833	0.930271	C4orf29	protein_cc
ENSG0000(	573.0152	-0.19512	0.064821	-3.01017	0.002611	0.005896	ZNF618	protein_cc
ENSG0000(	572.8399	0.015274	0.028014	0.545232	0.585594	0.671914	MED7	protein_cc
ENSG0000(	572.8236	-0.24427	0.064817	-3.76861	0.000164	0.000462	PTH1R	protein_cc
ENSG0000(	572.6152	-0.48456	0.067962	-7.12991	1.00E-12	1.04E-11	TAL1	protein_cc
ENSG0000(	572.31	-0.20347	0.037022	-5.49601	3.88E-08	1.96E-07	PIGP	protein_cc
ENSG0000(	572.1277	0.1805	0.027281	6.616278	3.68E-11	3.00E-10	ACTR5	protein_cc
ENSG0000(	572.0969	-0.43759	0.10667	-4.1023	4.09E-05	0.000128	PPAP2C	protein_cc
ENSG0000(	571.4433	-0.13971	0.049838	-2.80334	0.005058	0.010771	ACSL5	protein_cc
ENSG0000(	571.4144	0.338948	0.04098	8.271092	1.33E-16	2.64E-15	CTD-2037	lincRNA
ENSG0000(	571.2333	-0.16353	0.048211	-3.39204	0.000694	0.001741	HNRNPCP	pseudoge
ENSG0000(	571.2225	-0.24232	0.067484	-3.59081	0.00033	0.000881	PANK1	protein_cc
ENSG0000(	571.167	-0.24422	0.060138	-4.06105	4.89E-05	0.000151	KIF16B	protein_cc
ENSG0000(	570.9549	-0.45057	0.152519	-2.95417	0.003135	0.006961	AMER3	protein_cc
ENSG0000(	570.9546	0.270649	0.096318	2.809944	0.004955	0.010567	FAS	protein_cc
ENSG0000(	570.9182	0.086624	0.058293	1.486009	0.137277	0.202633	RP5-1085	lincRNA
ENSG0000(	570.3401	0.031606	0.031345	1.00832	0.313301	0.406711	ZMYM5	protein_cc
ENSG0000(	570.275	0.155175	0.043243	3.588444	0.000333	0.000888	C19orf44	protein_cc
ENSG0000(	570.1652	0.127314	0.053544	2.377752	0.017419	0.032886	SPSB2	protein_cc
ENSG0000(	570.1136	-0.22313	0.046944	-4.75316	2.00E-06	7.72E-06	LRRC37A1	pseudoge
ENSG0000(	569.866	-0.58655	0.104575	-5.6089	2.04E-08	1.08E-07	LINC0032(	lincRNA
ENSG0000(	569.2655	0.324671	0.056419	5.754676	8.68E-09	4.86E-08	RP1-152L	pseudoge
ENSG0000(	569.0238	0.011539	0.046164	0.249957	0.80262	0.854173	RP11-673	pseudoge
ENSG0000(	568.98	0.179565	0.030661	5.856382	4.73E-09	2.76E-08	TTI2	protein_cc
ENSG0000(	568.8108	0.219775	0.02845	7.72484	1.12E-14	1.61E-13	ERCC8	protein_cc
ENSG0000(	568.7663	0.781175	0.093957	8.314203	9.24E-17	1.89E-15	RPL18AP3	pseudoge
ENSG0000(	568.6976	-0.00686	0.041371	-0.16577	0.868338	0.905513	ATP7A	protein_cc
ENSG0000(	568.2582	0.659835	0.09028	7.308775	2.70E-13	3.06E-12	MYO5C	protein_cc
ENSG0000(	568.1456	0.143363	0.041087	3.489299	0.000484	0.001253	GNG10	protein_cc
ENSG0000(	567.8803	-1.68918	0.176735	-9.55774	1.20E-21	5.49E-20	CACNG3	protein_cc
ENSG0000(	567.3876	0.155639	0.044519	3.496036	0.000472	0.001224	ZFP82	protein_cc
ENSG0000(	567.3812	0.170017	0.039051	4.353726	1.34E-05	4.52E-05	BCL10	protein_cc
ENSG0000(	567.0837	-0.67238	0.127844	-5.25939	1.45E-07	6.67E-07	AFF2	protein_cc
ENSG0000(	566.4888	0.086428	0.052262	1.653763	0.098176	0.152061	ABHD15	protein_cc
ENSG0000(	566.4059	-0.26023	0.066267	-3.92708	8.60E-05	0.000255	IRAK3	protein_cc
ENSG0000(	566.1799	-0.15146	0.082189	-1.8428	0.065358	0.106709	MEX3B	protein_cc
ENSG0000(	566.1025	-0.47104	0.07708	-6.11107	9.90E-10	6.46E-09	KLF4	protein_cc
ENSG0000(	566.0206	-0.03771	0.131957	-0.28578	0.775049	0.831958	HES5	protein_cc
ENSG0000(	565.687	0.161801	0.056127	2.88277	0.003942	0.008587	TTF2	protein_cc
ENSG0000(	565.533	0.141231	0.033912	4.1647	3.12E-05	9.93E-05	C2orf76	protein_cc
ENSG0000(	565.5038	-0.46351	0.079352	-5.84113	5.18E-09	3.01E-08	SLC16A7	protein_cc
ENSG0000(	565.2983	-0.22887	0.05516	-4.14915	3.34E-05	0.000106	C1orf51	protein_cc
ENSG0000(	565.2352	0.360191	0.039101	9.211746	3.21E-20	1.13E-18	RP11-390	antisense
ENSG0000(	565.2337	0.030566	0.051196	0.597031	0.550486	0.640385	KCTD9	protein_cc
ENSG0000(	565.073	-0.25817	0.068348	-3.77728	0.000159	0.000447	KCNQ1	protein_cc
ENSG0000(	564.3732	0.263546	0.040032	6.583428	4.60E-11	3.69E-10	HSCB	protein_cc
ENSG0000(	564.3159	0.089827	0.028312	3.172742	0.00151	0.003565	ALKBH1	protein_cc
ENSG0000(	564.1428	0.144175	0.03667	3.93166	8.44E-05	0.00025	ZNF607	protein_cc
ENSG0000(	563.8283	-0.36511	0.087669	-4.16467	3.12E-05	9.93E-05	DNAH7	protein_cc
ENSG0000(	563.6882	-0.12713	0.043761	-2.90501	0.003672	0.008041	FRAT2	protein_cc
ENSG0000(	563.5411	-0.18417	0.075197	-2.44919	0.014318	0.027633	PDE3A	protein_cc
ENSG0000(	563.236	0.196564	0.04661	4.217249	2.47E-05	8.00E-05	WARS2	protein_cc
ENSG0000(	563.1307	0.183869	0.054627	3.365889	0.000763	0.0019	RAB33B	protein_cc
ENSG0000(	562.6431	0.314784	0.043493	7.237593	4.57E-13	4.98E-12	SPATA33	protein_cc
ENSG0000(	562.6088	0.101109	0.029788	3.39433	0.000688	0.001728	C1orf56	protein_cc

ENSG0000(	562.4337	-0.06854	0.029411	-2.33055	0.019777	0.036847	SLC25A51	protein_cc
ENSG0000(	562.3452	0.224582	0.063499	3.536766	0.000405	0.001064	CASP7	protein_cc
ENSG0000(	562.3135	0.205118	0.053866	3.807932	0.00014	0.000399	MYL5	protein_cc
ENSG0000(	562.2244	0.362706	0.098852	3.669177	0.000243	0.000665	EPHA2	protein_cc
ENSG0000(	561.5997	0.272612	0.08597	3.171022	0.001519	0.003584	HTATIP2	protein_cc
ENSG0000(	561.4886	0.283667	0.09923	2.858669	0.004254	0.0092	CMPK2	protein_cc
ENSG0000(	561.2899	-0.50142	0.052994	-9.46188	3.02E-21	1.28E-19	PARK2	protein_cc
ENSG0000(	561.2038	0.138706	0.036741	3.77523	0.00016	0.000451	FAM207A	protein_cc
ENSG0000(	561.1877	-0.20201	0.048569	-4.15926	3.19E-05	0.000101	RP11-774	lincRNA
ENSG0000(	561.0649	-0.45955	0.113006	-4.06658	4.77E-05	0.000147	GJC2	protein_cc
ENSG0000(	560.9973	0.388245	0.077693	4.997197	5.82E-07	2.44E-06	ZDHHC12	protein_cc
ENSG0000(	560.7686	0.255114	0.076907	3.31718	0.000909	0.002232	TMCO4	protein_cc
ENSG0000(	560.6734	0.162966	0.055401	2.94155	0.003266	0.007225	NBPF15	protein_cc
ENSG0000(	560.3461	-0.33717	0.114321	-2.94929	0.003185	0.007061	CDS1	protein_cc
ENSG0000(	560.2641	0.170634	0.044679	3.819088	0.000134	0.000383	ZSCAN12	protein_cc
ENSG0000(	560.1733	0.686309	0.063237	10.85295	1.93E-27	2.67E-25	FBXO5	protein_cc
ENSG0000(	560.1098	-0.06508	0.031884	-2.04119	0.041232	0.071269	PRDM15	protein_cc
ENSG0000(	559.9835	0.468543	0.126811	3.694825	0.00022	0.000605	GAPDHP1	pseudoge
ENSG0000(	559.9259	0.267204	0.045679	5.849656	4.93E-09	2.87E-08	NUDT19	protein_cc
ENSG0000(	559.8024	0.528357	0.079028	6.685734	2.30E-11	1.93E-10	NMI	protein_cc
ENSG0000(	559.5941	-0.19756	0.059528	-3.31868	0.000904	0.002222	ZCCHC18	protein_cc
ENSG0000(	559.5001	-1.18856	0.101996	-11.653	2.21E-31	6.55E-29	LINC0008	lincRNA
ENSG0000(	559.3888	1.12387	0.112006	10.03402	1.08E-23	7.40E-22	BUB1	protein_cc
ENSG0000(	559.2936	0.087407	0.039603	2.207082	0.027308	0.049274	TMEM53	protein_cc
ENSG0000(	559.2549	-0.10351	0.047025	-2.20121	0.027721	0.049956	SRSF12	protein_cc
ENSG0000(	559.0502	0.419431	0.05158	8.131615	4.24E-16	7.79E-15	SOX9-AS1	processed
ENSG0000(	559.0048	-0.21053	0.099067	-2.12514	0.033575	0.059338	SYT14	protein_cc
ENSG0000(	558.9992	0.275616	0.081051	3.400514	0.000673	0.001693	SLCO2A1	protein_cc
ENSG0000(	558.7249	0.086493	0.045587	1.897308	0.057787	0.095902	INVS	protein_cc
ENSG0000(	558.572	0.047954	0.068571	0.69934	0.48434	0.579386	RP11-390	antisense
ENSG0000(	558.4311	-0.21638	0.125407	-1.72539	0.084458	0.133366	MIR219-2	lincRNA
ENSG0000(	558.344	0.058032	0.064339	0.901978	0.367069	0.463189	MIS18BP1	protein_cc
ENSG0000(	558.2936	0.103907	0.055034	1.888066	0.059017	0.097659	TRPM4	protein_cc
ENSG0000(	558.1237	0.258121	0.031066	8.308923	9.66E-17	1.97E-15	ST7L	protein_cc
ENSG0000(	558.077	-0.33241	0.199312	-1.66779	0.095358	0.148278	IGHG1	IG_C_gene
ENSG0000(	558.041	-0.09093	0.108724	-0.8363	0.402984	0.499813	RSPH1	protein_cc
ENSG0000(	557.8524	0.126397	0.045996	2.748006	0.005996	0.012558	SAMD10	protein_cc
ENSG0000(	557.6903	0.703336	0.079754	8.818838	1.16E-18	3.18E-17	FANCD2	protein_cc
ENSG0000(	557.4677	0.014737	0.074955	0.196611	0.844132	0.886899	TRIM38	protein_cc
ENSG0000(	557.3776	-0.12266	0.072253	-1.69763	0.089577	0.140456	FRAT1	protein_cc
ENSG0000(	557.2509	0.795683	0.08892	8.948298	3.61E-19	1.08E-17	ARHGAP1	protein_cc
ENSG0000(	556.9553	-0.1688	0.051015	-3.30888	0.000937	0.002294	DCAF4	protein_cc
ENSG0000(	556.928	-0.10174	0.04479	-2.2716	0.023111	0.042435	SLC25A30	protein_cc
ENSG0000(	556.7812	-0.28561	0.071298	-4.00584	6.18E-05	0.000187	PRX	protein_cc
ENSG0000(	556.7533	-0.36417	0.128761	-2.82826	0.00468	0.010022	HPR	protein_cc
ENSG0000(	556.375	0.085341	0.034503	2.473423	0.013383	0.025977	SFMBT1	protein_cc
ENSG0000(	556.3629	-0.36104	0.061053	-5.91348	3.35E-09	2.01E-08	GDPD5	protein_cc
ENSG0000(	556.2629	0.009063	0.043375	0.208953	0.834485	0.879481	C8orf37	protein_cc
ENSG0000(	556.2591	0.364306	0.042072	8.659109	4.75E-18	1.20E-16	CMC1	protein_cc
ENSG0000(	555.9982	-0.14655	0.035394	-4.14056	3.46E-05	0.000109	AUH	protein_cc
ENSG0000(	555.9283	0.050706	0.085459	0.593343	0.552952	0.642688	FLRT3	protein_cc
ENSG0000(	555.6794	-0.22957	0.092383	-2.48493	0.012958	0.025221	LILRB1	protein_cc
ENSG0000(	555.6652	0.122677	0.043646	2.810735	0.004943	0.010545	RP11-658	antisense
ENSG0000(	555.5353	-0.76057	0.087558	-8.68637	3.74E-18	9.56E-17	KCNA2	protein_cc
ENSG0000(	555.4567	0.043171	0.026858	1.607388	0.107969	0.165024	GRK4	protein_cc
ENSG0000(	555.2496	1.312541	0.15731	8.343646	7.20E-17	1.50E-15	TMSB4XP8	pseudoge
ENSG0000(	554.7132	0.087349	0.054229	1.610727	0.107239	0.164097	LINC0068(	processed
ENSG0000(	554.5777	0.045475	0.041854	1.086509	0.277254	0.367467	ZNF821	protein_cc
ENSG0000(	553.8705	-1.0897	0.120384	-9.05186	1.41E-19	4.49E-18	SLIT2	protein_cc

ENSG0000(	553.4109	0.059116	0.06932	0.852804	0.393768	0.490335	VANGL1	protein_cc
ENSG0000(	553.376	0.00511	0.080326	0.063611	0.94928	0.965026	SLC44A3	protein_cc
ENSG0000(	552.9925	0.040375	0.034843	1.158771	0.24655	0.332954	ANKMY1	protein_cc
ENSG0000(	552.9367	0.12807	0.026969	4.748706	2.05E-06	7.87E-06	NDUFAF7	protein_cc
ENSG0000(	552.9005	0.565666	0.094325	5.996995	2.01E-09	1.25E-08	C9orf64	protein_cc
ENSG0000(	552.7494	-0.41959	0.088416	-4.74562	2.08E-06	7.99E-06	AC018647	lincRNA
ENSG0000(	552.5981	-0.16689	0.07076	-2.35851	0.018349	0.034458	BCAS4	protein_cc
ENSG0000(	552.1292	0.80709	0.084086	9.598407	8.12E-22	3.77E-20	KIF15	protein_cc
ENSG0000(	552.0391	-0.14993	0.094912	-1.57963	0.114191	0.17324	ITGAL	protein_cc
ENSG0000(	551.9736	0.192676	0.049424	3.898401	9.68E-05	0.000284	C5	protein_cc
ENSG0000(	551.9636	0.306098	0.069074	4.431464	9.36E-06	3.25E-05	PQLC3	protein_cc
ENSG0000(	551.6412	-0.34269	0.060027	-5.70893	1.14E-08	6.25E-08	MITF	protein_cc
ENSG0000(	551.5102	0.299144	0.062207	4.808872	1.52E-06	5.96E-06	CREB3L4	protein_cc
ENSG0000(	551.3589	0.200666	0.03037	6.607357	3.91E-11	3.18E-10	FAM86A	protein_cc
ENSG0000(	551.1495	0.119504	0.035415	3.374399	0.00074	0.001846	HSPBAP1	protein_cc
ENSG0000(	550.8601	-0.30596	0.068502	-4.46644	7.95E-06	2.79E-05	TINAGL1	protein_cc
ENSG0000(	550.7923	-0.13284	0.064138	-2.07113	0.038347	0.066863	ASPHD2	protein_cc
ENSG0000(	550.6978	0.269442	0.033922	7.942935	1.97E-15	3.25E-14	C5orf28	protein_cc
ENSG0000(	550.5841	0.243095	0.032708	7.432292	1.07E-13	1.29E-12	THG1L	protein_cc
ENSG0000(	550.3745	0.401534	0.077269	5.196545	2.03E-07	9.16E-07	ZNF713	protein_cc
ENSG0000(	550.2397	0.084804	0.060307	1.406201	0.159664	0.23067	NAAA	protein_cc
ENSG0000(	550.1732	-1.08705	0.142286	-7.63986	2.17E-14	2.96E-13	KCNT1	protein_cc
ENSG0000(	549.9301	-0.82096	0.103975	-7.89571	2.89E-15	4.62E-14	EPB41L4B	protein_cc
ENSG0000(	549.8091	-0.05495	0.071641	-0.76704	0.44306	0.539874	ABCA7	protein_cc
ENSG0000(	549.5487	-0.12871	0.038934	-3.30582	0.000947	0.002317	ZFAT	protein_cc
ENSG0000(	549.5399	0.012043	0.054135	0.22247	0.823948	0.870945	OSCP1	protein_cc
ENSG0000(	549.5164	0.239854	0.049141	4.880949	1.06E-06	4.25E-06	U2AF1L4	protein_cc
ENSG0000(	549.3049	-0.46347	0.090998	-5.09318	3.52E-07	1.53E-06	C2CD4C	protein_cc
ENSG0000(	549.2076	0.036146	0.081421	0.443942	0.657085	0.734552	DOK3	protein_cc
ENSG0000(	549.1887	0.686616	0.07475	9.185527	4.10E-20	1.42E-18	CKS1B	protein_cc
ENSG0000(	548.7233	0.000761	0.046889	0.016231	0.98705	0.991075	KIAA1731	protein_cc
ENSG0000(	548.6476	0.736457	0.092412	7.96931	1.60E-15	2.67E-14	PPIC	protein_cc
ENSG0000(	548.2062	0.31116	0.057353	5.425392	5.78E-08	2.85E-07	KIAA0895	protein_cc
ENSG0000(	548.0318	0.198626	0.050375	3.942981	8.05E-05	0.000239	DHRS4L2	protein_cc
ENSG0000(	547.7708	0.550479	0.122247	4.503026	6.70E-06	2.38E-05	SDC1	protein_cc
ENSG0000(	547.6976	-0.02646	0.044915	-0.58907	0.555816	0.645288	AIFM2	protein_cc
ENSG0000(	547.2927	0.117766	0.029128	4.043034	5.28E-05	0.000162	THAP6	protein_cc
ENSG0000(	547.0335	-0.18151	0.046578	-3.89695	9.74E-05	0.000285	SUPT3H	protein_cc
ENSG0000(	546.9561	0.091305	0.030685	2.97556	0.002925	0.006533	COQ6	protein_cc
ENSG0000(	546.9073	-1.56205	0.156018	-10.012	1.35E-23	9.09E-22	C11orf87	protein_cc
ENSG0000(	546.8101	0.111667	0.038218	2.921823	0.00348	0.007658	ZNF501	protein_cc
ENSG0000(	546.76	0.024143	0.029588	0.815976	0.414514	0.511506	ZNF16	protein_cc
ENSG0000(	546.7599	-0.07456	0.052801	-1.4121	0.157922	0.228434	RP11-18I1	processed
ENSG0000(	546.7463	-0.41397	0.072023	-5.74777	9.04E-09	5.05E-08	SNX32	protein_cc
ENSG0000(	546.7366	0.075589	0.028442	2.657704	0.007867	0.016096	ZNF287	protein_cc
ENSG0000(	546.67	-0.23288	0.076999	-3.02442	0.002491	0.005649	VWCE	protein_cc
ENSG0000(	546.6165	0.03117	0.091592	0.340317	0.733618	0.798151	HUNK	protein_cc
ENSG0000(	546.5539	0.20427	0.028602	7.141769	9.21E-13	9.64E-12	DUS4L	protein_cc
ENSG0000(	546.5021	-0.12361	0.071044	-1.73993	0.081871	0.129749	ITPR3	protein_cc
ENSG0000(	546.2332	0.226481	0.038508	5.881402	4.07E-09	2.40E-08	MSTO1	protein_cc
ENSG0000(	545.2389	0.076502	0.070673	1.082469	0.279044	0.369458	RGS17	protein_cc
ENSG0000(	544.9197	0.09353	0.030097	3.10761	0.001886	0.004372	SPATA5L1	protein_cc
ENSG0000(	544.8458	0.177469	0.017498	10.14218	3.59E-24	2.67E-22	RBM45	protein_cc
ENSG0000(	544.7583	0.072251	0.034967	2.066243	0.038806	0.067543	PECR	protein_cc
ENSG0000(	544.5822	0.032291	0.027304	1.182645	0.23695	0.321935	NFXL1	protein_cc
ENSG0000(	544.1907	-0.07356	0.107544	-0.68397	0.493992	0.588378	AC244102	protein_cc
ENSG0000(	544.0967	0.204902	0.118917	1.723067	0.084876	0.13396	THNSL2	protein_cc
ENSG0000(	544.0775	0.149141	0.036111	4.130036	3.63E-05	0.000114	ZNF616	protein_cc
ENSG0000(	543.9802	-0.04221	0.102117	-0.41332	0.679369	0.753198	SOX1	protein_cc

ENSG0000(	543.8263	-0.152	0.051477	-2.95282	0.003149	0.006988	AK9	protein_cc
ENSG0000(	542.8562	-0.70117	0.105284	-6.65981	2.74E-11	2.28E-10	PIEZO2	protein_cc
ENSG0000(	542.8385	-0.49938	0.16076	-3.10638	0.001894	0.004389	PCDHGB1	protein_cc
ENSG0000(	542.8074	0.418813	0.051462	8.138262	4.01E-16	7.39E-15	GEMIN7	protein_cc
ENSG0000(	542.5845	0.139306	0.040682	3.424278	0.000616	0.001563	ZNF660	protein_cc
ENSG0000(	542.5836	0.13512	0.100948	1.338508	0.180731	0.256112	ABCA5	protein_cc
ENSG0000(	542.5784	-0.22707	0.122517	-1.8534	0.063825	0.104559	MCHR1	protein_cc
ENSG0000(	542.55	0.143765	0.027921	5.14902	2.62E-07	1.16E-06	EXOSC3	protein_cc
ENSG0000(	542.0285	-0.38406	0.073687	-5.21201	1.87E-07	8.47E-07	CACNA1D	protein_cc
ENSG0000(	542.0043	-0.16225	0.06589	-2.46249	0.013798	0.026711	ANKFN1	protein_cc
ENSG0000(	541.8034	0.283241	0.034875	8.121699	4.60E-16	8.39E-15	RWDD4	protein_cc
ENSG0000(	541.4049	0.170491	0.056184	3.034532	0.002409	0.005478	TEX30	protein_cc
ENSG0000(	541.0108	-0.40345	0.072183	-5.58931	2.28E-08	1.20E-07	PRTG	protein_cc
ENSG0000(	540.8521	0.20104	0.059415	3.38366	0.000715	0.001791	C14orf80	protein_cc
ENSG0000(	540.5974	-0.14818	0.088703	-1.6705	0.09482	0.147525	SULT1A1	protein_cc
ENSG0000(	540.5533	0.134949	0.051371	2.626976	0.008615	0.017468	DHRS4	protein_cc
ENSG0000(	540.364	-0.41327	0.059887	-6.9008	5.17E-12	4.79E-11	PDE3B	protein_cc
ENSG0000(	540.1948	0.118757	0.076143	1.559664	0.118839	0.179299	MIR22HG	lincRNA
ENSG0000(	539.9541	0.111573	0.115189	0.968611	0.332739	0.427291	ST14	protein_cc
ENSG0000(	539.8358	0.27019	0.135007	2.001305	0.04536	0.077501	COL9A1	protein_cc
ENSG0000(	539.2952	0.350449	0.084326	4.155888	3.24E-05	0.000103	EPS8L2	protein_cc
ENSG0000(	539.1683	-0.90071	0.106899	-8.42581	3.58E-17	7.85E-16	RASGEF1A	protein_cc
ENSG0000(	539.0429	0.277413	0.055672	4.982993	6.26E-07	2.61E-06	C2orf81	protein_cc
ENSG0000(	538.8207	0.001114	0.078545	0.014183	0.988684	0.992182	BTK	protein_cc
ENSG0000(	538.5242	-0.29861	0.093689	-3.18727	0.001436	0.003403	LRRC3B	protein_cc
ENSG0000(	538.142	1.218345	0.124481	9.787368	1.28E-22	6.91E-21	CCNB2	protein_cc
ENSG0000(	538.122	0.175269	0.030038	5.834985	5.38E-09	3.11E-08	PTCD1	protein_cc
ENSG0000(	538.0657	-0.22975	0.044602	-5.15121	2.59E-07	1.15E-06	MBLAC2	protein_cc
ENSG0000(	537.9813	0.12461	0.037681	3.306987	0.000943	0.002308	ATG4A	protein_cc
ENSG0000(	537.9331	0.058119	0.042929	1.353834	0.175789	0.250335	HYI	protein_cc
ENSG0000(	537.5398	-0.50227	0.062948	-7.97911	1.47E-15	2.47E-14	LEPR	protein_cc
ENSG0000(	537.1398	0.073899	0.024301	3.041001	0.002358	0.005369	HELQ	protein_cc
ENSG0000(	537.1389	0.038662	0.026493	1.459336	0.144473	0.211773	LINS	protein_cc
ENSG0000(	536.9818	-0.00383	0.03063	-0.12496	0.900552	0.92946	ALG12	protein_cc
ENSG0000(	536.9801	-0.05053	0.048198	-1.04835	0.294479	0.386289	ZBED5-AS	antisense
ENSG0000(	536.7707	1.217401	0.113662	10.71074	9.06E-27	1.12E-24	CENPU	protein_cc
ENSG0000(	536.4564	0.169397	0.040771	4.154846	3.26E-05	0.000103	ING2	protein_cc
ENSG0000(	536.4102	-0.10661	0.044407	-2.4008	0.016359	0.031126	SMIM8	protein_cc
ENSG0000(	536.3451	0.198695	0.069858	2.844251	0.004452	0.009585	GALM	protein_cc
ENSG0000(	536.2661	0.281312	0.087235	3.224759	0.001261	0.003016	SIM2	protein_cc
ENSG0000(	536.2038	0.122823	0.032671	3.759407	0.00017	0.000478	CDPF1	protein_cc
ENSG0000(	535.8639	0.311183	0.031424	9.902707	4.05E-23	2.43E-21	ZNF557	protein_cc
ENSG0000(	535.617	0.475154	0.059869	7.936619	2.08E-15	3.41E-14	MCM8	protein_cc
ENSG0000(	535.3877	-0.01627	0.049497	-0.32877	0.742326	0.8056	TTC32	protein_cc
ENSG0000(	535.1841	0.423064	0.061254	6.906664	4.96E-12	4.62E-11	CCDC125	protein_cc
ENSG0000(	534.8158	-0.05192	0.05504	-0.94327	0.345541	0.440976	PRDM5	protein_cc
ENSG0000(	534.3567	0.010349	0.024056	0.430214	0.66704	0.743015	OXNAD1	protein_cc
ENSG0000(	534.2618	0.532548	0.103919	5.12465	2.98E-07	1.31E-06	RIN1	protein_cc
ENSG0000(	534.2228	0.085768	0.069258	1.238372	0.215578	0.297546	FGR	protein_cc
ENSG0000(	534.0807	0.099553	0.049885	1.995624	0.045975	0.078451	EOGT	protein_cc
ENSG0000(	534.0036	-0.64178	0.121776	-5.27014	1.36E-07	6.32E-07	PAK7	protein_cc
ENSG0000(	533.9071	0.422493	0.101119	4.178163	2.94E-05	9.39E-05	PNPLA4	protein_cc
ENSG0000(	533.5003	-0.37486	0.046201	-8.11358	4.91E-16	8.93E-15	ERCC6	protein_cc
ENSG0000(	533.3079	0.421139	0.053321	7.898231	2.83E-15	4.54E-14	BOLA3	protein_cc
ENSG0000(	533.0069	-0.9471	0.121894	-7.76987	7.86E-15	1.17E-13	RAB3B	protein_cc
ENSG0000(	532.7444	-0.19666	0.098046	-2.00574	0.044884	0.076781	HSD17B6	protein_cc
ENSG0000(	532.5336	0.476275	0.063283	7.526128	5.23E-14	6.68E-13	ZWILCH	protein_cc
ENSG0000(	532.4769	-0.36821	0.066779	-5.51385	3.51E-08	1.79E-07	PKDCC	protein_cc
ENSG0000(	531.7226	0.127483	0.023181	5.499513	3.81E-08	1.93E-07	THAP1	protein_cc

ENSG0000	531.247	0.235316	0.030171	7.799481	6.22E-15	9.41E-14	ZNF14	protein_co
ENSG0000	531.02	-0.41807	0.074825	-5.5873	2.31E-08	1.21E-07	TAGAP	protein_co
ENSG0000	530.9665	0.209938	0.083254	2.521644	0.011681	0.022983	PLA2G4A	protein_co
ENSG0000	530.9036	0.234005	0.047529	4.923433	8.50E-07	3.47E-06	ZNF524	protein_co
ENSG0000	530.7371	-0.5558	0.087736	-6.33496	2.37E-10	1.70E-09	RP11-588	sense_ove
ENSG0000	530.4634	0.157831	0.071998	2.192178	0.028367	0.05101	RELB	protein_co
ENSG0000	530.3523	0.138633	0.026454	5.240546	1.60E-07	7.34E-07	ZSCAN21	protein_co
ENSG0000	530.2793	0.214516	0.029176	7.352466	1.95E-13	2.25E-12	ELAC1	protein_co
ENSG0000	529.8552	0.204429	0.047795	4.277199	1.89E-05	6.26E-05	C1orf159	protein_co
ENSG0000	529.8398	0.088069	0.041821	2.105859	0.035217	0.061984	RP11-169	antisense
ENSG0000	529.5707	-0.79159	0.091038	-8.69515	3.46E-18	8.90E-17	PANX2	protein_co
ENSG0000	528.9638	-0.31506	0.068442	-4.60324	4.16E-06	1.53E-05	CD55	protein_co
ENSG0000	528.819	0.081677	0.04828	1.69174	0.090696	0.14193	RP11-46C	antisense
ENSG0000	528.7326	-0.3923	0.065736	-5.96777	2.41E-09	1.48E-08	CCDC69	protein_co
ENSG0000	528.6169	0.198971	0.042605	4.67018	3.01E-06	1.13E-05	IMMP2L	protein_co
ENSG0000	528.3146	-0.16931	0.106512	-1.58956	0.111935	0.170282	NID2	protein_co
ENSG0000	528.2878	0.600464	0.048758	12.31509	7.51E-35	4.61E-32	PMS2P1	pseudoge
ENSG0000	528.0624	-0.11311	0.044674	-2.53194	0.011343	0.022382	ANXA9	protein_co
ENSG0000	528.013	-0.68305	0.100962	-6.76538	1.33E-11	1.16E-10	ITPKA	protein_co
ENSG0000	528.0018	-0.33057	0.070237	-4.70648	2.52E-06	9.56E-06	FRG1B	protein_co
ENSG0000	527.78	-0.10967	0.049743	-2.20474	0.027473	0.04954	HMGB1P5	pseudoge
ENSG0000	527.5662	0.028216	0.099586	0.28333	0.776924	0.833523	RBM47	protein_co
ENSG0000	526.9915	0.033551	0.097059	0.345674	0.729588	0.794712	SPHK1	protein_co
ENSG0000	526.9171	0.166654	0.048738	3.419375	0.000628	0.001589	ZDHHC13	protein_co
ENSG0000	526.2397	-0.18757	0.043712	-4.29094	1.78E-05	5.90E-05	ANKRD16	protein_co
ENSG0000	526.0483	0.005919	0.049711	0.119064	0.905225	0.932902	ZNF234	protein_co
ENSG0000	525.829	0.016527	0.029206	0.565899	0.571462	0.659024	C12orf66	protein_co
ENSG0000	525.6442	0.551814	0.05372	10.27201	9.42E-25	7.92E-23	FBXO4	protein_co
ENSG0000	525.6258	0.353914	0.05343	6.623903	3.50E-11	2.86E-10	C19orf57	protein_co
ENSG0000	525.616	-0.13032	0.037351	-3.48909	0.000485	0.001254	TMLHE	protein_co
ENSG0000	525.572	0.624	0.063076	9.892811	4.47E-23	2.67E-21	ITGB3BP	protein_co
ENSG0000	525.4823	0.245857	0.037679	6.525099	6.80E-11	5.32E-10	MOSPD1	protein_co
ENSG0000	525.3507	-0.09467	0.074139	-1.27691	0.201633	0.281254	STXBP2	protein_co
ENSG0000	525.1865	0.108353	0.041098	2.636461	0.008378	0.017038	ZNF224	protein_co
ENSG0000	525.0701	0.019645	0.037562	0.523007	0.60097	0.685632	FAM161A	protein_co
ENSG0000	524.9857	0.085668	0.047076	1.819798	0.06879	0.111605	TMEM177	protein_co
ENSG0000	524.9186	0.429657	0.041244	10.41751	2.06E-25	1.95E-23	RP9	protein_co
ENSG0000	524.6308	0.073987	0.032337	2.287974	0.022139	0.040825	ZNF343	protein_co
ENSG0000	524.5951	1.162004	0.104464	11.12349	9.64E-29	1.75E-26	CCNA2	protein_co
ENSG0000	524.314	0.021053	0.033754	0.623701	0.532824	0.624287	EEF1E1	protein_co
ENSG0000	524.1153	-0.53477	0.0825	-6.48207	9.05E-11	6.95E-10	PLD1	protein_co
ENSG0000	523.9114	-0.60108	0.100364	-5.98903	2.11E-09	1.31E-08	PLCH1	protein_co
ENSG0000	523.8018	-0.34571	0.051253	-6.74508	1.53E-11	1.32E-10	AC096772	lincRNA
ENSG0000	522.8492	0.188638	0.044614	4.228225	2.36E-05	7.65E-05	NBPF10	protein_co
ENSG0000	522.8372	0.273237	0.082133	3.326752	0.000879	0.002164	GLI2	protein_co
ENSG0000	522.3627	0.27557	0.040697	6.7712	1.28E-11	1.11E-10	TMEM99	protein_co
ENSG0000	522.2615	0.519038	0.096739	5.365349	8.08E-08	3.88E-07	LYPD6	protein_co
ENSG0000	521.8926	-0.16904	0.058399	-2.89459	0.003797	0.008292	FGD5	protein_co
ENSG0000	521.7827	-1.22512	0.161391	-7.591	3.17E-14	4.20E-13	CAMK1G	protein_co
ENSG0000	521.6539	-0.39987	0.057748	-6.92432	4.38E-12	4.12E-11	FGD6	protein_co
ENSG0000	521.2612	0.937449	0.094358	9.934998	2.93E-23	1.82E-21	UBE2T	protein_co
ENSG0000	521.0594	1.343049	0.130921	10.25844	1.08E-24	8.88E-23	PKIB	protein_co
ENSG0000	520.9666	-0.30411	0.073029	-4.16419	3.12E-05	9.94E-05	TMC8	protein_co
ENSG0000	520.2458	-0.48827	0.146717	-3.32799	0.000875	0.002155	TMEM125	protein_co
ENSG0000	519.694	0.305276	0.043435	7.028388	2.09E-12	2.07E-11	ZNHIT2	protein_co
ENSG0000	519.6358	-0.29944	0.086947	-3.44393	0.000573	0.001463	MLXIPL	protein_co
ENSG0000	519.5742	-0.01208	0.097411	-0.12401	0.901309	0.93003	RP11-105	lincRNA
ENSG0000	519.5419	0.292199	0.024698	11.831	2.70E-32	9.56E-30	ZNF200	protein_co
ENSG0000	519.1445	0.270415	0.032893	8.221038	2.02E-16	3.90E-15	OXSM	protein_co

ENSG0000(	519.0468	0.222914	0.03389	6.577587	4.78E-11	3.82E-10	GEMIN2	protein_cc
ENSG0000(	518.9978	0.079802	0.022581	3.5341	0.000409	0.001074	NAIF1	protein_cc
ENSG0000(	518.6426	0.203459	0.035835	5.677626	1.37E-08	7.42E-08	SHQ1	protein_cc
ENSG0000(	518.2779	-0.08895	0.080022	-1.11157	0.266321	0.355259	YJEFN3	protein_cc
ENSG0000(	517.7926	0.07125	0.059197	1.203603	0.228743	0.312581	AC113189	antisense
ENSG0000(	517.6215	1.357978	0.184331	7.367056	1.74E-13	2.04E-12	IL13RA2	protein_cc
ENSG0000(	517.4306	0.015547	0.056607	0.274644	0.783589	0.838905	CYYR1	protein_cc
ENSG0000(	516.8919	-0.08062	0.051979	-1.55109	0.12088	0.181785	SRP14-AS	lincRNA
ENSG0000(	516.8419	-0.00297	0.047785	-0.06208	0.950501	0.965837	FEM1A	protein_cc
ENSG0000(	516.7306	0.137815	0.077284	1.783234	0.074548	0.119713	ADAMTS6	protein_cc
ENSG0000(	516.6074	0.937101	0.109834	8.531983	1.44E-17	3.36E-16	TK1	protein_cc
ENSG0000(	516.2997	0.260386	0.029843	8.725245	2.66E-18	6.94E-17	ANKRD49	protein_cc
ENSG0000(	516.299	-0.65959	0.077541	-8.50642	1.79E-17	4.13E-16	SYT3	protein_cc
ENSG0000(	516.2258	-0.17341	0.060379	-2.87204	0.004078	0.008856	TTC39B	protein_cc
ENSG0000(	515.8429	0.463284	0.06365	7.27857	3.37E-13	3.77E-12	FAM86DP	pseudoge
ENSG0000(	515.454	0.184086	0.043776	4.205225	2.61E-05	8.40E-05	MRPL38	protein_cc
ENSG0000(	515.3475	0.06175	0.123061	0.50178	0.615822	0.698854	MX2	protein_cc
ENSG0000(	515.3363	1.305338	0.118341	11.03032	2.73E-28	4.44E-26	ASF1B	protein_cc
ENSG0000(	515.3158	0.231153	0.055839	4.139643	3.48E-05	0.00011	AC016747	processed
ENSG0000(	514.9161	0.02367	0.038098	0.621285	0.534412	0.625774	GDAP2	protein_cc
ENSG0000(	514.6963	0.215537	0.083973	2.566733	0.010266	0.02045	FAM78B	protein_cc
ENSG0000(	514.383	0.106957	0.037276	2.869327	0.004113	0.008925	DPH6	protein_cc
ENSG0000(	514.3516	0.11788	0.085322	1.381586	0.167099	0.239819	MIR4458H	lincRNA
ENSG0000(	514.3369	0.059985	0.064877	0.924594	0.355177	0.450798	PACRG	protein_cc
ENSG0000(	514.0387	-0.0004	0.159482	-0.00253	0.997984	0.998823	XKR7	protein_cc
ENSG0000(	513.8152	0.298028	0.035073	8.49745	1.94E-17	4.43E-16	PMS2CL	pseudoge
ENSG0000(	513.7362	-0.52753	0.056346	-9.36234	7.80E-21	3.05E-19	AVPI1	protein_cc
ENSG0000(	513.7336	0.391332	0.136012	2.877192	0.004012	0.008727	SEMA3A	protein_cc
ENSG0000(	513.6086	0.062093	0.053772	1.15475	0.248193	0.334898	ZNRF2	protein_cc
ENSG0000(	513.4802	0.026356	0.049417	0.533329	0.593806	0.679165	PURG	protein_cc
ENSG0000(	513.464	-0.01624	0.035438	-0.45812	0.646864	0.726047	SNX16	protein_cc
ENSG0000(	513.3831	0.247493	0.030894	8.010986	1.14E-15	1.95E-14	FASTKD3	protein_cc
ENSG0000(	513.2402	-0.03605	0.03747	-0.96213	0.335986	0.430801	PPAPDC2	protein_cc
ENSG0000(	512.9628	0.136561	0.070101	1.948056	0.051408	0.086589	ARL4D	protein_cc
ENSG0000(	512.9145	-0.65228	0.096033	-6.7923	1.10E-11	9.71E-11	FAM189A:	protein_cc
ENSG0000(	512.7889	0.161658	0.067284	2.402618	0.016278	0.030996	FBXO6	protein_cc
ENSG0000(	512.7402	0.002635	0.056853	0.046349	0.963032	0.974693	RP11-473	antisense
ENSG0000(	512.5185	0.073978	0.030913	2.393073	0.016708	0.031712	PCBD2	protein_cc
ENSG0000(	512.4419	-0.03932	0.057023	-0.6896	0.490445	0.584848	PTPDC1	protein_cc
ENSG0000(	512.4288	-0.11032	0.039449	-2.79657	0.005165	0.010976	SLC24A1	protein_cc
ENSG0000(	512.3374	-0.70112	0.111307	-6.29895	3.00E-10	2.12E-09	RP11-61I1	antisense
ENSG0000(	512.0334	-0.10191	0.042033	-2.42445	0.015332	0.02936	IQCK	protein_cc
ENSG0000(	511.6188	0.167593	0.030888	5.425903	5.77E-08	2.84E-07	ANAPC10	protein_cc
ENSG0000(	511.6031	0.317377	0.042941	7.391026	1.46E-13	1.73E-12	SNAPC1	protein_cc
ENSG0000(	511.2722	0.045431	0.069235	0.656181	0.511708	0.604802	RAB3D	protein_cc
ENSG0000(	511.2054	-0.28494	0.100794	-2.82696	0.004699	0.010061	CA4	protein_cc
ENSG0000(	510.92	0.073585	0.056269	1.307737	0.190963	0.268438	KLHL17	protein_cc
ENSG0000(	510.7858	-0.15221	0.040974	-3.71474	0.000203	0.000563	EFCAB11	protein_cc
ENSG0000(	510.6404	-0.29655	0.087063	-3.40621	0.000659	0.001661	BTNL9	protein_cc
ENSG0000(	510.6129	0.294709	0.082707	3.563281	0.000366	0.00097	LATS2	protein_cc
ENSG0000(	510.5032	-0.67714	0.10043	-6.74244	1.56E-11	1.34E-10	BEGAIN	protein_cc
ENSG0000(	510.4128	-0.00877	0.02837	-0.30906	0.757272	0.817334	ZUFSP	protein_cc
ENSG0000(	510.3757	-0.99255	0.151057	-6.57069	5.01E-11	3.99E-10	WSCD2	protein_cc
ENSG0000(	510.3301	0.255265	0.061853	4.126942	3.68E-05	0.000116	MAP7D3	protein_cc
ENSG0000(	510.0295	0.105518	0.029713	3.55119	0.000383	0.001012	ZNF350	protein_cc
ENSG0000(	509.9743	-0.05778	0.094448	-0.61172	0.54072	0.631505	TMIE	protein_cc
ENSG0000(	509.9442	0.339875	0.04861	6.991823	2.71E-12	2.64E-11	LINC0033!	lincRNA
ENSG0000(	509.599	-0.97087	0.157919	-6.14787	7.85E-10	5.21E-09	PCDHGA1	protein_cc
ENSG0000(	509.4487	0.110502	0.039988	2.763366	0.005721	0.012038	LENG1	protein_cc

ENSG0000(	509.1061	-0.55746	0.068961	-8.08369	6.28E-16	1.12E-14	KLHL3	protein_cc
ENSG0000(	508.8678	-0.27281	0.123508	-2.20889	0.027182	0.049074	GPC5	protein_cc
ENSG0000(	508.7711	-0.44528	0.100907	-4.41274	1.02E-05	3.52E-05	CLEC3B	protein_cc
ENSG0000(	508.6795	0.211684	0.024677	8.578185	9.64E-18	2.32E-16	MTFMT	protein_cc
ENSG0000(	508.5303	-0.19216	0.075604	-2.54166	0.011033	0.021833	IL16	protein_cc
ENSG0000(	508.44	-0.58674	0.072917	-8.0467	8.51E-16	1.48E-14	PLCB4	protein_cc
ENSG0000(	508.2214	0.074602	0.039648	1.881592	0.059892	0.098915	TMEM117	protein_cc
ENSG0000(	508.1728	0.077448	0.038567	2.008136	0.044629	0.076408	SPICE1	protein_cc
ENSG0000(	508.0648	-0.07833	0.039868	-1.96476	0.049442	0.083662	ZNF382	protein_cc
ENSG0000(	508.0303	0.009637	0.031535	0.305607	0.759904	0.819486	RBM15	protein_cc
ENSG0000(	507.8343	-1.51052	0.164086	-9.20566	3.40E-20	1.19E-18	SLC30A3	protein_cc
ENSG0000(	507.7898	0.092766	0.102069	0.908854	0.363427	0.459426	EPHA3	protein_cc
ENSG0000(	507.6615	-0.56982	0.096995	-5.87476	4.23E-09	2.49E-08	TOX2	protein_cc
ENSG0000(	507.1123	0.100627	0.057941	1.736715	0.082438	0.130541	CCDC24	protein_cc
ENSG0000(	506.8793	-0.77359	0.161866	-4.77919	1.76E-06	6.84E-06	TSHR	protein_cc
ENSG0000(	506.7713	-1.21379	0.161812	-7.50119	6.32E-14	7.99E-13	GABRA4	protein_cc
ENSG0000(	506.7097	0.120885	0.029484	4.099968	4.13E-05	0.000129	C12orf73	protein_cc
ENSG0000(	506.4641	-0.82667	0.103051	-8.02195	1.04E-15	1.79E-14	VWA5B2	protein_cc
ENSG0000(	506.383	-0.02801	0.026335	-1.0637	0.287466	0.378683	METTL15	protein_cc
ENSG0000(	505.8232	-0.01626	0.043516	-0.37368	0.708642	0.777758	ZNF391	protein_cc
ENSG0000(	505.751	-0.33342	0.07889	-4.22643	2.37E-05	7.70E-05	LINC0069:	antisense
ENSG0000(	505.4561	0.108756	0.090585	1.200598	0.229907	0.31388	VAX2	protein_cc
ENSG0000(	505.4148	0.028002	0.050631	0.553054	0.580226	0.66726	TMEM133	protein_cc
ENSG0000(	505.411	0.154915	0.1339	1.156941	0.247296	0.333852	TACR1	protein_cc
ENSG0000(	504.9707	0.8214	0.077937	10.53934	5.69E-26	6.03E-24	AC093673	antisense
ENSG0000(	504.9351	-0.03343	0.073945	-0.45212	0.651181	0.729693	SLC52A3	protein_cc
ENSG0000(	504.9228	0.24832	0.042359	5.862348	4.56E-09	2.67E-08	ZNF771	protein_cc
ENSG0000(	504.5529	-0.04833	0.049871	-0.96911	0.332491	0.427115	IKZF2	protein_cc
ENSG0000(	504.0253	0.138628	0.177719	0.780039	0.435368	0.532399	LIF	protein_cc
ENSG0000(	503.6717	0.012351	0.159055	0.077653	0.938104	0.956787	IRX1	protein_cc
ENSG0000(	503.6689	-0.04337	0.041556	-1.04375	0.296601	0.388688	RP11-226	sense_ove
ENSG0000(	502.7948	0.19499	0.05997	3.25144	0.001148	0.002766	EMCN	protein_cc
ENSG0000(	502.7035	-0.1669	0.06152	-2.71294	0.006669	0.013845	ZNF185	protein_cc
ENSG0000(	502.5706	0.019264	0.083034	0.232007	0.816533	0.865396	LRIG3	protein_cc
ENSG0000(	502.3962	0.107901	0.042234	2.554844	0.010624	0.021098	THUMPD2	protein_cc
ENSG0000(	502.33	0.101574	0.043009	2.361713	0.018191	0.034196	ZNF286A	protein_cc
ENSG0000(	502.0731	0.393002	0.077672	5.059785	4.20E-07	1.80E-06	DUSP10	protein_cc
ENSG0000(	502.0023	0.838768	0.086926	9.649232	4.95E-22	2.39E-20	MXD3	protein_cc
ENSG0000(	501.8146	-0.14237	0.069647	-2.04425	0.040929	0.070819	RP11-25K	antisense
ENSG0000(	501.7406	0.932865	0.110434	8.447245	2.98E-17	6.64E-16	DTL	protein_cc
ENSG0000(	501.6884	-0.57249	0.066764	-8.57488	9.92E-18	2.38E-16	TBC1D30	protein_cc
ENSG0000(	501.486	-0.87653	0.105835	-8.28204	1.21E-16	2.43E-15	RASL11B	protein_cc
ENSG0000(	501.0299	0.106385	0.032223	3.301537	0.000962	0.002349	YARS2	protein_cc
ENSG0000(	500.8908	-0.42901	0.069468	-6.17567	6.59E-10	4.42E-09	RAI2	protein_cc
ENSG0000(	500.7927	-0.33711	0.111968	-3.0108	0.002606	0.005885	LGR5	protein_cc
ENSG0000(	500.6522	0.346154	0.094184	3.675293	0.000238	0.00065	RP11-834	lincRNA
ENSG0000(	500.1666	-0.47631	0.144349	-3.29974	0.000968	0.002364	ACAN	protein_cc
ENSG0000(	500.1265	-1.49844	0.160412	-9.3412	9.52E-21	3.68E-19	UNC13C	protein_cc
ENSG0000(	500.095	0.238765	0.039049	6.114525	9.68E-10	6.33E-09	FANCE	protein_cc
ENSG0000(	499.6874	0.077396	0.02321	3.334582	0.000854	0.002108	SLC38A9	protein_cc
ENSG0000(	499.667	0.880716	0.0982	8.968628	3.00E-19	9.07E-18	RP11-425	pseudoge
ENSG0000(	499.6339	0.36611	0.072131	5.07565	3.86E-07	1.67E-06	FCHSD1	protein_cc
ENSG0000(	499.547	0.045902	0.068419	0.670906	0.502281	0.596149	FAM110A	protein_cc
ENSG0000(	499.3945	1.031668	0.122304	8.435288	3.30E-17	7.26E-16	ZMYND1(	protein_cc
ENSG0000(	499.2962	0.272034	0.048454	5.614263	1.97E-08	1.05E-07	TTC30B	protein_cc
ENSG0000(	498.993	0.126494	0.051154	2.472826	0.013405	0.026017	AD00009(	antisense
ENSG0000(	498.658	0.402873	0.123953	3.250218	0.001153	0.002777	EDA2R	protein_cc
ENSG0000(	498.5272	0.012073	0.037417	0.322677	0.74694	0.809305	ANKRD39	protein_cc
ENSG0000(	498.5257	-0.55411	0.101262	-5.47199	4.45E-08	2.23E-07	ZC3H12B	protein_cc

ENSG0000(	497.8243	-0.59893	0.092874	-6.44879	1.13E-10	8.53E-10	MCTP1	protein_cc
ENSG0000(	497.8039	-0.15338	0.066612	-2.30265	0.021298	0.039401	DACT1	protein_cc
ENSG0000(	497.8014	-0.19256	0.050542	-3.80978	0.000139	0.000396	FBXL18	protein_cc
ENSG0000(	497.7848	-0.24813	0.089829	-2.76219	0.005742	0.012077	SLC13A3	protein_cc
ENSG0000(	497.2719	0.025509	0.02488	1.025284	0.305229	0.398206	ZFPL1	protein_cc
ENSG0000(	496.8449	-0.08852	0.047683	-1.85647	0.063386	0.103944	DDX26B	protein_cc
ENSG0000(	496.7186	0.081347	0.055524	1.46506	0.142905	0.209826	TTC39C	protein_cc
ENSG0000(	496.1067	-0.01605	0.029941	-0.53616	0.591851	0.677389	BRF2	protein_cc
ENSG0000(	495.6055	-0.10278	0.039316	-2.61434	0.00894	0.018057	OBFC1	protein_cc
ENSG0000(	495.2678	0.159219	0.03624	4.393505	1.12E-05	3.82E-05	ZNF653	protein_cc
ENSG0000(	495.087	0.211618	0.042868	4.93649	7.95E-07	3.26E-06	ZNF761	processed
ENSG0000(	494.8681	-0.08724	0.030375	-2.87215	0.004077	0.008854	ZNF554	protein_cc
ENSG0000(	494.8633	0.183595	0.033384	5.499451	3.81E-08	1.93E-07	C1orf50	protein_cc
ENSG0000(	494.8127	0.647454	0.07993	8.100232	5.49E-16	9.89E-15	FAM221A	protein_cc
ENSG0000(	494.8056	0.356307	0.046414	7.676652	1.63E-14	2.27E-13	MRPS24	protein_cc
ENSG0000(	494.6564	-0.03242	0.031772	-1.0203	0.307588	0.40066	CTB-131K	antisense
ENSG0000(	494.2243	-0.08172	0.069782	-1.17108	0.241567	0.327149	FOXC1	protein_cc
ENSG0000(	494.1644	0.299173	0.031688	9.441348	3.68E-21	1.53E-19	CCDC58	protein_cc
ENSG0000(	493.9423	-1.03606	0.113963	-9.09123	9.79E-20	3.20E-18	CACNA2D	protein_cc
ENSG0000(	493.8923	0.102162	0.074667	1.368232	0.17124	0.244856	TLR3	protein_cc
ENSG0000(	493.6805	-0.23501	0.043308	-5.42636	5.75E-08	2.83E-07	AMN1	protein_cc
ENSG0000(	493.5908	0.483599	0.043882	11.02046	3.05E-28	4.84E-26	RP9P	pseudoge
ENSG0000(	493.4099	-0.05588	0.036813	-1.5179	0.129039	0.192184	C17orf59	protein_cc
ENSG0000(	493.4076	0.803297	0.116274	6.908641	4.89E-12	4.56E-11	IQGAP3	protein_cc
ENSG0000(	493.1722	0.299007	0.112931	2.647694	0.008104	0.016535	COX7A1	protein_cc
ENSG0000(	493.0124	0.255038	0.076919	3.31566	0.000914	0.002244	C4orf48	protein_cc
ENSG0000(	493.007	-0.1752	0.042847	-4.0889	4.33E-05	0.000135	NUTM2A-	antisense
ENSG0000(	492.7288	0.271497	0.03241	8.376934	5.43E-17	1.16E-15	TMEM79	protein_cc
ENSG0000(	492.25	1.069662	0.088122	12.13837	6.61E-34	3.36E-31	GINS2	protein_cc
ENSG0000(	492.1269	0.37516	0.054803	6.845553	7.62E-12	6.90E-11	QPCTL	protein_cc
ENSG0000(	492.1255	0.593536	0.118641	5.002782	5.65E-07	2.37E-06	TFAP2A	protein_cc
ENSG0000(	491.7965	0.03363	0.047828	0.703143	0.481967	0.577162	AC242842	protein_cc
ENSG0000(	491.4325	0.059117	0.0528	1.119641	0.262867	0.351423	GPR19	protein_cc
ENSG0000(	490.8247	0.219219	0.042033	5.215376	1.83E-07	8.33E-07	CCDC77	protein_cc
ENSG0000(	490.4814	0.084954	0.048559	1.74949	0.080206	0.127524	RP11-73M	sense_intr
ENSG0000(	490.4184	-0.67067	0.065632	-10.2188	1.63E-24	1.30E-22	ITGA9	protein_cc
ENSG0000(	490.3589	0.291378	0.052453	5.555022	2.78E-08	1.44E-07	PUSL1	protein_cc
ENSG0000(	490.3215	-0.31671	0.061702	-5.13298	2.85E-07	1.26E-06	HSPA12B	protein_cc
ENSG0000(	490.118	-0.09575	0.036612	-2.61529	0.008915	0.018013	NIFK-AS1	antisense
ENSG0000(	489.7643	-0.33361	0.098855	-3.3747	0.000739	0.001845	DARC	protein_cc
ENSG0000(	489.7236	-1.37491	0.124095	-11.0795	1.58E-28	2.74E-26	PPP4R4	protein_cc
ENSG0000(	489.6284	-0.96384	0.105865	-9.10445	8.67E-20	2.86E-18	LRRC7	protein_cc
ENSG0000(	489.6157	1.709137	0.137525	12.42783	1.85E-35	1.29E-32	VAV3	protein_cc
ENSG0000(	489.4476	0.315724	0.049207	6.416213	1.40E-10	1.04E-09	RNASEH1-	antisense
ENSG0000(	489.3327	0.046739	0.039603	1.180172	0.237932	0.323087	ZNF841	protein_cc
ENSG0000(	489.2027	-0.00305	0.030178	-0.101	0.919552	0.943214	THAP10	protein_cc
ENSG0000(	489.0162	0.471506	0.117917	3.998608	6.37E-05	0.000193	ANGPT1	protein_cc
ENSG0000(	488.7627	0.034206	0.028263	1.210274	0.226174	0.309609	ZNF182	protein_cc
ENSG0000(	488.622	0.386688	0.089912	4.300753	1.70E-05	5.66E-05	SQRDL	protein_cc
ENSG0000(	488.3189	0.825515	0.088661	9.310922	1.27E-20	4.79E-19	PKMYT1	protein_cc
ENSG0000(	487.7746	-0.50678	0.06868	-7.3788	1.60E-13	1.88E-12	RUNDC3B	protein_cc
ENSG0000(	487.67	0.043845	0.027559	1.590976	0.111615	0.169895	FXN	protein_cc
ENSG0000(	487.4037	-0.41261	0.049681	-8.30508	9.97E-17	2.03E-15	PWWP2B	protein_cc
ENSG0000(	487.1731	0.309576	0.08727	3.547334	0.000389	0.001026	SLC25A43	protein_cc
ENSG0000(	487.1096	-0.02419	0.069024	-0.35047	0.725982	0.791645	PILRA	protein_cc
ENSG0000(	486.6043	-0.76234	0.099504	-7.66132	1.84E-14	2.54E-13	ZNF804A	protein_cc
ENSG0000(	486.3611	0.664234	0.087357	7.603694	2.88E-14	3.83E-13	RP11-318	antisense
ENSG0000(	485.8543	0.362271	0.155134	2.335209	0.019533	0.036442	SLC47A2	protein_cc
ENSG0000(	485.7405	0.352884	0.041694	8.463572	2.59E-17	5.84E-16	C4orf46	protein_cc

ENSG0000(	485.6716	-0.26823	0.099233	-2.703	0.006872	0.014224	DAB1	protein_cc
ENSG0000(	485.6046	0.539007	0.069801	7.722023	1.14E-14	1.65E-13	TRAF5	protein_cc
ENSG0000(	485.571	0.195738	0.026844	7.291744	3.06E-13	3.44E-12	ASTE1	protein_cc
ENSG0000(	485.4544	-0.07982	0.062975	-1.26746	0.20499	0.285121	UBAP1L	protein_cc
ENSG0000(	485.4341	0.267464	0.036878	7.252647	4.09E-13	4.50E-12	CCDC126	protein_cc
ENSG0000(	485.4128	-0.18916	0.039144	-4.83245	1.35E-06	5.34E-06	ZNF70	protein_cc
ENSG0000(	485.2927	0.121944	0.105568	1.155127	0.248038	0.334722	A2ML1	protein_cc
ENSG0000(	485.2706	-0.16256	0.053851	-3.0187	0.002539	0.005749	SHB	protein_cc
ENSG0000(	485.1098	-0.82784	0.1096	-7.55327	4.24E-14	5.50E-13	NR4A3	protein_cc
ENSG0000(	484.9366	-0.17266	0.11425	-1.51127	0.130721	0.194295	MT1G	protein_cc
ENSG0000(	484.913	0.317807	0.03742	8.492903	2.02E-17	4.59E-16	C19orf82	protein_cc
ENSG0000(	484.7478	0.290024	0.043585	6.654211	2.85E-11	2.36E-10	MMS22L	protein_cc
ENSG0000(	483.9823	0.186699	0.066669	2.800379	0.005104	0.010861	ZNF404	protein_cc
ENSG0000(	483.8315	-0.10637	0.060864	-1.74767	0.080521	0.127954	RP11-356.	processed
ENSG0000(	483.5943	-0.78413	0.117084	-6.69718	2.12E-11	1.79E-10	RALYL	protein_cc
ENSG0000(	483.3295	0.229056	0.08071	2.838	0.00454	0.009749	B3GALT1	protein_cc
ENSG0000(	483.3056	2.333621	0.148422	15.72287	1.05E-55	5.42E-52	AGAP2-AS	protein_cc
ENSG0000(	483.1343	-0.09174	0.04048	-2.26628	0.023434	0.042946	SLC7A6	protein_cc
ENSG0000(	483.0627	-0.47459	0.079973	-5.93435	2.95E-09	1.78E-08	TTN	protein_cc
ENSG0000(	482.4299	0.318617	0.038074	8.368247	5.85E-17	1.24E-15	MIS18A	protein_cc
ENSG0000(	482.2941	0.555638	0.05054	10.99395	4.09E-28	6.32E-26	CCZ1B	protein_cc
ENSG0000(	482.1919	0.044267	0.041163	1.075397	0.282197	0.372842	AC004706	pseudoge
ENSG0000(	482.1751	-0.44094	0.061272	-7.19639	6.18E-13	6.62E-12	RBMS3	protein_cc
ENSG0000(	482.092	-0.19892	0.096054	-2.0709	0.038368	0.066892	RGS20	protein_cc
ENSG0000(	481.9425	0.167622	0.041308	4.057815	4.95E-05	0.000152	AC009948	antisense
ENSG0000(	481.9224	-0.03638	0.054431	-0.66835	0.503908	0.597713	RASIP1	protein_cc
ENSG0000(	481.9026	0.016452	0.052542	0.313113	0.754195	0.814761	ARHGAP1	protein_cc
ENSG0000(	481.806	-0.29472	0.09225	-3.19479	0.001399	0.003323	KCNJ5	protein_cc
ENSG0000(	481.6613	0.445096	0.05094	8.737575	2.38E-18	6.28E-17	C14orf142	protein_cc
ENSG0000(	481.2736	-0.13807	0.054726	-2.52301	0.011636	0.022903	ERG	protein_cc
ENSG0000(	481.1922	0.269199	0.039943	6.739595	1.59E-11	1.37E-10	ZNF765	protein_cc
ENSG0000(	481.03	0.259618	0.035464	7.320614	2.47E-13	2.81E-12	TFB2M	protein_cc
ENSG0000(	480.8895	0.193716	0.058459	3.313701	0.000921	0.002258	C19orf55	protein_cc
ENSG0000(	480.848	0.47211	0.048437	9.746838	1.90E-22	1.00E-20	RNFT1	protein_cc
ENSG0000(	480.7429	0.003554	0.043882	0.080992	0.935449	0.95497	IFT74	protein_cc
ENSG0000(	480.6572	0.671143	0.111151	6.038107	1.56E-09	9.85E-09	DBX2	protein_cc
ENSG0000(	480.504	-0.0871	0.048674	-1.78947	0.073539	0.118309	LNP1	protein_cc
ENSG0000(	480.1914	0.159346	0.082053	1.941982	0.052139	0.087678	DUSP23	protein_cc
ENSG0000(	480.1476	0.142458	0.050588	2.816067	0.004862	0.01038	GLB1L	protein_cc
ENSG0000(	479.6259	0.139455	0.100248	1.391101	0.164195	0.236286	RAC2	protein_cc
ENSG0000(	479.5762	-0.19504	0.049214	-3.96319	7.40E-05	0.000221	ARSG	protein_cc
ENSG0000(	479.2393	-0.11546	0.052566	-2.19655	0.028053	0.050483	MYPOP	protein_cc
ENSG0000(	479.1768	-0.29326	0.229972	-1.2752	0.202237	0.281963	UTY	protein_cc
ENSG0000(	479.0848	-0.19507	0.078345	-2.48987	0.012779	0.024901	SMOC2	protein_cc
ENSG0000(	478.6871	0.249138	0.062217	4.004324	6.22E-05	0.000189	KIAA1614	protein_cc
ENSG0000(	478.6167	-0.14003	0.038533	-3.63393	0.000279	0.000755	BOLA3-AS	antisense
ENSG0000(	478.6123	-0.26967	0.143647	-1.87729	0.060478	0.09974	PPL	protein_cc
ENSG0000(	478.5876	0.082153	0.075839	1.083251	0.278697	0.369071	NOXA1	protein_cc
ENSG0000(	478.5616	-0.14838	0.058378	-2.54175	0.01103	0.021829	PDE5A	protein_cc
ENSG0000(	478.468	0.249045	0.032812	7.58998	3.20E-14	4.23E-13	CLP1	protein_cc
ENSG0000(	477.984	0.087482	0.148812	0.587873	0.556618	0.645945	HSPA6	protein_cc
ENSG0000(	477.7309	-0.26249	0.12352	-2.1251	0.033578	0.05934	IGFBP6	protein_cc
ENSG0000(	477.6048	-0.11483	0.116817	-0.983	0.325609	0.419773	SIX1	protein_cc
ENSG0000(	477.432	0.027489	0.037105	0.740847	0.458786	0.555177	OMA1	protein_cc
ENSG0000(	477.0905	0.090642	0.043783	2.070274	0.038427	0.066983	ZNF610	protein_cc
ENSG0000(	477.0259	0.047157	0.093386	0.504963	0.613585	0.696969	KLHL29	protein_cc
ENSG0000(	476.9456	-1.65542	0.165635	-9.99442	1.61E-23	1.06E-21	MAL2	protein_cc
ENSG0000(	476.9229	-0.56729	0.128627	-4.41033	1.03E-05	3.56E-05	HS3ST2	protein_cc
ENSG0000(	476.8306	-0.02381	0.043079	-0.55277	0.580421	0.667391	ATXN7L1	protein_cc

ENSG0000(	476.5946	0.557493	0.055178	10.10345	5.33E-24	3.86E-22	CCZ1	protein_cc
ENSG0000(	476.5513	-0.11079	0.044014	-2.51718	0.01183	0.023247	GPR75-AS	protein_cc
ENSG0000(	476.4114	-1.12312	0.124894	-8.99259	2.41E-19	7.42E-18	HTR2A	protein_cc
ENSG0000(	476.2346	-0.17256	0.049185	-3.50828	0.000451	0.001175	HIC2	protein_cc
ENSG0000(	476.1026	-1.05368	0.13301	-7.92181	2.34E-15	3.80E-14	CACNA1I	protein_cc
ENSG0000(	476.072	-0.63484	0.09545	-6.65104	2.91E-11	2.41E-10	JAKMIP3	protein_cc
ENSG0000(	476.0528	-0.22626	0.05906	-3.83112	0.000128	0.000366	TCP11L2	protein_cc
ENSG0000(	475.9044	0.074627	0.149576	0.498927	0.617831	0.700727	HAMP	protein_cc
ENSG0000(	475.839	0.131838	0.050557	2.607725	0.009115	0.018371	CTA-217C	lincRNA
ENSG0000(	475.805	-0.04348	0.074256	-0.58558	0.558159	0.647295	ARHGAP9	protein_cc
ENSG0000(	475.4243	-0.01993	0.032438	-0.61441	0.538943	0.629895	USP45	protein_cc
ENSG0000(	475.02	0.23625	0.029596	7.98253	1.43E-15	2.42E-14	PARP16	protein_cc
ENSG0000(	475.007	1.350498	0.171837	7.8592	3.87E-15	6.07E-14	DDIT4L	protein_cc
ENSG0000(	474.0893	0.182635	0.038771	4.710659	2.47E-06	9.38E-06	RABGEF1	protein_cc
ENSG0000(	473.7822	-0.14072	0.027566	-5.10474	3.31E-07	1.44E-06	KIAA1328	protein_cc
ENSG0000(	473.7343	0.343961	0.164162	2.095259	0.036148	0.063441	DLX6-AS1	antisense
ENSG0000(	473.5945	-0.81064	0.10986	-7.3788	1.60E-13	1.88E-12	VWC2	protein_cc
ENSG0000(	473.5844	-0.40886	0.058413	-6.99945	2.57E-12	2.51E-11	STAMBPL1	protein_cc
ENSG0000(	473.4447	0.248399	0.035287	7.039353	1.93E-12	1.93E-11	ZNF583	protein_cc
ENSG0000(	472.6575	0.156056	0.101643	1.535335	0.124701	0.186725	RP4-668G	antisense
ENSG0000(	472.5372	0.255179	0.079513	3.209254	0.001331	0.003172	KLHDC9	protein_cc
ENSG0000(	472.4443	-0.18973	0.046155	-4.11067	3.95E-05	0.000123	PPP1R3D	protein_cc
ENSG0000(	472.4082	-0.06548	0.045449	-1.44068	0.149676	0.218365	TBC1D32	protein_cc
ENSG0000(	472.3356	-1.19415	0.123366	-9.67976	3.68E-22	1.82E-20	CYP26B1	protein_cc
ENSG0000(	472.2923	0.605496	0.084518	7.164119	7.83E-13	8.28E-12	ARNTL2	protein_cc
ENSG0000(	472.2341	0.196551	0.062974	3.121166	0.001801	0.004193	KCNAB3	protein_cc
ENSG0000(	472.2302	-0.07984	0.085801	-0.93051	0.352105	0.447671	NCF4	protein_cc
ENSG0000(	472.1739	0.28501	0.045044	6.327345	2.49E-10	1.79E-09	GS1-124K	lincRNA
ENSG0000(	472.0723	0.180746	0.03731	4.844489	1.27E-06	5.05E-06	C8orf58	protein_cc
ENSG0000(	471.9879	-1.10127	0.111031	-9.91858	3.46E-23	2.10E-21	MATK	protein_cc
ENSG0000(	471.9113	-0.02121	0.036709	-0.57772	0.563455	0.651966	KIAA1009	protein_cc
ENSG0000(	471.6837	1.135106	0.188277	6.028922	1.65E-09	1.04E-08	CXCL10	protein_cc
ENSG0000(	471.5951	-0.98712	0.148987	-6.62551	3.46E-11	2.83E-10	HRH3	protein_cc
ENSG0000(	471.4341	-1.19408	0.097962	-12.1892	3.55E-34	1.87E-31	SAMD12	protein_cc
ENSG0000(	471.4004	-0.38459	0.07511	-5.12039	3.05E-07	1.34E-06	SLC6A12	protein_cc
ENSG0000(	471.3599	-0.37669	0.122227	-3.08187	0.002057	0.004737	EPHA5	protein_cc
ENSG0000(	471.283	-0.07536	0.069903	-1.07809	0.280996	0.371555	RP11-258	lincRNA
ENSG0000(	471.1468	0.131736	0.055035	2.393693	0.01668	0.031665	SMPDL3A	protein_cc
ENSG0000(	470.8089	-0.24866	0.05408	-4.59802	4.27E-06	1.56E-05	ZFHX2	protein_cc
ENSG0000(	470.7951	-0.27971	0.063028	-4.43791	9.08E-06	3.16E-05	INPP4B	protein_cc
ENSG0000(	470.7741	0.384372	0.039428	9.748742	1.87E-22	9.86E-21	ZNF700	protein_cc
ENSG0000(	470.5933	0.358921	0.040775	8.802407	1.34E-18	3.66E-17	ZNF682	protein_cc
ENSG0000(	470.4461	0.242871	0.070213	3.459068	0.000542	0.00139	BCL6B	protein_cc
ENSG0000(	470.3095	0.19926	0.038989	5.110686	3.21E-07	1.40E-06	POLG2	protein_cc
ENSG0000(	470.1945	-0.31155	0.1092	-2.85298	0.004331	0.009348	HOXD-AS	antisense
ENSG0000(	470.1285	-1.27566	0.190694	-6.68954	2.24E-11	1.88E-10	GJB6	protein_cc
ENSG0000(	469.9493	-0.11885	0.079688	-1.49138	0.135862	0.200862	MGMT	protein_cc
ENSG0000(	469.9081	0.335743	0.054232	6.190913	5.98E-10	4.03E-09	NPHP1	protein_cc
ENSG0000(	469.2854	0.085869	0.031012	2.768872	0.005625	0.011855	SRFBP1	protein_cc
ENSG0000(	469.0553	-0.01509	0.030958	-0.4874	0.625974	0.707913	MMAA	protein_cc
ENSG0000(	468.9048	0.104975	0.03476	3.019992	0.002528	0.005727	RP11-33B	pseudoge
ENSG0000(	468.6885	0.620798	0.070599	8.793271	1.45E-18	3.95E-17	TTC26	protein_cc
ENSG0000(	468.5167	0.114064	0.034836	3.274344	0.001059	0.002569	MARS2	protein_cc
ENSG0000(	468.1923	0.295916	0.041788	7.081357	1.43E-12	1.45E-11	TGDS	protein_cc
ENSG0000(	468.1329	0.094341	0.02307	4.089259	4.33E-05	0.000135	MED26	protein_cc
ENSG0000(	467.5495	-0.01082	0.083217	-0.13006	0.896516	0.926549	ARHGAP2	protein_cc
ENSG0000(	467.3944	-0.04747	0.066769	-0.71095	0.477118	0.572759	RP11-395	antisense
ENSG0000(	467.2368	-0.09175	0.060771	-1.50969	0.131124	0.194859	CTA-204B	antisense
ENSG0000(	466.8707	0.303371	0.045216	6.70939	1.95E-11	1.66E-10	VRK1	protein_cc

ENSG0000(	466.8278	-0.00514	0.070151	-0.07321	0.941636	0.959271	TRPV2	protein_cc
ENSG0000(	466.7415	-0.53397	0.101125	-5.28025	1.29E-07	6.00E-07	SYT9	protein_cc
ENSG0000(	466.6411	0.079927	0.16316	0.48987	0.624226	0.70646	S100A8	protein_cc
ENSG0000(	466.4743	0.413718	0.107253	3.857396	0.000115	0.000331	EVC	protein_cc
ENSG0000(	465.775	0.015083	0.033849	0.4456	0.655886	0.733591	ZNF526	protein_cc
ENSG0000(	465.7708	0.436516	0.035116	12.43067	1.78E-35	1.26E-32	GTF2IRD2I	protein_cc
ENSG0000(	465.75	0.413922	0.058656	7.056775	1.70E-12	1.71E-11	DYNLL1-A	antisense
ENSG0000(	465.4884	-0.59768	0.151021	-3.95759	7.57E-05	0.000226	DACH2	protein_cc
ENSG0000(	465.2917	0.341497	0.083887	4.070934	4.68E-05	0.000145	MUC1	protein_cc
ENSG0000(	465.2546	-0.20269	0.085845	-2.36109	0.018221	0.03424	HPSE	protein_cc
ENSG0000(	465.1316	-0.1305	0.039407	-3.31156	0.000928	0.002273	LACE1	protein_cc
ENSG0000(	465.1183	0.362957	0.055472	6.543058	6.03E-11	4.75E-10	GK	protein_cc
ENSG0000(	464.6085	0.17311	0.03117	5.553702	2.80E-08	1.45E-07	WDR4	protein_cc
ENSG0000(	464.3896	-0.11062	0.143889	-0.76878	0.442021	0.538837	RP11-231	lincRNA
ENSG0000(	464.3525	-0.16324	0.043206	-3.77831	0.000158	0.000446	CCNK	protein_cc
ENSG0000(	464.2615	0.079443	0.050354	1.57768	0.114639	0.173848	NPHP3	protein_cc
ENSG0000(	464.1124	0.077299	0.05838	1.324082	0.185476	0.261855	FKBP11	protein_cc
ENSG0000(	463.9582	0.343815	0.123732	2.77872	0.005457	0.011539	GALR1	protein_cc
ENSG0000(	463.7876	-0.02387	0.036347	-0.65679	0.511314	0.604475	PIGA	protein_cc
ENSG0000(	463.6768	0.137952	0.070178	1.965738	0.049329	0.083492	LPAR2	protein_cc
ENSG0000(	463.6197	1.009596	0.102478	9.851846	6.73E-23	3.88E-21	WDR62	protein_cc
ENSG0000(	463.5008	0.255779	0.040057	6.385402	1.71E-10	1.25E-09	MED30	protein_cc
ENSG0000(	463.1979	0.271018	0.058479	4.634467	3.58E-06	1.33E-05	TRADD	protein_cc
ENSG0000(	463.1646	0.10778	0.051337	2.099458	0.035777	0.062851	SLMO1	protein_cc
ENSG0000(	462.7538	0.261935	0.042228	6.202825	5.55E-10	3.75E-09	ZMYM1	protein_cc
ENSG0000(	462.3459	0.251588	0.047945	5.247456	1.54E-07	7.09E-07	TMEM234	protein_cc
ENSG0000(	462.2316	-0.22688	0.04027	-5.6339	1.76E-08	9.41E-08	DEXI	protein_cc
ENSG0000(	461.9093	0.117862	0.095885	1.229193	0.218999	0.30138	RP1-249H	lincRNA
ENSG0000(	461.0953	1.046421	0.130078	8.044575	8.65E-16	1.50E-14	ASPM	protein_cc
ENSG0000(	460.9965	0.097251	0.051379	1.892812	0.058383	0.096761	GUSBP1	pseudoge
ENSG0000(	460.9648	0.368368	0.103757	3.550305	0.000385	0.001015	EPSTI1	protein_cc
ENSG0000(	460.9152	0.485654	0.058347	8.323579	8.53E-17	1.75E-15	GEN1	protein_cc
ENSG0000(	460.3972	-0.36186	0.075143	-4.81562	1.47E-06	5.78E-06	TRAM1L1	protein_cc
ENSG0000(	459.716	0.066587	0.082854	0.803665	0.421591	0.51856	GSTT2B	protein_cc
ENSG0000(	459.615	0.079383	0.031817	2.494993	0.012596	0.024582	SPATA5	protein_cc
ENSG0000(	459.5625	-0.0839	0.033611	-2.49613	0.012556	0.024512	ARHGEF18	protein_cc
ENSG0000(	459.482	0.076383	0.074161	1.029951	0.303033	0.39573	ACOX2	protein_cc
ENSG0000(	459.2661	0.259059	0.050018	5.179306	2.23E-07	9.98E-07	DUSP15	protein_cc
ENSG0000(	459.2592	0.269831	0.041479	6.505193	7.76E-11	6.02E-10	NDUFV2	protein_cc
ENSG0000(	459.1498	0.574656	0.064666	8.886483	6.31E-19	1.81E-17	ZNF468	protein_cc
ENSG0000(	458.9854	-0.0552	0.064961	-0.84969	0.395499	0.492066	CYP51A1	protein_cc
ENSG0000(	458.8171	-0.45453	0.117727	-3.86092	0.000113	0.000327	IQCA1	protein_cc
ENSG0000(	458.6038	0.112543	0.090624	1.241857	0.214289	0.296007	FAHD2B	protein_cc
ENSG0000(	458.3127	-0.45878	0.131471	-3.48959	0.000484	0.001252	LPPR3	protein_cc
ENSG0000(	458.3103	-0.4529	0.088794	-5.10057	3.39E-07	1.47E-06	OBSCN	protein_cc
ENSG0000(	458.2924	-0.20116	0.033153	-6.06784	1.30E-09	8.31E-09	C10orf88	protein_cc
ENSG0000(	458.1834	0.236829	0.06866	3.449285	0.000562	0.001437	IRF9	protein_cc
ENSG0000(	458.1412	-0.05195	0.048334	-1.07475	0.282486	0.373139	CTA-445C	antisense
ENSG0000(	458.1299	0.846335	0.129617	6.52948	6.60E-11	5.17E-10	CHST8	protein_cc
ENSG0000(	458.1174	0.305843	0.057889	5.283222	1.27E-07	5.91E-07	LINC0017	lincRNA
ENSG0000(	458.111	0.202368	0.05136	3.940178	8.14E-05	0.000242	DTX2	protein_cc
ENSG0000(	458.0332	-0.76044	0.115928	-6.55959	5.40E-11	4.28E-10	SAMD11	protein_cc
ENSG0000(	457.7944	-0.04484	0.100745	-0.44507	0.656271	0.733881	CLEC7A	protein_cc
ENSG0000(	457.6662	-0.1017	0.038365	-2.65089	0.008028	0.016392	RP11-398	antisense
ENSG0000(	457.6476	0.293039	0.036911	7.939099	2.04E-15	3.34E-14	ZNF180	protein_cc
ENSG0000(	457.2991	0.003565	0.047325	0.075328	0.939954	0.958151	CEP19	protein_cc
ENSG0000(	457.269	0.131878	0.044044	2.994211	0.002752	0.006174	SUV39H2	protein_cc
ENSG0000(	457.2164	0.04813	0.068986	0.697685	0.485374	0.580281	RP11-981	lincRNA
ENSG0000(	457.1553	0.242869	0.05035	4.823601	1.41E-06	5.57E-06	RP13-582	antisense

ENSG0000(	457.1505	0.227842	0.054942	4.146942	3.37E-05	0.000107	RADIL	protein_cc
ENSG0000(	457.0695	0.100004	0.026723	3.742301	0.000182	0.000509	ZNF764	protein_cc
ENSG0000(	456.9865	-0.03883	0.067338	-0.57663	0.564192	0.652525	L3MBTL4	protein_cc
ENSG0000(	456.9336	-0.07568	0.028908	-2.61811	0.008842	0.017877	NAF1	protein_cc
ENSG0000(	456.7435	0.216588	0.088853	2.437597	0.014785	0.028427	PAWR	protein_cc
ENSG0000(	456.5283	-1.51932	0.155716	-9.75699	1.72E-22	9.13E-21	TBR1	protein_cc
ENSG0000(	456.5036	0.363404	0.042657	8.519237	1.61E-17	3.71E-16	COQ2	protein_cc
ENSG0000(	456.2916	0.34077	0.055962	6.089344	1.13E-09	7.33E-09	LINC0115:	lincRNA
ENSG0000(	456.1881	1.57392	0.169692	9.275136	1.77E-20	6.51E-19	CTD-3049	lincRNA
ENSG0000(	456.1638	0.195501	0.056442	3.463723	0.000533	0.001368	MIR3916	pseudoge
ENSG0000(	455.9234	0.042704	0.041347	1.032825	0.301686	0.394321	ATP6V1E2	protein_cc
ENSG0000(	455.5457	0.007118	0.173858	0.040939	0.967345	0.977422	HLA-DQA	protein_cc
ENSG0000(	455.4439	-0.6135	0.075994	-8.07296	6.86E-16	1.22E-14	NLRP3	protein_cc
ENSG0000(	455.441	0.307163	0.039712	7.734716	1.04E-14	1.51E-13	KTI12	protein_cc
ENSG0000(	454.9374	-0.39985	0.119195	-3.35455	0.000795	0.001974	RP11-713	sense_intr
ENSG0000(	454.6615	-0.48128	0.059369	-8.10653	5.21E-16	9.42E-15	RNF152	protein_cc
ENSG0000(	454.4616	0.028396	0.099819	0.284474	0.776047	0.832811	RP11-429	lincRNA
ENSG0000(	454.3705	0.009816	0.041696	0.235411	0.81389	0.863262	HDDC3	protein_cc
ENSG0000(	454.3502	-0.12689	0.106581	-1.19053	0.233839	0.31831	NTNG1	protein_cc
ENSG0000(	454.0607	0.696378	0.074004	9.410045	4.96E-21	2.01E-19	ZNF367	protein_cc
ENSG0000(	454.0174	0.142541	0.073534	1.938441	0.052569	0.088286	FAM43B	protein_cc
ENSG0000(	453.9295	0.226113	0.034374	6.578038	4.77E-11	3.81E-10	CEP57L1	protein_cc
ENSG0000(	453.485	0.072289	0.037538	1.925752	0.054135	0.090572	PYROXD1	protein_cc
ENSG0000(	453.3179	-0.39504	0.102162	-3.86676	0.00011	0.00032	FHL2	protein_cc
ENSG0000(	452.8328	0.112746	0.026794	4.207875	2.58E-05	8.31E-05	TIGD6	protein_cc
ENSG0000(	452.8251	-0.09328	0.08877	-1.05082	0.293342	0.385043	FAM50B	protein_cc
ENSG0000(	452.5217	0.192644	0.072586	2.654005	0.007954	0.016262	IZUMO4	protein_cc
ENSG0000(	452.3822	0.06801	0.035461	1.917909	0.055123	0.092033	ARMCX5	protein_cc
ENSG0000(	452.2854	0.216937	0.025921	8.369116	5.81E-17	1.23E-15	CBWD2	protein_cc
ENSG0000(	452.1162	0.055842	0.074189	0.752697	0.451632	0.548229	TEX9	protein_cc
ENSG0000(	452.008	0.387271	0.094278	4.107766	4.00E-05	0.000125	FZD6	protein_cc
ENSG0000(	451.8439	0.045801	0.080446	0.569345	0.569122	0.657044	ALPK1	protein_cc
ENSG0000(	451.7069	0.257645	0.051147	5.037348	4.72E-07	2.01E-06	PASK	protein_cc
ENSG0000(	451.6254	-0.17956	0.072582	-2.47388	0.013366	0.02595	PRR5	protein_cc
ENSG0000(	451.5708	-0.11402	0.068444	-1.66589	0.095736	0.148786	C12orf75	protein_cc
ENSG0000(	451.2093	-0.16041	0.108484	-1.47865	0.139233	0.205175	FREM1	protein_cc
ENSG0000(	451.0925	0.014647	0.100303	0.146029	0.883898	0.917105	PPP1R14C	protein_cc
ENSG0000(	451.0066	-0.15039	0.069044	-2.17816	0.029394	0.05269	SIGIRR	protein_cc
ENSG0000(	450.9603	-0.23206	0.127857	-1.81503	0.06952	0.112649	CORO6	protein_cc
ENSG0000(	450.9296	0.36611	0.078039	4.691391	2.71E-06	1.02E-05	GIMAP2	protein_cc
ENSG0000(	450.9083	-0.00935	0.054082	-0.17298	0.862669	0.901034	AGAP6	protein_cc
ENSG0000(	450.7191	0.118414	0.041477	2.854899	0.004305	0.009298	LINC0088:	lincRNA
ENSG0000(	450.6849	0.02886	0.049632	0.58148	0.560917	0.649797	EPOR	protein_cc
ENSG0000(	450.481	-0.02302	0.035612	-0.64634	0.518062	0.610732	AP001372	lincRNA
ENSG0000(	450.2983	-0.30569	0.057516	-5.31483	1.07E-07	5.04E-07	ZNF81	protein_cc
ENSG0000(	449.4807	-0.30375	0.112072	-2.71036	0.006721	0.01394	PDIA2	protein_cc
ENSG0000(	449.0578	0.065076	0.036987	1.759431	0.078504	0.125215	ZNF550	protein_cc
ENSG0000(	448.8903	-0.161	0.141963	-1.13409	0.256755	0.344619	SPON2	protein_cc
ENSG0000(	448.6241	0.273061	0.029574	9.233066	2.63E-20	9.39E-19	POC5	protein_cc
ENSG0000(	447.9893	-0.14203	0.070307	-2.02021	0.043362	0.074484	NHS	protein_cc
ENSG0000(	447.8377	0.070145	0.064027	1.095549	0.273276	0.363179	MIR497H(	antisense
ENSG0000(	447.7061	0.12021	0.0371	3.240172	0.001195	0.002869	CA5BP1	pseudoge
ENSG0000(	447.6935	0.797542	0.07235	11.02333	2.95E-28	4.75E-26	CDCA3	protein_cc
ENSG0000(	446.8533	-0.10237	0.034835	-2.93875	0.003295	0.007285	PEX7	protein_cc
ENSG0000(	446.8468	0.104871	0.022927	4.574116	4.78E-06	1.74E-05	TAMM41	protein_cc
ENSG0000(	446.8085	-0.25625	0.121103	-2.11593	0.03435	0.060599	THBD	protein_cc
ENSG0000(	446.7723	1.018613	0.099756	10.21102	1.77E-24	1.40E-22	CD58	protein_cc
ENSG0000(	446.7473	0.156588	0.03963	3.951205	7.78E-05	0.000232	ZNF112	protein_cc
ENSG0000(	446.3507	0.041728	0.048509	0.860207	0.389675	0.486272	PRICKLE4	protein_cc

ENSG0000(	446.2093	0.3322	0.055975	5.934788	2.94E-09	1.78E-08	MYBL1	protein_cc
ENSG0000(	446.1734	-1.17822	0.153868	-7.65736	1.90E-14	2.62E-13	GDF10	protein_cc
ENSG0000(	446.1462	0.668838	0.114887	5.821714	5.82E-09	3.35E-08	CYTL1	protein_cc
ENSG0000(	445.7103	0.088169	0.086494	1.019362	0.308031	0.401122	PYCARD	protein_cc
ENSG0000(	445.663	0.09756	0.038175	2.555605	0.0106	0.021056	TMCO6	protein_cc
ENSG0000(	445.6404	-0.36866	0.070595	-5.22222	1.77E-07	8.05E-07	CARD11	protein_cc
ENSG0000(	445.5986	0.474706	0.060726	7.817152	5.40E-15	8.25E-14	KIF9	protein_cc
ENSG0000(	445.4688	-0.1183	0.049029	-2.41287	0.015828	0.030215	FHIT	protein_cc
ENSG0000(	445.3225	0.080955	0.028059	2.885133	0.003912	0.008528	WDSUB1	protein_cc
ENSG0000(	445.2719	0.188395	0.027005	6.976239	3.03E-12	2.93E-11	ZCCHC4	protein_cc
ENSG0000(	445.2594	-1.17478	0.127378	-9.22278	2.89E-20	1.02E-18	TCEAL6	protein_cc
ENSG0000(	445.0028	0.787999	0.070003	11.25658	2.15E-29	4.33E-27	CENPH	protein_cc
ENSG0000(	444.8636	-0.80004	0.129506	-6.17765	6.51E-10	4.37E-09	UNC5D	protein_cc
ENSG0000(	444.7976	0.140335	0.077542	1.809784	0.070329	0.113773	RPL41P1	pseudoge
ENSG0000(	444.7556	0.599585	0.100995	5.936771	2.91E-09	1.76E-08	XKR8	protein_cc
ENSG0000(	444.6786	0.163068	0.094914	1.718062	0.085785	0.135216	OPLAH	protein_cc
ENSG0000(	444.4975	0.322626	0.039047	8.262566	1.43E-16	2.83E-15	ZNF461	protein_cc
ENSG0000(	444.321	-0.08622	0.091471	-0.94261	0.345878	0.441287	WNT7A	protein_cc
ENSG0000(	444.0103	0.141272	0.097313	1.451726	0.146578	0.214454	CLGN	protein_cc
ENSG0000(	443.3133	-0.38235	0.097591	-3.9179	8.93E-05	0.000263	NIPAL2	protein_cc
ENSG0000(	443.2293	0.790366	0.162611	4.860481	1.17E-06	4.68E-06	FTLP3	pseudoge
ENSG0000(	443.2214	-1.35681	0.127133	-10.6723	1.37E-26	1.65E-24	NOS1	protein_cc
ENSG0000(	443.0671	-0.0445	0.072185	-0.61654	0.537541	0.628632	AMY2B	protein_cc
ENSG0000(	442.2411	0.21053	0.067863	3.102264	0.00192	0.004444	LACTB2	protein_cc
ENSG0000(	442.2396	1.222908	0.122759	9.961836	2.24E-23	1.44E-21	NCAPG	protein_cc
ENSG0000(	442.1898	-0.03905	0.031765	-1.22943	0.218912	0.30129	RPP38	protein_cc
ENSG0000(	442.0912	0.058108	0.046636	1.245996	0.212766	0.29419	ZEB1-AS1	processed
ENSG0000(	441.829	0.152364	0.040813	3.733227	0.000189	0.000526	SLC10A7	protein_cc
ENSG0000(	441.8091	0.09548	0.108868	0.877024	0.380474	0.47703	PCDHB15	protein_cc
ENSG0000(	441.57	-0.11327	0.066214	-1.71066	0.087143	0.137133	USP37	protein_cc
ENSG0000(	441.3384	0.434482	0.105239	4.128535	3.65E-05	0.000115	ALK	protein_cc
ENSG0000(	441.3088	0.403107	0.064561	6.243833	4.27E-10	2.94E-09	MYCBP	protein_cc
ENSG0000(	441.0532	0.23217	0.036757	6.316361	2.68E-10	1.91E-09	LSM12	protein_cc
ENSG0000(	440.8099	0.447018	0.044233	10.10592	5.20E-24	3.80E-22	PRIM2	protein_cc
ENSG0000(	440.7257	-0.17728	0.145264	-1.2204	0.222315	0.305227	MET	protein_cc
ENSG0000(	440.5229	-0.5523	0.092559	-5.96696	2.42E-09	1.48E-08	ADAMTS8	protein_cc
ENSG0000(	440.1749	0.133367	0.035046	3.805461	0.000142	0.000402	THAP9	protein_cc
ENSG0000(	439.9699	0.059218	0.025168	2.352925	0.018626	0.034931	TRMT44	protein_cc
ENSG0000(	439.9548	-0.94645	0.102431	-9.2399	2.47E-20	8.85E-19	AKAP5	protein_cc
ENSG0000(	439.8649	-1.45578	0.122949	-11.8406	2.41E-32	8.69E-30	CDH8	protein_cc
ENSG0000(	439.7921	-0.19663	0.086715	-2.26756	0.023356	0.042828	HOGA1	protein_cc
ENSG0000(	439.7522	1.274548	0.11463	11.11876	1.02E-28	1.83E-26	SPC24	protein_cc
ENSG0000(	439.6744	-0.4147	0.103019	-4.0255	5.69E-05	0.000173	NOV	protein_cc
ENSG0000(	439.518	0.446438	0.093025	4.799102	1.59E-06	6.24E-06	HDHD3	protein_cc
ENSG0000(	439.4243	-1.09596	0.14955	-7.32834	2.33E-13	2.66E-12	RASAL1	protein_cc
ENSG0000(	439.3999	0.105868	0.06218	1.702618	0.08864	0.139205	HDAC10	protein_cc
ENSG0000(	439.2661	-0.13819	0.032029	-4.31446	1.60E-05	5.34E-05	ZNF839	protein_cc
ENSG0000(	439.2627	-1.07186	0.187838	-5.7063	1.15E-08	6.34E-08	FREM3	protein_cc
ENSG0000(	439.0664	-0.253	0.126666	-1.99739	0.045783	0.078152	PCDHGA1	protein_cc
ENSG0000(	438.8261	-0.20446	0.05951	-3.43573	0.000591	0.001504	TNFSF13	protein_cc
ENSG0000(	438.6562	-0.3234	0.132262	-2.44512	0.01448	0.027913	C7	protein_cc
ENSG0000(	438.507	0.071346	0.04583	1.556753	0.119529	0.180088	ZNF225	protein_cc
ENSG0000(	438.4945	0.221915	0.048759	4.551214	5.33E-06	1.92E-05	TMEM44	protein_cc
ENSG0000(	437.0795	0.105872	0.166958	0.634122	0.526001	0.617789	SEMA3E	protein_cc
ENSG0000(	436.9759	0.247829	0.086008	2.881469	0.003958	0.008617	QPCT	protein_cc
ENSG0000(	436.5201	-0.10416	0.063402	-1.64278	0.100429	0.155083	PROB1	protein_cc
ENSG0000(	436.477	-0.23835	0.04745	-5.02318	5.08E-07	2.15E-06	BAG2	protein_cc
ENSG0000(	435.9764	0.190878	0.031873	5.988652	2.12E-09	1.31E-08	GTPBP8	protein_cc
ENSG0000(	435.8153	-0.05341	0.080173	-0.66619	0.505292	0.599055	RP11-713	lincRNA

ENSG0000(	435.2474	-1.13857	0.141695	-8.03532	9.33E-16	1.61E-14	KCNA1	protein_cc
ENSG0000(	435.1915	0.12142	0.044652	2.719238	0.006543	0.0136	NRG2	protein_cc
ENSG0000(	435.1318	-0.13111	0.033369	-3.929	8.53E-05	0.000253	RP11-342	antisense
ENSG0000(	435.0284	-0.19724	0.039362	-5.01096	5.42E-07	2.28E-06	RP11-156	processed
ENSG0000(	434.7095	0.233394	0.075896	3.075177	0.002104	0.004833	PPIL6	protein_cc
ENSG0000(	434.6208	0.072064	0.094786	0.760275	0.44709	0.543872	PCED1B-A	processed
ENSG0000(	434.6139	0.175292	0.050134	3.496494	0.000471	0.001223	GBAP1	pseudoge
ENSG0000(	434.5598	1.30E-05	0.088986	0.000146	0.999883	0.99989	RP5-855F:	lincRNA
ENSG0000(	434.4343	0.011762	0.05915	0.198849	0.842381	0.885511	ZNF135	protein_cc
ENSG0000(	434.3269	0.575034	0.14039	4.095976	4.20E-05	0.000131	SLPI	protein_cc
ENSG0000(	434.2308	0.342264	0.035514	9.637534	5.55E-22	2.66E-20	ZNF599	protein_cc
ENSG0000(	434.2143	-0.47848	0.105905	-4.51803	6.24E-06	2.23E-05	GRHL3	protein_cc
ENSG0000(	433.9038	-0.01995	0.054398	-0.36681	0.713759	0.781715	DNAJC3-A	lincRNA
ENSG0000(	433.6502	0.1081	0.047383	2.281383	0.022526	0.041472	ENDOG	protein_cc
ENSG0000(	433.5455	0.156816	0.060897	2.575077	0.010022	0.02001	CLEC2D	protein_cc
ENSG0000(	433.336	-1.33476	0.140237	-9.51788	1.77E-21	7.85E-20	SLC6A15	protein_cc
ENSG0000(	433.3223	0.21268	0.038017	5.594298	2.22E-08	1.17E-07	GLMN	protein_cc
ENSG0000(	433.1584	1.125989	0.11695	9.627949	6.09E-22	2.89E-20	CDCA8	protein_cc
ENSG0000(	432.6155	0.044944	0.103239	0.435336	0.663318	0.74001	NFKBIZ	protein_cc
ENSG0000(	432.2591	0.080833	0.073624	1.097915	0.272242	0.362027	MFI2	protein_cc
ENSG0000(	432.1543	0.138168	0.097629	1.415235	0.157	0.227428	KCNMB1	protein_cc
ENSG0000(	431.7546	-0.08349	0.04772	-1.74962	0.080184	0.127511	CAPS2	protein_cc
ENSG0000(	431.3166	-1.16741	0.128332	-9.09682	9.30E-20	3.05E-18	LRFN5	protein_cc
ENSG0000(	431.2299	-0.23513	0.053384	-4.40451	1.06E-05	3.65E-05	YPEL1	protein_cc
ENSG0000(	431.1404	0.100744	0.083154	1.211539	0.225689	0.309086	C1orf106	protein_cc
ENSG0000(	430.9934	0.174678	0.055978	3.120451	0.001806	0.004201	EIF4A1	protein_cc
ENSG0000(	430.967	-0.54168	0.075868	-7.13976	9.35E-13	9.77E-12	NRROS	protein_cc
ENSG0000(	430.8743	0.433141	0.078551	5.514172	3.50E-08	1.79E-07	TLCD1	protein_cc
ENSG0000(	430.6971	-0.94866	0.138449	-6.85207	7.28E-12	6.61E-11	CDH18	protein_cc
ENSG0000(	430.4204	-0.86347	0.132442	-6.51962	7.05E-11	5.50E-10	ZCCHC12	protein_cc
ENSG0000(	429.964	1.515015	0.130587	11.60158	4.05E-31	1.16E-28	NOS2	protein_cc
ENSG0000(	429.8181	-0.24993	0.165042	-1.51436	0.129935	0.193274	CACNG2	protein_cc
ENSG0000(	429.8037	-0.07149	0.050515	-1.41527	0.156989	0.22742	AC239811	protein_cc
ENSG0000(	429.7796	0.239354	0.076517	3.128103	0.001759	0.004103	CXorf40A	protein_cc
ENSG0000(	429.7378	-0.0386	0.092645	-0.41664	0.67694	0.751234	TLR1	protein_cc
ENSG0000(	429.6428	-1.24224	0.152203	-8.16173	3.30E-16	6.16E-15	NEUROD2	protein_cc
ENSG0000(	429.3717	-0.08707	0.04093	-2.12737	0.033389	0.059053	CCNJ	protein_cc
ENSG0000(	429.337	0.062124	0.040375	1.538661	0.123887	0.185681	SCLT1	protein_cc
ENSG0000(	429.0908	-0.63318	0.066737	-9.4877	2.36E-21	1.02E-19	SH3BP5	protein_cc
ENSG0000(	428.7753	0.307093	0.051398	5.974802	2.30E-09	1.42E-08	GPS2	protein_cc
ENSG0000(	428.682	0.11443	0.088173	1.297788	0.19436	0.272497	ZNF662	protein_cc
ENSG0000(	428.6792	-0.01028	0.0545	-0.18862	0.850387	0.891784	PLEKHM1I	pseudoge
ENSG0000(	428.4238	0.288796	0.069695	4.143707	3.42E-05	0.000108	MECOM	protein_cc
ENSG0000(	428.3238	0.161991	0.034622	4.678849	2.88E-06	1.08E-05	ZNF595	protein_cc
ENSG0000(	428.2655	0.104699	0.077454	1.351754	0.176454	0.251064	LAMB3	protein_cc
ENSG0000(	427.9961	-0.0675	0.031511	-2.14199	0.032194	0.057146	PCGF6	protein_cc
ENSG0000(	427.7527	0.25423	0.043825	5.800975	6.59E-09	3.77E-08	ARSK	protein_cc
ENSG0000(	427.7346	-0.14902	0.064651	-2.30491	0.021172	0.039196	SH3BP5-A	antisense
ENSG0000(	427.5491	0.118272	0.035382	3.342754	0.00083	0.002052	HNRNPA1	protein_cc
ENSG0000(	427.4831	-0.56597	0.134833	-4.19754	2.70E-05	8.68E-05	SLC5A11	protein_cc
ENSG0000(	427.2788	0.185628	0.0373	4.976677	6.47E-07	2.70E-06	PIGL	protein_cc
ENSG0000(	427.0447	0.770069	0.128153	6.008992	1.87E-09	1.16E-08	ADAM33	protein_cc
ENSG0000(	426.974	-0.17811	0.031371	-5.67774	1.36E-08	7.41E-08	SLC7A6OS	protein_cc
ENSG0000(	426.7235	-0.21684	0.094798	-2.28741	0.022172	0.040882	EDN3	protein_cc
ENSG0000(	426.535	0.221525	0.047432	4.670334	3.01E-06	1.13E-05	MASTL	protein_cc
ENSG0000(	426.4692	0.20683	0.055384	3.734451	0.000188	0.000524	LINC0026!	lincRNA
ENSG0000(	426.3856	-0.01542	0.069739	-0.22111	0.825007	0.871681	WTIP	protein_cc
ENSG0000(	426.3814	0.252028	0.0325	7.754643	8.86E-15	1.30E-13	FAM103A:	protein_cc
ENSG0000(	426.3305	-0.25494	0.062	-4.11203	3.92E-05	0.000123	FAM174B	protein_cc

ENSG0000(	426.2754	-0.16075	0.05622	-2.85931	0.004246	0.009184	EIF5A2	protein_cc
ENSG0000(	425.599	0.46856	0.048816	9.598467	8.11E-22	3.77E-20	PRIM1	protein_cc
ENSG0000(	425.5701	0.008927	0.059099	0.151051	0.879935	0.914169	TTC21A	protein_cc
ENSG0000(	425.3834	0.253881	0.047772	5.314438	1.07E-07	5.05E-07	C6orf226	protein_cc
ENSG0000(	425.2469	0.056221	0.034696	1.620386	0.105149	0.161313	ZNF449	protein_cc
ENSG0000(	424.6205	0.445478	0.053243	8.366826	5.92E-17	1.25E-15	C18orf54	protein_cc
ENSG0000(	424.2749	-0.00012	0.029684	-0.00402	0.996794	0.997983	RP11-884	antisense
ENSG0000(	424.0784	0.901636	0.116843	7.716665	1.19E-14	1.71E-13	BUB1B	protein_cc
ENSG0000(	424.0376	0.228955	0.034399	6.655932	2.82E-11	2.33E-10	ASB16-AS	antisense
ENSG0000(	423.814	0.048024	0.023203	2.069786	0.038472	0.067057	NSUN3	protein_cc
ENSG0000(	423.7599	-0.01296	0.035984	-0.36008	0.718787	0.785883	ZNF546	protein_cc
ENSG0000(	423.4783	0.129443	0.031793	4.071362	4.67E-05	0.000145	TUBD1	protein_cc
ENSG0000(	423.4756	-0.34837	0.104688	-3.32775	0.000876	0.002157	JAK3	protein_cc
ENSG0000(	423.0475	0.266901	0.032404	8.236732	1.77E-16	3.46E-15	ZNF527	protein_cc
ENSG0000(	423.0465	-0.70261	0.1045	-6.72352	1.77E-11	1.51E-10	TSHZ2	protein_cc
ENSG0000(	422.7907	-0.01686	0.086979	-0.19379	0.846338	0.888628	PRAM1	protein_cc
ENSG0000(	422.6254	-0.16101	0.083784	-1.92167	0.054647	0.091339	RGL3	protein_cc
ENSG0000(	422.6181	-0.36518	0.086935	-4.20067	2.66E-05	8.57E-05	PDE10A	protein_cc
ENSG0000(	422.532	0.0615	0.031669	1.941939	0.052144	0.087681	DET1	protein_cc
ENSG0000(	422.5227	0.075913	0.036482	2.08084	0.037449	0.065458	KBTBD3	protein_cc
ENSG0000(	422.1962	0.288075	0.040874	7.047968	1.82E-12	1.82E-11	FADD	protein_cc
ENSG0000(	422.1352	-0.039	0.051924	-0.751	0.452651	0.54932	FAM66C	antisense
ENSG0000(	422.1296	-0.18794	0.094033	-1.99863	0.045648	0.077955	RP11-158	antisense
ENSG0000(	422.1046	-0.48425	0.09774	-4.95447	7.25E-07	3.00E-06	C10orf35	protein_cc
ENSG0000(	421.7491	0.207762	0.040015	5.192153	2.08E-07	9.36E-07	AVEN	protein_cc
ENSG0000(	421.7307	0.375076	0.060225	6.227889	4.73E-10	3.24E-09	MFI2-AS1	antisense
ENSG0000(	421.6587	0.287302	0.051397	5.589911	2.27E-08	1.19E-07	MIR4453	lincRNA
ENSG0000(	421.6169	0.157833	0.042484	3.715119	0.000203	0.000562	ZNF551	protein_cc
ENSG0000(	421.6048	0.300718	0.053078	5.665558	1.47E-08	7.92E-08	SLC38A6	protein_cc
ENSG0000(	421.4531	0.208947	0.039992	5.224759	1.74E-07	7.95E-07	CYP2R1	protein_cc
ENSG0000(	421.2903	-0.00468	0.05301	-0.08827	0.92966	0.95065	DDO	protein_cc
ENSG0000(	421.2168	0.200404	0.036714	5.458463	4.80E-08	2.39E-07	ZNF773	protein_cc
ENSG0000(	421.1599	0.356579	0.055234	6.455832	1.08E-10	8.16E-10	TRIM45	protein_cc
ENSG0000(	421.1075	-0.23966	0.076091	-3.14961	0.001635	0.003834	RASAL3	protein_cc
ENSG0000(	420.6457	-0.01155	0.076076	-0.15178	0.879357	0.913799	ADAMTS5	protein_cc
ENSG0000(	420.1525	0.953069	0.1069	8.915516	4.86E-19	1.42E-17	NCAPH	protein_cc
ENSG0000(	419.9971	0.448548	0.07183	6.244555	4.25E-10	2.93E-09	TAPSAR1	lincRNA
ENSG0000(	419.8138	-0.12479	0.060437	-2.06482	0.03894	0.067749	ZMYND15	protein_cc
ENSG0000(	419.7323	-0.37881	0.068191	-5.55507	2.78E-08	1.44E-07	EFNA3	protein_cc
ENSG0000(	419.5734	-0.07169	0.030231	-2.37146	0.017718	0.03337	UBE2V1	protein_cc
ENSG0000(	419.5314	0.748682	0.076729	9.757469	1.71E-22	9.09E-21	FANCA	protein_cc
ENSG0000(	419.3726	0.163024	0.026185	6.225951	4.79E-10	3.28E-09	BCDIN3D	protein_cc
ENSG0000(	419.2976	0.149217	0.039997	3.730678	0.000191	0.000531	IRAK1BP1	protein_cc
ENSG0000(	419.2063	0.016163	0.061521	0.262726	0.792762	0.846442	hsa-mir-1	protein_cc
ENSG0000(	419.0744	0.214671	0.080475	2.667545	0.007641	0.015676	AJUBA	protein_cc
ENSG0000(	419.0549	-0.37571	0.090577	-4.14799	3.35E-05	0.000106	EMB	protein_cc
ENSG0000(	418.4361	-0.41625	0.078888	-5.27654	1.32E-07	6.12E-07	TNRC6C-/	processed
ENSG0000(	418.373	-1.21852	0.134497	-9.05985	1.31E-19	4.19E-18	CNNM1	protein_cc
ENSG0000(	418.3096	0.209421	0.073902	2.833758	0.0046	0.009866	ADPRH	protein_cc
ENSG0000(	417.9336	-0.1375	0.071288	-1.92881	0.053754	0.090015	MTMR9LP	pseudoge
ENSG0000(	417.8921	0.310582	0.031161	9.967034	2.12E-23	1.37E-21	ZNF101	protein_cc
ENSG0000(	417.8329	-0.11815	0.056077	-2.10684	0.035131	0.061852	RP11-124	lincRNA
ENSG0000(	417.8002	0.003522	0.04433	0.079458	0.936669	0.955773	ZNRD1-A:	antisense
ENSG0000(	417.2329	-0.14595	0.047052	-3.10185	0.001923	0.00445	ATP6AP1L	protein_cc
ENSG0000(	417.1631	0.028915	0.043171	0.66978	0.502998	0.596892	FAM117A	protein_cc
ENSG0000(	417.0445	0.507316	0.129987	3.902836	9.51E-05	0.000279	CELSR1	protein_cc
ENSG0000(	416.9543	-0.23893	0.140143	-1.70487	0.088219	0.138627	HMX1	protein_cc
ENSG0000(	416.6686	-0.24581	0.223782	-1.09845	0.272009	0.361752	ZFY	protein_cc
ENSG0000(	416.6045	-0.31925	0.059174	-5.39508	6.85E-08	3.33E-07	GIMAP1	protein_cc

ENSG0000(	416.1977	0.200757	0.038777	5.177211	2.25E-07	1.01E-06	KRT8P12	pseudoge
ENSG0000(	416.1095	0.254989	0.085944	2.966931	0.003008	0.006702	SLC25A24	protein_cc
ENSG0000(	416.0586	0.191154	0.048229	3.963431	7.39E-05	0.000221	C21orf119	protein_cc
ENSG0000(	415.9214	0.238645	0.070921	3.364923	0.000766	0.001906	CLEC11A	protein_cc
ENSG0000(	415.4925	0.98933	0.11375	8.697412	3.40E-18	8.74E-17	GTSE1	protein_cc
ENSG0000(	414.3793	1.147807	0.131171	8.750462	2.12E-18	5.65E-17	KIF20A	protein_cc
ENSG0000(	414.24	-0.15339	0.045932	-3.33944	0.000839	0.002076	ARID3A	protein_cc
ENSG0000(	413.7893	0.086128	0.064803	1.329071	0.183824	0.259833	RP11-715.	sense_intr
ENSG0000(	413.7637	0.010021	0.036659	0.273365	0.784572	0.83955	COQ3	protein_cc
ENSG0000(	413.4409	-0.41145	0.067463	-6.09893	1.07E-09	6.93E-09	CTD-2514	antisense
ENSG0000(	413.3138	-1.02761	0.158784	-6.47173	9.69E-11	7.40E-10	SCN7A	protein_cc
ENSG0000(	413.2422	-0.32933	0.067567	-4.87413	1.09E-06	4.39E-06	TM6SF1	protein_cc
ENSG0000(	413.2165	0.160612	0.034908	4.601017	4.20E-06	1.54E-05	ZNF132	protein_cc
ENSG0000(	413.1864	-0.10309	0.105243	-0.97955	0.327307	0.421619	GPR183	protein_cc
ENSG0000(	413.0564	0.161529	0.067528	2.392035	0.016755	0.031787	ADA	protein_cc
ENSG0000(	413.0558	0.36683	0.034983	10.48602	1.00E-25	1.02E-23	TIPIN	protein_cc
ENSG0000(	412.9906	-0.35736	0.102527	-3.48557	0.000491	0.001269	DCHS2	protein_cc
ENSG0000(	412.9693	0.212074	0.036097	5.875155	4.22E-09	2.49E-08	NBPF11	protein_cc
ENSG0000(	412.7883	-0.28031	0.051886	-5.40253	6.57E-08	3.21E-07	RP11-545	antisense
ENSG0000(	412.4929	-0.05959	0.037567	-1.5862	0.112693	0.17127	C15orf61	protein_cc
ENSG0000(	412.299	0.216465	0.033803	6.40365	1.52E-10	1.12E-09	RP11-139	antisense
ENSG0000(	412.2421	-1.13428	0.130486	-8.6927	3.54E-18	9.07E-17	ISLR2	protein_cc
ENSG0000(	412.1582	1.014751	0.137156	7.398522	1.38E-13	1.64E-12	PDGFD	protein_cc
ENSG0000(	412.1114	-0.81687	0.087115	-9.37695	6.79E-21	2.67E-19	RP13-514	sense_ove
ENSG0000(	412.0332	-0.07108	0.032546	-2.18401	0.028961	0.051991	AC027763	protein_cc
ENSG0000(	411.6941	-0.26935	0.065517	-4.11112	3.94E-05	0.000123	GIMAP7	protein_cc
ENSG0000(	411.4905	-0.08813	0.048275	-1.8255	0.067925	0.110353	GNL3L	protein_cc
ENSG0000(	411.1708	0.162492	0.077845	2.087392	0.036853	0.064521	PROCR	protein_cc
ENSG0000(	411.0029	0.157572	0.036744	4.288394	1.80E-05	5.97E-05	ZBTB8A	protein_cc
ENSG0000(	410.9391	-0.99056	0.142297	-6.9612	3.37E-12	3.24E-11	CALB2	protein_cc
ENSG0000(	410.4995	-0.20832	0.072704	-2.86528	0.004166	0.009032	LYL1	protein_cc
ENSG0000(	410.4231	0.004829	0.038067	0.126848	0.899061	0.928418	TXNRD3	protein_cc
ENSG0000(	410.2816	-0.0771	0.038609	-1.99695	0.045831	0.078222	ZNF611	protein_cc
ENSG0000(	410.2671	0.044747	0.039558	1.131166	0.257985	0.345909	ZNF511	protein_cc
ENSG0000(	410.1883	-0.6232	0.087157	-7.15031	8.66E-13	9.09E-12	NR4A2	protein_cc
ENSG0000(	410.1598	-0.11196	0.047356	-2.36432	0.018063	0.033973	SMYD3	protein_cc
ENSG0000(	409.9118	-1.63828	0.14961	-10.9504	6.62E-28	9.86E-26	FRMPD4	protein_cc
ENSG0000(	409.8719	-0.1723	0.083366	-2.06679	0.038754	0.067462	GATA2	protein_cc
ENSG0000(	409.0462	-0.55425	0.117982	-4.6978	2.63E-06	9.94E-06	RP11-54C	lincRNA
ENSG0000(	408.9071	-0.12468	0.052179	-2.38945	0.016874	0.03199	SFXN2	protein_cc
ENSG0000(	408.4279	-0.0932	0.038549	-2.41763	0.015622	0.029853	L3MBTL3	protein_cc
ENSG0000(	408.2246	0.112875	0.055548	2.032032	0.04215	0.072661	FKBP7	protein_cc
ENSG0000(	407.8212	0.274286	0.119594	2.293475	0.021821	0.040283	SERINC2	protein_cc
ENSG0000(	407.7253	-0.39552	0.05496	-7.19653	6.18E-13	6.62E-12	ATP7B	protein_cc
ENSG0000(	407.6917	-1.48231	0.145125	-10.214	1.72E-24	1.36E-22	CBLN2	protein_cc
ENSG0000(	407.5772	0.206402	0.08002	2.579389	0.009898	0.019795	APOBR	protein_cc
ENSG0000(	407.3218	1.263873	0.259279	4.874568	1.09E-06	4.38E-06	PSPHP1	pseudoge
ENSG0000(	407.0702	-0.08235	0.077108	-1.06805	0.285498	0.376368	SLCO4A1	protein_cc
ENSG0000(	407.0232	0.106215	0.053741	1.976435	0.048106	0.081609	CLYBL	protein_cc
ENSG0000(	406.968	0.652833	0.107844	6.053508	1.42E-09	9.02E-09	XAF1	protein_cc
ENSG0000(	406.911	0.242586	0.05768	4.205759	2.60E-05	8.39E-05	RRN3P1	pseudoge
ENSG0000(	406.8187	0.529115	0.12433	4.255722	2.08E-05	6.84E-05	ZDHHC23	protein_cc
ENSG0000(	406.7504	-0.67816	0.09201	-7.37056	1.70E-13	1.99E-12	PNMA6C	protein_cc
ENSG0000(	406.4431	0.690762	0.168948	4.088593	4.34E-05	0.000135	IGF2BP2	protein_cc
ENSG0000(	406.428	0.894045	0.126829	7.049218	1.80E-12	1.80E-11	MAP3K7C	protein_cc
ENSG0000(	406.2673	0.081745	0.031065	2.631463	0.008502	0.017266	ZNF354B	protein_cc
ENSG0000(	406.2488	-0.34062	0.049837	-6.83478	8.21E-12	7.39E-11	ZNF774	protein_cc
ENSG0000(	406.2378	0.308017	0.037145	8.292257	1.11E-16	2.24E-15	ZNF596	protein_cc
ENSG0000(	405.8895	0.002809	0.056805	0.049445	0.960565	0.972986	ZNF737	protein_cc

ENSG0000(	405.6609	0.3377	0.063942	5.281349	1.28E-07	5.97E-07	CEP135	protein_cc
ENSG0000(	405.3697	-1.09053	0.134363	-8.11626	4.81E-16	8.76E-15	MKX	protein_cc
ENSG0000(	405.139	-0.38841	0.095633	-4.06141	4.88E-05	0.00015	POLR2F	protein_cc
ENSG0000(	405.0298	-0.03196	0.055025	-0.58081	0.56137	0.650126	PI4KAP2	pseudoge
ENSG0000(	405.0272	0.30375	0.081777	3.714372	0.000204	0.000564	PLEKHF1	protein_cc
ENSG0000(	404.9201	0.116663	0.047752	2.443093	0.014562	0.028056	ADCK5	protein_cc
ENSG0000(	404.8949	0.14532	0.033982	4.276445	1.90E-05	6.27E-05	PINX1	protein_cc
ENSG0000(	404.8452	0.037776	0.040076	0.942609	0.345881	0.441287	ZNF285	protein_cc
ENSG0000(	404.6579	0.371871	0.070468	5.277173	1.31E-07	6.10E-07	FZD5	protein_cc
ENSG0000(	404.5455	0.244684	0.035337	6.924258	4.38E-12	4.12E-11	ZNF35	protein_cc
ENSG0000(	404.5307	-0.22765	0.089457	-2.54481	0.010934	0.021659	ANKRD65	protein_cc
ENSG0000(	404.5057	-0.36541	0.043784	-8.34587	7.07E-17	1.47E-15	WDR31	protein_cc
ENSG0000(	404.2813	-0.72259	0.126048	-5.73268	9.89E-09	5.49E-08	BCL11A	protein_cc
ENSG0000(	404.21	-0.28	0.053492	-5.23451	1.65E-07	7.57E-07	RP11-309	lincRNA
ENSG0000(	404.094	0.242212	0.179665	1.348131	0.177616	0.252481	SLN	protein_cc
ENSG0000(	403.9713	0.165014	0.044862	3.678283	0.000235	0.000643	ANKRD32	protein_cc
ENSG0000(	403.8453	0.275777	0.073842	3.734702	0.000188	0.000523	GPR4	protein_cc
ENSG0000(	403.6198	0.138374	0.045351	3.051146	0.00228	0.005204	WASH6P	pseudoge
ENSG0000(	403.55	0.529922	0.054086	9.797817	1.15E-22	6.27E-21	SMIM4	protein_cc
ENSG0000(	403.0739	0.088109	0.029047	3.033352	0.002419	0.005499	XRCC4	protein_cc
ENSG0000(	403.0318	0.033649	0.098844	0.34043	0.733533	0.7981	GULP1	protein_cc
ENSG0000(	402.8407	-0.02844	0.111595	-0.25488	0.798819	0.851261	SEMA3F	protein_cc
ENSG0000(	402.2276	0.013237	0.040639	0.325721	0.744636	0.80731	ZNF141	protein_cc
ENSG0000(	402.1553	0.362098	0.075457	4.798716	1.60E-06	6.25E-06	N6AMT2	protein_cc
ENSG0000(	401.9061	0.348845	0.067906	5.137167	2.79E-07	1.23E-06	RP11-420	antisense
ENSG0000(	401.8544	0.387771	0.068256	5.681117	1.34E-08	7.28E-08	CCDC74A	protein_cc
ENSG0000(	401.7338	0.623854	0.061823	10.09102	6.05E-24	4.34E-22	CENPN	protein_cc
ENSG0000(	401.6764	0.1271	0.081854	1.55278	0.120476	0.181295	VILL	protein_cc
ENSG0000(	401.6411	-0.79942	0.093073	-8.58912	8.76E-18	2.12E-16	MACROD2	protein_cc
ENSG0000(	401.5741	0.377949	0.079264	4.768227	1.86E-06	7.20E-06	ABRACL	protein_cc
ENSG0000(	401.1969	0.228185	0.03616	6.310416	2.78E-10	1.98E-09	SPDYE3	protein_cc
ENSG0000(	401.082	-1.13528	0.161776	-7.01759	2.26E-12	2.22E-11	SLC32A1	protein_cc
ENSG0000(	401.0782	-0.60406	0.117282	-5.15048	2.60E-07	1.15E-06	CABP7	protein_cc
ENSG0000(	400.8717	-0.32892	0.143893	-2.28587	0.022262	0.04103	C1QL4	protein_cc
ENSG0000(	400.869	-0.20568	0.050792	-4.04954	5.13E-05	0.000158	ZNF843	protein_cc
ENSG0000(	400.8307	-0.58258	0.099404	-5.86071	4.61E-09	2.69E-08	KIAA1324	protein_cc
ENSG0000(	400.6624	-0.12601	0.055301	-2.27858	0.022692	0.041742	LIN7B	protein_cc
ENSG0000(	400.5984	0.090598	0.064504	1.404521	0.160164	0.231254	PRDM1	protein_cc
ENSG0000(	400.5958	-0.42176	0.06647	-6.34516	2.22E-10	1.60E-09	NRIP2	protein_cc
ENSG0000(	400.5316	0.334784	0.071573	4.677489	2.90E-06	1.09E-05	FAM211A	protein_cc
ENSG0000(	400.4187	0.015837	0.085176	0.18593	0.8525	0.893345	PERP	protein_cc
ENSG0000(	400.3884	0.286023	0.052376	5.460995	4.73E-08	2.36E-07	FGGY	protein_cc
ENSG0000(	400.2523	-0.3148	0.082269	-3.82645	0.00013	0.000372	CDH23	protein_cc
ENSG0000(	400.1106	-0.11272	0.037206	-3.02961	0.002449	0.005562	PGBD4	protein_cc
ENSG0000(	399.8245	-1.1299	0.134811	-8.38132	5.23E-17	1.12E-15	SLITRK4	protein_cc
ENSG0000(	399.7884	-0.37027	0.114822	-3.22468	0.001261	0.003017	FAM133A	protein_cc
ENSG0000(	399.7176	-0.08733	0.111129	-0.78586	0.431948	0.528927	SOX3	protein_cc
ENSG0000(	399.2962	0.04238	0.098987	0.428131	0.668556	0.744118	LRRC25	protein_cc
ENSG0000(	399.2619	-0.41421	0.057077	-7.25699	3.96E-13	4.37E-12	ARHGAP5	antisense
ENSG0000(	399.212	0.129021	0.066183	1.94945	0.051242	0.086338	ASGR1	protein_cc
ENSG0000(	399.017	0.216068	0.047252	4.572698	4.81E-06	1.75E-05	C9orf40	protein_cc
ENSG0000(	398.9954	0.669112	0.108373	6.174132	6.65E-10	4.46E-09	ENPEP	protein_cc
ENSG0000(	398.9262	-0.14256	0.065381	-2.18051	0.029219	0.052409	RRP7B	pseudoge
ENSG0000(	398.632	-1.24687	0.122233	-10.2008	1.97E-24	1.53E-22	COL26A1	polymorpl
ENSG0000(	398.6311	0.723657	0.091012	7.951255	1.85E-15	3.06E-14	EEF1A1P1	pseudoge
ENSG0000(	398.483	-0.57648	0.098136	-5.87431	4.25E-09	2.50E-08	ZNF204P	pseudoge
ENSG0000(	398.4309	0.814914	0.071347	11.42177	3.26E-30	7.65E-28	SGOL2	protein_cc
ENSG0000(	398.3511	0.16461	0.053036	3.103741	0.001911	0.004424	CTH	protein_cc
ENSG0000(	398.2925	-0.13594	0.033019	-4.1169	3.84E-05	0.00012	ZNF846	protein_cc

ENSG0000(	397.6662	1.038809	0.121292	8.564495	1.09E-17	2.58E-16	RP11-234.	pseudoge
ENSG0000(	397.5103	-0.10857	0.040402	-2.68724	0.007204	0.014847	RBM41	protein_cc
ENSG0000(	397.3243	-0.52897	0.158099	-3.34585	0.00082	0.002031	TMEM235	protein_cc
ENSG0000(	397.0878	0.831142	0.134116	6.197175	5.75E-10	3.88E-09	FBLN7	protein_cc
ENSG0000(	397.0871	0.072021	0.05257	1.369995	0.170689	0.24417	ZNF891	protein_cc
ENSG0000(	397.0314	-1.23154	0.15409	-7.99237	1.32E-15	2.25E-14	LRTM2	protein_cc
ENSG0000(	396.8345	-0.35057	0.060965	-5.75029	8.91E-09	4.98E-08	USP51	protein_cc
ENSG0000(	396.8055	0.530263	0.083171	6.375559	1.82E-10	1.33E-09	NFE2L3	protein_cc
ENSG0000(	396.4504	-0.23189	0.06488	-3.57406	0.000351	0.000935	PEAR1	protein_cc
ENSG0000(	396.3464	-0.02091	0.059175	-0.35339	0.723799	0.78983	AF131215	sense_intr
ENSG0000(	396.2455	1.328717	0.120209	11.05341	2.11E-28	3.58E-26	KIAA0101	protein_cc
ENSG0000(	395.9967	0.040444	0.053537	0.755437	0.449987	0.546814	NOSTRIN	protein_cc
ENSG0000(	395.9414	-0.63814	0.135391	-4.71333	2.44E-06	9.27E-06	SLC1A6	protein_cc
ENSG0000(	395.5804	0.129684	0.042452	3.054851	0.002252	0.005146	ZNF418	protein_cc
ENSG0000(	395.4195	0.209137	0.042103	4.967327	6.79E-07	2.82E-06	HSD17B7	protein_cc
ENSG0000(	395.3351	0.45113	0.048017	9.395238	5.71E-21	2.29E-19	MED31	protein_cc
ENSG0000(	395.0532	0.116416	0.048217	2.414422	0.01576	0.030093	FBF1	protein_cc
ENSG0000(	395.0057	0.258389	0.051881	4.98038	6.35E-07	2.65E-06	RP11-421	antisense
ENSG0000(	394.8448	0.075708	0.073216	1.034024	0.301125	0.393713	RP11-120.	antisense
ENSG0000(	394.763	-0.62571	0.118979	-5.259	1.45E-07	6.68E-07	EPHA10	protein_cc
ENSG0000(	394.37	0.068586	0.02517	2.724913	0.006432	0.013395	ABHD16A	protein_cc
ENSG0000(	394.162	-0.14286	0.069149	-2.066	0.038828	0.067579	RARB	protein_cc
ENSG0000(	394.1132	-0.31896	0.105237	-3.03092	0.002438	0.005539	LGI2	protein_cc
ENSG0000(	393.9675	0.253819	0.044306	5.728774	1.01E-08	5.60E-08	ZNF582-A	lincRNA
ENSG0000(	393.8276	0.981961	0.125668	7.813919	5.54E-15	8.46E-14	ARSJ	protein_cc
ENSG0000(	393.7804	0.083087	0.055187	1.50554	0.132185	0.196217	RDH13	protein_cc
ENSG0000(	393.7645	0.450473	0.088177	5.10873	3.24E-07	1.42E-06	PDIA5	protein_cc
ENSG0000(	393.6447	0.51781	0.062404	8.297649	1.06E-16	2.15E-15	WDHD1	protein_cc
ENSG0000(	393.4239	0.422563	0.07332	5.763248	8.25E-09	4.63E-08	CASP8	protein_cc
ENSG0000(	393.4159	0.063988	0.07807	0.819623	0.412431	0.509368	RAB27A	protein_cc
ENSG0000(	392.9266	0.213692	0.042955	4.974727	6.53E-07	2.72E-06	C5orf56	protein_cc
ENSG0000(	392.3156	-0.2151	0.08249	-2.60758	0.009118	0.018376	CMTM7	protein_cc
ENSG0000(	392.2594	-0.09257	0.13163	-0.70325	0.481898	0.577099	FPR3	protein_cc
ENSG0000(	391.9691	-0.01659	0.033069	-0.50172	0.615865	0.698875	RPL32P3	pseudoge
ENSG0000(	391.3322	-0.24292	0.087707	-2.7697	0.005611	0.011829	BLNK	protein_cc
ENSG0000(	390.8729	-0.10646	0.070554	-1.50885	0.131337	0.195113	SLC22A4	protein_cc
ENSG0000(	390.5985	-0.05066	0.050976	-0.99389	0.320274	0.414064	XRRA1	protein_cc
ENSG0000(	390.1696	-0.94785	0.122568	-7.73325	1.05E-14	1.52E-13	SPTB	protein_cc
ENSG0000(	389.7449	0.152632	0.068974	2.212889	0.026905	0.048637	BRICD5	protein_cc
ENSG0000(	389.7013	0.0127	0.034628	0.366746	0.713808	0.781748	ZBTB3	protein_cc
ENSG0000(	389.5021	-0.17641	0.046417	-3.80058	0.000144	0.00041	SLC35E4	protein_cc
ENSG0000(	389.2917	-0.15266	0.074218	-2.05687	0.039699	0.068927	KCNJ2	protein_cc
ENSG0000(	389.2481	0.001138	0.041803	0.027226	0.97828	0.984697	FUT10	protein_cc
ENSG0000(	389.1531	-0.20855	0.044456	-4.69121	2.72E-06	1.02E-05	AMER1	protein_cc
ENSG0000(	388.9631	0.061594	0.054341	1.133474	0.257015	0.344901	TMCC1-A	antisense
ENSG0000(	388.434	1.026842	0.119307	8.606709	7.52E-18	1.84E-16	KIF23	protein_cc
ENSG0000(	388.1117	1.267658	0.130194	9.73667	2.10E-22	1.09E-20	MELK	protein_cc
ENSG0000(	387.7426	0.010168	0.032403	0.313804	0.75367	0.81443	POP1	protein_cc
ENSG0000(	387.6972	-0.42768	0.084673	-5.05094	4.40E-07	1.88E-06	RAB26	protein_cc
ENSG0000(	387.6363	0.354905	0.043528	8.153583	3.53E-16	6.57E-15	ZNF486	protein_cc
ENSG0000(	387.0346	0.337136	0.072956	4.621062	3.82E-06	1.41E-05	CEP112	protein_cc
ENSG0000(	386.9908	0.12712	0.054661	2.325622	0.020039	0.037283	EIF3C	protein_cc
ENSG0000(	386.9735	0.136544	0.033443	4.082894	4.45E-05	0.000138	C15orf41	protein_cc
ENSG0000(	386.5701	-0.17596	0.064106	-2.7448	0.006055	0.012671	ADCY10P1	pseudoge
ENSG0000(	386.5286	0.651783	0.104278	6.250422	4.09E-10	2.83E-09	NMNAT3	protein_cc
ENSG0000(	386.4687	0.733473	0.095056	7.716196	1.20E-14	1.72E-13	CHAF1B	protein_cc
ENSG0000(	386.2789	0.283262	0.063478	4.462382	8.11E-06	2.84E-05	RP11-458	lincRNA
ENSG0000(	386.1655	0.004396	0.085743	0.051275	0.959107	0.97206	CD302	protein_cc
ENSG0000(	386.0339	-0.50853	0.071897	-7.07298	1.52E-12	1.53E-11	PRKG1	protein_cc

ENSG0000(	386.0172	-0.38355	0.130331	-2.94286	0.003252	0.007197	PCP4	protein_cc
ENSG0000(	385.9948	-0.20364	0.061382	-3.31759	0.000908	0.002229	AF131215	sense_intr
ENSG0000(	385.9491	-0.49882	0.092302	-5.40423	6.51E-08	3.18E-07	IL34	protein_cc
ENSG0000(	385.8189	-0.53206	0.103353	-5.14795	2.63E-07	1.17E-06	CAPN3	protein_cc
ENSG0000(	385.6708	0.684669	0.082074	8.342122	7.30E-17	1.52E-15	TMEM159	protein_cc
ENSG0000(	385.6391	-0.01296	0.070107	-0.18487	0.853332	0.893847	CARD9	protein_cc
ENSG0000(	385.5032	0.460907	0.136635	3.373285	0.000743	0.001853	FBN3	protein_cc
ENSG0000(	385.282	0.251345	0.074183	3.388166	0.000704	0.001764	NME5	protein_cc
ENSG0000(	385.2729	0.125328	0.034005	3.685561	0.000228	0.000626	AC025335	antisense
ENSG0000(	385.224	-0.07184	0.045713	-1.57166	0.116029	0.175683	EID2B	protein_cc
ENSG0000(	385.2031	0.082385	0.049956	1.649145	0.099118	0.153358	C1orf213	protein_cc
ENSG0000(	385.1773	0.231574	0.074046	3.12745	0.001763	0.004111	LRRC34	protein_cc
ENSG0000(	384.9855	0.251403	0.058897	4.268542	1.97E-05	6.48E-05	AC004381	protein_cc
ENSG0000(	384.9522	0.960662	0.091209	10.53255	6.12E-26	6.47E-24	AURKA	protein_cc
ENSG0000(	384.9368	-0.01802	0.082432	-0.21862	0.826945	0.87319	TNFAIP8L2	protein_cc
ENSG0000(	384.765	0.171098	0.03708	4.614303	3.94E-06	1.45E-05	PARS2	protein_cc
ENSG0000(	384.1696	0.660591	0.074745	8.837974	9.75E-19	2.71E-17	CDCA4	protein_cc
ENSG0000(	383.893	-0.43822	0.094591	-4.63279	3.61E-06	1.34E-05	JPH2	protein_cc
ENSG0000(	383.6456	-0.12504	0.049128	-2.54518	0.010922	0.021638	EHHADH	protein_cc
ENSG0000(	383.343	0.146042	0.03737	3.907979	9.31E-05	0.000274	ZNF790	protein_cc
ENSG0000(	383.0706	-0.41636	0.07504	-5.54857	2.88E-08	1.49E-07	ATP8B4	protein_cc
ENSG0000(	382.9066	-0.08058	0.064104	-1.25705	0.208737	0.289534	DAPK2	protein_cc
ENSG0000(	382.8339	-0.12334	0.04904	-2.51503	0.011902	0.023374	PAXBP1-A	antisense
ENSG0000(	382.5073	0.559538	0.122123	4.58177	4.61E-06	1.68E-05	TCEA3	protein_cc
ENSG0000(	382.2878	0.033547	0.04211	0.79665	0.425654	0.522728	CTD-2619	lincRNA
ENSG0000(	382.1608	0.055462	0.030617	1.81151	0.070062	0.113403	SWT1	protein_cc
ENSG0000(	382.1083	-0.15991	0.037884	-4.2211	2.43E-05	7.87E-05	ZNF619	protein_cc
ENSG0000(	382.0573	-0.25971	0.083637	-3.10519	0.001902	0.004405	GNA15	protein_cc
ENSG0000(	382.0191	0.068062	0.066964	1.016388	0.309444	0.402607	IKBKE	protein_cc
ENSG0000(	381.6194	1.241354	0.118834	10.44612	1.53E-25	1.48E-23	NDC80	protein_cc
ENSG0000(	381.6001	-1.06356	0.148818	-7.1467	8.89E-13	9.32E-12	SST	protein_cc
ENSG0000(	381.2884	-0.10155	0.094839	-1.07075	0.28428	0.374967	FZD8	protein_cc
ENSG0000(	381.2606	-0.16596	0.124899	-1.32873	0.183938	0.259968	RP5-857K	pseudoge
ENSG0000(	381.1981	0.790785	0.113719	6.953868	3.55E-12	3.39E-11	CASC5	protein_cc
ENSG0000(	381.1253	-0.0666	0.072309	-0.92103	0.357034	0.452763	GSDMB	protein_cc
ENSG0000(	381.1063	-0.0163	0.056242	-0.28978	0.771985	0.829391	C17orf97	protein_cc
ENSG0000(	381.0698	0.094539	0.112867	0.83762	0.402244	0.499016	NAT16	protein_cc
ENSG0000(	381.0336	1.188751	0.102892	11.55338	7.10E-31	1.97E-28	NUF2	protein_cc
ENSG0000(	380.993	0.207453	0.040261	5.152655	2.57E-07	1.14E-06	ZNF322	protein_cc
ENSG0000(	380.8592	0.190812	0.066356	2.875562	0.004033	0.008767	PPP1R14B	pseudoge
ENSG0000(	380.8314	1.256143	0.124919	10.05567	8.67E-24	6.07E-22	HJURP	protein_cc
ENSG0000(	380.7381	-0.48848	0.130013	-3.75715	0.000172	0.000482	CALB1	protein_cc
ENSG0000(	380.7286	-0.52152	0.06281	-8.30304	1.01E-16	2.06E-15	TESK2	protein_cc
ENSG0000(	380.5338	0.171566	0.035745	4.799704	1.59E-06	6.23E-06	IQCH-AS1	lincRNA
ENSG0000(	380.2761	-0.48191	0.061389	-7.85007	4.16E-15	6.48E-14	CASC2	antisense
ENSG0000(	380.2613	-1.42277	0.136233	-10.4436	1.57E-25	1.51E-23	SH2D5	protein_cc
ENSG0000(	379.8911	-0.10783	0.107633	-1.00185	0.316414	0.41004	RP11-552	lincRNA
ENSG0000(	379.7845	0.290477	0.082611	3.516192	0.000438	0.001143	MBNL3	protein_cc
ENSG0000(	379.6613	-0.16344	0.036624	-4.46264	8.10E-06	2.83E-05	AC007246	antisense
ENSG0000(	379.5929	0.589	0.100333	5.870433	4.35E-09	2.55E-08	APOBEC3(	protein_cc
ENSG0000(	379.5289	0.420001	0.099809	4.20803	2.58E-05	8.31E-05	OSBPL10	protein_cc
ENSG0000(	379.4719	0.550494	0.065729	8.375161	5.51E-17	1.17E-15	CBR3	protein_cc
ENSG0000(	379.3836	0.229719	0.065853	3.488375	0.000486	0.001257	HNRNPU-	antisense
ENSG0000(	379.3023	-1.14692	0.172432	-6.65145	2.90E-11	2.40E-10	TMEM132	protein_cc
ENSG0000(	379.2889	-0.19636	0.115632	-1.69816	0.089478	0.140339	CYB5R2	protein_cc
ENSG0000(	379.206	-0.33336	0.232332	-1.43483	0.151336	0.220482	TTTY15	lincRNA
ENSG0000(	379.0857	-0.03356	0.062665	-0.53558	0.592251	0.677772	PKD1P6	pseudoge
ENSG0000(	379.0617	0.195481	0.062917	3.10695	0.00189	0.004381	TTLL9	protein_cc
ENSG0000(	379.0612	0.187435	0.053767	3.486038	0.00049	0.001267	SLC25A19	protein_cc

ENSG0000(	378.8926	-0.0454	0.120783	-0.37591	0.706987	0.776387	HGF	protein_cc
ENSG0000(	378.1933	-0.51583	0.068804	-7.49714	6.52E-14	8.22E-13	RP11-113	lincRNA
ENSG0000(	378.0679	0.265812	0.050326	5.281756	1.28E-07	5.96E-07	LINC0046	processed
ENSG0000(	377.8932	0.445992	0.057886	7.704638	1.31E-14	1.86E-13	PKN3	protein_cc
ENSG0000(	377.4598	0.673469	0.159752	4.215713	2.49E-05	8.05E-05	GPX8	protein_cc
ENSG0000(	377.1474	0.238829	0.051934	4.59871	4.25E-06	1.56E-05	RP11-111	lincRNA
ENSG0000(	377.0952	0.097784	0.077894	1.255337	0.209356	0.290266	FOXF2	protein_cc
ENSG0000(	376.9215	-0.16417	0.053374	-3.07593	0.002098	0.004822	KLF11	protein_cc
ENSG0000(	376.86	-0.70473	0.137112	-5.13981	2.75E-07	1.21E-06	PTGS2	protein_cc
ENSG0000(	376.5581	-0.27515	0.077002	-3.57331	0.000353	0.000937	NABP1	protein_cc
ENSG0000(	376.5042	0.147438	0.091824	1.605653	0.10835	0.165557	LILRA2	protein_cc
ENSG0000(	376.4736	-1.04843	0.093932	-11.1616	6.29E-29	1.18E-26	DGKE	protein_cc
ENSG0000(	376.3259	-0.29403	0.082972	-3.5437	0.000395	0.001039	FAM182B	protein_cc
ENSG0000(	376.0224	0.21769	0.081588	2.668161	0.007627	0.015648	IRAK2	protein_cc
ENSG0000(	375.8484	1.089257	0.131008	8.314405	9.22E-17	1.88E-15	TROAP	protein_cc
ENSG0000(	375.1569	1.062838	0.122809	8.654406	4.95E-18	1.24E-16	H3F3AP4	pseudoge
ENSG0000(	374.9223	0.100425	0.032167	3.122019	0.001796	0.004182	CCDC142	protein_cc
ENSG0000(	374.8563	0.178246	0.040567	4.393902	1.11E-05	3.82E-05	DNAJC25	protein_cc
ENSG0000(	374.7905	-0.07366	0.031118	-2.36718	0.017924	0.033726	WDPCP	protein_cc
ENSG0000(	374.7592	0.052096	0.039965	1.303547	0.192388	0.270186	LINC0066	lincRNA
ENSG0000(	374.6554	0.081032	0.07259	1.116299	0.264294	0.353056	FAM43A	protein_cc
ENSG0000(	374.4198	0.230499	0.037179	6.199633	5.66E-10	3.83E-09	RPP21	protein_cc
ENSG0000(	374.1932	-0.58158	0.123656	-4.70322	2.56E-06	9.70E-06	MARVELD	protein_cc
ENSG0000(	373.8505	0.013803	0.030852	0.447394	0.654591	0.732515	KIAA0391	protein_cc
ENSG0000(	373.7307	1.199938	0.148339	8.089174	6.01E-16	1.07E-14	LINC0015	lincRNA
ENSG0000(	373.6634	0.333284	0.147859	2.254069	0.024192	0.044222	LOXL1	protein_cc
ENSG0000(	373.6563	-0.24386	0.060173	-4.05255	5.07E-05	0.000156	TMEM254	antisense
ENSG0000(	373.5963	0.026542	0.050878	0.521677	0.601895	0.686425	CCDC181	protein_cc
ENSG0000(	373.4448	-0.15285	0.085819	-1.78112	0.074893	0.120187	KLHL6	protein_cc
ENSG0000(	373.3986	-0.56553	0.118837	-4.7589	1.95E-06	7.52E-06	DHRS9	protein_cc
ENSG0000(	373.3912	0.057504	0.039965	1.438873	0.150186	0.219055	ZGPAT	protein_cc
ENSG0000(	373.2157	-0.17848	0.045674	-3.90781	9.31E-05	0.000274	KLHDC1	protein_cc
ENSG0000(	373.1983	-0.17937	0.054119	-3.31433	0.000919	0.002253	MATR3	protein_cc
ENSG0000(	373.0683	-0.45564	0.13976	-3.26017	0.001113	0.002689	KCNG1	protein_cc
ENSG0000(	372.8891	0.470661	0.069275	6.79409	1.09E-11	9.60E-11	PRR7	protein_cc
ENSG0000(	372.4366	0.987991	0.313518	3.151301	0.001625	0.003814	PLA2G2A	protein_cc
ENSG0000(	372.342	-0.49449	0.169736	-2.9133	0.003576	0.007852	TAC1	protein_cc
ENSG0000(	372.2646	0.147509	0.041268	3.574383	0.000351	0.000934	CBWD1	protein_cc
ENSG0000(	372.2039	-0.30363	0.089462	-3.39392	0.000689	0.00173	FAM155B	protein_cc
ENSG0000(	372.1827	-0.11404	0.051014	-2.23551	0.025384	0.046171	JHDM1D-	antisense
ENSG0000(	372.1797	0.02018	0.041208	0.489714	0.624337	0.70654	ZSCAN31	protein_cc
ENSG0000(	372.1605	-0.41477	0.139705	-2.96887	0.002989	0.006663	CBLN1	protein_cc
ENSG0000(	371.8623	0.33805	0.043145	7.835227	4.68E-15	7.23E-14	PPCDC	protein_cc
ENSG0000(	371.8559	0.150907	0.031278	4.824747	1.40E-06	5.54E-06	MCEE	protein_cc
ENSG0000(	371.8327	0.165085	0.037205	4.437143	9.12E-06	3.17E-05	PPAN	protein_cc
ENSG0000(	371.7212	0.04455	0.047967	0.928777	0.353005	0.448556	SPIN3	protein_cc
ENSG0000(	371.5172	-0.10915	0.091177	-1.19716	0.231245	0.315309	PDZRN4	protein_cc
ENSG0000(	371.3355	0.165604	0.029619	5.591162	2.26E-08	1.18E-07	WDR73	protein_cc
ENSG0000(	371.2791	-0.50414	0.083107	-6.06609	1.31E-09	8.39E-09	FBXO27	protein_cc
ENSG0000(	371.2513	0.354705	0.104036	3.409459	0.000651	0.001643	FOXD1	protein_cc
ENSG0000(	371.2501	0.339011	0.055	6.163883	7.10E-10	4.74E-09	PI4K2B	protein_cc
ENSG0000(	371.2486	1.152344	0.132275	8.711723	2.99E-18	7.76E-17	AURKB	protein_cc
ENSG0000(	371.2477	0.313356	0.078331	4.00041	6.32E-05	0.000191	DLL4	protein_cc
ENSG0000(	371.1318	0.209347	0.034356	6.093546	1.10E-09	7.16E-09	MTRF1	protein_cc
ENSG0000(	371.0452	0.445372	0.11837	3.762549	0.000168	0.000473	AMIGO2	protein_cc
ENSG0000(	370.8218	-0.41365	0.063781	-6.48553	8.84E-11	6.80E-10	LRMP	protein_cc
ENSG0000(	370.7536	-1.44282	0.178454	-8.08509	6.21E-16	1.11E-14	KCNS1	protein_cc
ENSG0000(	370.3861	0.292597	0.046755	6.258087	3.90E-10	2.70E-09	ZNF85	protein_cc
ENSG0000(	370.1493	0.697085	0.119733	5.822018	5.81E-09	3.34E-08	ALDH1L1-	antisense

ENSG0000(	369.8563	-0.26333	0.081583	-3.22781	0.001247	0.002987	RP11-75C	lincRNA
ENSG0000(	369.7454	0.142603	0.029381	4.853573	1.21E-06	4.84E-06	DUSP28	protein_cc
ENSG0000(	369.306	0.664785	0.12986	5.119261	3.07E-07	1.34E-06	CCDC8	protein_cc
ENSG0000(	368.9887	-0.51657	0.120698	-4.27985	1.87E-05	6.19E-05	S1PR5	protein_cc
ENSG0000(	368.4999	0.276833	0.046927	5.899249	3.65E-09	2.18E-08	KHDC1	protein_cc
ENSG0000(	368.3755	0.88592	0.117907	7.513731	5.75E-14	7.29E-13	ESPL1	protein_cc
ENSG0000(	368.2896	-0.15843	0.098179	-1.61367	0.1066	0.163227	SVEP1	protein_cc
ENSG0000(	368.0015	-0.25743	0.075353	-3.41631	0.000635	0.001606	GATSL3	protein_cc
ENSG0000(	367.978	0.212877	0.044034	4.834425	1.34E-06	5.29E-06	ZNF784	protein_cc
ENSG0000(	367.8557	-0.84427	0.149601	-5.64347	1.67E-08	8.93E-08	NELL1	protein_cc
ENSG0000(	367.8091	0.116725	0.026875	4.34323	1.40E-05	4.73E-05	ZKSCAN4	protein_cc
ENSG0000(	367.2368	0.000826	0.027737	0.029765	0.976255	0.983284	ZKSCAN3	protein_cc
ENSG0000(	367.1163	0.20601	0.065838	3.129032	0.001754	0.004091	TSNAXIP1	protein_cc
ENSG0000(	367.0718	-0.12706	0.06595	-1.92655	0.054036	0.090436	KANK3	protein_cc
ENSG0000(	366.908	-0.05608	0.118423	-0.47356	0.635815	0.716344	CDH1	protein_cc
ENSG0000(	366.8599	-0.02087	0.147262	-0.14174	0.887286	0.919761	ZFR2	protein_cc
ENSG0000(	366.7557	-0.08301	0.058086	-1.42916	0.152958	0.22249	ZFP69	protein_cc
ENSG0000(	366.6446	0.463304	0.062762	7.381917	1.56E-13	1.84E-12	MNS1	protein_cc
ENSG0000(	366.6237	0.223495	0.109915	2.033342	0.042018	0.072472	CPNE8	protein_cc
ENSG0000(	366.5164	-0.33154	0.096647	-3.43039	0.000603	0.001531	GALNT6	protein_cc
ENSG0000(	366.3018	0.315008	0.097118	3.243562	0.00118	0.002837	TBX15	protein_cc
ENSG0000(	365.9858	0.110078	0.067771	1.624257	0.104321	0.160204	CTC-524C	antisense
ENSG0000(	365.9765	-0.76766	0.127881	-6.00296	1.94E-09	1.21E-08	ACY3	protein_cc
ENSG0000(	365.8502	-0.97518	0.132245	-7.37406	1.66E-13	1.94E-12	PCDH11X	protein_cc
ENSG0000(	365.747	-0.0138	0.069396	-0.1989	0.842344	0.885495	TMEM91	protein_cc
ENSG0000(	365.6996	0.148579	0.048254	3.079106	0.002076	0.004778	ZFPM1	protein_cc
ENSG0000(	365.6276	0.195491	0.095784	2.040963	0.041254	0.071302	ADAM8	protein_cc
ENSG0000(	365.3677	0.018313	0.026204	0.69887	0.484633	0.579632	TIMM8A	protein_cc
ENSG0000(	365.1942	0.259454	0.119665	2.168169	0.030146	0.053873	F2RL1	protein_cc
ENSG0000(	365.1715	0.195918	0.037207	5.265613	1.40E-07	6.46E-07	PAM16	protein_cc
ENSG0000(	365.0219	-0.13039	0.047616	-2.73843	0.006173	0.0129	LYSMD4	protein_cc
ENSG0000(	365.0097	-0.40485	0.090563	-4.47033	7.81E-06	2.74E-05	C10orf90	protein_cc
ENSG0000(	364.8096	0.124848	0.036149	3.453757	0.000553	0.001414	RTN4IP1	protein_cc
ENSG0000(	364.8033	-0.1672	0.062963	-2.6555	0.007919	0.016195	PLD6	protein_cc
ENSG0000(	364.4334	-0.19476	0.036063	-5.40036	6.65E-08	3.24E-07	PRPF18	protein_cc
ENSG0000(	364.3072	-0.37435	0.04529	-8.26559	1.39E-16	2.76E-15	TTLL11	protein_cc
ENSG0000(	364.0716	-0.76923	0.095032	-8.09445	5.75E-16	1.03E-14	ANKRD34.	protein_cc
ENSG0000(	363.9843	-0.41672	0.0592	-7.03921	1.93E-12	1.93E-11	ZNF540	protein_cc
ENSG0000(	363.9813	0.116515	0.060859	1.914501	0.055556	0.092655	EPB41L4A	protein_cc
ENSG0000(	363.846	-0.10898	0.038355	-2.84144	0.004491	0.009659	TIGD2	protein_cc
ENSG0000(	363.7799	-0.26482	0.121043	-2.18786	0.02868	0.051529	ZNF385D	protein_cc
ENSG0000(	363.4781	-0.05055	0.052039	-0.97134	0.331377	0.425875	CGN	protein_cc
ENSG0000(	363.4145	0.15294	0.038708	3.951128	7.78E-05	0.000232	ZNF835	protein_cc
ENSG0000(	363.2862	-0.52406	0.122565	-4.27578	1.90E-05	6.29E-05	GREB1L	protein_cc
ENSG0000(	363.0582	-0.17222	0.051505	-3.34371	0.000827	0.002046	CDC14A	protein_cc
ENSG0000(	362.8516	-0.87343	0.168176	-5.19353	2.06E-07	9.30E-07	IRX2	protein_cc
ENSG0000(	362.7863	0.06093	0.05822	1.046546	0.295309	0.387205	MYCT1	protein_cc
ENSG0000(	362.7212	-0.78139	0.103137	-7.57627	3.56E-14	4.67E-13	LINC0098.	antisense
ENSG0000(	362.6712	0.125791	0.035218	3.571789	0.000355	0.000942	PSPN	protein_cc
ENSG0000(	362.5567	#######	0.054227	-0.00144	0.998854	0.99917	AP001816	protein_cc
ENSG0000(	362.2974	0.123746	0.065213	1.897558	0.057754	0.095862	GAB3	protein_cc
ENSG0000(	362.2175	-0.42502	0.079594	-5.33983	9.30E-08	4.43E-07	TESC	protein_cc
ENSG0000(	362.0509	0.231856	0.081908	2.830681	0.004645	0.009955	TCF7	protein_cc
ENSG0000(	361.8569	0.128477	0.043332	2.964957	0.003027	0.006739	NBR2	pseudoge
ENSG0000(	361.7863	-0.67145	0.107544	-6.24353	4.28E-10	2.95E-09	RXRG	protein_cc
ENSG0000(	361.7139	0.897122	0.099493	9.01695	1.93E-19	6.05E-18	CDC6	protein_cc
ENSG0000(	361.6199	0.11073	0.032657	3.390719	0.000697	0.001749	ZNF30	protein_cc
ENSG0000(	361.4981	0.059359	0.046875	1.266343	0.20539	0.285633	WASH3P	pseudoge
ENSG0000(	361.4579	0.234218	0.029234	8.011703	1.13E-15	1.94E-14	METTL18	protein_cc

ENSG0000(	361.2568	0.064056	0.067309	0.951674	0.341262	0.436383	SPACA6P	processed
ENSG0000(	361.2449	-0.00347	0.059859	-0.05795	0.953787	0.968028	NOD1	protein_cc
ENSG0000(	361.1692	0.29762	0.050482	5.895556	3.73E-09	2.22E-08	CTD-3184	antisense
ENSG0000(	361.0225	0.108169	0.068675	1.575093	0.115235	0.17464	TOR4A	protein_cc
ENSG0000(	360.885	0.559769	0.064292	8.706672	3.13E-18	8.10E-17	C21orf58	protein_cc
ENSG0000(	360.5238	0.105563	0.095931	1.100406	0.271155	0.360768	RP11-359	lincRNA
ENSG0000(	360.4468	-0.24124	0.083701	-2.88214	0.00395	0.008601	MAP3K8	protein_cc
ENSG0000(	360.4267	-0.13112	0.07502	-1.74775	0.080507	0.127938	JMJD7-PL	protein_cc
ENSG0000(	360.2895	0.282129	0.0407	6.931885	4.15E-12	3.92E-11	ZNF567	protein_cc
ENSG0000(	360.1295	0.546129	0.09314	5.863523	4.53E-09	2.65E-08	BATF2	protein_cc
ENSG0000(	360.0954	0.004179	0.023653	0.176672	0.859766	0.898709	GIN1	protein_cc
ENSG0000(	360.0555	0.189006	0.057402	3.292644	0.000992	0.002418	XXyac-YRI	pseudoge
ENSG0000(	360.054	0.125434	0.066707	1.880378	0.060057	0.099136	AGER	protein_cc
ENSG0000(	359.7765	0.074075	0.035461	2.08889	0.036718	0.064317	DFFB	protein_cc
ENSG0000(	359.6121	-0.13596	0.03753	-3.62276	0.000291	0.000786	SCAND2P	pseudoge
ENSG0000(	359.4608	-0.28279	0.075509	-3.74513	0.00018	0.000504	TBX3	protein_cc
ENSG0000(	359.2852	0.214541	0.106133	2.021431	0.043235	0.074294	COL4A4	protein_cc
ENSG0000(	359.1084	0.221076	0.042087	5.252782	1.50E-07	6.90E-07	RP11-448	lincRNA
ENSG0000(	359.0064	0.568939	0.107004	5.316971	1.06E-07	4.99E-07	TEAD3	protein_cc
ENSG0000(	358.9886	0.095014	0.033489	2.837221	0.004551	0.009772	C3orf33	protein_cc
ENSG0000(	358.9401	-0.02858	0.037146	-0.76949	0.441601	0.538414	ZNF624	protein_cc
ENSG0000(	358.6461	-0.30483	0.134954	-2.25876	0.023899	0.043729	FAM20A	protein_cc
ENSG0000(	358.164	0.001355	0.086243	0.015707	0.987468	0.991349	ESRRG	protein_cc
ENSG0000(	358.064	0.095528	0.037062	2.577503	0.009952	0.019891	ZNF417	protein_cc
ENSG0000(	357.9739	-0.0812	0.181631	-0.44704	0.654844	0.732744	IGKC	IG_C_gene
ENSG0000(	357.8294	0.605277	0.052995	11.42132	3.27E-30	7.65E-28	CKLF	protein_cc
ENSG0000(	357.7676	0.129531	0.04387	2.952616	0.003151	0.006991	SDHAP1	pseudoge
ENSG0000(	357.7645	0.162778	0.077065	2.112229	0.034667	0.061092	ZIC4	protein_cc
ENSG0000(	357.7571	0.160444	0.053462	3.001111	0.00269	0.006053	BBC3	protein_cc
ENSG0000(	357.4073	-0.37625	0.076516	-4.91728	8.78E-07	3.58E-06	GPT	protein_cc
ENSG0000(	357.3906	0.054743	0.058137	0.941625	0.346385	0.441834	RP13-516	lincRNA
ENSG0000(	357.3251	-0.18955	0.042369	-4.47376	7.69E-06	2.70E-05	RP11-175	antisense
ENSG0000(	357.3141	-0.19404	0.081881	-2.36979	0.017798	0.033505	RHBDL1	protein_cc
ENSG0000(	357.2193	0.498379	0.079566	6.263701	3.76E-10	2.62E-09	HELLS	protein_cc
ENSG0000(	357.1808	-0.54006	0.092792	-5.82013	5.88E-09	3.38E-08	MYH7B	protein_cc
ENSG0000(	357.144	0.147979	0.035854	4.127232	3.67E-05	0.000115	DZANK1	protein_cc
ENSG0000(	357.0758	0.096176	0.04446	2.163194	0.030526	0.054489	PARD6G	protein_cc
ENSG0000(	356.9898	-1.05983	0.113881	-9.30643	1.32E-20	4.97E-19	RLTPR	protein_cc
ENSG0000(	356.7934	0.370749	0.043379	8.546675	1.27E-17	2.98E-16	ZNF416	protein_cc
ENSG0000(	356.5243	-0.62684	0.129943	-4.82398	1.41E-06	5.56E-06	CD22	protein_cc
ENSG0000(	356.4229	0.035103	0.051572	0.680663	0.496085	0.590222	ZNF718	lincRNA
ENSG0000(	356.108	-0.2741	0.085483	-3.20644	0.001344	0.003201	WDR86	protein_cc
ENSG0000(	356.0277	-0.05399	0.054546	-0.98987	0.322237	0.416274	GLYCTK	protein_cc
ENSG0000(	355.9601	0.406343	0.131278	3.095293	0.001966	0.004542	COL23A1	protein_cc
ENSG0000(	355.5832	-0.02435	0.032697	-0.74457	0.456529	0.552966	TASP1	protein_cc
ENSG0000(	355.5818	-0.00764	0.09169	-0.08328	0.933629	0.953713	ELF4	protein_cc
ENSG0000(	355.5756	-0.33155	0.090551	-3.66149	0.000251	0.000683	LINC0100	processed
ENSG0000(	355.2223	0.01853	0.105875	0.175016	0.861067	0.899772	FAM179A	protein_cc
ENSG0000(	355.0939	0.279268	0.040404	6.911959	4.78E-12	4.46E-11	C20orf196	protein_cc
ENSG0000(	354.9837	0.241688	0.033202	7.279313	3.36E-13	3.75E-12	TFB1M	protein_cc
ENSG0000(	354.8767	0.018042	0.037957	0.475326	0.634555	0.715404	KB-431C1	antisense
ENSG0000(	354.7728	-0.02716	0.034022	-0.79828	0.424709	0.521801	INCA1	protein_cc
ENSG0000(	354.5502	-0.35792	0.071378	-5.01445	5.32E-07	2.25E-06	LYPD5	protein_cc
ENSG0000(	354.5146	-0.14244	0.111109	-1.28195	0.199859	0.27914	ANGPTL1	protein_cc
ENSG0000(	354.4468	-0.07304	0.035943	-2.03216	0.042138	0.072644	CENPBD1	protein_cc
ENSG0000(	354.4025	-0.01111	0.055863	-0.19882	0.842404	0.885512	CLUHP3	pseudoge
ENSG0000(	354.2884	0.056915	0.04764	1.194686	0.23221	0.316404	CCDC176	protein_cc
ENSG0000(	354.1975	0.598129	0.13993	4.274491	1.92E-05	6.32E-05	CTC-575D	pseudoge
ENSG0000(	354.1039	-0.19398	0.080512	-2.40931	0.015983	0.030486	RAMP2-A	lincRNA

ENSG0000(	353.6307	-0.14657	0.108112	-1.35571	0.175191	0.249621	DLEC1	protein_cc
ENSG0000(	353.3936	0.171516	0.064685	2.651572	0.008012	0.016363	IL27RA	protein_cc
ENSG0000(	353.3127	0.472121	0.088823	5.31529	1.06E-07	5.03E-07	SLC22A18	protein_cc
ENSG0000(	353.036	0.049874	0.03877	1.286402	0.198303	0.277199	CCDC121	protein_cc
ENSG0000(	352.7538	-0.25424	0.077672	-3.27318	0.001063	0.002578	BMP4	protein_cc
ENSG0000(	352.6471	0.595235	0.079686	7.469739	8.04E-14	9.94E-13	CDC25A	protein_cc
ENSG0000(	352.5291	0.153367	0.046813	3.276152	0.001052	0.002553	WASH2P	pseudoge
ENSG0000(	352.5087	0.013112	0.031324	0.418596	0.675512	0.750028	ZNF597	protein_cc
ENSG0000(	352.4273	-0.25371	0.11009	-2.30457	0.021191	0.039226	PABPC5	protein_cc
ENSG0000(	352.3297	0.166837	0.034899	4.780521	1.75E-06	6.80E-06	CLHC1	protein_cc
ENSG0000(	352.2185	-0.03023	0.029798	-1.01444	0.310374	0.403611	ZNF341	protein_cc
ENSG0000(	351.9999	-1.73893	0.16649	-10.4446	1.55E-25	1.49E-23	HCN1	protein_cc
ENSG0000(	351.9913	0.005661	0.078802	0.07184	0.942729	0.959957	PLB1	protein_cc
ENSG0000(	351.7628	0.818276	0.083746	9.770979	1.50E-22	8.03E-21	POC1A	protein_cc
ENSG0000(	351.686	0.727075	0.136037	5.344701	9.06E-08	4.32E-07	TMSB15A	protein_cc
ENSG0000(	351.5947	0.764766	0.114949	6.653085	2.87E-11	2.38E-10	PCOLCE2	protein_cc
ENSG0000(	351.484	-0.16114	0.060991	-2.6421	0.008239	0.016787	MAML3	protein_cc
ENSG0000(	351.0036	-1.38234	0.129914	-10.6405	1.93E-26	2.25E-24	HS3ST4	protein_cc
ENSG0000(	350.7933	-0.14277	0.102617	-1.39129	0.164137	0.236236	STPG1	protein_cc
ENSG0000(	350.7183	-0.19837	0.03273	-6.0609	1.35E-09	8.65E-09	RP11-773	lincRNA
ENSG0000(	350.6589	-0.45046	0.101627	-4.43247	9.32E-06	3.23E-05	RPS6KA6	protein_cc
ENSG0000(	350.5094	-0.4461	0.06053	-7.36987	1.71E-13	2.00E-12	DNAH10C	protein_cc
ENSG0000(	350.331	0.172375	0.034036	5.064535	4.09E-07	1.76E-06	COX10-AS	processed
ENSG0000(	349.885	-0.6981	0.084142	-8.29668	1.07E-16	2.16E-15	DSP	protein_cc
ENSG0000(	349.4264	0.538077	0.098554	5.459737	4.77E-08	2.38E-07	WDR78	protein_cc
ENSG0000(	349.3377	-0.41545	0.05726	-7.25546	4.00E-13	4.42E-12	SGPP2	protein_cc
ENSG0000(	349.3217	0.1637	0.073804	2.21803	0.026553	0.048095	CCDC74B	protein_cc
ENSG0000(	348.8286	0.069697	0.051411	1.355684	0.1752	0.249624	AP006621	protein_cc
ENSG0000(	348.7849	0.11555	0.033109	3.489986	0.000483	0.00125	ATG10	protein_cc
ENSG0000(	348.4121	-0.03986	0.04178	-0.95408	0.340044	0.435095	SFR1	protein_cc
ENSG0000(	348.3349	-0.21047	0.12785	-1.64625	0.099712	0.15414	KIF19	protein_cc
ENSG0000(	348.3317	0.200317	0.056343	3.555307	0.000378	0.000998	NBPF3	protein_cc
ENSG0000(	348.2838	0.091837	0.100526	0.913563	0.360947	0.456795	HRASLS	protein_cc
ENSG0000(	348.1632	0.04715	0.081498	0.578547	0.562895	0.651556	AC004540	lincRNA
ENSG0000(	348.1176	0.088106	0.077815	1.132241	0.257533	0.345439	DPY19L2P	pseudoge
ENSG0000(	347.5584	-0.02563	0.073543	-0.34856	0.72742	0.792792	ZNF571-A	antisense
ENSG0000(	347.1086	-0.089	0.059247	-1.50219	0.133049	0.197284	TMEM240	protein_cc
ENSG0000(	347.0752	-0.71518	0.126571	-5.65046	1.60E-08	8.60E-08	KCNK12	protein_cc
ENSG0000(	346.6117	0.174883	0.09268	1.886953	0.059167	0.097871	MAATS1	protein_cc
ENSG0000(	346.5397	0.348937	0.043283	8.061745	7.52E-16	1.32E-14	PSMC3IP	protein_cc
ENSG0000(	346.3697	0.077959	0.058012	1.343827	0.179004	0.254121	RNF157-A	antisense
ENSG0000(	346.3007	-0.37463	0.073337	-5.10839	3.25E-07	1.42E-06	C10orf128	protein_cc
ENSG0000(	346.072	0.032283	0.046651	0.692008	0.488932	0.583551	MORN1	protein_cc
ENSG0000(	346.0611	-0.14255	0.073077	-1.95062	0.051102	0.086141	GBGT1	protein_cc
ENSG0000(	345.9987	-0.14681	0.072244	-2.03215	0.042139	0.072644	FAM227A	protein_cc
ENSG0000(	345.9703	-0.26987	0.078147	-3.45335	0.000554	0.001416	EBI3	protein_cc
ENSG0000(	345.9196	0.243851	0.043481	5.608169	2.04E-08	1.08E-07	PDCD2L	protein_cc
ENSG0000(	345.8909	0.384188	0.054698	7.023838	2.16E-12	2.13E-11	ZNF93	protein_cc
ENSG0000(	345.6521	-0.21493	0.103644	-2.07377	0.038101	0.066474	RP11-284	antisense
ENSG0000(	345.6376	0.042542	0.05694	0.747143	0.454977	0.55159	B3GALT4	protein_cc
ENSG0000(	345.531	0.404462	0.143076	2.826895	0.0047	0.010062	RP11-750	pseudoge
ENSG0000(	345.3762	1.130133	0.121591	9.294573	1.48E-20	5.51E-19	CKAP2L	protein_cc
ENSG0000(	345.2068	-0.18185	0.064014	-2.84075	0.004501	0.009677	NOS3	protein_cc
ENSG0000(	345.0409	1.317172	0.117666	11.19413	4.36E-29	8.41E-27	GAS2L3	protein_cc
ENSG0000(	344.5834	0.070568	0.043411	1.625568	0.104042	0.15984	PHLDB3	protein_cc
ENSG0000(	344.5601	0.026216	0.131381	0.199543	0.841838	0.885076	LRAT	protein_cc
ENSG0000(	344.38	-0.03505	0.122452	-0.28623	0.774699	0.831733	ZNF300P1	pseudoge
ENSG0000(	344.3504	-0.32162	0.101718	-3.16191	0.001567	0.003689	NINJ2	protein_cc
ENSG0000(	344.193	-0.03638	0.055497	-0.65546	0.512169	0.60526	CROCCP3	pseudoge

ENSG0000(	344.1085	-0.01457	0.036062	-0.404	0.686209	0.759124	ZNF484	protein_cc
ENSG0000(	344.0606	-0.05025	0.041378	-1.21441	0.224591	0.307826	ZNF717	protein_cc
ENSG0000(	343.9621	-0.04793	0.07591	-0.6314	0.52778	0.619525	RP4-800G	pseudoge
ENSG0000(	343.8997	0.948261	0.095515	9.92789	3.15E-23	1.94E-21	SHCBP1	protein_cc
ENSG0000(	343.6642	-0.02685	0.03828	-0.70143	0.483034	0.578055	TRDMT1	protein_cc
ENSG0000(	343.5245	-1.4772	0.19841	-7.44519	9.68E-14	1.18E-12	WIF1	protein_cc
ENSG0000(	343.5147	0.374398	0.084084	4.452644	8.48E-06	2.96E-05	SP140L	protein_cc
ENSG0000(	343.1229	-0.41966	0.108182	-3.87917	0.000105	0.000305	RGS14	protein_cc
ENSG0000(	342.9826	0.624985	0.136735	4.570764	4.86E-06	1.76E-05	LRRN4CL	protein_cc
ENSG0000(	342.8144	0.022527	0.07295	0.308797	0.757476	0.817532	CARD6	protein_cc
ENSG0000(	342.7106	0.288689	0.077066	3.745977	0.00018	0.000502	STAG3L5P	pseudoge
ENSG0000(	342.664	0.154402	0.042214	3.657568	0.000255	0.000693	GNB1L	protein_cc
ENSG0000(	342.5024	-0.64004	0.106422	-6.01418	1.81E-09	1.13E-08	SYN3	protein_cc
ENSG0000(	342.4612	-0.04709	0.081106	-0.5806	0.561511	0.650265	PTPLAD2	protein_cc
ENSG0000(	342.4155	-0.06039	0.079957	-0.75532	0.450057	0.546882	AQP4-AS1	antisense
ENSG0000(	342.1925	0.027362	0.051824	0.52799	0.597506	0.682589	MTG1	protein_cc
ENSG0000(	342.1811	-0.05086	0.033264	-1.52893	0.126282	0.188796	DCLRE1C	protein_cc
ENSG0000(	342.1614	0.504253	0.046951	10.73996	6.61E-27	8.39E-25	DSCC1	protein_cc
ENSG0000(	342.1577	0.214397	0.035877	5.975954	2.29E-09	1.41E-08	FKBPL	protein_cc
ENSG0000(	342.0691	0.298929	0.052159	5.731107	9.98E-09	5.53E-08	NANP	protein_cc
ENSG0000(	341.9451	0.247182	0.067424	3.666061	0.000246	0.000672	TMEM160	protein_cc
ENSG0000(	341.7377	0.425188	0.063952	6.648541	2.96E-11	2.45E-10	RPL6P27	pseudoge
ENSG0000(	341.3945	-0.00244	0.044046	-0.05548	0.955757	0.969453	DUSP19	protein_cc
ENSG0000(	341.38	0.610275	0.053531	11.40037	4.16E-30	9.51E-28	HAUS8	protein_cc
ENSG0000(	341.0928	0.84671	0.087567	9.669282	4.07E-22	2.01E-20	TRIP13	protein_cc
ENSG0000(	340.9816	0.184491	0.070347	2.622609	0.008726	0.017668	RP11-395	lincRNA
ENSG0000(	340.946	-0.06908	0.060986	-1.13272	0.257332	0.345224	SMCO4	protein_cc
ENSG0000(	340.9232	0.128455	0.062874	2.043056	0.041047	0.070993	RP11-284	antisense
ENSG0000(	340.9047	-0.87357	0.124204	-7.03329	2.02E-12	2.01E-11	KCNH1	protein_cc
ENSG0000(	340.7391	0.233106	0.043939	5.305174	1.13E-07	5.30E-07	C11orf80	protein_cc
ENSG0000(	340.5343	0.825417	0.078394	10.52906	6.35E-26	6.68E-24	ORC6	protein_cc
ENSG0000(	340.4154	0.411634	0.053026	7.76291	8.30E-15	1.23E-13	CCDC107	protein_cc
ENSG0000(	340.4022	0.087731	0.101982	0.860266	0.389642	0.486258	SUSD2	protein_cc
ENSG0000(	340.4004	-1.23531	0.133148	-9.27774	1.73E-20	6.37E-19	RP1-111D	lincRNA
ENSG0000(	340.3591	0.384015	0.100235	3.831154	0.000128	0.000366	PILRB	protein_cc
ENSG0000(	340.3219	-0.42267	0.093285	-4.53096	5.87E-06	2.11E-05	WDR49	protein_cc
ENSG0000(	340.1822	-1.36581	0.130492	-10.4666	1.23E-25	1.24E-23	GLT1D1	protein_cc
ENSG0000(	339.7552	-0.02831	0.081673	-0.34667	0.72884	0.794171	NEB	protein_cc
ENSG0000(	339.7503	0.232338	0.129098	1.799703	0.071908	0.116007	B3GNT7	protein_cc
ENSG0000(	339.4708	0.474927	0.107944	4.399742	1.08E-05	3.72E-05	PLD5	protein_cc
ENSG0000(	339.4453	0.651426	0.057478	11.33348	8.96E-30	1.90E-27	CENPL	protein_cc
ENSG0000(	339.2613	2.169402	0.200939	10.79634	3.58E-27	4.77E-25	HOXB3	protein_cc
ENSG0000(	339.1601	0.581461	0.051128	11.37269	5.72E-30	1.27E-27	CTB-31O2	lincRNA
ENSG0000(	338.952	-0.34089	0.067829	-5.02567	5.02E-07	2.13E-06	PHLDB2	protein_cc
ENSG0000(	338.7196	-0.70149	0.132154	-5.30817	1.11E-07	5.21E-07	KCNJ6	protein_cc
ENSG0000(	338.6078	-0.27221	0.094435	-2.88249	0.003945	0.008592	RNF175	protein_cc
ENSG0000(	338.5748	0.387637	0.126607	3.061728	0.002201	0.005038	SECTM1	protein_cc
ENSG0000(	338.5613	0.132504	0.062344	2.125347	0.033558	0.059315	NPIPA1	protein_cc
ENSG0000(	338.4582	0.28775	0.040904	7.034749	2.00E-12	1.99E-11	GS1-124K	pseudoge
ENSG0000(	338.2946	-0.12894	0.077958	-1.65403	0.098122	0.151998	FOXO6	protein_cc
ENSG0000(	338.2513	-0.40014	0.100559	-3.97911	6.92E-05	0.000208	SLC22A15	protein_cc
ENSG0000(	338.2183	-0.42796	0.154848	-2.7637	0.005715	0.012027	CTHRC1	protein_cc
ENSG0000(	338.1794	0.845904	0.098514	8.586656	8.95E-18	2.17E-16	RP11-475	pseudoge
ENSG0000(	337.9701	-0.47303	0.070595	-6.7006	2.08E-11	1.75E-10	PRKCQ	protein_cc
ENSG0000(	337.886	0.171437	0.04574	3.748101	0.000178	0.000498	ZKSCAN7	protein_cc
ENSG0000(	337.8742	-0.51302	0.087719	-5.84844	4.96E-09	2.89E-08	MAPK13	protein_cc
ENSG0000(	337.3639	-0.10485	0.041841	-2.50584	0.012216	0.023921	CKMT2-A	antisense
ENSG0000(	337.1835	1.192087	0.109574	10.87928	1.45E-27	2.04E-25	LMO1	protein_cc
ENSG0000(	337.0966	0.629786	0.071651	8.789604	1.50E-18	4.07E-17	CASP6	protein_cc

ENSG0000(	336.8382	-0.35036	0.127007	-2.7586	0.005805	0.012198	IP6K3	protein_cc
ENSG0000(	336.5884	-0.08594	0.036001	-2.38715	0.01698	0.032163	ZSCAN2	protein_cc
ENSG0000(	336.5543	-0.22421	0.086509	-2.59172	0.00955	0.019168	MAGEL2	protein_cc
ENSG0000(	336.4909	0.139239	0.109514	1.271425	0.203578	0.283506	WFDC1	protein_cc
ENSG0000(	336.4107	0.354758	0.076017	4.666798	3.06E-06	1.14E-05	USP18	protein_cc
ENSG0000(	336.3039	0.317189	0.109028	2.909244	0.003623	0.007945	MYO5B	protein_cc
ENSG0000(	336.1693	0.000947	0.046124	0.020533	0.983618	0.988763	TMEM241	protein_cc
ENSG0000(	335.4212	0.16115	0.092485	1.742459	0.081428	0.129152	LINC0065	lincRNA
ENSG0000(	335.2669	0.612235	0.094869	6.453458	1.09E-10	8.28E-10	EVA1B	protein_cc
ENSG0000(	335.0369	0.283416	0.034971	8.104276	5.31E-16	9.58E-15	CDC26	protein_cc
ENSG0000(	334.9518	0.09207	0.028242	3.260018	0.001114	0.002691	ZSWIM3	protein_cc
ENSG0000(	334.6333	-1.39722	0.165158	-8.45987	2.68E-17	6.02E-16	SLC26A4-	antisense
ENSG0000(	334.5217	-0.10112	0.055903	-1.80883	0.070478	0.113969	CASP10	protein_cc
ENSG0000(	334.4894	0.470513	0.059812	7.866479	3.65E-15	5.75E-14	AC091729	antisense
ENSG0000(	334.4525	0.015214	0.044476	0.342073	0.732296	0.797092	LNX2	protein_cc
ENSG0000(	334.0013	0.201538	0.030313	6.648532	2.96E-11	2.45E-10	CEP76	protein_cc
ENSG0000(	333.8191	0.693591	0.108924	6.367668	1.92E-10	1.40E-09	RP11-632	pseudoge
ENSG0000(	333.805	0.324254	0.08876	3.653166	0.000259	0.000704	RP11-69E	antisense
ENSG0000(	333.7365	0.049308	0.04219	1.168706	0.242522	0.328269	B3GNTL1	protein_cc
ENSG0000(	333.6582	0.47224	0.0908	5.200856	1.98E-07	8.96E-07	ACCS	protein_cc
ENSG0000(	333.4807	-1.06422	0.114532	-9.29187	1.52E-20	5.63E-19	CYGB	protein_cc
ENSG0000(	333.3469	0.092086	0.038005	2.423004	0.015393	0.029463	C5orf55	protein_cc
ENSG0000(	333.3436	2.244135	0.182549	12.29331	9.84E-35	5.86E-32	DMRTA2	protein_cc
ENSG0000(	333.1313	-0.31989	0.117258	-2.7281	0.00637	0.013274	ADAMTSL	protein_cc
ENSG0000(	332.7858	0.206714	0.028706	7.201094	5.97E-13	6.42E-12	WDR53	protein_cc
ENSG0000(	332.6986	0.038122	0.032039	1.189856	0.234103	0.318614	TIRAP	protein_cc
ENSG0000(	332.4114	0.139392	0.076441	1.823527	0.068224	0.110772	MICA	protein_cc
ENSG0000(	332.1066	0.230685	0.060245	3.829134	0.000129	0.000368	AC009120	antisense
ENSG0000(	332.0681	-0.2064	0.069505	-2.96959	0.002982	0.006649	RP1-283E	pseudoge
ENSG0000(	331.879	0.237843	0.032908	7.227608	4.92E-13	5.34E-12	ADPRM	protein_cc
ENSG0000(	331.8571	-0.01935	0.040518	-0.47768	0.632879	0.713996	PDSS1	protein_cc
ENSG0000(	331.6754	1.219025	0.10715	11.37679	5.46E-30	1.23E-27	MTTP	protein_cc
ENSG0000(	331.6088	0.046635	0.062174	0.750078	0.453208	0.549785	PROCA1	protein_cc
ENSG0000(	331.4673	-0.20825	0.093432	-2.22891	0.02582	0.046888	CBS	protein_cc
ENSG0000(	331.443	0.329665	0.116001	2.841925	0.004484	0.009646	BIRC3	protein_cc
ENSG0000(	331.4	-0.1266	0.087002	-1.45514	0.14563	0.213264	HPGDS	protein_cc
ENSG0000(	331.3848	0.43361	0.156204	2.77592	0.005505	0.011628	SERPINA3	protein_cc
ENSG0000(	331.2679	0.07377	0.036273	2.033747	0.041977	0.072417	HDX	protein_cc
ENSG0000(	331.0687	-0.072	0.042222	-1.70528	0.088142	0.138524	PSTK	protein_cc
ENSG0000(	330.9446	-0.34666	0.123468	-2.80765	0.00499	0.010636	FOXP2	protein_cc
ENSG0000(	330.7945	1.090466	0.182713	5.968205	2.40E-09	1.47E-08	TCTEX1D1	protein_cc
ENSG0000(	330.7037	-0.00584	0.04864	-0.12016	0.904358	0.932264	TECTA	protein_cc
ENSG0000(	330.4457	-0.22812	0.044304	-5.1489	2.62E-07	1.16E-06	HSPA1L	protein_cc
ENSG0000(	330.2565	0.206477	0.049901	4.137759	3.51E-05	0.000111	BEND3	protein_cc
ENSG0000(	330.0864	0.332682	0.055461	5.998455	1.99E-09	1.24E-08	C4orf21	protein_cc
ENSG0000(	329.6831	0.036611	0.059789	0.61234	0.540313	0.631138	RP11-274	pseudoge
ENSG0000(	329.5224	0.327524	0.049032	6.679786	2.39E-11	2.01E-10	TCTEX1D2	protein_cc
ENSG0000(	329.4484	0.322674	0.052119	6.191121	5.97E-10	4.03E-09	CAMKMT	protein_cc
ENSG0000(	329.377	-0.65031	0.088172	-7.37551	1.64E-13	1.92E-12	PIP5K1B	protein_cc
ENSG0000(	329.3091	0.059444	0.035862	1.657573	0.097404	0.150996	FAM175A	protein_cc
ENSG0000(	329.1583	-0.49675	0.087233	-5.69449	1.24E-08	6.76E-08	LACC1	protein_cc
ENSG0000(	329.1466	-0.69014	0.09998	-6.90278	5.10E-12	4.73E-11	EXPH5	protein_cc
ENSG0000(	328.9828	0.303503	0.062168	4.881959	1.05E-06	4.23E-06	ST7-AS1	antisense
ENSG0000(	328.9366	-0.47384	0.074788	-6.33577	2.36E-10	1.70E-09	NANOS1	protein_cc
ENSG0000(	328.7773	-0.0125	0.050833	-0.24586	0.805792	0.856861	CTD-2366	antisense
ENSG0000(	328.3269	0.030938	0.051929	0.595785	0.551319	0.641189	LOH12CR	protein_cc
ENSG0000(	327.9597	1.25028	0.132728	9.419867	4.52E-21	1.85E-19	TSTD1	protein_cc
ENSG0000(	327.8634	-0.15242	0.050788	-3.00111	0.00269	0.006053	HSBP1L1	protein_cc
ENSG0000(	327.6239	0.152198	0.081117	1.876267	0.060619	0.099939	RP11-547	lincRNA

ENSG0000(	327.5447	-0.24209	0.087415	-2.76941	0.005616	0.011838	MEGF6	protein_cc
ENSG0000(	327.5345	0.155428	0.05017	3.098049	0.001948	0.004503	BBS12	protein_cc
ENSG0000(	327.2675	0.005967	0.031046	0.192186	0.847596	0.889697	SENP8	protein_cc
ENSG0000(	327.2361	-0.40463	0.102799	-3.93613	8.28E-05	0.000246	ANO4	protein_cc
ENSG0000(	327.1688	0.421631	0.083109	5.073233	3.91E-07	1.69E-06	RINL	protein_cc
ENSG0000(	327.0156	0.16976	0.044084	3.850809	0.000118	0.00034	CCDC61	protein_cc
ENSG0000(	326.9751	-0.0873	0.088667	-0.98457	0.324834	0.419037	TFEC	protein_cc
ENSG0000(	326.9667	0.435278	0.057702	7.54349	4.58E-14	5.89E-13	KDELC1	protein_cc
ENSG0000(	326.694	-0.11286	0.0936	-1.20576	0.227911	0.311599	SIMC1	protein_cc
ENSG0000(	326.4094	0.07345	0.035949	2.043201	0.041033	0.070975	TRMT10A	protein_cc
ENSG0000(	326.2591	0.202291	0.050342	4.01832	5.86E-05	0.000178	SDHAP3	pseudoge
ENSG0000(	325.7195	0.503059	0.042815	11.7497	7.09E-32	2.31E-29	GTF2IRD2	protein_cc
ENSG0000(	325.5897	-0.14715	0.048328	-3.04486	0.002328	0.005304	SRGAP2B	pseudoge
ENSG0000(	325.4432	-1.32039	0.129884	-10.1659	2.81E-24	2.13E-22	PART1	lincRNA
ENSG0000(	325.2061	0.828812	0.098603	8.40555	4.26E-17	9.21E-16	ITPRIPL1	protein_cc
ENSG0000(	325.0337	0.13031	0.132181	0.985848	0.324208	0.418373	LRRC2	protein_cc
ENSG0000(	324.9554	0.042249	0.04647	0.90917	0.36326	0.459243	AMMECR1	protein_cc
ENSG0000(	324.8942	0.301117	0.04194	7.179646	6.99E-13	7.45E-12	LY6G5C	protein_cc
ENSG0000(	324.8638	0.625816	0.097753	6.402019	1.53E-10	1.13E-09	HFE	protein_cc
ENSG0000(	324.5556	-0.17492	0.175867	-0.99463	0.319918	0.413681	TRH	protein_cc
ENSG0000(	324.262	0.397252	0.051728	7.679651	1.60E-14	2.23E-13	DLEU1	protein_cc
ENSG0000(	324.0676	0.216495	0.05862	3.693218	0.000221	0.000609	SPIN4	protein_cc
ENSG0000(	323.7355	0.223088	0.093595	2.383541	0.017147	0.032433	LINC0023	lincRNA
ENSG0000(	323.7159	0.327571	0.086215	3.799467	0.000145	0.000412	CCDC78	protein_cc
ENSG0000(	323.4821	-0.60748	0.102075	-5.95131	2.66E-09	1.62E-08	SH3TC2	protein_cc
ENSG0000(	323.4568	0.093351	0.048811	1.912514	0.05581	0.093015	ACYP1	protein_cc
ENSG0000(	323.1472	0.126528	0.049993	2.530905	0.011377	0.022436	B3GALNT2	protein_cc
ENSG0000(	323.1313	0.917055	0.090399	10.1445	3.51E-24	2.61E-22	RAD51AP1	protein_cc
ENSG0000(	323.0546	-1.18347	0.149663	-7.90757	2.62E-15	4.23E-14	CTD-2081	lincRNA
ENSG0000(	323.0264	0.419726	0.078482	5.348083	8.89E-08	4.25E-07	HSD3B7	protein_cc
ENSG0000(	322.5976	2.012454	0.215856	9.323116	1.13E-20	4.30E-19	SHOX2	protein_cc
ENSG0000(	322.4767	0.203706	0.080149	2.541591	0.011035	0.021836	RP11-864	pseudoge
ENSG0000(	322.3942	-0.49759	0.123813	-4.01889	5.85E-05	0.000178	TPBG	protein_cc
ENSG0000(	322.3778	0.077106	0.05897	1.30755	0.191026	0.268499	UBBP4	protein_cc
ENSG0000(	322.131	-0.05962	0.092572	-0.64406	0.519539	0.612122	CYP4F11	protein_cc
ENSG0000(	321.9896	-0.03938	0.042181	-0.93355	0.350538	0.44597	KIF9-AS1	antisense
ENSG0000(	321.8952	-0.12592	0.071563	-1.75957	0.078481	0.125187	HHAT	protein_cc
ENSG0000(	321.8203	-0.41181	0.082818	-4.97251	6.61E-07	2.75E-06	TET1	protein_cc
ENSG0000(	321.7827	-0.16261	0.041392	-3.9284	8.55E-05	0.000253	DTWD2	protein_cc
ENSG0000(	321.7019	0.980662	0.161829	6.059879	1.36E-09	8.70E-09	KCNE1L	protein_cc
ENSG0000(	321.6726	0.061317	0.037645	1.628839	0.103347	0.158969	ZNF17	protein_cc
ENSG0000(	321.4815	0.170526	0.065703	2.595403	0.009448	0.018977	MATN3	protein_cc
ENSG0000(	321.4442	-0.501	0.107814	-4.64692	3.37E-06	1.25E-05	NCR3LG1	protein_cc
ENSG0000(	321.3998	0.32741	0.062885	5.206467	1.92E-07	8.72E-07	RP11-631	antisense
ENSG0000(	321.3079	-0.40531	0.106235	-3.81521	0.000136	0.000388	PCDHGA2	protein_cc
ENSG0000(	321.0383	0.081566	0.080956	1.007537	0.313677	0.40709	ADSSL1	protein_cc
ENSG0000(	320.6627	0.408379	0.087973	4.642116	3.45E-06	1.28E-05	RCAN3	protein_cc
ENSG0000(	320.6543	-0.0943	0.102291	-0.92189	0.356586	0.452265	SSC5D	protein_cc
ENSG0000(	320.6117	0.797339	0.08293	9.614555	6.94E-22	3.27E-20	STIL	protein_cc
ENSG0000(	320.536	-0.28585	0.05564	-5.13744	2.79E-07	1.23E-06	NBEAL1	protein_cc
ENSG0000(	320.3345	-0.05246	0.097545	-0.53782	0.5907	0.67649	SLC4A11	protein_cc
ENSG0000(	320.2767	-0.11136	0.087314	-1.27535	0.202187	0.281903	CD33	protein_cc
ENSG0000(	320.1007	-0.86962	0.13754	-6.32265	2.57E-10	1.84E-09	PCP4L1	protein_cc
ENSG0000(	320.0682	0.079174	0.090315	0.876642	0.380681	0.477247	TTC18	protein_cc
ENSG0000(	320.0041	-0.02084	0.073161	-0.2849	0.775718	0.832567	RP11-977	sense_ove
ENSG0000(	319.9391	0.643164	0.143909	4.469229	7.85E-06	2.76E-05	GSX1	protein_cc
ENSG0000(	319.5025	0.220984	0.048547	4.552	5.31E-06	1.92E-05	TMEM17	protein_cc
ENSG0000(	319.4765	0.443753	0.046714	9.49941	2.11E-21	9.24E-20	C8orf76	protein_cc
ENSG0000(	319.0054	-0.1389	0.029695	-4.67744	2.90E-06	1.09E-05	KDM8	protein_cc

ENSG0000(	318.9928	0.335748	0.085392	3.931827	8.43E-05	0.00025	AQP6	protein_cc
ENSG0000(	318.7082	1.072839	0.094434	11.36078	6.56E-30	1.43E-27	SPC25	protein_cc
ENSG0000(	318.4775	-0.24335	0.063202	-3.85031	0.000118	0.00034	RP1-193H	lincRNA
ENSG0000(	318.0586	0.265389	0.043236	6.138156	8.35E-10	5.51E-09	TBC1D31	protein_cc
ENSG0000(	317.5962	-0.15692	0.102453	-1.53159	0.125623	0.18792	SLCO5A1	protein_cc
ENSG0000(	317.453	0.425802	0.045846	9.28761	1.58E-20	5.83E-19	LIN9	protein_cc
ENSG0000(	317.4524	0.084388	0.0842	1.002238	0.316228	0.409851	ITGA10	protein_cc
ENSG0000(	317.4423	0.219503	0.06477	3.388962	0.000702	0.001759	C9orf116	protein_cc
ENSG0000(	317.3762	0.119941	0.045486	2.636858	0.008368	0.017019	SLC25A35	protein_cc
ENSG0000(	317.2025	0.106572	0.053487	1.992473	0.046319	0.07896	KIF20B	protein_cc
ENSG0000(	317.1465	-0.03051	0.080933	-0.37694	0.706216	0.775747	USP32P3	pseudoge
ENSG0000(	317.0815	0.233686	0.064188	3.640669	0.000272	0.000737	CCDC102/	protein_cc
ENSG0000(	316.8534	0.069115	0.04366	1.583033	0.113414	0.172232	ZNF808	protein_cc
ENSG0000(	316.7836	0.307631	0.117139	2.626218	0.008634	0.017502	NXPH4	protein_cc
ENSG0000(	316.7623	-1.04262	0.087354	-11.9357	7.71E-33	3.14E-30	FMN1	protein_cc
ENSG0000(	316.7394	0.25481	0.058069	4.388044	1.14E-05	3.91E-05	CCDC151	protein_cc
ENSG0000(	316.1474	-0.61022	0.145801	-4.18529	2.85E-05	9.12E-05	GREM1	protein_cc
ENSG0000(	316.1167	-0.32746	0.104144	-3.1443	0.001665	0.003899	PTGIS	protein_cc
ENSG0000(	316.0369	0.188111	0.043969	4.278212	1.88E-05	6.23E-05	SMPD2	protein_cc
ENSG0000(	316.012	0.18551	0.060024	3.090575	0.001998	0.004609	SP110	protein_cc
ENSG0000(	315.8622	1.220753	0.168809	7.231554	4.77E-13	5.20E-12	EYA4	protein_cc
ENSG0000(	315.7222	0.317526	0.06498	4.886507	1.03E-06	4.14E-06	RPL7P9	pseudoge
ENSG0000(	315.4959	1.00532	0.178204	5.641406	1.69E-08	9.03E-08	CLEC5A	protein_cc
ENSG0000(	315.4036	-0.24138	0.060075	-4.01804	5.87E-05	0.000179	TIAF1	protein_cc
ENSG0000(	315.2797	-0.86923	0.156913	-5.5396	3.03E-08	1.56E-07	GRM5	protein_cc
ENSG0000(	315.0873	-1.52504	0.140584	-10.8479	2.04E-27	2.82E-25	DLGAP2	protein_cc
ENSG0000(	314.9664	0.490128	0.076465	6.409796	1.46E-10	1.08E-09	RP11-466	pseudoge
ENSG0000(	314.9223	0.106959	0.051703	2.068712	0.038573	0.067207	CDK11A	protein_cc
ENSG0000(	314.8319	0.777423	0.11954	6.503454	7.85E-11	6.07E-10	TOM1L1	protein_cc
ENSG0000(	314.7528	1.023932	0.107625	9.513929	1.84E-21	8.14E-20	CENPE	protein_cc
ENSG0000(	314.6614	0.04674	0.080203	0.582774	0.560046	0.64893	CD40	protein_cc
ENSG0000(	314.4547	0.019755	0.04837	0.408404	0.682977	0.756118	MCM3AP-	antisense
ENSG0000(	314.3672	0.285071	0.109606	2.600871	0.009299	0.018707	LY96	protein_cc
ENSG0000(	314.3289	-0.80028	0.07548	-10.6026	2.90E-26	3.25E-24	INPP5J	protein_cc
ENSG0000(	314.2408	-0.5528	0.130526	-4.23515	2.28E-05	7.43E-05	MRAP2	protein_cc
ENSG0000(	314.2328	-0.348	0.064165	-5.42344	5.85E-08	2.88E-07	PPARGC1I	protein_cc
ENSG0000(	313.9426	0.020011	0.067138	0.298054	0.765662	0.824097	CLIC2	protein_cc
ENSG0000(	313.7552	0.274503	0.133232	2.060343	0.039366	0.068405	VWA3A	protein_cc
ENSG0000(	313.6654	0.112594	0.040452	2.783407	0.005379	0.011393	ZNF571	protein_cc
ENSG0000(	313.6162	0.369594	0.038962	9.486134	2.40E-21	1.03E-19	ZNF674-A	lincRNA
ENSG0000(	313.5736	-1.14996	0.115334	-9.97073	2.05E-23	1.33E-21	GRM1	protein_cc
ENSG0000(	313.5576	-0.23303	0.054915	-4.24349	2.20E-05	7.18E-05	ZNF697	protein_cc
ENSG0000(	313.3345	0.024906	0.032005	0.778185	0.43646	0.533466	RNASEK	protein_cc
ENSG0000(	313.313	1.440934	0.151419	9.516198	1.80E-21	7.97E-20	TNFAIP6	protein_cc
ENSG0000(	313.1569	0.041988	0.037421	1.122061	0.261837	0.350319	RAB30-AS	lincRNA
ENSG0000(	313.0467	0.130311	0.043131	3.021287	0.002517	0.005704	RP11-418.	antisense
ENSG0000(	313.042	0.193354	0.06977	2.771301	0.005583	0.011775	CCNE2	protein_cc
ENSG0000(	313.02	0.253933	0.094154	2.697001	0.006997	0.014457	PRRT4	protein_cc
ENSG0000(	312.8537	0.108025	0.150102	0.719675	0.471725	0.567594	TMEM196	protein_cc
ENSG0000(	312.8352	0.50336	0.06335	7.945641	1.93E-15	3.18E-14	KATNAL2	protein_cc
ENSG0000(	312.378	0.102982	0.061184	1.683165	0.092343	0.144162	CCDC30	protein_cc
ENSG0000(	312.1641	-0.08495	0.044891	-1.89229	0.058452	0.096849	RP11-6N1	processed
ENSG0000(	311.5098	0.224394	0.09551	2.349418	0.018803	0.035212	RP5-857K	pseudoge
ENSG0000(	311.3196	0.472957	0.089821	5.265551	1.40E-07	6.46E-07	ZBTB42	protein_cc
ENSG0000(	311.0077	-0.01336	0.035609	-0.37506	0.707619	0.776997	MFSD9	protein_cc
ENSG0000(	310.9988	0.021783	0.039987	0.544752	0.585924	0.672196	TBC1D19	protein_cc
ENSG0000(	310.9536	0.136931	0.10199	1.342594	0.179404	0.254573	CERKL	protein_cc
ENSG0000(	310.8875	0.233065	0.046863	4.973278	6.58E-07	2.74E-06	ZNF628	protein_cc
ENSG0000(	310.8084	-0.05995	0.04216	-1.42186	0.155067	0.225066	DSTNP2	pseudoge

ENSG0000(	310.5644	-1.79082	0.181952	-9.84229	7.40E-23	4.20E-21	SLC6A7	protein_cc
ENSG0000(	310.1944	-0.55047	0.076017	-7.2414	4.44E-13	4.86E-12	DUOX1	protein_cc
ENSG0000(	310.1619	-0.23056	0.060479	-3.81229	0.000138	0.000392	RP11-359	antisense
ENSG0000(	310.1385	0.22647	0.031694	7.145547	8.96E-13	9.40E-12	LIPT1	protein_cc
ENSG0000(	310.0322	-1.25124	0.125211	-9.99303	1.63E-23	1.08E-21	NPM2	protein_cc
ENSG0000(	310.0302	0.391121	0.112102	3.488979	0.000485	0.001254	PRSS12	protein_cc
ENSG0000(	310.0292	-0.41419	0.082703	-5.00815	5.50E-07	2.32E-06	TLR5	protein_cc
ENSG0000(	309.7323	-0.02362	0.087714	-0.26933	0.787674	0.842168	GNB3	protein_cc
ENSG0000(	309.7233	0.346512	0.081341	4.259993	2.04E-05	6.72E-05	HAPLN3	protein_cc
ENSG0000(	309.6059	-0.30896	0.088225	-3.50189	0.000462	0.0012	CPED1	protein_cc
ENSG0000(	309.5501	0.07554	0.100819	0.749267	0.453696	0.550329	SIGLEC9	protein_cc
ENSG0000(	309.5235	-0.02363	0.076066	-0.31064	0.756078	0.816282	ITGA11	protein_cc
ENSG0000(	309.4672	0.094582	0.093855	1.00774	0.313579	0.406995	PHEX	protein_cc
ENSG0000(	309.3617	0.109404	0.064227	1.703393	0.088495	0.139009	RP11-660	antisense
ENSG0000(	309.2599	0.328805	0.051021	6.44446	1.16E-10	8.76E-10	AC012146	processed
ENSG0000(	309.241	0.221507	0.083763	2.644437	0.008183	0.01668	SMIM10	protein_cc
ENSG0000(	308.8302	0.411354	0.078328	5.251701	1.51E-07	6.93E-07	GJA4	protein_cc
ENSG0000(	308.5805	-0.21507	0.100655	-2.13673	0.03262	0.057842	RP6-201G	lincRNA
ENSG0000(	308.4988	-0.05635	0.045268	-1.24471	0.213238	0.294746	RP11-540	sense_intr
ENSG0000(	308.3135	0.106612	0.029937	3.561263	0.000369	0.000977	FCF1P2	pseudoge
ENSG0000(	308.3032	-0.09553	0.043343	-2.20393	0.027529	0.049632	ISPD	protein_cc
ENSG0000(	308.2624	-0.1522	0.046805	-3.25177	0.001147	0.002763	SLC35G1	protein_cc
ENSG0000(	308.1504	-1.34087	0.137029	-9.78532	1.30E-22	7.04E-21	RTN4RL1	protein_cc
ENSG0000(	308.0772	0.313163	0.103288	3.031945	0.00243	0.005522	PCDHB6	protein_cc
ENSG0000(	308.0542	0.739727	0.187839	3.938093	8.21E-05	0.000244	CA9	protein_cc
ENSG0000(	308.0351	0.315932	0.049586	6.371351	1.87E-10	1.36E-09	C1orf112	protein_cc
ENSG0000(	307.8124	0.071653	0.077236	0.92771	0.353558	0.449117	RP5-1057.	lincRNA
ENSG0000(	307.7582	-0.98953	0.095261	-10.3875	2.83E-25	2.59E-23	CYS1	protein_cc
ENSG0000(	307.6578	-0.19456	0.090087	-2.15963	0.030801	0.054917	LINC0063!	lincRNA
ENSG0000(	307.4888	0.136224	0.043296	3.146375	0.001653	0.003874	GGT1	protein_cc
ENSG0000(	307.4873	0.080758	0.038595	2.092444	0.036399	0.063827	AARSD1	protein_cc
ENSG0000(	307.1505	0.542692	0.076492	7.094743	1.30E-12	1.32E-11	PAXIP1-A!	protein_cc
ENSG0000(	307.0448	0.304584	0.062339	4.88591	1.03E-06	4.15E-06	CTA-29F1	antisense
ENSG0000(	306.7767	1.294069	0.121705	10.63282	2.10E-26	2.44E-24	PINLYP	protein_cc
ENSG0000(	306.7542	-0.23453	0.133905	-1.75148	0.079863	0.127081	NDNF	protein_cc
ENSG0000(	306.7047	1.127526	0.117323	9.610435	7.22E-22	3.39E-20	CDC45	protein_cc
ENSG0000(	306.55	0.049082	0.06397	0.767263	0.442925	0.539763	TCAP	protein_cc
ENSG0000(	306.487	-0.55698	0.117398	-4.74435	2.09E-06	8.04E-06	MLIP	protein_cc
ENSG0000(	306.4815	0.295086	0.083243	3.5449	0.000393	0.001035	SPAG8	protein_cc
ENSG0000(	306.4124	-0.01587	0.056128	-0.28266	0.777434	0.833799	SPRY3	protein_cc
ENSG0000(	306.1076	-0.26095	0.083094	-3.14048	0.001687	0.003946	DGAT2	protein_cc
ENSG0000(	306.1042	0.427913	0.043172	9.911721	3.70E-23	2.24E-21	CCT6P1	pseudoge
ENSG0000(	306.1007	0.552342	0.109004	5.067151	4.04E-07	1.74E-06	RLBP1	protein_cc
ENSG0000(	306.0617	-0.73759	0.138128	-5.33989	9.30E-08	4.43E-07	RSPO2	protein_cc
ENSG0000(	306.0411	0.302195	0.036716	8.230516	1.86E-16	3.62E-15	ZNF582	protein_cc
ENSG0000(	306.0128	0.383888	0.03495	10.98389	4.57E-28	6.98E-26	TEFM	protein_cc
ENSG0000(	305.9842	-0.5513	0.083064	-6.63709	3.20E-11	2.63E-10	KIF17	protein_cc
ENSG0000(	305.7193	0.419076	0.069247	6.051865	1.43E-09	9.10E-09	ZFHX4-AS	antisense
ENSG0000(	305.6762	-0.84205	0.096427	-8.73249	2.49E-18	6.54E-17	MYH15	protein_cc
ENSG0000(	305.4029	-0.22242	0.044858	-4.95844	7.11E-07	2.94E-06	DUSP18	protein_cc
ENSG0000(	305.2344	-0.45516	0.108891	-4.17995	2.92E-05	9.32E-05	EFNA5	protein_cc
ENSG0000(	305.0745	0.259165	0.10853	2.387955	0.016942	0.0321	TMEM229	protein_cc
ENSG0000(	304.9003	0.309629	0.125316	2.470792	0.013481	0.026149	GPR65	protein_cc
ENSG0000(	304.6278	-0.09283	0.046283	-2.00563	0.044896	0.076796	ACAP1	protein_cc
ENSG0000(	304.3505	-1.25326	0.137253	-9.131	6.79E-20	2.28E-18	FSTL4	protein_cc
ENSG0000(	304.3045	0.502601	0.063178	7.955334	1.79E-15	2.96E-14	KIAA1524	protein_cc
ENSG0000(	304.3042	0.405209	0.04502	9.000581	2.25E-19	6.92E-18	RPP40	protein_cc
ENSG0000(	304.2895	0.543626	0.05789	9.390631	5.96E-21	2.38E-19	DHRS13	protein_cc
ENSG0000(	304.2354	0.158773	0.023698	6.699852	2.09E-11	1.76E-10	ZBTB49	protein_cc

ENSG0000(	304.1949	-0.19166	0.103674	-1.84866	0.064507	0.105525	DCT	protein_cc
ENSG0000(	303.8991	-0.04258	0.04948	-0.86055	0.389488	0.486095	ZXDA	protein_cc
ENSG0000(	303.8378	1.322041	0.167073	7.912944	2.51E-15	4.06E-14	GDF15	protein_cc
ENSG0000(	303.6098	-0.97906	0.11447	-8.55298	1.20E-17	2.83E-16	CNTN4	protein_cc
ENSG0000(	303.3577	1.079155	0.154037	7.005837	2.46E-12	2.41E-11	CHIT1	protein_cc
ENSG0000(	303.2266	-0.09453	0.059768	-1.58165	0.113729	0.17264	U91328.19	lincRNA
ENSG0000(	303.1642	0.075503	0.028928	2.610046	0.009053	0.018262	ZNF79	protein_cc
ENSG0000(	303.0391	-0.24802	0.130306	-1.90337	0.056993	0.094728	GBX2	protein_cc
ENSG0000(	302.9738	0.018056	0.038002	0.475149	0.634681	0.715478	RP11-114	antisense
ENSG0000(	302.9243	0.187018	0.08393	2.228265	0.025863	0.046959	SOX18	protein_cc
ENSG0000(	302.9242	-0.6817	0.112194	-6.0761	1.23E-09	7.92E-09	CCDC64	protein_cc
ENSG0000(	302.816	0.235314	0.112679	2.088347	0.036767	0.064392	CDCP1	protein_cc
ENSG0000(	302.6524	-0.29821	0.06027	-4.94797	7.50E-07	3.09E-06	SNAI3-AS	antisense
ENSG0000(	302.5737	-0.25795	0.072522	-3.55689	0.000375	0.000992	CCDC160	protein_cc
ENSG0000(	302.5706	0.334965	0.175016	1.913909	0.055632	0.092755	SLC17A8	protein_cc
ENSG0000(	302.5546	-0.14054	0.176091	-0.79814	0.424791	0.52187	STC1	protein_cc
ENSG0000(	302.4722	0.119929	0.056579	2.119666	0.034034	0.06008	QRICH2	protein_cc
ENSG0000(	302.4261	0.205733	0.071239	2.887936	0.003878	0.008457	LIME1	protein_cc
ENSG0000(	302.2716	-0.07646	0.048241	-1.58495	0.112977	0.171663	AL807752	protein_cc
ENSG0000(	302.1684	-0.07881	0.044446	-1.77314	0.076205	0.122016	ARL6	protein_cc
ENSG0000(	302.1054	-0.40667	0.073128	-5.5611	2.68E-08	1.39E-07	BX322557	processed
ENSG0000(	302.0495	0.167771	0.047591	3.525297	0.000423	0.001108	THOC3	protein_cc
ENSG0000(	301.9334	-0.43892	0.113282	-3.8746	0.000107	0.00031	IRX3	protein_cc
ENSG0000(	301.5356	-0.04068	0.114632	-0.35487	0.722686	0.788971	SIGLEC14	protein_cc
ENSG0000(	301.4255	-0.52857	0.06985	-7.56724	3.81E-14	4.98E-13	WNT2B	protein_cc
ENSG0000(	301.3655	0.420063	0.276057	1.521656	0.128095	0.191049	IBSP	protein_cc
ENSG0000(	301.2048	0.000859	0.140832	0.006097	0.995135	0.996881	DLX1	protein_cc
ENSG0000(	301.0769	0.014049	0.113535	0.123744	0.901518	0.930132	ADAMTS7	protein_cc
ENSG0000(	300.8726	0.254405	0.040006	6.359125	2.03E-10	1.47E-09	LINC0052(	lincRNA
ENSG0000(	300.8614	0.331289	0.09584	3.456677	0.000547	0.001401	ADAMTSL	protein_cc
ENSG0000(	300.4483	0.227973	0.05537	4.117307	3.83E-05	0.00012	PPP4R1L	protein_cc
ENSG0000(	300.4264	0.104285	0.170136	0.612947	0.539911	0.630812	LEFTY2	protein_cc
ENSG0000(	300.271	0.302036	0.054713	5.520348	3.38E-08	1.73E-07	RP11-798	antisense
ENSG0000(	300.224	-0.4223	0.077036	-5.4819	4.21E-08	2.12E-07	SPTLC3	protein_cc
ENSG0000(	300.0527	0.585738	0.098615	5.939624	2.86E-09	1.73E-08	EEF1A1P1	pseudoge
ENSG0000(	299.9627	0.344425	0.073009	4.717598	2.39E-06	9.09E-06	TMEM220	protein_cc
ENSG0000(	299.9585	0.371718	0.093701	3.967079	7.28E-05	0.000218	FAM184B	protein_cc
ENSG0000(	299.8701	-0.10663	0.077463	-1.3765	0.168667	0.241733	B3GALT5	protein_cc
ENSG0000(	299.8366	0.03265	0.115047	0.283796	0.776567	0.833217	COL4A6	protein_cc
ENSG0000(	299.3512	0.166428	0.064834	2.566973	0.010259	0.02044	ENO3	protein_cc
ENSG0000(	299.2771	0.137904	0.039498	3.491425	0.00048	0.001244	ZNF235	protein_cc
ENSG0000(	299.218	0.188978	0.043174	4.377133	1.20E-05	4.10E-05	CTC-459F	lincRNA
ENSG0000(	299.089	0.380166	0.1164	3.266041	0.001091	0.002639	TEAD4	protein_cc
ENSG0000(	298.9933	-0.16074	0.047956	-3.35182	0.000803	0.001992	AC007551	lincRNA
ENSG0000(	298.9547	0.032736	0.046111	0.709955	0.477732	0.573245	ZNF239	protein_cc
ENSG0000(	298.7805	0.204696	0.043806	4.672781	2.97E-06	1.11E-05	PIGB	protein_cc
ENSG0000(	298.7459	0.011873	0.140659	0.084411	0.93273	0.953102	C5orf49	protein_cc
ENSG0000(	298.6931	0.076063	0.040522	1.877082	0.060507	0.099779	ZFP2	protein_cc
ENSG0000(	298.5758	-0.38319	0.059944	-6.39251	1.63E-10	1.20E-09	SMAD6	protein_cc
ENSG0000(	298.2369	0.32785	0.073187	4.479641	7.48E-06	2.64E-05	TICRR	protein_cc
ENSG0000(	297.8355	0.471719	0.087023	5.420631	5.94E-08	2.92E-07	RPL4P4	pseudoge
ENSG0000(	297.7559	-0.07256	0.075641	-0.95925	0.337433	0.43228	MIR3682	miRNA
ENSG0000(	297.6624	-1.41199	0.15185	-9.29858	1.42E-20	5.33E-19	TMEM155	protein_cc
ENSG0000(	297.5803	0.740866	0.079472	9.322374	1.14E-20	4.32E-19	AC007969	pseudoge
ENSG0000(	297.5022	0.696222	0.102457	6.795233	1.08E-11	9.53E-11	FAM65C	protein_cc
ENSG0000(	297.4572	0.169434	0.075403	2.247053	0.024637	0.044935	YDJC	protein_cc
ENSG0000(	297.4151	-0.1491	0.126813	-1.17576	0.239692	0.325039	ADAMTS1	protein_cc
ENSG0000(	297.273	-0.07322	0.053778	-1.36143	0.173378	0.24738	ICAM2	protein_cc
ENSG0000(	297.165	0.234562	0.042217	5.556047	2.76E-08	1.43E-07	ZNF845	protein_cc

ENSG0000	296.7278	-0.22992	0.07802	-2.947	0.003209	0.007108	LINC0053	pseudoge
ENSG0000	296.672	-0.27021	0.077623	-3.48104	0.000499	0.001289	TMEM106	protein_cc
ENSG0000	296.5611	0.259688	0.129612	2.003582	0.045115	0.077128	PCDHB3	protein_cc
ENSG0000	296.5581	-0.08288	0.026687	-3.10552	0.001899	0.004401	IKBKG	protein_cc
ENSG0000	295.6276	0.20686	0.03841	5.38559	7.22E-08	3.50E-07	ZSCAN22	protein_cc
ENSG0000	295.5979	-0.38795	0.051833	-7.48468	7.17E-14	8.97E-13	LL22NC03	antisense
ENSG0000	295.552	0.293189	0.073817	3.971856	7.13E-05	0.000214	DYRK3	protein_cc
ENSG0000	295.4786	0.146538	0.104708	1.399492	0.161665	0.233087	HKDC1	protein_cc
ENSG0000	295.3367	1.294287	0.156754	8.25682	1.50E-16	2.96E-15	HS3ST3B1	protein_cc
ENSG0000	295.1401	0.3198	0.047377	6.750138	1.48E-11	1.28E-10	CCDC138	protein_cc
ENSG0000	295.0332	0.085277	0.063824	1.336124	0.181509	0.257073	RELL1	protein_cc
ENSG0000	294.3152	0.342569	0.038117	8.987361	2.53E-19	7.75E-18	AC009506	antisense
ENSG0000	294.2832	-0.05781	0.104897	-0.55108	0.581581	0.668389	FIBCD1	protein_cc
ENSG0000	294.1578	0.087475	0.034457	2.538634	0.011129	0.022003	ZNF283	protein_cc
ENSG0000	294.1561	0.288961	0.046564	6.205645	5.45E-10	3.69E-09	PXN-AS1	antisense
ENSG0000	294.0881	0.625765	0.081967	7.634335	2.27E-14	3.08E-13	RP11-51C	pseudoge
ENSG0000	294.0563	0.584856	0.098975	5.909134	3.44E-09	2.06E-08	RPS2P46	pseudoge
ENSG0000	293.8439	-0.09878	0.065933	-1.49813	0.134099	0.198606	CDHR3	protein_cc
ENSG0000	293.7864	0.044794	0.045979	0.974235	0.32994	0.424332	ADAT2	protein_cc
ENSG0000	293.6518	-0.36164	0.071393	-5.06551	4.07E-07	1.75E-06	A4GALT	protein_cc
ENSG0000	293.5796	-0.05413	0.05905	-0.91666	0.359323	0.455091	SLC43A1	protein_cc
ENSG0000	293.4051	0.674738	0.062331	10.82506	2.62E-27	3.53E-25	LRR1	protein_cc
ENSG0000	293.3442	0.078917	0.035036	2.252457	0.024293	0.044386	LL22NC03	pseudoge
ENSG0000	293.0823	0.132015	0.119485	1.104867	0.269217	0.358538	AIM1	protein_cc
ENSG0000	293.0798	0.330786	0.096697	3.420834	0.000624	0.001581	KCNE3	protein_cc
ENSG0000	292.805	-0.52373	0.100353	-5.21887	1.80E-07	8.18E-07	DGCR9	lincRNA
ENSG0000	292.7678	-0.07379	0.090616	-0.81429	0.415478	0.512481	MILR1	protein_cc
ENSG0000	292.7188	0.259217	0.04128	6.27943	3.40E-10	2.38E-09	TM9SF1	protein_cc
ENSG0000	292.6387	0.246269	0.112635	2.186423	0.028785	0.051703	PLBD1	protein_cc
ENSG0000	292.6291	0.313518	0.03929	7.979554	1.47E-15	2.47E-14	ZNF77	protein_cc
ENSG0000	292.3281	0.112149	0.032546	3.445827	0.000569	0.001454	TIGD7	protein_cc
ENSG0000	292.1787	-0.35015	0.102136	-3.4283	0.000607	0.001542	ADAMTS2	protein_cc
ENSG0000	291.9856	-0.18127	0.052855	-3.42952	0.000605	0.001536	FAM47E	protein_cc
ENSG0000	291.9766	-0.42051	0.109346	-3.84573	0.00012	0.000346	CXCL2	protein_cc
ENSG0000	291.6742	0.159373	0.030612	5.206309	1.93E-07	8.72E-07	PUS10	protein_cc
ENSG0000	291.6547	-0.9018	0.14373	-6.27429	3.51E-10	2.46E-09	ST6GALNA	protein_cc
ENSG0000	291.5745	0.20242	0.065202	3.104489	0.001906	0.004414	RP11-504	lincRNA
ENSG0000	291.5165	-0.94013	0.108187	-8.68986	3.63E-18	9.29E-17	ADRA1A	protein_cc
ENSG0000	291.231	-0.16817	0.08936	-1.88193	0.059845	0.09885	SUSD3	protein_cc
ENSG0000	291.1773	-1.16593	0.119365	-9.76776	1.55E-22	8.26E-21	PLIN1	protein_cc
ENSG0000	291.1427	0.090682	0.057569	1.575179	0.115215	0.174619	LINC0086	lincRNA
ENSG0000	291.0474	-0.19168	0.068504	-2.79812	0.00514	0.010931	FAM212A	protein_cc
ENSG0000	290.929	0.109009	0.126673	0.860551	0.389486	0.486095	SLAMF8	protein_cc
ENSG0000	290.8915	-0.00217	0.119392	-0.01815	0.985523	0.990097	NEUROD1	protein_cc
ENSG0000	290.8852	-0.37508	0.099957	-3.75239	0.000175	0.000491	LINC0095	lincRNA
ENSG0000	290.8785	0.318357	0.041169	7.733012	1.05E-14	1.52E-13	ZNF829	protein_cc
ENSG0000	290.8059	0.044757	0.097195	0.460491	0.645164	0.724633	CD180	protein_cc
ENSG0000	290.7529	-1.02219	0.119813	-8.53154	1.44E-17	3.36E-16	HMGCLL1	protein_cc
ENSG0000	290.7483	0.0496	0.041962	1.18203	0.237194	0.322213	RP11-332	lincRNA
ENSG0000	290.7095	0.320502	0.067191	4.769983	1.84E-06	7.15E-06	HIGD1B	protein_cc
ENSG0000	290.6447	-0.11821	0.050559	-2.33799	0.019388	0.036202	ALG11	protein_cc
ENSG0000	290.582	-0.10907	0.060401	-1.80583	0.070945	0.114615	ADCY4	protein_cc
ENSG0000	290.4685	0.37485	0.036894	10.16018	2.99E-24	2.25E-22	ZNF433	protein_cc
ENSG0000	290.2695	0.141657	0.063179	2.242145	0.024952	0.045454	MC1R	protein_cc
ENSG0000	290.0533	-0.08042	0.063355	-1.26937	0.204309	0.284377	RP11-274	pseudoge
ENSG0000	289.8031	1.356771	0.140001	9.691133	3.29E-22	1.66E-20	DLGAP5	protein_cc
ENSG0000	289.5205	0.331914	0.044505	7.457862	8.79E-14	1.08E-12	CENPP	protein_cc
ENSG0000	289.4103	-0.43887	0.094359	-4.65113	3.30E-06	1.23E-05	FRMPD1	protein_cc
ENSG0000	289.3535	0.626542	0.108707	5.76359	8.23E-09	4.63E-08	SLC47A1	protein_cc

ENSG0000(	289.2799	0.627184	0.062803	9.986475	1.75E-23	1.15E-21	LINC0111(	lincRNA
ENSG0000(	289.2229	-0.14873	0.059052	-2.51866	0.01178	0.023158	TRAPPC5	protein_cc
ENSG0000(	289.0986	-0.21333	0.043797	-4.87091	1.11E-06	4.46E-06	RP11-676	pseudoge
ENSG0000(	288.9051	0.184089	0.10274	1.791796	0.073166	0.117819	ANPEP	protein_cc
ENSG0000(	288.7428	-0.13081	0.057573	-2.27215	0.023078	0.042387	CARD10	protein_cc
ENSG0000(	288.6639	-0.64028	0.104202	-6.14459	8.02E-10	5.30E-09	C10orf85	protein_cc
ENSG0000(	288.6285	-0.24413	0.162269	-1.50447	0.13246	0.196574	MMP19	protein_cc
ENSG0000(	288.5703	-1.06874	0.1403	-7.61749	2.59E-14	3.48E-13	PCDHGA3	protein_cc
ENSG0000(	288.4927	0.14531	0.080945	1.79518	0.072625	0.117049	NEXN	protein_cc
ENSG0000(	288.4077	-0.05493	0.058546	-0.93832	0.348082	0.443624	SGK494	protein_cc
ENSG0000(	288.0744	0.551699	0.065003	8.487276	2.12E-17	4.82E-16	SNHG9	lincRNA
ENSG0000(	287.9242	0.311392	0.065538	4.751301	2.02E-06	7.78E-06	LTB4R	protein_cc
ENSG0000(	287.9017	0.206187	0.053112	3.882089	0.000104	0.000302	PRSS27	protein_cc
ENSG0000(	287.5022	1.066314	0.122359	8.714633	2.92E-18	7.58E-17	TTK	protein_cc
ENSG0000(	286.7883	-0.31463	0.055819	-5.63662	1.73E-08	9.27E-08	PAOX	protein_cc
ENSG0000(	286.7539	0.127445	0.065429	1.947834	0.051435	0.086628	PCSK4	protein_cc
ENSG0000(	286.6717	-0.49516	0.161418	-3.06756	0.002158	0.004948	C5orf38	protein_cc
ENSG0000(	286.5896	-0.1087	0.115655	-0.93989	0.347276	0.442761	SPTSSB	protein_cc
ENSG0000(	286.4998	-0.26158	0.070392	-3.71599	0.000202	0.00056	PRKCQ-A!	lincRNA
ENSG0000(	286.4994	-0.17788	0.097793	-1.81895	0.068919	0.11179	PCDHGA4	protein_cc
ENSG0000(	286.4889	-0.45414	0.083877	-5.41437	6.15E-08	3.02E-07	RUNX2	protein_cc
ENSG0000(	286.1933	0.081525	0.049509	1.646663	0.099627	0.154043	RP11-455	antisense
ENSG0000(	286.0554	-0.53133	0.107277	-4.95288	7.31E-07	3.02E-06	GABRB1	protein_cc
ENSG0000(	285.7492	0.111099	0.039538	2.80993	0.004955	0.010567	ZNF669	protein_cc
ENSG0000(	285.5381	0.135544	0.045851	2.956182	0.003115	0.006919	APOM	protein_cc
ENSG0000(	285.3439	1.041737	0.136338	7.640831	2.16E-14	2.94E-13	CRNDE	lincRNA
ENSG0000(	285.2857	-0.09231	0.130242	-0.70876	0.478471	0.57393	CLVS1	protein_cc
ENSG0000(	285.0071	-0.70398	0.119828	-5.87495	4.23E-09	2.49E-08	MCF2	protein_cc
ENSG0000(	284.9256	-0.11095	0.085356	-1.29989	0.193639	0.271606	PLAGL1	protein_cc
ENSG0000(	284.7323	-0.35748	0.112935	-3.16536	0.001549	0.003649	KCTD4	protein_cc
ENSG0000(	284.4914	0.318309	0.100802	3.157766	0.00159	0.003737	FBP1	protein_cc
ENSG0000(	283.9701	-0.74457	0.076875	-9.68545	3.48E-22	1.74E-20	CBFA2T3	protein_cc
ENSG0000(	283.8232	0.079826	0.077274	1.033022	0.301594	0.394247	TUFT1	protein_cc
ENSG0000(	283.8138	-0.11464	0.035239	-3.25311	0.001141	0.002751	MROH8	protein_cc
ENSG0000(	283.6306	-0.03699	0.07045	-0.52503	0.59956	0.684422	ANKRD10	sense_intr
ENSG0000(	283.5818	-0.28134	0.084302	-3.33735	0.000846	0.002089	RP11-326	antisense
ENSG0000(	283.461	0.305494	0.169871	1.798393	0.072115	0.116314	UBXN10	protein_cc
ENSG0000(	283.4428	0.076177	0.070379	1.082377	0.279085	0.36949	LLGL2	protein_cc
ENSG0000(	283.307	0.090086	0.036897	2.441585	0.014623	0.028162	RWDD3	protein_cc
ENSG0000(	283.0404	0.113358	0.043993	2.57674	0.009974	0.019929	ENTPD3-/	antisense
ENSG0000(	283.0285	-0.10574	0.029483	-3.5865	0.000335	0.000894	METTL20	protein_cc
ENSG0000(	282.909	0.812959	0.121056	6.715564	1.87E-11	1.59E-10	E2F2	protein_cc
ENSG0000(	282.6842	0.271104	0.039798	6.811918	9.63E-12	8.58E-11	ZNF613	protein_cc
ENSG0000(	282.5194	0.002946	0.031448	0.093693	0.925353	0.9474	ZRANB3	protein_cc
ENSG0000(	282.4234	0.356777	0.050253	7.09966	1.25E-12	1.28E-11	SNHG10	antisense
ENSG0000(	282.4202	-1.49612	0.13	-11.5086	1.19E-30	3.15E-28	ANO3	protein_cc
ENSG0000(	282.1145	-0.03326	0.058859	-0.56502	0.572057	0.659507	EXO5	protein_cc
ENSG0000(	281.9788	0.005333	0.036539	0.145946	0.883964	0.917149	ZNF280C	protein_cc
ENSG0000(	281.9401	-0.0668	0.069	-0.96807	0.33301	0.427533	MYH3	protein_cc
ENSG0000(	281.9033	-0.03872	0.046097	-0.83994	0.400942	0.497746	MAP2K6	protein_cc
ENSG0000(	281.6964	-0.03145	0.099635	-0.31565	0.752266	0.813447	CRABP2	protein_cc
ENSG0000(	281.6727	-0.03117	0.03808	-0.81846	0.413092	0.509982	TAF5	protein_cc
ENSG0000(	281.5127	0.144723	0.163665	0.884261	0.376555	0.473012	GJB2	protein_cc
ENSG0000(	281.4538	0.254102	0.126483	2.008976	0.04454	0.076265	RP11-536	lincRNA
ENSG0000(	281.4502	-0.90845	0.138739	-6.5479	5.84E-11	4.60E-10	PRMT8	protein_cc
ENSG0000(	281.1351	-0.67686	0.172098	-3.93299	8.39E-05	0.000249	CBLN4	protein_cc
ENSG0000(	280.9187	-0.18457	0.081029	-2.27778	0.02274	0.041817	TNFRSF25	protein_cc
ENSG0000(	280.8506	0.053018	0.033402	1.587291	0.112447	0.170965	ZNF668	protein_cc
ENSG0000(	280.8132	-0.82224	0.103601	-7.93667	2.08E-15	3.41E-14	C1QTNF4	protein_cc

ENSG0000(	280.7168	0.124339	0.048961	2.539542	0.0111	0.021953	RPL23AP7	pseudoge
ENSG0000(	280.6453	-0.16253	0.100455	-1.61792	0.10568	0.162	CTD-2554	lincRNA
ENSG0000(	280.5831	-0.10687	0.078687	-1.3582	0.174401	0.248676	HLX	protein_cc
ENSG0000(	280.5474	0.781115	0.077302	10.10477	5.26E-24	3.82E-22	PLK4	protein_cc
ENSG0000(	280.5458	-0.84097	0.101428	-8.29132	1.12E-16	2.25E-15	VIPR1	protein_cc
ENSG0000(	280.4555	-0.06108	0.037997	-1.60735	0.107979	0.165032	C8orf44	protein_cc
ENSG0000(	280.3665	-0.41977	0.061804	-6.7919	1.11E-11	9.73E-11	ZNF467	protein_cc
ENSG0000(	280.3365	0.041723	0.113073	0.368991	0.712135	0.780456	ITGA4	protein_cc
ENSG0000(	280.0247	0.641139	0.101766	6.300115	2.97E-10	2.11E-09	BATF3	protein_cc
ENSG0000(	280.0068	0.406208	0.090682	4.479486	7.48E-06	2.64E-05	MIR24-2	lincRNA
ENSG0000(	280.0036	0.376402	0.054425	6.915907	4.65E-12	4.35E-11	ATAD5	protein_cc
ENSG0000(	279.6938	0.127204	0.053417	2.381359	0.017249	0.032592	AC074289	antisense
ENSG0000(	279.5355	-1.25447	0.135165	-9.28105	1.68E-20	6.19E-19	SV2C	protein_cc
ENSG0000(	279.3503	0.107567	0.038587	2.787626	0.00531	0.011257	RP11-115(	antisense
ENSG0000(	279.3154	0.257401	0.039485	6.519043	7.08E-11	5.52E-10	IQCC	protein_cc
ENSG0000(	279.0107	-0.11601	0.050734	-2.28659	0.02222	0.04096	C16orf86	protein_cc
ENSG0000(	278.9405	-2.29316	0.264332	-8.67529	4.13E-18	1.04E-16	RP11-111	lincRNA
ENSG0000(	278.9034	-0.11011	0.068166	-1.6154	0.106224	0.162712	AK8	protein_cc
ENSG0000(	278.8923	0.25892	0.14171	1.827113	0.067683	0.110041	SCRT2	protein_cc
ENSG0000(	278.8784	-0.43381	0.124679	-3.47941	0.000503	0.001296	RGS8	protein_cc
ENSG0000(	278.7662	0.096622	0.046303	2.086732	0.036912	0.064617	SLC25A15	protein_cc
ENSG0000(	278.6728	0.015106	0.039488	0.382562	0.702044	0.772279	LINC0047(	processed
ENSG0000(	278.4701	0.002884	0.067322	0.042841	0.965828	0.97649	AC005280	pseudoge
ENSG0000(	278.3808	-0.55415	0.076176	-7.27464	3.47E-13	3.87E-12	RP4-613B	antisense
ENSG0000(	278.3113	0.336516	0.117849	2.855489	0.004297	0.009283	SNAI2	protein_cc
ENSG0000(	278.3041	0.140096	0.048946	2.862221	0.004207	0.009113	RP11-111	lincRNA
ENSG0000(	277.6723	0.227116	0.083738	2.712209	0.006684	0.013871	TES	protein_cc
ENSG0000(	277.5147	-0.22813	0.069178	-3.29778	0.000975	0.002378	DENND1C	protein_cc
ENSG0000(	277.4908	-0.47767	0.098937	-4.82807	1.38E-06	5.45E-06	ADAMTS1	protein_cc
ENSG0000(	277.3938	0.156734	0.045714	3.428567	0.000607	0.001541	GLRX2	protein_cc
ENSG0000(	277.3083	0.045252	0.150597	0.300481	0.763811	0.822728	ALOX15B	protein_cc
ENSG0000(	277.209	0.319479	0.042611	7.497594	6.50E-14	8.20E-13	AGBL3	protein_cc
ENSG0000(	276.8187	0.005856	0.063754	0.091856	0.926813	0.948498	ARHGEF1!	protein_cc
ENSG0000(	276.7601	0.065868	0.064494	1.021295	0.307115	0.400155	PPP1R32	protein_cc
ENSG0000(	276.655	-0.25665	0.068506	-3.74633	0.000179	0.000502	ANKRD36(	protein_cc
ENSG0000(	276.5476	-0.35403	0.108801	-3.25391	0.001138	0.002745	RP11-105	lincRNA
ENSG0000(	276.501	0.172415	0.081747	2.10914	0.034932	0.061531	RP11-568	pseudoge
ENSG0000(	276.4176	-0.59499	0.122545	-4.85527	1.20E-06	4.80E-06	COLCA1	protein_cc
ENSG0000(	276.3974	0.501281	0.079086	6.338417	2.32E-10	1.67E-09	TPGS1	protein_cc
ENSG0000(	276.1744	0.012916	0.039955	0.32326	0.746498	0.808944	NUDT17	protein_cc
ENSG0000(	276.0425	0.339556	0.143793	2.361423	0.018205	0.034218	RIT2	protein_cc
ENSG0000(	275.7455	-0.19499	0.095534	-2.04102	0.041249	0.071295	SIRPB2	protein_cc
ENSG0000(	275.7151	0.593568	0.06515	9.110769	8.18E-20	2.71E-18	TRAIP	protein_cc
ENSG0000(	275.5866	-0.02263	0.036176	-0.62549	0.531649	0.623195	TMEM143	protein_cc
ENSG0000(	275.4891	0.290655	0.123755	2.348639	0.018842	0.035278	ARX	protein_cc
ENSG0000(	275.252	0.257425	0.077756	3.310669	0.000931	0.00228	SLC25A45	protein_cc
ENSG0000(	275.1676	-0.19704	0.129691	-1.51932	0.128681	0.191744	COL15A1	protein_cc
ENSG0000(	274.8464	0.479974	0.102544	4.680662	2.86E-06	1.08E-05	ISG20	protein_cc
ENSG0000(	274.5968	-0.1871	0.097773	-1.91356	0.055676	0.09281	ASPDH	protein_cc
ENSG0000(	274.3767	-0.13428	0.085104	-1.57789	0.114591	0.173788	ZDHHC11	protein_cc
ENSG0000(	274.273	0.354433	0.090439	3.919035	8.89E-05	0.000262	DPY19L2	protein_cc
ENSG0000(	274.0937	-0.14699	0.059699	-2.46226	0.013807	0.026726	RP11-694	pseudoge
ENSG0000(	274.0766	0.261104	0.088882	2.937643	0.003307	0.007309	FZD2	protein_cc
ENSG0000(	274.0495	0.145263	0.036925	3.933987	8.35E-05	0.000248	LIN37	protein_cc
ENSG0000(	273.8396	0.346244	0.042489	8.149	3.67E-16	6.80E-15	TRAPPC2F	protein_cc
ENSG0000(	273.8199	-0.47724	0.068609	-6.9559	3.50E-12	3.35E-11	RAB37	protein_cc
ENSG0000(	273.7455	0.35809	0.092413	3.874899	0.000107	0.00031	RTKN2	protein_cc
ENSG0000(	273.7333	-0.11067	0.067853	-1.63102	0.102886	0.158343	C1orf64	protein_cc
ENSG0000(	273.6735	-1.81046	0.198323	-9.12882	6.93E-20	2.32E-18	KCNV1	protein_cc

ENSG0000(	273.6707	-0.31887	0.064459	-4.94691	7.54E-07	3.10E-06	FILIP1	protein_cc
ENSG0000(	273.6148	0.11122	0.046477	2.393026	0.01671	0.031714	CTA-384D	antisense
ENSG0000(	273.5064	-0.05776	0.041313	-1.39801	0.162111	0.233663	CTB-55O6	antisense
ENSG0000(	273.4507	-0.33602	0.088115	-3.81339	0.000137	0.000391	TMEM257	protein_cc
ENSG0000(	273.4484	-0.15009	0.062999	-2.38234	0.017203	0.032519	OSGIN1	protein_cc
ENSG0000(	273.4247	0.039941	0.03557	1.122874	0.261491	0.349903	ZNF324B	protein_cc
ENSG0000(	273.3628	-0.07578	0.027461	-2.75962	0.005787	0.012165	RP11-400	antisense
ENSG0000(	273.2264	0.229508	0.069076	3.32256	0.000892	0.002193	MDH1B	protein_cc
ENSG0000(	273.2167	-0.1037	0.10884	-0.95277	0.340707	0.435809	CFD	protein_cc
ENSG0000(	273.0999	0.166202	0.06828	2.434124	0.014928	0.028659	MAP10	protein_cc
ENSG0000(	273.0721	0.395365	0.084621	4.672205	2.98E-06	1.12E-05	TIFA	protein_cc
ENSG0000(	272.786	-0.43853	0.076713	-5.71653	1.09E-08	6.00E-08	WNT5B	protein_cc
ENSG0000(	272.7065	0.004001	0.067278	0.059473	0.952575	0.96718	FUT4	protein_cc
ENSG0000(	272.555	0.196177	0.061838	3.172439	0.001512	0.003568	CTD-2287	pseudoge
ENSG0000(	272.4714	-0.49061	0.095449	-5.14003	2.75E-07	1.21E-06	BCL11B	protein_cc
ENSG0000(	272.2572	0.079594	0.039666	2.006617	0.044791	0.07665	LETM2	protein_cc
ENSG0000(	272.1859	-0.27451	0.128856	-2.13034	0.033143	0.058663	LINC0096(	lincRNA
ENSG0000(	271.8547	1.15955	0.109122	10.62614	2.25E-26	2.59E-24	SKA3	protein_cc
ENSG0000(	271.8491	-0.13296	0.03905	-3.40485	0.000662	0.001669	RP11-37B	lincRNA
ENSG0000(	271.8348	1.040996	0.115773	8.99167	2.44E-19	7.47E-18	SLC10A4	protein_cc
ENSG0000(	271.7846	-0.42617	0.111943	-3.80706	0.000141	0.0004	GPR123-A	antisense
ENSG0000(	271.6164	-1.56873	0.152902	-10.2597	1.07E-24	8.79E-23	OLFM3	protein_cc
ENSG0000(	271.6152	0.088387	0.041113	2.149853	0.031567	0.056141	ZNF813	protein_cc
ENSG0000(	271.5911	0.196806	0.077665	2.534047	0.011275	0.022265	SRPK3	protein_cc
ENSG0000(	271.5084	-0.05643	0.042731	-1.32063	0.186625	0.263267	VCPKMT	protein_cc
ENSG0000(	271.4608	-0.05489	0.057294	-0.95808	0.338024	0.432902	RP13-104	pseudoge
ENSG0000(	271.4528	-0.80357	0.103956	-7.72987	1.08E-14	1.56E-13	WDR11-A	antisense
ENSG0000(	271.3021	-0.13781	0.080487	-1.71223	0.086855	0.136742	NOMO3	protein_cc
ENSG0000(	271.0244	-0.22346	0.086396	-2.58645	0.009697	0.019427	HMCN1	protein_cc
ENSG0000(	270.8417	-0.41521	0.120523	-3.44506	0.000571	0.001458	FRAS1	protein_cc
ENSG0000(	270.8367	-0.00742	0.045338	-0.1637	0.869968	0.906654	MKLN1-A	processed
ENSG0000(	270.8187	-0.19171	0.050261	-3.81431	0.000137	0.00039	DNAJC27-	antisense
ENSG0000(	270.8175	0.121848	0.051137	2.382784	0.017182	0.032488	BX936347	pseudoge
ENSG0000(	270.6978	-0.19839	0.041046	-4.83337	1.34E-06	5.32E-06	AC006978	pseudoge
ENSG0000(	270.3984	0.55873	0.18849	2.964246	0.003034	0.006754	TREM1	protein_cc
ENSG0000(	269.8995	0.244212	0.053359	4.576798	4.72E-06	1.72E-05	CTD-3064	antisense
ENSG0000(	269.6739	0.309227	0.048067	6.433201	1.25E-10	9.40E-10	TMEM44-	antisense
ENSG0000(	269.6507	0.202279	0.103457	1.955205	0.050559	0.085324	CLEC2B	protein_cc
ENSG0000(	268.4837	-0.45402	0.077949	-5.82462	5.72E-09	3.30E-08	GHR	protein_cc
ENSG0000(	268.4094	0.32806	0.12396	2.646495	0.008133	0.016588	TC2N	protein_cc
ENSG0000(	268.2527	-0.0666	0.054995	-1.21109	0.225862	0.309282	RP11-4O1	sense_ove
ENSG0000(	268.1232	0.104662	0.052	2.012709	0.044145	0.075659	RUSC1-AS	antisense
ENSG0000(	267.9759	-0.26742	0.076533	-3.49414	0.000476	0.001232	RP11-384	lincRNA
ENSG0000(	267.9416	0.29629	0.043572	6.800045	1.05E-11	9.25E-11	ZSCAN5A	protein_cc
ENSG0000(	267.858	-0.55598	0.065877	-8.43974	3.18E-17	7.02E-16	RNF125	protein_cc
ENSG0000(	267.7862	-0.2666	0.094028	-2.8353	0.004578	0.009825	PLA2G7	protein_cc
ENSG0000(	267.6493	0.103812	0.035241	2.945737	0.003222	0.007136	ZNF543	protein_cc
ENSG0000(	267.3716	0.187377	0.064391	2.910012	0.003614	0.007927	RILP	protein_cc
ENSG0000(	267.1723	0.180099	0.128154	1.405328	0.159924	0.230964	DUSP9	protein_cc
ENSG0000(	267.0794	0.746086	0.128518	5.805304	6.42E-09	3.68E-08	TXLNB	protein_cc
ENSG0000(	266.9697	0.166796	0.040356	4.133078	3.58E-05	0.000113	CCDC157	protein_cc
ENSG0000(	266.6813	0.993633	0.094582	10.50556	8.14E-26	8.46E-24	XRCC6BP1	protein_cc
ENSG0000(	266.6705	0.674907	0.129136	5.226341	1.73E-07	7.89E-07	LRRC61	protein_cc
ENSG0000(	266.6588	0.292525	0.058302	5.017365	5.24E-07	2.21E-06	PDIA3P1	pseudoge
ENSG0000(	266.6527	0.401049	0.137769	2.911029	0.003602	0.007904	OASL	protein_cc
ENSG0000(	266.4236	0.065319	0.071605	0.912205	0.361661	0.457548	PDK3	protein_cc
ENSG0000(	266.2742	0.629497	0.080007	7.867984	3.60E-15	5.69E-14	RP11-169	pseudoge
ENSG0000(	266.1509	-1.01159	0.110978	-9.11529	7.85E-20	2.61E-18	ADRA2A	protein_cc
ENSG0000(	266.1219	-0.23993	0.054855	-4.37382	1.22E-05	4.15E-05	RAB41	protein_cc

ENSG0000(	265.9357	0.899905	0.095104	9.462363	3.01E-21	1.28E-19	CENPM	protein_cc
ENSG0000(	265.7006	-0.27484	0.072449	-3.79361	0.000148	0.000421	SYCP2	protein_cc
ENSG0000(	265.5037	1.602236	0.18117	8.843817	9.25E-19	2.58E-17	STAC	protein_cc
ENSG0000(	265.4448	-0.02961	0.054945	-0.53888	0.589966	0.675818	RP13-36G	sense_intr
ENSG0000(	265.4443	0.255699	0.055699	4.590761	4.42E-06	1.61E-05	WEE2-AS1	antisense
ENSG0000(	265.3967	-0.17228	0.142188	-1.21165	0.225647	0.309049	ST8SIA2	protein_cc
ENSG0000(	265.1834	-0.14263	0.054321	-2.62569	0.008647	0.017528	RP3-368A	sense_intr
ENSG0000(	264.945	1.147054	0.16967	6.760507	1.38E-11	1.19E-10	WISP1	protein_cc
ENSG0000(	264.9075	-0.20206	0.066661	-3.03122	0.002436	0.005535	ZFAND4	protein_cc
ENSG0000(	264.7519	-0.27058	0.090628	-2.98557	0.002831	0.006335	BMP6	protein_cc
ENSG0000(	264.7066	-0.45572	0.142926	-3.18849	0.00143	0.00339	VWC2L	protein_cc
ENSG0000(	264.6218	1.139844	0.10393	10.96739	5.48E-28	8.26E-26	CDKN3	protein_cc
ENSG0000(	264.5831	-0.06016	0.068806	-0.87437	0.381916	0.478547	HHEX	protein_cc
ENSG0000(	264.2666	-0.42681	0.065907	-6.47596	9.42E-11	7.21E-10	IQCJ-SCH	protein_cc
ENSG0000(	264.1761	0.410247	0.082239	4.988497	6.09E-07	2.54E-06	HIST1H2B	protein_cc
ENSG0000(	264.1678	1.075134	0.106036	10.1393	3.70E-24	2.74E-22	HMMR	protein_cc
ENSG0000(	264.0068	0.704582	0.127144	5.541611	3.00E-08	1.54E-07	HCG4P5	pseudoge
ENSG0000(	263.9743	0.148932	0.08085	1.842071	0.065465	0.106862	ADORA2B	protein_cc
ENSG0000(	263.8717	-0.26184	0.064484	-4.0606	4.89E-05	0.000151	KCNK6	protein_cc
ENSG0000(	263.6756	0.890934	0.10045	8.869423	7.35E-19	2.09E-17	RPS7P1	pseudoge
ENSG0000(	263.66	-0.07559	0.083893	-0.90098	0.3676	0.46376	SPN	protein_cc
ENSG0000(	263.642	0.029219	0.042922	0.680742	0.496035	0.590222	RP11-95D	lincRNA
ENSG0000(	263.6294	0.296301	0.048457	6.114668	9.68E-10	6.33E-09	RP13-103	antisense
ENSG0000(	263.5359	0.056802	0.036249	1.566995	0.117116	0.177048	ZNF620	protein_cc
ENSG0000(	263.5178	-0.04132	0.031307	-1.3198	0.186901	0.263594	GDPGP1	protein_cc
ENSG0000(	263.411	0.075575	0.047693	1.584601	0.113057	0.171759	ZBED3-AS	antisense
ENSG0000(	263.2465	0.165154	0.085966	1.921163	0.054711	0.091433	ARHGAP1	protein_cc
ENSG0000(	263.0871	0.462205	0.096925	4.768679	1.85E-06	7.19E-06	HIST1H2B	protein_cc
ENSG0000(	263.0425	-0.3235	0.105555	-3.06478	0.002178	0.004989	MRGPRF	protein_cc
ENSG0000(	262.9866	-0.07277	0.038597	-1.88535	0.059383	0.098165	NLRC3	polymorpl
ENSG0000(	262.5976	0.049124	0.038184	1.286504	0.198267	0.277162	RP11-213	pseudoge
ENSG0000(	262.5922	0.235812	0.062381	3.780219	0.000157	0.000442	DPY19L1P	pseudoge
ENSG0000(	262.1044	-0.58142	0.088168	-6.59441	4.27E-11	3.44E-10	PLEKHM3	protein_cc
ENSG0000(	262.0174	0.302837	0.042453	7.133427	9.79E-13	1.02E-11	ZNF230	protein_cc
ENSG0000(	262.0039	0.148993	0.035287	4.222299	2.42E-05	7.83E-05	TAF1A	protein_cc
ENSG0000(	261.9523	0.149151	0.049109	3.037127	0.002388	0.005434	ANKRD37	protein_cc
ENSG0000(	261.9306	1.561263	0.11195	13.94608	3.32E-44	6.83E-41	RP11-71N	lincRNA
ENSG0000(	261.7308	-0.02308	0.058925	-0.39167	0.695303	0.766606	FGF14-AS	lincRNA
ENSG0000(	261.577	0.111216	0.065841	1.689168	0.091187	0.142596	RP11-296	pseudoge
ENSG0000(	261.5612	0.023658	0.045196	0.523457	0.600656	0.685388	TOPORS-/	antisense
ENSG0000(	261.3906	-0.20784	0.081291	-2.55675	0.010565	0.020998	PRRG4	protein_cc
ENSG0000(	261.323	0.556761	0.121101	4.597494	4.28E-06	1.57E-05	SPEF1	protein_cc
ENSG0000(	261.2139	-0.00569	0.113134	-0.05029	0.959888	0.972526	ZBED6	protein_cc
ENSG0000(	260.9168	1.146004	0.122288	9.371382	7.16E-21	2.81E-19	FAM111B	protein_cc
ENSG0000(	260.9088	0.095843	0.063051	1.520096	0.128487	0.191536	CCDC39	protein_cc
ENSG0000(	260.7307	0.418863	0.063774	6.56796	5.10E-11	4.06E-10	B9D2	protein_cc
ENSG0000(	260.4535	0.061912	0.079071	0.782988	0.433634	0.530674	RP11-223	sense_ove
ENSG0000(	260.4044	0.510334	0.059773	8.537817	1.37E-17	3.20E-16	SETD9	protein_cc
ENSG0000(	260.1003	0.183306	0.039453	4.646224	3.38E-06	1.26E-05	ZNF256	protein_cc
ENSG0000(	260.0628	-0.31668	0.077429	-4.09001	4.31E-05	0.000134	SOGA3	protein_cc
ENSG0000(	260.0602	-0.07667	0.059928	-1.2794	0.200758	0.280196	ZNF337	protein_cc
ENSG0000(	260.0216	0.000788	0.048072	0.016398	0.986917	0.991062	C10orf25	protein_cc
ENSG0000(	260.0179	-0.18702	0.035353	-5.29022	1.22E-07	5.71E-07	IPPK	protein_cc
ENSG0000(	259.8706	-0.32281	0.116722	-2.76563	0.005681	0.011962	GRIP2	processed
ENSG0000(	259.8182	-0.27569	0.063019	-4.37474	1.22E-05	4.14E-05	AKAP3	protein_cc
ENSG0000(	259.7308	-0.01403	0.064552	-0.21734	0.82794	0.873995	SLC12A8	protein_cc
ENSG0000(	259.6726	0.530402	0.080054	6.62557	3.46E-11	2.83E-10	EEF1A1P9	pseudoge
ENSG0000(	259.6666	0.567489	0.085458	6.640557	3.13E-11	2.57E-10	BCL2L12	protein_cc
ENSG0000(	259.5138	-0.14532	0.042957	-3.38292	0.000717	0.001796	HSD17B1	protein_cc

ENSG0000(	259.2985	-0.2786	0.082875	-3.36173	0.000775	0.001927	SIX4	protein_cc
ENSG0000(	259.0066	0.530608	0.073349	7.234027	4.69E-13	5.11E-12	CHEK2	protein_cc
ENSG0000(	258.874	-0.03428	0.040482	-0.8469	0.39705	0.493644	TPK1	protein_cc
ENSG0000(	258.6529	0.260223	0.046985	5.538412	3.05E-08	1.57E-07	ZNF630	protein_cc
ENSG0000(	258.6071	0.109292	0.110962	0.984951	0.324648	0.418824	EMR2	protein_cc
ENSG0000(	258.3464	-0.13658	0.074305	-1.83812	0.066045	0.10769	C1orf94	protein_cc
ENSG0000(	258.2839	-0.60731	0.100077	-6.06841	1.29E-09	8.29E-09	MIPOL1	protein_cc
ENSG0000(	258.2512	1.21215	0.123663	9.802008	1.10E-22	6.06E-21	NEK2	protein_cc
ENSG0000(	257.7383	0.127487	0.076354	1.669684	0.094982	0.147743	SYTL3	protein_cc
ENSG0000(	257.4038	0.313925	0.043857	7.15797	8.19E-13	8.63E-12	C12orf45	protein_cc
ENSG0000(	257.3559	-1.07223	0.139133	-7.70652	1.29E-14	1.83E-13	ASIC2	protein_cc
ENSG0000(	256.9735	0.367374	0.116018	3.166534	0.001543	0.003636	COL4A3	protein_cc
ENSG0000(	256.9617	0.038234	0.088451	0.432265	0.665549	0.741906	RP11-332	antisense
ENSG0000(	256.8063	-0.18534	0.04341	-4.2696	1.96E-05	6.45E-05	BCKDHA	protein_cc
ENSG0000(	256.6833	-0.44355	0.083405	-5.31801	1.05E-07	4.96E-07	ZFP92	protein_cc
ENSG0000(	256.3328	0.428378	0.074617	5.741049	9.41E-09	5.23E-08	CUBN	protein_cc
ENSG0000(	256.2969	0.255854	0.036963	6.921996	4.45E-12	4.19E-11	RP5-1136	antisense
ENSG0000(	256.1528	0.196919	0.185102	1.063841	0.287401	0.378622	SERPINA5	protein_cc
ENSG0000(	256.1337	0.334722	0.064718	5.17198	2.32E-07	1.04E-06	TTC25	processed
ENSG0000(	256.1067	-0.08953	0.044008	-2.03443	0.041908	0.072304	ZNF487	protein_cc
ENSG0000(	255.8919	-0.55058	0.12162	-4.52708	5.98E-06	2.14E-05	GPR88	protein_cc
ENSG0000(	255.752	-0.19526	0.064639	-3.02075	0.002521	0.005713	LRRC6	protein_cc
ENSG0000(	255.4396	0.618084	0.172511	3.582867	0.00034	0.000906	HOXD8	protein_cc
ENSG0000(	255.2311	0.662994	0.088185	7.518252	5.55E-14	7.05E-13	RPL15P3	pseudoge
ENSG0000(	255.1427	-1.13712	0.151495	-7.50601	6.10E-14	7.71E-13	CLEC2L	protein_cc
ENSG0000(	255.1017	-0.12474	0.04559	-2.73607	0.006218	0.012985	RP11-111	protein_cc
ENSG0000(	255.0458	0.744109	0.121005	6.149391	7.78E-10	5.16E-09	MCM10	protein_cc
ENSG0000(	254.9813	0.227642	0.029165	7.805182	5.94E-15	9.00E-14	ZNF879	protein_cc
ENSG0000(	254.8995	0.026077	0.128297	0.203257	0.838934	0.882813	ANKRD22	protein_cc
ENSG0000(	254.1911	0.200179	0.111797	1.790556	0.073365	0.118061	PCDHA10	protein_cc
ENSG0000(	254.1568	-0.55939	0.097518	-5.73632	9.68E-09	5.38E-08	GALNT14	protein_cc
ENSG0000(	253.8812	-0.50643	0.132548	-3.82077	0.000133	0.00038	GPR12	protein_cc
ENSG0000(	253.8741	0.131284	0.074783	1.755539	0.079167	0.126111	PPP1R3G	protein_cc
ENSG0000(	253.8386	0.310911	0.072776	4.272162	1.94E-05	6.38E-05	STARD4-A	antisense
ENSG0000(	253.6066	0.110515	0.033767	3.272906	0.001064	0.002581	TMEM194	protein_cc
ENSG0000(	253.5196	-0.10018	0.128183	-0.7815	0.434507	0.531552	GDNF-AS	lincRNA
ENSG0000(	253.3665	1.717475	0.130877	13.12282	2.44E-39	3.13E-36	OTX1	protein_cc
ENSG0000(	253.2572	0.198208	0.041624	4.761824	1.92E-06	7.42E-06	C4orf36	protein_cc
ENSG0000(	253.2392	-0.06677	0.03793	-1.76033	0.078351	0.125029	AQP11	protein_cc
ENSG0000(	253.1429	0.002516	0.086464	0.029094	0.97679	0.983654	OSCAR	protein_cc
ENSG0000(	252.9857	-0.0322	0.040747	-0.79021	0.429404	0.526454	FAM227B	protein_cc
ENSG0000(	252.7865	-0.52279	0.074827	-6.98657	2.82E-12	2.73E-11	SLC9A5	protein_cc
ENSG0000(	252.6758	0.326516	0.052188	6.256518	3.94E-10	2.73E-09	POLR2J4	processed
ENSG0000(	252.5918	1.299083	0.134852	9.633375	5.78E-22	2.75E-20	RPS3AP6	pseudoge
ENSG0000(	252.4921	0.449602	0.129528	3.47109	0.000518	0.001333	TP73	protein_cc
ENSG0000(	252.4483	-0.09097	0.08895	-1.02266	0.306467	0.399478	BICC1	protein_cc
ENSG0000(	252.2026	0.208396	0.077298	2.695987	0.007018	0.014496	FLJ27365	protein_cc
ENSG0000(	251.9267	-0.54244	0.060632	-8.94629	3.68E-19	1.09E-17	SHE	protein_cc
ENSG0000(	251.8405	0.286221	0.061203	4.676589	2.92E-06	1.10E-05	NPIPP1	pseudoge
ENSG0000(	251.795	0.415555	0.078715	5.279261	1.30E-07	6.03E-07	ZBTB8B	protein_cc
ENSG0000(	251.7754	0.570115	0.081491	6.996052	2.63E-12	2.57E-11	RP11-543	pseudoge
ENSG0000(	251.7671	-0.16643	0.073	-2.27986	0.022616	0.041613	CTD-2587	sense_intr
ENSG0000(	251.7416	0.153487	0.025385	6.046404	1.48E-09	9.38E-09	ZNF674	protein_cc
ENSG0000(	251.6809	-0.55021	0.125493	-4.38438	1.16E-05	3.97E-05	GPR62	protein_cc
ENSG0000(	251.5557	-0.20505	0.079358	-2.58384	0.009771	0.019563	RP11-2E1	antisense
ENSG0000(	251.4692	-0.07493	0.063592	-1.17833	0.238664	0.323954	COLQ	protein_cc
ENSG0000(	251.4394	-0.33303	0.089087	-3.73823	0.000185	0.000517	GPR126	protein_cc
ENSG0000(	251.3314	0.066086	0.05194	1.272338	0.203253	0.28315	LPP-AS2	antisense
ENSG0000(	251.2716	-0.37357	0.095027	-3.93122	8.45E-05	0.00025	SAMD5	protein_cc

ENSG0000(	251.1277	0.128803	0.040591	3.173219	0.001508	0.00356	SASS6	protein_cc
ENSG0000(	251.1136	-0.48175	0.072307	-6.66249	2.69E-11	2.24E-10	LINC0093(	lincRNA
ENSG0000(	251.0937	-0.07249	0.053811	-1.34721	0.177914	0.252852	RP11-410	lincRNA
ENSG0000(	251.0433	-0.20223	0.138302	-1.46221	0.143682	0.210795	C8orf34	protein_cc
ENSG0000(	251.0007	-0.23589	0.10078	-2.34067	0.019249	0.03597	COLCA2	protein_cc
ENSG0000(	250.8006	-0.08711	0.036222	-2.40483	0.01618	0.030836	PET117	protein_cc
ENSG0000(	250.635	-0.05347	0.053163	-1.00577	0.314525	0.408016	FMO5	protein_cc
ENSG0000(	250.2307	0.18542	0.064581	2.871127	0.00409	0.008879	C7orf61	protein_cc
ENSG0000(	250.2057	0.233301	0.043863	5.318876	1.04E-07	4.94E-07	ZNF345	protein_cc
ENSG0000(	250.2015	0.203917	0.051794	3.937108	8.25E-05	0.000245	TPM3P9	pseudoge
ENSG0000(	249.8198	0.459171	0.064091	7.164341	7.82E-13	8.27E-12	KIF24	protein_cc
ENSG0000(	249.7381	-0.49079	0.112	-4.38206	1.18E-05	4.01E-05	ITGA8	protein_cc
ENSG0000(	249.53	0.164891	0.031755	5.192622	2.07E-07	9.34E-07	ZNF836	protein_cc
ENSG0000(	248.9558	0.054436	0.063664	0.855057	0.39252	0.489136	STAC3	protein_cc
ENSG0000(	248.6342	0.019046	0.031152	0.611386	0.540944	0.631713	HARBI1	protein_cc
ENSG0000(	248.44	0.161076	0.055784	2.887474	0.003883	0.008468	SLC19A3	protein_cc
ENSG0000(	248.3478	0.314398	0.067207	4.678092	2.90E-06	1.09E-05	DLGAP1-/	antisense
ENSG0000(	248.2898	-0.23212	0.077829	-2.98249	0.002859	0.006396	ZBTB20	protein_cc
ENSG0000(	248.2619	0.368344	0.082166	4.482909	7.36E-06	2.60E-05	RTP4	protein_cc
ENSG0000(	247.9664	-0.89742	0.112188	-7.99924	1.25E-15	2.13E-14	INHBA	protein_cc
ENSG0000(	247.7665	0.050217	0.077653	0.646689	0.517833	0.610507	KIAA1683	protein_cc
ENSG0000(	247.6671	-0.17572	0.064164	-2.73864	0.006169	0.012893	RP11-50E	antisense
ENSG0000(	247.6293	1.361778	0.111496	12.21371	2.63E-34	1.40E-31	CENPK	protein_cc
ENSG0000(	247.5101	0.285546	0.040254	7.093641	1.31E-12	1.33E-11	ZNF586	protein_cc
ENSG0000(	247.424	0.349252	0.053001	6.589575	4.41E-11	3.55E-10	PMM2	protein_cc
ENSG0000(	247.2133	0.067738	0.037221	1.819893	0.068775	0.111588	LRRC29	protein_cc
ENSG0000(	247.1168	0.362503	0.071425	5.075296	3.87E-07	1.67E-06	C7orf31	protein_cc
ENSG0000(	247.0922	0.001918	0.056072	0.034208	0.972711	0.980941	RP11-600	antisense
ENSG0000(	247.0685	0.090726	0.093281	0.972604	0.33075	0.425162	SCIMP	protein_cc
ENSG0000(	247.0122	0.268412	0.051328	5.229343	1.70E-07	7.77E-07	MAFG-AS	antisense
ENSG0000(	246.9326	0.347624	0.09272	3.749198	0.000177	0.000497	TMEM37	protein_cc
ENSG0000(	246.8404	0.166616	0.04965	3.355842	0.000791	0.001965	NAALADL	protein_cc
ENSG0000(	246.6877	0.923813	0.224241	4.119726	3.79E-05	0.000119	HP	protein_cc
ENSG0000(	246.4257	-0.22185	0.096295	-2.30389	0.021229	0.039293	RP1-239B	antisense
ENSG0000(	246.4142	0.19065	0.043875	4.345252	1.39E-05	4.69E-05	ZNF547	protein_cc
ENSG0000(	246.3965	0.193784	0.046621	4.156622	3.23E-05	0.000103	ZNF525	protein_cc
ENSG0000(	246.3196	0.237786	0.044446	5.350006	8.80E-08	4.20E-07	FAM122C	protein_cc
ENSG0000(	246.1864	-1.43582	0.106643	-13.4638	2.55E-41	3.89E-38	CCDC85A	protein_cc
ENSG0000(	246.182	0.018177	0.091352	0.198975	0.842282	0.885452	ACOT11	protein_cc
ENSG0000(	246.0585	-0.2208	0.061199	-3.60795	0.000309	0.000829	NR6A1	protein_cc
ENSG0000(	246.049	-0.40753	0.08884	-4.58719	4.49E-06	1.64E-05	KCNS3	protein_cc
ENSG0000(	246.0343	-0.11538	0.072739	-1.58625	0.112683	0.171265	C1orf132	processed
ENSG0000(	245.8347	0.245169	0.072887	3.363698	0.000769	0.001914	ZNF311	protein_cc
ENSG0000(	245.6246	-0.46642	0.13648	-3.4175	0.000632	0.001599	CNTNAP5	protein_cc
ENSG0000(	245.6154	-0.27773	0.079213	-3.50608	0.000455	0.001183	NFKBID	protein_cc
ENSG0000(	245.3777	-0.20206	0.058762	-3.43858	0.000585	0.00149	C2orf74	protein_cc
ENSG0000(	245.3559	-0.01917	0.044733	-0.42861	0.66821	0.743895	SLC25A5-	processed
ENSG0000(	245.3032	0.121671	0.038499	3.160402	0.001576	0.003706	AC007383	lincRNA
ENSG0000(	245.2786	-0.01329	0.059834	-0.22215	0.824196	0.871138	RP11-16P	antisense
ENSG0000(	245.1578	0.090545	0.052075	1.738749	0.082079	0.130044	RAD51-AS	processed
ENSG0000(	245.1498	-0.02788	0.074694	-0.37326	0.708955	0.777925	CEACAM1	protein_cc
ENSG0000(	245.0627	0.208238	0.133679	1.557744	0.119294	0.179846	FOSL1	protein_cc
ENSG0000(	245.0267	-0.03862	0.02398	-1.61041	0.107309	0.164179	MEMO1	protein_cc
ENSG0000(	244.8105	0.907751	0.090257	10.05745	8.52E-24	5.97E-22	RMI2	protein_cc
ENSG0000(	244.7504	-0.02377	0.040518	-0.58656	0.557497	0.64671	CEP72	protein_cc
ENSG0000(	244.7068	0.283136	0.06351	4.458107	8.27E-06	2.89E-05	RP11-423	pseudoge
ENSG0000(	244.6931	0.824522	0.11836	6.966205	3.26E-12	3.13E-11	FAM83D	protein_cc
ENSG0000(	244.3636	-0.2891	0.072287	-3.99942	6.35E-05	0.000192	SLC16A6	protein_cc
ENSG0000(	244.323	-0.29633	0.086101	-3.44161	0.000578	0.001475	CDH3	protein_cc

ENSG0000(	244.2729	-0.08613	0.062321	-1.38209	0.166944	0.239639	RP6-206I1	lincRNA
ENSG0000(	244.1235	-0.22876	0.044171	-5.17907	2.23E-07	9.99E-07	C22orf23	protein_cc
ENSG0000(	244.104	-0.66748	0.125218	-5.33055	9.79E-08	4.65E-07	EPHA7	protein_cc
ENSG0000(	244.0464	-0.03674	0.035472	-1.03566	0.300361	0.392826	SDCBP2-A	antisense
ENSG0000(	244.0139	0.474015	0.053121	8.92333	4.52E-19	1.33E-17	ZNF788	protein_cc
ENSG0000(	243.979	0.378611	0.104409	3.626238	0.000288	0.000776	TFR2	protein_cc
ENSG0000(	243.8332	-0.04336	0.101185	-0.42852	0.66827	0.743901	C10orf107	protein_cc
ENSG0000(	243.8303	-0.34773	0.076939	-4.51959	6.20E-06	2.21E-05	RELL2	protein_cc
ENSG0000(	243.8218	-0.03394	0.057569	-0.58954	0.555501	0.644976	OXCT1-AS	processed
ENSG0000(	243.579	0.869104	0.105406	8.245318	1.65E-16	3.23E-15	CTD-2521	lincRNA
ENSG0000(	243.5692	0.159516	0.043489	3.667978	0.000244	0.000668	ZNF396	protein_cc
ENSG0000(	243.5572	-0.65543	0.14924	-4.39177	1.12E-05	3.85E-05	PENK	protein_cc
ENSG0000(	243.4953	0.198561	0.048273	4.113264	3.90E-05	0.000122	PINK1-AS	antisense
ENSG0000(	243.4322	0.652923	0.109068	5.986391	2.15E-09	1.33E-08	AGMO	protein_cc
ENSG0000(	243.2931	-0.73082	0.081874	-8.9261	4.41E-19	1.30E-17	FAM83H	protein_cc
ENSG0000(	243.2213	-0.19089	0.068244	-2.7971	0.005156	0.010961	MIR568	miRNA
ENSG0000(	243.1591	0.87507	0.1134	7.71665	1.19E-14	1.71E-13	KIF14	protein_cc
ENSG0000(	243.0407	-0.1042	0.062079	-1.67858	0.093233	0.145348	AC034220	processed
ENSG0000(	242.9771	1.404248	0.190983	7.352725	1.94E-13	2.25E-12	IGF2BP3	protein_cc
ENSG0000(	242.907	0.269913	0.045539	5.927092	3.08E-09	1.86E-08	TMEM182	protein_cc
ENSG0000(	242.8991	0.701484	0.133411	5.258056	1.46E-07	6.71E-07	CD36	protein_cc
ENSG0000(	242.882	0.058513	0.128303	0.456057	0.648349	0.727218	ACP5	protein_cc
ENSG0000(	242.7288	0.14983	0.038194	3.922902	8.75E-05	0.000259	VMAC	protein_cc
ENSG0000(	242.5595	0.674237	0.096951	6.95442	3.54E-12	3.38E-11	NUAK2	protein_cc
ENSG0000(	242.4123	0.883371	0.109768	8.047606	8.44E-16	1.47E-14	RPL39P3	pseudoge
ENSG0000(	242.1596	-0.47585	0.084149	-5.65486	1.56E-08	8.40E-08	TNFRSF13	protein_cc
ENSG0000(	242.1418	0.644393	0.057935	11.12264	9.73E-29	1.76E-26	MTBP	protein_cc
ENSG0000(	242.063	0.061907	0.027406	2.258846	0.023893	0.043722	GMDS-AS	lincRNA
ENSG0000(	242.0447	-0.09095	0.088022	-1.03331	0.30146	0.394101	FKBP1B	protein_cc
ENSG0000(	241.717	0.102296	0.041889	2.442048	0.014604	0.028128	FANCM	protein_cc
ENSG0000(	241.6696	-0.53982	0.092992	-5.80496	6.44E-09	3.68E-08	SGK2	protein_cc
ENSG0000(	241.5072	0.461653	0.091826	5.027462	4.97E-07	2.11E-06	FAM109B	protein_cc
ENSG0000(	241.4855	-0.13401	0.092916	-1.44228	0.149223	0.217812	MTRNR2L	protein_cc
ENSG0000(	241.2519	-0.32671	0.086309	-3.78533	0.000154	0.000434	RPP25	protein_cc
ENSG0000(	241.2243	0.372243	0.096888	3.841997	0.000122	0.000351	F12	protein_cc
ENSG0000(	241.1752	0.198198	0.09586	2.067584	0.038679	0.067344	SMIM5	protein_cc
ENSG0000(	241.1656	0.241565	0.111828	2.160143	0.030762	0.054856	RP11-439	lincRNA
ENSG0000(	241.1162	-0.01829	0.045757	-0.39974	0.689346	0.761823	ARID3B	protein_cc
ENSG0000(	241.064	0.695421	0.194999	3.566281	0.000362	0.00096	ESM1	protein_cc
ENSG0000(	240.93	0.175297	0.076995	2.276741	0.022802	0.041916	IMPA2	protein_cc
ENSG0000(	240.4241	-0.01432	0.066997	-0.21375	0.830738	0.876408	AC144652	lincRNA
ENSG0000(	240.4165	-0.53353	0.071305	-7.48237	7.30E-14	9.12E-13	ZNF483	protein_cc
ENSG0000(	240.4019	-0.36919	0.118642	-3.11177	0.00186	0.004314	RANBP17	protein_cc
ENSG0000(	240.2108	-0.3473	0.113505	-3.05978	0.002215	0.005067	RP11-54C	lincRNA
ENSG0000(	240.1926	-0.27045	0.069468	-3.89312	9.90E-05	0.000289	ANKRD18I	pseudoge
ENSG0000(	240.1155	0.706102	0.060191	11.73102	8.84E-32	2.80E-29	ZNF530	protein_cc
ENSG0000(	239.8787	0.379977	0.10814	3.513749	0.000442	0.001153	MMRN1	protein_cc
ENSG0000(	239.7987	0.257258	0.050556	5.088617	3.61E-07	1.56E-06	RP11-730	pseudoge
ENSG0000(	239.518	0.337434	0.091991	3.668139	0.000244	0.000667	RP11-65J3	lincRNA
ENSG0000(	239.4381	-0.52152	0.079667	-6.54626	5.90E-11	4.65E-10	FAM101A	protein_cc
ENSG0000(	239.4283	-0.64148	0.154285	-4.15774	3.21E-05	0.000102	HMCN2	protein_cc
ENSG0000(	239.3964	-1.24821	0.105111	-11.8751	1.60E-32	5.91E-30	ADAMTSL	protein_cc
ENSG0000(	239.1174	-0.34154	0.093354	-3.65853	0.000254	0.000691	CSF2RB	protein_cc
ENSG0000(	239.0206	0.09332	0.066634	1.400489	0.161367	0.232738	LY6G5B	protein_cc
ENSG0000(	238.983	-0.04141	0.080709	-0.51302	0.607935	0.691662	BTBD19	protein_cc
ENSG0000(	238.9457	0.634074	0.093877	6.754303	1.44E-11	1.24E-10	PRPH	protein_cc
ENSG0000(	238.9153	-0.00337	0.060597	-0.05557	0.955681	0.9694	GIPC3	protein_cc
ENSG0000(	238.777	-1.15253	0.144538	-7.97386	1.54E-15	2.58E-14	FAM19A1	protein_cc
ENSG0000(	238.468	0.743709	0.126667	5.871383	4.32E-09	2.54E-08	AC016739	pseudoge

ENSG0000(	238.4304	-0.13818	0.060893	-2.26922	0.023255	0.042666	RP11-152	pseudoge
ENSG0000(	238.3743	0.095548	0.034238	2.790682	0.00526	0.011165	ZNF19	protein_cc
ENSG0000(	238.2163	1.646888	0.148032	11.1252	9.46E-29	1.73E-26	CHODL	protein_cc
ENSG0000(	238.058	0.907302	0.104124	8.7137	2.94E-18	7.64E-17	PPP1R1C	protein_cc
ENSG0000(	237.9371	0.280421	0.046673	6.008249	1.88E-09	1.17E-08	ZNF587B	protein_cc
ENSG0000(	237.7057	0.089816	0.118433	0.75837	0.448229	0.545016	C1orf194	protein_cc
ENSG0000(	237.6818	0.050298	0.076556	0.65702	0.511168	0.604355	C10orf11	protein_cc
ENSG0000(	237.6306	-0.13056	0.076924	-1.69727	0.089645	0.140547	RP11-958	pseudoge
ENSG0000(	237.2564	1.970964	0.183333	10.75072	5.88E-27	7.51E-25	CHRNA1	protein_cc
ENSG0000(	237.204	-0.18731	0.042716	-4.38508	1.16E-05	3.96E-05	AMACR	protein_cc
ENSG0000(	236.8634	0.247198	0.103736	2.38296	0.017174	0.032475	DENND2D	protein_cc
ENSG0000(	236.8063	0.037091	0.061099	0.607075	0.543801	0.634383	MPP3	protein_cc
ENSG0000(	236.7977	0.228143	0.103039	2.214141	0.026819	0.048516	ODF3B	protein_cc
ENSG0000(	236.7838	0.161702	0.065772	2.458528	0.013951	0.026973	RASSF7	protein_cc
ENSG0000(	236.5239	-0.41154	0.082029	-5.01702	5.25E-07	2.22E-06	AC005330	lincRNA
ENSG0000(	236.4552	0.004455	0.10008	0.04451	0.964498	0.975696	TRIM59	protein_cc
ENSG0000(	236.2936	0.12802	0.030663	4.175044	2.98E-05	9.51E-05	NUDT6	protein_cc
ENSG0000(	236.1918	0.005006	0.054873	0.091228	0.927311	0.948814	RP1-151F:	lincRNA
ENSG0000(	236.1288	0.173166	0.057788	2.996555	0.00273	0.006132	AC007405	lincRNA
ENSG0000(	236.0551	-0.05197	0.050002	-1.03934	0.298645	0.390906	ASPRV1	protein_cc
ENSG0000(	235.9483	0.637817	0.06778	9.410119	4.96E-21	2.01E-19	C16orf59	protein_cc
ENSG0000(	235.9252	0.156028	0.081675	1.910358	0.056087	0.093401	PTGES3P1	pseudoge
ENSG0000(	235.9097	0.035289	0.049952	0.706464	0.4799	0.575242	ZNF286B	protein_cc
ENSG0000(	235.8264	0.274676	0.076314	3.59927	0.000319	0.000855	TMEM255	protein_cc
ENSG0000(	235.7719	-0.02894	0.067469	-0.42887	0.66802	0.743767	RP3-368A	sense_intr
ENSG0000(	235.767	0.278757	0.094717	2.943044	0.00325	0.007193	ZIC5	protein_cc
ENSG0000(	235.6825	0.244858	0.054004	4.534074	5.79E-06	2.08E-05	MESP1	protein_cc
ENSG0000(	235.6777	-0.23062	0.051132	-4.51038	6.47E-06	2.30E-05	NDUFA6-/	processed
ENSG0000(	235.5203	-1.71815	0.177037	-9.70506	2.87E-22	1.46E-20	C1QL3	protein_cc
ENSG0000(	235.2946	0.173171	0.037263	4.647295	3.36E-06	1.25E-05	FAM86C2I	pseudoge
ENSG0000(	235.1765	0.002788	0.079556	0.035051	0.972039	0.980594	KCNQ1OT	antisense
ENSG0000(	235.1697	-0.05211	0.125881	-0.41397	0.678899	0.7529	FXYD1	protein_cc
ENSG0000(	235.0037	0.140115	0.037144	3.772241	0.000162	0.000456	ZNF782	protein_cc
ENSG0000(	234.9746	0.331398	0.054124	6.122897	9.19E-10	6.03E-09	RP11-488	lincRNA
ENSG0000(	234.9122	0.238363	0.079087	3.013946	0.002579	0.005828	HLA-L	pseudoge
ENSG0000(	234.7763	-0.20941	0.066568	-3.14587	0.001656	0.00388	ANKRD36	protein_cc
ENSG0000(	234.7702	0.303439	0.069046	4.394741	1.11E-05	3.80E-05	CA13	protein_cc
ENSG0000(	234.756	0.307039	0.04024	7.630199	2.34E-14	3.17E-13	ZSCAN16	protein_cc
ENSG0000(	234.6531	-0.4105	0.066246	-6.19657	5.77E-10	3.90E-09	CTC-203F	antisense
ENSG0000(	234.5914	-0.3943	0.099888	-3.94741	7.90E-05	0.000235	CYP2E1	protein_cc
ENSG0000(	234.5111	0.657546	0.184268	3.568433	0.000359	0.000953	DES	protein_cc
ENSG0000(	234.4171	-0.21058	0.050278	-4.18839	2.81E-05	9.01E-05	CCDC171	protein_cc
ENSG0000(	234.2763	0.649667	0.091675	7.086628	1.37E-12	1.40E-11	TLE6	protein_cc
ENSG0000(	234.2634	0.230856	0.041551	5.555973	2.76E-08	1.43E-07	CTD-2574	sense_intr
ENSG0000(	233.9435	0.131129	0.05673	2.31146	0.020807	0.038581	RP4-635E.	antisense
ENSG0000(	233.9335	0.382626	0.170843	2.239639	0.025114	0.045728	FCGR2B	protein_cc
ENSG0000(	233.5965	0.481362	0.106118	4.536121	5.73E-06	2.06E-05	EGFLAM	protein_cc
ENSG0000(	233.4678	0.006227	0.037589	0.165649	0.868433	0.905582	CD3EAP	protein_cc
ENSG0000(	232.5076	-0.22362	0.045577	-4.90636	9.28E-07	3.77E-06	RPH3AL	protein_cc
ENSG0000(	232.4529	0.749849	0.094444	7.939591	2.03E-15	3.33E-14	DNAJC22	protein_cc
ENSG0000(	232.3448	-0.60646	0.112349	-5.39798	6.74E-08	3.28E-07	AF131216	antisense
ENSG0000(	232.0938	-0.06111	0.048964	-1.24803	0.212022	0.293348	GPR135	protein_cc
ENSG0000(	232.0254	0.049777	0.09888	0.503407	0.614678	0.697903	PCDHGA6	protein_cc
ENSG0000(	232.0089	-0.64012	0.076111	-8.41037	4.09E-17	8.86E-16	ADRB2	protein_cc
ENSG0000(	232.0038	1.309873	0.145138	9.025013	1.80E-19	5.65E-18	TNFRSF11	protein_cc
ENSG0000(	231.8428	0.164307	0.029139	5.63869	1.71E-08	9.16E-08	FAM185A	protein_cc
ENSG0000(	231.7854	-0.34758	0.07475	-4.64993	3.32E-06	1.24E-05	KLHL11	protein_cc
ENSG0000(	231.7629	-0.06422	0.035445	-1.81194	0.069996	0.113314	AC074286	sense_ove
ENSG0000(	231.7368	0.298961	0.044735	6.682998	2.34E-11	1.96E-10	NEK8	protein_cc

ENSG0000(	231.7084	-0.07072	0.111242	-0.63577	0.524924	0.616824	SPEF2	protein_cc
ENSG0000(	231.5399	-0.04017	0.059497	-0.67512	0.499599	0.593493	RP11-274	pseudoge
ENSG0000(	231.4778	-0.70444	0.140572	-5.01123	5.41E-07	2.28E-06	PTPN3	protein_cc
ENSG0000(	231.4153	0.312565	0.049423	6.324307	2.54E-10	1.82E-09	POLR3G	protein_cc
ENSG0000(	231.3283	0.632496	0.087597	7.220542	5.18E-13	5.61E-12	RPL14P1	pseudoge
ENSG0000(	231.2094	-0.46739	0.090405	-5.17001	2.34E-07	1.05E-06	ADAMTS1	protein_cc
ENSG0000(	231.1398	-0.32222	0.084634	-3.80718	0.000141	0.0004	DSC2	protein_cc
ENSG0000(	231.0915	-0.1082	0.08384	-1.29059	0.196845	0.275492	SGMS2	protein_cc
ENSG0000(	231.0547	0.051526	0.03012	1.710715	0.087134	0.137128	PGBD2	protein_cc
ENSG0000(	231.0112	0.014255	0.063841	0.223292	0.823309	0.870603	EIF3FP3	pseudoge
ENSG0000(	230.8428	-0.73606	0.106109	-6.93683	4.01E-12	3.80E-11	PEBP4	protein_cc
ENSG0000(	230.8245	0.626301	0.104748	5.979112	2.24E-09	1.38E-08	TTC12	protein_cc
ENSG0000(	230.6641	0.10436	0.092913	1.123201	0.261352	0.349751	PCDP1	protein_cc
ENSG0000(	230.602	0.246915	0.051787	4.76794	1.86E-06	7.21E-06	RP11-617	antisense
ENSG0000(	230.5671	0.427474	0.086683	4.931448	8.16E-07	3.34E-06	AP000230	lincRNA
ENSG0000(	230.5228	0.213041	0.154562	1.378353	0.168094	0.241071	GPRC5A	protein_cc
ENSG0000(	229.9783	-0.15335	0.031577	-4.8564	1.20E-06	4.77E-06	GNRHR2	pseudoge
ENSG0000(	229.862	0.203473	0.035174	5.784845	7.26E-09	4.12E-08	ZNF222	protein_cc
ENSG0000(	229.7641	0.199812	0.039255	5.090069	3.58E-07	1.55E-06	ZNF684	protein_cc
ENSG0000(	229.7007	0.165366	0.055033	3.00485	0.002657	0.005988	ZNF781	protein_cc
ENSG0000(	229.6672	0.06095	0.048808	1.248773	0.211748	0.293068	KIF27	protein_cc
ENSG0000(	229.2955	0.09314	0.118452	0.786313	0.431684	0.528692	RP11-844	lincRNA
ENSG0000(	229.2523	-0.32567	0.090126	-3.61355	0.000302	0.000812	ADRB1	protein_cc
ENSG0000(	229.2429	-0.29659	0.158851	-1.86711	0.061886	0.101744	SLC13A5	protein_cc
ENSG0000(	229.236	-0.0782	0.06081	-1.28604	0.19843	0.277332	RP11-119	sense_ove
ENSG0000(	229.0302	-0.83424	0.121842	-6.84688	7.55E-12	6.84E-11	ACVR1C	protein_cc
ENSG0000(	228.8866	-0.18402	0.081336	-2.26248	0.023668	0.043336	HIC1	protein_cc
ENSG0000(	228.8055	-0.05938	0.042495	-1.39741	0.162291	0.233865	AC004893	antisense
ENSG0000(	228.6818	-0.00456	0.055621	-0.08202	0.934633	0.954407	RP11-262	lincRNA
ENSG0000(	228.5876	0.863347	0.114341	7.550644	4.33E-14	5.60E-13	RMST	processed
ENSG0000(	228.46	-0.56767	0.113858	-4.98579	6.17E-07	2.58E-06	NIPAL4	protein_cc
ENSG0000(	228.407	0.010155	0.041919	0.242253	0.808584	0.859012	C21orf67	protein_cc
ENSG0000(	228.3808	-0.17583	0.085232	-2.06301	0.039112	0.068007	ATP2A3	protein_cc
ENSG0000(	228.3344	-0.60308	0.137236	-4.39444	1.11E-05	3.81E-05	CPAMD8	protein_cc
ENSG0000(	228.3164	-0.2964	0.043904	-6.75115	1.47E-11	1.27E-10	RSG1	protein_cc
ENSG0000(	228.3108	0.163544	0.05727	2.855645	0.004295	0.009279	RP11-452	lincRNA
ENSG0000(	228.294	0.298971	0.052162	5.731607	9.95E-09	5.52E-08	DZIP1L	protein_cc
ENSG0000(	228.1718	0.231556	0.045108	5.133408	2.85E-07	1.25E-06	CXorf24	protein_cc
ENSG0000(	227.8088	-0.21925	0.111929	-1.95883	0.050132	0.084698	RP11-259	lincRNA
ENSG0000(	227.766	0.310766	0.040923	7.593984	3.10E-14	4.11E-13	SMN1	protein_cc
ENSG0000(	227.6976	0.14606	0.032914	4.437636	9.10E-06	3.16E-05	GET4	protein_cc
ENSG0000(	227.6157	1.011498	0.138862	7.284204	3.24E-13	3.62E-12	ADAMTS3	protein_cc
ENSG0000(	227.5229	0.266791	0.046731	5.709137	1.14E-08	6.25E-08	SHPK	protein_cc
ENSG0000(	227.4335	-0.80667	0.097819	-8.24656	1.63E-16	3.20E-15	RP11-441	antisense
ENSG0000(	227.3238	0.350788	0.046314	7.574047	3.62E-14	4.74E-13	ZNF701	protein_cc
ENSG0000(	227.2491	-0.22298	0.139153	-1.60238	0.109072	0.166481	ZDHHC8P	pseudoge
ENSG0000(	227.1384	-0.45788	0.086306	-5.30525	1.13E-07	5.29E-07	RERGL	protein_cc
ENSG0000(	227.1129	0.95223	0.146135	6.516077	7.22E-11	5.62E-10	PCDHGA1	protein_cc
ENSG0000(	226.958	0.245842	0.037129	6.621243	3.56E-11	2.91E-10	PITPNA-A	antisense
ENSG0000(	226.7128	0.571778	0.082794	6.906009	4.98E-12	4.64E-11	RPL13AP2	pseudoge
ENSG0000(	226.5014	-0.17899	0.060206	-2.97292	0.00295	0.006585	LINC0086:	lincRNA
ENSG0000(	226.4469	0.1742	0.145399	1.198083	0.230885	0.31493	RNASE2	protein_cc
ENSG0000(	226.3937	0.118241	0.038567	3.065847	0.002171	0.004974	AC133528	antisense
ENSG0000(	226.2275	-0.0595	0.060678	-0.98058	0.3268	0.421044	EIF4EBP3	protein_cc
ENSG0000(	226.1956	-0.21048	0.063518	-3.31368	0.000921	0.002258	XXbac-BP	antisense
ENSG0000(	226.1115	-0.14725	0.121581	-1.2111	0.225857	0.309282	SLC16A10	protein_cc
ENSG0000(	225.9626	-0.06907	0.048296	-1.43014	0.152676	0.222197	DPY19L2P	pseudoge
ENSG0000(	225.9049	-0.11779	0.087681	-1.34342	0.179136	0.254249	TNFRSF10	protein_cc
ENSG0000(	225.8752	-0.05257	0.088254	-0.59562	0.551428	0.641262	ZSCAN1	protein_cc

ENSG0000(	225.5019	-0.33916	0.080607	-4.2075	2.58E-05	8.33E-05	TLR6	protein_cc
ENSG0000(	225.3964	0.417824	0.079923	5.227803	1.72E-07	7.83E-07	DIAPH3	protein_cc
ENSG0000(	225.2802	-0.25251	0.059323	-4.25644	2.08E-05	6.82E-05	ARG2	protein_cc
ENSG0000(	225.2631	0.131581	0.12091	1.08825	0.276485	0.366637	SMIM18	protein_cc
ENSG0000(	225.1442	0.015239	0.07559	0.201601	0.840229	0.883858	AGAP10	protein_cc
ENSG0000(	225.0371	-0.85222	0.088709	-9.60696	7.47E-22	3.50E-20	PCDHAC2	protein_cc
ENSG0000(	224.7388	-0.30522	0.066123	-4.61593	3.91E-06	1.44E-05	TMEM232	protein_cc
ENSG0000(	224.6971	0.139947	0.083206	1.681932	0.092582	0.144497	FOXF1	protein_cc
ENSG0000(	224.688	0.128573	0.042578	3.019707	0.00253	0.005731	LINC0101:	lincRNA
ENSG0000(	224.683	-0.215	0.089001	-2.41564	0.015707	0.030002	CASZ1	protein_cc
ENSG0000(	224.5438	-1.05904	0.120808	-8.76633	1.85E-18	4.93E-17	GRM2	protein_cc
ENSG0000(	224.5331	0.199536	0.066013	3.022696	0.002505	0.005679	FAM71F2	protein_cc
ENSG0000(	224.4862	-0.62264	0.107969	-5.76682	8.08E-09	4.55E-08	CES4A	protein_cc
ENSG0000(	224.4074	-0.38152	0.093956	-4.06063	4.89E-05	0.000151	ANKRD29	protein_cc
ENSG0000(	224.2586	-0.68347	0.132126	-5.17291	2.30E-07	1.03E-06	CPXM2	protein_cc
ENSG0000(	224.1679	-0.07743	0.084139	-0.92028	0.357427	0.453163	ALS2CL	protein_cc
ENSG0000(	224.0279	-0.03306	0.034122	-0.96897	0.332562	0.427154	ZNF565	protein_cc
ENSG0000(	223.99	-0.35187	0.09888	-3.55857	0.000373	0.000986	BNC2	protein_cc
ENSG0000(	223.8414	0.041379	0.100436	0.411992	0.680346	0.753976	SYNC	protein_cc
ENSG0000(	223.7318	-0.61481	0.080884	-7.60112	2.94E-14	3.90E-13	HENMT1	protein_cc
ENSG0000(	223.7129	0.143037	0.067929	2.105699	0.035231	0.062006	RCN1P2	pseudoge
ENSG0000(	223.6829	0.096084	0.071837	1.337521	0.181053	0.256506	C4orf19	protein_cc
ENSG0000(	223.6212	0.235624	0.043221	5.451615	4.99E-08	2.48E-07	UCHL3	protein_cc
ENSG0000(	223.5642	-0.17618	0.0465	-3.78877	0.000151	0.000429	ZNF169	protein_cc
ENSG0000(	223.534	0.081203	0.067797	1.197727	0.231023	0.315048	CAHM	lincRNA
ENSG0000(	223.3614	-0.08486	0.049276	-1.72217	0.085038	0.134189	RP1-228H	sense_ove
ENSG0000(	223.2155	0.010239	0.080789	0.126735	0.89915	0.928455	PCED1B	protein_cc
ENSG0000(	223.2125	-0.08335	0.041567	-2.00511	0.044951	0.076877	RP11-582.	antisense
ENSG0000(	223.0044	0.128661	0.06015	2.138997	0.032436	0.057543	MYLK2	protein_cc
ENSG0000(	222.7619	0.37268	0.103125	3.613867	0.000302	0.000811	CD274	protein_cc
ENSG0000(	222.4071	0.194093	0.039692	4.889986	1.01E-06	4.07E-06	INO80B	protein_cc
ENSG0000(	222.2773	-0.05127	0.061474	-0.83407	0.404239	0.501128	MAP4K1	protein_cc
ENSG0000(	222.2473	0.41842	0.144157	2.902533	0.003702	0.0081	FAM110C	protein_cc
ENSG0000(	221.9436	-0.07726	0.069589	-1.11018	0.266922	0.355919	RP11-449	antisense
ENSG0000(	221.9299	0.814791	0.294759	2.764263	0.005705	0.012009	SAA1	protein_cc
ENSG0000(	221.8229	-0.3182	0.076921	-4.13669	3.52E-05	0.000111	RASGRP4	protein_cc
ENSG0000(	221.7956	1.085212	0.117567	9.230578	2.69E-20	9.57E-19	EXO1	protein_cc
ENSG0000(	221.6674	-0.4656	0.120607	-3.86043	0.000113	0.000327	LRRC10B	protein_cc
ENSG0000(	221.5714	-0.10684	0.07783	-1.37276	0.169828	0.243243	CPB2-AS1	antisense
ENSG0000(	221.4065	-0.05691	0.042425	-1.34137	0.179801	0.255029	KB-1507C	protein_cc
ENSG0000(	221.4056	0.136552	0.054327	2.513509	0.011954	0.023459	MTX1P1	pseudoge
ENSG0000(	221.3924	-0.03565	0.052278	-0.68193	0.495282	0.589544	RBM26-A!	antisense
ENSG0000(	221.258	0.541518	0.157844	3.430724	0.000602	0.00153	TEKT1	protein_cc
ENSG0000(	221.2221	0.442108	0.089619	4.933205	8.09E-07	3.31E-06	CTF1	protein_cc
ENSG0000(	221.0903	-0.10136	0.08106	-1.2504	0.211154	0.292373	HFM1	protein_cc
ENSG0000(	220.9963	0.896351	0.114082	7.85707	3.93E-15	6.16E-14	RPL7AP6	pseudoge
ENSG0000(	220.9378	-0.34042	0.078158	-4.35553	1.33E-05	4.49E-05	TRPV3	protein_cc
ENSG0000(	220.5112	-0.67478	0.082092	-8.21982	2.04E-16	3.93E-15	SLC2A12	protein_cc
ENSG0000(	220.4579	-0.04825	0.042163	-1.14448	0.252423	0.339715	PHF7	protein_cc
ENSG0000(	220.4485	-0.13113	0.068411	-1.91673	0.055272	0.092248	SLC26A1	protein_cc
ENSG0000(	220.3797	-0.33195	0.064621	-5.13692	2.79E-07	1.23E-06	RP11-305	sense_ove
ENSG0000(	220.2955	-0.0416	0.048744	-0.85346	0.393406	0.489989	CCT6B	protein_cc
ENSG0000(	220.2805	-0.21706	0.090035	-2.41079	0.015918	0.030371	ATP13A5	protein_cc
ENSG0000(	220.1889	-0.15985	0.049797	-3.20998	0.001327	0.003164	C8orf31	protein_cc
ENSG0000(	220.1011	0.445487	0.064867	6.867708	6.52E-12	5.96E-11	HNRNPA1	pseudoge
ENSG0000(	220.0125	-0.41213	0.082097	-5.01999	5.17E-07	2.19E-06	TFAP2E	protein_cc
ENSG0000(	219.9671	-0.73328	0.109005	-6.72703	1.73E-11	1.48E-10	FAM131C	protein_cc
ENSG0000(	219.8626	0.541651	0.096997	5.584215	2.35E-08	1.23E-07	RIBC1	protein_cc
ENSG0000(	219.7527	0.02956	0.028416	1.040257	0.29822	0.390511	TCEANC	protein_cc

ENSG0000(	219.7166	-0.18229	0.065296	-2.79174	0.005243	0.01113	LURAP1L	protein_cc
ENSG0000(	219.527	0.4633	0.047853	9.681668	3.61E-22	1.80E-20	CTD-2201	sense_ove
ENSG0000(	219.459	-0.48964	0.135132	-3.62341	0.000291	0.000784	C1QL2	protein_cc
ENSG0000(	219.4123	0.229362	0.044106	5.200229	1.99E-07	8.99E-07	SARS2	protein_cc
ENSG0000(	219.3009	0.182569	0.117224	1.557434	0.119368	0.179929	LINC0009?	lincRNA
ENSG0000(	219.2642	0.191206	0.083665	2.285389	0.02229	0.041078	CASC10	protein_cc
ENSG0000(	219.1624	-0.22016	0.060966	-3.61124	0.000305	0.000819	RASL11A	protein_cc
ENSG0000(	219.0058	0.083706	0.061093	1.370154	0.170639	0.244107	LRRC37A2	protein_cc
ENSG0000(	218.9043	0.005456	0.093511	0.058347	0.953473	0.967876	IFNLR1	protein_cc
ENSG0000(	218.8399	0.550934	0.082508	6.677343	2.43E-11	2.04E-10	EEF1A1P1!	pseudoge
ENSG0000(	218.7906	0.263257	0.05056	5.206874	1.92E-07	8.70E-07	PEX11G	protein_cc
ENSG0000(	218.7853	0.302286	0.068926	4.38565	1.16E-05	3.95E-05	AC087393	lincRNA
ENSG0000(	218.6864	0.050946	0.032775	1.554404	0.120088	0.180759	RP5-821D	antisense
ENSG0000(	218.5914	1.032797	0.089614	11.52493	9.88E-31	2.71E-28	C11orf82	protein_cc
ENSG0000(	218.5821	-0.85463	0.12108	-7.05839	1.68E-12	1.69E-11	FAM196B	protein_cc
ENSG0000(	218.2896	0.07447	0.048291	1.542102	0.123049	0.184594	CYB5RL	protein_cc
ENSG0000(	218.1291	-0.11413	0.062562	-1.82424	0.068116	0.110619	USHBP1	protein_cc
ENSG0000(	218.0344	0.295533	0.064721	4.566273	4.96E-06	1.80E-05	CCNE1	protein_cc
ENSG0000(	217.989	-1.26029	0.120656	-10.4454	1.54E-25	1.49E-23	LHX6	protein_cc
ENSG0000(	217.8482	-0.38495	0.093887	-4.10018	4.13E-05	0.000129	LAMA3	protein_cc
ENSG0000(	217.8446	0.894088	0.120821	7.400109	1.36E-13	1.62E-12	CEP55	protein_cc
ENSG0000(	217.6115	0.114828	0.118234	0.97119	0.331454	0.425946	HYDIN	protein_cc
ENSG0000(	217.424	-0.34682	0.115296	-3.00813	0.002629	0.005929	ITIH2	protein_cc
ENSG0000(	217.2359	-0.0252	0.069363	-0.36333	0.716356	0.784	RP11-305	antisense
ENSG0000(	217.0417	-0.21398	0.190108	-1.12555	0.260355	0.348643	TLX1	protein_cc
ENSG0000(	216.9489	-0.65265	0.144857	-4.50545	6.62E-06	2.35E-05	SLC22A6	protein_cc
ENSG0000(	216.8146	0.072508	0.102	0.71086	0.477171	0.572763	ONECUT2	protein_cc
ENSG0000(	216.6978	-0.15724	0.064494	-2.43807	0.014766	0.028397	EAF2	protein_cc
ENSG0000(	216.6637	-0.15656	0.092798	-1.68716	0.091572	0.143116	ARHGEF28	protein_cc
ENSG0000(	216.4987	-1.15714	0.151553	-7.63524	2.25E-14	3.06E-13	LINC0061	lincRNA
ENSG0000(	216.4935	-0.51689	0.151739	-3.40647	0.000658	0.00166	PCDHA4	protein_cc
ENSG0000(	216.4635	0.285798	0.057607	4.961137	7.01E-07	2.90E-06	DNA2	protein_cc
ENSG0000(	216.4547	0.863176	0.152725	5.651827	1.59E-08	8.54E-08	ARSF	protein_cc
ENSG0000(	216.4455	-0.64188	0.113979	-5.63162	1.79E-08	9.53E-08	JPH1	protein_cc
ENSG0000(	216.3067	1.476933	0.11713	12.60935	1.88E-36	1.61E-33	SOCS2-AS	processed
ENSG0000(	216.2868	-0.15345	0.070216	-2.18545	0.028856	0.051822	RP11-259	lincRNA
ENSG0000(	216.2017	-0.21511	0.08124	-2.6478	0.008102	0.016531	PRKAG2-A	antisense
ENSG0000(	216.119	0.396062	0.064108	6.178043	6.49E-10	4.36E-09	CEP152	protein_cc
ENSG0000(	216.0688	1.086556	0.117685	9.232774	2.64E-20	9.41E-19	ESCO2	protein_cc
ENSG0000(	216.0456	0.713973	0.092368	7.729637	1.08E-14	1.56E-13	CTD-2192	pseudoge
ENSG0000(	215.8293	0.236576	0.139881	1.691269	0.090785	0.142043	CLCF1	protein_cc
ENSG0000(	215.7911	0.053777	0.034619	1.553381	0.120332	0.181093	UBE3D	protein_cc
ENSG0000(	215.7204	-0.15462	0.077805	-1.98726	0.046893	0.079803	MID2	protein_cc
ENSG0000(	215.5345	0.471008	0.078027	6.036452	1.58E-09	9.94E-09	CRYGS	protein_cc
ENSG0000(	215.5039	-0.29833	0.075037	-3.9758	7.01E-05	0.000211	RP11-9G1	lincRNA
ENSG0000(	215.463	0.61447	0.128163	4.794424	1.63E-06	6.38E-06	NAMPTL	protein_cc
ENSG0000(	215.3846	-0.51988	0.142119	-3.65806	0.000254	0.000692	RP11-47I2	antisense
ENSG0000(	215.3719	-0.4482	0.073984	-6.05806	1.38E-09	8.79E-09	SLC30A10	protein_cc
ENSG0000(	215.3544	0.087114	0.121506	0.716951	0.473404	0.569215	C2orf40	protein_cc
ENSG0000(	215.2979	-0.42492	0.085497	-4.96994	6.70E-07	2.78E-06	LRRC73	protein_cc
ENSG0000(	215.1793	-0.41532	0.088598	-4.68772	2.76E-06	1.04E-05	TNFRSF11	protein_cc
ENSG0000(	215.0036	0.011783	0.172998	0.068109	0.945698	0.96217	CYP4F24P	pseudoge
ENSG0000(	214.9991	0.200356	0.097677	2.051205	0.040247	0.069774	PTHLH	protein_cc
ENSG0000(	214.9622	0.412882	0.097133	4.250676	2.13E-05	6.98E-05	NPR1	protein_cc
ENSG0000(	214.9498	0.250244	0.049224	5.083826	3.70E-07	1.60E-06	RP11-705	pseudoge
ENSG0000(	214.843	0.040027	0.063878	0.626616	0.530911	0.62256	PDXDC2P	processed
ENSG0000(	214.779	-0.07623	0.052549	-1.45072	0.146859	0.214811	INTS6-AS	antisense
ENSG0000(	214.6343	-0.3679	0.063906	-5.75688	8.57E-09	4.80E-08	REL	protein_cc
ENSG0000(	214.5615	-0.03002	0.082898	-0.36217	0.717227	0.784573	TTC40	protein_cc

ENSG0000(	214.4968	-0.03909	0.049384	-0.79158	0.428606	0.525678	SUGCT	protein_cc
ENSG0000(	214.2248	1.360091	0.128848	10.55578	4.78E-26	5.10E-24	E2F7	protein_cc
ENSG0000(	214.2052	0.267442	0.091937	2.908974	0.003626	0.007951	RUNX3	protein_cc
ENSG0000(	214.1423	0.738766	0.080506	9.176514	4.45E-20	1.54E-18	EME1	protein_cc
ENSG0000(	214.0749	-0.01423	0.040011	-0.35571	0.722061	0.78854	RP4-769N	processed
ENSG0000(	214.0723	0.14844	0.0485	3.060625	0.002209	0.005054	RP11-498	lincRNA
ENSG0000(	214.0183	0.007503	0.072176	0.103954	0.917206	0.941708	AL592494	lincRNA
ENSG0000(	214.0167	-0.21111	0.060971	-3.4625	0.000535	0.001373	CTB-36H1	sense_ove
ENSG0000(	213.9875	0.617483	0.083893	7.3604	1.83E-13	2.13E-12	RPL24P4	pseudoge
ENSG0000(	213.9421	-0.85599	0.115627	-7.40307	1.33E-13	1.58E-12	CRHBP	protein_cc
ENSG0000(	213.7942	-0.6328	0.079904	-7.91952	2.38E-15	3.86E-14	GSTO2	protein_cc
ENSG0000(	213.79	-1.29868	0.133695	-9.71379	2.63E-22	1.35E-20	PTPRR	protein_cc
ENSG0000(	213.7407	-0.64187	0.118585	-5.41275	6.21E-08	3.04E-07	CKMT1B	protein_cc
ENSG0000(	213.2934	-0.07863	0.058432	-1.34562	0.178424	0.25348	hsa-mir-6	processed
ENSG0000(	213.2889	-0.2761	0.066025	-4.18179	2.89E-05	9.25E-05	TMEM74B	protein_cc
ENSG0000(	213.2354	-0.39622	0.109638	-3.61385	0.000302	0.000811	AC002429	antisense
ENSG0000(	213.1946	-0.02819	0.066555	-0.42359	0.671865	0.746812	MLKL	protein_cc
ENSG0000(	212.9923	0.479033	0.088543	5.410172	6.30E-08	3.08E-07	MMP25	protein_cc
ENSG0000(	212.8922	0.092493	0.039923	2.316809	0.020514	0.038092	AC009133	antisense
ENSG0000(	212.8763	-0.22963	0.02965	-7.74474	9.58E-15	1.40E-13	RP11-102	antisense
ENSG0000(	212.8577	0.142917	0.058797	2.430674	0.015071	0.028905	TMEM102	protein_cc
ENSG0000(	212.7358	0.066009	0.074637	0.8844	0.376481	0.472932	GLIPR1L2	protein_cc
ENSG0000(	212.6737	-0.15418	0.053724	-2.86977	0.004108	0.008915	RP11-819	lincRNA
ENSG0000(	212.6556	0.165846	0.077139	2.149959	0.031558	0.056131	CCDC65	protein_cc
ENSG0000(	212.4733	-1.66475	0.161123	-10.3322	5.04E-25	4.46E-23	TRHDE	protein_cc
ENSG0000(	212.4191	-0.04658	0.046748	-0.99641	0.319051	0.412782	ZNF572	protein_cc
ENSG0000(	212.3632	-0.46144	0.065165	-7.08112	1.43E-12	1.45E-11	PIGCP1	pseudoge
ENSG0000(	212.3319	0.622502	0.115347	5.396769	6.79E-08	3.30E-07	RP4-665J	lincRNA
ENSG0000(	212.2204	0.367881	0.043719	8.414747	3.94E-17	8.55E-16	RP5-991G	antisense
ENSG0000(	212.0926	-0.10636	0.062633	-1.69806	0.089496	0.140356	XXbac-BP	lincRNA
ENSG0000(	211.9765	0.050472	0.050498	0.999491	0.317557	0.411262	KBTBD8	protein_cc
ENSG0000(	211.9258	-0.16702	0.05875	-2.84296	0.00447	0.00962	CATSPER2	protein_cc
ENSG0000(	211.7739	0.094474	0.123725	0.763579	0.445118	0.541938	CCL5	protein_cc
ENSG0000(	211.7267	0.363924	0.051075	7.125292	1.04E-12	1.08E-11	RAB11B-A	antisense
ENSG0000(	211.7168	-0.22368	0.058699	-3.81054	0.000139	0.000395	MCMDC2	protein_cc
ENSG0000(	211.6951	-0.00492	0.078124	-0.06293	0.949825	0.965341	VRK2	protein_cc
ENSG0000(	211.627	-0.31307	0.059851	-5.23075	1.69E-07	7.72E-07	LINC0088	lincRNA
ENSG0000(	211.4191	0.849754	0.093616	9.077016	1.12E-19	3.62E-18	PIF1	protein_cc
ENSG0000(	211.325	0.939077	0.153097	6.13388	8.58E-10	5.65E-09	GPX1P1	pseudoge
ENSG0000(	211.2978	-0.8078	0.085415	-9.45739	3.16E-21	1.33E-19	PDZD7	protein_cc
ENSG0000(	211.284	-1.01246	0.104399	-9.698	3.07E-22	1.56E-20	SLC2A4	protein_cc
ENSG0000(	211.1462	0.111703	0.072821	1.533937	0.125045	0.187192	SCML2	protein_cc
ENSG0000(	211.1042	-0.08076	0.15415	-0.52392	0.600332	0.685132	HLA-DQB	protein_cc
ENSG0000(	210.9526	-0.30303	0.083728	-3.61926	0.000295	0.000796	PMAIP1	protein_cc
ENSG0000(	210.8229	-0.03017	0.142657	-0.2115	0.832493	0.87781	AC053503	antisense
ENSG0000(	210.7471	0.850307	0.083537	10.1788	2.47E-24	1.88E-22	XRCC2	protein_cc
ENSG0000(	210.4487	0.14405	0.053311	2.70208	0.006891	0.014259	MSTO2P	pseudoge
ENSG0000(	210.1817	-0.21568	0.106703	-2.02128	0.043251	0.074318	LILRB2	protein_cc
ENSG0000(	209.9663	0.17929	0.041561	4.313858	1.60E-05	5.36E-05	CTD-2528	lincRNA
ENSG0000(	209.8283	-0.0714	0.072594	-0.98358	0.325324	0.419498	GPR133	protein_cc
ENSG0000(	209.7266	-0.40303	0.142552	-2.82726	0.004695	0.010053	CDH19	protein_cc
ENSG0000(	209.6372	0.173767	0.038834	4.474576	7.66E-06	2.70E-05	ZNF823	protein_cc
ENSG0000(	209.5344	-0.00735	0.062653	-0.11739	0.906554	0.933826	RP11-384	lincRNA
ENSG0000(	209.1786	0.080436	0.089795	0.895778	0.370371	0.466741	BEAN1	protein_cc
ENSG0000(	209.0117	0.156713	0.068155	2.299345	0.021485	0.03972	FAM229A	protein_cc
ENSG0000(	208.9937	0.361819	0.077902	4.644516	3.41E-06	1.27E-05	MSH5	protein_cc
ENSG0000(	208.6351	-0.00043	0.121602	-0.00351	0.997201	0.998269	MYO1G	protein_cc
ENSG0000(	208.5653	1.25962	0.145263	8.671304	4.27E-18	1.08E-16	DPP4	protein_cc
ENSG0000(	208.5439	-0.01997	0.041824	-0.4776	0.632936	0.714022	RP11-242	lincRNA

ENSG0000(	208.4795	-0.16717	0.092317	-1.81082	0.070169	0.113554	FCGR1B	protein_cc
ENSG0000(	208.3018	0.1536	0.045865	3.348991	0.000811	0.00201	AC074117	antisense
ENSG0000(	208.1763	0.083117	0.04101	2.026772	0.042686	0.073485	RP11-15H	processed
ENSG0000(	208.1236	0.271112	0.050608	5.357112	8.46E-08	4.05E-07	CTC-444N	lincRNA
ENSG0000(	208.12	0.249523	0.097857	2.549886	0.010776	0.021375	NAALAD2	protein_cc
ENSG0000(	207.8974	-0.77429	0.100486	-7.70549	1.30E-14	1.85E-13	CRHR1	protein_cc
ENSG0000(	207.8547	-0.49762	0.054172	-9.18597	4.08E-20	1.42E-18	RP11-439	antisense
ENSG0000(	207.7191	0.360226	0.038674	9.31431	1.23E-20	4.65E-19	GPR89A	protein_cc
ENSG0000(	207.6067	0.158853	0.032641	4.866753	1.13E-06	4.55E-06	FDXACB1	protein_cc
ENSG0000(	207.526	0.238146	0.039585	6.01607	1.79E-09	1.12E-08	CTD-2619	lincRNA
ENSG0000(	207.3781	0.243744	0.046045	5.2936	1.20E-07	5.62E-07	ZNF665	protein_cc
ENSG0000(	207.3231	0.592142	0.136058	4.352129	1.35E-05	4.56E-05	HTRA3	protein_cc
ENSG0000(	207.3229	-0.22421	0.066383	-3.37749	0.000732	0.001828	PKMP3	pseudoge
ENSG0000(	207.2226	0.110232	0.136085	0.810023	0.417927	0.514838	KDELR3	protein_cc
ENSG0000(	207.0635	0.006973	0.076825	0.090766	0.927679	0.94912	CYSLTR2	protein_cc
ENSG0000(	207.0001	1.027912	0.169804	6.053504	1.42E-09	9.02E-09	G0S2	protein_cc
ENSG0000(	206.92	0.136656	0.041614	3.283886	0.001024	0.00249	HEXIM2	protein_cc
ENSG0000(	206.9066	-0.28895	0.162355	-1.77973	0.07512	0.12049	C10orf105	protein_cc
ENSG0000(	206.9002	0.031235	0.069021	0.452545	0.650876	0.729455	CCDC96	protein_cc
ENSG0000(	206.7765	0.391255	0.134318	2.912909	0.003581	0.007861	LILRA4	protein_cc
ENSG0000(	206.6552	0.592647	0.113857	5.205196	1.94E-07	8.77E-07	GCK	protein_cc
ENSG0000(	206.4885	-0.06976	0.034006	-2.05148	0.04022	0.069734	C14orf28	protein_cc
ENSG0000(	206.4602	0.611318	0.09531	6.413982	1.42E-10	1.06E-09	MIR210HC	lincRNA
ENSG0000(	206.4325	0.068617	0.068111	1.00743	0.313728	0.407123	AP001469	antisense
ENSG0000(	206.4029	0.123578	0.047013	2.628568	0.008575	0.017393	ZNF124	protein_cc
ENSG0000(	206.3288	-0.19663	0.09443	-2.08226	0.037319	0.065253	RIPPLY2	protein_cc
ENSG0000(	206.3019	0.481455	0.119791	4.019133	5.84E-05	0.000178	ARSI	protein_cc
ENSG0000(	206.2956	0.093606	0.053775	1.740713	0.081734	0.129557	HYAL3	protein_cc
ENSG0000(	206.2584	-0.25198	0.183464	-1.37346	0.16961	0.242973	SLC7A10	protein_cc
ENSG0000(	206.2519	-0.36907	0.176702	-2.08869	0.036736	0.064346	PNOC	protein_cc
ENSG0000(	206.1816	0.429267	0.076388	5.61955	1.91E-08	1.02E-07	DLGAP1-/	antisense
ENSG0000(	206.1025	-0.25195	0.053923	-4.67234	2.98E-06	1.12E-05	RP11-359	antisense
ENSG0000(	206.0908	1.213685	0.126133	9.622259	6.44E-22	3.04E-20	RPS3AP26	pseudoge
ENSG0000(	205.6297	-0.04443	0.038211	-1.16276	0.244928	0.331122	ANKHD1	protein_cc
ENSG0000(	205.6203	-0.21062	0.040354	-5.21942	1.79E-07	8.16E-07	LINC0067	pseudoge
ENSG0000(	205.5977	0.234066	0.080466	2.908874	0.003627	0.007953	SIX5	protein_cc
ENSG0000(	205.5449	0.751602	0.084329	8.912688	4.98E-19	1.45E-17	BRCA2	protein_cc
ENSG0000(	205.5104	-0.07566	0.065644	-1.15264	0.249058	0.3359	TLCD2	protein_cc
ENSG0000(	205.4862	-0.4225	0.063056	-6.70047	2.08E-11	1.75E-10	EDA	protein_cc
ENSG0000(	205.2524	-0.1647	0.053746	-3.06438	0.002181	0.004995	AC093415	processed
ENSG0000(	205.2243	0.345166	0.05462	6.319453	2.62E-10	1.87E-09	EXOC3L1	protein_cc
ENSG0000(	205.2018	0.279179	0.072903	3.829453	0.000128	0.000368	RBPMS2	protein_cc
ENSG0000(	204.9432	0.775944	0.126402	6.138714	8.32E-10	5.49E-09	EFHC2	protein_cc
ENSG0000(	204.8273	0.526479	0.074833	7.035405	1.99E-12	1.98E-11	GINS4	protein_cc
ENSG0000(	204.7011	-0.06907	0.036673	-1.88332	0.059657	0.098583	BBS1	protein_cc
ENSG0000(	204.6425	-0.05275	0.055346	-0.9531	0.340541	0.435661	RP5-1198	lincRNA
ENSG0000(	204.5416	0.357858	0.037674	9.498762	2.12E-21	9.28E-20	ZNF443	protein_cc
ENSG0000(	204.331	-0.38764	0.111261	-3.48404	0.000494	0.001275	SERTAD4	protein_cc
ENSG0000(	204.3046	1.207962	0.141207	8.55453	1.18E-17	2.80E-16	DEPDC1	protein_cc
ENSG0000(	204.2902	-0.26948	0.106069	-2.54066	0.011064	0.021888	MYOT	protein_cc
ENSG0000(	204.1796	-0.11332	0.059985	-1.88911	0.058877	0.09745	PRKAR2A-	antisense
ENSG0000(	204.1639	0.021664	0.043465	0.498419	0.618189	0.701018	EIF3EP1	pseudoge
ENSG0000(	204.1225	-0.00896	0.050337	-0.17803	0.858701	0.897961	1-Sep	protein_cc
ENSG0000(	204.0854	-0.27292	0.103591	-2.63455	0.008425	0.017124	GCNT1	protein_cc
ENSG0000(	204.0782	0.249625	0.057761	4.32166	1.55E-05	5.18E-05	LINC0112!	antisense
ENSG0000(	203.9702	0.781155	0.097888	7.980107	1.46E-15	2.46E-14	TMEM154	protein_cc
ENSG0000(	203.9368	0.079251	0.192747	0.411168	0.680949	0.75434	IGHA1	IG_C_gene
ENSG0000(	203.9015	-0.25714	0.077228	-3.32956	0.00087	0.002144	RP11-334	antisense
ENSG0000(	203.8781	-0.18764	0.100335	-1.87018	0.061459	0.101121	RRAD	protein_cc

ENSG0000(	203.7095	0.478721	0.118814	4.029159	5.60E-05	0.000171	PCDHB8	protein_cc
ENSG0000(	203.5186	-0.03941	0.099952	-0.39428	0.693372	0.765113	DNAH5	protein_cc
ENSG0000(	203.5011	0.190372	0.097558	1.951361	0.051014	0.086015	SARDH	protein_cc
ENSG0000(	203.2753	0.022269	0.081092	0.274621	0.783607	0.838905	FUOM	protein_cc
ENSG0000(	203.1952	-0.43466	0.13504	-3.21874	0.001288	0.003076	FGF17	protein_cc
ENSG0000(	203.1117	0.227283	0.051288	4.431506	9.36E-06	3.25E-05	TMEM81	protein_cc
ENSG0000(	203.0934	-0.13767	0.071318	-1.93034	0.053564	0.089752	NUDT7	protein_cc
ENSG0000(	203.0863	-0.80688	0.129188	-6.2458	4.22E-10	2.91E-09	ZNF385B	protein_cc
ENSG0000(	202.8219	0.727965	0.092348	7.88284	3.20E-15	5.09E-14	LXN	protein_cc
ENSG0000(	202.7314	1.390637	0.171097	8.127774	4.37E-16	8.03E-15	DPEP1	protein_cc
ENSG0000(	202.6321	0.307618	0.098057	3.137149	0.001706	0.003988	MYO7B	protein_cc
ENSG0000(	202.5247	0.262498	0.049836	5.26718	1.39E-07	6.41E-07	RP11-629	antisense
ENSG0000(	202.4416	0.505521	0.08251	6.126759	8.97E-10	5.89E-09	ARSE	protein_cc
ENSG0000(	202.4372	0.126897	0.055696	2.278386	0.022704	0.04176	SLC25A10	protein_cc
ENSG0000(	202.4037	0.246819	0.071695	3.442641	0.000576	0.00147	RP5-1074	antisense
ENSG0000(	202.3894	-0.55941	0.086854	-6.44085	1.19E-10	8.96E-10	N4BP3	protein_cc
ENSG0000(	202.2471	0.06159	0.043163	1.426922	0.153602	0.223317	RMDN2	protein_cc
ENSG0000(	202.2166	0.344809	0.034391	10.02613	1.17E-23	7.95E-22	MRPL53	protein_cc
ENSG0000(	202.1759	-0.10966	0.119292	-0.91924	0.357972	0.453757	CCR5	protein_cc
ENSG0000(	201.9941	0.005346	0.110587	0.048342	0.961444	0.973589	KBTBD11-	lincRNA
ENSG0000(	201.9525	0.081081	0.056352	1.43883	0.150199	0.219065	VN1R1	protein_cc
ENSG0000(	201.9271	-0.57821	0.113272	-5.10464	3.31E-07	1.44E-06	FGF9	protein_cc
ENSG0000(	201.821	0.610209	0.088439	6.899782	5.21E-12	4.82E-11	RP11-289	pseudoge
ENSG0000(	201.7781	-0.13595	0.047576	-2.85756	0.004269	0.00923	ENTPD7	protein_cc
ENSG0000(	201.7343	0.259465	0.089425	2.901481	0.003714	0.008124	RP11-22B	pseudoge
ENSG0000(	201.5263	0.225635	0.073339	3.076623	0.002094	0.004813	AC005618	lincRNA
ENSG0000(	201.4951	1.708567	0.157554	10.84429	2.12E-27	2.92E-25	HOTAIRM	antisense
ENSG0000(	201.495	0.360378	0.059215	6.085952	1.16E-09	7.48E-09	H1FX-AS1	antisense
ENSG0000(	201.4925	-0.1565	0.096128	-1.62802	0.103521	0.15916	RERG	protein_cc
ENSG0000(	201.4707	0.302303	0.058502	5.167397	2.37E-07	1.06E-06	TCTE3	protein_cc
ENSG0000(	201.4517	-0.78023	0.122633	-6.36231	1.99E-10	1.44E-09	OPRD1	protein_cc
ENSG0000(	201.2239	-0.07862	0.104437	-0.75276	0.451594	0.548203	SNX20	protein_cc
ENSG0000(	201.1516	-0.18521	0.082509	-2.24469	0.024788	0.045184	ZC3H12A	protein_cc
ENSG0000(	201.0961	-0.37403	0.13223	-2.82863	0.004675	0.010012	CXCL5	protein_cc
ENSG0000(	201.0895	-0.05227	0.046439	-1.12549	0.260383	0.34867	METTL12	protein_cc
ENSG0000(	201.0666	-0.77087	0.100909	-7.63925	2.18E-14	2.97E-13	PLIN4	protein_cc
ENSG0000(	201.0314	0.161281	0.119153	1.353555	0.175878	0.250435	HRASLS5	protein_cc
ENSG0000(	201.0135	0.062411	0.045197	1.380871	0.167319	0.240059	GTF2H4	protein_cc
ENSG0000(	201.0065	0.32897	0.12227	2.690518	0.007134	0.014712	WDR16	protein_cc
ENSG0000(	200.9985	0.008685	0.091713	0.094701	0.924552	0.946816	SLC39A4	protein_cc
ENSG0000(	200.9324	0.199743	0.07697	2.595094	0.009457	0.018993	LINC0065	lincRNA
ENSG0000(	200.7399	0.333868	0.04402	7.584529	3.34E-14	4.40E-13	AP001258	lincRNA
ENSG0000(	200.7123	-0.05449	0.049931	-1.09141	0.275094	0.365158	ASAH2B	protein_cc
ENSG0000(	200.644	0.835816	0.100132	8.347174	6.99E-17	1.46E-15	RAD54L	protein_cc
ENSG0000(	200.6163	0.271477	0.06698	4.053108	5.05E-05	0.000155	PRR22	protein_cc
ENSG0000(	200.5697	0.071174	0.059937	1.18748	0.235038	0.319729	IQCD	protein_cc
ENSG0000(	200.4716	0.371116	0.047511	7.811145	5.67E-15	8.62E-14	ZNF69	protein_cc
ENSG0000(	200.3432	0.912778	0.109163	8.361583	6.19E-17	1.30E-15	BRIP1	protein_cc
ENSG0000(	200.2904	-0.25052	0.129473	-1.93495	0.052997	0.088932	CECR7	lincRNA
ENSG0000(	200.1397	0.136423	0.208235	0.655142	0.512377	0.605383	AC062021	lincRNA
ENSG0000(	200.1149	0.135918	0.043664	3.112804	0.001853	0.004301	CBWD5	protein_cc
ENSG0000(	199.7652	0.461229	0.107431	4.293258	1.76E-05	5.85E-05	SLFN13	protein_cc
ENSG0000(	199.7315	-0.1929	0.092251	-2.091	0.036528	0.064029	KCNQ4	protein_cc
ENSG0000(	199.6567	0.182854	0.040727	4.489721	7.13E-06	2.52E-05	FAM86EP	pseudoge
ENSG0000(	199.6008	0.240312	0.046697	5.146235	2.66E-07	1.18E-06	FDX1L	protein_cc
ENSG0000(	199.5741	-0.04441	0.075689	-0.58669	0.557415	0.646632	LINC0089	processed
ENSG0000(	199.3586	0.441676	0.111671	3.955162	7.65E-05	0.000228	MORN3	protein_cc
ENSG0000(	199.154	1.143412	0.109041	10.48604	1.00E-25	1.02E-23	DEPDC1B	protein_cc
ENSG0000(	199.1467	-0.04966	0.119878	-0.41425	0.678693	0.752733	HK3	protein_cc

ENSG0000(	199.076	0.128992	0.070815	1.821526	0.068527	0.111225	UCN	protein_co
ENSG0000(	198.8601	0.137959	0.082934	1.663473	0.096218	0.1494	SH3D21	protein_co
ENSG0000(	198.8402	0.067068	0.042593	1.574637	0.11534	0.174776	CTD-2186	lincRNA
ENSG0000(	198.7345	-0.10856	0.058332	-1.86113	0.062725	0.102991	RP11-389	sense_ove
ENSG0000(	198.4245	0.34993	0.038336	9.128074	6.97E-20	2.33E-18	NDUFA7	protein_co
ENSG0000(	198.4199	0.671244	0.099442	6.750101	1.48E-11	1.28E-10	EEF1A1P1:	pseudoge
ENSG0000(	198.3204	0.11092	0.049956	2.220377	0.026393	0.04784	RP11-345.	protein_co
ENSG0000(	198.2028	0.196987	0.040786	4.829757	1.37E-06	5.41E-06	PMS2P4	pseudoge
ENSG0000(	198.1648	0.447993	0.053729	8.337949	7.56E-17	1.57E-15	GS1-124K	lincRNA
ENSG0000(	198.1603	-0.30154	0.098198	-3.0707	0.002136	0.0049	ZNF826P	pseudoge
ENSG0000(	197.3772	0.178891	0.044337	4.034832	5.46E-05	0.000167	AF127936	lincRNA
ENSG0000(	197.3722	1.709207	0.229364	7.451937	9.20E-14	1.13E-12	HOXA10	protein_co
ENSG0000(	197.3033	1.001554	0.098513	10.16673	2.79E-24	2.12E-22	RAD51	protein_co
ENSG0000(	197.2918	0.021091	0.041652	0.50637	0.612597	0.696047	RAD51B	protein_co
ENSG0000(	197.271	-0.12266	0.043615	-2.81237	0.004918	0.010494	RP11-452	antisense
ENSG0000(	197.2485	-0.02693	0.095287	-0.28261	0.777479	0.833826	DNAAF1	protein_co
ENSG0000(	197.2043	0.170696	0.036546	4.670767	3.00E-06	1.12E-05	SCLY	protein_co
ENSG0000(	197.1733	-0.25666	0.060279	-4.25791	2.06E-05	6.78E-05	RP11-181	antisense
ENSG0000(	197.0528	-0.06236	0.046082	-1.35332	0.175952	0.250497	SDAD1P1	pseudoge
ENSG0000(	196.8442	1.064823	0.07817	13.6219	2.97E-42	5.09E-39	ANXA2R	protein_co
ENSG0000(	196.5425	-0.35735	0.073793	-4.84256	1.28E-06	5.09E-06	GXYLT2	protein_co
ENSG0000(	196.5367	0.15351	0.05979	2.567487	0.010244	0.020412	NANOS3	protein_co
ENSG0000(	196.1538	-0.5708	0.082079	-6.95431	3.54E-12	3.39E-11	DEGS2	protein_co
ENSG0000(	196.0398	-0.25784	0.122013	-2.11319	0.034584	0.060975	RP11-313.	pseudoge
ENSG0000(	196.0348	0.707415	0.090888	7.78334	7.06E-15	1.06E-13	RP11-571	lincRNA
ENSG0000(	195.9839	0.075445	0.105699	0.713769	0.47537	0.571211	TNFAIP8	protein_co
ENSG0000(	195.9711	1.120246	0.128367	8.726866	2.62E-18	6.85E-17	RP11-121	pseudoge
ENSG0000(	195.9649	-0.45483	0.06407	-7.09894	1.26E-12	1.29E-11	RNF39	protein_co
ENSG0000(	195.9058	0.327817	0.056595	5.792371	6.94E-09	3.95E-08	FAM86C1	protein_co
ENSG0000(	195.8765	-0.34297	0.057039	-6.01291	1.82E-09	1.14E-08	TAF4B	protein_co
ENSG0000(	195.7578	0.123095	0.047769	2.576863	0.00997	0.019923	ZNF497	protein_co
ENSG0000(	195.7407	0.167925	0.0541	3.103961	0.001909	0.004421	CHKB	protein_co
ENSG0000(	195.7322	0.07276	0.093291	0.779928	0.435433	0.532448	HCST	protein_co
ENSG0000(	195.6804	0.129104	0.045094	2.86299	0.004197	0.009092	RP11-355	antisense
ENSG0000(	195.6297	-0.18403	0.041146	-4.4727	7.72E-06	2.72E-05	AC003665	antisense
ENSG0000(	195.5815	0.825305	0.110701	7.455271	8.97E-14	1.10E-12	CLSPN	protein_co
ENSG0000(	195.2801	0.145461	0.155985	0.932531	0.351062	0.446526	ACTG2	protein_co
ENSG0000(	195.2465	0.175605	0.063167	2.779996	0.005436	0.011501	AC068282	lincRNA
ENSG0000(	195.1537	-0.13054	0.067315	-1.93931	0.052463	0.088137	GPLD1	protein_co
ENSG0000(	195.1176	-0.76333	0.102262	-7.46443	8.37E-14	1.03E-12	ADRA1B	protein_co
ENSG0000(	195.0829	-1.14002	0.130763	-8.71818	2.83E-18	7.37E-17	CPNE7	protein_co
ENSG0000(	195.0415	-0.01171	0.038278	-0.30596	0.759636	0.819326	RP11-110	antisense
ENSG0000(	195.04	-1.09113	0.138979	-7.85105	4.13E-15	6.43E-14	RBP4	protein_co
ENSG0000(	194.8911	0.103509	0.059378	1.743211	0.081297	0.128988	COMTD1	protein_co
ENSG0000(	194.8414	-0.33271	0.115937	-2.86972	0.004108	0.008916	SBSPON	protein_co
ENSG0000(	194.6402	0.930269	0.118751	7.833809	4.73E-15	7.31E-14	CARD16	protein_co
ENSG0000(	194.5528	-0.09572	0.04357	-2.197	0.02802	0.050427	RP11-137	processed
ENSG0000(	194.4958	-0.12386	0.079804	-1.552	0.120661	0.181535	GRM8	protein_co
ENSG0000(	194.4623	-0.1092	0.069388	-1.57378	0.115539	0.175038	DPEP2	protein_co
ENSG0000(	194.3023	-0.17017	0.053878	-3.15844	0.001586	0.003729	CCM2L	protein_co
ENSG0000(	194.2973	-0.04076	0.050498	-0.80707	0.419626	0.51656	RBKS	protein_co
ENSG0000(	194.2212	-0.15679	0.095837	-1.63603	0.101833	0.156963	PLEKHG4	protein_co
ENSG0000(	194.1642	0.126882	0.041629	3.047899	0.002304	0.005256	ZNF815P	pseudoge
ENSG0000(	194.0829	0.533521	0.121995	4.373295	1.22E-05	4.16E-05	RPL39L	protein_co
ENSG0000(	193.9506	0.028028	0.069204	0.405013	0.685468	0.758401	RTEL1-TN	protein_co
ENSG0000(	193.9132	1.648611	0.286749	5.749318	8.96E-09	5.00E-08	HOXC10	protein_co
ENSG0000(	193.7789	-0.58093	0.10679	-5.43994	5.33E-08	2.64E-07	FMO2	protein_co
ENSG0000(	193.6908	0.01647	0.042393	0.388498	0.697648	0.768532	RP11-348	lincRNA
ENSG0000(	193.6338	-1.26465	0.141518	-8.93628	4.03E-19	1.19E-17	SYT2	protein_co

ENSG0000	193.5866	0.286795	0.118176	2.426844	0.015231	0.02918	HSPB7	protein_cc
ENSG0000	193.524	0.084507	0.079257	1.066242	0.286314	0.377312	ACOT1	protein_cc
ENSG0000	193.5133	-0.50889	0.073127	-6.95893	3.43E-12	3.29E-11	DLEU7	protein_cc
ENSG0000	193.4397	-0.00334	0.054734	-0.06094	0.951407	0.966543	CCRN4L	protein_cc
ENSG0000	193.3562	1.595131	0.181264	8.800041	1.37E-18	3.73E-17	HOXB2	protein_cc
ENSG0000	193.2466	0.644963	0.109444	5.893082	3.79E-09	2.25E-08	C14orf23	protein_cc
ENSG0000	192.8532	0.570434	0.075658	7.539665	4.71E-14	6.06E-13	CCNJL	protein_cc
ENSG0000	192.6914	-0.09283	0.037413	-2.48116	0.013095	0.025468	RP11-121	lincRNA
ENSG0000	192.6175	0.515618	0.095624	5.392158	6.96E-08	3.38E-07	CCDC152	protein_cc
ENSG0000	192.6152	0.087679	0.095056	0.922393	0.356323	0.451974	CYTIP	protein_cc
ENSG0000	192.6019	-0.65535	0.151667	-4.32096	1.55E-05	5.20E-05	MSX2	protein_cc
ENSG0000	192.4649	-0.54835	0.151407	-3.62171	0.000293	0.000789	SCN9A	protein_cc
ENSG0000	192.128	0.258989	0.07955	3.255693	0.001131	0.002729	HAS3	protein_cc
ENSG0000	192.0342	0.503231	0.157656	3.191947	0.001413	0.003353	C9orf24	protein_cc
ENSG0000	192.0015	0.334505	0.076592	4.367342	1.26E-05	4.27E-05	GTF2H2	protein_cc
ENSG0000	191.9683	0.264512	0.0417	6.343223	2.25E-10	1.62E-09	ZNF284	protein_cc
ENSG0000	191.9677	-0.02345	0.111675	-0.20997	0.833693	0.87876	LANCL3	protein_cc
ENSG0000	191.9552	1.596676	0.200998	7.943753	1.96E-15	3.23E-14	CHRNA9	protein_cc
ENSG0000	191.84	0.408335	0.114297	3.572564	0.000354	0.00094	PTPN7	protein_cc
ENSG0000	191.6975	0.199224	0.054848	3.632288	0.000281	0.00076	RP11-326	lincRNA
ENSG0000	191.6705	-0.3806	0.100579	-3.78413	0.000154	0.000436	CCDC170	protein_cc
ENSG0000	191.5535	0.271618	0.115803	2.345509	0.019001	0.035554	C17orf72	protein_cc
ENSG0000	191.545	0.645621	0.098012	6.587138	4.48E-11	3.60E-10	TMEM221	protein_cc
ENSG0000	191.5219	-1.17036	0.119379	-9.80372	1.09E-22	5.97E-21	GDF7	protein_cc
ENSG0000	191.4734	-0.21174	0.108272	-1.95565	0.050507	0.085249	PTGER4	protein_cc
ENSG0000	191.4634	-0.13888	0.120044	-1.15689	0.247316	0.333866	SLC22A3	protein_cc
ENSG0000	191.4495	0.162306	0.028073	5.781619	7.40E-09	4.19E-08	ZNF57	protein_cc
ENSG0000	191.2869	0.157186	0.046369	3.389866	0.000699	0.001754	CTD-3074	antisense
ENSG0000	191.2792	-0.05909	0.086634	-0.68204	0.495216	0.589515	LRRC37A4	pseudoge
ENSG0000	191.2028	0.904497	0.146612	6.169308	6.86E-10	4.59E-09	LINC0111	lincRNA
ENSG0000	191.0961	0.134107	0.079865	1.679177	0.093117	0.145217	Z83851.1	lincRNA
ENSG0000	191.0476	0.52256	0.084539	6.181252	6.36E-10	4.27E-09	VASH2	protein_cc
ENSG0000	190.9294	-0.35256	0.073476	-4.79825	1.60E-06	6.27E-06	RAPH1	protein_cc
ENSG0000	190.9075	0.276176	0.05024	5.497111	3.86E-08	1.95E-07	CTD-3065	antisense
ENSG0000	190.8994	-0.21441	0.095828	-2.23742	0.025259	0.045956	SEC14L6	protein_cc
ENSG0000	190.7022	0.398471	0.050061	7.959718	1.72E-15	2.86E-14	CTD-3138	antisense
ENSG0000	190.4247	-0.54523	0.060916	-8.95061	3.54E-19	1.05E-17	GLUD2	protein_cc
ENSG0000	190.394	-0.00593	0.087475	-0.06774	0.945989	0.962394	CACNA2D	protein_cc
ENSG0000	190.3739	0.03907	0.088133	0.443303	0.657547	0.734948	PYROXD2	protein_cc
ENSG0000	190.2451	0.279532	0.121734	2.296257	0.021661	0.040014	FAM26F	protein_cc
ENSG0000	190.2154	-0.01522	0.084786	-0.17954	0.857517	0.89702	TMEM156	protein_cc
ENSG0000	189.9362	-0.58341	0.085409	-6.83078	8.45E-12	7.58E-11	TINCR	lincRNA
ENSG0000	189.8824	0.148878	0.057331	2.596819	0.009409	0.018906	HYLS1	protein_cc
ENSG0000	189.8508	0.49219	0.095639	5.14635	2.66E-07	1.18E-06	NPIPB5	protein_cc
ENSG0000	189.8405	0.670865	0.111575	6.01269	1.82E-09	1.14E-08	FAM115C	protein_cc
ENSG0000	189.833	-0.75776	0.138219	-5.4823	4.20E-08	2.11E-07	FNDC9	protein_cc
ENSG0000	189.6961	0.377335	0.0845	4.465505	7.99E-06	2.80E-05	CHCHD2P	pseudoge
ENSG0000	189.6204	0.490894	0.113102	4.340282	1.42E-05	4.79E-05	PRKCDBP	protein_cc
ENSG0000	189.5744	-0.00422	0.139061	-0.03033	0.975804	0.982951	OSM	protein_cc
ENSG0000	189.5242	0.231237	0.103658	2.230766	0.025697	0.046682	RP11-277	antisense
ENSG0000	189.4305	0.333064	0.056605	5.883985	4.01E-09	2.37E-08	STAP2	protein_cc
ENSG0000	189.3592	0.262925	0.085885	3.06137	0.002203	0.005043	GOLGA2P	pseudoge
ENSG0000	189.3472	0.271904	0.074523	3.648579	0.000264	0.000716	AP000473	lincRNA
ENSG0000	189.2696	-0.18934	0.080583	-2.34968	0.01879	0.035194	NKD2	protein_cc
ENSG0000	189.171	-0.7601	0.126611	-6.00342	1.93E-09	1.20E-08	XK	protein_cc
ENSG0000	189.151	0.762114	0.120159	6.342565	2.26E-10	1.63E-09	RAB42	protein_cc
ENSG0000	189.0849	0.116556	0.082401	1.414499	0.157216	0.227661	ASMTL-AS	antisense
ENSG0000	189.0511	0.432087	0.048727	8.867585	7.47E-19	2.12E-17	AC004980	pseudoge
ENSG0000	189.0107	0.307256	0.047836	6.423104	1.34E-10	9.98E-10	DLEU2	processed

ENSG0000(	188.9292	-0.35646	0.072205	-4.93678	7.94E-07	3.26E-06	CASS4	protein_cc
ENSG0000(	188.8906	-0.02243	0.087489	-0.25639	0.797649	0.850445	STX16-NP	protein_cc
ENSG0000(	188.7544	-0.23376	0.130637	-1.78941	0.073548	0.11832	TNNT1	protein_cc
ENSG0000(	188.7178	-0.12378	0.12279	-1.00807	0.313421	0.406815	PCDHA1	protein_cc
ENSG0000(	188.6588	-0.19006	0.097547	-1.94841	0.051365	0.086529	PIK3CG	protein_cc
ENSG0000(	188.6093	0.057773	0.147299	0.392218	0.694897	0.766282	HSPA7	pseudoge
ENSG0000(	188.4711	0.115176	0.066048	1.743813	0.081192	0.128871	PCDHB13	protein_cc
ENSG0000(	188.416	0.075904	0.10401	0.729778	0.465526	0.561466	WDR96	protein_cc
ENSG0000(	188.3025	0.181233	0.167379	1.082774	0.278909	0.369304	LHX9	protein_cc
ENSG0000(	188.2776	0.84411	0.094738	8.909912	5.11E-19	1.48E-17	RP11-3P1	pseudoge
ENSG0000(	188.2357	-0.06397	0.073586	-0.86934	0.384661	0.48134	ABCA17P	pseudoge
ENSG0000(	188.1605	-0.3184	0.060309	-5.27957	1.29E-07	6.03E-07	AC005003	protein_cc
ENSG0000(	187.7362	-0.02072	0.094428	-0.21939	0.826348	0.872718	B4GALNT3	protein_cc
ENSG0000(	187.467	-0.27905	0.057364	-4.86453	1.15E-06	4.59E-06	TMEM88	protein_cc
ENSG0000(	187.3665	0.295814	0.059568	4.965988	6.84E-07	2.84E-06	POLR2J3	protein_cc
ENSG0000(	187.1081	-0.55794	0.10945	-5.09765	3.44E-07	1.50E-06	RP11-284	lincRNA
ENSG0000(	187.0572	0.246621	0.044428	5.551042	2.84E-08	1.47E-07	ZNF491	protein_cc
ENSG0000(	187.0016	-0.23187	0.080045	-2.89677	0.00377	0.008239	FGF11	protein_cc
ENSG0000(	186.986	-0.04184	0.042582	-0.9825	0.325854	0.420049	CEP128	protein_cc
ENSG0000(	186.6235	0.116181	0.085971	1.351395	0.176569	0.251175	RP11-436	lincRNA
ENSG0000(	186.5152	-0.07785	0.046988	-1.65674	0.097572	0.151228	RP11-244	processed
ENSG0000(	186.4728	-0.00106	0.036674	-0.029	0.976864	0.983696	KDM4D	protein_cc
ENSG0000(	186.4079	0.550796	0.113478	4.853771	1.21E-06	4.83E-06	CISH	protein_cc
ENSG0000(	186.3767	0.449813	0.073241	6.141542	8.17E-10	5.40E-09	YBX1P10	pseudoge
ENSG0000(	185.9435	-0.04649	0.074252	-0.62616	0.531211	0.622806	OPN3	protein_cc
ENSG0000(	185.8559	0.756886	0.109176	6.932737	4.13E-12	3.90E-11	GPR3	protein_cc
ENSG0000(	185.8417	0.233409	0.048669	4.795887	1.62E-06	6.34E-06	CHAC2	protein_cc
ENSG0000(	185.6663	-0.15496	0.100817	-1.53701	0.12429	0.186232	ST6GALNA	protein_cc
ENSG0000(	185.6122	-0.176	0.057881	-3.04081	0.002359	0.005372	CCDC13	protein_cc
ENSG0000(	185.5877	-0.00566	0.078591	-0.072	0.942604	0.959852	C1orf228	protein_cc
ENSG0000(	185.5685	0.33408	0.05103	6.546694	5.88E-11	4.64E-10	CCT6P3	pseudoge
ENSG0000(	185.5066	-0.22261	0.08823	-2.52302	0.011635	0.022903	RGS18	protein_cc
ENSG0000(	185.5024	0.286847	0.067936	4.222294	2.42E-05	7.83E-05	C7orf63	protein_cc
ENSG0000(	185.4315	0.098747	0.212496	0.4647	0.642146	0.721962	LHX5	protein_cc
ENSG0000(	185.2574	-0.46095	0.124692	-3.6967	0.000218	0.000601	SLC25A21	protein_cc
ENSG0000(	185.0316	0.072386	0.05099	1.419604	0.155723	0.225833	RP11-45P	antisense
ENSG0000(	184.948	-0.24081	0.0974	-2.47234	0.013423	0.026047	ISM1	protein_cc
ENSG0000(	184.837	-0.18726	0.085221	-2.19738	0.027993	0.050385	EFHB	protein_cc
ENSG0000(	184.8286	-1.16287	0.109418	-10.6277	2.22E-26	2.57E-24	ABCG4	protein_cc
ENSG0000(	184.7943	-1.00149	0.136095	-7.35874	1.86E-13	2.15E-12	ENTPD3	protein_cc
ENSG0000(	184.7406	-0.48334	0.090837	-5.32095	1.03E-07	4.89E-07	ALDH1A2	protein_cc
ENSG0000(	184.6642	-0.73749	0.13099	-5.63016	1.80E-08	9.61E-08	ZMAT4	protein_cc
ENSG0000(	184.5367	0.286294	0.04496	6.367675	1.92E-10	1.40E-09	RP11-355(	sense_ove
ENSG0000(	184.4988	-0.4227	0.09625	-4.39172	1.12E-05	3.85E-05	KCTD8	protein_cc
ENSG0000(	184.3985	-0.87417	0.074026	-11.809	3.51E-32	1.21E-29	AC011747	antisense
ENSG0000(	184.3302	0.954883	0.128862	7.410107	1.26E-13	1.51E-12	RP11-36C	pseudoge
ENSG0000(	184.3158	0.089488	0.036026	2.483942	0.012994	0.025285	CHMP4A	protein_cc
ENSG0000(	184.1743	0.341691	0.098926	3.454015	0.000552	0.001413	ARHGAP1	protein_cc
ENSG0000(	184.13	-1.29942	0.136447	-9.52322	1.68E-21	7.49E-20	TNF	protein_cc
ENSG0000(	184.1156	0.990554	0.137288	7.215135	5.39E-13	5.82E-12	SH2D4A	protein_cc
ENSG0000(	184.0194	-0.31448	0.06666	-4.7177	2.39E-06	9.09E-06	DNAH10	protein_cc
ENSG0000(	183.4247	-0.81227	0.105884	-7.6713	1.70E-14	2.36E-13	TTPA	protein_cc
ENSG0000(	183.3704	0.346881	0.046548	7.452073	9.19E-14	1.13E-12	C1orf74	protein_cc
ENSG0000(	183.2754	-0.02263	0.091046	-0.2485	0.803744	0.85517	CITED4	protein_cc
ENSG0000(	183.237	-1.08862	0.119525	-9.10791	8.40E-20	2.78E-18	LRFN2	protein_cc
ENSG0000(	183.1053	0.379967	0.138988	2.733808	0.006261	0.013068	GBP5	protein_cc
ENSG0000(	183.0768	-0.72684	0.151708	-4.79108	1.66E-06	6.48E-06	PRKG2	protein_cc
ENSG0000(	182.8303	0.193765	0.059808	3.239805	0.001196	0.002873	RP11-384	pseudoge
ENSG0000(	182.7747	-0.54892	0.085287	-6.43612	1.23E-10	9.22E-10	NKAIN3	protein_cc

ENSG0000(	182.7679	0.09989	0.057977	1.72294	0.084899	0.133991	NUDT8	protein_cc
ENSG0000(	182.6738	-0.42383	0.102477	-4.13585	3.54E-05	0.000111	RNF207	protein_cc
ENSG0000(	182.5219	0.044219	0.041898	1.055394	0.291245	0.382755	RP11-282(	antisense
ENSG0000(	182.4893	-0.03362	0.079016	-0.42551	0.670466	0.745619	WFIKKN1	protein_cc
ENSG0000(	182.3341	-0.47598	0.083456	-5.70327	1.18E-08	6.45E-08	GNA14	protein_cc
ENSG0000(	182.1718	-0.37084	0.066581	-5.56969	2.55E-08	1.33E-07	ZNF385C	protein_cc
ENSG0000(	182.1278	0.873599	0.126626	6.899058	5.23E-12	4.85E-11	SNHG18	lincRNA
ENSG0000(	182.1208	0.84003	0.073929	11.36268	6.41E-30	1.41E-27	HNF4G	protein_cc
ENSG0000(	182.0445	-0.0296	0.131551	-0.22502	0.821967	0.869564	PCDHA3	protein_cc
ENSG0000(	181.9709	0.210492	0.069364	3.034591	0.002409	0.005478	SAMD13	protein_cc
ENSG0000(	181.842	0.171132	0.121681	1.406395	0.159607	0.230604	FST	protein_cc
ENSG0000(	181.8339	0.374722	0.09126	4.106103	4.02E-05	0.000126	TEKT2	protein_cc
ENSG0000(	181.7885	-0.28596	0.087257	-3.27726	0.001048	0.002544	LILRA1	protein_cc
ENSG0000(	181.7814	-0.11366	0.086685	-1.31119	0.189795	0.267065	RP11-578	pseudoge
ENSG0000(	181.6852	0.456716	0.081099	5.631555	1.79E-08	9.53E-08	RP11-848	pseudoge
ENSG0000(	181.6058	-0.18072	0.039979	-4.52031	6.18E-06	2.20E-05	RPL13P5	pseudoge
ENSG0000(	181.5081	0.590272	0.139778	4.222941	2.41E-05	7.81E-05	CD48	protein_cc
ENSG0000(	181.3947	-0.28808	0.048027	-5.99819	2.00E-09	1.24E-08	RP11-504	lincRNA
ENSG0000(	181.3885	0.371018	0.148954	2.490826	0.012745	0.024842	SPATA18	protein_cc
ENSG0000(	181.2956	0.166214	0.047882	3.471292	0.000518	0.001333	ZNF749	protein_cc
ENSG0000(	181.2326	0.108996	0.14334	0.760402	0.447014	0.543828	DHRS2	protein_cc
ENSG0000(	181.1253	0.121402	0.092709	1.309501	0.190365	0.267698	TRPC3	protein_cc
ENSG0000(	181.0988	0.460136	0.052202	8.814532	1.20E-18	3.30E-17	PYURF	protein_cc
ENSG0000(	180.892	0.135117	0.034159	3.955555	7.64E-05	0.000228	METTL25	protein_cc
ENSG0000(	180.8543	0.151787	0.048671	3.118637	0.001817	0.004224	RP11-635	antisense
ENSG0000(	180.8523	-0.01069	0.052326	-0.20432	0.838106	0.882245	RP5-882C	antisense
ENSG0000(	180.7541	-0.63439	0.079647	-7.96499	1.65E-15	2.75E-14	ABCA9	protein_cc
ENSG0000(	180.6689	-0.0828	0.074045	-1.11829	0.263442	0.352067	CTD-2517	processed
ENSG0000(	180.6121	0.270202	0.057439	4.704131	2.55E-06	9.66E-06	SLC16A1-	antisense
ENSG0000(	180.4409	0.040196	0.134153	0.29963	0.764459	0.823276	NRG1	protein_cc
ENSG0000(	180.3276	-0.8182	0.108185	-7.56299	3.94E-14	5.13E-13	DUSP2	protein_cc
ENSG0000(	180.2592	0.429814	0.054765	7.848284	4.22E-15	6.57E-14	RP11-126	antisense
ENSG0000(	180.1864	0.30694	0.060634	5.062182	4.14E-07	1.78E-06	ZNF816	protein_cc
ENSG0000(	180.0783	-0.04912	0.041842	-1.17406	0.240369	0.325742	HYPK	protein_cc
ENSG0000(	179.7732	0.218818	0.028336	7.722168	1.14E-14	1.64E-13	STARD7-A	processed
ENSG0000(	179.6603	-0.17598	0.075219	-2.33959	0.019305	0.036066	FHL5	protein_cc
ENSG0000(	179.6554	0.537518	0.064953	8.275501	1.28E-16	2.55E-15	CTB-25B1	processed
ENSG0000(	179.4913	-0.04077	0.096899	-0.42076	0.673928	0.748579	TLR10	protein_cc
ENSG0000(	179.4644	-0.00248	0.044353	-0.05602	0.955328	0.969184	TSGA10	protein_cc
ENSG0000(	179.4589	0.131868	0.05356	2.462044	0.013815	0.02674	RP11-644	protein_cc
ENSG0000(	179.4349	-0.54826	0.08097	-6.7711	1.28E-11	1.11E-10	CLIC5	protein_cc
ENSG0000(	179.3451	0.379502	0.085269	4.450621	8.56E-06	2.99E-05	AP000769	protein_cc
ENSG0000(	178.8988	0.329252	0.075173	4.379927	1.19E-05	4.05E-05	AC018738	pseudoge
ENSG0000(	178.8697	-0.24142	0.139191	-1.73445	0.082839	0.13107	COL21A1	protein_cc
ENSG0000(	178.7825	-0.10109	0.085496	-1.18244	0.23703	0.322034	RP11-706	protein_cc
ENSG0000(	178.7227	0.081143	0.049873	1.626984	0.103741	0.159449	Y_RNA	antisense
ENSG0000(	178.5536	-0.03876	0.052087	-0.74421	0.456749	0.5532	THAP7-AS	antisense
ENSG0000(	178.5279	-0.00677	0.048451	-0.13967	0.888919	0.92085	AP001062	antisense
ENSG0000(	178.4767	0.106957	0.104934	1.019282	0.308069	0.401147	PALM3	protein_cc
ENSG0000(	178.4096	0.201768	0.045485	4.435976	9.17E-06	3.18E-05	RP11-73E.	antisense
ENSG0000(	178.2498	0.127296	0.138639	0.918181	0.358524	0.454317	BCL2A1	protein_cc
ENSG0000(	178.055	-0.28648	0.073974	-3.87274	0.000108	0.000313	C19orf38	protein_cc
ENSG0000(	177.8161	-0.58402	0.082269	-7.09892	1.26E-12	1.29E-11	LONRF3	protein_cc
ENSG0000(	177.7104	0.250584	0.089418	2.802388	0.005073	0.010802	KAZALD1	protein_cc
ENSG0000(	177.2876	-0.48962	0.09108	-5.37572	7.63E-08	3.68E-07	SCUBE3	protein_cc
ENSG0000(	177.2415	0.140712	0.031177	4.513355	6.38E-06	2.27E-05	SPIN2B	protein_cc
ENSG0000(	177.1445	-0.32916	0.036452	-9.03004	1.72E-19	5.40E-18	AC006547	antisense
ENSG0000(	177.0157	-0.00401	0.063465	-0.06325	0.949566	0.965197	SLC25A34	protein_cc
ENSG0000(	176.9932	0.921493	0.107932	8.537713	1.37E-17	3.20E-16	ORC1	protein_cc

ENSG0000(	176.9789	-0.2147	0.100603	-2.13409	0.032836	0.058172	OTOGL	protein_cc
ENSG0000(	176.9371	-0.45636	0.136449	-3.34455	0.000824	0.00204	PLN	protein_cc
ENSG0000(	176.9154	0.235032	0.047899	4.906804	9.26E-07	3.76E-06	SPNS1	protein_cc
ENSG0000(	176.8615	0.064365	0.156163	0.412167	0.680217	0.753874	MFAP2	protein_cc
ENSG0000(	176.7467	0.366691	0.066929	5.478773	4.28E-08	2.15E-07	HAUS7	protein_cc
ENSG0000(	176.4602	1.89667	0.248106	7.644583	2.10E-14	2.87E-13	HOXA5	protein_cc
ENSG0000(	176.3595	-0.00541	0.077131	-0.07017	0.944055	0.96083	CKMT2	protein_cc
ENSG0000(	176.2246	-0.09396	0.051815	-1.81334	0.069779	0.113014	MAMSTR	protein_cc
ENSG0000(	176.2001	-0.76806	0.091643	-8.38103	5.25E-17	1.12E-15	RP11-434	lincRNA
ENSG0000(	176.167	0.110081	0.058291	1.88847	0.058963	0.097581	FTCDNL1	protein_cc
ENSG0000(	176.1629	0.523935	0.087885	5.96161	2.50E-09	1.53E-08	C8orf56	protein_cc
ENSG0000(	175.7007	-0.4181	0.126519	-3.30464	0.000951	0.002325	FAP	protein_cc
ENSG0000(	175.6261	0.661045	0.106927	6.182201	6.32E-10	4.25E-09	SLC26A10	protein_cc
ENSG0000(	175.5348	-0.11127	0.138322	-0.80439	0.421171	0.518136	SNX31	protein_cc
ENSG0000(	175.5259	1.659301	0.215066	7.715322	1.21E-14	1.73E-13	EN1	protein_cc
ENSG0000(	175.4921	0.233197	0.051718	4.508974	6.51E-06	2.32E-05	AC083799	pseudoge
ENSG0000(	175.4105	0.871111	0.133367	6.531688	6.50E-11	5.10E-10	HOXC4	protein_cc
ENSG0000(	175.3348	-1.45317	0.155257	-9.35975	7.99E-21	3.12E-19	CCKBR	protein_cc
ENSG0000(	175.2819	-0.65601	0.108162	-6.06508	1.32E-09	8.44E-09	CLMP	protein_cc
ENSG0000(	175.2606	0.519777	0.126627	4.104799	4.05E-05	0.000126	TWIST1	protein_cc
ENSG0000(	175.2387	-0.50284	0.078918	-6.37164	1.87E-10	1.36E-09	PDXP	protein_cc
ENSG0000(	175.1839	0.10113	0.049678	2.035701	0.04178	0.072114	RP11-83A	antisense
ENSG0000(	174.8718	1.022864	0.110641	9.244885	2.35E-20	8.47E-19	AC004453	pseudoge
ENSG0000(	174.8714	1.209281	0.121177	9.979495	1.87E-23	1.22E-21	SKA1	protein_cc
ENSG0000(	174.8401	0.449328	0.055294	8.126136	4.43E-16	8.13E-15	CCDC15	protein_cc
ENSG0000(	174.8105	0.345896	0.087504	3.952898	7.72E-05	0.00023	FTH1P20	pseudoge
ENSG0000(	174.5182	0.758783	0.110476	6.868296	6.50E-12	5.94E-11	LPIN3	protein_cc
ENSG0000(	174.49	-0.3635	0.092493	-3.92998	8.50E-05	0.000252	RP11-2E1	lincRNA
ENSG0000(	174.3765	-0.18383	0.073287	-2.50839	0.012128	0.02377	OVGP1	protein_cc
ENSG0000(	174.3488	-1.58175	0.156585	-10.1016	5.44E-24	3.93E-22	EMX1	protein_cc
ENSG0000(	174.3322	-0.27463	0.101649	-2.70173	0.006898	0.014273	LPAR4	protein_cc
ENSG0000(	174.2447	-0.46989	0.195666	-2.40148	0.016329	0.031077	PRKY	pseudoge
ENSG0000(	174.1525	-0.23523	0.086942	-2.70561	0.006818	0.014124	PLAC9	protein_cc
ENSG0000(	174.0723	0.26895	0.075006	3.585693	0.000336	0.000897	TBXA2R	protein_cc
ENSG0000(	174.0236	-0.29347	0.098868	-2.96832	0.002994	0.006674	FAM46C	protein_cc
ENSG0000(	174.0215	0.462189	0.060903	7.588912	3.23E-14	4.26E-13	ZNF726	protein_cc
ENSG0000(	174.0178	0.568596	0.120536	4.71722	2.39E-06	9.10E-06	PDCD1LG:	protein_cc
ENSG0000(	173.9871	0.112217	0.076777	1.461606	0.143849	0.210965	MFSD7	protein_cc
ENSG0000(	173.9598	1.245002	0.121333	10.26105	1.06E-24	8.72E-23	CLEC18B	protein_cc
ENSG0000(	173.7192	-0.1702	0.033441	-5.08974	3.59E-07	1.55E-06	STX18-AS	antisense
ENSG0000(	173.6474	0.504351	0.109536	4.604423	4.14E-06	1.52E-05	EIF5AL1	protein_cc
ENSG0000(	173.633	0.047255	0.075293	0.627611	0.530259	0.62199	AC012309	lincRNA
ENSG0000(	173.5844	-0.03976	0.090917	-0.43728	0.661911	0.738762	TTN-AS1	antisense
ENSG0000(	173.3706	-0.07396	0.047015	-1.57314	0.115687	0.17525	POLN	protein_cc
ENSG0000(	173.3473	0.309036	0.053315	5.796401	6.78E-09	3.86E-08	CTU1	protein_cc
ENSG0000(	173.3429	-0.35445	0.111808	-3.17017	0.001523	0.003593	CYP27C1	protein_cc
ENSG0000(	173.1852	0.129538	0.042537	3.045278	0.002325	0.005298	ACY1	protein_cc
ENSG0000(	173.0795	0.102539	0.046497	2.205269	0.027435	0.049484	CTC-241N	protein_cc
ENSG0000(	173.069	0.056063	0.037158	1.508779	0.131355	0.195125	AC005562	processed
ENSG0000(	173.0456	0.219902	0.082156	2.676622	0.007437	0.015289	WDR66	protein_cc
ENSG0000(	172.971	-0.96954	0.198779	-4.87751	1.07E-06	4.32E-06	DAO	protein_cc
ENSG0000(	172.959	0.007392	0.052478	0.140862	0.887979	0.920224	AC231657	protein_cc
ENSG0000(	172.9351	1.084933	0.133815	8.107704	5.16E-16	9.34E-15	GBP1P1	pseudoge
ENSG0000(	172.9242	0.49157	0.099974	4.91698	8.79E-07	3.58E-06	VWA3B	protein_cc
ENSG0000(	172.9199	0.117014	0.075283	1.554331	0.120106	0.180778	VMO1	protein_cc
ENSG0000(	172.8932	0.601565	0.067915	8.857641	8.17E-19	2.30E-17	AC006115	antisense
ENSG0000(	172.8593	0.160235	0.074776	2.142879	0.032123	0.057036	FAM218A	protein_cc
ENSG0000(	172.8437	0.144244	0.069414	2.078012	0.037708	0.065851	SYCE2	protein_cc
ENSG0000(	172.8207	0.11952	0.092811	1.287777	0.197824	0.276664	EGF	protein_cc

ENSG0000(	172.5745	0.748964	0.107816	6.946685	3.74E-12	3.56E-11	PVT1	processed
ENSG0000(	172.5367	-0.45211	0.088233	-5.12405	2.99E-07	1.31E-06	SLC31A2	protein_cc
ENSG0000(	172.4496	-0.03389	0.046413	-0.73023	0.465252	0.561234	RP11-85F:	antisense
ENSG0000(	172.2954	-0.10403	0.058973	-1.76406	0.077721	0.124163	C14orf64	protein_cc
ENSG0000(	171.9722	-0.12795	0.146466	-0.8736	0.382336	0.478913	IGFBPL1	protein_cc
ENSG0000(	171.9664	-0.25243	0.137938	-1.83	0.067249	0.109406	BDNF	protein_cc
ENSG0000(	171.7994	-0.05406	0.062111	-0.87046	0.384051	0.480739	PCAT6	antisense
ENSG0000(	171.67	-0.08875	0.098777	-0.89845	0.368948	0.465332	CFTR	protein_cc
ENSG0000(	171.6213	-0.67598	0.177043	-3.81814	0.000134	0.000384	ECEL1	protein_cc
ENSG0000(	171.5502	-0.23663	0.066538	-3.5563	0.000376	0.000994	RP11-677	antisense
ENSG0000(	171.5448	-0.33984	0.107415	-3.1638	0.001557	0.003667	PTPRH	protein_cc
ENSG0000(	171.5057	0.108849	0.076432	1.424137	0.154407	0.224352	RP11-397.	lincRNA
ENSG0000(	171.4276	-0.11331	0.049363	-2.29542	0.021709	0.040095	ZNF575	protein_cc
ENSG0000(	171.4247	-0.94732	0.131104	-7.22569	4.99E-13	5.41E-12	OGN	protein_cc
ENSG0000(	171.2631	0.081017	0.065587	1.235246	0.216739	0.298804	GOLGA6L!	protein_cc
ENSG0000(	171.1408	-0.24514	0.061201	-4.00539	6.19E-05	0.000188	PAQR5	protein_cc
ENSG0000(	171.0231	-0.20427	0.045111	-4.52821	5.95E-06	2.13E-05	RAP2C-AS	antisense
ENSG0000(	170.9833	0.029848	0.030875	0.966743	0.333672	0.428249	TTC4	protein_cc
ENSG0000(	170.8129	0.352871	0.077618	4.546227	5.46E-06	1.97E-05	SLC38A11	protein_cc
ENSG0000(	170.6514	0.316817	0.063956	4.953668	7.28E-07	3.01E-06	ULK4	protein_cc
ENSG0000(	170.637	-0.06749	0.09172	-0.73582	0.461842	0.557955	PRCD	protein_cc
ENSG0000(	170.6168	-0.99303	0.117391	-8.45915	2.69E-17	6.05E-16	TMEM200	protein_cc
ENSG0000(	170.5785	0.253888	0.114798	2.211605	0.026994	0.048776	C11orf70	protein_cc
ENSG0000(	170.3674	-0.06613	0.045877	-1.44136	0.149483	0.218137	C20ORF1:	protein_cc
ENSG0000(	170.3512	0.351714	0.102329	3.437078	0.000588	0.001498	C20orf26	protein_cc
ENSG0000(	170.1988	0.370753	0.085574	4.332542	1.47E-05	4.95E-05	ATP6V1B1	protein_cc
ENSG0000(	170.0225	-0.93864	0.149905	-6.26155	3.81E-10	2.65E-09	PRRG3	protein_cc
ENSG0000(	169.9581	-0.18328	0.063081	-2.90548	0.003667	0.008032	CDKL1	protein_cc
ENSG0000(	169.9423	0.414107	0.120137	3.446963	0.000567	0.001448	RSPH4A	protein_cc
ENSG0000(	169.7635	-0.29099	0.115507	-2.51922	0.011761	0.023126	FAM182A	processed
ENSG0000(	169.7024	0.665741	0.124844	5.332595	9.68E-08	4.60E-07	IGFBP7-AS	antisense
ENSG0000(	169.7001	0.205377	0.048021	4.276859	1.90E-05	6.26E-05	ZNF155	protein_cc
ENSG0000(	169.6376	1.781526	0.16168	11.01884	3.10E-28	4.88E-26	AC064875	processed
ENSG0000(	169.6038	0.827026	0.112634	7.342607	2.09E-13	2.41E-12	RP11-889	pseudoge
ENSG0000(	169.4823	-0.08307	0.060772	-1.36693	0.171647	0.245344	RP11-413	pseudoge
ENSG0000(	169.4528	0.077099	0.052806	1.460044	0.144278	0.211518	CTD-2006	antisense
ENSG0000(	169.2754	1.015607	0.188269	5.394447	6.87E-08	3.34E-07	C7orf57	protein_cc
ENSG0000(	169.1826	0.323064	0.055291	5.842957	5.13E-09	2.98E-08	EMC6	protein_cc
ENSG0000(	169.1486	0.137129	0.047876	2.864265	0.00418	0.009058	MBLAC1	protein_cc
ENSG0000(	169.1341	0.402605	0.121758	3.306606	0.000944	0.002311	RP11-551	pseudoge
ENSG0000(	169.1027	-0.15318	0.075228	-2.03624	0.041726	0.072044	HAAO	protein_cc
ENSG0000(	169.0238	0.543687	0.091417	5.947341	2.73E-09	1.66E-08	C5orf63	protein_cc
ENSG0000(	168.986	-0.1641	0.046434	-3.53407	0.000409	0.001074	ZSCAN20	protein_cc
ENSG0000(	168.89	-0.15004	0.099497	-1.50802	0.13155	0.195386	KLHL35	protein_cc
ENSG0000(	168.8593	-0.47051	0.099745	-4.71719	2.39E-06	9.11E-06	LINC0063:	protein_cc
ENSG0000(	168.7167	0.423141	0.052248	8.098706	5.55E-16	1.00E-14	AC092295	antisense
ENSG0000(	168.6899	-0.02251	0.056729	-0.3968	0.691518	0.763497	FMO4	protein_cc
ENSG0000(	168.6464	-0.06785	0.058508	-1.15961	0.246209	0.332601	AC006538	sense_intr
ENSG0000(	168.5556	0.167042	0.100477	1.662487	0.096415	0.149667	PAPPA2	protein_cc
ENSG0000(	168.5055	0.17802	0.103633	1.717797	0.085834	0.135284	DRD2	protein_cc
ENSG0000(	168.4774	-0.67139	0.103753	-6.47107	9.73E-11	7.43E-10	ARHGAP2	protein_cc
ENSG0000(	168.3913	0.212149	0.058182	3.646291	0.000266	0.000722	ZNF114	protein_cc
ENSG0000(	168.3901	-0.10951	0.128431	-0.85265	0.393856	0.4904	HSD11B1	protein_cc
ENSG0000(	168.3357	-0.23227	0.104936	-2.21344	0.026868	0.048585	PKD1P5	pseudoge
ENSG0000(	168.2427	0.053256	0.057351	0.928594	0.3531	0.448646	ARMC2	protein_cc
ENSG0000(	168.1514	0.346959	0.052284	6.636071	3.22E-11	2.65E-10	RP11-611	antisense
ENSG0000(	168.1204	-0.02411	0.07305	-0.33007	0.74135	0.80469	GRTP1	protein_cc
ENSG0000(	168.0669	-0.71761	0.117095	-6.12847	8.87E-10	5.84E-09	PRSS3	protein_cc
ENSG0000(	168.0156	-0.2638	0.045668	-5.77651	7.63E-09	4.31E-08	IGSF22	protein_cc

ENSG0000(	167.9785	-0.21318	0.125558	-1.69786	0.089534	0.140406	STRA6	protein_cc
ENSG0000(	167.9414	0.064099	0.097184	0.659565	0.509533	0.602838	ENPP1	protein_cc
ENSG0000(	167.8701	-0.23292	0.10252	-2.27192	0.023091	0.042406	PTGES	protein_cc
ENSG0000(	167.6896	0.010245	0.148802	0.068849	0.94511	0.961714	BDKRB2	protein_cc
ENSG0000(	167.6413	0.697654	0.106144	6.572699	4.94E-11	3.94E-10	SPATA17	protein_cc
ENSG0000(	167.5658	0.10022	0.069398	1.444138	0.1487	0.217157	EGFL8	protein_cc
ENSG0000(	167.0372	0.153138	0.060403	2.535292	0.011235	0.022195	TBX6	protein_cc
ENSG0000(	167.0086	0.342429	0.055719	6.145642	7.96E-10	5.27E-09	KB-1208A	antisense
ENSG0000(	166.8208	-0.03997	0.043036	-0.92881	0.352987	0.448556	RP11-613	antisense
ENSG0000(	166.7942	-0.2811	0.063724	-4.41125	1.03E-05	3.54E-05	HTR7P1	pseudoge
ENSG0000(	166.6929	-0.00104	0.068906	-0.01503	0.988011	0.991749	MROH6	protein_cc
ENSG0000(	166.6772	-0.58887	0.09225	-6.38341	1.73E-10	1.27E-09	RP11-64B	pseudoge
ENSG0000(	166.5686	-0.67167	0.150103	-4.47474	7.65E-06	2.69E-05	REM1	protein_cc
ENSG0000(	166.4771	-0.15948	0.073247	-2.17725	0.029462	0.052795	RP11-285	antisense
ENSG0000(	166.4325	-1.2309	0.117382	-10.4863	9.99E-26	1.02E-23	PCDHGA5	protein_cc
ENSG0000(	166.3486	-0.38295	0.09492	-4.03442	5.47E-05	0.000167	RP11-108	pseudoge
ENSG0000(	166.2633	-0.02003	0.047767	-0.41928	0.675015	0.749562	RP3-508I1	antisense
ENSG0000(	166.2446	-0.01735	0.073469	-0.23609	0.813364	0.862949	IL15RA	protein_cc
ENSG0000(	166.2071	-0.08324	0.034685	-2.39984	0.016402	0.0312	RP11-3J1(	pseudoge
ENSG0000(	166.204	0.038967	0.045695	0.852753	0.393796	0.49034	RP11-225	lincRNA
ENSG0000(	165.9478	0.093672	0.041679	2.247458	0.024611	0.044896	ZNF792	protein_cc
ENSG0000(	165.817	0.096294	0.056573	1.702104	0.088736	0.139308	ZNF699	protein_cc
ENSG0000(	165.5584	-0.31111	0.144653	-2.15075	0.031496	0.05603	CYP4F3	protein_cc
ENSG0000(	165.3627	0.512272	0.051393	9.967769	2.11E-23	1.37E-21	AC026271	pseudoge
ENSG0000(	165.3018	-0.21236	0.065892	-3.22295	0.001269	0.003035	ZNF192P1	pseudoge
ENSG0000(	165.1664	0.443556	0.062344	7.114705	1.12E-12	1.16E-11	ZNF850	protein_cc
ENSG0000(	165.1374	0.361828	0.070852	5.106781	3.28E-07	1.43E-06	ABCB4	protein_cc
ENSG0000(	165.1172	0.346938	0.045473	7.629466	2.36E-14	3.19E-13	GTF2H2C	protein_cc
ENSG0000(	165.1069	-0.39156	0.098588	-3.97166	7.14E-05	0.000214	SLC51B	protein_cc
ENSG0000(	165.0415	-0.12283	0.086699	-1.41677	0.156549	0.226879	KCNK13	protein_cc
ENSG0000(	164.9118	0.080584	0.051939	1.551499	0.120782	0.18167	RP5-890C	sense_intr
ENSG0000(	164.8464	0.073849	0.079024	0.934512	0.35004	0.44546	LY75	protein_cc
ENSG0000(	164.6193	0.377766	0.03798	9.94633	2.62E-23	1.65E-21	ZNF799	protein_cc
ENSG0000(	164.4927	-0.33509	0.078081	-4.29153	1.77E-05	5.89E-05	RP11-594	sense_ove
ENSG0000(	164.4437	-0.87354	0.114905	-7.60234	2.91E-14	3.87E-13	CLEC9A	protein_cc
ENSG0000(	164.2441	-0.25198	0.115407	-2.18341	0.029005	0.052059	GDNF	protein_cc
ENSG0000(	164.2411	0.388122	0.069267	5.603277	2.10E-08	1.11E-07	RP11-793	protein_cc
ENSG0000(	164.2266	0.729887	0.092542	7.887103	3.09E-15	4.93E-14	NPM1P27	pseudoge
ENSG0000(	164.1345	0.31322	0.075276	4.160954	3.17E-05	0.000101	HELB	protein_cc
ENSG0000(	164.0899	0.277771	0.049019	5.666587	1.46E-08	7.87E-08	IPO4	protein_cc
ENSG0000(	164.0279	0.256553	0.108088	2.373563	0.017617	0.033203	LINC0017(	lincRNA
ENSG0000(	163.9345	0.266431	0.047144	5.651419	1.59E-08	8.56E-08	RP4-758J1	lincRNA
ENSG0000(	163.6713	0.256846	0.040056	6.412186	1.43E-10	1.07E-09	ZNF442	protein_cc
ENSG0000(	163.6272	-0.36939	0.06466	-5.71277	1.11E-08	6.12E-08	CTD-2377	antisense
ENSG0000(	163.4678	0.742988	0.128662	5.774741	7.71E-09	4.36E-08	EN2	protein_cc
ENSG0000(	163.4655	0.230393	0.054603	4.219403	2.45E-05	7.92E-05	GPR35	protein_cc
ENSG0000(	163.4319	0.462523	0.048668	9.503543	2.03E-21	8.92E-20	PMS2P3	pseudoge
ENSG0000(	163.4287	0.087668	0.058516	1.498194	0.134083	0.198589	TIGD3	protein_cc
ENSG0000(	163.2096	0.12794	0.030272	4.226298	2.38E-05	7.71E-05	UGDH-AS	antisense
ENSG0000(	163.1569	-1.48535	0.129615	-11.4597	2.10E-30	5.37E-28	MTUS2	protein_cc
ENSG0000(	163.1448	0.27095	0.103568	2.616159	0.008893	0.017972	ANKRD45	protein_cc
ENSG0000(	163.1371	-0.11882	0.07899	-1.50423	0.132523	0.196655	RP11-121	pseudoge
ENSG0000(	162.9079	0.145032	0.045275	3.203348	0.001358	0.003232	RP11-495	lincRNA
ENSG0000(	162.8908	0.983193	0.12663	7.764327	8.21E-15	1.22E-13	TPI1P1	pseudoge
ENSG0000(	162.7575	0.36924	0.070396	5.245206	1.56E-07	7.16E-07	CCDC18	protein_cc
ENSG0000(	162.757	0.293586	0.091227	3.218184	0.00129	0.003082	SMN2	protein_cc
ENSG0000(	162.6179	0.968634	0.085155	11.37501	5.57E-30	1.24E-27	PARPBP	protein_cc
ENSG0000(	162.6147	-0.6161	0.086782	-7.09936	1.25E-12	1.29E-11	HCG11	lincRNA
ENSG0000(	162.5942	0.388267	0.111122	3.494046	0.000476	0.001233	C12orf55	protein_cc

ENSG0000(	162.4243	-0.02984	0.042466	-0.70277	0.482197	0.577298	RP11-522	antisense
ENSG0000(	162.3986	0.956673	0.095802	9.985936	1.76E-23	1.15E-21	CD101	protein_cc
ENSG0000(	162.2198	0.26804	0.064652	4.14589	3.38E-05	0.000107	LINC0092(	lincRNA
ENSG0000(	162.0868	0.52612	0.194286	2.70796	0.00677	0.014033	TRPM8	protein_cc
ENSG0000(	162.075	-0.01152	0.039713	-0.29005	0.771781	0.829228	SPATA24	protein_cc
ENSG0000(	161.7917	-0.01067	0.083722	-0.12744	0.898591	0.928087	RP11-526	lincRNA
ENSG0000(	161.7321	-0.10162	0.07503	-1.35434	0.175626	0.250154	CCDC180	protein_cc
ENSG0000(	161.7298	-0.12106	0.046368	-2.61074	0.009035	0.018229	RP11-337	antisense
ENSG0000(	161.7195	-0.20663	0.069961	-2.95357	0.003141	0.006974	RP11-490	lincRNA
ENSG0000(	161.6592	-1.06473	0.145676	-7.30892	2.69E-13	3.05E-12	NDST3	protein_cc
ENSG0000(	161.6499	-0.02292	0.051016	-0.44929	0.653223	0.731348	ZNF454	protein_cc
ENSG0000(	161.6441	0.300934	0.065594	4.587793	4.48E-06	1.63E-05	RP5-1159	lincRNA
ENSG0000(	161.6295	-0.02442	0.055669	-0.43874	0.660847	0.737702	KCNJ14	protein_cc
ENSG0000(	161.5818	-0.0659	0.067304	-0.97916	0.327502	0.42183	FBXO15	protein_cc
ENSG0000(	161.5723	-0.50243	0.070239	-7.1531	8.48E-13	8.93E-12	RP11-384	lincRNA
ENSG0000(	161.4357	0.18018	0.079789	2.258213	0.023932	0.043777	CMAHP	pseudoge
ENSG0000(	161.2436	0.112747	0.060817	1.853886	0.063755	0.104461	RP11-98I(	antisense
ENSG0000(	161.2149	0.034595	0.084947	0.407258	0.683818	0.756968	GCH1	protein_cc
ENSG0000(	161.1791	0.30939	0.045181	6.847829	7.50E-12	6.80E-11	CTD-2162	antisense
ENSG0000(	161.0783	-1.10177	0.138656	-7.94607	1.93E-15	3.17E-14	GRM7	protein_cc
ENSG0000(	161.0319	0.318265	0.103051	3.088419	0.002012	0.00464	ROR1	protein_cc
ENSG0000(	160.8787	-1.41678	0.1462	-9.69069	3.30E-22	1.66E-20	WNT10B	protein_cc
ENSG0000(	160.7692	0.420008	0.086109	4.877628	1.07E-06	4.32E-06	PIWIL4	protein_cc
ENSG0000(	160.7466	-0.13067	0.091768	-1.42394	0.154464	0.22441	CHSY3	protein_cc
ENSG0000(	160.7158	-1.53964	0.160784	-9.57579	1.01E-21	4.64E-20	KCNH5	protein_cc
ENSG0000(	160.4655	0.625226	0.057657	10.84381	2.13E-27	2.93E-25	KMT2E-A(	antisense
ENSG0000(	160.4609	0.003009	0.058868	0.051118	0.959232	0.972139	FAM90A1	protein_cc
ENSG0000(	160.3732	1.065166	0.12133	8.779081	1.65E-18	4.42E-17	CDCA2	protein_cc
ENSG0000(	160.3535	0.460852	0.115007	4.007176	6.14E-05	0.000186	ART3	protein_cc
ENSG0000(	160.3306	-0.28965	0.068878	-4.20532	2.61E-05	8.40E-05	RP11-218	antisense
ENSG0000(	160.3021	0.437064	0.063198	6.915738	4.65E-12	4.36E-11	RP11-440	lincRNA
ENSG0000(	160.2106	-1.07098	0.132666	-8.07276	6.87E-16	1.22E-14	KHDRBS2	protein_cc
ENSG0000(	159.6935	-0.30375	0.07437	-4.08433	4.42E-05	0.000137	BAIAP2L2	protein_cc
ENSG0000(	159.6282	-0.05235	0.051742	-1.01177	0.31165	0.404925	RP11-461	lincRNA
ENSG0000(	159.6174	-0.97817	0.124098	-7.88224	3.22E-15	5.11E-14	FEZF2	protein_cc
ENSG0000(	159.5866	-0.43174	0.105227	-4.10292	4.08E-05	0.000127	RP11-383	protein_cc
ENSG0000(	159.3991	-0.36872	0.092448	-3.98839	6.65E-05	0.000201	RP11-90P	lincRNA
ENSG0000(	159.3655	-0.43702	0.071486	-6.11337	9.76E-10	6.37E-09	GLT8D2	protein_cc
ENSG0000(	159.2179	0.73687	0.107457	6.857328	7.02E-12	6.39E-11	MT1L	pseudoge
ENSG0000(	159.0818	0.949632	0.164731	5.764741	8.18E-09	4.60E-08	ANXA2P2	pseudoge
ENSG0000(	159.0631	-0.33005	0.076955	-4.28887	1.80E-05	5.96E-05	ST6GALN/	protein_cc
ENSG0000(	158.9039	0.261417	0.05735	4.558268	5.16E-06	1.86E-05	CAPN10-/	antisense
ENSG0000(	158.8577	-0.25335	0.080035	-3.16546	0.001548	0.003648	RP11-127	lincRNA
ENSG0000(	158.7157	-0.08567	0.037757	-2.26909	0.023263	0.042678	BMS1P20	processed
ENSG0000(	158.6917	-0.0633	0.072415	-0.87411	0.382056	0.478664	HSD17B7F	pseudoge
ENSG0000(	158.5313	-0.14497	0.063722	-2.27505	0.022903	0.042089	NME9	protein_cc
ENSG0000(	158.4981	-0.33891	0.146934	-2.30652	0.021082	0.039049	OSR1	protein_cc
ENSG0000(	158.4711	-0.16688	0.080693	-2.06809	0.038632	0.067286	TRAF3IP3	protein_cc
ENSG0000(	158.4646	0.007288	0.130229	0.05596	0.955374	0.969184	TKTL1	protein_cc
ENSG0000(	158.3921	0.108308	0.035545	3.047089	0.002311	0.005269	ANKHD1-	protein_cc
ENSG0000(	158.2326	0.138605	0.051333	2.700138	0.006931	0.014336	ALKBH6	protein_cc
ENSG0000(	158.2287	-0.21726	0.090406	-2.40315	0.016254	0.030955	TMEM52B	protein_cc
ENSG0000(	157.8288	-0.23709	0.054616	-4.34111	1.42E-05	4.78E-05	SLC35E2	protein_cc
ENSG0000(	157.7773	-0.34526	0.063333	-5.45143	5.00E-08	2.48E-07	PROX1-A(	antisense
ENSG0000(	157.7747	0.143404	0.037785	3.795283	0.000147	0.000418	CTC-559E	sense_intr
ENSG0000(	157.5807	0.905764	0.203648	4.447682	8.68E-06	3.02E-05	TBX5	protein_cc
ENSG0000(	157.4265	-1.43309	0.163909	-8.74323	2.27E-18	6.00E-17	SERTM1	protein_cc
ENSG0000(	157.3978	0.746318	0.126405	5.904183	3.54E-09	2.12E-08	FAM181A-	processed
ENSG0000(	157.3015	1.423735	0.12909	11.02902	2.77E-28	4.48E-26	CENPA	protein_cc

ENSG0000(	157.2935	0.29235	0.089994	3.248548	0.00116	0.002791	BST1	protein_cc
ENSG0000(	157.292	0.436474	0.068199	6.400016	1.55E-10	1.15E-09	STAG3L5P	processed
ENSG0000(	156.9963	0.168575	0.100981	1.669378	0.095043	0.147832	IL2RG	protein_cc
ENSG0000(	156.9333	0.166141	0.038711	4.291837	1.77E-05	5.88E-05	MDP1	protein_cc
ENSG0000(	156.7973	0.005296	0.059523	0.088968	0.929107	0.950195	DEPDC7	protein_cc
ENSG0000(	156.7203	0.154001	0.079256	1.94307	0.052008	0.087493	ARMC12	protein_cc
ENSG0000(	156.719	0.089663	0.073799	1.214962	0.22438	0.307601	AC253572	protein_cc
ENSG0000(	156.6415	-0.04493	0.126998	-0.35382	0.723474	0.7896	CNTN6	protein_cc
ENSG0000(	156.4582	1.069439	0.146789	7.285564	3.20E-13	3.58E-12	RPS3AP5	pseudoge
ENSG0000(	156.4372	-0.09635	0.068991	-1.39655	0.162548	0.234195	AHRR	protein_cc
ENSG0000(	156.332	0.327969	0.067035	4.89251	9.96E-07	4.03E-06	C16orf71	protein_cc
ENSG0000(	156.2668	-0.2823	0.087881	-3.21226	0.001317	0.003142	LINC0114:	lincRNA
ENSG0000(	155.8577	-0.17391	0.094083	-1.84844	0.064539	0.105569	SPTBN5	protein_cc
ENSG0000(	155.8396	0.23946	0.052549	4.556853	5.19E-06	1.88E-05	CTD-2287	antisense
ENSG0000(	155.7908	0.157901	0.051085	3.090964	0.001995	0.004604	RP11-408	pseudoge
ENSG0000(	155.7832	-0.26335	0.101067	-2.60573	0.009168	0.018466	CHAC1	protein_cc
ENSG0000(	155.7363	-0.26455	0.146736	-1.80289	0.071406	0.115283	RP11-809	antisense
ENSG0000(	155.626	-0.70704	0.113633	-6.22209	4.91E-10	3.35E-09	GRIP1	protein_cc
ENSG0000(	155.3806	-1.39971	0.149873	-9.33934	9.69E-21	3.73E-19	RXFP1	protein_cc
ENSG0000(	155.3221	-0.43451	0.090442	-4.80427	1.55E-06	6.09E-06	SLC13A4	protein_cc
ENSG0000(	155.2261	1.148894	0.111685	10.28692	8.07E-25	6.83E-23	KIF18A	protein_cc
ENSG0000(	155.0856	0.639523	0.064714	9.88226	4.97E-23	2.93E-21	C17orf53	protein_cc
ENSG0000(	154.9209	0.037386	0.076733	0.487224	0.6261	0.708016	BCO2	protein_cc
ENSG0000(	154.7505	-0.00949	0.072449	-0.13101	0.895767	0.925984	CCDC11	protein_cc
ENSG0000(	154.7181	0.20683	0.056329	3.671802	0.000241	0.000659	KLRAP1	pseudoge
ENSG0000(	154.6484	0.137915	0.13678	1.008293	0.313314	0.406714	PODNL1	protein_cc
ENSG0000(	154.5543	-0.01767	0.086775	-0.2036	0.838663	0.882617	TEKT4P2	pseudoge
ENSG0000(	154.5423	-0.32485	0.121498	-2.67374	0.007501	0.015414	GPC3	protein_cc
ENSG0000(	154.491	-0.17749	0.065652	-2.70357	0.00686	0.014201	DMGDH	protein_cc
ENSG0000(	154.4355	-0.08894	0.060417	-1.47208	0.141	0.207371	LRRC56	protein_cc
ENSG0000(	154.3145	0.001456	0.121877	0.011947	0.990468	0.993512	GABRE	protein_cc
ENSG0000(	154.2449	-0.47713	0.081927	-5.82382	5.75E-09	3.31E-08	CABYR	protein_cc
ENSG0000(	154.2106	1.072072	0.126055	8.504792	1.82E-17	4.18E-16	NOX4	protein_cc
ENSG0000(	154.1117	0.104166	0.043416	2.399278	0.016427	0.03124	AC079922	lincRNA
ENSG0000(	153.9081	-0.42441	0.169469	-2.50432	0.012269	0.02401	SLC17A6	protein_cc
ENSG0000(	153.8099	-0.0687	0.082574	-0.83192	0.405452	0.502353	PSTPIP2	protein_cc
ENSG0000(	153.7435	0.540252	0.105303	5.130464	2.89E-07	1.27E-06	ZP3	protein_cc
ENSG0000(	153.7067	0.354658	0.088176	4.022162	5.77E-05	0.000176	KANSL1-A	antisense
ENSG0000(	153.6931	-0.32119	0.045833	-7.0078	2.42E-12	2.37E-11	RP11-378.	sense_ove
ENSG0000(	153.5808	-0.01179	0.09966	-0.11829	0.905835	0.933287	LINC0096(	lincRNA
ENSG0000(	153.5788	-0.01758	0.040388	-0.4352	0.663415	0.740039	RP11-53C	antisense
ENSG0000(	153.5484	0.320754	0.109797	2.921339	0.003485	0.00767	FAM132B	protein_cc
ENSG0000(	153.5134	0.513007	0.115872	4.427352	9.54E-06	3.30E-05	FTH1P8	pseudoge
ENSG0000(	153.422	0.250651	0.07957	3.150068	0.001632	0.003829	ZSCAN12I	pseudoge
ENSG0000(	153.2527	-0.27438	0.076139	-3.6037	0.000314	0.000841	IL12RB1	protein_cc
ENSG0000(	153.0127	0.103096	0.080181	1.285791	0.198516	0.277434	CCRL2	protein_cc
ENSG0000(	153.0125	-0.21921	0.043448	-5.04547	4.52E-07	1.93E-06	RP11-809	lincRNA
ENSG0000(	152.7535	-0.37018	0.101714	-3.63939	0.000273	0.000741	LINC0040:	lincRNA
ENSG0000(	152.703	0.591109	0.090282	6.547378	5.86E-11	4.62E-10	GGACT	protein_cc
ENSG0000(	152.681	-1.06069	0.143617	-7.38557	1.52E-13	1.79E-12	CDH7	protein_cc
ENSG0000(	152.6488	0.039926	0.068462	0.58318	0.559772	0.648708	NME2	protein_cc
ENSG0000(	152.5988	-0.25176	0.080152	-3.14107	0.001683	0.003939	VWA2	protein_cc
ENSG0000(	152.55	0.22159	0.040699	5.444622	5.19E-08	2.57E-07	RP5-1103	antisense
ENSG0000(	152.537	0.368301	0.091594	4.021027	5.79E-05	0.000176	MICB	protein_cc
ENSG0000(	152.4897	-0.33713	0.085686	-3.9345	8.34E-05	0.000247	GPR132	protein_cc
ENSG0000(	152.4582	0.216905	0.041776	5.192146	2.08E-07	9.36E-07	ARIH2OS	protein_cc
ENSG0000(	152.4144	0.059374	0.084259	0.704659	0.481022	0.576335	CRIP3	protein_cc
ENSG0000(	152.3407	0.312626	0.053754	5.815899	6.03E-09	3.46E-08	ARL6IP4	protein_cc
ENSG0000(	152.2283	0.279264	0.09295	3.004454	0.002661	0.005995	MYOZ1	protein_cc

ENSG0000(	152.0797	0.071417	0.075873	0.941268	0.346568	0.44204	ZC2HC1C	protein_cc
ENSG0000(	151.9851	0.529051	0.149193	3.546085	0.000391	0.00103	DLX5	protein_cc
ENSG0000(	151.9726	-0.00457	0.051216	-0.08932	0.928825	0.949962	RP3-449C	pseudoge
ENSG0000(	151.7828	-0.1323	0.060067	-2.20248	0.027631	0.049807	RP5-1042	sense_ove
ENSG0000(	151.7211	0.262748	0.071973	3.650659	0.000262	0.000711	AC018766	antisense
ENSG0000(	151.6365	0.66124	0.086069	7.682664	1.56E-14	2.18E-13	RP11-5C2	antisense
ENSG0000(	151.523	-0.6651	0.098638	-6.74283	1.55E-11	1.34E-10	CYP7B1	protein_cc
ENSG0000(	151.4404	1.269704	0.143359	8.8568	8.23E-19	2.32E-17	TMSB4XP4	pseudoge
ENSG0000(	151.4301	-0.11809	0.071679	-1.64752	0.099451	0.153827	NLRC4	protein_cc
ENSG0000(	151.3429	-0.13559	0.119822	-1.13158	0.25781	0.345732	ABCA6	protein_cc
ENSG0000(	151.3028	-0.01363	0.075651	-0.18021	0.856991	0.896721	ATP8B1	protein_cc
ENSG0000(	151.2964	0.162248	0.059626	2.721084	0.006507	0.013534	GDPD3	protein_cc
ENSG0000(	151.2724	-1.0111	0.124134	-8.14527	3.78E-16	7.00E-15	MUM1L1	protein_cc
ENSG0000(	151.1429	0.315819	0.123063	2.56633	0.010278	0.020472	MPZL2	protein_cc
ENSG0000(	151.1216	-0.1573	0.135229	-1.16319	0.244754	0.330964	IL7R	protein_cc
ENSG0000(	150.9386	0.742622	0.174871	4.246698	2.17E-05	7.09E-05	CTD-2328	pseudoge
ENSG0000(	150.8924	0.037628	0.068173	0.551951	0.580982	0.667861	RP11-163	lincRNA
ENSG0000(	150.8808	-0.15631	0.058019	-2.69404	0.007059	0.014572	ZFP69B	protein_cc
ENSG0000(	150.8436	0.231248	0.075138	3.077657	0.002086	0.004799	SERPINF2	protein_cc
ENSG0000(	150.8251	0.184299	0.055202	3.33863	0.000842	0.002081	RP5-894A	antisense
ENSG0000(	150.6359	0.143737	0.076408	1.881186	0.059947	0.09899	RP11-572	pseudoge
ENSG0000(	150.6306	-2.13594	0.200322	-10.6625	1.52E-26	1.82E-24	NEUROD6	protein_cc
ENSG0000(	150.5235	-0.3406	0.123004	-2.76904	0.005622	0.01185	SYTL5	protein_cc
ENSG0000(	150.3876	0.067169	0.068714	0.97752	0.328312	0.422741	MPZ	protein_cc
ENSG0000(	150.1584	-0.36867	0.13457	-2.73962	0.006151	0.012857	IL21R	protein_cc
ENSG0000(	150.077	-0.0112	0.04991	-0.22448	0.822387	0.869875	CTD-2015	antisense
ENSG0000(	149.7869	-0.46146	0.106718	-4.32409	1.53E-05	5.13E-05	RP11-797	pseudoge
ENSG0000(	149.7677	0.364261	0.042213	8.629119	6.18E-18	1.53E-16	CTC-534A	protein_cc
ENSG0000(	149.702	0.286921	0.071394	4.01885	5.85E-05	0.000178	FBXL19-A	antisense
ENSG0000(	149.6988	0.161949	0.087808	1.844349	0.065132	0.106391	BCMO1	protein_cc
ENSG0000(	149.6346	-0.15155	0.049407	-3.06733	0.00216	0.004951	RP5-1007	antisense
ENSG0000(	149.5229	0.018404	0.06942	0.265111	0.790924	0.844918	RAB39A	protein_cc
ENSG0000(	149.5142	-0.1246	0.079615	-1.56498	0.117588	0.177646	GPR160	protein_cc
ENSG0000(	149.3435	-0.09368	0.088682	-1.05639	0.290791	0.382267	KIAA1875	protein_cc
ENSG0000(	149.2628	0.628028	0.12626	4.974088	6.56E-07	2.73E-06	FOLR1	protein_cc
ENSG0000(	149.2057	-0.32885	0.059115	-5.56286	2.65E-08	1.38E-07	HSD17B1F	pseudoge
ENSG0000(	149.1219	-0.02274	0.085215	-0.26688	0.789565	0.843773	SLC17A9	protein_cc
ENSG0000(	148.9101	-0.15382	0.075771	-2.03003	0.042354	0.072962	RHBDL2	protein_cc
ENSG0000(	148.8865	0.028776	0.040067	0.718199	0.472634	0.568488	TMEM110	protein_cc
ENSG0000(	148.8742	0.058297	0.074362	0.783959	0.433064	0.530055	SLC2A9	protein_cc
ENSG0000(	148.839	-0.42607	0.06921	-6.1562	7.45E-10	4.96E-09	ADPRHL1	protein_cc
ENSG0000(	148.7614	0.746788	0.137026	5.449975	5.04E-08	2.50E-07	MTND1P2	pseudoge
ENSG0000(	148.734	0.59734	0.103255	5.785109	7.25E-09	4.11E-08	OLFML1	protein_cc
ENSG0000(	148.7184	-0.6659	0.093991	-7.08476	1.39E-12	1.42E-11	SLC6A13	protein_cc
ENSG0000(	148.6932	0.748582	0.130331	5.743715	9.26E-09	5.16E-08	TMEM71	protein_cc
ENSG0000(	148.5274	-0.44971	0.072836	-6.17421	6.65E-10	4.46E-09	RNF32	protein_cc
ENSG0000(	148.4612	-0.01074	0.141529	-0.07587	0.939519	0.957826	DAPL1	protein_cc
ENSG0000(	148.3963	-0.11478	0.038211	-3.00392	0.002665	0.006004	NRL	protein_cc
ENSG0000(	148.2873	0.010297	0.029914	0.344231	0.730673	0.795725	FBXO48	protein_cc
ENSG0000(	148.2149	0.228487	0.118931	1.921172	0.05471	0.091433	IL4I1	protein_cc
ENSG0000(	148.181	-0.54695	0.187447	-2.91787	0.003524	0.007747	PAX1	protein_cc
ENSG0000(	148.131	-0.99315	0.134864	-7.36409	1.78E-13	2.08E-12	GREM2	protein_cc
ENSG0000(	148.1199	0.303697	0.101272	2.998828	0.00271	0.006092	NCF1	protein_cc
ENSG0000(	147.9537	-0.46058	0.096673	-4.76427	1.90E-06	7.33E-06	EPHX4	protein_cc
ENSG0000(	147.9142	0.252892	0.135174	1.870868	0.061363	0.100989	RARRES1	protein_cc
ENSG0000(	147.8231	-0.1787	0.084504	-2.11473	0.034453	0.060763	IGFL4	protein_cc
ENSG0000(	147.7479	-0.76255	0.119691	-6.37094	1.88E-10	1.37E-09	CKMT1A	protein_cc
ENSG0000(	147.7298	0.349254	0.140494	2.485894	0.012923	0.025159	INMT	protein_cc
ENSG0000(	147.6969	-0.36298	0.096472	-3.76254	0.000168	0.000473	NPAS1	protein_cc

ENSG0000(	147.5711	-0.03008	0.079048	-0.3805	0.703573	0.773651	CTD-2636	antisense
ENSG0000(	147.4882	-0.21385	0.084964	-2.5169	0.011839	0.023262	CNKSR1	protein_cc
ENSG0000(	147.3947	0.579962	0.208111	2.78679	0.005323	0.011284	DSG2	protein_cc
ENSG0000(	147.3396	0.170189	0.04731	3.597282	0.000322	0.000861	FAM86JP	pseudoge
ENSG0000(	147.1986	0.353718	0.039845	8.877424	6.84E-19	1.95E-17	GPR89B	protein_cc
ENSG0000(	147.1795	0.067706	0.0584	1.15936	0.24631	0.332695	AC139100	protein_cc
ENSG0000(	146.9699	0.67131	0.135061	4.970432	6.68E-07	2.78E-06	IL20RA	protein_cc
ENSG0000(	146.8523	0.416737	0.082828	5.031328	4.87E-07	2.07E-06	MB21D1	protein_cc
ENSG0000(	146.8442	-0.12471	0.070706	-1.76375	0.077775	0.124239	PLS1	protein_cc
ENSG0000(	146.7429	0.146266	0.048712	3.002695	0.002676	0.006027	PARD6B	protein_cc
ENSG0000(	146.6648	-0.1971	0.12327	-1.59893	0.109837	0.167518	CNN1	protein_cc
ENSG0000(	146.5144	0.300643	0.05874	5.118196	3.08E-07	1.35E-06	RP5-827C	antisense
ENSG0000(	146.4771	0.611426	0.123077	4.967851	6.77E-07	2.81E-06	GNLY	protein_cc
ENSG0000(	146.3796	-0.13451	0.047784	-2.81486	0.00488	0.010417	CYB5D1	protein_cc
ENSG0000(	146.3635	0.452841	0.045584	9.934206	2.96E-23	1.83E-21	CTC-359D	pseudoge
ENSG0000(	146.2921	-0.14366	0.09556	-1.50334	0.132752	0.196923	MAPT-AS:	antisense
ENSG0000(	146.2678	0.409856	0.064576	6.346928	2.20E-10	1.58E-09	ZGLP1	protein_cc
ENSG0000(	146.2593	0.251111	0.051939	4.834692	1.33E-06	5.28E-06	AC016629	antisense
ENSG0000(	146.248	0.180366	0.044032	4.096238	4.20E-05	0.000131	C12orf60	protein_cc
ENSG0000(	145.9923	0.98168	0.132341	7.417812	1.19E-13	1.43E-12	RP11-119	antisense
ENSG0000(	145.7313	-0.2418	0.103326	-2.34018	0.019275	0.036015	DNAH6	protein_cc
ENSG0000(	145.7258	0.858894	0.104144	8.24719	1.62E-16	3.19E-15	CTB-63M2	pseudoge
ENSG0000(	145.604	-0.05558	0.058224	-0.95463	0.339765	0.434806	TOB1-AS1	processed
ENSG0000(	145.6028	1.158091	0.118039	9.811063	1.01E-22	5.58E-21	SGOL1	protein_cc
ENSG0000(	145.602	0.461211	0.06057	7.614496	2.65E-14	3.55E-13	RP11-66N	antisense
ENSG0000(	145.5103	0.04514	0.116332	0.388026	0.697997	0.76885	PLA2R1	protein_cc
ENSG0000(	145.4998	-0.56318	0.107497	-5.23898	1.61E-07	7.40E-07	PAPPA	protein_cc
ENSG0000(	145.4786	-0.37657	0.063875	-5.89549	3.74E-09	2.22E-08	ABCC6	protein_cc
ENSG0000(	145.4575	-0.03178	0.063851	-0.4977	0.618693	0.701415	SKOR1	protein_cc
ENSG0000(	145.406	0.922466	0.141147	6.535491	6.34E-11	4.98E-10	S100A3	protein_cc
ENSG0000(	145.3841	0.293771	0.033018	8.897289	5.72E-19	1.65E-17	RP11-422	lincRNA
ENSG0000(	145.3446	0.103824	0.056611	1.83399	0.066655	0.10856	RP6-109B	antisense
ENSG0000(	145.3124	0.032292	0.05147	0.627393	0.530402	0.622105	DFNB59	protein_cc
ENSG0000(	145.2657	-0.6487	0.088479	-7.33168	2.27E-13	2.60E-12	TRIM17	protein_cc
ENSG0000(	145.225	1.1261	0.214491	5.2501	1.52E-07	6.99E-07	HOXD9	protein_cc
ENSG0000(	145.1839	0.869254	0.078412	11.0857	1.47E-28	2.59E-26	POLE2	protein_cc
ENSG0000(	145.1614	-0.4983	0.048931	-10.1837	2.34E-24	1.81E-22	MBNL1-A	antisense
ENSG0000(	145.1198	0.42523	0.072127	5.895545	3.73E-09	2.22E-08	CTC-241F	antisense
ENSG0000(	145.0479	0.095054	0.122324	0.777074	0.437115	0.534124	CLEC4E	protein_cc
ENSG0000(	144.8554	0.026737	0.056375	0.474272	0.635306	0.716064	C15orf37	protein_cc
ENSG0000(	144.8012	0.024459	0.061277	0.39916	0.689775	0.762149	AGAP4	protein_cc
ENSG0000(	144.6744	0.472822	0.06374	7.417999	1.19E-13	1.43E-12	RAC1P2	pseudoge
ENSG0000(	144.5813	-0.3573	0.144638	-2.47031	0.013499	0.026183	ACTC1	protein_cc
ENSG0000(	144.4736	1.457587	0.151313	9.632935	5.80E-22	2.76E-20	HMGN2P5	pseudoge
ENSG0000(	144.4171	-0.13702	0.101857	-1.34518	0.178566	0.25363	AC144521	processed
ENSG0000(	144.374	-0.03364	0.090656	-0.37106	0.710592	0.77918	RP11-728	antisense
ENSG0000(	144.3538	-0.41469	0.058229	-7.12181	1.07E-12	1.10E-11	CTB-152G	antisense
ENSG0000(	144.2924	0.372867	0.070522	5.287228	1.24E-07	5.80E-07	MTFP1	protein_cc
ENSG0000(	144.1994	0.153576	0.039144	3.923405	8.73E-05	0.000258	C11orf48	protein_cc
ENSG0000(	144.1607	0.514281	0.184544	2.786764	0.005324	0.011284	DRC1	protein_cc
ENSG0000(	144.0763	-0.58963	0.085163	-6.9236	4.40E-12	4.14E-11	CRYBB1	protein_cc
ENSG0000(	144.0744	-0.94207	0.129492	-7.27511	3.46E-13	3.86E-12	SLC35F3	protein_cc
ENSG0000(	144.0165	-0.19032	0.071486	-2.66233	0.00776	0.015899	RP5-1021	antisense
ENSG0000(	144.0055	0.723115	0.071761	10.07678	7.00E-24	4.98E-22	RP11-303	sense_intr
ENSG0000(	143.9942	-0.04264	0.063364	-0.67288	0.501026	0.594947	RP11-228	antisense
ENSG0000(	143.9772	0.089917	0.065904	1.364366	0.172452	0.246278	TXNDC5	protein_cc
ENSG0000(	143.9724	-0.08187	0.049012	-1.67033	0.094855	0.147568	RP11-521	antisense
ENSG0000(	143.9378	0.14471	0.109195	1.325242	0.185091	0.261372	PDGFRL	protein_cc
ENSG0000(	143.9069	-0.74813	0.129235	-5.78888	7.09E-09	4.02E-08	CNTN5	protein_cc

ENSG0000(	143.904	1.775614	0.249691	7.111239	1.15E-12	1.18E-11	HOXD10	protein_cc
ENSG0000(	143.8852	-0.00912	0.064092	-0.14222	0.886908	0.919508	RP3-402G	antisense
ENSG0000(	143.7251	0.749717	0.081142	9.239517	2.48E-20	8.87E-19	CENPI	protein_cc
ENSG0000(	143.617	0.108275	0.039137	2.766591	0.005665	0.011933	KIAA1919	protein_cc
ENSG0000(	143.5818	0.095498	0.177665	0.537519	0.590909	0.676632	ZNF560	protein_cc
ENSG0000(	143.5543	0.219899	0.06198	3.547928	0.000388	0.001024	AP003774	protein_cc
ENSG0000(	143.4203	0.099837	0.150639	0.662757	0.507486	0.601108	LINC0064!	lincRNA
ENSG0000(	143.4181	0.268932	0.07745	3.472358	0.000516	0.001328	EFNA4	protein_cc
ENSG0000(	143.3992	0.119025	0.041263	2.884548	0.00392	0.008542	NBPF8	protein_cc
ENSG0000(	143.3074	0.142988	0.06178	2.314489	0.020641	0.038305	LA16c-35(	antisense
ENSG0000(	143.2139	0.039143	0.052453	0.746254	0.455514	0.552071	TMEM9B-	antisense
ENSG0000(	143.091	-1.58606	0.167282	-9.48134	2.51E-21	1.08E-19	KCNS2	protein_cc
ENSG0000(	143.0542	-0.22688	0.060322	-3.76112	0.000169	0.000475	LINC0098	lincRNA
ENSG0000(	143.0206	-0.18729	0.07399	-2.53135	0.011362	0.022413	GRHL1	protein_cc
ENSG0000(	142.9418	-0.3308	0.073129	-4.52351	6.08E-06	2.17E-05	RP11-66N	antisense
ENSG0000(	142.7879	-0.0639	0.101262	-0.631	0.528041	0.619743	VAC14-AS	antisense
ENSG0000(	142.6326	-0.00052	0.031509	-0.01645	0.986872	0.991062	RP3-475N	antisense
ENSG0000(	142.575	0.392255	0.110156	3.560895	0.00037	0.000978	LINC0090(	lincRNA
ENSG0000(	142.5615	-0.11062	0.072742	-1.52077	0.128317	0.191303	VSIG10L	protein_cc
ENSG0000(	142.5577	0.007976	0.165238	0.048271	0.9615	0.973622	AQP9	protein_cc
ENSG0000(	142.5229	0.007312	0.043282	0.168944	0.865841	0.903652	CTC-479C	protein_cc
ENSG0000(	142.5098	0.640138	0.120554	5.309968	1.10E-07	5.17E-07	KCNN4	protein_cc
ENSG0000(	142.4892	0.144655	0.04317	3.350812	0.000806	0.001998	CTD-2267	antisense
ENSG0000(	142.4871	0.775004	0.095731	8.095645	5.70E-16	1.02E-14	C4orf47	protein_cc
ENSG0000(	142.4616	-0.32694	0.09353	-3.49556	0.000473	0.001226	ENDOU	protein_cc
ENSG0000(	142.4476	-0.1868	0.056112	-3.32907	0.000871	0.002148	EFCAB6	protein_cc
ENSG0000(	142.4011	-0.47066	0.104459	-4.50564	6.62E-06	2.35E-05	RBP7	protein_cc
ENSG0000(	142.3961	0.37214	0.162711	2.28712	0.022189	0.04091	CXCL9	protein_cc
ENSG0000(	142.3958	0.407491	0.076152	5.351041	8.74E-08	4.18E-07	CCDC169	protein_cc
ENSG0000(	142.3655	-0.51824	0.114603	-4.52206	6.12E-06	2.19E-05	TDRD9	protein_cc
ENSG0000(	142.3387	-0.20181	0.070166	-2.87617	0.004025	0.008752	KIAA1024	protein_cc
ENSG0000(	142.2746	-0.34371	0.050872	-6.75645	1.41E-11	1.23E-10	LURAP1	protein_cc
ENSG0000(	142.2615	-1.3223	0.158295	-8.35342	6.63E-17	1.39E-15	GPR22	protein_cc
ENSG0000(	142.2099	0.543963	0.065085	8.357802	6.39E-17	1.34E-15	SRCRB4D	protein_cc
ENSG0000(	142.1886	0.224491	0.058955	3.807804	0.00014	0.000399	RP11-140	antisense
ENSG0000(	142.1439	0.796554	0.14253	5.588658	2.29E-08	1.20E-07	RP11-363	lincRNA
ENSG0000(	141.9212	-0.08189	0.088562	-0.92471	0.355115	0.450747	NOD2	protein_cc
ENSG0000(	141.8456	-0.52266	0.122946	-4.25114	2.13E-05	6.97E-05	KCNA5	protein_cc
ENSG0000(	141.7671	0.192691	0.036674	5.254101	1.49E-07	6.85E-07	MIR3654	protein_cc
ENSG0000(	141.752	-1.43674	0.177259	-8.10531	5.26E-16	9.50E-15	NGB	protein_cc
ENSG0000(	141.731	0.191225	0.060742	3.148145	0.001643	0.003852	RP11-308	processed
ENSG0000(	141.6951	0.562077	0.087656	6.412319	1.43E-10	1.06E-09	CENPW	protein_cc
ENSG0000(	141.6825	0.105671	0.09495	1.11291	0.265747	0.354583	KREMEN2	protein_cc
ENSG0000(	141.6782	-0.00633	0.046604	-0.13584	0.89195	0.923153	SPSB3	protein_cc
ENSG0000(	141.6774	-0.31906	0.065422	-4.87706	1.08E-06	4.33E-06	TSSK4	protein_cc
ENSG0000(	141.5952	-0.3161	0.082263	-3.84261	0.000122	0.000351	POMK	protein_cc
ENSG0000(	141.2449	-0.01026	0.039959	-0.25683	0.797308	0.850126	KTN1-AS1	antisense
ENSG0000(	141.2152	0.153572	0.038489	3.989987	6.61E-05	0.000199	ZNF410	protein_cc
ENSG0000(	141.1204	0.615588	0.065388	9.414365	4.76E-21	1.94E-19	C5orf34	protein_cc
ENSG0000(	141.0492	0.81557	0.134823	6.049191	1.46E-09	9.24E-09	C11orf63	protein_cc
ENSG0000(	141.0294	0.544287	0.125717	4.329462	1.49E-05	5.01E-05	AC068057	lincRNA
ENSG0000(	141.0195	1.270167	0.111407	11.40113	4.13E-30	9.50E-28	RP11-698	antisense
ENSG0000(	140.9896	0.403984	0.10226	3.950574	7.80E-05	0.000232	ZMYND12	protein_cc
ENSG0000(	140.9591	-0.77014	0.10398	-7.40655	1.30E-13	1.55E-12	ANXA3	protein_cc
ENSG0000(	140.9083	0.3982	0.127904	3.113271	0.00185	0.004295	PRF1	protein_cc
ENSG0000(	140.8978	0.019917	0.065973	0.301903	0.762726	0.821881	TNFSF4	protein_cc
ENSG0000(	140.8607	-0.11907	0.064388	-1.84921	0.064428	0.105424	CTA-963H	pseudoge
ENSG0000(	140.8386	0.235671	0.049643	4.7473	2.06E-06	7.92E-06	LIPE-AS1	antisense
ENSG0000(	140.7758	-0.66715	0.125701	-5.30741	1.11E-07	5.23E-07	GLIS1	protein_cc

ENSG0000(	140.731	0.285904	0.055901	5.11445	3.15E-07	1.38E-06	DNMT3B	protein_cc
ENSG0000(	140.7051	0.044496	0.075278	0.591086	0.554463	0.644044	DNASE1L2	protein_cc
ENSG0000(	140.5858	-0.26102	0.094604	-2.75911	0.005796	0.012182	LRRC37A6	pseudoge
ENSG0000(	140.5393	0.07395	0.131271	0.56334	0.573203	0.660551	MAGEE2	protein_cc
ENSG0000(	140.5112	0.308928	0.080204	3.851794	0.000117	0.000339	C9orf117	protein_cc
ENSG0000(	140.4324	-1.81072	0.204299	-8.86308	7.78E-19	2.20E-17	GPR26	protein_cc
ENSG0000(	140.3771	0.26557	0.122105	2.174922	0.029636	0.053068	GAPT	protein_cc
ENSG0000(	140.356	-0.48583	0.081339	-5.97293	2.33E-09	1.43E-08	RP11-750	antisense
ENSG0000(	140.352	0.482146	0.054273	8.883687	6.47E-19	1.85E-17	BORA	protein_cc
ENSG0000(	140.3487	-1.38423	0.134664	-10.2792	8.75E-25	7.39E-23	HOOK1	protein_cc
ENSG0000(	140.3476	-0.07054	0.051476	-1.37039	0.170566	0.244029	RP1-153G	lincRNA
ENSG0000(	140.3364	-0.01534	0.090303	-0.16992	0.865077	0.903021	ARL11	protein_cc
ENSG0000(	140.3226	0.130307	0.08259	1.577754	0.114622	0.173829	CCDC173	protein_cc
ENSG0000(	140.2862	0.753965	0.099096	7.608437	2.77E-14	3.71E-13	EEF1B2P3	pseudoge
ENSG0000(	140.2354	-0.99617	0.104619	-9.52188	1.70E-21	7.57E-20	GPR61	protein_cc
ENSG0000(	140.2047	-0.16954	0.092596	-1.83099	0.067102	0.109196	AC003102	antisense
ENSG0000(	140.1151	0.274635	0.195751	1.402981	0.160622	0.23181	RP4-765C	pseudoge
ENSG0000(	140.0767	0.128537	0.090311	1.423275	0.154657	0.224643	CASC1	protein_cc
ENSG0000(	140.0728	-0.31654	0.108141	-2.92714	0.003421	0.00754	GLB1L3	protein_cc
ENSG0000(	140.0415	-1.06637	0.142772	-7.46902	8.08E-14	9.99E-13	KCNK9	protein_cc
ENSG0000(	139.9983	0.040219	0.048357	0.831723	0.405565	0.502404	SRD5A3-A	processed
ENSG0000(	139.9785	-0.54949	0.063525	-8.65007	5.15E-18	1.29E-16	RP11-293	antisense
ENSG0000(	139.8947	0.07325	0.097096	0.754411	0.450603	0.547363	C21orf88	protein_cc
ENSG0000(	139.8793	0.285024	0.065172	4.373409	1.22E-05	4.16E-05	ZMYM6NB	protein_cc
ENSG0000(	139.7686	-0.39512	0.096429	-4.09754	4.18E-05	0.00013	PTGDR2	protein_cc
ENSG0000(	139.6398	-0.00706	0.04778	-0.14784	0.882469	0.916061	ZNF573	protein_cc
ENSG0000(	139.6207	0.388324	0.066589	5.831674	5.49E-09	3.17E-08	RP4-740C	processed
ENSG0000(	139.502	1.099165	0.118672	9.262197	2.00E-20	7.29E-19	CCER2	protein_cc
ENSG0000(	139.4744	-0.13036	0.076725	-1.69901	0.089317	0.140124	RP11-268.	protein_cc
ENSG0000(	139.2796	-0.04346	0.052251	-0.83175	0.405553	0.502404	XYLB	protein_cc
ENSG0000(	139.2121	0.00129	0.067372	0.019141	0.984729	0.989541	RASA4	protein_cc
ENSG0000(	139.1802	0.061148	0.064984	0.940977	0.346716	0.442202	UNC5CL	protein_cc
ENSG0000(	139.1212	-0.06504	0.076182	-0.85378	0.393228	0.489855	HSF2BP	protein_cc
ENSG0000(	139.1173	0.048415	0.090747	0.53352	0.593674	0.679079	GRAMD2	protein_cc
ENSG0000(	139.0855	-0.62452	0.090013	-6.93813	3.97E-12	3.77E-11	MYL3	protein_cc
ENSG0000(	139.0621	0.291264	0.093828	3.104229	0.001908	0.004418	FTH1P7	pseudoge
ENSG0000(	139.062	-0.3823	0.066216	-5.77344	7.77E-09	4.38E-08	RALGAPA1	pseudoge
ENSG0000(	139.0236	-0.90336	0.141738	-6.37348	1.85E-10	1.35E-09	RP11-267.	lincRNA
ENSG0000(	138.9943	0.395016	0.071101	5.55566	2.77E-08	1.43E-07	IGFLR1	protein_cc
ENSG0000(	138.9354	-0.25243	0.083991	-3.00537	0.002653	0.005979	AC122129	pseudoge
ENSG0000(	138.9179	-0.02435	0.148972	-0.16348	0.870144	0.906809	RP11-275	lincRNA
ENSG0000(	138.8826	0.012448	0.051254	0.242874	0.808103	0.858719	RP11-477	antisense
ENSG0000(	138.8298	-0.27451	0.152714	-1.79755	0.072248	0.116497	TAC3	protein_cc
ENSG0000(	138.7895	-0.10384	0.072881	-1.42473	0.154235	0.224125	RNLS	protein_cc
ENSG0000(	138.7467	-0.45243	0.082826	-5.46249	4.70E-08	2.34E-07	ZIC3	protein_cc
ENSG0000(	138.7413	0.141128	0.104061	1.35621	0.175032	0.249455	KYNU	protein_cc
ENSG0000(	138.7342	0.079906	0.066707	1.197878	0.230965	0.314989	IGSF5	protein_cc
ENSG0000(	138.7296	0.212543	0.0551	3.857369	0.000115	0.000331	RP11-16N	antisense
ENSG0000(	138.7128	-1.00015	0.13778	-7.25898	3.90E-13	4.31E-12	TNFSF18	protein_cc
ENSG0000(	138.6567	-0.76576	0.115791	-6.61324	3.76E-11	3.06E-10	NPY1R	protein_cc
ENSG0000(	138.5923	0.165539	0.046856	3.532929	0.000411	0.001079	SARNP	protein_cc
ENSG0000(	138.3913	1.015061	0.200186	5.070599	3.97E-07	1.71E-06	RP11-343	pseudoge
ENSG0000(	138.3906	0.462287	0.140087	3.300007	0.000967	0.002361	FAM46B	protein_cc
ENSG0000(	138.29	-1.12668	0.110596	-10.1874	2.26E-24	1.75E-22	CYP4X1	protein_cc
ENSG0000(	138.188	0.211849	0.085304	2.483459	0.013011	0.025316	KIRREL2	protein_cc
ENSG0000(	138.1273	-0.05863	0.08858	-0.66189	0.508042	0.601571	FAM26E	protein_cc
ENSG0000(	138.0367	-0.07328	0.061021	-1.20088	0.229799	0.313775	LINC0047	lincRNA
ENSG0000(	137.9733	-0.22629	0.058769	-3.85058	0.000118	0.00034	RP11-101	lincRNA
ENSG0000(	137.96	-0.83843	0.112675	-7.44113	9.98E-14	1.21E-12	POPDC3	protein_cc

ENSG0000(	137.8801	0.064109	0.174576	0.367226	0.713451	0.781482	IGHM	IG_C_gene
ENSG0000(	137.8755	-0.9612	0.120242	-7.99388	1.31E-15	2.22E-14	PKP2	protein_cc
ENSG0000(	137.8467	0.060013	0.0679	0.883848	0.376778	0.473234	RP11-88H	lincRNA
ENSG0000(	137.6605	0.517286	0.137187	3.770659	0.000163	0.000459	PCDHB17	protein_cc
ENSG0000(	137.6052	0.197823	0.032288	6.126751	8.97E-10	5.89E-09	CDKL3	protein_cc
ENSG0000(	137.5968	-0.42809	0.211193	-2.02702	0.04266	0.073451	OTOG	protein_cc
ENSG0000(	137.5778	0.008324	0.102013	0.081594	0.93497	0.954608	LINGO3	protein_cc
ENSG0000(	137.5735	1.28903	0.157024	8.209139	2.23E-16	4.28E-15	RP4-792G	antisense
ENSG0000(	137.4646	0.800564	0.088574	9.038407	1.59E-19	5.04E-18	MAEL	protein_cc
ENSG0000(	137.4306	0.014694	0.070776	0.207608	0.835535	0.88025	ZNF663P	pseudoge
ENSG0000(	137.3694	0.488944	0.14103	3.466954	0.000526	0.001353	SLC7A3	protein_cc
ENSG0000(	137.1626	-0.22991	0.138831	-1.65602	0.097719	0.151422	RP11-314	lincRNA
ENSG0000(	137.116	1.269725	0.116554	10.89385	1.23E-27	1.76E-25	CDC25C	protein_cc
ENSG0000(	137.1066	-0.11874	0.053858	-2.20477	0.02747	0.049539	TCEA1P2	pseudoge
ENSG0000(	137.0994	-1.50078	0.155291	-9.66429	4.28E-22	2.10E-20	GABRG3	protein_cc
ENSG0000(	137.0796	0.048171	0.042304	1.138685	0.254835	0.342433	RP11-127	antisense
ENSG0000(	136.933	-0.0867	0.082415	-1.05201	0.292794	0.384397	PPARG	protein_cc
ENSG0000(	136.7998	0.088792	0.078119	1.136623	0.255696	0.343366	CES3	protein_cc
ENSG0000(	136.6814	-0.05268	0.048723	-1.08118	0.279616	0.370086	RP11-182	lincRNA
ENSG0000(	136.6789	0.329967	0.059953	5.503771	3.72E-08	1.89E-07	FAM3C2	pseudoge
ENSG0000(	136.6752	0.322366	0.09372	3.439666	0.000582	0.001485	MST1P2	pseudoge
ENSG0000(	136.6412	0.15868	0.064887	2.445497	0.014465	0.02789	AC016700	lincRNA
ENSG0000(	136.6115	-0.052	0.219484	-0.23692	0.812719	0.862465	IGHG2	IG_C_gene
ENSG0000(	136.5838	-0.15687	0.045863	-3.4203	0.000626	0.001584	C9orf96	protein_cc
ENSG0000(	136.2632	0.406229	0.068082	5.966746	2.42E-09	1.48E-08	PIH1D2	protein_cc
ENSG0000(	136.0047	0.315147	0.049537	6.361886	1.99E-10	1.45E-09	PRR19	protein_cc
ENSG0000(	135.9981	0.144378	0.062559	2.307863	0.021007	0.038917	ELMO3	protein_cc
ENSG0000(	135.8427	-0.16287	0.062635	-2.60039	0.009312	0.018729	CTA-390C	sense_intr
ENSG0000(	135.8076	-0.32137	0.075626	-4.24942	2.14E-05	7.01E-05	ZNF366	protein_cc
ENSG0000(	135.8045	-0.14765	0.081132	-1.8199	0.068774	0.111588	AC093724	pseudoge
ENSG0000(	135.7372	0.633788	0.065723	9.643245	5.25E-22	2.53E-20	ZNF90	protein_cc
ENSG0000(	135.6184	-1.19823	0.148536	-8.06694	7.21E-16	1.27E-14	GPR83	protein_cc
ENSG0000(	135.5721	0.297426	0.06439	4.619144	3.85E-06	1.42E-05	RP11-486	lincRNA
ENSG0000(	135.5671	-0.1542	0.104941	-1.46942	0.14172	0.20828	KBTBD12	protein_cc
ENSG0000(	135.5527	-0.17381	0.107775	-1.61275	0.106798	0.163507	IRF6	protein_cc
ENSG0000(	135.5501	0.415532	0.060719	6.843523	7.73E-12	6.98E-11	FAM154B	protein_cc
ENSG0000(	135.5279	-0.57131	0.149045	-3.83313	0.000127	0.000363	CLCA4	protein_cc
ENSG0000(	135.4784	-1.36045	0.130048	-10.4612	1.30E-25	1.29E-23	STAT4	protein_cc
ENSG0000(	135.4762	0.587314	0.116443	5.043808	4.56E-07	1.95E-06	ADRA1D	protein_cc
ENSG0000(	135.3995	-0.60608	0.087871	-6.89737	5.30E-12	4.90E-11	CTD-2292	antisense
ENSG0000(	135.3781	0.252242	0.085743	2.941828	0.003263	0.00722	NEK5	protein_cc
ENSG0000(	135.2362	0.043656	0.066036	0.661096	0.50855	0.602022	RP11-415.	antisense
ENSG0000(	135.1844	-1.16581	0.123466	-9.44235	3.65E-21	1.52E-19	CDKL2	protein_cc
ENSG0000(	135.1492	0.342317	0.064173	5.3343	9.59E-08	4.56E-07	RP5-1068	lincRNA
ENSG0000(	135.0336	0.161602	0.068778	2.349623	0.018792	0.035196	CLEC4A	protein_cc
ENSG0000(	135.0116	0.046913	0.110103	0.426085	0.670046	0.745293	RP11-11N	lincRNA
ENSG0000(	135.0011	-0.23191	0.10643	-2.17901	0.029331	0.052583	FAM178B	protein_cc
ENSG0000(	135.0009	0.242404	0.039545	6.129857	8.80E-10	5.79E-09	RP11-395.	lincRNA
ENSG0000(	134.8459	0.359385	0.0888	4.04712	5.19E-05	0.000159	CETP	protein_cc
ENSG0000(	134.7499	-1.39867	0.156762	-8.92228	4.57E-19	1.34E-17	LINC0090(	lincRNA
ENSG0000(	134.6895	-0.32669	0.047656	-6.85518	7.12E-12	6.48E-11	BCDIN3D-	antisense
ENSG0000(	134.6288	-0.21072	0.065157	-3.23398	0.001221	0.002929	RASA4CP	pseudoge
ENSG0000(	134.6044	0.379705	0.041719	9.101536	8.91E-20	2.93E-18	AC009403	protein_cc
ENSG0000(	134.5586	0.182636	0.078997	2.311939	0.020781	0.038542	ZNF214	protein_cc
ENSG0000(	134.4977	0.047958	0.042097	1.13921	0.254616	0.342174	RP11-752	antisense
ENSG0000(	134.4851	0.06285	0.152933	0.410966	0.681098	0.754463	PCDHA11	protein_cc
ENSG0000(	134.4812	-0.61155	0.103108	-5.93114	3.01E-09	1.82E-08	IL1RAPL1	protein_cc
ENSG0000(	134.4007	0.343944	0.04753	7.236325	4.61E-13	5.03E-12	CTD-2006	processed
ENSG0000(	134.3313	0.071355	0.059746	1.194316	0.232355	0.31656	AC007318	pseudoge

ENSG0000(	134.3168	0.302306	0.120347	2.511952	0.012007	0.023558	CD69	protein_cc
ENSG0000(	134.3066	-0.34918	0.09979	-3.49912	0.000467	0.001212	AC006129	lincRNA
ENSG0000(	134.2229	-0.15737	0.055013	-2.86069	0.004227	0.00915	GPR146	protein_cc
ENSG0000(	134.2132	-0.07515	0.099756	-0.75332	0.451256	0.547918	SNAP25-/	antisense
ENSG0000(	134.1917	0.081556	0.130223	0.626282	0.53113	0.622728	PCDHA2	protein_cc
ENSG0000(	134.1894	-0.17267	0.155476	-1.11056	0.266759	0.355737	GLRA2	protein_cc
ENSG0000(	134.1679	0.550829	0.094347	5.838315	5.27E-09	3.05E-08	PRICKLE3	protein_cc
ENSG0000(	134.1537	0.146172	0.128233	1.139892	0.254331	0.34188	MMP11	protein_cc
ENSG0000(	134.1384	0.124521	0.042652	2.919473	0.003506	0.007713	AC007228	antisense
ENSG0000(	134.081	0.298258	0.048111	6.199353	5.67E-10	3.83E-09	RP11-378.	antisense
ENSG0000(	134.0725	0.314502	0.066085	4.75906	1.94E-06	7.51E-06	LINC0113	antisense
ENSG0000(	134.0015	0.255933	0.079617	3.214555	0.001306	0.003118	CD99P1	pseudoge
ENSG0000(	133.9726	-0.48689	0.166108	-2.93116	0.003377	0.00745	ARMC3	protein_cc
ENSG0000(	133.8782	-0.2309	0.058437	-3.95122	7.78E-05	0.000232	RP11-266	lincRNA
ENSG0000(	133.8774	0.114327	0.058643	1.94955	0.05123	0.086322	FBXO16	protein_cc
ENSG0000(	133.8682	-0.12269	0.102453	-1.19748	0.231121	0.315161	MAMDC2	protein_cc
ENSG0000(	133.7006	-0.70982	0.09248	-7.67539	1.65E-14	2.29E-13	TLL2	protein_cc
ENSG0000(	133.6633	-0.81507	0.104894	-7.77039	7.82E-15	1.17E-13	CHRM4	protein_cc
ENSG0000(	133.3971	0.263211	0.066813	3.939544	8.16E-05	0.000243	AF001548	antisense
ENSG0000(	133.2854	1.607134	0.186064	8.637543	5.74E-18	1.43E-16	AQP5	protein_cc
ENSG0000(	133.2845	-0.68496	0.108135	-6.33432	2.38E-10	1.71E-09	AGBL4	protein_cc
ENSG0000(	133.2812	0.10936	0.045884	2.383419	0.017153	0.03244	RP11-97C	antisense
ENSG0000(	133.2539	0.37578	0.066713	5.632741	1.77E-08	9.47E-08	AC009404	lincRNA
ENSG0000(	133.1119	0.533588	0.056644	9.419987	4.51E-21	1.85E-19	ZNF837	protein_cc
ENSG0000(	133.1075	-0.15944	0.130352	-1.22311	0.221287	0.303979	HCG22	lincRNA
ENSG0000(	133.0919	-0.27182	0.056331	-4.8254	1.40E-06	5.52E-06	RP1-111C	antisense
ENSG0000(	133.0555	0.281052	0.061174	4.594279	4.34E-06	1.59E-05	RP11-51J(	lincRNA
ENSG0000(	133.0228	-0.01882	0.077716	-0.24211	0.808692	0.859078	EFCAB4A	protein_cc
ENSG0000(	132.9691	-0.49362	0.104499	-4.72364	2.32E-06	8.84E-06	LCN12	protein_cc
ENSG0000(	132.9392	-0.28405	0.091187	-3.11505	0.001839	0.004271	CXorf21	protein_cc
ENSG0000(	132.9122	-0.34371	0.083394	-4.12153	3.76E-05	0.000118	AOC3	protein_cc
ENSG0000(	132.8506	0.654439	0.075686	8.646737	5.30E-18	1.32E-16	ARHGEF3(	protein_cc
ENSG0000(	132.8233	-0.40192	0.102804	-3.90962	9.24E-05	0.000272	BHMT2	protein_cc
ENSG0000(	132.7929	-0.09815	0.081551	-1.2035	0.228783	0.312616	PTK6	protein_cc
ENSG0000(	132.4707	0.104665	0.041109	2.546055	0.010895	0.021592	C17orf49	protein_cc
ENSG0000(	132.4587	0.683528	0.074959	9.118643	7.61E-20	2.53E-18	ST20	protein_cc
ENSG0000(	132.4437	1.011929	0.181798	5.566212	2.60E-08	1.35E-07	PITX1	protein_cc
ENSG0000(	132.4334	0.008114	0.09249	0.087727	0.930094	0.951022	CD300C	protein_cc
ENSG0000(	132.4252	-1.49899	0.115963	-12.9265	3.19E-38	3.65E-35	KCNJ12	protein_cc
ENSG0000(	132.4012	-1.28605	0.132446	-9.71	2.73E-22	1.39E-20	C3orf80	protein_cc
ENSG0000(	132.3293	0.454757	0.132255	3.438484	0.000585	0.001491	SHH	protein_cc
ENSG0000(	132.3104	0.146023	0.053332	2.737993	0.006182	0.012916	RP11-152	antisense
ENSG0000(	132.1328	-0.04527	0.06803	-0.66546	0.505757	0.599456	RP4-751H	antisense
ENSG0000(	132.0947	-0.59222	0.085241	-6.94765	3.71E-12	3.53E-11	SYPL2	protein_cc
ENSG0000(	131.9531	-0.11208	0.046951	-2.3871	0.016982	0.032166	RP11-378.	antisense
ENSG0000(	131.8812	-0.45352	0.043905	-10.3297	5.17E-25	4.55E-23	RP11-541	sense_ove
ENSG0000(	131.8797	0.116819	0.085861	1.36056	0.173653	0.247721	MIR503H(	lincRNA
ENSG0000(	131.817	-0.63721	0.137991	-4.61774	3.88E-06	1.43E-05	RP11-597	pseudoge
ENSG0000(	131.5387	0.817703	0.155188	5.269099	1.37E-07	6.35E-07	RGS22	protein_cc
ENSG0000(	131.4636	0.822441	0.102855	7.996121	1.28E-15	2.18E-14	POLQ	protein_cc
ENSG0000(	131.4204	0.463002	0.08488	5.454776	4.90E-08	2.44E-07	SLFN12	protein_cc
ENSG0000(	131.3777	0.046225	0.043065	1.073364	0.283108	0.373745	RP11-412	antisense
ENSG0000(	131.3514	-0.02442	0.081767	-0.2986	0.765242	0.823803	FANK1	protein_cc
ENSG0000(	131.283	-0.19632	0.066078	-2.97107	0.002968	0.006621	C4orf32	protein_cc
ENSG0000(	131.2557	2.501024	0.197716	12.64959	1.12E-36	9.84E-34	SP8	protein_cc
ENSG0000(	131.2481	0.291783	0.048401	6.028487	1.66E-09	1.04E-08	ZNF552	protein_cc
ENSG0000(	131.2378	0.21066	0.076879	2.740143	0.006141	0.012838	ARHGEF1(	protein_cc
ENSG0000(	131.2355	0.009702	0.054557	0.177825	0.85886	0.898059	NUDT13	protein_cc
ENSG0000(	131.2125	0.048061	0.054745	0.877913	0.379991	0.476512	GDF9	protein_cc

ENSG0000(	130.9074	-0.29796	0.040529	-7.35191	1.95E-13	2.26E-12	RP11-517	sense_ove
ENSG0000(	130.8682	-0.3824	0.120003	-3.18662	0.001439	0.003409	AKR1C2	protein_cc
ENSG0000(	130.7932	0.310743	0.122623	2.53414	0.011272	0.02226	RP5-912I1	sense_ove
ENSG0000(	130.765	-0.32418	0.102667	-3.15758	0.001591	0.003739	SLC1A7	protein_cc
ENSG0000(	130.6725	-0.32614	0.08727	-3.7371	0.000186	0.000519	C5AR2	protein_cc
ENSG0000(	130.6649	0.011983	0.072079	0.166246	0.867963	0.905345	TEX22	protein_cc
ENSG0000(	130.5106	0.010092	0.094329	0.106991	0.914796	0.940009	CCDC154	protein_cc
ENSG0000(	130.4243	-0.40344	0.05766	-6.9968	2.62E-12	2.56E-11	RP11-47A	lincRNA
ENSG0000(	130.2235	0.019934	0.065027	0.306547	0.759188	0.818942	PHC1P1	pseudoge
ENSG0000(	130.0959	0.203196	0.104115	1.95166	0.050979	0.085973	CD300LF	protein_cc
ENSG0000(	129.9836	1.191102	0.17379	6.853677	7.20E-12	6.55E-11	C6orf141	protein_cc
ENSG0000(	129.9349	0.379261	0.053873	7.039919	1.92E-12	1.92E-11	ZNF593	protein_cc
ENSG0000(	129.922	0.010728	0.076902	0.139504	0.889052	0.920918	RP11-549	antisense
ENSG0000(	129.8703	-0.52715	0.124345	-4.23939	2.24E-05	7.30E-05	RP11-10L	lincRNA
ENSG0000(	129.732	0.059632	0.110159	0.541328	0.588281	0.67442	PLIN5	protein_cc
ENSG0000(	129.6828	0.398182	0.057858	6.882061	5.90E-12	5.42E-11	LINC0100	antisense
ENSG0000(	129.6559	0.692623	0.066119	10.47539	1.12E-25	1.14E-23	RAD54B	protein_cc
ENSG0000(	129.2443	-0.12123	0.031351	-3.86703	0.00011	0.000319	NDST2	protein_cc
ENSG0000(	129.1805	0.919806	0.112471	8.178165	2.88E-16	5.43E-15	RPS23P8	pseudoge
ENSG0000(	129.1046	0.097193	0.033795	2.875993	0.004028	0.008756	CTD-3185	antisense
ENSG0000(	128.9739	0.034971	0.059678	0.586	0.557876	0.647076	RP1-266L	antisense
ENSG0000(	128.9686	0.407003	0.041805	9.735697	2.12E-22	1.10E-20	RP5-914P	antisense
ENSG0000(	128.9197	-0.11361	0.049793	-2.28164	0.022511	0.041448	CTC-429P	lincRNA
ENSG0000(	128.9131	1.484198	0.160449	9.250268	2.24E-20	8.11E-19	HOXB7	protein_cc
ENSG0000(	128.8663	-0.1096	0.080748	-1.35734	0.174674	0.249013	AC097724	antisense
ENSG0000(	128.8399	-1.31394	0.146116	-8.99245	2.42E-19	7.42E-18	PNMA5	protein_cc
ENSG0000(	128.7675	0.237867	0.053381	4.456039	8.35E-06	2.92E-05	IL17RE	protein_cc
ENSG0000(	128.7295	-0.33976	0.087605	-3.8783	0.000105	0.000306	CNTNAP3	protein_cc
ENSG0000(	128.6674	-0.06505	0.046434	-1.40096	0.161227	0.232569	C19orf71	protein_cc
ENSG0000(	128.6508	-0.57363	0.120198	-4.77234	1.82E-06	7.07E-06	DMKN	protein_cc
ENSG0000(	128.5805	-0.65333	0.136549	-4.78459	1.71E-06	6.67E-06	TMEM233	protein_cc
ENSG0000(	128.5718	0.245606	0.113664	2.160807	0.03071	0.054779	POM121L	pseudoge
ENSG0000(	128.5384	-0.26896	0.092355	-2.91227	0.003588	0.007876	MS4A14	protein_cc
ENSG0000(	128.5196	0.729083	0.124313	5.864908	4.49E-09	2.63E-08	PCDHGB3	protein_cc
ENSG0000(	128.5053	0.617927	0.096167	6.425532	1.31E-10	9.84E-10	S100A2	protein_cc
ENSG0000(	128.467	0.393094	0.093733	4.193765	2.74E-05	8.82E-05	TRPC6	protein_cc
ENSG0000(	128.4091	0.169044	0.040109	4.2146	2.50E-05	8.08E-05	MIR4519	antisense
ENSG0000(	128.3664	-0.1123	0.036083	-3.11234	0.001856	0.004307	DNAJC28	protein_cc
ENSG0000(	128.2546	-0.06611	0.041085	-1.60918	0.107577	0.164528	CTC-487N	antisense
ENSG0000(	128.221	0.068769	0.029126	2.361113	0.01822	0.034239	ZSCAN32	protein_cc
ENSG0000(	128.0525	0.956937	0.130598	7.327352	2.35E-13	2.68E-12	GAPDHP6	pseudoge
ENSG0000(	127.9079	0.234867	0.052888	4.440867	8.96E-06	3.12E-05	FLVCR1-A	lincRNA
ENSG0000(	127.8945	0.043583	0.094697	0.460238	0.645345	0.724797	CD72	protein_cc
ENSG0000(	127.8921	0.019288	0.137676	0.140095	0.888585	0.92062	RP11-986	protein_cc
ENSG0000(	127.8881	0.318573	0.060126	5.298458	1.17E-07	5.48E-07	EMC3-AS	pseudoge
ENSG0000(	127.8743	-0.9442	0.112938	-8.36038	6.25E-17	1.32E-15	RP11-701	lincRNA
ENSG0000(	127.8568	0.068211	0.066364	1.027845	0.304023	0.396833	RBFADN	processed
ENSG0000(	127.7783	0.223927	0.044135	5.073658	3.90E-07	1.68E-06	ASH1L-AS	processed
ENSG0000(	127.7664	0.687043	0.132664	5.178826	2.23E-07	1.00E-06	STEAP1	protein_cc
ENSG0000(	127.7378	-0.40224	0.139525	-2.88294	0.00394	0.008582	MME	protein_cc
ENSG0000(	127.6804	-0.36637	0.043512	-8.41992	3.77E-17	8.23E-16	CTD-2541	antisense
ENSG0000(	127.4955	-0.05314	0.035121	-1.51312	0.130248	0.193684	SLC51A	protein_cc
ENSG0000(	127.4655	-0.28287	0.071996	-3.92895	8.53E-05	0.000253	RP11-499	antisense
ENSG0000(	127.405	0.072714	0.038874	1.870523	0.061411	0.10106	USP27X-A	lincRNA
ENSG0000(	127.3832	-0.04222	0.092497	-0.45645	0.648064	0.727077	ANO1	protein_cc
ENSG0000(	127.1756	-0.05583	0.059444	-0.93923	0.347614	0.443114	MASP2	protein_cc
ENSG0000(	127.1614	0.011409	0.050049	0.22796	0.819677	0.867789	PAN3-AS	antisense
ENSG0000(	127.1544	-1.0803	0.15089	-7.1595	8.10E-13	8.54E-12	RP11-320	sense_ove
ENSG0000(	127.0699	0.130874	0.061845	2.116169	0.03433	0.060572	FBXL8	protein_cc

ENSG0000(	126.9082	0.091712	0.090705	1.011105	0.311966	0.405259	MTRNR2L8	protein_cc
ENSG0000(	126.8991	0.030425	0.043118	0.70564	0.480412	0.575755	ATP1A1OS	processed
ENSG0000(	126.8601	-0.33985	0.123331	-2.75557	0.005859	0.0123	HCN4	protein_cc
ENSG0000(	126.7525	0.968909	0.161222	6.009791	1.86E-09	1.16E-08	F2RL2	protein_cc
ENSG0000(	126.6461	-0.15703	0.098566	-1.59312	0.111134	0.169257	TNFSF8	protein_cc
ENSG0000(	126.6417	-0.07369	0.055073	-1.33798	0.180904	0.25634	RP11-815	sense_intr
ENSG0000(	126.5975	0.006711	0.068331	0.098207	0.921768	0.944835	RP11-395	sense_ove
ENSG0000(	126.5821	0.285678	0.045174	6.323901	2.55E-10	1.82E-09	CBR3-AS1	processed
ENSG0000(	126.5695	-0.04611	0.058496	-0.78821	0.430573	0.527587	EIF4BP6	pseudoge
ENSG0000(	126.5183	0.296407	0.124685	2.377241	0.017443	0.032925	FAR2P2	pseudoge
ENSG0000(	126.5046	2.846861	0.181832	15.65651	3.00E-55	1.37E-51	TRDC	TR_C_gene
ENSG0000(	126.4631	0.384706	0.073287	5.249334	1.53E-07	7.02E-07	CTD-2541	lincRNA
ENSG0000(	126.4434	0.215689	0.11286	1.911115	0.05599	0.093272	MORN5	protein_cc
ENSG0000(	126.3163	0.284304	0.073832	3.85071	0.000118	0.00034	RP11-452	pseudoge
ENSG0000(	126.3152	-1.09977	0.115461	-9.52506	1.65E-21	7.37E-20	MPP7	protein_cc
ENSG0000(	126.2005	0.068711	0.037246	1.844822	0.065063	0.106291	CTC-338M	antisense
ENSG0000(	126.1861	-0.9534	0.179522	-5.31078	1.09E-07	5.15E-07	ADAMTS2	protein_cc
ENSG0000(	126.168	-0.03775	0.080773	-0.46735	0.640251	0.72026	ZNF233	protein_cc
ENSG0000(	126.1677	0.059342	0.045857	1.294064	0.195643	0.274089	RP11-252	pseudoge
ENSG0000(	126.0342	0.659739	0.114661	5.753842	8.72E-09	4.88E-08	LCTL	protein_cc
ENSG0000(	126.0175	-0.17303	0.12971	-1.33398	0.182211	0.257863	RP11-615	antisense
ENSG0000(	126.0006	-0.19694	0.07883	-2.49835	0.012477	0.024378	DLK2	protein_cc
ENSG0000(	125.9737	0.233566	0.06756	3.45715	0.000546	0.001399	AC011526	lincRNA
ENSG0000(	125.9343	0.565629	0.106737	5.299272	1.16E-07	5.46E-07	ANG	protein_cc
ENSG0000(	125.9208	0.662778	0.075217	8.811533	1.23E-18	3.38E-17	C1orf53	protein_cc
ENSG0000(	125.8394	-0.38931	0.080352	-4.84502	1.27E-06	5.04E-06	CAPN9	protein_cc
ENSG0000(	125.7783	0.289779	0.055998	5.174805	2.28E-07	1.02E-06	C9orf169	protein_cc
ENSG0000(	125.7541	-0.05853	0.04759	-1.2299	0.218736	0.301078	ZNF23	protein_cc
ENSG0000(	125.7489	-0.38504	0.153343	-2.51099	0.012039	0.023616	CYP4F12	protein_cc
ENSG0000(	125.7448	-0.62785	0.153606	-4.08737	4.36E-05	0.000136	ALDH1A3	protein_cc
ENSG0000(	125.7365	0.095526	0.072539	1.316892	0.187875	0.264749	RP11-627	processed
ENSG0000(	125.7286	-0.52733	0.114569	-4.60272	4.17E-06	1.53E-05	MSC	protein_cc
ENSG0000(	125.7069	-0.13396	0.063626	-2.10539	0.035258	0.062037	CCNI2	protein_cc
ENSG0000(	125.6406	0.232715	0.04419	5.266244	1.39E-07	6.44E-07	KLRG1	protein_cc
ENSG0000(	125.5214	0.272105	0.068352	3.980924	6.86E-05	0.000207	FCRLB	protein_cc
ENSG0000(	125.4924	-0.39545	0.05737	-6.89305	5.46E-12	5.04E-11	SOX7	protein_cc
ENSG0000(	125.4837	0.199043	0.053232	3.739153	0.000185	0.000515	ACRC	protein_cc
ENSG0000(	125.455	0.006023	0.077935	0.077286	0.938396	0.95699	RP11-347	lincRNA
ENSG0000(	125.4385	0.183007	0.052168	3.508022	0.000451	0.001176	LENG8-AS	antisense
ENSG0000(	125.4152	1.732209	0.270025	6.41499	1.41E-10	1.05E-09	HOXA3	protein_cc
ENSG0000(	125.4097	0.186765	0.055487	3.365907	0.000763	0.0019	RP11-95P	lincRNA
ENSG0000(	125.4027	-0.13112	0.060197	-2.17812	0.029397	0.052691	RP11-372	antisense
ENSG0000(	125.3721	-0.9219	0.104347	-8.83499	1.00E-18	2.78E-17	LRRC3	protein_cc
ENSG0000(	125.3584	0.339687	0.047616	7.133865	9.76E-13	1.02E-11	POPDC2	protein_cc
ENSG0000(	125.2957	0.950359	0.102211	9.298052	1.43E-20	5.35E-19	COX6A1P2	protein_cc
ENSG0000(	125.2707	0.536728	0.058828	9.123662	7.26E-20	2.42E-18	TMPO-AS	antisense
ENSG0000(	125.26	-1.09709	0.181721	-6.03719	1.57E-09	9.90E-09	PVALB	protein_cc
ENSG0000(	125.2168	-0.16464	0.085654	-1.92211	0.054591	0.091257	SLC9A3	protein_cc
ENSG0000(	125.084	0.481252	0.089609	5.370597	7.85E-08	3.78E-07	RPL7P1	pseudoge
ENSG0000(	124.9824	-0.29582	0.07252	-4.07914	4.52E-05	0.00014	NUTM2D	protein_cc
ENSG0000(	124.9134	-0.11876	0.045813	-2.59235	0.009532	0.019138	CCDC183	protein_cc
ENSG0000(	124.8315	-0.36711	0.131554	-2.79058	0.005261	0.011167	FAM201A	antisense
ENSG0000(	124.7466	2.4094	0.242324	9.942873	2.71E-23	1.70E-21	AC005550	lincRNA
ENSG0000(	124.6803	-0.10306	0.070765	-1.45636	0.145294	0.212823	PROM2	protein_cc
ENSG0000(	124.6091	0.235103	0.058025	4.05177	5.08E-05	0.000156	RP11-486	lincRNA
ENSG0000(	124.5553	-0.30647	0.11241	-2.72641	0.006403	0.013336	HPN	protein_cc
ENSG0000(	124.4263	0.135838	0.071755	1.893081	0.058347	0.096706	PCDHB12	protein_cc
ENSG0000(	124.3753	0.987474	0.085761	11.51423	1.12E-30	3.01E-28	AC009501	antisense
ENSG0000(	124.3277	-0.30458	0.086256	-3.53107	0.000414	0.001085	RP11-326	antisense

ENSG0000(	124.2758	-0.0441	0.07883	-0.55942	0.575876	0.663129	PTPRG-AS	processed
ENSG0000(	124.2742	-0.24409	0.063876	-3.8213	0.000133	0.00038	ANKRD19	pseudoge
ENSG0000(	124.0836	0.131722	0.065549	2.009521	0.044482	0.076178	RP11-448	sense_ove
ENSG0000(	124.0206	0.180847	0.060811	2.973895	0.00294	0.006567	WHAMMF	pseudoge
ENSG0000(	123.9854	-0.22883	0.126934	-1.80276	0.071426	0.115312	TLR8	protein_cc
ENSG0000(	123.7278	0.019115	0.064721	0.295347	0.767729	0.825716	CLEC1A	protein_cc
ENSG0000(	123.7217	-1.15197	0.143584	-8.02298	1.03E-15	1.78E-14	DRD1	protein_cc
ENSG0000(	123.6913	-0.12097	0.062694	-1.92951	0.053668	0.089893	RP11-894	antisense
ENSG0000(	123.6458	0.124061	0.055894	2.219568	0.026448	0.047923	RP11-708.	antisense
ENSG0000(	123.5609	0.731366	0.094602	7.730984	1.07E-14	1.54E-13	ECSCR	protein_cc
ENSG0000(	123.5437	-0.50754	0.113321	-4.47879	7.51E-06	2.65E-05	ZNF812	protein_cc
ENSG0000(	123.485	0.204255	0.041754	4.891819	9.99E-07	4.04E-06	AC002467	antisense
ENSG0000(	123.4634	0.003186	0.080602	0.039532	0.968466	0.978195	LA16c-38(	lincRNA
ENSG0000(	123.445	-0.25642	0.065899	-3.89107	9.98E-05	0.000292	RP11-129	antisense
ENSG0000(	123.4388	-0.05945	0.069797	-0.85179	0.39433	0.490842	RP11-795	protein_cc
ENSG0000(	123.4335	0.073926	0.07878	0.938391	0.348044	0.443592	FGFR4	protein_cc
ENSG0000(	123.4241	0.19998	0.051015	3.92004	8.85E-05	0.000261	CBWD7	protein_cc
ENSG0000(	123.3743	-0.09918	0.110932	-0.89408	0.371278	0.467697	RP11-401	antisense
ENSG0000(	123.3454	0.811454	0.129061	6.287354	3.23E-10	2.28E-09	SPAG4	protein_cc
ENSG0000(	123.2891	-0.46747	0.074018	-6.3156	2.69E-10	1.92E-09	ATF7IP2	protein_cc
ENSG0000(	123.2209	0.9504	0.122747	7.742759	9.73E-15	1.42E-13	YBX1P1	pseudoge
ENSG0000(	123.2003	0.08205	0.09372	0.875483	0.381311	0.477935	KCNK7	protein_cc
ENSG0000(	123.1894	0.914012	0.139087	6.571517	4.98E-11	3.97E-10	C9orf171	protein_cc
ENSG0000(	123.0681	0.180495	0.055038	3.279457	0.00104	0.002525	RP11-146	antisense
ENSG0000(	123.0443	-1.31441	0.127213	-10.3324	5.03E-25	4.46E-23	CPNE9	protein_cc
ENSG0000(	123.0292	-0.08734	0.056344	-1.55005	0.121129	0.182086	HYKK	protein_cc
ENSG0000(	123.029	-0.10385	0.075765	-1.37073	0.170458	0.243916	CMTM8	protein_cc
ENSG0000(	122.9292	-0.2464	0.091352	-2.6973	0.00699	0.014445	CCDC144(	pseudoge
ENSG0000(	122.9282	-0.20719	0.08837	-2.34458	0.019049	0.035636	WNK4	protein_cc
ENSG0000(	122.9242	0.082527	0.068947	1.196957	0.231323	0.315405	TBC1D10(	protein_cc
ENSG0000(	122.884	0.323013	0.043495	7.426455	1.12E-13	1.35E-12	RP11-767	sense_intr
ENSG0000(	122.6441	0.907403	0.130912	6.931385	4.17E-12	3.93E-11	EVC2	protein_cc
ENSG0000(	122.6392	0.116468	0.107974	1.078665	0.280737	0.371306	P2RY6	protein_cc
ENSG0000(	122.6193	0.369576	0.112162	3.295011	0.000984	0.0024	ATAD3C	protein_cc
ENSG0000(	122.5867	0.129547	0.047587	2.722312	0.006483	0.013491	RP11-363	sense_ove
ENSG0000(	122.5336	0.48113	0.127166	3.783471	0.000155	0.000437	CD52	protein_cc
ENSG0000(	122.5171	-0.07876	0.121122	-0.65029	0.515505	0.608346	PI15	protein_cc
ENSG0000(	122.5167	0.468467	0.081039	5.780731	7.44E-09	4.21E-08	SMKR1	protein_cc
ENSG0000(	122.486	-0.1078	0.090626	-1.18945	0.234261	0.318798	MADCAM	protein_cc
ENSG0000(	122.4157	-0.03541	0.074067	-0.47812	0.632564	0.713737	CCDC13-/	antisense
ENSG0000(	122.2753	0.058616	0.104257	0.562225	0.573963	0.661278	TRPC4	protein_cc
ENSG0000(	122.0736	0.069795	0.041153	1.695981	0.08989	0.140877	LAMTOR5	antisense
ENSG0000(	122.0683	0.067428	0.05419	1.244288	0.213393	0.294924	MGAT2	protein_cc
ENSG0000(	122.0444	0.273386	0.066405	4.116963	3.84E-05	0.00012	CCND2-A	antisense
ENSG0000(	121.9534	0.395816	0.064694	6.118312	9.46E-10	6.19E-09	AP000473	lincRNA
ENSG0000(	121.8784	0.532661	0.063301	8.414743	3.94E-17	8.55E-16	C19orf40	protein_cc
ENSG0000(	121.878	-0.00792	0.086394	-0.09173	0.926912	0.948548	CD226	protein_cc
ENSG0000(	121.8341	0.480478	0.132799	3.618097	0.000297	0.000799	AOX1	protein_cc
ENSG0000(	121.8313	0.471221	0.091011	5.177643	2.25E-07	1.01E-06	HGFAC	protein_cc
ENSG0000(	121.6297	0.615366	0.099155	6.206095	5.43E-10	3.68E-09	RP11-218	lincRNA
ENSG0000(	121.5578	0.800643	0.155513	5.148414	2.63E-07	1.16E-06	TEX26	protein_cc
ENSG0000(	121.4933	0.192844	0.086239	2.236146	0.025342	0.046097	GAS2	protein_cc
ENSG0000(	121.3725	0.361123	0.104533	3.454613	0.000551	0.00141	ATG9B	protein_cc
ENSG0000(	121.3483	-0.28588	0.061911	-4.61751	3.88E-06	1.43E-05	SNAI3	protein_cc
ENSG0000(	121.3125	-0.28139	0.042966	-6.54913	5.79E-11	4.57E-10	CTD-2555	antisense
ENSG0000(	121.2212	0.45632	0.071799	6.355542	2.08E-10	1.50E-09	EXTL3-AS.	antisense
ENSG0000(	121.1886	-0.08965	0.095391	-0.93987	0.347284	0.442761	SPINT1	protein_cc
ENSG0000(	121.1538	-0.04777	0.081206	-0.58831	0.556324	0.64575	CPT1B	protein_cc
ENSG0000(	121.1445	0.406495	0.146865	2.767812	0.005643	0.011891	FGFBP2	protein_cc

ENSG0000(	121.126	0.66919	0.138787	4.821704	1.42E-06	5.62E-06	GRB14	protein_cc
ENSG0000(	121.1169	0.31908	0.050423	6.328027	2.48E-10	1.78E-09	AC022007	lincRNA
ENSG0000(	121.1115	-0.0171	0.056646	-0.30185	0.762764	0.821881	AC239799	protein_cc
ENSG0000(	121.0926	-0.3879	0.070497	-5.50241	3.75E-08	1.90E-07	RP11-192	protein_cc
ENSG0000(	121.0913	0.366974	0.057117	6.424982	1.32E-10	9.86E-10	ZNF503-A	processed
ENSG0000(	121.0698	-0.72034	0.098789	-7.29175	3.06E-13	3.44E-12	CORO2A	protein_cc
ENSG0000(	120.9791	1.52657	0.305094	5.003605	5.63E-07	2.37E-06	HOXD13	protein_cc
ENSG0000(	120.9715	0.271719	0.047666	5.700481	1.19E-08	6.55E-08	DDX47	protein_cc
ENSG0000(	120.9502	0.519742	0.094741	5.485939	4.11E-08	2.07E-07	DDX12P	pseudoge
ENSG0000(	120.9458	1.581292	0.162591	9.725596	2.35E-22	1.21E-20	MYBPH	protein_cc
ENSG0000(	120.8189	0.498974	0.105089	4.748123	2.05E-06	7.90E-06	TFAP2A-A	antisense
ENSG0000(	120.8048	0.137278	0.07439	1.845392	0.06498	0.106181	HAS2-AS1	antisense
ENSG0000(	120.7715	-0.025	0.037384	-0.66878	0.503636	0.597443	RP11-319	antisense
ENSG0000(	120.691	0.090496	0.053702	1.68516	0.091958	0.143631	AC025171	antisense
ENSG0000(	120.6888	0.317947	0.093918	3.385383	0.000711	0.001781	USP6	protein_cc
ENSG0000(	120.6454	-0.13353	0.076516	-1.74513	0.080962	0.128566	RP11-441	lincRNA
ENSG0000(	120.5851	-0.00339	0.066736	-0.05078	0.959505	0.972311	LRRC39	protein_cc
ENSG0000(	120.4933	-0.32482	0.067416	-4.81817	1.45E-06	5.71E-06	RP11-108	antisense
ENSG0000(	120.4921	-0.1587	0.041256	-3.84668	0.00012	0.000345	C16orf46	protein_cc
ENSG0000(	120.4499	0.295514	0.099991	2.955398	0.003123	0.006935	NPIPA5	protein_cc
ENSG0000(	120.438	0.716813	0.101823	7.039775	1.93E-12	1.92E-11	SBF2-AS1	antisense
ENSG0000(	120.2892	0.241026	0.058377	4.128778	3.65E-05	0.000115	hsa-mir-8	lincRNA
ENSG0000(	120.282	0.21859	0.149874	1.458491	0.144705	0.212091	NR0B1	protein_cc
ENSG0000(	120.2575	-0.09698	0.045586	-2.12738	0.033389	0.059053	HIATL2	protein_cc
ENSG0000(	120.221	0.154836	0.112387	1.377707	0.168294	0.241298	SIGLEC7	protein_cc
ENSG0000(	120.1926	-0.23875	0.066919	-3.56772	0.00036	0.000955	SOX17	protein_cc
ENSG0000(	120.0887	-0.6178	0.129664	-4.76463	1.89E-06	7.32E-06	HAR1A	lincRNA
ENSG0000(	120.0216	0.369675	0.058971	6.268758	3.64E-10	2.54E-09	RP11-48B	lincRNA
ENSG0000(	119.982	0.353233	0.060614	5.827554	5.62E-09	3.24E-08	MTHFS	protein_cc
ENSG0000(	119.9375	0.004995	0.076562	0.065238	0.947985	0.964043	RP11-521	antisense
ENSG0000(	119.9021	0.363881	0.079308	4.588203	4.47E-06	1.63E-05	LCA5L	protein_cc
ENSG0000(	119.8894	0.122137	0.059234	2.061935	0.039214	0.068173	TMEM86B	protein_cc
ENSG0000(	119.8373	0.185095	0.037625	4.91942	8.68E-07	3.54E-06	ANKAR	protein_cc
ENSG0000(	119.8288	0.014241	0.033956	0.419402	0.674922	0.749506	ZBTB11-A	antisense
ENSG0000(	119.8047	-1.11421	0.134645	-8.27519	1.28E-16	2.56E-15	GRM4	protein_cc
ENSG0000(	119.7748	-0.12163	0.051184	-2.37624	0.01749	0.032996	RP11-731	antisense
ENSG0000(	119.7442	-0.75659	0.152413	-4.96409	6.90E-07	2.86E-06	ADCYAP1	protein_cc
ENSG0000(	119.6292	-0.27373	0.0493	-5.55241	2.82E-08	1.46E-07	LINC0102	antisense
ENSG0000(	119.5479	-0.61674	0.132879	-4.64141	3.46E-06	1.28E-05	FLG-AS1	antisense
ENSG0000(	119.5195	-0.54988	0.106739	-5.15163	2.58E-07	1.15E-06	C1orf222	protein_cc
ENSG0000(	119.5081	0.574164	0.07973	7.201384	5.96E-13	6.41E-12	AC068535	lincRNA
ENSG0000(	119.3959	0.061943	0.060858	1.017841	0.308753	0.401848	RP11-61J1	lincRNA
ENSG0000(	119.3888	-0.37353	0.079185	-4.71714	2.39E-06	9.11E-06	RP11-508	lincRNA
ENSG0000(	119.2981	-0.10239	0.039243	-2.60912	0.009077	0.018308	RP11-158	antisense
ENSG0000(	119.1712	0.183973	0.064922	2.833732	0.004601	0.009867	AC132192	protein_cc
ENSG0000(	119.1514	0.143305	0.075993	1.885763	0.059327	0.09808	RP11-439	processed
ENSG0000(	119.0695	0.547906	0.081321	6.737569	1.61E-11	1.39E-10	LRRC46	protein_cc
ENSG0000(	119.0666	-0.53561	0.100764	-5.3155	1.06E-07	5.03E-07	RP11-624	antisense
ENSG0000(	119.0387	-0.76886	0.088071	-8.73003	2.55E-18	6.68E-17	TRPM6	protein_cc
ENSG0000(	118.9859	0.105909	0.119795	0.884089	0.376648	0.4731	AC005754	lincRNA
ENSG0000(	118.882	-0.4845	0.060698	-7.98222	1.44E-15	2.42E-14	CTD-2541	lincRNA
ENSG0000(	118.7905	-0.0514	0.100177	-0.51306	0.607911	0.691662	ECHDC3	protein_cc
ENSG0000(	118.4723	0.085902	0.055455	1.549034	0.121374	0.182406	OPN1SW	protein_cc
ENSG0000(	118.4605	-0.75611	0.161985	-4.66781	3.04E-06	1.14E-05	KRT17	protein_cc
ENSG0000(	118.4347	-0.30587	0.054194	-5.64399	1.66E-08	8.90E-08	LMCD1-A	antisense
ENSG0000(	118.3302	0.451536	0.145616	3.100865	0.00193	0.004463	C21orf33	protein_cc
ENSG0000(	118.3176	-0.22678	0.124746	-1.81791	0.069077	0.112028	AMICA1	protein_cc
ENSG0000(	118.2654	-0.41068	0.065041	-6.31413	2.72E-10	1.94E-09	RP11-513	antisense
ENSG0000(	118.2381	-0.05407	0.069828	-0.77437	0.438711	0.535612	AC138123	pseudoge

ENSG0000(	118.2112	-0.35848	0.154678	-2.31756	0.020473	0.038023	AC006262	processed
ENSG0000(	118.136	0.573214	0.097092	5.903812	3.55E-09	2.12E-08	APOBEC3I	protein_cc
ENSG0000(	118.0886	0.471244	0.086247	5.46386	4.66E-08	2.33E-07	SMAGP	protein_cc
ENSG0000(	117.9745	0.247842	0.092754	2.672046	0.007539	0.015488	RP11-556	pseudoge
ENSG0000(	117.9243	0.209537	0.10986	1.9073	0.056482	0.093985	CTB-113D	antisense
ENSG0000(	117.9241	-0.15529	0.053042	-2.92766	0.003415	0.007528	CTD-2047	antisense
ENSG0000(	117.8613	0.467158	0.06129	7.622065	2.50E-14	3.36E-13	C16orf93	protein_cc
ENSG0000(	117.7899	-0.4887	0.116731	-4.18654	2.83E-05	9.08E-05	CD163L1	protein_cc
ENSG0000(	117.6014	0.069075	0.055411	1.246594	0.212546	0.293925	RP11-686	lincRNA
ENSG0000(	117.5815	0.241344	0.084754	2.847596	0.004405	0.009494	FAM86B3I	pseudoge
ENSG0000(	117.581	-0.05402	0.048049	-1.12435	0.260863	0.34918	ZNF485	protein_cc
ENSG0000(	117.1214	-0.22057	0.066332	-3.32519	0.000884	0.002175	FAM13A-/	antisense
ENSG0000(	117.0539	-0.08681	0.078343	-1.10801	0.267858	0.356959	LINC0017:	processed
ENSG0000(	116.9135	0.056904	0.029679	1.917359	0.055192	0.092138	RBM34	protein_cc
ENSG0000(	116.7275	-0.08658	0.12687	-0.68245	0.494951	0.589231	CCDC178	protein_cc
ENSG0000(	116.6397	0.387458	0.067844	5.711018	1.12E-08	6.18E-08	ZNF600	protein_cc
ENSG0000(	116.6257	-0.28031	0.065198	-4.29932	1.71E-05	5.70E-05	EIF4BP7	pseudoge
ENSG0000(	116.6186	0.851069	0.093235	9.128249	6.96E-20	2.33E-18	GPR156	protein_cc
ENSG0000(	116.6118	-0.28456	0.089641	-3.17441	0.001501	0.003547	GPR63	protein_cc
ENSG0000(	116.6	-0.21305	0.044748	-4.76107	1.93E-06	7.44E-06	CTD-2260	protein_cc
ENSG0000(	116.578	0.253518	0.059269	4.277419	1.89E-05	6.25E-05	CCR10	protein_cc
ENSG0000(	116.5539	-0.22166	0.054345	-4.0787	4.53E-05	0.00014	RP11-84G	antisense
ENSG0000(	116.5272	-0.0607	0.078822	-0.77011	0.441232	0.538086	MUC20	protein_cc
ENSG0000(	116.4866	0.07924	0.04643	1.706661	0.087885	0.138178	XKR6	protein_cc
ENSG0000(	116.4362	0.023421	0.065509	0.357523	0.720701	0.78743	AC083843	lincRNA
ENSG0000(	116.3995	0.177938	0.062607	2.842126	0.004481	0.009641	C6orf52	protein_cc
ENSG0000(	116.3975	-0.07907	0.078944	-1.00158	0.316547	0.410187	LINC0089	antisense
ENSG0000(	116.2027	0.024845	0.052838	0.470205	0.638209	0.718391	RP11-259	antisense
ENSG0000(	116.1292	-0.21436	0.154946	-1.38347	0.166522	0.239133	RNF128	protein_cc
ENSG0000(	116.1077	0.372252	0.054122	6.877999	6.07E-12	5.57E-11	SNORA67	processed
ENSG0000(	116.074	1.053955	0.118314	8.908091	5.19E-19	1.51E-17	GALNT3	protein_cc
ENSG0000(	116.0531	-0.12082	0.054528	-2.21576	0.026708	0.048346	CCDC117	protein_cc
ENSG0000(	116.04	-0.56375	0.062304	-9.04836	1.45E-19	4.63E-18	RP4-657E	pseudoge
ENSG0000(	116.0308	-0.23249	0.061385	-3.78746	0.000152	0.000431	OCLN	protein_cc
ENSG0000(	115.9469	0.329097	0.133943	2.456986	0.014011	0.02708	CCDC135	protein_cc
ENSG0000(	115.8497	-0.22351	0.062447	-3.57923	0.000345	0.000917	GNG12-A	antisense
ENSG0000(	115.7684	-0.27749	0.057189	-4.85219	1.22E-06	4.87E-06	OTUB2	protein_cc
ENSG0000(	115.7414	1.477877	0.168127	8.790225	1.49E-18	4.05E-17	CXCL11	protein_cc
ENSG0000(	115.696	-0.20966	0.133074	-1.57551	0.115138	0.174508	CXCL3	protein_cc
ENSG0000(	115.5061	-0.13997	0.079927	-1.75122	0.079908	0.127144	ZSCAN23	protein_cc
ENSG0000(	115.4357	0.305697	0.090495	3.37807	0.00073	0.001825	DERL3	protein_cc
ENSG0000(	115.3625	-0.07343	0.06325	-1.16094	0.245666	0.332033	RP11-488	lincRNA
ENSG0000(	115.3332	0.17295	0.04412	3.919963	8.86E-05	0.000261	CTD-2583	sense_intr
ENSG0000(	115.325	0.157453	0.043662	3.606187	0.000311	0.000834	ALG10	protein_cc
ENSG0000(	115.2933	-0.09147	0.070977	-1.2887	0.197502	0.276298	RP11-393	antisense
ENSG0000(	115.2142	-0.02555	0.065089	-0.39251	0.694679	0.766083	AC139100	antisense
ENSG0000(	115.2132	0.280888	0.042343	6.633615	3.28E-11	2.69E-10	TSSK6	protein_cc
ENSG0000(	115.1923	-0.23445	0.051464	-4.55566	5.22E-06	1.89E-05	PTENP1	pseudoge
ENSG0000(	115.1603	0.730741	0.135746	5.383156	7.32E-08	3.54E-07	PLAC8	protein_cc
ENSG0000(	115.053	-0.11746	0.029589	-3.96955	7.20E-05	0.000216	CTAGE5	protein_cc
ENSG0000(	115.0198	-0.25918	0.187508	-1.38222	0.166903	0.239596	TMSB4Y	protein_cc
ENSG0000(	115.0139	-0.32293	0.088799	-3.6366	0.000276	0.000748	PLAG1	protein_cc
ENSG0000(	115.0082	-0.13509	0.0408	-3.31091	0.00093	0.002278	P2RX5-TA	protein_cc
ENSG0000(	114.9229	-0.82621	0.091492	-9.03036	1.71E-19	5.39E-18	STXBP5-A	antisense
ENSG0000(	114.8677	0.031236	0.05996	0.520947	0.602403	0.686791	CATSPER(	protein_cc
ENSG0000(	114.5886	-1.23781	0.118552	-10.441	1.61E-25	1.54E-23	RP11-66B	antisense
ENSG0000(	114.5601	0.649626	0.091108	7.130313	1.00E-12	1.04E-11	IGSF9	protein_cc
ENSG0000(	114.5275	0.341959	0.062735	5.450809	5.01E-08	2.49E-07	IQCH	protein_cc
ENSG0000(	114.4622	-0.27708	0.083579	-3.31524	0.000916	0.002247	CDHR2	protein_cc

ENSG0000(	114.4549	0.078749	0.126916	0.620476	0.534945	0.626219	PAX8	protein_cc
ENSG0000(	114.4476	0.049392	0.04976	0.992588	0.320911	0.41473	LINC0095(	lincRNA
ENSG0000(	114.4155	-0.24754	0.055212	-4.48345	7.34E-06	2.59E-05	MYLK4	protein_cc
ENSG0000(	114.3797	-0.03504	0.073955	-0.47375	0.635679	0.716269	PRSS53	protein_cc
ENSG0000(	114.3778	-0.14249	0.050246	-2.83584	0.004571	0.009811	RP11-166	lincRNA
ENSG0000(	114.3516	-0.39054	0.058038	-6.72899	1.71E-11	1.46E-10	C10orf12	protein_cc
ENSG0000(	114.3257	0.96424	0.129206	7.462816	8.47E-14	1.04E-12	CXorf28	protein_cc
ENSG0000(	114.3066	0.196342	0.108589	1.808131	0.070586	0.114121	FHAD1	protein_cc
ENSG0000(	114.137	0.144189	0.065187	2.211936	0.026971	0.048741	NAGS	protein_cc
ENSG0000(	114.1024	0.34953	0.087063	4.014665	5.95E-05	0.000181	FAM188B	protein_cc
ENSG0000(	114.0168	-0.00026	0.096064	-0.00271	0.997837	0.998712	MIR29A	lincRNA
ENSG0000(	114.0146	-0.20639	0.09362	-2.20454	0.027487	0.049562	ATP2C2	protein_cc
ENSG0000(	113.9298	0.063489	0.042517	1.493261	0.135369	0.200227	AC062029	antisense
ENSG0000(	113.8458	0.195438	0.087942	2.222345	0.02626	0.047615	FAIM3	protein_cc
ENSG0000(	113.796	-0.20805	0.065027	-3.19946	0.001377	0.003274	P2RY14	protein_cc
ENSG0000(	113.7892	0.199126	0.054308	3.666621	0.000246	0.000671	PDCD4-AS	antisense
ENSG0000(	113.7011	-0.2988	0.107366	-2.78301	0.005386	0.011405	LINC0092	lincRNA
ENSG0000(	113.6609	-0.19119	0.160622	-1.19032	0.233922	0.31841	RP11-116	sense_intr
ENSG0000(	113.6331	0.105108	0.10275	1.022952	0.306331	0.399339	RNF5P1	pseudoge
ENSG0000(	113.5405	0.565157	0.091005	6.210138	5.29E-10	3.60E-09	RP11-82L	sense_intr
ENSG0000(	113.5095	-0.03936	0.155301	-0.25347	0.799901	0.852121	DKK1	protein_cc
ENSG0000(	113.5052	0.318096	0.04593	6.925622	4.34E-12	4.09E-11	AC004967	pseudoge
ENSG0000(	113.4917	0.519152	0.092559	5.608884	2.04E-08	1.08E-07	AC027612	pseudoge
ENSG0000(	113.4887	-0.73157	0.111102	-6.58468	4.56E-11	3.66E-10	TG	protein_cc
ENSG0000(	113.4303	0.217849	0.036608	5.950842	2.67E-09	1.62E-08	RP11-251	antisense
ENSG0000(	113.3009	0.938309	0.081435	11.52224	1.02E-30	2.78E-28	SNRPGP2	pseudoge
ENSG0000(	113.1013	-0.03996	0.125096	-0.31945	0.749382	0.811266	PWP2	protein_cc
ENSG0000(	113.0394	0.100666	0.077513	1.298704	0.194045	0.272122	PDCL3P4	pseudoge
ENSG0000(	112.9946	0.588008	0.093904	6.261814	3.81E-10	2.65E-09	RDH5	protein_cc
ENSG0000(	112.6942	-0.21486	0.101701	-2.1127	0.034627	0.061034	TPTE2P1	pseudoge
ENSG0000(	112.691	0.514165	0.203959	2.520928	0.011705	0.023024	MMP7	protein_cc
ENSG0000(	112.6272	-0.02721	0.071817	-0.37892	0.704749	0.774611	IL12A	protein_cc
ENSG0000(	112.6172	-0.28227	0.072059	-3.91728	8.96E-05	0.000264	TDRD6	protein_cc
ENSG0000(	112.5498	0.257382	0.116291	2.213258	0.02688	0.048596	IGFBP1	protein_cc
ENSG0000(	112.4717	-0.11602	0.072355	-1.60354	0.108814	0.166136	SP140	protein_cc
ENSG0000(	112.286	0.036934	0.048983	0.754012	0.450842	0.547577	C2orf16	protein_cc
ENSG0000(	112.2536	1.981827	0.125227	15.82588	2.06E-56	1.21E-52	CYP27B1	protein_cc
ENSG0000(	112.1763	-0.09008	0.075921	-1.18648	0.235433	0.320192	RGMB-AS	antisense
ENSG0000(	112.1518	-1.17765	0.126598	-9.30229	1.37E-20	5.16E-19	TRPV6	protein_cc
ENSG0000(	112.1323	0.367963	0.05727	6.425076	1.32E-10	9.86E-10	C9orf50	protein_cc
ENSG0000(	112.0014	-0.10991	0.073538	-1.49465	0.135005	0.199746	PTPLA	protein_cc
ENSG0000(	111.9756	-0.41664	0.107844	-3.86332	0.000112	0.000324	SFTPC	protein_cc
ENSG0000(	111.9754	-0.32561	0.1141	-2.85374	0.004321	0.009329	AQP3	protein_cc
ENSG0000(	111.8618	0.613053	0.104283	5.878736	4.13E-09	2.44E-08	CHL1-AS2	processed
ENSG0000(	111.7806	1.72294	0.227306	7.579834	3.46E-14	4.55E-13	NKX2-5	protein_cc
ENSG0000(	111.7177	-0.53493	0.122572	-4.3642	1.28E-05	4.33E-05	RP11-54C	lincRNA
ENSG0000(	111.6473	0.151131	0.068213	2.215581	0.02672	0.048358	RP4-583P	protein_cc
ENSG0000(	111.6134	-0.2068	0.065046	-3.17926	0.001476	0.003492	RP11-799	antisense
ENSG0000(	111.6102	0.202069	0.100712	2.006403	0.044813	0.076679	CFB	protein_cc
ENSG0000(	111.5744	0.28454	0.056023	5.079032	3.79E-07	1.64E-06	FLT3LG	protein_cc
ENSG0000(	111.4768	1.305642	0.12669	10.3058	6.63E-25	5.67E-23	NSUN7	protein_cc
ENSG0000(	111.4746	0.331211	0.097392	3.400798	0.000672	0.001692	NOVA1-A	antisense
ENSG0000(	111.4354	0.25658	0.084924	3.021305	0.002517	0.005704	KLHL7-AS	lincRNA
ENSG0000(	111.3058	-0.19218	0.147371	-1.30403	0.192225	0.26998	IL1RN	protein_cc
ENSG0000(	111.2882	0.092397	0.051083	1.808753	0.070489	0.113982	RP11-685	antisense
ENSG0000(	111.0695	-1.35764	0.157628	-8.61294	7.12E-18	1.75E-16	PCDHGB4	protein_cc
ENSG0000(	110.831	-0.06066	0.047105	-1.28786	0.197796	0.276634	LINC0091(	lincRNA
ENSG0000(	110.7182	0.22391	0.036027	6.215146	5.13E-10	3.49E-09	PHOSPHC	protein_cc
ENSG0000(	110.7049	0.240361	0.055669	4.317679	1.58E-05	5.27E-05	TSPAN10	polymorpl

ENSG0000(	110.6702	0.012195	0.040389	0.301947	0.762692	0.821868	CCDC134	protein_cc
ENSG0000(	110.64	-0.48024	0.102221	-4.69801	2.63E-06	9.94E-06	PDE11A	protein_cc
ENSG0000(	110.5727	-0.15035	0.044497	-3.37879	0.000728	0.00182	AC141586	pseudoge
ENSG0000(	110.552	0.578373	0.093212	6.204949	5.47E-10	3.71E-09	TNN	protein_cc
ENSG0000(	110.5192	0.01307	0.036633	0.356774	0.721261	0.787896	SOS1-IT1	sense_intr
ENSG0000(	110.4755	-0.86707	0.130392	-6.6497	2.94E-11	2.43E-10	GS1-72M2	antisense
ENSG0000(	110.4025	0.064762	0.054285	1.192991	0.232873	0.317191	RNF139-A	lincRNA
ENSG0000(	110.3012	0.11776	0.062044	1.898011	0.057695	0.095779	TBX19	protein_cc
ENSG0000(	110.2217	0.005013	0.117832	0.042543	0.966066	0.976682	TRBC2	TR_C_gene
ENSG0000(	110.2072	-0.49987	0.06533	-7.65147	1.99E-14	2.73E-13	TP53TG5	protein_cc
ENSG0000(	110.1966	0.821057	0.09964	8.240239	1.72E-16	3.37E-15	RPL10AP6	pseudoge
ENSG0000(	110.016	0.021162	0.052108	0.406108	0.684663	0.757801	RP11-649.	antisense
ENSG0000(	109.8743	-0.06674	0.038403	-1.73787	0.082234	0.13026	RP11-867	protein_cc
ENSG0000(	109.8717	-0.03255	0.075801	-0.42944	0.667603	0.743481	RP1-59D1	antisense
ENSG0000(	109.8289	-0.21685	0.064721	-3.35051	0.000807	0.002	CTC-429P	sense_intr
ENSG0000(	109.7902	0.053821	0.040806	1.318951	0.187185	0.263918	AC097662	antisense
ENSG0000(	109.7515	0.278461	0.093926	2.964679	0.00303	0.006745	C18orf56	protein_cc
ENSG0000(	109.6603	-0.22311	0.096191	-2.31948	0.020369	0.037848	KLF5	protein_cc
ENSG0000(	109.6556	0.072834	0.098918	0.736305	0.461545	0.557678	KCNMB2	protein_cc
ENSG0000(	109.6234	0.852277	0.116302	7.328131	2.33E-13	2.67E-12	SOCS1	protein_cc
ENSG0000(	109.5262	1.430438	0.149418	9.57338	1.03E-21	4.74E-20	RASEF	protein_cc
ENSG0000(	109.51	0.084057	0.063914	1.315149	0.18846	0.265446	RP11-46A	antisense
ENSG0000(	109.5093	0.296668	0.066033	4.49272	7.03E-06	2.49E-05	CTD-2561	pseudoge
ENSG0000(	109.4976	-0.21764	0.062827	-3.46406	0.000532	0.001366	RP11-380	antisense
ENSG0000(	109.4912	-0.36267	0.130335	-2.78257	0.005393	0.011419	MSLN	protein_cc
ENSG0000(	109.2833	0.60455	0.105863	5.710671	1.13E-08	6.19E-08	SCXA	protein_cc
ENSG0000(	109.2632	0.159194	0.075468	2.109438	0.034907	0.061488	CDA	protein_cc
ENSG0000(	109.2573	0.093376	0.066953	1.394649	0.163122	0.234948	MARVELD	protein_cc
ENSG0000(	109.1929	0.398035	0.045682	8.713222	2.95E-18	7.67E-17	TRIM60P1	pseudoge
ENSG0000(	109.0463	0.599663	0.085548	7.009638	2.39E-12	2.35E-11	CTD-2267	protein_cc
ENSG0000(	108.9141	0.08778	0.03493	2.513008	0.011971	0.02349	RP11-143.	antisense
ENSG0000(	108.7753	-0.17054	0.083122	-2.05173	0.040195	0.069697	SATB2-AS	antisense
ENSG0000(	108.7674	-0.29423	0.121056	-2.43052	0.015077	0.028914	PAH	protein_cc
ENSG0000(	108.6728	-0.15262	0.045909	-3.32437	0.000886	0.002181	MRPS31P4	pseudoge
ENSG0000(	108.6715	0.033576	0.051669	0.649839	0.515796	0.608655	PPP5D1	protein_cc
ENSG0000(	108.6173	0.081997	0.050342	1.628809	0.103354	0.158973	RP11-333	antisense
ENSG0000(	108.5134	-0.14099	0.070623	-1.99635	0.045895	0.078322	A1BG-AS1	antisense
ENSG0000(	108.4567	-0.6283	0.096492	-6.51134	7.45E-11	5.79E-10	TMIGD2	protein_cc
ENSG0000(	108.4446	0.156553	0.080903	1.935067	0.052982	0.08891	HRCT1	protein_cc
ENSG0000(	108.3234	-0.28478	0.041681	-6.83245	8.35E-12	7.50E-11	ENTPD1-A	antisense
ENSG0000(	108.3077	0.04581	0.047589	0.96261	0.335743	0.430545	RP11-73K	antisense
ENSG0000(	108.264	-0.68163	0.092742	-7.34978	1.99E-13	2.29E-12	TBX18	protein_cc
ENSG0000(	108.0925	0.118595	0.046167	2.568801	0.010205	0.020338	AC069282	pseudoge
ENSG0000(	108.087	-0.15975	0.181974	-0.87789	0.380006	0.476517	KRT5	protein_cc
ENSG0000(	108.023	-0.08534	0.112128	-0.76112	0.446585	0.543434	PCDHGA7	protein_cc
ENSG0000(	108.0071	-0.00907	0.043872	-0.20666	0.836275	0.880826	C21orf49	protein_cc
ENSG0000(	107.9671	0.451184	0.076291	5.914029	3.34E-09	2.00E-08	AB019441	pseudoge
ENSG0000(	107.8268	0.003144	0.039697	0.079207	0.936868	0.955895	RP11-734	antisense
ENSG0000(	107.8081	0.971843	0.084847	11.45412	2.24E-30	5.66E-28	AKNAD1	protein_cc
ENSG0000(	107.7241	-0.06901	0.085737	-0.80486	0.420903	0.517898	FAM71E1	protein_cc
ENSG0000(	107.7027	-0.03911	0.115058	-0.33995	0.733896	0.79839	P2RY8	protein_cc
ENSG0000(	107.7022	0.071047	0.060783	1.16886	0.24246	0.328218	TOLLIP-AS	antisense
ENSG0000(	107.6183	-0.02098	0.073281	-0.2863	0.77465	0.831702	RP11-480.	lincRNA
ENSG0000(	107.5308	-0.69576	0.147645	-4.71239	2.45E-06	9.31E-06	CD209	protein_cc
ENSG0000(	107.53	0.464118	0.066094	7.022046	2.19E-12	2.16E-11	RP11-505	antisense
ENSG0000(	107.453	2.034244	0.212343	9.579972	9.71E-22	4.47E-20	HOXB4	protein_cc
ENSG0000(	107.3631	-0.16358	0.160169	-1.02129	0.307117	0.400155	RP11-958	pseudoge
ENSG0000(	107.3044	0.189692	0.092381	2.053381	0.040036	0.069449	MEIS3P1	pseudoge
ENSG0000(	107.2979	0.097819	0.097528	1.002986	0.315867	0.409461	LRRC43	protein_cc

ENSG0000(	107.2888	-0.20913	0.1084	-1.92927	0.053697	0.089937	WNT9A	protein_cc
ENSG0000(	107.2186	-0.13109	0.048359	-2.71079	0.006712	0.013924	LINC0063(	lincRNA
ENSG0000(	107.1355	-0.07186	0.054102	-1.32829	0.184082	0.260117	RP4-798A	lincRNA
ENSG0000(	107.1262	-0.22259	0.062639	-3.55359	0.00038	0.001004	RP3-399L	antisense
ENSG0000(	107.0764	0.232623	0.045763	5.083266	3.71E-07	1.61E-06	ABCA11P	pseudoge
ENSG0000(	107.0728	-0.56402	0.17642	-3.197	0.001389	0.003301	GJD2	protein_cc
ENSG0000(	106.9181	0.171039	0.057119	2.994424	0.00275	0.006171	RP11-499	lincRNA
ENSG0000(	106.8828	-0.23869	0.084533	-2.82366	0.004748	0.010155	CEACAM2	protein_cc
ENSG0000(	106.6002	-0.10502	0.078386	-1.33977	0.180322	0.255618	RP11-2E1	antisense
ENSG0000(	106.5958	-0.63829	0.102673	-6.21669	5.08E-10	3.46E-09	HS3ST5	protein_cc
ENSG0000(	106.5858	0.080208	0.110403	0.726505	0.467529	0.563386	TTC39A	protein_cc
ENSG0000(	106.5449	0.204511	0.07727	2.646724	0.008128	0.016578	RP11-430	lincRNA
ENSG0000(	106.5008	0.244768	0.049946	4.900633	9.55E-07	3.87E-06	CTD-2583	pseudoge
ENSG0000(	106.4068	-0.03719	0.226664	-0.16405	0.869689	0.906455	HOXA13	protein_cc
ENSG0000(	106.3211	-1.53329	0.168737	-9.08681	1.02E-19	3.33E-18	HTR5A	protein_cc
ENSG0000(	106.2902	-0.04857	0.071573	-0.67858	0.497404	0.591483	NPIPB4	protein_cc
ENSG0000(	106.2789	0.124481	0.074143	1.678925	0.093167	0.145269	RP11-274	lincRNA
ENSG0000(	106.2482	1.18647	0.144152	8.230681	1.86E-16	3.62E-15	DNAH11	protein_cc
ENSG0000(	106.1738	-0.17844	0.074744	-2.38729	0.016973	0.032154	BMS1P1	pseudoge
ENSG0000(	106.1561	0.093386	0.044816	2.08378	0.03718	0.065036	SIRT4	protein_cc
ENSG0000(	106.1504	-0.14447	0.089845	-1.60797	0.107842	0.164852	CYP2D6	protein_cc
ENSG0000(	106.0899	0.145222	0.032707	4.44016	8.99E-06	3.13E-05	OGFOD2	protein_cc
ENSG0000(	106.0052	0.581247	0.163419	3.55679	0.000375	0.000992	HBA1	protein_cc
ENSG0000(	105.996	0.45196	0.088041	5.133536	2.84E-07	1.25E-06	AC011242	pseudoge
ENSG0000(	105.9648	0.251895	0.061323	4.107674	4.00E-05	0.000125	RP1-20N2	antisense
ENSG0000(	105.9162	0.849976	0.119091	7.13719	9.53E-13	9.94E-12	LDHAP4	pseudoge
ENSG0000(	105.8658	0.531584	0.076742	6.926905	4.30E-12	4.05E-11	CYP3A5	protein_cc
ENSG0000(	105.8617	-0.06922	0.056989	-1.21459	0.224522	0.307764	RP11-13J1	sense_ove
ENSG0000(	105.8283	-0.13022	0.122899	-1.05959	0.289332	0.380705	EVA1A	protein_cc
ENSG0000(	105.8147	0.099627	0.082709	1.204545	0.228379	0.312177	RHCE	protein_cc
ENSG0000(	105.8045	0.34492	0.071322	4.836103	1.32E-06	5.25E-06	AC021188	antisense
ENSG0000(	105.7658	-0.20762	0.097193	-2.13617	0.032665	0.057907	CTD-2554	lincRNA
ENSG0000(	105.7614	-0.1876	0.068996	-2.71892	0.00655	0.013611	RP11-175	pseudoge
ENSG0000(	105.7147	-0.17773	0.155634	-1.14196	0.253472	0.340892	EMR1	protein_cc
ENSG0000(	105.6937	-0.51652	0.152312	-3.39117	0.000696	0.001746	EFCAB1	protein_cc
ENSG0000(	105.6509	-0.54162	0.090587	-5.97897	2.25E-09	1.38E-08	CCDC141	protein_cc
ENSG0000(	105.6443	-0.08872	0.051296	-1.72952	0.083716	0.132326	CTD-2010	pseudoge
ENSG0000(	105.6257	0.217854	0.082287	2.647484	0.008109	0.016544	RP11-175	pseudoge
ENSG0000(	105.4705	0.329311	0.045901	7.174323	7.27E-13	7.72E-12	RP11-565	sense_intr
ENSG0000(	105.3907	-0.02577	0.076079	-0.33869	0.734842	0.79925	ITGA2B	protein_cc
ENSG0000(	105.3467	0.201839	0.150348	1.342484	0.179439	0.254606	FCGR2C	polymorpl
ENSG0000(	105.3275	-0.15878	0.125838	-1.26179	0.207023	0.287563	KLRC3	protein_cc
ENSG0000(	105.2932	-0.9682	0.115068	-8.41411	3.96E-17	8.60E-16	VWA7	protein_cc
ENSG0000(	105.2852	-0.00924	0.078784	-0.1173	0.906624	0.933858	RIPK3	protein_cc
ENSG0000(	105.1826	0.20825	0.121914	1.708178	0.087603	0.137772	ASPN	protein_cc
ENSG0000(	105.0651	-1.4156	0.143243	-9.88257	4.95E-23	2.93E-21	VIP	protein_cc
ENSG0000(	105.0581	0.133165	0.048192	2.763244	0.005723	0.012042	TAS2R14	protein_cc
ENSG0000(	105.0329	0.067474	0.078029	0.864736	0.387184	0.483914	NPIPA7	protein_cc
ENSG0000(	105.0232	0.004069	0.039634	0.102664	0.918229	0.942324	RP11-727	antisense
ENSG0000(	105.0165	-0.04871	0.058643	-0.83054	0.406232	0.503037	MEF2C-AS	antisense
ENSG0000(	104.9865	-0.4582	0.110663	-4.14048	3.47E-05	0.000109	GJA5	protein_cc
ENSG0000(	104.9524	0.159533	0.061829	2.580234	0.009873	0.019752	RP11-262	lincRNA
ENSG0000(	104.9054	0.180827	0.040465	4.468743	7.87E-06	2.76E-05	ZNF763	protein_cc
ENSG0000(	104.888	-0.6702	0.100966	-6.63788	3.18E-11	2.62E-10	RNU6-52(	snRNA
ENSG0000(	104.8268	0.250059	0.038516	6.4923	8.45E-11	6.52E-10	MIPEPP3	pseudoge
ENSG0000(	104.7814	0.140335	0.055816	2.514245	0.011929	0.023417	RP11-452	lincRNA
ENSG0000(	104.7605	0.028245	0.110342	0.255974	0.797971	0.850678	MYOZ3	protein_cc
ENSG0000(	104.7549	-0.05918	0.124955	-0.47359	0.635789	0.716334	AP000525	processed
ENSG0000(	104.7018	0.364965	0.081522	4.476915	7.57E-06	2.67E-05	RP11-480	pseudoge

ENSG0000	104.5751	-0.63197	0.069759	-9.05933	1.31E-19	4.21E-18	RP11-760	lincRNA
ENSG0000	104.4869	-0.98278	0.132934	-7.39303	1.44E-13	1.70E-12	LILRB5	protein_cc
ENSG0000	104.4798	-0.20161	0.112624	-1.79014	0.073431	0.118161	FXYD2	protein_cc
ENSG0000	104.4433	0.139749	0.126214	1.107239	0.26819	0.357379	NPR3	protein_cc
ENSG0000	104.4002	-0.14032	0.222111	-0.63177	0.527538	0.619293	IGHG3	IG_C_gene
ENSG0000	104.369	0.097042	0.084257	1.151739	0.249428	0.336333	CYP2S1	protein_cc
ENSG0000	104.3322	0.393943	0.090779	4.339611	1.43E-05	4.81E-05	FOXS1	protein_cc
ENSG0000	104.3091	0.174438	0.060703	2.873639	0.004058	0.008814	CTC-338M	antisense
ENSG0000	104.2791	-0.57227	0.125477	-4.5608	5.10E-06	1.84E-05	RP11-126	lincRNA
ENSG0000	104.1601	-0.15454	0.073867	-2.09216	0.036424	0.063865	RP11-689	lincRNA
ENSG0000	104.1475	1.099394	0.093826	11.71737	1.04E-31	3.26E-29	OIP5	protein_cc
ENSG0000	104.1458	0.468832	0.062021	7.559202	4.06E-14	5.27E-13	APITD1	protein_cc
ENSG0000	104.1001	-0.24705	0.062647	-3.9435	8.03E-05	0.000239	RP1-283E	processed
ENSG0000	104.0762	0.168676	0.049624	3.399102	0.000676	0.001701	MYLK-AS1	antisense
ENSG0000	103.9479	-0.10377	0.07461	-1.39082	0.164281	0.236394	HMGN2P1	pseudoge
ENSG0000	103.9455	0.043208	0.059533	0.725793	0.467966	0.563814	ACRBP	protein_cc
ENSG0000	103.928	0.02077	0.10181	0.204006	0.838349	0.882422	RP11-279	lincRNA
ENSG0000	103.8861	0.199002	0.049752	3.999839	6.34E-05	0.000192	UCP3	protein_cc
ENSG0000	103.5632	0.631994	0.123161	5.131435	2.88E-07	1.27E-06	CYP19A1	protein_cc
ENSG0000	103.5441	0.374466	0.069582	5.381614	7.38E-08	3.57E-07	TEN1-CDK	processed
ENSG0000	103.4973	-0.09879	0.06513	-1.5168	0.129318	0.192517	RP11-458	lincRNA
ENSG0000	103.4959	-0.42848	0.074626	-5.74171	9.37E-09	5.22E-08	CTD-2647	sense_intr
ENSG0000	103.4775	0.809078	0.119031	6.797211	1.07E-11	9.42E-11	PSORS1C1	protein_cc
ENSG0000	103.3446	0.647364	0.085854	7.540267	4.69E-14	6.03E-13	PTMAP5	pseudoge
ENSG0000	103.32	-0.0943	0.131995	-0.71445	0.474948	0.570804	CCNA1	protein_cc
ENSG0000	103.3144	1.348318	0.128451	10.49679	8.94E-26	9.23E-24	NEIL3	protein_cc
ENSG0000	103.2658	-0.97929	0.09945	-9.84706	7.06E-23	4.06E-21	LHFPL1	protein_cc
ENSG0000	103.1919	0.062364	0.059464	1.04876	0.294288	0.386064	RP11-379	lincRNA
ENSG0000	103.1327	0.251848	0.095304	2.642564	0.008228	0.016768	DPY19L2P	pseudoge
ENSG0000	103.0859	-0.50463	0.080191	-6.2929	3.12E-10	2.20E-09	FZD9	protein_cc
ENSG0000	103.0417	0.503649	0.072195	6.976264	3.03E-12	2.93E-11	BTN2A3P	pseudoge
ENSG0000	103.0203	0.214057	0.061515	3.479775	0.000502	0.001294	TMEM45B	protein_cc
ENSG0000	102.9839	0.591588	0.152266	3.885227	0.000102	0.000298	PLEKHG4E	protein_cc
ENSG0000	102.9267	0.631578	0.121668	5.190993	2.09E-07	9.41E-07	EFCAB12	protein_cc
ENSG0000	102.9046	0.087628	0.088192	0.993611	0.320412	0.41419	HSPB2	protein_cc
ENSG0000	102.8804	0.127706	0.045471	2.808501	0.004977	0.01061	AC239860	protein_cc
ENSG0000	102.8783	0.661671	0.121282	5.455659	4.88E-08	2.43E-07	DPY19L2P	pseudoge
ENSG0000	102.792	0.756288	0.115317	6.558362	5.44E-11	4.31E-10	PPIAP29	pseudoge
ENSG0000	102.7833	-0.17445	0.060575	-2.87998	0.003977	0.008656	C16orf96	protein_cc
ENSG0000	102.7742	-0.07926	0.044768	-1.77057	0.076633	0.12261	CTD-2630	antisense
ENSG0000	102.7622	-0.19855	0.121034	-1.64041	0.10092	0.15576	SNAI1	protein_cc
ENSG0000	102.7058	-0.37535	0.156276	-2.40185	0.016312	0.031049	SLC14A2	protein_cc
ENSG0000	102.6717	0.079989	0.102674	0.779061	0.435944	0.532993	TMEM74	protein_cc
ENSG0000	102.6537	0.057231	0.133517	0.428643	0.668183	0.743885	CLEC4F	protein_cc
ENSG0000	102.6499	-1.4167	0.184464	-7.68007	1.59E-14	2.22E-13	TNNT2	protein_cc
ENSG0000	102.642	-0.19394	0.228864	-0.8474	0.39677	0.493416	TTTY14	lincRNA
ENSG0000	102.6332	-0.50901	0.078306	-6.50034	8.01E-11	6.20E-10	C15orf27	protein_cc
ENSG0000	102.6166	-0.68157	0.105576	-6.45574	1.08E-10	8.17E-10	PDE4C	protein_cc
ENSG0000	102.5935	-0.0015	0.079601	-0.01883	0.984975	0.989702	AC006547	protein_cc
ENSG0000	102.5841	-0.03361	0.064297	-0.52275	0.601146	0.685814	LRP5L	protein_cc
ENSG0000	102.5118	0.122927	0.04513	2.723861	0.006452	0.013431	PPT2-EGF	protein_cc
ENSG0000	102.4659	0.395167	0.094506	4.181397	2.90E-05	9.27E-05	SKIDA1	protein_cc
ENSG0000	102.3952	0.106467	0.051558	2.064978	0.038925	0.067728	AC002310	antisense
ENSG0000	102.3933	-0.06206	0.074712	-0.83067	0.406162	0.502967	RP11-434	lincRNA
ENSG0000	102.2496	-0.77128	0.140681	-5.48249	4.19E-08	2.11E-07	TLL1	protein_cc
ENSG0000	102.1426	0.6129	0.071975	8.515471	1.66E-17	3.83E-16	LINC0102	lincRNA
ENSG0000	102.1151	-1.84679	0.175902	-10.499	8.73E-26	9.05E-24	KIAA1239	protein_cc
ENSG0000	102.0857	0.500699	0.08167	6.130757	8.75E-10	5.76E-09	CTC-451P	pseudoge
ENSG0000	102.006	-0.24244	0.113036	-2.14475	0.031973	0.056792	CD8A	protein_cc

ENSG0000(	101.999	-0.0092	0.065765	-0.13996	0.88869	0.920706	HMGB1P1	pseudoge
ENSG0000(	101.9404	0.68075	0.096344	7.065829	1.60E-12	1.61E-11	HSP90AB3	pseudoge
ENSG0000(	101.922	0.270118	0.056465	4.783822	1.72E-06	6.70E-06	RP11-27I1	pseudoge
ENSG0000(	101.8227	0.077677	0.039874	1.948046	0.05141	0.086589	TSTD3	antisense
ENSG0000(	101.8045	-0.13217	0.066333	-1.99257	0.046309	0.078945	GNMT	protein_cc
ENSG0000(	101.8039	0.271621	0.034229	7.935417	2.10E-15	3.44E-14	ZNF563	protein_cc
ENSG0000(	101.6871	-0.23249	0.07684	-3.02559	0.002481	0.005629	CYSLTR1	protein_cc
ENSG0000(	101.6679	-0.12846	0.090111	-1.42554	0.154002	0.223859	STX11	protein_cc
ENSG0000(	101.6608	-0.72749	0.086123	-8.44709	2.99E-17	6.64E-16	SYT15	protein_cc
ENSG0000(	101.6354	-0.55256	0.113299	-4.877	1.08E-06	4.33E-06	POU3F1	protein_cc
ENSG0000(	101.619	0.421359	0.090819	4.639563	3.49E-06	1.30E-05	RP11-108	lincRNA
ENSG0000(	101.6185	0.265124	0.034724	7.635167	2.26E-14	3.06E-13	UBL7-AS1	antisense
ENSG0000(	101.5789	-0.16612	0.113953	-1.45782	0.144889	0.212323	AP000688	lincRNA
ENSG0000(	101.5408	-0.10961	0.119169	-0.91982	0.357668	0.45343	MTND6P4	pseudoge
ENSG0000(	101.4385	0.057113	0.037458	1.524726	0.127327	0.190159	CTD-2192	protein_cc
ENSG0000(	101.4338	-0.13689	0.134853	-1.01508	0.310069	0.403305	PNMT	protein_cc
ENSG0000(	101.4064	-0.64209	0.085902	-7.47463	7.74E-14	9.61E-13	GCNT4	protein_cc
ENSG0000(	101.3886	0.189953	0.119166	1.594022	0.110931	0.168973	ZBBX	protein_cc
ENSG0000(	101.2242	-0.1276	0.054068	-2.36	0.018275	0.034334	CCDC183-	antisense
ENSG0000(	101.2103	0.500741	0.127597	3.924404	8.69E-05	0.000257	RP1-140K	lincRNA
ENSG0000(	101.172	-0.51305	0.114704	-4.47285	7.72E-06	2.71E-05	CYP21A1F	pseudoge
ENSG0000(	101.1167	-0.53999	0.10501	-5.1423	2.71E-07	1.20E-06	PLK5	protein_cc
ENSG0000(	101.1094	0.549265	0.061296	8.960836	3.22E-19	9.66E-18	NSUN5P2	pseudoge
ENSG0000(	101.0545	0.020257	0.102663	0.197313	0.843583	0.886429	AC011899	antisense
ENSG0000(	101.046	0.118586	0.046696	2.539533	0.0111	0.021953	SLC16A13	protein_cc
ENSG0000(	101.0114	-0.47093	0.135138	-3.48483	0.000492	0.001272	OLFM4	protein_cc
ENSG0000(	101.0093	-0.17057	0.089453	-1.90678	0.056549	0.094083	C5orf64	protein_cc
ENSG0000(	101.0053	-0.0586	0.066177	-0.88547	0.375902	0.472408	OR7E38P	pseudoge
ENSG0000(	100.9573	-0.04563	0.07964	-0.57291	0.566706	0.654678	TRIM7	protein_cc
ENSG0000(	100.9344	-0.30374	0.085119	-3.56844	0.000359	0.000953	TBC1D8B	protein_cc
ENSG0000(	100.8986	-0.27641	0.109387	-2.5269	0.011507	0.022675	RP11-253	processed
ENSG0000(	100.8548	-0.14634	0.125203	-1.16879	0.242489	0.328235	RP11-166	protein_cc
ENSG0000(	100.8417	0.00867	0.050226	0.172622	0.862948	0.901257	RP11-118	processed
ENSG0000(	100.7635	0.023974	0.044506	0.538681	0.590107	0.675939	RP11-278	pseudoge
ENSG0000(	100.7531	0.302485	0.051536	5.869363	4.37E-09	2.57E-08	U47924.31	antisense
ENSG0000(	100.7501	0.473461	0.092827	5.100457	3.39E-07	1.48E-06	LINC0088(	lincRNA
ENSG0000(	100.7349	-0.44637	0.124445	-3.58691	0.000335	0.000893	SLC27A6	protein_cc
ENSG0000(	100.5899	-0.00813	0.055855	-0.14549	0.884328	0.917431	AC010536	protein_cc
ENSG0000(	100.5816	-0.0832	0.084463	-0.98505	0.324601	0.418789	RP3-466P	lincRNA
ENSG0000(	100.5753	-0.00171	0.076646	-0.02226	0.982238	0.987738	TSPAN32	protein_cc
ENSG0000(	100.5692	-0.4011	0.079545	-5.04241	4.60E-07	1.96E-06	NIPSNAP3	protein_cc
ENSG0000(	100.5156	0.155955	0.057878	2.694558	0.007048	0.014552	RP11-16E	lincRNA
ENSG0000(	100.5092	0.560781	0.144316	3.885781	0.000102	0.000297	GPR82	protein_cc
ENSG0000(	100.4507	0.033933	0.035277	0.961879	0.33611	0.430934	OAZ3	protein_cc
ENSG0000(	100.439	-0.49616	0.101534	-4.88668	1.03E-06	4.14E-06	AP000439	lincRNA
ENSG0000(	100.433	-0.85832	0.094635	-9.06983	1.19E-19	3.85E-18	ESYT3	protein_cc
ENSG0000(	100.4138	0.146059	0.05208	2.804502	0.005039	0.010735	KCTD21-A	antisense
ENSG0000(	100.4016	-0.37952	0.049464	-7.67275	1.68E-14	2.33E-13	CTD-2555	antisense
ENSG0000(	100.3887	-0.15571	0.055046	-2.82864	0.004675	0.010012	RP11-159	protein_cc
ENSG0000(	100.3516	-0.10806	0.045129	-2.39448	0.016644	0.031605	CATSPER2	pseudoge
ENSG0000(	100.311	0.554426	0.241179	2.298817	0.021515	0.039771	PI3	protein_cc
ENSG0000(	100.304	0.009442	0.150144	0.062886	0.949857	0.96535	CDH15	protein_cc
ENSG0000(	100.286	0.909102	0.112879	8.053743	8.03E-16	1.40E-14	RPS11P5	pseudoge
ENSG0000(	100.2777	-0.11862	0.070449	-1.6838	0.092221	0.143997	NKX3-1	protein_cc
ENSG0000(	100.2519	2.493712	0.238636	10.44986	1.47E-25	1.43E-23	OTP	protein_cc
ENSG0000(	100.2439	0.344424	0.07659	4.496987	6.89E-06	2.44E-05	FAM124B	protein_cc
ENSG0000(	100.2379	-0.04679	0.046575	-1.00453	0.315125	0.408665	SERHL2	protein_cc
ENSG0000(	100.1674	0.192072	0.050062	3.836692	0.000125	0.000358	RPS18P9	pseudoge
ENSG0000(	100.1562	-0.22855	0.050346	-4.53957	5.64E-06	2.03E-05	DENND2C	protein_cc

ENSG0000(	100.1401	0.056766	0.067557	0.840262	0.400762	0.497587	RP11-967	antisense
ENSG0000(	100.0947	0.027974	0.054794	0.510529	0.609681	0.693377	CBX3P2	pseudoge
ENSG0000(	100.0578	-0.07794	0.10854	-0.71808	0.472707	0.568559	MTND5P1	pseudoge
ENSG0000(	100.0141	-0.0204	0.083627	-0.24395	0.807269	0.858099	CTB-176F	antisense
ENSG0000(	99.948	0.125504	0.043659	2.874673	0.004044	0.008789	SSSCA1-A	antisense
ENSG0000(	99.87347	0.627763	0.202167	3.105172	0.001902	0.004405	ADAMDE(	protein_cc
ENSG0000(	99.74687	-0.65486	0.109034	-6.00605	1.90E-09	1.18E-08	RP11-168	lincRNA
ENSG0000(	99.71591	-0.05436	0.060955	-0.89181	0.372494	0.468942	ABHD1	protein_cc
ENSG0000(	99.69818	0.217742	0.12758	1.706713	0.087875	0.138168	NPIPB15	protein_cc
ENSG0000(	99.63482	0.088995	0.058086	1.532109	0.125495	0.187777	POT1-AS1	antisense
ENSG0000(	99.44703	-0.09459	0.050982	-1.8553	0.063554	0.104189	ZNF805	protein_cc
ENSG0000(	99.39087	-0.03621	0.038846	-0.93206	0.351304	0.446792	FTX	lincRNA
ENSG0000(	99.3541	-0.56164	0.120828	-4.64823	3.35E-06	1.25E-05	KY	protein_cc
ENSG0000(	99.33821	0.094855	0.04681	2.026394	0.042724	0.073545	NKAPP1	pseudoge
ENSG0000(	99.25986	0.193815	0.05852	3.311971	0.000926	0.00227	NFYC-AS1	antisense
ENSG0000(	99.24112	-0.23278	0.0875	-2.66034	0.007806	0.015985	C1orf204	protein_cc
ENSG0000(	99.23754	-0.12678	0.077525	-1.63529	0.101988	0.157156	RP11-408.	lincRNA
ENSG0000(	99.22453	-0.5544	0.130416	-4.25106	2.13E-05	6.97E-05	AC009227	antisense
ENSG0000(	99.16623	-0.40113	0.088776	-4.5184	6.23E-06	2.22E-05	RP11-513	antisense
ENSG0000(	99.14153	0.533626	0.083235	6.411074	1.44E-10	1.07E-09	C1QTNF2	protein_cc
ENSG0000(	99.03002	0.085493	0.040422	2.115029	0.034428	0.060725	HMGN3-/	antisense
ENSG0000(	99.01086	0.110481	0.048944	2.257287	0.02399	0.043875	PMF1-BGI	protein_cc
ENSG0000(	99.00647	0.768331	0.254682	3.016828	0.002554	0.005781	AC010970	pseudoge
ENSG0000(	98.97645	0.162406	0.122472	1.326069	0.184817	0.261048	SIRPB1	protein_cc
ENSG0000(	98.9324	0.240746	0.077156	3.120229	0.001807	0.004204	VPS9D1-/	antisense
ENSG0000(	98.85218	0.385534	0.054298	7.100324	1.24E-12	1.28E-11	RP11-539	lincRNA
ENSG0000(	98.75651	-1.15651	0.156193	-7.40439	1.32E-13	1.57E-12	SCARA5	protein_cc
ENSG0000(	98.75505	0.12841	0.150896	0.850985	0.394778	0.491325	RP5-1177	lincRNA
ENSG0000(	98.74806	-0.05106	0.088274	-0.57838	0.563007	0.651598	TREML1	protein_cc
ENSG0000(	98.73683	-0.19405	0.09728	-1.99473	0.046073	0.078595	DAPP1	protein_cc
ENSG0000(	98.60382	0.044111	0.102327	0.431082	0.666409	0.742513	DYNLRB2	protein_cc
ENSG0000(	98.49331	0.285702	0.04931	5.794059	6.87E-09	3.91E-08	CTD-3222	lincRNA
ENSG0000(	98.43012	-0.02664	0.043606	-0.61088	0.541278	0.632032	RP11-327	antisense
ENSG0000(	98.42085	-0.23695	0.097144	-2.43913	0.014723	0.028323	RP11-46H	lincRNA
ENSG0000(	98.34277	0.129053	0.124877	1.033436	0.3014	0.394035	GPR84	protein_cc
ENSG0000(	98.33571	0.225109	0.062366	3.609473	0.000307	0.000824	RP3-329A	lincRNA
ENSG0000(	98.33467	-0.21945	0.059964	-3.65967	0.000253	0.000688	FBXL22	protein_cc
ENSG0000(	98.32427	1.33267	0.116507	11.43859	2.68E-30	6.53E-28	FKBP9L	pseudoge
ENSG0000(	98.2619	0.214207	0.052141	4.108199	3.99E-05	0.000125	APLF	protein_cc
ENSG0000(	98.18463	0.414088	0.175566	2.358589	0.018345	0.034452	MAP3K19	protein_cc
ENSG0000(	98.1785	0.25356	0.057281	4.426571	9.57E-06	3.31E-05	LEAP2	protein_cc
ENSG0000(	98.13618	0.015219	0.048145	0.316112	0.751918	0.813177	ALOX12	protein_cc
ENSG0000(	98.05508	0.586327	0.125412	4.675209	2.94E-06	1.10E-05	FTH1P2	pseudoge
ENSG0000(	98.03447	-0.52847	0.149803	-3.52777	0.000419	0.001098	LINC0094:	lincRNA
ENSG0000(	97.96667	0.059512	0.077324	0.769648	0.441509	0.538324	RP11-21L:	sense_ove
ENSG0000(	97.91103	-0.14113	0.061315	-2.30169	0.021352	0.039492	RP11-536	antisense
ENSG0000(	97.89721	0.215861	0.152733	1.413322	0.157561	0.228072	DLX6	protein_cc
ENSG0000(	97.86651	-1.22442	0.174278	-7.02566	2.13E-12	2.11E-11	SOHLH1	protein_cc
ENSG0000(	97.79616	-0.02951	0.063123	-0.46752	0.640129	0.720197	AC007566	antisense
ENSG0000(	97.78106	0.111693	0.057964	1.92695	0.053986	0.090366	PLEKHA8F	pseudoge
ENSG0000(	97.77842	0.107318	0.048862	2.196341	0.028068	0.050503	CTC-559E	antisense
ENSG0000(	97.75632	0.090353	0.042233	2.139415	0.032402	0.057485	AP000254	antisense
ENSG0000(	97.71239	0.295029	0.090669	3.253893	0.001138	0.002745	TTC34	protein_cc
ENSG0000(	97.59769	0.073762	0.045242	1.630384	0.10302	0.158534	SSR4P1	pseudoge
ENSG0000(	97.54545	0.039923	0.057085	0.699357	0.484329	0.579386	RP4-756G	antisense
ENSG0000(	97.50557	-0.3143	0.056907	-5.52301	3.33E-08	1.70E-07	SH3RF3-A	lincRNA
ENSG0000(	97.43832	-0.33603	0.074847	-4.48957	7.14E-06	2.52E-05	C11orf45	protein_cc
ENSG0000(	97.38779	-0.021	0.03974	-0.52838	0.597238	0.682359	SPAG5-AS	processed
ENSG0000(	97.38239	-0.1303	0.088266	-1.47624	0.139881	0.205964	AC007228	lincRNA

ENSG0000(	97.32003	-0.21842	0.139021	-1.57113	0.116154	0.175834	BEND4	protein_cc
ENSG0000(	97.26984	-0.20776	0.074625	-2.78408	0.005368	0.011371	FAM115B	pseudoge
ENSG0000(	97.14292	-1.50832	0.163983	-9.19805	3.64E-20	1.27E-18	STYK1	protein_cc
ENSG0000(	97.13788	-0.08762	0.066269	-1.32224	0.18609	0.262584	RP11-254	lincRNA
ENSG0000(	97.05416	0.938151	0.079027	11.87127	1.67E-32	6.07E-30	RP1-65J1	lincRNA
ENSG0000(	97.04562	1.059918	0.088103	12.0304	2.46E-33	1.14E-30	KCTD14	protein_cc
ENSG0000(	97.04136	0.519194	0.144082	3.60347	0.000314	0.000842	TDO2	protein_cc
ENSG0000(	97.02067	-0.11386	0.064513	-1.76497	0.077568	0.123958	HRC	protein_cc
ENSG0000(	96.95473	0.312476	0.092656	3.372432	0.000745	0.001858	CLDN1	protein_cc
ENSG0000(	96.87296	0.320145	0.087075	3.676682	0.000236	0.000647	RP11-143.	lincRNA
ENSG0000(	96.81142	0.029202	0.123452	0.236546	0.813009	0.862639	VNN2	protein_cc
ENSG0000(	96.79214	-0.11768	0.115227	-1.02128	0.307122	0.400155	DACT2	protein_cc
ENSG0000(	96.7321	0.377535	0.062242	6.065601	1.31E-09	8.42E-09	NAALADL	protein_cc
ENSG0000(	96.59984	-0.28373	0.087137	-3.25618	0.001129	0.002725	FFAR4	protein_cc
ENSG0000(	96.58662	0.169504	0.151274	1.120506	0.262498	0.351079	FAM180A	protein_cc
ENSG0000(	96.57764	0.009827	0.134745	0.072929	0.941863	0.959359	BTC	protein_cc
ENSG0000(	96.53462	0.997825	0.098414	10.13901	3.71E-24	2.74E-22	MND1	protein_cc
ENSG0000(	96.50133	0.717244	0.116568	6.153032	7.60E-10	5.06E-09	ZBED6CL	protein_cc
ENSG0000(	96.4242	-0.16653	0.052284	-3.18507	0.001447	0.003427	UBAC2-AS	lincRNA
ENSG0000(	96.40152	-0.07143	0.068545	-1.04209	0.29737	0.389485	TSSK5P1	pseudoge
ENSG0000(	96.3809	0.299958	0.083571	3.589266	0.000332	0.000886	EFNA2	protein_cc
ENSG0000(	96.35058	-0.1721	0.146229	-1.17694	0.239218	0.324523	KCNH7	protein_cc
ENSG0000(	96.32325	0.719687	0.105351	6.831333	8.41E-12	7.56E-11	AC022431	pseudoge
ENSG0000(	96.29313	-1.29633	0.110732	-11.7069	1.17E-31	3.58E-29	HRH2	protein_cc
ENSG0000(	96.28977	0.415779	0.101709	4.087922	4.35E-05	0.000135	RP11-86H	lincRNA
ENSG0000(	96.152	0.022872	0.072787	0.314239	0.75334	0.814222	SAMD15	protein_cc
ENSG0000(	96.12765	0.117215	0.195252	0.600327	0.548288	0.638404	AC104135	lincRNA
ENSG0000(	96.05297	0.680213	0.130702	5.204296	1.95E-07	8.81E-07	NHLH1	protein_cc
ENSG0000(	96.00959	0.185553	0.057574	3.222843	0.001269	0.003036	TIGD4	protein_cc
ENSG0000(	96.00876	0.327232	0.04832	6.772138	1.27E-11	1.11E-10	STAG3L3	pseudoge
ENSG0000(	95.98624	0.026825	0.046709	0.574298	0.565766	0.653812	ZNF490	protein_cc
ENSG0000(	95.93276	-1.11422	0.157159	-7.08977	1.34E-12	1.37E-11	CDH9	protein_cc
ENSG0000(	95.85434	0.347393	0.050694	6.852809	7.24E-12	6.58E-11	AC005076	antisense
ENSG0000(	95.76209	0.274893	0.049303	5.57557	2.47E-08	1.29E-07	PIK3R2	protein_cc
ENSG0000(	95.73617	1.241191	0.159928	7.760926	8.43E-15	1.25E-13	RPS18P12	pseudoge
ENSG0000(	95.72118	-0.1437	0.0769	-1.86866	0.06167	0.101441	RP4-813D	lincRNA
ENSG0000(	95.71992	-1.46254	0.160645	-9.10415	8.69E-20	2.86E-18	KRT222	protein_cc
ENSG0000(	95.71656	0.506871	0.099107	5.114386	3.15E-07	1.38E-06	TRIM6	protein_cc
ENSG0000(	95.60793	-0.098	0.130375	-0.75168	0.452244	0.548875	TSPAN2	protein_cc
ENSG0000(	95.60108	-0.10087	0.061935	-1.62868	0.10338	0.159008	YEATS2-A	antisense
ENSG0000(	95.5974	0.881287	0.116406	7.570803	3.71E-14	4.85E-13	C6orf118	protein_cc
ENSG0000(	95.58782	-0.10709	0.126402	-0.8472	0.396884	0.493498	FCRLA	protein_cc
ENSG0000(	95.57151	-0.79654	0.092901	-8.57402	9.99E-18	2.40E-16	RAB11FIP	protein_cc
ENSG0000(	95.34167	-0.57425	0.140036	-4.10075	4.12E-05	0.000129	ROR2	protein_cc
ENSG0000(	95.32874	0.147051	0.102268	1.437903	0.150461	0.219363	MIR145	lincRNA
ENSG0000(	95.19879	0.128415	0.052808	2.431723	0.015027	0.028832	RP11-705	sense_intr
ENSG0000(	95.18307	0.157679	0.041042	3.841912	0.000122	0.000351	ZNF223	protein_cc
ENSG0000(	95.18249	-0.02372	0.053753	-0.4412	0.659068	0.736231	RP4-605C	processed
ENSG0000(	95.16759	0.192185	0.113805	1.688715	0.091274	0.142726	ZNF257	protein_cc
ENSG0000(	95.155	0.036047	0.080759	0.44635	0.655344	0.733124	GNGT2	protein_cc
ENSG0000(	95.10732	-0.03917	0.105734	-0.37046	0.71104	0.779609	FCGR1C	protein_cc
ENSG0000(	95.10119	-0.45913	0.104522	-4.39268	1.12E-05	3.84E-05	TDH	pseudoge
ENSG0000(	94.91647	-0.13593	0.164384	-0.82693	0.408277	0.505069	IL6	protein_cc
ENSG0000(	94.86871	0.044729	0.058326	0.766872	0.443158	0.539938	LL0XNC01	lincRNA
ENSG0000(	94.81794	-0.1601	0.109832	-1.45767	0.144932	0.212371	SLC26A4	protein_cc
ENSG0000(	94.81615	-0.11501	0.051364	-2.2391	0.02515	0.045788	RRN3P3	pseudoge
ENSG0000(	94.76307	1.108152	0.134579	8.234198	1.81E-16	3.52E-15	RPL5P34	pseudoge
ENSG0000(	94.74054	-0.91829	0.114062	-8.05085	8.22E-16	1.43E-14	AC141928	lincRNA
ENSG0000(	94.7211	0.954336	0.11681	8.169982	3.08E-16	5.78E-15	RP11-778	pseudoge

ENSG0000(	94.70556	-0.35651	0.089584	-3.97958	6.90E-05	0.000208	KLF8	protein_cc
ENSG0000(	94.64707	-0.12799	0.049404	-2.59073	0.009577	0.019216	RP11-78C	lincRNA
ENSG0000(	94.63288	-0.06103	0.040324	-1.51354	0.130143	0.193542	SLC9B1	protein_cc
ENSG0000(	94.58658	-0.32486	0.128569	-2.52673	0.011513	0.022685	RBM24	protein_cc
ENSG0000(	94.54514	0.060232	0.082623	0.728997	0.466004	0.56186	RP11-250	lincRNA
ENSG0000(	94.52947	-0.40983	0.081524	-5.02704	4.98E-07	2.11E-06	SPNS3	protein_cc
ENSG0000(	94.4149	1.395627	0.099864	13.97533	2.20E-44	4.77E-41	RP11-375	antisense
ENSG0000(	94.33933	-0.61048	0.157138	-3.885	0.000102	0.000298	PCDHA6	protein_cc
ENSG0000(	94.12293	0.22789	0.097201	2.344525	0.019051	0.035638	CACNG6	protein_cc
ENSG0000(	94.11559	0.396599	0.226059	1.754404	0.079361	0.126395	GALNT5	protein_cc
ENSG0000(	93.95772	2.475027	0.189525	13.05907	5.64E-39	7.00E-36	SCNN1B	protein_cc
ENSG0000(	93.95401	-0.17721	0.070207	-2.52403	0.011602	0.022843	CTD-2619	antisense
ENSG0000(	93.95335	0.520167	0.082969	6.26939	3.62E-10	2.53E-09	HSD11B2	protein_cc
ENSG0000(	93.86487	0.069115	0.100767	0.685892	0.492781	0.587208	RHOH	protein_cc
ENSG0000(	93.82878	-0.05814	0.089602	-0.64882	0.516457	0.609259	PPM1J	protein_cc
ENSG0000(	93.78923	-0.69627	0.110566	-6.29736	3.03E-10	2.14E-09	GPR68	protein_cc
ENSG0000(	93.71061	-0.23082	0.099637	-2.31661	0.020525	0.03811	AC004019	antisense
ENSG0000(	93.68747	0.096722	0.119615	0.808609	0.41874	0.5157	COL25A1	protein_cc
ENSG0000(	93.62669	0.267011	0.161913	1.649107	0.099126	0.153358	AC016708	pseudoge
ENSG0000(	93.61669	-0.53323	0.109399	-4.87419	1.09E-06	4.39E-06	STAR	protein_cc
ENSG0000(	93.61347	-0.24798	0.092102	-2.6924	0.007094	0.014639	SIGLEC11	protein_cc
ENSG0000(	93.61299	0.204898	0.106476	1.924349	0.054311	0.090844	ANKEF1	protein_cc
ENSG0000(	93.60983	-0.16534	0.217722	-0.7594	0.447614	0.544364	IL2RA	protein_cc
ENSG0000(	93.57692	0.350351	0.11355	3.085426	0.002033	0.004685	RP11-224	antisense
ENSG0000(	93.49468	0.099151	0.063893	1.551832	0.120702	0.181583	ANKLE1	protein_cc
ENSG0000(	93.49088	-1.16328	0.13035	-8.9243	4.49E-19	1.32E-17	KANK4	protein_cc
ENSG0000(	93.46031	0.207534	0.043202	4.803828	1.56E-06	6.11E-06	DDTL	protein_cc
ENSG0000(	93.42412	-0.09833	0.081959	-1.19971	0.230252	0.314241	VEGFC	protein_cc
ENSG0000(	93.34548	0.222526	0.049885	4.460805	8.17E-06	2.86E-05	CTA-211A	lincRNA
ENSG0000(	93.33226	0.332016	0.107889	3.077395	0.002088	0.004803	WFDC2	protein_cc
ENSG0000(	93.27903	-0.08111	0.072857	-1.11324	0.265607	0.354442	AC034228	antisense
ENSG0000(	93.03656	1.107256	0.119274	9.283315	1.64E-20	6.06E-19	E2F8	protein_cc
ENSG0000(	93.03501	-0.19687	0.094971	-2.07291	0.03818	0.066604	LINC0086!	lincRNA
ENSG0000(	93.0109	0.858602	0.111333	7.712034	1.24E-14	1.77E-13	APOBEC3I	protein_cc
ENSG0000(	92.99997	-0.06339	0.045252	-1.40078	0.161281	0.232639	NAPA-AS:	antisense
ENSG0000(	92.90312	-0.37723	0.149021	-2.53136	0.011362	0.022413	TRDN	protein_cc
ENSG0000(	92.89388	-0.56457	0.069802	-8.08816	6.06E-16	1.08E-14	CEBPA-AS	antisense
ENSG0000(	92.86652	0.798562	0.166083	4.808211	1.52E-06	5.98E-06	NPY2R	protein_cc
ENSG0000(	92.835	1.613377	0.148579	10.85869	1.81E-27	2.53E-25	SNRPGP1(	pseudoge
ENSG0000(	92.80278	0.26384	0.089107	2.960918	0.003067	0.006822	HLA-J	pseudoge
ENSG0000(	92.73286	-0.06846	0.044954	-1.523	0.12776	0.190657	RP1-197B	lincRNA
ENSG0000(	92.69961	0.136728	0.097647	1.400225	0.161446	0.232836	TNFRSF10	protein_cc
ENSG0000(	92.56816	0.032363	0.099763	0.3244	0.745636	0.808211	FNBP1P1	pseudoge
ENSG0000(	92.53677	-0.40677	0.071699	-5.67326	1.40E-08	7.60E-08	CDNF	protein_cc
ENSG0000(	92.53594	-0.104	0.072044	-1.44357	0.148859	0.217382	ZC3H12D	protein_cc
ENSG0000(	92.50627	-0.1842	0.104026	-1.77074	0.076604	0.122587	SERPINI2	protein_cc
ENSG0000(	92.40645	-0.10415	0.060539	-1.72034	0.085371	0.134637	GUSBP11	processed
ENSG0000(	92.34923	-0.07496	0.089509	-0.83746	0.402334	0.499113	GAS6-AS1	antisense
ENSG0000(	92.3365	0.397406	0.080658	4.927053	8.35E-07	3.41E-06	NAT1	protein_cc
ENSG0000(	92.32045	0.545644	0.177384	3.076066	0.002098	0.004821	WDR38	protein_cc
ENSG0000(	92.28633	0.190378	0.05123	3.716132	0.000202	0.00056	RP11-589	lincRNA
ENSG0000(	92.19736	-0.16843	0.097855	-1.72119	0.085216	0.134418	RP1-279N	pseudoge
ENSG0000(	92.12216	0.026763	0.047361	0.565081	0.572018	0.659481	RP11-712	antisense
ENSG0000(	92.10317	0.022765	0.069584	0.327155	0.743551	0.806461	RP11-203.	antisense
ENSG0000(	92.08139	-1.52276	0.188448	-8.08053	6.45E-16	1.15E-14	KB-1517D	antisense
ENSG0000(	92.00557	-0.42403	0.077429	-5.47628	4.34E-08	2.18E-07	AGMAT	protein_cc
ENSG0000(	91.95016	0.987146	0.102441	9.636212	5.62E-22	2.69E-20	MGARP	protein_cc
ENSG0000(	91.89453	0.084133	0.061075	1.377524	0.16835	0.241354	RP11-863	pseudoge
ENSG0000(	91.87303	0.209489	0.032272	6.491295	8.51E-11	6.56E-10	CTC-559E	processed

ENSG0000(	91.8248	-1.02387	0.120539	-8.49413	1.99E-17	4.55E-16	OMD	protein_cc
ENSG0000(	91.78489	-0.55552	0.092301	-6.0186	1.76E-09	1.10E-08	EML5	protein_cc
ENSG0000(	91.76727	-0.70741	0.098764	-7.16269	7.91E-13	8.35E-12	ADRA2C	protein_cc
ENSG0000(	91.70584	0.329729	0.096766	3.407493	0.000656	0.001654	RP11-160	lincRNA
ENSG0000(	91.66127	1.539572	0.134487	11.44773	2.41E-30	6.02E-28	CNPY1	protein_cc
ENSG0000(	91.64822	-0.04944	0.057148	-0.86518	0.386938	0.483708	ACVR2B-/	antisense
ENSG0000(	91.51591	0.085478	0.076025	1.124347	0.260866	0.34918	NACA3P	pseudoge
ENSG0000(	91.44115	0.353164	0.067574	5.226296	1.73E-07	7.89E-07	ERICH2	protein_cc
ENSG0000(	91.39326	-0.13502	0.068109	-1.98246	0.047427	0.080602	ZNF221	protein_cc
ENSG0000(	91.35793	0.23141	0.036066	6.416224	1.40E-10	1.04E-09	SWSAP1	protein_cc
ENSG0000(	91.30371	0.360304	0.05993	6.012032	1.83E-09	1.15E-08	LINC0032	lincRNA
ENSG0000(	91.28921	-0.48937	0.150591	-3.24964	0.001156	0.002781	MIR7-3H(	lincRNA
ENSG0000(	91.28611	0.008915	0.044386	0.200845	0.84082	0.884277	ZNF852	protein_cc
ENSG0000(	91.28594	0.511899	0.159013	3.219219	0.001285	0.003072	RASSF9	protein_cc
ENSG0000(	91.26651	0.178358	0.114386	1.55926	0.118935	0.179384	DCDC2	protein_cc
ENSG0000(	91.25744	0.124477	0.035933	3.46418	0.000532	0.001366	AC079807	antisense
ENSG0000(	91.21537	-0.58327	0.080538	-7.24211	4.42E-13	4.84E-12	TAS2R4	protein_cc
ENSG0000(	91.16342	-0.55386	0.084059	-6.58888	4.43E-11	3.57E-10	RP11-299	antisense
ENSG0000(	91.13072	0.942104	0.057189	16.47357	5.68E-61	5.84E-57	LINC0115	antisense
ENSG0000(	91.04573	0.032692	0.131343	0.248908	0.803432	0.854882	CTD-3064	antisense
ENSG0000(	90.92067	0.184087	0.060231	3.056329	0.002241	0.005122	CTD-3025	lincRNA
ENSG0000(	90.89307	0.252116	0.045984	5.48273	4.19E-08	2.11E-07	CTD-3131	lincRNA
ENSG0000(	90.8025	0.199834	0.056551	3.533695	0.00041	0.001076	CTC-479C	lincRNA
ENSG0000(	90.75144	-0.09589	0.174147	-0.55063	0.581884	0.668643	CRABP1	protein_cc
ENSG0000(	90.72381	0.120981	0.076234	1.586983	0.112516	0.17105	SH2D6	protein_cc
ENSG0000(	90.68815	0.153265	0.082393	1.860157	0.062863	0.103181	U73166.2	lincRNA
ENSG0000(	90.66768	-0.20991	0.055587	-3.77631	0.000159	0.000449	GLUD1P3	pseudoge
ENSG0000(	90.66527	0.563262	0.077458	7.27185	3.55E-13	3.95E-12	CCDC150	protein_cc
ENSG0000(	90.60354	0.019696	0.055718	0.353489	0.723722	0.789787	AC093495	antisense
ENSG0000(	90.55542	-0.10395	0.049591	-2.09609	0.036074	0.063331	RP11-15N	sense_ove
ENSG0000(	90.45169	-1.55361	0.157038	-9.89321	4.46E-23	2.66E-21	HIPK4	protein_cc
ENSG0000(	90.43113	-0.12031	0.052721	-2.28206	0.022486	0.041413	GVQW1	protein_cc
ENSG0000(	90.36301	-0.17627	0.083075	-2.12184	0.033851	0.059782	DRD4	protein_cc
ENSG0000(	90.35143	0.332353	0.080629	4.121996	3.76E-05	0.000118	AURKC	protein_cc
ENSG0000(	90.29176	0.626731	0.153067	4.094497	4.23E-05	0.000132	ABCA13	protein_cc
ENSG0000(	90.28729	0.512721	0.086162	5.950649	2.67E-09	1.63E-08	RP11-472	lincRNA
ENSG0000(	90.20648	1.417019	0.176975	8.006902	1.18E-15	2.01E-14	OTOS	protein_cc
ENSG0000(	90.16682	-0.17619	0.10033	-1.75613	0.079066	0.125979	CTSV	protein_cc
ENSG0000(	90.04658	0.035909	0.086781	0.413789	0.679029	0.752993	FMO3	protein_cc
ENSG0000(	89.98574	0.232609	0.10313	2.255493	0.024102	0.044068	RP11-195	protein_cc
ENSG0000(	89.97402	0.350337	0.085404	4.102109	4.09E-05	0.000128	HLA-K	pseudoge
ENSG0000(	89.97371	0.515411	0.065189	7.906435	2.65E-15	4.27E-14	SPCS2P4	pseudoge
ENSG0000(	89.93704	-0.27848	0.093936	-2.96456	0.003031	0.006747	RP11-182	pseudoge
ENSG0000(	89.86714	-0.18801	0.054821	-3.42964	0.000604	0.001535	HYAL1	protein_cc
ENSG0000(	89.85637	0.029521	0.083857	0.352046	0.724804	0.790674	DGKZP1	pseudoge
ENSG0000(	89.81626	0.166622	0.113415	1.469132	0.141797	0.208379	FOXD2-A	antisense
ENSG0000(	89.80442	-0.18941	0.060483	-3.13165	0.001738	0.004058	CTD-2132	sense_intr
ENSG0000(	89.76762	-0.40479	0.115322	-3.51007	0.000448	0.001168	CYP21A2	protein_cc
ENSG0000(	89.73636	-0.19848	0.077204	-2.57087	0.010144	0.020229	RP11-981	lincRNA
ENSG0000(	89.63428	0.117562	0.060296	1.949771	0.051203	0.086285	C9orf139	protein_cc
ENSG0000(	89.6313	0.176044	0.141327	1.245652	0.212892	0.294325	RP11-357	lincRNA
ENSG0000(	89.63034	0.422709	0.111821	3.780244	0.000157	0.000442	APOC2	protein_cc
ENSG0000(	89.62226	0.230272	0.07128	3.230533	0.001236	0.002962	HIST1H2B	protein_cc
ENSG0000(	89.5991	-0.12697	0.062137	-2.04335	0.041018	0.070956	MSS51	protein_cc
ENSG0000(	89.49734	-0.29358	0.116313	-2.52402	0.011602	0.022843	DNAH2	protein_cc
ENSG0000(	89.47051	-0.22049	0.057921	-3.80664	0.000141	0.000401	RP11-563	lincRNA
ENSG0000(	89.46476	-0.30301	0.086587	-3.49948	0.000466	0.00121	CNTF	protein_cc
ENSG0000(	89.25831	0.328915	0.054842	5.997547	2.00E-09	1.24E-08	RFESD	protein_cc
ENSG0000(	89.25199	-0.52041	0.118467	-4.39291	1.12E-05	3.83E-05	RP11-158	antisense

ENSG0000(	89.2367	0.262991	0.04505	5.837705	5.29E-09	3.06E-08	C16orf95	protein_cc
ENSG0000(	89.21398	0.044584	0.06934	0.642969	0.520244	0.612591	ZNF296	protein_cc
ENSG0000(	89.09564	0.216122	0.065887	3.280174	0.001037	0.00252	ATP2A1	protein_cc
ENSG0000(	89.03871	-0.15597	0.051154	-3.04896	0.002296	0.005239	RP11-552	sense_ove
ENSG0000(	88.90382	0.468639	0.05348	8.762935	1.90E-18	5.07E-17	RP11-162.	protein_cc
ENSG0000(	88.89444	0.848445	0.143782	5.90092	3.61E-09	2.16E-08	ACTBP2	pseudoge
ENSG0000(	88.87413	-0.28386	0.197319	-1.43857	0.150273	0.219151	RP1-35C2	lincRNA
ENSG0000(	88.86101	-1.07784	0.124969	-8.62486	6.42E-18	1.59E-16	GALNTL6	protein_cc
ENSG0000(	88.8509	-0.46744	0.081161	-5.75945	8.44E-09	4.73E-08	RP4-621B	lincRNA
ENSG0000(	88.84953	0.039913	0.140385	0.284308	0.776175	0.832926	CXCL1	protein_cc
ENSG0000(	88.84592	-0.20414	0.050527	-4.04026	5.34E-05	0.000163	USP46-AS	lincRNA
ENSG0000(	88.77837	0.718596	0.0959	7.493162	6.72E-14	8.45E-13	ANKRD53	protein_cc
ENSG0000(	88.75902	0.360279	0.068103	5.290185	1.22E-07	5.71E-07	RPL13AP2	pseudoge
ENSG0000(	88.7422	0.217112	0.127053	1.70884	0.087481	0.137584	LOXL4	protein_cc
ENSG0000(	88.69439	-0.47321	0.121937	-3.88073	0.000104	0.000303	RNF43	protein_cc
ENSG0000(	88.69428	-0.1531	0.119546	-1.28071	0.200295	0.279635	FZD10-AS	processed
ENSG0000(	88.66497	0.292855	0.084549	3.463733	0.000533	0.001368	FAM86B1	protein_cc
ENSG0000(	88.65499	-0.12363	0.047311	-2.61324	0.008969	0.018108	CNTD1	protein_cc
ENSG0000(	88.62659	0.140891	0.053995	2.609319	0.009072	0.018298	CTD-2017	lincRNA
ENSG0000(	88.58743	0.159875	0.085366	1.872816	0.061094	0.100622	C19orf35	protein_cc
ENSG0000(	88.5843	0.458533	0.067044	6.839304	7.96E-12	7.18E-11	HNRNPLP	pseudoge
ENSG0000(	88.55912	-0.10905	0.054587	-1.99772	0.045747	0.078094	CALML4	protein_cc
ENSG0000(	88.49115	0.224209	0.084465	2.654449	0.007944	0.016243	CTB-119C	antisense
ENSG0000(	88.40315	-0.6966	0.110236	-6.31913	2.63E-10	1.88E-09	RP11-710	antisense
ENSG0000(	88.32495	0.070929	0.068208	1.039888	0.298392	0.390674	CTB-31O2	processed
ENSG0000(	88.26373	-0.16082	0.104457	-1.53961	0.123656	0.185389	PNPLA3	protein_cc
ENSG0000(	88.25189	-0.48445	0.053725	-9.01719	1.93E-19	6.04E-18	RP11-517	lincRNA
ENSG0000(	88.17146	0.250554	0.044213	5.666977	1.45E-08	7.86E-08	WDR92	protein_cc
ENSG0000(	88.05762	0.380777	0.149627	2.544849	0.010932	0.021657	CSTA	protein_cc
ENSG0000(	88.03268	0.083705	0.072795	1.149874	0.250196	0.337247	RP5-966N	protein_cc
ENSG0000(	88.01726	0.211852	0.040506	5.230071	1.69E-07	7.74E-07	SEC24B-A	antisense
ENSG0000(	88.00932	-0.11464	0.096988	-1.18205	0.237188	0.322213	CD1D	protein_cc
ENSG0000(	87.97385	-0.01065	0.095653	-0.1113	0.911381	0.937402	HLA-G	protein_cc
ENSG0000(	87.92921	0.080541	0.043621	1.846372	0.064838	0.105961	RP11-10K	antisense
ENSG0000(	87.89866	-0.05553	0.133291	-0.41657	0.67699	0.75124	MMEL1	protein_cc
ENSG0000(	87.87331	-0.08823	0.041534	-2.12416	0.033656	0.059467	RP11-667	lincRNA
ENSG0000(	87.83226	0.609851	0.062978	9.683513	3.54E-22	1.77E-20	CTA-293F	antisense
ENSG0000(	87.72792	-0.18521	0.082145	-2.25465	0.024155	0.04416	SULT1B1	protein_cc
ENSG0000(	87.66677	0.492479	0.115687	4.256978	2.07E-05	6.80E-05	RP11-726	pseudoge
ENSG0000(	87.57955	-1.42434	0.141397	-10.0733	7.25E-24	5.13E-22	RSPO3	protein_cc
ENSG0000(	87.55049	0.048046	0.051072	0.940765	0.346825	0.442293	RP11-320	lincRNA
ENSG0000(	87.50582	-0.42326	0.076609	-5.52488	3.30E-08	1.69E-07	AC144831	lincRNA
ENSG0000(	87.50498	0.512331	0.089957	5.695283	1.23E-08	6.73E-08	AC025165	antisense
ENSG0000(	87.48274	-0.00131	0.041482	-0.03162	0.974772	0.982296	RP11-571	lincRNA
ENSG0000(	87.47656	0.979967	0.147834	6.628812	3.38E-11	2.77E-10	HPD	protein_cc
ENSG0000(	87.46918	-0.24454	0.097129	-2.51767	0.011813	0.023217	RP11-262	pseudoge
ENSG0000(	87.46404	-0.09792	0.060991	-1.60542	0.108402	0.165617	ANKRD36I	pseudoge
ENSG0000(	87.4584	0.368693	0.102949	3.581322	0.000342	0.000911	FXYD3	protein_cc
ENSG0000(	87.41555	0.107369	0.126925	0.845927	0.397594	0.494156	ITGB3	protein_cc
ENSG0000(	87.28848	0.178964	0.059666	2.99945	0.002705	0.006082	RP11-493	antisense
ENSG0000(	87.27835	0.052754	0.065328	0.807513	0.419371	0.516311	ATP8B3	protein_cc
ENSG0000(	87.26067	-0.16515	0.055047	-3.00009	0.002699	0.006071	AC241585	protein_cc
ENSG0000(	87.25967	0.012067	0.05345	0.225771	0.821379	0.869121	KIAA0825	protein_cc
ENSG0000(	87.22057	-0.24709	0.09468	-2.60976	0.00906	0.018277	PIWIL2	protein_cc
ENSG0000(	87.21175	0.005237	0.097748	0.053574	0.957275	0.970706	RIIAD1	protein_cc
ENSG0000(	87.19712	0.250818	0.046071	5.444112	5.21E-08	2.58E-07	RP11-299.	antisense
ENSG0000(	87.18533	-0.37929	0.093142	-4.07217	4.66E-05	0.000144	RP1-167F.	antisense
ENSG0000(	87.16166	-0.35817	0.084801	-4.22368	2.40E-05	7.79E-05	GPAT2	protein_cc
ENSG0000(	87.14417	-0.07032	0.124493	-0.56483	0.572191	0.659617	PTCHD4	protein_cc

ENSG0000(	87.13432	-0.18242	0.077524	-2.35313	0.018616	0.034914	CCNB3	protein_cc
ENSG0000(	87.12483	0.074553	0.055864	1.334548	0.182024	0.257652	ALG1L6P	pseudoge
ENSG0000(	87.11041	-0.9203	0.101978	-9.02449	1.81E-19	5.68E-18	TRABD2A	protein_cc
ENSG0000(	87.05293	0.520106	0.075684	6.87206	6.33E-12	5.80E-11	TECRP1	pseudoge
ENSG0000(	87.00292	-0.22161	0.051214	-4.32705	1.51E-05	5.07E-05	CTC-308K	antisense
ENSG0000(	86.98573	-0.17276	0.061264	-2.81998	0.004803	0.010264	RP11-337(	antisense
ENSG0000(	86.98563	-0.47185	0.09043	-5.2179	1.81E-07	8.22E-07	ZNF702P	pseudoge
ENSG0000(	86.92628	-0.12096	0.07138	-1.69461	0.090149	0.14123	RP11-15A	lincRNA
ENSG0000(	86.91247	0.474335	0.100729	4.709009	2.49E-06	9.45E-06	LINC0034(	protein_cc
ENSG0000(	86.90661	-0.22235	0.058278	-3.81544	0.000136	0.000388	GLTPD2	protein_cc
ENSG0000(	86.87564	1.586556	0.1858	8.539048	1.35E-17	3.18E-16	GSX2	protein_cc
ENSG0000(	86.84096	-0.09116	0.099761	-0.91382	0.360812	0.456681	CBLN3	protein_cc
ENSG0000(	86.82597	-0.67968	0.104368	-6.51237	7.40E-11	5.76E-10	SFTPD	protein_cc
ENSG0000(	86.71168	0.011312	0.056191	0.201318	0.84045	0.883994	RP11-324	antisense
ENSG0000(	86.65445	-0.10595	0.071166	-1.48876	0.136551	0.201714	RP11-71E	lincRNA
ENSG0000(	86.61843	-0.19359	0.073567	-2.63144	0.008502	0.017267	RP11-598	pseudoge
ENSG0000(	86.60051	0.285707	0.06848	4.172142	3.02E-05	9.63E-05	CFP	protein_cc
ENSG0000(	86.59006	0.661029	0.114599	5.76818	8.01E-09	4.51E-08	GSC	protein_cc
ENSG0000(	86.53972	4.594848	0.357622	12.84834	8.79E-38	9.03E-35	C6orf15	protein_cc
ENSG0000(	86.52481	-0.33062	0.055715	-5.93416	2.95E-09	1.79E-08	RP11-31F:	lincRNA
ENSG0000(	86.50416	-0.40014	0.101408	-3.94582	7.95E-05	0.000237	NOTUM	protein_cc
ENSG0000(	86.50128	0.318638	0.092751	3.435418	0.000592	0.001506	C16orf74	protein_cc
ENSG0000(	86.48203	0.324564	0.103608	3.132616	0.001733	0.004046	HCG4P7	pseudoge
ENSG0000(	86.44934	-0.11042	0.098241	-1.12395	0.261034	0.349393	RP11-8P1	lincRNA
ENSG0000(	86.42104	0.568579	0.081729	6.956902	3.48E-12	3.33E-11	RSPH9	protein_cc
ENSG0000(	86.35874	-0.00773	0.065161	-0.11866	0.905542	0.933157	AP003068	protein_cc
ENSG0000(	86.32621	0.346611	0.08713	3.978086	6.95E-05	0.000209	LBX2-AS1	antisense
ENSG0000(	86.31502	0.618075	0.091252	6.773301	1.26E-11	1.10E-10	CABP4	protein_cc
ENSG0000(	86.29849	0.089435	0.134365	0.665615	0.505657	0.59939	RP11-438	lincRNA
ENSG0000(	86.29432	0.181481	0.063728	2.847725	0.004403	0.009491	LINC0096:	lincRNA
ENSG0000(	86.27366	-0.2546	0.045995	-5.53533	3.11E-08	1.60E-07	RP11-582.	antisense
ENSG0000(	86.26969	0.079792	0.157967	0.505118	0.613476	0.696884	INSM2	protein_cc
ENSG0000(	86.25924	0.268221	0.114706	2.338328	0.01937	0.036174	NR5A2	protein_cc
ENSG0000(	86.25863	0.683955	0.23048	2.967527	0.003002	0.00669	HAND2	protein_cc
ENSG0000(	86.15216	0.172969	0.160682	1.076466	0.281719	0.37233	MARCO	protein_cc
ENSG0000(	86.07276	0.026085	0.125525	0.207808	0.835379	0.880153	DOK2	protein_cc
ENSG0000(	85.99484	-0.00231	0.060359	-0.03823	0.969503	0.978881	TUBA3FP	pseudoge
ENSG0000(	85.97446	-0.51443	0.146129	-3.52037	0.000431	0.001127	RP11-298	antisense
ENSG0000(	85.97054	-0.14131	0.047929	-2.94828	0.003195	0.007081	RP5-894A	sense_intr(
ENSG0000(	85.95477	0.030117	0.127701	0.235837	0.813559	0.863039	IL2RB	protein_cc
ENSG0000(	85.94178	-0.50584	0.110066	-4.59573	4.31E-06	1.58E-05	WNT4	protein_cc
ENSG0000(	85.85288	-0.15618	0.074909	-2.08495	0.037074	0.064874	PRSS51	antisense
ENSG0000(	85.83022	1.010577	0.116183	8.698147	3.37E-18	8.69E-17	PXDNL	protein_cc
ENSG0000(	85.73445	0.20785	0.058418	3.557983	0.000374	0.000988	RP11-284	lincRNA
ENSG0000(	85.73414	0.786871	0.096401	8.162482	3.28E-16	6.13E-15	TUBBP1	pseudoge
ENSG0000(	85.62385	0.309021	0.047305	6.532488	6.47E-11	5.08E-10	CTD-2331	pseudoge
ENSG0000(	85.56205	0.217625	0.05304	4.103001	4.08E-05	0.000127	FAM86FP	pseudoge
ENSG0000(	85.51212	0.689778	0.104179	6.62111	3.57E-11	2.91E-10	MYCBPAP	protein_cc
ENSG0000(	85.49444	-0.08957	0.058281	-1.53683	0.124335	0.186292	SERHL	processed
ENSG0000(	85.46187	0.208697	0.056577	3.688714	0.000225	0.000619	ANKRD23	protein_cc
ENSG0000(	85.43285	0.260968	0.128264	2.034611	0.04189	0.072275	ANKRD55	protein_cc
ENSG0000(	85.40592	-1.48307	0.146189	-10.1449	3.49E-24	2.61E-22	C4orf50	protein_cc
ENSG0000(	85.39784	0.177875	0.045385	3.91924	8.88E-05	0.000262	RP11-329	lincRNA
ENSG0000(	85.32998	1.12057	0.123216	9.094351	9.52E-20	3.12E-18	CHCHD2P	pseudoge
ENSG0000(	85.27433	0.076736	0.093326	0.822235	0.410943	0.507877	HCCAT3	antisense
ENSG0000(	85.22598	-0.05344	0.065949	-0.81027	0.417783	0.514737	PIP5KL1	protein_cc
ENSG0000(	85.21529	0.431924	0.055573	7.772203	7.71E-15	1.15E-13	RP11-848	antisense
ENSG0000(	85.20905	0.910429	0.105784	8.606472	7.53E-18	1.84E-16	RP11-12M	pseudoge
ENSG0000(	85.20224	-0.63853	0.142774	-4.47231	7.74E-06	2.72E-05	PTGFR	protein_cc

ENSG0000(	85.1014	-0.21459	0.067385	-3.18449	0.00145	0.003433	FOXO3B	pseudoge
ENSG0000(	85.09427	-0.15496	0.116487	-1.33025	0.183436	0.259356	VENTX	protein_cc
ENSG0000(	85.03762	-1.2945	0.149316	-8.66956	4.34E-18	1.10E-16	HTR2C	protein_cc
ENSG0000(	84.99864	0.575453	0.094296	6.102619	1.04E-09	6.79E-09	C8orf88	protein_cc
ENSG0000(	84.98834	-0.08383	0.046662	-1.79653	0.07241	0.116737	RP11-440	antisense
ENSG0000(	84.98283	-1.31979	0.125796	-10.4915	9.45E-26	9.74E-24	SHISA2	protein_cc
ENSG0000(	84.97505	0.217896	0.057414	3.795178	0.000148	0.000418	RP11-79P	lincRNA
ENSG0000(	84.81872	-0.48165	0.156983	-3.06819	0.002154	0.004938	CTB-36O1	pseudoge
ENSG0000(	84.81345	-0.28181	0.091498	-3.08001	0.00207	0.004764	FOXQ1	protein_cc
ENSG0000(	84.77074	0.476007	0.052651	9.040769	1.56E-19	4.94E-18	CTD-2256	antisense
ENSG0000(	84.74162	-0.29943	0.065903	-4.54354	5.53E-06	1.99E-05	RP11-96H	lincRNA
ENSG0000(	84.64325	0.277219	0.065133	4.256209	2.08E-05	6.82E-05	PRSS36	protein_cc
ENSG0000(	84.52718	0.603295	0.129161	4.670863	3.00E-06	1.12E-05	FTH1P11	pseudoge
ENSG0000(	84.48568	1.698344	0.284649	5.966445	2.42E-09	1.49E-08	HOXA2	protein_cc
ENSG0000(	84.40484	-0.28245	0.122533	-2.30511	0.02116	0.039182	COL13A1	protein_cc
ENSG0000(	84.39741	0.461121	0.075611	6.098631	1.07E-09	6.94E-09	ZNF724P	protein_cc
ENSG0000(	84.34897	-0.1854	0.065656	-2.82374	0.004747	0.010153	RANP4	pseudoge
ENSG0000(	84.33567	0.106432	0.045027	2.36371	0.018093	0.034026	RP11-77H	antisense
ENSG0000(	84.33385	0.105528	0.077427	1.362938	0.172902	0.24683	AC093627	lincRNA
ENSG0000(	84.32961	-0.12997	0.044435	-2.92486	0.003446	0.00759	RP11-428.	lincRNA
ENSG0000(	84.32766	-1.4833	0.154668	-9.59025	8.79E-22	4.07E-20	TCERG1L	protein_cc
ENSG0000(	84.29635	-0.18197	0.085392	-2.13098	0.033091	0.058583	AP001626	antisense
ENSG0000(	84.21813	-0.19981	0.10121	-1.9742	0.04836	0.081996	ATE1-AS1	antisense
ENSG0000(	84.18609	0.249313	0.07681	3.245831	0.001171	0.002816	RP11-552	antisense
ENSG0000(	84.17682	0.103882	0.062087	1.673173	0.094293	0.146816	AC116366	antisense
ENSG0000(	84.00378	-0.08057	0.094025	-0.85693	0.391481	0.488034	DCDC1	protein_cc
ENSG0000(	83.93434	0.299778	0.053959	5.555636	2.77E-08	1.43E-07	RP11-792.	antisense
ENSG0000(	83.89665	0.349654	0.107607	3.24937	0.001157	0.002784	RP11-307	lincRNA
ENSG0000(	83.8791	0.042658	0.067679	0.630295	0.528502	0.620195	INHA	protein_cc
ENSG0000(	83.83152	-0.61521	0.129412	-4.75389	2.00E-06	7.69E-06	ADAMTS1	protein_cc
ENSG0000(	83.83079	0.303662	0.075804	4.005869	6.18E-05	0.000187	FAM47E-S	protein_cc
ENSG0000(	83.79571	1.248101	0.117808	10.59437	3.16E-26	3.53E-24	SPINK8	protein_cc
ENSG0000(	83.69323	0.340551	0.119625	2.846815	0.004416	0.009514	SCAND3	protein_cc
ENSG0000(	83.65396	-1.15696	0.136482	-8.477	2.31E-17	5.24E-16	KCNA4	protein_cc
ENSG0000(	83.60015	0.214182	0.236413	0.905966	0.364954	0.461002	RP11-82C	antisense
ENSG0000(	83.56357	0.423928	0.100988	4.197819	2.69E-05	8.67E-05	HSD17B3	protein_cc
ENSG0000(	83.5443	1.776103	0.237437	7.480303	7.42E-14	9.24E-13	HOXA-AS	antisense
ENSG0000(	83.5346	0.039204	0.134301	0.291912	0.770354	0.827933	CD3E	protein_cc
ENSG0000(	83.53098	0.229279	0.040595	5.647971	1.62E-08	8.72E-08	RP11-10L	antisense
ENSG0000(	83.52939	0.184728	0.080647	2.290579	0.021988	0.040566	ADAT3	protein_cc
ENSG0000(	83.52253	-0.82756	0.099861	-8.28709	1.16E-16	2.33E-15	RP11-106	lincRNA
ENSG0000(	83.5202	-0.5345	0.075449	-7.08431	1.40E-12	1.42E-11	NHSL2	protein_cc
ENSG0000(	83.4282	0.499978	0.058342	8.56979	1.04E-17	2.48E-16	UFSP1	protein_cc
ENSG0000(	83.32707	2.252017	0.197529	11.40094	4.14E-30	9.50E-28	LINC0047	processed
ENSG0000(	83.30263	-0.14939	0.062217	-2.4011	0.016346	0.031105	RP11-677	pseudoge
ENSG0000(	83.26032	1.159329	0.146295	7.924575	2.29E-15	3.73E-14	LECT1	protein_cc
ENSG0000(	83.20675	-0.0108	0.074746	-0.14446	0.885141	0.918116	RP11-164.	processed
ENSG0000(	83.2039	0.019717	0.120997	0.162953	0.870556	0.907014	TTLL6	protein_cc
ENSG0000(	83.1994	0.810421	0.11093	7.305717	2.76E-13	3.12E-12	MIR4426	pseudoge
ENSG0000(	83.18589	0.529597	0.101469	5.219297	1.80E-07	8.17E-07	LINC0105	lincRNA
ENSG0000(	83.17879	-0.08828	0.083791	-1.05356	0.292085	0.383589	SMTNL1	protein_cc
ENSG0000(	83.09684	-0.97773	0.129852	-7.52953	5.09E-14	6.52E-13	NLRP2	protein_cc
ENSG0000(	83.07904	-0.65742	0.098949	-6.644	3.05E-11	2.52E-10	MYLK3	protein_cc
ENSG0000(	83.05563	0.376436	0.101356	3.714005	0.000204	0.000564	CCDC89	protein_cc
ENSG0000(	83.04952	0.440044	0.08439	5.214413	1.84E-07	8.37E-07	CCDC81	protein_cc
ENSG0000(	82.95552	-0.57961	0.18492	-3.1344	0.001722	0.004023	GLP1R	protein_cc
ENSG0000(	82.94122	-0.58595	0.132441	-4.4242	9.68E-06	3.35E-05	RP4-610C	lincRNA
ENSG0000(	82.92505	0.351521	0.056616	6.208902	5.34E-10	3.62E-09	C1orf229	protein_cc
ENSG0000(	82.90686	-0.16786	0.08848	-1.89714	0.05781	0.095935	AC073283	lincRNA

ENSG0000	82.84714	0.52146	0.076897	6.781309	1.19E-11	1.04E-10	MLLT4-AS	processed
ENSG0000	82.83155	0.466333	0.067465	6.912239	4.77E-12	4.45E-11	RP11-723	lincRNA
ENSG0000	82.83032	0.306752	0.085057	3.606421	0.00031	0.000833	CYP39A1	protein_cc
ENSG0000	82.81668	1.793983	0.268937	6.67065	2.55E-11	2.13E-10	HOXA4	protein_cc
ENSG0000	82.73766	-0.43422	0.094964	-4.57248	4.82E-06	1.75E-05	KCNH4	protein_cc
ENSG0000	82.5971	0.114496	0.109163	1.048851	0.294246	0.386053	RP1-79C4	lincRNA
ENSG0000	82.58869	-1.83665	0.186604	-9.84249	7.39E-23	4.20E-21	LL0XNC01	pseudoge
ENSG0000	82.5122	-0.03893	0.07722	-0.5042	0.614123	0.697387	RBM20	protein_cc
ENSG0000	82.45396	0.11317	0.052191	2.168375	0.03013	0.053852	CTD-2349	lincRNA
ENSG0000	82.41585	0.046568	0.072939	0.638457	0.523177	0.615174	TGM1	protein_cc
ENSG0000	82.33506	-0.2963	0.067651	-4.37989	1.19E-05	4.05E-05	GUCA1B	protein_cc
ENSG0000	82.25675	-0.74696	0.113409	-6.58647	4.50E-11	3.62E-10	RP11-364	pseudoge
ENSG0000	82.20724	0.358781	0.150731	2.380283	0.017299	0.032679	RHOD	protein_cc
ENSG0000	82.18275	-0.16063	0.111456	-1.44118	0.149533	0.218188	TEX15	protein_cc
ENSG0000	82.09512	0.077654	0.117469	0.661055	0.508577	0.602035	RP11-262	lincRNA
ENSG0000	82.07498	0.587839	0.074487	7.891871	2.98E-15	4.76E-14	RP1-1J6.2	antisense
ENSG0000	82.05126	0.272544	0.128934	2.113827	0.03453	0.060885	FER1L4	pseudoge
ENSG0000	81.99985	0.364488	0.049163	7.41384	1.23E-13	1.47E-12	ZNF559-Z	protein_cc
ENSG0000	81.91208	0.93652	0.118769	7.885209	3.14E-15	5.00E-14	ETV7	protein_cc
ENSG0000	81.85649	-0.83123	0.116986	-7.10532	1.20E-12	1.23E-11	PALM2	protein_cc
ENSG0000	81.85515	-0.1584	0.124087	-1.27654	0.201765	0.281401	ADAD2	protein_cc
ENSG0000	81.80982	-0.13505	0.103746	-1.30174	0.193004	0.270928	GIPR	protein_cc
ENSG0000	81.79762	0.557557	0.173958	3.205131	0.00135	0.003214	IGJ	protein_cc
ENSG0000	81.77422	-0.03726	0.086213	-0.43221	0.665591	0.741906	EFCC1	protein_cc
ENSG0000	81.75611	-0.1167	0.154035	-0.7576	0.44869	0.545495	FNDC1	protein_cc
ENSG0000	81.685	-0.33126	0.135728	-2.44064	0.014661	0.028221	DMRT2	protein_cc
ENSG0000	81.67623	0.021017	0.127217	0.165206	0.868782	0.905768	RP11-159.	pseudoge
ENSG0000	81.64843	0.201996	0.076198	2.650941	0.008027	0.016391	GALNT12	protein_cc
ENSG0000	81.62138	-0.33425	0.073932	-4.52109	6.15E-06	2.20E-05	TRAPPC12	antisense
ENSG0000	81.53698	-0.82373	0.150877	-5.45964	4.77E-08	2.38E-07	TRHDE-AS	processed
ENSG0000	81.50587	-0.18661	0.090744	-2.05643	0.039741	0.068987	PON3	protein_cc
ENSG0000	81.47454	0.596316	0.090307	6.603225	4.02E-11	3.26E-10	CHCHD2P	pseudoge
ENSG0000	81.45167	-0.45723	0.119302	-3.83253	0.000127	0.000364	RP1-63G5	sense_ove
ENSG0000	81.43928	0.149029	0.074071	2.011978	0.044222	0.075778	GOLGA8N	protein_cc
ENSG0000	81.38655	0.449917	0.168349	2.672518	0.007528	0.015468	SLC34A2	protein_cc
ENSG0000	81.34308	-0.45888	0.193254	-2.37449	0.017573	0.033129	MEPE	protein_cc
ENSG0000	81.32737	-0.41679	0.078116	-5.3355	9.53E-08	4.53E-07	RP11-451	protein_cc
ENSG0000	81.23034	0.222189	0.055967	3.970007	7.19E-05	0.000215	DYX1C1	protein_cc
ENSG0000	81.17979	-0.66329	0.148368	-4.47054	7.80E-06	2.74E-05	OR2L13	protein_cc
ENSG0000	81.17088	0.667879	0.140927	4.739197	2.15E-06	8.23E-06	NMU	protein_cc
ENSG0000	81.16123	-0.01821	0.137869	-0.13209	0.89491	0.925332	TRIM71	protein_cc
ENSG0000	81.16084	0.169869	0.049184	3.45372	0.000553	0.001414	RP11-793	antisense
ENSG0000	81.09182	-1.25925	0.156732	-8.03444	9.40E-16	1.62E-14	OPRK1	protein_cc
ENSG0000	81.01008	0.009264	0.050579	0.183153	0.854678	0.894983	CTC-260F	protein_cc
ENSG0000	80.99822	-0.2835	0.105033	-2.69915	0.006952	0.014373	RP11-285	lincRNA
ENSG0000	80.9704	0.014526	0.093048	0.156115	0.875942	0.911056	CD68	protein_cc
ENSG0000	80.96452	-1.73403	0.179854	-9.64131	5.35E-22	2.57E-20	RP11-286	lincRNA
ENSG0000	80.9488	1.050572	0.142654	7.364459	1.78E-13	2.07E-12	TPT1P9	pseudoge
ENSG0000	80.94548	-0.25407	0.04527	-5.61245	1.99E-08	1.06E-07	RP5-994D	antisense
ENSG0000	80.8192	1.017331	0.165776	6.136772	8.42E-10	5.55E-09	ADAMTS1	protein_cc
ENSG0000	80.80012	-1.24461	0.13525	-9.20229	3.50E-20	1.23E-18	GLP2R	protein_cc
ENSG0000	80.78025	-0.34343	0.088919	-3.8623	0.000112	0.000325	RHCG	protein_cc
ENSG0000	80.76933	0.115432	0.056207	2.053694	0.040005	0.069399	RP11-326	antisense
ENSG0000	80.7366	-0.09272	0.081788	-1.13363	0.256951	0.344826	CYP2D7P	polymorpl
ENSG0000	80.67808	0.084791	0.07802	1.086791	0.277129	0.36735	DPH6-AS1	lincRNA
ENSG0000	80.65998	-0.59245	0.062745	-9.4421	3.65E-21	1.52E-19	RP4-561L	lincRNA
ENSG0000	80.639	-1.89589	0.194703	-9.73736	2.09E-22	1.09E-20	KLK7	protein_cc
ENSG0000	80.63353	-1.51518	0.168348	-9.00027	2.25E-19	6.94E-18	LY86-AS1	antisense
ENSG0000	80.59628	-1.33178	0.129675	-10.2701	9.61E-25	8.03E-23	SLC7A4	protein_cc

ENSG0000(	80.58671	-0.09872	0.093227	-1.05888	0.289655	0.381052	ITGB2-AS:	antisense
ENSG0000(	80.57305	-0.53986	0.080114	-6.73862	1.60E-11	1.38E-10	FRK	protein_cc
ENSG0000(	80.54286	-0.41936	0.097499	-4.30119	1.70E-05	5.65E-05	GPR143	protein_cc
ENSG0000(	80.53245	-0.05193	0.077295	-0.67184	0.501687	0.595646	AC005519	sense_ove
ENSG0000(	80.5308	1.115749	0.118712	9.398815	5.52E-21	2.22E-19	MAB21L1	protein_cc
ENSG0000(	80.4607	-0.10652	0.076833	-1.38642	0.165617	0.238066	RP11-139	antisense
ENSG0000(	80.45209	-0.14048	0.105291	-1.33424	0.182127	0.257776	PRR16	protein_cc
ENSG0000(	80.44915	-0.1013	0.03932	-2.57624	0.009988	0.019953	RPL23AP5	pseudoge
ENSG0000(	80.36235	0.094541	0.133433	0.708528	0.478618	0.574056	RPSAP53	pseudoge
ENSG0000(	80.27254	-1.82419	0.19251	-9.47585	2.65E-21	1.13E-19	RP11-416	lincRNA
ENSG0000(	80.2388	-0.23133	0.056636	-4.08448	4.42E-05	0.000137	RP11-10N	antisense
ENSG0000(	80.21194	-0.25641	0.075815	-3.38212	0.000719	0.0018	RP11-274	pseudoge
ENSG0000(	80.14984	0.154645	0.100681	1.535993	0.12454	0.186524	RP11-597	antisense
ENSG0000(	80.14517	0.501829	0.08261	6.074693	1.24E-09	7.98E-09	GGN	protein_cc
ENSG0000(	80.13141	0.046875	0.074804	0.626638	0.530896	0.62256	ANO7	protein_cc
ENSG0000(	80.07083	0.317734	0.069551	4.56836	4.92E-06	1.78E-05	RP4-756H	sense_intr
ENSG0000(	80.04505	-1.21432	0.134786	-9.00925	2.07E-19	6.43E-18	RNF144A-	processed
ENSG0000(	79.96362	0.17556	0.050226	3.495398	0.000473	0.001227	TMEM262	protein_cc
ENSG0000(	79.95775	0.048045	0.070895	0.677692	0.497967	0.591947	LINC0024(	lincRNA
ENSG0000(	79.92922	0.972238	0.124937	7.781798	7.15E-15	1.07E-13	EIF4A1P1(	pseudoge
ENSG0000(	79.92114	0.09765	0.054072	1.805938	0.070928	0.114602	TMC4	protein_cc
ENSG0000(	79.89868	0.440635	0.063107	6.982357	2.90E-12	2.81E-11	YTHDF3-A	lincRNA
ENSG0000(	79.88073	0.37996	0.049755	7.636543	2.23E-14	3.03E-13	AC108488	lincRNA
ENSG0000(	79.88068	-0.13613	0.13916	-0.9782	0.327977	0.42235	KLRC4-KL	protein_cc
ENSG0000(	79.84951	-0.64	0.089083	-7.18431	6.75E-13	7.21E-12	ABHD12B	protein_cc
ENSG0000(	79.78998	0.993318	0.085479	11.62061	3.24E-31	9.38E-29	AC002456	antisense
ENSG0000(	79.76668	-0.06166	0.065034	-0.94804	0.343109	0.438321	GRID2IP	protein_cc
ENSG0000(	79.74899	-0.08289	0.057	-1.45419	0.145895	0.213583	SRXN1	protein_cc
ENSG0000(	79.71083	0.422156	0.079748	5.293594	1.20E-07	5.62E-07	RP11-436	lincRNA
ENSG0000(	79.67121	-0.4602	0.101649	-4.52738	5.97E-06	2.14E-05	C20orf203	lincRNA
ENSG0000(	79.62209	0.173456	0.080286	2.160462	0.030737	0.054819	RP4-794H	lincRNA
ENSG0000(	79.62	-0.25587	0.077885	-3.28524	0.001019	0.002479	SLC25A41	protein_cc
ENSG0000(	79.5957	0.034514	0.056693	0.608786	0.542666	0.633437	RP11-127	lincRNA
ENSG0000(	79.58718	-0.45979	0.072135	-6.37408	1.84E-10	1.34E-09	CARD14	protein_cc
ENSG0000(	79.57656	0.437561	0.111842	3.91231	9.14E-05	0.000269	COCH	protein_cc
ENSG0000(	79.5511	0.901002	0.204287	4.41048	1.03E-05	3.55E-05	C1orf158	protein_cc
ENSG0000(	79.54927	-0.06867	0.131109	-0.52376	0.600444	0.685241	CTB-43E1	lincRNA
ENSG0000(	79.49921	0.34023	0.084456	4.028496	5.61E-05	0.000171	C19orf18	protein_cc
ENSG0000(	79.49072	-0.21077	0.075002	-2.81014	0.004952	0.010562	RP11-875	antisense
ENSG0000(	79.42148	-0.03597	0.060763	-0.59199	0.553857	0.643468	LINC0027:	lincRNA
ENSG0000(	79.40706	0.890717	0.122616	7.264258	3.75E-13	4.15E-12	GLI1	protein_cc
ENSG0000(	79.30304	-0.12321	0.064557	-1.90851	0.056325	0.093771	RP11-509	lincRNA
ENSG0000(	79.23278	-0.80445	0.089874	-8.95083	3.53E-19	1.05E-17	DUSP27	protein_cc
ENSG0000(	79.20018	0.226277	0.057324	3.947307	7.90E-05	0.000235	RP11-98D	antisense
ENSG0000(	79.17315	-0.55739	0.07037	-7.92084	2.36E-15	3.83E-14	TNK1	protein_cc
ENSG0000(	79.14559	-0.05781	0.069487	-0.8319	0.405464	0.502353	RP11-77P	antisense
ENSG0000(	79.11332	-1.04341	0.134104	-7.78061	7.22E-15	1.08E-13	LINC0101	lincRNA
ENSG0000(	79.01626	0.168551	0.122856	1.371936	0.170083	0.243473	ACADL	protein_cc
ENSG0000(	78.93733	0.039072	0.055581	0.702976	0.482071	0.577205	RP11-783	lincRNA
ENSG0000(	78.89658	0.491627	0.079865	6.155747	7.47E-10	4.97E-09	AUNIP	protein_cc
ENSG0000(	78.81129	0.334955	0.098594	3.397311	0.000681	0.001711	FTH1P23	pseudoge
ENSG0000(	78.75095	0.470349	0.074337	6.327239	2.50E-10	1.79E-09	FSIP1	protein_cc
ENSG0000(	78.70351	0.182344	0.032397	5.6285	1.82E-08	9.70E-08	RP11-849	protein_cc
ENSG0000(	78.67038	-0.4672	0.085798	-5.44537	5.17E-08	2.56E-07	MST4	protein_cc
ENSG0000(	78.6098	-1.34957	0.144999	-9.30743	1.31E-20	4.94E-19	RP1-269M	antisense
ENSG0000(	78.59449	-0.5186	0.117562	-4.41127	1.03E-05	3.54E-05	CTD-2380	antisense
ENSG0000(	78.56035	-0.39397	0.094857	-4.15336	3.28E-05	0.000104	TCTE1	protein_cc
ENSG0000(	78.54614	-0.57398	0.061013	-9.40763	5.07E-21	2.05E-19	GIMAP5	protein_cc
ENSG0000(	78.51406	0.364571	0.085154	4.281315	1.86E-05	6.15E-05	RP11-317	antisense

ENSG0000(	78.49713	-0.06979	0.074731	-0.93391	0.350349	0.445784	LHX4	protein_cc
ENSG0000(	78.45618	0.442541	0.051862	8.533086	1.42E-17	3.33E-16	CTD-2270	lincRNA
ENSG0000(	78.45221	-0.36063	0.079768	-4.52098	6.16E-06	2.20E-05	RP11-517	antisense
ENSG0000(	78.44258	-0.36617	0.067233	-5.44623	5.14E-08	2.55E-07	DUSP8P5	pseudoge
ENSG0000(	78.37162	0.811077	0.109267	7.422899	1.15E-13	1.38E-12	EEF1A1P4	pseudoge
ENSG0000(	78.36694	-0.39534	0.101825	-3.88254	0.000103	0.000301	RP3-406A	lincRNA
ENSG0000(	78.23869	-0.18999	0.069479	-2.73458	0.006246	0.013041	TNFRSF10	protein_cc
ENSG0000(	78.207	-0.45259	0.05855	-7.72992	1.08E-14	1.56E-13	CTD-2292	lincRNA
ENSG0000(	78.16073	-0.97548	0.14303	-6.82005	9.10E-12	8.12E-11	FLG	protein_cc
ENSG0000(	78.10148	-0.37547	0.052981	-7.0869	1.37E-12	1.40E-11	AL022476	antisense
ENSG0000(	78.03751	0.044183	0.054736	0.807196	0.419554	0.516501	CMTM1	protein_cc
ENSG0000(	78.02772	0.622448	0.118373	5.25835	1.45E-07	6.70E-07	TMPRSS9	protein_cc
ENSG0000(	78.02441	0.712465	0.165118	4.314877	1.60E-05	5.33E-05	IRX5	protein_cc
ENSG0000(	78.02429	-0.03961	0.054079	-0.73248	0.463879	0.559988	RP5-864K	antisense
ENSG0000(	77.95263	0.025043	0.174896	0.143187	0.886142	0.918876	POU4F1	protein_cc
ENSG0000(	77.94983	-0.13479	0.14119	-0.9547	0.339728	0.434773	RBP3	protein_cc
ENSG0000(	77.94665	0.141038	0.048327	2.918391	0.003518	0.007736	ZNF137P	pseudoge
ENSG0000(	77.92184	0.616691	0.139811	4.410898	1.03E-05	3.55E-05	RP11-400	lincRNA
ENSG0000(	77.91099	0.35764	0.069752	5.127278	2.94E-07	1.29E-06	RP5-1159	lincRNA
ENSG0000(	77.87272	0.218	0.044758	4.87065	1.11E-06	4.46E-06	RP11-296	antisense
ENSG0000(	77.83819	-0.22165	0.219118	-1.01157	0.311741	0.405006	OR4N2	protein_cc
ENSG0000(	77.71358	-0.20724	0.053315	-3.88714	0.000101	0.000296	RPS11P6	pseudoge
ENSG0000(	77.67671	0.26082	0.09197	2.835941	0.004569	0.009808	PPP1R42	protein_cc
ENSG0000(	77.47511	-0.03119	0.087585	-0.35612	0.721752	0.788391	LAG3	protein_cc
ENSG0000(	77.44848	-0.74446	0.091929	-8.09822	5.58E-16	1.00E-14	SRL	protein_cc
ENSG0000(	77.44654	0.107883	0.041738	2.584782	0.009744	0.019515	RP11-267	antisense
ENSG0000(	77.44359	-0.15026	0.053004	-2.83487	0.004584	0.009837	CTD-2516	antisense
ENSG0000(	77.39304	-0.23132	0.146108	-1.5832	0.113377	0.172195	KLRC4	protein_cc
ENSG0000(	77.33155	0.867866	0.121847	7.122611	1.06E-12	1.10E-11	MT2P1	pseudoge
ENSG0000(	77.32958	0.429324	0.102998	4.168286	3.07E-05	9.78E-05	FTH1P16	pseudoge
ENSG0000(	77.29767	-0.18449	0.128643	-1.4341	0.151544	0.220761	USP32P1	pseudoge
ENSG0000(	77.29643	-0.60068	0.136134	-4.41242	1.02E-05	3.52E-05	DNMBP-A	antisense
ENSG0000(	77.27084	0.163152	0.04335	3.763547	0.000168	0.000471	FAM151B	protein_cc
ENSG0000(	77.24311	0.018252	0.145549	0.125398	0.900209	0.929152	FCGR3B	protein_cc
ENSG0000(	77.16484	-0.04991	0.071336	-0.69959	0.484183	0.579262	PATL2	protein_cc
ENSG0000(	77.15509	-0.60129	0.112455	-5.34693	8.95E-08	4.27E-07	VSTM5	protein_cc
ENSG0000(	77.13006	-0.76556	0.082941	-9.23014	2.70E-20	9.60E-19	UPP2	protein_cc
ENSG0000(	77.12308	-0.85567	0.111923	-7.64516	2.09E-14	2.86E-13	FRMPD2	protein_cc
ENSG0000(	77.04108	-1.18397	0.144657	-8.18469	2.73E-16	5.16E-15	PTGER3	protein_cc
ENSG0000(	77.0117	0.287642	0.103371	2.782627	0.005392	0.011418	NCF1C	pseudoge
ENSG0000(	77.0109	0.62717	0.112788	5.560596	2.69E-08	1.39E-07	RP11-680	antisense
ENSG0000(	76.96468	-0.6413	0.142315	-4.50621	6.60E-06	2.34E-05	TNFRSF8	protein_cc
ENSG0000(	76.91876	0.971695	0.12352	7.866721	3.64E-15	5.74E-14	DNAAF3	protein_cc
ENSG0000(	76.79902	0.533024	0.107257	4.969604	6.71E-07	2.79E-06	GPR157	protein_cc
ENSG0000(	76.79	0.591936	0.080193	7.381365	1.57E-13	1.85E-12	CTD-2162	protein_cc
ENSG0000(	76.76861	0.087562	0.069271	1.264053	0.206211	0.28661	AC009237	pseudoge
ENSG0000(	76.76515	0.279138	0.116528	2.395465	0.016599	0.031529	DNAI1	protein_cc
ENSG0000(	76.72583	0.202494	0.105803	1.913881	0.055635	0.092757	RP11-453	lincRNA
ENSG0000(	76.68965	-0.07081	0.144493	-0.49004	0.624109	0.706367	ITGBL1	protein_cc
ENSG0000(	76.67755	-0.11306	0.050802	-2.2255	0.026048	0.047257	CTC-429P	antisense
ENSG0000(	76.61253	-0.06391	0.108	-0.5918	0.553986	0.643581	MGAM	protein_cc
ENSG0000(	76.61074	0.013704	0.07412	0.184895	0.853311	0.893847	RP5-902P	lincRNA
ENSG0000(	76.56827	-0.54818	0.100298	-5.46555	4.61E-08	2.31E-07	P2RX5	protein_cc
ENSG0000(	76.51788	-0.7716	0.119247	-6.47059	9.76E-11	7.45E-10	SLC9A2	protein_cc
ENSG0000(	76.51484	-0.10461	0.109293	-0.95718	0.338477	0.433428	MUC4	protein_cc
ENSG0000(	76.48162	0.094825	0.033759	2.808845	0.004972	0.0106	AP000487	antisense
ENSG0000(	76.46999	0.280981	0.053831	5.219637	1.79E-07	8.15E-07	WASH7P	pseudoge
ENSG0000(	76.45757	-0.07115	0.092723	-0.76737	0.442859	0.539698	UPK2	protein_cc
ENSG0000(	76.42945	0.690165	0.149162	4.626959	3.71E-06	1.37E-05	FTLP2	pseudoge

ENSG0000(	76.42027	-0.04662	0.053045	-0.8788	0.37951	0.47609	RP11-110	lincRNA
ENSG0000(	76.41935	0.354892	0.095291	3.724294	0.000196	0.000544	CTD-3092	sense_intr
ENSG0000(	76.4181	0.09198	0.071784	1.281356	0.200069	0.279347	VTN	protein_cc
ENSG0000(	76.32897	0.15041	0.044369	3.389936	0.000699	0.001753	RP11-347	lincRNA
ENSG0000(	76.16457	0.366589	0.068778	5.330023	9.82E-08	4.66E-07	C15orf65	protein_cc
ENSG0000(	76.14431	-1.80665	0.260237	-6.94231	3.86E-12	3.66E-11	LINC0050	lincRNA
ENSG0000(	76.08215	-0.59351	0.083443	-7.11273	1.14E-12	1.17E-11	SMIM17	protein_cc
ENSG0000(	76.07829	0.425648	0.082547	5.156462	2.52E-07	1.12E-06	RPL4P5	pseudoge
ENSG0000(	76.06313	-0.20349	0.059236	-3.43527	0.000592	0.001507	ZNF460	protein_cc
ENSG0000(	76.02619	0.375814	0.065855	5.706704	1.15E-08	6.33E-08	PBX4	protein_cc
ENSG0000(	75.99459	-0.13036	0.054729	-2.38197	0.01722	0.032544	SYS1-DBN	protein_cc
ENSG0000(	75.95732	-1.10022	0.188027	-5.85139	4.87E-09	2.84E-08	HTR1A	protein_cc
ENSG0000(	75.93285	0.607767	0.10385	5.852337	4.85E-09	2.82E-08	ZC3HAV1	protein_cc
ENSG0000(	75.93173	-0.37084	0.087712	-4.22793	2.36E-05	7.65E-05	AIM2	protein_cc
ENSG0000(	75.91707	-0.05362	0.070308	-0.76266	0.445664	0.542443	RP5-1142	antisense
ENSG0000(	75.91092	-0.69661	0.083355	-8.35717	6.42E-17	1.35E-15	CCDC68	protein_cc
ENSG0000(	75.89401	-0.74583	0.116493	-6.40237	1.53E-10	1.13E-09	AC006466	pseudoge
ENSG0000(	75.8916	1.833928	0.256074	7.161702	7.97E-13	8.41E-12	HOXA7	protein_cc
ENSG0000(	75.86816	1.032822	0.105096	9.82745	8.58E-23	4.81E-21	ERCC6L	protein_cc
ENSG0000(	75.81546	-0.23791	0.081304	-2.9262	0.003431	0.007561	NAIP	protein_cc
ENSG0000(	75.80612	-0.01368	0.064262	-0.21295	0.831367	0.876914	SCGB1B2F	lincRNA
ENSG0000(	75.77361	-0.13718	0.061603	-2.22682	0.025959	0.047117	UBE2Q2P3	pseudoge
ENSG0000(	75.70533	-0.21231	0.110084	-1.92864	0.053776	0.090038	GLYCTK-A	processed
ENSG0000(	75.68724	0.143208	0.111356	1.286039	0.19843	0.277332	NKG7	protein_cc
ENSG0000(	75.66352	-0.09035	0.056978	-1.58578	0.11279	0.171404	SPATA25	protein_cc
ENSG0000(	75.64418	0.223622	0.039476	5.664707	1.47E-08	7.96E-08	RP11-266	pseudoge
ENSG0000(	75.61005	-0.01976	0.121912	-0.16205	0.871266	0.907447	ICOSLG	protein_cc
ENSG0000(	75.57589	-0.07777	0.093569	-0.83118	0.405872	0.502728	ALG1L	protein_cc
ENSG0000(	75.56557	0.033296	0.064769	0.514082	0.607195	0.691065	AC027601	antisense
ENSG0000(	75.48854	0.020411	0.08696	0.234718	0.814428	0.863699	LINC0112	lincRNA
ENSG0000(	75.48279	-0.20676	0.108017	-1.91419	0.055596	0.092706	C21orf37	protein_cc
ENSG0000(	75.41579	-0.21932	0.083883	-2.61462	0.008933	0.018045	RP11-400	sense_intr
ENSG0000(	75.39696	-0.10082	0.101277	-0.99547	0.319508	0.413203	RP11-776	lincRNA
ENSG0000(	75.27364	0.579147	0.094624	6.120534	9.33E-10	6.11E-09	CTD-2341	lincRNA
ENSG0000(	75.24082	0.160917	0.049876	3.226323	0.001254	0.003001	FAM86HP	pseudoge
ENSG0000(	75.20697	-0.11737	0.081672	-1.43707	0.150698	0.219685	RP11-6O2	antisense
ENSG0000(	75.19417	-0.0994	0.050415	-1.97158	0.048657	0.082481	CTD-2319	processed
ENSG0000(	75.17514	-3.00804	0.244354	-12.3102	7.98E-35	4.83E-32	CARTPT	protein_cc
ENSG0000(	75.13236	0.166272	0.114375	1.453743	0.146018	0.213718	ARMC4	protein_cc
ENSG0000(	75.11642	-0.81879	0.124104	-6.59761	4.18E-11	3.37E-10	OTOF	protein_cc
ENSG0000(	75.10215	0.338127	0.049569	6.821337	9.02E-12	8.06E-11	ATP6V1C2	protein_cc
ENSG0000(	75.09052	0.211244	0.040481	5.218391	1.80E-07	8.20E-07	NOX1	protein_cc
ENSG0000(	75.06888	-0.50658	0.180263	-2.81021	0.004951	0.010561	NDST4	protein_cc
ENSG0000(	75.02641	0.598438	0.14611	4.095802	4.21E-05	0.000131	RP11-599	pseudoge
ENSG0000(	75.01717	0.000927	0.057485	0.016119	0.987139	0.991121	RP11-329	lincRNA
ENSG0000(	74.95409	0.239934	0.068781	3.488357	0.000486	0.001257	RP11-214	lincRNA
ENSG0000(	74.83443	-0.10222	0.043841	-2.33168	0.019718	0.036744	ASB14	protein_cc
ENSG0000(	74.80795	-0.55564	0.095932	-5.79203	6.95E-09	3.96E-08	PLA1A	protein_cc
ENSG0000(	74.77395	0.794245	0.095181	8.344588	7.15E-17	1.49E-15	RP11-159	pseudoge
ENSG0000(	74.70449	0.624952	0.084598	7.387303	1.50E-13	1.77E-12	RP11-424	pseudoge
ENSG0000(	74.64792	0.35991	0.073424	4.901771	9.50E-07	3.85E-06	RP11-147	pseudoge
ENSG0000(	74.6089	0.084513	0.040531	2.08512	0.037058	0.064851	SNX15	protein_cc
ENSG0000(	74.59314	-0.91358	0.101668	-8.98589	2.57E-19	7.84E-18	BSPRY	protein_cc
ENSG0000(	74.58607	0.088878	0.047087	1.887548	0.059087	0.097762	RP11-467	antisense
ENSG0000(	74.58458	-0.59116	0.071425	-8.27663	1.27E-16	2.53E-15	FUT1	protein_cc
ENSG0000(	74.55084	-0.90433	0.154534	-5.85201	4.86E-09	2.83E-08	BHLHE22	protein_cc
ENSG0000(	74.38686	-0.12903	0.061637	-2.09335	0.036318	0.063701	C15orf62	protein_cc
ENSG0000(	74.37098	-0.99333	0.139196	-7.13616	9.60E-13	1.00E-11	GSDMC	protein_cc
ENSG0000(	74.35442	-0.39491	0.055714	-7.08804	1.36E-12	1.39E-11	ACKR2	protein_cc

ENSG0000(	74.32601	0.197804	0.053455	3.700393	0.000215	0.000593	RP11-723(	antisense
ENSG0000(	74.2946	-0.10809	0.068373	-1.58087	0.113908	0.172853	CTC-471F	pseudoge
ENSG0000(	74.24701	-0.61139	0.087724	-6.96944	3.18E-12	3.06E-11	B3GNT4	protein_cc
ENSG0000(	74.13711	0.054206	0.070473	0.769171	0.441792	0.53859	NPIPB3	protein_cc
ENSG0000(	74.11135	-0.11062	0.045546	-2.42884	0.015147	0.029037	IL10RB-AS	antisense
ENSG0000(	74.06132	-0.11296	0.046468	-2.43091	0.015061	0.02889	CTD-2547	antisense
ENSG0000(	73.98949	-0.37434	0.083468	-4.48484	7.30E-06	2.58E-05	RSPO4	protein_cc
ENSG0000(	73.98078	-0.70619	0.131565	-5.36765	7.98E-08	3.84E-07	CCDC144I	pseudoge
ENSG0000(	73.95136	-1.9178	0.160593	-11.942	7.15E-33	3.00E-30	SOWAHB	protein_cc
ENSG0000(	73.90466	0.020658	0.042509	0.485952	0.627001	0.708763	RP1-30M3	antisense
ENSG0000(	73.8542	0.570938	0.09443	6.046176	1.48E-09	9.39E-09	RP11-641	pseudoge
ENSG0000(	73.84124	0.298538	0.057387	5.202178	1.97E-07	8.91E-07	HESX1	protein_cc
ENSG0000(	73.75657	-0.36432	0.090543	-4.02369	5.73E-05	0.000175	ATP1A4	protein_cc
ENSG0000(	73.74797	0.440499	0.081613	5.397389	6.76E-08	3.29E-07	RP11-103(	antisense
ENSG0000(	73.65725	-0.21915	0.078344	-2.79733	0.005153	0.010954	PHOSPHC	protein_cc
ENSG0000(	73.64505	0.08931	0.055079	1.621483	0.104914	0.161024	AC005253	antisense
ENSG0000(	73.59903	0.060908	0.039327	1.548749	0.121442	0.182483	RP11-35G	antisense
ENSG0000(	73.59481	0.74308	0.110351	6.733775	1.65E-11	1.42E-10	RP11-103	pseudoge
ENSG0000(	73.56111	1.097145	0.166197	6.601488	4.07E-11	3.29E-10	RP3-460G	lincRNA
ENSG0000(	73.49961	-0.368	0.106172	-3.46605	0.000528	0.001357	LINC0040	lincRNA
ENSG0000(	73.45578	0.115387	0.079728	1.447257	0.147825	0.21604	RTDR1	protein_cc
ENSG0000(	73.43715	0.313941	0.08821	3.559029	0.000372	0.000985	WBSCR27	protein_cc
ENSG0000(	73.40179	-0.09078	0.100419	-0.90397	0.366012	0.462097	PKD1L2	polymorpl
ENSG0000(	73.39428	0.568426	0.128766	4.414397	1.01E-05	3.49E-05	MYZAP	protein_cc
ENSG0000(	73.38674	-0.09253	0.08966	-1.03197	0.302084	0.394767	RP11-420(	lincRNA
ENSG0000(	73.37963	0.069866	0.050272	1.389756	0.164603	0.236802	RP11-727	processed
ENSG0000(	73.34528	-1.3662	0.160768	-8.49798	1.93E-17	4.41E-16	RP11-389	sense_ove
ENSG0000(	73.31421	-0.26754	0.102967	-2.59833	0.009368	0.018831	PABPC1P4	pseudoge
ENSG0000(	73.2378	1.475527	0.170072	8.675899	4.10E-18	1.04E-16	MAP1LC3(	protein_cc
ENSG0000(	73.21758	0.508547	0.155005	3.280837	0.001035	0.002514	RP11-524	antisense
ENSG0000(	73.21233	-0.58742	0.144248	-4.07232	4.65E-05	0.000144	CD300LG	protein_cc
ENSG0000(	73.18909	-0.34794	0.049805	-6.98602	2.83E-12	2.74E-11	TCP10L	protein_cc
ENSG0000(	73.18416	-0.51737	0.095118	-5.43922	5.35E-08	2.65E-07	CTD-2210	protein_cc
ENSG0000(	73.1673	-0.36738	0.062899	-5.84088	5.19E-09	3.01E-08	RP11-383	lincRNA
ENSG0000(	73.12686	-0.12753	0.066943	-1.90499	0.056781	0.094411	TMEM51-	processed
ENSG0000(	73.10257	0.112811	0.067899	1.661463	0.096621	0.149963	RPL9P29	pseudoge
ENSG0000(	73.04845	0.024008	0.056071	0.42818	0.66852	0.744099	RP11-796	antisense
ENSG0000(	72.99581	-0.3968	0.076308	-5.19993	1.99E-07	9.00E-07	M1AP	protein_cc
ENSG0000(	72.9865	-0.07143	0.16716	-0.4273	0.669162	0.744592	RP1-293L(	antisense
ENSG0000(	72.98462	-0.44827	0.114786	-3.90529	9.41E-05	0.000276	RP11-180	lincRNA
ENSG0000(	72.97045	0.103146	0.038933	2.649293	0.008066	0.016464	AL136419	processed
ENSG0000(	72.93665	-0.44796	0.067888	-6.59846	4.15E-11	3.36E-10	AC093110	antisense
ENSG0000(	72.93612	-0.23085	0.081269	-2.84056	0.004504	0.00968	ERVMER3	protein_cc
ENSG0000(	72.81414	-0.00297	0.067786	-0.04375	0.965103	0.976116	RP11-204	lincRNA
ENSG0000(	72.80029	0.025936	0.059787	0.43381	0.664426	0.740926	PRPS1P2	pseudoge
ENSG0000(	72.79018	-0.98571	0.114221	-8.62989	6.14E-18	1.52E-16	RBM11	protein_cc
ENSG0000(	72.73679	0.002777	0.062158	0.044669	0.964371	0.97562	ZNF337-A	antisense
ENSG0000(	72.7034	-0.03084	0.129781	-0.2376	0.812189	0.862014	CCDC108	protein_cc
ENSG0000(	72.63311	0.195105	0.138262	1.411132	0.158206	0.228772	CLEC17A	protein_cc
ENSG0000(	72.62062	0.371749	0.106926	3.476702	0.000508	0.001308	SLC16A11	protein_cc
ENSG0000(	72.59258	-0.62318	0.176113	-3.53852	0.000402	0.001058	IL1RL1	protein_cc
ENSG0000(	72.54875	-1.74488	0.179086	-9.74323	1.97E-22	1.03E-20	PCDH11Y	protein_cc
ENSG0000(	72.53684	0.143707	0.061674	2.330085	0.019802	0.036887	RP11-97C	sense_intr
ENSG0000(	72.43639	-0.79259	0.119939	-6.60831	3.89E-11	3.16E-10	RTBDN	protein_cc
ENSG0000(	72.3982	-0.47229	0.10605	-4.45345	8.45E-06	2.95E-05	DIO3OS	lincRNA
ENSG0000(	72.35571	-0.21241	0.105235	-2.01842	0.043548	0.074778	ASB4	protein_cc
ENSG0000(	72.24466	-0.06134	0.080469	-0.76233	0.445861	0.542633	RHEBL1	protein_cc
ENSG0000(	72.2159	0.254186	0.075443	3.369248	0.000754	0.001878	RP11-996	antisense
ENSG0000(	72.20153	-0.01478	0.084264	-0.17541	0.860756	0.899516	RP11-92G	lincRNA

ENSG0000(	72.19812	0.032101	0.14966	0.214494	0.830161	0.87589	COL2A1	protein_cc
ENSG0000(	72.14956	0.517718	0.146797	3.526773	0.000421	0.001102	CES1	protein_cc
ENSG0000(	72.11252	0.51144	0.128527	3.979247	6.91E-05	0.000208	KRBOX1	protein_cc
ENSG0000(	72.02901	0.324824	0.089758	3.618868	0.000296	0.000797	ZNF730	protein_cc
ENSG0000(	72.02888	-0.23277	0.078846	-2.95226	0.003155	0.006998	C19orf81	protein_cc
ENSG0000(	72.00883	-0.03016	0.12419	-0.24289	0.808091	0.858719	CLUL1	protein_cc
ENSG0000(	71.95385	-0.87676	0.119598	-7.33085	2.29E-13	2.62E-12	ASB2	protein_cc
ENSG0000(	71.9192	-0.44981	0.130458	-3.4479	0.000565	0.001443	RP3-395M	lincRNA
ENSG0000(	71.91701	0.140027	0.056697	2.469734	0.013521	0.026221	CBWD3	protein_cc
ENSG0000(	71.9133	-0.29017	0.088144	-3.29202	0.000995	0.002423	NEK10	protein_cc
ENSG0000(	71.87858	-0.34035	0.143331	-2.37455	0.01757	0.033125	INHBA-AS	antisense
ENSG0000(	71.87833	-0.65794	0.133103	-4.94304	7.69E-07	3.16E-06	RAB6C-AS	pseudoge
ENSG0000(	71.85028	0.268939	0.06152	4.371544	1.23E-05	4.20E-05	LSMEM1	protein_cc
ENSG0000(	71.83451	-0.87846	0.120826	-7.2705	3.58E-13	3.98E-12	PPEF1	protein_cc
ENSG0000(	71.77448	0.611553	0.095168	6.42607	1.31E-10	9.81E-10	RP11-20C	pseudoge
ENSG0000(	71.768	0.43514	0.088414	4.921603	8.58E-07	3.50E-06	NEURL3	pseudoge
ENSG0000(	71.76171	0.381292	0.095782	3.980831	6.87E-05	0.000207	MBOAT1	protein_cc
ENSG0000(	71.72149	-0.37513	0.077512	-4.83958	1.30E-06	5.17E-06	RP11-125	antisense
ENSG0000(	71.62519	0.091859	0.045138	2.035075	0.041843	0.07221	RP11-539	lincRNA
ENSG0000(	71.61274	0.758167	0.090866	8.343794	7.19E-17	1.50E-15	RPS15AP1	pseudoge
ENSG0000(	71.6124	0.284451	0.09762	2.913865	0.00357	0.007839	WARS2-IT	antisense
ENSG0000(	71.58844	0.214615	0.063632	3.372758	0.000744	0.001856	EFCAB4B	protein_cc
ENSG0000(	71.58789	0.367043	0.074105	4.953043	7.31E-07	3.02E-06	RP11-638	lincRNA
ENSG0000(	71.57977	-0.46076	0.096117	-4.79373	1.64E-06	6.40E-06	RP11-480	lincRNA
ENSG0000(	71.53705	-0.1349	0.088136	-1.53061	0.125866	0.188242	TPH1	protein_cc
ENSG0000(	71.51734	-0.01653	0.066065	-0.25028	0.80237	0.854008	RP3-402G	antisense
ENSG0000(	71.50235	0.292335	0.094824	3.082936	0.00205	0.004721	ADIRF-AS	processed
ENSG0000(	71.4802	-0.00905	0.072551	-0.12469	0.900767	0.929541	RP11-66N	antisense
ENSG0000(	71.44415	0.909886	0.112739	8.070698	6.99E-16	1.24E-14	BEST4	protein_cc
ENSG0000(	71.43075	-0.21962	0.046088	-4.76524	1.89E-06	7.30E-06	CTC-205M	sense_ove
ENSG0000(	71.35897	-0.16575	0.092872	-1.78472	0.074306	0.119375	RP11-54D	pseudoge
ENSG0000(	71.35494	0.255134	0.145697	1.751131	0.079923	0.12716	RP11-347	lincRNA
ENSG0000(	71.34451	-0.10411	0.139296	-0.7474	0.454824	0.551485	VDR	protein_cc
ENSG0000(	71.29307	-0.12091	0.100933	-1.19795	0.230935	0.314971	SYNPO2L	protein_cc
ENSG0000(	71.26768	-0.50945	0.061756	-8.24943	1.59E-16	3.13E-15	ABCC11	protein_cc
ENSG0000(	71.12417	-0.23289	0.147317	-1.58085	0.113911	0.172853	RP11-153	pseudoge
ENSG0000(	71.10027	0.032603	0.076011	0.428919	0.667982	0.743762	PPM1N	protein_cc
ENSG0000(	71.05011	-0.10014	0.08521	-1.17521	0.23991	0.325247	MOV10L1	protein_cc
ENSG0000(	71.00613	0.593423	0.080525	7.369428	1.71E-13	2.00E-12	DIRC3	protein_cc
ENSG0000(	70.93106	1.439299	0.090199	15.95695	2.55E-57	1.75E-53	RP11-156	antisense
ENSG0000(	70.8302	-0.72038	0.122336	-5.88855	3.90E-09	2.31E-08	DPP10-AS	antisense
ENSG0000(	70.82054	-0.10385	0.052299	-1.9857	0.047066	0.080055	CTD-2547	pseudoge
ENSG0000(	70.81431	-0.01938	0.064083	-0.30234	0.762389	0.821634	EFCAB13	protein_cc
ENSG0000(	70.76926	0.058053	0.065153	0.891018	0.372919	0.469405	AC007292	antisense
ENSG0000(	70.63698	0.014154	0.06907	0.204917	0.837637	0.881899	RP11-876	antisense
ENSG0000(	70.63109	-0.49023	0.092437	-5.30336	1.14E-07	5.34E-07	ARHGEF16	protein_cc
ENSG0000(	70.57245	-1.41119	0.168709	-8.36463	6.03E-17	1.27E-15	CHRNA2	protein_cc
ENSG0000(	70.56408	-0.58149	0.09269	-6.2735	3.53E-10	2.47E-09	RP11-273	lincRNA
ENSG0000(	70.55744	0.411343	0.111536	3.687981	0.000226	0.000621	CNTNAP3	protein_cc
ENSG0000(	70.54844	0.23269	0.075916	3.0651	0.002176	0.004984	AC005785	processed
ENSG0000(	70.54104	0.40606	0.078111	5.198464	2.01E-07	9.07E-07	C8orf48	protein_cc
ENSG0000(	70.54043	0.309157	0.051842	5.963413	2.47E-09	1.51E-08	CTD-2554	pseudoge
ENSG0000(	70.49593	-0.25137	0.076804	-3.27286	0.001065	0.002581	ST3GAL6-	antisense
ENSG0000(	70.49146	0.155081	0.049727	3.118652	0.001817	0.004224	RP3-467N	antisense
ENSG0000(	70.48794	0.2174	0.065885	3.2997	0.000968	0.002364	CLDND2	protein_cc
ENSG0000(	70.48464	-0.02874	0.052615	-0.54623	0.584907	0.67131	RP11-102	pseudoge
ENSG0000(	70.47615	0.007287	0.079051	0.09218	0.926555	0.948418	AC093620	antisense
ENSG0000(	70.45005	0.153536	0.04896	3.135918	0.001713	0.004004	C19orf73	protein_cc
ENSG0000(	70.42938	0.206244	0.073912	2.790389	0.005264	0.011172	GS1-259H	lincRNA

ENSG0000(	70.40747	-0.7596	0.104149	-7.29342	3.02E-13	3.40E-12	ADAMTS1	protein_cc
ENSG0000(	70.3631	1.004802	0.148253	6.777621	1.22E-11	1.07E-10	RPL12P4	pseudoge
ENSG0000(	70.31781	0.938291	0.301443	3.112665	0.001854	0.004303	PAX3	protein_cc
ENSG0000(	70.29237	-0.17205	0.052176	-3.2974	0.000976	0.002381	ZEB2-AS1	antisense
ENSG0000(	70.2875	0.433314	0.063316	6.843651	7.72E-12	6.98E-11	RP11-696	antisense
ENSG0000(	70.21846	-0.16737	0.090621	-1.84696	0.064753	0.105851	PCDHB11	protein_cc
ENSG0000(	70.19685	-0.78755	0.165466	-4.75956	1.94E-06	7.49E-06	RP11-707	pseudoge
ENSG0000(	70.16725	0.039687	0.052991	0.74893	0.453899	0.550485	RP11-529	sense_ove
ENSG0000(	70.11178	0.816553	0.118099	6.91414	4.71E-12	4.40E-11	CTC-297N	antisense
ENSG0000(	70.10512	-0.19189	0.099097	-1.93639	0.05282	0.088657	CD6	protein_cc
ENSG0000(	70.09882	0.01543	0.082278	0.187535	0.851241	0.892362	ZNF578	protein_cc
ENSG0000(	70.08422	0.365655	0.055936	6.536963	6.28E-11	4.93E-10	RP11-548	antisense
ENSG0000(	70.03304	0.0105	0.046042	0.228061	0.819599	0.867746	AL645728	protein_cc
ENSG0000(	69.97069	1.10052	0.148266	7.422597	1.15E-13	1.38E-12	NCMAP	protein_cc
ENSG0000(	69.96772	-0.17797	0.074192	-2.39881	0.016449	0.031271	TMEM139	protein_cc
ENSG0000(	69.94067	0.261489	0.059011	4.431203	9.37E-06	3.25E-05	SRRM2-AS	antisense
ENSG0000(	69.92476	0.426271	0.057202	7.45209	9.19E-14	1.13E-12	MAMDC2-	antisense
ENSG0000(	69.88474	0.895982	0.104612	8.564804	1.08E-17	2.58E-16	HNRNPKP	pseudoge
ENSG0000(	69.86159	-0.05258	0.079809	-0.65886	0.509985	0.603233	RP11-848	lincRNA
ENSG0000(	69.85576	-0.01146	0.079931	-0.14343	0.885948	0.918768	RP11-496	pseudoge
ENSG0000(	69.84275	0.165922	0.091632	1.810738	0.070181	0.11357	CTD-3157	lincRNA
ENSG0000(	69.82572	-0.18588	0.10473	-1.77486	0.075921	0.121609	EMR4P	pseudoge
ENSG0000(	69.78839	-0.20308	0.113147	-1.79483	0.07268	0.117134	STEAP4	protein_cc
ENSG0000(	69.71953	-0.17629	0.052735	-3.34288	0.000829	0.002052	CCDC148	protein_cc
ENSG0000(	69.70127	0.232748	0.055119	4.222617	2.41E-05	7.82E-05	RP11-18H	lincRNA
ENSG0000(	69.6315	0.04697	0.038636	1.215701	0.224099	0.307275	RP11-216	antisense
ENSG0000(	69.6202	0.497686	0.090458	5.501849	3.76E-08	1.91E-07	IQUB	protein_cc
ENSG0000(	69.60768	0.585812	0.088886	6.590573	4.38E-11	3.53E-10	AC147651	lincRNA
ENSG0000(	69.60044	-0.29434	0.067201	-4.37996	1.19E-05	4.05E-05	ANO2	protein_cc
ENSG0000(	69.54785	0.227727	0.147831	1.540462	0.123448	0.185111	CD2	protein_cc
ENSG0000(	69.50954	0.884956	0.151907	5.825658	5.69E-09	3.28E-08	TGM5	protein_cc
ENSG0000(	69.48303	-0.00723	0.067074	-0.10779	0.914165	0.9395	RP11-804	lincRNA
ENSG0000(	69.47766	-0.35552	0.077608	-4.58099	4.63E-06	1.69E-05	GVINP1	pseudoge
ENSG0000(	69.42863	-1.14921	0.130214	-8.8255	1.09E-18	3.01E-17	CCDC177	protein_cc
ENSG0000(	69.35789	1.373909	0.170183	8.073108	6.85E-16	1.22E-14	FEZF1-AS	antisense
ENSG0000(	69.34033	0.062573	0.101839	0.614432	0.53893	0.629895	DDX11L2	pseudoge
ENSG0000(	69.30256	0.890592	0.11701	7.611233	2.71E-14	3.63E-13	PTMAP2	pseudoge
ENSG0000(	69.26154	-0.56782	0.144264	-3.93596	8.29E-05	0.000246	RP11-547	antisense
ENSG0000(	69.24254	-0.24795	0.068378	-3.62617	0.000288	0.000777	RP11-290	lincRNA
ENSG0000(	69.2018	0.596272	0.177712	3.355269	0.000793	0.001969	FOXD3	protein_cc
ENSG0000(	69.19901	-0.20953	0.077362	-2.70845	0.00676	0.014014	TMEFF1	protein_cc
ENSG0000(	69.1839	-0.47022	0.091524	-5.13765	2.78E-07	1.23E-06	EYS	protein_cc
ENSG0000(	69.15027	0.747613	0.124185	6.020159	1.74E-09	1.09E-08	MUC12	protein_cc
ENSG0000(	69.13737	0.335409	0.047298	7.09145	1.33E-12	1.35E-11	CIRBP-AS	antisense
ENSG0000(	69.0785	0.832961	0.112048	7.433962	1.05E-13	1.28E-12	GSG2	protein_cc
ENSG0000(	69.0695	0.301963	0.097091	3.110098	0.00187	0.004337	CCDC122	protein_cc
ENSG0000(	69.03687	-0.00979	0.100915	-0.09702	0.922713	0.945592	AVPR1A	protein_cc
ENSG0000(	69.00611	-0.33726	0.107477	-3.13802	0.001701	0.003977	RP11-110	protein_cc
ENSG0000(	68.95541	0.36028	0.083564	4.311438	1.62E-05	5.41E-05	AC009005	antisense
ENSG0000(	68.9515	-0.01762	0.055348	-0.3183	0.750257	0.811892	RP5-1065	antisense
ENSG0000(	68.94898	-1.10039	0.090355	-12.1785	4.05E-34	2.11E-31	C17orf107	protein_cc
ENSG0000(	68.9425	-0.18876	0.087094	-2.16732	0.03021	0.053979	FENDRR	lincRNA
ENSG0000(	68.93107	-0.48827	0.06433	-7.59006	3.20E-14	4.23E-13	TMPPE	protein_cc
ENSG0000(	68.87422	0.144587	0.103704	1.394225	0.16325	0.235115	GPR114	protein_cc
ENSG0000(	68.84453	0.030091	0.039073	0.77011	0.441235	0.538086	ZNF205-A	antisense
ENSG0000(	68.80216	-0.63511	0.134371	-4.72656	2.28E-06	8.73E-06	RP11-482	antisense
ENSG0000(	68.74755	0.469	0.106837	4.389852	1.13E-05	3.88E-05	RP11-85M	lincRNA
ENSG0000(	68.74173	0.175818	0.183184	0.959787	0.337162	0.432026	NEUROD4	protein_cc
ENSG0000(	68.69886	-0.1743	0.057779	-3.01661	0.002556	0.005784	F10	protein_cc

ENSG0000(	68.69817	0.11334	0.067922	1.668692	0.095178	0.148027	LRRC37A5	pseudoge
ENSG0000(	68.69435	-0.0239	0.095508	-0.25027	0.802382	0.854008	RP4-625H	lincRNA
ENSG0000(	68.68988	-1.65641	0.191625	-8.644	5.43E-18	1.35E-16	HTR5A-AS	protein_cc
ENSG0000(	68.63436	0.474699	0.19467	2.438482	0.014749	0.02837	RP11-84A	antisense
ENSG0000(	68.49478	0.056825	0.040374	1.407487	0.159283	0.230168	AC099850	antisense
ENSG0000(	68.46782	-0.24317	0.081954	-2.96714	0.003006	0.006697	SYCP2L	protein_cc
ENSG0000(	68.39011	-0.17776	0.069686	-2.55093	0.010743	0.021316	AP000662	sense_ove
ENSG0000(	68.36809	-0.01294	0.092025	-0.14065	0.888144	0.920326	CILP	protein_cc
ENSG0000(	68.35595	1.984298	0.171379	11.57839	5.30E-31	1.51E-28	WNT16	protein_cc
ENSG0000(	68.35266	-0.25491	0.083285	-3.06074	0.002208	0.005053	CTD-2020	antisense
ENSG0000(	68.29627	0.830403	0.102971	8.064419	7.36E-16	1.30E-14	AGBL2	protein_cc
ENSG0000(	68.2731	0.246616	0.13765	1.791618	0.073194	0.117847	C2orf50	protein_cc
ENSG0000(	68.23003	-0.20568	0.075845	-2.71192	0.006689	0.013881	KCNJ2-AS	antisense
ENSG0000(	68.21192	-0.16461	0.060557	-2.7182	0.006564	0.013638	RP11-722	lincRNA
ENSG0000(	68.13167	-0.04392	0.073309	-0.59912	0.549093	0.639181	MANEA-A	antisense
ENSG0000(	68.13006	-0.88751	0.10772	-8.23906	1.74E-16	3.39E-15	PGR	protein_cc
ENSG0000(	68.11171	0.210215	0.050629	4.152105	3.29E-05	0.000104	SERF1B	protein_cc
ENSG0000(	68.09325	0.495555	0.093241	5.314754	1.07E-07	5.04E-07	SP6	protein_cc
ENSG0000(	68.07765	1.10611	0.118972	9.297229	1.44E-20	5.39E-19	SLC26A7	protein_cc
ENSG0000(	68.01013	-0.02168	0.097814	-0.22166	0.824578	0.871368	AC016757	protein_cc
ENSG0000(	67.98383	-0.18481	0.071556	-2.58277	0.009801	0.019618	AC144530	pseudoge
ENSG0000(	67.97459	-0.31212	0.139341	-2.23996	0.025094	0.045696	NHLH2	protein_cc
ENSG0000(	67.82373	-0.21518	0.06819	-3.15556	0.001602	0.003764	CTD-2313	processed
ENSG0000(	67.80765	-0.02729	0.108216	-0.25221	0.80088	0.852807	LILRB3	protein_cc
ENSG0000(	67.76031	0.376535	0.091933	4.095761	4.21E-05	0.000131	ZAP70	protein_cc
ENSG0000(	67.66574	0.075735	0.06713	1.128187	0.259241	0.34731	AKR7A3	protein_cc
ENSG0000(	67.62285	-0.08268	0.119509	-0.69185	0.48903	0.583634	TMEM26	protein_cc
ENSG0000(	67.61006	0.482025	0.06015	8.013741	1.11E-15	1.91E-14	RP11-390	lincRNA
ENSG0000(	67.59684	-0.69803	0.091644	-7.61669	2.60E-14	3.50E-13	SYDE2	protein_cc
ENSG0000(	67.5753	0.179717	0.056894	3.158812	0.001584	0.003725	INE1	sense_intr
ENSG0000(	67.5535	0.177219	0.076658	2.311825	0.020787	0.03855	CTD-2537	antisense
ENSG0000(	67.55322	0.185048	0.065572	2.822054	0.004772	0.010202	RP11-209	antisense
ENSG0000(	67.52394	-0.17382	0.094179	-1.84566	0.064941	0.106121	CYP17A1	protein_cc
ENSG0000(	67.48299	-0.36631	0.098013	-3.73735	0.000186	0.000518	RP11-74E	pseudoge
ENSG0000(	67.44682	-0.16584	0.045118	-3.67573	0.000237	0.000649	RP4-714D	sense_ove
ENSG0000(	67.41025	-0.18037	0.093103	-1.93737	0.0527	0.088482	HIST3H2B	protein_cc
ENSG0000(	67.39682	0.858094	0.085839	9.996553	1.58E-23	1.05E-21	MTFR2	protein_cc
ENSG0000(	67.38573	0.118609	0.066643	1.779768	0.075114	0.120485	FOXN3-AS	antisense
ENSG0000(	67.30296	0.257777	0.130113	1.981174	0.047572	0.080824	RP11-676	lincRNA
ENSG0000(	67.30149	0.144972	0.066679	2.174166	0.029693	0.05316	RP11-264	processed
ENSG0000(	67.27467	-0.4299	0.069025	-6.22818	4.72E-10	3.23E-09	BZRAP1-A	antisense
ENSG0000(	67.27077	-0.15464	0.077174	-2.00381	0.04509	0.077092	CHKB-CPT	protein_cc
ENSG0000(	67.26536	-0.51623	0.140109	-3.68449	0.000229	0.000628	CTD-2303	processed
ENSG0000(	67.25693	0.990808	0.110261	8.98602	2.56E-19	7.83E-18	RPL41P2	pseudoge
ENSG0000(	67.22367	-0.70541	0.091356	-7.72159	1.15E-14	1.65E-13	TNFSF9	protein_cc
ENSG0000(	67.20385	0.314971	0.084458	3.729312	0.000192	0.000534	MYCNOS	antisense
ENSG0000(	67.13665	-1.11523	0.134974	-8.26253	1.43E-16	2.83E-15	SIAH3	protein_cc
ENSG0000(	67.11947	0.381772	0.075169	5.078849	3.80E-07	1.64E-06	MPZL3	protein_cc
ENSG0000(	67.06696	-0.04151	0.067722	-0.61302	0.539863	0.630773	CTB-118N	antisense
ENSG0000(	67.0583	-0.59477	0.107797	-5.5175	3.44E-08	1.75E-07	CASQ2	protein_cc
ENSG0000(	67.04399	0.198112	0.083898	2.361327	0.01821	0.034224	KLKB1	protein_cc
ENSG0000(	67.01265	-1.17512	0.150153	-7.82613	5.03E-15	7.74E-14	CA7	protein_cc
ENSG0000(	66.95219	0.096668	0.059375	1.628097	0.103504	0.159148	RP11-127	lincRNA
ENSG0000(	66.93879	-0.25405	0.060256	-4.2162	2.48E-05	8.03E-05	AC027307	protein_cc
ENSG0000(	66.92224	0.362173	0.071652	5.054627	4.31E-07	1.85E-06	RP11-227	lincRNA
ENSG0000(	66.88477	-0.0765	0.046511	-1.6447	0.100033	0.154577	AC093642	pseudoge
ENSG0000(	66.87815	-0.22249	0.06557	-3.39313	0.000691	0.001735	THPO	protein_cc
ENSG0000(	66.85608	0.586992	0.076106	7.712772	1.23E-14	1.76E-13	TM6SF2	protein_cc
ENSG0000(	66.81304	-0.14619	0.083106	-1.75906	0.078567	0.125296	JMJD1C-A	antisense

ENSG0000(	66.80423	0.003011	0.059888	0.050282	0.959898	0.972526	RP11-373	antisense
ENSG0000(	66.79614	0.442433	0.050873	8.696753	3.42E-18	8.79E-17	RP11-977(	antisense
ENSG0000(	66.75115	0.361499	0.100847	3.584633	0.000338	0.0009	AC093642	lincRNA
ENSG0000(	66.74702	-0.14642	0.133761	-1.09467	0.273659	0.363606	CCNO	protein_cc
ENSG0000(	66.7341	-0.24413	0.108421	-2.25171	0.02434	0.044458	RP11-328.	antisense
ENSG0000(	66.66754	0.481951	0.0794	6.069911	1.28E-09	8.21E-09	COX4I2	protein_cc
ENSG0000(	66.66671	-0.50933	0.078889	-6.45625	1.07E-10	8.14E-10	NHLRC1	protein_cc
ENSG0000(	66.64273	-0.39617	0.066822	-5.92873	3.05E-09	1.84E-08	AOC2	protein_cc
ENSG0000(	66.59013	0.402186	0.069974	5.747653	9.05E-09	5.05E-08	HNRNPA3	pseudoge
ENSG0000(	66.56683	-0.02848	0.077475	-0.36755	0.713208	0.781278	CTB-178M	antisense
ENSG0000(	66.56334	0.007993	0.069413	0.115154	0.908323	0.935062	FAM110D	protein_cc
ENSG0000(	66.51372	-0.58203	0.106374	-5.47152	4.46E-08	2.24E-07	SLC26A9	protein_cc
ENSG0000(	66.47687	0.163787	0.08876	1.845275	0.064998	0.1062	POLRMTP	pseudoge
ENSG0000(	66.45242	0.212174	0.077093	2.752176	0.00592	0.012416	AC005197	antisense
ENSG0000(	66.41287	0.403334	0.084734	4.760013	1.94E-06	7.48E-06	HHIP-AS1	antisense
ENSG0000(	66.40831	-0.00201	0.062923	-0.03202	0.974458	0.982124	MURC	protein_cc
ENSG0000(	66.3841	-0.48166	0.125922	-3.82508	0.000131	0.000374	AP003039	lincRNA
ENSG0000(	66.28844	-0.09864	0.123338	-0.79975	0.423856	0.520909	RP11-182.	antisense
ENSG0000(	66.28677	-0.07134	0.090921	-0.78458	0.432697	0.52967	RPL7P38	pseudoge
ENSG0000(	66.2844	0.224189	0.086644	2.587469	0.009668	0.019376	RP5-1092.	antisense
ENSG0000(	66.25696	-0.36128	0.090604	-3.98745	6.68E-05	0.000201	PCDHAC1	protein_cc
ENSG0000(	66.25086	0.317576	0.109752	2.893583	0.003809	0.008317	IL15	protein_cc
ENSG0000(	66.22945	-1.13377	0.120488	-9.40983	4.97E-21	2.01E-19	NPFFR1	protein_cc
ENSG0000(	66.19613	-0.32736	0.083154	-3.93684	8.26E-05	0.000245	NTN5	protein_cc
ENSG0000(	66.16751	0.376117	0.139937	2.687759	0.007193	0.014826	WDR63	protein_cc
ENSG0000(	66.15395	-0.42593	0.070199	-6.06743	1.30E-09	8.33E-09	RP11-303	antisense
ENSG0000(	66.14776	0.581088	0.100318	5.792432	6.94E-09	3.95E-08	MAK	protein_cc
ENSG0000(	66.11965	0.512706	0.068823	7.449637	9.36E-14	1.15E-12	KCNMB3	protein_cc
ENSG0000(	66.10676	-0.09046	0.067276	-1.34455	0.178772	0.253861	RP11-398(	lincRNA
ENSG0000(	66.09123	-0.20343	0.080071	-2.54066	0.011064	0.021888	NCAM1-A	antisense
ENSG0000(	66.08478	-0.25877	0.056514	-4.57878	4.68E-06	1.70E-05	SLC4A5	protein_cc
ENSG0000(	66.0846	0.100853	0.057405	1.756887	0.078937	0.125817	AC079630	antisense
ENSG0000(	66.00599	0.082566	0.158882	0.519668	0.603295	0.687503	TFPI2	protein_cc
ENSG0000(	65.97437	0.326979	0.044641	7.32462	2.40E-13	2.73E-12	RP5-886K	antisense
ENSG0000(	65.88892	-0.40732	0.07079	-5.75391	8.72E-09	4.88E-08	UBE2Q2P1	pseudoge
ENSG0000(	65.83603	0.009444	0.066122	0.142834	0.886421	0.919049	AC000068	antisense
ENSG0000(	65.8201	-0.34875	0.109344	-3.18943	0.001426	0.00338	AL592528	antisense
ENSG0000(	65.80737	-0.0687	0.071056	-0.96683	0.333629	0.428219	AC105020	protein_cc
ENSG0000(	65.79829	-0.4709	0.091887	-5.12475	2.98E-07	1.31E-06	SLC16A8	protein_cc
ENSG0000(	65.79476	-0.36369	0.090659	-4.01162	6.03E-05	0.000183	ACOT4	protein_cc
ENSG0000(	65.78443	0.361464	0.101046	3.577238	0.000347	0.000924	EFCAB10	protein_cc
ENSG0000(	65.76716	-0.05722	0.074676	-0.76621	0.44355	0.54032	VN1R83P	pseudoge
ENSG0000(	65.7482	-0.03835	0.050971	-0.75236	0.451832	0.548423	RAB40A	protein_cc
ENSG0000(	65.74316	-0.09675	0.080375	-1.20368	0.228714	0.312552	DAND5	protein_cc
ENSG0000(	65.7295	-0.2967	0.130366	-2.27589	0.022853	0.042002	RP11-3N2	pseudoge
ENSG0000(	65.63764	-0.05604	0.129228	-0.43367	0.664526	0.741017	LRIT2	protein_cc
ENSG0000(	65.61913	0.272785	0.06339	4.303278	1.68E-05	5.60E-05	GRAP	protein_cc
ENSG0000(	65.58311	0.486332	0.138459	3.512467	0.000444	0.001158	DTHD1	protein_cc
ENSG0000(	65.51478	-0.02274	0.076951	-0.2955	0.767608	0.825671	AC073343	protein_cc
ENSG0000(	65.51263	0.483626	0.072099	6.707844	1.98E-11	1.67E-10	DYNC1I2P	pseudoge
ENSG0000(	65.46218	0.277883	0.083564	3.32539	0.000883	0.002173	USP2-AS1	antisense
ENSG0000(	65.44208	-0.06734	0.076522	-0.88	0.378857	0.475438	RP13-650.	antisense
ENSG0000(	65.41993	0.049843	0.069819	0.713895	0.475292	0.571134	CTD-2666	lincRNA
ENSG0000(	65.31625	-0.06775	0.04429	-1.5296	0.126116	0.188582	RP11-73M	sense_intr
ENSG0000(	65.22386	0.101679	0.103157	0.985679	0.324291	0.418456	SERTAD4-	antisense
ENSG0000(	65.22203	-0.15701	0.081734	-1.92097	0.054735	0.091467	ALDH8A1	protein_cc
ENSG0000(	65.17565	-0.69692	0.117303	-5.94119	2.83E-09	1.72E-08	MYOZ2	protein_cc
ENSG0000(	65.1754	-0.32045	0.089766	-3.56984	0.000357	0.000948	RP11-116	sense_ove
ENSG0000(	65.16212	-0.22201	0.086409	-2.56932	0.01019	0.020311	CTA-228A	lincRNA

ENSG0000(	65.15841	-0.30987	0.051173	-6.05536	1.40E-09	8.93E-09	KLLN	protein_cc
ENSG0000(	65.13383	0.415542	0.059752	6.954475	3.54E-12	3.38E-11	RP11-199	sense_intr
ENSG0000(	65.12937	-0.03963	0.104428	-0.37951	0.704307	0.774325	NPIPB11	protein_cc
ENSG0000(	65.04995	-0.51564	0.14406	-3.57933	0.000344	0.000917	CHRM5	protein_cc
ENSG0000(	65.04937	-0.49539	0.114757	-4.31682	1.58E-05	5.29E-05	LTK	protein_cc
ENSG0000(	65.00404	0.001697	0.059658	0.02845	0.977303	0.983956	CDH26	protein_cc
ENSG0000(	64.94906	0.03567	0.096441	0.369861	0.711486	0.779994	RP11-545.	pseudoge
ENSG0000(	64.94243	-0.08817	0.123281	-0.71517	0.474505	0.570338	DLGAP1-/	antisense
ENSG0000(	64.93283	-1.43125	0.17751	-8.06291	7.45E-16	1.31E-14	RP11-438	sense_ove
ENSG0000(	64.90545	-0.71045	0.069775	-10.1819	2.39E-24	1.84E-22	AC007743	antisense
ENSG0000(	64.8968	-0.09545	0.066201	-1.4418	0.14936	0.217997	ACER2	protein_cc
ENSG0000(	64.85139	-0.37018	0.099325	-3.72699	0.000194	0.000539	SPESP1	protein_cc
ENSG0000(	64.83588	-1.86156	0.19323	-9.63389	5.75E-22	2.74E-20	MAS1	protein_cc
ENSG0000(	64.80779	-0.00076	0.062243	-0.01215	0.990304	0.99342	ZNF32-AS	antisense
ENSG0000(	64.7907	-0.39994	0.111323	-3.59259	0.000327	0.000876	AL022393	pseudoge
ENSG0000(	64.78135	0.270788	0.047989	5.64272	1.67E-08	8.96E-08	PDF	protein_cc
ENSG0000(	64.76687	0.086694	0.181289	0.478207	0.632503	0.713707	ZNF676	protein_cc
ENSG0000(	64.76031	-0.01522	0.062388	-0.24392	0.80729	0.858099	RP11-876	sense_intr
ENSG0000(	64.75214	0.44543	0.081	5.49913	3.82E-08	1.93E-07	WDR93	protein_cc
ENSG0000(	64.7098	-0.52192	0.091627	-5.69615	1.23E-08	6.70E-08	RP11-126	processed
ENSG0000(	64.6766	0.160432	0.128714	1.246425	0.212608	0.294001	CYP11A1	protein_cc
ENSG0000(	64.64454	0.892882	0.10516	8.490674	2.05E-17	4.68E-16	RPS7P11	pseudoge
ENSG0000(	64.62395	0.328131	0.050805	6.45862	1.06E-10	8.03E-10	RP1-95L4.	pseudoge
ENSG0000(	64.56482	-0.59202	0.076766	-7.71207	1.24E-14	1.77E-13	RP5-1142.	antisense
ENSG0000(	64.46367	-0.46817	0.114576	-4.08611	4.39E-05	0.000136	RP11-384	lincRNA
ENSG0000(	64.41081	-0.11088	0.062623	-1.77062	0.076624	0.122605	ESR1	protein_cc
ENSG0000(	64.40252	-0.174	0.055392	-3.14132	0.001682	0.003936	CFL1P1	pseudoge
ENSG0000(	64.34584	0.495848	0.107647	4.606256	4.10E-06	1.51E-05	EID3	protein_cc
ENSG0000(	64.32161	-0.47895	0.102241	-4.68448	2.81E-06	1.06E-05	CORT	protein_cc
ENSG0000(	64.31835	0.142227	0.061677	2.306003	0.02111	0.039095	TMEM27	protein_cc
ENSG0000(	64.31723	0.844606	0.106639	7.920262	2.37E-15	3.84E-14	CTB-79E8	pseudoge
ENSG0000(	64.29107	0.255608	0.117153	2.181834	0.029122	0.052254	MOCOS	protein_cc
ENSG0000(	64.2295	1.02184	0.109305	9.348477	8.89E-21	3.45E-19	LRRC36	protein_cc
ENSG0000(	64.22133	-1.20907	0.124594	-9.70411	2.90E-22	1.47E-20	PRPH2	protein_cc
ENSG0000(	64.15409	-0.263	0.054106	-4.86084	1.17E-06	4.67E-06	AP000708	protein_cc
ENSG0000(	64.14003	0.486628	0.155116	3.137187	0.001706	0.003988	DLX2	protein_cc
ENSG0000(	64.12621	0.031945	0.078931	0.404715	0.685687	0.758607	RP11-464	lincRNA
ENSG0000(	64.09599	1.12465	0.133612	8.417258	3.85E-17	8.40E-16	RPL24P8	pseudoge
ENSG0000(	64.09402	-0.35371	0.06736	-5.25105	1.51E-07	6.96E-07	RGAG1	protein_cc
ENSG0000(	64.03756	-0.28298	0.101578	-2.78585	0.005339	0.011313	CHRNA7	protein_cc
ENSG0000(	63.98944	0.122713	0.052578	2.333922	0.0196	0.036551	RP11-456	lincRNA
ENSG0000(	63.97405	-0.45613	0.166289	-2.74298	0.006088	0.012736	GS1-18A1	sense_ove
ENSG0000(	63.96224	1.355019	0.143021	9.474246	2.69E-21	1.15E-19	CTB-175P	lincRNA
ENSG0000(	63.95347	-0.73872	0.096454	-7.65879	1.88E-14	2.59E-13	MLK4	protein_cc
ENSG0000(	63.88904	-0.43522	0.078291	-5.55898	2.71E-08	1.41E-07	ABO	processed
ENSG0000(	63.84448	-0.13613	0.060524	-2.24916	0.024502	0.044725	RP11-230	lincRNA
ENSG0000(	63.82282	0.056654	0.085795	0.660347	0.509031	0.602414	RP11-797	pseudoge
ENSG0000(	63.80913	-0.33277	0.100035	-3.3265	0.000879	0.002165	FTCD	protein_cc
ENSG0000(	63.78633	-0.03478	0.104451	-0.33302	0.739117	0.802775	SLC5A9	protein_cc
ENSG0000(	63.71992	0.002315	0.066194	0.034967	0.972106	0.980595	RP11-849	antisense
ENSG0000(	63.63821	0.100197	0.049852	2.009882	0.044444	0.076116	RP11-176	protein_cc
ENSG0000(	63.62979	0.367863	0.062325	5.902357	3.58E-09	2.14E-08	NDUFV2P.	pseudoge
ENSG0000(	63.58926	0.740426	0.147961	5.004191	5.61E-07	2.36E-06	FTH1P10	pseudoge
ENSG0000(	63.54266	0.089973	0.080664	1.115401	0.264679	0.353421	SLC6A16	protein_cc
ENSG0000(	63.52454	0.090782	0.032166	2.822268	0.004769	0.010197	AC083899	pseudoge
ENSG0000(	63.51693	-1.13508	0.136389	-8.32243	8.62E-17	1.77E-15	DKK2	protein_cc
ENSG0000(	63.51563	0.022424	0.13083	0.171399	0.86391	0.902101	PPP1R17	protein_cc
ENSG0000(	63.50144	-0.0406	0.068021	-0.59692	0.550564	0.640438	AP000350	protein_cc
ENSG0000(	63.50079	0.004044	0.098278	0.04115	0.967177	0.977324	CD7	protein_cc

ENSG0000(	63.44864	0.284975	0.053633	5.313397	1.08E-07	5.08E-07	RP11-529	processed
ENSG0000(	63.40821	0.483201	0.121952	3.962226	7.43E-05	0.000222	COLEC11	protein_cc
ENSG0000(	63.38604	0.207981	0.077263	2.691867	0.007105	0.014659	TMEM217	protein_cc
ENSG0000(	63.36139	-0.26747	0.093541	-2.85942	0.004244	0.009182	RP11-158	antisense
ENSG0000(	63.32833	-0.15406	0.055965	-2.75285	0.005908	0.012393	AC068580	antisense
ENSG0000(	63.32361	1.100525	0.121319	9.071311	1.18E-19	3.81E-18	NODAL	protein_cc
ENSG0000(	63.32159	0.983018	0.125643	7.823869	5.12E-15	7.86E-14	RPL35P5	pseudoge
ENSG0000(	63.31562	-0.0323	0.055279	-0.58434	0.558992	0.64806	CTC-448F	pseudoge
ENSG0000(	63.28951	-0.29393	0.100943	-2.91186	0.003593	0.007885	LINC0031(	lincRNA
ENSG0000(	63.2659	-0.35325	0.118838	-2.97253	0.002954	0.006593	RP11-267	lincRNA
ENSG0000(	63.23555	0.442773	0.104865	4.222296	2.42E-05	7.83E-05	ULBP2	protein_cc
ENSG0000(	63.19486	0.144182	0.068244	2.112738	0.034623	0.061031	C22orf34	protein_cc
ENSG0000(	63.16884	0.13157	0.054123	2.430936	0.01506	0.028889	ASB16	protein_cc
ENSG0000(	63.12032	0.130987	0.055091	2.37764	0.017424	0.032894	RP4-612B	antisense
ENSG0000(	63.09024	0.016517	0.087652	0.188444	0.850529	0.891887	RPS4XP22	pseudoge
ENSG0000(	63.0436	-0.29453	0.077588	-3.79613	0.000147	0.000417	RP13-977.	lincRNA
ENSG0000(	62.9867	-0.01064	0.054884	-0.19379	0.84634	0.888628	RTEL1	protein_cc
ENSG0000(	62.95222	-0.24961	0.069316	-3.60107	0.000317	0.000849	RP11-247.	antisense
ENSG0000(	62.94783	0.042889	0.10369	0.413621	0.679152	0.753018	HMSD	protein_cc
ENSG0000(	62.86963	0.02477	0.073153	0.338607	0.734906	0.799299	HCG4P3	pseudoge
ENSG0000(	62.85572	0.241967	0.053838	4.494384	6.98E-06	2.47E-05	HNRNPA1	pseudoge
ENSG0000(	62.85251	0.920948	0.087109	10.57235	4.00E-26	4.36E-24	SKP1P1	pseudoge
ENSG0000(	62.82154	0.190402	0.052135	3.652073	0.00026	0.000707	RP5-1112	antisense
ENSG0000(	62.79233	0.493023	0.088107	5.595723	2.20E-08	1.16E-07	RPL3P2	pseudoge
ENSG0000(	62.78375	0.264353	0.031934	8.278223	1.25E-16	2.50E-15	GAS5-AS1	antisense
ENSG0000(	62.67032	-0.61643	0.08656	-7.12143	1.07E-12	1.11E-11	TMC1	protein_cc
ENSG0000(	62.60327	-0.54672	0.11578	-4.72203	2.34E-06	8.91E-06	C17orf102	protein_cc
ENSG0000(	62.58315	0.25192	0.099707	2.526606	0.011517	0.02269	RP6-99M1	lincRNA
ENSG0000(	62.56075	-0.00254	0.086003	-0.02955	0.976425	0.983387	LINC0010(	lincRNA
ENSG0000(	62.55322	-0.08723	0.150855	-0.57821	0.563123	0.651694	CPSF1P1	pseudoge
ENSG0000(	62.54574	0.109012	0.110551	0.986077	0.324095	0.418255	FAM177B	protein_cc
ENSG0000(	62.53605	-0.06425	0.063036	-1.01919	0.308114	0.401193	RP11-344	lincRNA
ENSG0000(	62.52657	-0.06724	0.065655	-1.0242	0.305743	0.398686	RP11-643	antisense
ENSG0000(	62.4743	0.254933	0.088762	2.872111	0.004077	0.008854	CTD-2201	antisense
ENSG0000(	62.44021	0.180411	0.074559	2.419711	0.015533	0.029703	ADAM32	protein_cc
ENSG0000(	62.40382	-0.17073	0.050502	-3.38071	0.000723	0.001809	ACAD11	protein_cc
ENSG0000(	62.3912	-0.20839	0.085586	-2.43486	0.014898	0.028614	RP11-344	lincRNA
ENSG0000(	62.38315	0.216044	0.111565	1.936481	0.052809	0.088641	GLYATL2	protein_cc
ENSG0000(	62.38285	-0.35813	0.084174	-4.25461	2.09E-05	6.87E-05	RP11-118	lincRNA
ENSG0000(	62.35434	0.242944	0.067017	3.625089	0.000289	0.00078	CTB-50E1	pseudoge
ENSG0000(	62.33263	0.049295	0.040592	1.214396	0.224597	0.307826	AC074138	lincRNA
ENSG0000(	62.30883	0.330112	0.080722	4.089476	4.32E-05	0.000134	CTD-2517	antisense
ENSG0000(	62.30596	0.471817	0.144599	3.26293	0.001103	0.002666	ITK	protein_cc
ENSG0000(	62.25914	0.022558	0.059785	0.37732	0.705936	0.775543	RP11-46J2	antisense
ENSG0000(	62.25628	-0.24128	0.07709	-3.1298	0.001749	0.004081	RP11-531.	antisense
ENSG0000(	62.21978	0.040195	0.068555	0.586324	0.557658	0.64686	CTD-2574	antisense
ENSG0000(	62.17154	0.074724	0.106889	0.699082	0.484501	0.579498	DBH-AS1	antisense
ENSG0000(	62.12674	0.09969	0.044934	2.218576	0.026516	0.048036	RP5-997D	antisense
ENSG0000(	62.11301	-0.2951	0.076934	-3.83578	0.000125	0.00036	SLC16A5	protein_cc
ENSG0000(	62.08384	-0.16957	0.066665	-2.54362	0.010971	0.021723	RP4-565E(	sense_intr
ENSG0000(	62.06917	0.812757	0.074124	10.96488	5.64E-28	8.46E-26	LINC0032	lincRNA
ENSG0000(	62.04698	-0.49071	0.069841	-7.02617	2.12E-12	2.10E-11	LINC0051!	antisense
ENSG0000(	62.0436	-0.58624	0.160573	-3.65091	0.000261	0.00071	WNT2	protein_cc
ENSG0000(	61.98898	0.491728	0.114739	4.285599	1.82E-05	6.04E-05	HIST1H2A	protein_cc
ENSG0000(	61.96827	-0.60446	0.134561	-4.49205	7.05E-06	2.50E-05	ZFP57	protein_cc
ENSG0000(	61.93719	0.094919	0.054447	1.743325	0.081277	0.128962	RN7SL832	lincRNA
ENSG0000(	61.92884	0.148832	0.087612	1.698764	0.089364	0.140186	BCRP3	pseudoge
ENSG0000(	61.92246	-2.65437	0.286982	-9.24925	2.26E-20	8.18E-19	LINC0100	lincRNA
ENSG0000(	61.85567	-0.27857	0.080939	-3.4417	0.000578	0.001474	CTD-2008	pseudoge

ENSG0000(	61.83745	0.816788	0.160687	5.083112	3.71E-07	1.61E-06	PHLDA2	protein_cc
ENSG0000(	61.79899	-0.47314	0.093629	-5.0533	4.34E-07	1.86E-06	IGSF10	protein_cc
ENSG0000(	61.75908	-0.06044	0.077904	-0.7758	0.43787	0.534855	RP11-420.	lincRNA
ENSG0000(	61.71166	0.041769	0.050909	0.820462	0.411953	0.50885	NEURL2	protein_cc
ENSG0000(	61.69226	0.123024	0.152911	0.804549	0.42108	0.518096	RP11-10B	pseudoge
ENSG0000(	61.66084	-0.61352	0.115967	-5.29048	1.22E-07	5.70E-07	PLCXD2	protein_cc
ENSG0000(	61.60327	0.001185	0.127442	0.009299	0.99258	0.995023	KRT18	protein_cc
ENSG0000(	61.58595	-0.5191	0.117328	-4.4243	9.68E-06	3.35E-05	TSPAN8	protein_cc
ENSG0000(	61.57469	-1.15062	0.148331	-7.75712	8.69E-15	1.28E-13	CDH12	protein_cc
ENSG0000(	61.51344	0.117294	0.046398	2.527993	0.011472	0.022611	TAPT1-AS	antisense
ENSG0000(	61.47421	-0.3925	0.073116	-5.36815	7.95E-08	3.83E-07	LINC0115!	lincRNA
ENSG0000(	61.44769	0.180861	0.057023	3.171716	0.001515	0.003576	RPL23P2	pseudoge
ENSG0000(	61.42791	0.673577	0.071545	9.414794	4.74E-21	1.94E-19	PMS2P5	pseudoge
ENSG0000(	61.4208	0.959282	0.137386	6.98241	2.90E-12	2.81E-11	CTD-2192	protein_cc
ENSG0000(	61.42035	1.081956	0.139569	7.752111	9.04E-15	1.33E-13	PLEK2	protein_cc
ENSG0000(	61.40782	0.382636	0.082082	4.661646	3.14E-06	1.17E-05	CTD-2510	antisense
ENSG0000(	61.40764	-0.25594	0.042963	-5.95723	2.57E-09	1.57E-08	NR1I3	protein_cc
ENSG0000(	61.38085	0.049972	0.15566	0.32103	0.748188	0.810273	IL1R2	protein_cc
ENSG0000(	61.36906	-1.08013	0.120138	-8.99074	2.46E-19	7.53E-18	SYNDIG1L	protein_cc
ENSG0000(	61.35393	0.503157	0.098138	5.127015	2.94E-07	1.29E-06	CACNG1	protein_cc
ENSG0000(	61.33658	0.078501	0.13107	0.598924	0.549224	0.639253	LPO	protein_cc
ENSG0000(	61.29189	-0.52049	0.116985	-4.44921	8.62E-06	3.01E-05	THEM5	protein_cc
ENSG0000(	61.2899	0.235108	0.163233	1.440319	0.149777	0.218497	MAPK15	protein_cc
ENSG0000(	61.28984	-0.38148	0.066982	-5.69524	1.23E-08	6.74E-08	RP11-612	lincRNA
ENSG0000(	61.28027	-1.48838	0.157165	-9.47015	2.79E-21	1.19E-19	RP11-863	lincRNA
ENSG0000(	61.2673	-0.55259	0.133966	-4.12483	3.71E-05	0.000117	TMEM88B	protein_cc
ENSG0000(	61.2335	-0.34053	0.076061	-4.4771	7.57E-06	2.67E-05	RP1-179N	antisense
ENSG0000(	61.19809	-0.08822	0.124908	-0.70628	0.480013	0.575361	IL1A	protein_cc
ENSG0000(	61.187	-0.08512	0.090966	-0.93579	0.349384	0.444832	C16orf54	protein_cc
ENSG0000(	61.17508	-0.08433	0.091588	-0.92075	0.357181	0.452908	DNAH12	protein_cc
ENSG0000(	61.15839	1.175395	0.17664	6.654192	2.85E-11	2.36E-10	RP11-124	antisense
ENSG0000(	61.13734	0.428997	0.070145	6.11582	9.61E-10	6.28E-09	PMFBP1	protein_cc
ENSG0000(	61.09721	0.876481	0.250212	3.502956	0.00046	0.001196	HOXA11	protein_cc
ENSG0000(	61.07987	0.135421	0.080299	1.68646	0.091707	0.1433	LINC0068!	antisense
ENSG0000(	61.07118	-0.92488	0.107416	-8.61029	7.29E-18	1.79E-16	TPBGL	protein_cc
ENSG0000(	61.06168	0.369709	0.055724	6.63466	3.25E-11	2.67E-10	RAD9B	protein_cc
ENSG0000(	60.99958	0.333165	0.068953	4.831766	1.35E-06	5.36E-06	CTB-92J2	sense_intr
ENSG0000(	60.97107	-0.76952	0.128434	-5.99153	2.08E-09	1.29E-08	FABP6	protein_cc
ENSG0000(	60.94069	-0.32705	0.089395	-3.65844	0.000254	0.000691	TRPV4	protein_cc
ENSG0000(	60.88169	-0.68465	0.146401	-4.67655	2.92E-06	1.10E-05	RP11-392	processed
ENSG0000(	60.86768	-1.43963	0.147708	-9.74646	1.91E-22	1.01E-20	EPHA6	protein_cc
ENSG0000(	60.81944	-0.07373	0.057159	-1.28984	0.197105	0.275828	OR7E7P	pseudoge
ENSG0000(	60.81408	0.233647	0.108461	2.154205	0.031224	0.055592	SHISA3	protein_cc
ENSG0000(	60.76717	-0.08787	0.057982	-1.51548	0.129652	0.192951	CTD-2173	pseudoge
ENSG0000(	60.72547	-0.26728	0.076027	-3.5156	0.000439	0.001145	RP11-506	antisense
ENSG0000(	60.7204	-0.65689	0.114774	-5.72335	1.04E-08	5.77E-08	KCNA3	protein_cc
ENSG0000(	60.69981	-0.15924	0.042384	-3.75702	0.000172	0.000482	AC073254	antisense
ENSG0000(	60.69071	0.781911	0.140345	5.57136	2.53E-08	1.32E-07	RP11-363	antisense
ENSG0000(	60.64853	0.122125	0.054725	2.23162	0.02564	0.046586	RP11-260	antisense
ENSG0000(	60.58669	-1.75096	0.226611	-7.72672	1.10E-14	1.59E-13	CTXN3	protein_cc
ENSG0000(	60.5758	0.507374	0.126127	4.02273	5.75E-05	0.000175	BANK1	protein_cc
ENSG0000(	60.53724	-0.03318	0.107739	-0.30797	0.758105	0.818082	MB	protein_cc
ENSG0000(	60.52386	-0.24845	0.120226	-2.06656	0.038776	0.067497	C8orf12	protein_cc
ENSG0000(	60.50102	0.021157	0.074719	0.283158	0.777056	0.833546	RP11-434	lincRNA
ENSG0000(	60.4959	0.055966	0.071197	0.786068	0.431827	0.528841	RDH16	protein_cc
ENSG0000(	60.48977	-0.71832	0.094259	-7.62071	2.52E-14	3.39E-13	RP11-379	antisense
ENSG0000(	60.43788	1.82164	0.315093	5.781278	7.41E-09	4.20E-08	HOXD11	protein_cc
ENSG0000(	60.43485	0.200538	0.044998	4.456591	8.33E-06	2.91E-05	MRPL45P2	pseudoge
ENSG0000(	60.41933	0.144201	0.075294	1.915181	0.055469	0.092541	KRTCAP3	protein_cc

ENSG0000(	60.38114	0.527383	0.150466	3.505	0.000457	0.001187	CCDC42B	protein_cc
ENSG0000(	60.34064	0.101747	0.066368	1.533081	0.125256	0.187446	TVP23C	protein_cc
ENSG0000(	60.2801	-0.05859	0.037558	-1.55985	0.118795	0.179239	RP11-449	lincRNA
ENSG0000(	60.2703	-0.12577	0.067148	-1.87303	0.061064	0.100576	AC005154	pseudoge
ENSG0000(	60.26641	0.446428	0.067568	6.607122	3.92E-11	3.18E-10	RP11-295	antisense
ENSG0000(	60.26368	0.77786	0.148407	5.241397	1.59E-07	7.31E-07	ASB5	protein_cc
ENSG0000(	60.18253	0.21401	0.050714	4.219913	2.44E-05	7.91E-05	MIR621	miRNA
ENSG0000(	60.16646	-0.00362	0.122449	-0.02959	0.976392	0.983387	RP11-527	lincRNA
ENSG0000(	60.14054	0.383072	0.086647	4.421046	9.82E-06	3.39E-05	RP11-611	antisense
ENSG0000(	60.12602	-0.29243	0.053437	-5.47244	4.44E-08	2.23E-07	CTD-2530	antisense
ENSG0000(	60.12395	-0.07351	0.074487	-0.98693	0.323678	0.41787	FAM167B	protein_cc
ENSG0000(	60.10979	0.663293	0.088293	7.51244	5.80E-14	7.35E-13	RP11-170	pseudoge
ENSG0000(	60.10331	-0.15964	0.058973	-2.70704	0.006789	0.014069	GOLGA2P	pseudoge
ENSG0000(	60.08599	0.145849	0.058979	2.472899	0.013402	0.026012	LTB4R2	protein_cc
ENSG0000(	60.06766	0.981985	0.151829	6.467707	9.95E-11	7.58E-10	RP11-416	lincRNA
ENSG0000(	60.06344	0.006213	0.130878	0.047473	0.962136	0.974074	RP11-315	antisense
ENSG0000(	60.06209	-0.04269	0.083803	-0.5094	0.61047	0.694159	CTC-255N	antisense
ENSG0000(	60.0529	-0.13497	0.051341	-2.62889	0.008566	0.017381	DGCR11	sense_intr
ENSG0000(	60.04823	0.295378	0.049422	5.976633	2.28E-09	1.40E-08	CTD-2124	pseudoge
ENSG0000(	60.01393	-0.25318	0.115165	-2.19843	0.027918	0.050268	MEG9	lincRNA
ENSG0000(	60.01206	0.308566	0.111264	2.773288	0.005549	0.011711	STEAP1B	protein_cc
ENSG0000(	59.97331	0.409191	0.100587	4.068022	4.74E-05	0.000146	TNFRSF4	protein_cc
ENSG0000(	59.96822	-0.12623	0.216383	-0.58337	0.559646	0.64858	DLK1	protein_cc
ENSG0000(	59.93288	0.325765	0.149963	2.172305	0.029833	0.05338	CHRDL2	protein_cc
ENSG0000(	59.92172	0.606701	0.090443	6.708098	1.97E-11	1.67E-10	AC092675	protein_cc
ENSG0000(	59.91906	1.11792	0.105833	10.56301	4.42E-26	4.76E-24	RP11-218	lincRNA
ENSG0000(	59.90453	-0.04199	0.074646	-0.56249	0.573779	0.661103	RP11-81A	lincRNA
ENSG0000(	59.89626	0.433598	0.127692	3.395666	0.000685	0.00172	RP11-561	antisense
ENSG0000(	59.83946	0.478964	0.163371	2.931753	0.003371	0.007437	PAX5	protein_cc
ENSG0000(	59.82318	-0.57846	0.12812	-4.51498	6.33E-06	2.26E-05	SOSTDC1	protein_cc
ENSG0000(	59.81767	-1.12877	0.174296	-6.47617	9.41E-11	7.20E-10	EGR4	protein_cc
ENSG0000(	59.81406	2.342632	0.342145	6.846896	7.55E-12	6.84E-11	HOTAIR	antisense
ENSG0000(	59.77793	-0.77795	0.129945	-5.98674	2.14E-09	1.32E-08	CHAD	protein_cc
ENSG0000(	59.74925	0.763023	0.126167	6.047721	1.47E-09	9.31E-09	AL162151	pseudoge
ENSG0000(	59.67637	0.190935	0.052774	3.617943	0.000297	0.0008	AC125232	pseudoge
ENSG0000(	59.62051	-0.32771	0.108725	-3.01411	0.002577	0.005826	LINC0060	lincRNA
ENSG0000(	59.6142	0.114615	0.073111	1.567681	0.116956	0.176859	CTD-2031	antisense
ENSG0000(	59.6089	-0.1648	0.112151	-1.46946	0.141708	0.208277	RP11-159	antisense
ENSG0000(	59.5164	0.208418	0.085375	2.441204	0.014638	0.028186	ANGPTL6	protein_cc
ENSG0000(	59.50693	0.73996	0.095687	7.733092	1.05E-14	1.52E-13	RP11-31F	sense_intr
ENSG0000(	59.50504	-0.35666	0.096886	-3.68126	0.000232	0.000636	CTA-134P	antisense
ENSG0000(	59.49954	-0.53138	0.103601	-5.12914	2.91E-07	1.28E-06	MYO15A	protein_cc
ENSG0000(	59.45431	0.057101	0.08219	0.694748	0.487213	0.581956	CTB-129C	antisense
ENSG0000(	59.42269	0.19089	0.053854	3.544563	0.000393	0.001036	RP11-339	antisense
ENSG0000(	59.39788	-0.19481	0.093226	-2.08967	0.036647	0.064219	OXER1	protein_cc
ENSG0000(	59.35522	-0.09083	0.109593	-0.82879	0.407226	0.504063	AMER2-A	lincRNA
ENSG0000(	59.35246	-0.37809	0.053739	-7.03563	1.98E-12	1.98E-11	RP11-507	antisense
ENSG0000(	59.34397	-1.2293	0.128931	-9.53458	1.51E-21	6.74E-20	RP11-586	lincRNA
ENSG0000(	59.3303	0.204094	0.216003	0.944864	0.344728	0.440062	IGLC2	IG_C_gene
ENSG0000(	59.30681	0.04992	0.138509	0.360414	0.718538	0.785652	AK7	protein_cc
ENSG0000(	59.29897	0.032517	0.067577	0.481183	0.630386	0.71174	RP11-452	antisense
ENSG0000(	59.24385	0.573379	0.134195	4.272714	1.93E-05	6.37E-05	RP13-735	lincRNA
ENSG0000(	59.24317	-0.43778	0.134492	-3.25506	0.001134	0.002735	XIRP1	protein_cc
ENSG0000(	59.05736	0.195342	0.081842	2.386814	0.016995	0.032187	HLA-F-AS	processed
ENSG0000(	59.01857	0.053257	0.05026	1.059627	0.289314	0.380705	PRR4	protein_cc
ENSG0000(	59.01055	0.203617	0.087324	2.331735	0.019715	0.03674	EGFEM1P	pseudoge
ENSG0000(	59.00228	0.380589	0.133499	2.850866	0.00436	0.009404	KMO	protein_cc
ENSG0000(	58.97007	-0.13639	0.06332	-2.15393	0.031246	0.055626	TEX14	protein_cc
ENSG0000(	58.93557	0.071275	0.043206	1.649633	0.099018	0.153222	Z97634.3	pseudoge

ENSG0000(	58.90793	-0.59892	0.082618	-7.24925	4.19E-13	4.61E-12	AF127936	lincRNA
ENSG0000(	58.89239	0.15414	0.048065	3.206909	0.001342	0.003196	CTC-329D	antisense
ENSG0000(	58.87669	-0.08745	0.178085	-0.49104	0.623398	0.705825	RP11-89N	lincRNA
ENSG0000(	58.82969	0.260729	0.100612	2.591438	0.009558	0.019183	LINC0029(	lincRNA
ENSG0000(	58.82042	-0.09276	0.072537	-1.27879	0.200971	0.280446	LINC0093	lincRNA
ENSG0000(	58.79119	-0.06911	0.081548	-0.84743	0.396754	0.493411	GAS6-AS2	lincRNA
ENSG0000(	58.76558	0.549351	0.067344	8.157362	3.42E-16	6.38E-15	RP11-22P	antisense
ENSG0000(	58.7607	0.293133	0.035743	8.201032	2.38E-16	4.57E-15	CTC-325H	antisense
ENSG0000(	58.69294	-0.20981	0.038427	-5.45993	4.76E-08	2.38E-07	ZCWPW2	protein_cc
ENSG0000(	58.68545	0.431198	0.092639	4.654578	3.25E-06	1.21E-05	LRRC66	protein_cc
ENSG0000(	58.56348	-0.02517	0.043707	-0.57584	0.564721	0.652975	CCDC7	protein_cc
ENSG0000(	58.50398	-0.05403	0.112135	-0.48183	0.629927	0.711308	AIRE	protein_cc
ENSG0000(	58.49843	-0.25084	0.074572	-3.36375	0.000769	0.001914	RP11-265	lincRNA
ENSG0000(	58.49138	0.132903	0.112805	1.17816	0.238733	0.324015	CXCR2	protein_cc
ENSG0000(	58.48563	-0.70125	0.099119	-7.07482	1.50E-12	1.51E-11	RP1-18D1	antisense
ENSG0000(	58.46164	-0.04906	0.162288	-0.3023	0.762425	0.821645	TNFSF14	protein_cc
ENSG0000(	58.45759	-0.20633	0.065081	-3.17045	0.001522	0.003591	CTD-2083	antisense
ENSG0000(	58.43899	0.156837	0.090115	1.74041	0.081787	0.129626	AC104655	lincRNA
ENSG0000(	58.41126	0.343844	0.056932	6.039537	1.55E-09	9.77E-09	MIR940	lincRNA
ENSG0000(	58.37743	0.238391	0.068972	3.456362	0.000548	0.001402	SPATA6L	protein_cc
ENSG0000(	58.37505	-0.5895	0.144094	-4.0911	4.29E-05	0.000134	RP11-559	antisense
ENSG0000(	58.36143	0.820381	0.239372	3.427224	0.00061	0.001547	HOXC6	protein_cc
ENSG0000(	58.34964	1.081804	0.120951	8.944117	3.75E-19	1.11E-17	RPS2P32	pseudoge
ENSG0000(	58.34336	-0.26464	0.093687	-2.82475	0.004732	0.010124	EPCAM	protein_cc
ENSG0000(	58.24053	0.328792	0.058212	5.648205	1.62E-08	8.70E-08	AC010761	antisense
ENSG0000(	58.24014	-0.43593	0.094664	-4.60505	4.12E-06	1.51E-05	CTD-2536	lincRNA
ENSG0000(	58.19047	-0.36236	0.059377	-6.10274	1.04E-09	6.79E-09	ARHGEF3:	protein_cc
ENSG0000(	58.17642	0.123678	0.051829	2.386257	0.017021	0.032232	RP3-324C	pseudoge
ENSG0000(	58.16717	-0.16081	0.091936	-1.7492	0.080256	0.127598	TMEM200	protein_cc
ENSG0000(	58.14182	1.177134	0.150932	7.799122	6.23E-15	9.43E-14	RP11-69L	pseudoge
ENSG0000(	58.13268	0.115356	0.042636	2.705602	0.006818	0.014124	ZNF165	protein_cc
ENSG0000(	58.11744	0.536576	0.093631	5.73075	1.00E-08	5.54E-08	CASC14	lincRNA
ENSG0000(	58.09701	-0.46553	0.133638	-3.48347	0.000495	0.001278	POU6F2	protein_cc
ENSG0000(	58.07684	0.273134	0.048226	5.663575	1.48E-08	8.01E-08	TBCAP1	pseudoge
ENSG0000(	58.076	0.17623	0.049106	3.58875	0.000332	0.000887	AC004166	pseudoge
ENSG0000(	58.05652	0.843875	0.281314	2.99976	0.002702	0.006076	HOXB13	protein_cc
ENSG0000(	58.01784	-0.74598	0.148121	-5.03626	4.75E-07	2.02E-06	CCL19	protein_cc
ENSG0000(	58.01327	-0.42764	0.121844	-3.50971	0.000449	0.001169	LINC0032:	antisense
ENSG0000(	58.00668	-0.28619	0.122344	-2.33921	0.019325	0.036095	AP000350	lincRNA
ENSG0000(	57.99864	0.083505	0.049202	1.697196	0.08966	0.140549	GEMIN8P4	pseudoge
ENSG0000(	57.96949	0.618796	0.166067	3.726179	0.000194	0.00054	GAL	protein_cc
ENSG0000(	57.96226	-0.79433	0.133295	-5.95915	2.54E-09	1.55E-08	AC091878	antisense
ENSG0000(	57.95074	0.077373	0.052545	1.472509	0.140884	0.207243	SHBG	protein_cc
ENSG0000(	57.94112	-0.12237	0.110588	-1.10651	0.268508	0.357709	RP11-539	antisense
ENSG0000(	57.93191	-0.58638	0.119924	-4.88965	1.01E-06	4.08E-06	PON1	protein_cc
ENSG0000(	57.92579	-0.79567	0.117504	-6.77146	1.27E-11	1.11E-10	CTC-525D	processed
ENSG0000(	57.92293	0.089397	0.070625	1.26579	0.205588	0.285861	RP11-111	sense_intr
ENSG0000(	57.89433	0.240111	0.047446	5.060731	4.18E-07	1.79E-06	RP11-863	protein_cc
ENSG0000(	57.89178	0.393063	0.145455	2.702298	0.006886	0.014251	RP11-290	lincRNA
ENSG0000(	57.74851	-0.40983	0.159527	-2.56906	0.010198	0.020325	AC009014	lincRNA
ENSG0000(	57.74598	0.958987	0.118832	8.070118	7.02E-16	1.24E-14	RPS2P7	pseudoge
ENSG0000(	57.72194	-2.08451	0.188772	-11.0425	2.38E-28	3.98E-26	CNGB1	protein_cc
ENSG0000(	57.70304	-0.22052	0.102909	-2.14287	0.032124	0.057036	GPR45	protein_cc
ENSG0000(	57.70018	-0.90424	0.16237	-5.56901	2.56E-08	1.33E-07	LINC0069	lincRNA
ENSG0000(	57.62292	-0.11011	0.066472	-1.65647	0.097627	0.151286	GHRL	protein_cc
ENSG0000(	57.60827	-0.0653	0.047806	-1.36595	0.171954	0.245682	WDR88	protein_cc
ENSG0000(	57.51382	-0.04948	0.063079	-0.78442	0.432795	0.529774	CTD-2132	sense_intr
ENSG0000(	57.4987	-0.03213	0.055677	-0.57716	0.563833	0.652201	RP11-179	antisense
ENSG0000(	57.49343	0.067681	0.127714	0.529946	0.59615	0.681399	MTATP8P	pseudoge

ENSG0000(	57.39396	-0.46118	0.070532	-6.53867	6.21E-11	4.88E-10	MSANTD1	protein_cc
ENSG0000(	57.31797	-0.2851	0.093604	-3.04576	0.002321	0.005291	LRRC37A7	pseudoge
ENSG0000(	57.31071	-0.47181	0.084234	-5.60119	2.13E-08	1.12E-07	MIR4442	miRNA
ENSG0000(	57.283	0.275068	0.081242	3.385789	0.00071	0.001778	RP11-966	antisense
ENSG0000(	57.27645	0.068021	0.044248	1.53729	0.124222	0.186136	CCNT2-AS	antisense
ENSG0000(	57.26761	-0.22704	0.108829	-2.08617	0.036963	0.06469	RP11-73C	antisense
ENSG0000(	57.21882	-0.18047	0.090191	-2.00101	0.045391	0.077552	CLDN9	protein_cc
ENSG0000(	57.18291	-0.19426	0.079754	-2.4357	0.014863	0.028554	MID1IP1-/	antisense
ENSG0000(	57.18149	0.03787	0.065084	0.581863	0.560659	0.649574	ROCK1P1	pseudoge
ENSG0000(	57.16526	-0.54992	0.162111	-3.39223	0.000693	0.00174	CCL8	protein_cc
ENSG0000(	57.15626	-0.35145	0.055628	-6.3178	2.65E-10	1.89E-09	GP1BA	protein_cc
ENSG0000(	57.14249	0.114804	0.074955	1.531631	0.125614	0.187918	TNKS2-AS	antisense
ENSG0000(	57.13406	0.493158	0.075121	6.564823	5.21E-11	4.14E-10	AC017104	processed
ENSG0000(	57.09324	0.007849	0.06359	0.123432	0.901765	0.930268	FBXO24	protein_cc
ENSG0000(	57.08509	-1.12223	0.134291	-8.35673	6.45E-17	1.35E-15	RP11-13K	lincRNA
ENSG0000(	57.07951	0.012113	0.085795	0.141183	0.887726	0.920074	MIR4477A	miRNA
ENSG0000(	57.07399	0.122688	0.040791	3.007763	0.002632	0.005936	RP11-286	protein_cc
ENSG0000(	57.03909	-0.05353	0.0528	-1.01388	0.310642	0.403884	MEF2BNB	protein_cc
ENSG0000(	56.99239	1.318022	0.140551	9.377523	6.75E-21	2.66E-19	ARL9	protein_cc
ENSG0000(	56.91234	0.085367	0.06844	1.247326	0.212278	0.293643	BGLAP	protein_cc
ENSG0000(	56.91157	-1.10026	0.163771	-6.71831	1.84E-11	1.57E-10	FAM153B	protein_cc
ENSG0000(	56.89942	-0.07142	0.046722	-1.52872	0.126333	0.188866	FUT2	protein_cc
ENSG0000(	56.89227	0.22856	0.190078	1.202453	0.229188	0.313086	PLEKHS1	protein_cc
ENSG0000(	56.88033	-0.43739	0.071082	-6.15332	7.59E-10	5.05E-09	BAIAP2L1	protein_cc
ENSG0000(	56.81448	-0.47878	0.158306	-3.02441	0.002491	0.005649	NYAP2	protein_cc
ENSG0000(	56.80941	-0.06191	0.110302	-0.56124	0.574637	0.66192	HCG23	antisense
ENSG0000(	56.80895	-0.06733	0.073659	-0.91407	0.360679	0.456546	RP11-271	pseudoge
ENSG0000(	56.80122	0.308106	0.067504	4.564246	5.01E-06	1.82E-05	RHPN1-AS	antisense
ENSG0000(	56.78834	-0.3036	0.08477	-3.58145	0.000342	0.00091	RP3-325F:	antisense
ENSG0000(	56.75293	-0.25397	0.116406	-2.18172	0.02913	0.052266	SGCG	protein_cc
ENSG0000(	56.6524	0.048417	0.105818	0.457556	0.647272	0.726326	AF186192	pseudoge
ENSG0000(	56.63557	0.961105	0.113129	8.495684	1.97E-17	4.49E-16	AC079250	pseudoge
ENSG0000(	56.62359	0.029131	0.12248	0.237844	0.812002	0.86186	FAM83G	protein_cc
ENSG0000(	56.61899	0.834116	0.096062	8.683116	3.85E-18	9.82E-17	IL7	protein_cc
ENSG0000(	56.60733	0.216256	0.069671	3.103964	0.001909	0.004421	AC084219	antisense
ENSG0000(	56.57575	-1.34755	0.160117	-8.41604	3.89E-17	8.48E-16	LINGO2	protein_cc
ENSG0000(	56.57089	0.282478	0.119044	2.372896	0.017649	0.033259	FAM225A	lincRNA
ENSG0000(	56.57014	0.210302	0.059563	3.530745	0.000414	0.001087	HCG27	protein_cc
ENSG0000(	56.49274	0.256785	0.111869	2.295404	0.02171	0.040095	CLDN23	protein_cc
ENSG0000(	56.48611	0.615002	0.123855	4.965496	6.85E-07	2.84E-06	NKX6-1	protein_cc
ENSG0000(	56.44613	-0.73128	0.12662	-5.77539	7.68E-09	4.34E-08	PCDHA5	protein_cc
ENSG0000(	56.42249	-0.29442	0.117741	-2.50061	0.012398	0.024239	IKZF3	protein_cc
ENSG0000(	56.39761	0.367104	0.160186	2.291735	0.021921	0.040456	HS3ST3A1	protein_cc
ENSG0000(	56.36062	0.136032	0.063834	2.131022	0.033087	0.058579	AKR7L	polymorpl
ENSG0000(	56.34504	0.114993	0.032831	3.502572	0.000461	0.001197	RBM14-RI	protein_cc
ENSG0000(	56.33758	0.116779	0.06846	1.705815	0.088042	0.138394	RP11-54C	pseudoge
ENSG0000(	56.3215	-0.03378	0.091272	-0.37008	0.711322	0.779836	PTPRVP	pseudoge
ENSG0000(	56.30741	0.594659	0.088844	6.693272	2.18E-11	1.84E-10	CCDC153	protein_cc
ENSG0000(	56.26088	-0.66276	0.080827	-8.19972	2.41E-16	4.61E-15	RP4-575N	antisense
ENSG0000(	56.2447	-0.02224	0.079026	-0.28142	0.778391	0.834572	RP11-44N	lincRNA
ENSG0000(	56.23453	-0.07521	0.064656	-1.16319	0.244751	0.330964	AP000347	pseudoge
ENSG0000(	56.20949	-0.13891	0.088098	-1.57682	0.114837	0.174116	RP11-211	lincRNA
ENSG0000(	56.17061	0.119907	0.08672	1.382692	0.166759	0.239415	CD247	protein_cc
ENSG0000(	56.14667	-0.31224	0.12097	-2.58114	0.009848	0.019703	AL078585	protein_cc
ENSG0000(	56.14643	-1.43352	0.16665	-8.60197	7.84E-18	1.91E-16	KCNB2	protein_cc
ENSG0000(	56.12814	0.104847	0.087475	1.198596	0.230685	0.314702	CTD-3088	protein_cc
ENSG0000(	56.08234	-0.58001	0.091369	-6.348	2.18E-10	1.57E-09	BFSP1	protein_cc
ENSG0000(	56.08182	0.318989	0.097783	3.262197	0.001106	0.002672	FSIP2	protein_cc
ENSG0000(	56.0578	0.843374	0.091548	9.212413	3.19E-20	1.12E-18	RPS3AP47	pseudoge

ENSG0000(	56.05137	-0.24433	0.085684	-2.85151	0.004351	0.009388	AC133785	antisense
ENSG0000(	56.04642	-0.82788	0.114867	-7.20731	5.71E-13	6.15E-12	CDR1	protein_cc
ENSG0000(	56.01744	0.126855	0.113223	1.120399	0.262544	0.351117	RP11-588(	antisense
ENSG0000(	56.00969	0.240653	0.062302	3.862683	0.000112	0.000325	RP5-890E.	processed
ENSG0000(	55.94231	1.00585	0.120135	8.372659	5.63E-17	1.20E-15	RPS7P10	pseudoge
ENSG0000(	55.93608	0.007919	0.076508	0.103511	0.917557	0.94193	LINC0092(	lincRNA
ENSG0000(	55.93548	0.108171	0.094704	1.142195	0.253373	0.340792	CTC-459F	lincRNA
ENSG0000(	55.91766	0.794872	0.232773	3.414791	0.000638	0.001614	APCDD1L	protein_cc
ENSG0000(	55.9131	0.357319	0.052077	6.861372	6.82E-12	6.21E-11	HNRNPCP	pseudoge
ENSG0000(	55.91029	0.041664	0.068902	0.604683	0.545389	0.635875	ZNF876P	pseudoge
ENSG0000(	55.90091	-0.34272	0.115561	-2.96569	0.00302	0.006724	COL24A1	protein_cc
ENSG0000(	55.79033	-0.09093	0.134252	-0.67731	0.498211	0.592168	VIT	protein_cc
ENSG0000(	55.77149	0.079901	0.132507	0.602991	0.546514	0.636934	VSX1	protein_cc
ENSG0000(	55.77028	0.089462	0.040615	2.202663	0.027619	0.049787	CTD-2013	antisense
ENSG0000(	55.72711	-0.11894	0.050119	-2.37325	0.017632	0.033228	RP11-250	lincRNA
ENSG0000(	55.69662	1.773145	0.218035	8.132377	4.21E-16	7.75E-15	FAM183A	protein_cc
ENSG0000(	55.68705	-0.22595	0.073687	-3.06628	0.002167	0.004968	BMP8A	protein_cc
ENSG0000(	55.66291	0.416725	0.088926	4.686186	2.78E-06	1.05E-05	SYCE1L	protein_cc
ENSG0000(	55.61493	-0.33089	0.09227	-3.58605	0.000336	0.000896	GHRHR	protein_cc
ENSG0000(	55.6061	-1.71776	0.144065	-11.9235	8.93E-33	3.50E-30	CCBE1	protein_cc
ENSG0000(	55.60593	0.056163	0.062546	0.897937	0.369219	0.465531	URAHP	pseudoge
ENSG0000(	55.57802	0.138624	0.050872	2.724946	0.006431	0.013394	CTD-2538	lincRNA
ENSG0000(	55.57409	-0.07552	0.040608	-1.85975	0.062921	0.103266	LSM12P1	pseudoge
ENSG0000(	55.56941	-0.05185	0.087964	-0.58946	0.555555	0.645021	SOWAHD	protein_cc
ENSG0000(	55.54816	0.259935	0.047293	5.496299	3.88E-08	1.96E-07	RP11-345.	pseudoge
ENSG0000(	55.53959	0.256013	0.072768	3.518187	0.000435	0.001135	CTD-2561	processed
ENSG0000(	55.50556	0.462273	0.166028	2.784305	0.005364	0.011364	BPIFB2	protein_cc
ENSG0000(	55.48464	0.297121	0.051191	5.80421	6.47E-09	3.70E-08	CCT8P1	pseudoge
ENSG0000(	55.46309	-0.08495	0.08726	-0.97357	0.330269	0.424623	FAM66B	lincRNA
ENSG0000(	55.45785	0.284825	0.106521	2.67388	0.007498	0.015409	ELOVL2-A	antisense
ENSG0000(	55.39808	-0.36062	0.069606	-5.18088	2.21E-07	9.90E-07	RP11-503	antisense
ENSG0000(	55.39763	-0.0289	0.048333	-0.59787	0.549928	0.639901	RP11-73M	antisense
ENSG0000(	55.33363	0.468576	0.0629	7.449532	9.37E-14	1.15E-12	RP11-106	antisense
ENSG0000(	55.33311	1.043632	0.166709	6.260202	3.84E-10	2.67E-09	HOXD-AS	antisense
ENSG0000(	55.32416	0.803573	0.115207	6.975049	3.06E-12	2.95E-11	RP11-169	pseudoge
ENSG0000(	55.31234	-0.11435	0.051635	-2.21466	0.026783	0.048464	RP11-296	pseudoge
ENSG0000(	55.30379	0.098442	0.077468	1.270747	0.203819	0.283792	SEMA3B-/	antisense
ENSG0000(	55.30342	1.744344	0.152691	11.42405	3.17E-30	7.54E-28	LINC0066!	lincRNA
ENSG0000(	55.29397	1.015876	0.137035	7.413273	1.23E-13	1.48E-12	RP11-761	pseudoge
ENSG0000(	55.28337	0.173736	0.097708	1.778116	0.075385	0.120877	PCDHB18	pseudoge
ENSG0000(	55.23385	-0.10023	0.069468	-1.44289	0.14905	0.21763	RP11-156	protein_cc
ENSG0000(	55.17331	0.678032	0.09554	7.096845	1.28E-12	1.31E-11	RPL7AP30	pseudoge
ENSG0000(	55.13676	0.504821	0.065731	7.68007	1.59E-14	2.22E-13	RP11-168.	pseudoge
ENSG0000(	55.09967	0.766003	0.110928	6.905434	5.01E-12	4.65E-11	AC147651	antisense
ENSG0000(	55.08984	0.177437	0.089699	1.978148	0.047912	0.081321	FAM162B	protein_cc
ENSG0000(	55.04797	-0.00092	0.141496	-0.00652	0.994797	0.996669	WFIKKN2	protein_cc
ENSG0000(	55.00536	0.101267	0.080475	1.258372	0.208257	0.288976	FAM166B	protein_cc
ENSG0000(	54.979	-0.47297	0.127407	-3.7123	0.000205	0.000568	CCDC144/	protein_cc
ENSG0000(	54.9406	-1.02837	0.12653	-8.12752	4.38E-16	8.04E-15	PTH2R	protein_cc
ENSG0000(	54.93166	0.469722	0.096079	4.88894	1.01E-06	4.09E-06	PABPC3	protein_cc
ENSG0000(	54.8847	1.362164	0.139612	9.756769	1.73E-22	9.13E-21	RP11-270(	pseudoge
ENSG0000(	54.87931	1.063088	0.123916	8.579071	9.56E-18	2.30E-16	FAUP1	pseudoge
ENSG0000(	54.87647	-0.58786	0.079019	-7.43946	1.01E-13	1.23E-12	TMEM72-.	antisense
ENSG0000(	54.85927	0.3767	0.087346	4.312716	1.61E-05	5.38E-05	EIF4BP3	pseudoge
ENSG0000(	54.8391	0.20367	0.058604	3.475344	0.00051	0.001314	AC004985	pseudoge
ENSG0000(	54.82872	-0.46643	0.101351	-4.60207	4.18E-06	1.53E-05	CTD-2049	antisense
ENSG0000(	54.82663	0.172136	0.051691	3.330096	0.000868	0.002141	USP3-AS1	antisense
ENSG0000(	54.79613	-0.16116	0.12513	-1.28795	0.197762	0.276596	PCDHA7	protein_cc
ENSG0000(	54.78061	-0.24374	0.10224	-2.38404	0.017124	0.032395	VGLL3	protein_cc

ENSG0000(	54.77156	-0.58275	0.146959	-3.96542	7.33E-05	0.000219	VAX1	protein_cc
ENSG0000(	54.73336	0.240632	0.187605	1.282647	0.199616	0.278829	OSR2	protein_cc
ENSG0000(	54.72932	-0.15794	0.103022	-1.5331	0.125252	0.187446	AC140912	lincRNA
ENSG0000(	54.71713	0.202489	0.054583	3.709737	0.000207	0.000573	RP11-301(	antisense
ENSG0000(	54.71638	0.795007	0.103024	7.716697	1.19E-14	1.71E-13	LRGUK	protein_cc
ENSG0000(	54.7097	-0.90815	0.122735	-7.39922	1.37E-13	1.63E-12	NT5C1A	protein_cc
ENSG0000(	54.70785	0.45809	0.101358	4.519506	6.20E-06	2.21E-05	C9orf47	protein_cc
ENSG0000(	54.64077	-0.21162	0.049588	-4.2675	1.98E-05	6.51E-05	GPR113	protein_cc
ENSG0000(	54.62581	0.061335	0.05682	1.079465	0.28038	0.37093	ZNF157	protein_cc
ENSG0000(	54.56041	-0.22602	0.150474	-1.50202	0.133093	0.197319	NTSR1	protein_cc
ENSG0000(	54.53171	0.059912	0.067114	0.892691	0.372023	0.468463	RP3-395M	processed
ENSG0000(	54.48474	-0.00148	0.122888	-0.01201	0.990419	0.993487	ALX4	protein_cc
ENSG0000(	54.41893	0.158204	0.053568	2.953326	0.003144	0.006978	RP1-43E1:	antisense
ENSG0000(	54.40704	-1.35042	0.13982	-9.65826	4.54E-22	2.21E-20	CHRM2	protein_cc
ENSG0000(	54.37204	0.023838	0.051172	0.465835	0.641334	0.721218	RP11-305	antisense
ENSG0000(	54.34449	0.327525	0.126156	2.596192	0.009426	0.018936	AC008060	protein_cc
ENSG0000(	54.34005	0.041481	0.05691	0.728888	0.46607	0.561891	LINC0063{	lincRNA
ENSG0000(	54.31408	-0.18975	0.049608	-3.82503	0.000131	0.000374	BTF3L4P2	pseudoge
ENSG0000(	54.3001	0.093215	0.098261	0.94865	0.342799	0.43798	ALB	protein_cc
ENSG0000(	54.28681	0.513502	0.233356	2.200513	0.027771	0.050043	HMGA2	protein_cc
ENSG0000(	54.2614	-0.01428	0.047036	-0.30369	0.761361	0.820735	LINC0042:	lincRNA
ENSG0000(	54.2152	-0.17637	0.062995	-2.79979	0.005114	0.010879	DLG5-AS1	antisense
ENSG0000(	54.19521	0.06523	0.054262	1.202116	0.229318	0.313212	NOXRED1	protein_cc
ENSG0000(	54.1611	0.000414	0.065876	0.006279	0.994991	0.996808	ZMIZ1-AS	antisense
ENSG0000(	54.13739	-0.25358	0.061404	-4.1297	3.63E-05	0.000114	RHOF	protein_cc
ENSG0000(	54.0897	-0.29264	0.077248	-3.78836	0.000152	0.000429	DAZAP2P:	pseudoge
ENSG0000(	54.05481	0.940326	0.136553	6.886165	5.73E-12	5.28E-11	ALDH3A1	protein_cc
ENSG0000(	54.04909	0.319995	0.086678	3.69177	0.000223	0.000612	RP11-230	antisense
ENSG0000(	54.04766	0.286443	0.06548	4.374509	1.22E-05	4.14E-05	RP11-157	antisense
ENSG0000(	54.04325	-0.15464	0.064296	-2.40512	0.016167	0.030815	RP3-402G	antisense
ENSG0000(	54.0136	0.010925	0.080521	0.135681	0.892073	0.923211	LTB	protein_cc
ENSG0000(	54.00355	0.730434	0.117331	6.225407	4.80E-10	3.28E-09	SC22CB-1	pseudoge
ENSG0000(	53.98597	0.012897	0.130014	0.099193	0.920985	0.944433	FABP4	protein_cc
ENSG0000(	53.97931	0.190647	0.054014	3.529587	0.000416	0.001091	RP11-304	antisense
ENSG0000(	53.95815	0.161691	0.068743	2.352129	0.018666	0.034993	RP11-479	lincRNA
ENSG0000(	53.94309	0.112497	0.146348	0.768692	0.442076	0.538889	MEDAG	protein_cc
ENSG0000(	53.93777	0.004242	0.071605	0.059236	0.952764	0.967325	TLDC2	protein_cc
ENSG0000(	53.92701	-0.59837	0.078284	-7.64356	2.11E-14	2.89E-13	RP11-113	pseudoge
ENSG0000(	53.8993	0.317086	0.090554	3.501643	0.000462	0.001201	AC098614	pseudoge
ENSG0000(	53.89853	-0.0296	0.071178	-0.41584	0.67753	0.751727	HNRNPUL	protein_cc
ENSG0000(	53.83572	-0.01012	0.076593	-0.13207	0.894933	0.925332	SIGLEC16	pseudoge
ENSG0000(	53.75292	0.022112	0.047793	0.462652	0.643614	0.723339	RP4-584D	antisense
ENSG0000(	53.75026	-0.10893	0.067336	-1.61776	0.105715	0.162039	RP11-707	antisense
ENSG0000(	53.71873	-0.14299	0.087244	-1.63896	0.101221	0.15614	LINC0087	lincRNA
ENSG0000(	53.70916	0.250174	0.072128	3.468453	0.000523	0.001346	AC004463	antisense
ENSG0000(	53.68492	0.183298	0.088188	2.078496	0.037664	0.06579	HPGD	protein_cc
ENSG0000(	53.64897	-0.2621	0.103904	-2.52251	0.011652	0.022933	MIR142	antisense
ENSG0000(	53.60924	-1.10244	0.148102	-7.44376	9.79E-14	1.19E-12	MT-TF	Mt_tRNA
ENSG0000(	53.60862	0.204096	0.090964	2.243698	0.024852	0.045292	CLDN7	protein_cc
ENSG0000(	53.59844	-0.09308	0.12499	-0.7447	0.456453	0.552907	KLRK1	protein_cc
ENSG0000(	53.59825	-0.02744	0.049226	-0.55742	0.577241	0.66444	RAB43	protein_cc
ENSG0000(	53.59088	0.30654	0.073535	4.168635	3.06E-05	9.77E-05	AC018766	antisense
ENSG0000(	53.58428	0.168156	0.142748	1.177994	0.238799	0.324073	LOXL1-AS	antisense
ENSG0000(	53.55676	0.642465	0.105494	6.090088	1.13E-09	7.30E-09	RP3-417G	pseudoge
ENSG0000(	53.55557	-0.34017	0.069781	-4.87479	1.09E-06	4.37E-06	RGPD8	protein_cc
ENSG0000(	53.55546	0.145967	0.105426	1.384554	0.166189	0.238746	RP11-465	pseudoge
ENSG0000(	53.54862	1.00818	0.13157	7.6627	1.82E-14	2.52E-13	GAPDHP6	pseudoge
ENSG0000(	53.4909	0.559448	0.091813	6.093319	1.11E-09	7.17E-09	RPS20P14	pseudoge
ENSG0000(	53.47895	0.364957	0.085867	4.250267	2.14E-05	6.99E-05	EPHA1	protein_cc

ENSG0000(	53.47342	-0.40016	0.178828	-2.23767	0.025243	0.045933	SYCE1	protein_cc
ENSG0000(	53.29997	0.656522	0.143872	4.563228	5.04E-06	1.82E-05	EXOC3L2	protein_cc
ENSG0000(	53.27394	0.47936	0.06399	7.491146	6.83E-14	8.57E-13	RP11-443	antisense
ENSG0000(	53.18725	0.266986	0.174901	1.526499	0.126886	0.18959	RNU4-2	snRNA
ENSG0000(	53.18111	0.873628	0.152794	5.717688	1.08E-08	5.96E-08	TRIM29	protein_cc
ENSG0000(	53.16582	-0.56467	0.105471	-5.35383	8.61E-08	4.12E-07	IL18R1	protein_cc
ENSG0000(	53.14896	0.393484	0.1185	3.320541	0.000898	0.002208	FOXN4	protein_cc
ENSG0000(	53.12643	0.293543	0.043263	6.785105	1.16E-11	1.02E-10	LIPT2	protein_cc
ENSG0000(	53.09482	-0.08098	0.067638	-1.19723	0.231217	0.315281	ADM5	protein_cc
ENSG0000(	53.08878	0.505751	0.065914	7.672858	1.68E-14	2.33E-13	GRM6	protein_cc
ENSG0000(	53.05429	-0.09883	0.069937	-1.41309	0.15763	0.22814	RP11-68I1	sense_ove
ENSG0000(	53.0134	0.034342	0.070243	0.488902	0.624911	0.707002	LA16c-OS	lincRNA
ENSG0000(	53.01333	0.283246	0.05374	5.270622	1.36E-07	6.30E-07	ZNF66	protein_cc
ENSG0000(	53.00369	0.275781	0.065572	4.205781	2.60E-05	8.39E-05	RP4-761J1	lincRNA
ENSG0000(	52.98323	-0.12022	0.134663	-0.89276	0.371987	0.468433	ACPP	protein_cc
ENSG0000(	52.98153	0.401534	0.043995	9.126854	7.05E-20	2.36E-18	C9orf147	protein_cc
ENSG0000(	52.97179	0.140041	0.08297	1.687852	0.09144	0.142952	RP11-12A	lincRNA
ENSG0000(	52.97155	-0.49848	0.086959	-5.73242	9.90E-09	5.49E-08	RP11-154	antisense
ENSG0000(	52.9434	-0.21102	0.059318	-3.55751	0.000374	0.00099	SENCR	antisense
ENSG0000(	52.93412	0.108903	0.091824	1.185995	0.235624	0.320398	KCP	processed
ENSG0000(	52.87162	0.803706	0.101104	7.949291	1.88E-15	3.10E-14	PPP1R36	protein_cc
ENSG0000(	52.86724	-0.23225	0.077993	-2.97784	0.002903	0.006489	SNORD11	lincRNA
ENSG0000(	52.85171	-0.01589	0.117122	-0.1357	0.892059	0.923211	RP11-867	processed
ENSG0000(	52.84324	0.129978	0.095497	1.36106	0.173495	0.24753	SGCA	protein_cc
ENSG0000(	52.78427	-0.02461	0.131861	-0.18663	0.851954	0.892927	WDR65	protein_cc
ENSG0000(	52.71201	0.629018	0.080336	7.829795	4.89E-15	7.52E-14	RPL24P2	pseudoge
ENSG0000(	52.71068	-0.2964	0.117627	-2.5198	0.011742	0.02309	AGPAT9	protein_cc
ENSG0000(	52.70487	0.181493	0.046686	3.88757	0.000101	0.000296	RP11-817	lincRNA
ENSG0000(	52.66847	-0.27624	0.144547	-1.91105	0.055998	0.09328	MFAP5	protein_cc
ENSG0000(	52.66215	-0.76078	0.134129	-5.67196	1.41E-08	7.65E-08	PTPN20A	protein_cc
ENSG0000(	52.659	0.585697	0.077776	7.530591	5.05E-14	6.48E-13	CTC-360G	lincRNA
ENSG0000(	52.64538	-0.36142	0.124333	-2.90683	0.003651	0.008001	CTD-3065	antisense
ENSG0000(	52.62844	0.391132	0.15925	2.456084	0.014046	0.027139	ALPK2	protein_cc
ENSG0000(	52.62749	0.09681	0.095813	1.010402	0.312303	0.405614	LIPC	protein_cc
ENSG0000(	52.62636	0.200856	0.063855	3.145486	0.001658	0.003884	LRP4-AS1	antisense
ENSG0000(	52.61744	0.160387	0.099132	1.617921	0.10568	0.162	NCF1B	pseudoge
ENSG0000(	52.58999	0.6137	0.09319	6.585453	4.54E-11	3.64E-10	AC010226	antisense
ENSG0000(	52.5834	-0.22387	0.070586	-3.1716	0.001516	0.003577	AC092835	lincRNA
ENSG0000(	52.55798	0.189967	0.097303	1.95233	0.050899	0.085853	HIST1H2B	protein_cc
ENSG0000(	52.54481	-0.23283	0.079641	-2.92347	0.003462	0.007621	NTRK1	protein_cc
ENSG0000(	52.54443	0.251112	0.053087	4.730232	2.24E-06	8.58E-06	GS1-124K	pseudoge
ENSG0000(	52.53166	0.196128	0.064597	3.036187	0.002396	0.005451	LINC0011!	lincRNA
ENSG0000(	52.42674	-0.03964	0.062622	-0.63296	0.526759	0.618574	RP1-151F	antisense
ENSG0000(	52.39837	-0.13903	0.045167	-3.07819	0.002083	0.004791	RPGRIP1	protein_cc
ENSG0000(	52.39256	-0.0044	0.046004	-0.09572	0.923741	0.946404	RASAL2-A	lincRNA
ENSG0000(	52.38214	0.764806	0.102987	7.426216	1.12E-13	1.35E-12	FKBP1C	protein_cc
ENSG0000(	52.3774	0.166698	0.042607	3.912493	9.13E-05	0.000269	RP11-35G	antisense
ENSG0000(	52.3771	0.352127	0.054765	6.42976	1.28E-10	9.59E-10	MED14-A!	antisense
ENSG0000(	52.36855	0.935374	0.123531	7.57196	3.68E-14	4.81E-13	TPT1P4	pseudoge
ENSG0000(	52.36822	0.371287	0.040905	9.076717	1.12E-19	3.63E-18	ZNF709	protein_cc
ENSG0000(	52.36729	0.208258	0.088972	2.340719	0.019247	0.035967	ADAMTS9	antisense
ENSG0000(	52.36474	-0.36539	0.099785	-3.66172	0.000251	0.000683	RP11-338	lincRNA
ENSG0000(	52.30548	0.269271	0.078209	3.44299	0.000575	0.001468	TTC23L	protein_cc
ENSG0000(	52.28399	-0.13203	0.056063	-2.35496	0.018525	0.034756	DBIL5P	pseudoge
ENSG0000(	52.27482	0.931424	0.113775	8.186525	2.69E-16	5.10E-15	RP5-827C	pseudoge
ENSG0000(	52.24742	0.036403	0.053745	0.677316	0.498206	0.592168	RP11-30K	lincRNA
ENSG0000(	52.2459	-0.09568	0.086027	-1.11224	0.266034	0.354919	RP11-475	antisense
ENSG0000(	52.1844	0.630182	0.076671	8.219323	2.05E-16	3.95E-15	HPX	protein_cc
ENSG0000(	52.17782	-0.64329	0.084296	-7.63131	2.32E-14	3.14E-13	SLC5A12	protein_cc

ENSG0000	52.17227	-0.46044	0.116205	-3.96233	7.42E-05	0.000222	GUSBP5	pseudoge
ENSG0000	52.15245	-0.26799	0.160719	-1.66748	0.09542	0.148363	FPR2	protein_cc
ENSG0000	52.10692	-0.37137	0.082099	-4.52342	6.08E-06	2.18E-05	RP11-733	lincRNA
ENSG0000	52.08064	-0.28171	0.078967	-3.56737	0.000361	0.000956	RP11-315	antisense
ENSG0000	52.03725	0.093628	0.05416	1.728723	0.083859	0.132532	AC010894	lincRNA
ENSG0000	52.0191	-0.04718	0.065091	-0.72476	0.468602	0.564486	BSN-AS2	lincRNA
ENSG0000	52.01037	1.69807	0.344889	4.923533	8.50E-07	3.47E-06	HOXC13	protein_cc
ENSG0000	51.9403	0.118502	0.057696	2.053904	0.039985	0.069368	SPATA9	protein_cc
ENSG0000	51.8998	0.376228	0.082813	4.543109	5.54E-06	1.99E-05	SAPCD1	protein_cc
ENSG0000	51.889	0.151832	0.140566	1.080148	0.280076	0.370587	SFN	protein_cc
ENSG0000	51.81943	-0.0764	0.063989	-1.19389	0.232523	0.316767	RP11-6J2	lincRNA
ENSG0000	51.7874	-0.09762	0.08519	-1.14593	0.251826	0.33906	SRGAP2D	protein_cc
ENSG0000	51.73836	-0.06854	0.053033	-1.29233	0.196241	0.274815	RP11-432	antisense
ENSG0000	51.72886	-0.02453	0.057432	-0.42713	0.669287	0.74467	FOXP3	protein_cc
ENSG0000	51.72802	0.229467	0.059252	3.872693	0.000108	0.000313	PRORSD1	pseudoge
ENSG0000	51.70502	0.395747	0.054714	7.232991	4.72E-13	5.15E-12	RWDD4P2	pseudoge
ENSG0000	51.67455	0.493224	0.114136	4.321355	1.55E-05	5.19E-05	RP11-222	lincRNA
ENSG0000	51.66818	0.169112	0.070342	2.404147	0.01621	0.030883	GNRH1	protein_cc
ENSG0000	51.64556	-0.31569	0.080179	-3.93736	8.24E-05	0.000245	RP11-446	pseudoge
ENSG0000	51.60353	0.694122	0.103588	6.700772	2.07E-11	1.75E-10	EEF1A1P8	pseudoge
ENSG0000	51.60265	0.379545	0.072597	5.228122	1.71E-07	7.82E-07	RP11-127	lincRNA
ENSG0000	51.5964	0.185216	0.071477	2.591261	0.009562	0.019192	RPL21P28	pseudoge
ENSG0000	51.59142	0.694012	0.096908	7.161537	7.98E-13	8.42E-12	SOHLH2	protein_cc
ENSG0000	51.58972	0.047703	0.05647	0.844757	0.398247	0.494848	RP1-257A	antisense
ENSG0000	51.58188	0.464615	0.102807	4.519311	6.20E-06	2.21E-05	APOBEC3	protein_cc
ENSG0000	51.55339	-0.30421	0.055909	-5.44112	5.29E-08	2.62E-07	CTB-32O	lincRNA
ENSG0000	51.53023	-1.24034	0.119098	-10.4145	2.13E-25	1.99E-23	KRTAP5-A	antisense
ENSG0000	51.52943	0.027119	0.096698	0.280447	0.779135	0.835166	CDC42EP	protein_cc
ENSG0000	51.52218	0.177581	0.117791	1.507596	0.131658	0.195532	LAMP3	protein_cc
ENSG0000	51.51858	-0.85339	0.156542	-5.45149	4.99E-08	2.48E-07	PCDHA9	protein_cc
ENSG0000	51.51441	0.455695	0.098612	4.621073	3.82E-06	1.41E-05	RP11-108	pseudoge
ENSG0000	51.49923	0.265192	0.049478	5.359768	8.33E-08	3.99E-07	AC093627	lincRNA
ENSG0000	51.47336	0.026054	0.042114	0.618649	0.536148	0.627294	NDUFB2-A	antisense
ENSG0000	51.46401	-0.26045	0.080966	-3.21672	0.001297	0.003096	KL	protein_cc
ENSG0000	51.45721	-0.31698	0.075119	-4.21968	2.45E-05	7.92E-05	AC118758	protein_cc
ENSG0000	51.43042	-0.56323	0.075607	-7.44934	9.38E-14	1.15E-12	ST8SIA6	protein_cc
ENSG0000	51.3803	0.416422	0.075866	5.488904	4.04E-08	2.04E-07	RP11-148	antisense
ENSG0000	51.37829	-0.22109	0.101102	-2.18685	0.028754	0.051652	MROH7	protein_cc
ENSG0000	51.358	0.727262	0.091787	7.923391	2.31E-15	3.76E-14	PSMC1P1	pseudoge
ENSG0000	51.35319	0.170049	0.088933	1.91211	0.055862	0.09309	PPIEL	pseudoge
ENSG0000	51.34248	1.093056	0.137211	7.96622	1.64E-15	2.73E-14	AP000330	antisense
ENSG0000	51.30128	-0.05049	0.063081	-0.80037	0.423498	0.520547	RP11-426	lincRNA
ENSG0000	51.29423	-0.10656	0.146877	-0.72554	0.468121	0.563967	AC079305	lincRNA
ENSG0000	51.27026	0.187042	0.093027	2.01062	0.044366	0.075995	U2AF1	protein_cc
ENSG0000	51.26964	0.213067	0.077462	2.750607	0.005948	0.012469	ALS2CR12	protein_cc
ENSG0000	51.2248	0.056284	0.053429	1.053431	0.292143	0.383653	RP5-1024	antisense
ENSG0000	51.21582	0.095194	0.074897	1.271003	0.203727	0.283676	AC005329	antisense
ENSG0000	51.20036	-0.12573	0.114329	-1.09976	0.271437	0.361108	VNN1	protein_cc
ENSG0000	51.18512	-0.23514	0.077663	-3.02766	0.002465	0.005594	POMC	protein_cc
ENSG0000	51.16139	-1.21447	0.115338	-10.5296	6.31E-26	6.65E-24	SCUBE1	protein_cc
ENSG0000	51.15826	0.432502	0.095137	4.546098	5.46E-06	1.97E-05	RP11-119	antisense
ENSG0000	51.10996	-0.01209	0.048987	-0.2468	0.805062	0.85635	C17orf104	protein_cc
ENSG0000	51.1038	-0.40955	0.113999	-3.59256	0.000327	0.000876	FAM222A-	antisense
ENSG0000	51.06917	0.356241	0.061426	5.799538	6.65E-09	3.80E-08	RP11-100	lincRNA
ENSG0000	51.03034	0.936879	0.121323	7.722166	1.14E-14	1.64E-13	RPS14P3	pseudoge
ENSG0000	51.03023	-1.00955	0.107903	-9.35613	8.27E-21	3.22E-19	SLC9A7P1	pseudoge
ENSG0000	51.02255	-0.03213	0.048914	-0.65695	0.511214	0.604392	SCGB2B2	protein_cc
ENSG0000	51.00519	0.01934	0.079443	0.24345	0.807657	0.858362	TSPAN1	protein_cc
ENSG0000	50.99827	-0.39243	0.086372	-4.54352	5.53E-06	1.99E-05	THSD7B	protein_cc

ENSG0000(	50.97478	-0.36259	0.050142	-7.23114	4.79E-13	5.21E-12	C22orf26	protein_cc
ENSG0000(	50.89636	0.248892	0.049657	5.012244	5.38E-07	2.27E-06	MTCP1	protein_cc
ENSG0000(	50.88223	0.068488	0.099757	0.686554	0.492364	0.58683	RP11-21A	antisense
ENSG0000(	50.83757	0.748508	0.168596	4.439644	9.01E-06	3.13E-05	RASSF10	pseudoge
ENSG0000(	50.8318	-0.12692	0.070979	-1.78806	0.073767	0.118616	SLC2A1-A	lincRNA
ENSG0000(	50.81978	0.296807	0.054848	5.411486	6.25E-08	3.06E-07	CTC-471J1	sense_ove
ENSG0000(	50.80986	0.398557	0.117101	3.40352	0.000665	0.001676	FRRS1	protein_cc
ENSG0000(	50.80888	-0.61667	0.138686	-4.44653	8.73E-06	3.04E-05	RPRML	protein_cc
ENSG0000(	50.79689	0.352926	0.099029	3.563852	0.000365	0.000968	HIST1H4H	protein_cc
ENSG0000(	50.79056	0.187438	0.070289	2.666679	0.00766	0.015712	MEI1	protein_cc
ENSG0000(	50.74503	0.539197	0.077968	6.915611	4.66E-12	4.36E-11	AC000089	pseudoge
ENSG0000(	50.65798	0.492747	0.070175	7.021706	2.19E-12	2.16E-11	PIPSL	pseudoge
ENSG0000(	50.64114	0.094454	0.087302	1.081912	0.279292	0.369716	NUDT16P1	pseudoge
ENSG0000(	50.62682	0.441776	0.172798	2.5566	0.01057	0.021003	TMPRSS3	protein_cc
ENSG0000(	50.62612	-0.26551	0.08893	-2.98562	0.00283	0.006335	IGFALS	protein_cc
ENSG0000(	50.61941	1.145866	0.116591	9.828111	8.52E-23	4.79E-21	AC003090	lincRNA
ENSG0000(	50.61158	-0.0019	0.121732	-0.01557	0.987579	0.991412	RPLP0P2	pseudoge
ENSG0000(	50.60962	-1.24806	0.130606	-9.55589	1.23E-21	5.56E-20	DCTN1-AS	antisense
ENSG0000(	50.57369	-0.4535	0.124659	-3.6379	0.000275	0.000745	AC083864	lincRNA
ENSG0000(	50.55973	-0.90274	0.110076	-8.20111	2.38E-16	4.57E-15	SCN5A	protein_cc
ENSG0000(	50.52375	-0.30966	0.072193	-4.28937	1.79E-05	5.94E-05	RP5-968J1	lincRNA
ENSG0000(	50.51422	0.170488	0.062942	2.708668	0.006755	0.014006	C1orf101	protein_cc
ENSG0000(	50.51125	0.17999	0.061228	2.939665	0.003286	0.007267	RP11-552	antisense
ENSG0000(	50.48453	-0.31807	0.071227	-4.46549	7.99E-06	2.80E-05	FAM66D	antisense
ENSG0000(	50.47199	-0.02869	0.115676	-0.24799	0.80414	0.855503	RP4-610C	lincRNA
ENSG0000(	50.45766	-0.65273	0.09142	-7.13991	9.34E-13	9.76E-12	CTD-2020	antisense
ENSG0000(	50.43825	0.031425	0.04391	0.715681	0.474188	0.570034	RP11-770	antisense
ENSG0000(	50.37861	-0.4083	0.065368	-6.24619	4.21E-10	2.90E-09	RP11-588	antisense
ENSG0000(	50.34829	-0.18925	0.080548	-2.34959	0.018794	0.035197	LINC0088	antisense
ENSG0000(	50.295	0.022886	0.059351	0.385605	0.699789	0.770457	MAP3K14-	antisense
ENSG0000(	50.24736	0.688478	0.099068	6.949578	3.66E-12	3.49E-11	EIF4HP1	pseudoge
ENSG0000(	50.23696	-0.65347	0.132056	-4.94847	7.48E-07	3.08E-06	COL6A4P2	pseudoge
ENSG0000(	50.23501	-0.19603	0.076849	-2.55079	0.010748	0.021323	HSP90AB2	pseudoge
ENSG0000(	50.20005	0.197037	0.07648	2.576331	0.009986	0.019949	RP11-632	pseudoge
ENSG0000(	50.1847	0.703495	0.182003	3.865298	0.000111	0.000322	GJA3	protein_cc
ENSG0000(	50.18371	-0.42087	0.083935	-5.01425	5.32E-07	2.25E-06	SSBP3-AS	protein_cc
ENSG0000(	50.15519	0.205385	0.142041	1.445951	0.148191	0.216491	SLAMF7	protein_cc
ENSG0000(	50.15344	-0.11424	0.102585	-1.11358	0.265461	0.354316	LINC0117(	lincRNA
ENSG0000(	50.14932	0.21601	0.120944	1.786034	0.074094	0.119063	RP11-307	antisense
ENSG0000(	50.14706	-0.21226	0.092045	-2.30609	0.021105	0.039089	DNM1P51	pseudoge
ENSG0000(	50.13451	1.834406	0.264347	6.939387	3.94E-12	3.74E-11	HOXA9	protein_cc
ENSG0000(	50.11793	-0.77492	0.187659	-4.12941	3.64E-05	0.000114	GOLGA6L1	protein_cc
ENSG0000(	50.10004	0.832828	0.105698	7.879339	3.29E-15	5.22E-14	YBX1P2	pseudoge
ENSG0000(	50.09636	0.705197	0.098502	7.159227	8.11E-13	8.55E-12	CTD-2270	pseudoge
ENSG0000(	50.0634	0.226224	0.040387	5.601394	2.13E-08	1.12E-07	RP11-977	antisense
ENSG0000(	50.04907	0.187893	0.071195	2.63912	0.008312	0.016916	RP11-336	antisense
ENSG0000(	50.01406	0.160541	0.105853	1.516642	0.129357	0.192554	RP11-557	lincRNA
ENSG0000(	49.99698	0.407396	0.130425	3.123608	0.001786	0.004162	PTPN22	protein_cc
ENSG0000(	49.98661	0.462877	0.153792	3.009763	0.002615	0.005902	CPA4	protein_cc
ENSG0000(	49.96968	0.412664	0.081245	5.079226	3.79E-07	1.64E-06	AP1S3	protein_cc
ENSG0000(	49.91445	-0.38073	0.135213	-2.81574	0.004867	0.010391	INSRR	protein_cc
ENSG0000(	49.91436	-0.14187	0.062506	-2.26971	0.023225	0.042624	RP1-140A	antisense
ENSG0000(	49.9108	0.070164	0.118941	0.589904	0.555255	0.644782	LCK	protein_cc
ENSG0000(	49.90415	0.331004	0.071483	4.630512	3.65E-06	1.35E-05	LYG1	protein_cc
ENSG0000(	49.88425	-0.16493	0.07444	-2.21562	0.026717	0.048355	C5orf27	protein_cc
ENSG0000(	49.87241	0.359045	0.087014	4.1263	3.69E-05	0.000116	ARTN	protein_cc
ENSG0000(	49.82457	0.378355	0.121337	3.118228	0.001819	0.004229	MCOLN2	protein_cc
ENSG0000(	49.82402	0.229621	0.091948	2.497306	0.012514	0.024439	DCST2	protein_cc
ENSG0000(	49.82091	-0.4634	0.107386	-4.31529	1.59E-05	5.33E-05	RP11-416	antisense

ENSG0000(	49.80146	-0.33065	0.047567	-6.95124	3.62E-12	3.45E-11	RP11-342	sense_intr
ENSG0000(	49.79043	0.123542	0.100582	1.228269	0.219346	0.301776	FOXL1	protein_cc
ENSG0000(	49.76609	-0.05819	0.07842	-0.74204	0.458062	0.554495	RP11-848	sense_intr
ENSG0000(	49.74265	0.013293	0.146629	0.090656	0.927766	0.949161	EEF1GP1	pseudoge
ENSG0000(	49.73974	0.044121	0.101149	0.436199	0.662692	0.739413	PARP15	protein_cc
ENSG0000(	49.71082	0.194166	0.04631	4.192714	2.76E-05	8.86E-05	RP11-423	sense_intr
ENSG0000(	49.70651	0.256155	0.063876	4.010176	6.07E-05	0.000184	FBXL13	protein_cc
ENSG0000(	49.6959	0.509518	0.077875	6.542748	6.04E-11	4.76E-10	RP11-572	lincRNA
ENSG0000(	49.65675	-0.10022	0.065579	-1.52816	0.126473	0.189041	TAS1R3	protein_cc
ENSG0000(	49.61631	-0.31562	0.133845	-2.35809	0.018369	0.034491	CHRNA3	protein_cc
ENSG0000(	49.6115	0.380849	0.052301	7.281942	3.29E-13	3.68E-12	PFN4	protein_cc
ENSG0000(	49.60504	0.135821	0.05648	2.404758	0.016183	0.03084	RP11-105	lincRNA
ENSG0000(	49.59258	-0.51562	0.085938	-5.99989	1.97E-09	1.23E-08	SCN11A	protein_cc
ENSG0000(	49.5233	0.26351	0.128508	2.050528	0.040313	0.069871	RNF212	protein_cc
ENSG0000(	49.5138	-0.05997	0.074242	-0.80775	0.419233	0.516199	CCDC17	protein_cc
ENSG0000(	49.40362	0.215727	0.15701	1.373972	0.16945	0.24277	RP11-664	protein_cc
ENSG0000(	49.38852	-0.04832	0.067896	-0.71168	0.476661	0.572328	KB-1732A	lincRNA
ENSG0000(	49.38584	-0.09485	0.063946	-1.48321	0.138019	0.20359	CCDC87	protein_cc
ENSG0000(	49.37657	0.138774	0.103897	1.33569	0.181651	0.257247	CTSW	protein_cc
ENSG0000(	49.36546	0.134493	0.096785	1.389598	0.164651	0.236843	HLA-DOB	protein_cc
ENSG0000(	49.338	0.123293	0.06521	1.890705	0.058664	0.097148	RP3-322G	antisense
ENSG0000(	49.32729	-0.66991	0.092765	-7.22157	5.14E-13	5.57E-12	MYADML2	protein_cc
ENSG0000(	49.31918	0.190071	0.072863	2.608622	0.009091	0.018331	MORF4L2-	antisense
ENSG0000(	49.31762	0.287745	0.082971	3.467993	0.000524	0.001348	PKD1L1	protein_cc
ENSG0000(	49.31432	0.007534	0.053066	0.14198	0.887096	0.919633	RP11-281	antisense
ENSG0000(	49.27197	-0.65174	0.114485	-5.69275	1.25E-08	6.82E-08	PKD2L1	protein_cc
ENSG0000(	49.25431	-0.14634	0.051154	-2.86082	0.004225	0.009147	CCDC62	protein_cc
ENSG0000(	49.22296	0.134077	0.063981	2.095568	0.036121	0.063396	AC073343	antisense
ENSG0000(	49.22259	0.799419	0.143983	5.552171	2.82E-08	1.46E-07	CCDC37	protein_cc
ENSG0000(	49.22024	-0.09624	0.093703	-1.02707	0.304388	0.397248	RP4-555D	lincRNA
ENSG0000(	49.15515	0.59608	0.084302	7.070797	1.54E-12	1.56E-11	CHRNA5	protein_cc
ENSG0000(	49.15313	0.110535	0.126138	0.876303	0.380865	0.477434	HTR1D	protein_cc
ENSG0000(	49.15111	0.302499	0.061117	4.949536	7.44E-07	3.07E-06	RP11-153	pseudoge
ENSG0000(	49.14227	0.404918	0.137107	2.953289	0.003144	0.006978	RP11-93B	antisense
ENSG0000(	49.08563	-0.39675	0.072298	-5.48767	4.07E-08	2.06E-07	KB-1507C	lincRNA
ENSG0000(	49.08294	-0.15415	0.097473	-1.58142	0.113783	0.172703	PRSS50	protein_cc
ENSG0000(	49.05922	0.116128	0.051974	2.234363	0.025459	0.046288	RP11-568	lincRNA
ENSG0000(	48.96052	0.230561	0.065702	3.509202	0.000449	0.001171	BHLHE40-	antisense
ENSG0000(	48.95467	0.391551	0.069583	5.627121	1.83E-08	9.77E-08	MSH5-SA	protein_cc
ENSG0000(	48.91275	-0.4598	0.119733	-3.84019	0.000123	0.000354	TGFBR3L	protein_cc
ENSG0000(	48.91018	0.110524	0.121671	0.90838	0.363678	0.459657	BATF	protein_cc
ENSG0000(	48.90415	0.56886	0.127291	4.468957	7.86E-06	2.76E-05	OSTM1-A	lincRNA
ENSG0000(	48.86436	0.213062	0.069656	3.058778	0.002222	0.005083	CEL	protein_cc
ENSG0000(	48.80248	-0.04977	0.051553	-0.96546	0.334316	0.428981	RP11-165.	antisense
ENSG0000(	48.80036	0.142994	0.06715	2.129462	0.033216	0.058774	RP11-235	antisense
ENSG0000(	48.78043	-0.2156	0.059982	-3.59439	0.000325	0.00087	EPX	protein_cc
ENSG0000(	48.75496	0.107433	0.044939	2.390611	0.01682	0.0319	DEPDC4	protein_cc
ENSG0000(	48.75485	0.931173	0.119945	7.763315	8.27E-15	1.23E-13	SP5	protein_cc
ENSG0000(	48.7481	0.52805	0.245898	2.147434	0.031759	0.056446	SAA2	protein_cc
ENSG0000(	48.73156	0.13209	0.033066	3.994778	6.48E-05	0.000196	C7orf25	protein_cc
ENSG0000(	48.72869	0.164092	0.057412	2.858153	0.004261	0.009214	CTC-366B	lincRNA
ENSG0000(	48.72071	-0.24949	0.061956	-4.02681	5.65E-05	0.000173	RGL4	protein_cc
ENSG0000(	48.69066	0.408038	0.108162	3.772463	0.000162	0.000456	TAAR3	pseudoge
ENSG0000(	48.68658	0.358843	0.085715	4.18644	2.83E-05	9.08E-05	CLDN4	protein_cc
ENSG0000(	48.66059	0.065276	0.088159	0.740432	0.459038	0.555318	RP11-341	antisense
ENSG0000(	48.61748	0.239416	0.146937	1.629377	0.103233	0.15883	UBD	protein_cc
ENSG0000(	48.56002	-0.12042	0.075867	-1.58719	0.11247	0.170994	ARL17B	protein_cc
ENSG0000(	48.53948	-0.15136	0.153873	-0.98366	0.325283	0.419485	RP11-1C8	antisense
ENSG0000(	48.52957	-0.15106	0.063038	-2.39635	0.016559	0.03146	RP11-429.	antisense

ENSG0000(	48.5085	-0.20424	0.10741	-1.9015	0.057237	0.095096	ANGPT4	protein_cc
ENSG0000(	48.49858	0.248692	0.124743	1.993639	0.046192	0.078778	ZBP1	protein_cc
ENSG0000(	48.48619	-2.07227	0.221865	-9.34023	9.61E-21	3.71E-19	MCHR2	protein_cc
ENSG0000(	48.47641	-0.19286	0.083071	-2.32167	0.020251	0.037649	KEL	protein_cc
ENSG0000(	48.45929	0.575689	0.129472	4.446447	8.73E-06	3.04E-05	F2RL3	protein_cc
ENSG0000(	48.44507	-0.57146	0.135653	-4.21264	2.52E-05	8.15E-05	ANKRD18.	protein_cc
ENSG0000(	48.40018	0.619355	0.100863	6.140529	8.22E-10	5.43E-09	RPL3P7	pseudoge
ENSG0000(	48.35884	-0.49301	0.125803	-3.91888	8.90E-05	0.000262	ADH1B	protein_cc
ENSG0000(	48.32496	0.078383	0.076502	1.02459	0.305557	0.398507	AC090587	sense_ove
ENSG0000(	48.3139	0.004057	0.047217	0.08593	0.931522	0.952128	CTA-253N	pseudoge
ENSG0000(	48.31387	-0.21439	0.078346	-2.7365	0.00621	0.012971	LINC0085(	lincRNA
ENSG0000(	48.30807	-0.2583	0.074292	-3.47684	0.000507	0.001307	EFCAB5	protein_cc
ENSG0000(	48.28937	0.180934	0.061098	2.961394	0.003062	0.006812	AC004158	lincRNA
ENSG0000(	48.28714	0.478092	0.062857	7.606077	2.83E-14	3.77E-13	RP11-715.	antisense
ENSG0000(	48.26489	-0.01131	0.066064	-0.17114	0.864117	0.902271	RP11-351	lincRNA
ENSG0000(	48.23919	-0.47057	0.077132	-6.10082	1.06E-09	6.86E-09	MIR3615	antisense
ENSG0000(	48.22337	-0.50999	0.124662	-4.09095	4.30E-05	0.000134	CCSER1	protein_cc
ENSG0000(	48.21787	0.486586	0.066754	7.289223	3.12E-13	3.50E-12	YY2	protein_cc
ENSG0000(	48.20193	-0.06946	0.095036	-0.73088	0.464852	0.560883	GPR97	protein_cc
ENSG0000(	48.16024	0.873345	0.131626	6.635025	3.24E-11	2.66E-10	RP11-112.	pseudoge
ENSG0000(	48.15362	-0.03151	0.0758	-0.41575	0.677589	0.751772	GCHFR	protein_cc
ENSG0000(	48.13701	-0.46951	0.096906	-4.84503	1.27E-06	5.04E-06	C9orf129	protein_cc
ENSG0000(	48.11384	0.395169	0.059804	6.607688	3.90E-11	3.17E-10	RP11-428.	lincRNA
ENSG0000(	48.11275	0.337706	0.126854	2.662165	0.007764	0.015905	RP11-561	antisense
ENSG0000(	48.10768	-0.85316	0.105859	-8.05946	7.66E-16	1.35E-14	TCHH	protein_cc
ENSG0000(	48.10347	0.194142	0.059187	3.280149	0.001038	0.00252	ARL17A	protein_cc
ENSG0000(	48.04802	-0.22345	0.152667	-1.46365	0.14329	0.210302	NMUR2	protein_cc
ENSG0000(	47.99392	0.283604	0.083983	3.376905	0.000733	0.001831	FTH1P3	pseudoge
ENSG0000(	47.9937	-0.41733	0.123264	-3.38569	0.00071	0.001779	RP1-223B	lincRNA
ENSG0000(	47.97657	0.496911	0.0888	5.595823	2.20E-08	1.16E-07	DOK7	protein_cc
ENSG0000(	47.93537	0.539308	0.153058	3.523551	0.000426	0.001114	COX20P1	pseudoge
ENSG0000(	47.92932	0.076601	0.051388	1.490644	0.136055	0.201104	CTB-179K	antisense
ENSG0000(	47.92705	0.648526	0.09979	6.498931	8.09E-11	6.25E-10	TPRG1	protein_cc
ENSG0000(	47.92031	0.135108	0.103263	1.308391	0.190741	0.268181	CUZD1	protein_cc
ENSG0000(	47.91201	0.165142	0.038019	4.343643	1.40E-05	4.73E-05	RP11-973	sense_intr
ENSG0000(	47.90061	0.692014	0.09064	7.634749	2.26E-14	3.07E-13	RPL35P1	pseudoge
ENSG0000(	47.86738	0.045035	0.047078	0.956598	0.33877	0.433722	RP11-267	lincRNA
ENSG0000(	47.83262	0.822666	0.114032	7.214319	5.42E-13	5.86E-12	RP11-553	pseudoge
ENSG0000(	47.82691	0.84253	0.11634	7.241937	4.42E-13	4.84E-12	RPS2P55	pseudoge
ENSG0000(	47.8268	0.026497	0.113206	0.234057	0.81494	0.864109	CST7	protein_cc
ENSG0000(	47.81473	-0.53497	0.114887	-4.65646	3.22E-06	1.20E-05	RP11-286	antisense
ENSG0000(	47.80133	0.089902	0.075876	1.184844	0.236079	0.320911	RP11-250	lincRNA
ENSG0000(	47.8009	-0.13037	0.148399	-0.87852	0.379664	0.476171	GSDMA	protein_cc
ENSG0000(	47.75039	1.011485	0.123574	8.185288	2.72E-16	5.14E-15	RP11-254	pseudoge
ENSG0000(	47.72496	-0.09028	0.067818	-1.33126	0.183103	0.258939	RP11-216	processed
ENSG0000(	47.72029	-0.20109	0.137393	-1.4636	0.143302	0.210312	MIR137HC	lincRNA
ENSG0000(	47.68841	0.293824	0.146681	2.003148	0.045161	0.077201	GZMA	protein_cc
ENSG0000(	47.66233	0.176281	0.055872	3.155103	0.001604	0.003769	TMEM256	protein_cc
ENSG0000(	47.64537	-0.70299	0.153031	-4.5938	4.35E-06	1.59E-05	MTND4P2	pseudoge
ENSG0000(	47.63209	-0.50673	0.139878	-3.62264	0.000292	0.000786	PKP1	protein_cc
ENSG0000(	47.62775	-0.90574	0.124583	-7.27016	3.59E-13	3.99E-12	F7	protein_cc
ENSG0000(	47.6177	-0.00207	0.086605	-0.02389	0.980939	0.986649	LRRIQ3	protein_cc
ENSG0000(	47.60916	0.211918	0.065149	3.252835	0.001143	0.002754	AC018755	pseudoge
ENSG0000(	47.60003	-0.19927	0.06621	-3.00972	0.002615	0.005903	SMG7-AS.	processed
ENSG0000(	47.59881	0.015247	0.065789	0.231762	0.816723	0.865554	AC018638	pseudoge
ENSG0000(	47.59318	-0.41805	0.076898	-5.43643	5.44E-08	2.68E-07	RP11-430	lincRNA
ENSG0000(	47.57129	0.82545	0.113956	7.243601	4.37E-13	4.79E-12	RP11-627	pseudoge
ENSG0000(	47.51368	0.633145	0.17731	3.570834	0.000356	0.000945	SCGN	protein_cc
ENSG0000(	47.49232	0.904983	0.11231	8.057877	7.76E-16	1.36E-14	CTB-33G1	pseudoge

ENSG0000(	47.4667	0.233929	0.037995	6.156868	7.42E-10	4.94E-09	RP11-297	processed
ENSG0000(	47.45229	0.282639	0.077036	3.668916	0.000244	0.000666	RP11-563	lincRNA
ENSG0000(	47.4381	0.620436	0.124393	4.987726	6.11E-07	2.55E-06	ERP27	protein_cc
ENSG0000(	47.4066	0.310155	0.078142	3.969122	7.21E-05	0.000216	RP11-646	lincRNA
ENSG0000(	47.40545	0.052861	0.101129	0.52271	0.601176	0.685829	CTD-2308	antisense
ENSG0000(	47.40472	0.421869	0.056007	7.532461	4.98E-14	6.39E-13	RP11-872.	antisense
ENSG0000(	47.39264	0.077777	0.072794	1.068452	0.285317	0.376166	ESRRB	protein_cc
ENSG0000(	47.33545	-0.01615	0.099416	-0.16241	0.870982	0.907277	HES7	protein_cc
ENSG0000(	47.33338	0.295817	0.059945	4.934839	8.02E-07	3.29E-06	IL20RB	protein_cc
ENSG0000(	47.31847	0.105036	0.054516	1.926699	0.054017	0.090407	TPI1P2	pseudoge
ENSG0000(	47.23563	-0.13317	0.063825	-2.08655	0.036929	0.064641	MRPS31P5	pseudoge
ENSG0000(	47.23004	0.322489	0.081334	3.965003	7.34E-05	0.00022	KLK14	protein_cc
ENSG0000(	47.2283	0.000467	0.050336	0.00927	0.992604	0.995023	RPL5P30	pseudoge
ENSG0000(	47.20495	-0.00158	0.046119	-0.03435	0.972598	0.980874	RP11-46D	lincRNA
ENSG0000(	47.17697	0.197743	0.068436	2.889473	0.003859	0.008419	RP1-261D	antisense
ENSG0000(	47.15392	0.197066	0.052118	3.781146	0.000156	0.000441	AC004221	lincRNA
ENSG0000(	47.12138	0.238924	0.052858	4.520152	6.18E-06	2.21E-05	RP11-266	lincRNA
ENSG0000(	47.11895	-0.17401	0.113091	-1.53867	0.123886	0.185681	AC010980	pseudoge
ENSG0000(	47.11807	0.668433	0.096099	6.955665	3.51E-12	3.36E-11	PABPC4L	protein_cc
ENSG0000(	47.1169	0.076877	0.052368	1.468005	0.142103	0.208775	RP11-343	lincRNA
ENSG0000(	47.10285	0.045995	0.041883	1.098165	0.272132	0.361893	RP11-15A	antisense
ENSG0000(	47.0604	-0.18092	0.092022	-1.96602	0.049296	0.083453	FAM66A	processed
ENSG0000(	47.04581	0.727805	0.101092	7.199415	6.05E-13	6.49E-12	RPL13AP7	pseudoge
ENSG0000(	47.02489	1.3353	0.143628	9.296923	1.45E-20	5.40E-19	PPIAP31	pseudoge
ENSG0000(	47.00282	-0.00996	0.084175	-0.11835	0.90579	0.933272	RP11-488	antisense
ENSG0000(	47.00134	-0.01142	0.068883	-0.16578	0.868334	0.905513	AMDHD1	protein_cc
ENSG0000(	46.99869	0.20906	0.059304	3.525219	0.000423	0.001108	RP11-385	antisense
ENSG0000(	46.98856	0.22091	0.060623	3.643998	0.000268	0.000728	VN1R81P	pseudoge
ENSG0000(	46.91261	0.338654	0.094556	3.581512	0.000342	0.00091	XPNPEP2	protein_cc
ENSG0000(	46.91234	-0.14963	0.064621	-2.31546	0.020588	0.038216	RP11-283	antisense
ENSG0000(	46.90669	0.757908	0.106135	7.140982	9.27E-13	9.69E-12	EEF1A1P1(	pseudoge
ENSG0000(	46.90159	0.102371	0.054601	1.874897	0.060807	0.100217	RP11-849	lincRNA
ENSG0000(	46.89287	0.184579	0.062148	2.96999	0.002978	0.006642	AC009120	pseudoge
ENSG0000(	46.88848	-0.03745	0.111469	-0.33596	0.736898	0.800915	MYRFL	protein_cc
ENSG0000(	46.88356	-1.25688	0.154044	-8.15927	3.37E-16	6.28E-15	DLGAP1-/	antisense
ENSG0000(	46.88022	0.674911	0.059537	11.33604	8.70E-30	1.85E-27	DDX11-AS	antisense
ENSG0000(	46.85243	0.732768	0.103047	7.110999	1.15E-12	1.19E-11	EEF1A1P2.	pseudoge
ENSG0000(	46.81797	-0.27454	0.153014	-1.79421	0.07278	0.117272	CLEC10A	protein_cc
ENSG0000(	46.80096	-0.10532	0.053719	-1.96052	0.049935	0.084407	AC004987	pseudoge
ENSG0000(	46.78554	0.052661	0.151219	0.348247	0.727655	0.793006	ZNF208	protein_cc
ENSG0000(	46.78152	-0.07188	0.078653	-0.91388	0.360779	0.456654	RP3-412A	antisense
ENSG0000(	46.77183	-1.34124	0.168424	-7.96349	1.67E-15	2.78E-14	SDR16C5	protein_cc
ENSG0000(	46.76166	-0.34251	0.084706	-4.04346	5.27E-05	0.000161	RP11-489	lincRNA
ENSG0000(	46.75054	0.75253	0.14757	5.099472	3.41E-07	1.48E-06	CCDC19	protein_cc
ENSG0000(	46.73054	2.309356	0.32146	7.183964	6.77E-13	7.23E-12	HOXC11	protein_cc
ENSG0000(	46.73035	-0.53229	0.156354	-3.4044	0.000663	0.001672	CLIC6	protein_cc
ENSG0000(	46.72964	-0.2269	0.062215	-3.64697	0.000265	0.000721	RP11-425	antisense
ENSG0000(	46.7271	-0.33275	0.096923	-3.43317	0.000597	0.001517	FOXC2	protein_cc
ENSG0000(	46.69997	-1.29531	0.134426	-9.63585	5.64E-22	2.70E-20	SLC6A20	protein_cc
ENSG0000(	46.61112	0.06852	0.08983	0.762765	0.445604	0.542385	LPAL2	pseudoge
ENSG0000(	46.60298	-0.21704	0.103817	-2.09055	0.036568	0.064096	YBX2	protein_cc
ENSG0000(	46.58579	0.758146	0.179679	4.219458	2.45E-05	7.92E-05	RP11-92A	antisense
ENSG0000(	46.51761	0.251176	0.08526	2.946007	0.003219	0.00713	RP11-680	antisense
ENSG0000(	46.51392	1.026366	0.114346	8.975976	2.81E-19	8.54E-18	AC010468	pseudoge
ENSG0000(	46.51236	0.464837	0.112238	4.141514	3.45E-05	0.000109	CDKN2B-/	antisense
ENSG0000(	46.50922	0.030108	0.073844	0.407723	0.683477	0.75661	RP11-573	pseudoge
ENSG0000(	46.46189	0.276086	0.073771	3.742461	0.000182	0.000509	XXbac-BP	pseudoge
ENSG0000(	46.44596	0.633758	0.114601	5.530122	3.20E-08	1.64E-07	LINC0060(	lincRNA
ENSG0000(	46.44501	0.087527	0.109427	0.799865	0.423789	0.520842	SUCNR1	protein_cc

ENSG0000(	46.40226	-0.09486	0.048225	-1.9671	0.049172	0.083267	RP11-303	processed
ENSG0000(	46.39117	-0.62518	0.104237	-5.99773	2.00E-09	1.24E-08	WNT9B	protein_cc
ENSG0000(	46.34756	-0.12071	0.059232	-2.03788	0.041562	0.071778	AC137932	protein_cc
ENSG0000(	46.34578	-0.37703	0.099855	-3.77575	0.00016	0.00045	KCNG2	protein_cc
ENSG0000(	46.32872	-0.67292	0.102671	-6.5541	5.60E-11	4.43E-10	RP6-65G2	lincRNA
ENSG0000(	46.31399	0.036763	0.080894	0.454457	0.6495	0.728171	RP11-46F:	antisense
ENSG0000(	46.30041	0.338526	0.086668	3.905992	9.38E-05	0.000276	HMGB1P1	pseudoge
ENSG0000(	46.28902	-0.0203	0.092352	-0.21986	0.82598	0.872396	FAM24B	protein_cc
ENSG0000(	46.25686	0.130544	0.111604	1.169704	0.24212	0.327822	RP11-190.	antisense
ENSG0000(	46.20026	-0.94951	0.12206	-7.77901	7.31E-15	1.09E-13	EPN3	protein_cc
ENSG0000(	46.19765	0.917797	0.109938	8.348345	6.92E-17	1.45E-15	RPL9P7	pseudoge
ENSG0000(	46.19764	-0.04616	0.061968	-0.74492	0.456319	0.552793	AC010976	antisense
ENSG0000(	46.19576	-0.0623	0.087093	-0.71534	0.474397	0.570241	CTD-2008	lincRNA
ENSG0000(	46.18629	-0.0225	0.048932	-0.45978	0.645677	0.725071	RP5-1061	lincRNA
ENSG0000(	46.18277	-0.08201	0.060682	-1.35141	0.176565	0.251175	CTD-2002	sense_ove
ENSG0000(	46.18027	-0.01761	0.046249	-0.38085	0.703316	0.773408	RP1-317E:	antisense
ENSG0000(	46.14881	-0.8827	0.106389	-8.29696	1.07E-16	2.16E-15	RP11-133	lincRNA
ENSG0000(	46.14374	0.555448	0.08	6.943112	3.84E-12	3.64E-11	MIR181A2	antisense
ENSG0000(	46.1314	-0.60178	0.080017	-7.52063	5.45E-14	6.94E-13	CYP2B7P	pseudoge
ENSG0000(	46.11788	-0.05078	0.09461	-0.53675	0.591441	0.677071	BIRC7	protein_cc
ENSG0000(	46.09576	-0.40133	0.076387	-5.25394	1.49E-07	6.85E-07	RP11-192	sense_intr
ENSG0000(	46.08866	-0.17034	0.072865	-2.33776	0.0194	0.036221	MATN1-A	antisense
ENSG0000(	46.08642	0.699909	0.111342	6.286106	3.26E-10	2.29E-09	HIST1H2B.	protein_cc
ENSG0000(	46.05221	0.128095	0.072702	1.761923	0.078082	0.124687	RP11-923	lincRNA
ENSG0000(	45.99408	-0.11609	0.0497	-2.3359	0.019497	0.036385	RP11-572	antisense
ENSG0000(	45.98315	0.193402	0.066287	2.917659	0.003527	0.007751	FAM212B-	antisense
ENSG0000(	45.9569	0.104835	0.152977	0.685299	0.493156	0.587569	PDZK1IP1	protein_cc
ENSG0000(	45.93737	-0.01806	0.071267	-0.25341	0.799952	0.852128	HMGB3P2	pseudoge
ENSG0000(	45.93176	-0.04313	0.140525	-0.30695	0.758885	0.818711	FAM180B	protein_cc
ENSG0000(	45.92807	-0.25382	0.058608	-4.33076	1.49E-05	4.99E-05	C17orf67	protein_cc
ENSG0000(	45.92027	-0.33035	0.058512	-5.6458	1.64E-08	8.82E-08	SUGT1P3	pseudoge
ENSG0000(	45.87789	1.159499	0.122564	9.460351	3.07E-21	1.30E-19	MIR155H(	lincRNA
ENSG0000(	45.84965	-0.02309	0.044945	-0.51368	0.607475	0.691289	RP1-234P	lincRNA
ENSG0000(	45.84941	-0.02039	0.056695	-0.35957	0.719169	0.78622	RP11-572	lincRNA
ENSG0000(	45.838	-0.14356	0.046135	-3.11174	0.00186	0.004314	AC092594	lincRNA
ENSG0000(	45.83322	-0.20229	0.095783	-2.11196	0.03469	0.06113	SYTL1	protein_cc
ENSG0000(	45.79867	-0.07996	0.062809	-1.27312	0.202976	0.282822	ARHGAP3	antisense
ENSG0000(	45.79316	-0.57445	0.147125	-3.90452	9.44E-05	0.000277	C1orf168	protein_cc
ENSG0000(	45.79199	-0.62606	0.10898	-5.74467	9.21E-09	5.13E-08	DUOXA1	protein_cc
ENSG0000(	45.78775	-1.19679	0.176058	-6.7977	1.06E-11	9.39E-11	CTB-1I21.	lincRNA
ENSG0000(	45.78556	0.74125	0.087164	8.504129	1.83E-17	4.20E-16	LINC0066	lincRNA
ENSG0000(	45.7669	-0.09145	0.083751	-1.09195	0.274854	0.36491	FZD10	protein_cc
ENSG0000(	45.74695	0.166159	0.066253	2.50793	0.012144	0.023796	CTD-2514	pseudoge
ENSG0000(	45.71947	-0.52054	0.081432	-6.39234	1.63E-10	1.20E-09	BTBD8	protein_cc
ENSG0000(	45.68984	0.283736	0.093753	3.026426	0.002475	0.005614	CCDC114	protein_cc
ENSG0000(	45.66942	-0.0149	0.098896	-0.15062	0.880275	0.91438	RP11-375	lincRNA
ENSG0000(	45.65573	0.077141	0.051593	1.495184	0.134867	0.19957	KLHL41	protein_cc
ENSG0000(	45.62168	-0.22213	0.097784	-2.27168	0.023106	0.04243	RP11-262	pseudoge
ENSG0000(	45.58266	-0.02038	0.089885	-0.22672	0.820645	0.868478	CTD-2651	antisense
ENSG0000(	45.57864	-0.28073	0.06399	-4.38714	1.15E-05	3.93E-05	TFP1	pseudoge
ENSG0000(	45.56943	0.893607	0.136722	6.535953	6.32E-11	4.96E-10	RP11-157.	lincRNA
ENSG0000(	45.56245	0.938752	0.112301	8.359251	6.31E-17	1.33E-15	BZW1P2	pseudoge
ENSG0000(	45.54262	-0.19045	0.079498	-2.39564	0.016591	0.031517	C1orf220	protein_cc
ENSG0000(	45.54117	0.025209	0.086659	0.290898	0.771129	0.828658	LINC0092	lincRNA
ENSG0000(	45.46764	0.827433	0.156819	5.276364	1.32E-07	6.12E-07	KRT7	protein_cc
ENSG0000(	45.35249	-0.31334	0.080842	-3.87599	0.000106	0.000309	A2M-AS1	antisense
ENSG0000(	45.34647	0.663924	0.115381	5.754202	8.71E-09	4.87E-08	MYB	protein_cc
ENSG0000(	45.33034	0.495627	0.145103	3.415693	0.000636	0.001609	EBF3	protein_cc
ENSG0000(	45.32938	0.573681	0.092294	6.215816	5.11E-10	3.48E-09	RP1-278E.	pseudoge

ENSG0000	45.32448	-0.07	0.061535	-1.13754	0.255314	0.34292	CTB-131B	processed
ENSG0000	45.31798	0.324579	0.0617	5.260588	1.44E-07	6.63E-07	SPDYA	protein_cc
ENSG0000	45.30806	-0.10603	0.055285	-1.91788	0.055126	0.092034	KRBA2	protein_cc
ENSG0000	45.30363	0.696907	0.081731	8.526863	1.50E-17	3.49E-16	RPSAP9	pseudoge
ENSG0000	45.29077	-0.09213	0.082078	-1.1225	0.26165	0.350081	PRDM6	protein_cc
ENSG0000	45.24584	-0.24889	0.0798	-3.11892	0.001815	0.004221	RAB11FIP1	pseudoge
ENSG0000	45.2311	0.315384	0.084578	3.728926	0.000192	0.000535	RP11-157	antisense
ENSG0000	45.22171	-0.0038	0.086388	-0.04399	0.964908	0.975986	RP11-861	antisense
ENSG0000	45.21448	0.047205	0.079105	0.596739	0.550681	0.640538	GCKR	protein_cc
ENSG0000	45.17195	1.803333	0.302233	5.966692	2.42E-09	1.48E-08	RP11-189	lincRNA
ENSG0000	45.16871	-0.29949	0.068081	-4.39901	1.09E-05	3.74E-05	SEC1P	pseudoge
ENSG0000	45.16754	-0.07842	0.083082	-0.94391	0.345217	0.440631	AC109826	lincRNA
ENSG0000	45.13508	0.5471	0.096203	5.686938	1.29E-08	7.05E-08	HSPA8P1	pseudoge
ENSG0000	45.13178	0.017486	0.059218	0.295289	0.767773	0.825721	RP11-81N	lincRNA
ENSG0000	45.08254	0.296286	0.053348	5.553831	2.79E-08	1.45E-07	CHRNA10	protein_cc
ENSG0000	45.0609	-0.4913	0.107051	-4.5894	4.45E-06	1.62E-05	RP11-343.	antisense
ENSG0000	45.04283	0.654674	0.153267	4.271456	1.94E-05	6.40E-05	RIBC2	protein_cc
ENSG0000	44.99984	-0.09609	0.074629	-1.28763	0.197875	0.276708	ZNF534	protein_cc
ENSG0000	44.99963	-0.06757	0.146205	-0.46219	0.643944	0.723559	KRT19	protein_cc
ENSG0000	44.99328	0.943907	0.12978	7.273118	3.51E-13	3.91E-12	CDC42P6	pseudoge
ENSG0000	44.97723	0.043406	0.089521	0.484877	0.627764	0.709352	WFDC3	protein_cc
ENSG0000	44.97413	-0.05889	0.122386	-0.48117	0.630399	0.71174	RP11-626	lincRNA
ENSG0000	44.89371	-0.44181	0.072986	-6.05335	1.42E-09	9.02E-09	AC007970	antisense
ENSG0000	44.82875	-0.19773	0.114527	-1.7265	0.084257	0.133085	LRRC63	protein_cc
ENSG0000	44.80575	0.094814	0.108953	0.87023	0.384175	0.480864	RP11-403	lincRNA
ENSG0000	44.79281	-0.38103	0.086382	-4.41096	1.03E-05	3.55E-05	TEX29	protein_cc
ENSG0000	44.78029	0.091357	0.0742	1.231226	0.218238	0.300493	FOXL2	protein_cc
ENSG0000	44.72196	-0.25461	0.082464	-3.08748	0.002019	0.004654	FAM132A	protein_cc
ENSG0000	44.70141	-0.09268	0.083758	-1.10651	0.268504	0.357709	PLA2G4B	protein_cc
ENSG0000	44.69665	-0.14693	0.069238	-2.12213	0.033827	0.059748	TAS2R5	protein_cc
ENSG0000	44.68673	-0.02944	0.081668	-0.36048	0.718486	0.785637	LA16c-38	lincRNA
ENSG0000	44.59751	0.673747	0.094692	7.115152	1.12E-12	1.15E-11	CALM2P2	pseudoge
ENSG0000	44.59681	-0.66381	0.085758	-7.74049	9.90E-15	1.44E-13	RP11-701	sense_intr
ENSG0000	44.59169	0.481092	0.091544	5.255306	1.48E-07	6.81E-07	ALOX12P2	pseudoge
ENSG0000	44.589	1.554181	0.281007	5.530752	3.19E-08	1.63E-07	SIX6	protein_cc
ENSG0000	44.55335	0.468037	0.058984	7.934918	2.11E-15	3.45E-14	CTD-2325	lincRNA
ENSG0000	44.52141	-1.064	0.124771	-8.52766	1.49E-17	3.47E-16	THEMIS	protein_cc
ENSG0000	44.50777	-0.1328	0.05365	-2.47525	0.013314	0.025862	NUS1P1	pseudoge
ENSG0000	44.49498	0.408352	0.074983	5.445902	5.15E-08	2.55E-07	EPS8L1	protein_cc
ENSG0000	44.48027	0.354806	0.069096	5.134976	2.82E-07	1.24E-06	HNRNPA1	pseudoge
ENSG0000	44.45215	0.005412	0.060565	0.08936	0.928795	0.949962	RP11-206	lincRNA
ENSG0000	44.42785	0.37768	0.102258	3.693414	0.000221	0.000609	MST1L	pseudoge
ENSG0000	44.42076	-0.41927	0.117509	-3.568	0.00036	0.000954	RP11-243.	lincRNA
ENSG0000	44.40028	-0.37524	0.081282	-4.61657	3.90E-06	1.44E-05	FAT2	protein_cc
ENSG0000	44.37041	-0.14886	0.137981	-1.07883	0.280663	0.371244	IL10	protein_cc
ENSG0000	44.32332	-0.24777	0.068485	-3.61785	0.000297	0.0008	RP11-122	antisense
ENSG0000	44.31764	-1.17529	0.123234	-9.53706	1.47E-21	6.61E-20	PAK6	protein_cc
ENSG0000	44.25776	0.071812	0.041039	1.749853	0.080144	0.127474	RP11-849	antisense
ENSG0000	44.25668	0.368748	0.071898	5.128761	2.92E-07	1.28E-06	SPATA3-A	lincRNA
ENSG0000	44.24849	-0.1142	0.047568	-2.40085	0.016357	0.031124	PTGES3P3	pseudoge
ENSG0000	44.24557	0.65565	0.094775	6.91799	4.58E-12	4.29E-11	RPL7AP66	pseudoge
ENSG0000	44.20989	-0.18948	0.101069	-1.87479	0.060821	0.100233	RP11-179.	lincRNA
ENSG0000	44.20982	1.195208	0.152278	7.848858	4.20E-15	6.54E-14	C15orf48	protein_cc
ENSG0000	44.19895	-0.17458	0.053251	-3.27836	0.001044	0.002535	AGBL5-IT1	sense_intr
ENSG0000	44.17563	-0.08934	0.111999	-0.79766	0.425068	0.522165	FAM150B	protein_cc
ENSG0000	44.14744	-0.97111	0.135808	-7.15058	8.64E-13	9.08E-12	DCLK3	protein_cc
ENSG0000	44.12996	-0.45208	0.124582	-3.62876	0.000285	0.00077	VWFP1	pseudoge
ENSG0000	44.09258	-0.34159	0.136083	-2.51017	0.012067	0.023662	FCN1	protein_cc
ENSG0000	44.09051	0.079348	0.076235	1.040831	0.297954	0.390213	AC093668	miRNA

ENSG0000(	44.08524	0.830934	0.137141	6.058993	1.37E-09	8.74E-09	ZNF474	protein_cc
ENSG0000(	44.08035	0.20446	0.070307	2.908118	0.003636	0.00797	AP001347	antisense
ENSG0000(	44.07339	-0.11013	0.061486	-1.7911	0.073277	0.117948	FPGT-TNN	protein_cc
ENSG0000(	43.94555	0.179373	0.070842	2.532022	0.011341	0.022378	RP11-274	lincRNA
ENSG0000(	43.91253	-0.72525	0.076942	-9.42584	4.27E-21	1.76E-19	SEPHS1P6	pseudoge
ENSG0000(	43.86616	0.333383	0.095071	3.506659	0.000454	0.001181	ASIP	protein_cc
ENSG0000(	43.86407	0.111283	0.114936	0.968216	0.332937	0.427491	LILRA6	protein_cc
ENSG0000(	43.86107	-0.63254	0.117968	-5.36199	8.23E-08	3.95E-07	LDLRAD4-	antisense
ENSG0000(	43.85356	0.859132	0.150452	5.710354	1.13E-08	6.20E-08	RP11-436	lincRNA
ENSG0000(	43.83631	0.255635	0.056028	4.562606	5.05E-06	1.83E-05	RP5-1125.	lincRNA
ENSG0000(	43.83582	-0.08587	0.053716	-1.59856	0.109917	0.167628	RP6-74O6	lincRNA
ENSG0000(	43.8169	-0.53724	0.091233	-5.88869	3.89E-09	2.31E-08	NKAPL	protein_cc
ENSG0000(	43.81191	-0.07754	0.055713	-1.39184	0.163972	0.236047	CTC-428H	antisense
ENSG0000(	43.77431	-0.20437	0.162318	-1.2591	0.207994	0.288669	CCR2	protein_cc
ENSG0000(	43.7459	0.308471	0.08861	3.481226	0.000499	0.001288	ASB9	protein_cc
ENSG0000(	43.74166	0.0714	0.036795	1.940488	0.05232	0.087926	RP11-712	protein_cc
ENSG0000(	43.70998	-0.37799	0.085041	-4.44474	8.80E-06	3.06E-05	RP1-90L6.	lincRNA
ENSG0000(	43.69534	0.276309	0.044775	6.171076	6.78E-10	4.55E-09	SPTY2D1-	processed
ENSG0000(	43.67854	0.154427	0.12563	1.229226	0.218987	0.301373	COL17A1	protein_cc
ENSG0000(	43.6763	-1.81925	0.17861	-10.1856	2.30E-24	1.78E-22	FADS6	protein_cc
ENSG0000(	43.64627	0.322492	0.050829	6.344636	2.23E-10	1.61E-09	RP11-307	antisense
ENSG0000(	43.60863	0.898179	0.195494	4.594411	4.34E-06	1.59E-05	TBX5-AS1	antisense
ENSG0000(	43.59632	1.418011	0.21568	6.574622	4.88E-11	3.90E-10	GPR1	protein_cc
ENSG0000(	43.52645	0.030408	0.053886	0.564302	0.572549	0.659936	RP11-755	antisense
ENSG0000(	43.50408	0.723896	0.099356	7.285889	3.20E-13	3.58E-12	TAS1R1	protein_cc
ENSG0000(	43.50165	1.079122	0.119068	9.063055	1.27E-19	4.08E-18	TBX1	protein_cc
ENSG0000(	43.47244	0.038692	0.039948	0.968539	0.332775	0.427324	MEMO1P1	pseudoge
ENSG0000(	43.45795	-0.05289	0.116625	-0.45353	0.650166	0.728799	P2RX1	protein_cc
ENSG0000(	43.43855	0.373351	0.064656	5.774437	7.72E-09	4.36E-08	RP11-29H	pseudoge
ENSG0000(	43.40441	0.363915	0.072172	5.042293	4.60E-07	1.96E-06	NPPA-AS1	antisense
ENSG0000(	43.30671	0.242075	0.078355	3.089445	0.002005	0.004626	GTF2H2B	pseudoge
ENSG0000(	43.29279	-0.05076	0.069246	-0.73308	0.46351	0.559641	PROSER2	protein_cc
ENSG0000(	43.28822	0.954468	0.090196	10.58213	3.61E-26	3.94E-24	10-Mar	protein_cc
ENSG0000(	43.2834	0.228915	0.084599	2.705892	0.006812	0.014113	HIST2H2B	protein_cc
ENSG0000(	43.27908	0.755868	0.192883	3.918796	8.90E-05	0.000263	FAM159B	protein_cc
ENSG0000(	43.27031	-0.46747	0.13014	-3.59208	0.000328	0.000877	MTATP8P2	pseudoge
ENSG0000(	43.27021	0.638933	0.104724	6.101096	1.05E-09	6.85E-09	GPR39	protein_cc
ENSG0000(	43.26923	0.015854	0.063491	0.249702	0.802818	0.854348	AC012358	pseudoge
ENSG0000(	43.26629	0.091944	0.089242	1.030273	0.302882	0.39557	LINC0089	antisense
ENSG0000(	43.24726	0.824076	0.110864	7.433185	1.06E-13	1.28E-12	EEF1A1P7	pseudoge
ENSG0000(	43.23591	0.005684	0.162892	0.034897	0.972162	0.980627	SIK1	protein_cc
ENSG0000(	43.21064	0.677428	0.132756	5.102822	3.35E-07	1.46E-06	RUFY4	protein_cc
ENSG0000(	43.16561	-0.48347	0.073029	-6.62019	3.59E-11	2.93E-10	RP4-673D	pseudoge
ENSG0000(	43.16344	-0.25849	0.082956	-3.116	0.001833	0.004259	AANAT	protein_cc
ENSG0000(	43.12159	-0.15483	0.074971	-2.0652	0.038904	0.067694	REM2	protein_cc
ENSG0000(	43.11206	-0.15852	0.085523	-1.85349	0.063813	0.104543	C2orf73	protein_cc
ENSG0000(	43.11181	-0.17947	0.11034	-1.6265	0.103844	0.15957	AC016735	lincRNA
ENSG0000(	43.09793	0.303254	0.05681	5.338078	9.39E-08	4.47E-07	RP11-37C	antisense
ENSG0000(	43.07298	0.002653	0.075125	0.035315	0.971828	0.980497	S1PR4	protein_cc
ENSG0000(	43.06691	-0.07885	0.100999	-0.78074	0.434953	0.532019	RP11-475.	pseudoge
ENSG0000(	43.0567	0.054397	0.040924	1.329222	0.183775	0.259772	HNRNPKP	pseudoge
ENSG0000(	43.03522	-0.15176	0.152015	-0.99834	0.318117	0.41178	SFRP5	protein_cc
ENSG0000(	42.98617	-0.34662	0.083654	-4.14353	3.42E-05	0.000108	CTC-277H	processed
ENSG0000(	42.9859	0.278217	0.185829	1.497166	0.13435	0.198935	FAM81B	protein_cc
ENSG0000(	42.90982	-0.19	0.107949	-1.76013	0.078386	0.125066	RP11-848	pseudoge
ENSG0000(	42.90158	-0.14364	0.063989	-2.24474	0.024785	0.04518	RP11-140	lincRNA
ENSG0000(	42.88444	0.102162	0.047764	2.138898	0.032444	0.057554	RNASEK-(	protein_cc
ENSG0000(	42.87894	-0.31062	0.072387	-4.29102	1.78E-05	5.90E-05	RP11-348	antisense
ENSG0000(	42.87751	0.130903	0.075133	1.742283	0.081459	0.129186	AC105053	lincRNA

ENSG0000(	42.87224	0.814511	0.107229	7.59602	3.05E-14	4.05E-13	RP11-40C	pseudoge
ENSG0000(	42.87021	-0.20531	0.105036	-1.95464	0.050625	0.085426	CCDC147-	antisense
ENSG0000(	42.84443	0.456803	0.05922	7.713726	1.22E-14	1.74E-13	RP11-127	antisense
ENSG0000(	42.83627	0.152799	0.089389	1.70938	0.087381	0.137453	PRELID2	protein_cc
ENSG0000(	42.83269	0.579818	0.073096	7.932301	2.15E-15	3.52E-14	AC007238	pseudoge
ENSG0000(	42.82146	-0.00359	0.095703	-0.0375	0.970087	0.979207	P2RX6P	pseudoge
ENSG0000(	42.81198	0.007184	0.05092	0.141091	0.887798	0.920106	RP13-20L	antisense
ENSG0000(	42.79697	-0.62549	0.108551	-5.76212	8.31E-09	4.66E-08	snoU13	lincRNA
ENSG0000(	42.79054	0.552638	0.097402	5.673788	1.40E-08	7.58E-08	ESR2	protein_cc
ENSG0000(	42.78137	-0.912	0.114744	-7.9481	1.89E-15	3.13E-14	CTD-2555	lincRNA
ENSG0000(	42.76514	-0.62833	0.085618	-7.33878	2.16E-13	2.47E-12	RP11-38L	antisense
ENSG0000(	42.75892	0.048254	0.063486	0.760082	0.447206	0.543948	ZNF252P-	antisense
ENSG0000(	42.74453	0.726259	0.103644	7.007242	2.43E-12	2.38E-11	ELF3	protein_cc
ENSG0000(	42.7377	0.206699	0.081347	2.540942	0.011055	0.021873	AC062017	protein_cc
ENSG0000(	42.73104	-0.17108	0.072904	-2.34667	0.018942	0.03545	RPS10P7	lincRNA
ENSG0000(	42.71782	-0.04654	0.065112	-0.71472	0.474783	0.570656	MIR3936	miRNA
ENSG0000(	42.71433	0.257489	0.094437	2.726574	0.0064	0.013331	TEKT3	protein_cc
ENSG0000(	42.67392	0.873837	0.109969	7.946206	1.92E-15	3.17E-14	LEMD1	protein_cc
ENSG0000(	42.607	-0.28748	0.086662	-3.31724	0.000909	0.002232	RP11-445	antisense
ENSG0000(	42.59909	-0.70121	0.175115	-4.00428	6.22E-05	0.000189	RP11-469	lincRNA
ENSG0000(	42.5848	1.267807	0.146937	8.628259	6.23E-18	1.54E-16	RPS10P3	pseudoge
ENSG0000(	42.57719	-0.05212	0.062769	-0.83033	0.406352	0.503112	C2ORF15	protein_cc
ENSG0000(	42.55985	-0.46372	0.13397	-3.4614	0.000537	0.001378	NKX1-2	protein_cc
ENSG0000(	42.55035	0.179265	0.050453	3.553134	0.000381	0.001005	RP11-502	lincRNA
ENSG0000(	42.5398	0.434872	0.122868	3.539355	0.000401	0.001055	RP11-686	lincRNA
ENSG0000(	42.50766	0.571476	0.075298	7.589574	3.21E-14	4.24E-13	RP4-758J1	lincRNA
ENSG0000(	42.50178	0.000449	0.068883	0.006514	0.994803	0.996669	RP11-346	lincRNA
ENSG0000(	42.43972	0.268132	0.121013	2.215734	0.02671	0.048346	DMRTA1	protein_cc
ENSG0000(	42.43711	-0.06111	0.06518	-0.93753	0.348486	0.443964	LL0XNC01	lincRNA
ENSG0000(	42.43494	0.089358	0.059504	1.501713	0.133171	0.197424	RP11-173	pseudoge
ENSG0000(	42.4295	0.278666	0.111379	2.501969	0.01235	0.024152	RP11-4B1	lincRNA
ENSG0000(	42.41101	0.825343	0.119059	6.932224	4.14E-12	3.91E-11	EEF1A1P1	pseudoge
ENSG0000(	42.39659	-0.3498	0.097084	-3.60311	0.000314	0.000843	ENPP7P4	pseudoge
ENSG0000(	42.39109	0.826682	0.103573	7.981627	1.44E-15	2.43E-14	CTD-2033	pseudoge
ENSG0000(	42.37732	-0.12951	0.069627	-1.86001	0.062884	0.10321	CSPG4P12	pseudoge
ENSG0000(	42.37445	-0.7513	0.1386	-5.42059	5.94E-08	2.92E-07	MCTP2	protein_cc
ENSG0000(	42.36881	-1.37248	0.138367	-9.91912	3.44E-23	2.09E-21	ZNF831	protein_cc
ENSG0000(	42.36714	-0.53995	0.086791	-6.22129	4.93E-10	3.37E-09	WBP2NL	protein_cc
ENSG0000(	42.34068	0.076258	0.052358	1.456478	0.145261	0.212792	APITD1-C	protein_cc
ENSG0000(	42.25069	-0.79411	0.132945	-5.97322	2.33E-09	1.43E-08	CPB1	protein_cc
ENSG0000(	42.21679	-0.47129	0.112102	-4.20412	2.62E-05	8.44E-05	RP4-719C	antisense
ENSG0000(	42.21011	0.209553	0.061285	3.419339	0.000628	0.001589	IMPG2	protein_cc
ENSG0000(	42.18099	0.708912	0.14397	4.924041	8.48E-07	3.46E-06	ROPN1L	protein_cc
ENSG0000(	42.14508	0.312221	0.074507	4.190485	2.78E-05	8.93E-05	CITF22-92	lincRNA
ENSG0000(	42.13903	-0.34894	0.054249	-6.43215	1.26E-10	9.46E-10	CYP2T2P	pseudoge
ENSG0000(	42.13704	-0.5619	0.104759	-5.3638	8.15E-08	3.91E-07	RP11-234	antisense
ENSG0000(	42.09736	-0.14371	0.054301	-2.64647	0.008134	0.016588	XXbac-B4	sense_intr
ENSG0000(	42.09061	0.034518	0.054152	0.637433	0.523843	0.615799	RP11-299	lincRNA
ENSG0000(	42.08939	-0.40443	0.103741	-3.89851	9.68E-05	0.000284	AC007375	protein_cc
ENSG0000(	42.08435	-0.55337	0.082752	-6.68707	2.28E-11	1.91E-10	GSN-AS1	antisense
ENSG0000(	42.05444	0.128213	0.047551	2.696324	0.007011	0.014483	RP11-50C	antisense
ENSG0000(	42.0485	-0.66793	0.111074	-6.01341	1.82E-09	1.14E-08	TIGIT	protein_cc
ENSG0000(	42.04494	-0.13334	0.082965	-1.60718	0.108014	0.16508	MT-TY	Mt_tRNA
ENSG0000(	42.03058	-0.05082	0.039643	-1.28184	0.199899	0.279168	AC105760	antisense
ENSG0000(	42.01884	0.17779	0.109673	1.621087	0.104999	0.161125	IL11	protein_cc
ENSG0000(	42.01445	0.598385	0.164287	3.642315	0.00027	0.000733	CLEC12A	protein_cc
ENSG0000(	42.01262	0.147781	0.045912	3.218809	0.001287	0.003075	CTD-3099	sense_intr
ENSG0000(	42.00116	-0.28964	0.081018	-3.57506	0.00035	0.000931	NKPD1	protein_cc
ENSG0000(	41.91526	-0.16869	0.08274	-2.0388	0.04147	0.071633	ASAH2	protein_cc

ENSG0000(	41.91424	-0.12313	0.081742	-1.50629	0.131992	0.195969	RASA4DP	pseudoge
ENSG0000(	41.90884	0.123104	0.072323	1.702148	0.088728	0.139301	RP11-513	lincRNA
ENSG0000(	41.89634	-0.03141	0.050614	-0.62061	0.534857	0.626163	UBE2FP3	pseudoge
ENSG0000(	41.89126	0.806599	0.106518	7.572399	3.66E-14	4.80E-13	RPSAP54	pseudoge
ENSG0000(	41.88281	1.86929	0.200433	9.32625	1.10E-20	4.20E-19	FEZF1	protein_cc
ENSG0000(	41.88274	1.449863	0.292248	4.961073	7.01E-07	2.90E-06	HOXC9	protein_cc
ENSG0000(	41.87983	-0.02426	0.073389	-0.33059	0.740956	0.804389	RP11-173	pseudoge
ENSG0000(	41.86393	-0.02657	0.058248	-0.45607	0.648339	0.727218	RP11-407	antisense
ENSG0000(	41.86108	0.422929	0.068618	6.163499	7.12E-10	4.75E-09	MCF2L-AS	antisense
ENSG0000(	41.85355	-0.24222	0.110359	-2.19488	0.028172	0.050673	DNAH14	protein_cc
ENSG0000(	41.84938	-0.17265	0.085879	-2.01033	0.044396	0.076041	RP13-39P	antisense
ENSG0000(	41.84616	0.230882	0.120266	1.919761	0.054888	0.09169	ESPN	protein_cc
ENSG0000(	41.84323	-0.27524	0.234333	-1.17457	0.240169	0.325502	IGLC3	IG_C_gene
ENSG0000(	41.84232	0.002454	0.077466	0.031674	0.974732	0.98228	HPDL	protein_cc
ENSG0000(	41.84148	-0.20823	0.053102	-3.92133	8.81E-05	0.00026	SEC63P1	pseudoge
ENSG0000(	41.81456	0.818226	0.167305	4.890623	1.01E-06	4.06E-06	SPAG17	protein_cc
ENSG0000(	41.7726	0.43147	0.149875	2.878861	0.003991	0.008684	OSTN	protein_cc
ENSG0000(	41.75987	-0.22274	0.092953	-2.39629	0.016562	0.031464	FGF20	protein_cc
ENSG0000(	41.7441	0.267594	0.077331	3.460363	0.000539	0.001383	LEKR1	protein_cc
ENSG0000(	41.73795	0.544337	0.177251	3.071003	0.002133	0.004896	CXCL13	protein_cc
ENSG0000(	41.69399	-0.3955	0.08839	-4.47446	7.66E-06	2.70E-05	VSIG2	protein_cc
ENSG0000(	41.68408	-1.29289	0.142625	-9.065	1.25E-19	4.02E-18	AC011524	lincRNA
ENSG0000(	41.65748	0.440628	0.082938	5.312764	1.08E-07	5.10E-07	ABCC6P1	pseudoge
ENSG0000(	41.65541	-0.10502	0.072302	-1.4525	0.146363	0.214169	FAM53A	protein_cc
ENSG0000(	41.60622	-0.12596	0.061973	-2.03242	0.042111	0.072605	NAPSA	protein_cc
ENSG0000(	41.58626	-0.03757	0.087244	-0.43063	0.666735	0.742775	AC024937	pseudoge
ENSG0000(	41.58527	0.668876	0.156355	4.277933	1.89E-05	6.24E-05	ULBP1	protein_cc
ENSG0000(	41.58185	0.397174	0.07423	5.350556	8.77E-08	4.19E-07	RPL7P23	pseudoge
ENSG0000(	41.56826	0.193657	0.058759	3.295761	0.000982	0.002394	RP5-968P	antisense
ENSG0000(	41.56243	-0.03249	0.088658	-0.36641	0.714056	0.781978	RP11-106	processed
ENSG0000(	41.54733	0.370262	0.083326	4.44356	8.85E-06	3.08E-05	LINC0056!	lincRNA
ENSG0000(	41.53868	0.16058	0.056108	2.861959	0.00421	0.009119	RP11-442	lincRNA
ENSG0000(	41.49638	-0.14135	0.072335	-1.95409	0.050691	0.085523	C3orf72	protein_cc
ENSG0000(	41.48833	-0.00202	0.065101	-0.03097	0.975291	0.982651	LINC0106:	antisense
ENSG0000(	41.48551	-0.83321	0.140021	-5.95058	2.67E-09	1.63E-08	CTC-458G	lincRNA
ENSG0000(	41.46936	1.023822	0.137721	7.434046	1.05E-13	1.28E-12	RP11-678	lincRNA
ENSG0000(	41.43985	-0.25947	0.083735	-3.09875	0.001943	0.004492	LSMEM2	protein_cc
ENSG0000(	41.42679	0.155003	0.047238	3.28131	0.001033	0.002511	OLA1P1	pseudoge
ENSG0000(	41.42555	0.256401	0.056572	4.532308	5.83E-06	2.09E-05	KDM4A-A	antisense
ENSG0000(	41.42164	1.116667	0.187065	5.969423	2.38E-09	1.46E-08	HILS1	pseudoge
ENSG0000(	41.41392	-0.83593	0.089718	-9.31725	1.19E-20	4.53E-19	LINC0114(	lincRNA
ENSG0000(	41.40822	-0.25139	0.078816	-3.18964	0.001425	0.003377	AC006129	lincRNA
ENSG0000(	41.3837	0.112207	0.068265	1.643699	0.100238	0.154842	RP11-524	antisense
ENSG0000(	41.37502	0.343491	0.062932	5.458132	4.81E-08	2.40E-07	CTD-2369	protein_cc
ENSG0000(	41.35761	0.196986	0.059711	3.298977	0.00097	0.002369	RP11-154.	antisense
ENSG0000(	41.35586	-0.62162	0.093279	-6.66406	2.66E-11	2.22E-10	USP44	protein_cc
ENSG0000(	41.32869	0.043443	0.081214	0.534922	0.592704	0.678215	CD81-AS1	processed
ENSG0000(	41.27096	0.322455	0.050673	6.363428	1.97E-10	1.43E-09	RP11-634	antisense
ENSG0000(	41.26835	0.161116	0.051652	3.119266	0.001813	0.004216	AC012442	3prime_ov
ENSG0000(	41.2674	-0.09839	0.078103	-1.25976	0.207755	0.288424	CTD-2383	antisense
ENSG0000(	41.25041	-0.0709	0.113016	-0.62731	0.530453	0.622148	C3orf67	protein_cc
ENSG0000(	41.24975	-0.47304	0.079562	-5.9455	2.76E-09	1.67E-08	IGF1	protein_cc
ENSG0000(	41.24668	1.046369	0.129316	8.091583	5.89E-16	1.06E-14	CTD-3035	pseudoge
ENSG0000(	41.24173	0.603394	0.098477	6.127249	8.94E-10	5.88E-09	RP11-592	pseudoge
ENSG0000(	41.23914	0.597981	0.072567	8.240448	1.72E-16	3.36E-15	FANCB	protein_cc
ENSG0000(	41.22011	-0.15886	0.077467	-2.05063	0.040303	0.069857	LINC0064!	antisense
ENSG0000(	41.20203	-0.5837	0.099359	-5.87467	4.24E-09	2.49E-08	HTR7	protein_cc
ENSG0000(	41.19801	-0.42279	0.076916	-5.49685	3.87E-08	1.96E-07	RP11-283	antisense
ENSG0000(	41.17358	0.320404	0.055375	5.786059	7.21E-09	4.09E-08	PRRG2	protein_cc

ENSG0000(	41.16941	0.195719	0.078167	2.503857	0.012285	0.024038	RP11-191	lincRNA
ENSG0000(	41.16108	-0.72304	0.110318	-6.55418	5.59E-11	4.42E-10	DMBT1	protein_cc
ENSG0000(	41.14055	-0.69403	0.119145	-5.82506	5.71E-09	3.29E-08	FAM19A4	protein_cc
ENSG0000(	41.1373	-0.16373	0.083644	-1.95746	0.050293	0.084936	SFTA1P	lincRNA
ENSG0000(	41.13404	0.380948	0.073462	5.185667	2.15E-07	9.67E-07	RP4-673M	antisense
ENSG0000(	41.12094	-0.52971	0.06812	-7.7761	7.48E-15	1.12E-13	LINC0113!	lincRNA
ENSG0000(	41.10197	-0.49026	0.080623	-6.08086	1.20E-09	7.71E-09	YWHAZP5	pseudoge
ENSG0000(	41.10135	-0.1478	0.075761	-1.95087	0.051072	0.086098	LINC0085	processed
ENSG0000(	41.09798	0.936649	0.105083	8.913397	4.95E-19	1.44E-17	HMGN2P3	pseudoge
ENSG0000(	41.07112	-0.04664	0.055634	-0.83842	0.401795	0.49858	DPY19L1P	pseudoge
ENSG0000(	41.07046	-1.39036	0.167679	-8.29179	1.12E-16	2.25E-15	CRH	protein_cc
ENSG0000(	41.04064	-0.03788	0.064843	-0.58413	0.559134	0.648149	TLE1P1	pseudoge
ENSG0000(	40.97658	-0.10632	0.069682	-1.52587	0.127043	0.189776	ENPP3	protein_cc
ENSG0000(	40.93468	-0.01325	0.057631	-0.22994	0.818138	0.866651	PTGES3L	protein_cc
ENSG0000(	40.92727	0.671697	0.109669	6.124741	9.08E-10	5.96E-09	RP11-603.	pseudoge
ENSG0000(	40.91625	0.44232	0.072077	6.136796	8.42E-10	5.55E-09	RP11-545	antisense
ENSG0000(	40.9107	-0.03182	0.078984	-0.40282	0.687079	0.759861	RP5-826L	lincRNA
ENSG0000(	40.90895	0.213276	0.061632	3.460485	0.000539	0.001383	CTC-444N	antisense
ENSG0000(	40.90649	-0.04786	0.130355	-0.36714	0.713513	0.781529	RNASE3	protein_cc
ENSG0000(	40.90328	0.062436	0.034719	1.798325	0.072126	0.116326	ZNF564	protein_cc
ENSG0000(	40.90113	0.883759	0.116357	7.595254	3.07E-14	4.08E-13	RP1-292B	pseudoge
ENSG0000(	40.88794	0.816115	0.088431	9.228812	2.74E-20	9.71E-19	FBXO43	protein_cc
ENSG0000(	40.88321	0.382902	0.094235	4.063292	4.84E-05	0.000149	INTS4L1	pseudoge
ENSG0000(	40.87294	1.434922	0.169949	8.443256	3.09E-17	6.83E-16	HOXA1	protein_cc
ENSG0000(	40.86222	1.005085	0.134305	7.483572	7.23E-14	9.04E-13	GAPDHP4	pseudoge
ENSG0000(	40.81229	0.370952	0.154868	2.395273	0.016608	0.031542	AE000661	processed
ENSG0000(	40.78948	0.703482	0.107186	6.563222	5.27E-11	4.18E-10	EEF1A1P1	pseudoge
ENSG0000(	40.76843	0.66973	0.095507	7.01235	2.34E-12	2.30E-11	CNTD2	protein_cc
ENSG0000(	40.74022	-0.71076	0.101822	-6.98046	2.94E-12	2.85E-11	JAZF1-AS	antisense
ENSG0000(	40.73256	0.37062	0.133261	2.781153	0.005417	0.011464	SLC18A1	protein_cc
ENSG0000(	40.71756	-0.05699	0.13241	-0.43038	0.666916	0.742937	EBF2	protein_cc
ENSG0000(	40.69446	0.059461	0.058736	1.012355	0.311369	0.404649	AC009950	antisense
ENSG0000(	40.63768	0.252737	0.115102	2.195764	0.028109	0.050566	MYO3B	protein_cc
ENSG0000(	40.63422	1.224267	0.178885	6.84389	7.71E-12	6.97E-11	RP11-300	lincRNA
ENSG0000(	40.63191	0.593904	0.146215	4.061847	4.87E-05	0.00015	GAS2L2	protein_cc
ENSG0000(	40.61306	0.030107	0.07335	0.410453	0.681474	0.754778	MIR647	miRNA
ENSG0000(	40.58344	0.390254	0.188802	2.067007	0.038733	0.067432	LINC0064	lincRNA
ENSG0000(	40.5793	0.158801	0.059245	2.680404	0.007353	0.015135	SRRM5	protein_cc
ENSG0000(	40.55322	-0.65192	0.098478	-6.61999	3.59E-11	2.93E-10	RP11-445	antisense
ENSG0000(	40.55237	0.429067	0.071448	6.005303	1.91E-09	1.19E-08	ESRRAP2	pseudoge
ENSG0000(	40.54652	0.11929	0.067202	1.775092	0.075883	0.121557	RP1-199J3	pseudoge
ENSG0000(	40.53051	0.272584	0.1263	2.158234	0.03091	0.055092	RP11-320	pseudoge
ENSG0000(	40.5158	-0.61282	0.094202	-6.50538	7.75E-11	6.01E-10	RP1-232L	lincRNA
ENSG0000(	40.46173	-0.12424	0.09297	-1.33632	0.181444	0.257016	ITGB7	protein_cc
ENSG0000(	40.45827	-0.01282	0.08894	-0.14418	0.885356	0.918293	ROPN1B	protein_cc
ENSG0000(	40.43341	0.100353	0.048739	2.058996	0.039495	0.068609	NAP1L1P1	pseudoge
ENSG0000(	40.41346	1.436162	0.171271	8.385341	5.06E-17	1.08E-15	HOXB-AS	antisense
ENSG0000(	40.39968	-0.26947	0.12142	-2.21929	0.026467	0.047953	SLC25A21	protein_cc
ENSG0000(	40.36003	0.083948	0.053911	1.557153	0.119434	0.179985	LINC0047	processed
ENSG0000(	40.3457	0.610372	0.085155	7.167776	7.62E-13	8.07E-12	RP11-312.	pseudoge
ENSG0000(	40.3292	-0.44314	0.103747	-4.27131	1.94E-05	6.41E-05	LRG1	protein_cc
ENSG0000(	40.31896	0.106047	0.088702	1.195537	0.231877	0.316035	SNORD10	snoRNA
ENSG0000(	40.28893	0.540648	0.130236	4.151287	3.31E-05	0.000105	RP11-380	pseudoge
ENSG0000(	40.2821	0.212655	0.094607	2.247778	0.02459	0.044866	MT-TC	Mt_tRNA
ENSG0000(	40.27413	0.575407	0.07315	7.866119	3.66E-15	5.76E-14	CDC20P1	pseudoge
ENSG0000(	40.26514	0.450496	0.168354	2.675881	0.007453	0.015321	MNX1	protein_cc
ENSG0000(	40.24981	-0.48934	0.22483	-2.17649	0.029519	0.05288	RP11-424	lincRNA
ENSG0000(	40.24786	-0.10869	0.086316	-1.25924	0.207943	0.288647	CCDC147	protein_cc
ENSG0000(	40.21842	0.80627	0.099655	8.090611	5.94E-16	1.06E-14	SETP14	pseudoge

ENSG0000(	40.21582	0.031234	0.07236	0.431649	0.665996	0.742194	WI2-2942.	lincRNA
ENSG0000(	40.20754	0.261601	0.094559	2.766536	0.005666	0.011933	MYBPHL	protein_cc
ENSG0000(	40.19899	-1.1949	0.139082	-8.59139	8.59E-18	2.08E-16	RP11-433.	lincRNA
ENSG0000(	40.19386	0.668285	0.089143	7.496758	6.54E-14	8.24E-13	AD000092	pseudoge
ENSG0000(	40.18727	-0.13114	0.072384	-1.81171	0.070031	0.113365	AL359878	protein_cc
ENSG0000(	40.17061	0.04443	0.077697	0.571843	0.567429	0.65542	DND1P1	pseudoge
ENSG0000(	40.17037	0.265331	0.050716	5.231723	1.68E-07	7.68E-07	RP11-7F1{	lincRNA
ENSG0000(	40.12029	-0.03672	0.107861	-0.3404	0.733557	0.798106	RP11-24F:	antisense
ENSG0000(	40.11534	0.22692	0.069925	3.245218	0.001174	0.002822	RP1-104C	lincRNA
ENSG0000(	40.10646	0.138132	0.077084	1.791973	0.073137	0.117783	A1BG	protein_cc
ENSG0000(	40.06284	0.130417	0.073116	1.783703	0.074472	0.119613	RP11-317	lincRNA
ENSG0000(	40.03714	-0.17993	0.138774	-1.29659	0.194771	0.273026	CR1	protein_cc
ENSG0000(	40.03705	0.142814	0.063001	2.266848	0.023399	0.042888	IDH1-AS1	antisense
ENSG0000(	40.03064	0.798099	0.097504	8.185295	2.72E-16	5.14E-15	FAM159A	protein_cc
ENSG0000(	40.01567	0.428894	0.068294	6.280112	3.38E-10	2.37E-09	RP11-446	pseudoge
ENSG0000(	39.98068	-0.22923	0.08042	-2.85045	0.004366	0.009415	RP11-88H	sense_intr
ENSG0000(	39.96578	-0.0611	0.057854	-1.05604	0.290948	0.38245	CTD-2619	lincRNA
ENSG0000(	39.91658	-0.66359	0.079037	-8.39597	4.62E-17	9.92E-16	PLEKHD1	protein_cc
ENSG0000(	39.91133	-0.67249	0.075891	-8.86117	7.92E-19	2.23E-17	RP11-326	lincRNA
ENSG0000(	39.91106	-0.13146	0.067819	-1.93834	0.052582	0.088303	U52111.1	antisense
ENSG0000(	39.90669	0.04971	0.091966	0.540524	0.588836	0.674915	PCDHB19I	pseudoge
ENSG0000(	39.88866	0.186825	0.063268	2.952914	0.003148	0.006986	FAAH2	protein_cc
ENSG0000(	39.88203	-0.05292	0.10618	-0.49844	0.618174	0.701018	LINC0057	lincRNA
ENSG0000(	39.87159	0.901253	0.130994	6.880106	5.98E-12	5.50E-11	EIF3CL	protein_cc
ENSG0000(	39.85381	0.109197	0.082544	1.322903	0.185868	0.262334	SMIM2-AS	antisense
ENSG0000(	39.85211	0.57798	0.078588	7.354537	1.92E-13	2.22E-12	FAM72B	protein_cc
ENSG0000(	39.8418	-0.67858	0.096278	-7.0481	1.81E-12	1.82E-11	TDGF1	protein_cc
ENSG0000(	39.81903	-0.41933	0.067526	-6.2098	5.31E-10	3.60E-09	RP11-61N	pseudoge
ENSG0000(	39.81037	0.229526	0.087073	2.636008	0.008389	0.017056	RP11-174	sense_ove
ENSG0000(	39.79504	-0.55638	0.102559	-5.42497	5.80E-08	2.85E-07	C19orf77	protein_cc
ENSG0000(	39.79126	1.040098	0.125298	8.300966	1.03E-16	2.09E-15	RPL5P4	pseudoge
ENSG0000(	39.78292	0.123269	0.097747	1.261095	0.207275	0.287855	PRR15	protein_cc
ENSG0000(	39.77478	0.230567	0.04412	5.225948	1.73E-07	7.90E-07	HCG25	antisense
ENSG0000(	39.76482	0.545657	0.062196	8.77314	1.74E-18	4.66E-17	IPO9-AS1	antisense
ENSG0000(	39.7363	-1.28975	0.135056	-9.54974	1.30E-21	5.89E-20	GNG13	protein_cc
ENSG0000(	39.71688	-0.05821	0.08677	-0.6709	0.502285	0.596149	RP11-760	lincRNA
ENSG0000(	39.71225	-0.22024	0.047681	-4.61895	3.86E-06	1.42E-05	ZRANB2-/	processed
ENSG0000(	39.67129	0.401368	0.131379	3.055034	0.00225	0.005143	ADM2	protein_cc
ENSG0000(	39.66625	1.126916	0.148108	7.60872	2.77E-14	3.70E-13	GAPDHP6	pseudoge
ENSG0000(	39.64213	-0.394	0.082617	-4.769	1.85E-06	7.18E-06	LINC0029{	lincRNA
ENSG0000(	39.61783	0.245064	0.125406	1.954162	0.050682	0.085511	IFI30	protein_cc
ENSG0000(	39.61516	0.337921	0.097491	3.466164	0.000528	0.001356	GPR64	protein_cc
ENSG0000(	39.61063	0.144107	0.070046	2.057304	0.039657	0.06887	RP11-112.	lincRNA
ENSG0000(	39.58626	0.468524	0.065133	7.19329	6.32E-13	6.77E-12	CTB-113I2	lincRNA
ENSG0000(	39.57043	0.651175	0.09527	6.835069	8.20E-12	7.38E-11	RP11-848	pseudoge
ENSG0000(	39.55592	-0.17579	0.061683	-2.84995	0.004373	0.009429	RP5-1050	antisense
ENSG0000(	39.5217	-0.45348	0.1634	-2.77528	0.005515	0.011649	11-Mar	protein_cc
ENSG0000(	39.49913	-0.36538	0.087965	-4.15365	3.27E-05	0.000104	RP11-463	lincRNA
ENSG0000(	39.49028	1.943355	0.226017	8.598283	8.09E-18	1.97E-16	FABP5P7	pseudoge
ENSG0000(	39.48302	-0.284	0.081825	-3.47087	0.000519	0.001334	KLRD1	protein_cc
ENSG0000(	39.47663	0.404718	0.076711	5.27591	1.32E-07	6.14E-07	RP11-680.	antisense
ENSG0000(	39.4599	-0.30523	0.122221	-2.49731	0.012514	0.024439	AL450992	antisense
ENSG0000(	39.42155	0.89131	0.1282	6.952495	3.59E-12	3.42E-11	RPL37AP1	pseudoge
ENSG0000(	39.41954	0.484993	0.061736	7.85595	3.97E-15	6.21E-14	RP11-276	lincRNA
ENSG0000(	39.41331	-0.32478	0.057681	-5.63068	1.80E-08	9.58E-08	RP11-324	pseudoge
ENSG0000(	39.3959	-0.17055	0.082948	-2.05609	0.039774	0.069039	KIAA0408	protein_cc
ENSG0000(	39.39176	0.423415	0.08871	4.773032	1.81E-06	7.04E-06	RAB17	protein_cc
ENSG0000(	39.39156	-0.01639	0.046366	-0.35343	0.723767	0.789816	CTC-471F	pseudoge
ENSG0000(	39.36256	-0.04148	0.076699	-0.54085	0.588612	0.674697	STARD5	protein_cc

ENSG0000(	39.36118	0.482765	0.117548	4.10698	4.01E-05	0.000125	RP5-1024	antisense
ENSG0000(	39.34047	0.777524	0.15934	4.879666	1.06E-06	4.27E-06	NKX3-2	protein_cc
ENSG0000(	39.31716	0.170226	0.076166	2.234939	0.025421	0.046229	CTD-2035	lincRNA
ENSG0000(	39.31482	-0.49129	0.062596	-7.8485	4.21E-15	6.56E-14	YWHAZP4	pseudoge
ENSG0000(	39.31146	0.821349	0.238252	3.447389	0.000566	0.001446	HOXA11-/	antisense
ENSG0000(	39.30655	-0.10212	0.135983	-0.75094	0.452687	0.549348	KIAA0087	protein_cc
ENSG0000(	39.30524	-0.05405	0.107141	-0.50446	0.613935	0.697232	CD5	protein_cc
ENSG0000(	39.26683	-0.64671	0.1031	-6.27266	3.55E-10	2.48E-09	LINC0051!	lincRNA
ENSG0000(	39.25476	0.254526	0.156284	1.628614	0.103395	0.159013	MT1H	protein_cc
ENSG0000(	39.24973	0.239831	0.099951	2.39949	0.016418	0.031225	ACRV1	protein_cc
ENSG0000(	39.2497	-0.24494	0.065638	-3.73173	0.00019	0.000529	RP4-789D	lincRNA
ENSG0000(	39.23009	0.16456	0.055755	2.951454	0.003163	0.007015	LA16c-31:	processed
ENSG0000(	39.22	-0.00637	0.099345	-0.06416	0.948841	0.964627	BMS1P10	pseudoge
ENSG0000(	39.18259	-0.53698	0.098523	-5.45025	5.03E-08	2.50E-07	LINC0020:	lincRNA
ENSG0000(	39.17676	-0.09435	0.052861	-1.78489	0.07428	0.119337	C11orf65	protein_cc
ENSG0000(	39.17513	-1.34473	0.164983	-8.15072	3.62E-16	6.71E-15	EIF4E1B	protein_cc
ENSG0000(	39.16223	-0.00023	0.094346	-0.00242	0.998072	0.998823	RP11-146	protein_cc
ENSG0000(	39.15556	0.635891	0.077855	8.16759	3.15E-16	5.89E-15	CTD-2031	pseudoge
ENSG0000(	39.13725	0.691389	0.084217	8.209596	2.22E-16	4.27E-15	ABCA12	protein_cc
ENSG0000(	39.1035	-0.34816	0.08885	-3.91855	8.91E-05	0.000263	SPATA22	protein_cc
ENSG0000(	39.08319	-0.07134	0.122765	-0.58113	0.561154	0.650012	KLHDC7B	protein_cc
ENSG0000(	39.06864	-0.14558	0.115006	-1.26586	0.205564	0.285845	PAQR9	protein_cc
ENSG0000(	39.06159	0.105095	0.057369	1.831925	0.066963	0.109004	RP11-680	pseudoge
ENSG0000(	39.05586	-0.3739	0.084062	-4.44796	8.67E-06	3.02E-05	AC110619	protein_cc
ENSG0000(	39.0479	-1.42803	0.145719	-9.7999	1.13E-22	6.16E-21	PABPC1L2	protein_cc
ENSG0000(	39.04329	0.786148	0.109138	7.20325	5.88E-13	6.33E-12	EEF1A1P2	pseudoge
ENSG0000(	39.02775	0.017795	0.109603	0.162356	0.871026	0.907297	AC005336	pseudoge
ENSG0000(	39.01224	-0.03705	0.070008	-0.52923	0.596645	0.681886	MEIG1	protein_cc
ENSG0000(	39.01223	0.066817	0.08464	0.789426	0.429863	0.526811	SH2D7	protein_cc
ENSG0000(	38.98923	0.110836	0.052916	2.094562	0.03621	0.063531	RP5-1112	antisense
ENSG0000(	38.95752	1.418182	0.13664	10.37896	3.09E-25	2.83E-23	SNRPEP4	pseudoge
ENSG0000(	38.94872	0.212139	0.127117	1.668856	0.095146	0.147982	C2orf62	protein_cc
ENSG0000(	38.93239	-0.02481	0.133567	-0.18578	0.852618	0.893418	KRT8	protein_cc
ENSG0000(	38.90937	-0.00713	0.049807	-0.14315	0.88617	0.918882	RP11-158	lincRNA
ENSG0000(	38.87787	0.261455	0.103749	2.520076	0.011733	0.023075	PTGIR	protein_cc
ENSG0000(	38.87631	0.121684	0.044545	2.731744	0.0063	0.013141	RP11-111	pseudoge
ENSG0000(	38.87245	0.275708	0.06109	4.513172	6.39E-06	2.27E-05	RPS4XP16	pseudoge
ENSG0000(	38.86869	-0.00451	0.122769	-0.03673	0.970703	0.979684	MIMT1	lincRNA
ENSG0000(	38.8664	0.282041	0.095662	2.948322	0.003195	0.007081	OR7E14P	pseudoge
ENSG0000(	38.86527	0.215606	0.06486	3.324192	0.000887	0.002182	GS1-124K	pseudoge
ENSG0000(	38.84537	1.303494	0.183559	7.101245	1.24E-12	1.27E-11	TCF23	protein_cc
ENSG0000(	38.83288	0.269861	0.052543	5.136022	2.81E-07	1.24E-06	AC144449	antisense
ENSG0000(	38.82474	-0.28293	0.096979	-2.91745	0.003529	0.007755	RP11-196	antisense
ENSG0000(	38.81582	0.336092	0.147355	2.280837	0.022558	0.04152	CXorf64	protein_cc
ENSG0000(	38.81311	1.237757	0.107802	11.48172	1.63E-30	4.24E-28	PCDHB1	protein_cc
ENSG0000(	38.80723	0.13351	0.110111	1.212507	0.225318	0.308661	HCAR1	protein_cc
ENSG0000(	38.80289	-0.13356	0.153401	-0.87065	0.383943	0.480623	SP9	protein_cc
ENSG0000(	38.78532	-0.1183	0.044852	-2.6376	0.008349	0.016986	FAM156A	protein_cc
ENSG0000(	38.77922	0.681677	0.095947	7.104756	1.21E-12	1.24E-11	H3F3C	protein_cc
ENSG0000(	38.77327	-0.19282	0.066873	-2.88339	0.003934	0.008572	AP001053	antisense
ENSG0000(	38.76381	-0.04051	0.051547	-0.78584	0.431961	0.528927	C20orf202	protein_cc
ENSG0000(	38.7637	0.328269	0.101743	3.226444	0.001253	0.003	MT1XP1	pseudoge
ENSG0000(	38.75956	0.969321	0.152775	6.344774	2.23E-10	1.61E-09	ESPNL	protein_cc
ENSG0000(	38.74655	-0.24715	0.079359	-3.11428	0.001844	0.004281	CTXN2	protein_cc
ENSG0000(	38.74319	0.504826	0.160003	3.155103	0.001604	0.003769	LRRIQ1	protein_cc
ENSG0000(	38.72868	-0.26003	0.075181	-3.45865	0.000543	0.001392	RP11-149	antisense
ENSG0000(	38.66356	-0.6346	0.094231	-6.73446	1.65E-11	1.41E-10	ACTRT3	protein_cc
ENSG0000(	38.66213	-0.25995	0.192439	-1.35082	0.176753	0.251385	DAW1	protein_cc
ENSG0000(	38.66117	0.731712	0.083816	8.729957	2.55E-18	6.68E-17	ETV2	protein_cc

ENSG0000(	38.62196	-0.08689	0.07109	-1.22222	0.221624	0.304395	RP11-329	lincRNA
ENSG0000(	38.5869	1.031697	0.144285	7.15039	8.65E-13	9.09E-12	GAPDHP7	pseudoge
ENSG0000(	38.54571	-0.29203	0.099076	-2.94754	0.003203	0.007097	RP11-34P	pseudoge
ENSG0000(	38.44382	0.315171	0.110323	2.856814	0.004279	0.00925	FTH1P5	pseudoge
ENSG0000(	38.4369	0.319473	0.060347	5.293927	1.20E-07	5.61E-07	DGUOK-A	antisense
ENSG0000(	38.42474	-0.515	0.075649	-6.80775	9.91E-12	8.81E-11	RP11-147	lincRNA
ENSG0000(	38.42223	-0.74906	0.109604	-6.8342	8.25E-12	7.42E-11	RP11-268	lincRNA
ENSG0000(	38.4042	0.673066	0.092809	7.252184	4.10E-13	4.52E-12	EEF1DP1	pseudoge
ENSG0000(	38.39171	1.176545	0.227926	5.161968	2.44E-07	1.09E-06	IDO1	protein_cc
ENSG0000(	38.34418	-0.11916	0.077006	-1.54735	0.121778	0.182954	RP11-101	lincRNA
ENSG0000(	38.34185	0.027967	0.057595	0.485579	0.627265	0.708964	RP11-472	pseudoge
ENSG0000(	38.32408	0.503109	0.095932	5.244414	1.57E-07	7.19E-07	RP11-783	antisense
ENSG0000(	38.30198	0.284728	0.05823	4.889722	1.01E-06	4.08E-06	FAM72A	protein_cc
ENSG0000(	38.29777	0.208997	0.092013	2.271376	0.023124	0.042457	RNF224	protein_cc
ENSG0000(	38.28538	0.079683	0.072181	1.103931	0.269623	0.358974	RP3-342P	pseudoge
ENSG0000(	38.26782	-0.23105	0.087894	-2.62877	0.00857	0.017385	AC090945	pseudoge
ENSG0000(	38.26616	-0.34845	0.07816	-4.45814	8.27E-06	2.89E-05	PPP1R26-	antisense
ENSG0000(	38.25534	0.006453	0.055575	0.116109	0.907566	0.934494	CTB-33O1	antisense
ENSG0000(	38.24988	0.569153	0.075147	7.573877	3.62E-14	4.75E-13	RRN3P2	pseudoge
ENSG0000(	38.23575	-0.09418	0.063914	-1.47351	0.140614	0.206898	RP11-186	antisense
ENSG0000(	38.21761	0.051285	0.050356	1.018445	0.308467	0.401563	RP11-642	pseudoge
ENSG0000(	38.21593	0.740765	0.121396	6.102033	1.05E-09	6.81E-09	RP11-195	antisense
ENSG0000(	38.21383	0.356092	0.106667	3.338335	0.000843	0.002083	C1RL-AS1	antisense
ENSG0000(	38.19434	0.075127	0.064771	1.15989	0.246094	0.332502	RP1-286D	antisense
ENSG0000(	38.17751	-0.01164	0.088966	-0.13086	0.895885	0.92606	PVRIG	protein_cc
ENSG0000(	38.17014	-0.51357	0.198827	-2.58299	0.009795	0.019607	CNN2P8	pseudoge
ENSG0000(	38.12955	-0.2421	0.071246	-3.39807	0.000679	0.001707	PROX2	protein_cc
ENSG0000(	38.12322	0.306566	0.06098	5.027353	4.97E-07	2.11E-06	RP11-752	pseudoge
ENSG0000(	38.12175	-0.04532	0.110227	-0.41119	0.680932	0.75434	RP11-174	lincRNA
ENSG0000(	38.06067	0.086538	0.045189	1.915001	0.055492	0.092571	ZACN	protein_cc
ENSG0000(	38.0537	-0.18258	0.06186	-2.9516	0.003161	0.007012	PITRM1-A	processed
ENSG0000(	38.05129	-0.13266	0.151385	-0.87632	0.380858	0.477434	HPN-AS1	antisense
ENSG0000(	38.01334	0.350399	0.089597	3.910813	9.20E-05	0.000271	TP53AIP1	protein_cc
ENSG0000(	37.98712	-0.11869	0.122897	-0.96573	0.334179	0.428832	SH2D2A	protein_cc
ENSG0000(	37.93709	-0.04055	0.074399	-0.54506	0.585713	0.67201	CHRNE	protein_cc
ENSG0000(	37.93475	-0.28714	0.059273	-4.84434	1.27E-06	5.05E-06	RP11-77K	pseudoge
ENSG0000(	37.9107	-0.98379	0.146108	-6.7333	1.66E-11	1.42E-10	RP11-807	lincRNA
ENSG0000(	37.86268	-0.63691	0.106677	-5.97043	2.37E-09	1.45E-08	FLT3	protein_cc
ENSG0000(	37.85348	0.175536	0.04236	4.143869	3.41E-05	0.000108	RP11-33C	lincRNA
ENSG0000(	37.84388	0.007173	0.121467	0.05905	0.952913	0.967451	MLPH	protein_cc
ENSG0000(	37.83104	-0.05088	0.233124	-0.21825	0.827233	0.873427	TLX1NB	protein_cc
ENSG0000(	37.82783	0.74342	0.088863	8.365946	5.96E-17	1.26E-15	PSME2P2	pseudoge
ENSG0000(	37.78279	0.139606	0.06713	2.079649	0.037558	0.065621	LINC0024	lincRNA
ENSG0000(	37.77541	-0.19846	0.115759	-1.71442	0.086451	0.136168	AL132709	lincRNA
ENSG0000(	37.72539	-0.89128	0.124859	-7.13827	9.45E-13	9.87E-12	HAS1	protein_cc
ENSG0000(	37.71008	-1.3484	0.150614	-8.95266	3.47E-19	1.04E-17	TRPC5	protein_cc
ENSG0000(	37.70881	0.769852	0.115823	6.6468	3.00E-11	2.47E-10	UBBP1	pseudoge
ENSG0000(	37.69312	-0.08814	0.134219	-0.65671	0.511365	0.604501	RP11-261	antisense
ENSG0000(	37.68963	0.900845	0.116762	7.71524	1.21E-14	1.73E-13	EEF1A1P3	pseudoge
ENSG0000(	37.67917	-0.20166	0.108191	-1.86391	0.062334	0.102402	RP11-341	antisense
ENSG0000(	37.67693	0.574469	0.081939	7.010889	2.37E-12	2.33E-11	YWHAEP5	pseudoge
ENSG0000(	37.676	-0.22555	0.108253	-2.08351	0.037204	0.065073	ITGB6	protein_cc
ENSG0000(	37.61138	0.217698	0.058693	3.709128	0.000208	0.000575	AC012358	antisense
ENSG0000(	37.60961	-0.21009	0.157957	-1.33002	0.183512	0.259446	ATP6V0A4	protein_cc
ENSG0000(	37.60864	-0.19914	0.073712	-2.70159	0.006901	0.014278	SPATA41	lincRNA
ENSG0000(	37.60435	-0.17896	0.061586	-2.9058	0.003663	0.008024	TPTE2P5	pseudoge
ENSG0000(	37.57635	-0.66715	0.158056	-4.22097	2.43E-05	7.88E-05	HSPB3	protein_cc
ENSG0000(	37.57142	0.110563	0.06873	1.608673	0.107688	0.164643	RP11-820	processed
ENSG0000(	37.56726	-0.16048	0.117911	-1.36107	0.173492	0.24753	FOXD2	protein_cc

ENSG0000(	37.54103	0.012438	0.070937	0.175335	0.860816	0.899556	RP11-585	antisense
ENSG0000(	37.53946	0.06519	0.078242	0.833174	0.404747	0.501621	RFPL3S	protein_cc
ENSG0000(	37.5292	0.63504	0.088643	7.164036	7.83E-13	8.28E-12	RP11-356	antisense
ENSG0000(	37.52018	-0.14973	0.111331	-1.34492	0.178652	0.253734	P4HA3	protein_cc
ENSG0000(	37.50642	1.362221	0.267421	5.093916	3.51E-07	1.52E-06	TFAP2B	protein_cc
ENSG0000(	37.50177	-0.25321	0.087941	-2.87935	0.003985	0.008671	SLC6A1-A	antisense
ENSG0000(	37.49824	0.14504	0.044019	3.294922	0.000984	0.0024	CKLF-CMT	protein_cc
ENSG0000(	37.49563	0.716098	0.109542	6.537188	6.27E-11	4.93E-10	C20orf201	protein_cc
ENSG0000(	37.48997	0.14217	0.154042	0.92293	0.356044	0.451647	FCER1A	protein_cc
ENSG0000(	37.47851	-0.24317	0.122265	-1.98888	0.046714	0.079541	RP11-161	lincRNA
ENSG0000(	37.46303	-1.12342	0.109431	-10.266	1.00E-24	8.33E-23	GLS2	protein_cc
ENSG0000(	37.46059	1.288277	0.125362	10.27648	8.99E-25	7.58E-23	RP11-299	lincRNA
ENSG0000(	37.44589	0.058119	0.079655	0.72963	0.465616	0.561541	RP11-147	antisense
ENSG0000(	37.43885	-0.51166	0.101444	-5.04377	4.56E-07	1.95E-06	RP11-707.	lincRNA
ENSG0000(	37.39065	-0.06453	0.114478	-0.56367	0.572978	0.660384	LAMC2	protein_cc
ENSG0000(	37.36813	-0.60748	0.075972	-7.99614	1.28E-15	2.18E-14	C1QTNF7	protein_cc
ENSG0000(	37.32874	0.027868	0.088747	0.314018	0.753507	0.814339	SLC34A3	protein_cc
ENSG0000(	37.31313	0.924609	0.112218	8.239365	1.73E-16	3.39E-15	RP3-375P	pseudoge
ENSG0000(	37.26132	-0.17686	0.099889	-1.7706	0.076627	0.122605	WISP2	protein_cc
ENSG0000(	37.23963	-0.06179	0.087942	-0.70257	0.482323	0.577357	GJC3	protein_cc
ENSG0000(	37.219	-0.46409	0.124326	-3.73285	0.000189	0.000527	AC138430	processed
ENSG0000(	37.2042	-0.24234	0.145555	-1.66497	0.095918	0.14903	ZNF98	protein_cc
ENSG0000(	37.18982	-0.66352	0.090397	-7.34004	2.14E-13	2.45E-12	ADAMTSL	protein_cc
ENSG0000(	37.17529	0.003412	0.117724	0.028982	0.976879	0.983696	TSLP	protein_cc
ENSG0000(	37.16568	0.251025	0.108759	2.308084	0.020994	0.038898	AP001046	lincRNA
ENSG0000(	37.13332	0.834206	0.100712	8.28308	1.20E-16	2.41E-15	RP11-464	pseudoge
ENSG0000(	37.10685	0.025958	0.068807	0.377259	0.705981	0.775548	RP11-256	antisense
ENSG0000(	37.09813	0.03947	0.048466	0.814389	0.415422	0.512452	CTD-2623	pseudoge
ENSG0000(	37.09557	0.521857	0.096415	5.412628	6.21E-08	3.04E-07	CTD-2126	sense_ove
ENSG0000(	37.04813	0.329005	0.109905	2.993544	0.002758	0.006186	AC144835	lincRNA
ENSG0000(	37.03743	1.490828	0.14464	10.30714	6.54E-25	5.61E-23	FAM19A3	protein_cc
ENSG0000(	37.02687	-0.25591	0.091221	-2.80533	0.005026	0.010709	PRKXP1	pseudoge
ENSG0000(	37.01695	0.089049	0.077962	1.14221	0.253367	0.340792	HMGN2P1	pseudoge
ENSG0000(	37.01531	-0.1505	0.087933	-1.71148	0.086992	0.136921	RP11-553.	protein_cc
ENSG0000(	37.0131	0.376146	0.091542	4.109009	3.97E-05	0.000124	RP11-598	antisense
ENSG0000(	37.00518	0.178134	0.219094	0.813052	0.416188	0.513157	IGHA2	IG_C_gene
ENSG0000(	37.00362	-0.24422	0.063254	-3.86087	0.000113	0.000327	RP11-407	pseudoge
ENSG0000(	36.96625	-0.36234	0.117035	-3.09603	0.001961	0.004532	RP11-54C	lincRNA
ENSG0000(	36.94512	0.052076	0.047292	1.101172	0.270822	0.360371	WASH4P	protein_cc
ENSG0000(	36.94485	1.280981	0.16828	7.612212	2.69E-14	3.61E-13	ISL2	protein_cc
ENSG0000(	36.91903	0.540827	0.121508	4.450975	8.55E-06	2.98E-05	FTH1P12	pseudoge
ENSG0000(	36.90078	0.822293	0.106671	7.708696	1.27E-14	1.81E-13	ATP5EP2	protein_cc
ENSG0000(	36.88803	0.037192	0.065334	0.569255	0.569183	0.657096	RPL17P50	pseudoge
ENSG0000(	36.87973	-0.21378	0.249063	-0.85836	0.390695	0.487291	IGKV3-20	IG_V_gene
ENSG0000(	36.85842	1.216911	0.134578	9.042427	1.53E-19	4.87E-18	RANP1	pseudoge
ENSG0000(	36.81202	0.992471	0.111922	8.867492	7.48E-19	2.12E-17	RP11-212	pseudoge
ENSG0000(	36.75728	-0.07508	0.073845	-1.01672	0.309286	0.402463	RP11-288	pseudoge
ENSG0000(	36.75417	0.276021	0.076244	3.620258	0.000294	0.000793	XXbac-B5	pseudoge
ENSG0000(	36.75201	0.17948	0.084002	2.136626	0.032628	0.057854	GRIK1-AS	antisense
ENSG0000(	36.7472	0.136155	0.065314	2.084619	0.037104	0.064925	CTD-2165	antisense
ENSG0000(	36.74201	0.221259	0.125599	1.761624	0.078133	0.124753	CTD-2325	antisense
ENSG0000(	36.73521	-0.45107	0.126979	-3.55231	0.000382	0.001008	RP11-85C	antisense
ENSG0000(	36.73237	0.222629	0.081158	2.743145	0.006085	0.012731	MIR17HG	processed
ENSG0000(	36.67363	0.122716	0.082428	1.488778	0.136546	0.201714	AP006621	antisense
ENSG0000(	36.6679	-0.01982	0.07811	-0.2538	0.79965	0.852004	RP11-575	antisense
ENSG0000(	36.66483	-0.25851	0.078769	-3.28189	0.001031	0.002506	CTD-2561	lincRNA
ENSG0000(	36.6202	-0.0111	0.056024	-0.19804	0.843012	0.886065	VN1R108F	pseudoge
ENSG0000(	36.61199	0.510437	0.139594	3.656573	0.000256	0.000696	MT1A	protein_cc
ENSG0000(	36.60361	0.409965	0.069667	5.884606	3.99E-09	2.36E-08	AC093106	pseudoge

ENSG0000(	36.52994	0.150276	0.060667	2.477077	0.013246	0.025738	AC093818	antisense
ENSG0000(	36.51791	0.268274	0.049644	5.403972	6.52E-08	3.18E-07	CTD-2007	lincRNA
ENSG0000(	36.51647	-0.02268	0.081698	-0.27765	0.781281	0.836921	RP11-196	lincRNA
ENSG0000(	36.50792	0.436539	0.078448	5.564724	2.63E-08	1.36E-07	AP000459	lincRNA
ENSG0000(	36.50602	-0.08607	0.078874	-1.09122	0.275174	0.365241	CCDC110	protein_cc
ENSG0000(	36.45878	0.184214	0.070727	2.604589	0.009198	0.018527	HCG15	antisense
ENSG0000(	36.44431	0.118665	0.217775	0.544895	0.585826	0.672102	SKOR2	protein_cc
ENSG0000(	36.44279	0.284466	0.071214	3.9945	6.48E-05	0.000196	RP11-312	antisense
ENSG0000(	36.41751	-0.14225	0.073779	-1.92812	0.053841	0.090141	RP11-345	antisense
ENSG0000(	36.38631	0.247817	0.08932	2.774477	0.005529	0.011672	RP11-693	pseudoge
ENSG0000(	36.3737	0.26329	0.107167	2.456833	0.014017	0.02709	CTD-2291	lincRNA
ENSG0000(	36.36625	-1.34804	0.139925	-9.63406	5.74E-22	2.74E-20	PABPC1L2	protein_cc
ENSG0000(	36.36625	-0.30395	0.085861	-3.53997	0.0004	0.001053	RP11-295	lincRNA
ENSG0000(	36.35805	-0.70329	0.130419	-5.39255	6.95E-08	3.37E-07	RTP1	protein_cc
ENSG0000(	36.35606	-0.03437	0.08826	-0.38947	0.696931	0.767969	LIPH	protein_cc
ENSG0000(	36.34764	0.235999	0.078235	3.016529	0.002557	0.005785	CTD-3099	sense_intr
ENSG0000(	36.33833	-0.21916	0.091713	-2.38965	0.016864	0.031973	EPPK1	protein_cc
ENSG0000(	36.32066	0.327609	0.179809	1.82198	0.068458	0.111126	BARHL1	protein_cc
ENSG0000(	36.30092	0.021616	0.095204	0.227053	0.820383	0.86829	GNG8	protein_cc
ENSG0000(	36.29692	-1.03268	0.165289	-6.24776	4.16E-10	2.88E-09	DCAF12L2	protein_cc
ENSG0000(	36.29387	0.402549	0.069188	5.818228	5.95E-09	3.42E-08	RP11-23J9	processed
ENSG0000(	36.2793	-0.3073	0.205323	-1.49668	0.134477	0.19908	KALP	pseudoge
ENSG0000(	36.27116	-0.14993	0.073789	-2.03187	0.042167	0.072684	ACTA1	protein_cc
ENSG0000(	36.26872	-0.86556	0.12975	-6.67101	2.54E-11	2.12E-10	RP11-13K	lincRNA
ENSG0000(	36.26554	-0.01879	0.070894	-0.26499	0.791016	0.844973	RP11-839	antisense
ENSG0000(	36.24651	-0.47255	0.083629	-5.65054	1.60E-08	8.60E-08	TMEM30B	protein_cc
ENSG0000(	36.24099	-0.19968	0.067307	-2.96674	0.00301	0.006705	ZNRF2P2	pseudoge
ENSG0000(	36.23757	0.749509	0.115533	6.487419	8.73E-11	6.72E-10	SALL4	protein_cc
ENSG0000(	36.2299	-0.16922	0.073694	-2.29631	0.021658	0.04001	PDPK2	pseudoge
ENSG0000(	36.22662	-0.40668	0.102003	-3.98691	6.69E-05	0.000202	MUC6	protein_cc
ENSG0000(	36.21984	0.291367	0.07714	3.777124	0.000159	0.000448	RP11-645	lincRNA
ENSG0000(	36.21599	0.059644	0.150119	0.397313	0.691136	0.76326	CXorf22	protein_cc
ENSG0000(	36.214	-0.21662	0.092768	-2.33506	0.01954	0.036454	RP11-521	lincRNA
ENSG0000(	36.21192	0.051174	0.052743	0.970253	0.331921	0.426493	RP11-177	processed
ENSG0000(	36.16064	-0.13405	0.057517	-2.33064	0.019772	0.036839	RNASEH2I	antisense
ENSG0000(	36.1278	-1.36834	0.15658	-8.73894	2.35E-18	6.21E-17	RP11-728	antisense
ENSG0000(	36.11925	0.199166	0.076146	2.615574	0.008908	0.017998	CTC-273B	lincRNA
ENSG0000(	36.09757	-0.37309	0.05952	-6.26832	3.65E-10	2.54E-09	RP11-135.	antisense
ENSG0000(	36.06989	-0.04394	0.067053	-0.65534	0.51225	0.605305	AC006946	protein_cc
ENSG0000(	36.0545	0.320405	0.052149	6.143995	8.05E-10	5.32E-09	SMC5-AS:	antisense
ENSG0000(	36.05036	0.0475	0.075233	0.631363	0.527803	0.619534	RP11-203	antisense
ENSG0000(	36.04128	-0.44195	0.063355	-6.97588	3.04E-12	2.93E-11	RP11-543	lincRNA
ENSG0000(	36.0267	-0.00114	0.06872	-0.01653	0.986808	0.991025	RP3-402G	antisense
ENSG0000(	35.99434	-0.11749	0.066099	-1.77747	0.07549	0.121021	RP5-1126	3prime_ov
ENSG0000(	35.96697	-0.33543	0.085204	-3.93677	8.26E-05	0.000245	TJP3	protein_cc
ENSG0000(	35.96575	-1.22077	0.130931	-9.32377	1.12E-20	4.28E-19	CRHR2	protein_cc
ENSG0000(	35.94675	-0.09367	0.069228	-1.3531	0.176022	0.250571	ABCC2	protein_cc
ENSG0000(	35.94644	-0.37829	0.069922	-5.41015	6.30E-08	3.08E-07	PKDREJ	protein_cc
ENSG0000(	35.94625	0.394756	0.112811	3.499279	0.000467	0.001211	RAB38	protein_cc
ENSG0000(	35.93868	0.185887	0.05794	3.20826	0.001335	0.003183	XXbac-B1	lincRNA
ENSG0000(	35.91924	-0.23082	0.073917	-3.12271	0.001792	0.004174	AL021068	pseudoge
ENSG0000(	35.91903	-0.25241	0.053986	-4.67551	2.93E-06	1.10E-05	SUGT1P	pseudoge
ENSG0000(	35.90193	-0.09741	0.078796	-1.23628	0.216354	0.298408	RP11-182	lincRNA
ENSG0000(	35.89328	0.782654	0.114404	6.841147	7.86E-12	7.09E-11	HSPB1P1	pseudoge
ENSG0000(	35.855	-0.05233	0.067949	-0.77013	0.441223	0.538086	SH3GL1P1	pseudoge
ENSG0000(	35.82291	0.02125	0.070672	0.300679	0.763659	0.822587	SCARNA1	antisense
ENSG0000(	35.80674	0.578852	0.186676	3.10083	0.00193	0.004463	G6PC2	protein_cc
ENSG0000(	35.77544	-0.46041	0.121767	-3.78105	0.000156	0.000441	IFLTD1	protein_cc
ENSG0000(	35.77059	0.134494	0.07127	1.887105	0.059146	0.097849	ITIH4	protein_cc

ENSG0000(	35.75718	0.485478	0.06806	7.133057	9.82E-13	1.02E-11	AC012314	antisense
ENSG0000(	35.70537	-0.0961	0.070447	-1.36408	0.172541	0.246383	DLEU2L	protein_cc
ENSG0000(	35.70202	-0.07922	0.13865	-0.57138	0.567739	0.655723	CTD-2534	lincRNA
ENSG0000(	35.67596	0.886112	0.106307	8.335412	7.72E-17	1.60E-15	SRP9P1	pseudoge
ENSG0000(	35.63187	0.073596	0.088246	0.833992	0.404285	0.501171	APOC1P1	pseudoge
ENSG0000(	35.61775	-0.298	0.098195	-3.03482	0.002407	0.005474	POU2AF1	protein_cc
ENSG0000(	35.58382	-0.28264	0.061592	-4.58897	4.45E-06	1.63E-05	RP11-981(	sense_ove
ENSG0000(	35.5531	0.047758	0.064495	0.740488	0.459004	0.55531	BRWD1-IT	sense_intr
ENSG0000(	35.53126	0.928204	0.139242	6.666131	2.63E-11	2.19E-10	FAM225B	lincRNA
ENSG0000(	35.52791	2.106476	0.134617	15.64791	3.43E-55	1.41E-51	RP11-745(	antisense
ENSG0000(	35.52401	-0.45287	0.08735	-5.18451	2.17E-07	9.72E-07	CNGA4	protein_cc
ENSG0000(	35.51308	-0.31893	0.091686	-3.47848	0.000504	0.0013	WNT11	protein_cc
ENSG0000(	35.51244	1.122034	0.118483	9.470034	2.80E-21	1.19E-19	UBE2SP1	pseudoge
ENSG0000(	35.50568	0.035867	0.049199	0.729023	0.465987	0.56186	LINC0085:	antisense
ENSG0000(	35.49366	1.002448	0.098065	10.22226	1.58E-24	1.26E-22	RP11-651	pseudoge
ENSG0000(	35.48497	0.009489	0.052711	0.18001	0.857145	0.896858	RP11-477	lincRNA
ENSG0000(	35.46508	-0.06748	0.083225	-0.81086	0.417444	0.514443	NTN3	protein_cc
ENSG0000(	35.42908	-0.20009	0.085155	-2.34973	0.018787	0.035192	UBL4B	protein_cc
ENSG0000(	35.39037	0.270749	0.153745	1.761031	0.078233	0.124868	ZNF99	protein_cc
ENSG0000(	35.38213	-0.20015	0.104096	-1.92276	0.05451	0.091139	C1orf145	protein_cc
ENSG0000(	35.38093	0.502744	0.071504	7.031024	2.05E-12	2.04E-11	HIF1A-AS:	lincRNA
ENSG0000(	35.34006	-0.37657	0.134263	-2.80475	0.005036	0.010727	FCAR	protein_cc
ENSG0000(	35.335	-0.04865	0.097892	-0.49697	0.619207	0.701901	RP1-8B1.4	antisense
ENSG0000(	35.32516	-0.79122	0.103566	-7.63979	2.18E-14	2.96E-13	MIR4500H	lincRNA
ENSG0000(	35.29548	-0.6787	0.103557	-6.55386	5.61E-11	4.43E-10	WI2-8151	lincRNA
ENSG0000(	35.2811	-0.13749	0.121313	-1.13332	0.25708	0.344951	AC069213	pseudoge
ENSG0000(	35.25672	-0.31026	0.088962	-3.48755	0.000487	0.00126	RP11-60A	lincRNA
ENSG0000(	35.23396	-0.17697	0.110291	-1.6046	0.108581	0.165836	GPR55	protein_cc
ENSG0000(	35.2272	-1.06815	0.107659	-9.92165	3.35E-23	2.04E-21	SLC26A8	protein_cc
ENSG0000(	35.21806	-0.96142	0.229528	-4.18869	2.81E-05	9.00E-05	MYOD1	protein_cc
ENSG0000(	35.21013	0.389742	0.065194	5.978219	2.26E-09	1.39E-08	RP11-181(	protein_cc
ENSG0000(	35.20361	0.569425	0.123558	4.608556	4.05E-06	1.49E-05	RP11-966	antisense
ENSG0000(	35.19428	0.035377	0.063946	0.553227	0.580108	0.667199	RP11-550	pseudoge
ENSG0000(	35.15468	0.250407	0.153229	1.634208	0.102215	0.157458	PCDHA13	protein_cc
ENSG0000(	35.12994	0.088902	0.104499	0.850741	0.394913	0.491479	RP11-278	lincRNA
ENSG0000(	35.12706	0.041891	0.157193	0.266494	0.789859	0.843999	RP11-1C8	lincRNA
ENSG0000(	35.12674	0.123952	0.05594	2.215783	0.026706	0.048346	RP1-313I6	antisense
ENSG0000(	35.10833	0.23425	0.07861	2.979879	0.002884	0.006448	RP11-73M	antisense
ENSG0000(	35.10574	-0.11192	0.082814	-1.35151	0.176533	0.251144	CCDC158	protein_cc
ENSG0000(	35.10312	-0.13604	0.129969	-1.04672	0.29523	0.387113	RP11-800.	sense_ove
ENSG0000(	35.09275	0.039269	0.08145	0.482132	0.629712	0.711124	RP11-110.	protein_cc
ENSG0000(	35.09086	-0.16666	0.069924	-2.38351	0.017149	0.032434	HHATL-AS	antisense
ENSG0000(	35.04469	0.505397	0.069002	7.324365	2.40E-13	2.74E-12	RP11-163	pseudoge
ENSG0000(	35.02731	0.025945	0.088605	0.292819	0.769661	0.827321	RP11-326(	lincRNA
ENSG0000(	35.01475	0.110462	0.116449	0.948588	0.34283	0.438006	RP11-216	pseudoge
ENSG0000(	35.00823	0.155832	0.082453	1.889945	0.058765	0.097289	RDH12	protein_cc
ENSG0000(	35.00215	-0.04985	0.121675	-0.40967	0.682045	0.755228	CCL28	protein_cc
ENSG0000(	34.98392	-0.00575	0.054891	-0.10471	0.916608	0.941212	DSTNP1	pseudoge
ENSG0000(	34.97285	0.38541	0.202597	1.902345	0.057126	0.094923	RP11-197	lincRNA
ENSG0000(	34.96357	-0.01587	0.051113	-0.31055	0.756144	0.816282	RP11-311	pseudoge
ENSG0000(	34.93718	-0.37087	0.070884	-5.23208	1.68E-07	7.67E-07	ASB3	protein_cc
ENSG0000(	34.93154	-0.16094	0.096749	-1.66351	0.09621	0.149393	RP11-113(	pseudoge
ENSG0000(	34.89365	-0.41073	0.086082	-4.77142	1.83E-06	7.10E-06	CTC-425C	sense_intr
ENSG0000(	34.89322	-0.40848	0.083648	-4.88337	1.04E-06	4.20E-06	IBA57-AS:	protein_cc
ENSG0000(	34.88445	-0.20156	0.044245	-4.55564	5.22E-06	1.89E-05	RP11-195	antisense
ENSG0000(	34.8806	-0.20967	0.051638	-4.06035	4.90E-05	0.000151	C15orf38-	protein_cc
ENSG0000(	34.87944	-0.45162	0.113467	-3.98021	6.89E-05	0.000207	BTBD16	protein_cc
ENSG0000(	34.86056	1.033876	0.130861	7.900536	2.78E-15	4.47E-14	RPSAP15	pseudoge
ENSG0000(	34.84135	1.27059	0.197235	6.442022	1.18E-10	8.90E-10	OR51E1	protein_cc

ENSG0000(	34.83135	-0.21571	0.135121	-1.5964	0.110401	0.168242	SULT1C2	protein_cc
ENSG0000(	34.81922	0.09482	0.167159	0.567244	0.570549	0.658285	HMGA1P7	pseudoge
ENSG0000(	34.79192	0.021405	0.142169	0.150558	0.880324	0.91438	RP11-467	lincRNA
ENSG0000(	34.76792	0.664139	0.092029	7.216598	5.33E-13	5.77E-12	AC009245	pseudoge
ENSG0000(	34.76087	0.138081	0.17474	0.79021	0.429405	0.526454	IGHG4	IG_C_gene
ENSG0000(	34.74442	-1.49226	0.156439	-9.5389	1.44E-21	6.51E-20	AC062028	lincRNA
ENSG0000(	34.73469	1.02579	0.253738	4.042715	5.28E-05	0.000162	GATA4	protein_cc
ENSG0000(	34.73091	0.296076	0.065506	4.519857	6.19E-06	2.21E-05	CTD-3162	lincRNA
ENSG0000(	34.71914	-0.27807	0.124303	-2.23703	0.025284	0.046001	FGL1	protein_cc
ENSG0000(	34.70653	0.976569	0.141259	6.913347	4.73E-12	4.42E-11	RP11-558	antisense
ENSG0000(	34.69847	0.1745	0.063948	2.728783	0.006357	0.013249	LL21NC02	sense_intr
ENSG0000(	34.68946	-0.17961	0.164877	-1.08935	0.275999	0.366135	CD300E	protein_cc
ENSG0000(	34.6774	0.908122	0.125793	7.219175	5.23E-13	5.66E-12	CTC-467M	lincRNA
ENSG0000(	34.67559	0.157901	0.080959	1.950392	0.051129	0.086185	AC007277	antisense
ENSG0000(	34.67433	-0.03704	0.068963	-0.53715	0.591163	0.676866	NHLRC4	protein_cc
ENSG0000(	34.67418	-0.13165	0.082796	-1.59005	0.111823	0.170143	ABCA4	protein_cc
ENSG0000(	34.65413	0.381302	0.076496	4.984568	6.21E-07	2.59E-06	RP3-508I1	antisense
ENSG0000(	34.65007	-0.12786	0.087244	-1.46556	0.142769	0.209649	MEFV	protein_cc
ENSG0000(	34.64214	0.307113	0.059089	5.197437	2.02E-07	9.12E-07	AC007405	lincRNA
ENSG0000(	34.62774	-0.01693	0.047222	-0.35858	0.719911	0.786882	TMEM56-	protein_cc
ENSG0000(	34.60644	-0.10481	0.059594	-1.75881	0.07861	0.125349	C10ORF68	protein_cc
ENSG0000(	34.60445	-1.34192	0.169234	-7.92939	2.20E-15	3.59E-14	FAM163A	protein_cc
ENSG0000(	34.58543	-0.30686	0.124701	-2.46076	0.013864	0.026822	TNXA	pseudoge
ENSG0000(	34.58459	-1.50933	0.181165	-8.33126	8.00E-17	1.65E-15	CTD-2023	lincRNA
ENSG0000(	34.57742	-0.49073	0.077994	-6.29194	3.14E-10	2.21E-09	TACR2	protein_cc
ENSG0000(	34.55107	0.138191	0.066805	2.06857	0.038586	0.067227	RP11-342	antisense
ENSG0000(	34.54908	-0.12297	0.124643	-0.98654	0.323868	0.418014	AC006946	lincRNA
ENSG0000(	34.51387	-0.08782	0.066725	-1.3161	0.188139	0.265049	AP001092	antisense
ENSG0000(	34.50905	0.110355	0.072511	1.521913	0.128031	0.190967	RP11-390	antisense
ENSG0000(	34.50007	0.143361	0.048896	2.931936	0.003369	0.007433	RP11-7O1	antisense
ENSG0000(	34.47458	-0.37711	0.099461	-3.79149	0.00015	0.000424	IRF4	protein_cc
ENSG0000(	34.46654	-0.61803	0.085277	-7.24732	4.25E-13	4.67E-12	GIPC2	protein_cc
ENSG0000(	34.37928	0.101273	0.058619	1.727643	0.084052	0.132796	RP11-390	sense_intr
ENSG0000(	34.36125	-0.09634	0.059257	-1.62584	0.103983	0.159763	AC007038	antisense
ENSG0000(	34.32184	-0.10617	0.059981	-1.77004	0.076721	0.122728	CTC-336P	antisense
ENSG0000(	34.30639	-0.65174	0.129586	-5.02942	4.92E-07	2.09E-06	LINC0094(	lincRNA
ENSG0000(	34.30509	-1.0295	0.13693	-7.51848	5.54E-14	7.04E-13	MIR770	miRNA
ENSG0000(	34.2924	-0.92804	0.155856	-5.95447	2.61E-09	1.59E-08	FAM153A	protein_cc
ENSG0000(	34.2844	0.063262	0.057259	1.104844	0.269227	0.35854	RP11-170	antisense
ENSG0000(	34.28378	0.127213	0.146067	0.870928	0.383793	0.480474	AC079354	protein_cc
ENSG0000(	34.27957	-0.02863	0.052253	-0.5479	0.583762	0.67022	GLIPR1L1	protein_cc
ENSG0000(	34.27192	-0.30104	0.09331	-3.22623	0.001254	0.003002	ABCA10	protein_cc
ENSG0000(	34.26717	-0.09552	0.065281	-1.46327	0.143393	0.21043	RP11-464	antisense
ENSG0000(	34.2601	1.262266	0.162376	7.773738	7.62E-15	1.14E-13	TMSB4XP1	pseudoge
ENSG0000(	34.23045	0.401042	0.065714	6.102844	1.04E-09	6.78E-09	RP11-9L18	pseudoge
ENSG0000(	34.20195	-0.00945	0.071683	-0.13185	0.895106	0.925441	CD27	protein_cc
ENSG0000(	34.19796	0.070561	0.07549	0.934711	0.349937	0.445371	RP11-488	pseudoge
ENSG0000(	34.19126	0.477955	0.065742	7.270159	3.59E-13	3.99E-12	RAG1	protein_cc
ENSG0000(	34.17018	-0.80518	0.158204	-5.08952	3.59E-07	1.56E-06	NAP1L6	protein_cc
ENSG0000(	34.16742	-0.47034	0.102157	-4.60406	4.14E-06	1.52E-05	TRABD2B	protein_cc
ENSG0000(	34.14388	0.86571	0.114007	7.593489	3.11E-14	4.13E-13	LEF1-AS1	processed
ENSG0000(	34.14007	0.059187	0.075921	0.779577	0.43564	0.532669	HSPB9	protein_cc
ENSG0000(	34.12743	0.631225	0.176036	3.585774	0.000336	0.000896	LHX3	protein_cc
ENSG0000(	34.06303	-0.13386	0.092534	-1.44658	0.148014	0.216263	RP11-156	lincRNA
ENSG0000(	34.05617	-0.20798	0.057753	-3.60112	0.000317	0.000849	CTC-490E	lincRNA
ENSG0000(	34.05336	0.51453	0.087722	5.865438	4.48E-09	2.62E-08	RP11-57A	lincRNA
ENSG0000(	34.03649	-1.43002	0.184496	-7.75095	9.12E-15	1.34E-13	LINC0049(	lincRNA
ENSG0000(	34.03193	0.269632	0.046186	5.837953	5.28E-09	3.06E-08	AC073063	pseudoge
ENSG0000(	34.02886	0.855588	0.11076	7.724684	1.12E-14	1.62E-13	XRCC6P2	pseudoge

ENSG0000(	34.02781	0.191458	0.063835	2.999246	0.002706	0.006085	AC079922	pseudoge
ENSG0000(	34.02207	0.270733	0.050902	5.318668	1.05E-07	4.95E-07	RP11-867(	lincRNA
ENSG0000(	34.00395	0.191636	0.079599	2.407531	0.016061	0.03062	RP11-120	lincRNA
ENSG0000(	34.00369	0.425496	0.085173	4.995682	5.86E-07	2.46E-06	CRIP1	protein_cc
ENSG0000(	33.99988	-0.07387	0.128489	-0.57489	0.565365	0.653515	NPPC	protein_cc
ENSG0000(	33.97312	0.436264	0.078361	5.567378	2.59E-08	1.34E-07	UBE2MP1	pseudoge
ENSG0000(	33.96242	-0.32293	0.069455	-4.64944	3.33E-06	1.24E-05	DKFZP667	protein_cc
ENSG0000(	33.94307	0.158291	0.069803	2.26769	0.023348	0.042815	RP11-264	antisense
ENSG0000(	33.93785	-0.34172	0.140343	-2.43489	0.014896	0.028613	HLA-DPB2	pseudoge
ENSG0000(	33.89604	0.267457	0.12092	2.211855	0.026977	0.048749	RP11-503	lincRNA
ENSG0000(	33.89279	-0.09017	0.077379	-1.16534	0.24388	0.329945	RP11-348	lincRNA
ENSG0000(	33.87157	-0.63026	0.099734	-6.31937	2.63E-10	1.87E-09	ARHGEF5	protein_cc
ENSG0000(	33.86517	-0.71285	0.174003	-4.09679	4.19E-05	0.000131	NPAS4	protein_cc
ENSG0000(	33.8502	-0.23209	0.079078	-2.93491	0.003336	0.007369	TATDN2P2	pseudoge
ENSG0000(	33.8501	0.623335	0.100353	6.211435	5.25E-10	3.57E-09	AC004067	antisense
ENSG0000(	33.83554	-0.26553	0.066406	-3.9985	6.37E-05	0.000193	MST1R	protein_cc
ENSG0000(	33.82755	0.148729	0.08031	1.851941	0.064034	0.104864	RP11-465	antisense
ENSG0000(	33.81097	0.333491	0.083336	4.001758	6.29E-05	0.00019	AC074212	antisense
ENSG0000(	33.80938	0.359165	0.064448	5.57298	2.50E-08	1.31E-07	CECR5-AS	antisense
ENSG0000(	33.79802	-0.30749	0.042706	-7.20018	6.01E-13	6.46E-12	RP11-245.	lincRNA
ENSG0000(	33.79113	0.085879	0.154424	0.556122	0.578127	0.665274	OR2I1P	pseudoge
ENSG0000(	33.77597	0.101496	0.097333	1.042768	0.297056	0.389162	RP3-510C	antisense
ENSG0000(	33.76936	0.009929	0.105085	0.094486	0.924723	0.94692	AC012507	antisense
ENSG0000(	33.75619	-0.24452	0.090232	-2.70995	0.006729	0.013957	AP002954	antisense
ENSG0000(	33.75359	0.096875	0.047288	2.048634	0.040498	0.070159	RP11-421	lincRNA
ENSG0000(	33.74827	-0.06745	0.074258	-0.90835	0.363693	0.459663	FOXD4	protein_cc
ENSG0000(	33.74735	-0.44824	0.086934	-5.15615	2.52E-07	1.12E-06	AC005082	lincRNA
ENSG0000(	33.74234	0.133848	0.07697	1.738964	0.082041	0.129989	RP11-98D	lincRNA
ENSG0000(	33.7349	-0.11281	0.068949	-1.63612	0.101815	0.156947	RP11-326	antisense
ENSG0000(	33.73224	-0.4899	0.112731	-4.34571	1.39E-05	4.68E-05	DBH	protein_cc
ENSG0000(	33.71951	0.291455	0.061281	4.756047	1.97E-06	7.62E-06	UBE2E1-A	antisense
ENSG0000(	33.71802	0.181798	0.051721	3.514968	0.00044	0.001148	BNIPL	protein_cc
ENSG0000(	33.69617	-0.45667	0.099943	-4.56932	4.89E-06	1.78E-05	CTD-2315	lincRNA
ENSG0000(	33.68554	-0.08694	0.079821	-1.08919	0.276069	0.366192	RP11-56B	pseudoge
ENSG0000(	33.65713	0.364414	0.070484	5.170138	2.34E-07	1.04E-06	RP1-40E1(	antisense
ENSG0000(	33.62268	0.357251	0.071035	5.029222	4.92E-07	2.09E-06	RP11-3D4	antisense
ENSG0000(	33.60441	0.059325	0.126229	0.469978	0.638371	0.718514	CD96	protein_cc
ENSG0000(	33.57872	-0.01049	0.056517	-0.18553	0.852814	0.893577	AK3P3	pseudoge
ENSG0000(	33.57071	-0.68048	0.115506	-5.89135	3.83E-09	2.27E-08	PRG4	protein_cc
ENSG0000(	33.55488	-0.25173	0.056905	-4.42369	9.70E-06	3.36E-05	RP11-506	lincRNA
ENSG0000(	33.5273	0.088352	0.071139	1.241968	0.214248	0.295971	LRRC37A	protein_cc
ENSG0000(	33.52441	0.93878	0.132388	7.091107	1.33E-12	1.36E-11	RPL5P1	pseudoge
ENSG0000(	33.5185	0.887907	0.113738	7.806611	5.87E-15	8.91E-14	RP11-680	pseudoge
ENSG0000(	33.49665	0.629448	0.089626	7.023013	2.17E-12	2.14E-11	CSAG1	protein_cc
ENSG0000(	33.49351	-0.27664	0.122553	-2.2573	0.023989	0.043875	RP11-742	processed
ENSG0000(	33.48739	0.977072	0.142608	6.851455	7.31E-12	6.64E-11	PFN1P1	pseudoge
ENSG0000(	33.48167	0.00245	0.0736	0.033292	0.973442	0.981413	HEATR4	protein_cc
ENSG0000(	33.47024	-0.04113	0.057851	-0.71104	0.477059	0.572722	C9orf43	protein_cc
ENSG0000(	33.46704	0.36408	0.156549	2.325655	0.020037	0.037282	LILRA5	protein_cc
ENSG0000(	33.46175	-0.00118	0.070053	-0.01686	0.986545	0.990809	AC015849	antisense
ENSG0000(	33.4228	-0.37475	0.099873	-3.75221	0.000175	0.000491	GJB5	protein_cc
ENSG0000(	33.39519	-0.13824	0.054616	-2.53107	0.011372	0.022428	RP11-73M	sense_intr
ENSG0000(	33.37945	-0.04183	0.049626	-0.84298	0.399242	0.495965	RP11-124	antisense
ENSG0000(	33.37281	-0.53722	0.182082	-2.95042	0.003173	0.007038	C21orf128	protein_cc
ENSG0000(	33.33844	-0.12914	0.058101	-2.22272	0.026235	0.047573	RP6-109B	antisense
ENSG0000(	33.32776	0.235519	0.100669	2.339542	0.019307	0.03607	DUSP5P1	pseudoge
ENSG0000(	33.30532	-0.19057	0.042882	-4.44408	8.83E-06	3.07E-05	RP11-379	lincRNA
ENSG0000(	33.30308	0.28169	0.058232	4.837368	1.32E-06	5.22E-06	TMSB15B	protein_cc
ENSG0000(	33.29997	0.159559	0.053409	2.987503	0.002813	0.006299	PRR7-AS1	antisense

ENSG0000(	33.28246	0.04029	0.132272	0.304597	0.760673	0.820144	ALOX12B	protein_cc
ENSG0000(	33.28093	0.001486	0.046762	0.031779	0.974648	0.982228	RP11-203	antisense
ENSG0000(	33.27607	0.239955	0.06318	3.797955	0.000146	0.000414	ZNF833P	lincRNA
ENSG0000(	33.27225	0.808204	0.119118	6.784912	1.16E-11	1.02E-10	RP1-40E1(	lincRNA
ENSG0000(	33.2411	1.144496	0.148226	7.721283	1.15E-14	1.65E-13	MAGED4B	protein_cc
ENSG0000(	33.24073	0.334871	0.060513	5.533871	3.13E-08	1.61E-07	RP3-337H	antisense
ENSG0000(	33.23493	-0.05019	0.063707	-0.78789	0.430763	0.52779	RP11-132.	antisense
ENSG0000(	33.22472	-0.21652	0.071112	-3.04476	0.002329	0.005306	ADAM1B	pseudoge
ENSG0000(	33.22205	-0.33181	0.093959	-3.53146	0.000413	0.001084	IDI2-AS1	antisense
ENSG0000(	33.21189	-0.95781	0.123492	-7.75606	8.76E-15	1.29E-13	DIO3	protein_cc
ENSG0000(	33.1881	0.390051	0.113416	3.439128	0.000584	0.001488	NPIPA3	protein_cc
ENSG0000(	33.18752	0.207633	0.097204	2.13606	0.032675	0.057921	ASCL2	protein_cc
ENSG0000(	33.1866	0.074295	0.157017	0.473166	0.636095	0.71658	C1orf87	protein_cc
ENSG0000(	33.15878	0.154127	0.073772	2.089233	0.036687	0.064277	CTC-542B	lincRNA
ENSG0000(	33.1544	0.073766	0.154524	0.477378	0.633093	0.714138	DYDC2	protein_cc
ENSG0000(	33.12975	0.006414	0.061091	0.104987	0.916386	0.941101	RP11-261(	lincRNA
ENSG0000(	33.11845	-1.06993	0.140698	-7.60442	2.86E-14	3.81E-13	RFPL2	protein_cc
ENSG0000(	33.10142	-0.10117	0.087518	-1.15599	0.247685	0.33429	NPIPB6	protein_cc
ENSG0000(	33.08324	0.51578	0.066043	7.809715	5.73E-15	8.71E-14	AC099522	pseudoge
ENSG0000(	33.07713	-0.06426	0.065197	-0.98568	0.324292	0.418456	RP11-33B	lincRNA
ENSG0000(	33.05628	-0.07223	0.104	-0.69454	0.487343	0.582087	INHBC	protein_cc
ENSG0000(	33.0451	-0.34484	0.09	-3.83159	0.000127	0.000365	NEBL-AS1	antisense
ENSG0000(	32.97853	-1.33214	0.157186	-8.47491	2.35E-17	5.32E-16	AC244230	protein_cc
ENSG0000(	32.96222	0.291941	0.104122	2.803831	0.00505	0.010756	C11orf21	protein_cc
ENSG0000(	32.9541	-0.27346	0.082713	-3.30611	0.000946	0.002315	CTD-2353	antisense
ENSG0000(	32.94664	0.019489	0.05477	0.35583	0.721968	0.788501	RP11-42I1	lincRNA
ENSG0000(	32.92721	-0.32906	0.076988	-4.27413	1.92E-05	6.33E-05	RP11-266	lincRNA
ENSG0000(	32.90686	0.407757	0.07833	5.205667	1.93E-07	8.75E-07	LRRFIP1P1	pseudoge
ENSG0000(	32.89946	0.150003	0.045649	3.285992	0.001016	0.002473	RP11-134!	lincRNA
ENSG0000(	32.89307	0.706146	0.092344	7.646927	2.06E-14	2.82E-13	SNORA76	lincRNA
ENSG0000(	32.89154	0.459232	0.110982	4.137891	3.51E-05	0.000111	CDC42BP(	protein_cc
ENSG0000(	32.88403	1.049448	0.145891	7.193375	6.32E-13	6.77E-12	APCDD1L-	processed
ENSG0000(	32.88299	-1.39343	0.174468	-7.98673	1.39E-15	2.34E-14	XX-CR54.:	protein_cc
ENSG0000(	32.87244	0.262701	0.078755	3.335688	0.000851	0.002101	RP13-516	antisense
ENSG0000(	32.85947	0.818092	0.133234	6.140257	8.24E-10	5.44E-09	MACC1	protein_cc
ENSG0000(	32.85663	-0.34315	0.083876	-4.09117	4.29E-05	0.000134	ACTA2-AS	antisense
ENSG0000(	32.85007	-0.41717	0.104646	-3.98653	6.70E-05	0.000202	RP11-238	lincRNA
ENSG0000(	32.81057	0.056405	0.082936	0.680104	0.496439	0.590591	C19orf45	protein_cc
ENSG0000(	32.80634	-0.13285	0.070037	-1.89688	0.057843	0.095983	RP11-111	pseudoge
ENSG0000(	32.80485	0.203571	0.064035	3.179049	0.001478	0.003495	TIPARP-A!	antisense
ENSG0000(	32.80041	0.158683	0.052108	3.04529	0.002325	0.005298	SUMO2P1	pseudoge
ENSG0000(	32.78485	-0.0209	0.130387	-0.16032	0.872628	0.908528	SLAMF6	protein_cc
ENSG0000(	32.76932	0.007758	0.055534	0.139689	0.888906	0.92085	RP1-122P	lincRNA
ENSG0000(	32.76522	1.066683	0.124879	8.541747	1.32E-17	3.11E-16	NME2P1	pseudoge
ENSG0000(	32.73559	0.034741	0.049812	0.697452	0.48552	0.580388	RP11-109	lincRNA
ENSG0000(	32.72727	0.094819	0.070461	1.345694	0.178401	0.253457	LRIT3	protein_cc
ENSG0000(	32.71468	0.34758	0.085598	4.060606	4.89E-05	0.000151	VIM-AS1	antisense
ENSG0000(	32.70119	-0.37881	0.118643	-3.19288	0.001409	0.003343	SHISA8	protein_cc
ENSG0000(	32.69119	0.123364	0.091906	1.342293	0.179501	0.25466	SULT2B1	protein_cc
ENSG0000(	32.67223	-0.31917	0.197072	-1.61957	0.105326	0.161541	RP11-706(	lincRNA
ENSG0000(	32.66945	0.330317	0.121154	2.726421	0.006403	0.013336	GDF5	protein_cc
ENSG0000(	32.66147	-0.16663	0.045069	-3.69722	0.000218	0.0006	RP11-550	processed
ENSG0000(	32.65301	0.179513	0.071186	2.521728	0.011678	0.02298	RP11-55K	antisense
ENSG0000(	32.64774	-0.48036	0.110008	-4.36659	1.26E-05	4.28E-05	DGCR10	sense_intr
ENSG0000(	32.645	-0.39362	0.083827	-4.69568	2.66E-06	1.00E-05	ITIH3	protein_cc
ENSG0000(	32.63745	0.238934	0.075278	3.174002	0.001504	0.003551	RP11-342	lincRNA
ENSG0000(	32.61477	0.243795	0.068451	3.561619	0.000369	0.000976	TMEM110	protein_cc
ENSG0000(	32.58907	-0.12935	0.070594	-1.83231	0.066905	0.108928	KB-1125A	antisense
ENSG0000(	32.57704	0.157757	0.056587	2.787854	0.005306	0.01125	RP11-197	processed

ENSG0000(	32.56559	3.138933	0.238773	13.1461	1.79E-39	2.38E-36	14-Sep	protein_cc
ENSG0000(	32.56203	0.088878	0.061405	1.447407	0.147783	0.215987	STEAP3-A	antisense
ENSG0000(	32.55437	-0.47154	0.127347	-3.70282	0.000213	0.000588	CTD-2561	antisense
ENSG0000(	32.5496	0.111212	0.116545	0.954242	0.339961	0.435013	CDK15	protein_cc
ENSG0000(	32.54701	-0.13755	0.092352	-1.48943	0.136375	0.20152	RP11-23N	antisense
ENSG0000(	32.51771	-0.42915	0.074353	-5.7718	7.84E-09	4.42E-08	ALDOB	protein_cc
ENSG0000(	32.49515	0.037964	0.059255	0.640685	0.521727	0.613892	ACTG1P1	pseudoge
ENSG0000(	32.49125	-0.04652	0.057162	-0.81389	0.415711	0.512707	MPRIPP1	pseudoge
ENSG0000(	32.48926	0.38723	0.121684	3.182259	0.001461	0.003458	NPW	protein_cc
ENSG0000(	32.48776	-0.33073	0.096391	-3.43116	0.000601	0.001527	ASGR2	protein_cc
ENSG0000(	32.4874	-0.63342	0.101641	-6.23197	4.61E-10	3.16E-09	TRGC2	TR_C_gene
ENSG0000(	32.4719	-0.09362	0.166818	-0.56122	0.574649	0.66192	RP1-153P	pseudoge
ENSG0000(	32.46689	-0.17391	0.205095	-0.84797	0.396453	0.493082	AREG	protein_cc
ENSG0000(	32.46507	-0.01062	0.124754	-0.08514	0.932152	0.952653	TMPRSS2	protein_cc
ENSG0000(	32.46358	0.401461	0.070553	5.690197	1.27E-08	6.92E-08	RP11-314.	pseudoge
ENSG0000(	32.46187	0.408551	0.063864	6.397228	1.58E-10	1.17E-09	HNRNPCP	pseudoge
ENSG0000(	32.45932	0.115473	0.076965	1.500329	0.133529	0.197876	LL0XNC01	antisense
ENSG0000(	32.45355	0.187455	0.08163	2.296397	0.021653	0.040003	AC008746	lincRNA
ENSG0000(	32.44549	-0.34227	0.064662	-5.29323	1.20E-07	5.63E-07	C3orf83	protein_cc
ENSG0000(	32.43555	0.695718	0.141331	4.92261	8.54E-07	3.49E-06	GATA3	protein_cc
ENSG0000(	32.42243	0.181325	0.058946	3.076133	0.002097	0.00482	CTD-2083	processed
ENSG0000(	32.40248	-0.13035	0.076262	-1.70919	0.087416	0.137488	RP11-165.	lincRNA
ENSG0000(	32.39946	-0.87609	0.108377	-8.08368	6.28E-16	1.12E-14	STRCP1	pseudoge
ENSG0000(	32.39807	-0.13425	0.085522	-1.56973	0.116477	0.176265	ZNF556	protein_cc
ENSG0000(	32.38867	0.124047	0.065712	1.887738	0.059061	0.097725	CTC-260E	antisense
ENSG0000(	32.38408	-0.27781	0.072923	-3.8096	0.000139	0.000396	A2MP1	pseudoge
ENSG0000(	32.3783	0.027311	0.056133	0.486534	0.626588	0.708452	KCNE2	protein_cc
ENSG0000(	32.36059	0.762842	0.111175	6.861652	6.81E-12	6.21E-11	EEF1A1P2!	pseudoge
ENSG0000(	32.35849	0.161761	0.080864	2.000396	0.045458	0.077655	SPATA4	protein_cc
ENSG0000(	32.35542	#######	0.159453	-0.00022	0.999823	0.99989	KCNK5	protein_cc
ENSG0000(	32.35202	0.079839	0.109523	0.728975	0.466017	0.56186	PMEL	protein_cc
ENSG0000(	32.3463	-0.29343	0.126796	-2.31422	0.020656	0.038329	TWIST2	protein_cc
ENSG0000(	32.34407	-0.23924	0.073439	-3.25768	0.001123	0.002712	FBXW12	protein_cc
ENSG0000(	32.34329	-0.2382	0.057313	-4.15612	3.24E-05	0.000103	LINC0053!	antisense
ENSG0000(	32.32748	-0.23565	0.062271	-3.78422	0.000154	0.000436	RP11-102	lincRNA
ENSG0000(	32.32334	0.563934	0.103128	5.468298	4.54E-08	2.27E-07	RTN3P1	pseudoge
ENSG0000(	32.3136	-0.12846	0.049199	-2.61111	0.009025	0.018211	RP11-284	antisense
ENSG0000(	32.30225	0.027869	0.055578	0.501445	0.616058	0.699045	AC068831	antisense
ENSG0000(	32.2933	0.937006	0.107622	8.706481	3.13E-18	8.11E-17	RP11-16F	pseudoge
ENSG0000(	32.29102	0.61929	0.085537	7.240013	4.49E-13	4.91E-12	AC010729	3prime_ov
ENSG0000(	32.27516	0.4235	0.083333	5.082026	3.73E-07	1.62E-06	C9orf170	protein_cc
ENSG0000(	32.27291	0.132855	0.074352	1.786841	0.073963	0.11888	RP11-16E	lincRNA
ENSG0000(	32.26855	0.072415	0.072202	1.002952	0.315884	0.409469	RP11-15A	antisense
ENSG0000(	32.26508	-0.46308	0.068098	-6.80011	1.05E-11	9.25E-11	NOTCH2N	protein_cc
ENSG0000(	32.24685	0.094206	0.076481	1.231754	0.218041	0.300282	RP11-95C	antisense
ENSG0000(	32.23513	0.075104	0.076552	0.981078	0.326554	0.420766	RP11-196	antisense
ENSG0000(	32.23272	-0.02118	0.06543	-0.32367	0.746191	0.808706	RP11-367	lincRNA
ENSG0000(	32.23142	0.516608	0.158747	3.254275	0.001137	0.002742	UBXN10-A	antisense
ENSG0000(	32.22304	-0.13588	0.0624	-2.1776	0.029436	0.05275	FTOP1	pseudoge
ENSG0000(	32.21911	-0.03707	0.059366	-0.62447	0.532317	0.623802	RP11-130	antisense
ENSG0000(	32.21772	0.025476	0.070844	0.359604	0.719143	0.78622	RP4-671G	antisense
ENSG0000(	32.21111	0.460847	0.099364	4.637994	3.52E-06	1.30E-05	RP11-290	lincRNA
ENSG0000(	32.18166	-0.20994	0.067004	-3.13328	0.001729	0.004038	CTC-246B	sense_ove
ENSG0000(	32.17946	0.480056	0.129514	3.706582	0.00021	0.00058	LDLRAD2	protein_cc
ENSG0000(	32.17111	0.09045	0.059219	1.527382	0.126666	0.189295	CTD-2410	lincRNA
ENSG0000(	32.15102	-0.76193	0.160871	-4.73629	2.18E-06	8.34E-06	RP11-531.	lincRNA
ENSG0000(	32.1373	-0.18169	0.043264	-4.19964	2.67E-05	8.60E-05	RP11-51F	pseudoge
ENSG0000(	32.13016	0.125746	0.053557	2.347878	0.018881	0.035345	RP1-309F	processed
ENSG0000(	32.12237	0.186866	0.110381	1.692919	0.090471	0.141648	SLC28A1	protein_cc

ENSG0000(	32.10055	0.348093	0.146136	2.381975	0.01722	0.032544	NR1H4	protein_cc
ENSG0000(	32.0987	0.177251	0.066681	2.658213	0.007856	0.016073	RP11-715.	lincRNA
ENSG0000(	32.08534	-0.77377	0.104803	-7.38309	1.55E-13	1.83E-12	ATOH7	protein_cc
ENSG0000(	32.08206	0.530883	0.091737	5.787032	7.16E-09	4.07E-08	RP11-430	lincRNA
ENSG0000(	32.05896	0.821611	0.091142	9.014628	1.98E-19	6.15E-18	PPIAP11	pseudoge
ENSG0000(	32.02714	-0.0427	0.057897	-0.73745	0.46085	0.557084	KB-431C1	antisense
ENSG0000(	32.02342	-0.40755	0.081853	-4.97911	6.39E-07	2.66E-06	ALMS1-IT	sense_intr
ENSG0000(	32.01272	-0.64799	0.274987	-2.35645	0.018451	0.03463	ANKRD62	processed
ENSG0000(	32.00197	0.010245	0.080281	0.127614	0.898454	0.927993	RP11-474	antisense
ENSG0000(	31.99065	-0.18316	0.081585	-2.24498	0.024769	0.045153	NUTM2A	protein_cc
ENSG0000(	31.98141	0.327426	0.071695	4.566902	4.95E-06	1.80E-05	CDK3	protein_cc
ENSG0000(	31.97707	0.399143	0.103376	3.861099	0.000113	0.000327	PROK1	protein_cc
ENSG0000(	31.96862	1.271319	0.147722	8.60616	7.55E-18	1.85E-16	RP11-522	lincRNA
ENSG0000(	31.96196	-0.018	0.068116	-0.2643	0.791549	0.84541	RP11-110	antisense
ENSG0000(	31.96031	-0.12503	0.117969	-1.05982	0.289226	0.380624	ENO4	protein_cc
ENSG0000(	31.95736	0.400673	0.059196	6.768555	1.30E-11	1.13E-10	RP11-727	antisense
ENSG0000(	31.95223	0.2681	0.072515	3.697142	0.000218	0.0006	AC074212	antisense
ENSG0000(	31.95134	-0.06234	0.100014	-0.6233	0.533089	0.624545	AC016582	lincRNA
ENSG0000(	31.93368	-0.16517	0.058145	-2.84069	0.004502	0.009678	XXbac-B4	pseudoge
ENSG0000(	31.91852	0.870967	0.134582	6.471665	9.69E-11	7.41E-10	HABP2	protein_cc
ENSG0000(	31.91713	-0.35599	0.064329	-5.53388	3.13E-08	1.61E-07	AC012074	lincRNA
ENSG0000(	31.89468	-0.03069	0.0826	-0.3715	0.710263	0.778986	AC072052	pseudoge
ENSG0000(	31.87065	-0.06377	0.096523	-0.66071	0.508797	0.602192	XXbac-BP	lincRNA
ENSG0000(	31.85724	-0.27688	0.079174	-3.49714	0.00047	0.00122	LINC0108	lincRNA
ENSG0000(	31.85399	0.244858	0.168601	1.452288	0.146422	0.214248	CD5L	protein_cc
ENSG0000(	31.85037	0.132551	0.061386	2.159306	0.030826	0.054955	PDZK1	protein_cc
ENSG0000(	31.84905	-0.98545	0.119255	-8.2634	1.42E-16	2.81E-15	RHOV	protein_cc
ENSG0000(	31.84312	-0.54257	0.126066	-4.30381	1.68E-05	5.59E-05	MPO	protein_cc
ENSG0000(	31.8372	0.202482	0.07074	2.862344	0.004205	0.00911	AC006942	antisense
ENSG0000(	31.80383	-0.34887	0.070666	-4.93685	7.94E-07	3.26E-06	ALMS1P	pseudoge
ENSG0000(	31.78414	0.107855	0.055892	1.929684	0.053646	0.089869	RP11-353	lincRNA
ENSG0000(	31.77918	-0.35833	0.112736	-3.17846	0.001481	0.003501	RP11-783	processed
ENSG0000(	31.77194	-0.92846	0.150837	-6.1554	7.49E-10	4.98E-09	NXPH2	protein_cc
ENSG0000(	31.74989	0.011193	0.053629	0.208721	0.834666	0.879605	RP11-573	antisense
ENSG0000(	31.74957	-0.57145	0.109761	-5.20634	1.93E-07	8.72E-07	CELF2-AS	antisense
ENSG0000(	31.70432	0.739424	0.093364	7.919814	2.38E-15	3.86E-14	RPL29P11	pseudoge
ENSG0000(	31.68502	-0.22955	0.213051	-1.07743	0.281289	0.371898	RP11-80F	pseudoge
ENSG0000(	31.6703	0.156708	0.062342	2.513688	0.011948	0.02345	RP5-940J	antisense
ENSG0000(	31.66843	0.645649	0.170877	3.778436	0.000158	0.000445	AC108142	antisense
ENSG0000(	31.66802	0.135937	0.058361	2.329225	0.019847	0.036969	AC068134	pseudoge
ENSG0000(	31.66628	-0.01889	0.045267	-0.41722	0.676515	0.750844	RP11-692	antisense
ENSG0000(	31.66135	-0.40966	0.137878	-2.9712	0.002966	0.006619	AL022344	lincRNA
ENSG0000(	31.653	0.52763	0.063112	8.360174	6.26E-17	1.32E-15	RP11-977	antisense
ENSG0000(	31.64978	0.08751	0.057618	1.518799	0.128813	0.191917	RP11-83N	lincRNA
ENSG0000(	31.63007	0.002015	0.050493	0.039914	0.968162	0.978055	AC000123	antisense
ENSG0000(	31.6276	0.019908	0.063306	0.314467	0.753166	0.814121	TTC36	protein_cc
ENSG0000(	31.61972	0.214366	0.094001	2.280472	0.02258	0.041556	CEACAM1	protein_cc
ENSG0000(	31.61477	1.412015	0.181069	7.798211	6.28E-15	9.49E-14	TMSB4XP2	pseudoge
ENSG0000(	31.58175	-0.18897	0.140545	-1.34455	0.17877	0.253861	CTC-523E	lincRNA
ENSG0000(	31.57973	-0.14851	0.07619	-1.94919	0.051273	0.086383	PVRL3-AS	antisense
ENSG0000(	31.5773	-0.03407	0.077649	-0.43874	0.660853	0.737702	LEFTY1	protein_cc
ENSG0000(	31.56022	-0.06703	0.051739	-1.29554	0.195135	0.273481	RP11-16E	lincRNA
ENSG0000(	31.54344	0.182662	0.040323	4.529988	5.90E-06	2.11E-05	AC005224	antisense
ENSG0000(	31.51911	0.09584	0.076759	1.248579	0.211819	0.293127	GZMM	protein_cc
ENSG0000(	31.50169	-0.22892	0.078466	-2.91744	0.003529	0.007755	PDE6C	protein_cc
ENSG0000(	31.45	-0.33349	0.127257	-2.62058	0.008778	0.017762	SMIM6	protein_cc
ENSG0000(	31.43906	0.523185	0.085683	6.106093	1.02E-09	6.66E-09	RP11-693.	lincRNA
ENSG0000(	31.43846	-0.44013	0.14025	-3.13817	0.0017	0.003975	KLHL14	protein_cc
ENSG0000(	31.43043	0.379168	0.094183	4.025882	5.68E-05	0.000173	RP11-262	pseudoge

ENSG0000(	31.4124	-0.77093	0.105333	-7.31897	2.50E-13	2.84E-12	TMEM191	processed
ENSG0000(	31.40801	0.064259	0.068178	0.942525	0.345924	0.441328	RP11-131	pseudoge
ENSG0000(	31.4037	0.157929	0.089911	1.756509	0.079002	0.125897	AC114730	antisense
ENSG0000(	31.3993	-0.1775	0.079152	-2.24252	0.024928	0.045418	TUBB1	protein_cc
ENSG0000(	31.38144	0.173815	0.143686	1.209692	0.226397	0.309859	KCNG4	protein_cc
ENSG0000(	31.35874	-0.30744	0.089136	-3.44916	0.000562	0.001437	ASCL5	protein_cc
ENSG0000(	31.35038	1.272026	0.157377	8.082665	6.34E-16	1.13E-14	MAGED4	protein_cc
ENSG0000(	31.34565	0.146173	0.065652	2.22647	0.025983	0.047152	RP11-629	lincRNA
ENSG0000(	31.32904	-0.04094	0.069154	-0.59199	0.553855	0.643468	RP11-755	antisense
ENSG0000(	31.32394	-0.33976	0.085444	-3.97636	7.00E-05	0.00021	AC010127	antisense
ENSG0000(	31.26895	0.34253	0.068343	5.011924	5.39E-07	2.27E-06	CTB-161K	antisense
ENSG0000(	31.24921	-0.30467	0.085484	-3.564	0.000365	0.000968	BPI	protein_cc
ENSG0000(	31.20383	-0.18408	0.0573	-3.21259	0.001315	0.003138	RP13-216	lincRNA
ENSG0000(	31.19524	-0.45719	0.102458	-4.46221	8.11E-06	2.84E-05	AC006129	lincRNA
ENSG0000(	31.19454	-0.10733	0.051707	-2.07574	0.037918	0.066172	RP13-941	lincRNA
ENSG0000(	31.18786	0.310441	0.065203	4.761117	1.93E-06	7.44E-06	HAVCR1P	pseudoge
ENSG0000(	31.18486	0.44139	0.074537	5.921789	3.18E-09	1.91E-08	AC002398	antisense
ENSG0000(	31.16814	-0.18649	0.107278	-1.73841	0.082138	0.130126	CD207	protein_cc
ENSG0000(	31.14631	0.296171	0.057795	5.124515	2.98E-07	1.31E-06	AC004951	pseudoge
ENSG0000(	31.14035	-0.50519	0.101642	-4.9703	6.68E-07	2.78E-06	SNORD10	sense_intr
ENSG0000(	31.1255	-0.11157	0.067332	-1.65707	0.097506	0.151142	CTA-363E	antisense
ENSG0000(	31.10314	0.263076	0.121286	2.169059	0.030078	0.053775	CTD-2195	pseudoge
ENSG0000(	31.08217	-0.29076	0.11484	-2.53186	0.011346	0.022385	TMEM150	protein_cc
ENSG0000(	31.07896	-0.39212	0.055521	-7.06251	1.64E-12	1.64E-11	RP11-549.	sense_intr
ENSG0000(	31.07185	1.298787	0.274171	4.737137	2.17E-06	8.31E-06	HOXC8	protein_cc
ENSG0000(	31.06246	-0.70152	0.085398	-8.21474	2.13E-16	4.09E-15	RP11-384	sense_intr
ENSG0000(	31.05681	0.133933	0.052345	2.558651	0.010508	0.020896	C5orf66	protein_cc
ENSG0000(	31.05001	-0.25984	0.077022	-3.37355	0.000742	0.001851	KCNIP2-A	antisense
ENSG0000(	31.04919	0.125243	0.110662	1.131759	0.257736	0.345688	RP11-809	pseudoge
ENSG0000(	31.03038	0.506522	0.124253	4.07654	4.57E-05	0.000142	FNDC7	protein_cc
ENSG0000(	31.02682	1.330521	0.120474	11.04406	2.34E-28	3.93E-26	EGFR-AS1	antisense
ENSG0000(	31.02027	-0.4593	0.093331	-4.92116	8.60E-07	3.51E-06	ODF3L2	protein_cc
ENSG0000(	31.01744	0.52116	0.055355	9.414804	4.74E-21	1.94E-19	CTD-2521	antisense
ENSG0000(	31.01655	0.317185	0.070084	4.525755	6.02E-06	2.15E-05	RP11-579	lincRNA
ENSG0000(	31.01086	0.012644	0.109047	0.115947	0.907694	0.934556	TAT	protein_cc
ENSG0000(	31.00529	-0.00414	0.092138	-0.0449	0.964186	0.975571	RP3-405J1	sense_intr
ENSG0000(	31.00245	0.352661	0.099393	3.548127	0.000388	0.001023	TPTE2P3	pseudoge
ENSG0000(	30.99135	0.772971	0.082372	9.383958	6.35E-21	2.52E-19	LINC0113	lincRNA
ENSG0000(	30.99006	-0.51676	0.072034	-7.17373	7.30E-13	7.75E-12	RP4-545K	sense_ove
ENSG0000(	30.98738	0.138135	0.066649	2.072569	0.038212	0.066654	ANO7P1	pseudoge
ENSG0000(	30.98516	-0.00501	0.060702	-0.08255	0.93421	0.954055	TAS2R15	pseudoge
ENSG0000(	30.95276	0.582435	0.092171	6.319073	2.63E-10	1.88E-09	HSP90B2F	pseudoge
ENSG0000(	30.93831	0.583477	0.124598	4.682884	2.83E-06	1.06E-05	RP3-510D	lincRNA
ENSG0000(	30.91838	0.440225	0.075186	5.855116	4.77E-09	2.78E-08	RP11-307	antisense
ENSG0000(	30.90793	-0.01501	0.053355	-0.28141	0.778398	0.834572	ZNF818P	pseudoge
ENSG0000(	30.90778	-0.05698	0.059646	-0.95523	0.339461	0.434482	TOP3B	protein_cc
ENSG0000(	30.90394	0.357423	0.136211	2.624043	0.008689	0.017601	CTB-171A	antisense
ENSG0000(	30.9025	-0.75335	0.144025	-5.23066	1.69E-07	7.72E-07	HTR4	protein_cc
ENSG0000(	30.90201	0.403453	0.075994	5.309009	1.10E-07	5.19E-07	RP11-178	antisense
ENSG0000(	30.89957	-0.07161	0.053623	-1.33547	0.181723	0.257341	RP11-330	sense_intr
ENSG0000(	30.87147	-0.00433	0.114065	-0.03794	0.969734	0.979014	RP11-153	pseudoge
ENSG0000(	30.86436	0.07693	0.079616	0.966265	0.333912	0.428529	AP000255	lincRNA
ENSG0000(	30.8612	0.029265	0.045321	0.645723	0.518458	0.611147	RP5-884G	antisense
ENSG0000(	30.85144	0.777706	0.081908	9.494861	2.21E-21	9.61E-20	SNRPEP2	pseudoge
ENSG0000(	30.82932	0.08427	0.074888	1.125276	0.260472	0.348771	RP11-41C	protein_cc
ENSG0000(	30.81567	0.234954	0.06423	3.657985	0.000254	0.000692	DCAF4L1	protein_cc
ENSG0000(	30.81013	0.345511	0.121556	2.842415	0.004477	0.009634	TP63	protein_cc
ENSG0000(	30.79331	0.617092	0.077549	7.95747	1.76E-15	2.92E-14	AC107983	pseudoge
ENSG0000(	30.79219	-0.51304	0.087329	-5.87473	4.24E-09	2.49E-08	CTD-2575	pseudoge

ENSG0000	30.79043	-0.43519	0.152153	-2.86019	0.004234	0.009163	RP11-231	lincRNA
ENSG0000	30.78857	-0.11865	0.065001	-1.82528	0.067959	0.110399	LINC0100	lincRNA
ENSG0000	30.78664	-0.10935	0.210045	-0.52061	0.602635	0.686941	CSF3	protein_cc
ENSG0000	30.74067	-0.37522	0.067867	-5.52879	3.22E-08	1.65E-07	RP11-100	processed
ENSG0000	30.73033	-1.53549	0.150817	-10.1812	2.41E-24	1.85E-22	PIK3C2G	protein_cc
ENSG0000	30.72896	-0.02499	0.060858	-0.41055	0.681403	0.754741	ECT2L	protein_cc
ENSG0000	30.71891	-0.10935	0.062571	-1.74755	0.080543	0.127984	RPL34-AS	lincRNA
ENSG0000	30.70951	0.060712	0.059694	1.017047	0.309131	0.402275	AP003068	pseudoge
ENSG0000	30.70856	0.100408	0.070349	1.427285	0.153498	0.223204	RP11-362	antisense
ENSG0000	30.69259	-0.13707	0.154699	-0.88607	0.375578	0.472174	KIF25-AS1	lincRNA
ENSG0000	30.68343	0.00934	0.101683	0.09185	0.926818	0.948498	LINC0070	lincRNA
ENSG0000	30.67977	0.029317	0.06912	0.424141	0.671463	0.746447	RP11-48B	lincRNA
ENSG0000	30.67528	0.57359	0.07777	7.375436	1.64E-13	1.92E-12	RP1-228P	pseudoge
ENSG0000	30.67414	-0.56526	0.099585	-5.67619	1.38E-08	7.48E-08	AC002451	antisense
ENSG0000	30.66699	0.558877	0.079169	7.059307	1.67E-12	1.68E-11	SPATA1	pseudoge
ENSG0000	30.63732	-0.00806	0.110362	-0.07305	0.941766	0.959355	MEIS3P2	pseudoge
ENSG0000	30.62243	-0.69593	0.15321	-4.54234	5.56E-06	2.00E-05	NPY5R	protein_cc
ENSG0000	30.58362	-0.0701	0.098356	-0.71272	0.476016	0.57172	MAP3K15	protein_cc
ENSG0000	30.57967	0.769059	0.164827	4.665858	3.07E-06	1.15E-05	LINC0094	lincRNA
ENSG0000	30.57222	0.092152	0.066111	1.39389	0.163351	0.235228	RP11-388	sense_intr
ENSG0000	30.54167	0.455009	0.082489	5.516008	3.47E-08	1.77E-07	TOB2P1	pseudoge
ENSG0000	30.53074	-0.06609	0.075601	-0.87425	0.381981	0.478599	COX5BP6	pseudoge
ENSG0000	30.5304	-0.15264	0.062308	-2.4497	0.014298	0.027596	EIF4HP2	pseudoge
ENSG0000	30.52867	-0.00719	0.069855	-0.10293	0.91802	0.942167	AC009120	lincRNA
ENSG0000	30.49442	-0.10109	0.068126	-1.48385	0.137848	0.203375	TNNC2	protein_cc
ENSG0000	30.48065	-0.23012	0.257251	-0.89453	0.371037	0.467422	IGKV4-1	IG_V_gene
ENSG0000	30.45512	0.314148	0.080797	3.888119	0.000101	0.000295	CTD-2619	processed
ENSG0000	30.42608	0.063913	0.056629	1.128624	0.259057	0.34712	AC098820	processed
ENSG0000	30.41444	-0.1842	0.058561	-3.14537	0.001659	0.003886	RP11-505	protein_cc
ENSG0000	30.41063	0.299572	0.085667	3.496941	0.000471	0.001221	RP11-483	sense_intr
ENSG0000	30.38277	0.327992	0.147974	2.216547	0.026654	0.04826	CD3D	protein_cc
ENSG0000	30.36337	-0.41619	0.102319	-4.0676	4.75E-05	0.000147	RP11-21A	antisense
ENSG0000	30.3435	0.136292	0.127423	1.069604	0.284798	0.375578	SLC16A12	protein_cc
ENSG0000	30.32537	0.386268	0.057347	6.735686	1.63E-11	1.40E-10	BRD7P2	pseudoge
ENSG0000	30.31907	0.107587	0.048613	2.213153	0.026887	0.048607	RP11-315	antisense
ENSG0000	30.30008	-0.17271	0.066799	-2.58553	0.009723	0.019476	RP11-107	lincRNA
ENSG0000	30.27795	-0.52903	0.095302	-5.55111	2.84E-08	1.47E-07	RP11-554	antisense
ENSG0000	30.27707	0.529523	0.07659	6.913755	4.72E-12	4.41E-11	RP11-5C2	antisense
ENSG0000	30.27552	0.562353	0.124779	4.506775	6.58E-06	2.34E-05	TSSC2	pseudoge
ENSG0000	30.26647	0.086404	0.049964	1.729338	0.083749	0.132371	ARPC4-TT	protein_cc
ENSG0000	30.26515	0.678986	0.085446	7.946393	1.92E-15	3.17E-14	RBBP4P1	pseudoge
ENSG0000	30.23999	-0.38423	0.079207	-4.85098	1.23E-06	4.90E-06	TNNI3K	protein_cc
ENSG0000	30.21737	-0.09827	0.109614	-0.89652	0.369978	0.466316	AC105053	antisense
ENSG0000	30.2079	0.436357	0.069441	6.283848	3.30E-10	2.32E-09	ZNF816-Z	pseudoge
ENSG0000	30.19608	0.203251	0.087158	2.331984	0.019702	0.036717	RP11-304	sense_ove
ENSG0000	30.19421	0.078436	0.042	1.86751	0.06183	0.101668	RP11-100	lincRNA
ENSG0000	30.17113	-0.24654	0.052557	-4.6909	2.72E-06	1.03E-05	PSMD6-A	antisense
ENSG0000	30.16094	-0.08952	0.050189	-1.78363	0.074483	0.119627	FAM21FP	pseudoge
ENSG0000	30.15992	-0.21057	0.060669	-3.47073	0.000519	0.001335	RP11-111	lincRNA
ENSG0000	30.13389	-0.0233	0.174705	-0.13338	0.893895	0.924631	IGKV3-11	IG_V_gene
ENSG0000	30.132	0.359548	0.097847	3.674576	0.000238	0.000652	APOC4-A	protein_cc
ENSG0000	30.11628	0.162869	0.0867	1.87852	0.06031	0.099514	TMEM249	protein_cc
ENSG0000	30.11272	0.026989	0.081104	0.332777	0.739303	0.802934	AC109829	protein_cc
ENSG0000	30.1101	0.050531	0.050897	0.992807	0.320804	0.414618	CHKB-AS1	antisense
ENSG0000	30.1091	2.233404	0.173652	12.86135	7.43E-38	8.04E-35	AL009178	protein_cc
ENSG0000	30.0994	0.295475	0.082077	3.599955	0.000318	0.000853	RP11-495	pseudoge
ENSG0000	30.08714	0.859514	0.100223	8.576026	9.82E-18	2.36E-16	HNRNPKP	pseudoge
ENSG0000	30.0712	-0.21551	0.063469	-3.39548	0.000685	0.001721	RP11-204	pseudoge
ENSG0000	30.05309	0.173579	0.096964	1.790135	0.073432	0.118161	XXyac-YX	antisense

ENSG0000(	30.05164	-0.0903	0.067115	-1.34542	0.178489	0.253547	RP11-134	lincRNA
ENSG0000(	30.05062	-0.12698	0.08507	-1.49262	0.135536	0.200424	LA16c-39(	antisense
ENSG0000(	30.00905	0.125136	0.078371	1.596725	0.110327	0.168152	RP11-274	sense_ove
ENSG0000(	29.98592	-1.23992	0.137597	-9.01124	2.04E-19	6.33E-18	RP11-766	lincRNA
ENSG0000(	29.97783	-0.57238	0.087282	-6.55782	5.46E-11	4.32E-10	LINC0062:	sense_ove
ENSG0000(	29.97648	0.480643	0.087907	5.467614	4.56E-08	2.28E-07	RP11-367.	lincRNA
ENSG0000(	29.97206	0.405274	0.074078	5.470893	4.48E-08	2.24E-07	FAM186A	protein_cc
ENSG0000(	29.96601	-0.31302	0.073332	-4.26845	1.97E-05	6.48E-05	TDRD10	protein_cc
ENSG0000(	29.95468	-1.87719	0.171684	-10.934	7.93E-28	1.17E-25	ANKRD34(	protein_cc
ENSG0000(	29.95224	0.648081	0.107426	6.032795	1.61E-09	1.02E-08	CD79B	protein_cc
ENSG0000(	29.95063	-0.98504	0.134278	-7.33587	2.20E-13	2.53E-12	SH3RF2	protein_cc
ENSG0000(	29.94686	0.356964	0.091425	3.904436	9.44E-05	0.000277	C11orf35	protein_cc
ENSG0000(	29.9442	0.029793	0.059173	0.503491	0.614619	0.697855	RP11-196(	sense_intr(
ENSG0000(	29.93248	0.101354	0.057602	1.759573	0.07848	0.125187	RP5-855D	sense_intr(
ENSG0000(	29.90782	0.033483	0.177608	0.188522	0.850467	0.891845	SCGB1D2	protein_cc
ENSG0000(	29.89074	-0.03725	0.054612	-0.68203	0.495219	0.589515	RP11-536(	antisense
ENSG0000(	29.87783	-0.30869	0.089824	-3.43664	0.000589	0.0015	LYPD3	protein_cc
ENSG0000(	29.87253	0.301625	0.091924	3.281242	0.001034	0.002511	RP11-353	lincRNA
ENSG0000(	29.86321	-0.27966	0.110348	-2.53429	0.011267	0.022252	RP11-422	protein_cc
ENSG0000(	29.85988	0.358678	0.104952	3.417539	0.000632	0.001599	KIAA1257	protein_cc
ENSG0000(	29.85392	0.830528	0.105504	7.872031	3.49E-15	5.51E-14	HSPD1P1	pseudoge
ENSG0000(	29.85251	0.037296	0.076662	0.486498	0.626614	0.708457	LINC0039:	lincRNA
ENSG0000(	29.84136	-0.18256	0.058151	-3.1394	0.001693	0.00396	CTD-2017	antisense
ENSG0000(	29.83607	0.796659	0.105457	7.554315	4.21E-14	5.46E-13	RPL35P2	pseudoge
ENSG0000(	29.82364	0.112801	0.058165	1.939325	0.052462	0.088137	RP11-295	sense_intr(
ENSG0000(	29.82176	0.22528	0.072279	3.11682	0.001828	0.004248	RP11-134	antisense
ENSG0000(	29.81216	0.059717	0.10294	0.58011	0.561841	0.650556	AP001172	lincRNA
ENSG0000(	29.80458	-0.1592	0.069351	-2.29553	0.021703	0.040086	TDRD12	protein_cc
ENSG0000(	29.79883	-0.51298	0.07338	-6.9907	2.74E-12	2.66E-11	RP11-840	antisense
ENSG0000(	29.76983	0.073426	0.070645	1.039358	0.298638	0.390906	RP11-548	lincRNA
ENSG0000(	29.76977	0.180721	0.061261	2.950036	0.003177	0.007045	RP11-803	lincRNA
ENSG0000(	29.7575	-0.07835	0.066989	-1.16953	0.242191	0.327896	RP11-77P	lincRNA
ENSG0000(	29.74581	0.389854	0.090068	4.328432	1.50E-05	5.04E-05	RP11-195	processed
ENSG0000(	29.74378	-0.33298	0.090336	-3.686	0.000228	0.000625	DHDH	protein_cc
ENSG0000(	29.73614	0.171976	0.092828	1.852628	0.063936	0.104717	SIGLEC5	protein_cc
ENSG0000(	29.70578	0.326491	0.071555	4.562787	5.05E-06	1.83E-05	AC010883	antisense
ENSG0000(	29.70455	0.52886	0.086365	6.123566	9.15E-10	6.00E-09	SNRPGP1!	pseudoge
ENSG0000(	29.68775	0.979012	0.110188	8.884885	6.40E-19	1.83E-17	C2orf66	protein_cc
ENSG0000(	29.68352	-0.96615	0.107666	-8.9736	2.87E-19	8.72E-18	TMEM236	protein_cc
ENSG0000(	29.67915	-0.02884	0.126846	-0.22738	0.820132	0.868084	PIGR	protein_cc
ENSG0000(	29.67144	-0.30066	0.116783	-2.5745	0.010038	0.020039	PCDHA12	protein_cc
ENSG0000(	29.65706	-0.05355	0.088876	-0.60249	0.54685	0.637253	LINC0085:	antisense
ENSG0000(	29.63298	-0.51368	0.091593	-5.60825	2.04E-08	1.08E-07	RAB6C	protein_cc
ENSG0000(	29.63112	0.066634	0.041103	1.621142	0.104987	0.161119	RP11-944	antisense
ENSG0000(	29.62963	0.369464	0.087537	4.220638	2.44E-05	7.89E-05	ST13P6	pseudoge
ENSG0000(	29.62164	0.156563	0.038701	4.045442	5.22E-05	0.00016	AC097500	lincRNA
ENSG0000(	29.61146	0.251239	0.077798	3.229372	0.001241	0.002973	RP11-382	lincRNA
ENSG0000(	29.60213	0.189168	0.056393	3.354473	0.000795	0.001975	AC002553	protein_cc
ENSG0000(	29.58305	0.193277	0.115578	1.672261	0.094473	0.147068	TTC29	protein_cc
ENSG0000(	29.57116	0.730906	0.136388	5.359031	8.37E-08	4.01E-07	PTCRA	protein_cc
ENSG0000(	29.55704	-0.00107	0.067037	-0.01597	0.987256	0.991209	CCDC116	protein_cc
ENSG0000(	29.53124	0.838371	0.112444	7.455931	8.92E-14	1.10E-12	SELV	protein_cc
ENSG0000(	29.5299	0.146899	0.059599	2.46479	0.013709	0.026557	RP11-316(	antisense
ENSG0000(	29.52974	-0.1607	0.075462	-2.12953	0.03321	0.058767	RP4-740C	sense_intr(
ENSG0000(	29.5244	-0.51148	0.102912	-4.97009	6.69E-07	2.78E-06	STAB2	protein_cc
ENSG0000(	29.51497	0.199977	0.061804	3.235646	0.001214	0.002913	RP11-104	antisense
ENSG0000(	29.4642	0.324017	0.077991	4.154518	3.26E-05	0.000103	YWHAZP3	pseudoge
ENSG0000(	29.45786	-0.203	0.108227	-1.8757	0.060696	0.100059	CD244	protein_cc
ENSG0000(	29.45117	-0.14329	0.084774	-1.69027	0.090976	0.142303	KB-208E9.	lincRNA

ENSG0000(	29.44874	-0.49169	0.238277	-2.06354	0.039062	0.067934	SMCP	protein_cc
ENSG0000(	29.43285	0.194156	0.072889	2.663711	0.007728	0.01584	SYNE4	protein_cc
ENSG0000(	29.42472	0.604773	0.133819	4.519328	6.20E-06	2.21E-05	FTH1P4	pseudoge
ENSG0000(	29.41751	0.222025	0.059975	3.701973	0.000214	0.00059	CTD-2619	antisense
ENSG0000(	29.40175	-0.35224	0.136302	-2.58423	0.00976	0.019544	TFAP2C	protein_cc
ENSG0000(	29.39941	0.776739	0.11507	6.750174	1.48E-11	1.28E-10	EIF2S3L	protein_cc
ENSG0000(	29.39872	0.094364	0.05354	1.762512	0.077983	0.124547	IL23A	protein_cc
ENSG0000(	29.3856	0.029902	0.083328	0.358841	0.719714	0.786708	RP11-656	antisense
ENSG0000(	29.38138	1.457101	0.198736	7.331837	2.27E-13	2.60E-12	TERT	protein_cc
ENSG0000(	29.3367	0.010589	0.058896	0.179789	0.857318	0.896971	CTD-2033	lincRNA
ENSG0000(	29.32844	-0.26558	0.061151	-4.34296	1.41E-05	4.74E-05	RP4-728D	antisense
ENSG0000(	29.32722	0.470235	0.098042	4.796238	1.62E-06	6.33E-06	XKR9	protein_cc
ENSG0000(	29.32617	-0.86791	0.165021	-5.25937	1.45E-07	6.67E-07	ABCC12	protein_cc
ENSG0000(	29.32182	0.690377	0.125279	5.510697	3.57E-08	1.82E-07	FCN3	protein_cc
ENSG0000(	29.3177	0.576597	0.14053	4.103024	4.08E-05	0.000127	RP11-347(	protein_cc
ENSG0000(	29.31571	0.335388	0.094829	3.536785	0.000405	0.001064	RNASE4	protein_cc
ENSG0000(	29.30992	-0.20949	0.058976	-3.55207	0.000382	0.001009	RP11-374	lincRNA
ENSG0000(	29.29791	0.454575	0.082584	5.50437	3.70E-08	1.88E-07	RP11-431	antisense
ENSG0000(	29.26476	-0.17118	0.125569	-1.36322	0.172814	0.246725	SIX2	protein_cc
ENSG0000(	29.26228	-0.45862	0.097694	-4.69448	2.67E-06	1.01E-05	NRG4	protein_cc
ENSG0000(	29.25873	-0.62481	0.147159	-4.24581	2.18E-05	7.11E-05	CCT7P2	pseudoge
ENSG0000(	29.25291	0.455409	0.069782	6.52618	6.75E-11	5.28E-10	AC093391	antisense
ENSG0000(	29.24612	-0.22295	0.077708	-2.86909	0.004117	0.008932	GOLGA6L!	pseudoge
ENSG0000(	29.22826	0.266067	0.089778	2.963599	0.003041	0.006766	AC002480	antisense
ENSG0000(	29.21421	-0.50422	0.070747	-7.12707	1.03E-12	1.06E-11	RP11-586	antisense
ENSG0000(	29.19239	0.192518	0.117118	1.643801	0.100217	0.154815	TNFRSF18	protein_cc
ENSG0000(	29.1918	-0.17937	0.079756	-2.24893	0.024517	0.044743	RP11-483	antisense
ENSG0000(	29.17458	-0.5296	0.177299	-2.98704	0.002817	0.006307	LBP	protein_cc
ENSG0000(	29.1645	1.704984	0.116721	14.60731	2.52E-48	7.98E-45	CTD-2035	lincRNA
ENSG0000(	29.15684	0.223987	0.093362	2.399121	0.016434	0.031249	POM121B	pseudoge
ENSG0000(	29.13097	-0.17533	0.085037	-2.06181	0.039225	0.06819	DLEU7-AS	antisense
ENSG0000(	29.12612	0.286843	0.05546	5.172083	2.31E-07	1.03E-06	RP11-231(	lincRNA
ENSG0000(	29.07911	-0.20636	0.043692	-4.72312	2.32E-06	8.86E-06	RP11-264	antisense
ENSG0000(	29.07667	0.714078	0.105947	6.739952	1.58E-11	1.36E-10	EEF1A1P2	pseudoge
ENSG0000(	29.07405	0.040629	0.048652	0.835093	0.403665	0.500536	RP11-64K	pseudoge
ENSG0000(	29.07374	0.290046	0.172258	1.683784	0.092223	0.143997	UPK3BL	protein_cc
ENSG0000(	29.06335	0.306969	0.061109	5.023291	5.08E-07	2.15E-06	RP11-179(	pseudoge
ENSG0000(	29.05647	-0.02414	0.064129	-0.37647	0.706565	0.776057	RP5-855D	antisense
ENSG0000(	29.04676	0.233573	0.047596	4.907419	9.23E-07	3.75E-06	ZNF197-A	antisense
ENSG0000(	29.04318	-0.3498	0.067265	-5.20033	1.99E-07	8.99E-07	RP11-258	lincRNA
ENSG0000(	29.03981	0.433327	0.076675	5.651466	1.59E-08	8.55E-08	PRRT3-AS	antisense
ENSG0000(	29.03684	0.928816	0.107731	8.62159	6.60E-18	1.63E-16	RNPS1P1	pseudoge
ENSG0000(	29.03277	-0.65342	0.1505	-4.34165	1.41E-05	4.76E-05	COL19A1	protein_cc
ENSG0000(	29.01898	0.318296	0.097162	3.275933	0.001053	0.002555	SMPDL3B	protein_cc
ENSG0000(	28.99785	0.875969	0.139382	6.284685	3.29E-10	2.31E-09	RPL37P2	pseudoge
ENSG0000(	28.99709	0.428369	0.062669	6.835366	8.18E-12	7.36E-11	C12orf61	protein_cc
ENSG0000(	28.99607	1.148747	0.153411	7.48802	6.99E-14	8.77E-13	C2orf70	protein_cc
ENSG0000(	28.97415	0.272932	0.059715	4.570588	4.86E-06	1.77E-05	BCL2L15	protein_cc
ENSG0000(	28.97035	-0.29599	0.091129	-3.24801	0.001162	0.002796	CTA-14H9	lincRNA
ENSG0000(	28.96728	-0.0716	0.084521	-0.84717	0.3969	0.493502	NUTM2B	protein_cc
ENSG0000(	28.954	1.324596	0.197442	6.708796	1.96E-11	1.66E-10	TAGLN2P1	pseudoge
ENSG0000(	28.95292	-0.18293	0.133829	-1.36691	0.171654	0.245346	CD28	protein_cc
ENSG0000(	28.94966	0.917086	0.156065	5.8763	4.20E-09	2.47E-08	C15orf26	protein_cc
ENSG0000(	28.92655	0.139462	0.033882	4.116153	3.85E-05	0.000121	TMED6	protein_cc
ENSG0000(	28.90916	-0.16486	0.055403	-2.97571	0.002923	0.006531	RP11-736	antisense
ENSG0000(	28.90859	0.204265	0.064852	3.149707	0.001634	0.003833	RP11-321	lincRNA
ENSG0000(	28.905	0.090532	0.046256	1.957172	0.050327	0.084986	RP11-307	lincRNA
ENSG0000(	28.90252	0.317214	0.077752	4.079804	4.51E-05	0.00014	CTD-3220	lincRNA
ENSG0000(	28.89847	0.411383	0.061399	6.70013	2.08E-11	1.76E-10	RP11-500(	antisense

ENSG0000(	28.89816	0.002607	0.061471	0.042403	0.966177	0.976747	DNLZ	protein_cc
ENSG0000(	28.89721	-0.63817	0.08773	-7.2742	3.48E-13	3.88E-12	CACNA1F	protein_cc
ENSG0000(	28.87914	-0.17819	0.072903	-2.44422	0.014517	0.027975	RP11-363.	lincRNA
ENSG0000(	28.87221	0.011462	0.056998	0.201095	0.840625	0.884117	RP11-314	lincRNA
ENSG0000(	28.86438	0.016434	0.07499	0.219154	0.82653	0.872859	ERAS	protein_cc
ENSG0000(	28.81283	-0.3707	0.08929	-4.15166	3.30E-05	0.000105	CILP2	protein_cc
ENSG0000(	28.75271	0.495573	0.161643	3.065846	0.002171	0.004974	AP000696	lincRNA
ENSG0000(	28.74542	0.375568	0.117386	3.199419	0.001377	0.003274	CLECL1	protein_cc
ENSG0000(	28.74511	0.076424	0.059476	1.284962	0.198805	0.277791	FAM129C	protein_cc
ENSG0000(	28.74314	-0.36608	0.088049	-4.15765	3.22E-05	0.000102	RP11-573	lincRNA
ENSG0000(	28.73517	0.009107	0.048929	0.18613	0.852343	0.893243	RP5-994D	lincRNA
ENSG0000(	28.72479	0.312635	0.07595	4.116341	3.85E-05	0.000121	ST13P5	pseudoge
ENSG0000(	28.72286	-1.49783	0.135016	-11.0938	1.34E-28	2.40E-26	RP11-497	lincRNA
ENSG0000(	28.71338	0.589955	0.085051	6.936444	4.02E-12	3.81E-11	RP11-110	lincRNA
ENSG0000(	28.70933	0.101848	0.102309	0.995498	0.319494	0.413198	VTCN1	protein_cc
ENSG0000(	28.69894	-0.0016	0.06945	-0.023	0.981654	0.987247	RP11-203.	processed
ENSG0000(	28.69228	0.787165	0.102046	7.7138	1.22E-14	1.74E-13	PTMAP4	pseudoge
ENSG0000(	28.68071	-0.11346	0.086401	-1.31317	0.189126	0.266275	AC004448	lincRNA
ENSG0000(	28.67999	0.537095	0.058104	9.243617	2.38E-20	8.56E-19	RP11-204	antisense
ENSG0000(	28.66933	0.151229	0.07888	1.917214	0.055211	0.092165	RP11-285.	antisense
ENSG0000(	28.6592	-0.11897	0.054495	-2.18319	0.029022	0.052086	RP11-98D	antisense
ENSG0000(	28.65545	-1.24205	0.15601	-7.96135	1.70E-15	2.83E-14	CLEC4G	protein_cc
ENSG0000(	28.65041	0.063565	0.094296	0.674098	0.500249	0.594178	WNT6	protein_cc
ENSG0000(	28.61443	-1.352	0.171281	-7.89348	2.94E-15	4.70E-14	PAX7	protein_cc
ENSG0000(	28.60512	-0.26873	0.082939	-3.24006	0.001195	0.00287	CHRFAM7	protein_cc
ENSG0000(	28.60163	0.047515	0.107535	0.441859	0.658591	0.735836	RP4-607I7	antisense
ENSG0000(	28.58305	-0.20971	0.077402	-2.70938	0.006741	0.013979	AC115522	lincRNA
ENSG0000(	28.55562	-0.25209	0.113166	-2.22759	0.025908	0.047033	IZUMO1	protein_cc
ENSG0000(	28.52593	0.285293	0.06601	4.321978	1.55E-05	5.18E-05	MEF2B	protein_cc
ENSG0000(	28.52477	-0.0345	0.051736	-0.66687	0.504854	0.59868	LA16c-42!	sense_ove
ENSG0000(	28.51373	0.012451	0.0823	0.151286	0.87975	0.914101	CEP170P1	pseudoge
ENSG0000(	28.50183	-0.45192	0.095719	-4.72135	2.34E-06	8.93E-06	WDR86-A	processed
ENSG0000(	28.49964	0.60387	0.070837	8.524752	1.53E-17	3.55E-16	RP11-61A	lincRNA
ENSG0000(	28.49509	-0.49179	0.103948	-4.73111	2.23E-06	8.54E-06	RP11-764	pseudoge
ENSG0000(	28.46117	-0.01669	0.051406	-0.32464	0.745456	0.808038	CTC-510F	antisense
ENSG0000(	28.44944	-0.10656	0.102902	-1.03551	0.300431	0.392893	ANKRD18	pseudoge
ENSG0000(	28.44909	-0.04256	0.061427	-0.69286	0.488399	0.583067	GUSBP4	pseudoge
ENSG0000(	28.42607	-1.40725	0.157036	-8.96134	3.21E-19	9.63E-18	RP11-215	lincRNA
ENSG0000(	28.42574	-0.27721	0.12951	-2.14049	0.032315	0.057343	NRAP	protein_cc
ENSG0000(	28.41879	0.319008	0.126866	2.514521	0.011919	0.023403	SDR42E2	protein_cc
ENSG0000(	28.41213	-0.19229	0.097965	-1.96279	0.049671	0.084015	RP11-396	lincRNA
ENSG0000(	28.39565	0.213666	0.118626	1.801183	0.071674	0.115657	ZNF492	protein_cc
ENSG0000(	28.38375	-0.55863	0.105105	-5.315	1.07E-07	5.04E-07	C2orf71	protein_cc
ENSG0000(	28.37788	0.715508	0.081618	8.76656	1.84E-18	4.93E-17	CTC-246B	lincRNA
ENSG0000(	28.37041	0.394736	0.096858	4.075405	4.59E-05	0.000142	RP4-569M	antisense
ENSG0000(	28.36984	-0.25193	0.099477	-2.53258	0.011323	0.022347	RP13-616	lincRNA
ENSG0000(	28.35338	0.04814	0.09199	0.52332	0.600752	0.685463	GS1-393G	protein_cc
ENSG0000(	28.35324	0.204451	0.066056	3.09513	0.001967	0.004544	GS1-124K	lincRNA
ENSG0000(	28.3463	-0.76368	0.134061	-5.69656	1.22E-08	6.69E-08	MT-TL1	Mt_tRNA
ENSG0000(	28.33085	0.154136	0.14266	1.080443	0.279945	0.370461	CXCR6	protein_cc
ENSG0000(	28.30677	0.942351	0.135859	6.936259	4.03E-12	3.81E-11	RP11-61L	pseudoge
ENSG0000(	28.3064	-0.04006	0.044422	-0.90174	0.367192	0.463317	RP5-1000	pseudoge
ENSG0000(	28.2941	0.133447	0.080495	1.657829	0.097352	0.150927	CA5A	protein_cc
ENSG0000(	28.28516	0.153951	0.068784	2.238175	0.02521	0.045881	RP11-762	pseudoge
ENSG0000(	28.26474	0.290302	0.086954	3.338573	0.000842	0.002081	AP003025	antisense
ENSG0000(	28.25388	0.322775	0.057238	5.639209	1.71E-08	9.14E-08	RP11-61L	sense_intr
ENSG0000(	28.2311	0.02317	0.071986	0.321873	0.747549	0.809815	RP11-399	lincRNA
ENSG0000(	28.22342	0.177588	0.072449	2.451211	0.014238	0.027487	AHCTF1P1	pseudoge
ENSG0000(	28.21421	-0.17764	0.046388	-3.82942	0.000128	0.000368	RP11-320	pseudoge

ENSG0000(	28.21062	-0.081	0.054122	-1.49656	0.134508	0.199119	RP11-88I1	antisense
ENSG0000(	28.19004	0.502465	0.09266	5.422684	5.87E-08	2.89E-07	RP1-159A	pseudoge
ENSG0000(	28.18414	0.205217	0.06477	3.168392	0.001533	0.003615	SCARNA1!	processed
ENSG0000(	28.17505	-0.4298	0.119314	-3.60227	0.000315	0.000846	TRIM54	protein_cc
ENSG0000(	28.16207	0.446591	0.093833	4.759444	1.94E-06	7.50E-06	PROC	protein_cc
ENSG0000(	28.16145	-0.48728	0.079892	-6.09932	1.07E-09	6.92E-09	AC087163	pseudoge
ENSG0000(	28.14361	0.06986	0.110743	0.63083	0.528152	0.619855	RP11-745	antisense
ENSG0000(	28.1236	0.367545	0.085244	4.311675	1.62E-05	5.41E-05	FER1L5	pseudoge
ENSG0000(	28.11687	0.008409	0.141831	0.05929	0.952721	0.967304	CYP4B1	protein_cc
ENSG0000(	28.11304	-0.02435	0.047557	-0.51212	0.608567	0.692302	RP11-17M	antisense
ENSG0000(	28.10434	0.379991	0.099293	3.826957	0.00013	0.000371	KLRB1	protein_cc
ENSG0000(	28.08726	-0.35649	0.130247	-2.73701	0.0062	0.012953	RP11-150	lincRNA
ENSG0000(	28.07452	0.221025	0.04943	4.471465	7.77E-06	2.73E-05	RP11-48B	pseudoge
ENSG0000(	28.07156	0.004906	0.104345	0.04702	0.962498	0.97432	MYO18B	protein_cc
ENSG0000(	28.06535	-0.25884	0.086827	-2.98107	0.002872	0.006424	RP11-379	antisense
ENSG0000(	28.06119	-1.1479	0.126312	-9.08777	1.01E-19	3.30E-18	BARX2	protein_cc
ENSG0000(	28.0409	-0.7324	0.131946	-5.55074	2.84E-08	1.47E-07	HOXD1	protein_cc
ENSG0000(	28.04032	0.26686	0.050962	5.236423	1.64E-07	7.49E-07	AC135048	processed
ENSG0000(	28.0365	-0.29976	0.113571	-2.63937	0.008306	0.016906	RP11-521	lincRNA
ENSG0000(	28.02627	-0.91579	0.120133	-7.62317	2.48E-14	3.34E-13	RP11-5P1	pseudoge
ENSG0000(	28.01942	-1.15657	0.140523	-8.23051	1.86E-16	3.62E-15	SLC27A2	protein_cc
ENSG0000(	28.00523	-0.07275	0.054941	-1.32406	0.185484	0.261855	RP1-191J1	antisense
ENSG0000(	27.9958	0.006368	0.076695	0.083033	0.933825	0.953818	RP11-388	antisense
ENSG0000(	27.99446	-0.06227	0.089064	-0.69918	0.484442	0.579454	RP11-723	protein_cc
ENSG0000(	27.9926	0.446277	0.143671	3.106242	0.001895	0.004391	TACSTD2	protein_cc
ENSG0000(	27.99023	-0.17489	0.079003	-2.21369	0.02685	0.048559	CTC-498M	lincRNA
ENSG0000(	27.97824	0.839909	0.12784	6.57	5.03E-11	4.01E-10	RP11-887	pseudoge
ENSG0000(	27.96282	-0.61266	0.072997	-8.39294	4.74E-17	1.02E-15	RP11-574	antisense
ENSG0000(	27.95866	-0.0768	0.08567	-0.89648	0.369999	0.466329	AC092667	antisense
ENSG0000(	27.89468	-0.07275	0.071	-1.02461	0.305548	0.398507	RP11-496	lincRNA
ENSG0000(	27.88016	-0.14209	0.099097	-1.43389	0.151604	0.220841	TREH	protein_cc
ENSG0000(	27.86914	0.478092	0.06509	7.345052	2.06E-13	2.37E-12	RPL23AP2	pseudoge
ENSG0000(	27.84521	-0.617	0.153832	-4.01088	6.05E-05	0.000184	AC007163	lincRNA
ENSG0000(	27.84454	-0.26803	0.085198	-3.14601	0.001655	0.003878	PRR26	protein_cc
ENSG0000(	27.8367	0.803246	0.127468	6.301566	2.95E-10	2.09E-09	CTC-297N	lincRNA
ENSG0000(	27.82734	0.376342	0.130118	2.892312	0.003824	0.008348	RP11-475.	pseudoge
ENSG0000(	27.82067	-0.02107	0.068742	-0.30653	0.7592	0.818942	AS3MT	protein_cc
ENSG0000(	27.81622	0.745296	0.134434	5.543971	2.96E-08	1.52E-07	ACTBP7	pseudoge
ENSG0000(	27.79953	0.959136	0.13812	6.944218	3.81E-12	3.62E-11	RP11-372	pseudoge
ENSG0000(	27.79507	-0.06637	0.069155	-0.95976	0.337174	0.432028	XXcos-LU(	antisense
ENSG0000(	27.76557	0.196041	0.040494	4.841226	1.29E-06	5.13E-06	C2orf15	protein_cc
ENSG0000(	27.73609	0.407643	0.074599	5.464426	4.64E-08	2.32E-07	RP11-10C	lincRNA
ENSG0000(	27.71315	0.176309	0.067163	2.625083	0.008663	0.017556	RBM44	protein_cc
ENSG0000(	27.70891	0.536304	0.073898	7.257322	3.95E-13	4.36E-12	GLDCP1	pseudoge
ENSG0000(	27.69867	0.106994	0.095548	1.1198	0.262799	0.351378	NFE2	protein_cc
ENSG0000(	27.69378	0.679936	0.105152	6.466228	1.00E-10	7.65E-10	EPHX3	protein_cc
ENSG0000(	27.66573	0.073812	0.058837	1.254508	0.209657	0.290624	RP11-60I3	antisense
ENSG0000(	27.66197	0.230205	0.054728	4.206353	2.60E-05	8.37E-05	AC016292	pseudoge
ENSG0000(	27.6017	0.203128	0.101301	2.005185	0.044943	0.076866	IL21R-AS1	antisense
ENSG0000(	27.58911	0.437158	0.066435	6.580251	4.70E-11	3.76E-10	CTD-2201	lincRNA
ENSG0000(	27.58318	-0.44487	0.111038	-4.00646	6.16E-05	0.000187	DUOX2	protein_cc
ENSG0000(	27.56647	-0.71026	0.11814	-6.01206	1.83E-09	1.15E-08	CYP26A1	protein_cc
ENSG0000(	27.55846	0.113287	0.072207	1.568929	0.116665	0.176484	RBP5	protein_cc
ENSG0000(	27.55518	0.357048	0.079031	4.517821	6.25E-06	2.23E-05	RPS24P8	pseudoge
ENSG0000(	27.54886	0.272462	0.106951	2.547552	0.010848	0.021507	RP4-562J1	lincRNA
ENSG0000(	27.5384	-0.2029	0.08664	-2.34184	0.019189	0.035871	RP11-113	lincRNA
ENSG0000(	27.52972	-0.01204	0.072443	-0.16614	0.868047	0.905409	TAS2R20	protein_cc
ENSG0000(	27.52534	0.038987	0.072659	0.536583	0.591556	0.677183	AP001437	antisense
ENSG0000(	27.52082	0.183277	0.055818	3.283494	0.001025	0.002493	RP11-482	antisense

ENSG0000(	27.51388	0.35565	0.100941	3.523345	0.000426	0.001115	RP13-20L:	lincRNA
ENSG0000(	27.51378	0.054372	0.083908	0.647989	0.516992	0.60975	PLAC8L1	protein_cc
ENSG0000(	27.5065	0.071657	0.131326	0.545644	0.585311	0.671642	OR7A5	protein_cc
ENSG0000(	27.50629	0.081345	0.083523	0.97392	0.330096	0.424427	SLA2	protein_cc
ENSG0000(	27.48924	0.757084	0.104665	7.233423	4.71E-13	5.13E-12	EIF2S2P4	pseudoge
ENSG0000(	27.46575	0.608677	0.102744	5.924213	3.14E-09	1.89E-08	BNIP3P1	pseudoge
ENSG0000(	27.45838	1.820348	0.150779	12.07292	1.47E-33	7.19E-31	HGD	protein_cc
ENSG0000(	27.45763	-0.50079	0.112712	-4.44312	8.87E-06	3.09E-05	RP11-66B	lincRNA
ENSG0000(	27.45505	1.179435	0.117465	10.04072	1.01E-23	6.95E-22	CLEC18A	protein_cc
ENSG0000(	27.45268	0.157176	0.098105	1.602123	0.109128	0.166548	TTC16	protein_cc
ENSG0000(	27.44556	0.080048	0.08255	0.969688	0.332202	0.426828	RBM22P2	pseudoge
ENSG0000(	27.43538	-0.13655	0.054323	-2.51359	0.011951	0.023456	RP11-96K	antisense
ENSG0000(	27.40582	0.229183	0.080948	2.831231	0.004637	0.009939	SPACA6P-	lincRNA
ENSG0000(	27.39825	0.303603	0.068364	4.44098	8.96E-06	3.11E-05	RP11-298	antisense
ENSG0000(	27.37242	-0.60111	0.134458	-4.47057	7.80E-06	2.74E-05	FUT3	protein_cc
ENSG0000(	27.36885	0.377225	0.109117	3.457077	0.000546	0.001399	CTB-51J2:	antisense
ENSG0000(	27.36201	0.228678	0.08226	2.779944	0.005437	0.011502	LL09NC01	lincRNA
ENSG0000(	27.34981	-0.07315	0.084972	-0.8609	0.389294	0.485912	RP1-159A	antisense
ENSG0000(	27.34875	0.064293	0.069455	0.925675	0.354615	0.45021	CMTM2	protein_cc
ENSG0000(	27.34797	-0.0679	0.095916	-0.70788	0.479021	0.5744	GRAP2	protein_cc
ENSG0000(	27.34673	-0.78249	0.17509	-4.46909	7.86E-06	2.76E-05	SELE	protein_cc
ENSG0000(	27.31103	0.139975	0.070422	1.987662	0.046849	0.079738	AC241952	protein_cc
ENSG0000(	27.30544	0.084541	0.142509	0.59323	0.553027	0.642733	AC131097	protein_cc
ENSG0000(	27.29189	0.039721	0.097626	0.406868	0.684105	0.757244	LINC0090	lincRNA
ENSG0000(	27.29023	-0.07562	0.064889	-1.16544	0.24384	0.329911	GLUD1P2	pseudoge
ENSG0000(	27.27548	0.012431	0.0628	0.197953	0.843082	0.886067	RP11-802	antisense
ENSG0000(	27.2702	0.192462	0.063889	3.012442	0.002592	0.005855	RP11-152	antisense
ENSG0000(	27.26823	-0.56783	0.157764	-3.59926	0.000319	0.000855	RP11-245.	processed
ENSG0000(	27.26319	-0.12792	0.048609	-2.6317	0.008496	0.017257	KB-1410C	lincRNA
ENSG0000(	27.25529	0.322553	0.061606	5.235761	1.64E-07	7.52E-07	AC011290	pseudoge
ENSG0000(	27.25387	0.367431	0.062666	5.863328	4.54E-09	2.65E-08	RP11-166	lincRNA
ENSG0000(	27.25221	0.247277	0.049666	4.97876	6.40E-07	2.67E-06	CATSPER3	protein_cc
ENSG0000(	27.23645	-0.20074	0.07953	-2.52409	0.0116	0.022841	RP11-756	pseudoge
ENSG0000(	27.22907	0.999376	0.180408	5.539544	3.03E-08	1.56E-07	CTD-2316	lincRNA
ENSG0000(	27.19281	0.437667	0.129711	3.374187	0.00074	0.001847	GZMB	protein_cc
ENSG0000(	27.18888	0.250806	0.058959	4.253883	2.10E-05	6.89E-05	GHRLOS	antisense
ENSG0000(	27.16036	0.227649	0.050723	4.488091	7.19E-06	2.54E-05	SNURF	protein_cc
ENSG0000(	27.14069	0.051302	0.076981	0.666418	0.505144	0.598937	CACNA1C	antisense
ENSG0000(	27.13686	-0.31232	0.07895	-3.95595	7.62E-05	0.000227	RP1-265C	pseudoge
ENSG0000(	27.1113	0.010778	0.075071	0.143567	0.885842	0.918704	DUTP6	pseudoge
ENSG0000(	27.10978	-0.50384	0.104772	-4.80891	1.52E-06	5.96E-06	AC018804	pseudoge
ENSG0000(	27.09486	-0.06066	0.080278	-0.75559	0.449897	0.54672	RARRES2F	pseudoge
ENSG0000(	27.09081	0.053943	0.059052	0.913492	0.360984	0.456815	RP11-347	antisense
ENSG0000(	27.0844	0.619219	0.095894	6.457326	1.07E-10	8.10E-10	RP11-286	pseudoge
ENSG0000(	27.08125	0.967634	0.143458	6.745075	1.53E-11	1.32E-10	PKMP1	pseudoge
ENSG0000(	27.06997	0.007743	0.056995	0.135862	0.89193	0.923153	CTA-292E	antisense
ENSG0000(	27.06263	0.863475	0.125245	6.894302	5.41E-12	5.00E-11	RP11-807	lincRNA
ENSG0000(	27.05128	0.044436	0.061328	0.724571	0.468716	0.564553	CTD-3138	sense_intr
ENSG0000(	27.04616	-0.07659	0.099047	-0.77331	0.439341	0.536222	LGALS4	protein_cc
ENSG0000(	27.04351	-0.06944	0.054325	-1.27816	0.201192	0.280725	SEC14L1P:	pseudoge
ENSG0000(	27.03058	-0.89837	0.139484	-6.44069	1.19E-10	8.97E-10	CAPN13	protein_cc
ENSG0000(	27.02018	-1.30225	0.142203	-9.1577	5.30E-20	1.82E-18	RP11-247	protein_cc
ENSG0000(	27.01646	0.665942	0.08602	7.741675	9.81E-15	1.43E-13	C17orf82	protein_cc
ENSG0000(	27.00455	1.507605	0.180487	8.352975	6.66E-17	1.39E-15	RP11-565	antisense
ENSG0000(	26.99873	-0.22388	0.223349	-1.00236	0.316171	0.409803	MMP1	protein_cc
ENSG0000(	26.99277	0.032981	0.073339	0.449703	0.652925	0.731153	RP11-218	lincRNA
ENSG0000(	26.98847	0.072938	0.067428	1.081716	0.279379	0.369807	CTC-550B	antisense
ENSG0000(	26.98079	0.408651	0.127414	3.207264	0.00134	0.003193	ADAMTS7	pseudoge
ENSG0000(	26.96985	0.082092	0.075541	1.086713	0.277164	0.367372	RP11-809	antisense

ENSG0000(	26.96642	0.433302	0.115169	3.762321	0.000168	0.000473	DNAI2	protein_co
ENSG0000(	26.95876	0.04571	0.103856	0.440124	0.659847	0.736899	RP11-806	antisense
ENSG0000(	26.95043	0.801138	0.118961	6.73448	1.65E-11	1.41E-10	NR2F2-AS	antisense
ENSG0000(	26.94364	-0.56366	0.1106	-5.09639	3.46E-07	1.51E-06	ANKRD2	protein_co
ENSG0000(	26.93663	-0.0114	0.065965	-0.17285	0.862772	0.901095	ZNRF2P1	pseudoge
ENSG0000(	26.9306	0.383839	0.100791	3.808281	0.00014	0.000398	RP11-503	protein_co
ENSG0000(	26.92148	0.094732	0.067612	1.40112	0.161178	0.232506	RP11-468	protein_co
ENSG0000(	26.90551	-0.40714	0.13088	-3.11078	0.001866	0.004328	GDF6	protein_co
ENSG0000(	26.87354	-0.11438	0.083085	-1.37668	0.168611	0.241669	RP4-777D	antisense
ENSG0000(	26.86196	-1.74034	0.161305	-10.7891	3.87E-27	5.14E-25	CALHM1	protein_co
ENSG0000(	26.85895	0.415274	0.063107	6.580508	4.69E-11	3.76E-10	RP11-672	lincRNA
ENSG0000(	26.85894	0.106458	0.068397	1.55646	0.119599	0.180174	CTD-2203	antisense
ENSG0000(	26.85385	1.790327	0.298183	6.004122	1.92E-09	1.20E-08	HOXA6	protein_co
ENSG0000(	26.8507	0.134469	0.083931	1.602142	0.109124	0.166547	RP11-713	antisense
ENSG0000(	26.85016	0.181789	0.065882	2.759308	0.005792	0.012176	EEF1G	protein_co
ENSG0000(	26.84143	0.28238	0.082454	3.424711	0.000615	0.001561	SNORD14	snoRNA
ENSG0000(	26.81333	0.72398	0.155359	4.660036	3.16E-06	1.18E-05	RP11-806	lincRNA
ENSG0000(	26.80439	-0.35937	0.059744	-6.01512	1.80E-09	1.13E-08	KIF28P	pseudoge
ENSG0000(	26.80067	-0.59201	0.133414	-4.43735	9.11E-06	3.16E-05	HAR1B	lincRNA
ENSG0000(	26.79062	0.097801	0.055081	1.775592	0.0758	0.121439	ACSM3	protein_co
ENSG0000(	26.78757	-0.78989	0.233121	-3.38832	0.000703	0.001763	SLC18A3	protein_co
ENSG0000(	26.78069	-0.32491	0.063143	-5.14559	2.67E-07	1.18E-06	RP11-326	sense_intr
ENSG0000(	26.75465	-0.09962	0.065549	-1.51981	0.128559	0.191622	RP5-1073	antisense
ENSG0000(	26.7526	-0.16557	0.09766	-1.6954	0.09	0.141023	RP4-669P	pseudoge
ENSG0000(	26.75	-0.04237	0.138201	-0.30662	0.759134	0.818914	MS4A4E	protein_co
ENSG0000(	26.74453	-0.58177	0.07564	-7.69131	1.46E-14	2.05E-13	RP11-350	antisense
ENSG0000(	26.73624	-0.16545	0.095176	-1.7384	0.082141	0.130126	RP11-373	lincRNA
ENSG0000(	26.72661	0.017589	0.047032	0.373983	0.708417	0.777583	RP11-15L	antisense
ENSG0000(	26.71884	-0.00489	0.061702	-0.07919	0.936881	0.955895	CHST5	protein_co
ENSG0000(	26.71622	1.52612	0.153954	9.912805	3.66E-23	2.22E-21	FBXO39	protein_co
ENSG0000(	26.71412	0.402574	0.085551	4.705683	2.53E-06	9.60E-06	SLC7A9	protein_co
ENSG0000(	26.71313	0.213057	0.063011	3.381277	0.000721	0.001805	RP11-91J1	antisense
ENSG0000(	26.71244	0.376482	0.113575	3.314827	0.000917	0.00225	RP5-1120	antisense
ENSG0000(	26.70848	0.06156	0.053714	1.146081	0.251762	0.339007	RP11-345	pseudoge
ENSG0000(	26.70498	0.134053	0.106392	1.259994	0.207672	0.288328	AC002398	lincRNA
ENSG0000(	26.69487	-0.16695	0.096221	-1.7351	0.082723	0.130923	RP11-381	antisense
ENSG0000(	26.69332	0.867106	0.135245	6.411353	1.44E-10	1.07E-09	AC005592	antisense
ENSG0000(	26.68395	2.122165	0.255764	8.297369	1.06E-16	2.15E-15	MTHFD2P	pseudoge
ENSG0000(	26.67663	-0.55243	0.068734	-8.03724	9.19E-16	1.59E-14	VN1R20P	pseudoge
ENSG0000(	26.67293	0.416485	0.068533	6.077162	1.22E-09	7.87E-09	HSD17B13	protein_co
ENSG0000(	26.66797	0.090479	0.052536	1.722245	0.085025	0.134174	RP3-400B	lincRNA
ENSG0000(	26.65908	-0.05581	0.066481	-0.83943	0.401227	0.49804	RP11-181	pseudoge
ENSG0000(	26.65639	-0.0049	0.067906	-0.07221	0.942432	0.959725	LMOD3	protein_co
ENSG0000(	26.65226	0.026465	0.052223	0.506772	0.612314	0.695776	RP11-347	pseudoge
ENSG0000(	26.65084	-0.09861	0.088401	-1.11554	0.264619	0.353364	RBMS3-AS	antisense
ENSG0000(	26.63883	1.908575	0.277591	6.875488	6.18E-12	5.67E-11	HOXA-AS	antisense
ENSG0000(	26.62555	0.014036	0.074085	0.189452	0.849739	0.891423	NAMA	lincRNA
ENSG0000(	26.62247	0.120415	0.086839	1.386651	0.165548	0.237975	POU5F1B	protein_co
ENSG0000(	26.62027	3.122084	0.212931	14.66245	1.12E-48	3.84E-45	MAB21L2	protein_co
ENSG0000(	26.61714	0.507125	0.253761	1.998439	0.045669	0.077978	KRT75	protein_co
ENSG0000(	26.60713	-0.08509	0.098118	-0.86724	0.385813	0.482489	DQX1	protein_co
ENSG0000(	26.60548	0.492792	0.063724	7.733233	1.05E-14	1.52E-13	RP1-178F	antisense
ENSG0000(	26.59121	0.205525	0.135141	1.520817	0.128306	0.191293	GOLT1A	protein_co
ENSG0000(	26.57775	0.26157	0.165744	1.578155	0.11453	0.173702	CCL20	protein_co
ENSG0000(	26.57583	-0.30874	0.075198	-4.10562	4.03E-05	0.000126	C3orf20	protein_co
ENSG0000(	26.57543	-0.05582	0.088065	-0.6338	0.52621	0.61799	CACNA1G	antisense
ENSG0000(	26.57295	-0.12284	0.042519	-2.88893	0.003866	0.008432	RP11-894	protein_co
ENSG0000(	26.56641	0.531552	0.090553	5.870092	4.36E-09	2.56E-08	AC074391	lincRNA
ENSG0000(	26.56563	-0.92914	0.106328	-8.73845	2.36E-18	6.23E-17	SPAG6	protein_co

ENSG0000(	26.54766	0.484675	0.085039	5.699462	1.20E-08	6.59E-08	RP11-714	lincRNA
ENSG0000(	26.54365	-0.35969	0.110906	-3.24324	0.001182	0.00284	RP11-122	antisense
ENSG0000(	26.53766	-0.66515	0.133796	-4.97139	6.65E-07	2.77E-06	RS1	protein_cc
ENSG0000(	26.53079	-0.12572	0.054385	-2.31159	0.0208	0.038569	LINC0111	protein_cc
ENSG0000(	26.52588	0.092798	0.05616	1.652369	0.098459	0.152443	RP11-264	antisense
ENSG0000(	26.5249	0.51286	0.083459	6.145064	7.99E-10	5.29E-09	RP11-215.	pseudoge
ENSG0000(	26.50933	-0.01562	0.07374	-0.21181	0.832253	0.877602	LINC0060	lincRNA
ENSG0000(	26.4704	-0.16764	0.120404	-1.39235	0.163818	0.235842	AZU1	protein_cc
ENSG0000(	26.43835	0.193174	0.042875	4.505518	6.62E-06	2.35E-05	CTC-512J	pseudoge
ENSG0000(	26.4336	0.053909	0.042279	1.275073	0.202284	0.282019	RP11-195	antisense
ENSG0000(	26.42882	0.327891	0.071089	4.6124	3.98E-06	1.46E-05	FAM92A1I	pseudoge
ENSG0000(	26.41058	-0.21343	0.057476	-3.71341	0.000204	0.000566	CTD-2245	lincRNA
ENSG0000(	26.40668	-0.61131	0.118249	-5.16967	2.35E-07	1.05E-06	AC093590	lincRNA
ENSG0000(	26.39558	-0.18907	0.065686	-2.87842	0.003997	0.008695	CTB-113P	lincRNA
ENSG0000(	26.39494	0.324464	0.065862	4.926429	8.37E-07	3.42E-06	RP11-166	lincRNA
ENSG0000(	26.38658	-0.92586	0.125882	-7.35495	1.91E-13	2.21E-12	ADTRP	protein_cc
ENSG0000(	26.38579	0.92484	0.118294	7.818181	5.36E-15	8.20E-14	RP11-466	pseudoge
ENSG0000(	26.36037	-0.25979	0.084402	-3.07797	0.002084	0.004794	DOCK9-A	antisense
ENSG0000(	26.35698	0.180954	0.099995	1.80962	0.070355	0.113805	RP11-259	lincRNA
ENSG0000(	26.34481	0.213368	0.047669	4.476066	7.60E-06	2.68E-05	RP5-1024	antisense
ENSG0000(	26.33797	0.549455	0.081854	6.712596	1.91E-11	1.62E-10	AC069513	lincRNA
ENSG0000(	26.31187	0.189941	0.052269	3.633909	0.000279	0.000755	RP11-119	antisense
ENSG0000(	26.30431	0.845681	0.125556	6.735465	1.63E-11	1.40E-10	RP11-364	pseudoge
ENSG0000(	26.29098	-0.51166	0.123896	-4.12974	3.63E-05	0.000114	LINC0057	lincRNA
ENSG0000(	26.27786	-0.33787	0.095433	-3.54035	0.0004	0.001051	ZBTB20-A	lincRNA
ENSG0000(	26.27098	0.581479	0.176706	3.290664	0.001	0.002434	HOXD4	protein_cc
ENSG0000(	26.25877	-0.02308	0.059368	-0.38879	0.69743	0.768354	ADAM21	protein_cc
ENSG0000(	26.25217	-1.5836	0.168817	-9.38057	6.56E-21	2.59E-19	DRD5	protein_cc
ENSG0000(	26.24025	0.711811	0.095315	7.468021	8.14E-14	1.01E-12	RP11-267.	pseudoge
ENSG0000(	26.23746	0.002881	0.043199	0.066697	0.946823	0.963052	NHEJ1	protein_cc
ENSG0000(	26.23339	0.265325	0.04692	5.654844	1.56E-08	8.40E-08	CNBD2	protein_cc
ENSG0000(	26.22326	-0.23162	0.052129	-4.4432	8.86E-06	3.08E-05	LINC0056	lincRNA
ENSG0000(	26.22168	-0.00569	0.121646	-0.0468	0.962676	0.974477	METTL24	protein_cc
ENSG0000(	26.20729	0.08206	0.094838	0.86527	0.386891	0.483675	B3GNT8	protein_cc
ENSG0000(	26.18287	0.192274	0.105425	1.823789	0.068184	0.110712	RP11-553.	antisense
ENSG0000(	26.15429	0.065812	0.05894	1.11659	0.26417	0.352924	RP11-350	lincRNA
ENSG0000(	26.14864	-0.15896	0.105698	-1.50389	0.132609	0.196762	RHD	protein_cc
ENSG0000(	26.14657	0.234278	0.047812	4.899932	9.59E-07	3.88E-06	RP11-580	antisense
ENSG0000(	26.12413	0.091986	0.054912	1.675146	0.093906	0.146313	RP11-96K	antisense
ENSG0000(	26.11418	0.513389	0.050105	10.24623	1.23E-24	1.00E-22	ZNF20	protein_cc
ENSG0000(	26.10773	0.225413	0.074532	3.024383	0.002491	0.005649	LAT	protein_cc
ENSG0000(	26.10419	-0.811	0.106728	-7.59876	2.99E-14	3.97E-13	ALOXE3	protein_cc
ENSG0000(	26.10034	1.02311	0.15233	6.716422	1.86E-11	1.59E-10	RPL34P18	pseudoge
ENSG0000(	26.09603	0.666358	0.099383	6.704921	2.02E-11	1.71E-10	RPL7AP11	pseudoge
ENSG0000(	26.08038	0.707477	0.147813	4.7863	1.70E-06	6.62E-06	LINC0048	lincRNA
ENSG0000(	26.07379	0.290889	0.066338	4.384988	1.16E-05	3.96E-05	C8G	protein_cc
ENSG0000(	26.07242	0.109373	0.061435	1.780292	0.075028	0.120371	RP11-529	pseudoge
ENSG0000(	26.06563	-0.62137	0.101242	-6.13742	8.39E-10	5.53E-09	RP11-365	sense_intr
ENSG0000(	26.04619	-0.44038	0.114091	-3.85992	0.000113	0.000328	RP11-569	lincRNA
ENSG0000(	26.04238	-0.7334	0.147764	-4.96332	6.93E-07	2.87E-06	RP11-886	lincRNA
ENSG0000(	26.0369	0.404902	0.087688	4.617529	3.88E-06	1.43E-05	PDE6G	protein_cc
ENSG0000(	26.02865	0.794136	0.094528	8.401044	4.43E-17	9.53E-16	RP11-146	pseudoge
ENSG0000(	26.0277	-0.25865	0.102853	-2.51473	0.011912	0.023393	RP11-372	antisense
ENSG0000(	26.02603	0.408044	0.156099	2.614	0.008949	0.018072	RP11-148	lincRNA
ENSG0000(	26.01759	0.120675	0.07857	1.535891	0.124565	0.186547	CTC-325H	pseudoge
ENSG0000(	26.00227	0.090122	0.051712	1.742782	0.081372	0.129082	AL357515	miRNA
ENSG0000(	26.00189	0.101206	0.075541	1.339752	0.180326	0.255618	AC006042	antisense
ENSG0000(	25.97555	-0.83562	0.142166	-5.87773	4.16E-09	2.45E-08	SGCZ	protein_cc
ENSG0000(	25.96919	-0.00244	0.14998	-0.01628	0.987007	0.991068	CXCR1	protein_cc

ENSG0000(	25.95605	0.272558	0.134235	2.03046	0.04231	0.072899	GZMH	protein_cc
ENSG0000(	25.93708	-0.03537	0.062854	-0.56281	0.573566	0.66092	CD160	protein_cc
ENSG0000(	25.93278	-0.06448	0.057712	-1.11725	0.263887	0.35258	C10orf111	protein_cc
ENSG0000(	25.93081	0.092074	0.094803	0.971208	0.331445	0.425946	RP11-302	lincRNA
ENSG0000(	25.92566	-0.47151	0.086313	-5.46286	4.69E-08	2.34E-07	SMTNL2	protein_cc
ENSG0000(	25.91282	0.087914	0.160911	0.546348	0.584827	0.671274	NLRP4	protein_cc
ENSG0000(	25.8984	0.025425	0.084944	0.299309	0.764704	0.823475	CCND2-A	antisense
ENSG0000(	25.88713	0.267698	0.063125	4.240726	2.23E-05	7.26E-05	AC079305	antisense
ENSG0000(	25.87757	-0.20227	0.074803	-2.70405	0.00685	0.014184	AC005932	antisense
ENSG0000(	25.86565	0.528574	0.062405	8.470075	2.45E-17	5.54E-16	RPL10P15	pseudoge
ENSG0000(	25.86426	-1.3296	0.183223	-7.25672	3.97E-13	4.38E-12	KLHL1	protein_cc
ENSG0000(	25.84447	-0.44271	0.095358	-4.64267	3.44E-06	1.28E-05	PRLR	protein_cc
ENSG0000(	25.84323	1.04564	0.114229	9.15392	5.49E-20	1.88E-18	SUMO2P1	pseudoge
ENSG0000(	25.84172	-0.05945	0.059501	-0.99915	0.317724	0.411415	RP11-325	sense_intr
ENSG0000(	25.82843	-0.31654	0.099095	-3.19432	0.001402	0.003328	RP11-728	pseudoge
ENSG0000(	25.82686	1.01884	0.127529	7.989084	1.36E-15	2.30E-14	AC022210	pseudoge
ENSG0000(	25.82574	0.436641	0.048628	8.97919	2.73E-19	8.32E-18	CTD-2342	antisense
ENSG0000(	25.82307	0.064762	0.068626	0.943691	0.345328	0.440732	RP11-234	lincRNA
ENSG0000(	25.77156	0.996388	0.1389	7.173397	7.32E-13	7.77E-12	AGTR1	protein_cc
ENSG0000(	25.76328	0.369701	0.092894	3.979812	6.90E-05	0.000207	RP11-54D	pseudoge
ENSG0000(	25.75047	0.775909	0.167879	4.621849	3.80E-06	1.40E-05	TECTB	protein_cc
ENSG0000(	25.7454	0.05358	0.050121	1.069011	0.285065	0.375882	RP11-33B	pseudoge
ENSG0000(	25.74321	0.538125	0.085538	6.291052	3.15E-10	2.22E-09	RP11-368	protein_cc
ENSG0000(	25.73818	2.269712	0.193408	11.73533	8.40E-32	2.68E-29	AC002454	antisense
ENSG0000(	25.73488	0.189563	0.081245	2.333224	0.019636	0.036611	RP11-15J1	pseudoge
ENSG0000(	25.71675	0.160719	0.046281	3.472701	0.000515	0.001326	UXT-AS1	antisense
ENSG0000(	25.71386	-0.15183	0.07297	-2.08076	0.037456	0.065468	AC136289	pseudoge
ENSG0000(	25.71379	0.594092	0.109794	5.410953	6.27E-08	3.07E-07	RP11-159	lincRNA
ENSG0000(	25.70921	-0.07664	0.085231	-0.89918	0.368556	0.464866	RN7SL138	misc_RNA
ENSG0000(	25.70838	1.160385	0.137642	8.430446	3.44E-17	7.55E-16	RP3-340B	pseudoge
ENSG0000(	25.70244	0.718911	0.074846	9.605199	7.60E-22	3.55E-20	RPS27P25	pseudoge
ENSG0000(	25.69974	0.114597	0.056913	2.013542	0.044058	0.075537	YIPF7	protein_cc
ENSG0000(	25.67745	0.425209	0.107925	3.939841	8.15E-05	0.000242	ARHGAP1	protein_cc
ENSG0000(	25.67651	-0.2204	0.108503	-2.03125	0.042229	0.072778	CCL22	protein_cc
ENSG0000(	25.67273	0.342511	0.098331	3.483233	0.000495	0.001279	SYCE3	protein_cc
ENSG0000(	25.66494	0.84002	0.13243	6.343126	2.25E-10	1.62E-09	EEF1A1P1	pseudoge
ENSG0000(	25.65335	-0.08995	0.065328	-1.37689	0.168548	0.241612	DACT3-AS	antisense
ENSG0000(	25.61845	-0.4851	0.090147	-5.38122	7.40E-08	3.58E-07	RP11-752	antisense
ENSG0000(	25.61839	-0.16871	0.102121	-1.65206	0.098522	0.152535	RP11-423	antisense
ENSG0000(	25.61438	0.806556	0.143723	5.611894	2.00E-08	1.06E-07	ACTBP11	pseudoge
ENSG0000(	25.60383	0.904301	0.120266	7.519157	5.51E-14	7.01E-13	RPL19P12	pseudoge
ENSG0000(	25.59654	0.705314	0.070749	9.969304	2.08E-23	1.35E-21	RP11-298	pseudoge
ENSG0000(	25.58801	0.310786	0.09369	3.317176	0.000909	0.002232	AC009299	pseudoge
ENSG0000(	25.58512	0.79793	0.102679	7.7711	7.78E-15	1.16E-13	PGK1P2	pseudoge
ENSG0000(	25.56341	-0.48533	0.11859	-4.09249	4.27E-05	0.000133	KCNH1-IT	sense_intr
ENSG0000(	25.54855	-0.76449	0.118392	-6.4573	1.07E-10	8.10E-10	GPR150	protein_cc
ENSG0000(	25.5428	-0.10112	0.082617	-1.22393	0.220979	0.303657	RP11-930	lincRNA
ENSG0000(	25.53173	-0.08127	0.062434	-1.30166	0.193033	0.270949	RP11-316	antisense
ENSG0000(	25.52603	-0.08032	0.06481	-1.23927	0.215246	0.297149	RP11-177.	lincRNA
ENSG0000(	25.52198	0.003986	0.054178	0.073577	0.941347	0.959095	RP11-574	antisense
ENSG0000(	25.52114	-0.07902	0.064248	-1.22993	0.218724	0.301077	TEX21P	pseudoge
ENSG0000(	25.5112	-0.2585	0.046752	-5.52912	3.22E-08	1.65E-07	RP11-697	protein_cc
ENSG0000(	25.50851	-0.08503	0.072686	-1.16981	0.242079	0.327788	RAET1G	protein_cc
ENSG0000(	25.50134	-0.5833	0.173706	-3.35798	0.000785	0.001951	C20orf166	antisense
ENSG0000(	25.49649	0.462445	0.070908	6.521759	6.95E-11	5.43E-10	MIR25	miRNA
ENSG0000(	25.49527	-0.13786	0.120573	-1.1434	0.252873	0.340242	CCR7	protein_cc
ENSG0000(	25.49426	-1.51183	0.185633	-8.14419	3.82E-16	7.06E-15	HTR1E	protein_cc
ENSG0000(	25.49036	0.103095	0.049969	2.063168	0.039097	0.067984	AC007292	antisense
ENSG0000(	25.45892	0.211238	0.093599	2.256833	0.024019	0.043925	RP11-598	lincRNA

ENSG0000(	25.45762	-0.47066	0.135976	-3.46135	0.000537	0.001379	RP11-373	sense_intr(
ENSG0000(	25.44096	0.641657	0.081649	7.858724	3.88E-15	6.09E-14	GNAS-AS:	antisense
ENSG0000(	25.41899	0.366247	0.062748	5.836826	5.32E-09	3.08E-08	AC005498	lincRNA
ENSG0000(	25.40967	0.348316	0.085472	4.075185	4.60E-05	0.000142	RP11-686	pseudoge
ENSG0000(	25.40881	0.523118	0.110737	4.723948	2.31E-06	8.83E-06	RP11-118	lincRNA
ENSG0000(	25.39004	0.731096	0.104244	7.013331	2.33E-12	2.29E-11	TUBB2BP1	pseudoge
ENSG0000(	25.37828	0.186363	0.090118	2.06798	0.038642	0.067298	AC000068	antisense
ENSG0000(	25.37272	1.714816	0.172028	9.968235	2.10E-23	1.36E-21	RP11-19E:	lincRNA
ENSG0000(	25.36754	-0.10071	0.092147	-1.09292	0.274428	0.364441	ADPGK-A:	antisense
ENSG0000(	25.36356	0.154165	0.162456	0.94896	0.342641	0.43783	ANKUB1	protein_cc
ENSG0000(	25.3583	-0.53676	0.143453	-3.74175	0.000183	0.00051	ANKRD36I	pseudoge
ENSG0000(	25.3543	-0.75119	0.11373	-6.60502	3.97E-11	3.22E-10	HBQ1	protein_cc
ENSG0000(	25.35142	-0.05663	0.066037	-0.8576	0.391111	0.487691	CTB-31N1	sense_intr(
ENSG0000(	25.32769	-0.1685	0.071932	-2.34258	0.019151	0.035805	RP11-348	antisense
ENSG0000(	25.31944	0.312265	0.09887	3.158326	0.001587	0.00373	TNNI2	protein_cc
ENSG0000(	25.30988	0.094782	0.094901	0.99874	0.317921	0.411616	OTOA	protein_cc
ENSG0000(	25.30348	-0.33706	0.111219	-3.03056	0.002441	0.005545	NGF	protein_cc
ENSG0000(	25.30269	-0.05112	0.047552	-1.07506	0.282349	0.373018	RP11-26J:	sense_intr(
ENSG0000(	25.29087	1.108877	0.144029	7.698973	1.37E-14	1.94E-13	H3F3AP6	pseudoge
ENSG0000(	25.28889	-0.0638	0.043335	-1.47222	0.140962	0.207336	FAM185BI	pseudoge
ENSG0000(	25.2865	0.012578	0.06888	0.182603	0.855109	0.895312	RP11-347(	sense_intr(
ENSG0000(	25.24863	0.203016	0.073796	2.751065	0.00594	0.012455	RP11-736	lincRNA
ENSG0000(	25.24107	0.104658	0.064818	1.614643	0.106388	0.162946	RP11-434	protein_cc
ENSG0000(	25.23948	0.967172	0.093294	10.36697	3.50E-25	3.18E-23	RP11-448	lincRNA
ENSG0000(	25.23508	0.04101	0.056925	0.720427	0.471262	0.567168	RP11-314	antisense
ENSG0000(	25.23251	-0.4948	0.152585	-3.24278	0.001184	0.002844	PAPL	protein_cc
ENSG0000(	25.22115	0.062548	0.111499	0.560978	0.574813	0.662075	TGM3	protein_cc
ENSG0000(	25.20658	-0.17253	0.126302	-1.36604	0.171925	0.245666	P2RY2	protein_cc
ENSG0000(	25.20054	-0.03313	0.087319	-0.37938	0.704408	0.774362	XXbac-BP	lincRNA
ENSG0000(	25.19981	0.132174	0.062818	2.104058	0.035373	0.062214	RP11-646	lincRNA
ENSG0000(	25.19286	0.006916	0.177222	0.039025	0.96887	0.978444	FCER2	protein_cc
ENSG0000(	25.18623	0.245036	0.066468	3.68654	0.000227	0.000624	RP3-522D	lincRNA
ENSG0000(	25.17276	-0.00481	0.053869	-0.08933	0.928819	0.949962	FBXW11P:	pseudoge
ENSG0000(	25.16621	0.415196	0.056326	7.371255	1.69E-13	1.98E-12	GTF2IRD2	pseudoge
ENSG0000(	25.15882	-0.21455	0.07166	-2.99405	0.002753	0.006177	RP11-849	protein_cc
ENSG0000(	25.15668	-0.24893	0.088395	-2.81616	0.00486	0.010378	KLHL31	protein_cc
ENSG0000(	25.15229	-0.03605	0.050489	-0.71408	0.475178	0.571041	RP11-111.	lincRNA
ENSG0000(	25.15022	0.175802	0.073126	2.404083	0.016213	0.030884	LA16c-32!	lincRNA
ENSG0000(	25.14917	0.021483	0.114361	0.187848	0.850996	0.892241	TAC4	protein_cc
ENSG0000(	25.14648	0.020569	0.10889	0.188894	0.850176	0.891631	AC006042	pseudoge
ENSG0000(	25.14575	-0.24758	0.132538	-1.86799	0.061763	0.101577	PCDHA8	protein_cc
ENSG0000(	25.14467	0.959467	0.106163	9.037715	1.60E-19	5.06E-18	AC016734	pseudoge
ENSG0000(	25.1438	0.7669	0.094395	8.124332	4.50E-16	8.23E-15	EIF1AXP1	pseudoge
ENSG0000(	25.13641	0.894607	0.111286	8.038788	9.07E-16	1.57E-14	RP6-105D	pseudoge
ENSG0000(	25.12949	0.366201	0.068254	5.365231	8.08E-08	3.88E-07	RP11-727	antisense
ENSG0000(	25.12307	-0.1616	0.111047	-1.45525	0.1456	0.213236	RP11-339	pseudoge
ENSG0000(	25.11341	0.095538	0.071544	1.335364	0.181757	0.257367	AD000684	sense_intr(
ENSG0000(	25.11275	-0.18407	0.079781	-2.30713	0.021048	0.038988	RNY3P8	misc_RNA
ENSG0000(	25.10747	-0.21855	0.068455	-3.19266	0.00141	0.003345	PHBP9	pseudoge
ENSG0000(	25.10396	0.093549	0.052925	1.767564	0.077134	0.12334	MIS18A-A	antisense
ENSG0000(	25.10285	-0.3121	0.060554	-5.15408	2.55E-07	1.13E-06	PDZD9	protein_cc
ENSG0000(	25.09892	0.954196	0.133707	7.136489	9.57E-13	9.99E-12	HSPA8P5	pseudoge
ENSG0000(	25.0937	1.217775	0.131976	9.227282	2.78E-20	9.83E-19	AC007682	lincRNA
ENSG0000(	25.09036	-0.08281	0.095561	-0.86656	0.386186	0.482897	SRMS	protein_cc
ENSG0000(	25.08104	0.069053	0.065763	1.05002	0.293709	0.385451	WEE2	protein_cc
ENSG0000(	25.07979	0.117721	0.074422	1.581806	0.113694	0.172606	RP11-383	lincRNA
ENSG0000(	25.07424	0.345088	0.109129	3.162217	0.001566	0.003685	RP11-255.	pseudoge
ENSG0000(	25.06623	0.423191	0.099255	4.263698	2.01E-05	6.61E-05	RP11-620.	lincRNA
ENSG0000(	25.06185	0.570193	0.113601	5.019249	5.19E-07	2.19E-06	LRRC71	protein_cc

ENSG0000(	25.0567	-0.04553	0.064684	-0.70392	0.481481	0.576734	RP11-532	antisense
ENSG0000(	25.05355	-0.00692	0.118774	-0.05827	0.953535	0.967886	MGC1095	protein_cc
ENSG0000(	25.05334	-1.23908	0.127261	-9.73653	2.11E-22	1.09E-20	EPHA8	protein_cc
ENSG0000(	25.05299	-0.18987	0.084067	-2.25854	0.023912	0.043748	NPM1P25	pseudoge
ENSG0000(	25.04465	0.243823	0.070827	3.442525	0.000576	0.00147	RP11-637.	lincRNA
ENSG0000(	25.03915	-1.49375	0.254607	-5.86691	4.44E-09	2.60E-08	LMX1A	protein_cc
ENSG0000(	25.03525	0.506306	0.076935	6.580914	4.68E-11	3.75E-10	RP11-893	antisense
ENSG0000(	25.02357	0.302073	0.051837	5.827404	5.63E-09	3.24E-08	ZHX1-C8(	protein_cc
ENSG0000(	25.02295	-0.14381	0.090217	-1.59407	0.11092	0.168962	RP11-757	lincRNA
ENSG0000(	25.01966	-0.27414	0.091241	-3.00453	0.00266	0.005994	RP11-359.	lincRNA
ENSG0000(	25.00902	-0.19291	0.089464	-2.15626	0.031063	0.055341	GAL3ST2	protein_cc
ENSG0000(	24.9701	0.124686	0.055017	2.266328	0.023431	0.042943	AL121761	protein_cc
ENSG0000(	24.94607	-0.08964	0.046468	-1.92897	0.053734	0.089989	RP1-30M3	antisense
ENSG0000(	24.93767	-0.26696	0.128577	-2.07626	0.03787	0.066113	AC092162	lincRNA
ENSG0000(	24.9312	0.49064	0.101318	4.842585	1.28E-06	5.09E-06	GS1-44D2	pseudoge
ENSG0000(	24.92665	-0.04521	0.049564	-0.9122	0.361663	0.457548	RP13-131	antisense
ENSG0000(	24.87974	-0.08397	0.137832	-0.60919	0.542401	0.633146	OR7D2	protein_cc
ENSG0000(	24.87933	0.386963	0.085182	4.542776	5.55E-06	2.00E-05	RP11-686	processed
ENSG0000(	24.87793	-0.00166	0.071049	-0.02333	0.981384	0.987025	C6orf163	protein_cc
ENSG0000(	24.86632	-0.0898	0.074761	-1.20113	0.2297	0.31367	RP1-168L	antisense
ENSG0000(	24.86586	-0.29879	0.085975	-3.47535	0.00051	0.001314	CYP2D8P	pseudoge
ENSG0000(	24.85788	1.091046	0.14499	7.524956	5.27E-14	6.73E-13	RP11-516.	pseudoge
ENSG0000(	24.85328	0.294975	0.113366	2.601978	0.009269	0.018655	RP11-220	antisense
ENSG0000(	24.85082	2.622075	0.299983	8.740737	2.32E-18	6.12E-17	HOXB8	protein_cc
ENSG0000(	24.84987	1.563974	0.156829	9.972513	2.01E-23	1.31E-21	AC010729	lincRNA
ENSG0000(	24.84039	0.075595	0.053527	1.412284	0.157867	0.228378	HCG17	lincRNA
ENSG0000(	24.83917	0.009373	0.052253	0.17938	0.857639	0.897101	RP11-74E	antisense
ENSG0000(	24.83281	-0.221	0.075056	-2.94448	0.003235	0.007162	AC005042	antisense
ENSG0000(	24.82978	-0.15719	0.065443	-2.40196	0.016307	0.031043	CTD-2373	sense_ove
ENSG0000(	24.82942	-0.00664	0.065063	-0.10211	0.918669	0.942624	RP11-465	antisense
ENSG0000(	24.8229	-0.59947	0.13067	-4.58767	4.48E-06	1.64E-05	CACNA1S	protein_cc
ENSG0000(	24.81708	0.061057	0.068951	0.885508	0.375883	0.472398	CTC-325H	lincRNA
ENSG0000(	24.81272	-0.39373	0.089364	-4.40595	1.05E-05	3.62E-05	RP11-71E	lincRNA
ENSG0000(	24.80235	0.167109	0.081912	2.040102	0.04134	0.071429	SLC22A18	protein_cc
ENSG0000(	24.79627	0.182794	0.059576	3.068269	0.002153	0.004937	STAG3L1	pseudoge
ENSG0000(	24.77852	-0.01141	0.107914	-0.10578	0.915759	0.940669	AC007405	antisense
ENSG0000(	24.77844	0.170081	0.074046	2.296967	0.021621	0.039946	TAS2R19	protein_cc
ENSG0000(	24.76244	0.358206	0.094361	3.796112	0.000147	0.000417	RP11-60A	lincRNA
ENSG0000(	24.74215	-0.07925	0.071201	-1.11307	0.265677	0.354512	RP11-230	lincRNA
ENSG0000(	24.74164	0.156873	0.122439	1.281237	0.200111	0.279397	FAM3B	protein_cc
ENSG0000(	24.73517	-0.00537	0.061328	-0.08757	0.930222	0.951106	RP1-266L	sense_intr
ENSG0000(	24.73302	0.365353	0.083321	4.384891	1.16E-05	3.96E-05	RP11-26J3	lincRNA
ENSG0000(	24.71431	0.147988	0.058135	2.545593	0.010909	0.021618	RP3-388M	antisense
ENSG0000(	24.71265	-0.57479	0.16007	-3.59084	0.00033	0.000881	RP11-673	lincRNA
ENSG0000(	24.70532	0.453242	0.061504	7.369262	1.72E-13	2.01E-12	GS1-257G	pseudoge
ENSG0000(	24.70519	-0.08225	0.087729	-0.93756	0.348469	0.443955	FBXW10	protein_cc
ENSG0000(	24.6891	1.335032	0.171251	7.795746	6.40E-15	9.67E-14	SCNN1G	protein_cc
ENSG0000(	24.68787	-0.10513	0.050743	-2.07178	0.038286	0.066771	AP000487	antisense
ENSG0000(	24.68341	0.930624	0.12122	7.67713	1.63E-14	2.27E-13	EIF4A1P2	pseudoge
ENSG0000(	24.67037	-0.92936	0.108541	-8.56232	1.11E-17	2.62E-16	PNLDC1	protein_cc
ENSG0000(	24.66775	0.903046	0.194157	4.651106	3.30E-06	1.23E-05	RP11-893	antisense
ENSG0000(	24.62784	-0.05067	0.055759	-0.90872	0.363496	0.459471	RP11-531	antisense
ENSG0000(	24.62589	0.149734	0.16047	0.933097	0.35077	0.446237	AC093802	protein_cc
ENSG0000(	24.61586	-0.20446	0.087927	-2.32535	0.020053	0.037309	C20orf197	protein_cc
ENSG0000(	24.6132	-0.59321	0.114371	-5.18671	2.14E-07	9.62E-07	RP5-1039	antisense
ENSG0000(	24.58397	0.377972	0.066963	5.644476	1.66E-08	8.88E-08	RP11-473	sense_ove
ENSG0000(	24.58382	0.160367	0.07352	2.181265	0.029164	0.052316	CAPN14	protein_cc
ENSG0000(	24.57026	0.011863	0.105893	0.112023	0.910805	0.936938	ZNF695	protein_cc
ENSG0000(	24.55063	0.008949	0.10301	0.086876	0.93077	0.951525	RP11-556	antisense

ENSG0000(	24.52807	-0.23504	0.10317	-2.27815	0.022718	0.041782	CTA-268H	lincRNA
ENSG0000(	24.51556	0.488533	0.08513	5.738696	9.54E-09	5.30E-08	RP4-798A	lincRNA
ENSG0000(	24.50992	-0.02595	0.040401	-0.6424	0.520611	0.612911	SYNJ2BP-(	processed
ENSG0000(	24.5083	-0.20025	0.073564	-2.7221	0.006487	0.013498	RP11-188(	pseudoge
ENSG0000(	24.49871	-0.25067	0.094903	-2.64135	0.008258	0.016821	RP11-864	antisense
ENSG0000(	24.49641	0.324084	0.093501	3.466094	0.000528	0.001357	EDARADD	protein_cc
ENSG0000(	24.45472	-0.40685	0.114831	-3.54299	0.000396	0.001042	KCNH6	protein_cc
ENSG0000(	24.45442	-0.07654	0.080805	-0.94726	0.343504	0.438717	MIR143HC	lincRNA
ENSG0000(	24.44956	-0.33576	0.105305	-3.18842	0.001431	0.00339	PROSER2-	antisense
ENSG0000(	24.44368	-1.33737	0.154816	-8.63847	5.70E-18	1.42E-16	OCA2	protein_cc
ENSG0000(	24.44256	-0.05065	0.072671	-0.697	0.485801	0.580674	RPL23AP6	pseudoge
ENSG0000(	24.44003	-0.03987	0.069164	-0.57646	0.564305	0.652618	ADAM20P	lincRNA
ENSG0000(	24.43772	0.239962	0.086263	2.78174	0.005407	0.011445	ANKRD7	protein_cc
ENSG0000(	24.42145	-0.0855	0.059662	-1.43306	0.151842	0.221148	MAGOH2	pseudoge
ENSG0000(	24.38605	1.236218	0.161142	7.671585	1.70E-14	2.35E-13	RPS19P1	pseudoge
ENSG0000(	24.38449	-0.04077	0.069021	-0.59072	0.554709	0.644292	RP11-137(	antisense
ENSG0000(	24.37958	0.528617	0.123515	4.279789	1.87E-05	6.19E-05	RP11-48F:	lincRNA
ENSG0000(	24.37046	-0.01515	0.071242	-0.21262	0.831626	0.87712	RP11-388(	lincRNA
ENSG0000(	24.36544	0.463652	0.070492	6.57738	4.79E-11	3.83E-10	RP11-791(	lincRNA
ENSG0000(	24.35594	-0.21515	0.146967	-1.46396	0.143205	0.210197	EGFL6	protein_cc
ENSG0000(	24.34809	-0.13032	0.250219	-0.52083	0.602487	0.686829	IGKV1-5	IG_V_gene
ENSG0000(	24.3307	-0.46301	0.113251	-4.08833	4.34E-05	0.000135	RCVRN	protein_cc
ENSG0000(	24.31768	-0.03416	0.050567	-0.67555	0.499323	0.59325	XXbac-B4	antisense
ENSG0000(	24.31114	-1.61832	0.15594	-10.3778	3.13E-25	2.86E-23	FRMPD2P:	pseudoge
ENSG0000(	24.31014	-0.42032	0.121471	-3.46023	0.00054	0.001384	MEP1A	protein_cc
ENSG0000(	24.28621	0.245065	0.113596	2.157334	0.03098	0.055208	RP11-305	pseudoge
ENSG0000(	24.26845	-0.24524	0.157479	-1.55728	0.119405	0.179966	GUCA1A	protein_cc
ENSG0000(	24.2666	-0.30941	0.080566	-3.84045	0.000123	0.000353	CTA-212D	antisense
ENSG0000(	24.26376	0.345814	0.064663	5.347939	8.90E-08	4.25E-07	RP13-582(	antisense
ENSG0000(	24.26176	0.271672	0.071156	3.817974	0.000135	0.000384	C12orf79	protein_cc
ENSG0000(	24.25282	-0.28065	0.053949	-5.20223	1.97E-07	8.90E-07	FAM228A	protein_cc
ENSG0000(	24.24909	0.201501	0.077282	2.607347	0.009125	0.018386	LGALS2	protein_cc
ENSG0000(	24.24909	0.132262	0.083262	1.588503	0.112173	0.17058	RP11-473	lincRNA
ENSG0000(	24.23614	0.282586	0.13841	2.041653	0.041186	0.07121	GSTT2	protein_cc
ENSG0000(	24.23047	0.294241	0.067409	4.36503	1.27E-05	4.31E-05	RP11-435(	antisense
ENSG0000(	24.22642	0.975833	0.10047	9.712714	2.66E-22	1.36E-20	AC004797	pseudoge
ENSG0000(	24.22241	0.361561	0.138145	2.617256	0.008864	0.01792	RP11-747	sense_intr
ENSG0000(	24.21871	-0.34509	0.076002	-4.54046	5.61E-06	2.02E-05	RP11-307(	antisense
ENSG0000(	24.21349	0.541803	0.075	7.224048	5.05E-13	5.48E-12	RP3-337H	pseudoge
ENSG0000(	24.2045	0.246737	0.04775	5.167277	2.38E-07	1.06E-06	OSGEPL1-	antisense
ENSG0000(	24.2034	-0.03404	0.081652	-0.41688	0.676764	0.751073	RP11-175	lincRNA
ENSG0000(	24.18472	-0.33734	0.085746	-3.93414	8.35E-05	0.000248	BAAT	protein_cc
ENSG0000(	24.17209	-0.12968	0.111077	-1.16751	0.243003	0.328876	RP11-54C	antisense
ENSG0000(	24.16997	-0.40187	0.099102	-4.05506	5.01E-05	0.000154	RP11-364	lincRNA
ENSG0000(	24.16844	-0.33077	0.09722	-3.40226	0.000668	0.001683	CYP24A1	protein_cc
ENSG0000(	24.15473	0.189849	0.134355	1.413034	0.157646	0.228147	MGC4294	protein_cc
ENSG0000(	24.13252	0.717588	0.116387	6.165506	7.03E-10	4.69E-09	AC005682	processed
ENSG0000(	24.13192	0.097729	0.100332	0.974059	0.330027	0.424385	RP3-369A	lincRNA
ENSG0000(	24.11936	0.041294	0.081731	0.505242	0.613389	0.696823	RP11-17E:	lincRNA
ENSG0000(	24.11809	0.188019	0.063294	2.970548	0.002973	0.006632	RP11-438	lincRNA
ENSG0000(	24.10443	-0.09369	0.097323	-0.96264	0.335729	0.430545	RP11-309	lincRNA
ENSG0000(	24.10056	-0.13687	0.06586	-2.0782	0.037691	0.065827	TSSK3	protein_cc
ENSG0000(	24.08976	-0.35826	0.075594	-4.73924	2.15E-06	8.23E-06	IMPG1	protein_cc
ENSG0000(	24.08642	0.329644	0.133705	2.465456	0.013684	0.02651	ST7-AS2	antisense
ENSG0000(	24.07195	0.091243	0.051803	1.761345	0.07818	0.124799	RP11-178(	lincRNA
ENSG0000(	24.0579	0.161271	0.053506	3.014059	0.002578	0.005827	AC005229	miRNA
ENSG0000(	24.05483	-0.1539	0.075162	-2.04761	0.040598	0.070312	MATN1	protein_cc
ENSG0000(	24.05088	0.108847	0.080461	1.352797	0.176121	0.250676	ABCC6P2	pseudoge
ENSG0000(	24.04353	-0.32257	0.139574	-2.31112	0.020826	0.038612	CA15P1	pseudoge

ENSG0000(	24.02713	0.094518	0.063477	1.48901	0.136485	0.201657	GUSBP2	pseudoge
ENSG0000(	24.01512	0.081945	0.118263	0.692908	0.488367	0.583058	CD300LB	protein_cc
ENSG0000(	24.00608	-0.23502	0.171813	-1.3679	0.171344	0.244988	COX6CP1	pseudoge
ENSG0000(	24.00447	0.617972	0.103041	5.997326	2.01E-09	1.25E-08	RPL23P8	pseudoge
ENSG0000(	24.00215	-0.0489	0.069498	-0.70368	0.481633	0.576865	RP11-471	antisense
ENSG0000(	23.996	0.503335	0.132589	3.796199	0.000147	0.000417	GFI1B	protein_cc
ENSG0000(	23.98853	-0.43764	0.094112	-4.65019	3.32E-06	1.23E-05	OR2H2	protein_cc
ENSG0000(	23.98553	0.379358	0.086155	4.403195	1.07E-05	3.67E-05	RP11-293.	antisense
ENSG0000(	23.9682	-0.20738	0.149332	-1.38869	0.164927	0.237199	AGAP7	protein_cc
ENSG0000(	23.96316	-0.10952	0.078759	-1.39051	0.164374	0.236511	RP3-388E.	antisense
ENSG0000(	23.96196	-0.20699	0.08803	-2.35139	0.018703	0.035049	RP1-130H	protein_cc
ENSG0000(	23.9603	0.089727	0.047188	1.901461	0.057242	0.0951	AP001462	lincRNA
ENSG0000(	23.94657	-0.75853	0.099917	-7.59163	3.16E-14	4.18E-13	TUBA8	protein_cc
ENSG0000(	23.94581	0.001411	0.110284	0.012796	0.989791	0.993002	ACTR3C	protein_cc
ENSG0000(	23.94488	0.284524	0.052289	5.441424	5.29E-08	2.62E-07	CITF22-1A	lincRNA
ENSG0000(	23.94467	0.251251	0.054648	4.59767	4.27E-06	1.56E-05	RP11-533	lincRNA
ENSG0000(	23.93812	0.053335	0.188308	0.283233	0.776999	0.833523	TMC3	protein_cc
ENSG0000(	23.93506	0.098593	0.130335	0.756458	0.449375	0.546182	RP11-317	lincRNA
ENSG0000(	23.92534	-1.05064	0.136848	-7.67741	1.62E-14	2.26E-13	AC012593	lincRNA
ENSG0000(	23.92433	-0.38152	0.08934	-4.27047	1.95E-05	6.43E-05	AKR1E2	protein_cc
ENSG0000(	23.92082	0.52093	0.096024	5.42498	5.80E-08	2.85E-07	PHEX-AS1	antisense
ENSG0000(	23.91944	-0.34466	0.106445	-3.23793	0.001204	0.002891	CCDC26	lincRNA
ENSG0000(	23.91132	2.022293	0.24661	8.200376	2.40E-16	4.59E-15	VGLL2	protein_cc
ENSG0000(	23.89847	0.429422	0.081975	5.238468	1.62E-07	7.42E-07	CSNK2A3	protein_cc
ENSG0000(	23.89473	-0.68069	0.119875	-5.67834	1.36E-08	7.39E-08	RP11-13A	lincRNA
ENSG0000(	23.89288	-0.21168	0.075632	-2.79884	0.005129	0.010908	RP4-734G	antisense
ENSG0000(	23.89044	-0.92409	0.12068	-7.65737	1.90E-14	2.62E-13	RP11-930	antisense
ENSG0000(	23.87971	0.165378	0.087825	1.883032	0.059696	0.098643	HIST2H2B.	pseudoge
ENSG0000(	23.87943	-0.06307	0.064082	-0.98422	0.325008	0.419222	CORIN	protein_cc
ENSG0000(	23.86839	-0.00334	0.09454	-0.03537	0.971782	0.980485	HIST4H4	protein_cc
ENSG0000(	23.83429	-1.2957	0.114433	-11.3228	1.01E-29	2.12E-27	IL12RB2	protein_cc
ENSG0000(	23.82857	0.176934	0.087337	2.025883	0.042777	0.073608	TMC2	protein_cc
ENSG0000(	23.80435	-0.08808	0.204683	-0.43032	0.666959	0.742965	CCDC129	protein_cc
ENSG0000(	23.79443	-0.04627	0.084203	-0.54946	0.582692	0.669384	RP11-271	lincRNA
ENSG0000(	23.79225	0.699428	0.1502	4.656653	3.21E-06	1.20E-05	VWA5B1	protein_cc
ENSG0000(	23.79119	0.298717	0.047291	6.316548	2.67E-10	1.91E-09	RP11-422	antisense
ENSG0000(	23.78988	-0.07367	0.111983	-0.6579	0.510604	0.603792	CTGLF12P	pseudoge
ENSG0000(	23.78374	0.31473	0.074429	4.228586	2.35E-05	7.63E-05	RP11-44N	processed
ENSG0000(	23.7817	-0.24969	0.060793	-4.10727	4.00E-05	0.000125	RP11-29G	processed
ENSG0000(	23.78128	-0.38534	0.124689	-3.09042	0.001999	0.004611	KIF25	protein_cc
ENSG0000(	23.77915	-0.15699	0.086896	-1.80658	0.070827	0.114457	RP11-338	lincRNA
ENSG0000(	23.77321	-1.04583	0.117093	-8.93162	4.20E-19	1.24E-17	KLHL34	protein_cc
ENSG0000(	23.76919	0.394906	0.093228	4.235929	2.28E-05	7.41E-05	SSUH2	protein_cc
ENSG0000(	23.75056	0.340562	0.06521	5.222512	1.77E-07	8.04E-07	RP4-682C	pseudoge
ENSG0000(	23.74899	0.046665	0.057524	0.811228	0.417235	0.514247	DTX2P1	pseudoge
ENSG0000(	23.74503	0.830367	0.227188	3.654977	0.000257	0.0007	HAND2-A	antisense
ENSG0000(	23.74015	0.108763	0.097775	1.112387	0.265972	0.354848	RP11-44M	pseudoge
ENSG0000(	23.72986	0.110892	0.092135	1.203572	0.228755	0.312587	AP001627	antisense
ENSG0000(	23.72048	0.474464	0.130438	3.637459	0.000275	0.000746	RP11-395	antisense
ENSG0000(	23.71569	-0.03701	0.058603	-0.63153	0.527692	0.619457	CTD-3203	protein_cc
ENSG0000(	23.71245	-0.63442	0.107065	-5.92554	3.11E-09	1.87E-08	TRIM58	protein_cc
ENSG0000(	23.70817	0.654913	0.13685	4.78561	1.70E-06	6.64E-06	ITGB1P1	pseudoge
ENSG0000(	23.70801	-0.00323	0.045461	-0.07099	0.943404	0.960367	CTD-2145	lincRNA
ENSG0000(	23.67996	-0.03225	0.088927	-0.36263	0.716884	0.784323	LINC0059!	lincRNA
ENSG0000(	23.67864	-0.30116	0.097864	-3.07728	0.002089	0.004804	CYP2A6	protein_cc
ENSG0000(	23.67532	-0.29807	0.123103	-2.42134	0.015463	0.02959	HLA-V	pseudoge
ENSG0000(	23.66871	-0.15564	0.067426	-2.30827	0.020984	0.038883	RP11-333	lincRNA
ENSG0000(	23.66766	1.83901	0.147357	12.47997	9.60E-36	7.59E-33	RP11-696	lincRNA
ENSG0000(	23.66059	0.063798	0.038097	1.674635	0.094006	0.14643	ALKBH3-A	antisense

ENSG0000(	23.6509	-0.18716	0.091442	-2.04676	0.040682	0.070434	SULT1A2	protein_cc
ENSG0000(	23.63566	0.274856	0.048673	5.647027	1.63E-08	8.76E-08	ZNF625	protein_cc
ENSG0000(	23.63016	-0.27706	0.087471	-3.16747	0.001538	0.003625	CTD-2002	antisense
ENSG0000(	23.62926	-0.15747	0.168839	-0.93265	0.351001	0.44649	PPBP	protein_cc
ENSG0000(	23.62656	-0.21125	0.070319	-3.0042	0.002663	0.005999	RP11-65J2	lincRNA
ENSG0000(	23.62479	0.745866	0.157257	4.742967	2.11E-06	8.09E-06	RP11-100	antisense
ENSG0000(	23.62316	0.240107	0.107175	2.240329	0.02507	0.045654	DNASE2B	protein_cc
ENSG0000(	23.60162	0.187096	0.093536	2.000252	0.045473	0.077679	TRPC5OS	antisense
ENSG0000(	23.60066	-0.39469	0.065194	-6.05414	1.41E-09	8.99E-09	SLC10A5	protein_cc
ENSG0000(	23.59717	0.289465	0.12846	2.253353	0.024237	0.044299	CTB-118N	pseudoge
ENSG0000(	23.59639	0.715326	0.07355	9.725678	2.34E-22	1.21E-20	RP11-386	pseudoge
ENSG0000(	23.59538	0.380757	0.115179	3.305787	0.000947	0.002317	KB-1980E(	protein_cc
ENSG0000(	23.59523	-0.1011	0.159522	-0.63379	0.526217	0.61799	ADAM29	protein_cc
ENSG0000(	23.59042	-0.25849	0.064139	-4.03012	5.57E-05	0.00017	PRC1-AS1	antisense
ENSG0000(	23.58337	-0.1052	0.060424	-1.74105	0.081675	0.129478	RP11-981	antisense
ENSG0000(	23.55315	0.249386	0.04695	5.31172	1.09E-07	5.12E-07	RP11-85K	processed
ENSG0000(	23.55223	0.272406	0.067095	4.060036	4.91E-05	0.000151	RP11-506	antisense
ENSG0000(	23.55156	0.112687	0.161432	0.698044	0.48515	0.580131	GZMK	protein_cc
ENSG0000(	23.54984	0.081657	0.049066	1.664211	0.09607	0.149214	RP11-546	lincRNA
ENSG0000(	23.54022	-0.64681	0.087475	-7.39424	1.42E-13	1.69E-12	ACAP2-IT	sense_intr
ENSG0000(	23.53613	-0.04853	0.10442	-0.4648	0.642073	0.721911	RP11-535	lincRNA
ENSG0000(	23.53448	-0.29482	0.092024	-3.20376	0.001356	0.003228	RP11-960	lincRNA
ENSG0000(	23.49869	0.197704	0.086574	2.283635	0.022393	0.041255	RP11-304	sense_intr
ENSG0000(	23.49252	-0.5323	0.117488	-4.53065	5.88E-06	2.11E-05	PGA5	protein_cc
ENSG0000(	23.48174	0.16373	0.064709	2.530248	0.011398	0.022474	SNORA33	snoRNA
ENSG0000(	23.47672	-0.3097	0.059958	-5.16527	2.40E-07	1.07E-06	RP11-317	antisense
ENSG0000(	23.47553	-0.0559	0.069019	-0.80998	0.417953	0.514854	RNA5SP28	rRNA
ENSG0000(	23.45767	-0.29892	0.078353	-3.81501	0.000136	0.000389	TEC	protein_cc
ENSG0000(	23.45668	0.682148	0.089725	7.602614	2.90E-14	3.86E-13	RP11-274	pseudoge
ENSG0000(	23.44338	0.093159	0.102588	0.908092	0.36383	0.459807	MKRN7P	pseudoge
ENSG0000(	23.43497	0.316336	0.080291	3.939869	8.15E-05	0.000242	AC115617	pseudoge
ENSG0000(	23.42368	-0.00348	0.054243	-0.0642	0.948809	0.964619	CTD-2206	pseudoge
ENSG0000(	23.42337	-0.77864	0.121338	-6.41713	1.39E-10	1.04E-09	LINC0116(	lincRNA
ENSG0000(	23.42147	0.0739	0.068578	1.077615	0.281205	0.371818	GRK1	protein_cc
ENSG0000(	23.4024	0.153355	0.107798	1.422607	0.15485	0.224861	GLYATL1	protein_cc
ENSG0000(	23.40121	0.197371	0.054988	3.589358	0.000331	0.000885	AC093677	protein_cc
ENSG0000(	23.38965	0.101356	0.194056	0.522304	0.601459	0.686113	RP11-268	lincRNA
ENSG0000(	23.37758	-0.07142	0.067243	-1.0621	0.288192	0.379482	RP11-396	antisense
ENSG0000(	23.366	0.719312	0.112654	6.38514	1.71E-10	1.25E-09	RPSAP47	pseudoge
ENSG0000(	23.36289	-0.13458	0.056398	-2.38619	0.017024	0.032234	RP11-248	sense_intr
ENSG0000(	23.34223	0.906587	0.098704	9.18488	4.12E-20	1.43E-18	BANF1P3	pseudoge
ENSG0000(	23.33838	-0.18009	0.063976	-2.8149	0.004879	0.010416	RP11-51J9	lincRNA
ENSG0000(	23.33541	-1.31472	0.196057	-6.70581	2.00E-11	1.70E-10	GPR6	protein_cc
ENSG0000(	23.33343	-0.83131	0.11024	-7.54091	4.67E-14	6.01E-13	EHF	protein_cc
ENSG0000(	23.27864	0.087606	0.052768	1.660197	0.096875	0.150301	ABHD14A	protein_cc
ENSG0000(	23.2688	-0.05914	0.094596	-0.62523	0.531817	0.623356	LINC0115(	lincRNA
ENSG0000(	23.26548	0.143644	0.059488	2.414661	0.01575	0.030076	ASS1P12	pseudoge
ENSG0000(	23.26026	0.296002	0.16306	1.815294	0.069479	0.112601	C1orf110	protein_cc
ENSG0000(	23.23638	0.672423	0.174086	3.862597	0.000112	0.000325	CYP4F29P	pseudoge
ENSG0000(	23.2363	-0.20984	0.131315	-1.59801	0.110042	0.167798	IGLV1-51	IG_V_gene
ENSG0000(	23.23283	0.326643	0.067064	4.870603	1.11E-06	4.46E-06	RP1-315G	lincRNA
ENSG0000(	23.21697	-0.59875	0.113316	-5.2839	1.26E-07	5.89E-07	RP11-95C	lincRNA
ENSG0000(	23.21567	1.174907	0.098923	11.87698	1.56E-32	5.83E-30	RP5-1024	antisense
ENSG0000(	23.21357	-0.12057	0.062077	-1.94232	0.052098	0.087627	RP11-325	sense_intr
ENSG0000(	23.21292	-0.33993	0.116077	-2.92852	0.003406	0.007509	TDRD5	protein_cc
ENSG0000(	23.20013	-0.09815	0.058092	-1.6895	0.091124	0.142513	RP11-576	antisense
ENSG0000(	23.19886	0.024261	0.112887	0.214911	0.829836	0.875614	TNNI3	protein_cc
ENSG0000(	23.19732	0.344091	0.073558	4.677807	2.90E-06	1.09E-05	POU5F1	protein_cc
ENSG0000(	23.18108	0.327335	0.159094	2.057498	0.039638	0.068841	RP11-575	lincRNA

ENSG0000(	23.17664	0.210789	0.113025	1.864979	0.062184	0.10218	ACKR4	protein_cc
ENSG0000(	23.17126	-0.34006	0.100586	-3.38078	0.000723	0.001808	TNFSF15	protein_cc
ENSG0000(	23.1622	-0.30044	0.091127	-3.29698	0.000977	0.002384	AP000783	protein_cc
ENSG0000(	23.16088	0.882931	0.101768	8.675918	4.10E-18	1.04E-16	PSMA6P1	pseudoge
ENSG0000(	23.15364	0.244937	0.171293	1.429932	0.152737	0.222255	LUCAT1	lincRNA
ENSG0000(	23.14929	-0.40847	0.103317	-3.95354	7.70E-05	0.00023	RP11-363.	lincRNA
ENSG0000(	23.14053	0.656833	0.093311	7.039213	1.93E-12	1.93E-11	RP11-367(	pseudoge
ENSG0000(	23.13936	0.234051	0.078735	2.972645	0.002952	0.006591	RP1-104C	lincRNA
ENSG0000(	23.13682	0.079955	0.104909	0.76214	0.445977	0.542742	AC022182	lincRNA
ENSG0000(	23.12492	0.109595	0.099408	1.102469	0.270258	0.359679	RP11-8L8.	lincRNA
ENSG0000(	23.11969	0.694434	0.101169	6.864096	6.69E-12	6.11E-11	LAMB4	protein_cc
ENSG0000(	23.1174	-0.33243	0.066461	-5.0019	5.68E-07	2.39E-06	CDK2AP2I	pseudoge
ENSG0000(	23.1146	0.130559	0.073066	1.78686	0.07396	0.11888	AC005356	lincRNA
ENSG0000(	23.10061	-0.21134	0.068738	-3.0746	0.002108	0.004842	RP1-265C	lincRNA
ENSG0000(	23.09926	0.268205	0.042853	6.258767	3.88E-10	2.69E-09	RP11-159	processed
ENSG0000(	23.09501	0.155538	0.07441	2.09029	0.036592	0.064132	ITGB1BP2	protein_cc
ENSG0000(	23.07767	0.715445	0.091915	7.783741	7.04E-15	1.06E-13	GCSHP5	pseudoge
ENSG0000(	23.06732	0.082166	0.050311	1.633154	0.102437	0.157752	RBMXP4	pseudoge
ENSG0000(	23.04352	0.069757	0.057966	1.203395	0.228823	0.31266	RRH	protein_cc
ENSG0000(	23.03915	0.265809	0.105574	2.517746	0.011811	0.023213	GRIN3B	protein_cc
ENSG0000(	23.03814	0.458143	0.070436	6.504419	7.80E-11	6.04E-10	TXNDC2	protein_cc
ENSG0000(	23.0288	-0.32064	0.109868	-2.91844	0.003518	0.007735	MEG8	lincRNA
ENSG0000(	23.01029	0.622969	0.108983	5.716218	1.09E-08	6.01E-08	RP11-390	pseudoge
ENSG0000(	23.00114	0.032615	0.06662	0.489565	0.624442	0.706589	RP11-504	pseudoge
ENSG0000(	22.98272	0.293365	0.079244	3.702034	0.000214	0.00059	UBQLN4P	pseudoge
ENSG0000(	22.97162	-0.23783	0.069653	-3.41445	0.000639	0.001616	RP11-23J(	processed
ENSG0000(	22.96724	-0.11613	0.074468	-1.55949	0.11888	0.179345	RP11-379	antisense
ENSG0000(	22.96042	-0.02348	0.089377	-0.26276	0.792737	0.846437	HS6ST1P1	pseudoge
ENSG0000(	22.95851	0.143031	0.070293	2.034775	0.041873	0.072256	RP4-614C	lincRNA
ENSG0000(	22.93636	-0.06804	0.044971	-1.51291	0.130303	0.193752	CTD-2213	antisense
ENSG0000(	22.91975	-0.19199	0.129692	-1.48033	0.138784	0.204601	RP11-110(	lincRNA
ENSG0000(	22.91466	0.094066	0.070533	1.333657	0.182316	0.257985	USH2A	protein_cc
ENSG0000(	22.90966	-0.061	0.067186	-0.9079	0.363933	0.45991	AF146191	lincRNA
ENSG0000(	22.87457	0.083251	0.086639	0.960904	0.3366	0.431441	CDIPT-AS	antisense
ENSG0000(	22.83188	-1.02054	0.125773	-8.11415	4.89E-16	8.90E-15	COL10A1	protein_cc
ENSG0000(	22.81697	-0.05419	0.064666	-0.83796	0.402055	0.498857	THRB-AS1	processed
ENSG0000(	22.81675	-0.02041	0.09045	-0.2256	0.821513	0.869218	BVES-AS1	antisense
ENSG0000(	22.81429	0.059807	0.055592	1.075819	0.282008	0.372644	RP11-466	antisense
ENSG0000(	22.81307	-0.00396	0.055858	-0.07098	0.943412	0.960367	RNU6-51(	snRNA
ENSG0000(	22.80547	0.974031	0.133677	7.286468	3.18E-13	3.56E-12	MATN4	protein_cc
ENSG0000(	22.8032	-0.23881	0.10784	-2.21445	0.026798	0.048487	NOX5	protein_cc
ENSG0000(	22.78084	0.713778	0.092863	7.686394	1.51E-14	2.12E-13	RP11-435	pseudoge
ENSG0000(	22.77922	0.12137	0.04583	2.648278	0.00809	0.01651	PFKFB1	protein_cc
ENSG0000(	22.7757	0.080124	0.048277	1.659673	0.09698	0.15043	RP11-527.	antisense
ENSG0000(	22.77563	0.253806	0.103452	2.453376	0.014152	0.027333	RP11-806(	processed
ENSG0000(	22.75784	-0.98652	0.140534	-7.01977	2.22E-12	2.19E-11	MC4R	protein_cc
ENSG0000(	22.75473	0.028246	0.06758	0.417968	0.67597	0.750401	RP11-295	protein_cc
ENSG0000(	22.73806	0.338057	0.062186	5.436256	5.44E-08	2.69E-07	AC142528	antisense
ENSG0000(	22.73336	-0.87657	0.179633	-4.8798	1.06E-06	4.27E-06	CLEC4GP1	pseudoge
ENSG0000(	22.72939	1.15063	0.168363	6.834206	8.25E-12	7.42E-11	LRRC9	protein_cc
ENSG0000(	22.72813	0.415872	0.111152	3.741475	0.000183	0.000511	RP11-4K3.	pseudoge
ENSG0000(	22.71215	0.434071	0.079025	5.492843	3.96E-08	2.00E-07	LLNLR-24(	lincRNA
ENSG0000(	22.69432	0.765997	0.108074	7.087686	1.36E-12	1.39E-11	EEF1A1P3	pseudoge
ENSG0000(	22.67177	-0.76992	0.096199	-8.00333	1.21E-15	2.06E-14	DPYS	protein_cc
ENSG0000(	22.65526	0.211228	0.083366	2.533738	0.011285	0.022281	RP11-396(	lincRNA
ENSG0000(	22.65105	-0.48183	0.095472	-5.04682	4.49E-07	1.92E-06	CDHR5	protein_cc
ENSG0000(	22.65025	0.156695	0.122175	1.282551	0.19965	0.278866	RP11-785(	antisense
ENSG0000(	22.64022	0.655528	0.181671	3.608321	0.000308	0.000828	RP11-424	lincRNA
ENSG0000(	22.63388	-0.76922	0.197166	-3.90137	9.57E-05	0.000281	KNCN	protein_cc

ENSG0000(	22.63243	0.143683	0.057207	2.511614	0.012018	0.023579	CTD-2286	sense_intr(
ENSG0000(	22.62112	0.746989	0.117084	6.379937	1.77E-10	1.29E-09	RP11-567(	pseudoge
ENSG0000(	22.62056	-0.00517	0.042238	-0.12236	0.902615	0.930866	RP11-459	antisense
ENSG0000(	22.61392	-0.4484	0.089052	-5.03532	4.77E-07	2.03E-06	ZDHHC19	protein_cc
ENSG0000(	22.61159	0.223121	0.105811	2.108677	0.034973	0.061594	RP11-345	lincRNA
ENSG0000(	22.60246	-1.58072	0.175499	-9.00699	2.12E-19	6.55E-18	KIF12	protein_cc
ENSG0000(	22.58184	0.165805	0.114798	1.444318	0.148649	0.217099	LINC0068:	lincRNA
ENSG0000(	22.57828	0.840269	0.189448	4.435354	9.19E-06	3.19E-05	KISS1R	protein_cc
ENSG0000(	22.57667	0.525643	0.10055	5.22767	1.72E-07	7.83E-07	DNAJB13	protein_cc
ENSG0000(	22.57297	-0.23875	0.093279	-2.55955	0.010481	0.020847	TPPP2	protein_cc
ENSG0000(	22.56959	0.25491	0.087094	2.926824	0.003424	0.007547	ICAM3	protein_cc
ENSG0000(	22.56543	0.316007	0.081561	3.874477	0.000107	0.000311	AC093388	antisense
ENSG0000(	22.56153	0.630818	0.114059	5.530629	3.19E-08	1.64E-07	RP11-366	pseudoge
ENSG0000(	22.54447	-0.53505	0.087237	-6.13328	8.61E-10	5.67E-09	RP11-169	lincRNA
ENSG0000(	22.54203	-0.61144	0.101728	-6.01049	1.85E-09	1.16E-08	ZNF215	protein_cc
ENSG0000(	22.5203	1.14773	0.15218	7.54193	4.63E-14	5.96E-13	LINC0088(	lincRNA
ENSG0000(	22.51485	0.840484	0.12008	6.999397	2.57E-12	2.51E-11	RP11-838	lincRNA
ENSG0000(	22.51386	0.664755	0.125552	5.294655	1.19E-07	5.59E-07	PRSS30P	pseudoge
ENSG0000(	22.48502	-0.31691	0.069817	-4.53915	5.65E-06	2.03E-05	MRVI1-AS	antisense
ENSG0000(	22.4778	0.274729	0.121787	2.255825	0.024082	0.044032	ALX3	protein_cc
ENSG0000(	22.46912	-0.12731	0.068181	-1.86729	0.061861	0.101709	EBLN2	protein_cc
ENSG0000(	22.46358	-0.35073	0.136201	-2.57511	0.010021	0.02001	KCNQ1DN	antisense
ENSG0000(	22.44109	0.083294	0.066292	1.256471	0.208945	0.289774	AP4B1-AS	antisense
ENSG0000(	22.43575	0.255368	0.052601	4.854767	1.21E-06	4.81E-06	RP11-366	antisense
ENSG0000(	22.43186	-0.17508	0.056355	-3.1068	0.001891	0.004383	RP11-553	pseudoge
ENSG0000(	22.431	0.147413	0.066892	2.20376	0.027541	0.049652	RP11-602	lincRNA
ENSG0000(	22.42967	-0.22219	0.073581	-3.01967	0.002531	0.005731	RP11-799	lincRNA
ENSG0000(	22.42627	0.256621	0.075398	3.403572	0.000665	0.001676	RP11-329.	pseudoge
ENSG0000(	22.42286	-0.26933	0.080183	-3.35896	0.000782	0.001945	CTD-2126	lincRNA
ENSG0000(	22.4224	0.237053	0.12374	1.915729	0.0554	0.092435	RP11-152	processed
ENSG0000(	22.41929	-0.05202	0.124035	-0.41937	0.674945	0.749506	RP11-245(	lincRNA
ENSG0000(	22.41671	-0.16958	0.072352	-2.34384	0.019086	0.035697	RP4-647C	antisense
ENSG0000(	22.40958	0.315591	0.118674	2.659312	0.00783	0.016026	CSPG4P13	pseudoge
ENSG0000(	22.40489	-0.19785	0.089847	-2.20206	0.027661	0.049857	WNT5A-A	antisense
ENSG0000(	22.39761	-0.63616	0.074233	-8.56977	1.04E-17	2.48E-16	AFAP1-AS	antisense
ENSG0000(	22.39533	0.239067	0.049526	4.827099	1.39E-06	5.48E-06	AF129075	sense_intr(
ENSG0000(	22.39003	0.130292	0.129483	1.006246	0.314297	0.407747	CYP2C8	protein_cc
ENSG0000(	22.38921	0.487131	0.106439	4.576606	4.73E-06	1.72E-05	GUCY2C	protein_cc
ENSG0000(	22.38843	-0.8501	0.169159	-5.02546	5.02E-07	2.13E-06	GALNTL5	protein_cc
ENSG0000(	22.3787	0.419718	0.082341	5.097308	3.45E-07	1.50E-06	RP11-420	pseudoge
ENSG0000(	22.37612	0.081106	0.063505	1.277158	0.201546	0.281144	RP11-80K	lincRNA
ENSG0000(	22.3659	0.86265	0.111204	7.757391	8.67E-15	1.28E-13	RPS19P3	pseudoge
ENSG0000(	22.35391	-1.46879	0.168171	-8.7339	2.46E-18	6.47E-17	LINC0110(	lincRNA
ENSG0000(	22.35148	-0.08553	0.075466	-1.13332	0.257081	0.344951	RP11-62H	pseudoge
ENSG0000(	22.34943	0.494162	0.156143	3.164795	0.001552	0.003655	RP11-707(	pseudoge
ENSG0000(	22.34065	-0.26182	0.084238	-3.10807	0.001883	0.004365	RP11-567	processed
ENSG0000(	22.3339	-0.31617	0.116148	-2.72214	0.006486	0.013497	FAM83H-/	lincRNA
ENSG0000(	22.3278	-0.21842	0.089818	-2.43181	0.015024	0.028827	LINC0015!	lincRNA
ENSG0000(	22.3224	0.279891	0.074409	3.761509	0.000169	0.000474	RPL21P75	pseudoge
ENSG0000(	22.32003	1.175097	0.147221	7.981837	1.44E-15	2.43E-14	RP11-447	pseudoge
ENSG0000(	22.31082	0.055635	0.059344	0.937503	0.3485	0.443967	RP11-888	sense_intr(
ENSG0000(	22.30846	-0.16416	0.068453	-2.39816	0.016478	0.031318	AC132008	pseudoge
ENSG0000(	22.29074	0.24077	0.075594	3.185022	0.001447	0.003427	RP11-768	lincRNA
ENSG0000(	22.27789	-0.30802	0.074463	-4.1365	3.53E-05	0.000111	RP11-116(	pseudoge
ENSG0000(	22.26882	0.093382	0.141071	0.66195	0.508003	0.601571	C3orf52	protein_cc
ENSG0000(	22.26642	0.013005	0.079385	0.163828	0.869867	0.906578	AC156455	processed
ENSG0000(	22.25111	-0.14703	0.165777	-0.88691	0.375129	0.47171	PRRX2	protein_cc
ENSG0000(	22.24937	0.137451	0.073464	1.871006	0.061344	0.100965	MCCC1-A	antisense
ENSG0000(	22.24662	-0.05066	0.082315	-0.61543	0.538274	0.629346	RP11-54A	antisense

ENSG0000(	22.23955	-0.10449	0.077141	-1.35454	0.175564	0.250083	SIRPAP1	pseudoge
ENSG0000(	22.23803	-0.23389	0.111062	-2.10594	0.03521	0.061974	RP5-884C	lincRNA
ENSG0000(	22.23013	-0.24794	0.106576	-2.3264	0.019997	0.037217	C2CD4D	protein_cc
ENSG0000(	22.22821	0.402764	0.141612	2.844133	0.004453	0.009588	RP11-352	pseudoge
ENSG0000(	22.22264	0.350683	0.07974	4.397852	1.09E-05	3.75E-05	AC046143	antisense
ENSG0000(	22.2037	-0.28501	0.085464	-3.33491	0.000853	0.002106	RP13-225	antisense
ENSG0000(	22.18139	-2.59686	0.404043	-6.42718	1.30E-10	9.74E-10	DEFB119	protein_cc
ENSG0000(	22.17985	-0.84345	0.176523	-4.77814	1.77E-06	6.88E-06	PRSS16	protein_cc
ENSG0000(	22.17598	-0.19399	0.108452	-1.7887	0.073663	0.118467	CD200R1	protein_cc
ENSG0000(	22.17514	-0.24018	0.168555	-1.42492	0.15418	0.224061	RP1-153P	lincRNA
ENSG0000(	22.17124	-0.01933	0.159287	-0.12133	0.903433	0.931483	CXCR2P1	pseudoge
ENSG0000(	22.1568	-0.21501	0.063239	-3.39987	0.000674	0.001697	CTD-3203	antisense
ENSG0000(	22.15218	-0.60295	0.07448	-8.09549	5.70E-16	1.03E-14	C3orf49	protein_cc
ENSG0000(	22.14604	1.135815	0.125616	9.041998	1.54E-19	4.89E-18	ALDH7A1I	pseudoge
ENSG0000(	22.14136	0.276929	0.096455	2.871067	0.004091	0.00888	RP11-478	pseudoge
ENSG0000(	22.12888	-0.53314	0.110183	-4.83866	1.31E-06	5.19E-06	MORC1	protein_cc
ENSG0000(	22.11747	-0.28221	0.143231	-1.97029	0.048805	0.082703	RP4-630C	antisense
ENSG0000(	22.10929	0.346587	0.091506	3.787605	0.000152	0.00043	1-Dec	protein_cc
ENSG0000(	22.08902	-0.75523	0.109518	-6.89595	5.35E-12	4.95E-11	FGF18	protein_cc
ENSG0000(	22.08604	-0.01917	0.067587	-0.28367	0.776666	0.833302	RP11-177	pseudoge
ENSG0000(	22.08245	1.303129	0.138044	9.439979	3.73E-21	1.55E-19	RP4-592A	pseudoge
ENSG0000(	22.06944	0.690635	0.107896	6.400962	1.54E-10	1.14E-09	PEBP1P2	pseudoge
ENSG0000(	22.04788	-0.18162	0.07904	-2.29778	0.021575	0.039875	AC096670	antisense
ENSG0000(	22.02686	-0.31983	0.085748	-3.72993	0.000192	0.000533	RP11-712	pseudoge
ENSG0000(	22.02417	-0.20352	0.053864	-3.77841	0.000158	0.000446	RP5-933K	lincRNA
ENSG0000(	22.02224	-0.30317	0.174809	-1.7343	0.082865	0.1311	RP11-953	lincRNA
ENSG0000(	22.01781	0.318965	0.083706	3.810521	0.000139	0.000395	AC074091	protein_cc
ENSG0000(	22.0125	0.106812	0.07228	1.477751	0.139474	0.205442	RP11-365	antisense
ENSG0000(	22.00159	-0.2254	0.102026	-2.20921	0.02716	0.049046	RASA4B	protein_cc
ENSG0000(	21.99835	0.228553	0.052678	4.338688	1.43E-05	4.82E-05	TLK2P1	pseudoge
ENSG0000(	21.99759	0.246536	0.095109	2.592143	0.009538	0.019147	RP11-802	antisense
ENSG0000(	21.99609	0.763143	0.110948	6.878387	6.05E-12	5.56E-11	RP11-100	pseudoge
ENSG0000(	21.98181	0.135783	0.055177	2.460856	0.013861	0.026819	RP11-435	antisense
ENSG0000(	21.9786	-0.27071	0.055425	-4.8843	1.04E-06	4.18E-06	PP2D1	protein_cc
ENSG0000(	21.97825	-0.01692	0.060786	-0.27836	0.780736	0.83644	MIR635	miRNA
ENSG0000(	21.96816	0.153298	0.068996	2.221841	0.026294	0.047673	RP11-533	lincRNA
ENSG0000(	21.9458	0.274234	0.07665	3.577735	0.000347	0.000923	TRIM73	protein_cc
ENSG0000(	21.93929	0.252003	0.054527	4.621653	3.81E-06	1.41E-05	RP11-255	lincRNA
ENSG0000(	21.9277	-0.26396	0.146388	-1.80313	0.071367	0.115225	ALAS2	protein_cc
ENSG0000(	21.92725	-0.35841	0.083723	-4.28088	1.86E-05	6.16E-05	SLC12A1	protein_cc
ENSG0000(	21.9221	0.68999	0.075248	9.169519	4.75E-20	1.64E-18	SUB1P1	pseudoge
ENSG0000(	21.91255	0.22331	0.070114	3.184945	0.001448	0.003428	RP11-480	lincRNA
ENSG0000(	21.91218	-0.37513	0.130077	-2.88392	0.003928	0.008558	SMYD1	protein_cc
ENSG0000(	21.90853	-1.46003	0.180433	-8.09182	5.88E-16	1.05E-14	LINC0112	lincRNA
ENSG0000(	21.90804	0.06969	0.109899	0.634126	0.525998	0.617789	RP11-231	pseudoge
ENSG0000(	21.90366	0.346431	0.063148	5.486006	4.11E-08	2.07E-07	RP11-468	pseudoge
ENSG0000(	21.89158	-0.28716	0.153844	-1.86657	0.061962	0.101848	KCNJ15	protein_cc
ENSG0000(	21.8905	0.241283	0.065426	3.687846	0.000226	0.000621	CTA-984G	antisense
ENSG0000(	21.88974	-0.73827	0.071492	-10.3267	5.34E-25	4.68E-23	RP11-730	pseudoge
ENSG0000(	21.88046	0.123105	0.064539	1.907442	0.056463	0.093965	ZNF32-AS	antisense
ENSG0000(	21.87763	0.391063	0.146217	2.674532	0.007483	0.01538	C2CD4A	protein_cc
ENSG0000(	21.87152	-0.19843	0.098	-2.02484	0.042884	0.073777	ANKRD31	protein_cc
ENSG0000(	21.86744	-0.26103	0.078997	-3.30431	0.000952	0.002328	RP6-74O6	lincRNA
ENSG0000(	21.85304	-0.22539	0.086133	-2.61678	0.008876	0.01794	SCARNA9	antisense
ENSG0000(	21.8465	-0.22373	0.06725	-3.3268	0.000878	0.002164	CTD-2639	lincRNA
ENSG0000(	21.82776	0.252839	0.064484	3.920977	8.82E-05	0.00026	RP11-127	lincRNA
ENSG0000(	21.80696	0.098145	0.1041	0.942794	0.345786	0.441245	IL24	protein_cc
ENSG0000(	21.80353	-0.28854	0.060904	-4.73754	2.16E-06	8.30E-06	RP11-324	antisense
ENSG0000(	21.80113	-0.39278	0.099307	-3.95523	7.65E-05	0.000228	FGF22	protein_cc

ENSG0000(	21.7992	-0.46228	0.089563	-5.16149	2.45E-07	1.09E-06	AC098823	lincRNA
ENSG0000(	21.79528	-0.10258	0.098878	-1.03744	0.299532	0.391842	TNK2-AS1	antisense
ENSG0000(	21.78425	-0.98233	0.082109	-11.9638	5.50E-33	2.38E-30	OR8T1P	pseudoge
ENSG0000(	21.78015	-0.41743	0.113922	-3.66415	0.000248	0.000677	RP11-815.	pseudoge
ENSG0000(	21.7729	0.255027	0.106951	2.384529	0.017101	0.032359	S100P	protein_cc
ENSG0000(	21.73579	0.126093	0.076635	1.645376	0.099892	0.154389	TMEM191	lincRNA
ENSG0000(	21.72468	-1.18237	0.123727	-9.55634	1.22E-21	5.55E-20	FAM87A	lincRNA
ENSG0000(	21.71165	-0.33349	0.111225	-2.99828	0.002715	0.006101	ADAMTS9	antisense
ENSG0000(	21.7116	0.518375	0.079038	6.558574	5.43E-11	4.31E-10	NACAP1	pseudoge
ENSG0000(	21.70669	0.594793	0.100063	5.9442	2.78E-09	1.69E-08	AC005754	antisense
ENSG0000(	21.69936	0.422184	0.082617	5.110111	3.22E-07	1.41E-06	RP11-10C	pseudoge
ENSG0000(	21.68081	0.385106	0.074251	5.186573	2.14E-07	9.62E-07	NBPF2P	pseudoge
ENSG0000(	21.67546	1.165778	0.140397	8.303418	1.01E-16	2.06E-15	AC093616	pseudoge
ENSG0000(	21.67438	-0.0819	0.057861	-1.41544	0.15694	0.227357	RP11-767(	antisense
ENSG0000(	21.67427	0.05137	0.093628	0.54866	0.583239	0.66979	CD79A	protein_cc
ENSG0000(	21.66998	-0.92462	0.110896	-8.33769	7.58E-17	1.57E-15	RP4-735C	antisense
ENSG0000(	21.66355	-0.33425	0.141234	-2.36663	0.017951	0.033773	PCK1	protein_cc
ENSG0000(	21.65693	0.599931	0.152088	3.944629	7.99E-05	0.000238	UPK3A	protein_cc
ENSG0000(	21.65407	0.00934	0.087386	0.10688	0.914884	0.940029	AC008746	antisense
ENSG0000(	21.64364	1.474644	0.153132	9.629899	5.98E-22	2.84E-20	AC004562	lincRNA
ENSG0000(	21.63922	0.01881	0.060856	0.309085	0.757257	0.817334	CTD-2196	sense_intr(
ENSG0000(	21.63831	-0.0488	0.085888	-0.56819	0.569908	0.657711	RP11-57H	antisense
ENSG0000(	21.63726	0.620594	0.088645	7.000855	2.54E-12	2.49E-11	RP11-161	antisense
ENSG0000(	21.63079	-0.5711	0.159503	-3.58047	0.000343	0.000913	RP11-158.	sense_ove
ENSG0000(	21.62686	0.160782	0.084267	1.907998	0.056392	0.093862	RP13-188.	lincRNA
ENSG0000(	21.61715	-0.06026	0.059929	-1.00547	0.314673	0.408169	CTD-2555	processed
ENSG0000(	21.59848	1.099377	0.164931	6.665665	2.63E-11	2.20E-10	SLAMF9	protein_cc
ENSG0000(	21.59816	0.065332	0.129715	0.503658	0.614502	0.69776	PDCD1	protein_cc
ENSG0000(	21.59289	-0.3662	0.098901	-3.70265	0.000213	0.000588	AC011515	pseudoge
ENSG0000(	21.58317	-0.0332	0.082644	-0.40167	0.687926	0.760552	RP11-85A	antisense
ENSG0000(	21.57474	-0.50779	0.123068	-4.12611	3.69E-05	0.000116	GRP	protein_cc
ENSG0000(	21.5665	0.153799	0.070118	2.193435	0.028276	0.050851	RPL21P11(	pseudoge
ENSG0000(	21.56525	0.125229	0.062518	2.003094	0.045167	0.077207	RP11-173	lincRNA
ENSG0000(	21.56219	-0.29631	0.084236	-3.5176	0.000435	0.001138	RP11-770.	protein_cc
ENSG0000(	21.56109	0.229174	0.067908	3.374777	0.000739	0.001844	RP5-943J(	lincRNA
ENSG0000(	21.54626	0.023384	0.069371	0.33709	0.736049	0.800224	UTS2B	protein_cc
ENSG0000(	21.54279	-0.16305	0.060507	-2.69475	0.007044	0.014544	RP4-539M	sense_intr(
ENSG0000(	21.53286	0.015972	0.045559	0.35057	0.725911	0.791609	CTD-2619	lincRNA
ENSG0000(	21.53178	0.739651	0.111007	6.66312	2.68E-11	2.23E-10	SERBP1P5	pseudoge
ENSG0000(	21.5258	-0.98306	0.154949	-6.34439	2.23E-10	1.61E-09	ANKRD18I	protein_cc
ENSG0000(	21.52452	0.220779	0.056931	3.877994	0.000105	0.000306	ANKRD61	protein_cc
ENSG0000(	21.52368	0.071567	0.082973	0.862534	0.388394	0.48509	TAS2R31	protein_cc
ENSG0000(	21.52074	-0.04259	0.068757	-0.61944	0.535626	0.62682	RP3-441A	antisense
ENSG0000(	21.51981	0.545441	0.12828	4.251971	2.12E-05	6.94E-05	RP11-128	lincRNA
ENSG0000(	21.51766	0.159544	0.085231	1.871908	0.061219	0.100792	RP11-84C	antisense
ENSG0000(	21.51318	-0.1502	0.137031	-1.09609	0.27304	0.362924	INSC	protein_cc
ENSG0000(	21.50662	0.59748	0.068395	8.735777	2.42E-18	6.38E-17	C9orf163	protein_cc
ENSG0000(	21.49947	0.089987	0.080249	1.121352	0.262138	0.350632	RP11-236	lincRNA
ENSG0000(	21.48944	0.180439	0.088024	2.049878	0.040376	0.069972	INHBE	protein_cc
ENSG0000(	21.48314	-0.05096	0.066995	-0.76068	0.446848	0.543706	RP11-110	pseudoge
ENSG0000(	21.48251	0.85037	0.099622	8.535963	1.39E-17	3.25E-16	RP11-3P1	lincRNA
ENSG0000(	21.46675	0.398584	0.106837	3.730777	0.000191	0.000531	RP11-368	lincRNA
ENSG0000(	21.44775	0.02927	0.075202	0.389221	0.697113	0.768107	RP11-273	antisense
ENSG0000(	21.415	-0.19242	0.062955	-3.05645	0.00224	0.005121	RP11-302	pseudoge
ENSG0000(	21.40895	-0.11178	0.089638	-1.24702	0.212389	0.293758	RP11-295	lincRNA
ENSG0000(	21.39851	-0.2065	0.122146	-1.69057	0.09092	0.142231	SMCR5	antisense
ENSG0000(	21.39277	-0.1541	0.056142	-2.74486	0.006054	0.01267	RP11-574	lincRNA
ENSG0000(	21.39273	0.058853	0.180694	0.325708	0.744645	0.80731	RP11-356(	lincRNA
ENSG0000(	21.38399	-0.06207	0.058399	-1.06285	0.287851	0.379107	RP3-467L(	sense_intr(

ENSG0000(	21.37349	-0.16479	0.094525	-1.74333	0.081276	0.128962	AP006222	lincRNA
ENSG0000(	21.37024	0.413042	0.071171	5.803486	6.50E-09	3.71E-08	RP4-730K	pseudoge
ENSG0000(	21.36762	1.071868	0.115894	9.24868	2.27E-20	8.20E-19	RP11-351.	lincRNA
ENSG0000(	21.36567	-0.06168	0.134142	-0.45978	0.645671	0.725071	C2CD4B	protein_cc
ENSG0000(	21.3574	0.208105	0.091782	2.267377	0.023367	0.042841	TPTE2	protein_cc
ENSG0000(	21.35544	0.054008	0.061798	0.873946	0.382147	0.478735	CACTIN-A	antisense
ENSG0000(	21.35054	0.853549	0.115996	7.358426	1.86E-13	2.16E-12	RPL13AP6	pseudoge
ENSG0000(	21.35031	0.063159	0.073091	0.864106	0.387529	0.484194	RP5-858B	antisense
ENSG0000(	21.34744	-0.86383	0.146756	-5.88617	3.95E-09	2.34E-08	SLC38A4	protein_cc
ENSG0000(	21.34638	-0.07893	0.07515	-1.05032	0.29357	0.385294	RP11-797.	sense_intr
ENSG0000(	21.33062	0.86268	0.187694	4.596205	4.30E-06	1.57E-05	LCN9	protein_cc
ENSG0000(	21.32792	2.816332	0.227861	12.35987	4.31E-35	2.73E-32	PITX2	protein_cc
ENSG0000(	21.3227	-0.23086	0.092586	-2.49342	0.012652	0.024676	RP11-446	lincRNA
ENSG0000(	21.30736	0.904454	0.124384	7.271493	3.56E-13	3.95E-12	RPL10AP2	pseudoge
ENSG0000(	21.2999	0.744136	0.223344	3.331794	0.000863	0.002128	RP11-328	lincRNA
ENSG0000(	21.29955	0.045427	0.056752	0.800445	0.423453	0.520507	SLC23A1	protein_cc
ENSG0000(	21.29602	-0.19826	0.108467	-1.8278	0.067579	0.10989	ANXA13	protein_cc
ENSG0000(	21.29598	-0.06925	0.16	-0.43281	0.665152	0.741634	BLK	protein_cc
ENSG0000(	21.28792	-0.20585	0.126142	-1.63193	0.102695	0.158091	RP11-171.	antisense
ENSG0000(	21.28401	0.132209	0.07524	1.757169	0.078889	0.125765	RP11-546.	lincRNA
ENSG0000(	21.28034	-0.47157	0.115131	-4.09593	4.20E-05	0.000131	PKP3	protein_cc
ENSG0000(	21.26251	0.458277	0.133559	3.431274	0.000601	0.001527	RP11-958	lincRNA
ENSG0000(	21.26139	-0.3476	0.080875	-4.29806	1.72E-05	5.73E-05	AC010733	lincRNA
ENSG0000(	21.25323	0.757829	0.09567	7.921242	2.35E-15	3.82E-14	RP11-832	pseudoge
ENSG0000(	21.22374	0.217402	0.08401	2.58782	0.009659	0.019359	SCARNA1:	snoRNA
ENSG0000(	21.22363	0.00054	0.067754	0.007974	0.993637	0.995768	RP3-468K	pseudoge
ENSG0000(	21.21861	0.238592	0.106785	2.23433	0.025461	0.046288	RP11-319	sense_intr
ENSG0000(	21.21634	0.616909	0.103736	5.946935	2.73E-09	1.66E-08	RP11-490	pseudoge
ENSG0000(	21.20437	0.521706	0.181306	2.877485	0.004009	0.00872	HOXB6	protein_cc
ENSG0000(	21.20132	0.461757	0.051488	8.968221	3.01E-19	9.09E-18	AC115618	protein_cc
ENSG0000(	21.19869	0.60237	0.125378	4.804422	1.55E-06	6.09E-06	GAPDHP7	pseudoge
ENSG0000(	21.18766	0.495497	0.138987	3.565053	0.000364	0.000964	WNT10A	protein_cc
ENSG0000(	21.1727	-0.05725	0.100579	-0.56916	0.569245	0.65713	RP11-307	antisense
ENSG0000(	21.16812	0.018535	0.08228	0.225271	0.821768	0.869421	CTD-2324	lincRNA
ENSG0000(	21.16019	0.204684	0.094947	2.155759	0.031102	0.055394	CAPN8	protein_cc
ENSG0000(	21.15044	-0.0103	0.138662	-0.07426	0.940806	0.958714	RP11-20I2	antisense
ENSG0000(	21.14578	-0.28632	0.057214	-5.00434	5.61E-07	2.36E-06	CETN4P	pseudoge
ENSG0000(	21.14298	-0.17934	0.059373	-3.02064	0.002522	0.005715	LINC0093:	pseudoge
ENSG0000(	21.14183	-0.06609	0.065024	-1.01647	0.309406	0.40257	RP4-657D	antisense
ENSG0000(	21.11755	0.147941	0.074497	1.985872	0.047047	0.080032	CTB-66B2	antisense
ENSG0000(	21.09829	-0.57775	0.095405	-6.05574	1.40E-09	8.91E-09	CYP1B1-A	antisense
ENSG0000(	21.0901	-0.3476	0.14262	-2.43723	0.0148	0.028451	STK19P	pseudoge
ENSG0000(	21.07382	-0.28793	0.185091	-1.55563	0.119795	0.180403	CXCL6	protein_cc
ENSG0000(	21.07019	-0.06627	0.101838	-0.65079	0.515185	0.60802	PARD3-AS	antisense
ENSG0000(	21.06075	0.161457	0.096732	1.669117	0.095094	0.147907	RP11-455	lincRNA
ENSG0000(	21.05054	0.179381	0.086614	2.071032	0.038356	0.066876	RP13-270	antisense
ENSG0000(	21.04368	0.103819	0.084523	1.228295	0.219336	0.301772	GPBAR1	protein_cc
ENSG0000(	21.02183	0.224722	0.065945	3.407714	0.000655	0.001653	AP000688	sense_intr
ENSG0000(	21.00977	-0.10987	0.062376	-1.76145	0.078162	0.124792	TUBA3D	protein_cc
ENSG0000(	21.0072	-0.8556	0.109499	-7.81371	5.55E-15	8.47E-14	KRT86	protein_cc
ENSG0000(	20.99685	0.41184	0.060484	6.80911	9.82E-12	8.73E-11	SPG20OS	protein_cc
ENSG0000(	20.99496	-0.51294	0.1115	-4.60035	4.22E-06	1.55E-05	RP11-127	pseudoge
ENSG0000(	20.97246	-0.24895	0.112758	-2.20784	0.027255	0.04919	SLC28A3	protein_cc
ENSG0000(	20.9496	0.060215	0.072315	0.832688	0.405021	0.501885	RP11-15B	antisense
ENSG0000(	20.9468	-0.40956	0.142647	-2.87111	0.00409	0.008879	APOB	protein_cc
ENSG0000(	20.94462	0.81463	0.10613	7.675756	1.64E-14	2.29E-13	CACYBPP2	pseudoge
ENSG0000(	20.91118	-0.31431	0.110569	-2.84269	0.004473	0.009627	Z83001.1	antisense
ENSG0000(	20.90517	0.117455	0.073453	1.599036	0.109813	0.167486	RP11-128	pseudoge
ENSG0000(	20.90046	0.091141	0.105751	0.861846	0.388772	0.485452	CCDC162I	pseudoge

ENSG0000	20.89817	0.31563	0.068123	4.633262	3.60E-06	1.33E-05	CTD-2350	antisense
ENSG0000	20.88472	0.845046	0.117767	7.175583	7.20E-13	7.66E-12	NACA2	protein_cc
ENSG0000	20.88186	0.185377	0.071123	2.606407	0.00915	0.018433	RP11-472	antisense
ENSG0000	20.8674	-0.00016	0.070663	-0.00228	0.998181	0.998885	RP11-444	protein_cc
ENSG0000	20.86231	0.82979	0.082949	10.00358	1.47E-23	9.83E-22	WBP1LP2	pseudoge
ENSG0000	20.8613	-0.22035	0.064529	-3.41484	0.000638	0.001613	NONOP2	pseudoge
ENSG0000	20.85719	0.039638	0.060081	0.659736	0.509423	0.602777	FAM186B	protein_cc
ENSG0000	20.84786	-0.36369	0.119159	-3.05212	0.002272	0.00519	LA16c-36	antisense
ENSG0000	20.8436	0.142591	0.107047	1.332042	0.182846	0.25862	CTB-12O2	lincRNA
ENSG0000	20.82404	0.042546	0.064807	0.656503	0.511501	0.604626	RP11-873	lincRNA
ENSG0000	20.81533	0.198728	0.071153	2.792983	0.005222	0.01109	KB-1572G	processed
ENSG0000	20.81053	0.088407	0.135369	0.653082	0.513704	0.60662	DNASE1L3	protein_cc
ENSG0000	20.80847	-0.14108	0.116723	-1.20871	0.226774	0.310282	DNM1P46	pseudoge
ENSG0000	20.80636	0.089977	0.076649	1.173887	0.24044	0.325816	LINC0116	lincRNA
ENSG0000	20.80379	-0.00873	0.059484	-0.14669	0.883374	0.916815	RP11-390	lincRNA
ENSG0000	20.80027	-0.40124	0.089797	-4.46835	7.88E-06	2.77E-05	MTMR8	protein_cc
ENSG0000	20.79726	0.242129	0.071984	3.363666	0.000769	0.001914	RP11-318	lincRNA
ENSG0000	20.79369	-0.30738	0.146644	-2.0961	0.036074	0.063331	LYPD6B	protein_cc
ENSG0000	20.79	0.005187	0.05089	0.101916	0.918823	0.942709	XXbac-BP	antisense
ENSG0000	20.75706	0.13131	0.049192	2.669353	0.0076	0.015598	CTC-428G	antisense
ENSG0000	20.75445	0.78155	0.113684	6.874756	6.21E-12	5.70E-11	EEF1GP5	pseudoge
ENSG0000	20.74434	0.168259	0.071889	2.340554	0.019255	0.03598	TM4SF19-	antisense
ENSG0000	20.73013	-0.53074	0.085288	-6.22288	4.88E-10	3.34E-09	LY6K	protein_cc
ENSG0000	20.72628	1.100251	0.150304	7.320156	2.48E-13	2.82E-12	GAPDHP3	pseudoge
ENSG0000	20.72053	-0.05614	0.096606	-0.58111	0.561164	0.650012	FAM151A	protein_cc
ENSG0000	20.71386	-0.32154	0.114419	-2.8102	0.004951	0.010561	NXNL2	protein_cc
ENSG0000	20.71271	0.214126	0.062942	3.401954	0.000669	0.001685	STK24-AS	antisense
ENSG0000	20.71029	-1.21263	0.188539	-6.43176	1.26E-10	9.48E-10	RP11-672	processed
ENSG0000	20.70679	0.242969	0.088578	2.743001	0.006088	0.012736	STK31	protein_cc
ENSG0000	20.70646	0.382837	0.115416	3.317029	0.00091	0.002233	RP11-231	protein_cc
ENSG0000	20.69419	-0.39688	0.071574	-5.54503	2.94E-08	1.51E-07	RP11-12J1	protein_cc
ENSG0000	20.69231	-0.21383	0.137295	-1.55742	0.119371	0.179929	CTB-61M7	lincRNA
ENSG0000	20.68687	-0.38059	0.095174	-3.99884	6.37E-05	0.000193	AC005220	antisense
ENSG0000	20.67524	0.570564	0.097348	5.861075	4.60E-09	2.69E-08	MPP4	protein_cc
ENSG0000	20.6732	0.840107	0.114126	7.361202	1.82E-13	2.12E-12	RBM8B	pseudoge
ENSG0000	20.67235	0.600191	0.119451	5.024562	5.05E-07	2.14E-06	SLC9C2	protein_cc
ENSG0000	20.66644	0.16364	0.149381	1.095452	0.273318	0.363212	TRBV28	TR_V_gene
ENSG0000	20.65423	0.176554	0.060596	2.913624	0.003573	0.007845	CTD-2587	lincRNA
ENSG0000	20.65211	-0.2857	0.183372	-1.55805	0.119223	0.179745	ADARB2-A	antisense
ENSG0000	20.6443	0.0974	0.066684	1.460611	0.144122	0.211312	LL09NC01	antisense
ENSG0000	20.63976	0.133437	0.104119	1.281578	0.199991	0.279258	GTSF1	protein_cc
ENSG0000	20.63262	-0.25387	0.07853	-3.23281	0.001226	0.00294	ADORA2A	antisense
ENSG0000	20.62926	0.494649	0.113404	4.361834	1.29E-05	4.37E-05	RP11-407	processed
ENSG0000	20.62548	0.193632	0.056465	3.429207	0.000605	0.001537	AC104532	antisense
ENSG0000	20.62445	-0.94163	0.147551	-6.38174	1.75E-10	1.28E-09	HTR6	protein_cc
ENSG0000	20.61933	0.711141	0.094531	7.522823	5.36E-14	6.84E-13	NPM1P39	pseudoge
ENSG0000	20.61838	-1.81971	0.151415	-12.018	2.86E-33	1.31E-30	FBXO40	protein_cc
ENSG0000	20.60819	0.557199	0.094118	5.920251	3.21E-09	1.93E-08	LBX2	protein_cc
ENSG0000	20.60026	-0.43835	0.081398	-5.38532	7.23E-08	3.50E-07	KLB	protein_cc
ENSG0000	20.59177	-0.40837	0.058105	-7.02809	2.09E-12	2.08E-11	AP000275	protein_cc
ENSG0000	20.5751	0.516493	0.111313	4.640025	3.48E-06	1.29E-05	ZNF541	protein_cc
ENSG0000	20.57321	0.910184	0.128622	7.076439	1.48E-12	1.50E-11	RP11-392	pseudoge
ENSG0000	20.57146	0.200251	0.069273	2.890763	0.003843	0.008387	RP11-458	pseudoge
ENSG0000	20.56654	0.614262	0.07245	8.47843	2.28E-17	5.18E-16	RP11-126	antisense
ENSG0000	20.54544	0.483036	0.146659	3.293603	0.000989	0.00241	MUC3A	protein_cc
ENSG0000	20.54255	0.962303	0.132939	7.238671	4.53E-13	4.95E-12	RP11-253	pseudoge
ENSG0000	20.53531	-0.05048	0.07911	-0.63804	0.523449	0.615371	AC002059	sense_intr
ENSG0000	20.53467	-0.15787	0.085759	-1.84091	0.065635	0.107114	SLC35E1P	pseudoge
ENSG0000	20.53338	-0.07528	0.045647	-1.64912	0.099124	0.153358	RP11-215	antisense

ENSG0000(	20.52899	-0.17759	0.107021	-1.65937	0.097041	0.150503	C16orf92	protein_cc
ENSG0000(	20.5161	0.155974	0.061688	2.528422	0.011458	0.022587	CTD-2012	lincRNA
ENSG0000(	20.51429	0.161201	0.108806	1.48154	0.138463	0.204201	RP4-564F:	lincRNA
ENSG0000(	20.5021	0.747328	0.100527	7.434139	1.05E-13	1.28E-12	RPS4XP6	pseudoge
ENSG0000(	20.49891	0.027834	0.09065	0.307053	0.758803	0.818664	RP11-101.	lincRNA
ENSG0000(	20.49143	0.206124	0.117057	1.760882	0.078258	0.1249	RP11-38M	lincRNA
ENSG0000(	20.48431	-0.08899	0.053047	-1.67766	0.093414	0.145597	TTLL13	protein_cc
ENSG0000(	20.46673	0.413389	0.067284	6.143913	8.05E-10	5.32E-09	RP11-98G	lincRNA
ENSG0000(	20.45999	-0.82168	0.117603	-6.98689	2.81E-12	2.73E-11	CYP1A1	protein_cc
ENSG0000(	20.452	0.364008	0.068382	5.32314	1.02E-07	4.83E-07	CTD-2184	antisense
ENSG0000(	20.44882	-0.18529	0.061133	-3.03091	0.002438	0.005539	RP11-415	antisense
ENSG0000(	20.44292	-0.27931	0.084591	-3.30189	0.00096	0.002346	SLC5A4	protein_cc
ENSG0000(	20.44078	0.43106	0.071069	6.065384	1.32E-09	8.43E-09	AP001412	antisense
ENSG0000(	20.43575	-0.60711	0.067711	-8.96623	3.07E-19	9.24E-18	RP11-298.	sense_ove
ENSG0000(	20.43367	-0.42676	0.076657	-5.56706	2.59E-08	1.35E-07	UPB1	protein_cc
ENSG0000(	20.43205	-0.10211	0.083738	-1.21945	0.222675	0.305609	OR7E29P	pseudoge
ENSG0000(	20.42159	0.195161	0.059044	3.305342	0.000949	0.00232	RP11-100	lincRNA
ENSG0000(	20.42094	0.265167	0.091638	2.893625	0.003808	0.008316	AC016738	antisense
ENSG0000(	20.41476	-0.06956	0.071935	-0.96694	0.333576	0.428179	AC139530	miRNA
ENSG0000(	20.40894	0.115185	0.056576	2.035925	0.041758	0.072087	RP11-680	antisense
ENSG0000(	20.39754	0.024277	0.087221	0.278341	0.780751	0.83644	ARHGEF7-	antisense
ENSG0000(	20.38113	-0.07038	0.073116	-0.96259	0.335755	0.430545	RP11-656	antisense
ENSG0000(	20.37618	0.130912	0.073435	1.782696	0.074636	0.119839	ACSBG2	protein_cc
ENSG0000(	20.37526	-0.22723	0.068314	-3.32621	0.00088	0.002167	AC137932	antisense
ENSG0000(	20.36942	0.37014	0.05865	6.310971	2.77E-10	1.97E-09	RP5-1165	processed
ENSG0000(	20.35637	-0.80613	0.146385	-5.5069	3.65E-08	1.86E-07	LRRC38	protein_cc
ENSG0000(	20.34205	0.220821	0.071692	3.080122	0.002069	0.004763	AL031663	protein_cc
ENSG0000(	20.3341	0.341564	0.078491	4.35161	1.35E-05	4.57E-05	ANKDD1B	protein_cc
ENSG0000(	20.32685	-0.99925	0.122292	-8.17099	3.06E-16	5.74E-15	LINC0099(	lincRNA
ENSG0000(	20.32323	0.402774	0.097346	4.13756	3.51E-05	0.000111	RP4-791C	pseudoge
ENSG0000(	20.31934	0.399985	0.079414	5.036721	4.74E-07	2.01E-06	BOLA2B	protein_cc
ENSG0000(	20.31924	-0.24908	0.140952	-1.76709	0.077212	0.123456	RP11-219.	protein_cc
ENSG0000(	20.31057	0.756204	0.102348	7.388556	1.48E-13	1.76E-12	RP1-241P	protein_cc
ENSG0000(	20.29556	0.232749	0.076859	3.028277	0.00246	0.005584	TREX2	protein_cc
ENSG0000(	20.28973	-0.03958	0.196562	-0.20134	0.840436	0.883994	LRRC15	protein_cc
ENSG0000(	20.2869	-0.06665	0.083832	-0.79501	0.426607	0.523726	RP11-111	sense_intr
ENSG0000(	20.2837	0.252539	0.057505	4.391604	1.13E-05	3.85E-05	RP11-20E:	lincRNA
ENSG0000(	20.27634	1.46436	0.160092	9.147013	5.85E-20	1.99E-18	AC096559	lincRNA
ENSG0000(	20.26364	-0.66541	0.083625	-7.95704	1.76E-15	2.92E-14	RP11-690	sense_ove
ENSG0000(	20.25567	0.021158	0.05424	0.390088	0.696471	0.767627	CTD-3199	pseudoge
ENSG0000(	20.23813	-0.11708	0.060981	-1.91988	0.054873	0.091667	RP1-92O1	antisense
ENSG0000(	20.23094	-0.31003	0.244221	-1.26946	0.204277	0.284346	IGLL5	protein_cc
ENSG0000(	20.22876	-0.54956	0.102897	-5.34088	9.25E-08	4.41E-07	RP11-44N	lincRNA
ENSG0000(	20.22382	-0.17422	0.076631	-2.27344	0.023	0.042257	RP4-669L:	lincRNA
ENSG0000(	20.22317	-0.09454	0.098786	-0.95704	0.338546	0.433475	RP11-121	lincRNA
ENSG0000(	20.22224	-0.34138	0.08472	-4.02954	5.59E-05	0.000171	CTC-332L	lincRNA
ENSG0000(	20.21882	-0.23985	0.057751	-4.15311	3.28E-05	0.000104	AF131216	antisense
ENSG0000(	20.21658	0.135062	0.05783	2.335506	0.019517	0.03642	RP11-473	pseudoge
ENSG0000(	20.21131	0.579781	0.098113	5.909319	3.44E-09	2.06E-08	EEF1A1P1	pseudoge
ENSG0000(	20.20977	0.198869	0.072916	2.727365	0.006384	0.013302	RP11-551	lincRNA
ENSG0000(	20.20844	-0.01589	0.103493	-0.15352	0.877988	0.912745	DNM3OS	antisense
ENSG0000(	20.20634	0.656546	0.089774	7.313339	2.61E-13	2.96E-12	RP11-288	pseudoge
ENSG0000(	20.20048	-0.08738	0.064957	-1.34514	0.178579	0.253639	SERPINE3	protein_cc
ENSG0000(	20.19832	-0.37323	0.080463	-4.63851	3.51E-06	1.30E-05	RNU1-12:	snRNA
ENSG0000(	20.19394	0.845585	0.207872	4.067821	4.75E-05	0.000147	CACNG5	protein_cc
ENSG0000(	20.19196	-0.01363	0.092125	-0.14797	0.882369	0.91598	RP1-37N7	lincRNA
ENSG0000(	20.191	0.057099	0.058514	0.975812	0.329157	0.423564	RP6-109B	lincRNA
ENSG0000(	20.18368	-0.12941	0.100249	-1.29089	0.196744	0.275378	LINC0101:	lincRNA
ENSG0000(	20.1809	0.177759	0.073694	2.412133	0.01586	0.030268	AC092171	antisense

ENSG0000(	20.17613	0.835396	0.120932	6.908002	4.92E-12	4.58E-11	UBE2D3P1	pseudoge
ENSG0000(	20.16291	-0.09592	0.119956	-0.79964	0.423918	0.52097	CEACAM4	protein_cc
ENSG0000(	20.16112	0.878423	0.09923	8.852386	8.57E-19	2.41E-17	RP11-571	lincRNA
ENSG0000(	20.13044	0.408241	0.129903	3.142663	0.001674	0.00392	C10orf55	protein_cc
ENSG0000(	20.12615	0.142687	0.068334	2.088094	0.036789	0.064426	MIR3685	miRNA
ENSG0000(	20.12351	-0.28503	0.047739	-5.97063	2.36E-09	1.45E-08	MYCBP2-/	antisense
ENSG0000(	20.12275	-0.24662	0.088419	-2.78919	0.005284	0.011211	CTC-378H	antisense
ENSG0000(	20.11847	0.833604	0.132482	6.292193	3.13E-10	2.21E-09	SCML4	protein_cc
ENSG0000(	20.11718	0.263465	0.055993	4.705285	2.54E-06	9.61E-06	RP11-387	antisense
ENSG0000(	20.10122	0.623182	0.15053	4.139925	3.47E-05	0.00011	ATP5F1P5	pseudoge
ENSG0000(	20.09872	-0.22393	0.098306	-2.27785	0.022735	0.041811	RP11-255	pseudoge
ENSG0000(	20.08633	0.095852	0.067824	1.413251	0.157582	0.228087	RP5-940J5	antisense
ENSG0000(	20.08243	0.167823	0.070509	2.380162	0.017305	0.032688	PLGLB1	protein_cc
ENSG0000(	20.05234	0.662973	0.143369	4.624233	3.76E-06	1.39E-05	ULBP3	protein_cc
ENSG0000(	20.05081	-0.15594	0.092579	-1.68437	0.092111	0.143837	CTC-498M	lincRNA
ENSG0000(	20.04888	0.363118	0.060122	6.039644	1.54E-09	9.77E-09	RP11-223.	sense_ove
ENSG0000(	20.04744	0.209659	0.103598	2.023781	0.042993	0.073942	RP11-107	lincRNA
ENSG0000(	20.04609	0.022124	0.106788	0.207176	0.835872	0.88056	TBX21	protein_cc
ENSG0000(	20.03754	0.351207	0.082134	4.276002	1.90E-05	6.29E-05	RP11-304	antisense
ENSG0000(	20.03727	-0.03252	0.057603	-0.56448	0.572431	0.659845	CTD-2081	lincRNA
ENSG0000(	20.03283	-0.38234	0.079884	-4.78612	1.70E-06	6.63E-06	ZNRF3	protein_cc
ENSG0000(	20.00105	-0.28765	0.130829	-2.19869	0.0279	0.050237	LINGO4	protein_cc
ENSG0000(	19.9815	-1.33758	0.150768	-8.87174	7.20E-19	2.05E-17	TYRP1	protein_cc
ENSG0000(	19.981	-0.53236	0.070916	-7.50687	6.06E-14	7.66E-13	RGS9BP	protein_cc
ENSG0000(	19.97817	-0.72978	0.121501	-6.00639	1.90E-09	1.18E-08	CD8BP	pseudoge
ENSG0000(	19.97697	-0.44668	0.075213	-5.93882	2.87E-09	1.74E-08	RP11-548	pseudoge
ENSG0000(	19.97229	0.076436	0.069173	1.104998	0.26916	0.358474	TMEM31	protein_cc
ENSG0000(	19.95833	0.608892	0.194505	3.130479	0.001745	0.004073	AC116614	antisense
ENSG0000(	19.95343	-0.01915	0.093944	-0.20387	0.838454	0.882466	RP11-398	pseudoge
ENSG0000(	19.95284	0.029143	0.078203	0.372658	0.709403	0.778291	CTC-360J:	pseudoge
ENSG0000(	19.94565	-0.72308	0.094518	-7.65016	2.01E-14	2.75E-13	SPDEF	protein_cc
ENSG0000(	19.93693	0.232372	0.069414	3.347648	0.000815	0.002019	RP13-890	antisense
ENSG0000(	19.93394	0.398981	0.077411	5.154091	2.55E-07	1.13E-06	RP11-134	lincRNA
ENSG0000(	19.92466	-0.0476	0.074722	-0.63701	0.524119	0.616089	GOLGA8R	protein_cc
ENSG0000(	19.9159	-0.30565	0.082054	-3.72505	0.000195	0.000542	AF127936	lincRNA
ENSG0000(	19.90579	-0.40391	0.099555	-4.05718	4.97E-05	0.000153	NLRP14	protein_cc
ENSG0000(	19.88807	0.501027	0.085166	5.882939	4.03E-09	2.38E-08	RP3-418C	lincRNA
ENSG0000(	19.88727	-0.08516	0.129397	-0.65815	0.510444	0.603655	AC013275	antisense
ENSG0000(	19.87391	0.112573	0.059949	1.877831	0.060404	0.099638	AC010642	protein_cc
ENSG0000(	19.86588	-0.00793	0.07178	-0.1105	0.91201	0.937851	C6orf25	protein_cc
ENSG0000(	19.83031	0.139492	0.069772	1.99926	0.04558	0.077852	RP5-855D	antisense
ENSG0000(	19.81859	0.390404	0.076177	5.124948	2.98E-07	1.31E-06	RPL10P3	pseudoge
ENSG0000(	19.81647	0.585563	0.107254	5.459596	4.77E-08	2.38E-07	CPA2	protein_cc
ENSG0000(	19.80827	-0.57822	0.095839	-6.0333	1.61E-09	1.01E-08	QRFPR	protein_cc
ENSG0000(	19.7939	-0.0842	0.077869	-1.08135	0.279544	0.370013	ZBTB32	protein_cc
ENSG0000(	19.79009	0.009718	0.077711	0.125059	0.900477	0.929405	MIR146A	lincRNA
ENSG0000(	19.78943	0.175871	0.090154	1.950776	0.051084	0.086115	RP11-409	pseudoge
ENSG0000(	19.77901	0.431619	0.075266	5.734584	9.78E-09	5.43E-08	AQPEP	protein_cc
ENSG0000(	19.77156	0.040565	0.073417	0.552528	0.580587	0.667525	RP4-635E.	antisense
ENSG0000(	19.76761	-0.94837	0.147965	-6.40944	1.46E-10	1.08E-09	LCN15	protein_cc
ENSG0000(	19.75185	0.241469	0.056958	4.239404	2.24E-05	7.30E-05	DTX2P1-L	processed
ENSG0000(	19.75098	0.061048	0.086169	0.708473	0.478652	0.574064	DNAJC9-/	antisense
ENSG0000(	19.73456	-0.25551	0.123144	-2.07491	0.037995	0.066297	PHBP4	pseudoge
ENSG0000(	19.72946	-0.22804	0.064789	-3.51966	0.000432	0.00113	AC004471	lincRNA
ENSG0000(	19.72618	0.008267	0.062135	0.133042	0.89416	0.924874	CTC-523E	lincRNA
ENSG0000(	19.70134	0.816081	0.110485	7.386366	1.51E-13	1.78E-12	RP11-490	pseudoge
ENSG0000(	19.70067	0.436914	0.079053	5.526816	3.26E-08	1.67E-07	RP1-89D4	pseudoge
ENSG0000(	19.69954	-0.28211	0.057167	-4.93488	8.02E-07	3.29E-06	RP11-554	lincRNA
ENSG0000(	19.69736	-0.85467	0.123024	-6.94723	3.73E-12	3.54E-11	TACR3	protein_cc

ENSG0000(	19.68921	0.97614	0.134999	7.230741	4.80E-13	5.22E-12	HSPA8P9	pseudoge
ENSG0000(	19.6884	0.443573	0.125818	3.525513	0.000423	0.001107	AC017076	antisense
ENSG0000(	19.68354	0.334312	0.055419	6.032436	1.62E-09	1.02E-08	CTD-2310	antisense
ENSG0000(	19.67574	0.361652	0.073102	4.947191	7.53E-07	3.10E-06	RP4-635E	antisense
ENSG0000(	19.67132	-0.20746	0.107246	-1.93446	0.053056	0.089016	RP11-353	lincRNA
ENSG0000(	19.66057	0.265606	0.051962	5.111555	3.20E-07	1.40E-06	RP11-126	protein_cc
ENSG0000(	19.65275	0.794255	0.126424	6.282471	3.33E-10	2.34E-09	RP11-334	pseudoge
ENSG0000(	19.6395	-0.27434	0.065492	-4.18885	2.80E-05	8.99E-05	PZP	protein_cc
ENSG0000(	19.63305	-0.39771	0.096314	-4.12934	3.64E-05	0.000114	RP11-70L	antisense
ENSG0000(	19.62628	-0.19758	0.138786	-1.42361	0.15456	0.224534	HLA-DQB	antisense
ENSG0000(	19.624	-0.02414	0.122157	-0.19763	0.843331	0.88626	AC114877	lincRNA
ENSG0000(	19.62296	0.276093	0.072737	3.795748	0.000147	0.000418	HYI-AS1	antisense
ENSG0000(	19.61639	-0.23387	0.06042	-3.87067	0.000109	0.000315	SYCP3	protein_cc
ENSG0000(	19.61192	0.077334	0.136391	0.567001	0.570714	0.658438	SLC10A6	protein_cc
ENSG0000(	19.60776	0.229953	0.108145	2.126345	0.033475	0.059186	SLC52A1	protein_cc
ENSG0000(	19.60416	0.023579	0.076737	0.307267	0.75864	0.818553	RP11-80H	lincRNA
ENSG0000(	19.6013	-0.16363	0.055729	-2.93612	0.003323	0.007343	CTD-2530	antisense
ENSG0000(	19.58662	-0.01023	0.069378	-0.14738	0.88283	0.916366	FBXW4P1	pseudoge
ENSG0000(	19.57796	0.00859	0.047948	0.179155	0.857816	0.897241	SUCLA2-A	antisense
ENSG0000(	19.56246	-0.20757	0.08813	-2.35526	0.01851	0.034734	RP11-338	lincRNA
ENSG0000(	19.55781	-0.11203	0.084043	-1.33295	0.182548	0.258277	KLF1	protein_cc
ENSG0000(	19.55431	0.373531	0.113591	3.288387	0.001008	0.002453	RP11-134	lincRNA
ENSG0000(	19.54961	0.369448	0.091995	4.015961	5.92E-05	0.00018	CRYBB2	protein_cc
ENSG0000(	19.54655	-1.6027	0.170322	-9.40981	4.97E-21	2.01E-19	CIDEA	protein_cc
ENSG0000(	19.54547	-0.20422	0.164172	-1.24392	0.21353	0.295088	C6orf223	protein_cc
ENSG0000(	19.54116	-1.11939	0.142345	-7.86394	3.72E-15	5.86E-14	AC011322	pseudoge
ENSG0000(	19.53218	-0.18469	0.179632	-1.02815	0.30388	0.39671	RP11-109	pseudoge
ENSG0000(	19.52976	0.077731	0.088128	0.882027	0.377762	0.474296	LTA	protein_cc
ENSG0000(	19.52793	0.187452	0.063923	2.932486	0.003363	0.007421	RP11-188	antisense
ENSG0000(	19.52628	0.673037	0.113343	5.938067	2.88E-09	1.75E-08	LDHBP2	pseudoge
ENSG0000(	19.50839	-0.10398	0.082652	-1.258	0.20839	0.289131	AL161668	lincRNA
ENSG0000(	19.49517	0.441571	0.130714	3.378141	0.00073	0.001824	HSH2D	protein_cc
ENSG0000(	19.49507	-1.32529	0.147099	-9.00955	2.07E-19	6.42E-18	CTC-471C	lincRNA
ENSG0000(	19.47244	0.529192	0.24162	2.190179	0.028511	0.051237	KRT16	protein_cc
ENSG0000(	19.46966	-0.16432	0.109698	-1.4979	0.134158	0.198687	GBP7	protein_cc
ENSG0000(	19.46265	-0.23451	0.058173	-4.03128	5.55E-05	0.000169	RP11-303	antisense
ENSG0000(	19.45922	-0.06502	0.093872	-0.69264	0.488535	0.583196	RP13-467	antisense
ENSG0000(	19.45832	0.008025	0.116629	0.06881	0.945141	0.961722	C1orf111	protein_cc
ENSG0000(	19.45489	-0.01819	0.075119	-0.24213	0.808681	0.859078	RP11-787	antisense
ENSG0000(	19.44912	0.540631	0.169132	3.196502	0.001391	0.003306	AC009336	lincRNA
ENSG0000(	19.44604	0.12727	0.074753	1.702543	0.088654	0.139216	RP11-206	lincRNA
ENSG0000(	19.44504	-0.10374	0.128141	-0.80957	0.418188	0.515082	RP11-496	lincRNA
ENSG0000(	19.44055	-0.32011	0.102676	-3.11769	0.001823	0.004236	RP4-755D	antisense
ENSG0000(	19.43905	0.287017	0.120384	2.384184	0.017117	0.032385	NAT2	protein_cc
ENSG0000(	19.43344	-0.01373	0.087135	-0.15757	0.874794	0.910207	CNFN	protein_cc
ENSG0000(	19.42279	0.422548	0.100113	4.2207	2.44E-05	7.88E-05	RP11-621	lincRNA
ENSG0000(	19.41858	-0.13155	0.092795	-1.41764	0.156296	0.226561	FIGF	protein_cc
ENSG0000(	19.41419	-0.0257	0.098234	-0.26165	0.793594	0.84705	LETM1P2	pseudoge
ENSG0000(	19.40982	0.047174	0.251516	0.187556	0.851224	0.892362	IGLV2-14	IG_V_gene
ENSG0000(	19.40715	0.137575	0.150384	0.914823	0.360284	0.456182	MCOLN3	protein_cc
ENSG0000(	19.40386	0.241949	0.057113	4.236358	2.27E-05	7.39E-05	C20orf144	protein_cc
ENSG0000(	19.40186	0.539013	0.107887	4.996069	5.85E-07	2.45E-06	RP11-496	antisense
ENSG0000(	19.38871	0.135253	0.10784	1.254202	0.209769	0.290732	MTRNR2L	protein_cc
ENSG0000(	19.38535	0.350342	0.078335	4.472356	7.74E-06	2.72E-05	AC005306	antisense
ENSG0000(	19.38344	-0.21405	0.191876	-1.11556	0.264613	0.353364	CAPSL	protein_cc
ENSG0000(	19.38175	0.053984	0.100924	0.534896	0.592721	0.678216	TTLL2	protein_cc
ENSG0000(	19.38139	-0.33606	0.069675	-4.82322	1.41E-06	5.58E-06	RP11-56M	pseudoge
ENSG0000(	19.37955	0.161231	0.116718	1.381367	0.167166	0.23989	XXbac-BP	lincRNA
ENSG0000(	19.37827	0.382571	0.108619	3.522125	0.000428	0.00112	KLRC1	protein_cc

ENSG0000(	19.37364	0.03571	0.073318	0.487057	0.626218	0.708091	AC087793	miRNA
ENSG0000(	19.37198	0.328032	0.059482	5.514802	3.49E-08	1.78E-07	RP11-332	lincRNA
ENSG0000(	19.36045	-0.54935	0.119481	-4.59779	4.27E-06	1.56E-05	PRSS55	protein_cc
ENSG0000(	19.35583	-0.04705	0.051528	-0.91304	0.361222	0.457088	RP11-697	pseudoge
ENSG0000(	19.35398	0.017126	0.073158	0.2341	0.814908	0.864097	RP11-461.	sense_ove
ENSG0000(	19.34964	-0.21502	0.08781	-2.44867	0.014338	0.027666	RP11-111	pseudoge
ENSG0000(	19.34439	0.130953	0.141792	0.923559	0.355716	0.451315	CD8B	protein_cc
ENSG0000(	19.33666	0.01865	0.069598	0.267968	0.788724	0.843027	CTC-251D	antisense
ENSG0000(	19.33624	1.06395	0.130585	8.14758	3.71E-16	6.88E-15	RPS3AP49	pseudoge
ENSG0000(	19.33526	0.534428	0.070746	7.554193	4.21E-14	5.47E-13	CTD-2201	antisense
ENSG0000(	19.3349	0.149473	0.083131	1.79803	0.072172	0.116397	RP11-480	lincRNA
ENSG0000(	19.33031	-0.54609	0.109125	-5.00427	5.61E-07	2.36E-06	RP3-523C	lincRNA
ENSG0000(	19.32733	0.24712	0.141011	1.75249	0.07969	0.12684	OPTC	protein_cc
ENSG0000(	19.32572	-1.28948	0.175261	-7.35748	1.87E-13	2.17E-12	TMEM215	protein_cc
ENSG0000(	19.32555	0.607778	0.094384	6.439424	1.20E-10	9.04E-10	MAGEA12	protein_cc
ENSG0000(	19.31995	-0.06109	0.065758	-0.92899	0.352892	0.448465	AC079145	antisense
ENSG0000(	19.31819	0.146643	0.048511	3.022874	0.002504	0.005676	HMGN2P4	pseudoge
ENSG0000(	19.31732	-0.41546	0.114314	-3.63441	0.000279	0.000754	AP000345	lincRNA
ENSG0000(	19.30824	0.900769	0.097391	9.249017	2.27E-20	8.19E-19	RP11-382.	protein_cc
ENSG0000(	19.30047	0.061163	0.068101	0.898121	0.369121	0.465469	AC007773	antisense
ENSG0000(	19.29762	0.358697	0.110146	3.256562	0.001128	0.002722	PCOLCE-/	antisense
ENSG0000(	19.29535	-0.04136	0.065225	-0.63418	0.525964	0.617781	RP11-544	antisense
ENSG0000(	19.29484	0.439904	0.08495	5.178398	2.24E-07	1.00E-06	AC093609	lincRNA
ENSG0000(	19.29476	-0.08995	0.070511	-1.27573	0.202052	0.281743	RP1L1	protein_cc
ENSG0000(	19.28774	0.220305	0.096996	2.271274	0.02313	0.042465	LRCOL1	lincRNA
ENSG0000(	19.28713	0.711641	0.107083	6.645671	3.02E-11	2.49E-10	HNRNPA3	pseudoge
ENSG0000(	19.28548	0.252868	0.058162	4.347641	1.38E-05	4.64E-05	PRMT5-AS	antisense
ENSG0000(	19.28114	-0.0616	0.11815	-0.52139	0.602092	0.686572	LINC0111	lincRNA
ENSG0000(	19.27309	-0.04279	0.059096	-0.72414	0.468979	0.564835	RP11-677	antisense
ENSG0000(	19.27103	-0.21189	0.075175	-2.81857	0.004824	0.010305	RP11-386	antisense
ENSG0000(	19.26289	0.071178	0.06757	1.053394	0.292161	0.383664	C3orf35	protein_cc
ENSG0000(	19.23884	-0.3085	0.073547	-4.19463	2.73E-05	8.79E-05	RP11-439.	antisense
ENSG0000(	19.23859	0.158079	0.075109	2.104672	0.03532	0.062136	MIR570	miRNA
ENSG0000(	19.23163	-0.38383	0.094062	-4.08063	4.49E-05	0.000139	RP11-392	antisense
ENSG0000(	19.21748	1.062015	0.185923	5.712135	1.12E-08	6.14E-08	RP11-489	antisense
ENSG0000(	19.21662	-0.19128	0.080069	-2.38897	0.016896	0.032028	FAM86B2	protein_cc
ENSG0000(	19.20902	-0.27175	0.094124	-2.88711	0.003888	0.008477	RP5-998N	lincRNA
ENSG0000(	19.20774	-0.24002	0.075448	-3.18128	0.001466	0.003469	KRT18P34	pseudoge
ENSG0000(	19.17812	0.552287	0.071697	7.703093	1.33E-14	1.88E-13	NPM1P6	pseudoge
ENSG0000(	19.1767	0.477775	0.128528	3.71727	0.000201	0.000558	LINC0027	lincRNA
ENSG0000(	19.17444	0.325345	0.069586	4.675431	2.93E-06	1.10E-05	AC018720	pseudoge
ENSG0000(	19.17178	-0.04497	0.094042	-0.47814	0.632548	0.713737	MTRNR2L	protein_cc
ENSG0000(	19.16312	0.036817	0.085453	0.430849	0.666578	0.74262	CCIN	protein_cc
ENSG0000(	19.16064	0.282398	0.076742	3.679813	0.000233	0.00064	RP11-27N	lincRNA
ENSG0000(	19.15218	0.159359	0.086266	1.847293	0.064705	0.105785	AC072062	antisense
ENSG0000(	19.14727	0.28685	0.086849	3.302848	0.000957	0.002339	YWHAZP2	pseudoge
ENSG0000(	19.14543	0.571992	0.090607	6.312887	2.74E-10	1.95E-09	ACTL10	protein_cc
ENSG0000(	19.13789	0.565401	0.082272	6.872308	6.32E-12	5.79E-11	TMEM238	protein_cc
ENSG0000(	19.1349	1.425573	0.293113	4.863556	1.15E-06	4.61E-06	RP11-742	antisense
ENSG0000(	19.12965	0.234726	0.063523	3.695144	0.00022	0.000605	RP11-468	lincRNA
ENSG0000(	19.12739	-0.75894	0.21354	-3.55411	0.000379	0.001002	KCNMB3P	pseudoge
ENSG0000(	19.11008	-0.38465	0.074754	-5.14549	2.67E-07	1.18E-06	RP11-455	antisense
ENSG0000(	19.10771	0.069661	0.087887	0.792618	0.428	0.525092	RP4-758J1	antisense
ENSG0000(	19.10645	-1.00424	0.099898	-10.0526	8.95E-24	6.22E-22	TRIM72	protein_cc
ENSG0000(	19.10353	0.928349	0.122525	7.576796	3.54E-14	4.65E-13	RPS4XP11	pseudoge
ENSG0000(	19.08517	-0.05398	0.084107	-0.64185	0.52097	0.613247	S100Z	protein_cc
ENSG0000(	19.07926	-0.24713	0.102236	-2.41726	0.015638	0.029882	NUP210L	protein_cc
ENSG0000(	19.07655	-0.91984	0.163805	-5.61545	1.96E-08	1.04E-07	FAM153C	protein_cc
ENSG0000(	19.07598	0.586093	0.116344	5.037575	4.71E-07	2.01E-06	CATSPER1	protein_cc

ENSG0000(	19.06688	0.45742	0.114577	3.992243	6.55E-05	0.000198	AHSG	protein_cc
ENSG0000(	19.0584	-0.30996	0.111158	-2.78847	0.005296	0.011231	HTR2B	protein_cc
ENSG0000(	19.05609	-0.69441	0.077822	-8.92314	4.53E-19	1.33E-17	RP3-467L:	antisense
ENSG0000(	19.05523	-0.43291	0.123441	-3.50703	0.000453	0.00118	RP11-258(	pseudoge
ENSG0000(	19.04522	0.234904	0.0652	3.602808	0.000315	0.000844	RP11-510	antisense
ENSG0000(	19.04495	0.269928	0.112278	2.404105	0.016212	0.030883	RP5-921G	antisense
ENSG0000(	19.04313	-0.15134	0.104092	-1.45395	0.145961	0.21365	RP11-344	antisense
ENSG0000(	19.03801	-0.7321	0.099603	-7.3502	1.98E-13	2.28E-12	CTD-2269	antisense
ENSG0000(	19.02802	0.164498	0.07314	2.249091	0.024507	0.044726	RP1-199J:	antisense
ENSG0000(	19.02228	0.058037	0.080304	0.722718	0.469853	0.565722	RP11-532	lincRNA
ENSG0000(	19.00674	0.311594	0.065462	4.7599	1.94E-06	7.48E-06	CTD-3195	antisense
ENSG0000(	19.0006	-0.214	0.061333	-3.48919	0.000484	0.001253	WI2-1528	pseudoge
ENSG0000(	18.9894	-0.01959	0.088318	-0.22184	0.824437	0.87128	APOBEC3I	protein_cc
ENSG0000(	18.97753	-0.44715	0.15884	-2.81513	0.004876	0.010409	GATA6	protein_cc
ENSG0000(	18.97553	-0.05015	0.078084	-0.64222	0.520733	0.61302	MIR92B	miRNA
ENSG0000(	18.96689	0.01782	0.076486	0.232978	0.815779	0.864797	GPR158-A	antisense
ENSG0000(	18.96591	-0.11073	0.0746	-1.48428	0.137734	0.203229	RP1-149A	lincRNA
ENSG0000(	18.9643	0.776166	0.119946	6.470944	9.74E-11	7.43E-10	RP11-244.	pseudoge
ENSG0000(	18.96303	0.415765	0.076625	5.425997	5.76E-08	2.84E-07	HNRNPA1	pseudoge
ENSG0000(	18.95976	0.191982	0.082105	2.338244	0.019375	0.03618	RP11-498	lincRNA
ENSG0000(	18.92838	1.164929	0.115149	10.11674	4.66E-24	3.41E-22	RP11-87G	processed
ENSG0000(	18.92775	-0.41545	0.066623	-6.23586	4.49E-10	3.09E-09	RP11-333	lincRNA
ENSG0000(	18.90942	-0.51737	0.105523	-4.9029	9.44E-07	3.83E-06	RP11-806	antisense
ENSG0000(	18.90325	0.056344	0.109919	0.512593	0.608236	0.691982	LINC0096!	lincRNA
ENSG0000(	18.9024	0.988237	0.135239	7.307342	2.72E-13	3.09E-12	C20orf195	protein_cc
ENSG0000(	18.9024	0.049993	0.144552	0.345845	0.729459	0.794593	RP11-598	antisense
ENSG0000(	18.90179	0.674971	0.129541	5.210474	1.88E-07	8.54E-07	LINC0060:	lincRNA
ENSG0000(	18.9011	-0.41998	0.246711	-1.70232	0.088696	0.139256	PARP4P3	pseudoge
ENSG0000(	18.89857	-0.59431	0.110341	-5.38615	7.20E-08	3.49E-07	RP11-138	lincRNA
ENSG0000(	18.89353	-0.23155	0.081824	-2.82979	0.004658	0.00998	CTD-2235	lincRNA
ENSG0000(	18.89339	-0.38433	0.091638	-4.19399	2.74E-05	8.81E-05	RP11-116	lincRNA
ENSG0000(	18.87087	0.006113	0.066594	0.091789	0.926866	0.948524	RP11-762	sense_intr(
ENSG0000(	18.87064	-0.15855	0.092085	-1.72173	0.085118	0.134295	FAM85A	antisense
ENSG0000(	18.86989	0.531743	0.1006	5.285712	1.25E-07	5.84E-07	RPL5P23	pseudoge
ENSG0000(	18.86303	-0.3246	0.089366	-3.63223	0.000281	0.00076	RP11-266	pseudoge
ENSG0000(	18.8601	0.084769	0.068488	1.237719	0.21582	0.297822	RP11-356	sense_intr(
ENSG0000(	18.8439	0.323893	0.094973	3.410389	0.000649	0.001638	AC007966	antisense
ENSG0000(	18.83613	-1.62584	0.179626	-9.05123	1.41E-19	4.51E-18	RP11-321	lincRNA
ENSG0000(	18.83612	0.274305	0.056866	4.823727	1.41E-06	5.57E-06	CTC-232P	antisense
ENSG0000(	18.83321	-0.65853	0.269137	-2.44682	0.014412	0.027796	ANKRD62	pseudoge
ENSG0000(	18.83107	0.06991	0.066616	1.04944	0.293975	0.385751	RP11-84C	lincRNA
ENSG0000(	18.82846	0.225146	0.119194	1.888908	0.058904	0.097488	RP11-481	pseudoge
ENSG0000(	18.8275	-0.41905	0.103627	-4.04389	5.26E-05	0.000161	ERVW-1	protein_cc
ENSG0000(	18.81711	0.027834	0.085871	0.324142	0.745831	0.808401	AC096574	processed
ENSG0000(	18.79459	-0.05371	0.049933	-1.07567	0.282073	0.37269	RP11-700.	antisense
ENSG0000(	18.78065	-0.36049	0.092416	-3.90069	9.59E-05	0.000281	MTL5	protein_cc
ENSG0000(	18.78032	0.526558	0.094031	5.599822	2.15E-08	1.13E-07	RP11-879	lincRNA
ENSG0000(	18.77335	-0.33611	0.070715	-4.75299	2.00E-06	7.72E-06	ALS2CR11	protein_cc
ENSG0000(	18.76677	-0.17649	0.094758	-1.86249	0.062534	0.102701	SMIM1	protein_cc
ENSG0000(	18.75256	0.801199	0.118298	6.772714	1.26E-11	1.10E-10	VDAC1P1	pseudoge
ENSG0000(	18.73878	-2.06536	0.208443	-9.90854	3.82E-23	2.30E-21	HTR3B	protein_cc
ENSG0000(	18.73261	0.193107	0.057285	3.370982	0.000749	0.001867	AC005740	antisense
ENSG0000(	18.72914	-0.02626	0.075497	-0.34777	0.72801	0.793351	RP11-109	lincRNA
ENSG0000(	18.72398	0.206938	0.068237	3.032623	0.002424	0.00551	JSRP1	protein_cc
ENSG0000(	18.72203	0.054597	0.09259	0.589659	0.555419	0.644918	CLIC3	protein_cc
ENSG0000(	18.72104	-0.02512	0.055373	-0.45358	0.650128	0.728789	HMGB3P2	pseudoge
ENSG0000(	18.71978	0.07988	0.132713	0.601899	0.547241	0.637583	LIPM	protein_cc
ENSG0000(	18.71544	0.066746	0.064832	1.02953	0.303231	0.395963	CTD-2154	antisense
ENSG0000(	18.713	-0.26644	0.100468	-2.65197	0.008002	0.016348	CTD-2008	lincRNA

ENSG0000(	18.70721	0.004245	0.069207	0.061335	0.951093	0.966295	GS1-166A	lincRNA
ENSG0000(	18.70532	0.253035	0.123004	2.057122	0.039674	0.068892	FMO1	protein_cc
ENSG0000(	18.70511	-0.02855	0.066831	-0.42725	0.6692	0.744613	RP11-288	antisense
ENSG0000(	18.6994	0.433282	0.127418	3.400469	0.000673	0.001694	RP11-536	lincRNA
ENSG0000(	18.6839	-0.2659	0.113212	-2.34869	0.018839	0.035275	RP11-326	lincRNA
ENSG0000(	18.67784	0.670486	0.173671	3.860676	0.000113	0.000327	TPSB2	polymorpl
ENSG0000(	18.67394	-0.57201	0.073852	-7.74529	9.54E-15	1.40E-13	RP11-253	lincRNA
ENSG0000(	18.67087	-1.07502	0.126385	-8.50591	1.80E-17	4.14E-16	IL1RL2	protein_cc
ENSG0000(	18.66184	0.078701	0.126286	0.623197	0.533155	0.624586	KCNK15	protein_cc
ENSG0000(	18.65548	0.330589	0.1351	2.446986	0.014406	0.027784	MIR4461	pseudoge
ENSG0000(	18.65501	0.090068	0.083132	1.083436	0.278615	0.368995	PCDHGA8	protein_cc
ENSG0000(	18.65268	0.470603	0.098355	4.78472	1.71E-06	6.67E-06	UBQLNL	protein_cc
ENSG0000(	18.64874	1.295638	0.173056	7.486804	7.06E-14	8.84E-13	FEV	protein_cc
ENSG0000(	18.64241	-0.03159	0.064201	-0.49205	0.622686	0.705087	C6orf165	protein_cc
ENSG0000(	18.64226	0.163237	0.126761	1.287755	0.197831	0.276665	RP11-288	antisense
ENSG0000(	18.63599	0.270856	0.065176	4.155772	3.24E-05	0.000103	RP11-91G	lincRNA
ENSG0000(	18.63539	0.083241	0.137359	0.606008	0.544509	0.635011	RP11-277	lincRNA
ENSG0000(	18.62018	0.345878	0.093	3.719121	0.0002	0.000554	C9orf173	protein_cc
ENSG0000(	18.61833	-0.0124	0.088413	-0.14023	0.88848	0.92054	RP4-798P	processed
ENSG0000(	18.6172	0.270705	0.071565	3.782621	0.000155	0.000438	LRRC69	protein_cc
ENSG0000(	18.6162	0.074195	0.059247	1.252295	0.210462	0.291543	RP11-505	antisense
ENSG0000(	18.6138	0.085017	0.05761	1.475738	0.140014	0.206134	RP11-573	pseudoge
ENSG0000(	18.59093	0.03708	0.055374	0.66962	0.5031	0.596961	RP11-298	antisense
ENSG0000(	18.56246	-1.1294	0.181176	-6.23373	4.55E-10	3.13E-09	AC011516	lincRNA
ENSG0000(	18.559	-0.67306	0.118196	-5.69444	1.24E-08	6.76E-08	AKR1B15	protein_cc
ENSG0000(	18.54622	-0.03073	0.074616	-0.41188	0.680426	0.753984	CTAGE7P	pseudoge
ENSG0000(	18.53931	0.79986	0.109781	7.285994	3.19E-13	3.57E-12	ATF4P3	pseudoge
ENSG0000(	18.53599	-0.03806	0.08097	-0.47	0.638352	0.718512	CEP164P1	pseudoge
ENSG0000(	18.51918	0.370553	0.070699	5.241298	1.59E-07	7.31E-07	RP11-42C	lincRNA
ENSG0000(	18.51689	-0.06536	0.085486	-0.76452	0.44456	0.541386	RP5-1091	antisense
ENSG0000(	18.50186	0.973621	0.142254	6.844255	7.69E-12	6.95E-11	RP11-378	pseudoge
ENSG0000(	18.49447	0.027108	0.068693	0.394626	0.693119	0.764875	AC004076	protein_cc
ENSG0000(	18.49233	-0.18851	0.077007	-2.44799	0.014366	0.027716	RN7SL268	misc_RNA
ENSG0000(	18.48511	0.057085	0.108594	0.525674	0.599115	0.684085	DDC	protein_cc
ENSG0000(	18.47119	-0.76623	0.097517	-7.85745	3.92E-15	6.15E-14	TRGC1	TR_C_gen
ENSG0000(	18.46808	-0.0227	0.065051	-0.34895	0.727127	0.792515	RP11-536	antisense
ENSG0000(	18.46595	0.108809	0.083353	1.305403	0.191756	0.269396	RP11-560	lincRNA
ENSG0000(	18.46136	-0.04712	0.103956	-0.45323	0.65038	0.728959	RP11-613	lincRNA
ENSG0000(	18.46045	1.181882	0.141377	8.359813	6.28E-17	1.32E-15	RP11-101	lincRNA
ENSG0000(	18.45987	0.085772	0.134337	0.638488	0.523156	0.615168	CD3G	protein_cc
ENSG0000(	18.45499	0.102996	0.081172	1.268869	0.204488	0.284581	AC104653	lincRNA
ENSG0000(	18.45045	0.060096	0.09769	0.615167	0.538444	0.629491	BLOC1S5-	protein_cc
ENSG0000(	18.44961	-0.28132	0.149121	-1.8865	0.059227	0.097948	RP1-193H	lincRNA
ENSG0000(	18.44229	-1.92633	0.190903	-10.0906	6.08E-24	4.35E-22	RP11-87E	antisense
ENSG0000(	18.41612	0.39801	0.110842	3.590798	0.00033	0.000881	UMODL1	protein_cc
ENSG0000(	18.40017	0.162631	0.063575	2.558083	0.010525	0.020924	RP11-455	antisense
ENSG0000(	18.39587	-0.63546	0.112405	-5.65334	1.57E-08	8.47E-08	RP11-466	antisense
ENSG0000(	18.3927	0.026231	0.161797	0.162124	0.871208	0.907441	C1DP1	pseudoge
ENSG0000(	18.38035	-0.19871	0.053705	-3.70011	0.000216	0.000594	RAD21-AS	antisense
ENSG0000(	18.37714	0.163519	0.049819	3.282255	0.00103	0.002503	PRH1	protein_cc
ENSG0000(	18.37536	-0.35839	0.083506	-4.29186	1.77E-05	5.88E-05	MYO1A	protein_cc
ENSG0000(	18.37145	-0.68766	0.122378	-5.61912	1.92E-08	1.02E-07	MTATP6P	pseudoge
ENSG0000(	18.37016	0.164013	0.114463	1.432892	0.151889	0.221201	ZNF295-A	lincRNA
ENSG0000(	18.36781	0.645481	0.117872	5.476134	4.35E-08	2.18E-07	RP4-794H	lincRNA
ENSG0000(	18.36615	-0.02494	0.069575	-0.35843	0.72002	0.786917	RP11-68L	antisense
ENSG0000(	18.36342	0.424643	0.074834	5.674486	1.39E-08	7.55E-08	DHFRP1	pseudoge
ENSG0000(	18.3548	-0.53574	0.071674	-7.47474	7.74E-14	9.60E-13	CTD-2325	antisense
ENSG0000(	18.34472	-0.24996	0.089257	-2.80049	0.005102	0.010858	GSG1	protein_cc
ENSG0000(	18.33718	0.251464	0.085081	2.955598	0.003121	0.006931	RP11-139	lincRNA

ENSG0000(	18.33539	0.475584	0.060847	7.816021	5.45E-15	8.33E-14	TMEM253	protein_cc
ENSG0000(	18.33385	0.686035	0.080756	8.495121	1.98E-17	4.51E-16	RPL26P30	pseudoge
ENSG0000(	18.3273	0.479001	0.109202	4.38638	1.15E-05	3.94E-05	WWTR1-A	antisense
ENSG0000(	18.31805	-0.35673	0.058056	-6.14468	8.01E-10	5.30E-09	MAP2K4P1	pseudoge
ENSG0000(	18.31203	0.611158	0.140547	4.348411	1.37E-05	4.63E-05	LIMS3	protein_cc
ENSG0000(	18.30076	0.287916	0.096307	2.989546	0.002794	0.006262	CTA-223H	lincRNA
ENSG0000(	18.29828	-0.1251	0.13126	-0.95308	0.340549	0.435661	AL589743	lincRNA
ENSG0000(	18.2926	0.182083	0.070326	2.589139	0.009622	0.019294	RP11-21L	antisense
ENSG0000(	18.28379	1.394896	0.198362	7.032075	2.03E-12	2.02E-11	AC004057	pseudoge
ENSG0000(	18.28211	-0.10404	0.075906	-1.37062	0.170494	0.243951	POU5F1P3	pseudoge
ENSG0000(	18.27439	0.469204	0.142454	3.293713	0.000989	0.00241	SNORA73	snoRNA
ENSG0000(	18.27362	1.039496	0.119617	8.690174	3.62E-18	9.27E-17	RPS3AP25	pseudoge
ENSG0000(	18.27349	0.11995	0.060026	1.99831	0.045683	0.077995	MFSD2B	protein_cc
ENSG0000(	18.26593	0.197434	0.197588	0.999221	0.317688	0.411393	BMP5	protein_cc
ENSG0000(	18.25144	-0.0928	0.135198	-0.68638	0.492475	0.586934	RFX8	protein_cc
ENSG0000(	18.23452	0.004474	0.080226	0.055767	0.955528	0.969304	AP000347	pseudoge
ENSG0000(	18.23365	0.08557	0.071365	1.199051	0.230508	0.314502	RP11-498	lincRNA
ENSG0000(	18.23336	-0.20904	0.075021	-2.78643	0.005329	0.011295	AC138035	lincRNA
ENSG0000(	18.22858	0.134682	0.07415	1.816349	0.069317	0.112369	WISP3	protein_cc
ENSG0000(	18.21738	-0.19067	0.05639	-3.38129	0.000721	0.001805	RP4-785G	pseudoge
ENSG0000(	18.21236	0.175879	0.107551	1.635302	0.101986	0.157156	LINC0070	lincRNA
ENSG0000(	18.19258	-0.12095	0.075218	-1.60795	0.107845	0.164852	RP11-589	sense_intr
ENSG0000(	18.19049	-0.17077	0.099481	-1.71661	0.086051	0.135599	TCF15	protein_cc
ENSG0000(	18.1836	0.316239	0.072918	4.33688	1.45E-05	4.86E-05	PABPC1P3	pseudoge
ENSG0000(	18.1466	0.66452	0.167873	3.958481	7.54E-05	0.000225	HOXD3	protein_cc
ENSG0000(	18.14646	-0.76166	0.106351	-7.16178	7.96E-13	8.41E-12	RP11-564	pseudoge
ENSG0000(	18.14012	-0.45218	0.077791	-5.81284	6.14E-09	3.52E-08	PDLIM1P4	pseudoge
ENSG0000(	18.13909	0.747829	0.12972	5.764927	8.17E-09	4.60E-08	HSPA8P8	pseudoge
ENSG0000(	18.13846	-0.01425	0.120494	-0.11828	0.90585	0.933287	RP3-518E	antisense
ENSG0000(	18.12244	-0.96311	0.138865	-6.9356	4.05E-12	3.83E-11	EDAR	protein_cc
ENSG0000(	18.11747	0.214986	0.089778	2.394647	0.016636	0.031592	GFI1	protein_cc
ENSG0000(	18.11441	-0.33348	0.076889	-4.33719	1.44E-05	4.85E-05	RP11-513	antisense
ENSG0000(	18.10727	0.524476	0.080932	6.480424	9.15E-11	7.02E-10	GTF2IRD1	pseudoge
ENSG0000(	18.10413	-0.13872	0.047031	-2.94943	0.003184	0.007058	AC005255	pseudoge
ENSG0000(	18.10315	-0.36746	0.122404	-3.00206	0.002682	0.006037	AP000344	pseudoge
ENSG0000(	18.10279	0.105937	0.049236	2.151604	0.031429	0.055924	SAP30L-A	antisense
ENSG0000(	18.10171	0.078532	0.073211	1.072679	0.283415	0.37408	RP11-571	lincRNA
ENSG0000(	18.09998	0.072931	0.074303	0.981539	0.326327	0.420515	RP11-440	lincRNA
ENSG0000(	18.09897	0.051986	0.065126	0.798237	0.424733	0.521815	KB-226F1.	lincRNA
ENSG0000(	18.09436	-0.0359	0.063414	-0.56605	0.571362	0.659001	RP11-141	processed
ENSG0000(	18.09108	1.088351	0.125656	8.661379	4.66E-18	1.17E-16	RP11-318	pseudoge
ENSG0000(	18.08918	0.123508	0.085069	1.451863	0.14654	0.214413	CTD-2012	lincRNA
ENSG0000(	18.08745	0.105774	0.06175	1.712924	0.086727	0.136555	CTD-2349	sense_intr
ENSG0000(	18.08707	-0.35337	0.250366	-1.41143	0.158117	0.228676	IGLV1-40	IG_V_gene
ENSG0000(	18.0847	-0.44249	0.153582	-2.88114	0.003962	0.008626	AC079776	pseudoge
ENSG0000(	18.07514	-0.39395	0.103545	-3.80464	0.000142	0.000404	RP11-34P	pseudoge
ENSG0000(	18.07284	-0.01432	0.113436	-0.12623	0.899554	0.928709	PSCA	protein_cc
ENSG0000(	18.06937	0.684005	0.094536	7.235422	4.64E-13	5.06E-12	ATF4P4	pseudoge
ENSG0000(	18.06909	-0.04744	0.126072	-0.37633	0.706673	0.776103	TNFRSF9	protein_cc
ENSG0000(	18.0686	0.011678	0.08236	0.141792	0.887244	0.91974	SLC5A2	protein_cc
ENSG0000(	18.05895	0.426017	0.072787	5.852917	4.83E-09	2.82E-08	RP5-977B	antisense
ENSG0000(	18.04945	-0.14716	0.102973	-1.42914	0.152964	0.222491	PAPPA-AS	antisense
ENSG0000(	18.04359	0.29501	0.072103	4.091514	4.29E-05	0.000133	AC007461	lincRNA
ENSG0000(	18.04112	-0.10764	0.091729	-1.17345	0.240617	0.326013	RP11-14N	lincRNA
ENSG0000(	18.04073	-0.03328	0.096897	-0.34346	0.731253	0.79621	CTD-2523	antisense
ENSG0000(	18.03908	-0.01084	0.064208	-0.16887	0.865902	0.903654	RP11-346	lincRNA
ENSG0000(	18.00063	-0.2768	0.086177	-3.212	0.001318	0.003144	RP11-444	lincRNA
ENSG0000(	17.99971	0.454748	0.123786	3.673656	0.000239	0.000654	DKKL1	protein_cc
ENSG0000(	17.993	-0.86296	0.182638	-4.72495	2.30E-06	8.79E-06	C6	protein_cc

ENSG0000(	17.98569	0.367916	0.122697	2.998566	0.002713	0.006096	GBP6	protein_cc
ENSG0000(	17.98403	-0.04311	0.113625	-0.37944	0.704364	0.774333	CTLA4	protein_cc
ENSG0000(	17.9731	-0.21682	0.056825	-3.81564	0.000136	0.000388	CTD-2530	sense_intr
ENSG0000(	17.9712	0.004787	0.094322	0.050756	0.95952	0.972311	RP11-133	antisense
ENSG0000(	17.94319	-0.46729	0.07097	-6.5843	4.57E-11	3.67E-10	RP11-446	lincRNA
ENSG0000(	17.92981	-1.42427	0.168197	-8.46787	2.50E-17	5.64E-16	KCNG3	protein_cc
ENSG0000(	17.92807	0.185021	0.11443	1.616902	0.105899	0.162279	RP11-475	pseudoge
ENSG0000(	17.92357	0.387785	0.073631	5.266569	1.39E-07	6.43E-07	HNRNPA1	pseudoge
ENSG0000(	17.91629	-0.08082	0.080231	-1.00736	0.31376	0.40714	SLC28A2	protein_cc
ENSG0000(	17.90798	0.138381	0.055455	2.495367	0.012583	0.024559	KCNRG	protein_cc
ENSG0000(	17.90124	0.214729	0.089443	2.400726	0.016363	0.031131	TAF7L	protein_cc
ENSG0000(	17.90021	0.206145	0.042042	4.903293	9.42E-07	3.82E-06	RP11-453	pseudoge
ENSG0000(	17.89672	0.915203	0.141142	6.484275	8.92E-11	6.85E-10	RP11-436	pseudoge
ENSG0000(	17.88315	0.078136	0.128861	0.606354	0.54428	0.634816	IL18RAP	protein_cc
ENSG0000(	17.86982	-0.05086	0.089648	-0.56729	0.570514	0.658266	ZIM2	protein_cc
ENSG0000(	17.86856	-1.0734	0.164839	-6.51178	7.43E-11	5.78E-10	RP11-116	antisense
ENSG0000(	17.86622	-0.46	0.166648	-2.7603	0.005775	0.012141	RP11-116	lincRNA
ENSG0000(	17.85858	0.390804	0.130214	3.001248	0.002689	0.006051	SLC35F4	protein_cc
ENSG0000(	17.85092	-0.15202	0.088315	-1.72134	0.08519	0.13439	NADK2-A	antisense
ENSG0000(	17.84138	-0.16838	0.06756	-2.49227	0.012693	0.02475	RP11-440	antisense
ENSG0000(	17.83886	-1.76607	0.157633	-11.2037	3.91E-29	7.62E-27	AC061961	antisense
ENSG0000(	17.83883	0.089458	0.065481	1.366157	0.17189	0.245623	AC016907	antisense
ENSG0000(	17.83078	-0.33258	0.090661	-3.66842	0.000244	0.000667	RP11-219	antisense
ENSG0000(	17.83067	-0.64999	0.14697	-4.42261	9.75E-06	3.37E-05	SSTR5	protein_cc
ENSG0000(	17.82962	0.977186	0.127332	7.674318	1.66E-14	2.31E-13	TPT1P6	pseudoge
ENSG0000(	17.82669	0.139857	0.060225	2.322244	0.02022	0.037596	OCIAD1-A	antisense
ENSG0000(	17.82099	0.099687	0.0797	1.25077	0.211018	0.292205	RP11-293	lincRNA
ENSG0000(	17.81666	-1.41956	0.21014	-6.75531	1.43E-11	1.23E-10	RP11-325	lincRNA
ENSG0000(	17.81404	0.860278	0.105455	8.157788	3.41E-16	6.36E-15	HIST2H2A	protein_cc
ENSG0000(	17.81109	-0.04919	0.070212	-0.70052	0.483602	0.578685	MIR4292	miRNA
ENSG0000(	17.80562	0.283738	0.055543	5.108431	3.25E-07	1.42E-06	AC006378	antisense
ENSG0000(	17.79781	-0.43436	0.10691	-4.06283	4.85E-05	0.00015	AL133493	lincRNA
ENSG0000(	17.79682	0.165928	0.068162	2.434295	0.014921	0.028649	UBOX5-A	antisense
ENSG0000(	17.79203	0.729941	0.085221	8.565312	1.08E-17	2.57E-16	FAM72D	protein_cc
ENSG0000(	17.78573	0.19729	0.068104	2.896889	0.003769	0.008236	RP11-137	pseudoge
ENSG0000(	17.76941	0.376039	0.071691	5.245259	1.56E-07	7.16E-07	RP13-270	antisense
ENSG0000(	17.76458	0.336839	0.065531	5.140135	2.75E-07	1.21E-06	AC009948	pseudoge
ENSG0000(	17.75942	0.201806	0.107498	1.877307	0.060476	0.09974	AC083867	lincRNA
ENSG0000(	17.75628	0.157446	0.060784	2.59024	0.009591	0.01924	DENND5B	antisense
[truncated: 646,529 more chars]
